# Changes in the spatial distribution of the under-five mortality rate: Small-area analysis of 122 DHS surveys in 262 subregions of 35 countries in Africa

**DOI:** 10.1371/journal.pone.0210645

**Published:** 2019-01-22

**Authors:** Zehang Li, Yuan Hsiao, Jessica Godwin, Bryan D. Martin, Jon Wakefield, Samuel J. Clark, with support from the United Nations Inter-agency Group for Child Mortality Estimation and its technical advisory group

**Affiliations:** 1 Department of Biostatistics, Yale School of Public Health, New Haven, Connecticut, United States of America; 2 Department of Statistics, University of Washington, Seattle, Washington, United States of America; 3 Department of Biostatistics, University of Washington, Seattle, Washington, United States of America; 4 Department of Sociology, University of Washington, Seattle, Washington, United States of America; 5 Department of Sociology, The Ohio State University, Columbus, Ohio, United States of America; 6 MRC/Wits Rural Public Health and Health Transitions Research Unit (Agincourt), School of Public Health, Faculty of Health Sciences, University of the Witwatersrand, Johannesburg, South Africa; The University of Warwick, UNITED KINGDOM

## Abstract

The under-five mortality rate (U5MR) is a critical and widely available population health indicator. Both the MDGs and SDGs define targets for improvement in the U5MR, and the SDGs require spatial disaggregation of indicators. We estimate trends in the U5MR for Admin-1 subnational areas using 122 DHS surveys in 35 countries in Africa and assess progress toward the MDG target reductions for each subnational region and each country as a whole. In each country, direct weighted estimates of the U5MR from each survey are calculated and combined into a single estimate for each Admin-1 region across five-year periods. Our method fully accounts for the sample design of each survey. The region-time-specific estimates are smoothed using a Bayesian, space-time model that produces more precise estimates (when compared to the direct estimates) at a one-year scale that are consistent with each other in both space and time. The resulting estimated distributions of the U5MR are summarized and used to assess subnational progress toward the MDG 4 target of two-thirds reduction in the U5MR during 1990–2015. Our space-time modeling approach is tractable and can be readily applied to a large collection of sample survey data. Subnational, regional spatial heterogeneity in the levels and trends in the U5MR vary considerably across Africa. There is no generalizable pattern between spatial heterogeneity and levels or trends in the U5MR. Subnational, small-area estimates of the U5MR: (i) identify subnational regions where interventions are still necessary and those where improvement is well under way; and (ii) countries where there is very little spatial variation and others where there are important differences between subregions in both levels and trends. More work is necessary to improve both the data sources and methods necessary to adequately measure subnational progress toward the SDG child survival targets.

## Introduction

Mortality is the most direct and important indicator of health at the population level, but globally 62 percent of deaths are unreported, mostly in Africa [1]. We know very little about the timing or cause of those deaths—how many potential years of life are lost and what diseases or conditions are the primary killers—and that critically limits our ability to evaluate and monitor population health and target interventions in the settings where they are needed most. In many areas without fully functioning vital statistics systems there are data from household surveys and censuses over the past several decades that can be used to estimate measures of child mortality at reasonable geographic granularity, including large parts of Africa. The under-five mortality rate (U5MR), _5_*q*_0_, is often used in conjunction with model life tables to extrapolate mortality indicators at other ages [2].

Child survival is inherently important in its own right, and both the Millennium Development Goals (MDG) [3, 4] and the Sustainable Development Goals (SDG) [5, 6] contain specific targets for the improvement of child survival. MDG 4 targeted a two-thirds proportional reduction in child mortality between 1990 and 2015. The globe as a whole missed this target with an estimated 53 percent reduction, and only two major world regions—East Asia and the Pacific and Latin America and the Caribbean—and 62/195 (32 percent) countries are estimated to have reached the target. Sub-Saharan Africa saw an overall estimated reduction of 24–39 percent in East and Southern Africa and 12 percent in West and Central Africa. There was an estimated 67 percent reduction in Asia as a whole with South Asia contributing 60 percent and East Asia and the Pacific 79 percent [7].

SDG goal 3 redefines the child mortality target to include fixed numerical targets for neonatal mortality (NMR) (≤ 12/1,000 live births) and U5MR (< 25/1,000 live births) and the elimination of preventable child deaths between birth and age five by 2030. The SDG framework as a whole requires *inclusion*—attention to subgroups that may be missing from traditional measurement systems or different in consequential ways from the population as a whole. In the SDG resolution [5, 8] this is operationalized in paragraph 74.g as the disaggregation of indicators along important dimensions that are relevant to ‘national context’:

They will be rigorous and based on evidence, informed by country-led evaluations and data which is high-quality, accessible, timely, reliable and disaggregated by income, sex, age, race, ethnicity, migration status, disability and *geographic location* [emphasis added] and other characteristics relevant in national contexts.

Disaggregation in space is equivalent to producing estimates for subnational areas. These can coincide with political/administrative boundaries or be effectively ‘continuously’ varying surfaces at a fine-grained spatial scale, e.g. a 1×1 km grid. National-level estimates are primarily useful for comparing nations and aggregating across large world regions, and hence their natural audience is international policy makers and donors. In contrast, subnational estimates shift focus away from the international to the national and subnational levels giving national and local policy makers and funders a useful new description of what is happening at the geographic and administrative levels where consequential decisions are typically made. Subnational variation in levels and trends can be revealed and addressed, and specific places with poor, satisfactory, or excellent levels or trends can be identified and targeted for improvement or used as exemplars.

Interest in small-area, subnational estimates of child mortality grew throughout the MDG era as it became clear that national-level estimates masked the heterogeneous progress of smaller geographical areas within countries. Early studies explored different methodological approaches to estimating the U5MR [9] and quantifying the effects of space [10, 11], while others demonstrated that a variety of data sources with geographical information can be used to learn about the subnational distribution of the U5MR—household surveys, most commonly USAID’s Demographic and Health Surveys (DHS); [9, 11–18] censuses; [10, 19] and fine-grained administrative data such as municipalities [20] and districts [21].

Most studies of subnational levels and trends in either the NMR or the U5MR reveal important variation in both. A common finding is that spatial variation persists after controlling for other determinants, and that the spatial effects often cross administrative/political boundaries, such as national borders, confirming that local conditions are important components of the risk of dying for young children [13, 19, 22–24]. While at the individual level, there are well known associations between child mortality and child, mother, household, and community variables (e.g., [25]), these variables are not available at all locations and universally available covariates usually have less strong associations [26, 27]. In a study of 28 African countries using DHS survey data, Burke and colleagues [12] find that 76 percent of the overall variation in the U5MR is accounted for by within-country variation. Working with data from Brazilian municipalities, Sousa and coworkers [20] find large differences in both the levels and rates of improvement of the NMR and the U5MR defined by geography and wealth, with the overall result that poorer, more rural communities are being ‘left behind’ and are progressively worse compared to wealthier urban areas. Likewise, Arku et al. [23] find that cross-district inequality in the U5MR increased over time as the overall U5MR fell. Quantifying geographic inequality in either levels or rates of improvement has been an important feature of many studies [15, 21]. In this vein De and Dhar [14] apply concentration curve approaches to the states of India and discover that the poorer states are more homogeneous, and Hosseinpoor and colleagues [16] test a variety of inequality measures and conclude that the majority lead to the same conclusions—spatial inequality appears robust to the common metrics used to measure it.

Two groups have produced comprehensive estimates of subnational child mortality for Africa that include spatially-defined covariates (we do not include a study of sub-Saharan Africa based on kernel density estimation [28] since this approach is exploratory rather than inferential.) Pezzulo et al. [18] use DHS data to estimate the ‘child mortality rate’, the probability of dying before age five for those who survive to age one, _4_q_1_, in 255 subnational areas defined by the DHS in 27 African countries. Using an area-based conditional autoregressive spatial model [29], the study investigates risk and protective factors expected to affect child mortality that include a range of geospatial covariates. Corroborating the earlier work done in single countries, the authors find important spatial variation net of the other predictors and identify maternal literacy as a key correlate of child mortality across Africa.

The Institute for Health Metrics and Evaluation (IHME) expand on this work to include a wide variety of additional data (235 survey and census data sets) and build a ‘continuous’ space model with 5×5 km resolution across 46 countries in Africa to estimate both the NMR and the U5MR [30]. This study reveals significant local and regional variation in both levels and rates of improvement in both indicators. The product of this work is a set of time-specific continuous surfaces of estimated NMR and U5MR with uncertainty. The authors conclude that many small localities and whole regions within Africa will have to sustain unrealistic rates of improvement in both the NMR and U5MR in order to reach the SDG 3.2 targets, and that their fine-grained spatial estimates provide valuable information to inform geographical targeting of interventions.

Both comprehensive studies of child mortality are limited in important ways. Household sample surveys are important data sources for both, but neither study accounts appropriately for the complex survey design that is used for the constituent surveys. Hence, the overall uncertainty of the space-time estimates is questionable. In a single-country study of Tanzania, we describe how this can be done [11].

Over the past few decades HIV has dramatically increased the mortality of reproductive-aged people in East and Southern Africa, and this leads to consequential bias in household sample survey estimates of both child and adult mortality. HIV spreads within families from spouse-to-spouse and mother-to-child which strongly correlates the risk of dying within families. The high-risk families are less likely to be included in household sample surveys because the mother is more likely to be dead and thus either missing from the sampling frame or unavailable to respond to the interview, and this leads to dramatic underestimates of mortality. Mortality measures derived from household sample surveys (e.g. DHS, UNICEF’s Multiple Indicator Cluster Surveys—MICS) for populations affected by HIV must correct for this bias. Neither Pezzulo et al. nor IHME do this, with the likely consequence that their estimates are too low in countries affected by HIV.

The IHME study is flawed in additional ways. When working with surveys with a complex design, such as the DHS, the model must acknowledge the design, and this is not straightforward for the continuous spatial model. As discussed in [27], the continuous spatial model is conceptually appealing because it allows inference at any level of aggregation, but in practice it requires far more fine-tuning and is less robust than the discrete spatial alternative. The modeling approach used by IHME does not allow inference in the sense that uncertainty in the covariate fitting procedure is not acknowledged in their uncertainty surfaces. The mortality of children of different ages is estimated separately using four models. The final U5MR is estimated from the fits of the four age models as if these are independent, but this is incorrect since each model fit is based on the same children. Finally, the IHME work utilizes non-standard and statistically unjustified approaches to estimating mortality from summary birth histories and calculating the overall standard error on their estimates.

In this paper we present direct, discrete space-time estimates of the U5MR in subnational geographic areas using data from DHS surveys conducted in Africa. We employ the approach we developed earlier [11] to fully account for survey design effects on the uncertainty in place-time-specific U5MR estimates. We combine place-time-specific, synthetic-cohort, direct estimates of the U5MR in a Bayesian space-time smoothing model with place, time, and place×time interactions to account for spatial and temporal correlation and identify separate place, time, and interaction effects. Using the space-time results, we identify spatial heterogeneity in levels and rates of decline and assess progress toward the MDG 4 targets for subnational and national areas annually from 1980–2019 and in five-year periods 1990–1994 to 2015–2019—we project U5MR to complete the series through 2019. Finally, we compare our national-level estimates to those produced by the UN-IGME [7] and IHME [30, 31].

## Methods

Additional details describing the data and methods are available in the supporting information file S1 File.

### Data sources

Data were drawn from the publicly available DHS surveys described in Table S1.1 in S1 File, the supporting information file: 122 surveys in 35 countries in Africa including 0⋅9 million children who contributed 156 million child-months of exposure. We selected every DHS survey in Africa except those that are not publicly available or those with data quality issues. See Table S1.2 in S1 File, the supporting information file, for a list of excluded countries. The DHS uses complex, multi-stage, cluster sample surveys that interview women aged 15–49 years. The details of the survey design vary across countries and DHS provides survey weights for each woman. Except for the early surveys, most identify the cluster where each woman was found, and some of the recent surveys have jittered GPS information. DHS surveys include a detailed birth history for each women that provides detailed information on the dates of birth and death for each of the women’s children. The birth history data were used to identify all completed or terminal child-months lived by every child reported by each adult female respondent. For each survey, data describing child-months were organized into a new dataset with one row per child month. Covariates defined for each child month included: an ID for the child; the date when the month started; the child’s age at the beginning of the month; a binary variable indicating if the child died during the month; the survey weight and sample information associated with the child; and geographic identifiers for the child’s household, if available. The national-level UN-IGME [32, 33] estimates are used to benchmark our final estimates.

### Statistical analysis

The approach we take, based on previous work [34, 35], is to model the direct estimate (in the language of Small Area Estimation (SAE), see [36]) of U5MR by decomposing the underlying mean of this estimate into space and time components. This discrete model approach has a long and successful history in spatial epidemiology [37, 38]. Statistical analysis proceeded in two steps as described by Mercer et al [11]. Full details can be found in the supporting information file S1 File.

First, discrete time survival analysis [39, 40] was used to estimate age-specific monthly probabilities of dying in age groups 0, 1–11, 12–23, 24–35, 36–47, and 48–59 completed months using weighted logistic regression. Following previous work [35], we picked one-year age groups with a separate age group for the first month, to account for the comparatively larger number of neonatal deaths. The weights account for the two-stage cluster design of the DHS surveys. The weight attached to each respondent reflects the sampling probability as well as a non-response adjustment. Consequently the variances associated with these estimates also reflect the survey design and the related uncertainty is propagated through to the estimates of the U5MR and its standard errors, which are the inputs to our smoothing model. For each survey, a separate weighted logistic regression model was estimated for each place and time period, resulting in survey-place-time-age-specific estimated monthly probabilities of dying with variance estimates that fully accounted for the specific design of the survey. Each model included child months that began in the specified time period at the specified place. Space was aggregated into Admin-1 geographical units and time into five-year periods from 1980–2014. Admin 0 and Admin 1 refer to administrative boundaries at the national level and the first sub-national level, respectively. The majority of DHS are designed so that there are sufficient samples to produce results that are representative at the Admin 1 level. Using a synthetic cohort approach and the standard life table definition of _5_q_0_, the estimated monthly probabilities of dying were used to construct estimates for the U5MR, $\hat{{}_{5}{\text{q}}_{0}}$, and the variance of each $\hat{{}_{5}{\text{q}}_{0}}$ was calculated via the delta method approach based on the estimated covariance matrices produced by the logistic regression models used to obtain the monthly probabilities of dying [11]. Because most countries have more than one DHS survey, during a given time period many places had U5MR estimates derived from multiple surveys. These were combined into a single place-time-specific $\hat{{}_{5}{\text{q}}_{0}}$ by constructing an inverse-variance weighted average of the survey-specific estimates—a standard meta-analysis approach (this operation is carried out on the logistic scale). Finally, to correct the bias caused by HIV, described above, we applied HIV prevalence-calibrated correction factors to the $\hat{{}_{5}{\text{q}}_{0}}$ as described by Walker et al [41].

In the second step a Bayesian space-time smoothing model was fit to the entire collection of place-time-specific $\hat{{}_{5}{\text{q}}_{0}}$ for each country. The objective of this step is to share information between near neighbors in both space and time to produce new estimates that are more stable and *smooth* in both space and time simultaneously, i.e., with smaller variance and higher signal to noise ratio. We require a model for the true _5_q_0_. So that the model can operate unconstrained over the range (−∞, ∞), we let λ_*it*_ = log[_5_q_0,*it*_/(1−_5_ *q*_0,*it*_)], where *i* indexes place and *t* time. On the logit scale, the model is:

$$\begin{array}{c} \lambda_{it}=\mu +\alpha_{t}+\gamma_{t}+\theta_{i}+\phi_{i}+\delta_{it}. \end{array}$$ (1)

Hence, the logit-transformed _5_q_0_ at each place and time are modeled as the sum of place and time-specific terms whose estimated distributions provide information about time trends (*γ*_*t*_) and random (unstructured) terms in time (*α*_*t*_), spatial trends (*ϕ*_*t*_) and random (unstructured) terms in space (*θ*_*i*_), and space-time structured interaction (*δ*_*it*_). The constant term (*μ*) captures the overall level. Hence, for each of space and time there are two components, one smooth (structured) and one unstructured, with each reflecting different sources of variation—the former being unobserved risk factors that vary smoothly and the latter random ‘shocks’ that are specific to that place or time only. Independent, unstructured variation in place (*θ*_*i*_) and time (*α*_*t*_) was identified by two terms independently drawn from normal distributions with mean 0 and variances estimated through the Bayesian inference setup; hence the amount of smoothing is estimated directly from the data, and uniquely for each country.

Because trends in time are likely to vary across spatial areas, a space-time interaction is needed. As with the main effects of time and space, these terms are not imposed on the fit, but rather the data choose the level of interaction required (the results show this, with some countries exhibiting very little interaction). We expect some smoothness in time and space for the interaction terms, and this is encoded in the form we assume for the interaction. This is particularly useful for predictions because we obtain area-specific temporal trends, leaning on recent trends in the observed data in those areas.

Temporal and spatial smoothing were conducted using two additional place (*ϕ*_*i*_) and time (*γ*_*t*_) specific terms. The space term was modeled using an intrinsic conditional autoregressive (ICAR) model [42], a generalization of the random walk of order 1 (RW1) model to space. Similarly, the time term was modeled using a random walk of order 2 (RW2) model at one-year intervals. In RW1 models each value depends on its nearest neighbors, and in RW2 models each value depends on both its nearest neighbors and those neighbors’ nearest neighbors. Since they are based on a greater number of neighbors, RW2 models generally encourage more smoothing. The interaction of time and place (*δ*_*it*_) was modeled assuming that the temporal (RW2) and spatial (ICAR) structured effects interact [43]. Consequently, the interactions have structure in time and space. In our setup each logit-transformed value depends on its nearest neighbors in space and its nearest two neighbors in both the past and future along the time axis. In what follows this model is referred to as the ‘subnational model’.

In order to compare our time trend results to those produced by other organizations at the *national level* [7, 31], we fit an analogous, separate model that ignores space by dropping the three spatial terms from Eq (1). This model is effectively a national-level model for the time trend, and in what follows it is referred to as the ‘national model’. The weighted estimates are benchmarked so that at the national level they closely follow those of UN-IGME [32, 33], see the supporting information file S1 File.

Analysis was conducted in the R statistical programming environment [44]. In the first step the logistic regression models were fit using the svyglm function in the survey [45] package. In the second step the space-time smoothing models were fit using the integrated nested Laplace approximation (INLA) [46] method implemented in the R-INLA package [47]. Running Microsoft’s version of R (Microsoft R Open v3.4.2 [48]) on an Apple Macbook Pro laptop computer (mid-2012 model, 2⋅6 GHz Intel Core i7 CPU, 8 GB 1600 MHz DDR3 RAM) and starting with data reshaped into child-months, calculations in each step require about 2⋅5 hours, or about 5 hours in total, for all 35 countries.

The MDG time frame is the twenty-five years between 1990–2015. Our analysis is organized in one- and five-year periods from 1980 to whenever the most recent data are available, usually 2010-2016. In order to replicate the MDG period, we made a probabilistic projection of the U5MR through 2019 using the results from our fitted models. The temporal behavior of this projection at the national level is governed by the one-year time scale RW2 model. We calculated MDG metrics using the annual estimates for 1990 and projected values for 2015.

The results we present include summaries of the estimated distributions of U5MR by place and time for each country. Using those we calculate proportional rates of decline in U5MR at both national and subnational levels to identify geographic regions that reached the MDG 4 target of 67 percent reduction in U5MR. We also present the fraction of overall variance explained by each of the terms in Eq (1). Those values indicate how important the space, time, interaction and independent effects are in producing the space-time variation in the U5MR.

### Data sharing

All data are from publicly available Demographic and Health Surveys (DHS), available at https://dhsprogram.com/data/. The software we developed for this study is available as an open source package for the R statistical programming environment, available at https://cran.r-project.org/package=SUMMER [49]. All of the R code used to produce the results described in this article is available at https://github.com/richardli/AfricaU5MR.

## Results

The supporting information file S1 File contains a wide range of results that are not included or described here, including s set of large tables containing all of the numerical estimates for each country and subnational region through time, along with comparable estimates from other organizations. Throughout this section, grey shading on maps indicates countries where estimates were not produced.

### Space and time heterogeneity

Recall that the space-time smoothing model in Eq (1) is fit for each country separately on a dataset of $\hat{{}_{5}{\text{q}}_{0}}$ created using all of the DHS surveys with geographic information for each country. For each country, Table 1 contains the fraction of overall variability in (the logit of) $\hat{{}_{5}{\text{q}}_{0}}$ explained by each of the terms in the space-time model. The ‘RW2’ column corresponds to time trends (structured temporal); ‘ICAR’ to spatial trends (structured spatial); ‘Space S’ (unstructured spatial), ‘Time T’ (unstructured temporal), and ‘RW2×ICAR’ (structured space-time interaction). Note that the percentages are with respect to the spatial (Admin-1) and temporal (yearly) scales at which we fit the model. So, for example, we cannot discern the level of spatial variability below the Admin-1 level with this analysis.

**Table 1:** Variance component proportions (percent).

Country	RW2 $\sigma_{\gamma }^{2}$	ICAR $\sigma_{\phi }^{2}$	RW2×ICAR $\sigma_{\delta }^{2}$	Unstructured Effects	
				Space (S)$\sigma_{\theta }^{2}$	Time (T) $\sigma_{\alpha }^{2}$
Angola	6⋅0	56⋅1	36⋅3	1⋅4	0⋅3
Benin	65⋅5	27⋅4	3⋅8	2⋅7	0⋅5
Burkina Faso	56⋅9	30⋅9	8⋅7	3⋅0	0⋅6
Burundi	47⋅5	35⋅6	14⋅7	1⋅8	0⋅4
Cameroon	29⋅2	65⋅3	2⋅7	2⋅4	0⋅4
Chad	36⋅9	41⋅4	17⋅7	3⋅3	0⋅6
Comoros	64⋅9	16⋅0	17⋅0	1⋅6	0⋅5
Congo	72⋅0	13⋅5	11⋅4	2⋅4	0⋅6
Côte d’Ivoire	25⋅7	54⋅8	16⋅7	2⋅5	0⋅4
DRC	53⋅9	28⋅1	15⋅4	2⋅1	0⋅4
Egypt	80⋅2	14⋅9	3⋅7	0⋅9	0⋅2
Ethiopia	70⋅8	24⋅6	3⋅4	1⋅0	0⋅2
Gabon	51⋅4	30⋅4	13⋅6	3⋅7	0⋅8
Gambia	66⋅2	18⋅6	14⋅0	0⋅9	0⋅2
Ghana	56⋅5	34⋅5	5⋅9	2⋅6	0⋅5
Guinea	62⋅7	31⋅5	4⋅6	1⋅1	0⋅2
Kenya	31⋅5	48⋅1	18⋅4	1⋅7	0⋅3
Lesotho	28⋅0	28⋅2	38⋅1	4⋅6	1⋅1
Liberia	84⋅2	6⋅9	7⋅3	1⋅3	0⋅3
Madagascar	72⋅7	16⋅2	9⋅4	1⋅4	0⋅3
Malawi	87⋅0	11⋅1	0⋅6	1⋅0	0⋅2
Mali	42⋅8	50⋅4	5⋅4	1⋅2	0⋅2
Morocco	83⋅0	8⋅8	7⋅0	1⋅0	0⋅2
Mozambique	65⋅2	21⋅8	11⋅8	1⋅0	0⋅2
Namibia	44⋅5	32⋅5	20⋅2	2⋅2	0⋅6
Niger	57⋅1	30⋅9	10⋅3	1⋅4	0⋅3
Nigeria	26⋅7	65⋅4	5⋅7	1⋅9	0⋅3
Rwanda	83⋅7	12⋅5	2⋅5	1⋅0	0⋅2
Senegal	72⋅5	23⋅0	3⋅0	1⋅2	0⋅2
Sierra Leone	59⋅4	24⋅7	13⋅1	2⋅3	0⋅5
Tanzania	75⋅8	17⋅5	5⋅3	1⋅2	0⋅2
Togo	53⋅4	39⋅9	3⋅6	2⋅5	0⋅5
Uganda	87⋅1	8⋅7	2⋅7	1⋅3	0⋅3
Zambia	75⋅0	19⋅2	3⋅8	1⋅6	0⋅3
Zimbabwe	45⋅2	44⋅3	7⋅7	2⋅3	0⋅5
Mean	57⋅7	29⋅5	10⋅4	1⋅9	0⋅4

Across all countries the time term (RW2) accounts for an average of 58 percent of the total variability. This indicates that most countries have experienced important, consistent temporal trends—consistently down except for some areas in Angola, Côte d’Ivoire, Lesotho, and Zimbabwe, see Table 2. The variability captured by the structured space term (ICAR) is also consistently important accounting for an average of 30 percent of total variability. The distribution of values in the ICAR column suggests that countries fall into one of two groups: the majority where space plays an important role in explaining the U5MR, and a few where space appears to be insignificant, accounting for only a few percent of total variability—Liberia, Morocco, and Uganda.

**Table 2:** Subnational MDG 4 target achievement.

Country	Regions Achieved	Percent Achieved	Median Reduction	Reduction Range
Angola	1/18	5⋅6	0⋅098	[-0⋅63, 0⋅88]
Benin	0/6	0⋅0	0⋅465	[0⋅38, 0⋅57]
Burkina Faso	0/4	0⋅0	0⋅599	[0⋅41, 0⋅65]
Burundi	1/5	20⋅0	0⋅637	[0⋅44, 0⋅69]
Cameroon	0/5	0⋅0	0⋅367	[0⋅22, 0⋅43]
Chad	0/8	0⋅0	0⋅353	[0⋅08, 0⋅53]
Comoros	0/3	0⋅0	0⋅335	[0⋅10, 0⋅41]
Congo	1/4	25⋅0	0⋅621	[0⋅39, 0⋅69]
Côte d’Ivoire	0/11	0⋅0	0⋅323	[-0⋅01, 0⋅59]
DRC	1/11	9⋅1	0⋅501	[0⋅27, 0⋅78]
**Egypt**	2/4	50⋅0	0⋅608	[0⋅51, 0⋅74]
**Ethiopia**	10/11	90⋅9	0⋅711	[0⋅58, 0⋅80]
Gabon	0/5	0⋅0	0⋅364	[0⋅14, 0⋅52]
Gambia	4/6	66⋅7	0⋅673	[0⋅36, 0⋅82]
Ghana	0/8	0⋅0	0⋅568	[0⋅35, 0⋅61]
Guinea	3/5	60⋅0	0⋅699	[0⋅40, 0⋅73]
Kenya	3/8	37⋅5	0⋅509	[0⋅04, 0⋅75]
Lesotho	0/10	0⋅0	0⋅195	[-0⋅44, 0⋅46]
**Liberia**	4/5	80⋅0	0⋅748	[0⋅45, 0⋅78]
**Madagascar**	6/6	100⋅0	0⋅804	[0⋅69, 0⋅89]
**Malawi**	3/3	100⋅0	0⋅717	[0⋅71, 0⋅73]
Mali	1/4	25⋅0	0⋅617	[0⋅46, 0⋅74]
**Morocco**	4/7	57⋅1	0⋅714	[0⋅56, 0⋅81]
**Mozambique**	6/11	54⋅6	0⋅679	[0⋅25, 0⋅80]
Namibia	1/13	7⋅7	0⋅443	[0⋅03, 0⋅68]
**Niger**	3/6	50⋅0	0⋅706	[0⋅47, 0⋅84]
Nigeria	0/6	0⋅0	0⋅466	[0⋅22, 0⋅58]
**Rwanda**	5/5	100⋅0	0⋅789	[0⋅71, 0⋅80]
**Senegal**	6/11	54⋅6	0⋅704	[0⋅59, 0⋅76]
Sierra Leone	0/4	0⋅0	0⋅554	[0⋅33, 0⋅66]
**Tanzania**	16/20	80⋅0	0⋅755	[0⋅55, 0⋅85]
Togo	0/6	0⋅0	0⋅449	[0⋅35, 0⋅55]
**Uganda**	3/4	75⋅0	0⋅711	[0⋅67, 0⋅74]
Zambia	4/9	44⋅4	0⋅658	[0⋅60, 0⋅76]
Zimbabwe	0/10	0⋅0	0⋅032	[-0⋅14, 0⋅34]

*Note*: Countries that achieved the MDG 4 target at the national level based on results from the national model are listed in boldface type; countries where no subnational region achieved the target are shaded in red; and countries where all subregions achieved the goal are shaded in green. “Reduction Range” is based on the minimum and maximum of the subnational reductions in each country.

**Fig 1:**
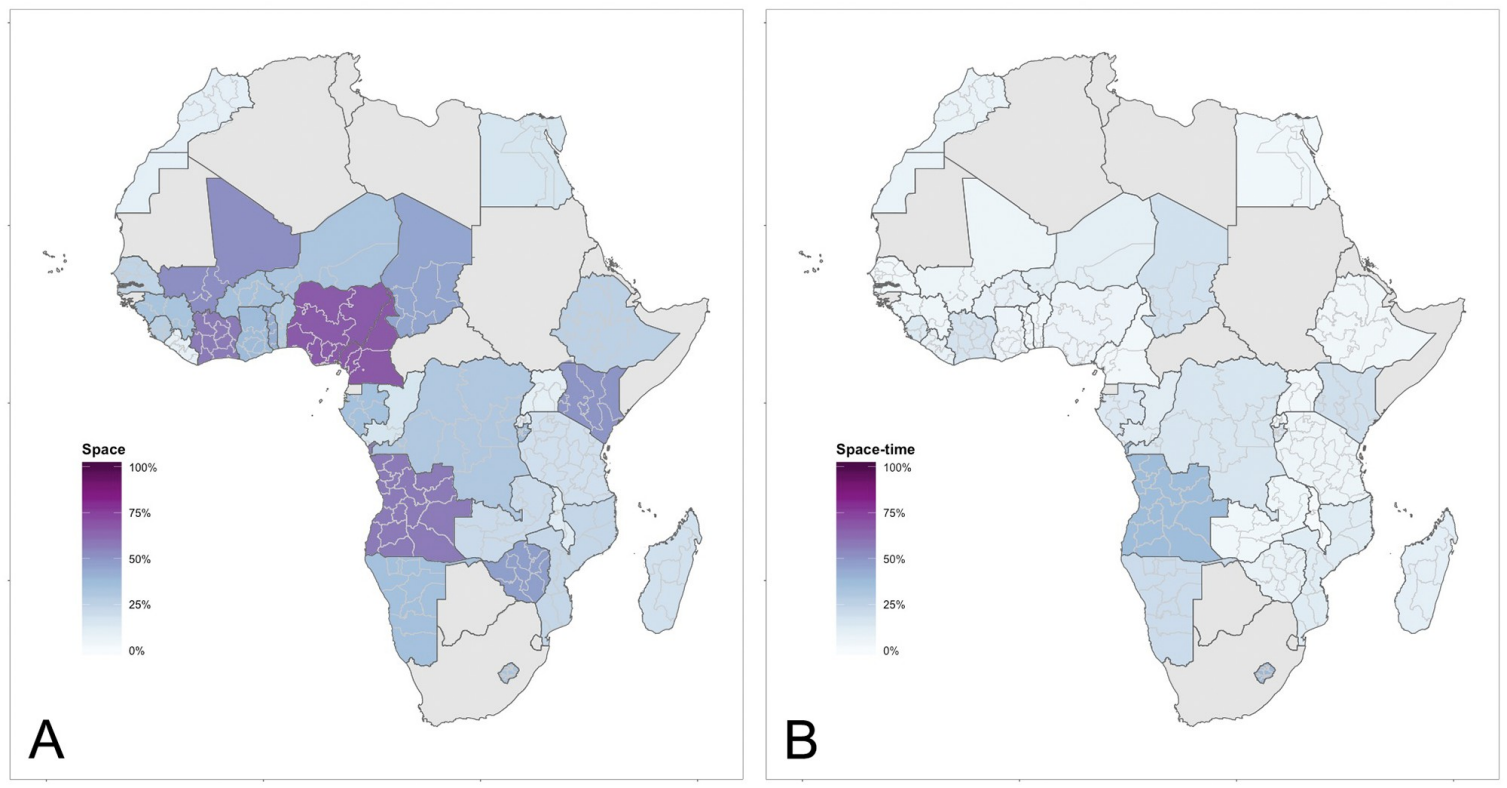
**Africa:** Proportion of variation explained by effects involving space. A: Spatial Variation. B: Space×time interaction variation.

Fig 1 displays the proportion of variation explained by the spatial terms. Fig 1A corresponds to the structured spatial (ICAR) and independent spatial (S) variation, given by the sum of the posterior marginal variance of *θ*_*i*_ and *ϕ*_*i*_ in Eq (1). The West Africa region has more spatial structure in general compared to the Central, East and Southern Africa regions, except for Kenya, Angola, and Zimbabwe. The unstructured effects contribute much less to total variation, with the unstructured spatial effects generally slightly more important compared to the unstructured time effects, on average 1⋅9 percent compared to 0⋅4 percent.

The time×space interaction (RW2×ICAR) explains on average 10⋅4 percent of overall variation, but there is substantial variability from a low of 0⋅6 percent to a high of 38⋅1 percent. Fig 1B displays this and confirms that there is no clear regional patterning of the space time interaction. A number of countries have space time interaction values around 15–20 percent, and three countries have particularly high values around 30 percent: Angola, Lesotho, and Namibia. This suggests that specific regions within these countries had unusual values during specific time periods that required an additional effect beyond the main effects of space (applies to each region at all times) and time (applies to each time period at all regions). These unusual regions did not change like the other regions in their country, and it may be substantively interesting to further investigate these countries and determine exactly which regions were involved and what was happening to them.

The supporting information file S1 File contains a wide range of additional subnational results for each country: (i) smoothed regional estimates of the U5MR through time, (ii) unsmoothed, direct regional estimates from each DHS survey, (iii) a comparison of the smoothed versus unsmoothed regional direct estimates, (iv) one-year and five-year estimates by regions, and (v) cross-validation results.

### Assessment of MDG 4 progress in subnational regions

#### Numerical assessment of progress on MDG 4

Table 2 contains a national-level summary of progress toward the MDG 4 target for *the subnational regions within each country*. The first two columns contain the estimated number of regions that achieved the target compared to the number of regions in total and that fraction as a percent. Three countries achieved the target reduction in all regions, nineteen countries achieved the target in some but not all regions, and thirteen countries did not achieve the target in any regions.

The last two columns in Table 2 summarize the proportional reductions achieved by the regions within each country, the median, and minimum to maximum range of those values. The countries that achieved the target as a whole generally had comfortable median regional reductions of 75 percent or more. For countries that did not achieve the goal, regional performance was mixed and highly variable, ranging from only one region (several countries) to a majority (Ethiopia) of regions achieving the target and median reductions of 9⋅8 to 75⋅5 percent. In countries where no subregions achieved the target, performance was generally poor with median regional reductions from 3⋅2 (Zimbabwe) to 59⋅9 (Burkina Faso) percent.

#### Visual comparison of 2015 to 1990

**Fig 2:**
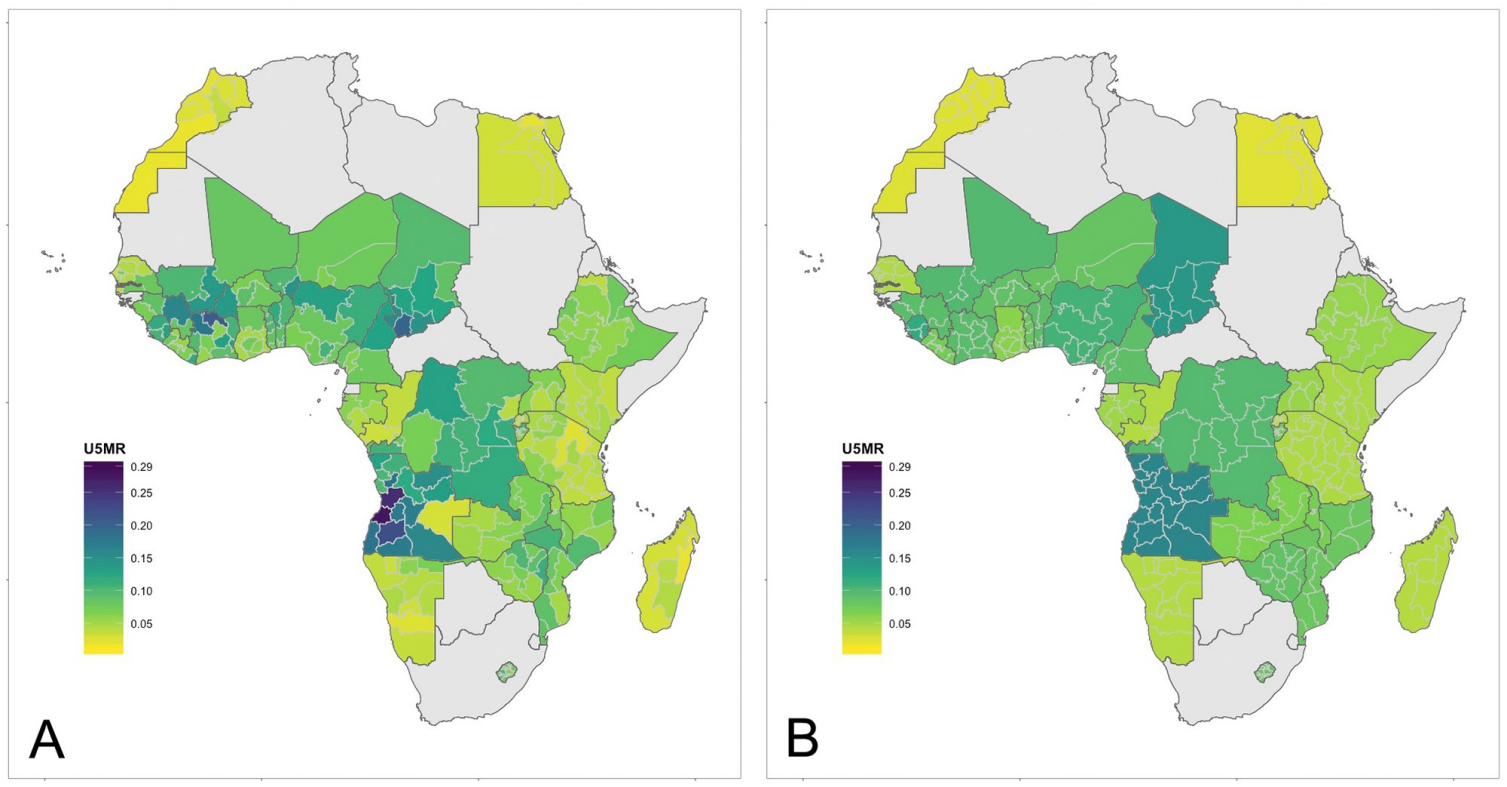
Projected U5MR in 2015. Green colors from 0⋅01 to 0⋅19. A: Projection using subnational model. B: Projection using national model.

Fig 2A and 2B show the projected U5MR in 2015 using the subnational and national models. There is a clear regional pattern to these values with higher levels in Central and West Africa and significant spatial heterogeneity within a number of countries.

**Fig 3:**
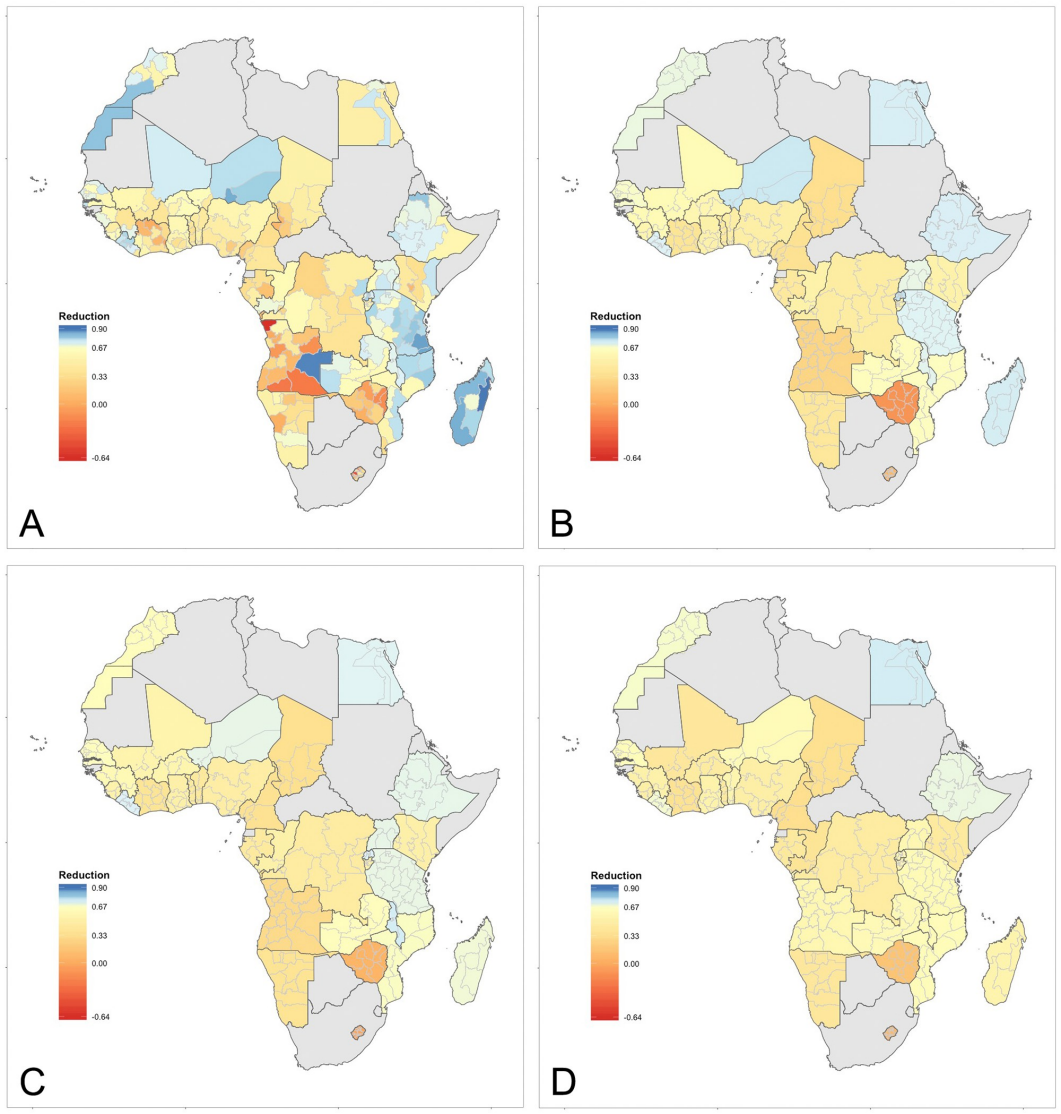
Reduction in the U5MR between 1990 and 2015. The top row displays our estimates. Blue colors reach the target level of 67%; yellow-red do not; scale from -0⋅64–0⋅9. A: Subnational model. B: National model. C: UN-IGME B-3 estimates [32]. D: IHME GBD estimates [31].

Fig 3A and 3B display reductions in the U5MR between 1990 and 2015 from the subnational and national models. The overall regional patterning persists with larger reductions in areas with lower U5MR in 2015—East, Southern and parts of North Africa. Within countries that achieved the MDG 4 target reductions at the national level, there is some heterogeneity across Admin-1 areas in the overall level of reduction—Egypt, Ethiopia, Liberia, Madagascar, Malawi, Morocco, Mozambique, Niger, Rwanda, Senegal, Tanzania, and Uganda. For countries that did not achieve the target nationally and have mixed performance at the Admin-1 level, there is pronounced regional heterogeneity—Angola, Burundi, Congo, DRC, Gambia, Guinea, Kenya, Mali, Mozambique, Namibia, Senegal, and Zambia. The remaining countries did not achieve the target nationally and had no subregions that achieved the target, and for most of these there appears to be less variability in the rate of reduction, with all Admin-1 regions doing poorly together. The pictures in these figures visually confirm the story told in Table 1. The supporting information file S1 File contains maps of regional reductions in U5MR through time for each country.

### Comparison to published estimates at national level

Both UN-IGME [32] and IHME [31] have produced national-level estimates of the U5MR over time. Fig 3 displays the overall reductions in the U5MR for the period 1990 to 2015 estimated by us at the subnational (Fig 3A) and national (Fig 3B) levels and the UN-IGME (Fig 3C) and IHME (Fig 3D) estimates at the national level. The national-level figures are all qualitatively similar, although we estimate a slightly worse improvement in Zimbabwe. The supporting information file S1 File contains national-level estimates using both RW1 and RW2 specifications for the temporal model compared to estimates from both UN-IGME [7] and IHME [31].

## Discussion

Our findings reveal a wide variety of subnational levels and trends in the U5MR in Africa. At the broadest level the U5MR has been falling consistently almost everywhere, but the rate of change and most recent levels vary considerably. In large regions, especially West and Central (Fig 2A and 2B), the U5MR is still high and recent reductions missed the MDG 4 target (Fig 3A and 3B). Unlike the finding of Sousa et al. [20], subnational heterogeneity in Africa is larger in areas with higher U5MR where reductions have been modest. In East and Southern Africa where the overall U5MR is lower, spatial variability is also generally lower.

We carried out extensive cross-validation model checks that are summarized in the supporting information file S1 File. Specifically, we systematically held out data (the weighted estimates) from Admin-1 areas and time periods, and then predicted these data using the model fitted to the remaining data. A variety of checks were then performed for each country: coverage of 95% interval estimates; examination of bias against time to look for systematic model failure. The checks did not reveal any problems with the models overall. For example, the coverage was 95% on average, with all but 2 countries having coverages between 89 and 100 percent.

Our results do not produce a generalizable relationship between the level and rate of change of the U5MR and subnational variation. Based on this it will be important to consider each country by itself and use subnational estimates to motivate and guide better understanding of the local risk and protective factors affecting the risk of dying for young children, so that locally-relevant interventions can be targeted to specific regional populations. This will allow for the possibility that overall interventions strategies are more cost effective, feasible (operate in smaller geographic areas), and hopefully because of all of that, more effective.

Our overall modeling approach has several advantages: (i) in comparison to other work using similar data to produce subnational estimates of the U5MR [18, 30], our approach is relatively straightforward so that it runs quickly on everyday computers, can be explained easily, and its results readily understood; (ii) it transparently acknowledges the sample design of each complex survey so that design-induced uncertainty is propagated through to our final estimates and the estimates are correctly adjusted to account for the complex sampling design of the DHS studies; (iii) it adjusts for the bias created by HIV epidemics in survey-based measures of mortality; (iv) it is based on well-understood space-time interaction [43] Gaussian Markov Random Field (GMRF) models [50] that have been used extensively in spatial epidemiology; and (v) the method has been validated in the statistics community [11] which provides users with confidence that the statistical methodology is sound. Finally, we provide a freely available, open source package for the R statistical programming environment (SUMMER: Spatio-Temporal Under-Five Mortality Methods for Estimation in R) to implement the method so that anyone can replicate our work or conduct a similar analysis using household survey data [49].

This work also has limitations: (i) foremost, we only make use of data from DHS surveys which limits the number of countries, time periods, and geographic resolution of our work; (ii) we do not make direct use of summary birth history data which excludes census data sources and some surveys, and prevents us from accessing the much finer geographic scale that is possible with census data; (iii) we do not make estimates at geographic scales below the Admin-1 level, and this potentially prevents us from identifying and characterizing important spatial heterogeneity at sub-Admin-1 levels; (iv) we do not include spatial covariates that may allow the space-time models to be more accurate and reveal more spatial and temporal structure; (v) we only produce estimates for the U5MR rather than a variety of mortality indicators pertaining to other age groups, such as the NMR and various age-specific rates covering all ages; (vi) we do not consider the effects of within-country; (vii) we have not incorporated covariates; and (viii) we do not attempt to incorporate cause of death to produce estimates of cause-specific mortality rates, something suggested by the SDG 3.2 target that calls for elimination of all *preventable* child deaths; ‘preventable’ in this sense presumably requiring categorization of deaths by cause. Our current work is addressing these limitations.

## Supporting information

S1 File Detailed data, methods, and results. The supporting information file is a PDF document that contains a full and detailed presentation of the data, methods, and all results in numerical form, including the complete validation study.
(PDF)

## References

[1] Mikkelsen L, Phillips DE, AbouZahr C, Setel PW, De Savigny D, Lozano R, et al A global assessment of civil registration and vital statistics systems: monitoring data quality and progress. The Lancet. 2015;386(10001):1395–1406. 10.1016/S0140-6736(15)60171-4 25971218

[2] Li N. Estimating Life Tables for Developing Countries. https://goo.gl/N2fMZx: United Nations Department of Economic and Social Affairs Population Division; 2015. 2014/4.

[3] United Nations General Assembly. Keeping the promise: united to achieve the Millennium Development Goals; 2010. Resolution Adopted by the General Assembly: 65/1.https://goo.gl/GK2AhU.

[4] United Nations. Millennium Development Goals; 2017.

[5] United Nations General Assembly. Transforming our world: the 2030 Agenda for Sustainable Development; 2015. Resolution Adopted by the General Assembly on 25 September 2015: 70/1.https://goo.gl/UBddEC.

[6] United Nations. Sustainable Development Goals; 2017.

[7] You D, Hug L, Ejdemyr S, Idele P, Hogan D, Mathers C, et al Global, regional, and national levels and trends in under-5 mortality between 1990 and 2015, with scenario-based projections to 2030: a systematic analysis by the UN Inter-agency Group for Child Mortality Estimation. The Lancet. 2015;386(10010):2275–2286. 10.1016/S0140-6736(15)00120-8 26361942

[8] United Nations. Sustainable development knowledge platform: transforming our world: the 2030 agenda for sustainable development; 2017.

[9] Dwyer-Lindgren L, Kakungu F, Hangoma P, Ng M, Wang H, Flaxman AD, et al Estimation of district-level under-5 mortality in Zambia using birth history data, 1980–2010. Spatial and Spatio-Temporal Epidemiology. 2014;11:89–107. 10.1016/j.sste.2014.09.002 25457599

[10] Kazembe L, Clarke A, Kandala NB. Childhood mortality in sub-Saharan Africa: cross-sectional insight into small-scale geographical inequalities from Census data. BMJ Open. 2012;2(5):e001421 10.1136/bmjopen-2012-001421 23089207 PMC3488715

[11] Mercer LD, Wakefield J, Pantazis A, Lutambi AM, Masanja H, Clark S. Space-time smoothing of complex survey data: small area estimation for child mortality. The Annals of Applied Statistics. 2015;9(4):1889–1905. 10.1214/15-AOAS872 27468328 PMC4959836

[12] Burke M, Heft-Neal S, Bendavid E. Sources of variation in under-5 mortality across Sub-Saharan Africa: a spatial analysis. The Lancet Global Health. 2016;4(12):e936–e945. 10.1016/S2214-109X(16)30212-1 27793587

[13] Chin B, Montana L, Basagaña X. Spatial modeling of geographic inequalities in infant and child mortality across Nepal. Health & Place. 2011;17(4):929–936. 10.1016/j.healthplace.2011.04.006 21555234

[14] De P, Dhar A. Inequality in child mortality across different states of India: a comparative study. Journal of Child Health Care. 2013;17(4):397–409. 10.1177/1367493512468359 23435164

[15] Hodge A, Firth S, Marthias T, Jimenez-Soto E. Location matters: trends in inequalities in child mortality in Indonesia. Evidence from repeated cross-sectional surveys. PloS One. 2014;9(7):e103597 10.1371/journal.pone.0103597 25061950 PMC4111602

[16] Hosseinpoor AR, Bergen N, Barros AJ, Wong KL, Boerma T, Victora CG. Monitoring subnational regional inequalities in health: measurement approaches and challenges. International Journal for Equity in Health. 2016;15(1):18 10.1186/s12939-016-0307-y 26822991 PMC4730638

[17] Jimenez-Soto E, Durham J, Hodge A. Entrenched geographical and socioeconomic disparities in child mortality: trends in absolute and relative inequalities in Cambodia. PloS One. 2014;9(10):e109044 10.1371/journal.pone.0109044 25295528 PMC4189958

[18] Pezzulo C, Bird T, Utazi EC, Sorichetta A, Tatem AJ, Yourkavitch J, et al. Geospatial modeling of child mortality across 27 countries in Sub-Saharan Africa. USAID, DHS Program; 2016. 13. Available from: https://goo.gl/AMk6zC.

[19] Bauze AE, Tran LN, Nguyen KH, Firth S, Jimenez-Soto E, Dwyer-Lindgren L, et al Equity and geography: the case of child mortality in Papua New Guinea. PLoS One. 2012;7(5):e37861 10.1371/journal.pone.0037861 22662238 PMC3360603

[20] Sousa A, Hill K, Dal Poz MR. Sub-national assessment of inequality trends in neonatal and child mortality in Brazil. International Journal for Equity in Health. 2010;9(1):21 10.1186/1475-9276-9-21 20815875 PMC2944212

[21] Ram U, Jha P, Ram F, Kumar K, Awasthi S, Shet A, et al Neonatal, 1–59 month, and under-5 mortality in 597 Indian districts, 2001 to 2012: estimates from national demographic and mortality surveys. The Lancet Global Health. 2013;1(4):e219–e226. 10.1016/S2214-109X(13)70073-1 25104347

[22] Amouzou A, Kozuki N, Gwatkin DR. Where is the gap?: the contribution of disparities within developing countries to global inequalities in under-five mortality. BMC Public Health. 2014;14(1):216 10.1186/1471-2458-14-216 24581032 PMC3945794

[23] Arku RE, Bennett JE, Castro MC, Agyeman-Duah K, Mintah SE, Ware JH, et al Geographical inequalities and social and environmental risk factors for under-five mortality in Ghana in 2000 and 2010: bayesian spatial analysis of census data. PLoS Medicine. 2016;13(6):e1002038 10.1371/journal.pmed.1002038 27327774 PMC4915620

[24] Bhutta ZA. Mapping the geography of child mortality: a key step in addressing disparities. The Lancet Global Health. 2016;4(12):e877–e878. 10.1016/S2214-109X(16)30264-9 27793588

[25] Balk D, Pullum T, Storeygard A, Greenwell F, Neuman M. A spatial analysis of childhood mortality in West Africa. Population, Space and Place. 2004;10:175–216. 10.1002/psp.328

[26] Golding N, Burstein R, Longbottom J, Browne AJ, Fullman N, Osgood-Zimmerman A, et al Mapping under-5 and neonatal mortality in Africa, 2000–15: a baseline analysis for the Sustainable Development Goals. The Lancet. 2017;. 10.1016/S0140-6736(17)31758-0 PMC5687451 28958464

[27] Wakefield J, Fuglstad GA, Riebler A, Godwin J, Wilson K, Clark SJ. Estimating under five mortality in space and time in a developing world context. Statistical Methods in Medical Research. 2018;. 10.1177/0962280218767988 29671377 PMC6599729

[28] Burke M, Heft-Neal S, Bendavid E. Sources of variation in under-5 mortality across sub-Saharan Africa: a spatial analysis. The Lancet Global Health. 2016;4:e936–e945. 10.1016/S2214-109X(16)30212-1 27793587

[29] Leroux BG, Lei X, Breslow N. Estimation of disease rates in small areas: A new mixed model for spatial dependence In: Halloran ME, Berry DA, editors. Statistical Models in Epidemiology, the Environment and Clinical Trials. New York: Springer; 1999 p. 179–192.

[30] Golding N, Burstein R, Longbottom J, Browne AJ, Fullman N, Osgood-Zimmerman A, et al Mapping under-5 and neonatal mortality in Africa, 2000–15: a baseline analysis for the Sustainable Development Goals. The Lancet. 2017;390(10108):2171–2182. 10.1016/S0140-6736(17)31758-0 PMC5687451 28958464

[31] GBD 2015 Child Mortality Collaborators. Global, regional, national, and selected subnational levels of stillbirths, neonatal, infant, and under-5 mortality, 1980-2015: a systematic analysis for the Global Burden of Disease Study 2015. The Lancet. 2016;388(10053):1725–1774. 10.1016/S0140-6736(16)31575-6 PMC5224696 27733285

[32] United Nations Inter-agency Group for Child Mortality Estimation (UN IGME). Levels & trends in child mortality: report 2017. Estimates developed by the UN Inter-agency Group for Child Mortality Estimation. New York: United Nations Children’s Fund; 2017.

[33] Alkema L, New JR, et al Global estimation of child mortality using a Bayesian B-spline bias-reduction model. The Annals of Applied Statistics. 2014;8(4):2122–2149. 10.1214/14-AOAS768

[34] Chen C, Wakefield J, Lumley T. The use of sample weights in Bayesian hierarchical models for small area estimation. Spatial and Spatio-Temporal Epidemiology. 2014;11:33–43. 10.1016/j.sste.2014.07.002 25457595 PMC4357363

[35] Mercer L, Wakefield J, Pantazis A, Lutambi A, Mosanja H, Clark S. Small area estimation of childhood of childhood mortality in the absence of vital registration. Annals of Applied Statistics. 2015;9:1889–1905. 27468328 10.1214/15-AOAS872 PMC4959836

[36] Rao JNK, Molina I. Small Area Estimation, Second Edition New York: John Wiley; 2015.

[37] Elliott P, Wakefield JC, Best NG, Briggs DJ. Spatial Epidemiology: Methods and Applications. Oxford: Oxford University Press; 2000.

[38] Waller LA, Gotway CA. Applied Spatial Statistics for Public Health Data. John Wiley and Sons; 2004.

[39] Allison PD. Discrete-time methods for the analysis of event histories. Sociological Methodology. 1982;13:61–98. 10.2307/270718

[40] Allison PD. Event history and survival analysis: Regression for longitudinal event data. vol. 46 SAGE Publications; 2014.

[41] Walker N, Hill K, Zhao F. Child mortality estimation: methods used to adjust for bias due to AIDS in estimating trends in under-five mortality. PLoS Medicine. 2012;9(8):e1001298 10.1371/journal.pmed.1001298 22952437 PMC3429377

[42] Besag J, York J, Mollié A. Bayesian image restoration with two applications in spatial statistics. Annals of the Institute of Statistics and Mathematics. 1991;43:1–59. 10.1007/BF00116466

[43] Knorr-Held L. Bayesian modelling of inseparable space-time variation in disease risk. Statistics in Medicine. 2000;19:2555–2567. 10.1002/1097-0258(20000915/30)19:17/18%3C2555::AID-SIM587%3E3.0.CO;2-%23 10960871

[44] R Core Team. R: a language and environment for statistical computing; 2017. Available from: https://www.R-project.org/.

[45] Lumley T. survey: analysis of complex survey samples; 2018. Available from: https://cran.r-project.org/package=survey.

[46] Rue H, Martino S, Chopin N. Approximate Bayesian inference for latent Gaussian models using integrated nested Laplace approximations (with discussion). Journal of the Royal Statistical Society, Series B. 2009;71:319–392. 10.1111/j.1467-9868.2008.00700.x

[47] Rue H, et al. R-INLA; 2017. Available from: http://www.r-inla.org.

[48] Microsoft. Microsoft R Open: The Enhanced R Distribution; 2017. Available from: https://mran.microsoft.com/open/.

[49] Martin BD, Li ZR, Hsiao Y, Godwin J, Wakefield J, Clark SJ. SUMMER: Spatio-Temporal Under-Five Mortality Methods for Estimation; 2018. Available from: https://CRAN.R-project.org/package=SUMMER.

[50] Rue H, Held L. Gaussian Markov random fields: theory and application. Boca Raton: Chapman and Hall/CRC Press; 2005.

