# Supplementary material for: Changes in the spatial distribution of the under-five mortality rate: Small-area analysis of 122 DHS surveys in 262 subregions of 35 countries in Africa
Source: PLoS One. 2019 Jan 22;14(1):e0210645. doi: 10.1371/journal.pone.0210645 (PMC6342310; doi:10.1371/journal.pone.0210645)
Supplement: S1 File — The supporting information file is a PDF document that contains a full and detailed presentation of the data, methods, and all results in numerical form, including the complete validation study. (PDF) [file pone.0210645.s001.pdf]

Supporting Information for “Changes in the Spatial Distribution of the Under-Five Mortality Rate: Small-area Analysis of 122 DHS Surveys in 262 Subregions of 35 Countries in Africa”

Zehang Li[1], Yuan Hsiao[2,4], Jessica Godwin[2], Bryan D. Martin[2], Jon Wakefield[2,3], Samuel J. Clark[5,6,*], with support from the United Nations Inter-agency Group for Child Mortality Estimation and its technical advisory group[7]

[1]Department of Biostatistics, Yale School of Public Health, New Haven, Connecticut, USA
[2]Department of Statistics, University of Washington, Seattle, Washington, USA
[3]Department of Biostatistics, University of Washington, Seattle, Washington, USA
[4]Department of Sociology, University of Washington, Seattle, Washington, USA
[5]Department of Sociology, The Ohio State University, Columbus, Ohio, USA
[6]MRC/Wits Rural Public Health and Health Transitions Research Unit (Agincourt), School of Public Health, Faculty of Health Sciences, University of the Witwatersrand, Johannesburg, South Africa
[7]Membership list can be found in the Acknowledgments section of the article
[*]Correspondance to `work@samclark.net`

January 10, 2019

Contents

1 Data **1**

1.1 Data Summary 1
1.2 List of Excluded Countries 4
1.3 Sampling Designs 5
1.4 Adjustments to Geographic Boundaries 9

2 Methods: Models and Estimation **12**

2.1 Overview 12
2.2 Discrete-Hazards Model 12
2.3 Meta-Analysis Estimator 13
2.4 HIV Adjustment 14
2.5 Space-Time Smoothing Model 14
2.6 Estimating the Yearly Model 16
2.7 Decomposition of Variance 18
2.8 Benchmark with UN B3 Model 18
2.9 Cross Validation Analysis 18
2.10 Previous Approaches 19
2.11 Software 20

3 Full Results **21**

3.1 Partitioning of Variability 21
3.2 Summary of MDG Goals 22
3.3 Cross Validation Summary 27
3.4 Benchmarking Summary 27
3.5 All Results by Country 30
3.5.1 Angola 30
3.5.2 Benin 38
3.5.3 Burkina Faso 46
3.5.4 Burundi 54
3.5.5 Cameroon 62
3.5.6 Chad 70
3.5.7 Comoros 78
3.5.8 Congo 86
3.5.9 Côte d'Ivoire 94
3.5.10 DRC 102
3.5.11 Egypt 110
3.5.12 Ethiopia 118
3.5.13 Gabon 126
3.5.14 Gambia 134
3.5.15 Ghana 142
3.5.16 Guinea 150

3.5.17 Kenya 158
3.5.18 Lesotho 166
3.5.19 Liberia 174
3.5.20 Madagascar 182
3.5.21 Malawi 190
3.5.22 Mali 198
3.5.23 Morocco 206
3.5.24 Mozambique 214
3.5.25 Namibia 222
3.5.26 Niger 230
3.5.27 Nigeria 238
3.5.28 Rwanda 246
3.5.29 Senegal 254
3.5.30 Sierra Leone 262
3.5.31 Tanzania 270
3.5.32 Togo 278
3.5.33 Uganda 286
3.5.34 Zambia 294
3.5.35 Zimbabwe 302
3.6 Table of All Results: 5-year Periods 310
3.7 Table of All Results: 1-year Periods 387

4 Bibliography **617**

1 Data

1.1 Data Summary

The data come from the publicly available Demographic and Health Surveys (DHS) from 35 countries, available at `https://dhsprogram.com/data/`. For each country we utilized the data from surveys on birth histories of mothers and transformed them into person-months for the survival analysis model. For each person-month the survival state of the child is recorded. The number of cases (i.e., mothers) and number of person-months is presented below in Table S1.1.

Table S1.1: Summary of cases and person-months.

Country	Survey year	N of cases	N of person-months
1. Angola	2015	10,538	1,741,353
2. Benin	1996	4,097	661,362
	2001	4,405	768,995
	2006	13,308	2,429,752
3. Burkino Faso	1993	4,701	650,675
	1999	48,87	846,306
	2003	9,175	1,702,785
	2010	12,842	2,413,100
4. Burundi	2010	5,666	1,017,262
5. Cameroon	1998	37,92	690,851
	2004	7,396	1,313,003
	2011	10,852	1,909,136
6. Chad	2004	4,444	869,500
	2015	14,100	3,186,337
7. Comoros	1996	1,641	291,733
	2012	2,804	536,067
8. Congo	2005	4,943	717,107
	2012	8,786	1,491,413
9. Côte d'Ivoire	2011	7,075	1,193,702
10. DRC	2007	6,965	1,256,301
	2014	13,450	2,475,936
11. Egypt	1988	7,357	753,140
	1992	8,687	1,195,371
	1995	13,120	2,049,192
	2000	14,084	2,312,753

Continued on next page . . .

Country	Survey year	N of cases	N of person-months
	2003	8,268	1,395,527
	2005	17,542	2,923,043
	2008	14,775	2,407,743
	2014	15,444	21,76,420
12. Ethiopia	2000	9,796	1,276,952
	2005	9,249	1,552,396
	2011	10,876	1,988,678
	2016	10,273	1,945,158
13. Gabon	2000	4,257	690,603
	2012	6,316	1,112,847
14. Gambia	2013	6,742	1,229,602
15. Ghana	1988	3,237	307,082
	1993	3,308	426,494
	1998	3,355	506,353
	2003	3,871	648,853
	2008	3,186	534,445
	2014	6,290	1,061,769
16. Guinea	1999	5,249	850,741
	2005	6,179	1,134,133
	2012	6,752	1,214,406
17. Kenya	1993	5,220	802,033
	1998	5,566	936,517
	2003	5,706	960,136
	2008	5,793	1,001,697
	2014	22,703	3,985,496
18. Lesotho	2005	4,821	667,174
	2010	5,189	677,736
	2014	4,285	527,696
19. Liberia	2007	5,662	941,805
	2013	7,438	1,355,562
20. Madagascar	1992	4,032	539,463
	1997	4,927	758,111
	2004	5,801	945,740
	2009	12,872	2,297,858
21. Malawi	1992	3,717	6785,00
	2000	9,865	1,614,164

Continued on next page ...

Country	Survey year	N of cases	N of person-months
	2004	9,295	1,536,546
	2010	17,545	3,143,534
	2015	18,088	3,099,545
22. Mali	1987	2,462	223,280
	1995	7,415	1,175,483
	2001	10,340	1,867,518
	2006	11,203	2,095,845
23. Morroco	1987	4,886	497,138
	1992	4,800	683,144
	2003	8,641	1,521,614
24. Mozambique	2003	9,275	1,445,615
	2011	10,240	1,635,503
25. Namibia	2000	4,525	614,323
	2007	6,592	925,124
	2013	6,244	850,301
26. Niger	1992	4,933	691,098
	1998	5,879	1,018,743
	2006	7,202	1,475,757
	2012	8,327	1,647,200
27. Nigeria	1990	6,119	757,104
	2003	4,979	912,303
	2008	22,930	4,387,420
	2013	27,067	5,286,553
28. Rwanda	2000	6,289	1,089,540
	2005	6,898	1,273,176
	2008	4,647	824,193
	2010	8,043	1,411,541
	2015	8,712	1,408,731
29. Senegal	1992	4,225	613,232
	1997	5,988	1,044,021
	2005	9,440	1,734,846
	2010	10,037	1,820,068
	2012	5,312	961,360
	2014	11,272	2,060,197
	2015	5,779	1,049,703
	2016	5,694	1,038,192

Continued on next page ...

Country	Survey year	N of cases	N of person-months
30. Sierra Leone	2013	11,981	1,991,192
31. Tanzania	1996	5,767	866,646
	1999	2,513	397,551
	2005	6,158	1,035,459
	2008	5,082	875,028
	2010	5,885	1,056,150
	2012	4,464	253,256
	2015	8,019	1,409,897
32. Togo	1998	6,190	1,033,222
	2013	6,928	1,236,117
33. Uganda	1989	3,378	374,086
	1995	5,254	779,853
	2001	5,466	959,959
	2006	6,236	1,276,899
	2011	6,182	1,263,007
34. Zambia	1992	4,480	828,014
	1996	5,414	956,917
	2001	5,168	911,253
	2007	5,010	858,902
	2014	12,391	2,303,680
35. Zimbabwe	1994	4,387	813,190
	1999	4,207	688,904
	2006	5,977	903,002
	2011	6,332	865,210
	2015	6,972	949,685
Total	122	919,295	156,124,351

1.2 List of Excluded Countries

Table S1.2 summarizes countries excluded from this study and the reasons why.

Table S1.2: List of excluded countries.

Country	Reasons for exclusion
Botswana	Survey available only at year 1988
Cape Verde	Survey not publicly available
Central African Republic	Survey available only at year 1994-1995

Continued on next page . . .

Country	Reasons for exclusion
Equatorial Guinea	Survey not publicly available
Eritrea	Survey not publicly available
Mauritania	Survey available only at year 2000–2001
Sao Tome and Principe	Survey available only at year 2008–2009
South Africa	Survey available only at year 1998
Sudan	Survey available only at year 1989–1990
Swaziland	Survey available only at year 2006–2007
Tunisia	Survey available only at year 1988

1.3 Sampling Designs

As the goal of DHS is to not only provide estimates at the national level, but often also at the regional or urban/rural level, DHS utilizes sampling techniques that involve two-stage or multi-stage stratified sampling. We follow the final reports of DHS and account for the sampling designs to ensure our estimates weight the probabilities of case selection appropriately. When the strata variables are directly available from the data, we use the strata variable directly (usually *v023* in the data). When a direct strata variable cannot be found in the original data, we referenced the final reports and created the strata variable (e.g., interaction term between *v024* [region] & *v025* [urban/rural] if the sampling design is a two-stage stratified sample between region and urban/rural status). In surveys where the final report indicates that multi-stage sampling was implemented but the strata variable cannot be created from the data, we create the strata variable at the highest stratum available. For instance, if the survey implemented a three-stage stratified sampling using region → urban/rural → urban type, but the third stratum (i.e., urban type) is not available in the data, we create the strata variable at the second level (i.e., urban/rural). A complete list of strata variables for each is presented in Table S1.3.

Table S1.3: List of sampling strata variables.

Country	Survey year	Strata variable
Angola	2015	v023
Benin	1996	interaction between v024 & v025
	2001	v023
	2006	v023
Burkino Faso	1993	interaction between v024 & v025
	1999	interaction between v024 & v025
	2003	interaction between v024 & v025
	2010	interaction between v024 & v025
Burundi	2010	interaction between v024 & v025

Continued on next page ...

Country	Survey year	Strata variable
Cameroon	1998	interaction between v024 & v025
	2004	v023
	2011	interaction between v024 & v025
Chad	2004	interaction between snewzon & v025
	2015	interaction between v024 & v025
Comoros	1996	interaction between v024 & v025
	2012	v023
Congo	2005	interaction between v024 & v025
	2012	interaction between v024 & v025
Côte d'Ivoire	2011	v023
DRC	2007	interaction between v024 & v025
	2014	interaction between v024 & v025
Egypt	1988	interaction between v024 & v102
	1992	interaction between v024 & v025
	1995	interaction between v024 & v025
	2000	interaction between v024 & v025
	2003	interaction between v024 & v025
	2005	interaction between v024 & v025
	2008	interaction between v024 & v025
	2014	interaction between v024 & v025
Ethiopia	2000	v023
	2005	v023
	2011	v023
	2016	v023
Gabon	2000	v023
	2012	interaction between v024 & v025
Gambia	2013	interaction between v024 & v025
Ghana	1989	interaction between v101 & v025
	1993	interaction between v024 & v025
	1998	v023
	2003	interaction between v024 & v025
	2008	interaction between v024 & v025
	2014	interaction between v024 & v025
Guinea	1999	v023
	2005	interaction between v024 & v025

Continued on next page . . .

Country	Survey year	Strata variable
	2012	v023
Kenya	1993	v023
	1998	v023
	2003	interaction between v024 & v025
	2008	interaction between v024 & v025
	2014	interaction between v024 & v025
Lesotho	2005	interaction between v024 & v025
	2010	v023
	2014	v023
Liberia	2007	interaction between v024 & v025
	2013	v023
Madagascar	1992	interaction between v024 & v025
	1997	v023
	2004	v023
	2009	v023
Malawi	1992	v023
	2000	interaction between v024 & v025
	2004	interaction between v024 & v025
	2010	interaction between v024 & v025
	2015	v023
Mali	1987	interaction between v101 & v102
	1995	v023
	2001	interaction between v024 & v025
	2006	interaction between v024 & v025
Morroco	1987	interaction between v101 & v102
	1992	interaction between v024 & v025
	2003	v023
Mozambique	2003	interaction between v024 & v025
	2011	v023
Namibia	2000	v023
	2007	interaction between v024 & v025
	2013	v023
Niger	1992	interaction between v024 & v025
	1998	interaction between v024 & v025
	2006	interaction between v024 & v025
	2012	interaction between v024 & v025

Continued on next page . . .

Country	Survey year	Strata variable
Nigeria	1990	interaction between v024 & v025
	2003	interaction between v024 & v025
	2008	interaction between v024 & v025
	2013	interaction between v024 & v025
Rwanda	2000	interaction between v024 & v025
	2005	interaction between v024 & v025
	2008	interaction between v024 & v025
	2010	interaction between v024 & v025
	2015	v023
Senegal	1992	interaction between v024 & v025
	1997	interaction between v024 & v025
	2005	interaction between v024 & v025
	2010	interaction between v024 & v025
	2012	v023
	2014	v023
	2015	v023
	2016	v023
Sierra Leone	2013	v023
Tanzania	1996	interaction between v024 & v025
	1999	interaction between v024 & v025
	2005	interaction between v024 & v025
	2010	interaction between v024 & v025
	2015	interaction between v024 & v025
Togo	1998	interaction between v024 & v025
	2013	interaction between v024 & v025
Uganda	1989	interaction between v101 & v102
	1995	interaction between v024 & v025
	2001	interaction between v024 & v025
	2006	interaction between v023 & v025
	2011	interaction between v024 & v025
Zambia	1992	interaction between v024 & v025
	1996	interaction between v024 & v025
	2001	interaction between v024 & v025
	2007	interaction between v024 & v025
	2014	interaction between v024 & v025
Zimbabwe	1994	v023
	1999	v023

Continued on next page ...

Country	Survey year	Strata variable
	2006	interaction between v024 & v025
	2011	v023
	2015	v023

1.4 Adjustments to Geographic Boundaries

Table S1.4 summarizes the geographic boundaries used in this study for all 35 countries and special notes for additional processing in some of the surveys. For countries with subnational region boundary splits or merges in different surveys, we map all observations to a common set of regions whenever possible, which usually results in merging finer regions to large ones. For region redraws that cannot be cast to a common set of boundaries, we used GPS data to map observations to the adopted boundaries. For cases where region redraws cannot be cast to a common set and GPS data is not available, we attempt to map to the closest boundary possible if the issue is minor and drop the survey if the issue is severe, as noted in Table S1.4.

Table S1.4: Maps used for each country with notes on resolution of geographical boundary issues.

Country	Map	Notes
Angola	2015	
Benin	2001	
Burkina Faso	1999	Ouagadougou merged into Central/South.
Burundi	2010	
Cameroon	1998	
Chad	2004	Due to boundary redrawing, we used GPS data to map year 2015.
Comoros	2012	Assumed the three islands are geographically connected.
Congo	2005	Assumed Brazzaville and Pointe-Noire are the same between year 2005 and year 2011.
Côte d'Ivoire	2011	
DRC	2013	
Egypt	1988	Uses groups of admin 1 areas. Assumes 2014 Frontier Governorates are comparable despite exclusion of two of the five Frontier Governorates.

Continued on next page ...

Country	Map	Notes
Ethiopia	2011	
Gabon	2000	
Gambia	2013	Merges the Kanifing region into Banjul
Ghana	1988	
Guinea	1999	Due to boundary redrawing, we used GPS data to map years 2005 and 2012.
Kenya	2014	
Lesotho	2014	
Liberia	2013	The 1986 DHS survey was excluded because of significant boundary redrawing.
Madagascar	2004	
Malawi	1992	
Mali	1987	
Morocco	1992	
Mozambique	2011	
Namibia	2013	The 1992 DHS survey was excluded because of significant boundary redrawing.
Niger	1998	
Nigeria	2013	Due to boundary redrawing , we used GPS data to map year 1990
Rwanda	2015	For year 2000, assumes Kigali city is the same as 2015 & Kigali rural is East region. Estimates should be interpreted with caution. Due to boundary redrawing , we used GPS data to map year 2005.
Senegal	2005	Due to boundary redrawing , we used GPS data to map years 1992 and 1997
Sierra Leone	2013	
Tanzania	1996	
Togo	1998	
Uganda	2000	
Zambia	2007	

Continued on next page . . .

Country	Map	Notes
Zimbabwe	2010	The 1988 DHS was excluded because it was only representative at the national level.

2 Methods: Models and Estimation

2.1 Overview

The available data are relatively sparse in space and time and so in order to produce reliable U5MR estimates at the subnational level we use space-time smoothing methods. Such methods are standard in spatial epidemiology [31] but here analysis is complicated by the stratified, cluster design of the DHS. This design must be acknowledged to avoid bias and obtain an appropriate measure of uncertainty.

The classic paradigm for the analysis of complex survey data is design-based inference [18]. We use this well-established theory and, following previous work [20, 29] (in which the model was proposed and extensively tested on Tanzania and Kenya, respectively), we obtain a (weighted) estimate of the logit U5MR with its associated design-based standard error. This is a small-area estimation problem and we first combine all the data in a particular area to obtain this estimate. We then take as likelihood the asymptotic (large-sample) distribution of the estimator of the logit of U5MR (we take the logit to improve the normal approximation). If we let y_{it} be the logit of the weighted estimator in area i and time period t then we have as likelihood y_{it} distributed as

$$\mathrm{N}(\lambda_{it}, \widehat{V}_{it}),$$

where $\widehat{V}_{it}$ is the appropriate estimated variance that accounts for the sample design (and is treated as known), and λ_{it} is the mean, to which we apply a (Bayesian) space-time smoothing model. We first describe how we obtain the logit of U5MR.

2.2 Discrete-Hazards Model

Throughout the following we use *month* as the unit of time. We adopt a discrete hazard model with age groups

$$[0, 1), [1, 12), [12, 24), [24, 36), [36, 48), [48, 60)$$

months. The first year is split into the first month when a child is particularly vulnerable and the remainder of the first year. The remaining 4 age groups cover the subsequent years until age 5 years. The use of unequally-spaced age groups means that the notation is a little finicky. Since a month is the shortest time unit considered, each child's data is expanded to provide up to 60 records, one for each month from birth, with a binary indicator of survival or death. We use standard demographic notation so that ${}_nq_x$ is the probability of death in $[x, x+n)$, given survival to x. The starting ages for the intervals, x_a, take the values $(0, 1, 12, 24, 36, 48)$ for $a = 1, \dots, 6$, and the widths of the intervals, n_a, take the values $(1, 11, 12, 12, 12, 12)$ for $a = 1, \dots, 6$. In a generic area and period and for a particular survey, the U5MR may be calculated over age groups a as

$${}_{60}q_0 = 1 - \prod_{a=1}^{6} \left(1 - {}_{n_a}q_{x_a}\right).$$

Let ${}_{n_a}q_{x_a}^{its}$ be the probability of an under-five death in county i during time period t in age group $[x_a, x_a + n_a)$ for survey s. Detailed argument in [2] show that the contributions for a generic child correspond to the product of up to 60 Bernoulli likelihoods. In county i, time period t and survey s, we therefore fit a weighted logistic regression to estimate the parameters of the following model:

$$\log\left(\frac{{}_1q_x^{its}}{1 - {}_1q_x^{its}}\right) = \beta_a^{its}, \tag{1}$$

for $x \in [x_a, x_a + n_a)$, $a = 1, \ldots, 6$. Consequently, $\exp(\beta_a)$ is the conditional odds of death for a month x contained in $[x_a, x_a + n_a)$, given survival until the end of month $x - 1$ in $[x_a, x_a + n_a)$. The weighting in the logistic regression accounts for the multistage cluster sampling design and produces design unbiased estimators along with an appropriate design-based variance [5]. The design-based model (1) is fitted in the `survey` package in `R`. This gives us a design-based *direct estimate*, $\widehat{\beta}_a^{its}$, for each county i, time period t, survey s and age group a, as well as a design-based covariance matrix for the vector $\boldsymbol{\beta}^{its} = (\beta_1^{its}, \ldots, \beta_6^{its})$. Note, (1) implies

$${}_1\widehat{q}_x^{\,its} = \frac{\exp(\widehat{\beta}_a^{its})}{1 + \exp(\widehat{\beta}_a^{its})},$$

i.e., the hazard of dying in $[x, x + 1)$ for $x \in [x_a, x_a + n_a)$, and so

$${}_{60}\widehat{q}_0^{\,its} = 1 - \prod_{a=1}^{6} \left(1 - {}_1\widehat{q}_{x_a}^{\,its}\right)^{n_a}.$$

Using the delta method we calculate the design-based variance of the $\text{logit}({}_{60}\widehat{q}_0^{\,its})$ values, $\widehat{V}_{\text{DES},its}$, that reflects the sampling scheme, details in [20].

To summarize, we form estimates and standard errors for each area crossed with 5-year time period, and for each survey s. The problem is that the standard errors are often large, because of the small sample sizes when we cross areas with time periods. Generally speaking, U5MR rates in nearby areas will tend to show similarity, and rates in adjacent periods will also tend to be similar. We leverage this similarity in the space-time models we describe in Section 2.5.

2.3 Meta-Analysis Estimator

The direct estimate of child mortality in survey s for county i and in time period t is denoted ${}_{60}\widehat{q}_0^{\,its}$. In general multiple surveys s will produce estimates in area i and period t, so we create a combined estimate with an appropriate variance. The estimator we produce weights each survey's contribution to area i and period t by the inverse of its variance, i.e., estimates

with larger variance receive smaller weight:

$$
{}_{60}\widehat{q}_0^{\,it} = \text{expit}\left(\sum_{s=1}^{S_t} \underbrace{\left[\frac{\widehat{V}_{\text{DES},its}^{-1}}{\sum_{s=1}^{S_t} \widehat{V}_{\text{DES},its}^{-1}} \right]}_{\text{Weight for survey } s} \text{logit}({}_{60}\widehat{q}_0^{\,its}) \right), \tag{2}
$$

where the expit function is defined as $\text{expit}(x) = \exp(x)/[1 + \exp(x)]$ and S_t is the number of surveys contributing to time period t. We then calculate the associated design-based variance, under the assumption of independence between surveys,

$$
\widehat{V}_{\text{DES},it} = \frac{1}{\sum_{s=1}^{S_t} \widehat{V}_{\text{DES},its}^{-1}}.
$$

The intuition is that the precision of the combined estimate is the sum of the precisions from each of the constituent surveys. We might refer to ${}_{60}\widehat{q}_0^{\,it}$ as a meta-analysis estimator, since its construction is identical to the fixed effects estimator that is used to combine information from multiple sources.

2.4 HIV Adjustment

In countries with high prevalence of HIV, and this can lead to serious bias in estimates of U5MR, particularly before ART treatment became widely available. Pre-treatment HIV positive women had a high risk of dying, and such women who had given birth were therefore less likely to appear in surveys. The children of HIV positive women are also more likely to die before age of 5 compared those born to HIV negative women, and therefore we expect to underestimate U5MR if we do not adjust for the missing women (in statistical parlance, the missing data are non-ignorable).

Following the cohort component projection model method described in [30], we obtained the estimated ratio, $\widehat{r}_{ts}$ of the reported U5MR to "true" U5MR for each survey s and 5-year period t.

2.5 Space-Time Smoothing Model

We follow [20] and fit a space-time smoothing model to the meta-analysis area-period estimate. This model allows us to share information between the direct estimates at the county level to result in more reliable (lower mean squared error) final estimates. Since U5MR estimates are in $[0, 1]$ we take the logit transform in order to obtain a variable that is on the whole real line and which can be more reliably modeled via a normal distribution. To summarize, we take as data (often referred to as *the logit* in what follows),

$$
y_{it} = \log\left(\frac{{}_{60}\widehat{q}_0^{\,it}}{1 - {}_{60}\widehat{q}_0^{\,it}} \right).
$$

The data model is

$$y_{it}|\lambda_{it} \sim \text{N}(\lambda_{it}, \widehat{V}_{\text{DES},it}),$$

where λ_{it} is the logit of the true U5MR in county i and period t, and we emphasize that $\widehat{V}_{\text{DES},it}$ is known. Note that if everyone is sampled in an area then $V_{\text{DES},its} = 0$, the space-time smoothing prior has no impact, and and we recover the finite population mean, i.e. the true U5MR. This assumes that the prior gives positive mass to any value that ${}_{60}q_0{}^{it}$ might take, which is true of our prior. Note that

$${}_{60}q_0{}^{it} \quad = \quad \frac{\exp(\lambda_{it})}{1+\exp(\lambda_{it})} \tag{3}$$

is the U5MR estimate in county i and period t.

We share information between contiguous neighbors and close time periods. The way such sharing is carried out is by specifying probability distributions that penalize estimates of ${}_{60}q_0{}^{it}$ that are very different in areas that are geographically and/or temporally close. Hence, similarity in estimates is encouraged. We stress that all values are allowed, but those that are alike are encouraged by the prior. We decompose λ_{it} into temporal, spatial and space-time components:

$$\lambda_{it} = \mu + \alpha_t + \gamma_t + \theta_i + \phi_i + \delta_{it}. \tag{4}$$

Both time and space have two ingredients, an independent term that picks up "random shocks" and a smoothing term. The parameters α_t and γ_t model the logit of U5MR over time, and θ_i and ϕ_i model the logit in space, with δ_{it} being the interaction between time and space which allows additional flexibility. Apart from the intercept μ, the remaining parameters are treated as random effects having independent zero mean normal distributions with constant variances; the distinguishing feature being that the variance (smoothing parameter) is estimated from the data. For all random effects associated with time, we estimate them as the average of 5 random effects on the yearly scale. This allows us to obtain both estimates on 5-year periods and estimates on the yearly scale. We refer to this model as the "yearly model", and describe the estimation details in the next subsection. There are many advantages to using a *linear hierarchical model* like this. We now give details on each of these constituent terms. We emphasize that t in this model refers to period (which is the scale at which the data enter), and in what follows (and in the next section) we explain how we translate the underlying yearly model into a model for periods.

- μ: fixed intercept with a relatively flat prior.
- α_t, θ_i: independent random effects, i.e. effects with no structure. These random effects all have the same form of distribution; we write $\text{N}(0, \sigma^2)$ for a generic random effects distribution. In the Bayesian approach to inference we place priors on each variance, σ^2 here, and this, along with the data, allows the amount of smoothing to be estimated. This variance determines the amount of smoothing with small/large values favoring

large/small amounts of smoothing. The period random effect α_t is estimated as the average of 5 yearly random effects with the same distribution. We describe the details of the yearly model in the next subsection.

- γ_t: temporal smoothing random effects; we consider random walk models of orders 2 (RW2) [24] on the yearly scale. There is a variance parameter that again controls the amount of smoothing.
- ϕ_i: spatial smoothing random effects; we take as local spatial smoothing model an intrinsic conditional autoregressive (ICAR) model [3]. This model is a generalization of the RW1 model to space. This generalization is problematic on data that do not conveniently fall on a grid. We need to specify what we mean by neighbors in space, and we take a conventional definition as sharing a common boundary, but we could specify alternative definitions based on distance or cultural similarity. So in the ICAR model we use, dependencies are assumed to extend to areas that are next to each other. Again, we have an additional variance parameter to estimate.
- δ_{it}: spatial-temporal interaction random effects; We use the type IV interaction described in [14], that assumes the spatially and temporally structured effects interact at the yearly level.

The model was fitted with the accurate and fast integrated nested Laplace approximation (INLA) method [25], which has an implementation in the `R` software.

This method has a number of advantages over other approaches:

- It transparently acknowledges the sampling design,
- it is based on well-understand Gaussian Markov Random Field (GMRF) models that have seen extensive use in spatial epidemiology [24], and
- the method has been validated in the statistics community [20].

2.6 Estimating the Yearly Model

The temporal random effects we considered in the model are all assumed to be on the yearly scale, while the direct estimates, y_{it}, is only observed on the 5-year period. In this subsection, we describe the procedure in fitting the yearly random effects from the aggregated data. We

denote the vector of random effects on the period scale to be

$$\alpha_t = \sum_{t'=5(t-1)+1}^{5t} \tilde{\alpha}_{t'} \quad (5)$$

$$\gamma_t = \sum_{t'=5(t-1)+1}^{5t} \tilde{\gamma}_{t'} \quad (6)$$

$$\delta_{it} = \sum_{t'=5(t-1)+1}^{5t} \tilde{\delta}_{it'}. \quad (7)$$

Random walk models are Markovian, meaning that dependencies between terms (here $\tilde{\gamma}_{t'}$) are assumed to be local. In a RW2 model, $\tilde{\gamma}_{t'}$ depends on second-order neighbors $\tilde{\gamma}_{t'-2}, \tilde{\gamma}_{t'-1}$ and $\tilde{\gamma}_{t'+1}, \tilde{\gamma}_{t'+2}$.

Take $\boldsymbol{\gamma}$ and $\tilde{\boldsymbol{\gamma}}$ for an example. $\tilde{\boldsymbol{\gamma}}$ follow a random walk model of order k, so that for some unknown precision κ,

$$\pi(\tilde{\boldsymbol{\gamma}}) = (2\pi)^{-(n-k)/2}\kappa^{(n-k)/2} \exp\left[-\frac{1}{2}\kappa\tilde{\boldsymbol{\gamma}}_1^T \mathbf{R}\tilde{\boldsymbol{\gamma}}_1\right], \quad (8)$$

where $\boldsymbol{R}$ is the dependency matrix of a RW of order k, scaled by the generalized variance of the vector $\tilde{\boldsymbol{\gamma}}$ fixing $\kappa = 1$. Since $\tilde{\boldsymbol{\gamma}} = \boldsymbol{A}\boldsymbol{\gamma}$, where

$$\mathbf{A} = \begin{bmatrix} 1/5 & 1/5 & 1/5 & 1/5 & 1/5 & 0 & \cdots & \cdots \\ 0 & \cdots & 1/5 & 1/5 & 1/5 & 1/5 & 1/5 & \cdots \\ \cdots & \cdots & \cdots & \cdots & \cdots & \cdots & \cdots & \\ \cdots & \cdots & 0 & 1/5 & 1/5 & 1/5 & 1/5 & 1/5 \end{bmatrix}, \quad (9)$$

we approximate this deterministic linear relationship with the conditional distribution

$$\pi(\tilde{\boldsymbol{\gamma}}|\boldsymbol{\gamma}) \sim \mathrm{N}\left(\boldsymbol{A}\boldsymbol{\gamma}, \frac{1}{\tau}\boldsymbol{I}\right)$$

where τ is a fixed high value. In this way, the joint distribution of $[\tilde{\boldsymbol{\gamma}} \quad \boldsymbol{\gamma}]^T$ is multivariate normal with precision matrix

$$\mathbf{Q} = \begin{bmatrix} \kappa\mathbf{R} + \tau\mathbf{A}^T\mathbf{A} & -\tau\mathbf{A}^T \\ -\tau\mathbf{A} & \tau\mathbf{I} \end{bmatrix}. \quad (10)$$

For $\boldsymbol{\alpha}$, we only need to change $\boldsymbol{R}$ above to be the identity matrix. For $\boldsymbol{\delta}$, under the type IV interaction defined in [14], the distribution of $vec(\boldsymbol{\delta})$ is a multivariate normal with precision matrix $\boldsymbol{R}_{RW} \otimes \boldsymbol{R}_{ICAR}$, where $\otimes$ denotes the Kronecker product; $\boldsymbol{R}_{RW}$ and $\boldsymbol{R}_{ICAR}$ denotes the precision matrix of the RW and ICAR random effects respectively.

2.7 Decomposition of Variance

First, in order for the model to be identifiable, we need to impose constraints on each group of random effects to sum to zero. The total varaince of the observed data can be decomposed into

$$var(\lambda_{it}) = \sigma^2_\alpha + \sigma^2_\gamma + \sigma^2_\theta + \sigma^2_\phi + \sigma^2_\delta \tag{11}$$

Each of the σ^2 quantity is the variance of a random effect. We calculated the estimates of these posterior marginal variances from the posterior marginal distribution of (a generic) random effect u with dimension m to be the empirical median of $\hat{\sigma}^2_u = \frac{\sum_{i=1}^m (u_i - \bar{u})^2}{m-1}$.

2.8 Benchmark with UN B3 Model

The final results were obtained with an additional adjustment step to account for the difference between the smoothed estimates and the estimates from the B3 model [1] on the national-level. We first fitted a national-level model with fixed and random effects corresponding to time trend only, i.e., μ, β, α_t, and γ_t. We calculated the estimated ratio, $\hat{r}_t$ of the posterior median U5MR from the national model to median U5MR from B3 model for each 5-year period t. We then adjusted the direct estimates ${}_{60}\widehat{q}_0^{\,it}$ by $\widehat{r}_t$ for each region i to obtain ${}_{60}\widetilde{q}_0^{\,it} = {}_{60}\widehat{q}_0^{\,it}/\hat{r}_t$ and calculated its variance on the logit scale accordingly. The final smoothed national and subnational model estimates were calculated using ${}_{60}\widetilde{q}_0^{\,it}$ in place of ${}_{60}\widehat{q}_0^{\,it}$.

2.9 Cross Validation Analysis

We conducted a cross validation study for each country. We systematicallly held out observations from one region in one time period, fit the model using the rest of the data, and compared the projected $\hat{\lambda}_{-it}$ (estimate with data omitted from area i and period t) to the direct estimates y_{it} for this region in the held-out time period. In each iteration, we obtained the marginal posterior distribution of λ_{-it}, with posterior expectation $\hat{\lambda}_{-it}$ and variance $\hat{\sigma}^2_{-it}$. To examine the frequentist accuracy of the posterior expectation, we assume $\widehat{V}_{\text{DES},it}$ is known, and then the posterior distribution of the bias between the projection and the held-out observation, i.e., $\hat{\lambda}_{-it} - y_{it}$, is approximately $\text{N}(0, \widehat{V}_{\text{DES},it} + \hat{\sigma}^2_{-it})$. Thus we can evaluate the coverage probability of the 95% posterior interval

$$\hat{\lambda}_{-it} - y_{it} \pm 1.96\sqrt{\widehat{V}_{\text{DES},it} + \hat{\sigma}^2_{-it}}.$$

We also examine the scaled bias

$$\frac{\hat{\lambda}_{-it} - y_{it}}{\sqrt{\widehat{V}_{\text{DES},it} + \hat{\sigma}^2_{-it}}},$$

and plot against time t to see if there are systematic deviations from the reference horizontal line at zero.

The full results for each country are presented in the results section 3.5.

2.10 Previous Approaches

Dwyer and colleagues [9] compare various spatial models for U5MR modeling in Zambia using DHS data. In their approach, the logit of the U5MR is modeled as normally distributed, but with a single common variance across all studies, which is clearly inappropriate since it does not acknowledge the differing effective sample sizes in each area. Computation was carried out using the integrated nested Laplace approximation (INLA) of [25]. [20] analyzed DHS data from 22 regions in Tanzania and assumed a likelihood in which the logit of the weighted (design) estimator was assumed to be normally distributed with variance given by the design variance. A discrete space, discrete time (5-year intervals) interaction model [14] was used to smooth the mean of this distribution, with implementation via INLA. Wakefield and colleagues [29] compare discrete and continuous spatial models in the context of U5MR estimation in Kenya. Pezzulo et al. [21] model ${}_4q_1$ across 27 countries in sub-Saharan Africa, at the Admin 1 level. Estimation was based on the most recent DHS with the log weighted U5MR estimators assumed to be normally distributed with spatial smoothing being carried out via the model of [16]. Extensive covariate modeling was carried out with potential variables being averaged within areas, and also allowing interactions by large regions (with three regions in total). As with all approaches that include covariates at the area level, the associations at the area-level cannot be transferred to the individual-level as this opens up the possibility of the ecological fallacy [28].

In other contexts, methods for small-area estimation [23] using spatial smoothing models have been proposed by a number of authors including [8], [35], [22], [7], [27] and [32]. Notably, these approaches all utilize spatial models at the area level, whereas the model we propose models space continuously.

Burke and co-workers [6] follow a different approach to modeling U5MR across sub-Saharan Africa. Kernel density estimation (KDE) is carried out with surfaces produced at a geographical scale of approximately 10km$\times$10km. This approach follows [15] who used the same method in the context of HIV prevalence estimation. Inference, including producing uncertainty surfaces, is difficult to obtain with KDE and the approach has been found to be inferior, when considering prediction at unsampled locations, to Bayesian geostatistical modeling [13].

More recently, IHME (Golding et al. [12]) carried out subnational estimation of U5MR for sub-Saharan Africa, with a continuously indexed spatial model. Four separate models were fitted to the age groups 0–1 months, 1–11 months, 12–35 months, 36–59 months, with the subsequent estimates being combined to give the U5MR. This combination is done by taking draws from the posteriors assuming they are independent, which is not correct, since they are based on the same children. Data from a variety of sources are included in the analysis including both full birth history (FBH) and summary birth history (SBH) data. FBH data include information for all children on the times of birth and death, if the latter occurs before the time of the survey, and these are the data we utilize from the DHS. SBH data consist of the number of children ever born, and the number who have died, along with the age of the mother. The FBH data are modeled as binomial with no explicit correction for

the survey design. The SBH data are also assumed to be binomially distributed, with an artificial response and denominator created through an elaborate procedure with a heuristic justification. A space-time smoothing model is specified via the stochastic partial differential equations (SPDEs) formulation of [17]. The same space-time covariance parameters are assumed for the whole of Africa. There is no adjustment for mothers lost to HIV, which can lead to serious underestimation in countries (such as Kenya and Malawi) with HIV epidemics. Estimates in each spatial grid cell are adjusted so that the national total agrees with the Global Burden of Disease (GBD) estimates. Covariates are also modeled.

Gething et al. [11] describe the use of DHS data to construct surfaces of: access to HIV testing in women, stunting in children, anemia prevalence in children and access to improved sanitation. For each outcome and each country the following procedure was carried out. A collection of 17 covariates were examined. Initially, simple linear regression was used taking three versions (the original, the square and the square root) of each of the 17 variables. Cross-validation was then used to reduce these to a subset of 17 terms. Two-way interactions for these 17 were added to the collection to give $289 = 17 \times 17$ additional terms. This complete set was reduced to 20, again via cross-validation. Then the resultant potential $2^{20} - 1$ models, that were combinations of these 20 terms, were compared.

Bhatt et al. [4] use an approach known as stacked generalization [34] in which multiple predicting algorithms are weighted to produce a final prediction. This approach is closely related to the more general super-learner approach [26]. This approach has optimality properties for prediction but has a lack of interpretability, and the model is not suitable for predictions into the future. There is also no way that uncertainty in the estimation procedure can be incorporated into interval estimates for the surface. A similar approach was used by [12].

The most recent GBD [10] produced national estimates for 195 countries and territories over the period 1970–2016. Some of the constituent data in the study of [12] do not contain GPS locations, but rather the administrative region within which the clusters were sampled. In this case, Golding et al. (2017, Supplementary Materials, Section 8) assign the data to a set of points selected within the area, where the points are obtained through k-means clustering. This approach is, at best, an approximation, since one needs to take a mixture over the likelihoods at each potential location, see [33].

2.11 Software

The software we developed for this study is available as an open source package for the `R` statistical programming environment, available at `https://cran.r-project.org/package=SUMMER` [19]. All of the `R` code used to produce the results described in this article is available at `https://github.com/richardli/AfricaU5MR`.

3 Full Results

3.1 Partitioning of Variability

Table S1.5 presents the partitioning of variability among the random effect terms in the space-time model.

Table S1.5: Variance component proportions for each country.

country	RW2 ($\sigma^2_{\gamma t}$)	ICAR($\sigma^2_{\phi_i}$)	IID space ($\sigma^2_{\theta_i}$)	IID time ($\sigma^2_{\alpha t}$)	space time ($\sigma^2_{\delta_{it}}$)
Angola	6%	56.1%	1.4%	0.3%	36.3%
Benin	65.5%	27.4%	2.7%	0.5%	3.8%
Burkina Faso	56.9%	30.9%	3%	0.6%	8.7%
Burundi	47.5%	35.6%	1.8%	0.4%	14.7%
Cameroon	29.2%	65.3%	2.4%	0.4%	2.7%
Chad	36.9%	41.4%	3.3%	0.6%	17.7%
Comoros	64.9%	16%	1.6%	0.5%	17%
Congo	72%	13.5%	2.4%	0.6%	11.4%
Côte d'Ivoire	25.7%	54.8%	2.5%	0.4%	16.7%
DRC	53.9%	28.1%	2.1%	0.4%	15.4%
Egypt	80.2%	14.9%	0.9%	0.2%	3.7%
Ethiopia	70.8%	24.6%	1%	0.2%	3.4%
Gabon	51.4%	30.4%	3.7%	0.8%	13.6%
Gambia	66.2%	18.6%	0.9%	0.2%	14%
Ghana	56.5%	34.5%	2.6%	0.5%	5.9%
Guinea	62.7%	31.5%	1.1%	0.2%	4.6%
Kenya	31.5%	48.1%	1.7%	0.3%	18.4%
Lesotho	28%	28.2%	4.6%	1.1%	38.1%
Liberia	84.2%	6.9%	1.3%	0.3%	7.3%
Madagascar	72.7%	16.2%	1.4%	0.3%	9.4%
Malawi	87%	11.1%	1%	0.2%	0.6%
Mali	42.8%	50.4%	1.2%	0.2%	5.4%
Morocco	83%	8.8%	1%	0.2%	7%
Mozambique	65.2%	21.8%	1%	0.2%	11.8%
Namibia	44.5%	32.5%	2.2%	0.6%	20.2%
Niger	57.1%	30.9%	1.4%	0.3%	10.3%
Nigeria	26.7%	65.4%	1.9%	0.3%	5.7%
Rwanda	83.7%	12.5%	1%	0.2%	2.5%
Senegal	72.5%	23%	1.2%	0.2%	3%
Sierra Leone	59.4%	24.7%	2.3%	0.5%	13.1%
Tanzania	75.8%	17.5%	1.2%	0.2%	5.3%
Togo	53.4%	39.9%	2.5%	0.5%	3.6%
Uganda	87.1%	8.7%	1.3%	0.3%	2.7%
Zambia	75%	19.2%	1.6%	0.3%	3.8%

Continued on next page ...

country	RW2 ($\sigma^2_{\gamma t}$)	ICAR($\sigma^2_{\phi_i}$)	IID space ($\sigma^2_{\theta_i}$)	IID time ($\sigma^2_{\alpha_t}$)	space time ($\sigma^2_{\delta_{it}}$)
Zimbabwe	45.2%	44.3%	2.3%	0.5%	7.7%

3.2 Summary of MDG Goals

Table S1.6 summarizes the MDG4 status by country.

Table S1.6: MDG4 goal achievement status for subnational regions.

Country	MDG4 achieved	Percent achieved	Median reduction	[Min, Max]
Angola	1/18	5.56%	0.098	[-0.63,0.88]
Benin	0/6	0%	0.465	[0.38,0.57]
Burkina Faso	0/4	0%	0.599	[0.41,0.65]
Burundi	1/5	20%	0.637	[0.44,0.69]
Cameroon	0/5	0%	0.367	[0.22,0.43]
Chad	0/8	0%	0.353	[0.08,0.53]
Comoros	0/3	0%	0.335	[0.10,0.41]
Congo	1/4	25%	0.621	[0.39,0.69]
Côte d'Ivoire	0/11	0%	0.323	[-0.01,0.59]
DRC	1/11	9.09%	0.501	[0.27,0.78]
Egypt	2/4	50%	0.608	[0.51,0.74]
Ethiopia	10/11	90.91%	0.711	[0.58,0.80]
Gabon	0/5	0%	0.364	[0.14,0.52]
Gambia	4/6	66.67%	0.673	[0.36,0.82]
Ghana	0/8	0%	0.568	[0.35,0.61]
Guinea	3/5	60%	0.699	[0.40,0.73]
Kenya	3/8	37.5%	0.509	[0.04,0.75]
Lesotho	0/10	0%	0.195	[-0.44,0.46]
Liberia	4/5	80%	0.748	[0.45,0.78]
Madagascar	6/6	100%	0.804	[0.69,0.89]
Malawi	3/3	100%	0.717	[0.71,0.73]
Mali	1/4	25%	0.617	[0.46,0.74]
Morocco	4/7	57.14%	0.714	[0.56,0.81]
Mozambique	6/11	54.55%	0.679	[0.25,0.80]
Namibia	1/13	7.69%	0.443	[0.03,0.68]
Niger	3/6	50%	0.706	[0.47,0.84]
Nigeria	0/6	0%	0.466	[0.22,0.58]
Rwanda	5/5	100%	0.789	[0.71,0.80]
Senegal	6/11	54.55%	0.704	[0.59,0.76]
Sierra Leone	0/4	0%	0.554	[0.33,0.66]
Tanzania	16/20	80%	0.755	[0.55,0.85]
Togo	0/6	0%	0.449	[0.35,0.55]
Uganda	3/4	75%	0.711	[0.67,0.74]
Zambia	4/9	44.44%	0.658	[0.60,0.76]

Continued on next page ...

Country	MDG4 achieved	Percent achieved	Median reduction	[Min, Max]
Zimbabwe	0/10	0%	0.032	[-0.14,0.34]

Figures S1.1 to S1.4 show the projected U5MR and the projected reduction of U5MR at year of 2015 and the time period of 2015-2019 compared to that of 1990 respectively. In addition to the subnational model results, we include the comparison to the RW2 only model fitted to the combined national data, i.e., without subnational spatial smoothing, after benchmarking with UN estimates. We also compare our results with UN (B3) estimates described in You et al. (2015) and IHME estimates based on GBD 2015 Child Mortality Collaborators (2016) for the comparisons with 2015 estimates.

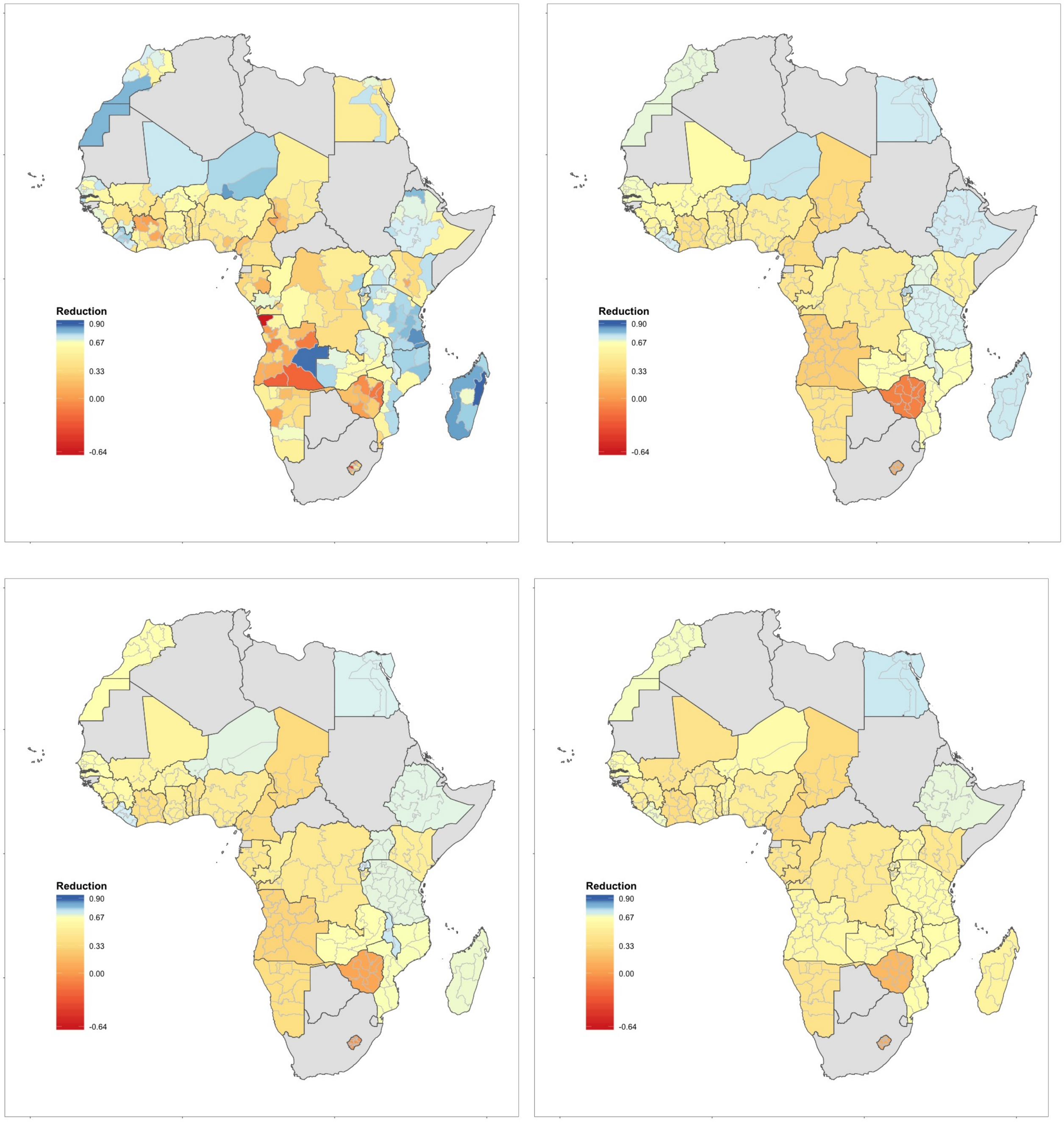

Figure S1.1: Reduction of U5MR from 1990 to 2015 estimated by different methods. Upper left: Subnational model. Upper right: National model. Lower left: UN B-3 estimates. Lower right: IHME GBD estimates

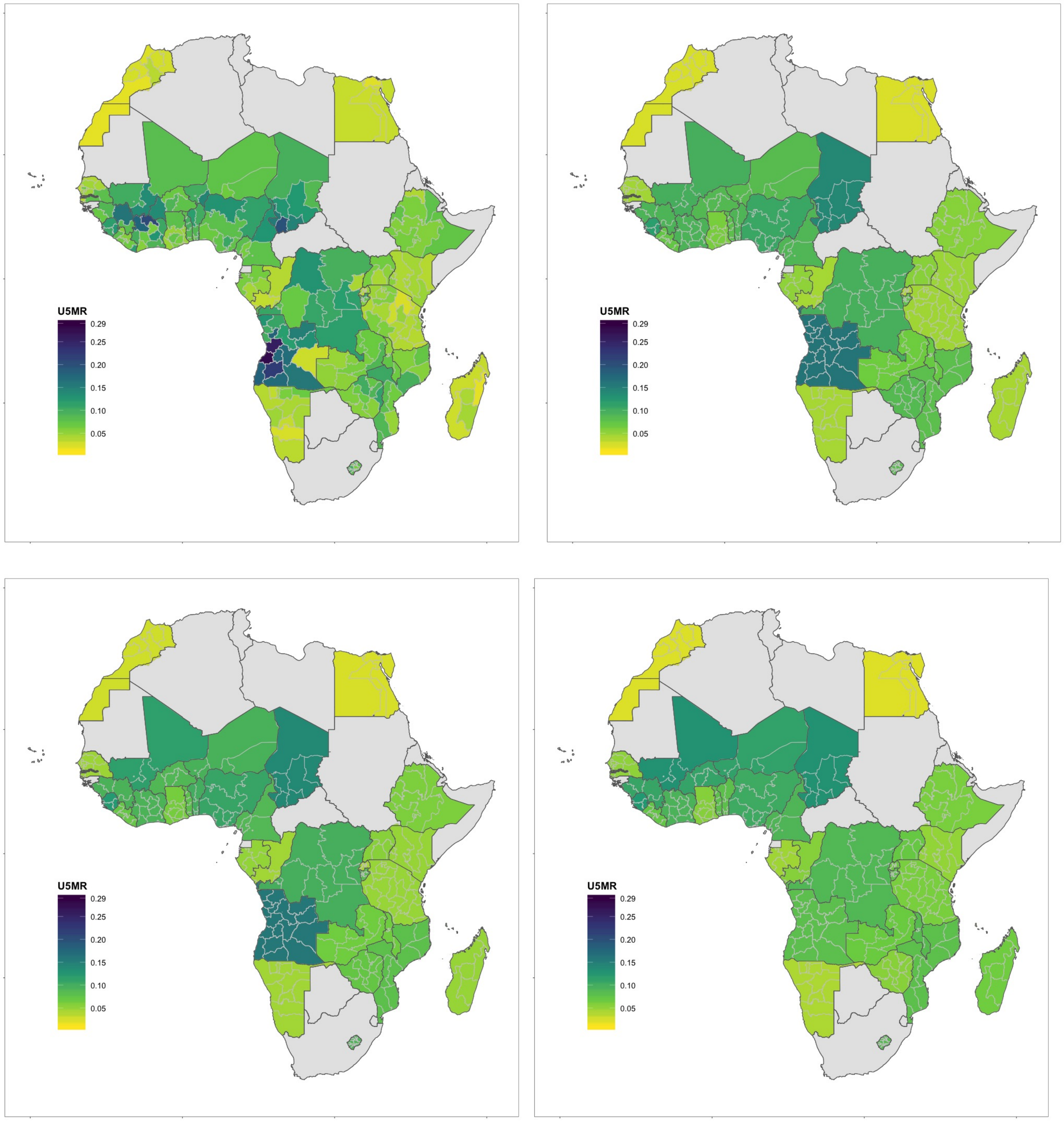

Figure S1.2: Projection of U5MR for 2015 by different methods. Upper left: Subnational model. Upper right: National model. Lower left: UN B-3 estimates. Lower right: IHME GBD estimates

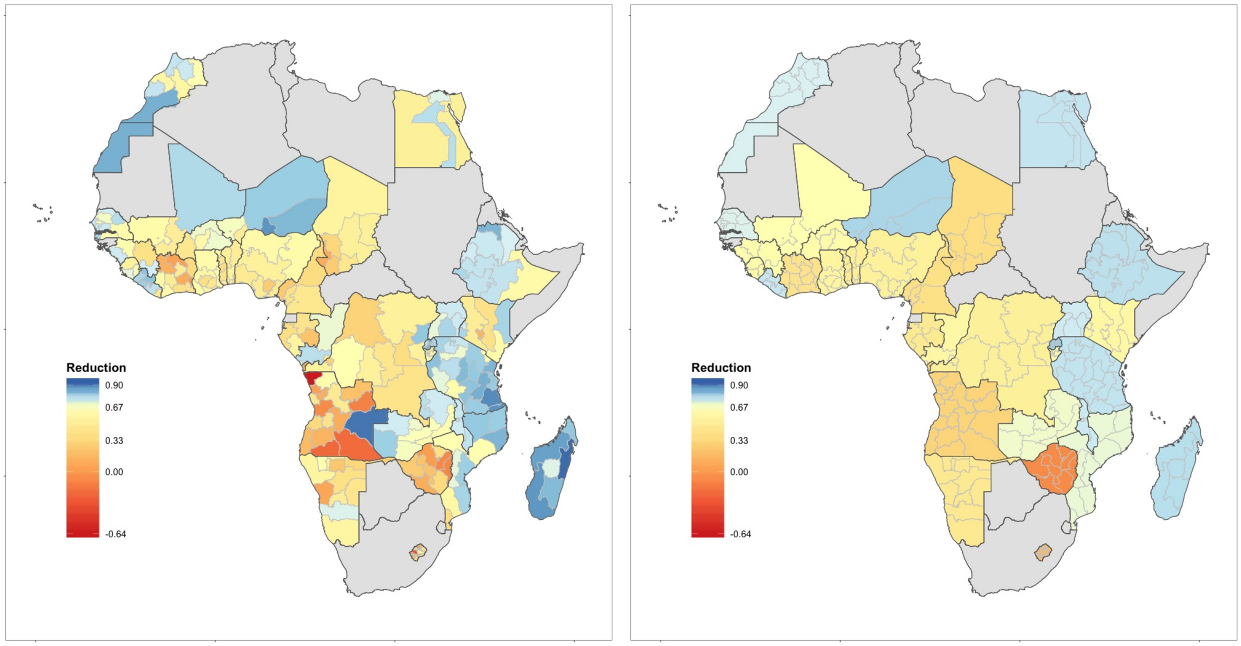

Figure S1.3: Reduction of U5MR from 1990 to 2015-2019 period estimated by different methods. Left: Subnational model. Right: National model.

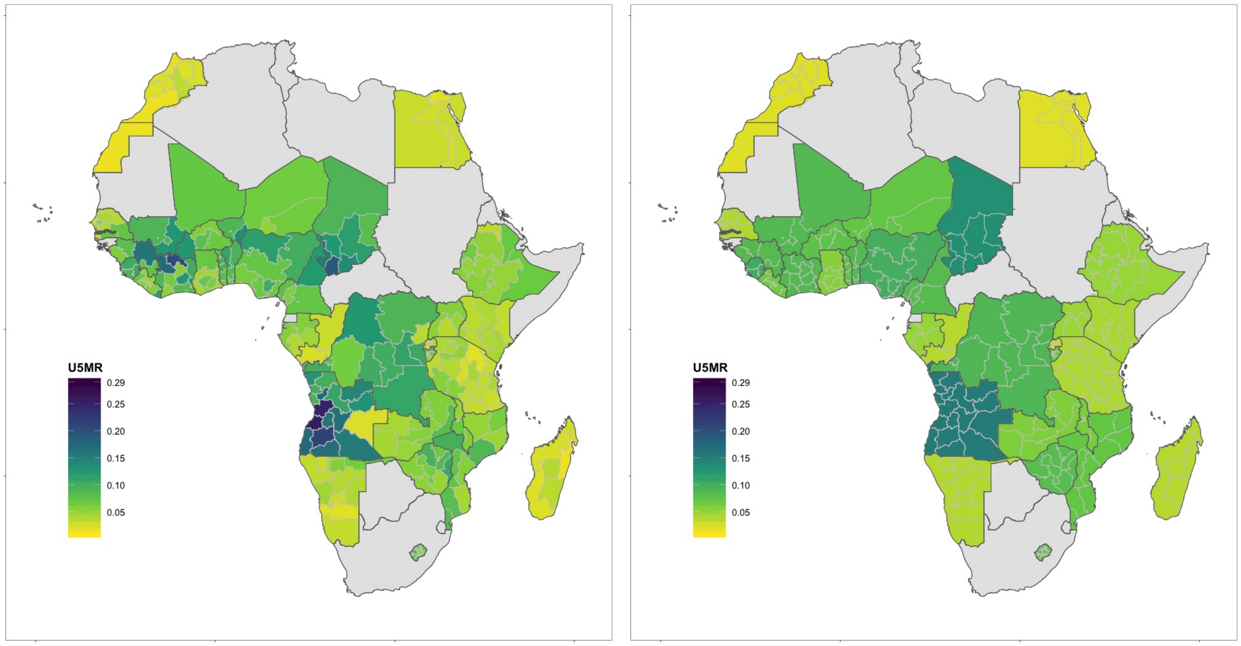

Figure S1.4: Projection of U5MR for 2015-2019 period by different methods. Left: Subnational model. Right: National model.

3.3 Cross Validation Summary

Figure S1.5 shows the distribution of the cross validation bias combined for all the regions in the study. Table S1.7 summarizes the cross validation results for each country. In general, the scaled bias measure behaves as we would expect if the model is correct (approximately like a standard normal). The plots of the rescaled bias against time for individual countries also do not show systematic patterns, which would be evidence of missing trends. Finally, the coverage of our 95% interval estimates is generally good (averaging 94%).

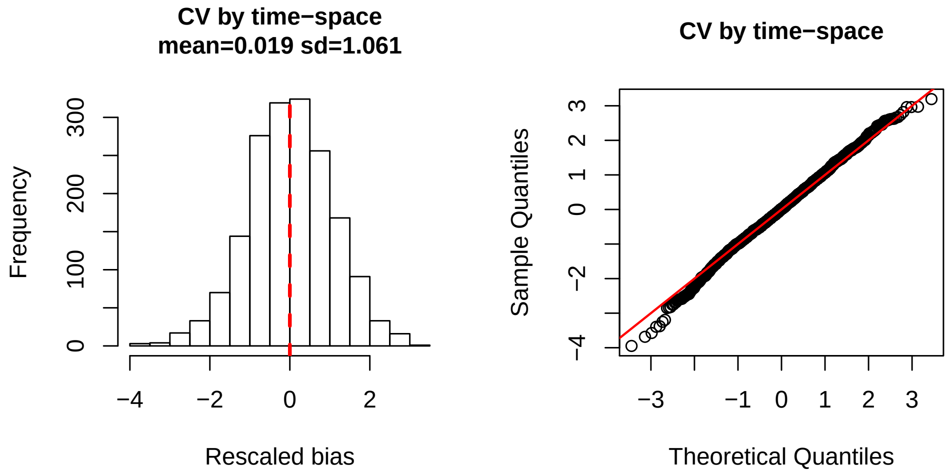

Figure S1.5: Histogram and QQ-plot of the rescaled difference between the smoothed estimates and the direct estimates. The differences between the two estimates are scaled by the square root of the total variance of the two estimates.

3.4 Benchmarking Summary

The final results were obtained with an additional adjustment step to account for the difference between the smoothed estimates and the estimates from the B3 model [1] on the national-level. Table S1.8 summarizes the adjustment factor $\hat{r}_t$, the ratio of the unadjusted direct estimates divided by the direct estimates after benchmarking, in each country and five year periods.

Table S1.7: Coverage of the 95% posterior credible interval for the logit of the direct estimates, mean and standard deviation of the rescaled bias under two cross validation schemes. The biases are scaled by the estimated standard deviation of the difference between the smoothed estimates and the direct estimates.

Country	coverage	Average bias	*sd*(bias)
Angola	0.94	0.06	1.11
Benin	0.94	0.01	1.01
Burkina Faso	1.00	0.00	0.75
Burundi	0.93	0.00	1.17
Cameroon	1.00	0.01	0.81
Chad	0.96	0.02	0.99
Comoros	1.00	-0.01	0.72
Congo	0.86	-0.00	1.33
Côte d'Ivoire	0.96	0.05	0.97
DRC	0.91	0.01	1.04
Egypt	0.96	-0.00	0.99
Ethiopia	0.92	0.02	1.06
Gabon	0.94	-0.00	1.11
Gambia	0.98	0.01	0.99
Ghana	0.94	0.02	1.08
Guinea	0.97	0.02	0.88
Kenya	0.95	0.00	1.06
Lesotho	0.99	0.03	0.87
Liberia	1.00	0.02	0.79
Madagascar	0.89	-0.01	1.18
Malawi	1.00	0.00	1.04
Mali	0.96	0.01	0.94
Morocco	0.97	-0.00	1.02
Mozambique	0.95	0.03	1.06
Namibia	0.90	0.06	1.09
Niger	0.89	-0.00	1.19
Nigeria	0.98	0.00	0.95
Rwanda	0.69	-0.02	1.58
Senegal	0.91	0.02	1.14
Sierra Leone	0.96	-0.01	0.97
Tanzania	0.90	0.03	1.24
Togo	0.95	0.01	1.00
Uganda	0.96	-0.01	1.06
Zambia	0.89	0.02	1.14
Zimbabwe	0.90	0.03	1.12
Average	0.94	0.01	1.04

Table S1.8: Ratio of the posterior median U5MR from the national model to median U5MR from B3 model for each 5-year period in each country before benchmarking. Values greater than 1 indicate the direct estimates are adjusted downwards after benchmarking, and vice versa.

Country	80-84	85-89	90-94	95-99	00-04	05-09	10-14	Average
Benin	1.05	1.02	1.00	1.03	1.00	0.88		1.00
Angola	1.58	1.08	0.98	0.94	0.76	0.54	0.39	0.90
Burkina Faso	1.02	0.98	1.00	1.01	0.99	0.95		0.99
Burundi	1.07	1.05	1.12	1.27	1.26	0.89		1.11
Cameroon	0.94	0.95	1.01	0.97	1.02	1.09	1.06	1.01
Chad	0.93	0.86	0.90	1.00	0.97	0.96	0.86	0.92
Comoros	0.94	0.86	0.83	0.69	0.53	0.50	0.66	0.72
Congo	0.95	0.98	0.99	1.08	0.98	0.90	1.22	1.01
Côte d'Ivoire	0.83	0.92	0.91	0.94	0.98	0.98	0.94	0.93
DRC	0.81	0.83	0.98	1.01	0.95	0.94	0.96	0.93
Egypt	0.98	0.95	1.00	0.96	0.94	0.97		0.97
Ethiopia	1.01	0.99	1.04	0.99	1.00	1.03	1.13	1.03
Gabon	0.95	0.86	0.96	0.88	0.74	0.78	1.38	0.94
Gambia	0.77	0.67	0.67	0.70	0.76	0.73	0.67	0.71
Ghana	0.98	0.96	0.95	0.95	0.96	0.98	0.88	0.95
Guinea	0.98	0.94	0.98	0.97	1.01	1.10	1.02	1.00
Kenya	0.97	0.94	0.94	0.88	0.88	0.83	0.92	0.91
Lesotho	0.82	0.86	0.97	0.83	0.95	0.96	0.91	0.90
Liberia	0.95	0.96	1.03	0.97	0.96	0.94	1.13	0.99
Madagascar	1.04	1.02	0.97	0.96	0.94	0.93		0.98
Malawi	0.98	0.96	0.95	0.95	0.97	0.97	0.86	0.95
Mali	0.96	0.99	0.99	1.04	1.00	1.08		1.01
Morocco	0.95	0.95	0.97	1.02	0.97			0.97
Mozambique	0.98	0.94	0.94	0.95	0.92	0.85	0.94	0.93
Namibia	0.88	0.87	0.92	0.85	0.97	1.02	1.06	0.94
Niger	0.98	0.97	0.95	0.96	1.00	0.97		0.97
Nigeria	1.00	1.01	0.99	1.02	1.02	1.03	0.97	1.01
Rwanda	0.98	1.01	1.16	0.96	0.99	0.94	0.92	1.00
Senegal	0.99	0.96	0.97	0.98	0.93	0.93	1.00	0.97
Sierra Leone	1.01	1.06	0.95	0.91	0.98	1.02	0.92	0.98
Tanzania	0.95	0.95	1.00	1.00	0.99	1.10	1.24	1.03
Togo	1.04	1.01	1.06	1.03	1.00	0.99	1.01	1.02
Uganda	0.96	0.90	0.90	0.95	1.01	1.11	1.34	1.03
Zambia	0.99	0.97	0.96	0.90	0.91	0.85	0.96	0.93
Zimbabwe	0.97	0.92	0.84	0.74	0.72	0.86		0.84
Average	0.98	0.95	0.97	0.95	0.94	0.93	0.98	0.96

3.5 All Results by Country

3.5.1 Angola

DHS surveys were conducted in Angola in 2015.

We fit both the RW2 only model to the combined national data, and compare the time trend at national level with the estimates produced by the UN and IHME in Figure S1.6. We then adjusted the combined national data to the UN estimates of U5MR, and refit the models on the benchmarked data.

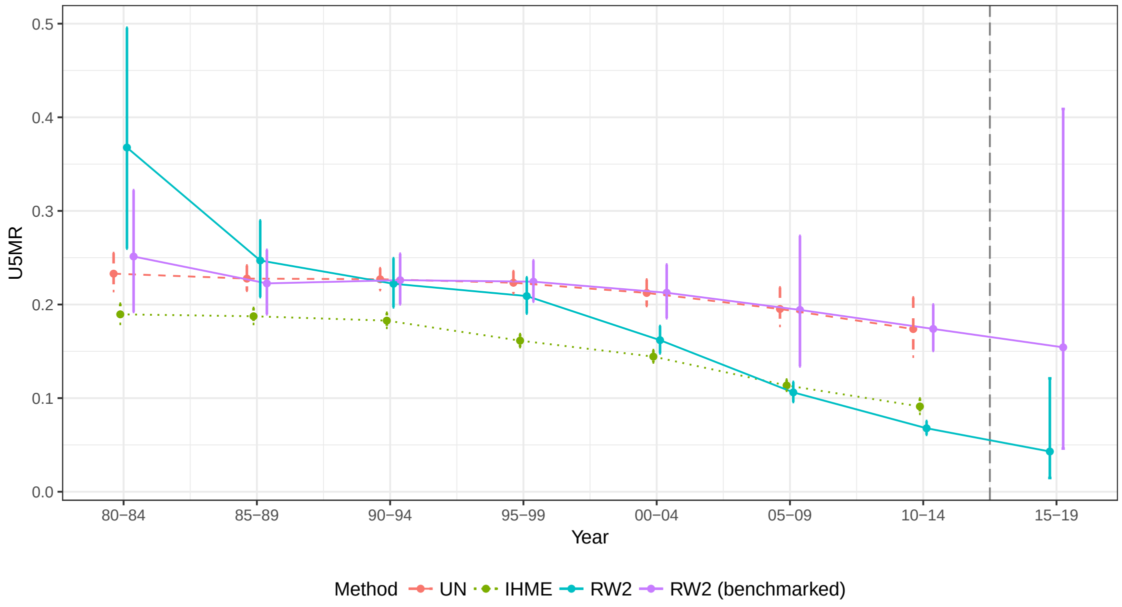

Figure S1.6: Angola: Temporal national trends along with UN (B3) estimates described in You et al. (2015) and IHME estimates based on GBD 2015 Child Mortality Collaborators (2016). RW2 represents the smoothed national estimates using the original data before benchmarking with UN estimates. RW2-adj represents the smoothed national estimates using the benchmarked data.

We fit the RW2 model to the benchmarked data in each area. We compare the results in Figure S1.7 to S1.11. Figure S1.7 compares the smoothed estimates against the direct estimates. Figure S1.8 and Figure S1.9 show the posterior median estimates of U5MR in each region over time and the reductions from 1990 period respectively. Figure S1.10 shows the smoothed estimates by region over time and Figure S1.11 compares the smoothed estimates with direct estimates from each survey for each region over time.

We further assess the RW2 model by holding out some observations, and compare the projections to the direct estimates in these holdout observations. Figure S1.12 compares the predicted estimates for the out-of-sample observations with the direct estimates by holding out observations from each area in each time period. Figure S1.13 compares the histogram of the bias rescaled by the total variance in the cross validation studies. Figure S1.14 compares the rescaled bias by region and time

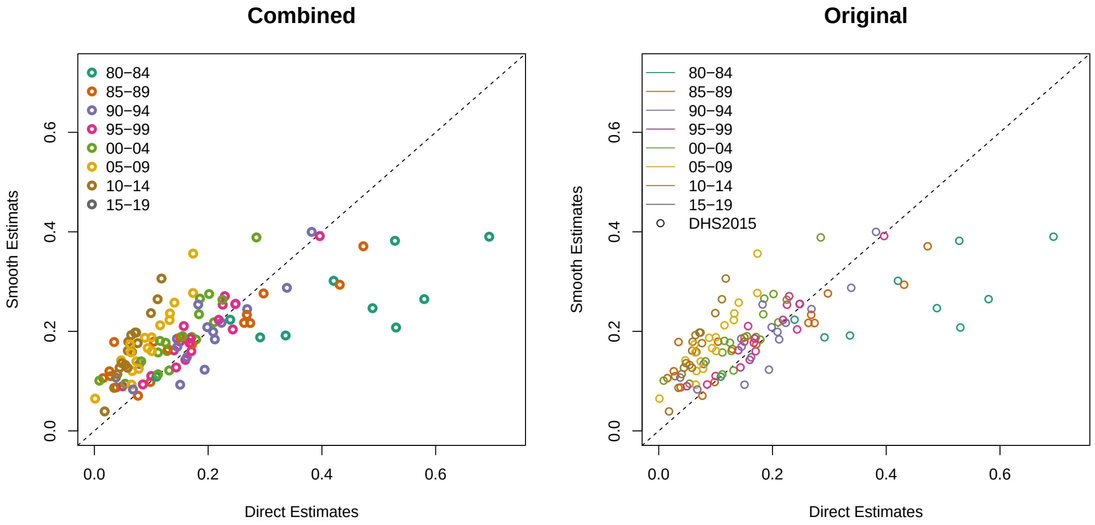

Figure S1.7: Angola: Smooth versus direct Admin 1 estimates. Left: Combined (meta-analysis) survey estimate against combined direct estimates. Right: Combined (meta-analysis) survey estimate against direct estimates from each survey.

periods.

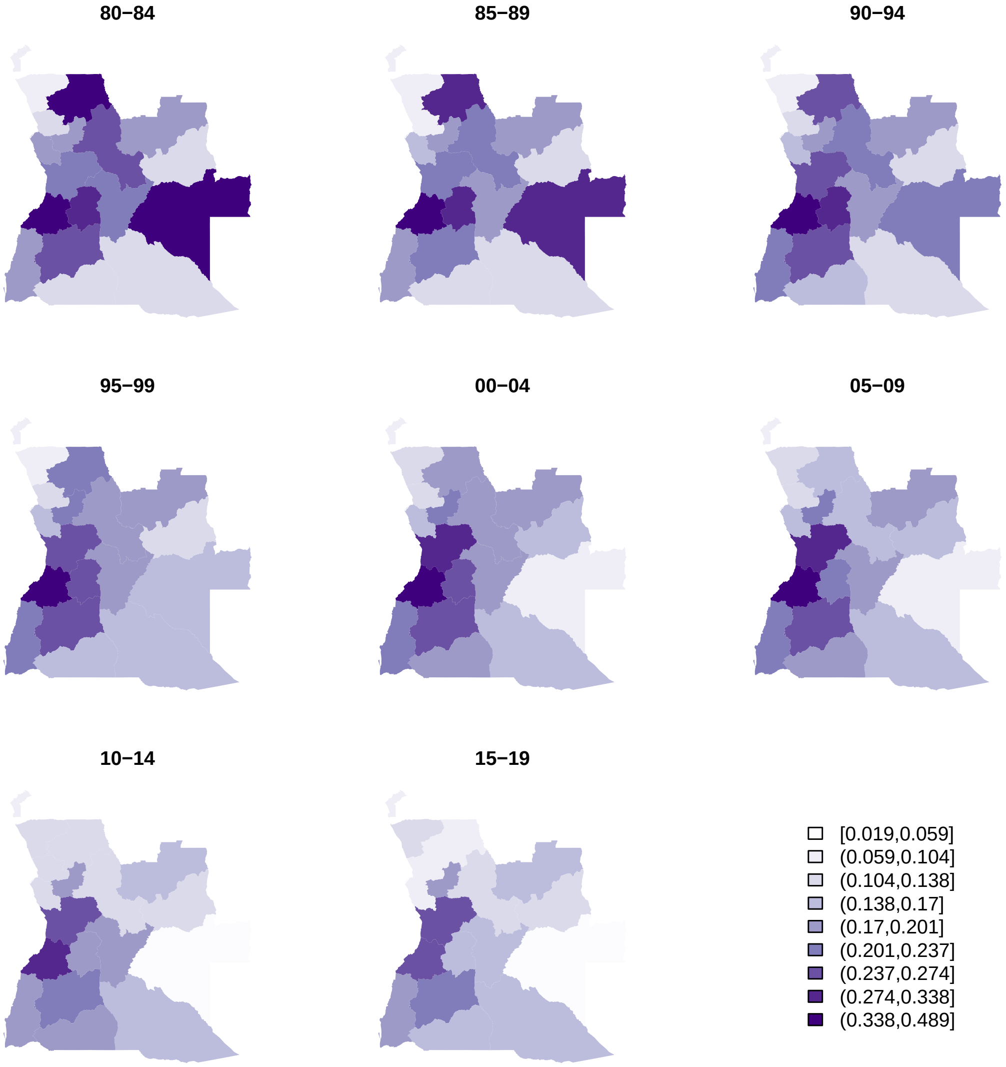

Figure S1.8: Angola: Maps of posterior medians over time.

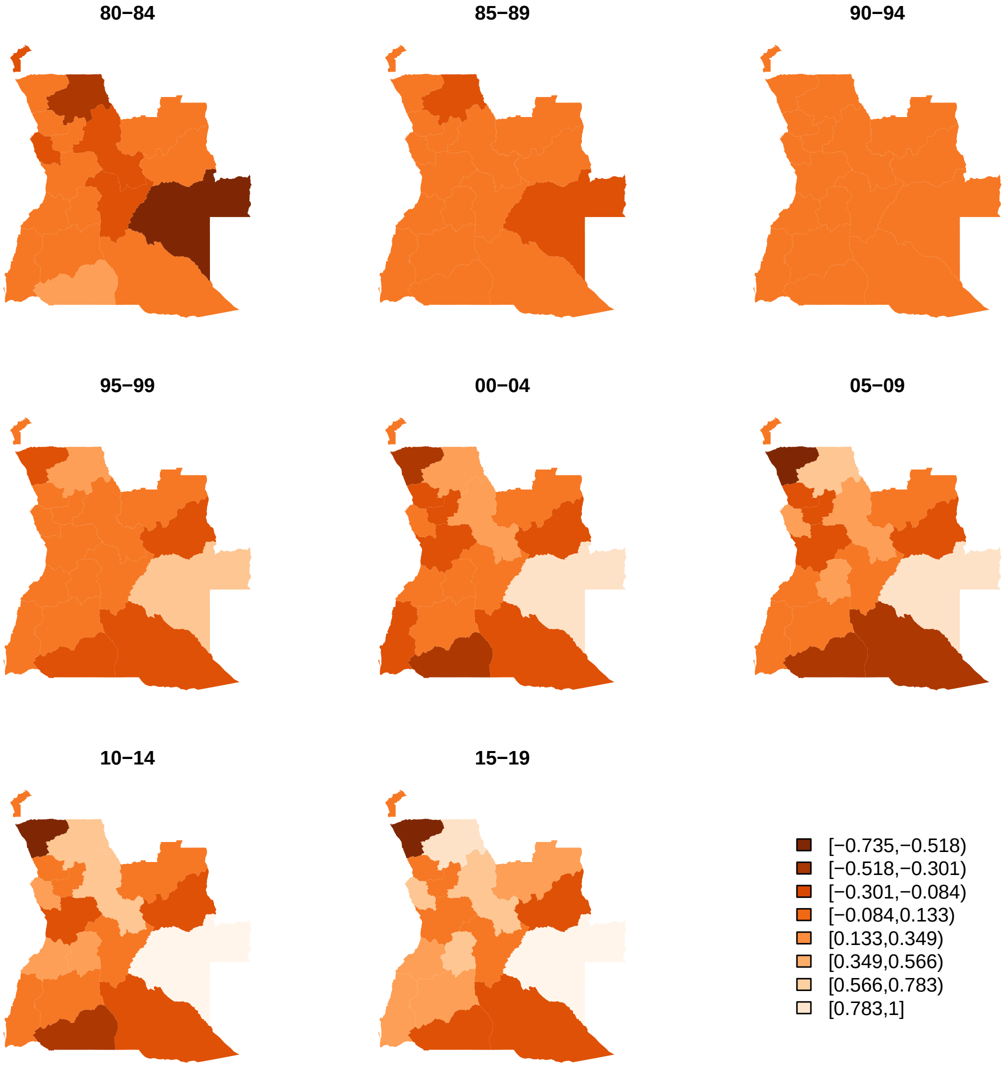

Figure S1.9: Angola: Maps of reduction of posterior median U5MR in each five-year period compared to 1990 over time.

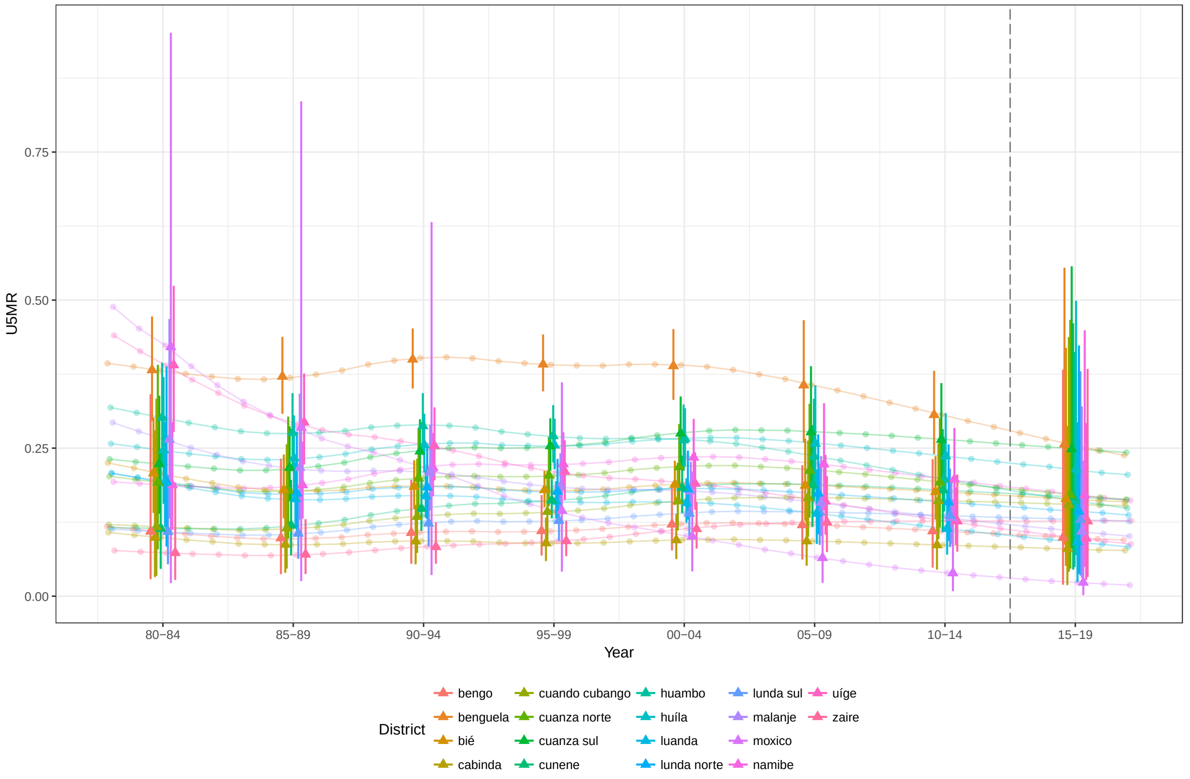

Figure S1.10: Angola: Smoothed regional estimates over time. The line indicates yearly posterior median estimates and error bars indicate 95 % posterior credible interval at each time period.

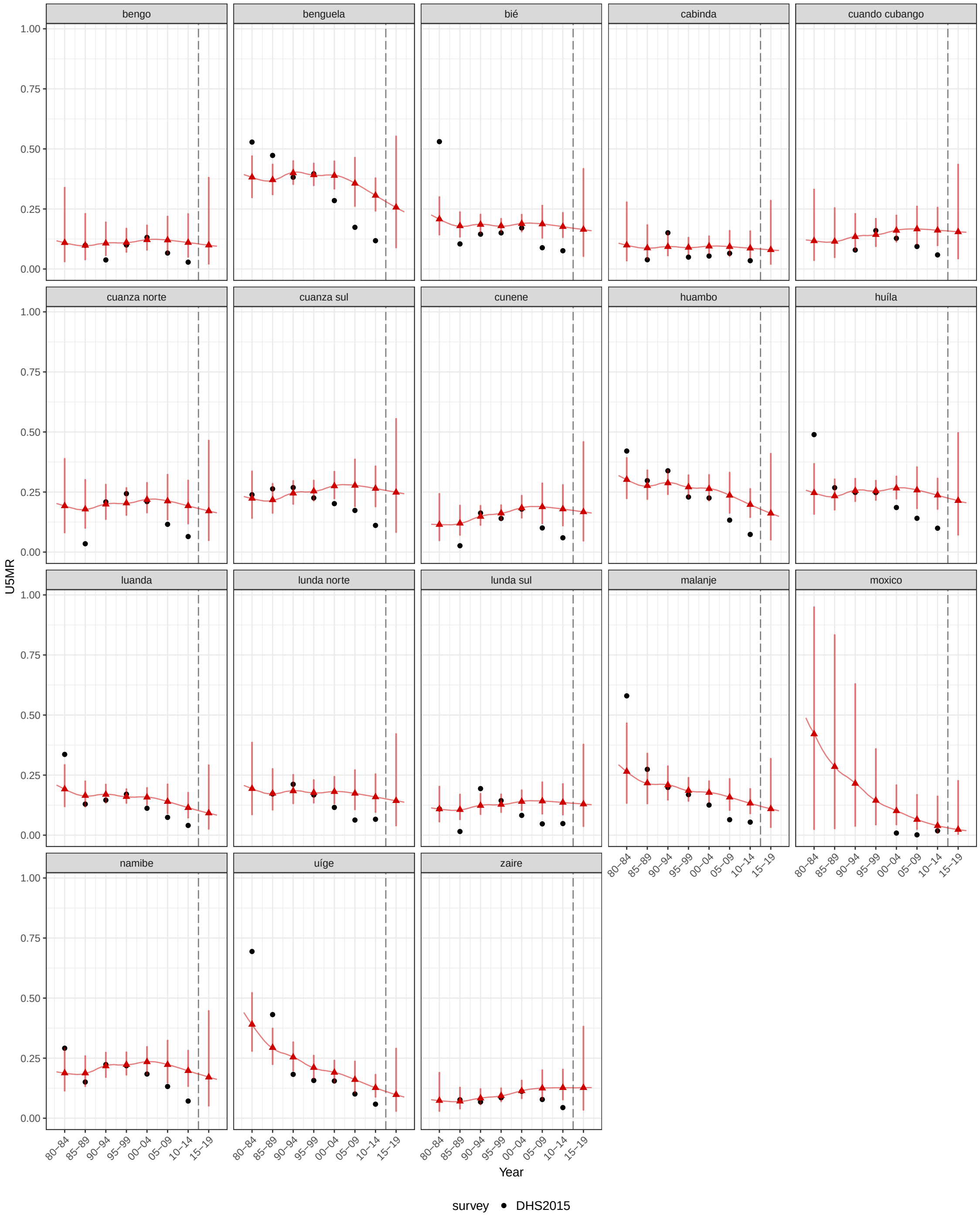

Figure S1.11: Angola: Smoothed regional estimates over time compared to the direct estimates from each surveys. Direct estimates are not benchmarked with UN estimates. The line indicates posterior median and error bars indicate 95% posterior credible interval.

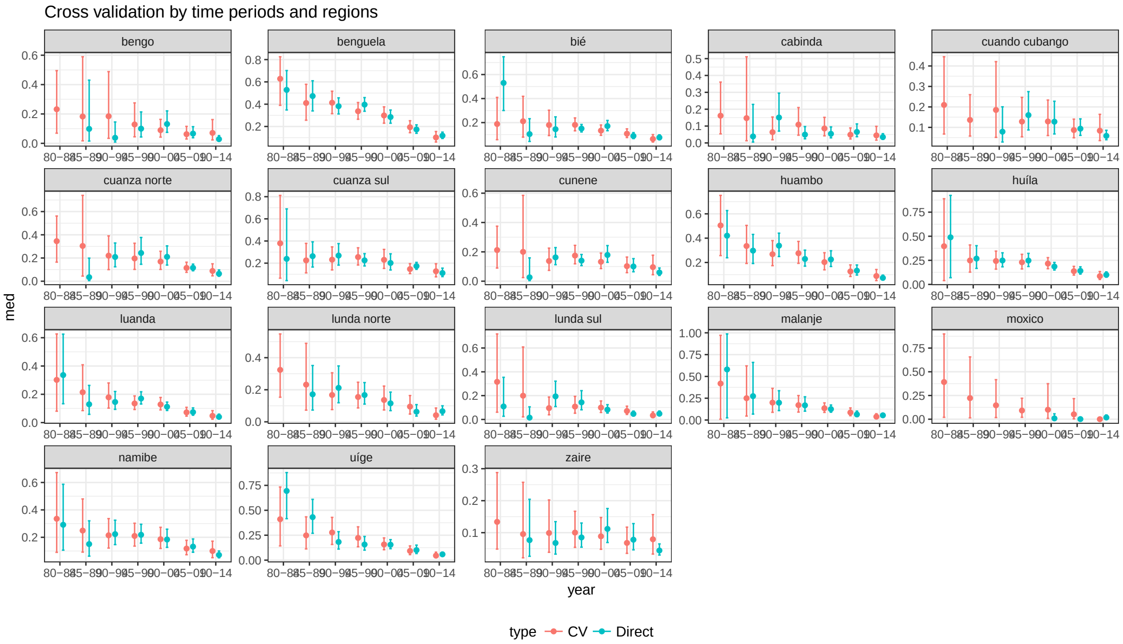

Figure S1.12: Angola: Out-of-sample predictions along with direct estimates in the cross validation study where data from one region in each time period is held out and predicted using the rest of the data.

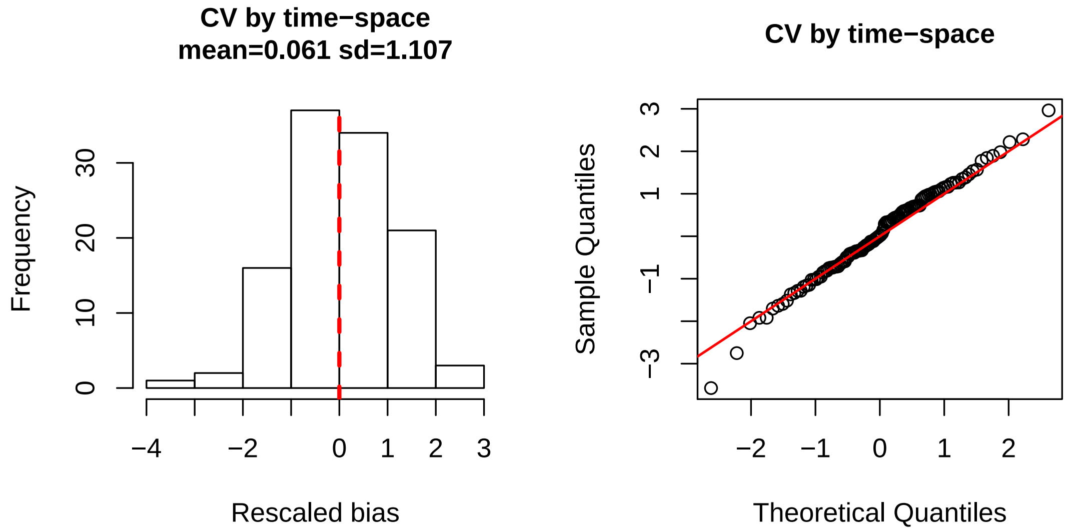

Figure S1.13: Angola: Histogram and QQ-plot of the rescaled difference between the smoothed estimates and the direct estimates in the cross validation study. The differences between the two estimates are rescaled by the square root of the total variance of the two estimates.

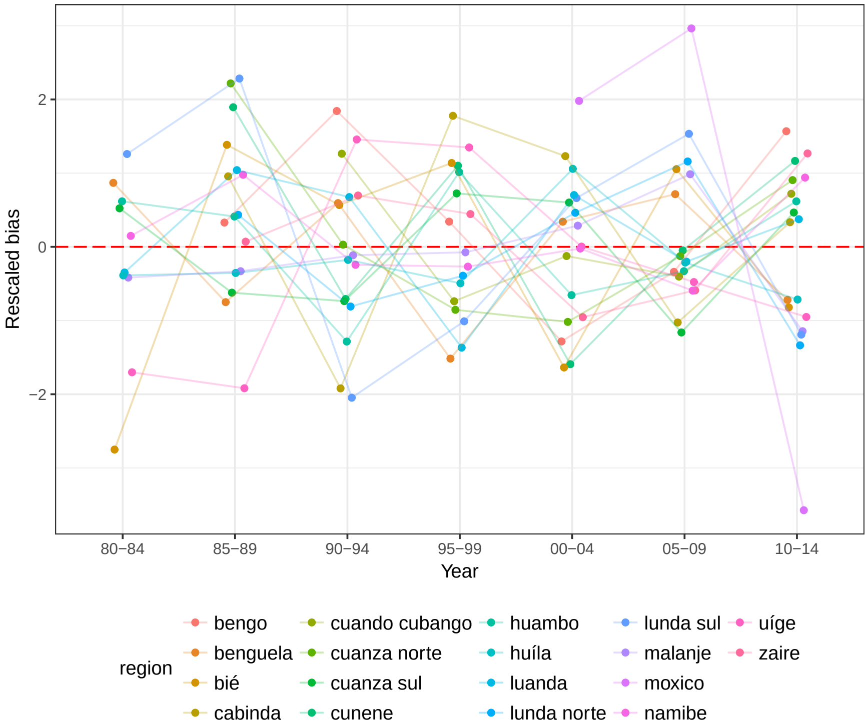

Figure S1.14: Angola: Line plot of the difference between smoothed estimates and the direct estimates in the cross validation study. The differences between the two estimates are rescaled by the square root of the total variance of the two estimates.

3.5.2 Benin

DHS surveys were conducted in Benin in 1996, 2001, and 2006.

We fit both the RW2 only model to the combined national data, and compare the time trend at national level with the estimates produced by the UN and IHME in Figure S1.15. We then adjusted the combined national data to the UN estimates of U5MR, and refit the models on the benchmarked data.

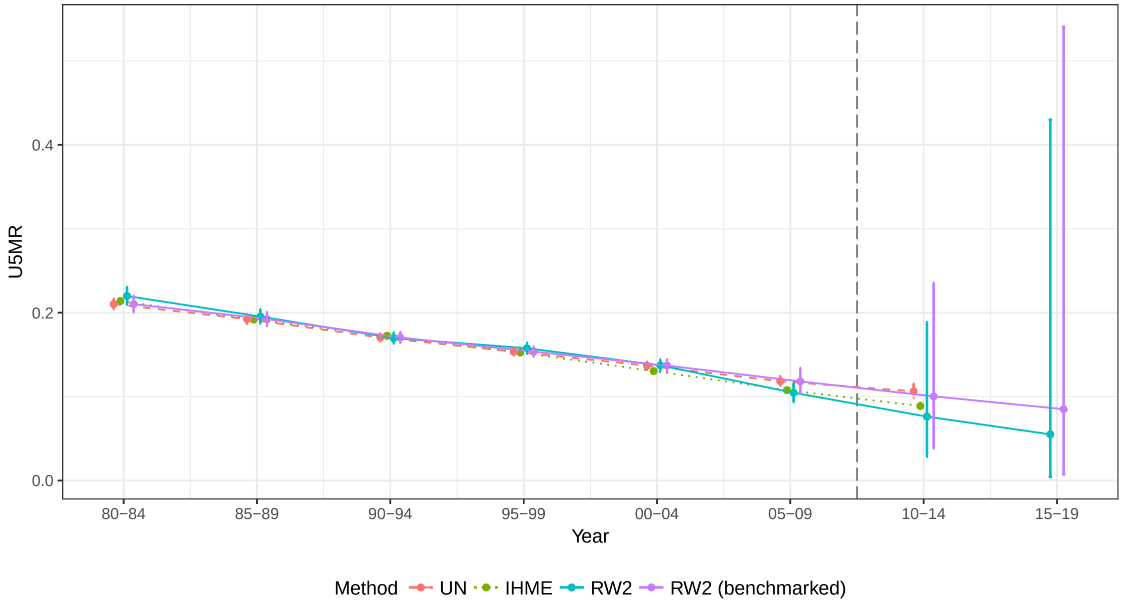

Figure S1.15: Benin: Temporal national trends along with UN (B3) estimates described in You et al. (2015) and IHME estimates based on GBD 2015 Child Mortality Collaborators (2016). RW2 represents the smoothed national estimates using the original data before benchmarking with UN estimates. RW2-adj represents the smoothed national estimates using the benchmarked data.

We fit the RW2 model to the benchmarked data in each area. We compare the results in Figure S1.16 to S1.20. Figure S1.16 compares the smoothed estimates against the direct estimates. Figure S1.17 and Figure S1.18 show the posterior median estimates of U5MR in each region over time and the reductions from 1990 period respectively. Figure S1.19 shows the smoothed estimates by region over time and Figure S1.20 compares the smoothed estimates with direct estimates from each survey for each region over time.

We further assess the RW2 model by holding out some observations, and compare the projections to the direct estimates in these holdout observations. Figure S1.21 compares the predicted estimates for the out-of-sample observations with the direct estimates by holding out observations from each area in each time period. Figure S1.22 compares the histogram of the bias rescaled by the total variance in the cross validation studies. Figure S1.23 compares the rescaled bias by region and time periods.

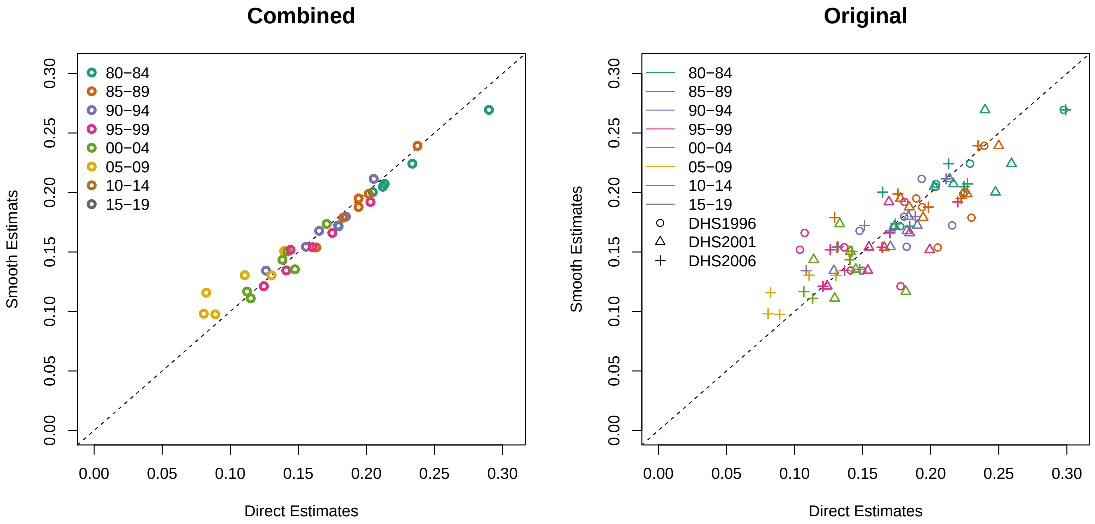

Figure S1.16: Benin: Smooth versus direct Admin 1 estimates. Left: Combined (meta-analysis) survey estimate against combined direct estimates. Right: Combined (meta-analysis) survey estimate against direct estimates from each survey.

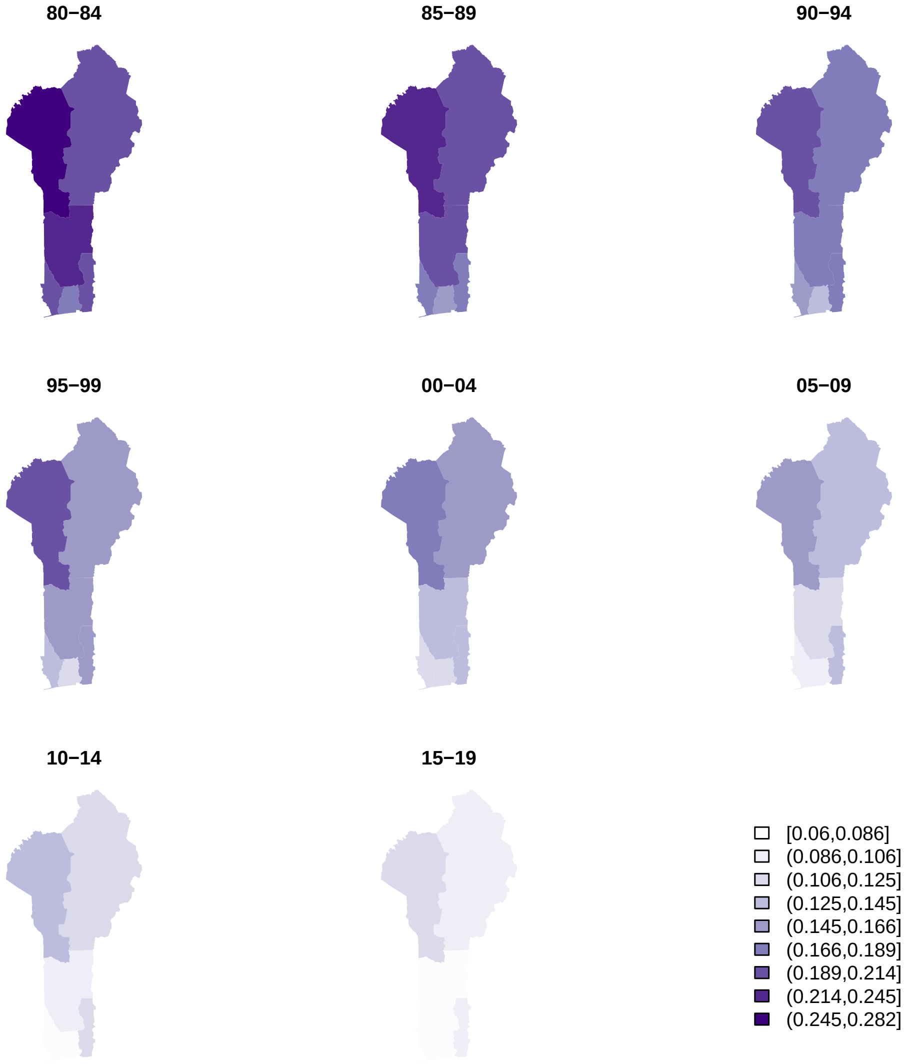

Figure S1.17: Benin: Maps of posterior medians over time.

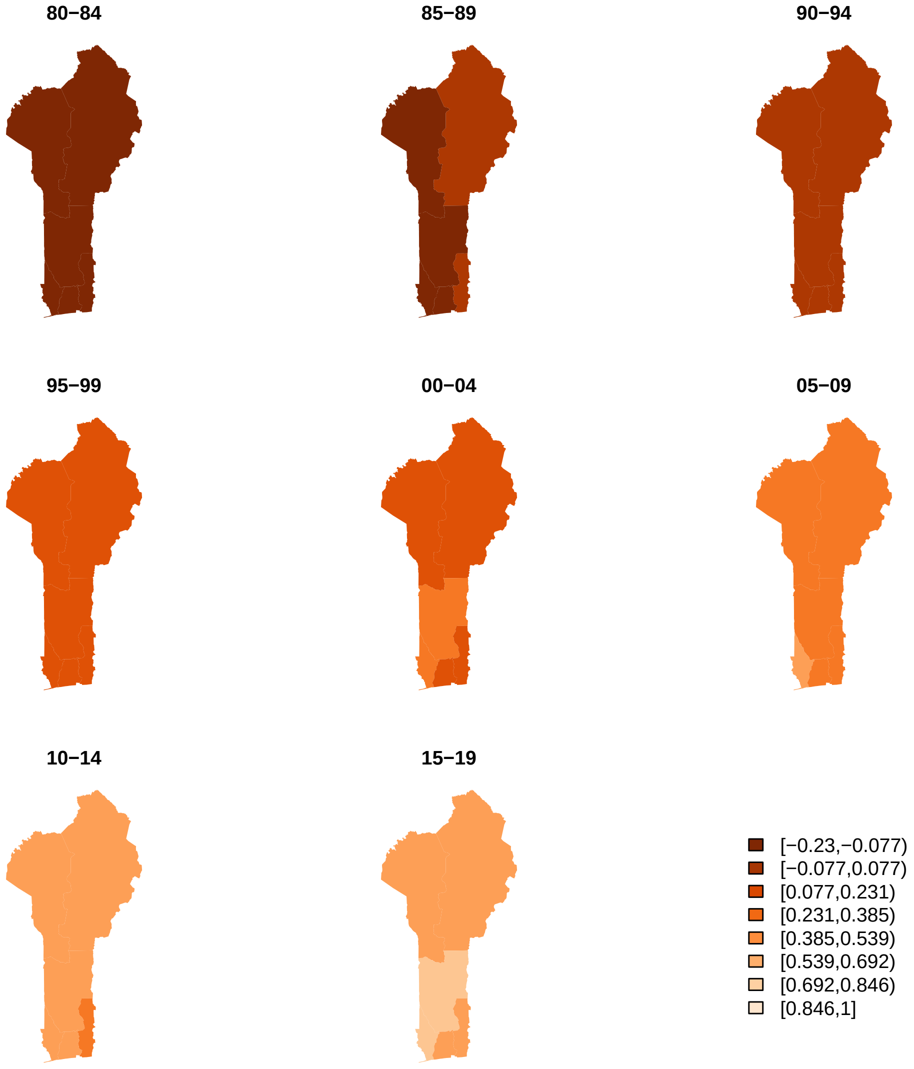

Figure S1.18: Benin: Maps of reduction of posterior median U5MR in each five-year period compared to 1990 over time.

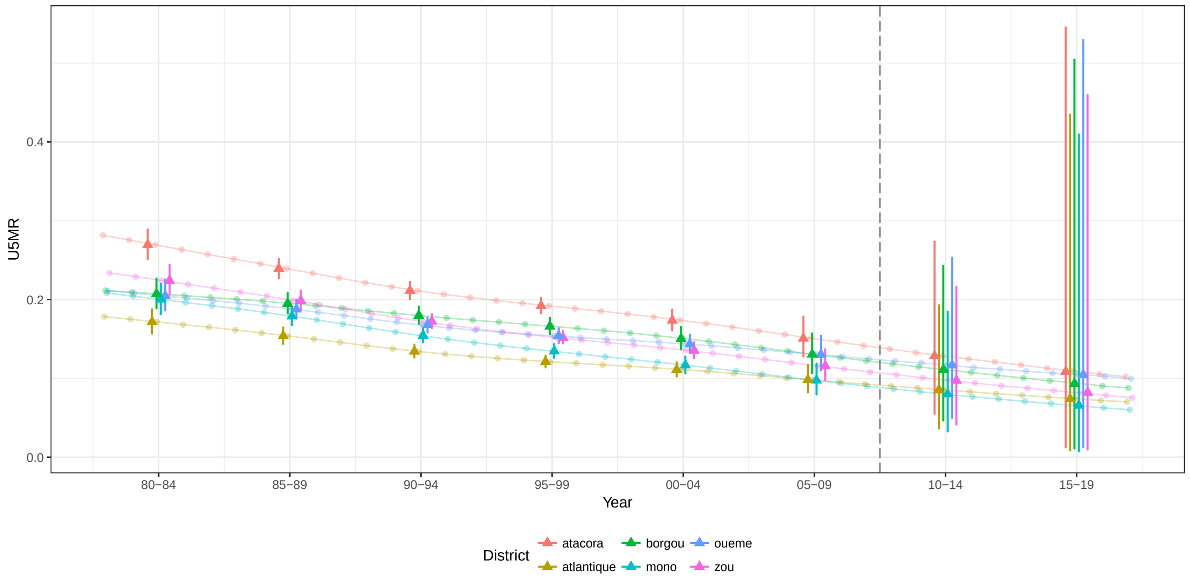

Figure S1.19: Benin: Smoothed regional estimates over time. The line indicates yearly posterior median estimates and error bars indicate 95 % posterior credible interval at each time period.

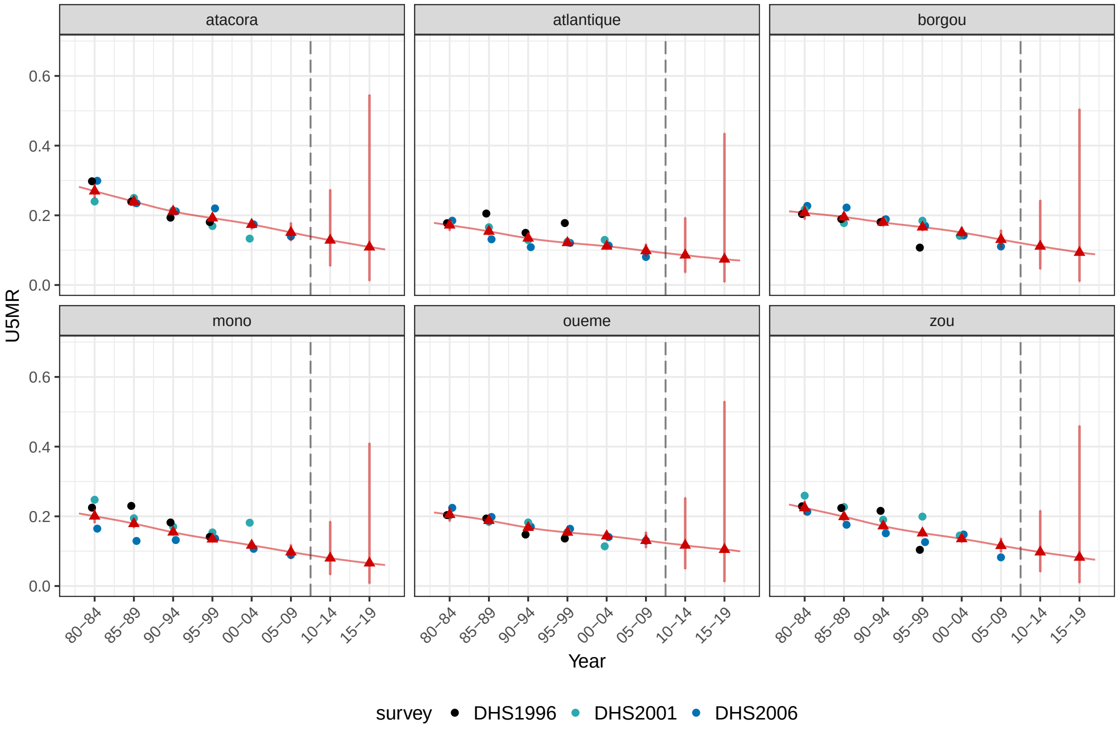

Figure S1.20: Benin: Smoothed regional estimates over time compared to the direct estimates from each surveys. Direct estimates are not benchmarked with UN estimates. The line indicates posterior median and error bars indicate 95% posterior credible interval.

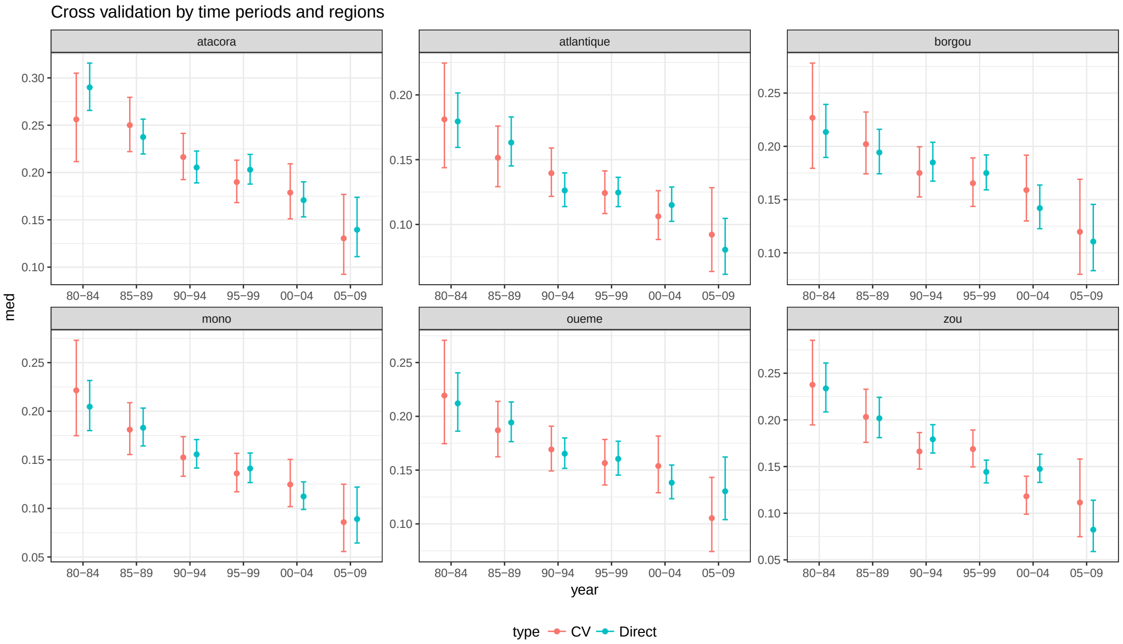

Figure S1.21: Benin: Out-of-sample predictions along with direct estimates in the cross validation study where data from one region in each time period is held out and predicted using the rest of the data.

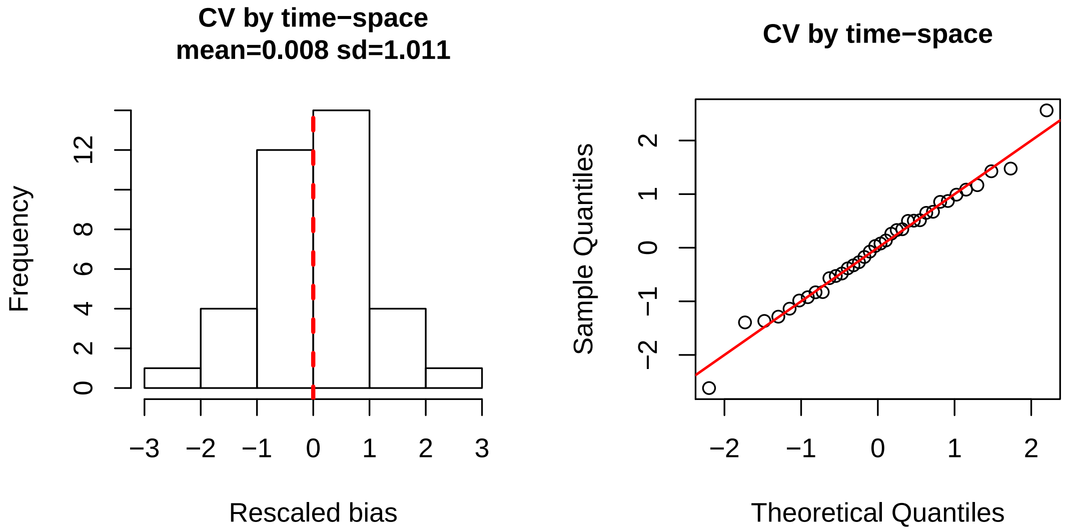

Figure S1.22: Benin: Histogram and QQ-plot of the rescaled difference between the smoothed estimates and the direct estimates in the cross validation study. The differences between the two estimates are rescaled by the square root of the total variance of the two estimates.

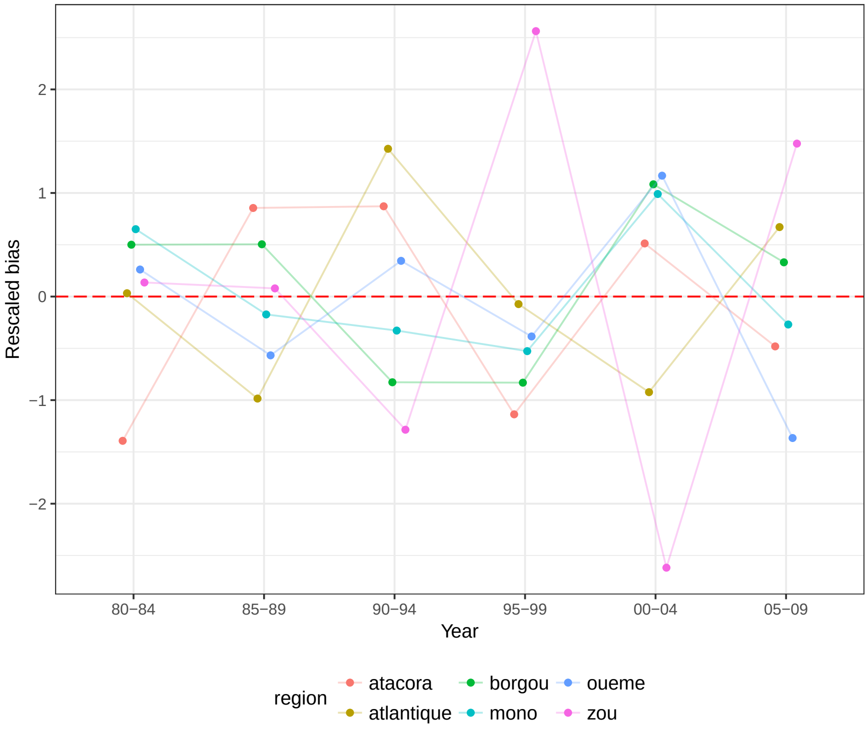

Figure S1.23: Benin: Line plot of the difference between smoothed estimates and the direct estimates in the cross validation study. The differences between the two estimates are rescaled by the square root of the total variance of the two estimates.

3.5.3 Burkina Faso

DHS surveys were conducted in Burkina Faso in 1993, 1999, 2003, and 2010.

We fit both the RW2 only model to the combined national data, and compare the time trend at national level with the estimates produced by the UN and IHME in Figure S1.24. We then adjusted the combined national data to the UN estimates of U5MR, and refit the models on the benchmarked data.

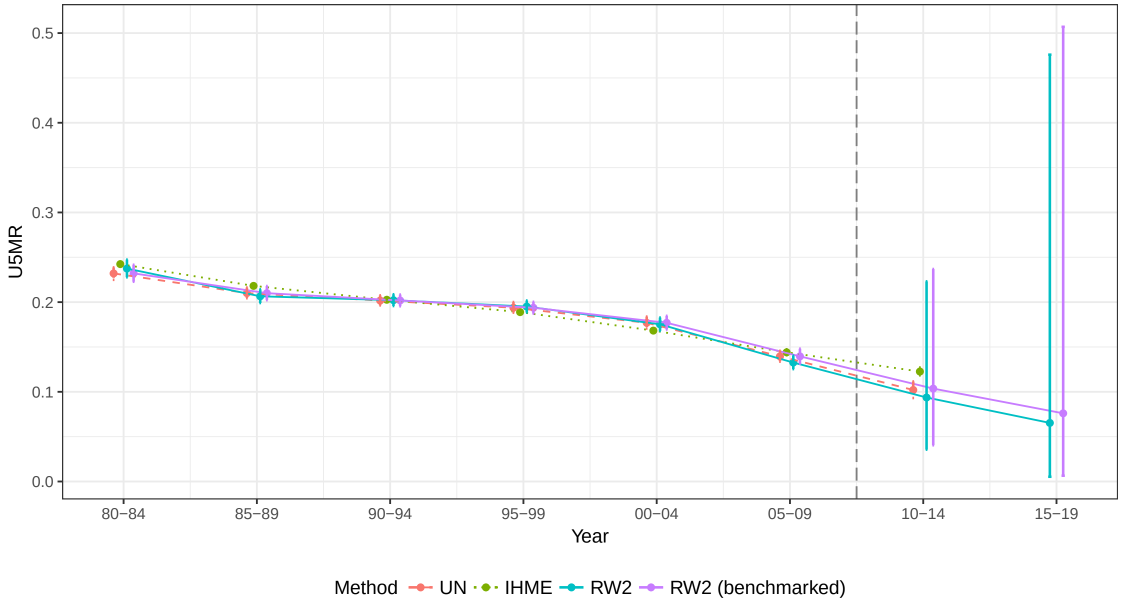

Figure S1.24: Burkina Faso: Temporal national trends along with UN (B3) estimates described in You et al. (2015) and IHME estimates based on GBD 2015 Child Mortality Collaborators (2016). RW2 represents the smoothed national estimates using the original data before benchmarking with UN estimates. RW2-adj represents the smoothed national estimates using the benchmarked data.

We fit the RW2 model to the benchmarked data in each area. We compare the results in Figure S1.25 to S1.29. Figure S1.25 compares the smoothed estimates against the direct estimates. Figure S1.26 and Figure S1.27 show the posterior median estimates of U5MR in each region over time and the reductions from 1990 period respectively. Figure S1.28 shows the smoothed estimates by region over time and Figure S1.29 compares the smoothed estimates with direct estimates from each survey for each region over time.

We further assess the RW2 model by holding out some observations, and compare the projections to the direct estimates in these holdout observations. Figure S1.30 compares the predicted estimates for the out-of-sample observations with the direct estimates by holding out observations from each area in each time period. Figure S1.31 compares the histogram of the bias rescaled by the total variance in the cross validation studies. Figure S1.32 compares the rescaled bias by region and time periods.

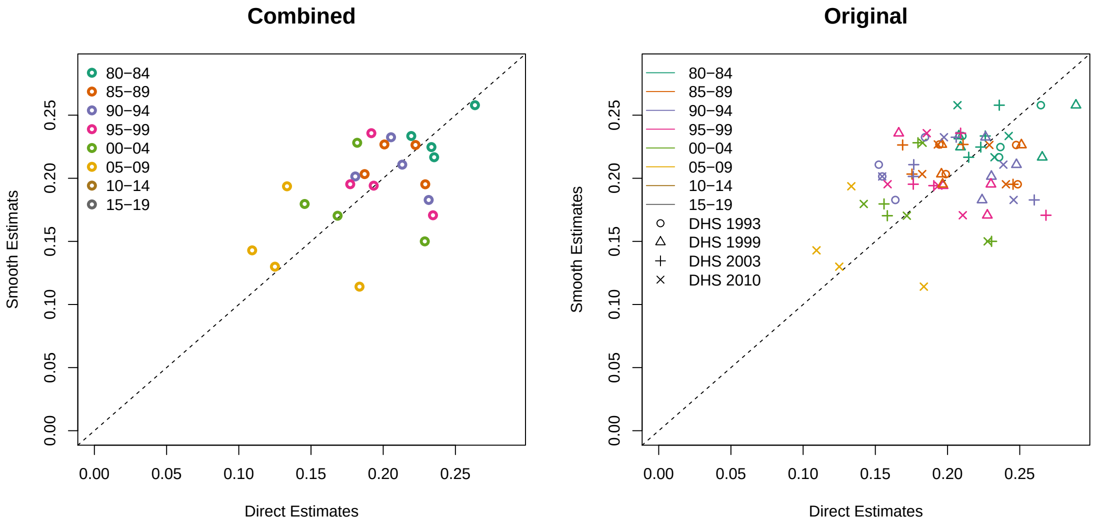

Figure S1.25: Burkina Faso: Smooth versus direct Admin 1 estimates. Left: Combined (meta-analysis) survey estimate against combined direct estimates. Right: Combined (meta-analysis) survey estimate against direct estimates from each survey.

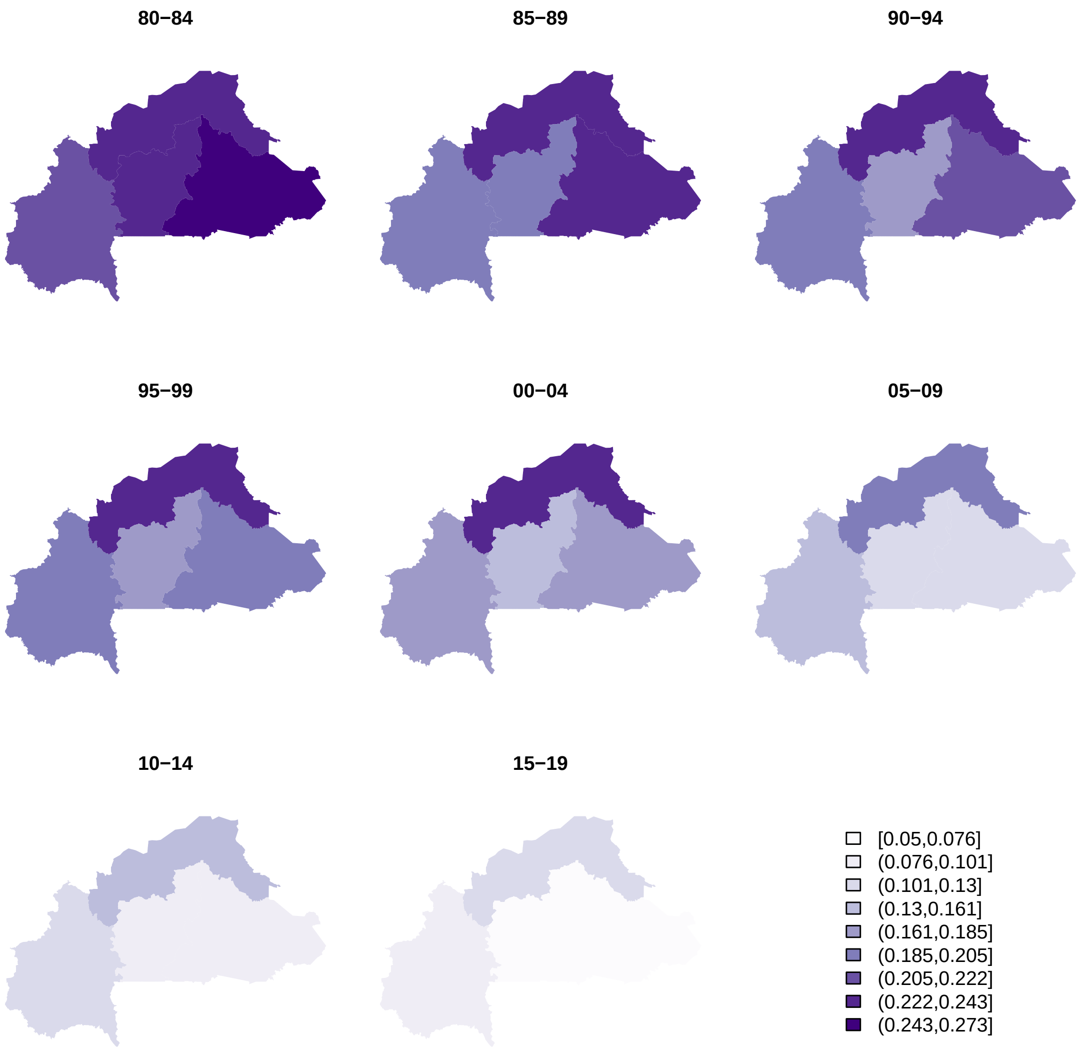

Figure S1.26: Burkina Faso: Maps of posterior medians over time.

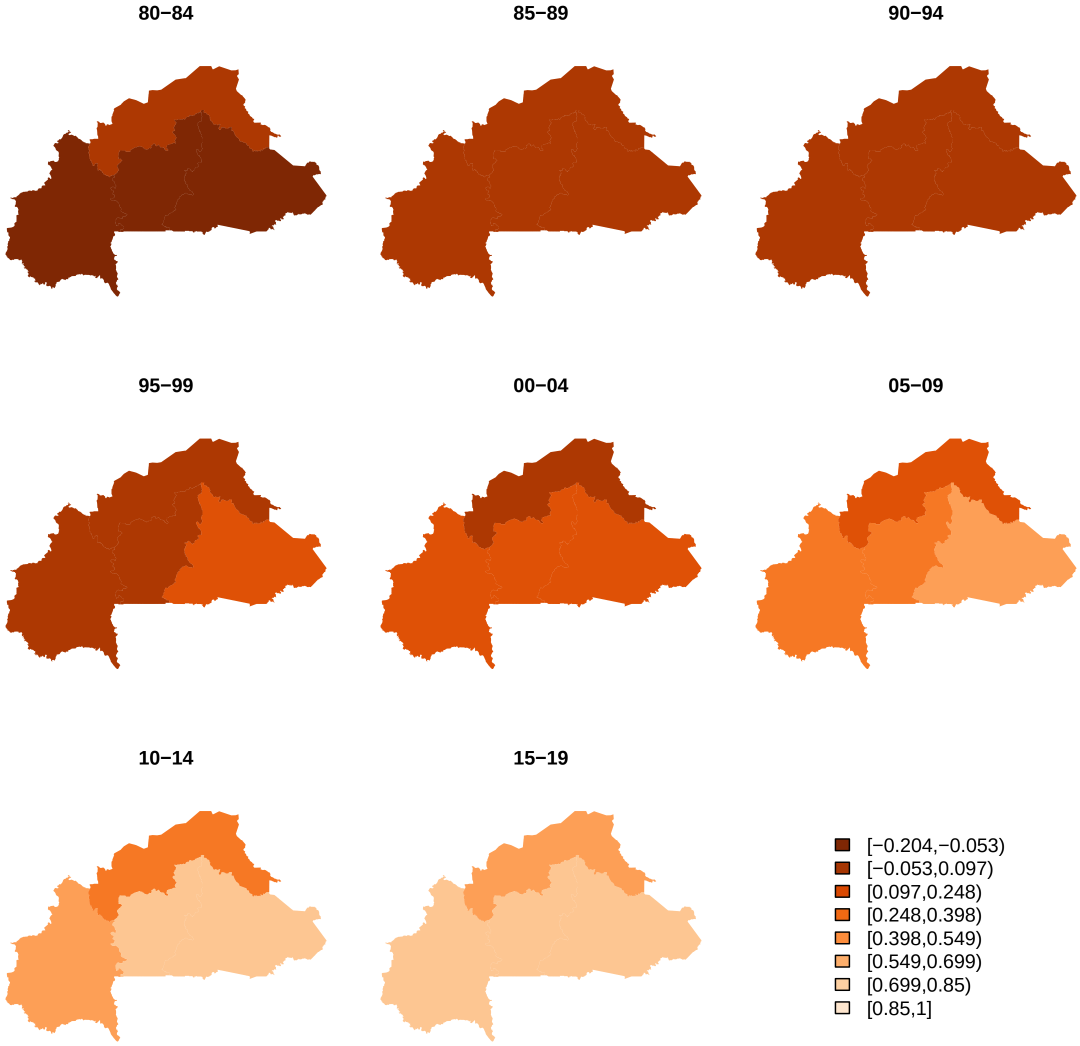

Figure S1.27: Burkina Faso: Maps of reduction of posterior median U5MR in each five-year period compared to 1990 over time.

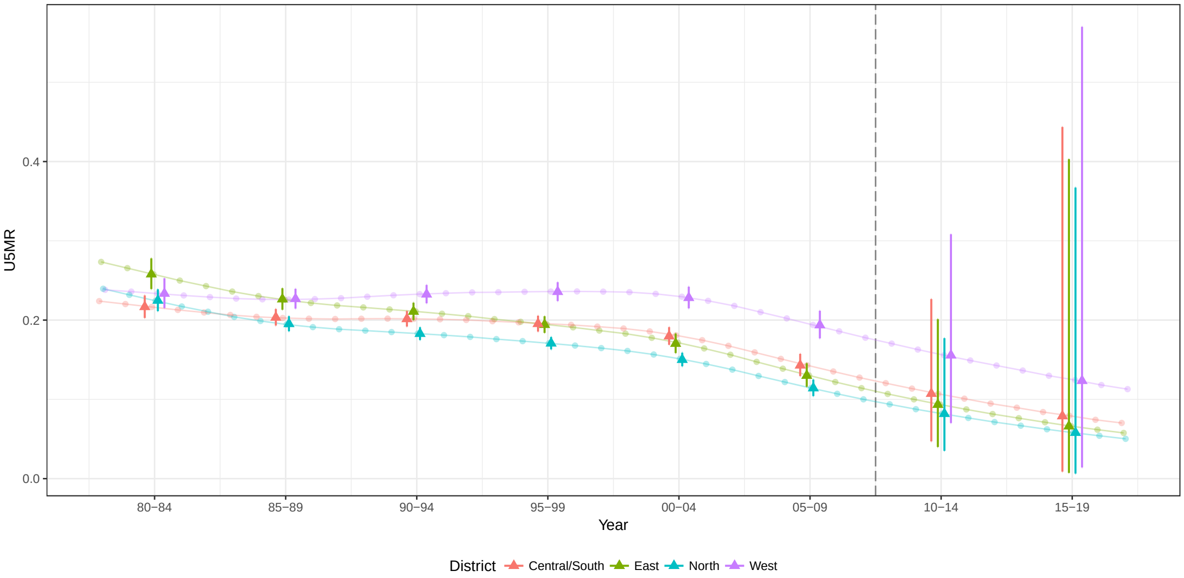

Figure S1.28: Burkina Faso: Smoothed regional estimates over time. The line indicates yearly posterior median estimates and error bars indicate 95 % posterior credible interval at each time period.

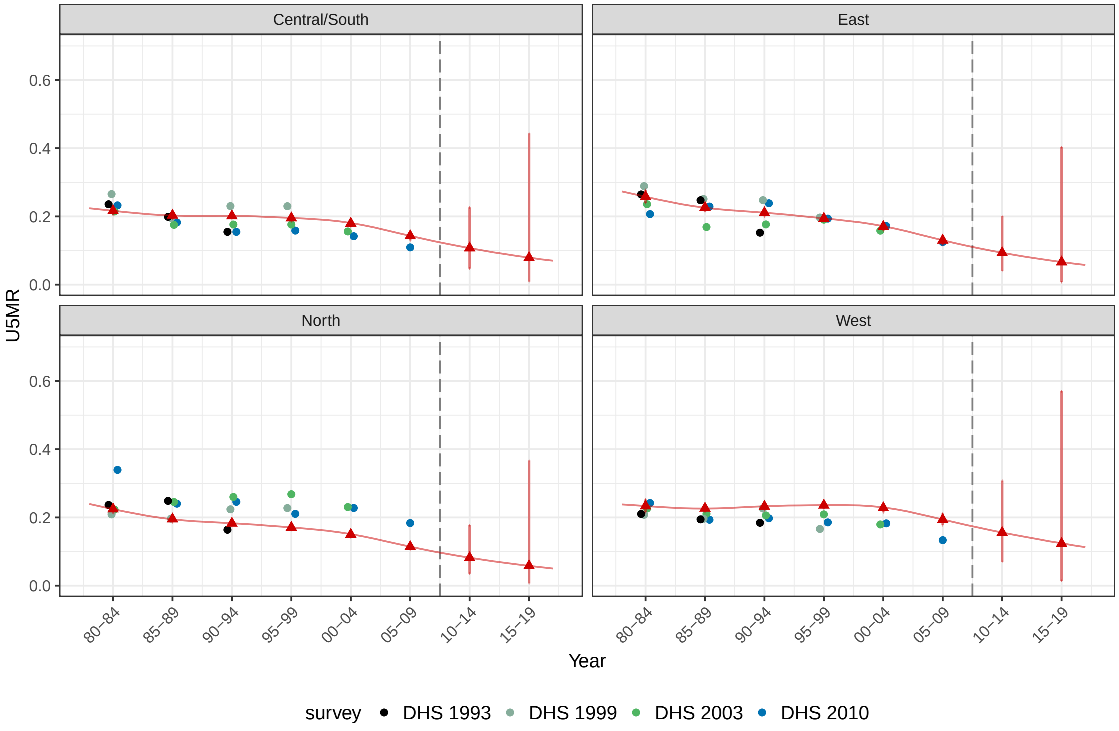

Figure S1.29: Burkina Faso: Smoothed regional estimates over time compared to the direct estimates from each surveys. Direct estimates are not benchmarked with UN estimates. The line indicates posterior median and error bars indicate 95% posterior credible interval.

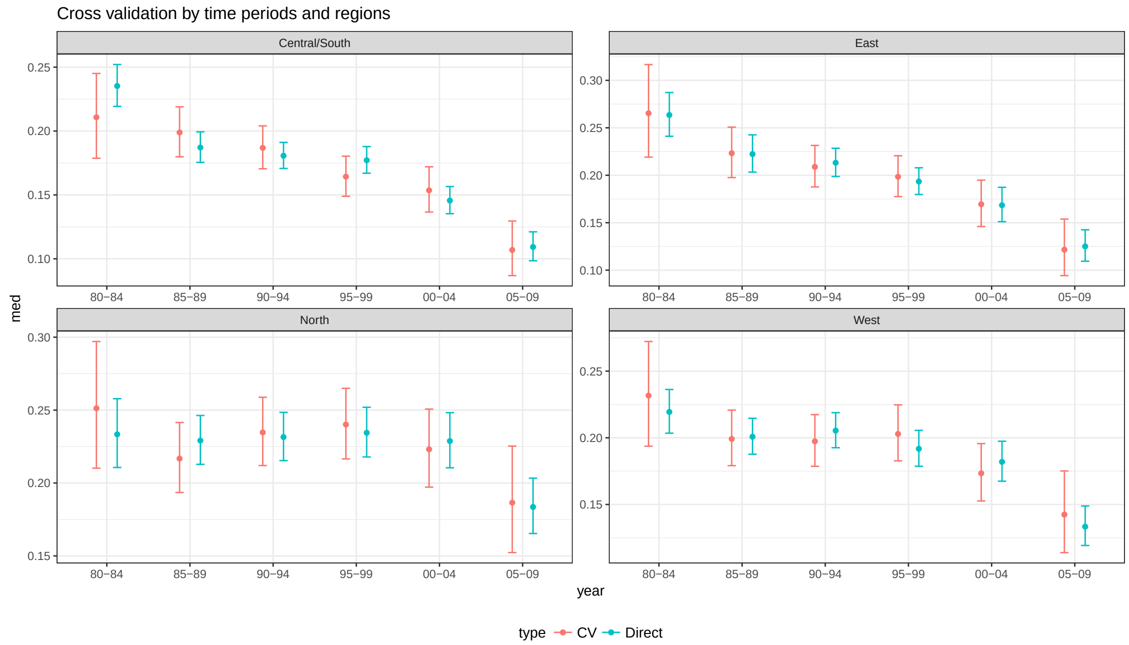

Figure S1.30: Burkina Faso: Out-of-sample predictions along with direct estimates in the cross validation study where data from one region in each time period is held out and predicted using the rest of the data.

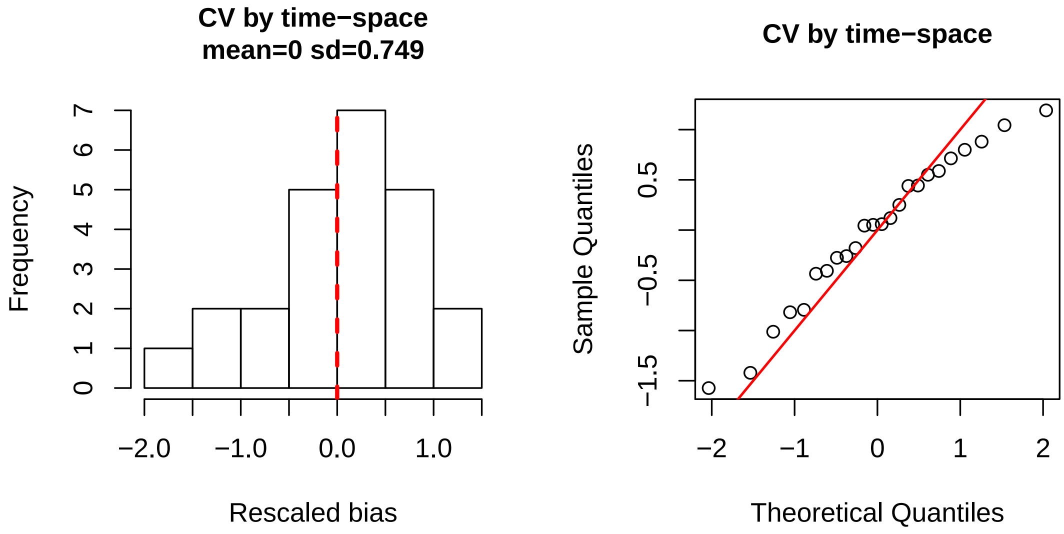

Figure S1.31: Burkina Faso: Histogram and QQ-plot of the rescaled difference between the smoothed estimates and the direct estimates in the cross validation study. The differences between the two estimates are rescaled by the square root of the total variance of the two estimates.

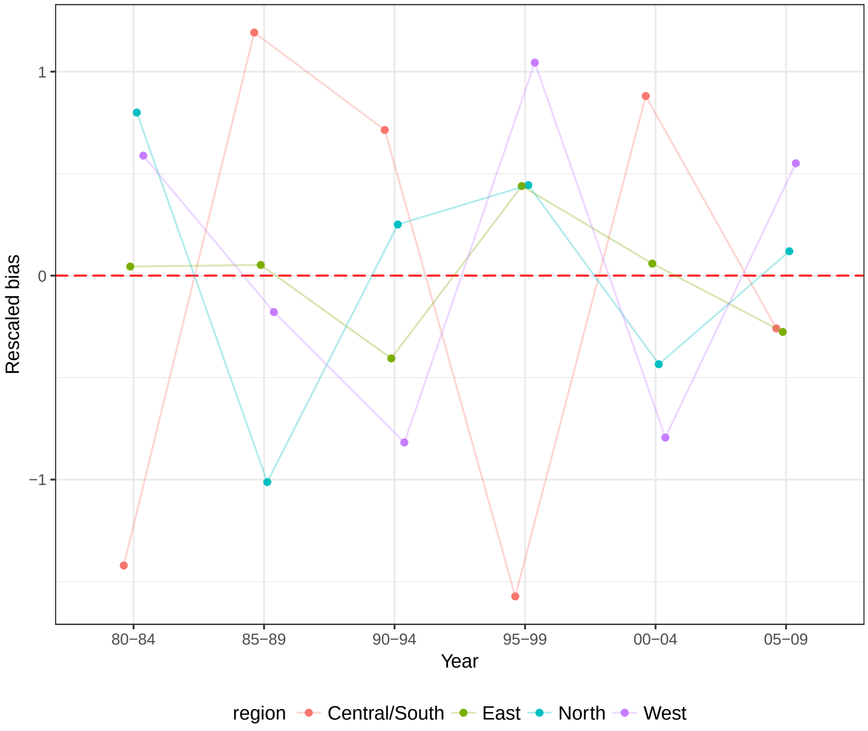

Figure S1.32: Burkina Faso: Line plot of the difference between smoothed estimates and the direct estimates in the cross validation study. The differences between the two estimates are rescaled by the square root of the total variance of the two estimates.

3.5.4 Burundi

DHS surveys were conducted in Burundi in 2010.

We fit both the RW2 only model to the combined national data, and compare the time trend at national level with the estimates produced by the UN and IHME in Figure S1.33. We then adjusted the combined national data to the UN estimates of U5MR, and refit the models on the benchmarked data.

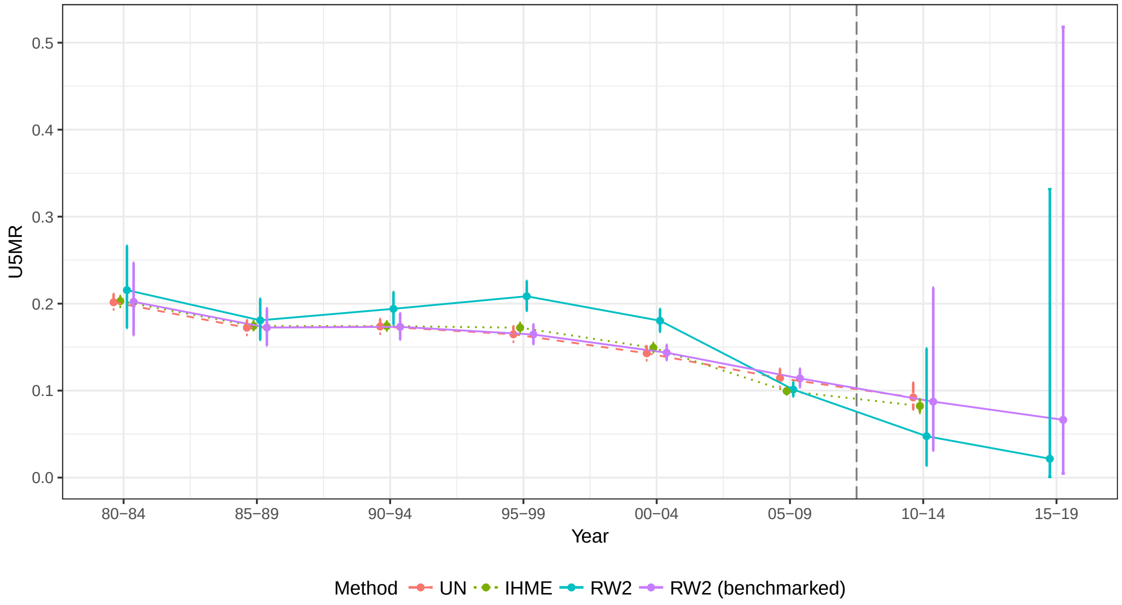

Figure S1.33: Burundi: Temporal national trends along with UN (B3) estimates described in You et al. (2015) and IHME estimates based on GBD 2015 Child Mortality Collaborators (2016). RW2 represents the smoothed national estimates using the original data before benchmarking with UN estimates. RW2-adj represents the smoothed national estimates using the benchmarked data.

We fit the RW2 model to the benchmarked data in each area. We compare the results in Figure S1.34 to S1.38. Figure S1.34 compares the smoothed estimates against the direct estimates. Figure S1.35 and Figure S1.36 show the posterior median estimates of U5MR in each region over time and the reductions from 1990 period respectively. Figure S1.37 shows the smoothed estimates by region over time and Figure S1.38 compares the smoothed estimates with direct estimates from each survey for each region over time.

We further assess the RW2 model by holding out some observations, and compare the projections to the direct estimates in these holdout observations. Figure S1.39 compares the predicted estimates for the out-of-sample observations with the direct estimates by holding out observations from each area in each time period. Figure S1.40 compares the histogram of the bias rescaled by the total variance in the cross validation studies. Figure S1.41 compares the rescaled bias by region and time periods.

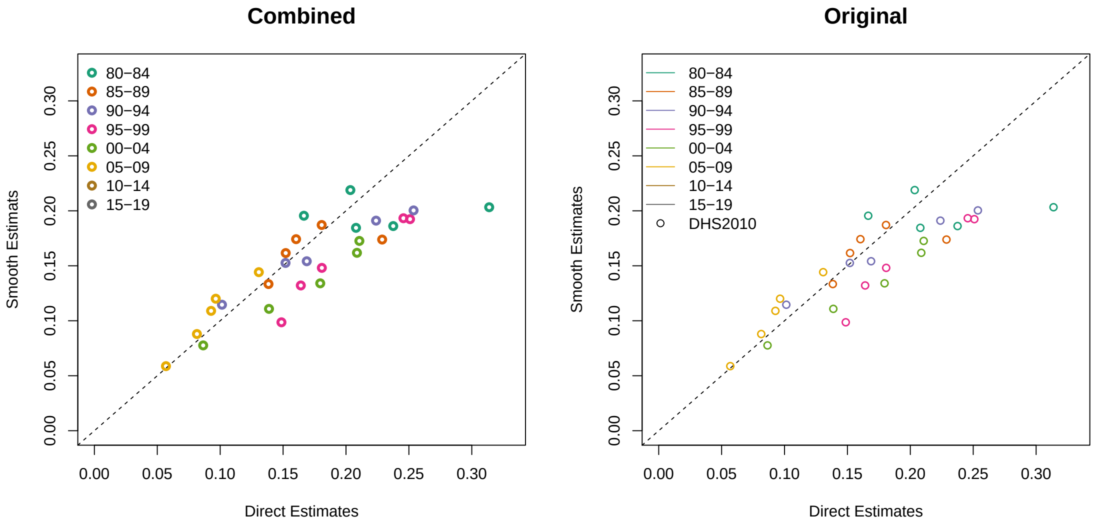

Figure S1.34: Burundi: Smooth versus direct Admin 1 estimates. Left: Combined (meta-analysis) survey estimate against combined direct estimates. Right: Combined (meta-analysis) survey estimate against direct estimates from each survey.

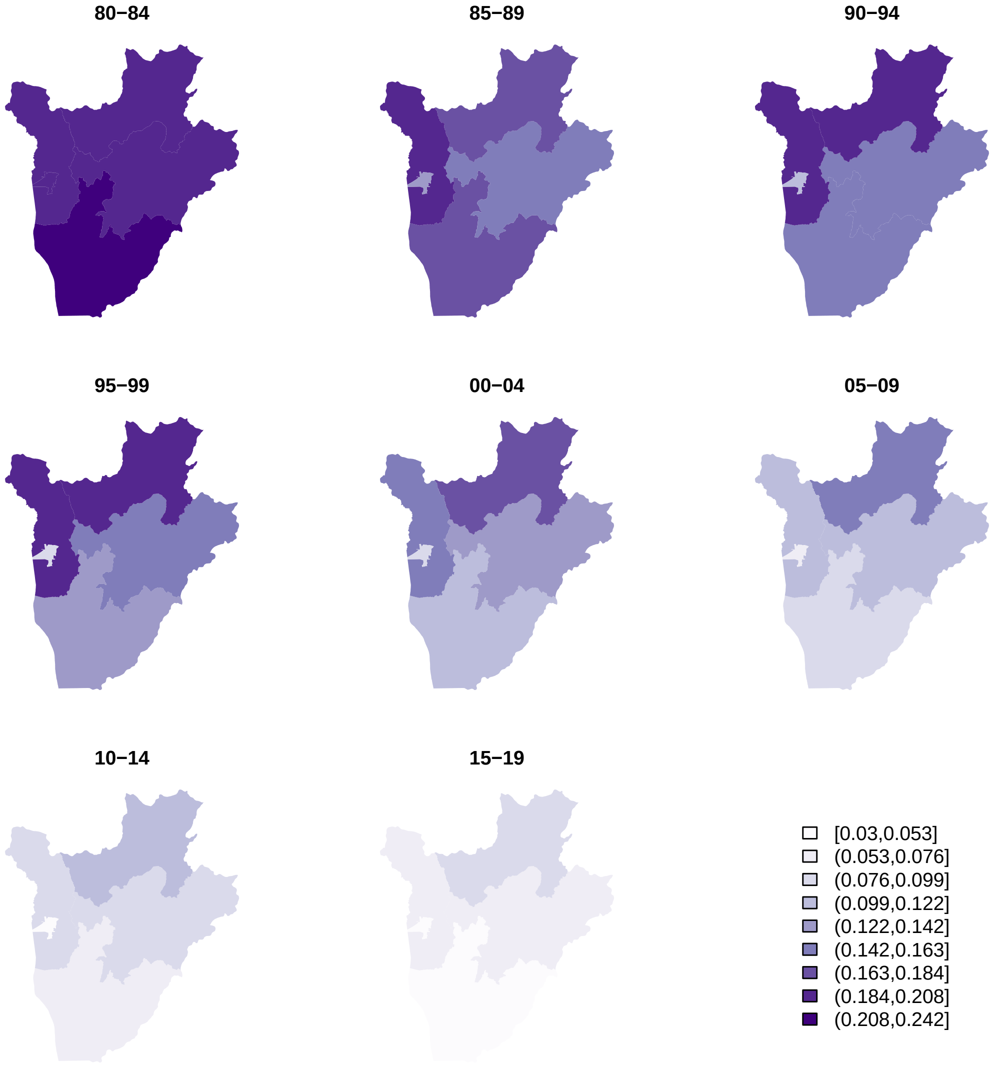

Figure S1.35: Burundi: Maps of posterior medians over time.

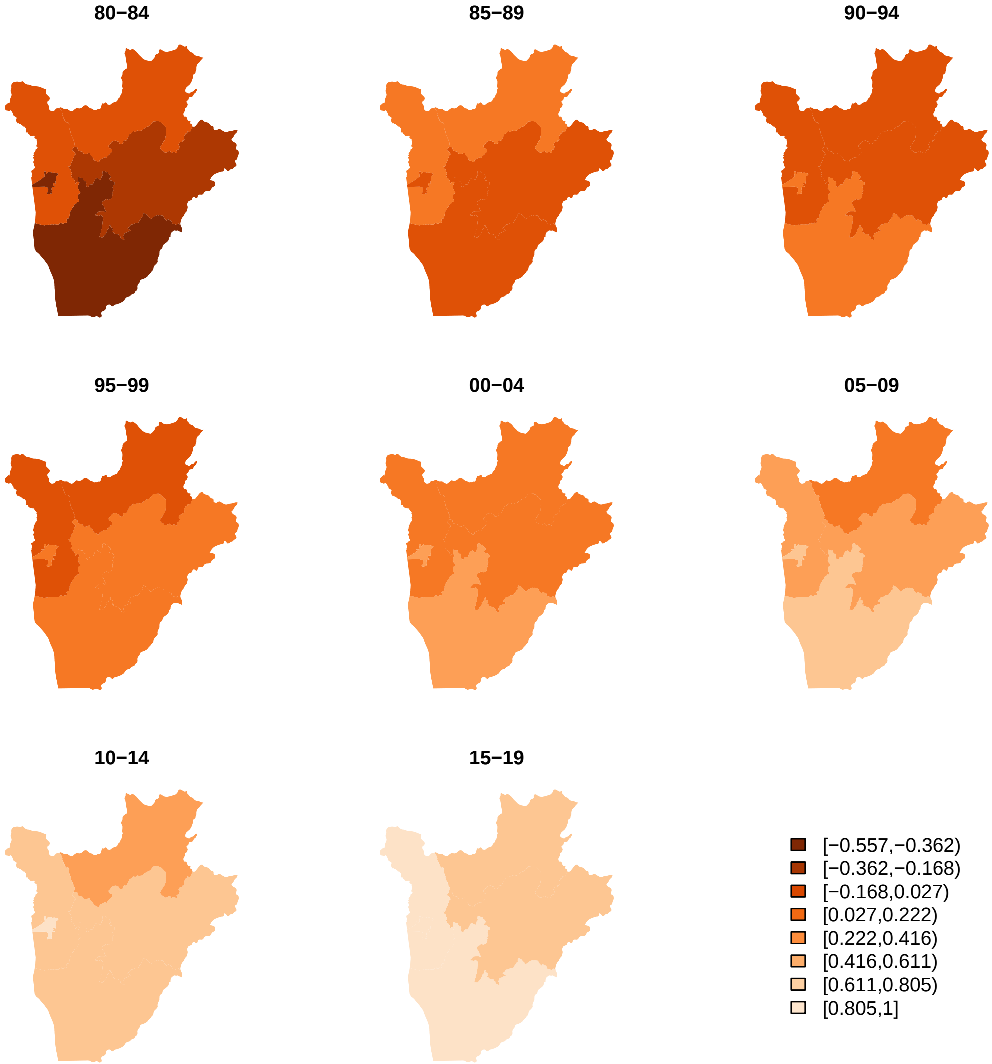

Figure S1.36: Burundi: Maps of reduction of posterior median U5MR in each five-year period compared to 1990 over time.

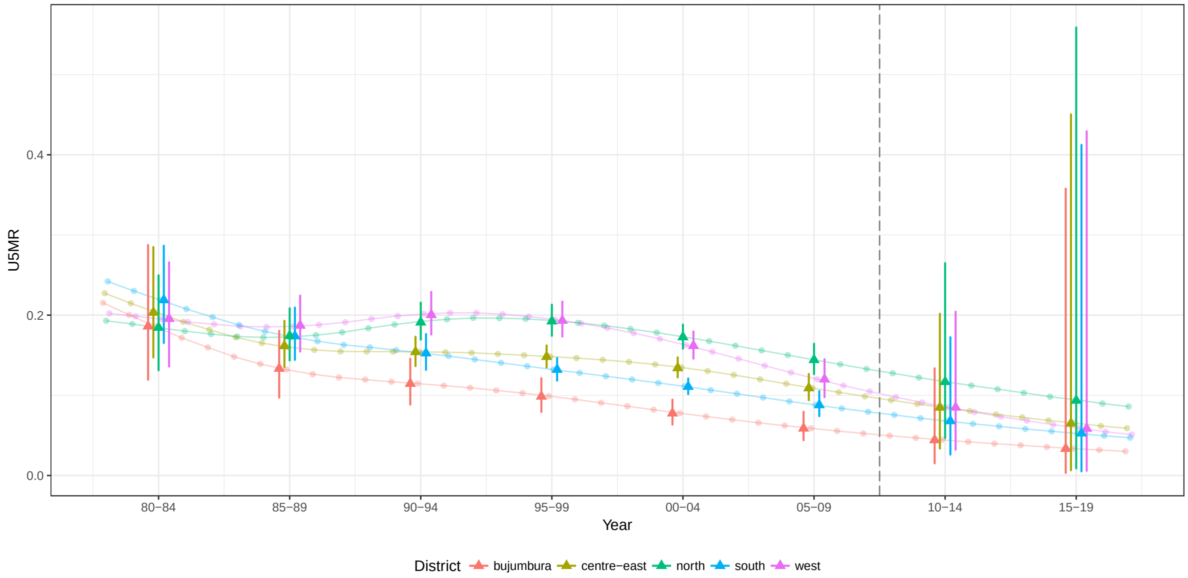

Figure S1.37: Burundi: Smoothed regional estimates over time. The line indicates yearly posterior median estimates and error bars indicate 95 % posterior credible interval at each time period.

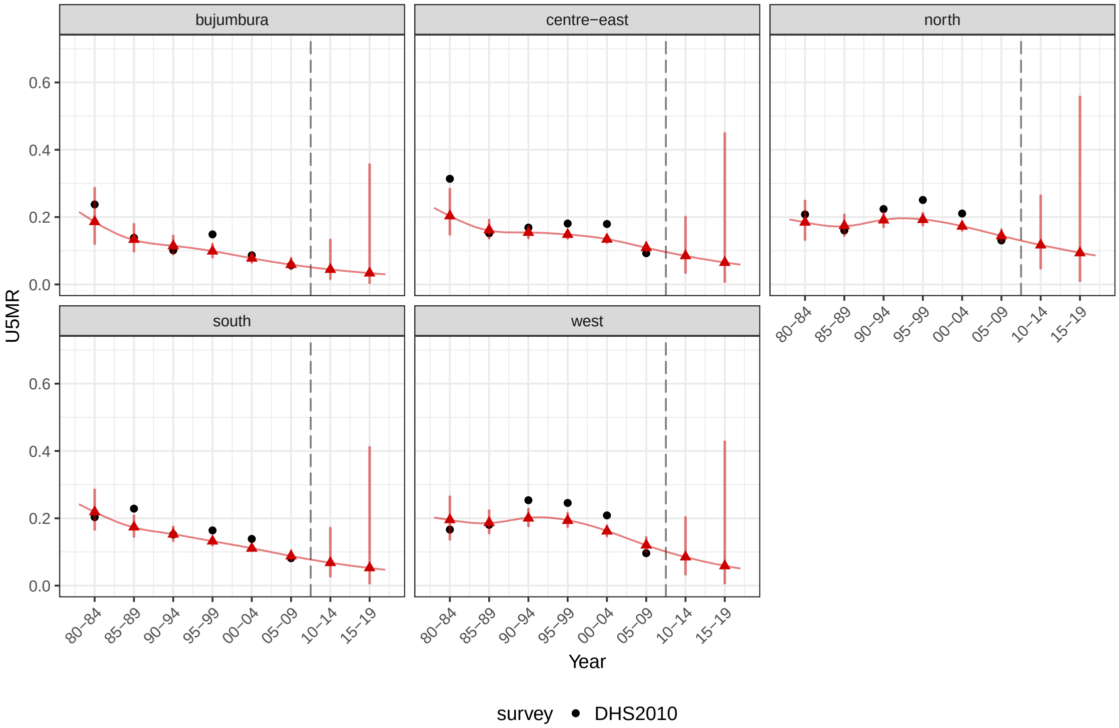

Figure S1.38: Burundi: Smoothed regional estimates over time compared to the direct estimates from each surveys. Direct estimates are not benchmarked with UN estimates. The line indicates posterior median and error bars indicate 95% posterior credible interval.

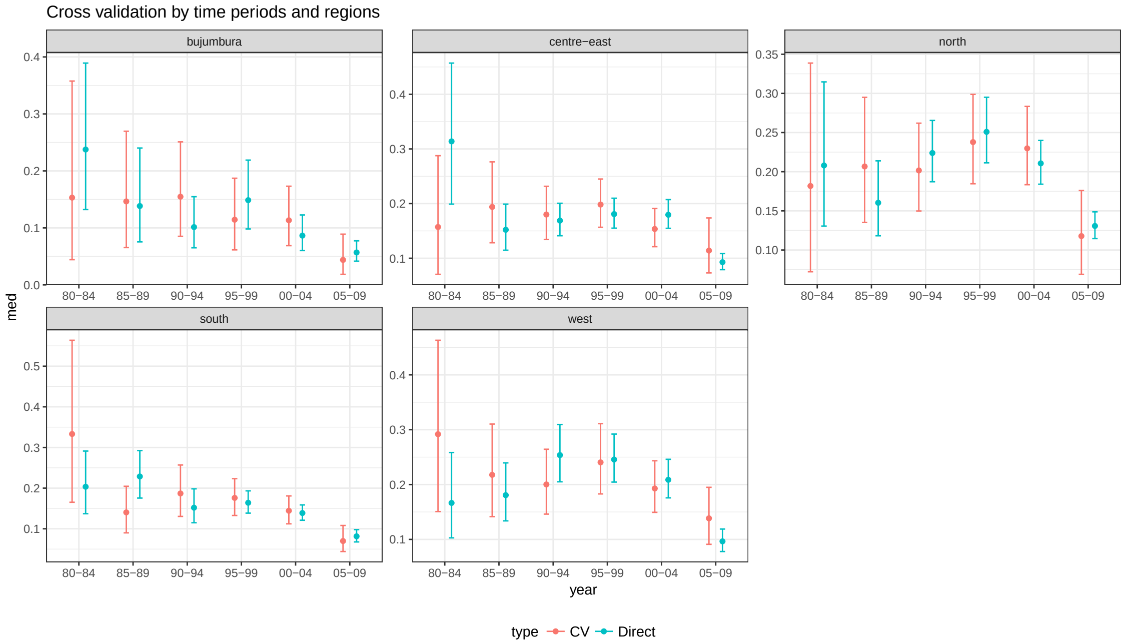

Figure S1.39: Burundi: Out-of-sample predictions along with direct estimates in the cross validation study where data from one region in each time period is held out and predicted using the rest of the data.

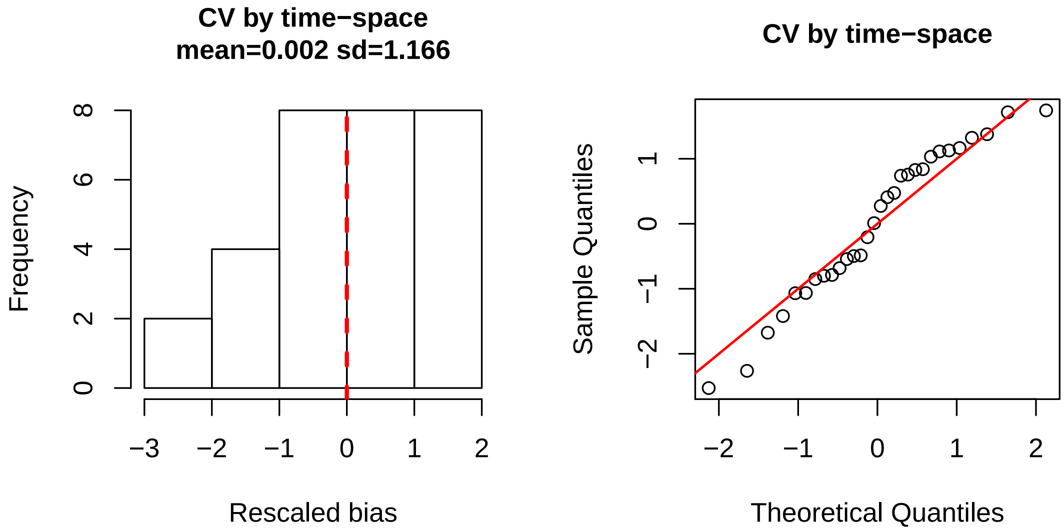

Figure S1.40: Burundi: Histogram and QQ-plot of the rescaled difference between the smoothed estimates and the direct estimates in the cross validation study. The differences between the two estimates are rescaled by the square root of the total variance of the two estimates.

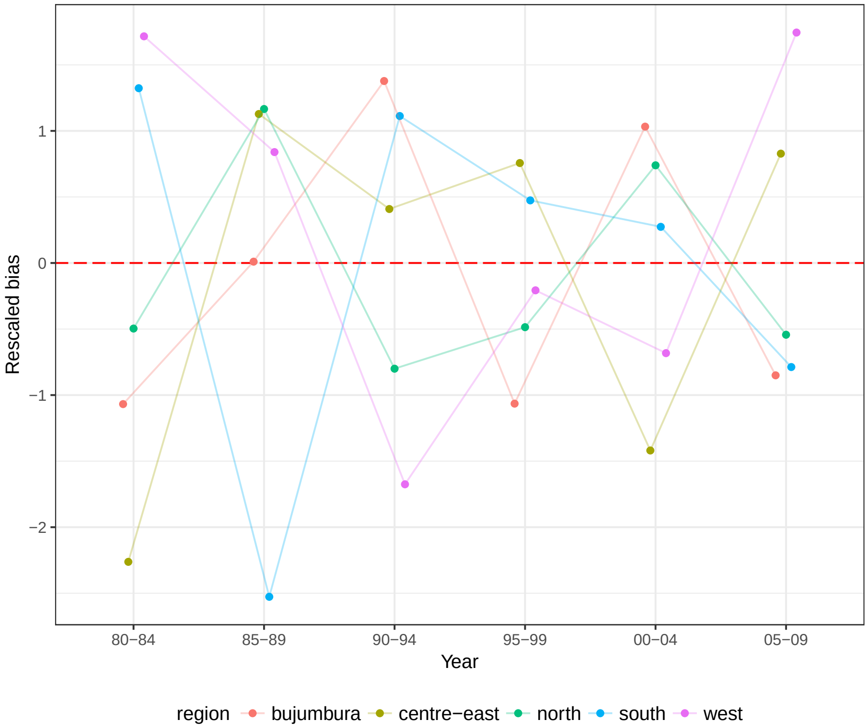

Figure S1.41: Burundi: Line plot of the difference between smoothed estimates and the direct estimates in the cross validation study. The differences between the two estimates are rescaled by the square root of the total variance of the two estimates.

3.5.5 Cameroon

DHS surveys were conducted in Cameroon in 1998, 2004, and 2011.

We fit both the RW2 only model to the combined national data, and compare the time trend at national level with the estimates produced by the UN and IHME in Figure S1.42. We then adjusted the combined national data to the UN estimates of U5MR, and refit the models on the benchmarked data.

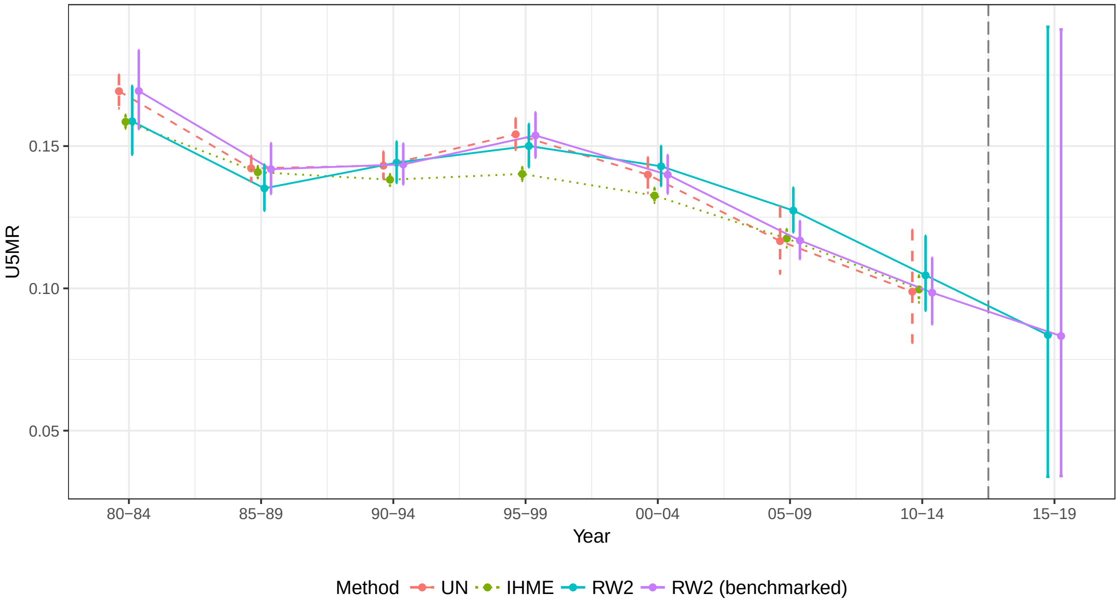

Figure S1.42: Cameroon: Temporal national trends along with UN (B3) estimates described in You et al. (2015) and IHME estimates based on GBD 2015 Child Mortality Collaborators (2016). RW2 represents the smoothed national estimates using the original data before benchmarking with UN estimates. RW2-adj represents the smoothed national estimates using the benchmarked data.

We fit the RW2 model to the benchmarked data in each area. We compare the results in Figure S1.43 to S1.47. Figure S1.43 compares the smoothed estimates against the direct estimates. Figure S1.44 and Figure S1.45 show the posterior median estimates of U5MR in each region over time and the reductions from 1990 period respectively. Figure S1.46 shows the smoothed estimates by region over time and Figure S1.47 compares the smoothed estimates with direct estimates from each survey for each region over time.

We further assess the RW2 model by holding out some observations, and compare the projections to the direct estimates in these holdout observations. Figure S1.48 compares the predicted estimates for the out-of-sample observations with the direct estimates by holding out observations from each area in each time period. Figure S1.49 compares the histogram of the bias rescaled by the total variance in the cross validation studies. Figure S1.50 compares the rescaled bias by region and time periods.

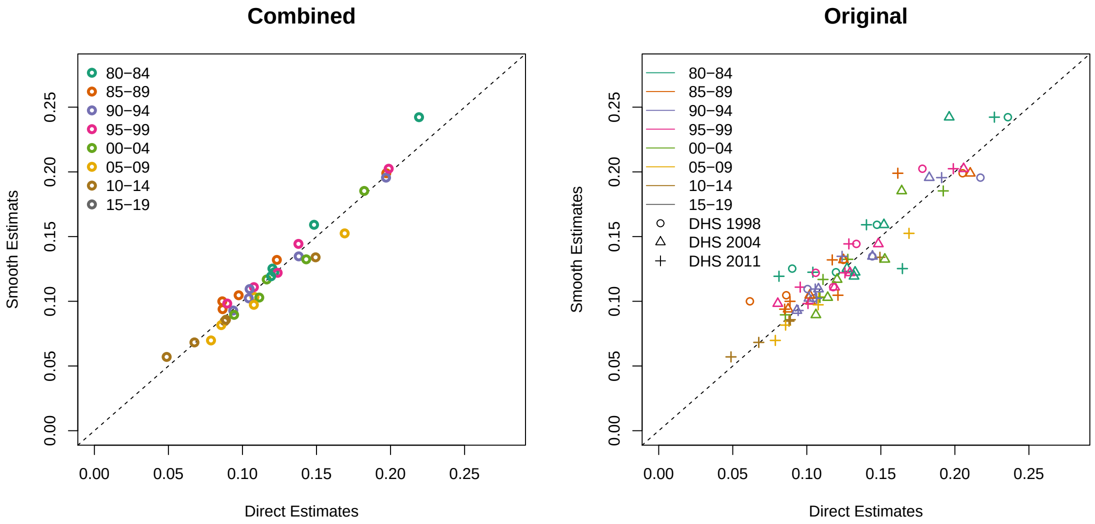

Figure S1.43: Cameroon: Smooth versus direct Admin 1 estimates. Left: Combined (meta-analysis) survey estimate against combined direct estimates. Right: Combined (meta-analysis) survey estimate against direct estimates from each survey.

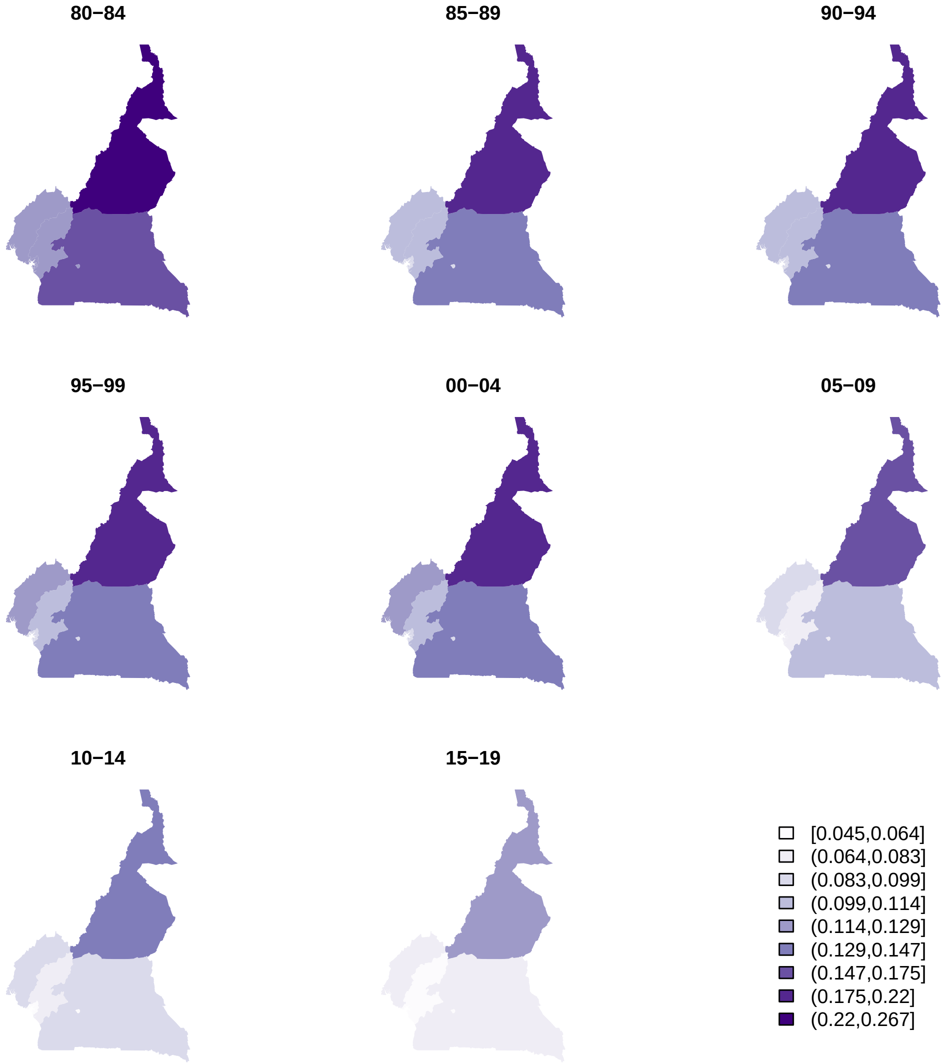

Figure S1.44: Cameroon: Maps of posterior medians over time.

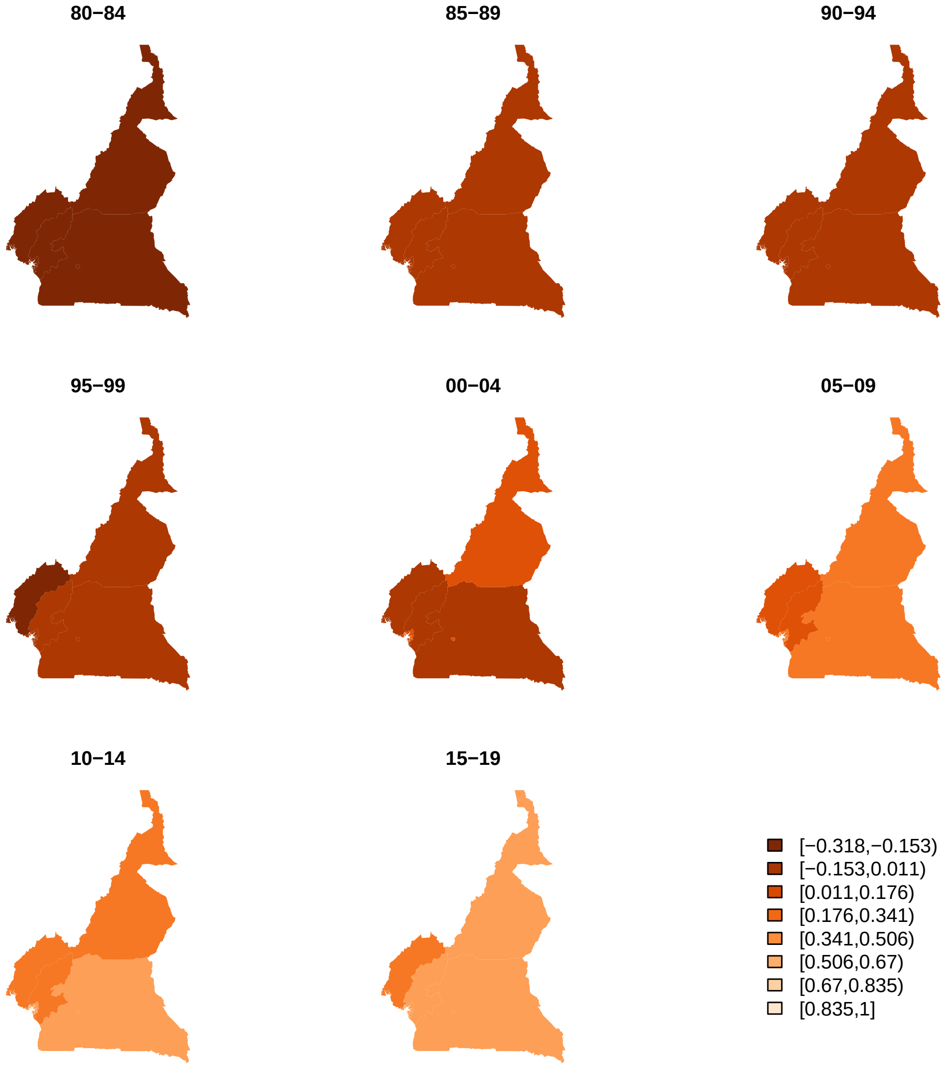

Figure S1.45: Cameroon: Maps of reduction of posterior median U5MR in each five-year period compared to 1990 over time.

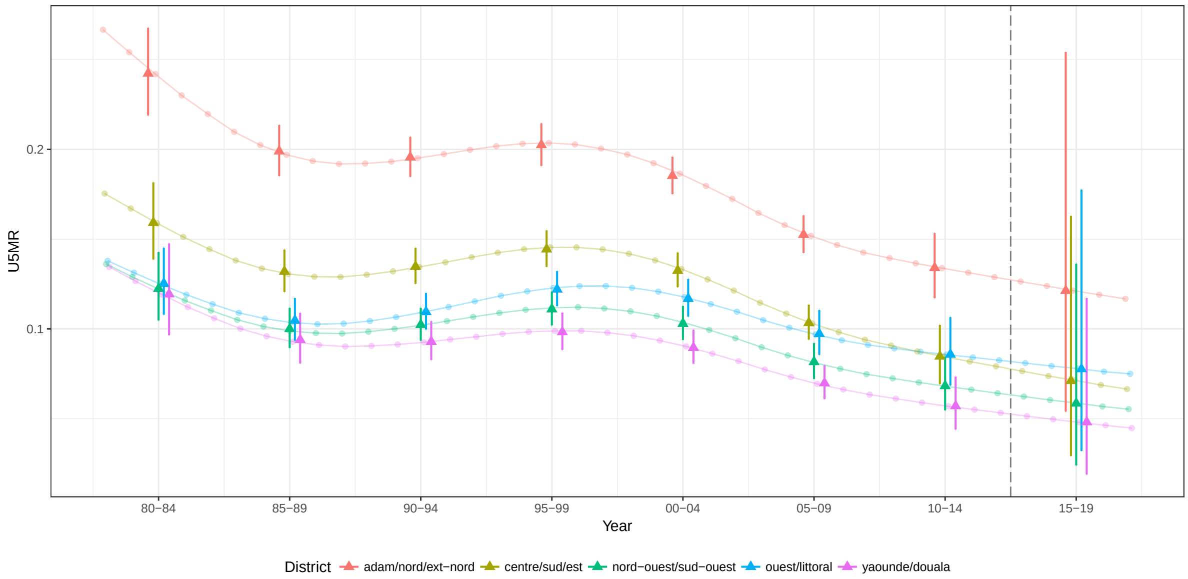

Figure S1.46: Cameroon: Smoothed regional estimates over time. The line indicates yearly posterior median estimates and error bars indicate 95 % posterior credible interval at each time period.

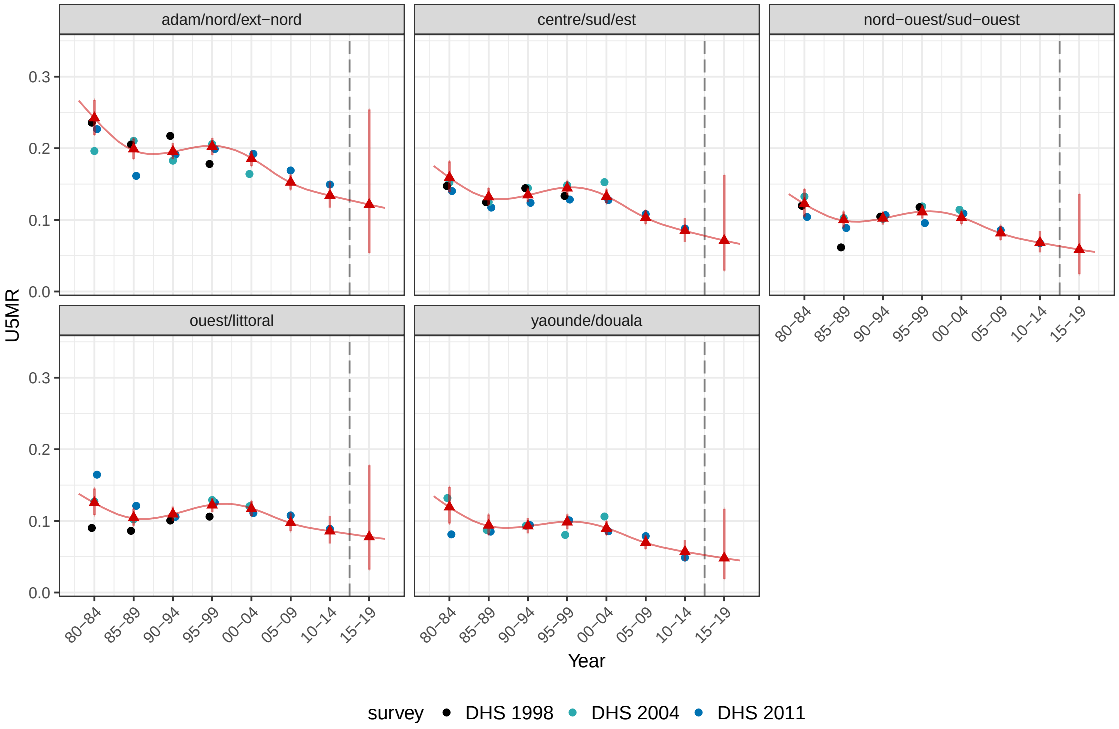

Figure S1.47: Cameroon: Smoothed regional estimates over time compared to the direct estimates from each surveys. Direct estimates are not benchmarked with UN estimates. The line indicates posterior median and error bars indicate 95% posterior credible interval.

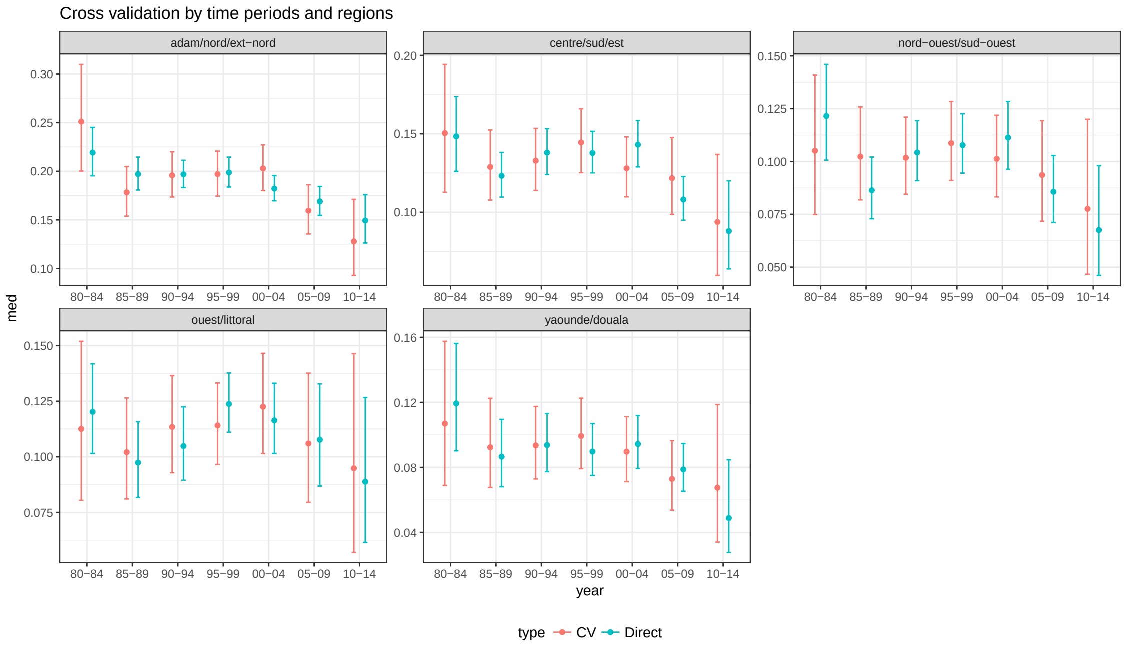

Figure S1.48: Cameroon: Out-of-sample predictions along with direct estimates in the cross validation study where data from one region in each time period is held out and predicted using the rest of the data.

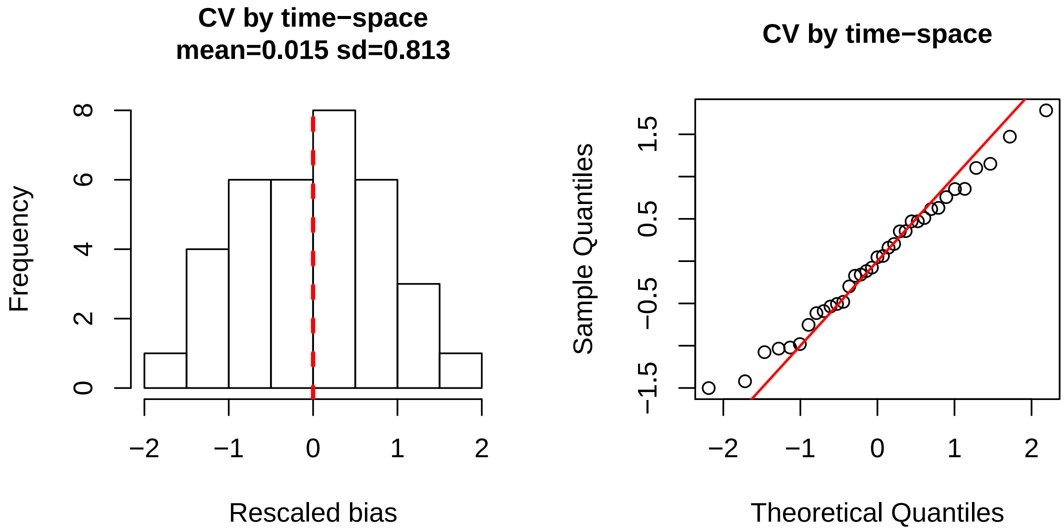

Figure S1.49: Cameroon: Histogram and QQ-plot of the rescaled difference between the smoothed estimates and the direct estimates in the cross validation study. The differences between the two estimates are rescaled by the square root of the total variance of the two estimates.

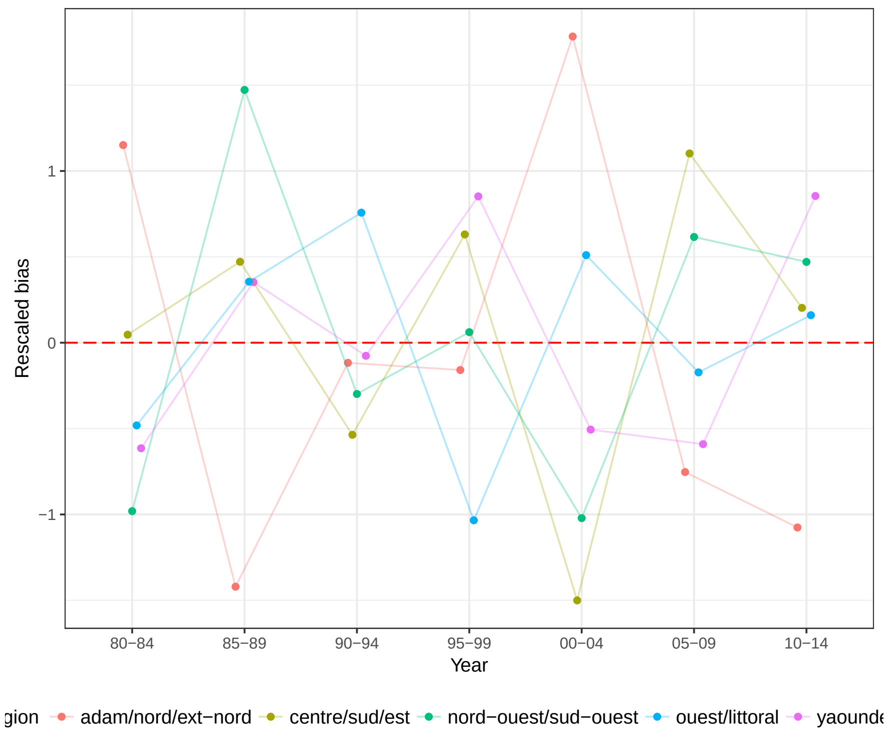

Figure S1.50: Cameroon: Line plot of the difference between smoothed estimates and the direct estimates in the cross validation study. The differences between the two estimates are rescaled by the square root of the total variance of the two estimates.

3.5.6 Chad

DHS surveys were conducted in Chad in 2004, and 2015.

We fit both the RW2 only model to the combined national data, and compare the time trend at national level with the estimates produced by the UN and IHME in Figure S1.51. We then adjusted the combined national data to the UN estimates of U5MR, and refit the models on the benchmarked data.

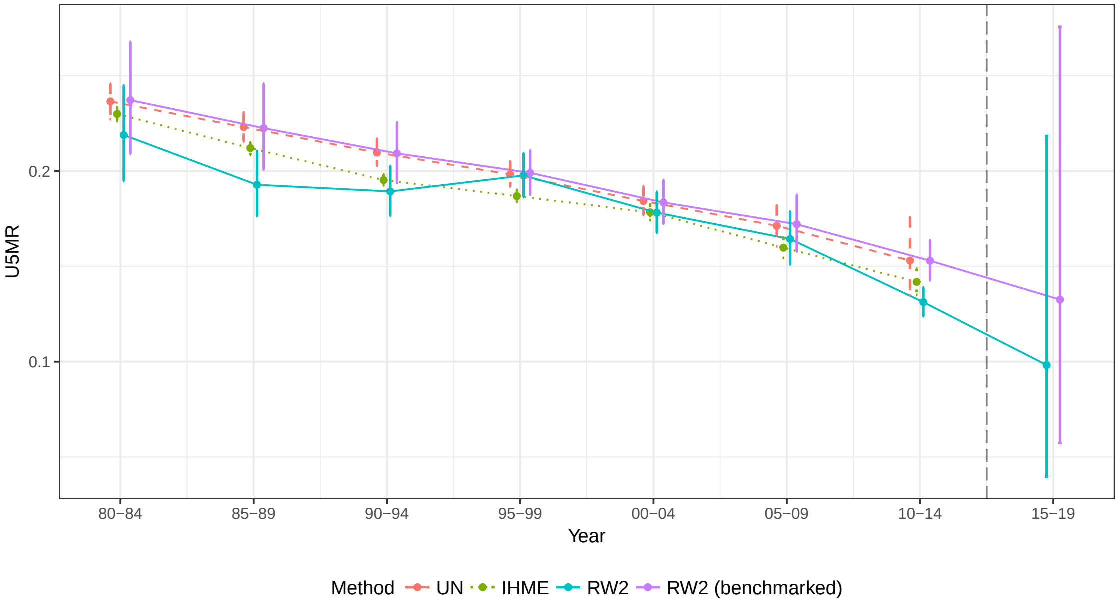

Figure S1.51: Chad: Temporal national trends along with UN (B3) estimates described in You et al. (2015) and IHME estimates based on GBD 2015 Child Mortality Collaborators (2016). RW2 represents the smoothed national estimates using the original data before benchmarking with UN estimates. RW2-adj represents the smoothed national estimates using the benchmarked data.

We fit the RW2 model to the benchmarked data in each area. We compare the results in Figure S1.52 to S1.56. Figure S1.52 compares the smoothed estimates against the direct estimates. Figure S1.53 and Figure S1.54 show the posterior median estimates of U5MR in each region over time and the reductions from 1990 period respectively. Figure S1.55 shows the smoothed estimates by region over time and Figure S1.56 compares the smoothed estimates with direct estimates from each survey for each region over time.

We further assess the RW2 model by holding out some observations, and compare the projections to the direct estimates in these holdout observations. Figure S1.57 compares the predicted estimates for the out-of-sample observations with the direct estimates by holding out observations from each area in each time period. Figure S1.58 compares the histogram of the bias rescaled by the total variance in the cross validation studies. Figure S1.59 compares the rescaled bias by region and time periods.

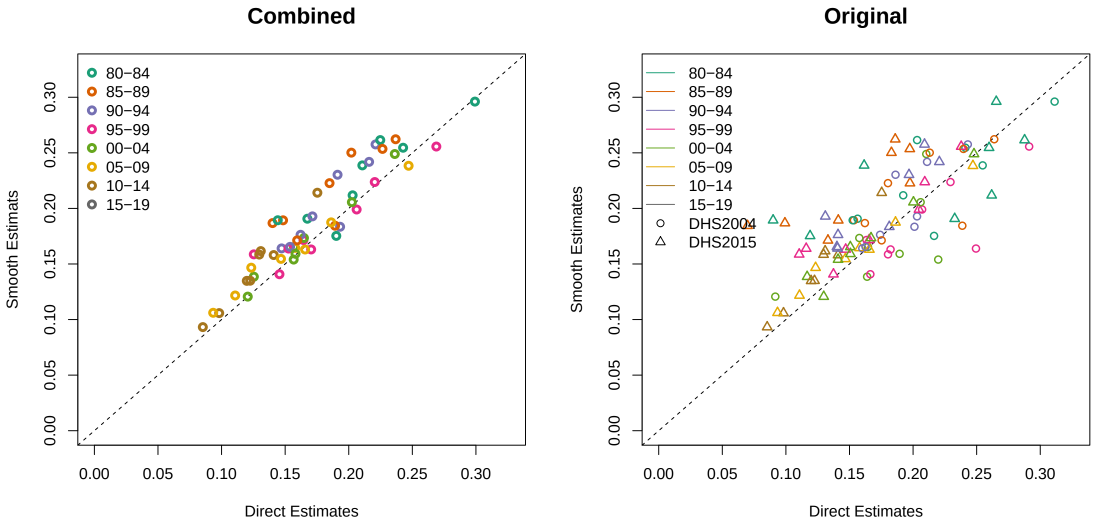

Figure S1.52: Chad: Smooth versus direct Admin 1 estimates. Left: Combined (meta-analysis) survey estimate against combined direct estimates. Right: Combined (meta-analysis) survey estimate against direct estimates from each survey.

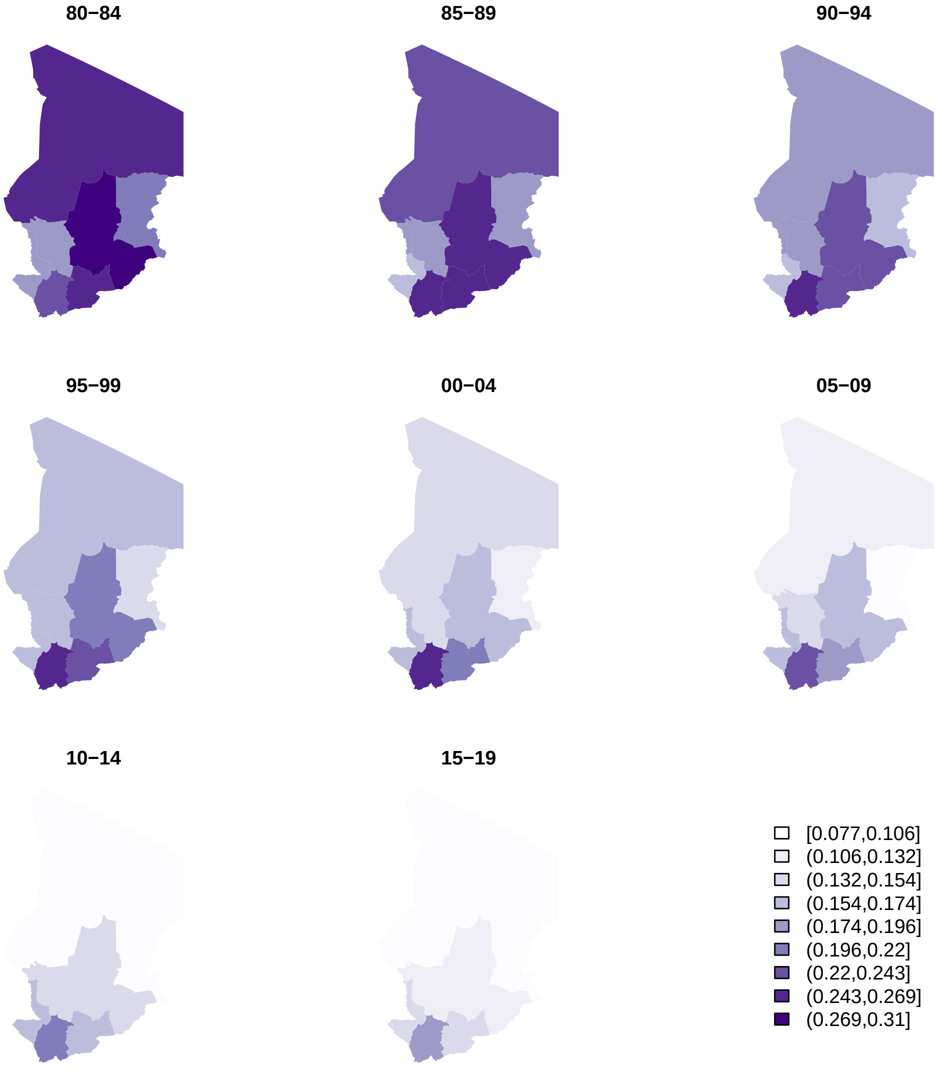

Figure S1.53: Chad: Maps of posterior medians over time.

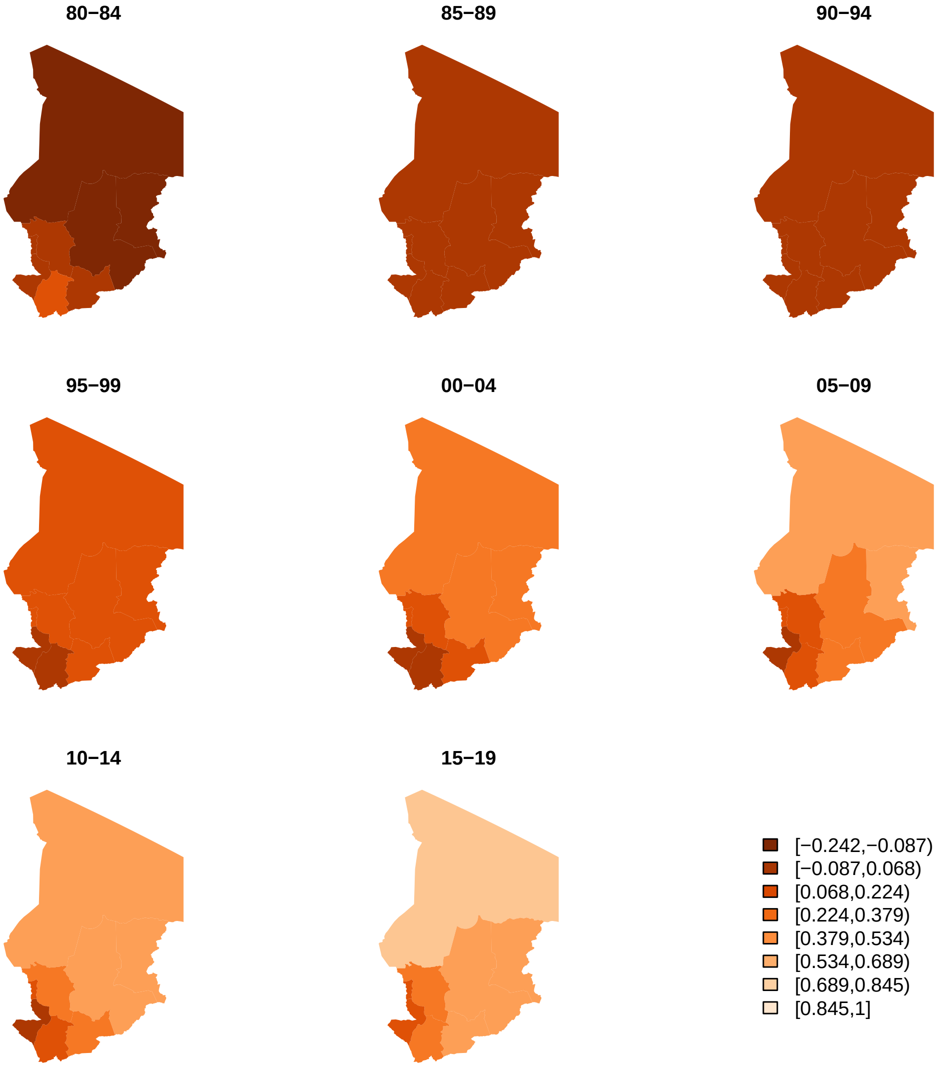

Figure S1.54: Chad: Maps of reduction of posterior median U5MR in each five-year period compared to 1990 over time.

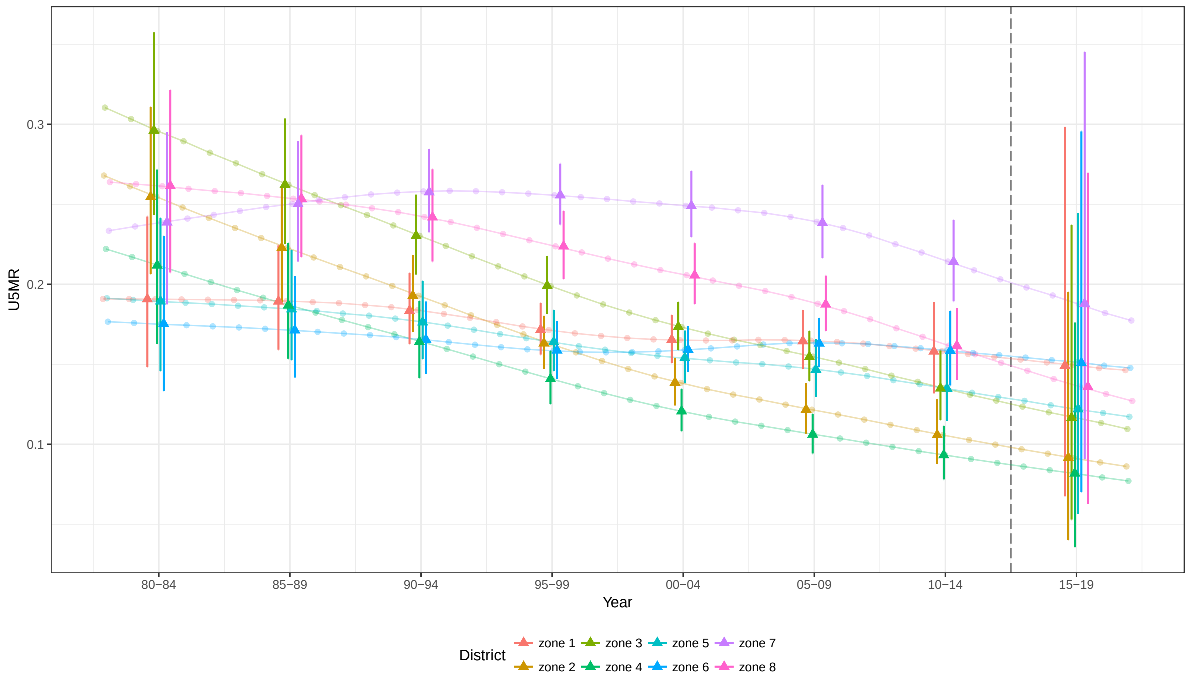

Figure S1.55: Chad: Smoothed regional estimates over time. The line indicates yearly posterior median estimates and error bars indicate 95 % posterior credible interval at each time period.

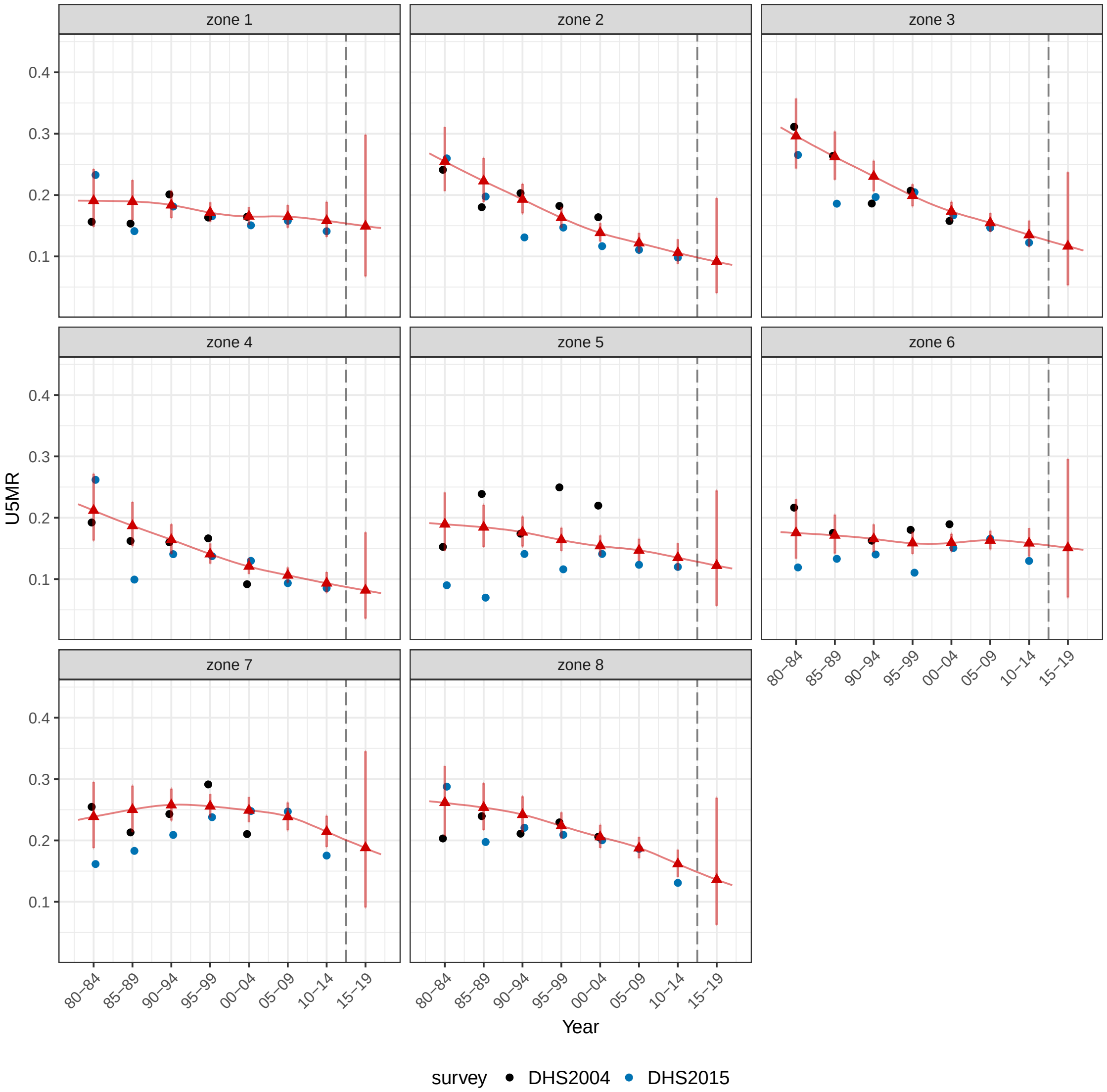

Figure S1.56: Chad: Smoothed regional estimates over time compared to the direct estimates from each surveys. Direct estimates are not benchmarked with UN estimates. The line indicates posterior median and error bars indicate 95% posterior credible interval.

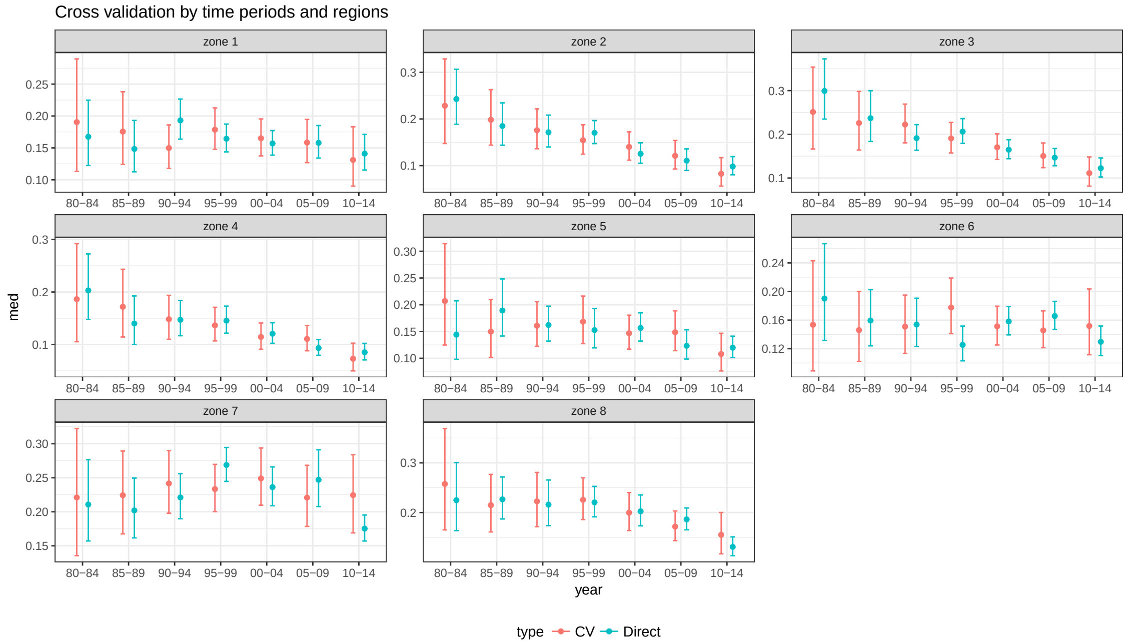

Figure S1.57: Chad: Out-of-sample predictions along with direct estimates in the cross validation study where data from one region in each time period is held out and predicted using the rest of the data.

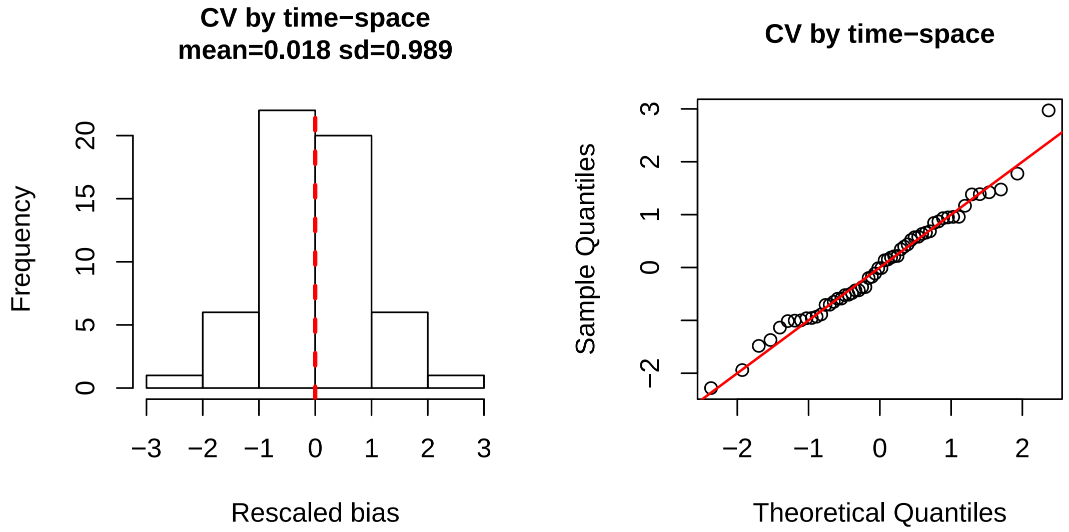

Figure S1.58: Chad: Histogram and QQ-plot of the rescaled difference between the smoothed estimates and the direct estimates in the cross validation study. The differences between the two estimates are rescaled by the square root of the total variance of the two estimates.

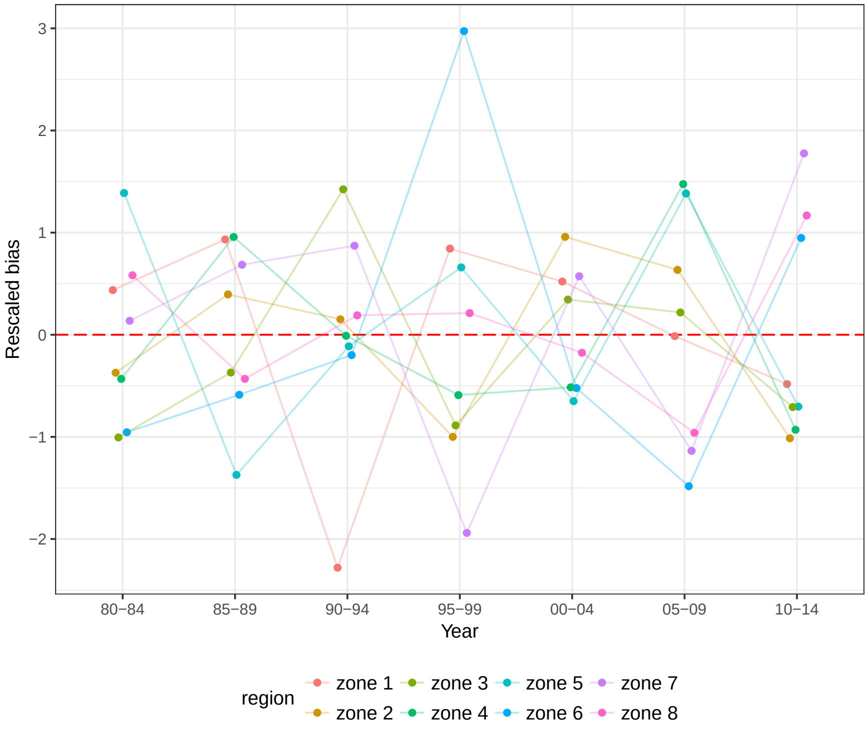

Figure S1.59: Chad: Line plot of the difference between smoothed estimates and the direct estimates in the cross validation study. The differences between the two estimates are rescaled by the square root of the total variance of the two estimates.

3.5.7 Comoros

DHS surveys were conducted in Comoros in 1996, and 2012.

We fit both the RW2 only model to the combined national data, and compare the time trend at national level with the estimates produced by the UN and IHME in Figure S1.60. We then adjusted the combined national data to the UN estimates of U5MR, and refit the models on the benchmarked data.

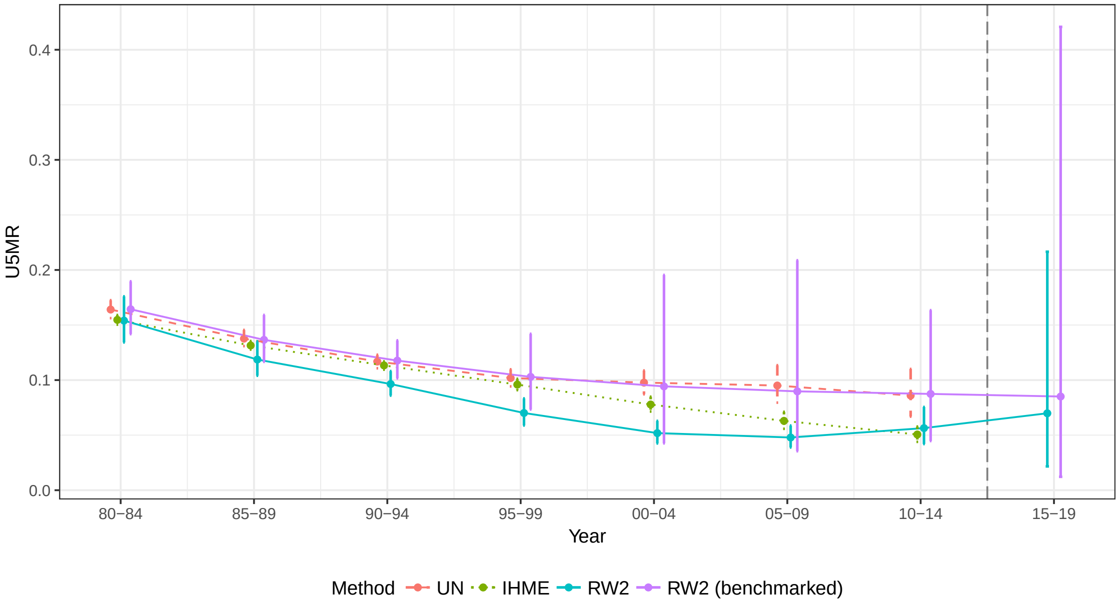

Figure S1.60: Comoros: Temporal national trends along with UN (B3) estimates described in You et al. (2015) and IHME estimates based on GBD 2015 Child Mortality Collaborators (2016). RW2 represents the smoothed national estimates using the original data before benchmarking with UN estimates. RW2-adj represents the smoothed national estimates using the benchmarked data.

We fit the RW2 model to the benchmarked data in each area. We compare the results in Figure S1.61 to S1.65. Figure S1.61 compares the smoothed estimates against the direct estimates. Figure S1.62 and Figure S1.63 show the posterior median estimates of U5MR in each region over time and the reductions from 1990 period respectively. Figure S1.64 shows the smoothed estimates by region over time and Figure S1.65 compares the smoothed estimates with direct estimates from each survey for each region over time.

We further assess the RW2 model by holding out some observations, and compare the projections to the direct estimates in these holdout observations. Figure S1.66 compares the predicted estimates for the out-of-sample observations with the direct estimates by holding out observations from each area in each time period. Figure S1.67 compares the histogram of the bias rescaled by the total variance in the cross validation studies. Figure S1.68 compares the rescaled bias by region and time periods.

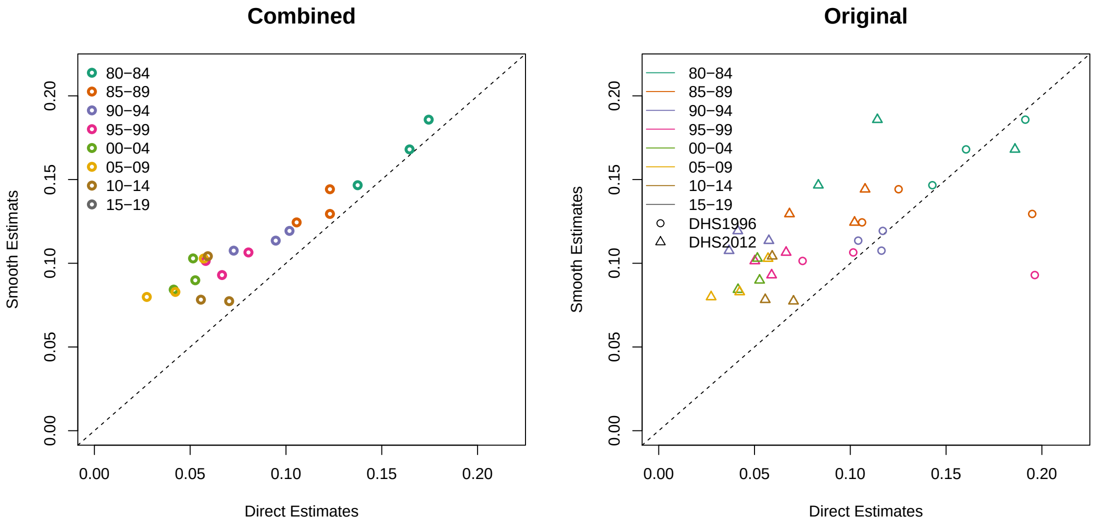

Figure S1.61: Comoros: Smooth versus direct Admin 1 estimates. Left: Combined (meta-analysis) survey estimate against combined direct estimates. Right: Combined (meta-analysis) survey estimate against direct estimates from each survey.

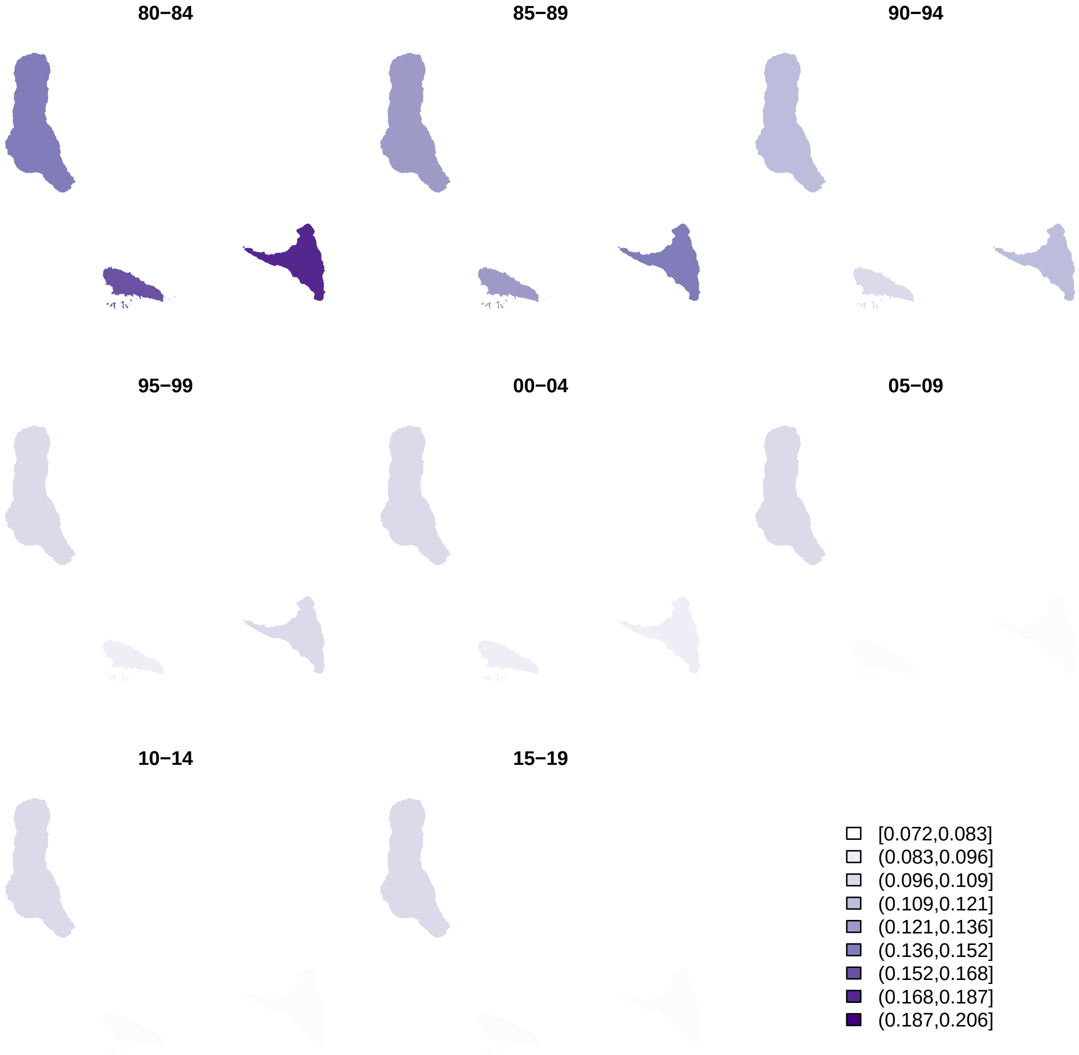

Figure S1.62: Comoros: Maps of posterior medians over time.

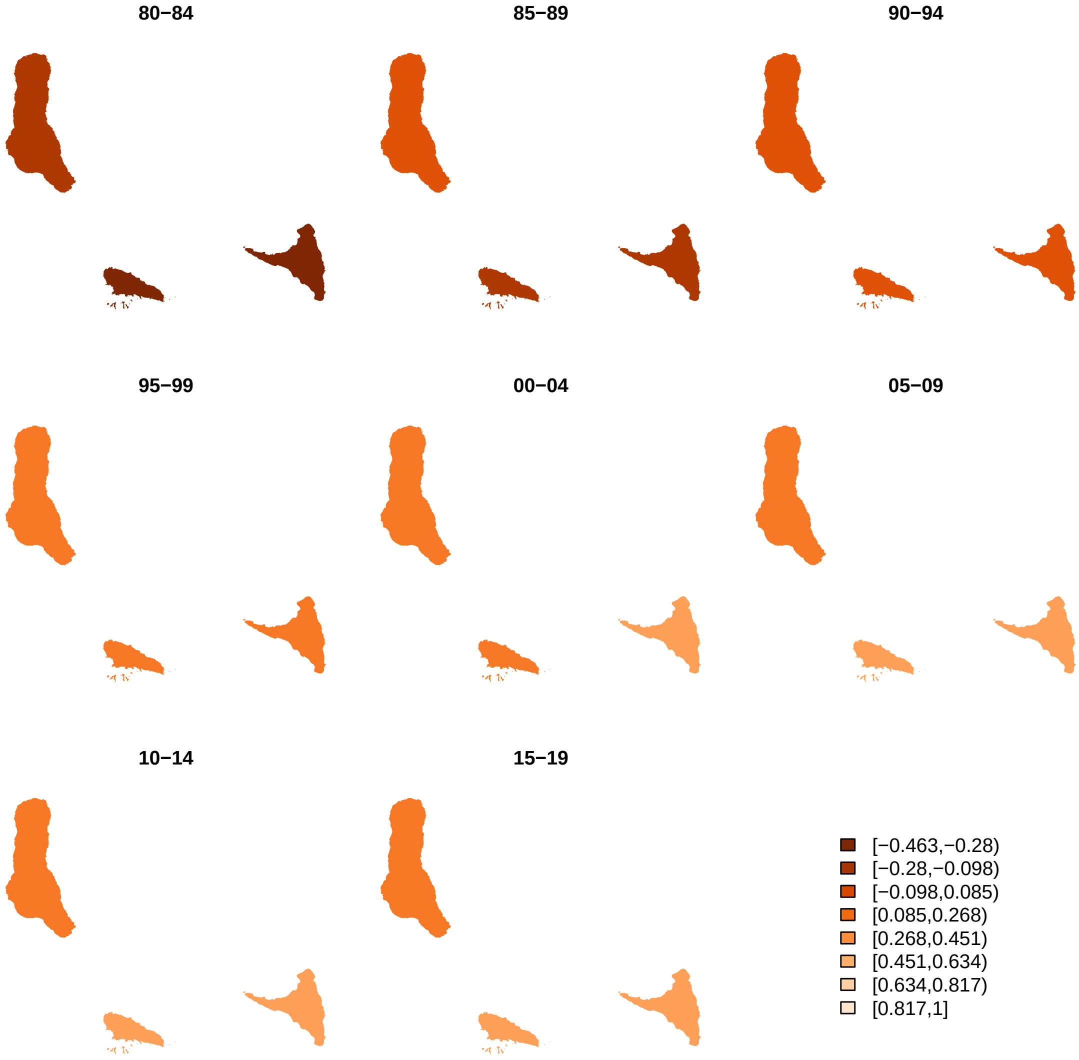

Figure S1.63: Comoros: Maps of reduction of posterior median U5MR in each five-year period compared to 1990 over time.

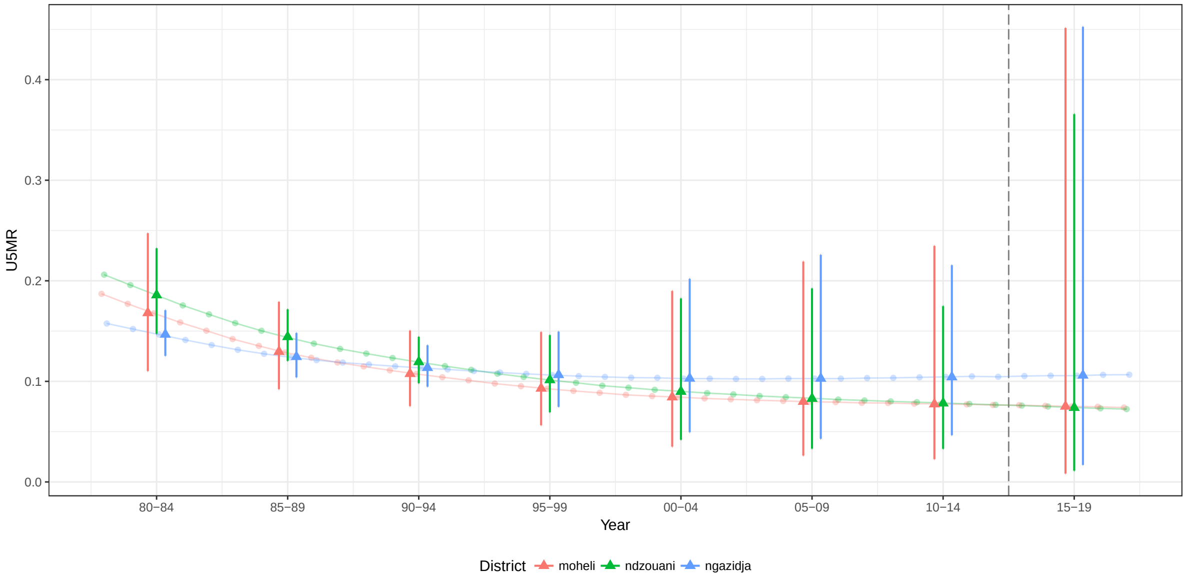

Figure S1.64: Comoros: Smoothed regional estimates over time. The line indicates yearly posterior median estimates and error bars indicate 95 % posterior credible interval at each time period.

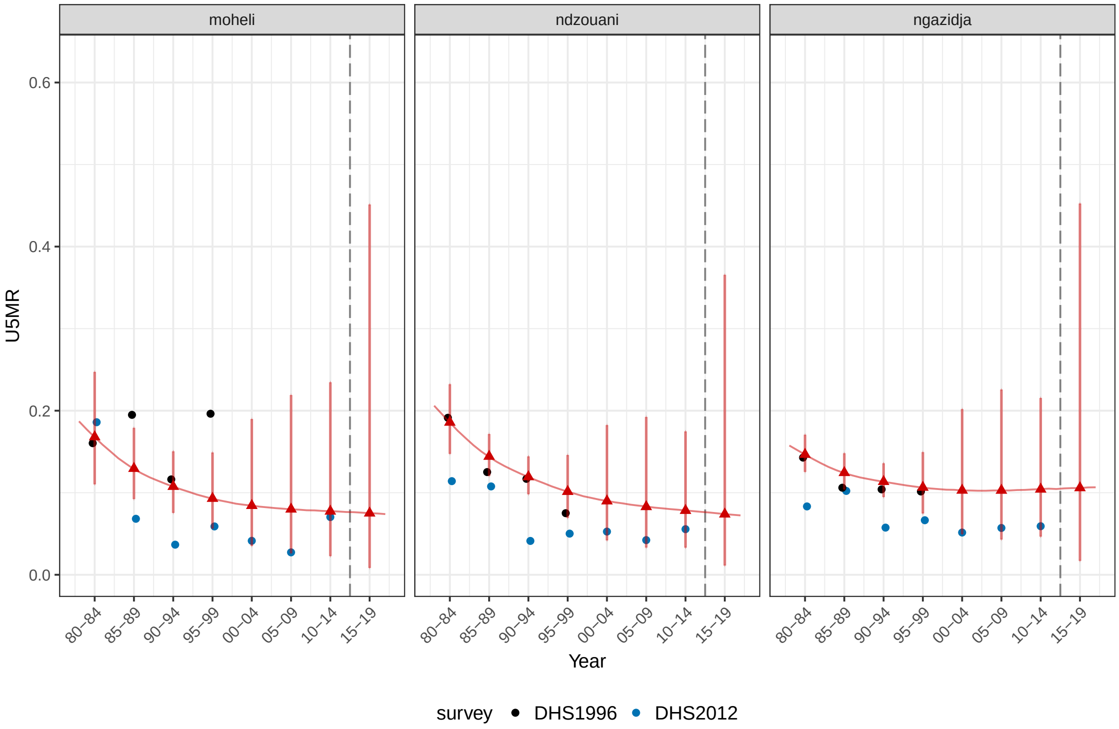

Figure S1.65: Comoros: Smoothed regional estimates over time compared to the direct estimates from each surveys. Direct estimates are not benchmarked with UN estimates. The line indicates posterior median and error bars indicate 95% posterior credible interval.

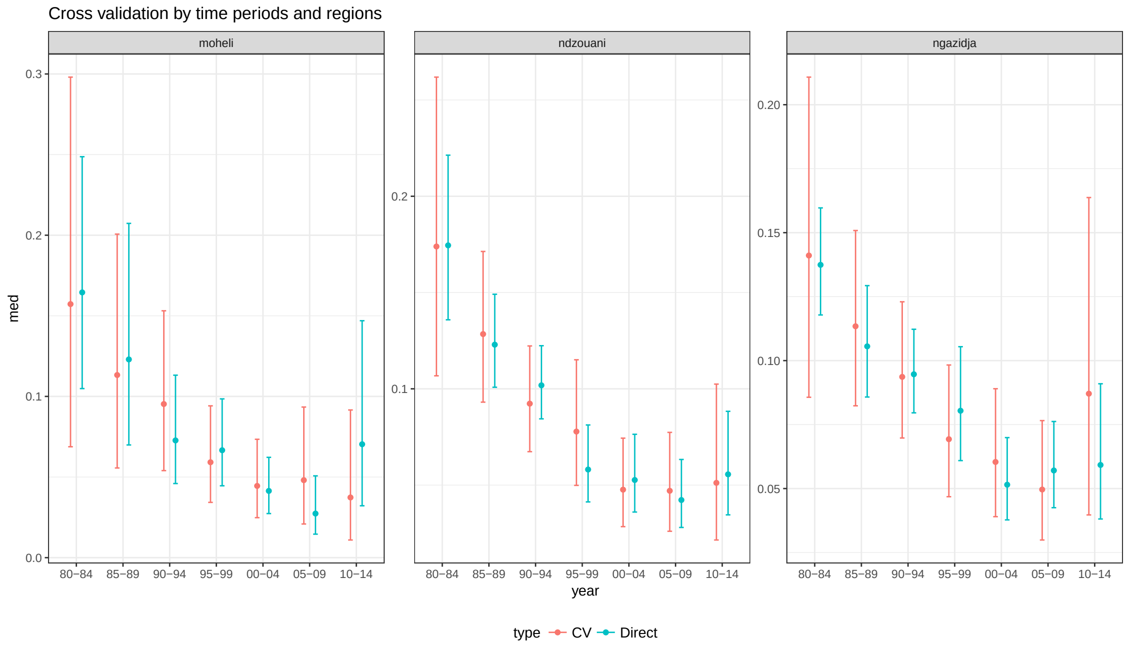

Figure S1.66: Comoros: Out-of-sample predictions along with direct estimates in the cross validation study where data from one region in each time period is held out and predicted using the rest of the data.

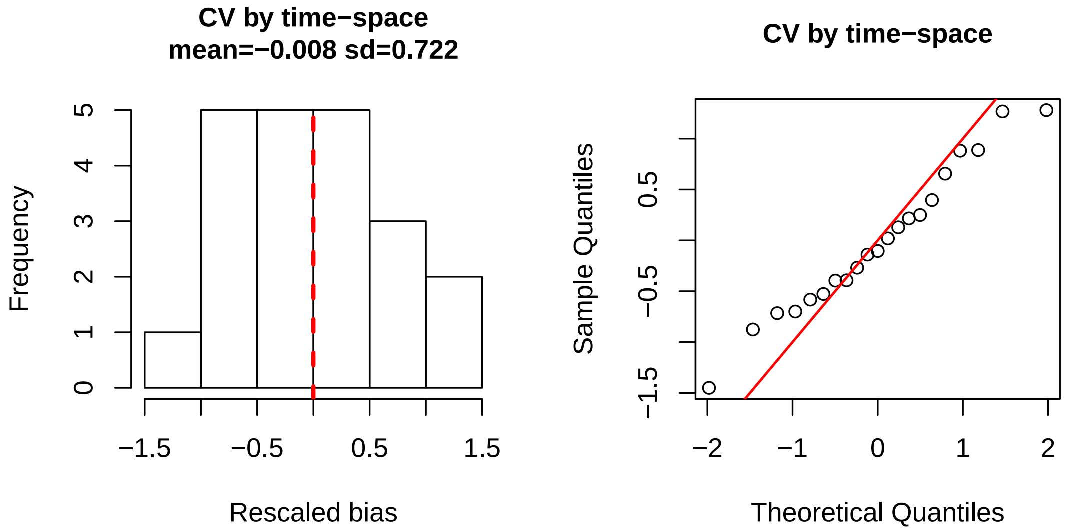

Figure S1.67: Comoros: Histogram and QQ-plot of the rescaled difference between the smoothed estimates and the direct estimates in the cross validation study. The differences between the two estimates are rescaled by the square root of the total variance of the two estimates.

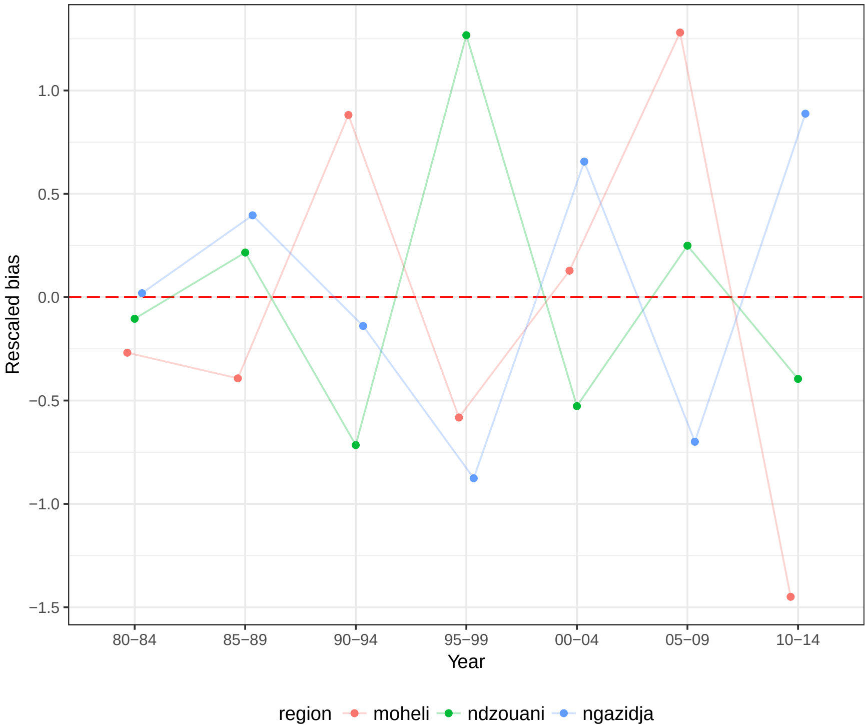

Figure S1.68: Comoros: Line plot of the difference between smoothed estimates and the direct estimates in the cross validation study. The differences between the two estimates are rescaled by the square root of the total variance of the two estimates.

3.5.8 Congo

DHS surveys were conducted in Congo in 2005, and 2012.

We fit both the RW2 only model to the combined national data, and compare the time trend at national level with the estimates produced by the UN and IHME in Figure S1.69. We then adjusted the combined national data to the UN estimates of U5MR, and refit the models on the benchmarked data.

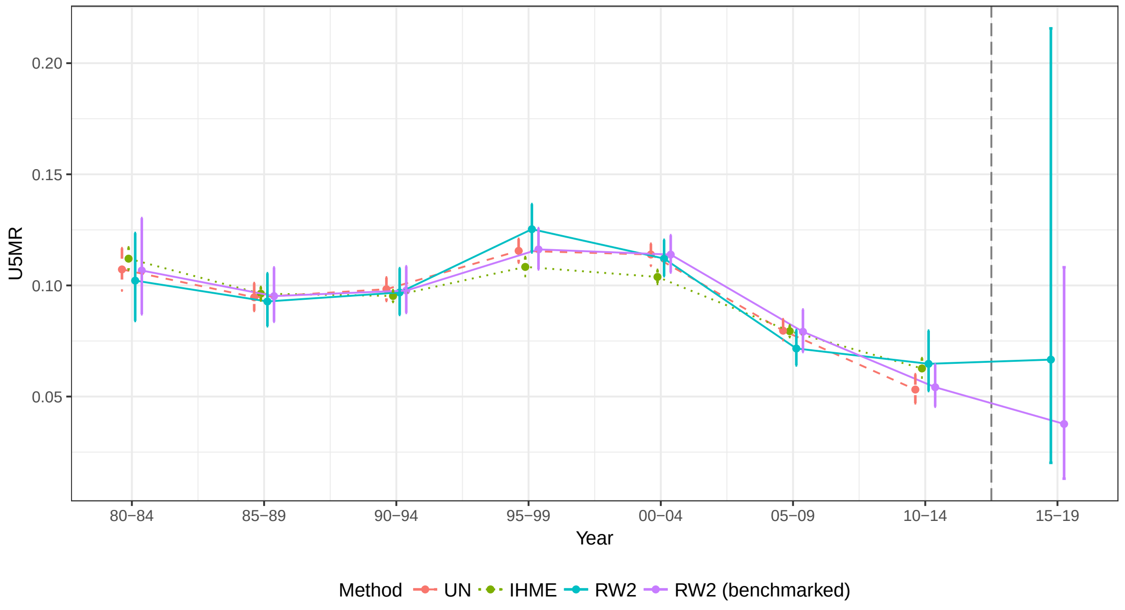

Figure S1.69: Congo: Temporal national trends along with UN (B3) estimates described in You et al. (2015) and IHME estimates based on GBD 2015 Child Mortality Collaborators (2016). RW2 represents the smoothed national estimates using the original data before benchmarking with UN estimates. RW2-adj represents the smoothed national estimates using the benchmarked data.

We fit the RW2 model to the benchmarked data in each area. We compare the results in Figure S1.70 to S1.74. Figure S1.70 compares the smoothed estimates against the direct estimates. Figure S1.71 and Figure S1.72 show the posterior median estimates of U5MR in each region over time and the reductions from 1990 period respectively. Figure S1.73 shows the smoothed estimates by region over time and Figure S1.74 compares the smoothed estimates with direct estimates from each survey for each region over time.

We further assess the RW2 model by holding out some observations, and compare the projections to the direct estimates in these holdout observations. Figure S1.75 compares the predicted estimates for the out-of-sample observations with the direct estimates by holding out observations from each area in each time period. Figure S1.76 compares the histogram of the bias rescaled by the total variance in the cross validation studies. Figure S1.77 compares the rescaled bias by region and time periods.

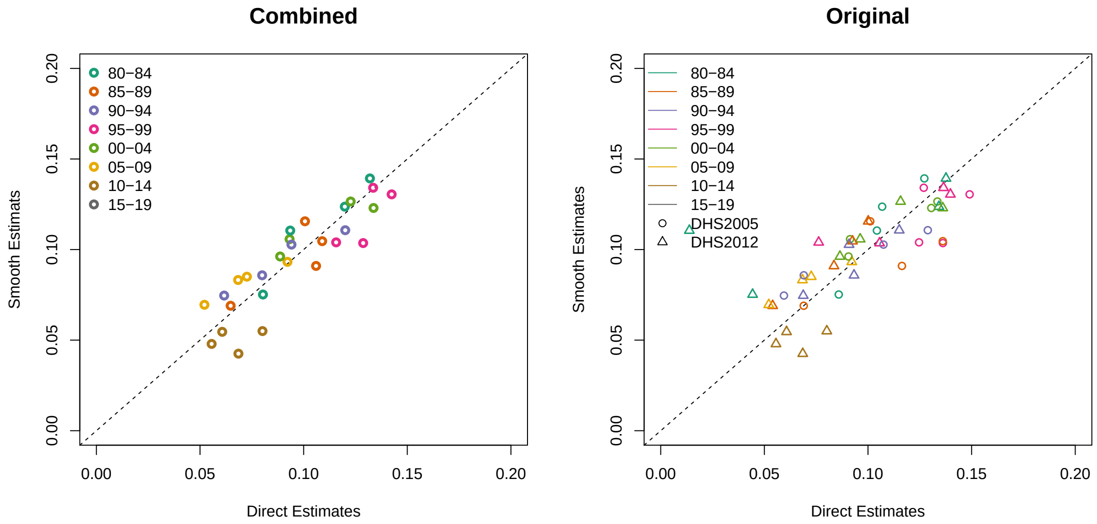

Figure S1.70: Congo: Smooth versus direct Admin 1 estimates. Left: Combined (meta-analysis) survey estimate against combined direct estimates. Right: Combined (meta-analysis) survey estimate against direct estimates from each survey.

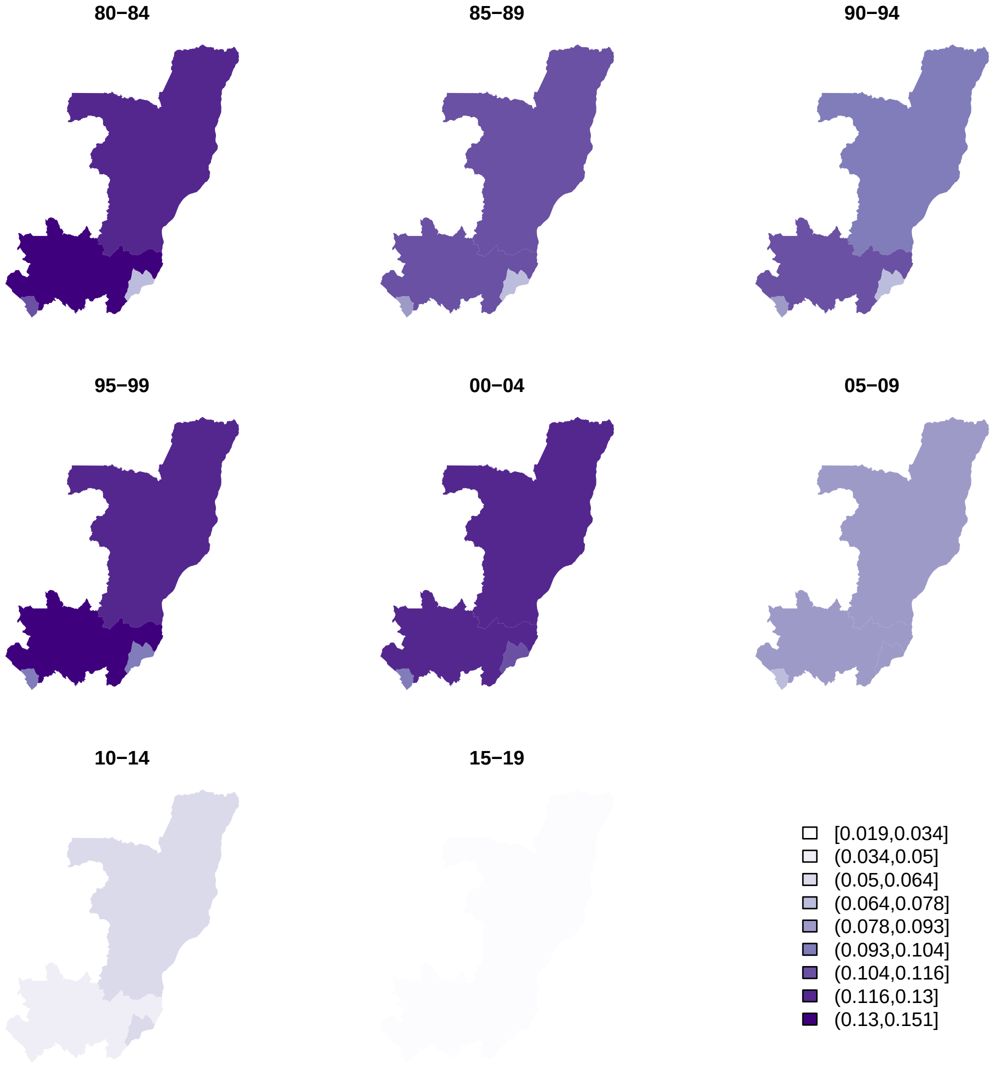

Figure S1.71: Congo: Maps of posterior medians over time.

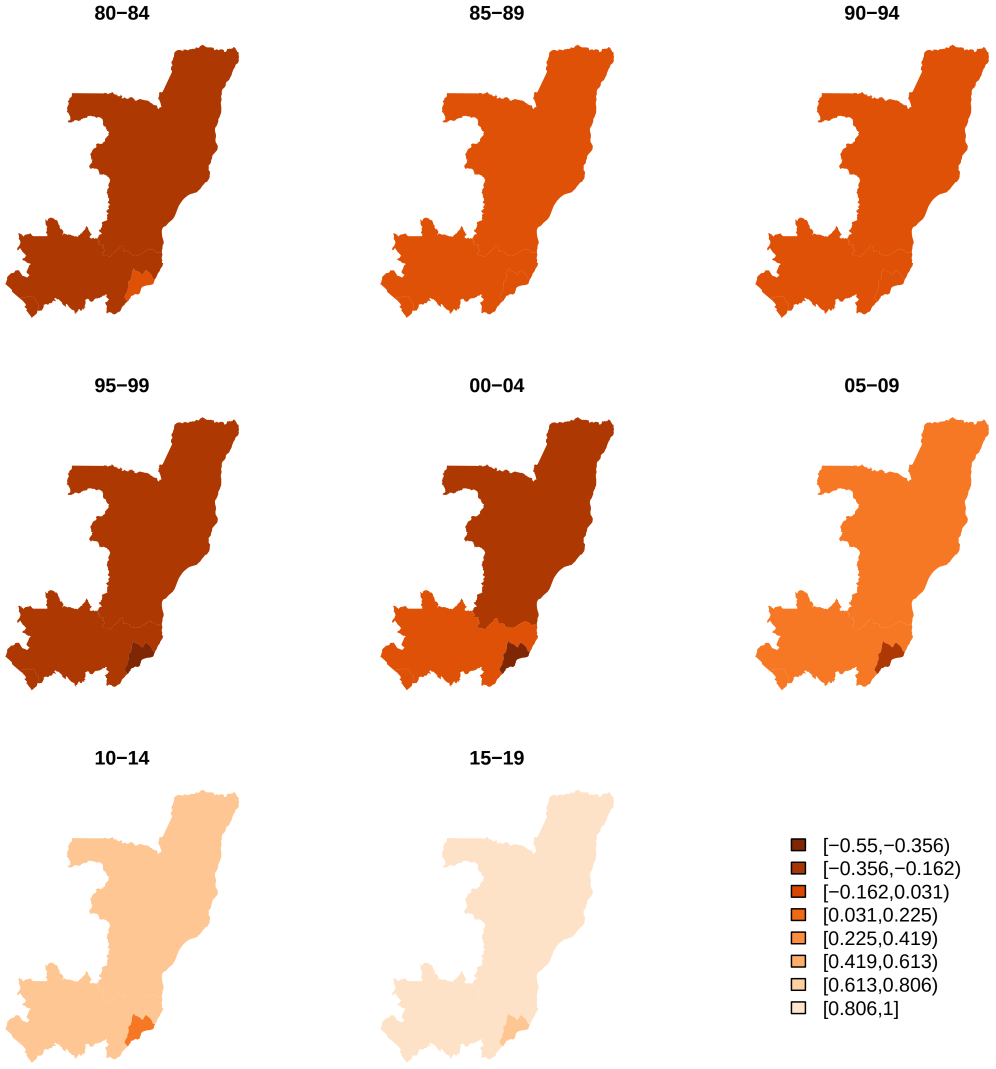

Figure S1.72: Congo: Maps of reduction of posterior median U5MR in each five-year period compared to 1990 over time.

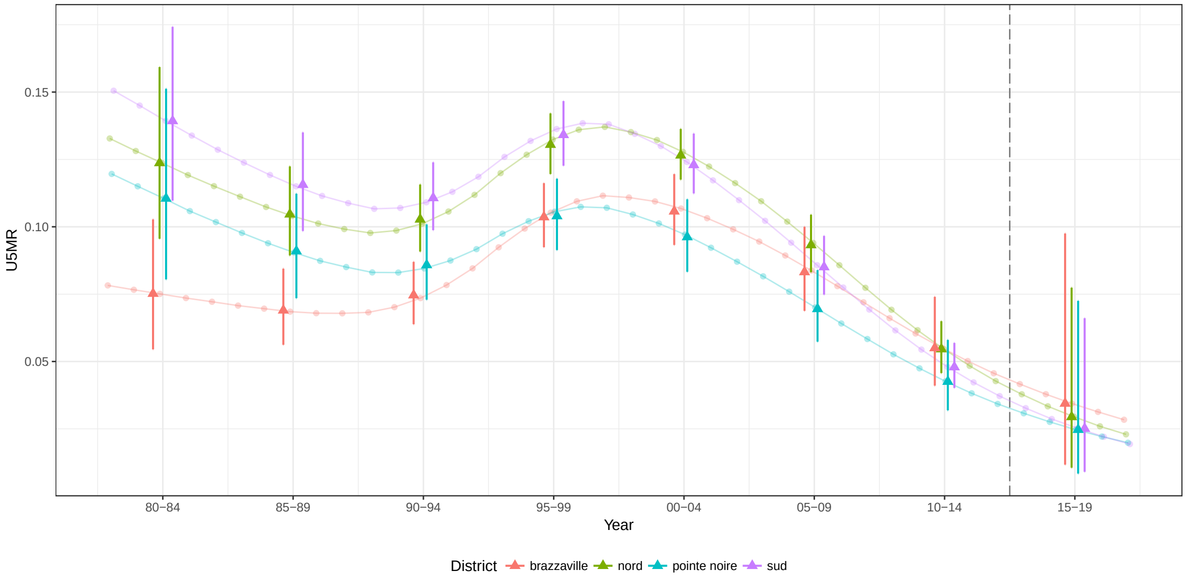

Figure S1.73: Congo: Smoothed regional estimates over time. The line indicates yearly posterior median estimates and error bars indicate 95 % posterior credible interval at each time period.

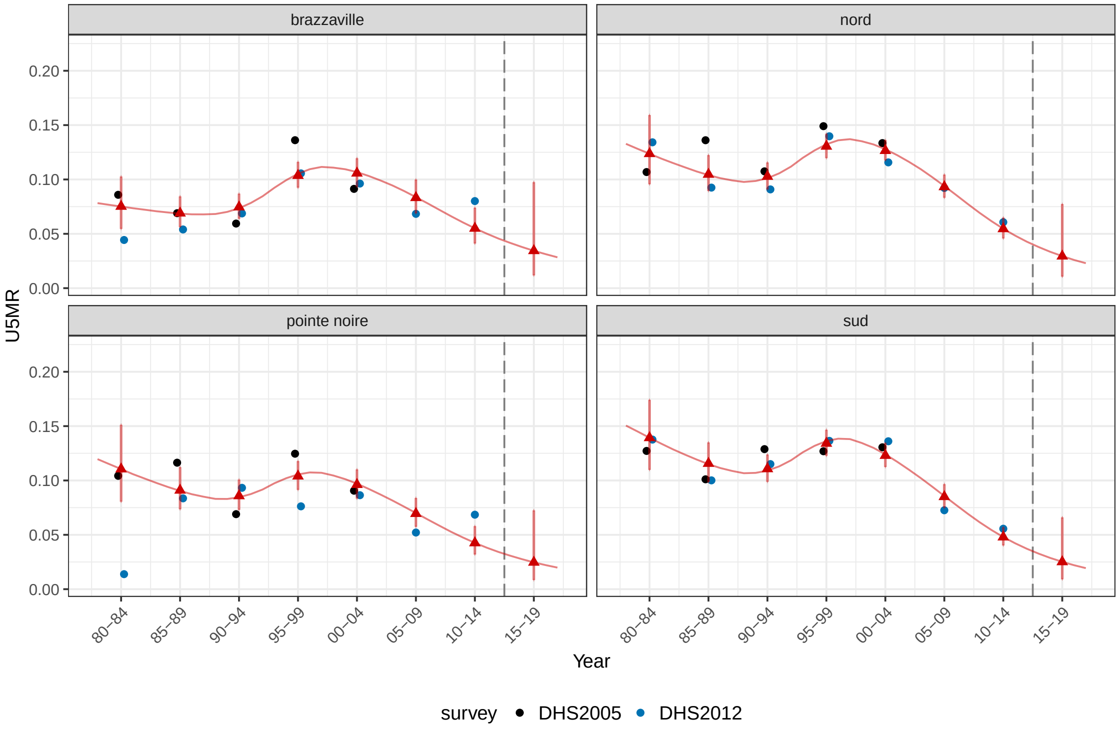

Figure S1.74: Congo: Smoothed regional estimates over time compared to the direct estimates from each surveys. Direct estimates are not benchmarked with UN estimates. The line indicates posterior median and error bars indicate 95% posterior credible interval.

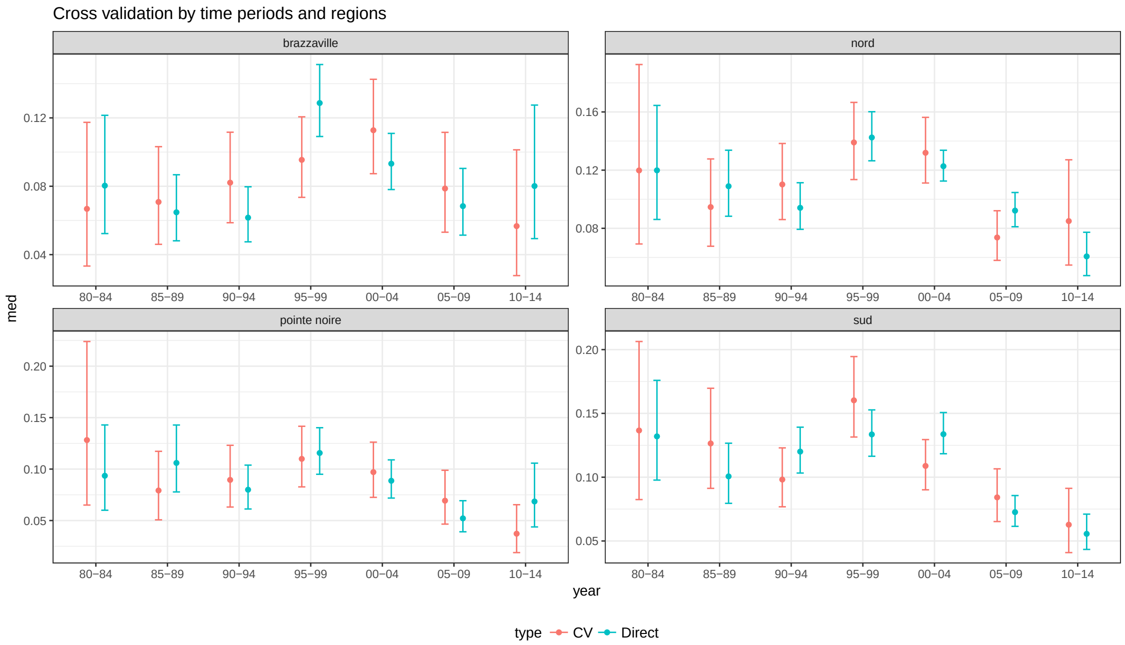

Figure S1.75: Congo: Out-of-sample predictions along with direct estimates in the cross validation study where data from one region in each time period is held out and predicted using the rest of the data.

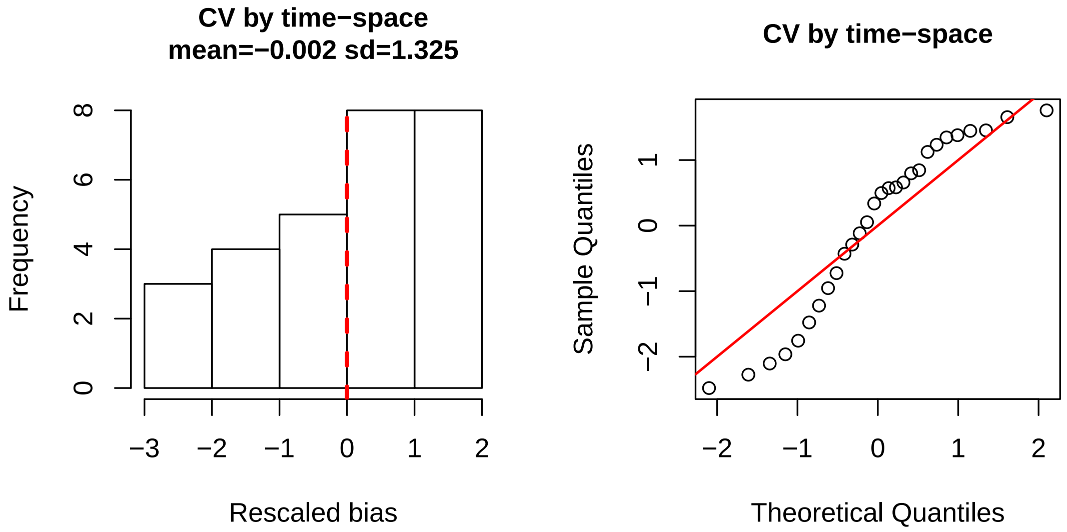

Figure S1.76: Congo: Histogram and QQ-plot of the rescaled difference between the smoothed estimates and the direct estimates in the cross validation study. The differences between the two estimates are rescaled by the square root of the total variance of the two estimates.

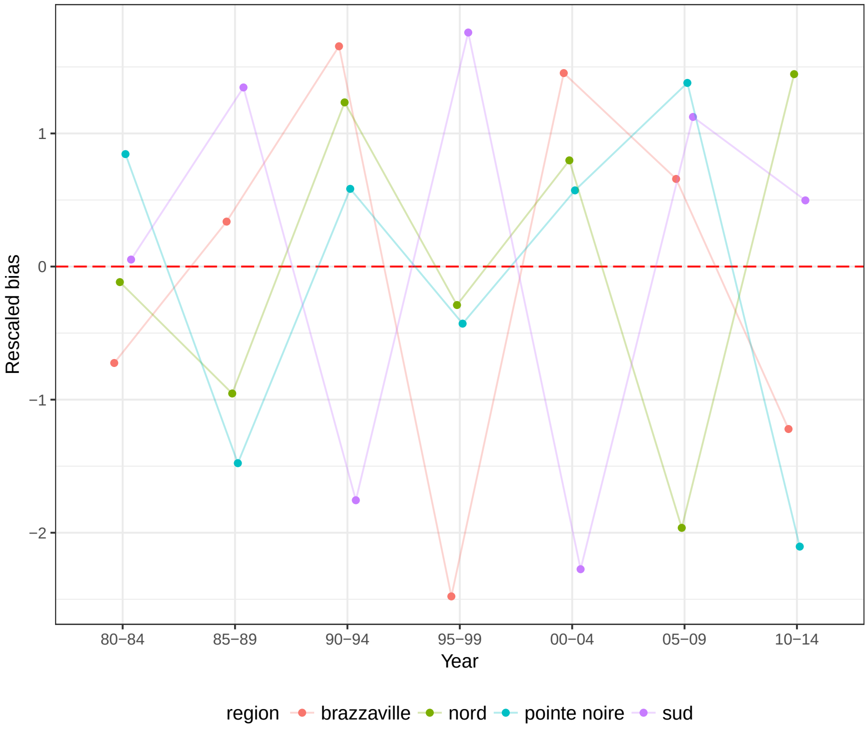

Figure S1.77: Congo: Line plot of the difference between smoothed estimates and the direct estimates in the cross validation study. The differences between the two estimates are rescaled by the square root of the total variance of the two estimates.

3.5.9 Côte d'Ivoire

DHS surveys were conducted in Côte d'Ivoire in 2011.

We fit both the RW2 only model to the combined national data, and compare the time trend at national level with the estimates produced by the UN and IHME in Figure S1.78. We then adjusted the combined national data to the UN estimates of U5MR, and refit the models on the benchmarked data.

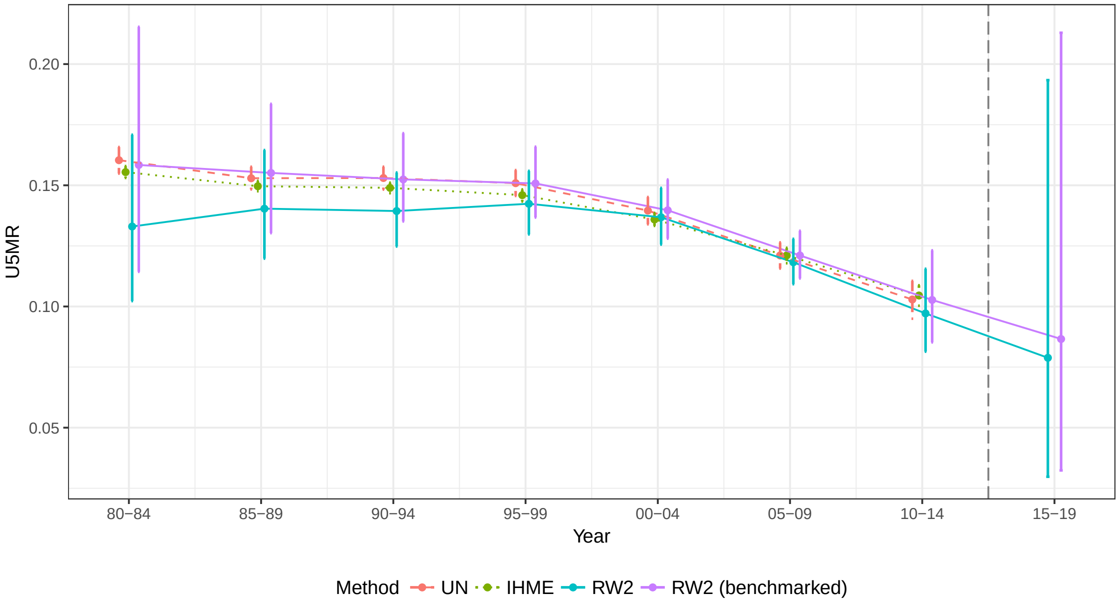

Figure S1.78: Côte d'Ivoire: Temporal national trends along with UN (B3) estimates described in You et al. (2015) and IHME estimates based on GBD 2015 Child Mortality Collaborators (2016). RW2 represents the smoothed national estimates using the original data before benchmarking with UN estimates. RW2-adj represents the smoothed national estimates using the benchmarked data.

We fit the RW2 model to the benchmarked data in each area. We compare the results in Figure S1.79 to S1.83. Figure S1.79 compares the smoothed estimates against the direct estimates. Figure S1.80 and Figure S1.81 show the posterior median estimates of U5MR in each region over time and the reductions from 1990 period respectively. Figure S1.82 shows the smoothed estimates by region over time and Figure S1.83 compares the smoothed estimates with direct estimates from each survey for each region over time.

We further assess the RW2 model by holding out some observations, and compare the projections to the direct estimates in these holdout observations. Figure S1.84 compares the predicted estimates for the out-of-sample observations with the direct estimates by holding out observations from each area in each time period. Figure S1.85 compares the histogram of the bias rescaled by the total variance in the cross validation studies. Figure S1.86 compares the rescaled bias by region and time periods.

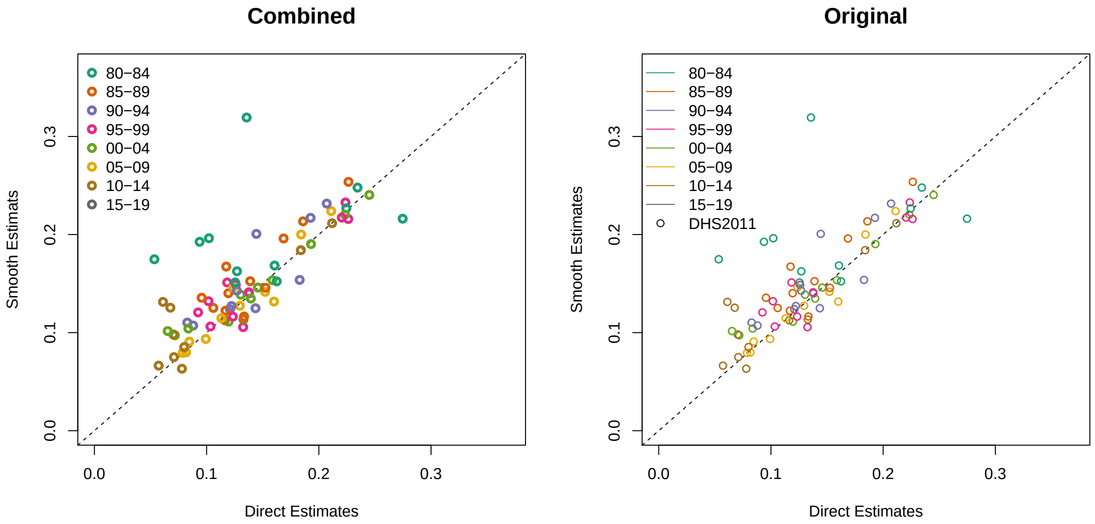

Figure S1.79: Côte d'Ivoire: Smooth versus direct Admin 1 estimates. Left: Combined (meta-analysis) survey estimate against combined direct estimates. Right: Combined (meta-analysis) survey estimate against direct estimates from each survey.

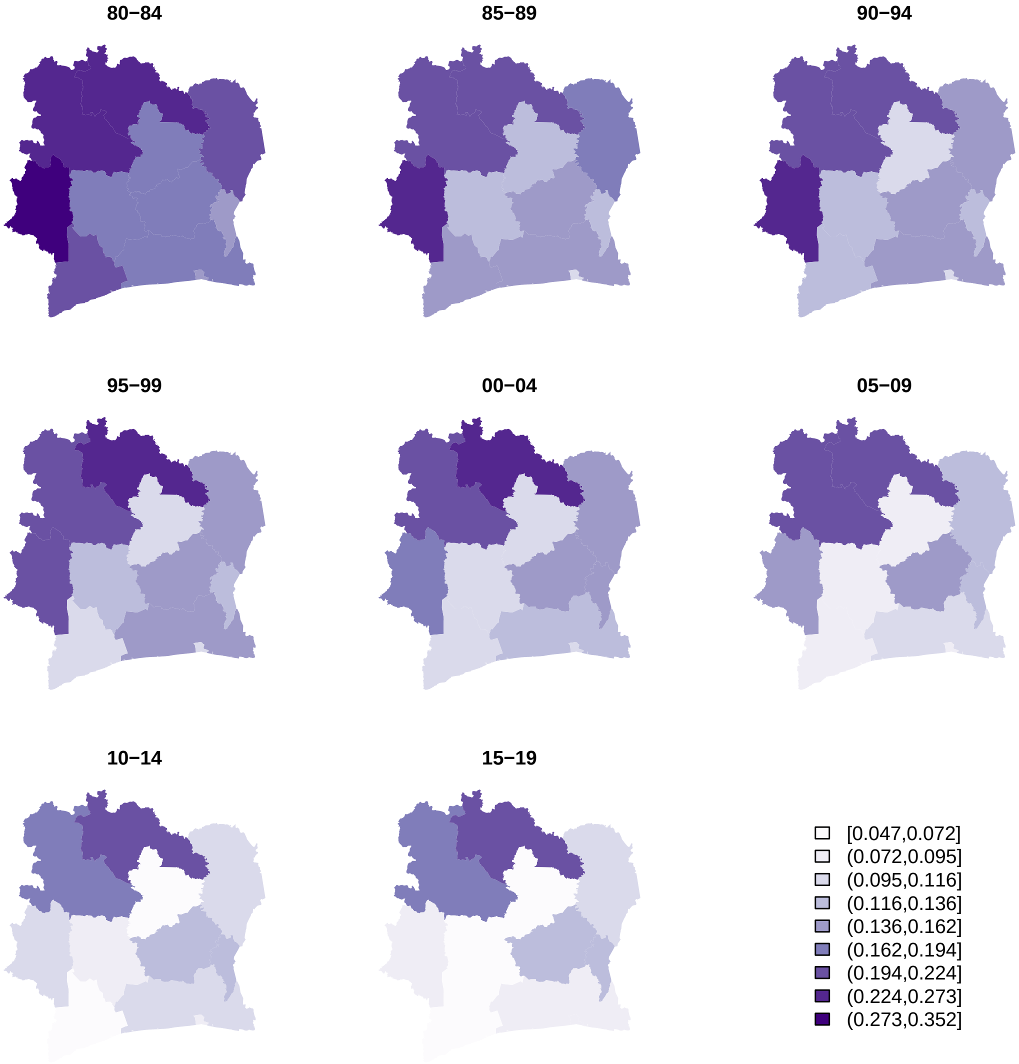

Figure S1.80: Côte d'Ivoire: Maps of posterior medians over time.

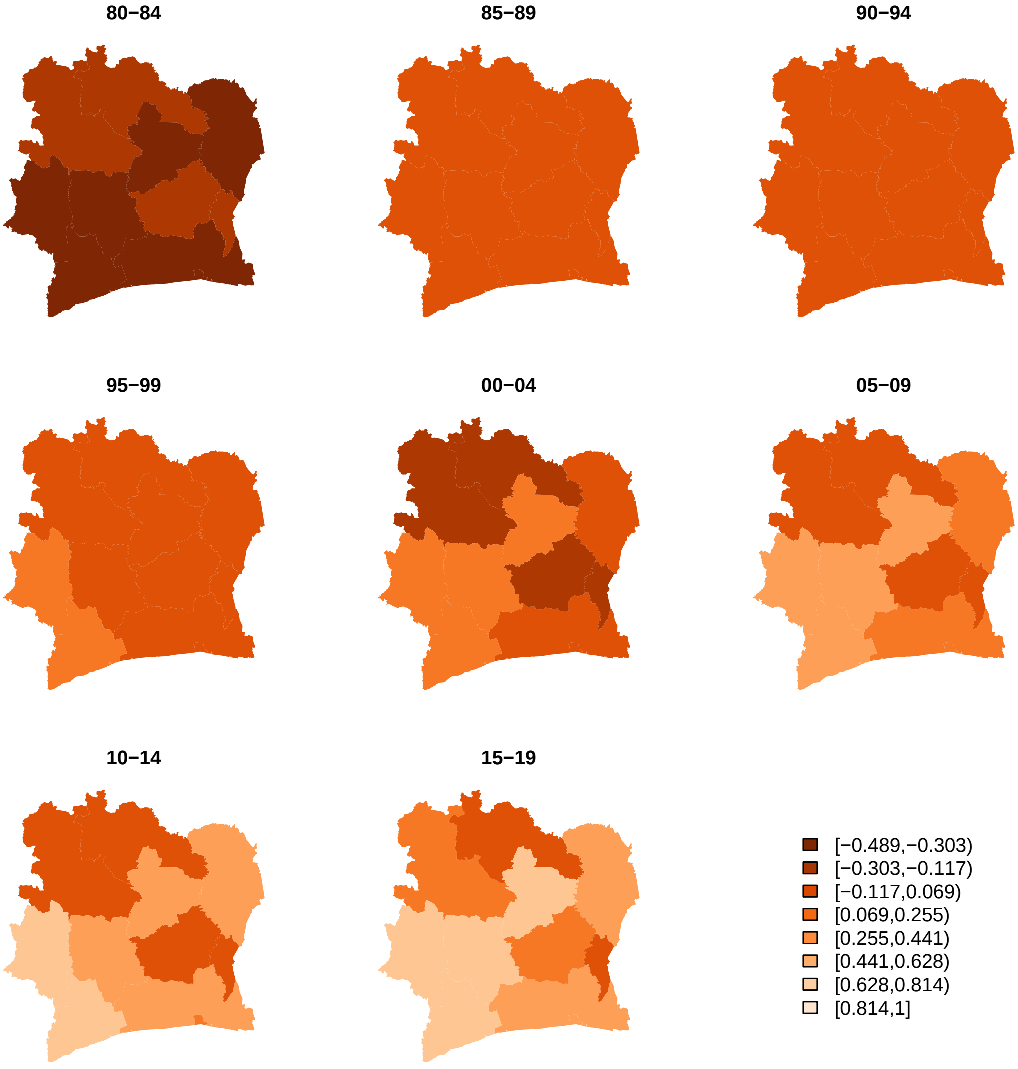

Figure S1.81: Côte d'Ivoire: Maps of reduction of posterior median U5MR in each five-year period compared to 1990 over time.

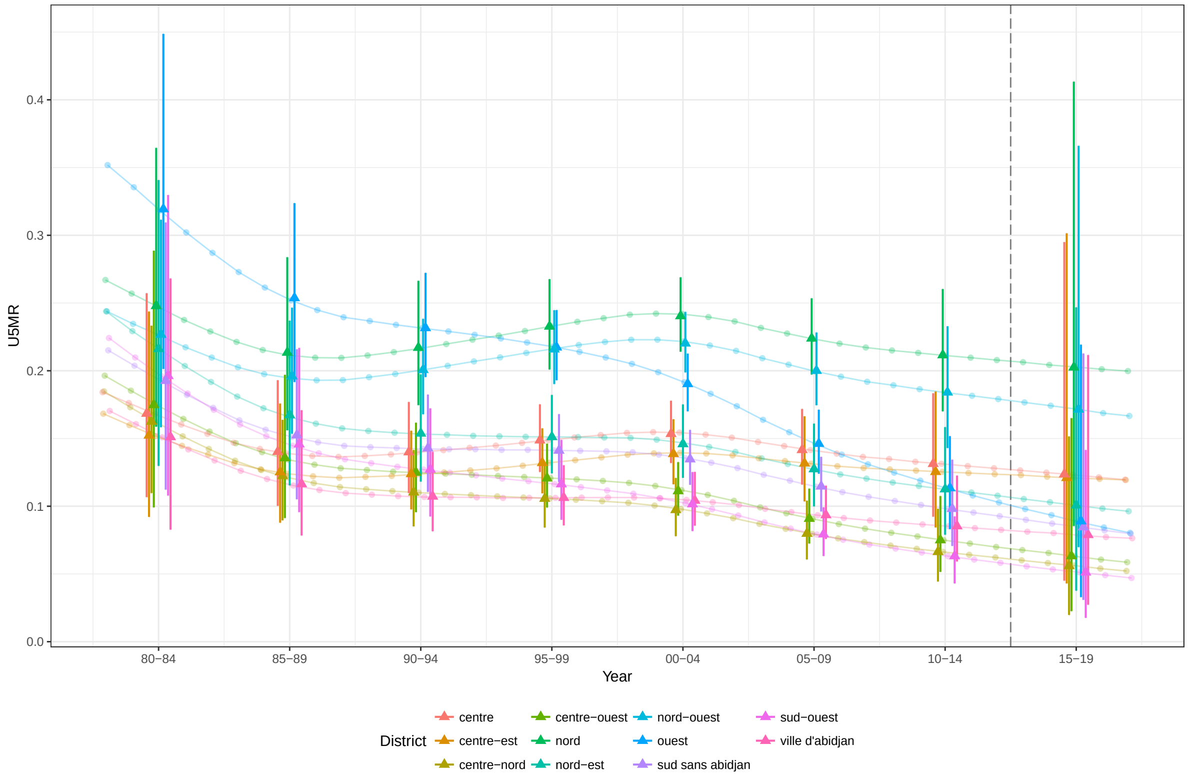

Figure S1.82: Côte d'Ivoire: Smoothed regional estimates over time. The line indicates yearly posterior median estimates and error bars indicate 95 % posterior credible interval at each time period.

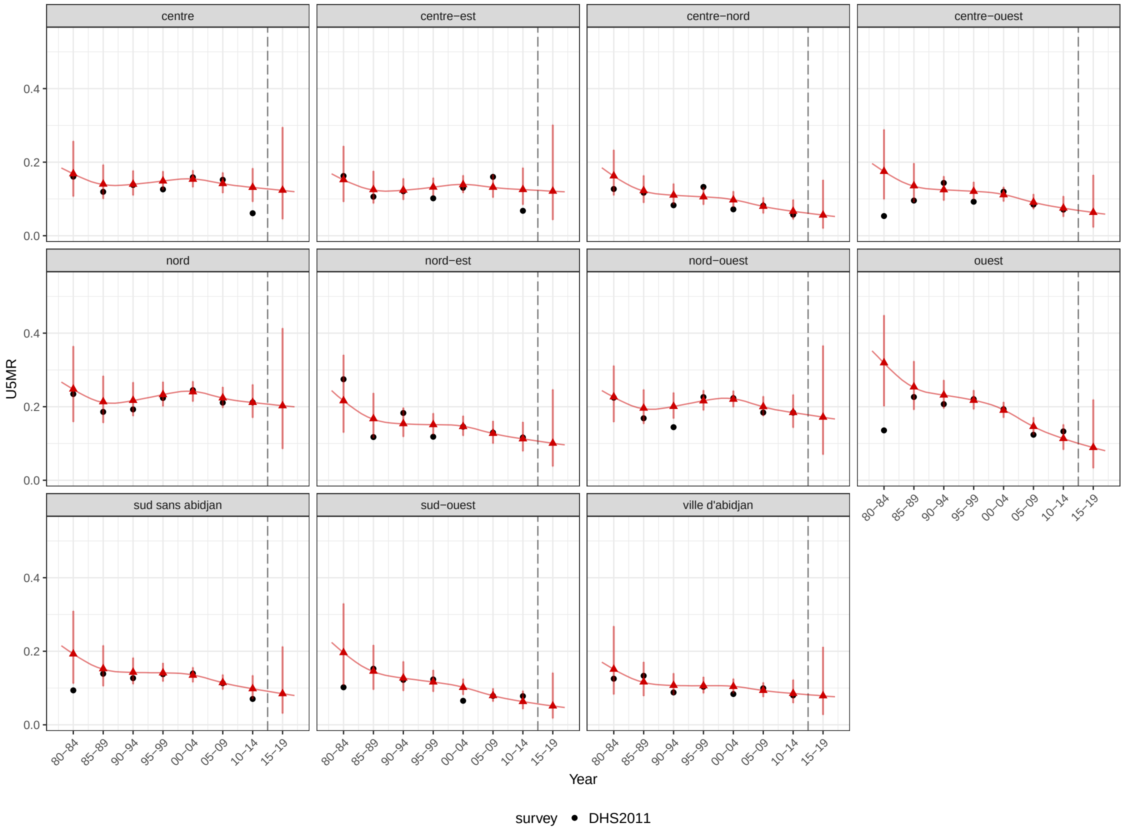

Figure S1.83: Côte d'Ivoire: Smoothed regional estimates over time compared to the direct estimates from each surveys. Direct estimates are not benchmarked with UN estimates. The line indicates posterior median and error bars indicate 95% posterior credible interval.

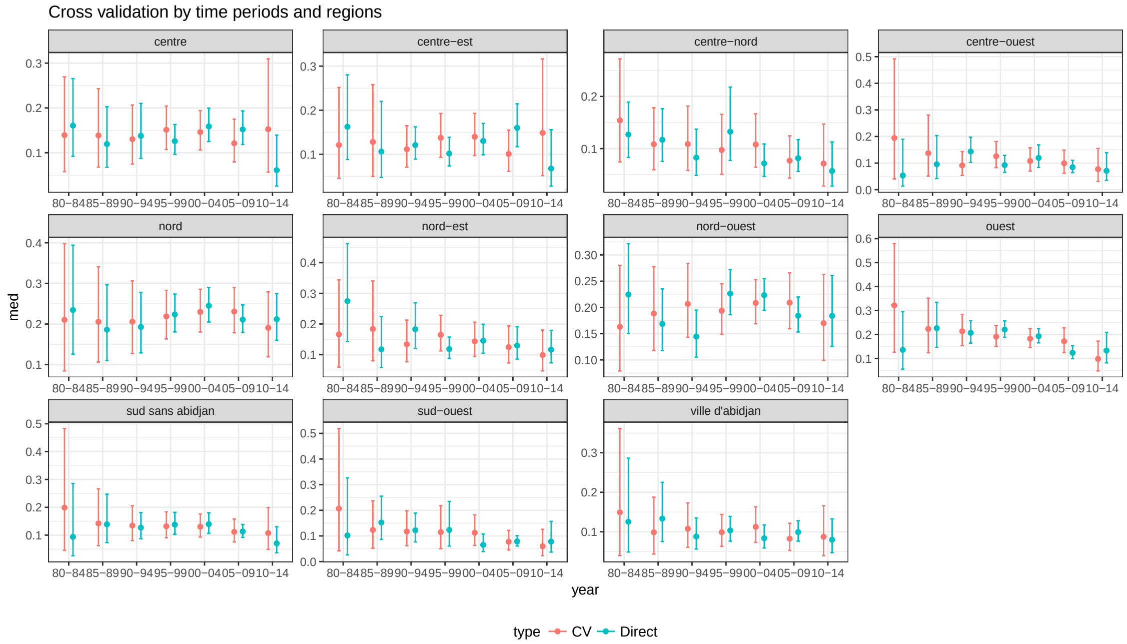

Figure S1.84: Côte d'Ivoire: Out-of-sample predictions along with direct estimates in the cross validation study where data from one region in each time period is held out and predicted using the rest of the data.

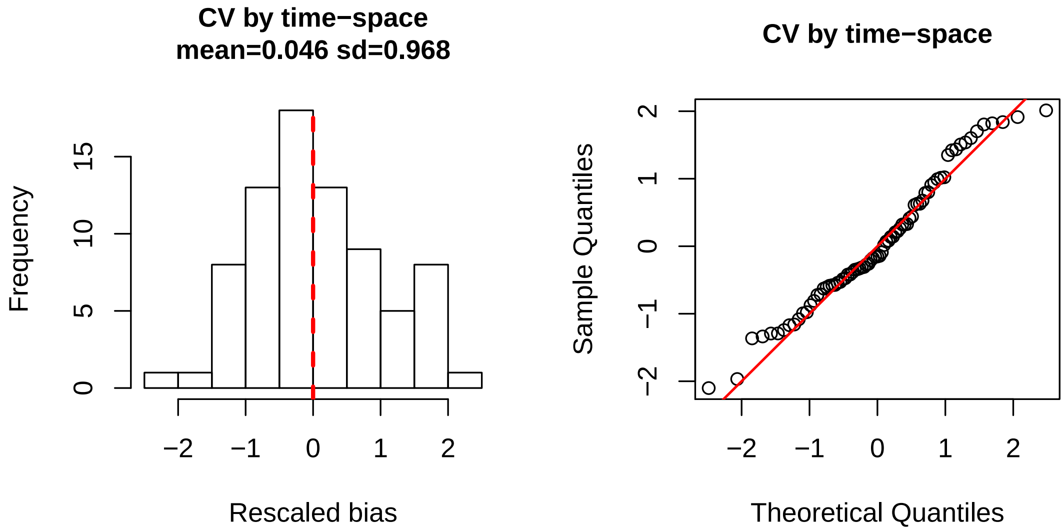

Figure S1.85: Côte d'Ivoire: Histogram and QQ-plot of the rescaled difference between the smoothed estimates and the direct estimates in the cross validation study. The differences between the two estimates are rescaled by the square root of the total variance of the two estimates.

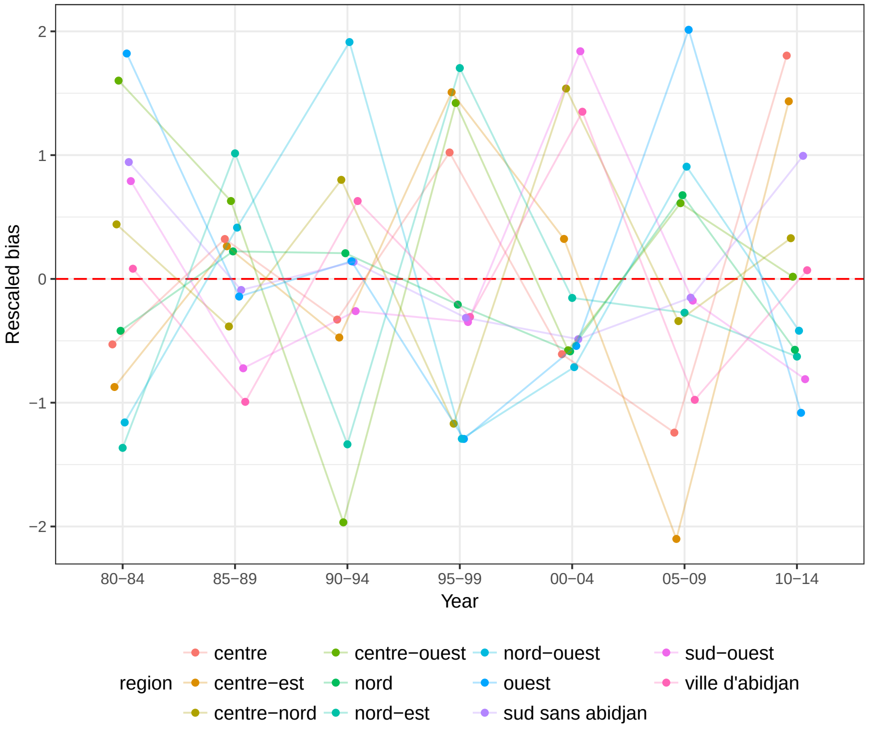

Figure S1.86: Côte d'Ivoire: Line plot of the difference between smoothed estimates and the direct estimates in the cross validation study. The differences between the two estimates are rescaled by the square root of the total variance of the two estimates.

3.5.10 DRC

DHS surveys were conducted in DRC in 2007, and 2014.

We fit both the RW2 only model to the combined national data, and compare the time trend at national level with the estimates produced by the UN and IHME in Figure S1.87. We then adjusted the combined national data to the UN estimates of U5MR, and refit the models on the benchmarked data.

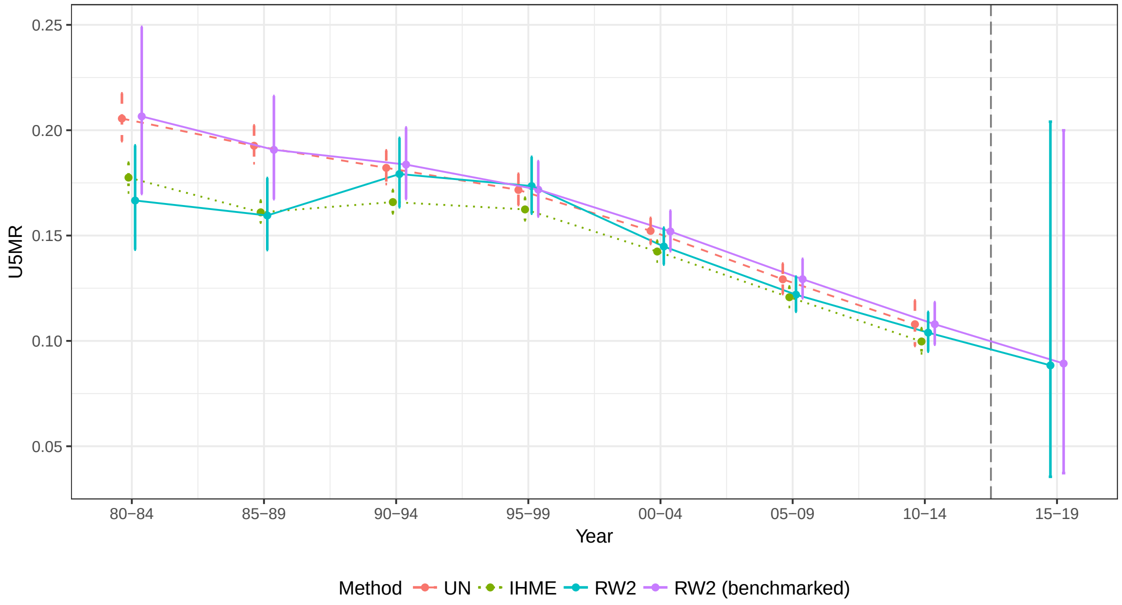

Figure S1.87: DRC: Temporal national trends along with UN (B3) estimates described in You et al. (2015) and IHME estimates based on GBD 2015 Child Mortality Collaborators (2016). RW2 represents the smoothed national estimates using the original data before benchmarking with UN estimates. RW2-adj represents the smoothed national estimates using the benchmarked data.

We fit the RW2 model to the benchmarked data in each area. We compare the results in Figure S1.88 to S1.92. Figure S1.88 compares the smoothed estimates against the direct estimates. Figure S1.89 and Figure S1.90 show the posterior median estimates of U5MR in each region over time and the reductions from 1990 period respectively. Figure S1.91 shows the smoothed estimates by region over time and Figure S1.92 compares the smoothed estimates with direct estimates from each survey for each region over time.

We further assess the RW2 model by holding out some observations, and compare the projections to the direct estimates in these holdout observations. Figure S1.93 compares the predicted estimates for the out-of-sample observations with the direct estimates by holding out observations from each area in each time period. Figure S1.94 compares the histogram of the bias rescaled by the total variance in the cross validation studies. Figure S1.95 compares the rescaled bias by region and time periods.

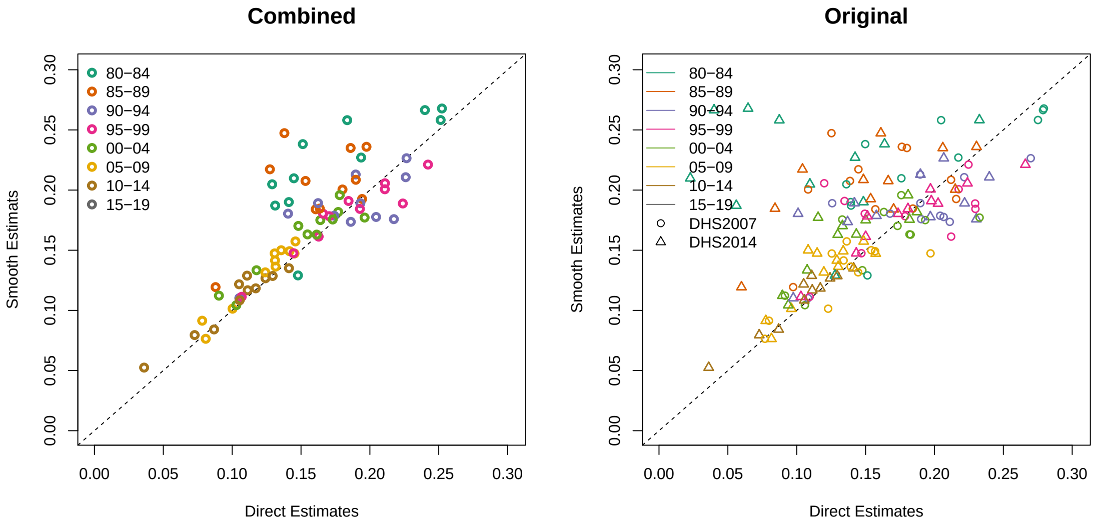

Figure S1.88: DRC: Smooth versus direct Admin 1 estimates. Left: Combined (meta-analysis) survey estimate against combined direct estimates. Right: Combined (meta-analysis) survey estimate against direct estimates from each survey.

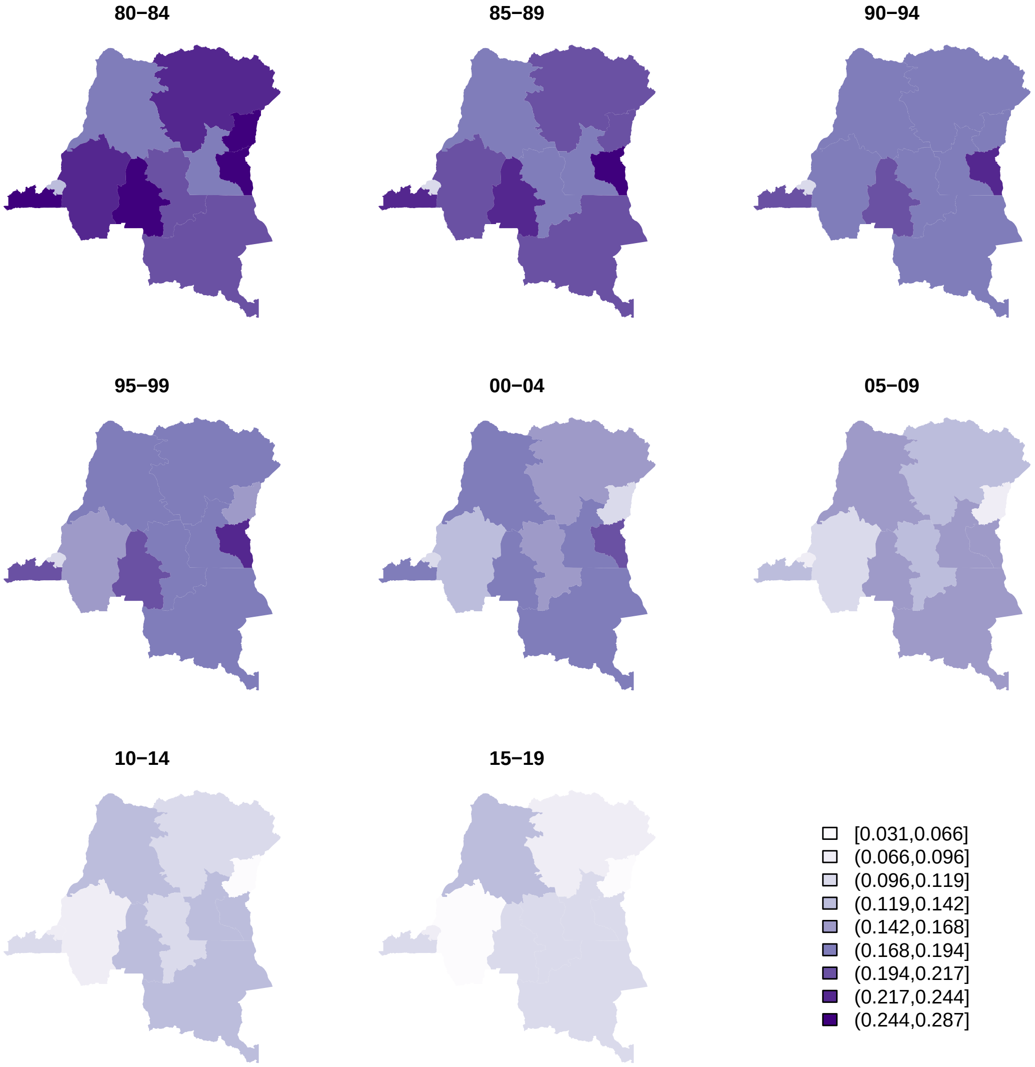

Figure S1.89: DRC: Maps of posterior medians over time.

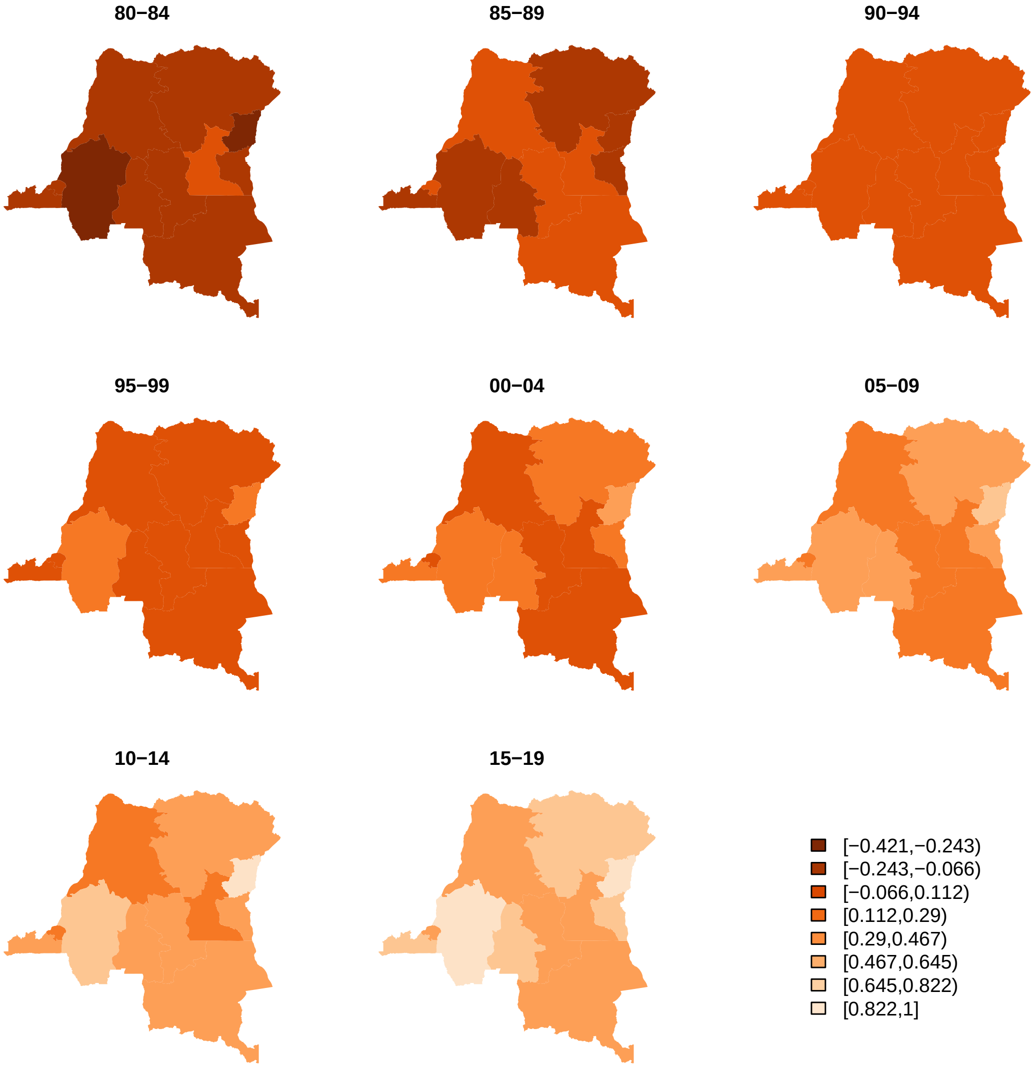

Figure S1.90: DRC: Maps of reduction of posterior median U5MR in each five-year period compared to 1990 over time.

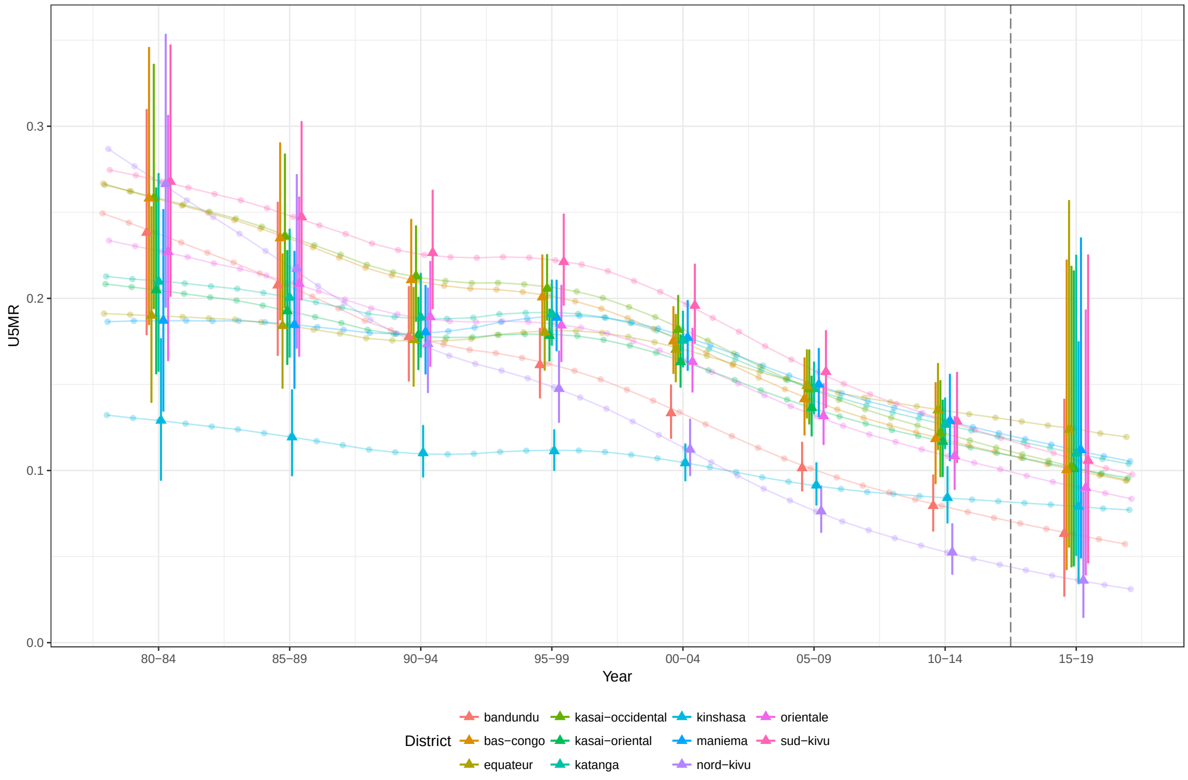

Figure S1.91: DRC: Smoothed regional estimates over time. The line indicates yearly posterior median estimates and error bars indicate 95 % posterior credible interval at each time period.

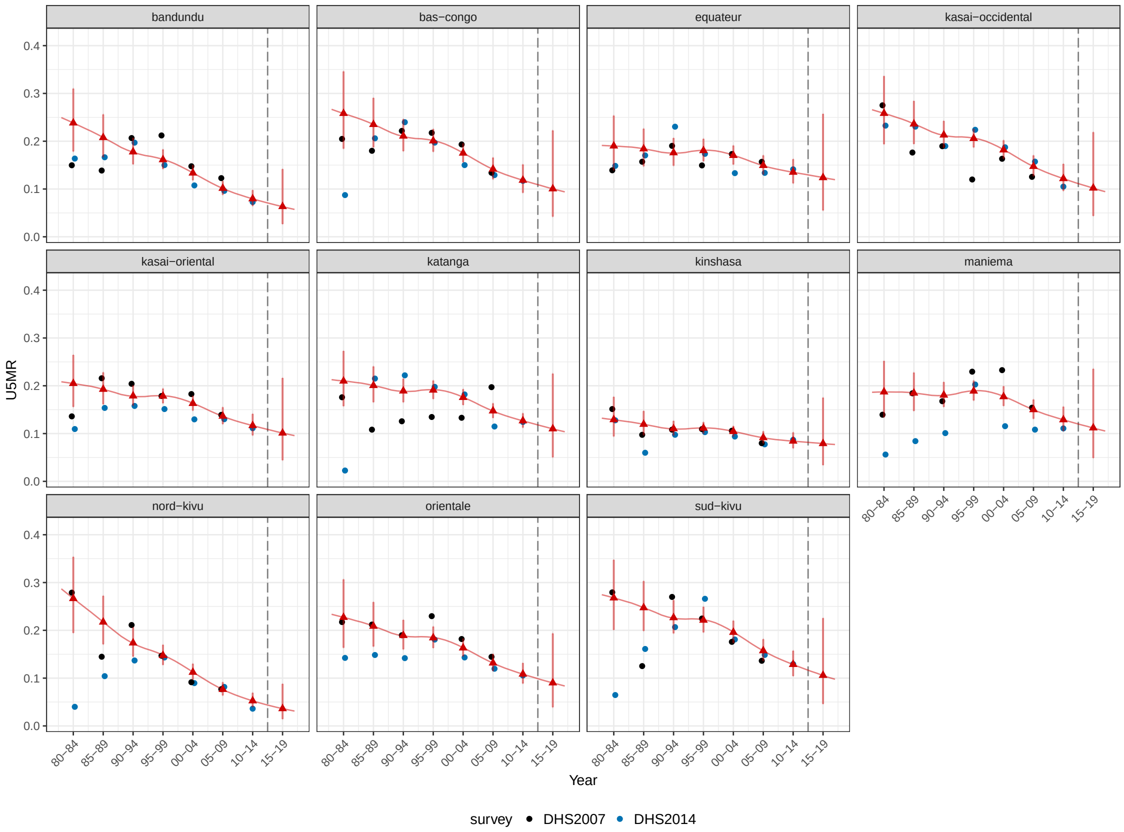

Figure S1.92: DRC: Smoothed regional estimates over time compared to the direct estimates from each surveys. Direct estimates are not benchmarked with UN estimates. The line indicates posterior median and error bars indicate 95% posterior credible interval.

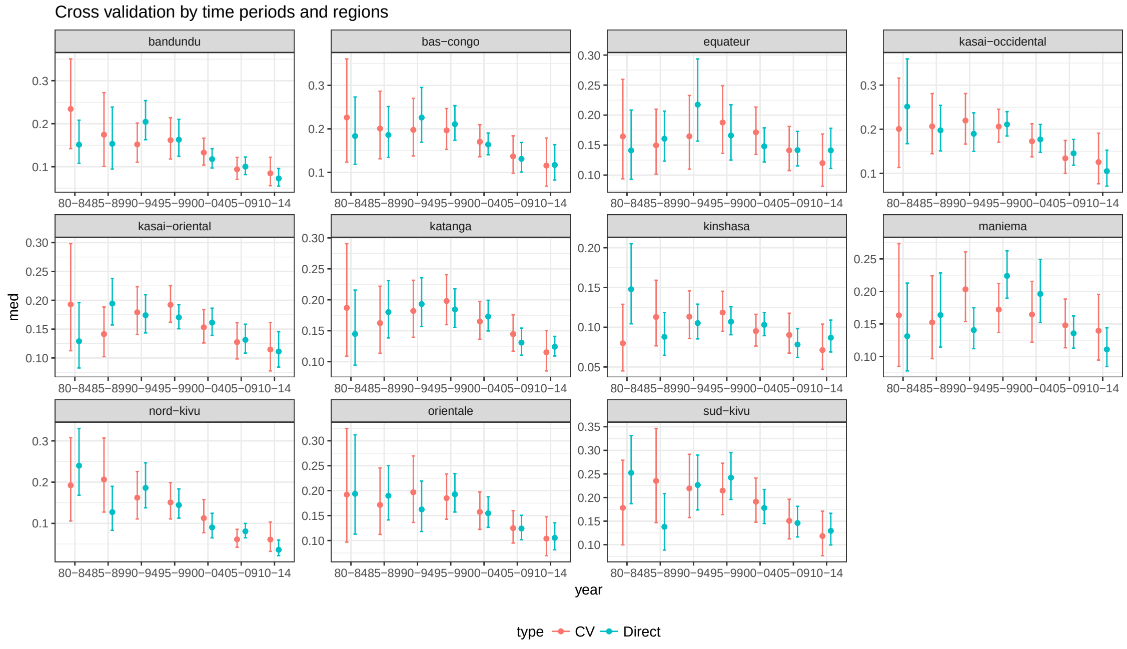

Figure S1.93: DRC: Out-of-sample predictions along with direct estimates in the cross validation study where data from one region in each time period is held out and predicted using the rest of the data.

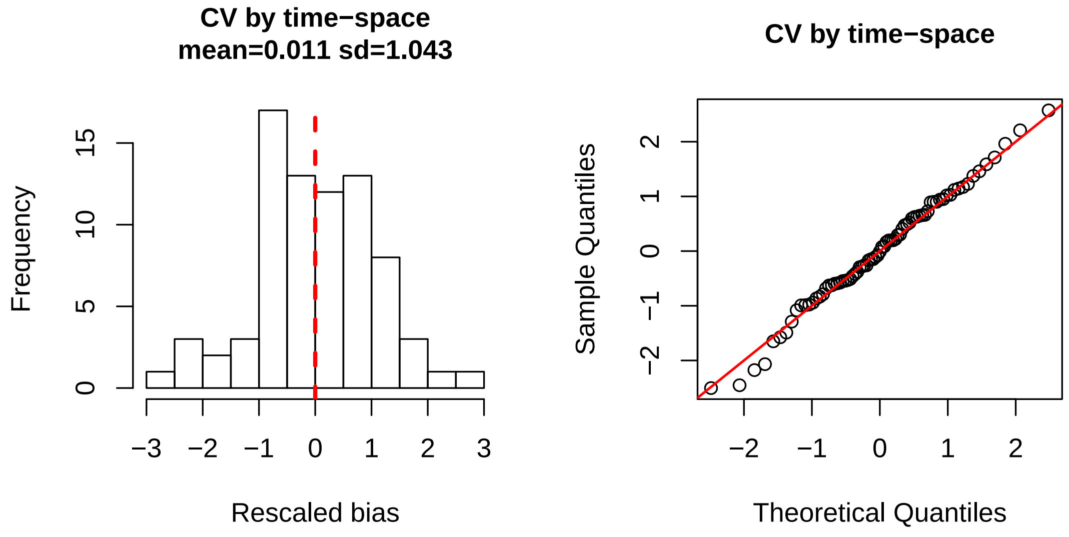

Figure S1.94: DRC: Histogram and QQ-plot of the rescaled difference between the smoothed estimates and the direct estimates in the cross validation study. The differences between the two estimates are rescaled by the square root of the total variance of the two estimates.

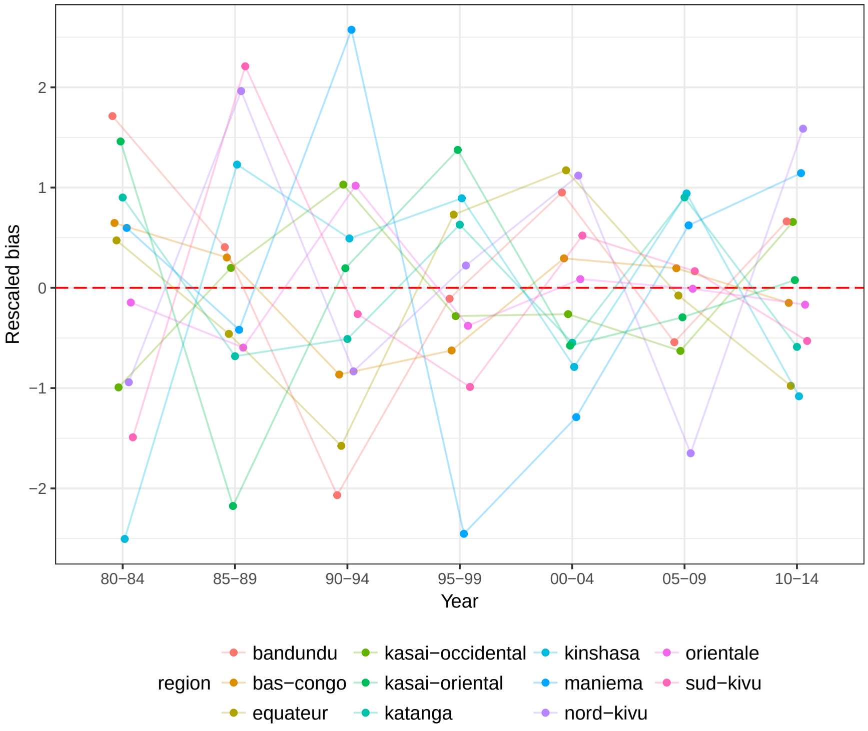

Figure S1.95: DRC: Line plot of the difference between smoothed estimates and the direct estimates in the cross validation study. The differences between the two estimates are rescaled by the square root of the total variance of the two estimates.

3.5.11 Egypt

DHS surveys were conducted in Egypt in 1988, 1992, 1995, 2000, 2003, 2005, 2008, and 2014.

We fit both the RW2 only model to the combined national data, and compare the time trend at national level with the estimates produced by the UN and IHME in Figure S1.96. We then adjusted the combined national data to the UN estimates of U5MR, and refit the models on the benchmarked data.

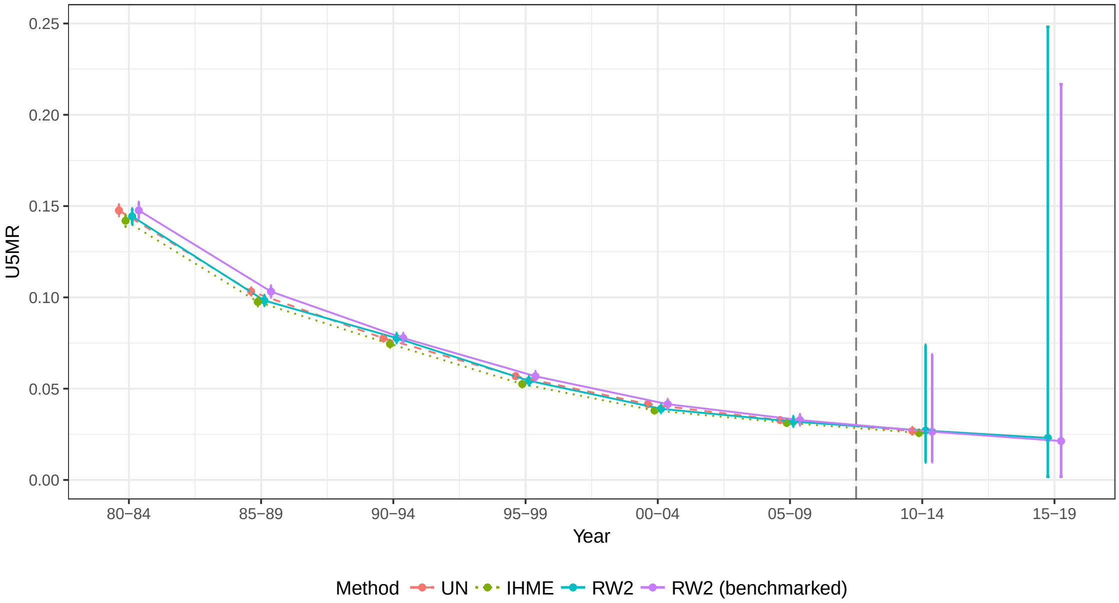

Figure S1.96: Egypt: Temporal national trends along with UN (B3) estimates described in You et al. (2015) and IHME estimates based on GBD 2015 Child Mortality Collaborators (2016). RW2 represents the smoothed national estimates using the original data before benchmarking with UN estimates. RW2-adj represents the smoothed national estimates using the benchmarked data.

We fit the RW2 model to the benchmarked data in each area. We compare the results in Figure S1.97 to S1.101. Figure S1.97 compares the smoothed estimates against the direct estimates. Figure S1.98 and Figure S1.99 show the posterior median estimates of U5MR in each region over time and the reductions from 1990 period respectively. Figure S1.100 shows the smoothed estimates by region over time and Figure S1.101 compares the smoothed estimates with direct estimates from each survey for each region over time.

We further assess the RW2 model by holding out some observations, and compare the projections to the direct estimates in these holdout observations. Figure S1.102 compares the predicted estimates for the out-of-sample observations with the direct estimates by holding out observations from each area in each time period. Figure S1.103 compares the histogram of the bias rescaled by the total variance in the cross validation studies. Figure S1.104 compares the rescaled bias by region and time periods.

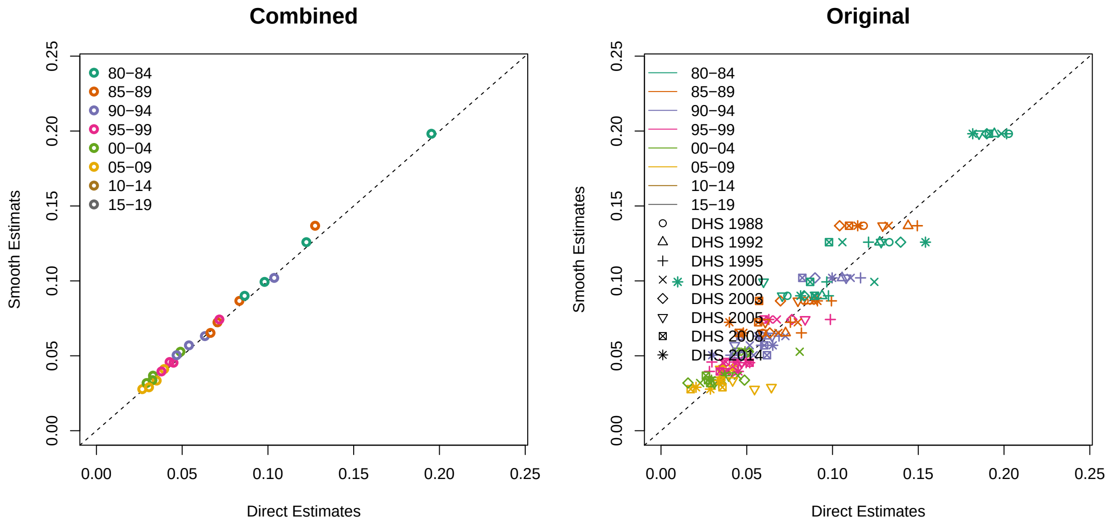

Figure S1.97: Egypt: Smooth versus direct Admin 1 estimates. Left: Combined (meta-analysis) survey estimate against combined direct estimates. Right: Combined (meta-analysis) survey estimate against direct estimates from each survey.

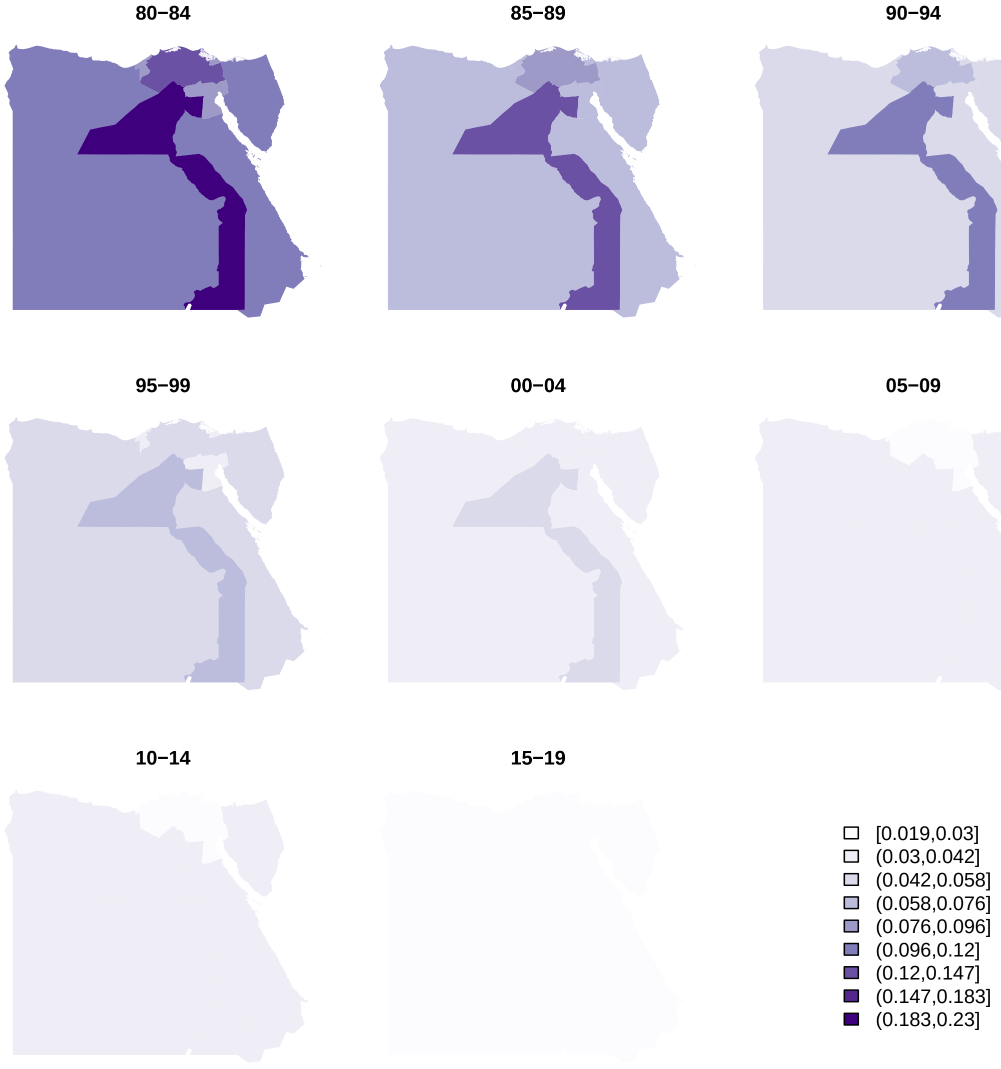

Figure S1.98: Egypt: Maps of posterior medians over time.

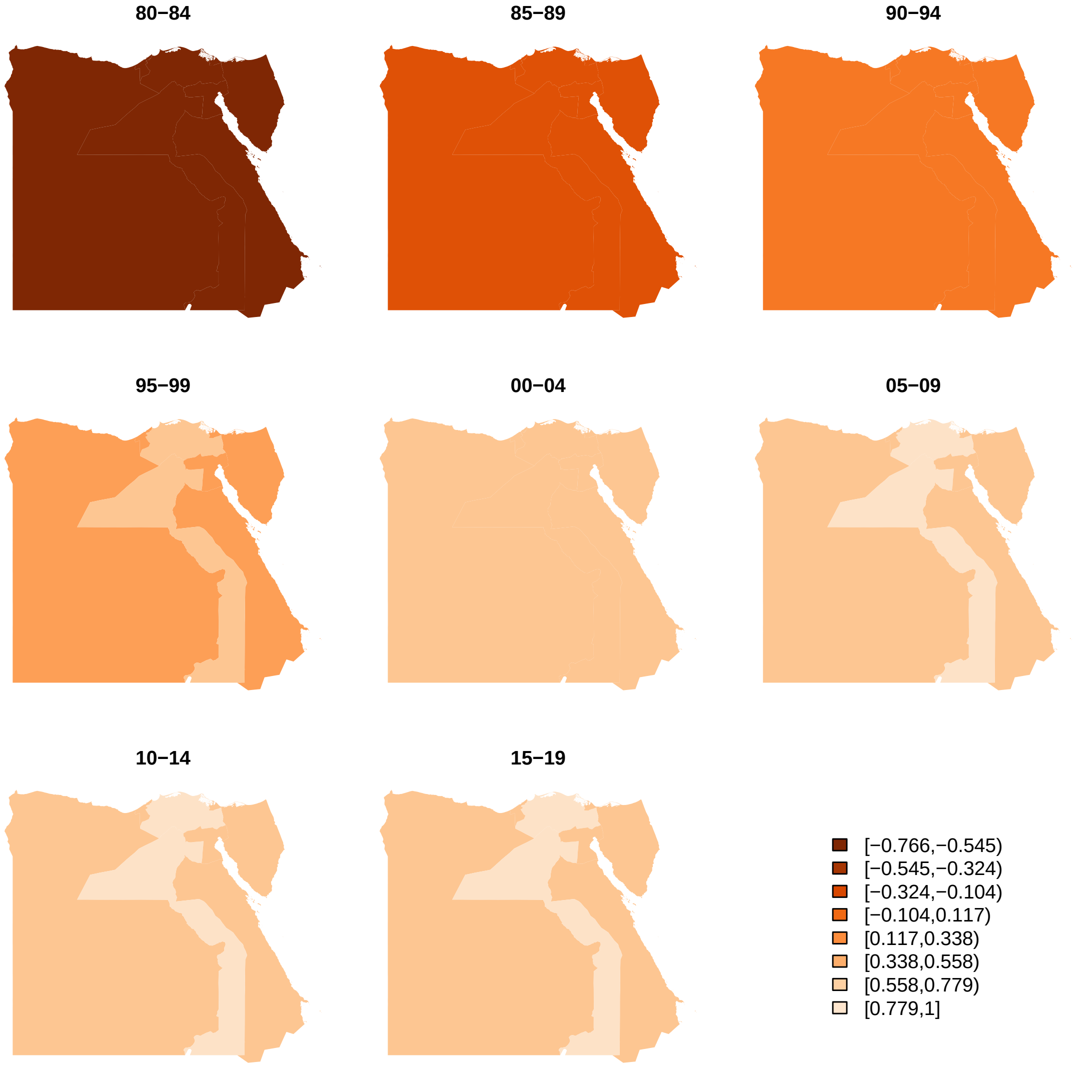

Figure S1.99: Egypt: Maps of reduction of posterior median U5MR in each five-year period compared to 1990 over time.

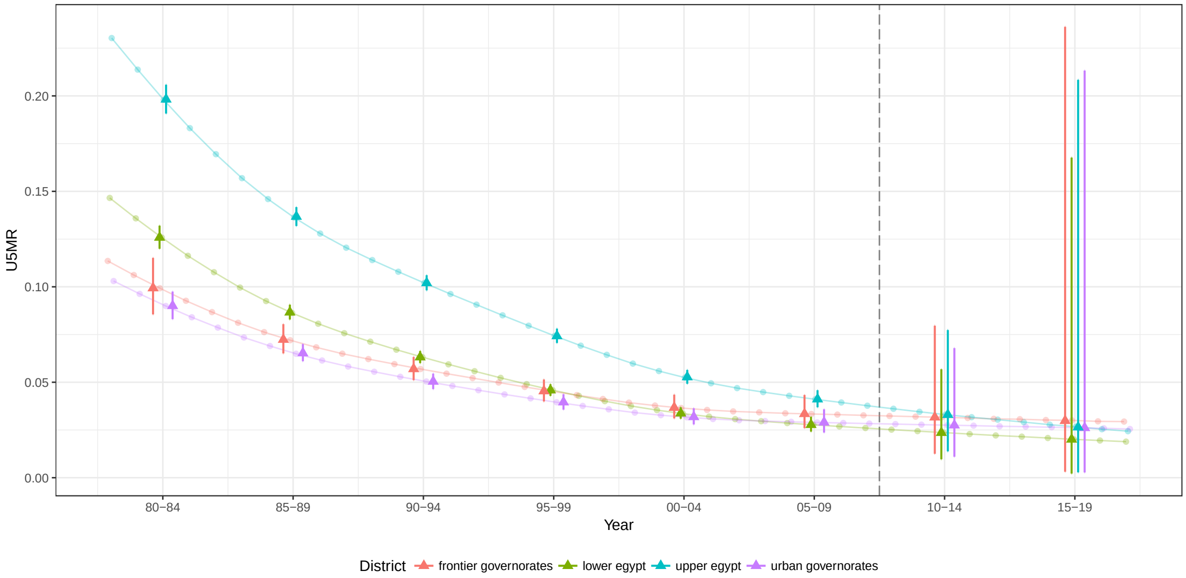

Figure S1.100: Egypt: Smoothed regional estimates over time. The line indicates yearly posterior median estimates and error bars indicate 95 % posterior credible interval at each time period.

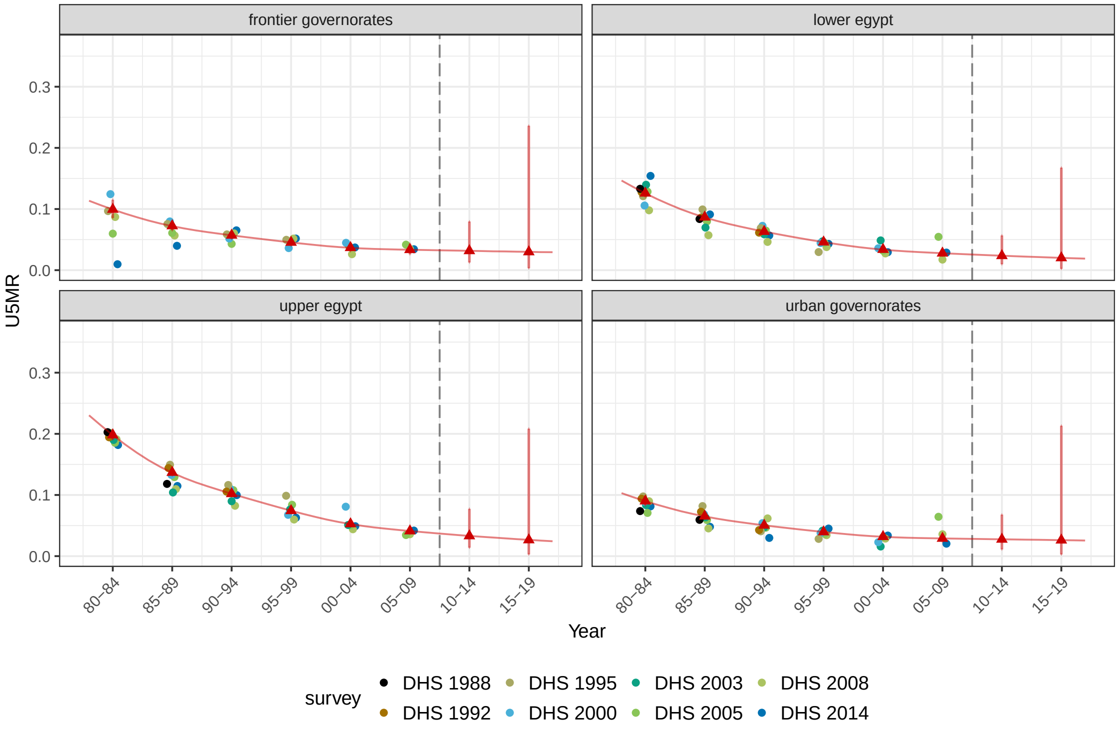

Figure S1.101: Egypt: Smoothed regional estimates over time compared to the direct estimates from each surveys. Direct estimates are not benchmarked with UN estimates. The line indicates posterior median and error bars indicate 95% posterior credible interval.

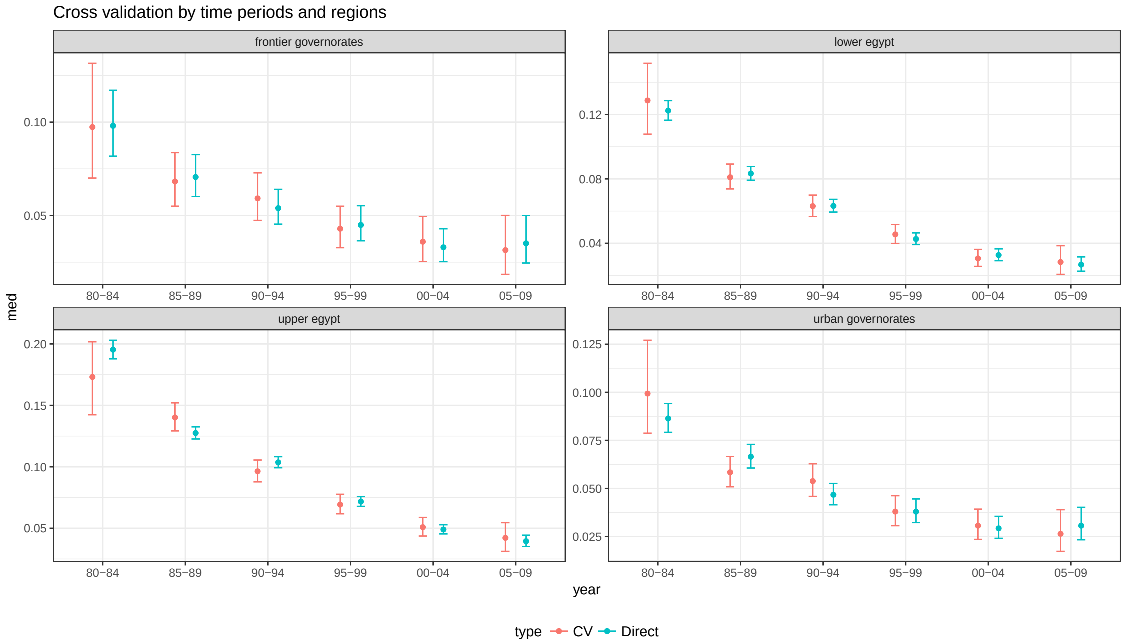

Figure S1.102: Egypt: Out-of-sample predictions along with direct estimates in the cross validation study where data from one region in each time period is held out and predicted using the rest of the data.

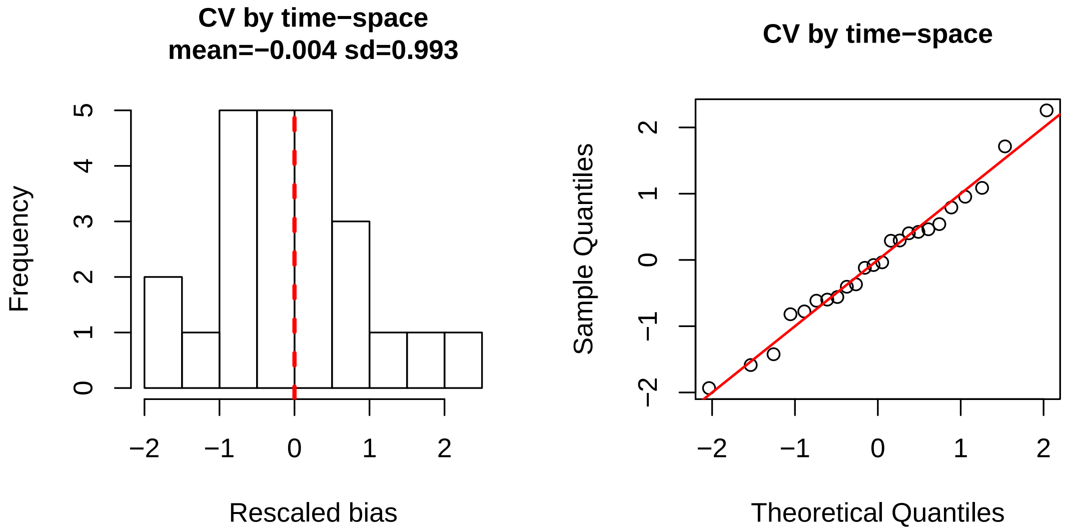

Figure S1.103: Egypt: Histogram and QQ-plot of the rescaled difference between the smoothed estimates and the direct estimates in the cross validation study. The differences between the two estimates are rescaled by the square root of the total variance of the two estimates.

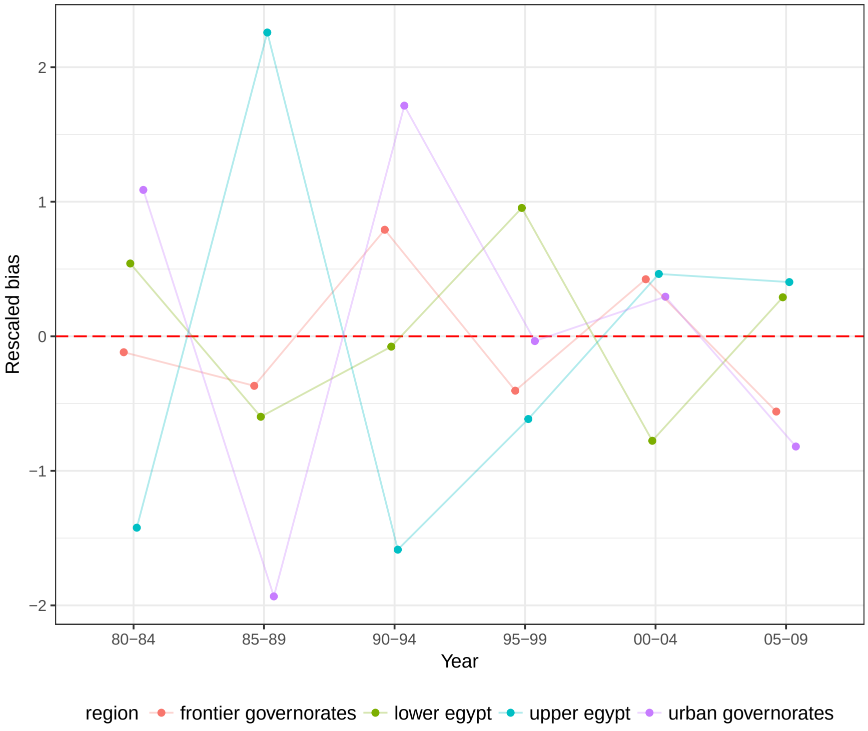

Figure S1.104: Egypt: Line plot of the difference between smoothed estimates and the direct estimates in the cross validation study. The differences between the two estimates are rescaled by the square root of the total variance of the two estimates.

3.5.12 Ethiopia

DHS surveys were conducted in Ethiopia in 2000, 2005, 2011, and 2016.

We fit both the RW2 only model to the combined national data, and compare the time trend at national level with the estimates produced by the UN and IHME in Figure S1.105. We then adjusted the combined national data to the UN estimates of U5MR, and refit the models on the benchmarked data.

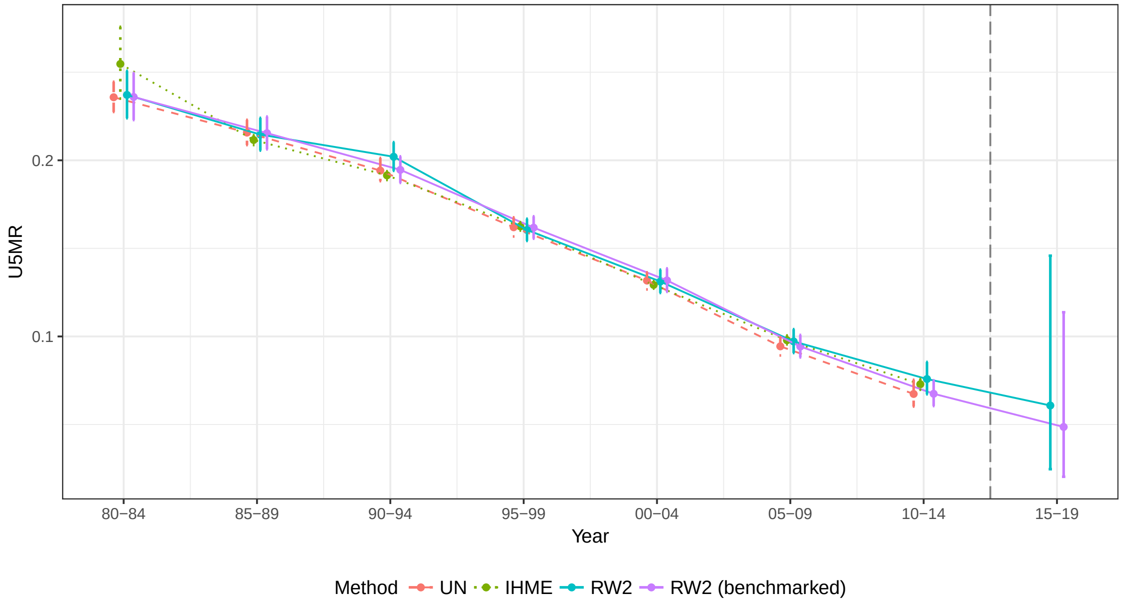

Figure S1.105: Ethiopia: Temporal national trends along with UN (B3) estimates described in You et al. (2015) and IHME estimates based on GBD 2015 Child Mortality Collaborators (2016). RW2 represents the smoothed national estimates using the original data before benchmarking with UN estimates. RW2-adj represents the smoothed national estimates using the benchmarked data.

We fit the RW2 model to the benchmarked data in each area. We compare the results in Figure S1.106 to S1.110. Figure S1.106 compares the smoothed estimates against the direct estimates. Figure S1.107 and Figure S1.108 show the posterior median estimates of U5MR in each region over time and the reductions from 1990 period respectively. Figure S1.109 shows the smoothed estimates by region over time and Figure S1.110 compares the smoothed estimates with direct estimates from each survey for each region over time.

We further assess the RW2 model by holding out some observations, and compare the projections to the direct estimates in these holdout observations. Figure S1.111 compares the predicted estimates for the out-of-sample observations with the direct estimates by holding out observations from each area in each time period. Figure S1.112 compares the histogram of the bias rescaled by the total variance in the cross validation studies. Figure S1.113 compares the rescaled bias by region and time periods.

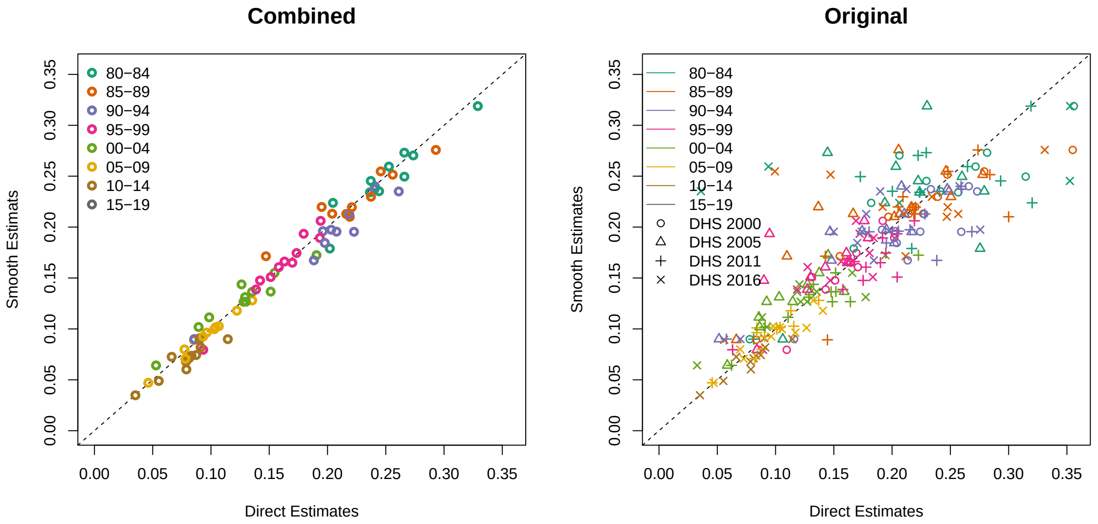

Figure S1.106: Ethiopia: Smooth versus direct Admin 1 estimates. Left: Combined (meta-analysis) survey estimate against combined direct estimates. Right: Combined (meta-analysis) survey estimate against direct estimates from each survey.

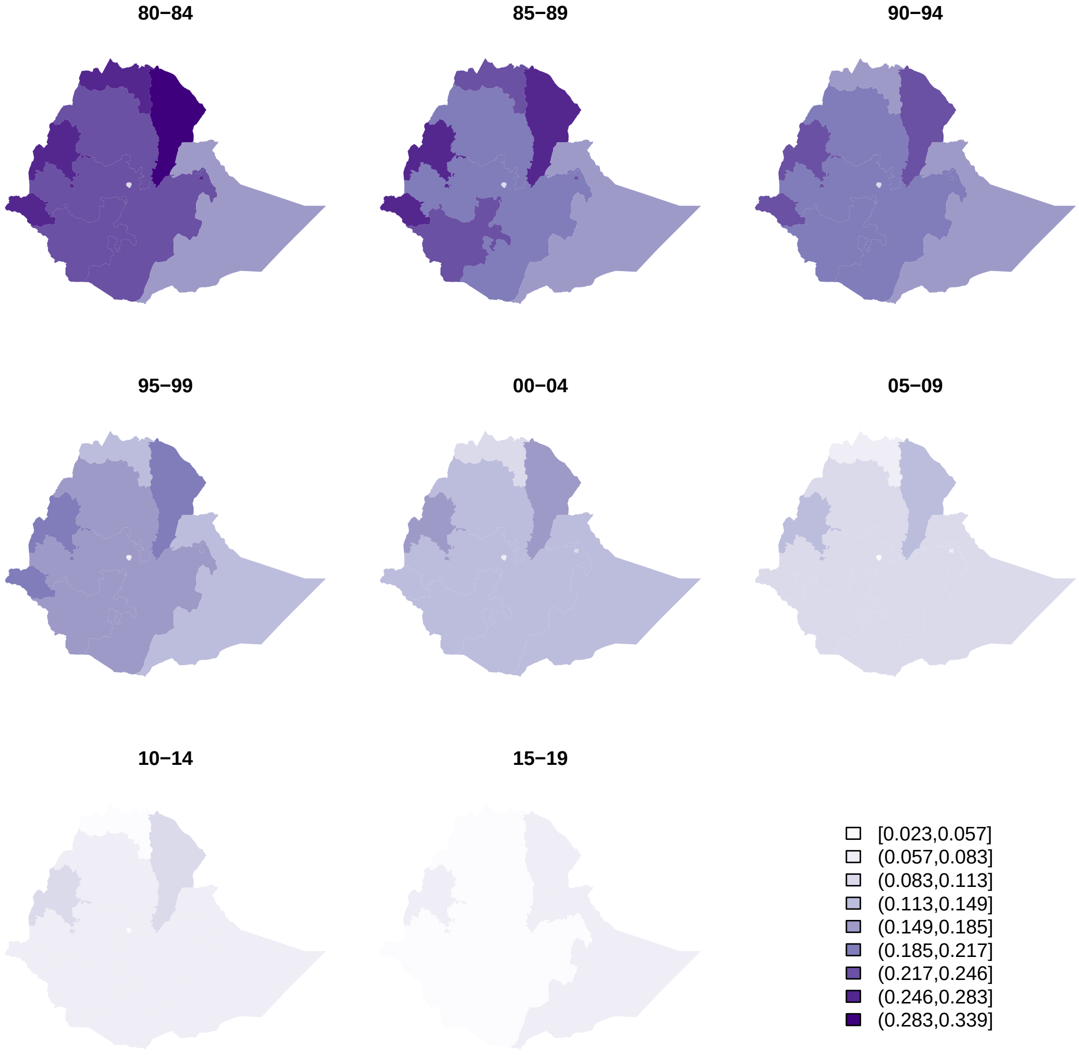

Figure S1.107: Ethiopia: Maps of posterior medians over time.

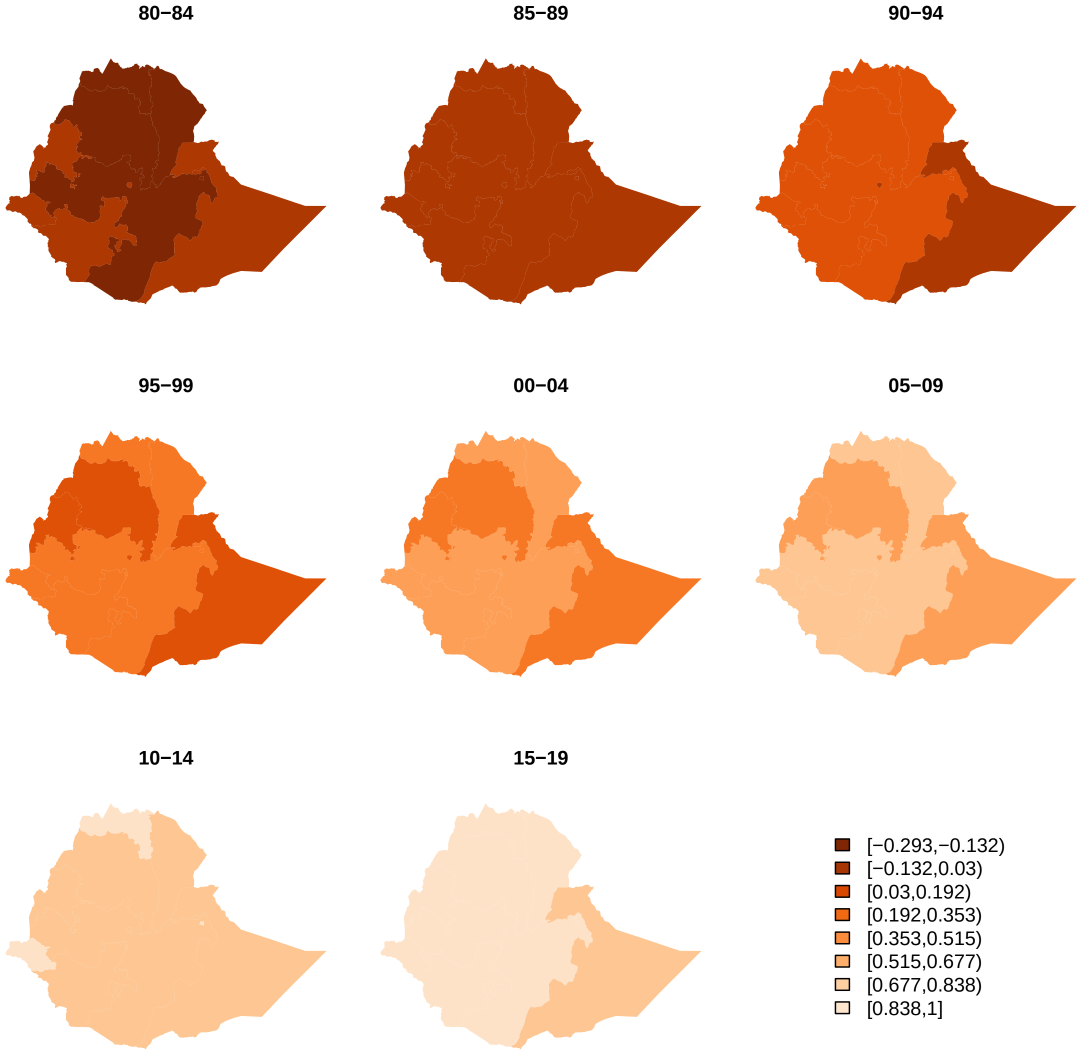

Figure S1.108: Ethiopia: Maps of reduction of posterior median U5MR in each five-year period compared to 1990 over time.

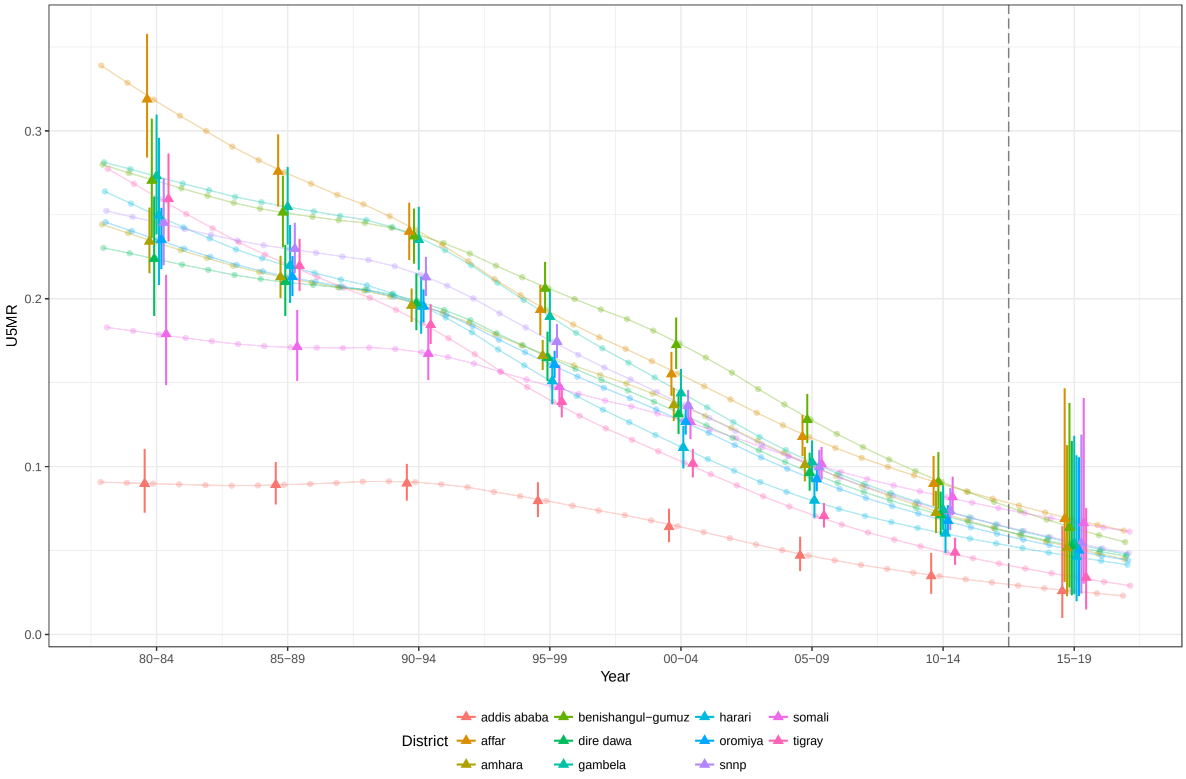

Figure S1.109: Ethiopia: Smoothed regional estimates over time. The line indicates yearly posterior median estimates and error bars indicate 95 % posterior credible interval at each time period.

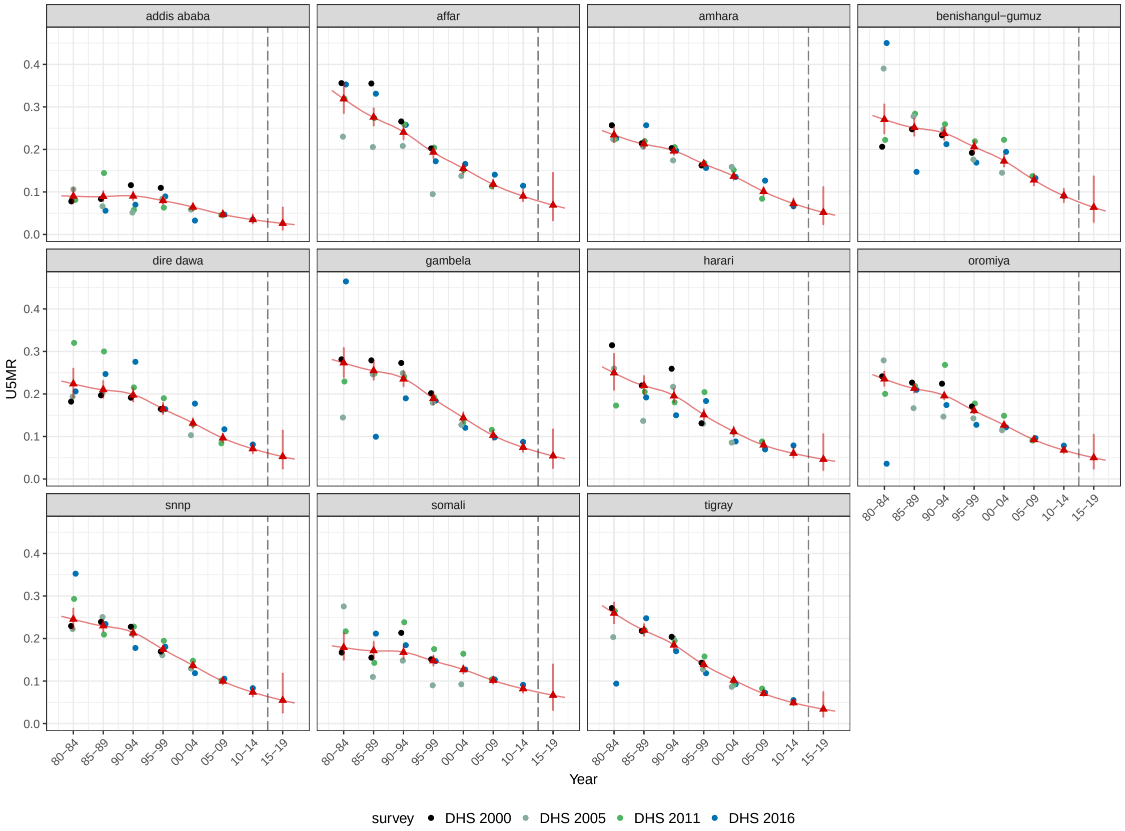

Figure S1.110: Ethiopia: Smoothed regional estimates over time compared to the direct estimates from each surveys. Direct estimates are not benchmarked with UN estimates. The line indicates posterior median and error bars indicate 95% posterior credible interval.

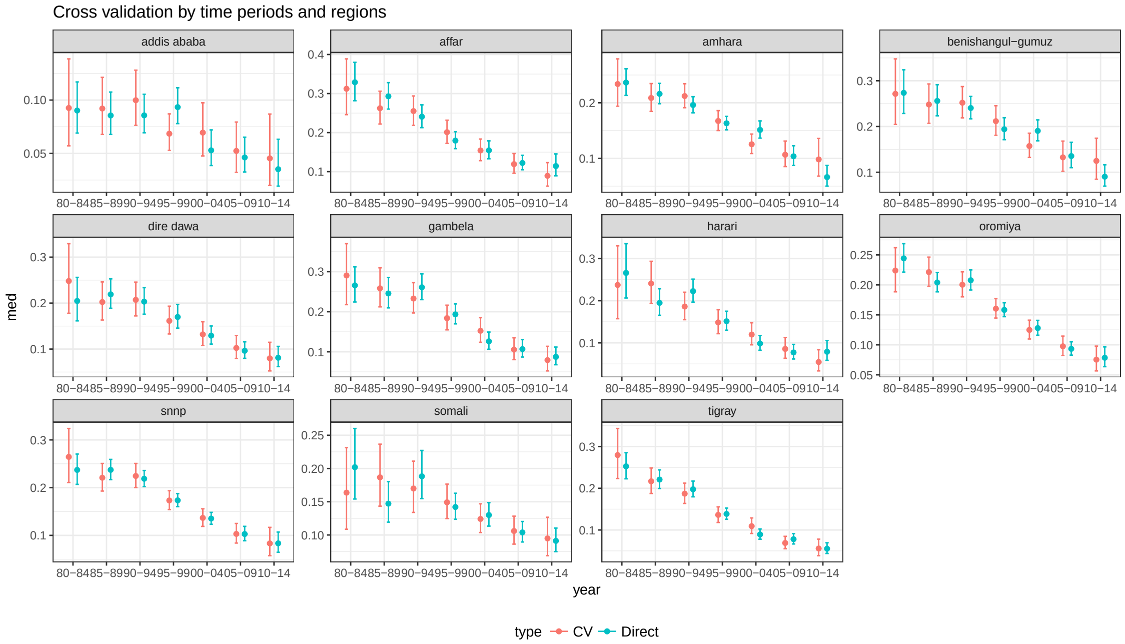

Figure S1.111: Ethiopia: Out-of-sample predictions along with direct estimates in the cross validation study where data from one region in each time period is held out and predicted using the rest of the data.

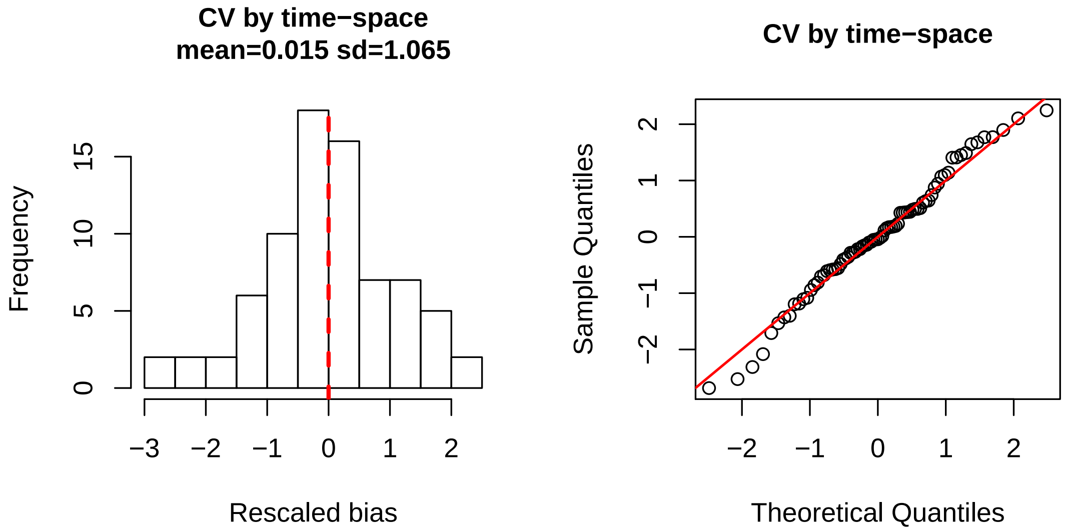

Figure S1.112: Ethiopia: Histogram and QQ-plot of the rescaled difference between the smoothed estimates and the direct estimates in the cross validation study. The differences between the two estimates are rescaled by the square root of the total variance of the two estimates.

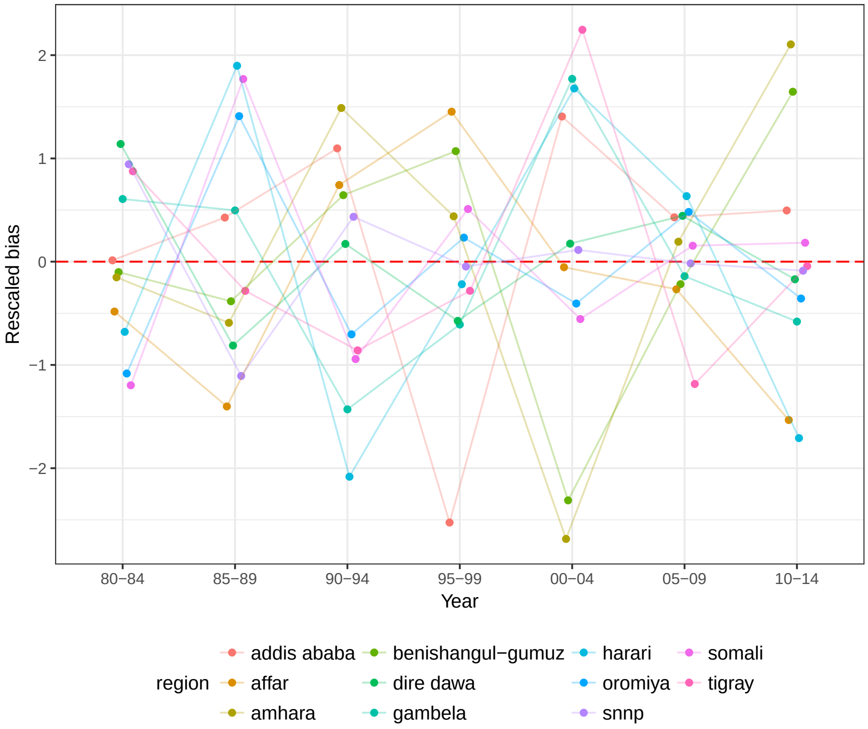

Figure S1.113: Ethiopia: Line plot of the difference between smoothed estimates and the direct estimates in the cross validation study. The differences between the two estimates are rescaled by the square root of the total variance of the two estimates.

3.5.13 Gabon

DHS surveys were conducted in Gabon in 2000, and 2012.

We fit both the RW2 only model to the combined national data, and compare the time trend at national level with the estimates produced by the UN and IHME in Figure S1.114. We then adjusted the combined national data to the UN estimates of U5MR, and refit the models on the benchmarked data.

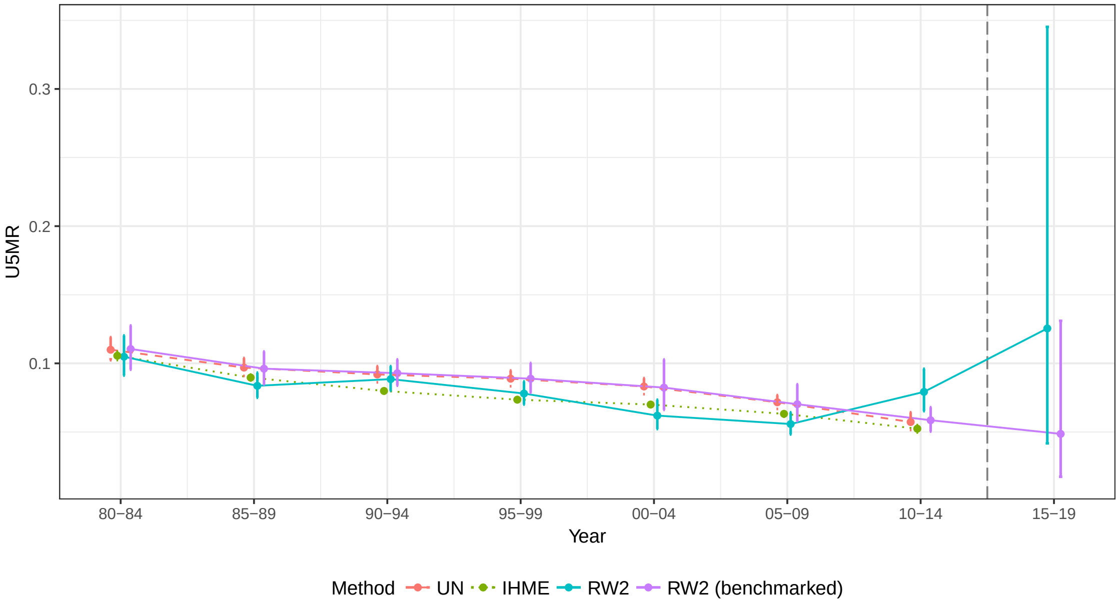

Figure S1.114: Gabon: Temporal national trends along with UN (B3) estimates described in You et al. (2015) and IHME estimates based on GBD 2015 Child Mortality Collaborators (2016). RW2 represents the smoothed national estimates using the original data before benchmarking with UN estimates. RW2-adj represents the smoothed national estimates using the benchmarked data.

We fit the RW2 model to the benchmarked data in each area. We compare the results in Figure S1.115 to S1.119. Figure S1.115 compares the smoothed estimates against the direct estimates. Figure S1.116 and Figure S1.117 show the posterior median estimates of U5MR in each region over time and the reductions from 1990 period respectively. Figure S1.118 shows the smoothed estimates by region over time and Figure S1.119 compares the smoothed estimates with direct estimates from each survey for each region over time.

We further assess the RW2 model by holding out some observations, and compare the projections to the direct estimates in these holdout observations. Figure S1.120 compares the predicted estimates for the out-of-sample observations with the direct estimates by holding out observations from each area in each time period. Figure S1.121 compares the histogram of the bias rescaled by the total variance in the cross validation studies. Figure S1.122 compares the rescaled bias by region and time periods.

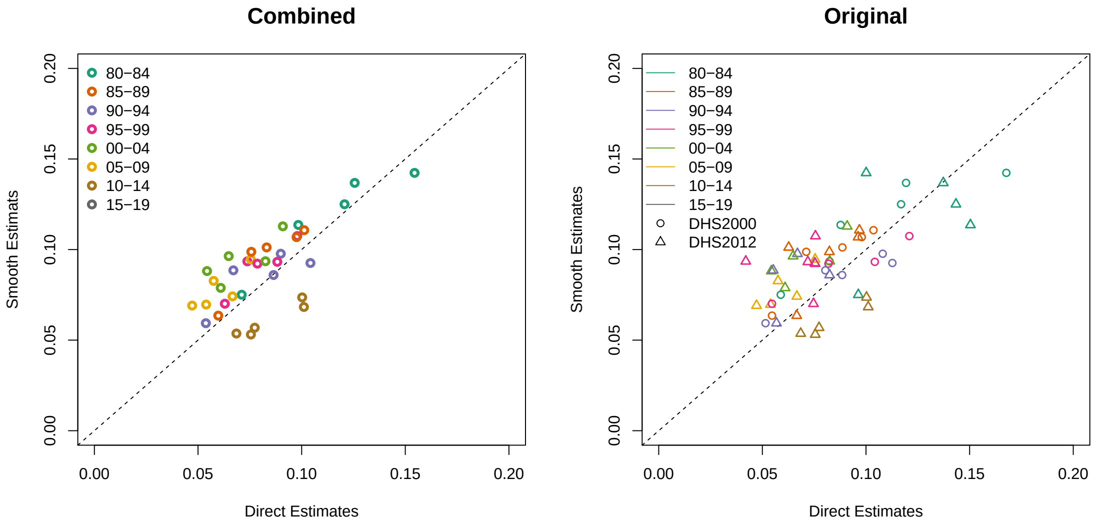

Figure S1.115: Gabon: Smooth versus direct Admin 1 estimates. Left: Combined (meta-analysis) survey estimate against combined direct estimates. Right: Combined (meta-analysis) survey estimate against direct estimates from each survey.

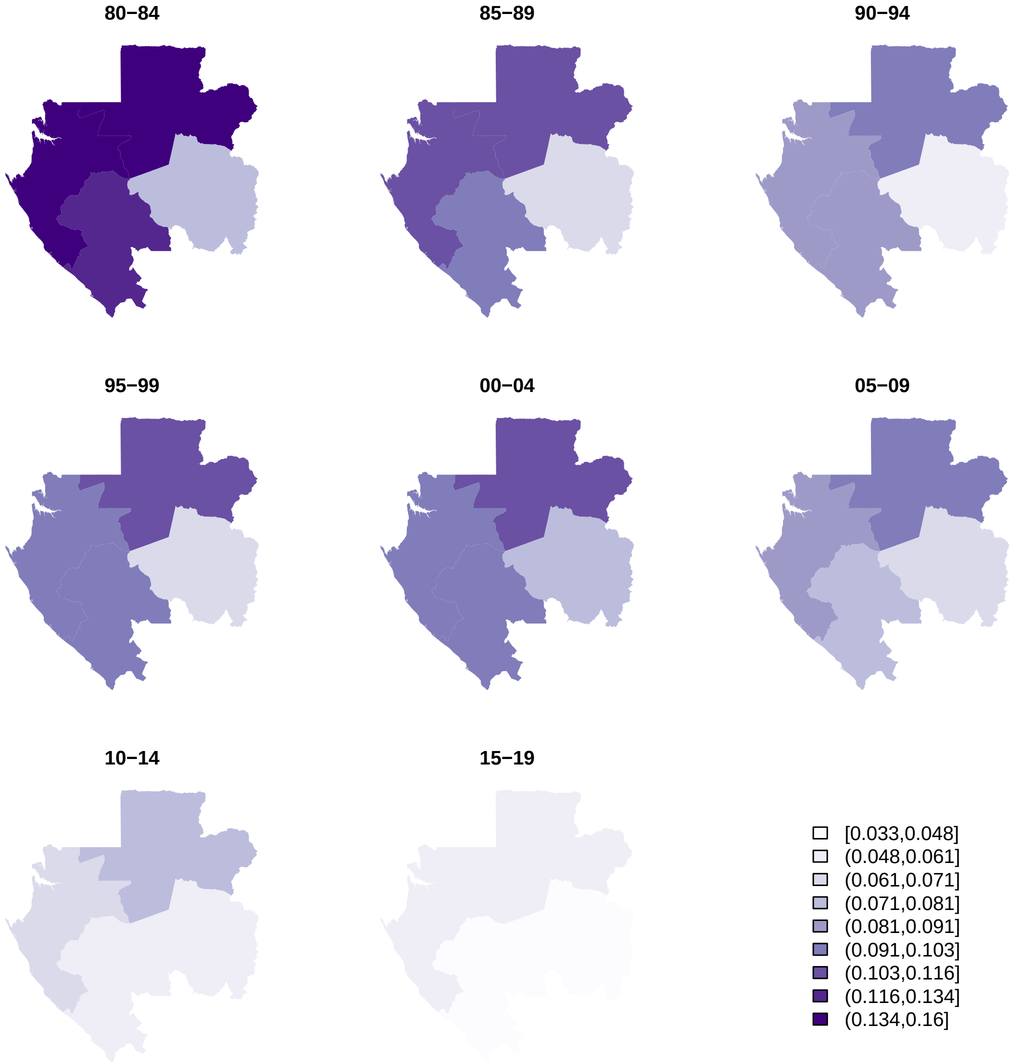

Figure S1.116: Gabon: Maps of posterior medians over time.

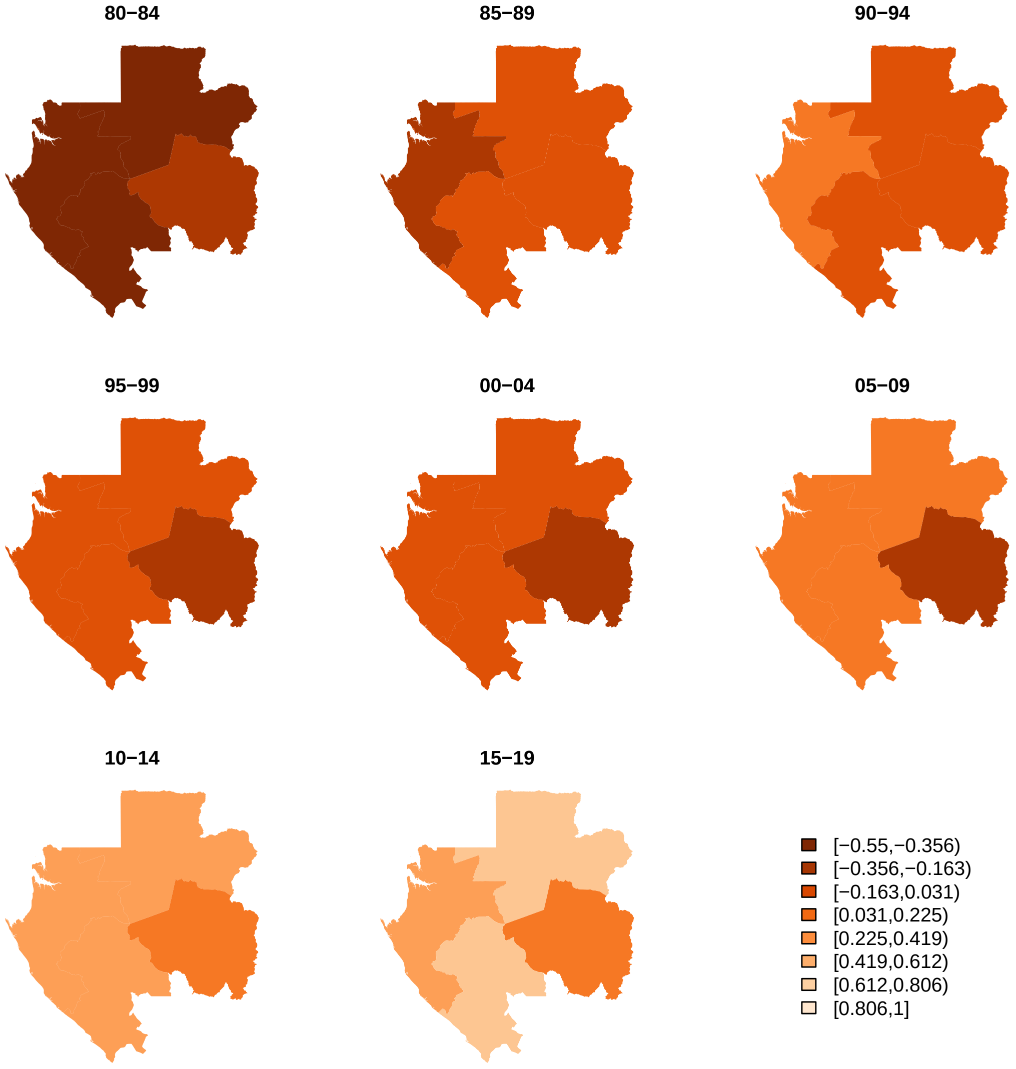

Figure S1.117: Gabon: Maps of reduction of posterior median U5MR in each five-year period compared to 1990 over time.

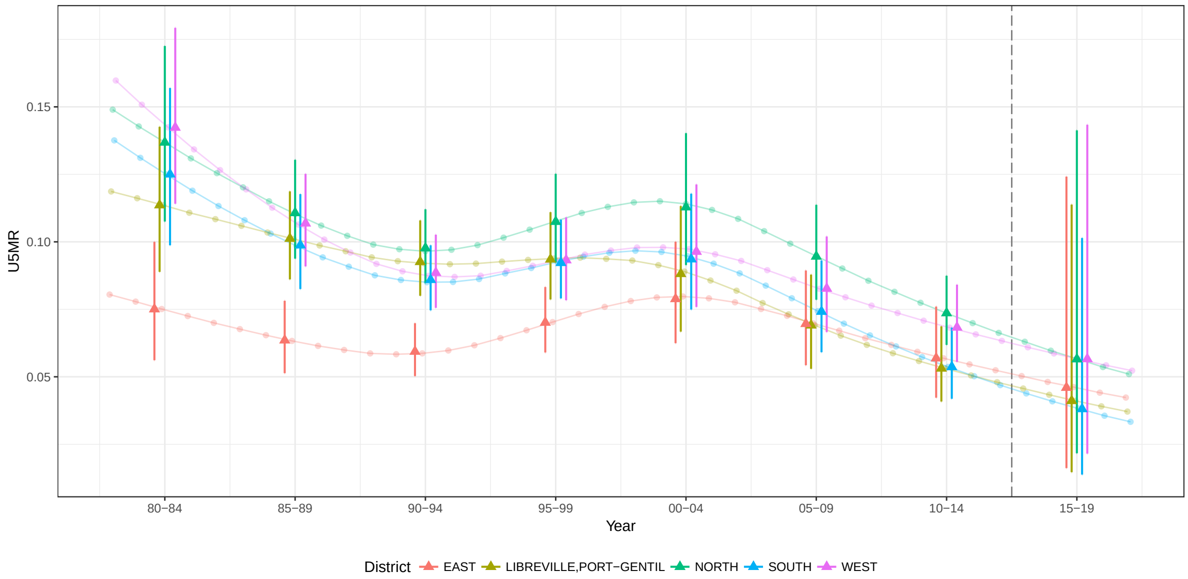

Figure S1.118: Gabon: Smoothed regional estimates over time. The line indicates yearly posterior median estimates and error bars indicate 95 % posterior credible interval at each time period.

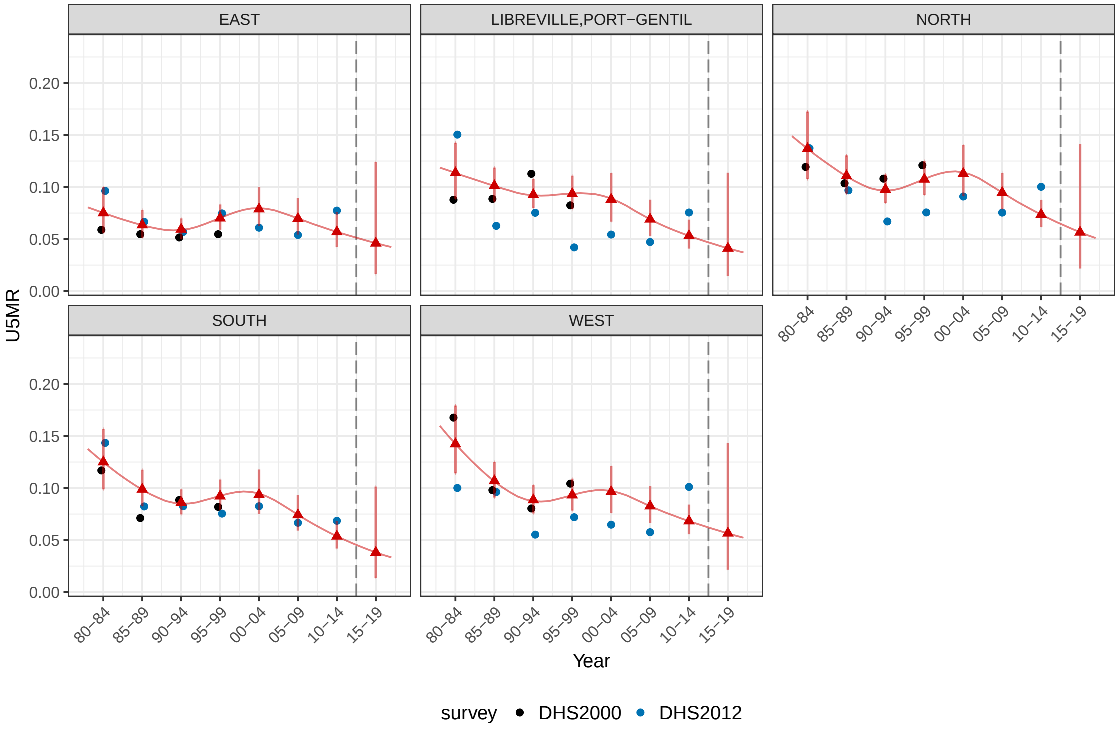

Figure S1.119: Gabon: Smoothed regional estimates over time compared to the direct estimates from each surveys. Direct estimates are not benchmarked with UN estimates. The line indicates posterior median and error bars indicate 95% posterior credible interval.

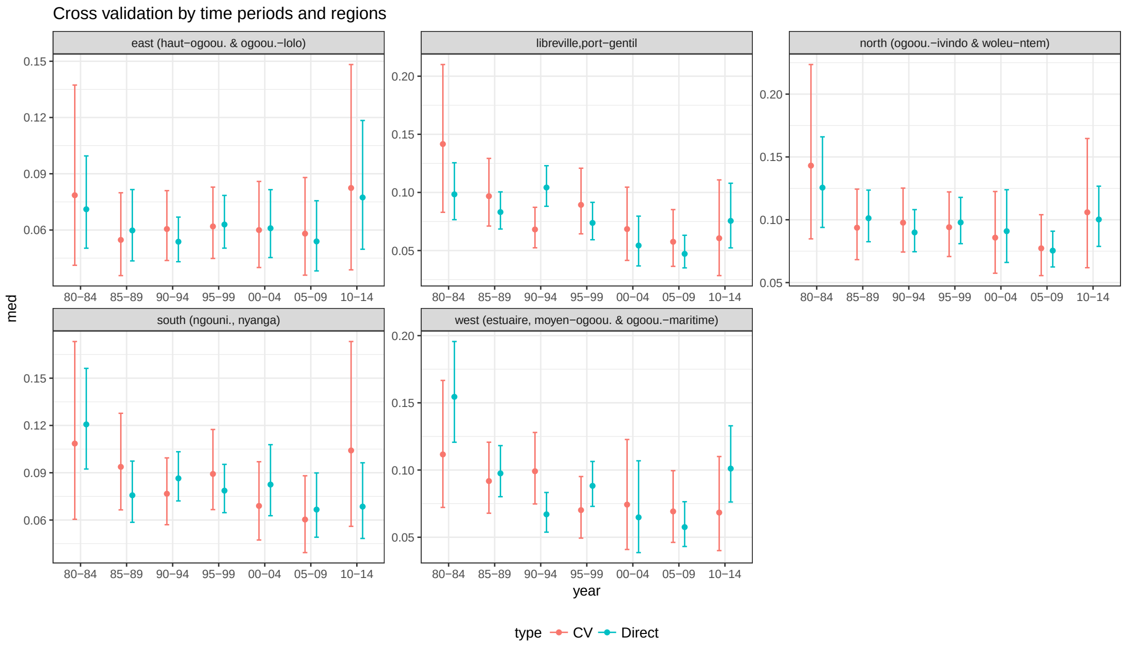

Figure S1.120: Gabon: Out-of-sample predictions along with direct estimates in the cross validation study where data from one region in each time period is held out and predicted using the rest of the data.

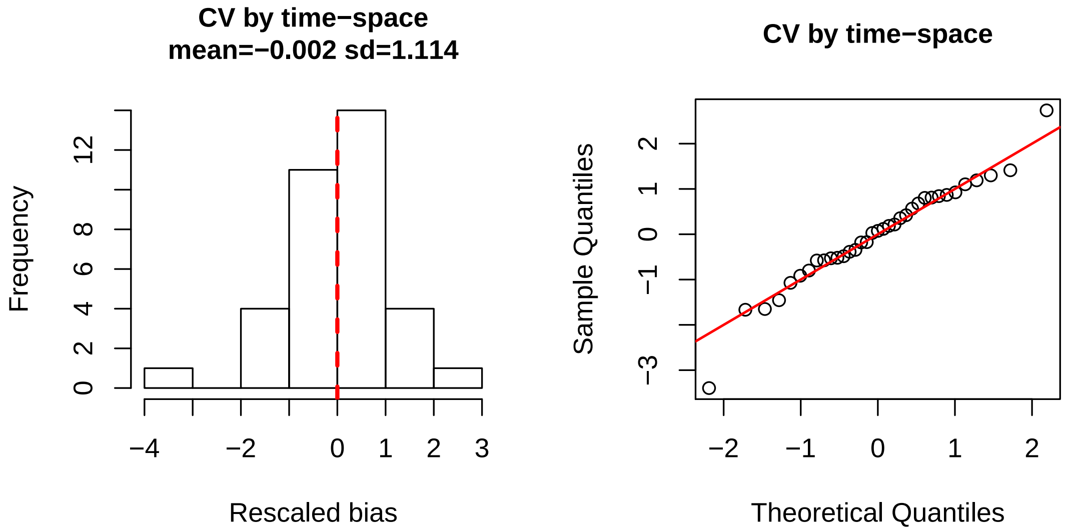

Figure S1.121: Gabon: Histogram and QQ-plot of the rescaled difference between the smoothed estimates and the direct estimates in the cross validation study. The differences between the two estimates are rescaled by the square root of the total variance of the two estimates.

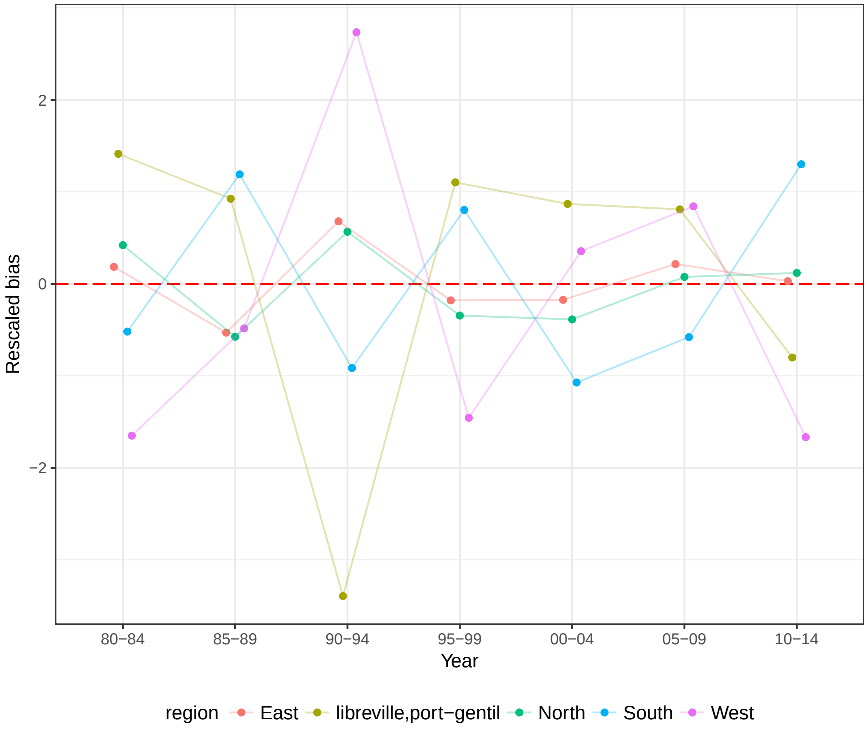

Figure S1.122: Gabon: Line plot of the difference between smoothed estimates and the direct estimates in the cross validation study. The differences between the two estimates are rescaled by the square root of the total variance of the two estimates.

3.5.14 Gambia

DHS surveys were conducted in Gambia in 2013.

We fit both the RW2 only model to the combined national data, and compare the time trend at national level with the estimates produced by the UN and IHME in Figure S1.123. We then adjusted the combined national data to the UN estimates of U5MR, and refit the models on the benchmarked data.

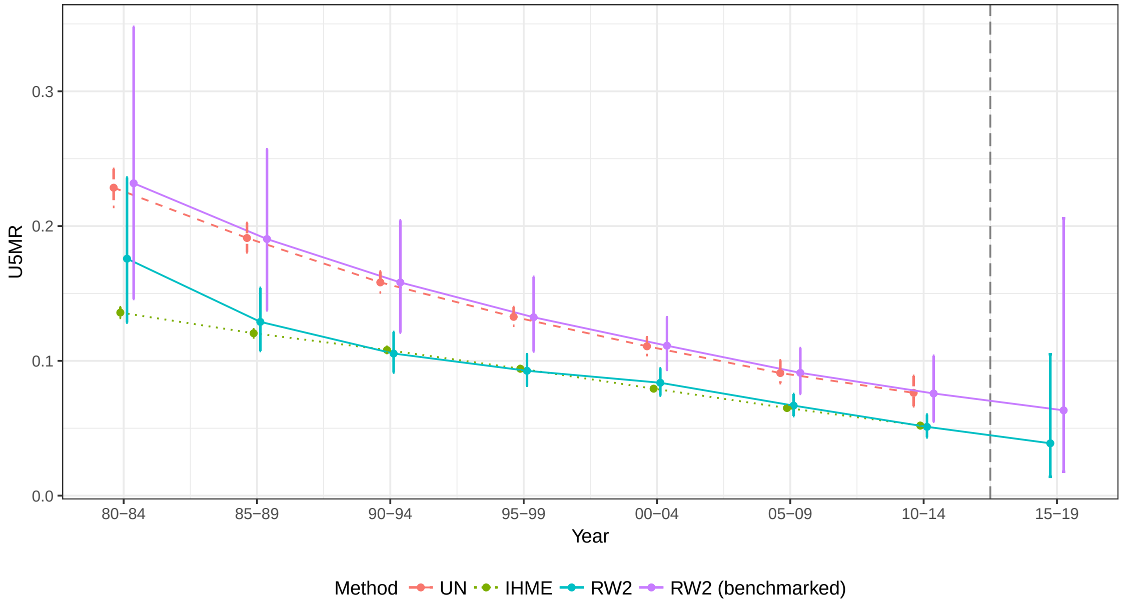

Figure S1.123: Gambia: Temporal national trends along with UN (B3) estimates described in You et al. (2015) and IHME estimates based on GBD 2015 Child Mortality Collaborators (2016). RW2 represents the smoothed national estimates using the original data before benchmarking with UN estimates. RW2-adj represents the smoothed national estimates using the benchmarked data.

We fit the RW2 model to the benchmarked data in each area. We compare the results in Figure S1.124 to S1.128. Figure S1.124 compares the smoothed estimates against the direct estimates. Figure S1.125 and Figure S1.126 show the posterior median estimates of U5MR in each region over time and the reductions from 1990 period respectively. Figure S1.127 shows the smoothed estimates by region over time and Figure S1.128 compares the smoothed estimates with direct estimates from each survey for each region over time.

We further assess the RW2 model by holding out some observations, and compare the projections to the direct estimates in these holdout observations. Figure S1.129 compares the predicted estimates for the out-of-sample observations with the direct estimates by holding out observations from each area in each time period. Figure S1.130 compares the histogram of the bias rescaled by the total variance in the cross validation studies. Figure S1.131 compares the rescaled bias by region and time periods.

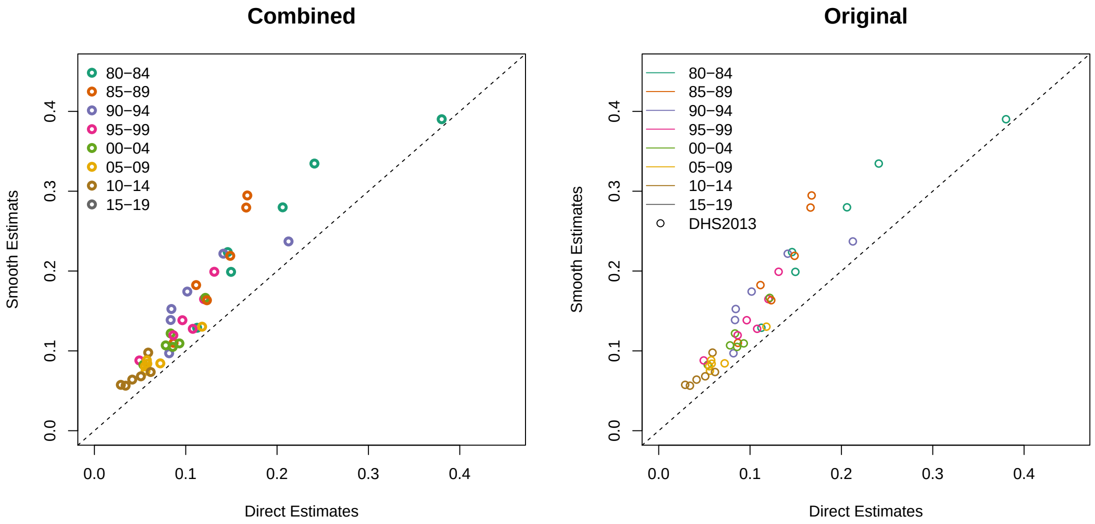

Figure S1.124: Gambia: Smooth versus direct Admin 1 estimates. Left: Combined (meta-analysis) survey estimate against combined direct estimates. Right: Combined (meta-analysis) survey estimate against direct estimates from each survey.

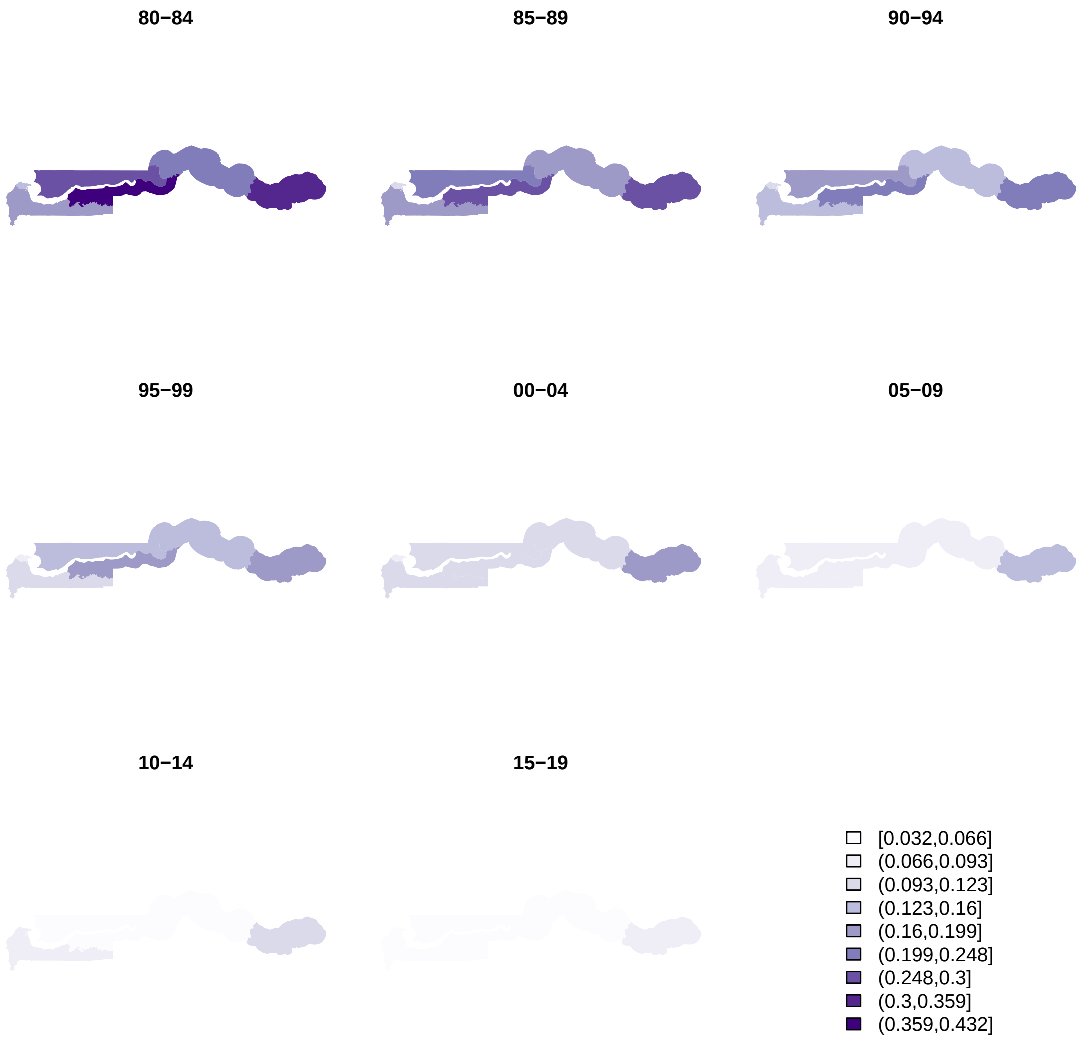

Figure S1.125: Gambia: Maps of posterior medians over time.

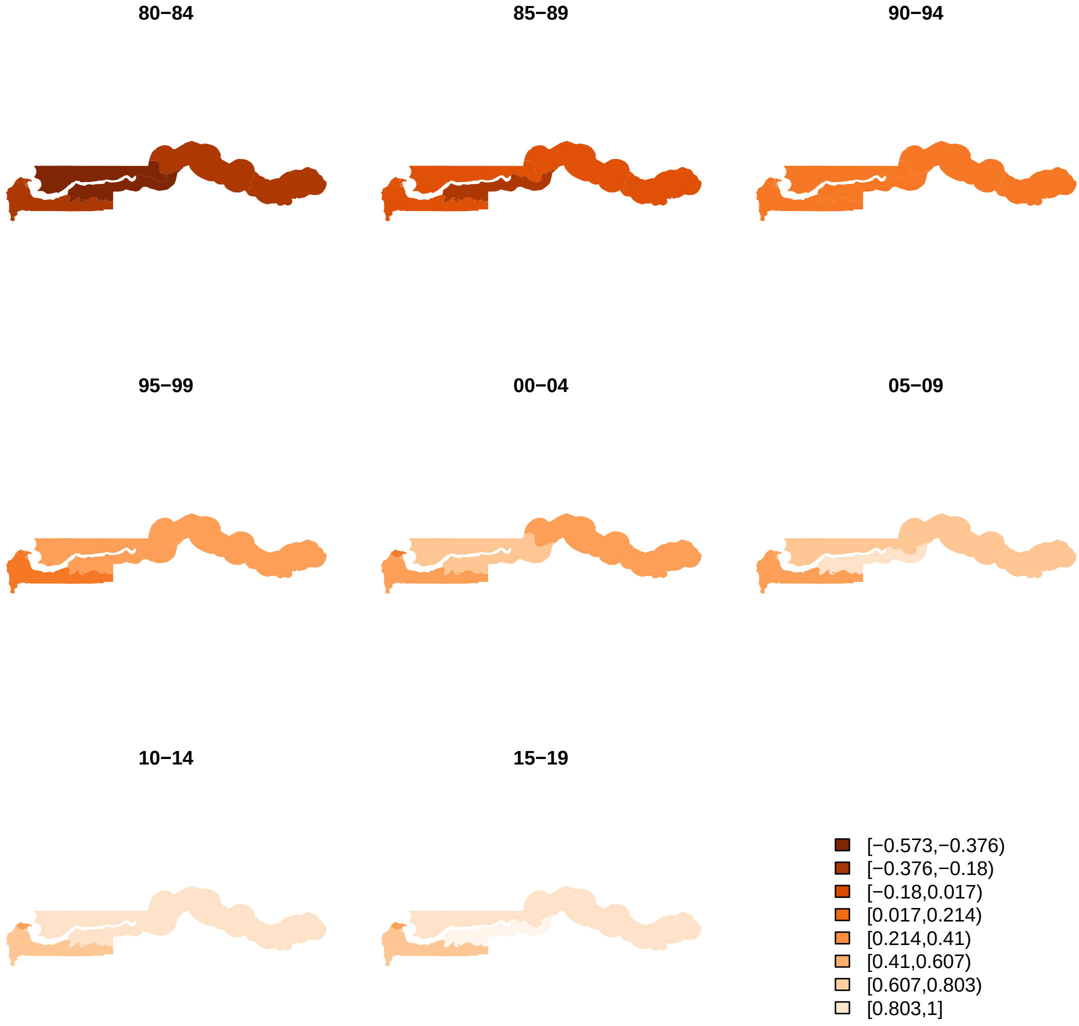

Figure S1.126: Gambia: Maps of reduction of posterior median U5MR in each five-year period compared to 1990 over time.

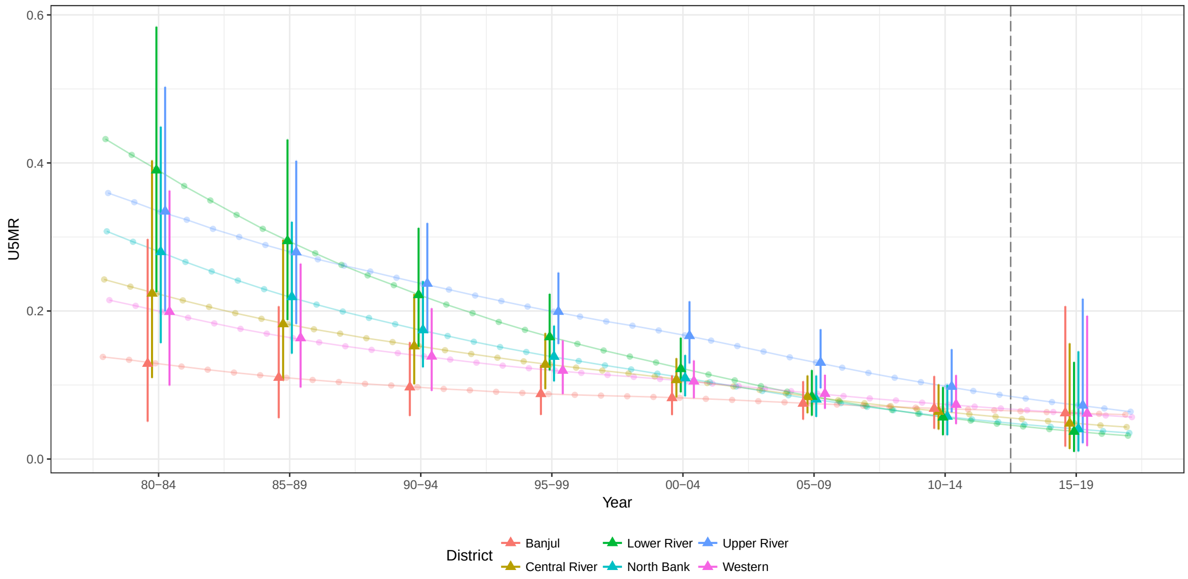

Figure S1.127: Gambia: Smoothed regional estimates over time. The line indicates yearly posterior median estimates and error bars indicate 95 % posterior credible interval at each time period.

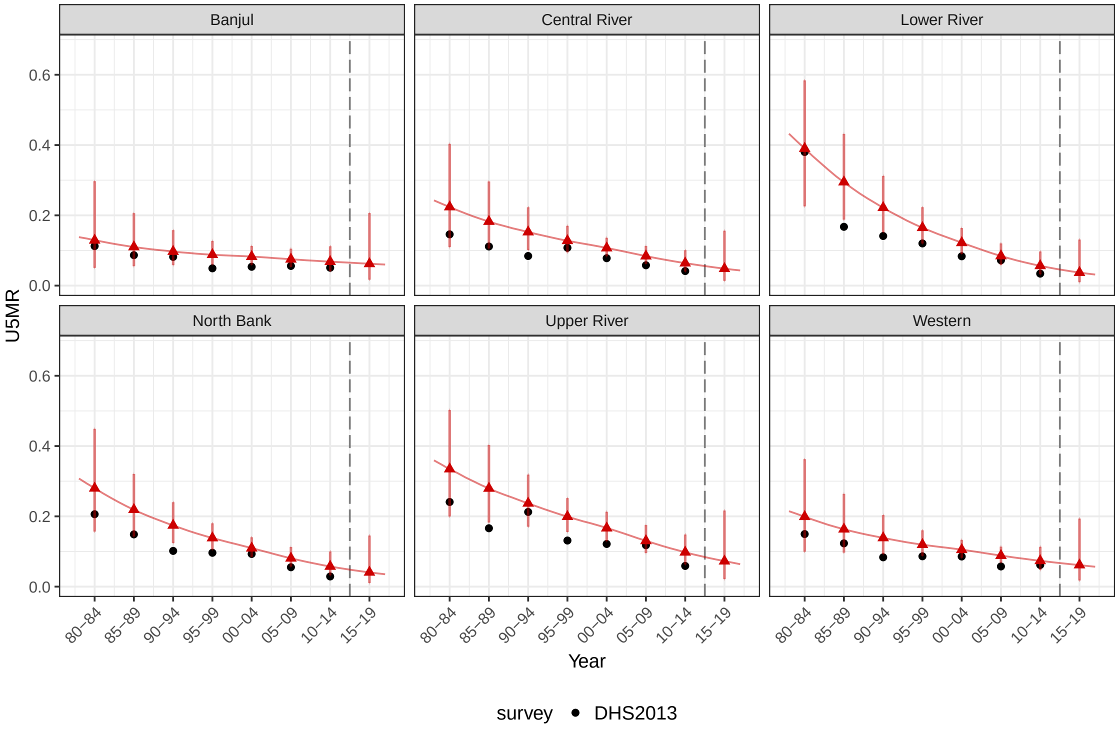

Figure S1.128: Gambia: Smoothed regional estimates over time compared to the direct estimates from each surveys. Direct estimates are not benchmarked with UN estimates. The line indicates posterior median and error bars indicate 95% posterior credible interval.

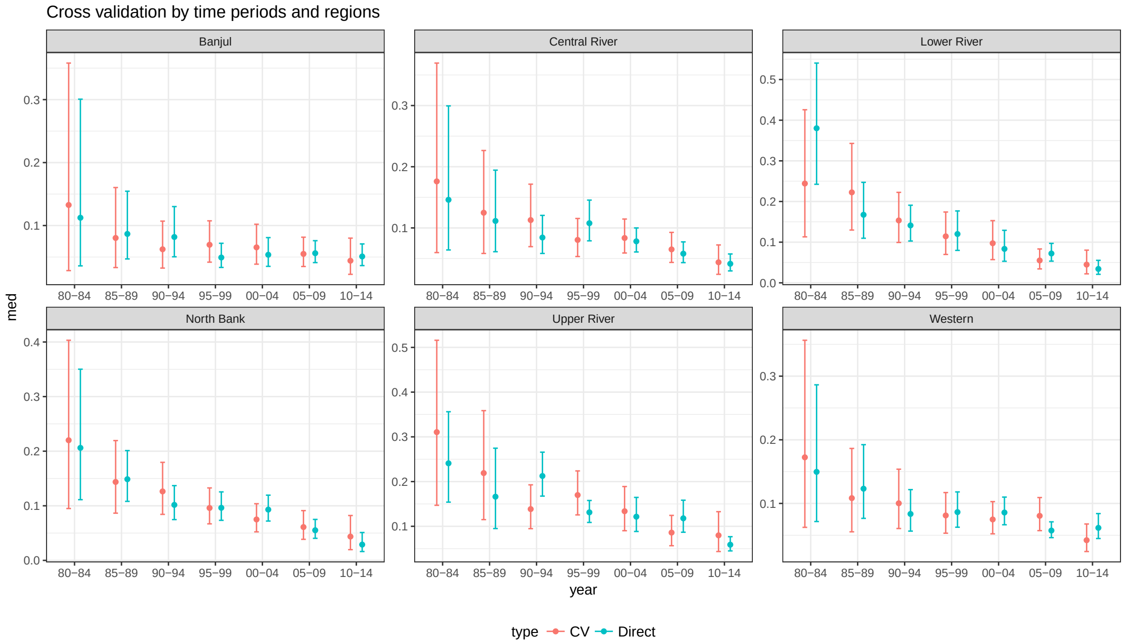

Figure S1.129: Gambia: Out-of-sample predictions along with direct estimates in the cross validation study where data from one region in each time period is held out and predicted using the rest of the data.

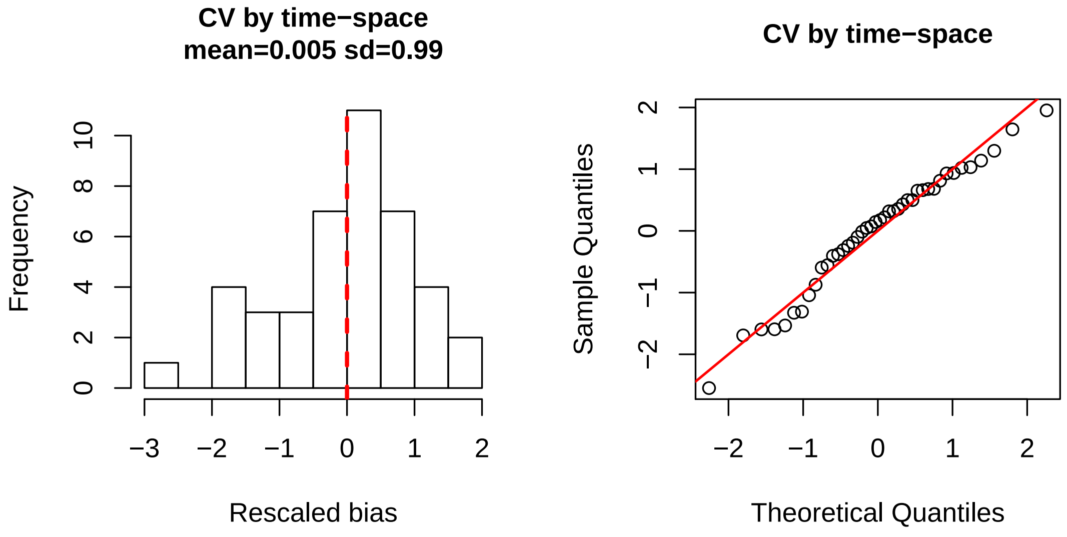

Figure S1.130: Gambia: Histogram and QQ-plot of the rescaled difference between the smoothed estimates and the direct estimates in the cross validation study. The differences between the two estimates are rescaled by the square root of the total variance of the two estimates.

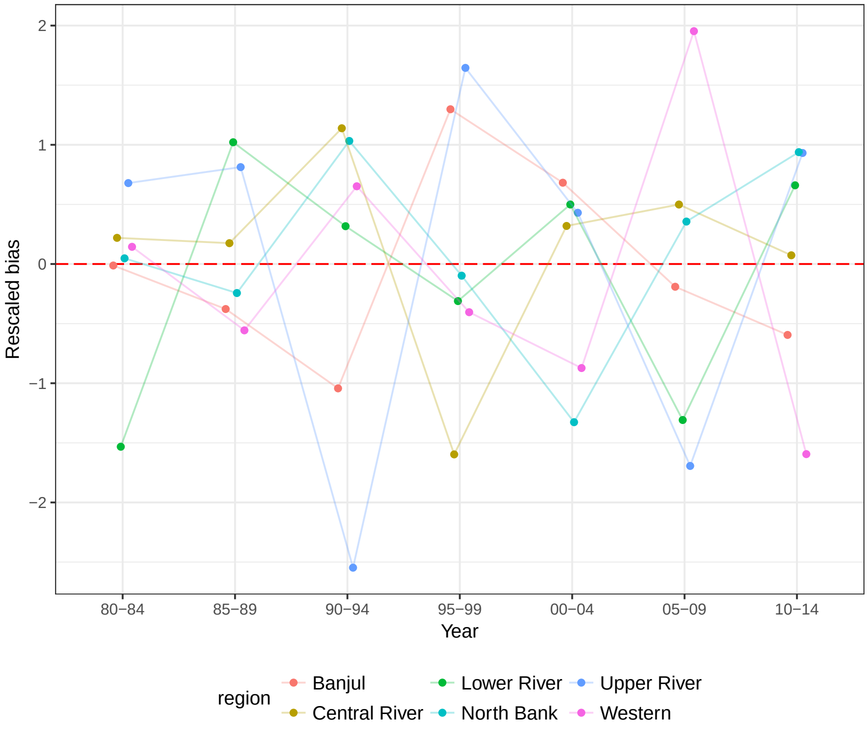

Figure S1.131: Gambia: Line plot of the difference between smoothed estimates and the direct estimates in the cross validation study. The differences between the two estimates are rescaled by the square root of the total variance of the two estimates.

3.5.15 Ghana

DHS surveys were conducted in Ghana in 1989, 1993, 1998, 2003, 2008, and 2014.

We fit both the RW2 only model to the combined national data, and compare the time trend at national level with the estimates produced by the UN and IHME in Figure S1.132. We then adjusted the combined national data to the UN estimates of U5MR, and refit the models on the benchmarked data.

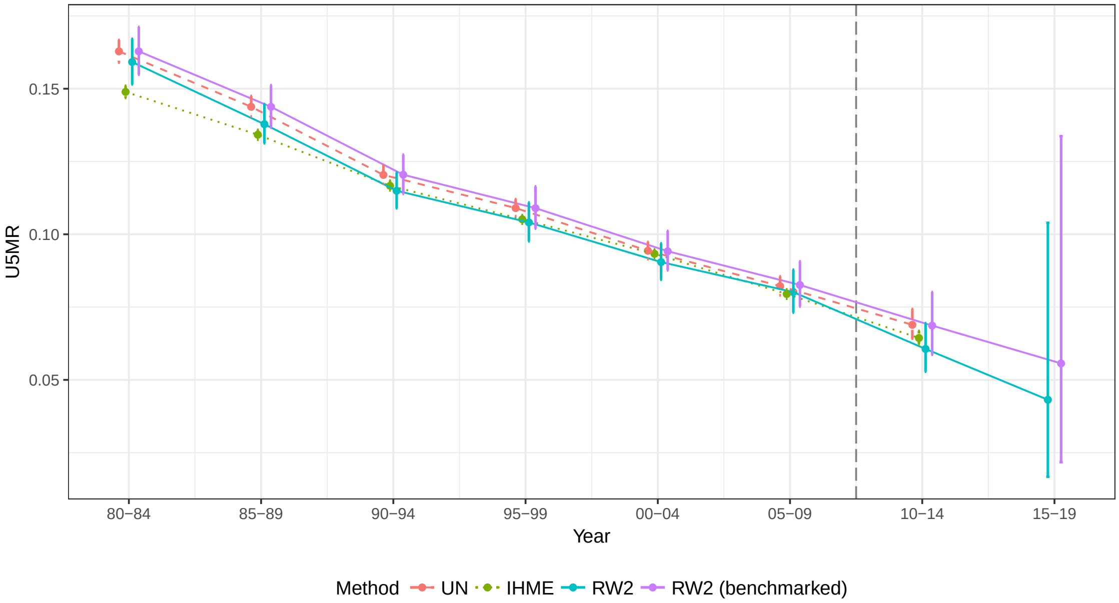

Figure S1.132: Ghana: Temporal national trends along with UN (B3) estimates described in You et al. (2015) and IHME estimates based on GBD 2015 Child Mortality Collaborators (2016). RW2 represents the smoothed national estimates using the original data before benchmarking with UN estimates. RW2-adj represents the smoothed national estimates using the benchmarked data.

We fit the RW2 model to the benchmarked data in each area. We compare the results in Figure S1.133 to S1.137. Figure S1.133 compares the smoothed estimates against the direct estimates. Figure S1.134 and Figure S1.135 show the posterior median estimates of U5MR in each region over time and the reductions from 1990 period respectively. Figure S1.136 shows the smoothed estimates by region over time and Figure S1.137 compares the smoothed estimates with direct estimates from each survey for each region over time.

We further assess the RW2 model by holding out some observations, and compare the projections to the direct estimates in these holdout observations. Figure S1.138 compares the predicted estimates for the out-of-sample observations with the direct estimates by holding out observations from each area in each time period. Figure S1.139 compares the histogram of the bias rescaled by the total variance in the cross validation studies. Figure S1.140 compares the rescaled bias by region and time periods.

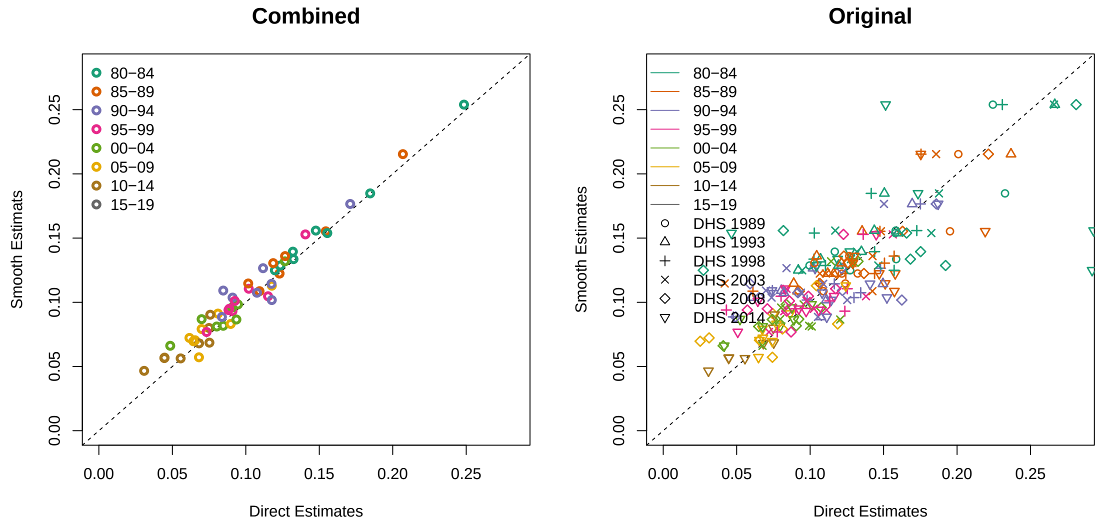

Figure S1.133: Ghana: Smooth versus direct Admin 1 estimates. Left: Combined (meta-analysis) survey estimate against combined direct estimates. Right: Combined (meta-analysis) survey estimate against direct estimates from each survey.

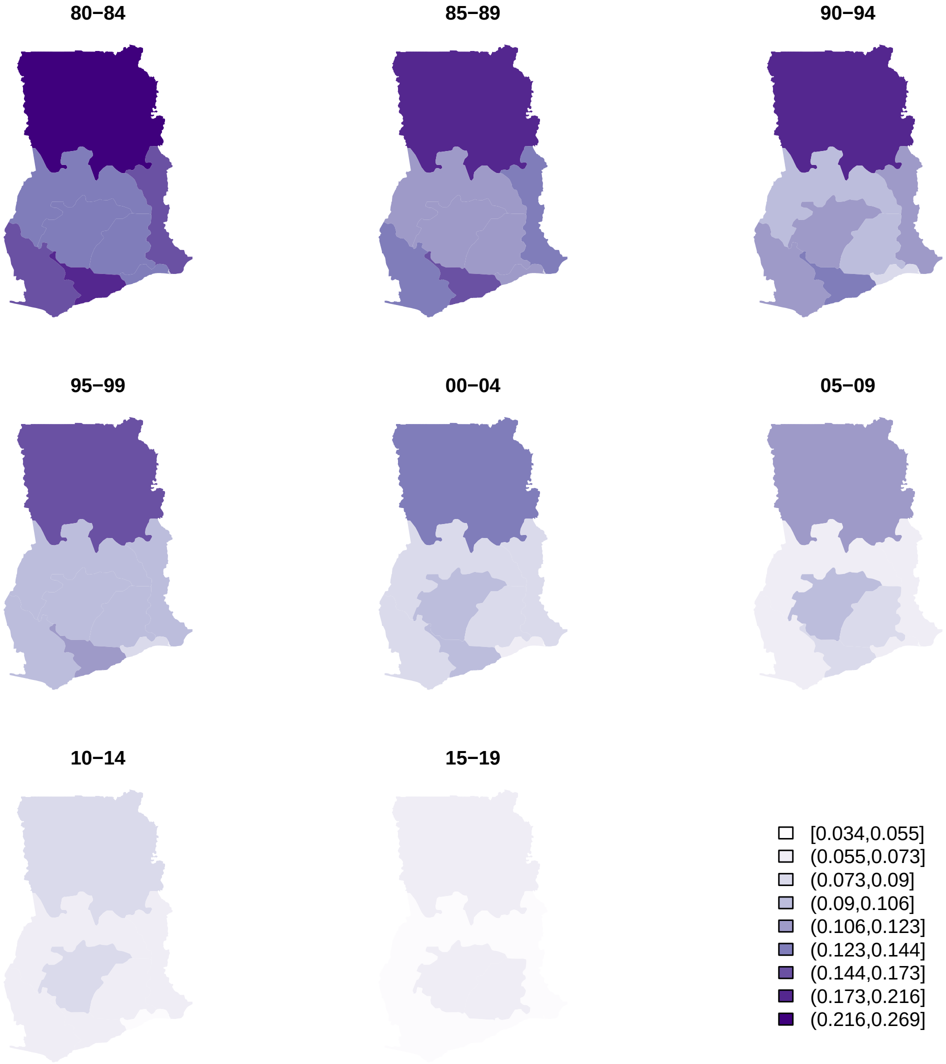

Figure S1.134: Ghana: Maps of posterior medians over time.

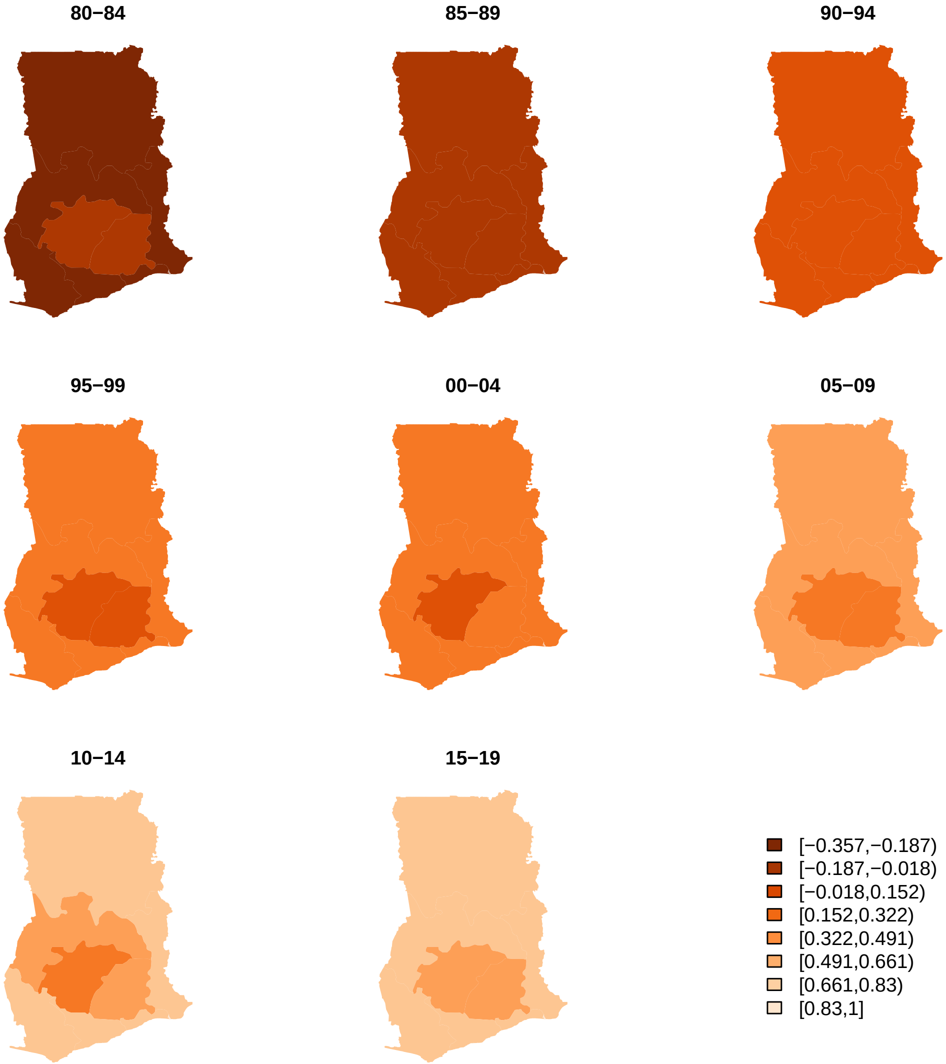

Figure S1.135: Ghana: Maps of reduction of posterior median U5MR in each five-year period compared to 1990 over time.

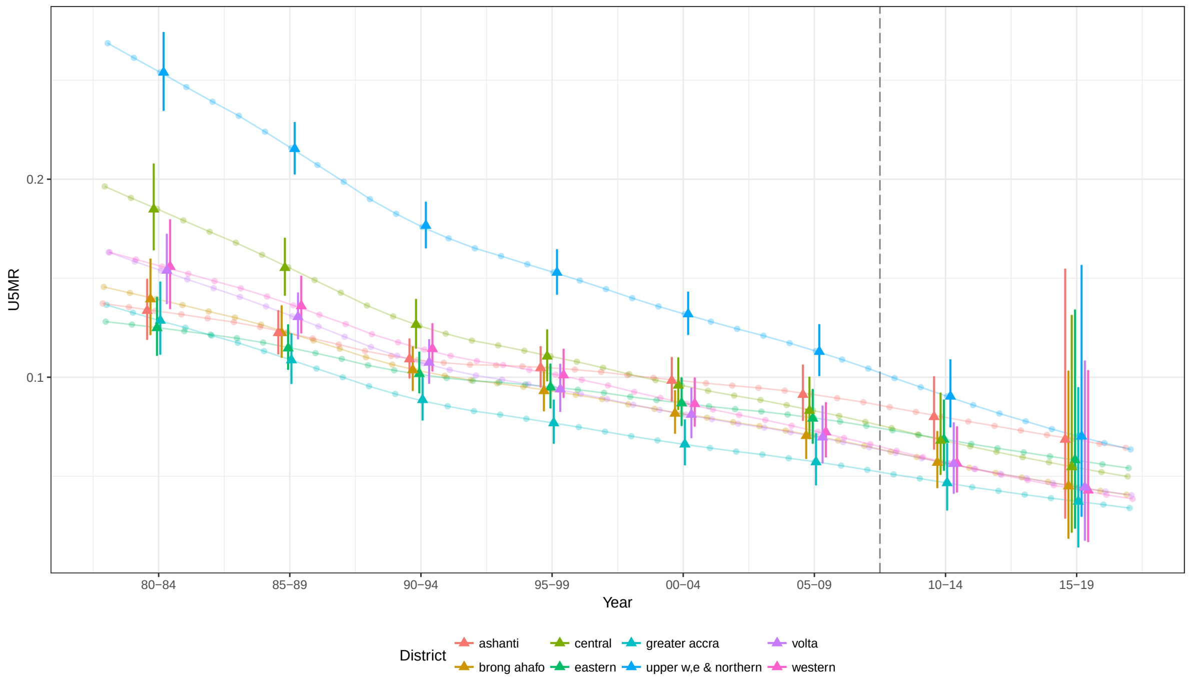

Figure S1.136: Ghana: Smoothed regional estimates over time. The line indicates yearly posterior median estimates and error bars indicate 95 % posterior credible interval at each time period.

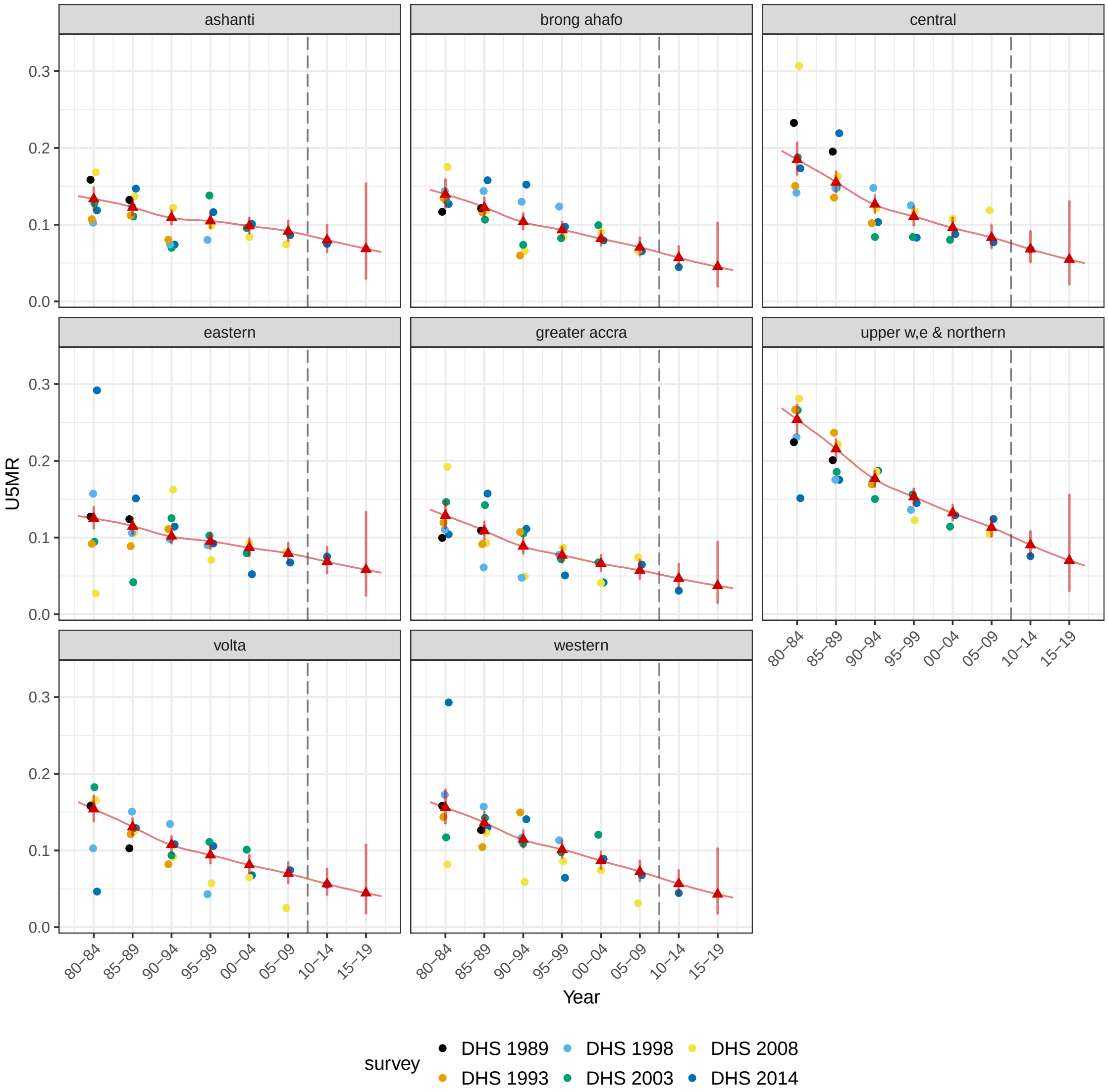

Figure S1.137: Ghana: Smoothed regional estimates over time compared to the direct estimates from each surveys. Direct estimates are not benchmarked with UN estimates. The line indicates posterior median and error bars indicate 95% posterior credible interval.

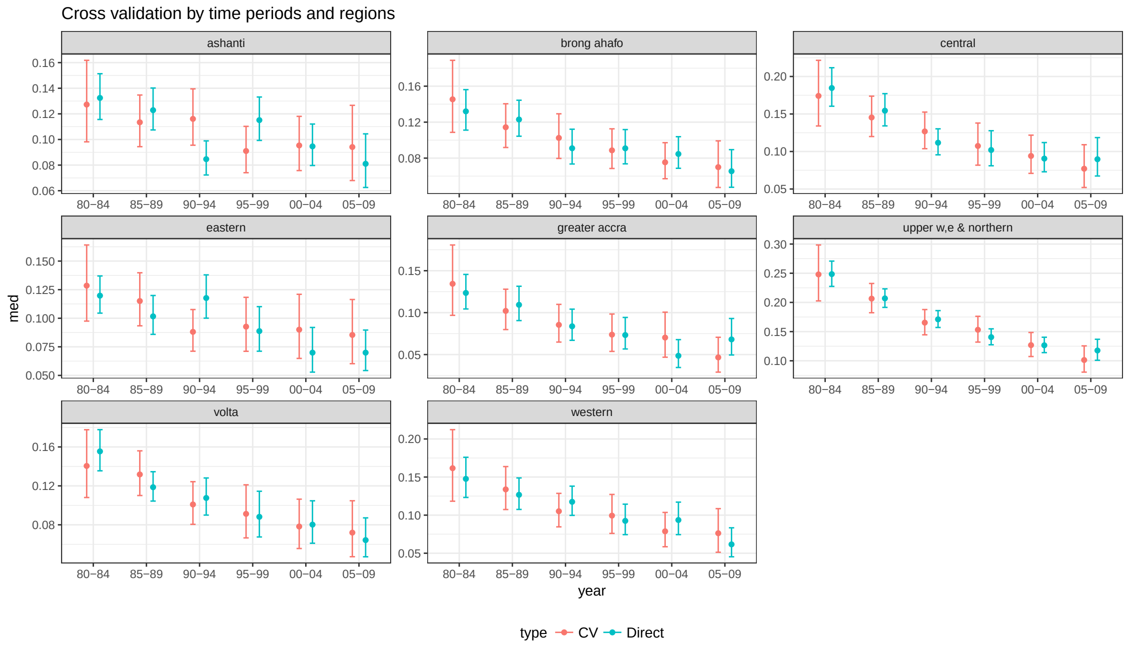

Figure S1.138: Ghana: Out-of-sample predictions along with direct estimates in the cross validation study where data from one region in each time period is held out and predicted using the rest of the data.

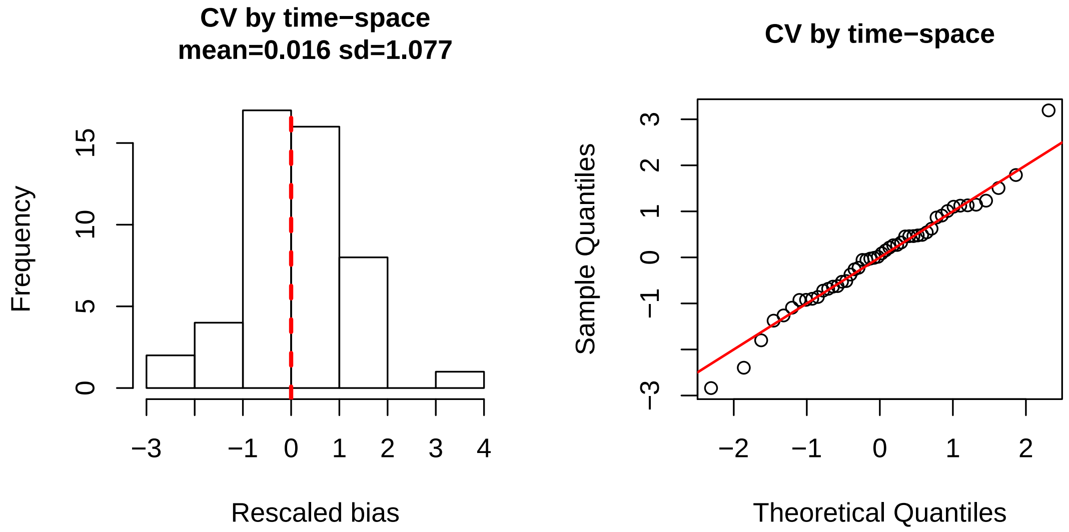

Figure S1.139: Ghana: Histogram and QQ-plot of the rescaled difference between the smoothed estimates and the direct estimates in the cross validation study. The differences between the two estimates are rescaled by the square root of the total variance of the two estimates.

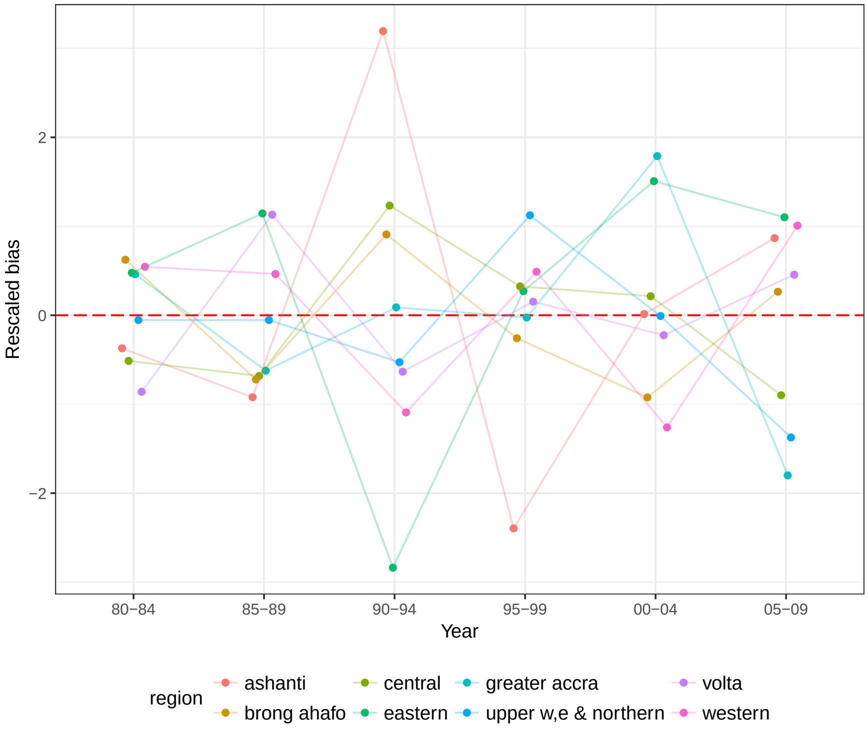

Figure S1.140: Ghana: Line plot of the difference between smoothed estimates and the direct estimates in the cross validation study. The differences between the two estimates are rescaled by the square root of the total variance of the two estimates.

3.5.16 Guinea

DHS surveys were conducted in Guinea in 1999, 2005, and 2012.

We fit both the RW2 only model to the combined national data, and compare the time trend at national level with the estimates produced by the UN and IHME in Figure S1.141. We then adjusted the combined national data to the UN estimates of U5MR, and refit the models on the benchmarked data.

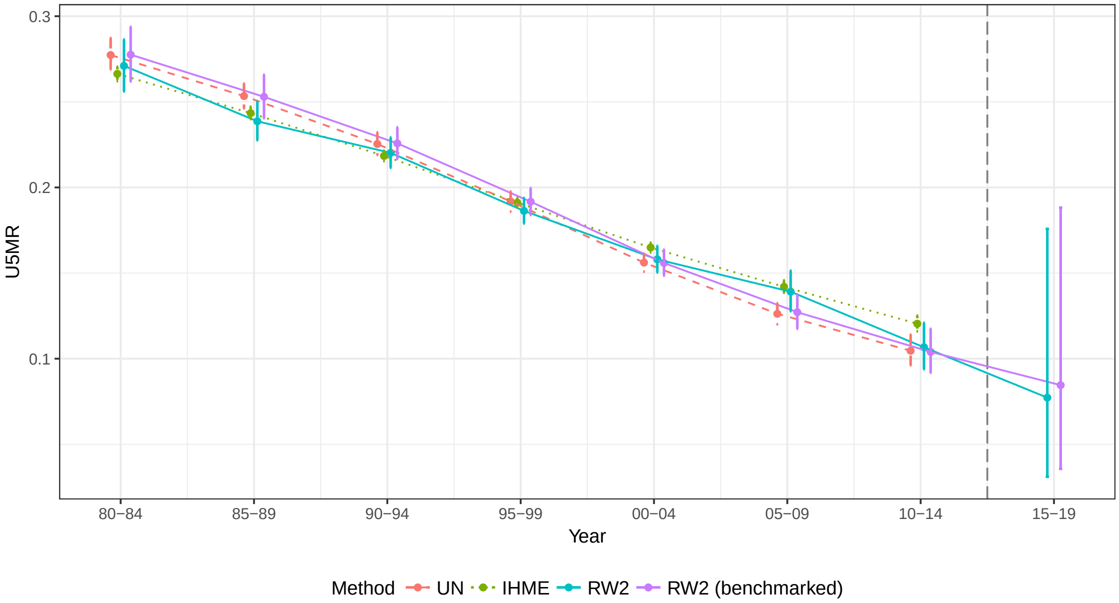

Figure S1.141: Guinea: Temporal national trends along with UN (B3) estimates described in You et al. (2015) and IHME estimates based on GBD 2015 Child Mortality Collaborators (2016). RW2 represents the smoothed national estimates using the original data before benchmarking with UN estimates. RW2-adj represents the smoothed national estimates using the benchmarked data.

We fit the RW2 model to the benchmarked data in each area. We compare the results in Figure S1.142 to S1.146. Figure S1.142 compares the smoothed estimates against the direct estimates. Figure S1.143 and Figure S1.144 show the posterior median estimates of U5MR in each region over time and the reductions from 1990 period respectively. Figure S1.145 shows the smoothed estimates by region over time and Figure S1.146 compares the smoothed estimates with direct estimates from each survey for each region over time.

We further assess the RW2 model by holding out some observations, and compare the projections to the direct estimates in these holdout observations. Figure S1.147 compares the predicted estimates for the out-of-sample observations with the direct estimates by holding out observations from each area in each time period. Figure S1.148 compares the histogram of the bias rescaled by the total variance in the cross validation studies. Figure S1.149 compares the rescaled bias by region and time periods.

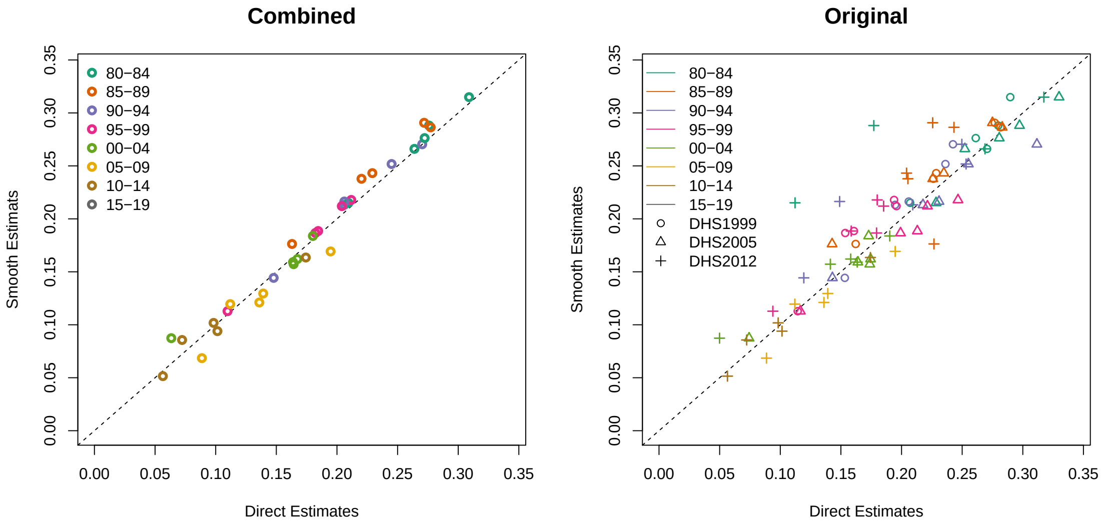

Figure S1.142: Guinea: Smooth versus direct Admin 1 estimates. Left: Combined (meta-analysis) survey estimate against combined direct estimates. Right: Combined (meta-analysis) survey estimate against direct estimates from each survey.

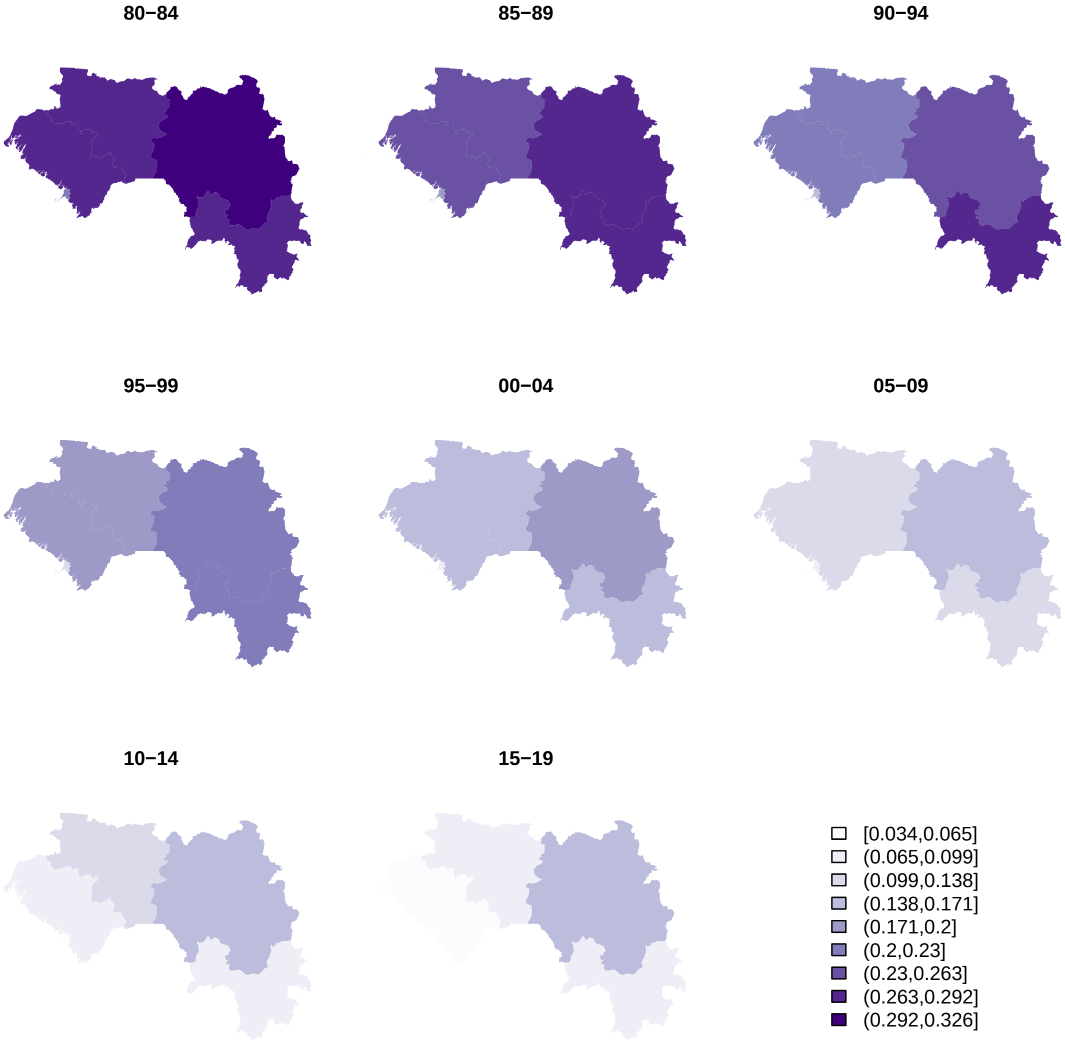

Figure S1.143: Guinea: Maps of posterior medians over time.

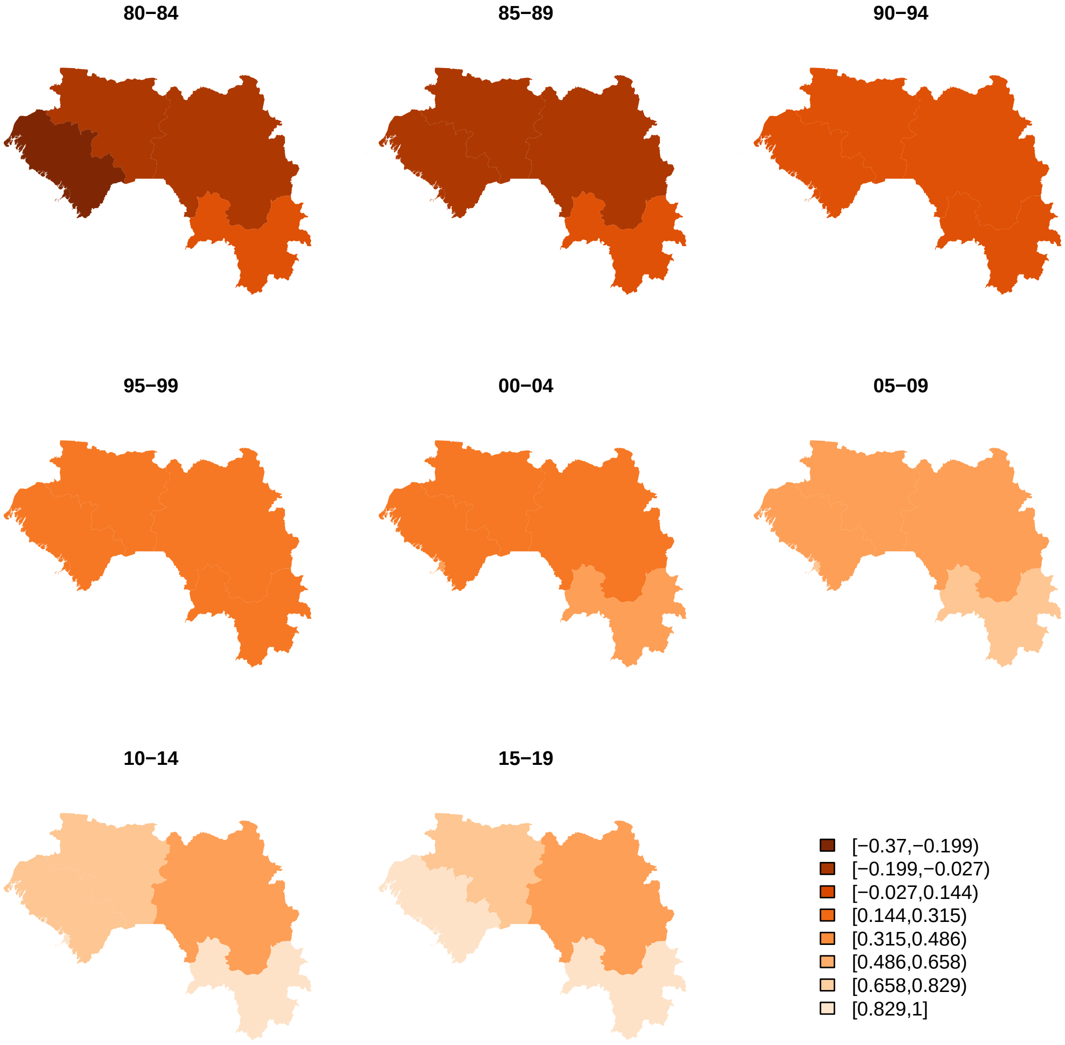

Figure S1.144: Guinea: Maps of reduction of posterior median U5MR in each five-year period compared to 1990 over time.

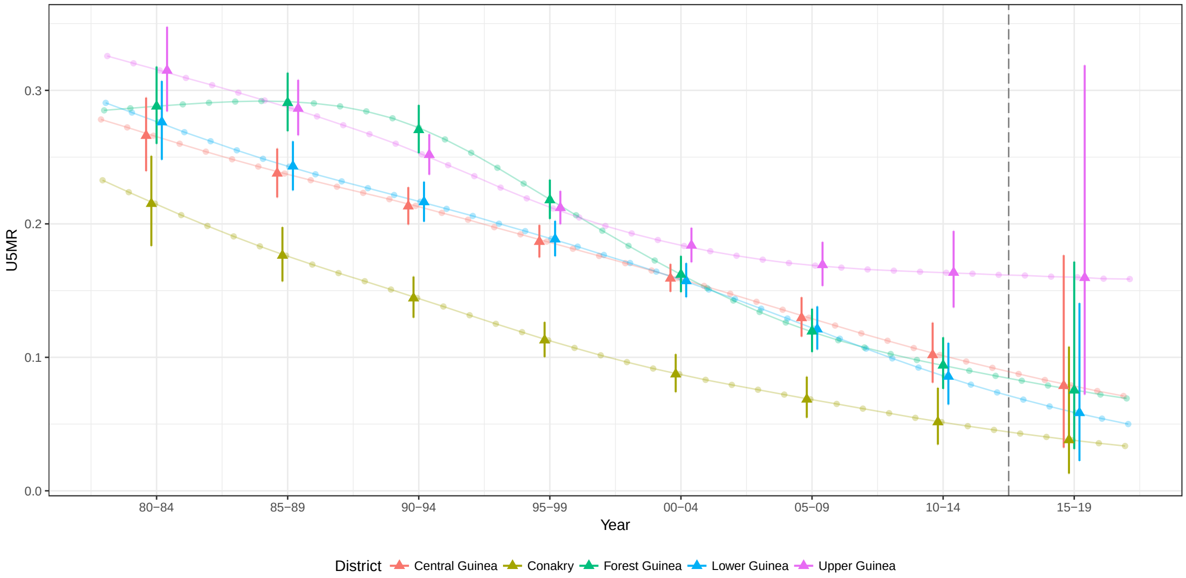

Figure S1.145: Guinea: Smoothed regional estimates over time. The line indicates yearly posterior median estimates and error bars indicate 95 % posterior credible interval at each time period.

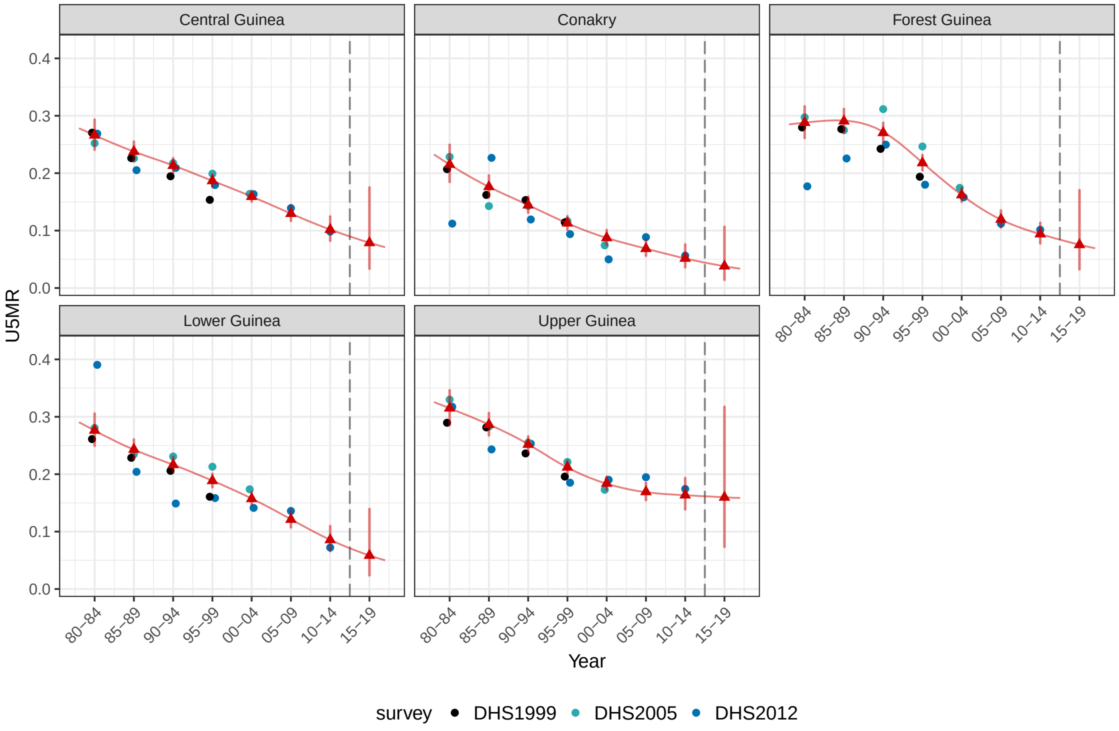

Figure S1.146: Guinea: Smoothed regional estimates over time compared to the direct estimates from each surveys. Direct estimates are not benchmarked with UN estimates. The line indicates posterior median and error bars indicate 95% posterior credible interval.

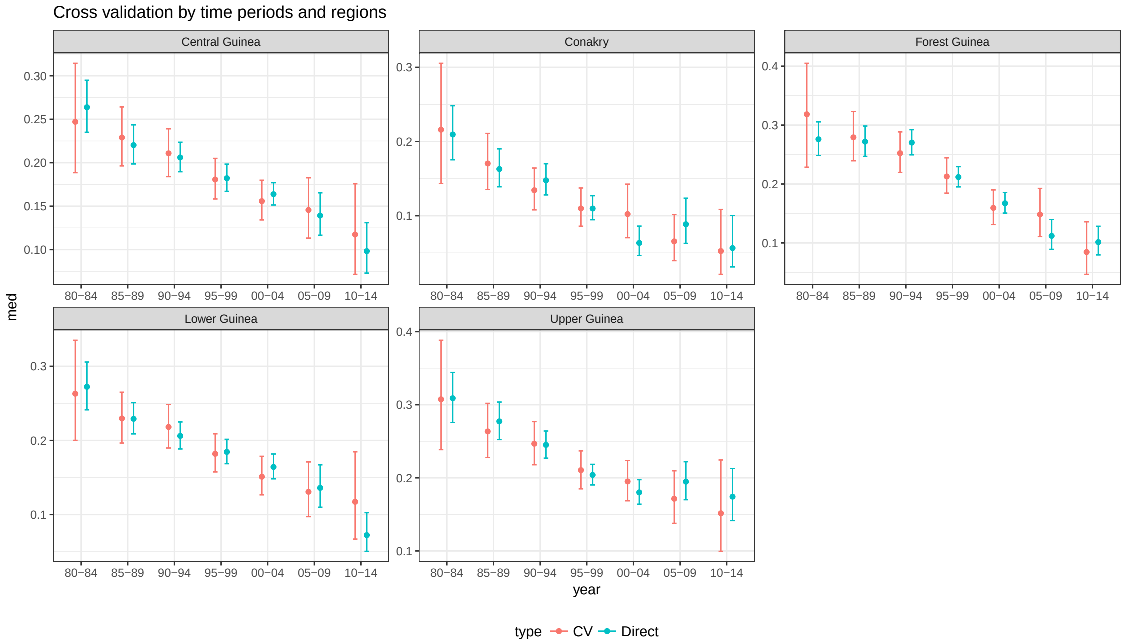

Figure S1.147: Guinea: Out-of-sample predictions along with direct estimates in the cross validation study where data from one region in each time period is held out and predicted using the rest of the data.

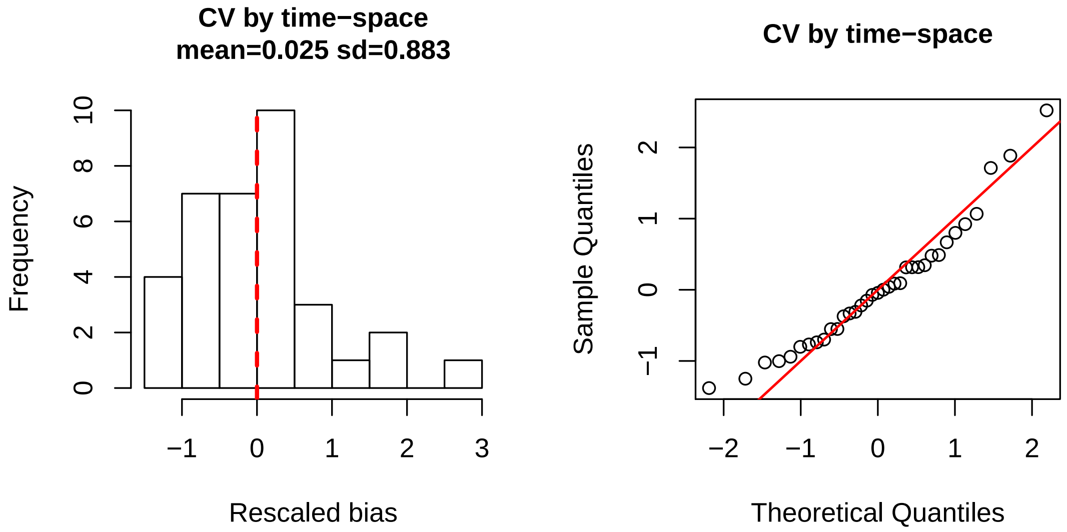

Figure S1.148: Guinea: Histogram and QQ-plot of the rescaled difference between the smoothed estimates and the direct estimates in the cross validation study. The differences between the two estimates are rescaled by the square root of the total variance of the two estimates.

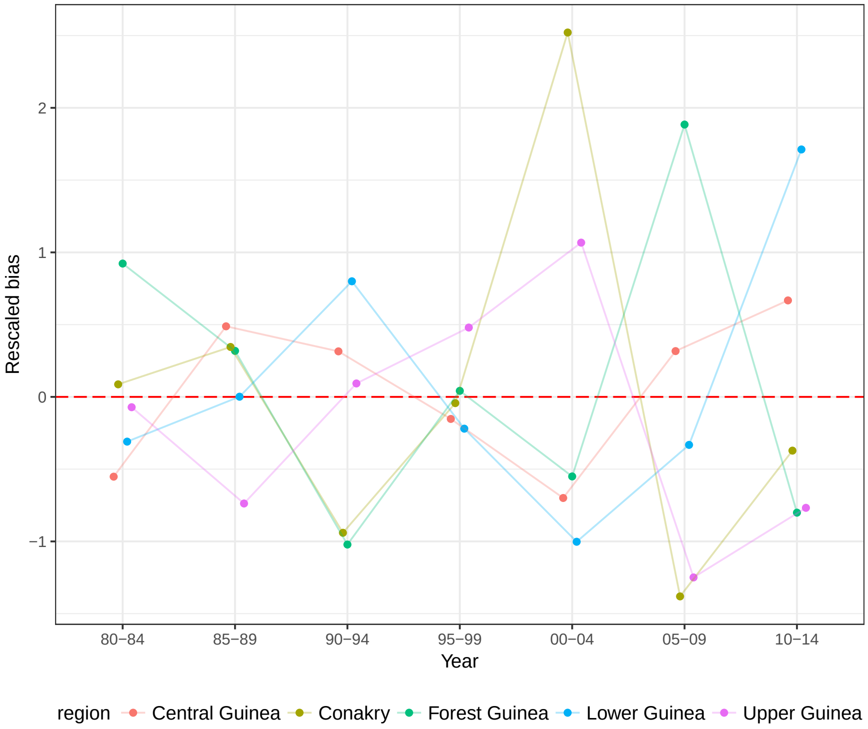

Figure S1.149: Guinea: Line plot of the difference between smoothed estimates and the direct estimates in the cross validation study. The differences between the two estimates are rescaled by the square root of the total variance of the two estimates.

3.5.17 Kenya

DHS surveys were conducted in Kenya in 1993, 1998, 2003, 2008, and 2014.

We fit both the RW2 only model to the combined national data, and compare the time trend at national level with the estimates produced by the UN and IHME in Figure S1.150. We then adjusted the combined national data to the UN estimates of U5MR, and refit the models on the benchmarked data.

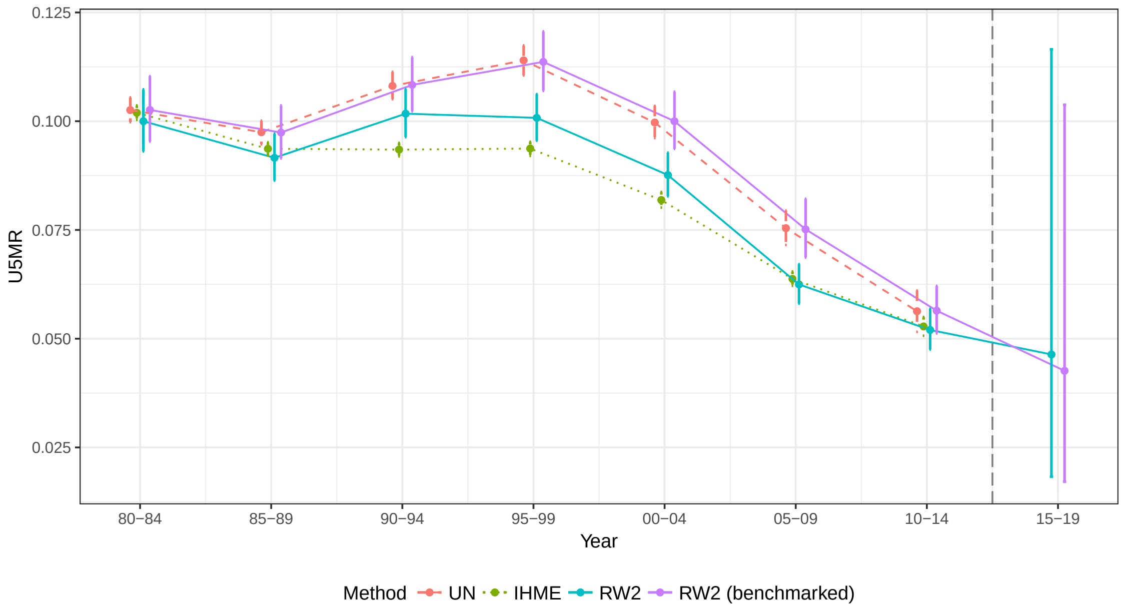

Figure S1.150: Kenya: Temporal national trends along with UN (B3) estimates described in You et al. (2015) and IHME estimates based on GBD 2015 Child Mortality Collaborators (2016). RW2 represents the smoothed national estimates using the original data before benchmarking with UN estimates. RW2-adj represents the smoothed national estimates using the benchmarked data.

We fit the RW2 model to the benchmarked data in each area. We compare the results in Figure S1.151 to S1.155. Figure S1.151 compares the smoothed estimates against the direct estimates. Figure S1.152 and Figure S1.153 show the posterior median estimates of U5MR in each region over time and the reductions from 1990 period respectively. Figure S1.154 shows the smoothed estimates by region over time and Figure S1.155 compares the smoothed estimates with direct estimates from each survey for each region over time.

We further assess the RW2 model by holding out some observations, and compare the projections to the direct estimates in these holdout observations. Figure S1.156 compares the predicted estimates for the out-of-sample observations with the direct estimates by holding out observations from each area in each time period. Figure S1.157 compares the histogram of the bias rescaled by the total variance in the cross validation studies. Figure S1.158 compares the rescaled bias by region and time periods.

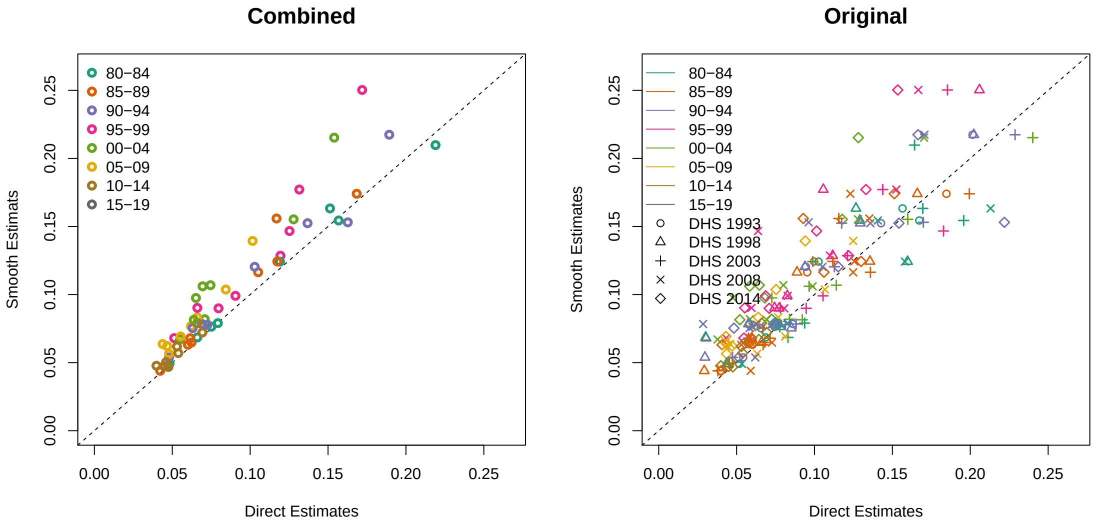

Figure S1.151: Kenya: Smooth versus direct Admin 1 estimates. Left: Combined (meta-analysis) survey estimate against combined direct estimates. Right: Combined (meta-analysis) survey estimate against direct estimates from each survey.

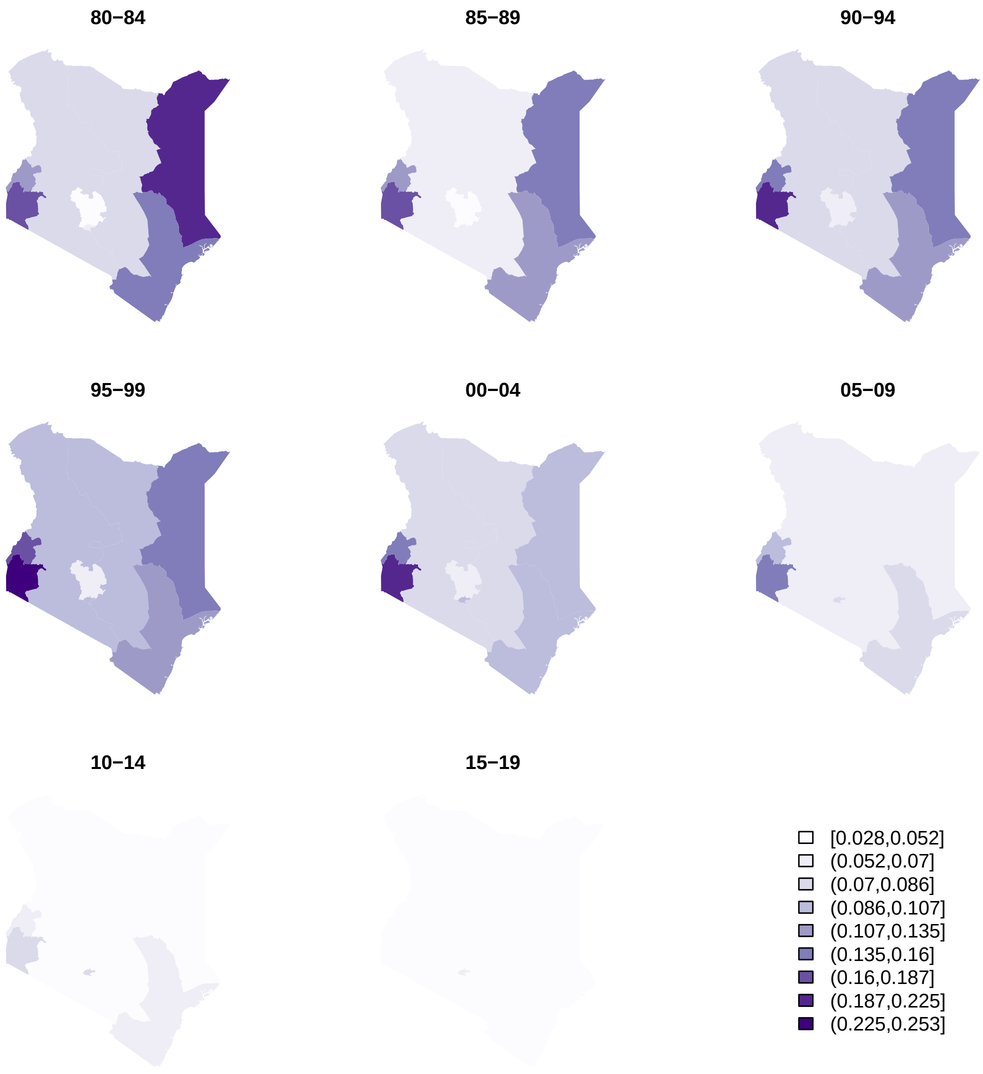

Figure S1.152: Kenya: Maps of posterior medians over time.

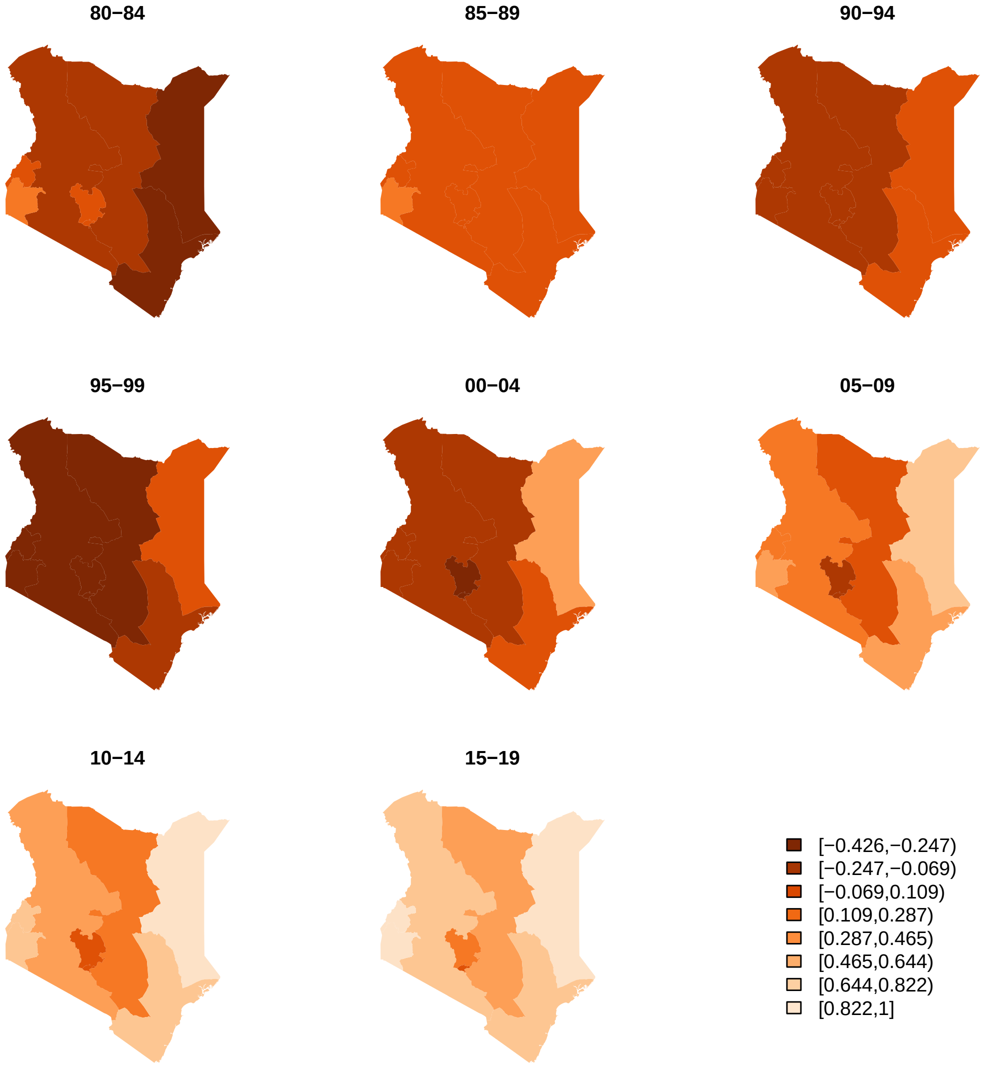

Figure S1.153: Kenya: Maps of reduction of posterior median U5MR in each five-year period compared to 1990 over time.

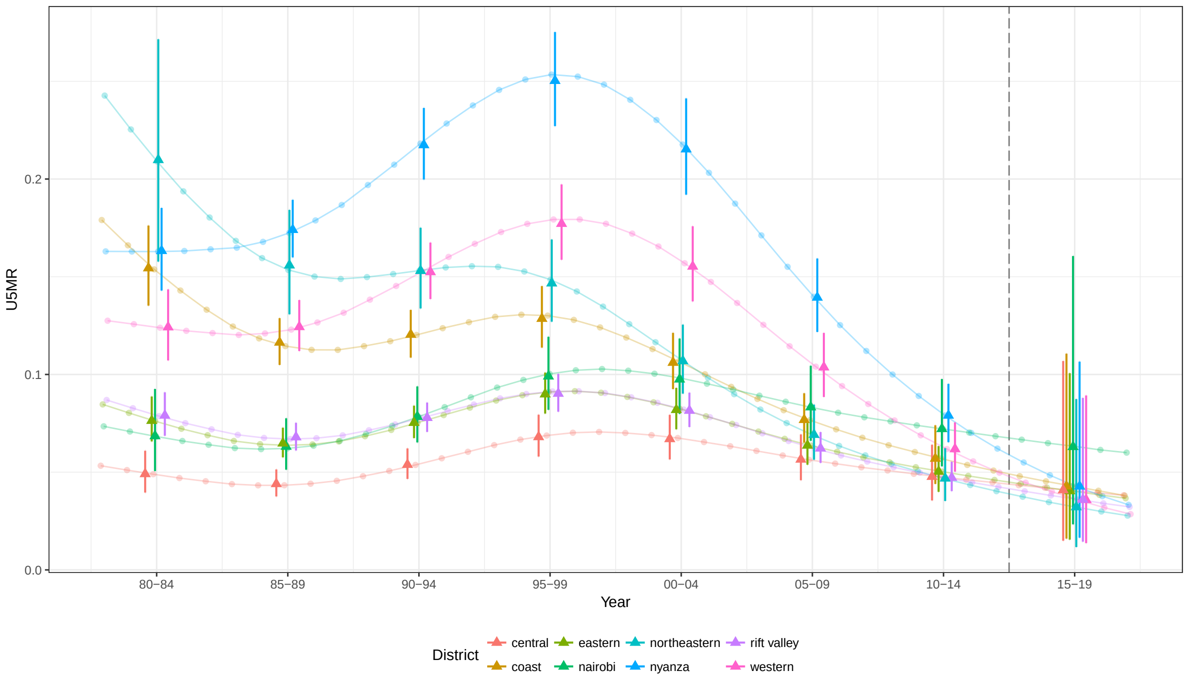

Figure S1.154: Kenya: Smoothed regional estimates over time. The line indicates yearly posterior median estimates and error bars indicate 95 % posterior credible interval at each time period.

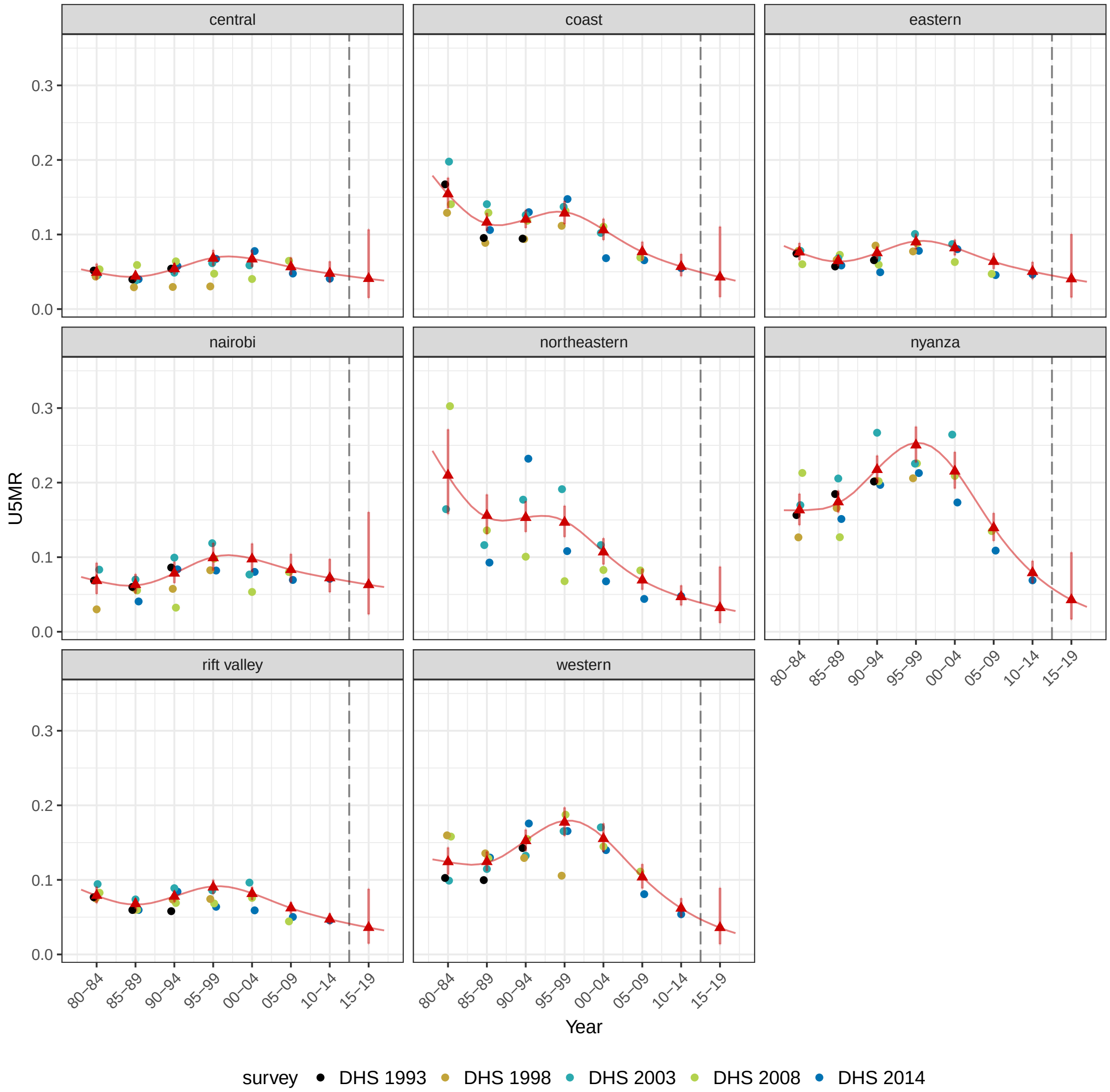

Figure S1.155: Kenya: Smoothed regional estimates over time compared to the direct estimates from each surveys. Direct estimates are not benchmarked with UN estimates. The line indicates posterior median and error bars indicate 95% posterior credible interval.

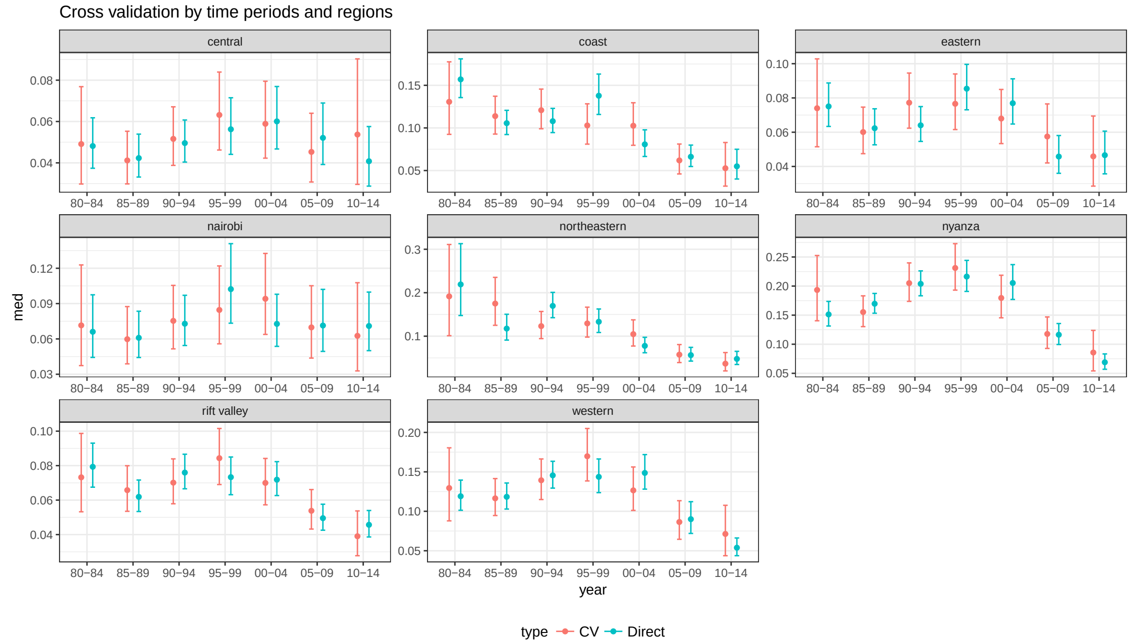

Figure S1.156: Kenya: Out-of-sample predictions along with direct estimates in the cross validation study where data from one region in each time period is held out and predicted using the rest of the data.

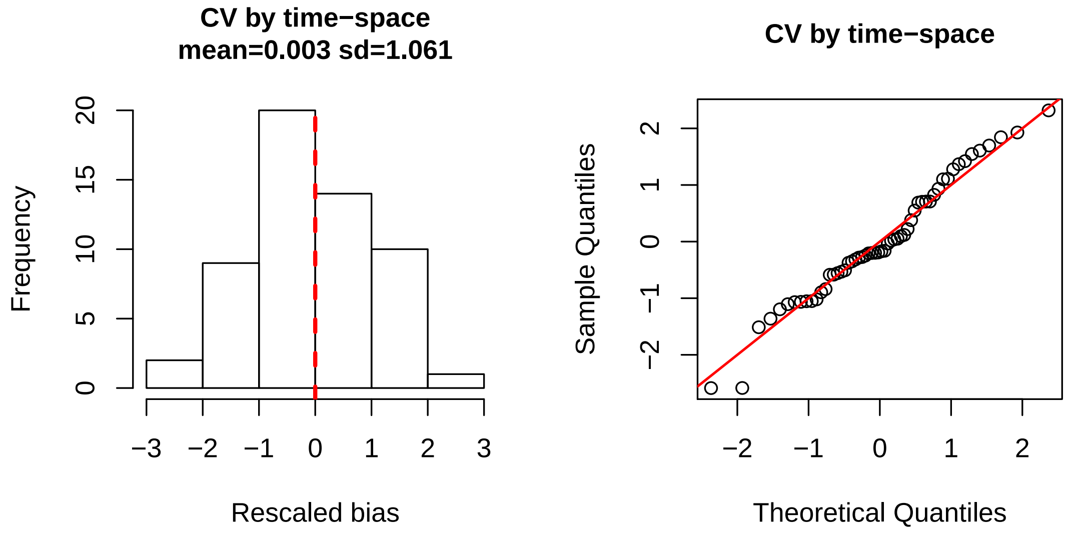

Figure S1.157: Kenya: Histogram and QQ-plot of the rescaled difference between the smoothed estimates and the direct estimates in the cross validation study. The differences between the two estimates are rescaled by the square root of the total variance of the two estimates.

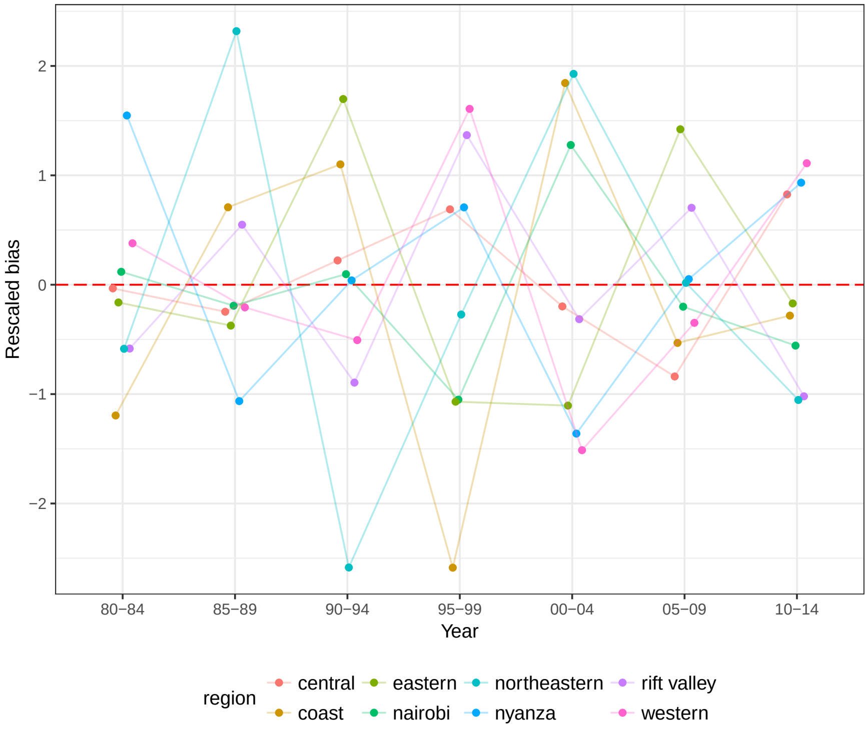

Figure S1.158: Kenya: Line plot of the difference between smoothed estimates and the direct estimates in the cross validation study. The differences between the two estimates are rescaled by the square root of the total variance of the two estimates.

3.5.18 Lesotho

DHS surveys were conducted in Lesotho in 2005, 2010, and 2014.

We fit both the RW2 only model to the combined national data, and compare the time trend at national level with the estimates produced by the UN and IHME in Figure S1.159. We then adjusted the combined national data to the UN estimates of U5MR, and refit the models on the benchmarked data.

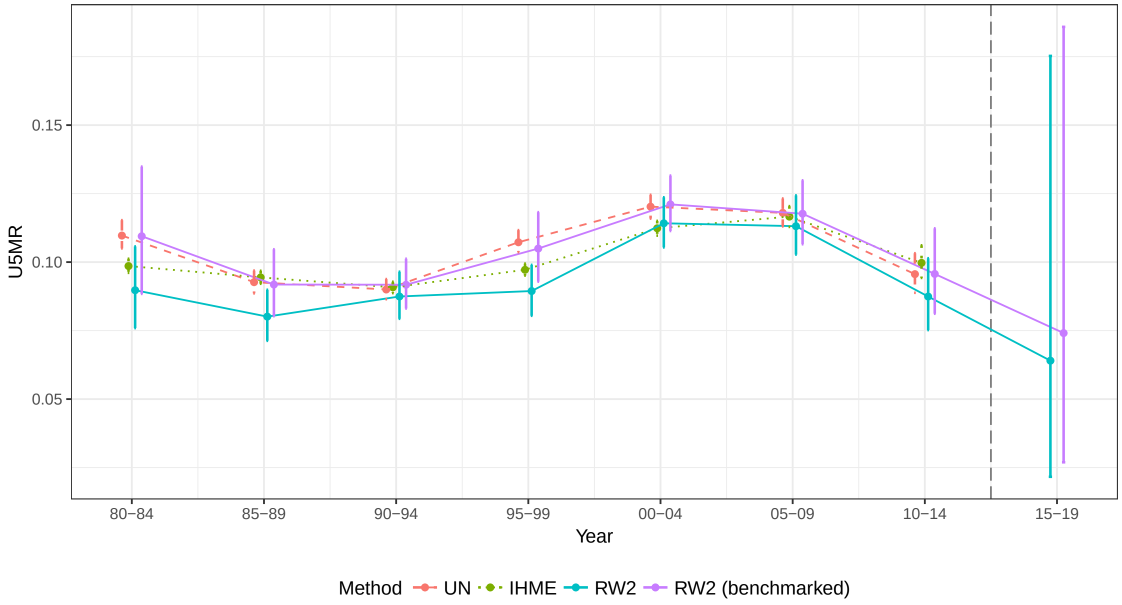

Figure S1.159: Lesotho: Temporal national trends along with UN (B3) estimates described in You et al. (2015) and IHME estimates based on GBD 2015 Child Mortality Collaborators (2016). RW2 represents the smoothed national estimates using the original data before benchmarking with UN estimates. RW2-adj represents the smoothed national estimates using the benchmarked data.

We fit the RW2 model to the benchmarked data in each area. We compare the results in Figure S1.160 to S1.164. Figure S1.160 compares the smoothed estimates against the direct estimates. Figure S1.161 and Figure S1.162 show the posterior median estimates of U5MR in each region over time and the reductions from 1990 period respectively. Figure S1.163 shows the smoothed estimates by region over time and Figure S1.164 compares the smoothed estimates with direct estimates from each survey for each region over time.

We further assess the RW2 model by holding out some observations, and compare the projections to the direct estimates in these holdout observations. Figure S1.165 compares the predicted estimates for the out-of-sample observations with the direct estimates by holding out observations from each area in each time period. Figure S1.166 compares the histogram of the bias rescaled by the total variance in the cross validation studies. Figure S1.167 compares the rescaled bias by region and time periods.

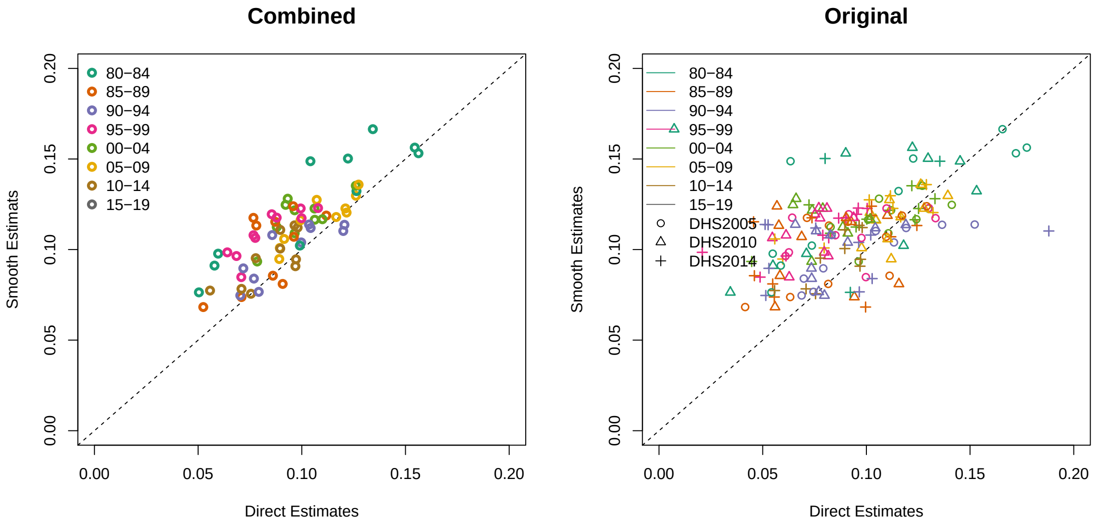

Figure S1.160: Lesotho: Smooth versus direct Admin 1 estimates. Left: Combined (meta-analysis) survey estimate against combined direct estimates. Right: Combined (meta-analysis) survey estimate against direct estimates from each survey.

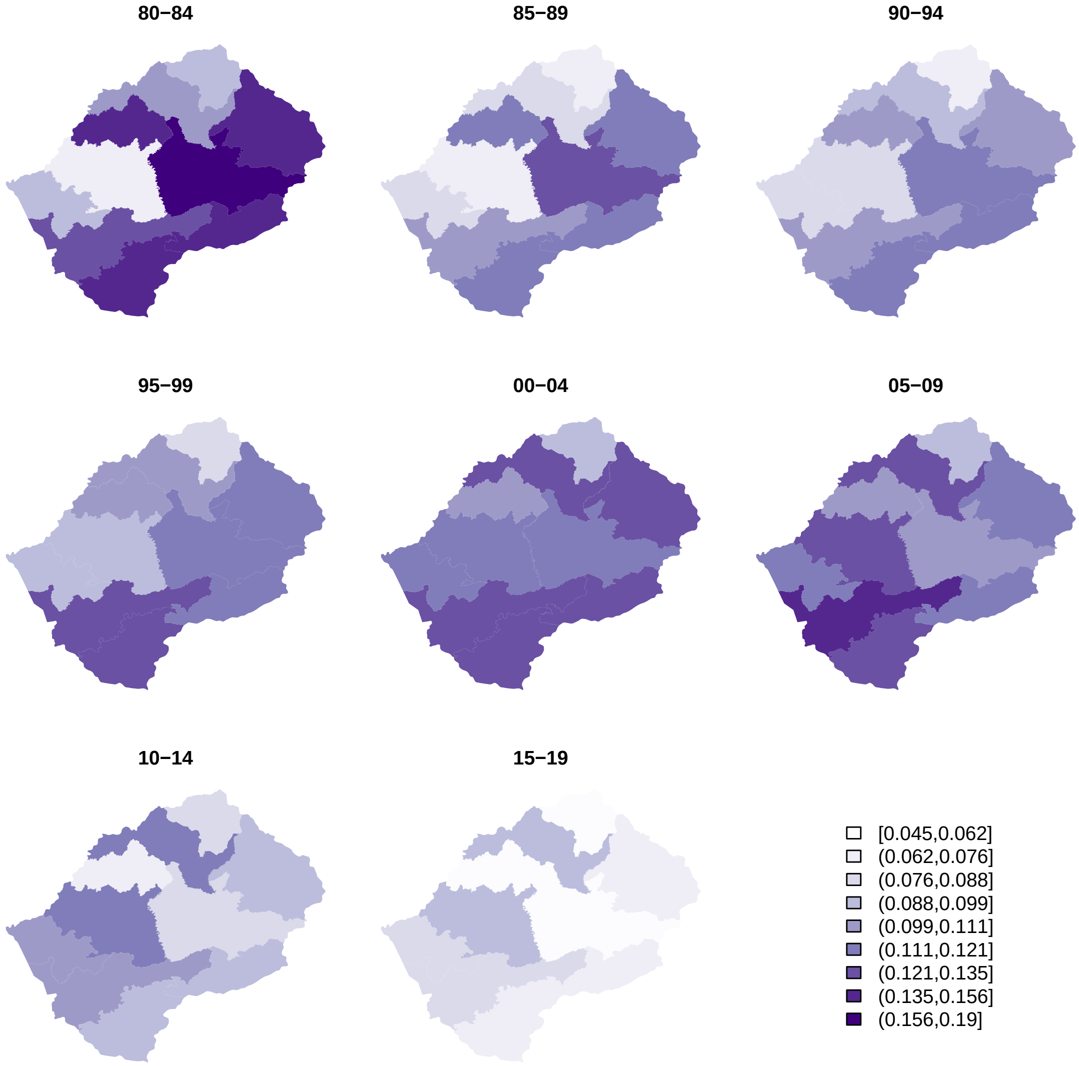

Figure S1.161: Lesotho: Maps of posterior medians over time.

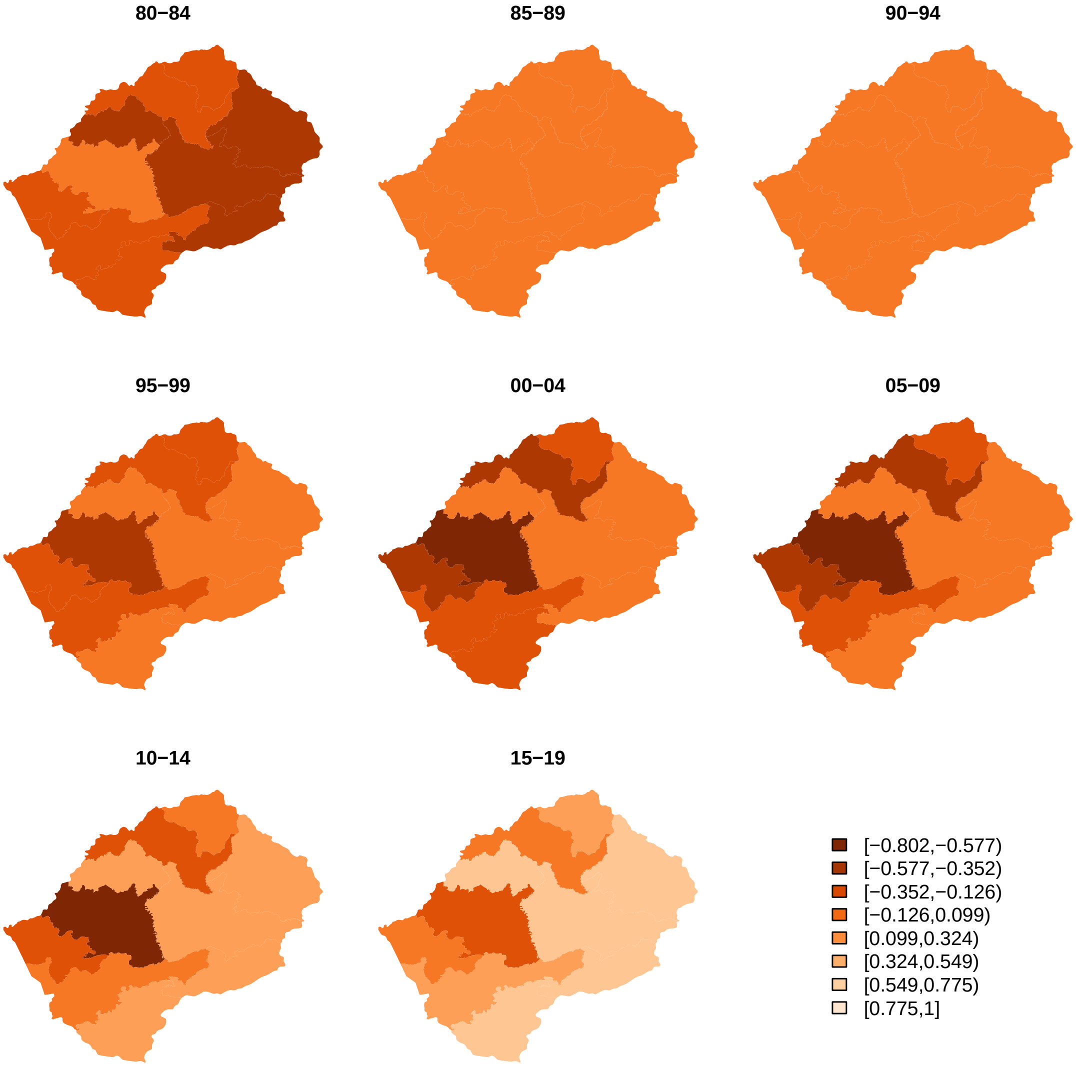

Figure S1.162: Lesotho: Maps of reduction of posterior median U5MR in each five-year period compared to 1990 over time.

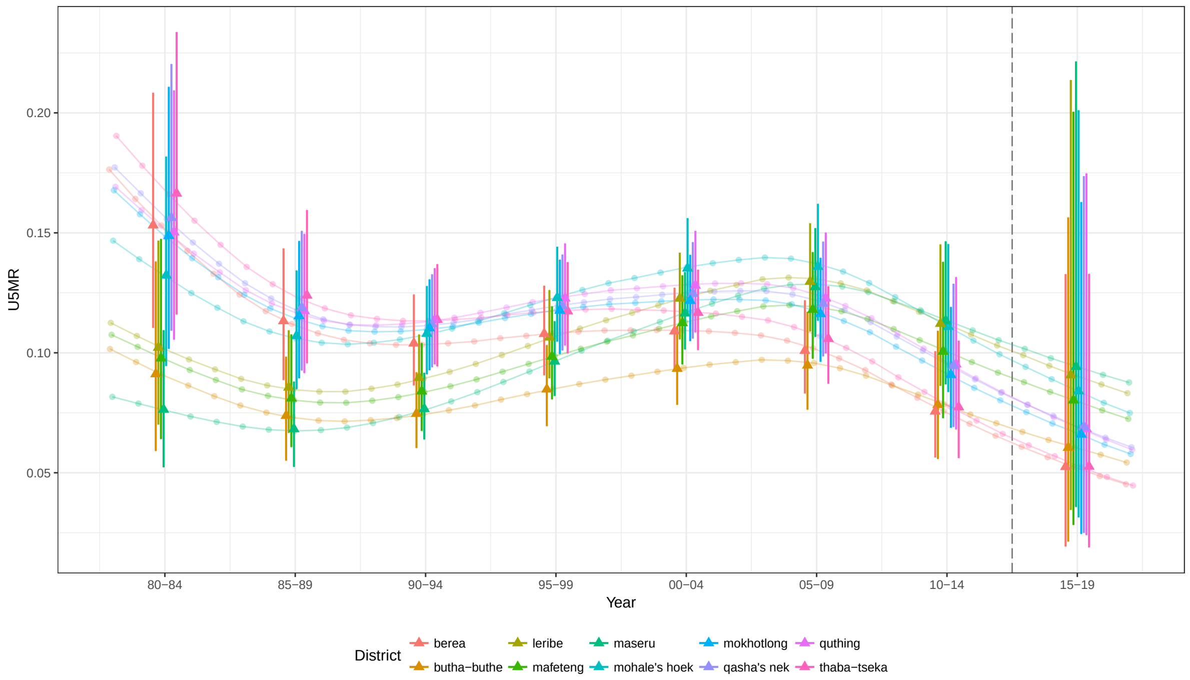

Figure S1.163: Lesotho: Smoothed regional estimates over time. The line indicates yearly posterior median estimates and error bars indicate 95 % posterior credible interval at each time period.

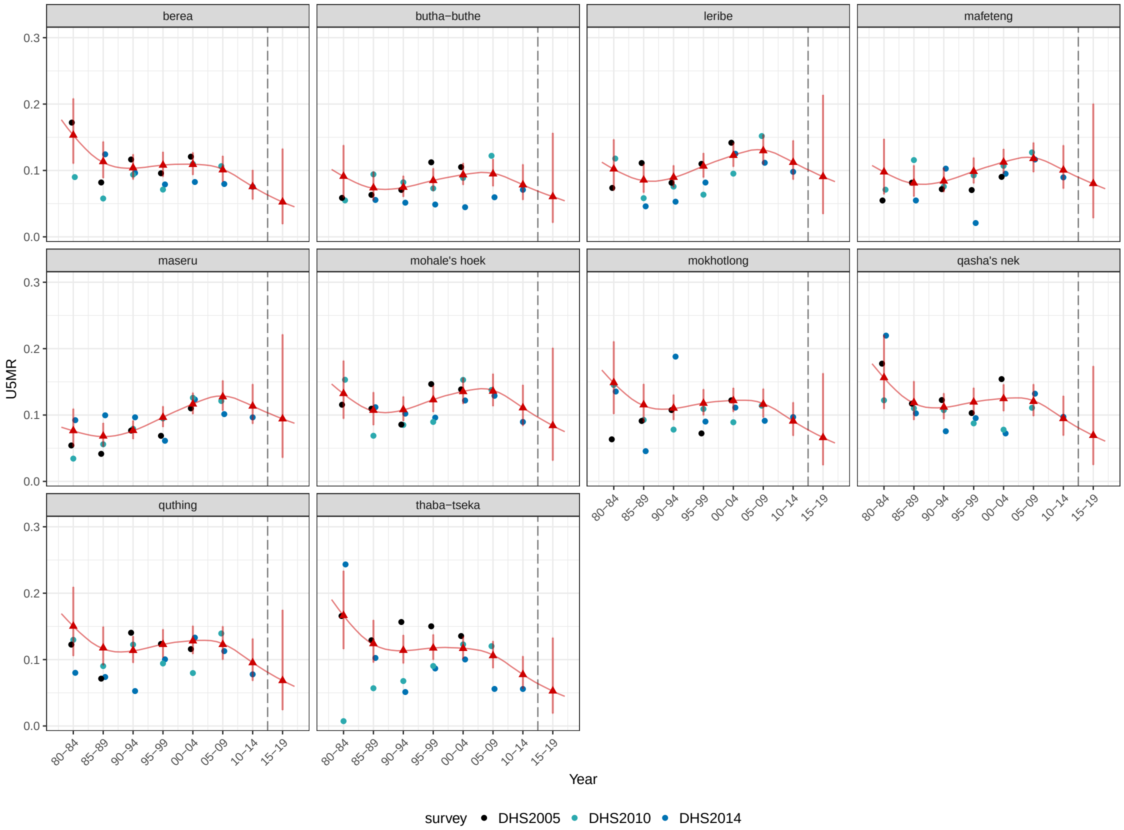

Figure S1.164: Lesotho: Smoothed regional estimates over time compared to the direct estimates from each surveys. Direct estimates are not benchmarked with UN estimates. The line indicates posterior median and error bars indicate 95% posterior credible interval.

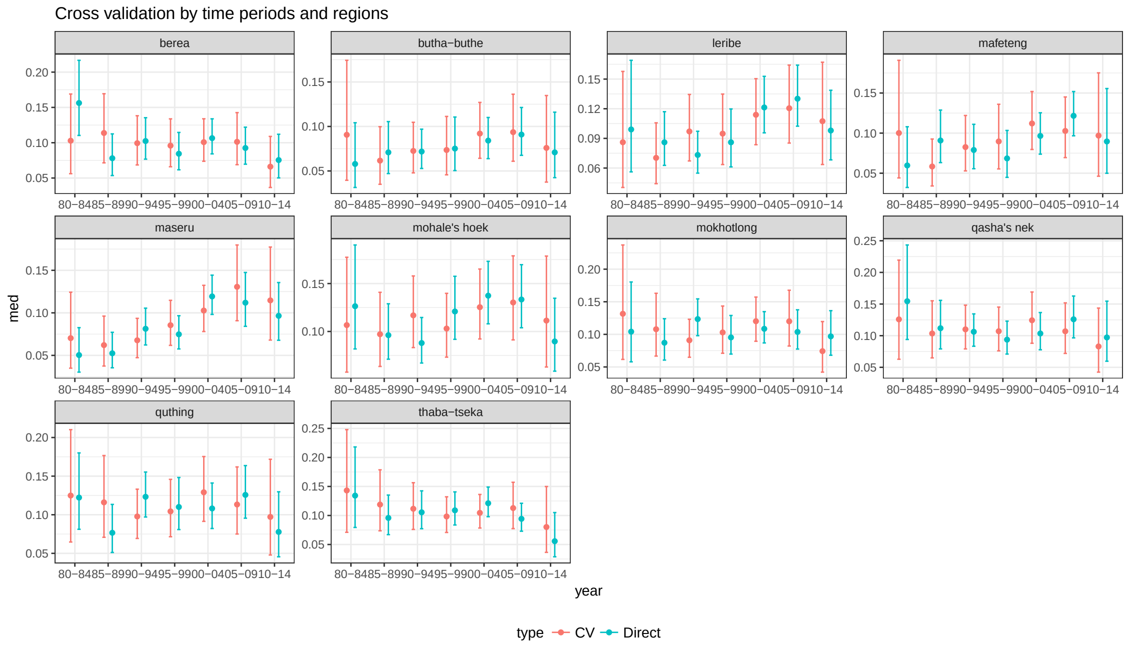

Figure S1.165: Lesotho: Out-of-sample predictions along with direct estimates in the cross validation study where data from one region in each time period is held out and predicted using the rest of the data.

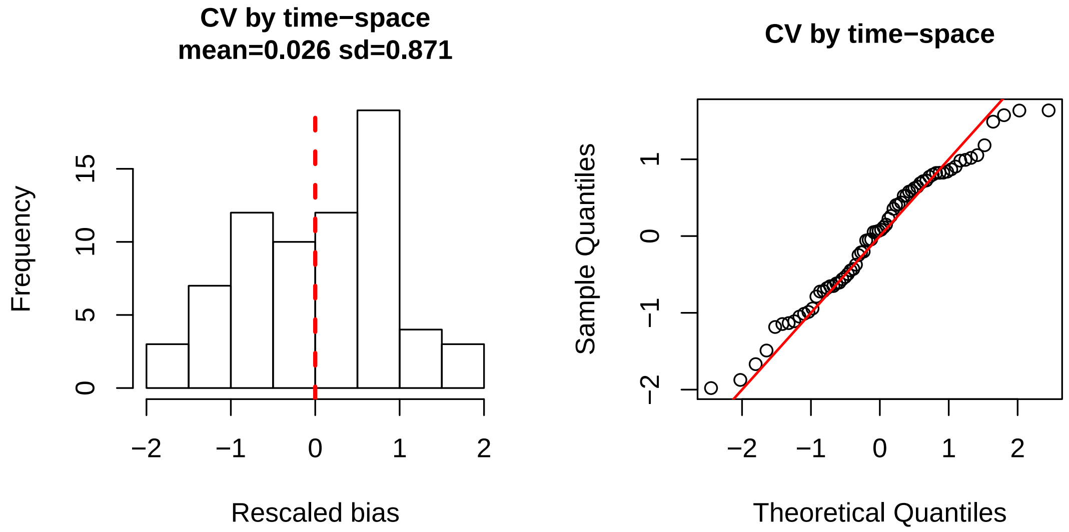

Figure S1.166: Lesotho: Histogram and QQ-plot of the rescaled difference between the smoothed estimates and the direct estimates in the cross validation study. The differences between the two estimates are rescaled by the square root of the total variance of the two estimates.

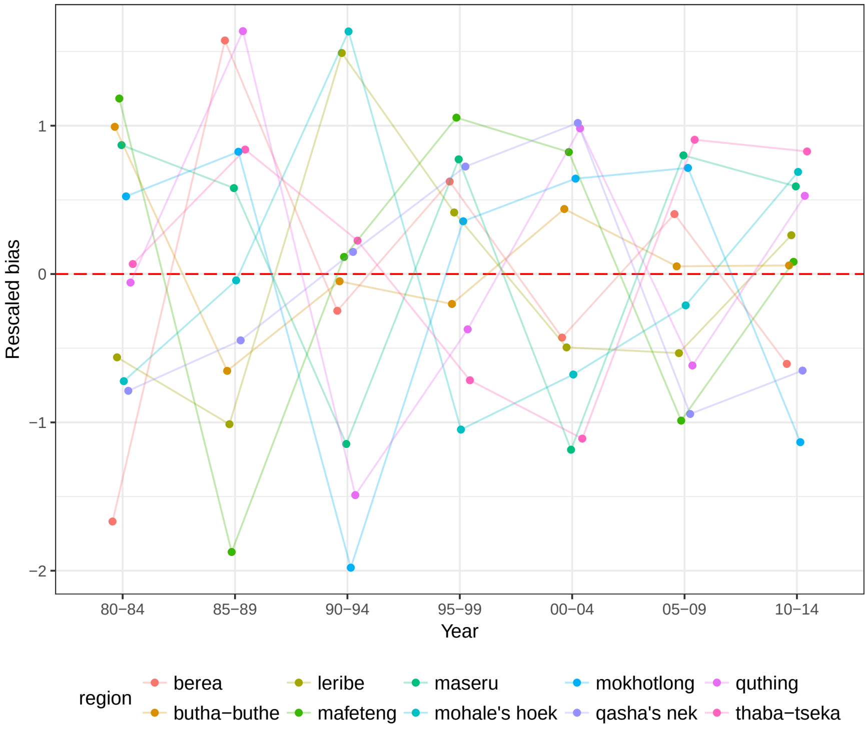

Figure S1.167: Lesotho: Line plot of the difference between smoothed estimates and the direct estimates in the cross validation study. The differences between the two estimates are rescaled by the square root of the total variance of the two estimates.

3.5.19 Liberia

DHS surveys were conducted in Liberia in 2007, and 2013.

We fit both the RW2 only model to the combined national data, and compare the time trend at national level with the estimates produced by the UN and IHME in Figure S1.168. We then adjusted the combined national data to the UN estimates of U5MR, and refit the models on the benchmarked data.

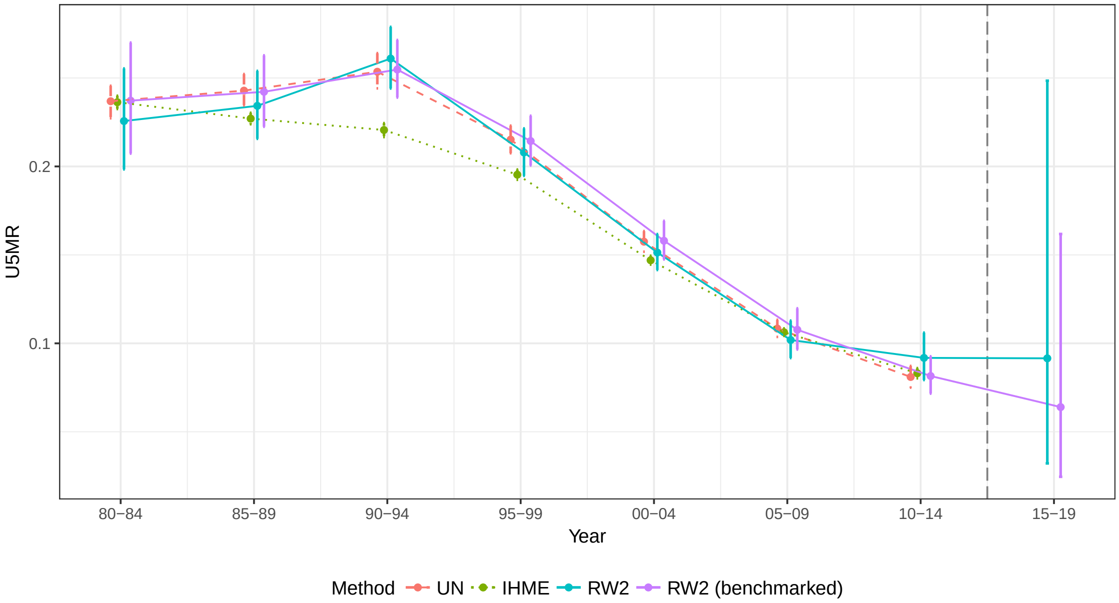

Figure S1.168: Liberia: Temporal national trends along with UN (B3) estimates described in You et al. (2015) and IHME estimates based on GBD 2015 Child Mortality Collaborators (2016). RW2 represents the smoothed national estimates using the original data before benchmarking with UN estimates. RW2-adj represents the smoothed national estimates using the benchmarked data.

We fit the RW2 model to the benchmarked data in each area. We compare the results in Figure S1.169 to S1.173. Figure S1.169 compares the smoothed estimates against the direct estimates. Figure S1.170 and Figure S1.171 show the posterior median estimates of U5MR in each region over time and the reductions from 1990 period respectively. Figure S1.172 shows the smoothed estimates by region over time and Figure S1.173 compares the smoothed estimates with direct estimates from each survey for each region over time.

We further assess the RW2 model by holding out some observations, and compare the projections to the direct estimates in these holdout observations. Figure S1.174 compares the predicted estimates for the out-of-sample observations with the direct estimates by holding out observations from each area in each time period. Figure S1.175 compares the histogram of the bias rescaled by the total variance in the cross validation studies. Figure S1.176 compares the rescaled bias by region and time periods.

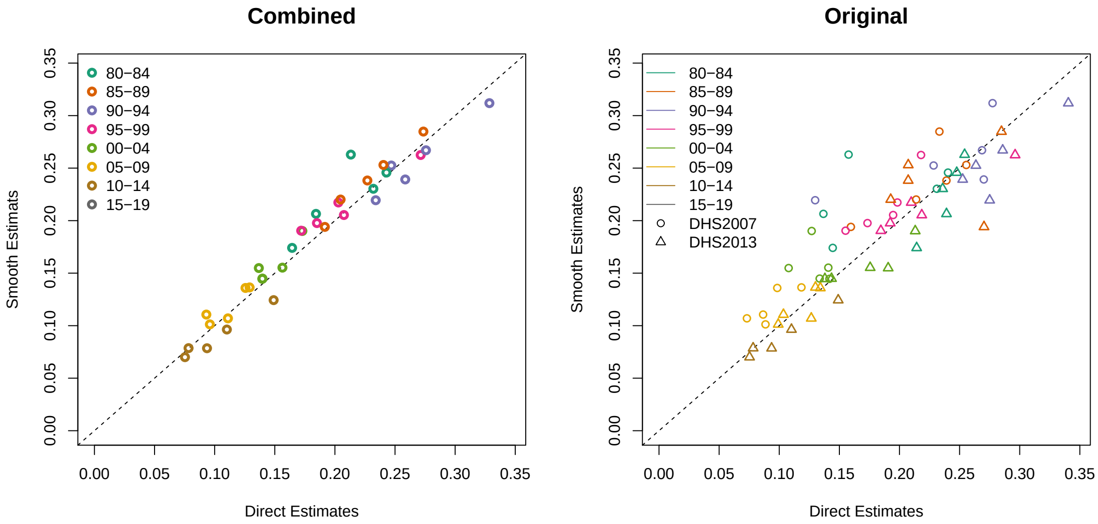

Figure S1.169: Liberia: Smooth versus direct Admin 1 estimates. Left: Combined (meta-analysis) survey estimate against combined direct estimates. Right: Combined (meta-analysis) survey estimate against direct estimates from each survey.

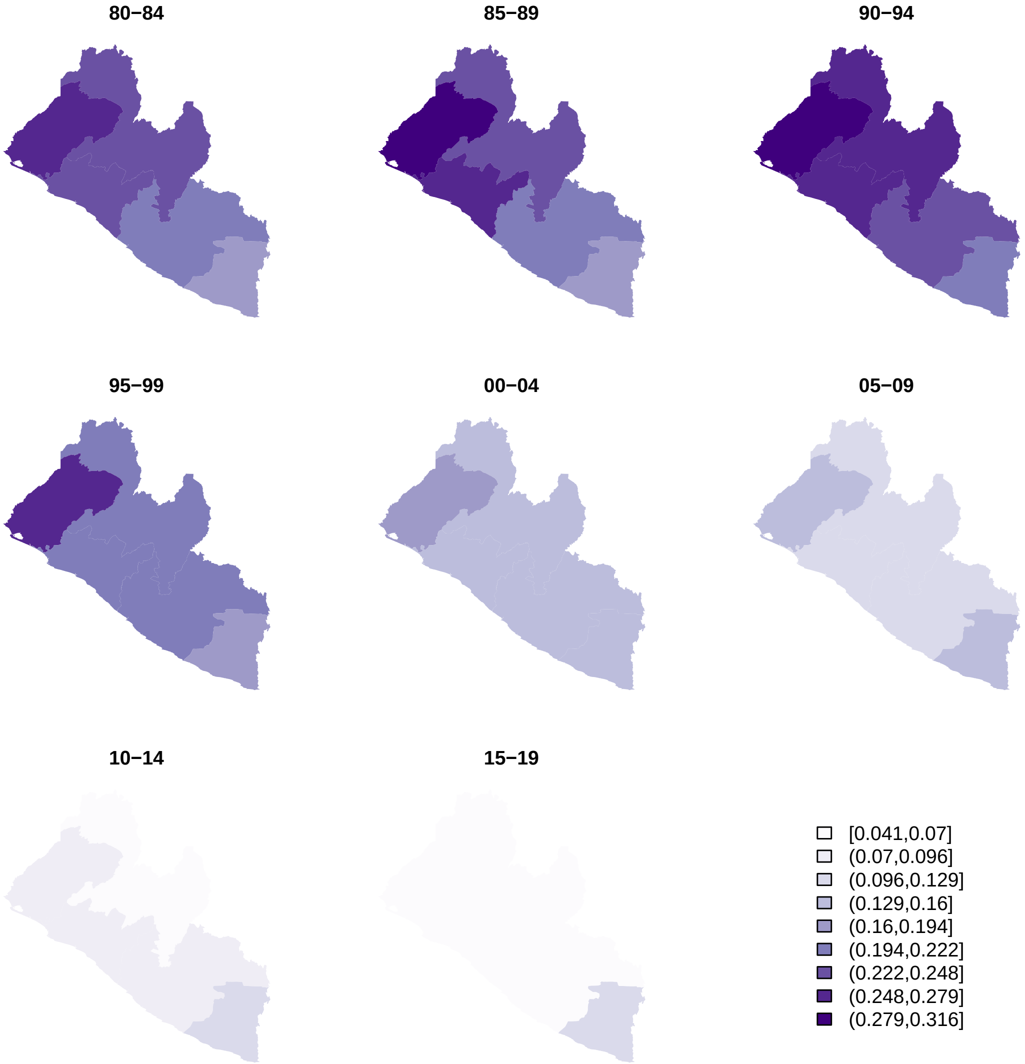

Figure S1.170: Liberia: Maps of posterior medians over time.

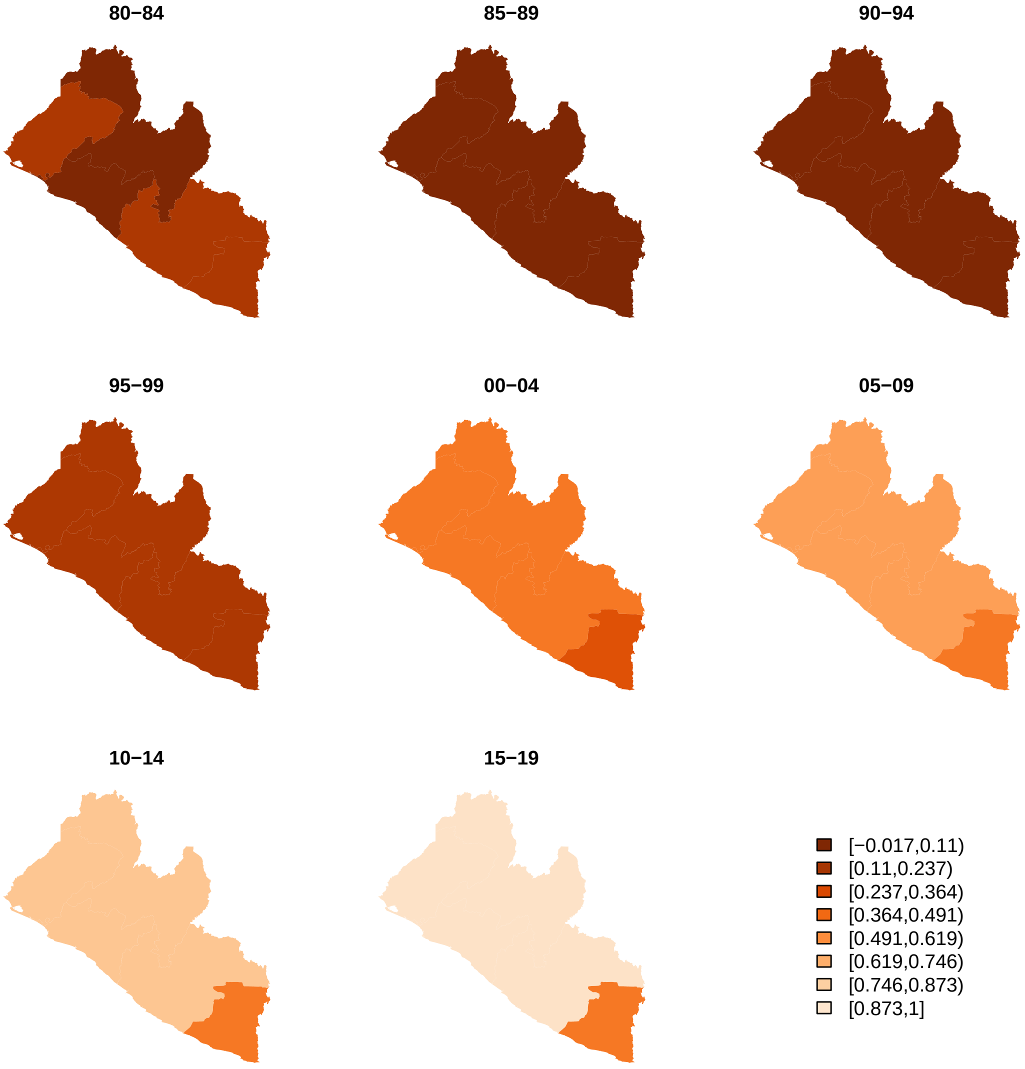

Figure S1.171: Liberia: Maps of reduction of posterior median U5MR in each five-year period compared to 1990 over time.

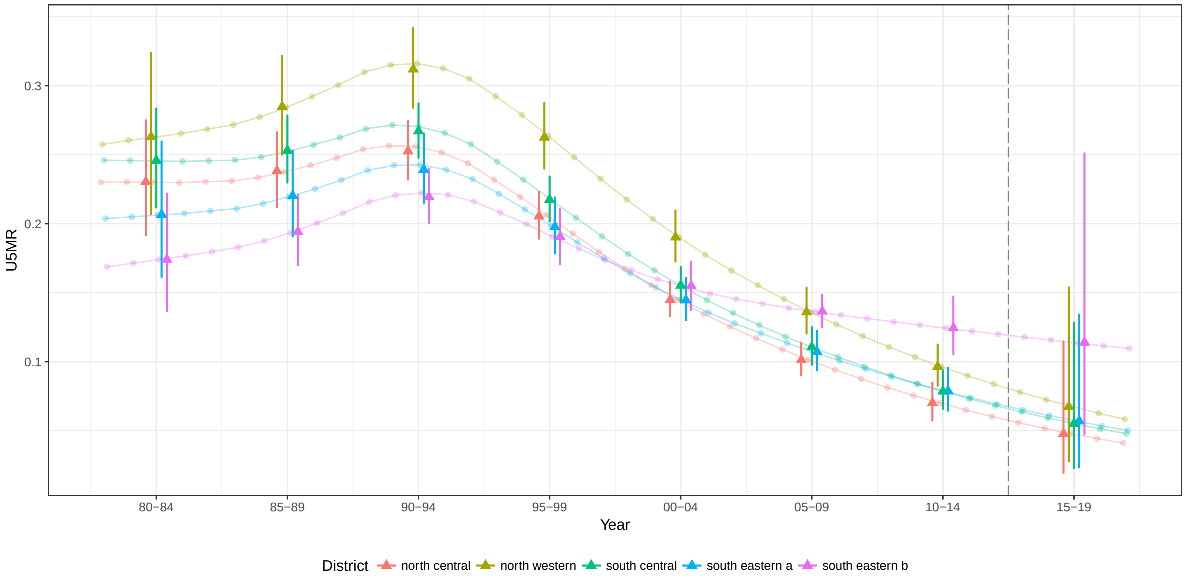

Figure S1.172: Liberia: Smoothed regional estimates over time. The line indicates yearly posterior median estimates and error bars indicate 95 % posterior credible interval at each time period.

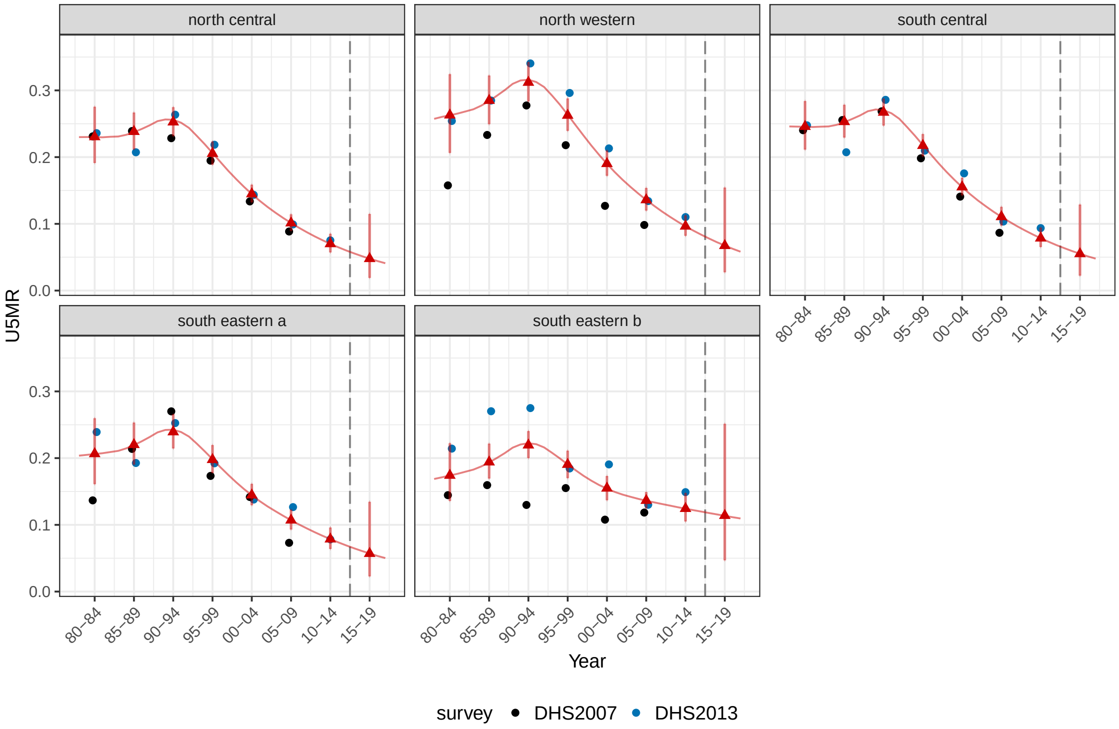

Figure S1.173: Liberia: Smoothed regional estimates over time compared to the direct estimates from each surveys. Direct estimates are not benchmarked with UN estimates. The line indicates posterior median and error bars indicate 95% posterior credible interval.

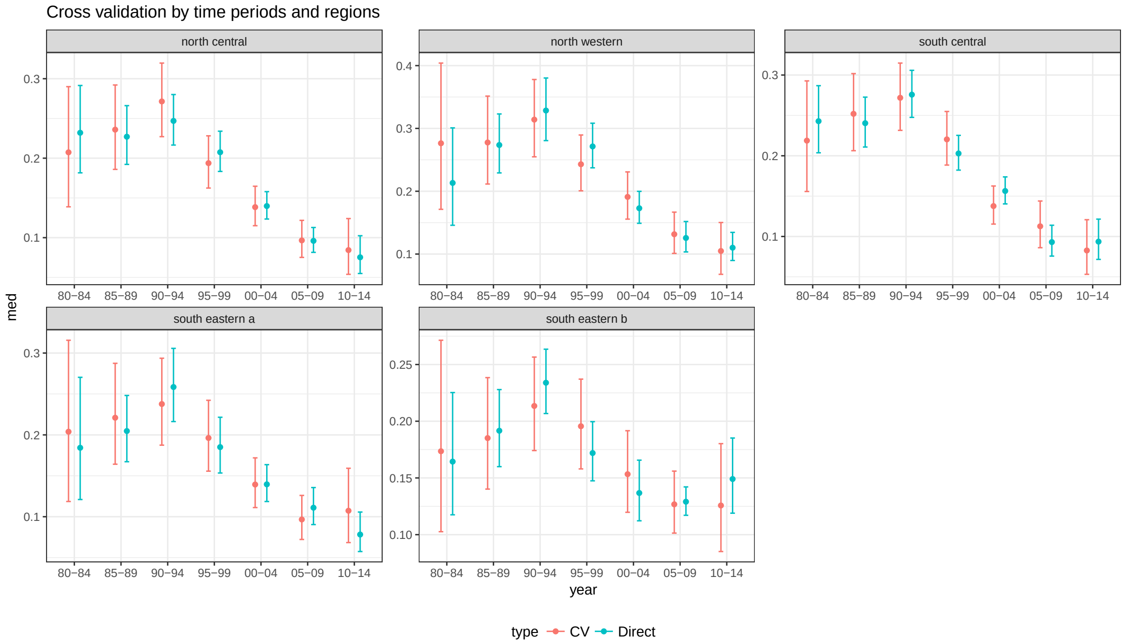

Figure S1.174: Liberia: Out-of-sample predictions along with direct estimates in the cross validation study where data from one region in each time period is held out and predicted using the rest of the data.

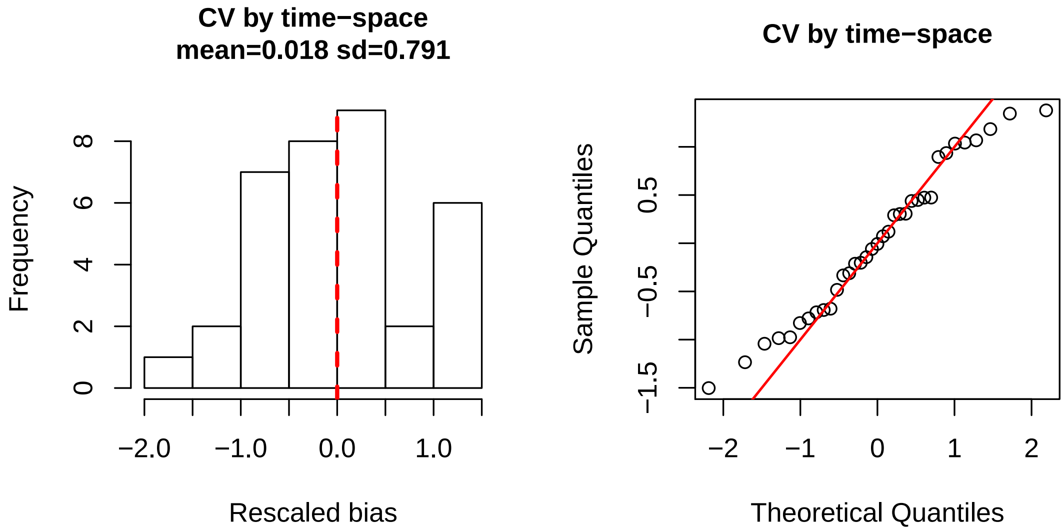

Figure S1.175: Liberia: Histogram and QQ-plot of the rescaled difference between the smoothed estimates and the direct estimates in the cross validation study. The differences between the two estimates are rescaled by the square root of the total variance of the two estimates.

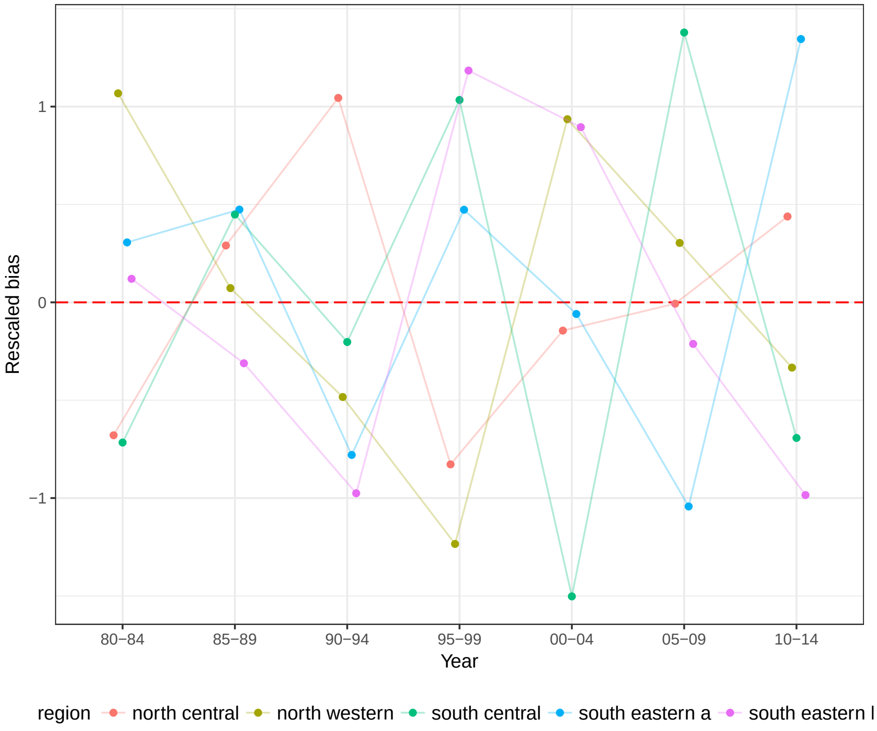

Figure S1.176: Liberia: Line plot of the difference between smoothed estimates and the direct estimates in the cross validation study. The differences between the two estimates are rescaled by the square root of the total variance of the two estimates.

3.5.20 Madagascar

DHS surveys were conducted in Madagascar in 1992, 1997, 2004, and 2009.

We fit both the RW2 only model to the combined national data, and compare the time trend at national level with the estimates produced by the UN and IHME in Figure S1.177. We then adjusted the combined national data to the UN estimates of U5MR, and refit the models on the benchmarked data.

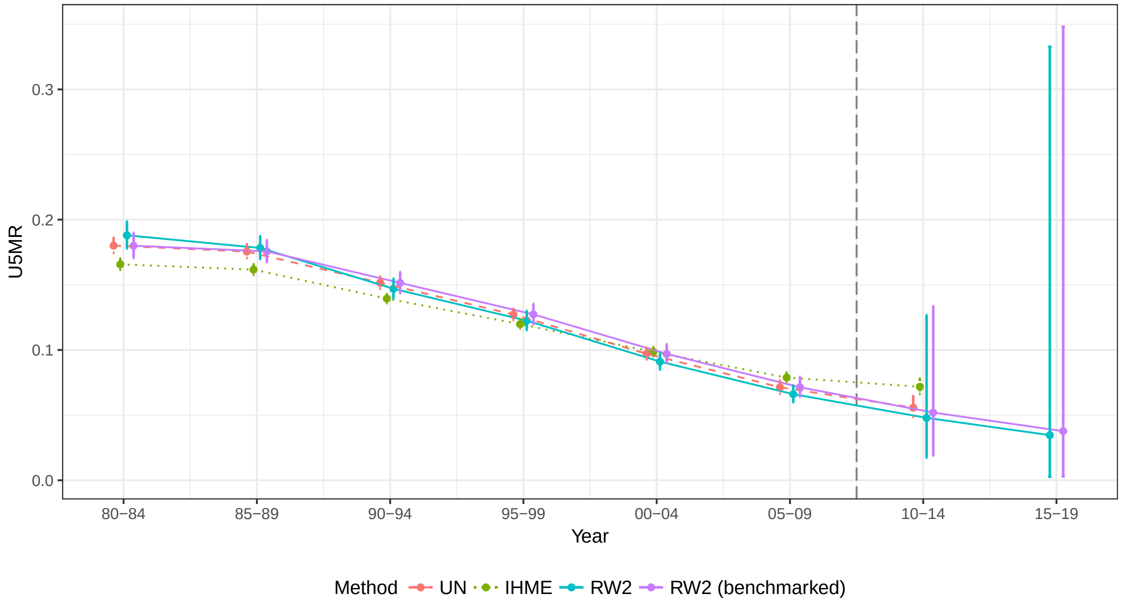

Figure S1.177: Madagascar: Temporal national trends along with UN (B3) estimates described in You et al. (2015) and IHME estimates based on GBD 2015 Child Mortality Collaborators (2016). RW2 represents the smoothed national estimates using the original data before benchmarking with UN estimates. RW2-adj represents the smoothed national estimates using the benchmarked data.

We fit the RW2 model to the benchmarked data in each area. We compare the results in Figure S1.178 to S1.182. Figure S1.178 compares the smoothed estimates against the direct estimates. Figure S1.179 and Figure S1.180 show the posterior median estimates of U5MR in each region over time and the reductions from 1990 period respectively. Figure S1.181 shows the smoothed estimates by region over time and Figure S1.182 compares the smoothed estimates with direct estimates from each survey for each region over time.

We further assess the RW2 model by holding out some observations, and compare the projections to the direct estimates in these holdout observations. Figure S1.183 compares the predicted estimates for the out-of-sample observations with the direct estimates by holding out observations from each area in each time period. Figure S1.184 compares the histogram of the bias rescaled by the total variance in the cross validation studies. Figure S1.185 compares the rescaled bias by region and time periods.

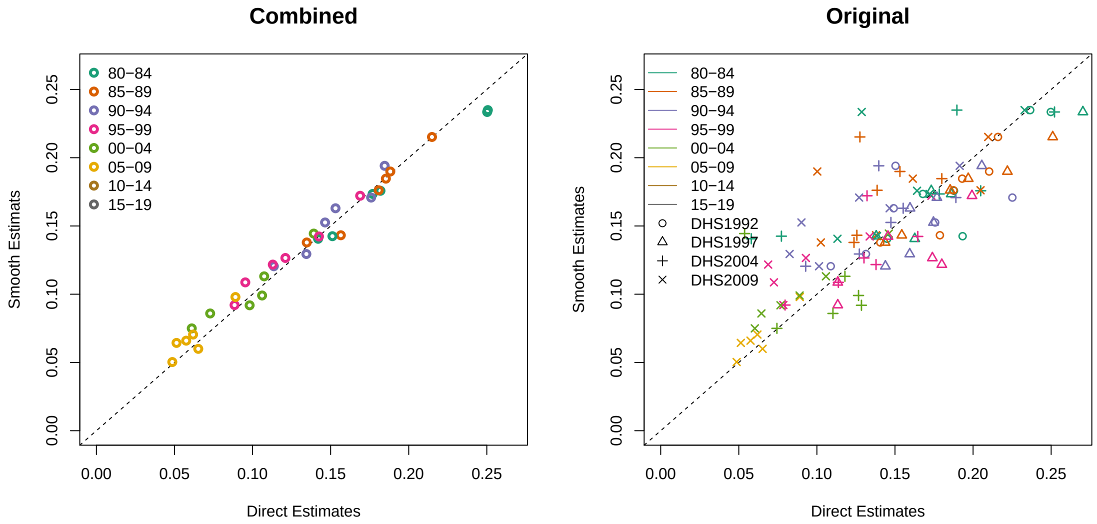

Figure S1.178: Madagascar: Smooth versus direct Admin 1 estimates. Left: Combined (meta-analysis) survey estimate against combined direct estimates. Right: Combined (meta-analysis) survey estimate against direct estimates from each survey.

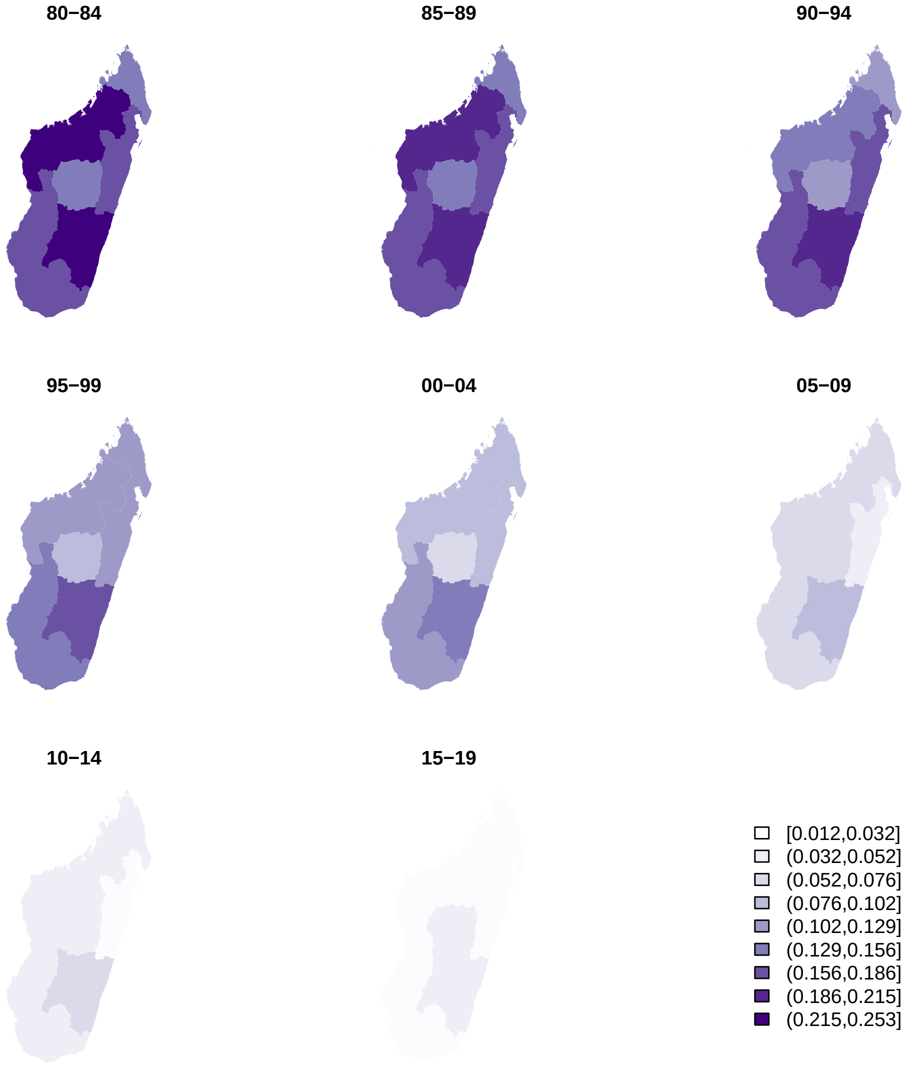

Figure S1.179: Madagascar: Maps of posterior medians over time.

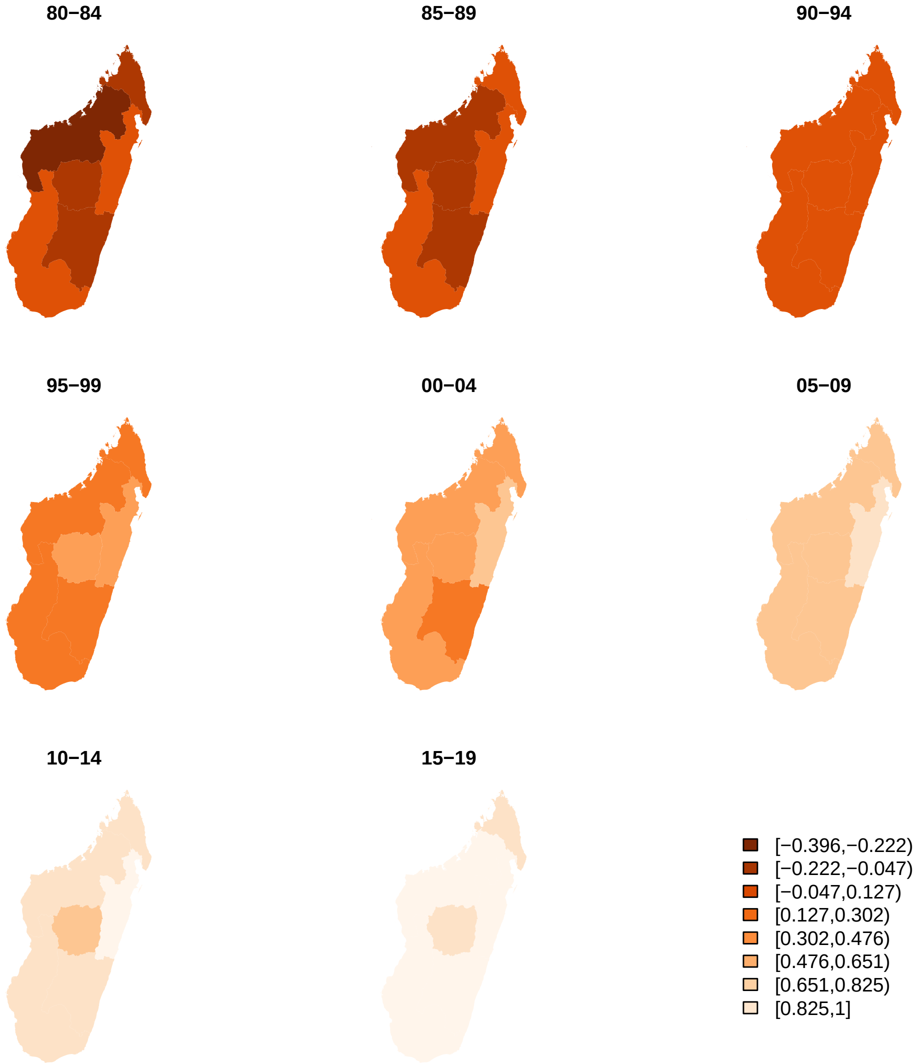

Figure S1.180: Madagascar: Maps of reduction of posterior median U5MR in each five-year period compared to 1990 over time.

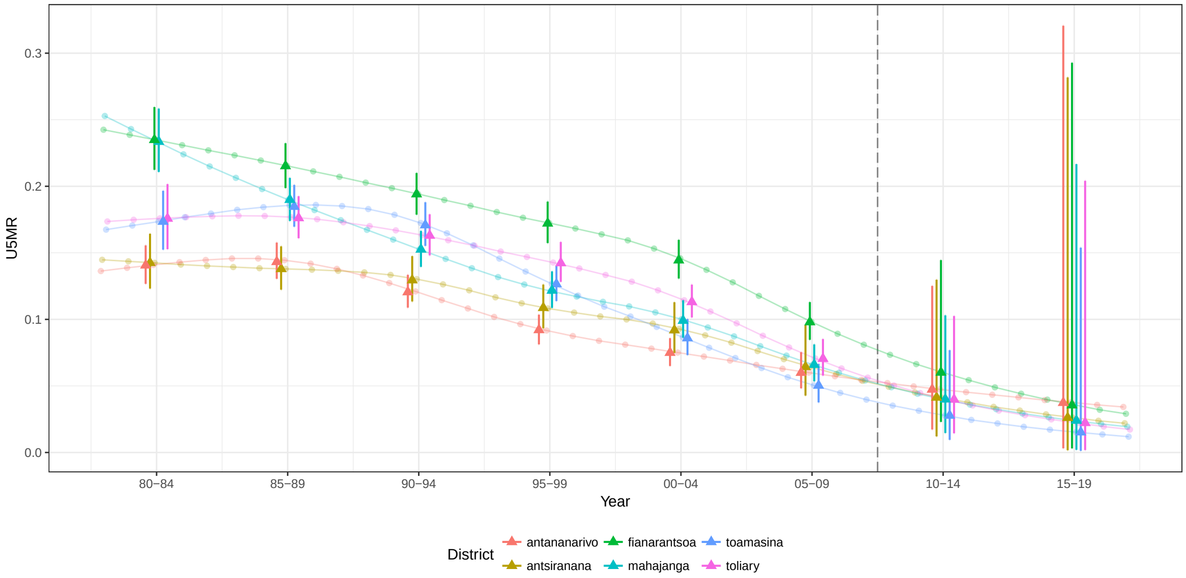

Figure S1.181: Madagascar: Smoothed regional estimates over time. The line indicates yearly posterior median estimates and error bars indicate 95 % posterior credible interval at each time period.

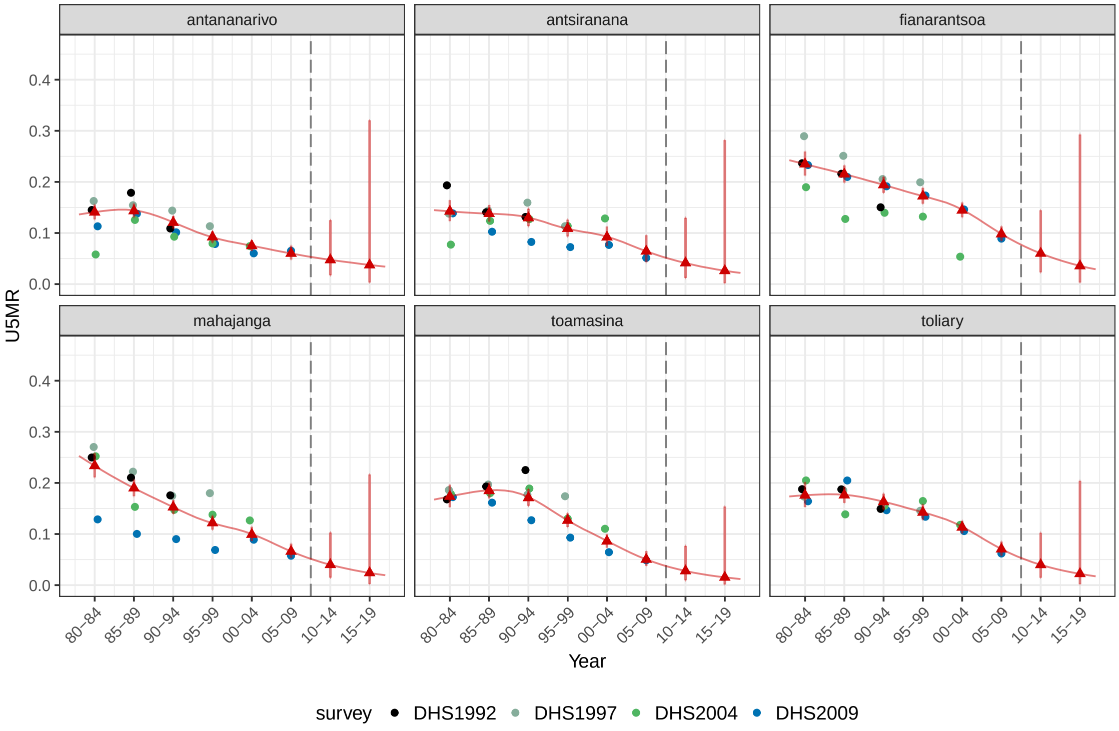

Figure S1.182: Madagascar: Smoothed regional estimates over time compared to the direct estimates from each surveys. Direct estimates are not benchmarked with UN estimates. The line indicates posterior median and error bars indicate 95% posterior credible interval.

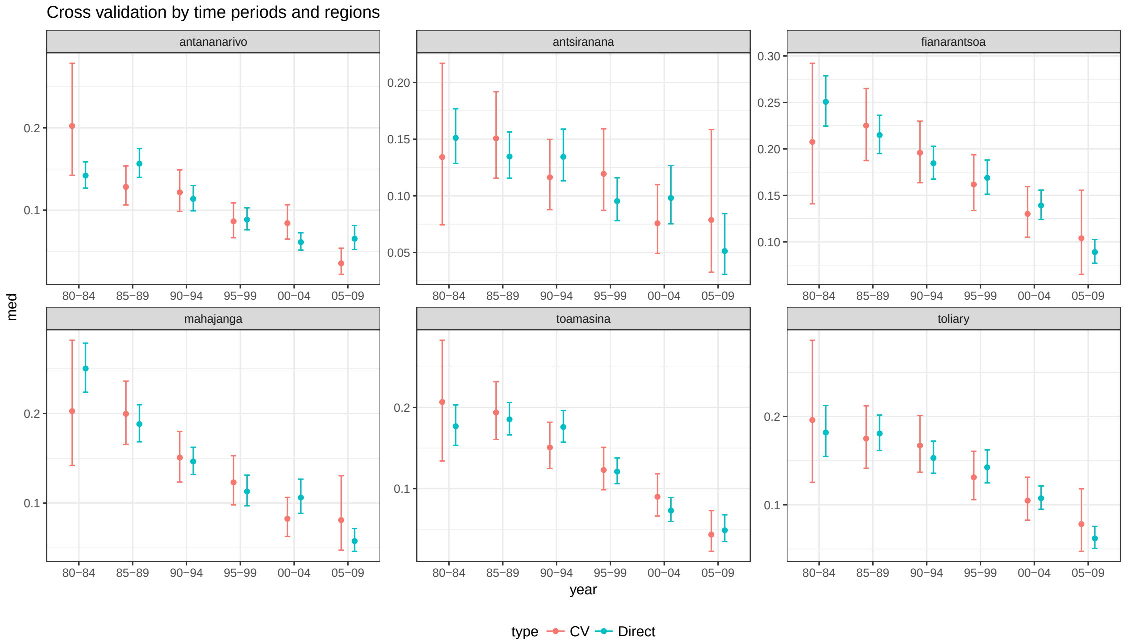

Figure S1.183: Madagascar: Out-of-sample predictions along with direct estimates in the cross validation study where data from one region in each time period is held out and predicted using the rest of the data.

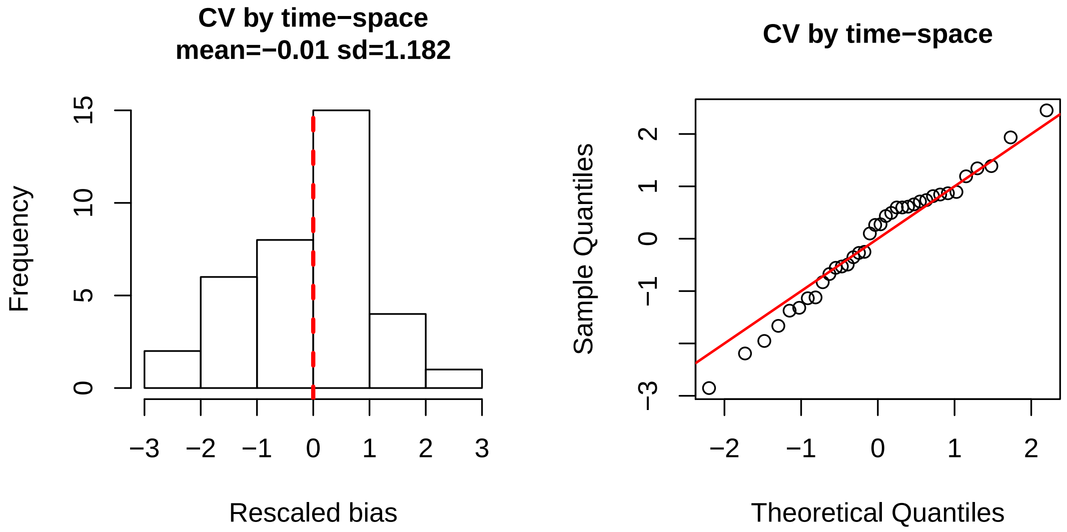

Figure S1.184: Madagascar: Histogram and QQ-plot of the rescaled difference between the smoothed estimates and the direct estimates in the cross validation study. The differences between the two estimates are rescaled by the square root of the total variance of the two estimates.

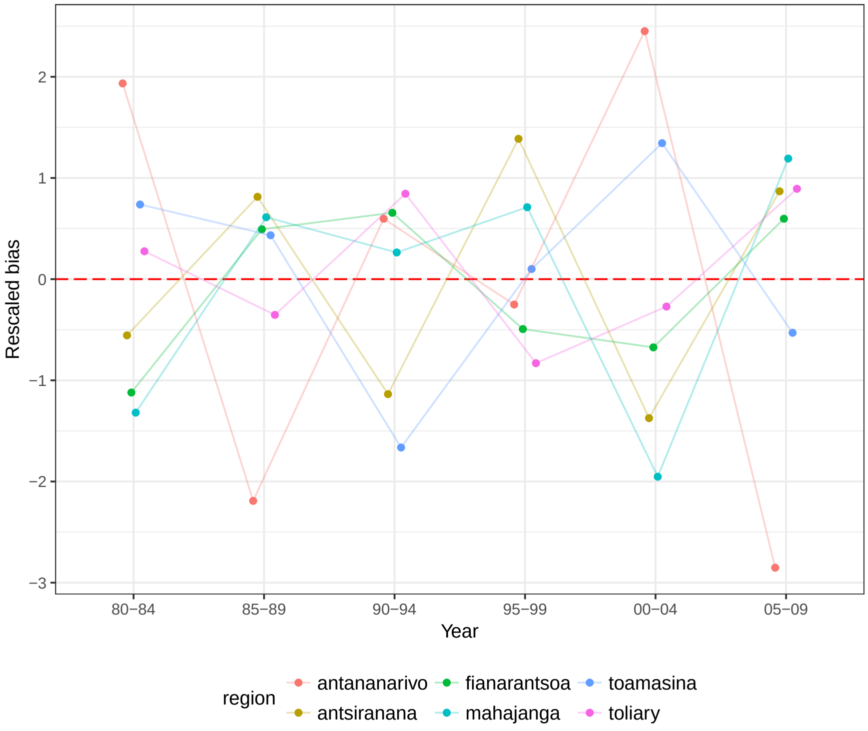

Figure S1.185: Madagascar: Line plot of the difference between smoothed estimates and the direct estimates in the cross validation study. The differences between the two estimates are rescaled by the square root of the total variance of the two estimates.

3.5.21 Malawi

DHS surveys were conducted in Malawi in 1992, 2000, 2004, 2010, and 2015.

We fit both the RW2 only model to the combined national data, and compare the time trend at national level with the estimates produced by the UN and IHME in Figure S1.186. We then adjusted the combined national data to the UN estimates of U5MR, and refit the models on the benchmarked data.

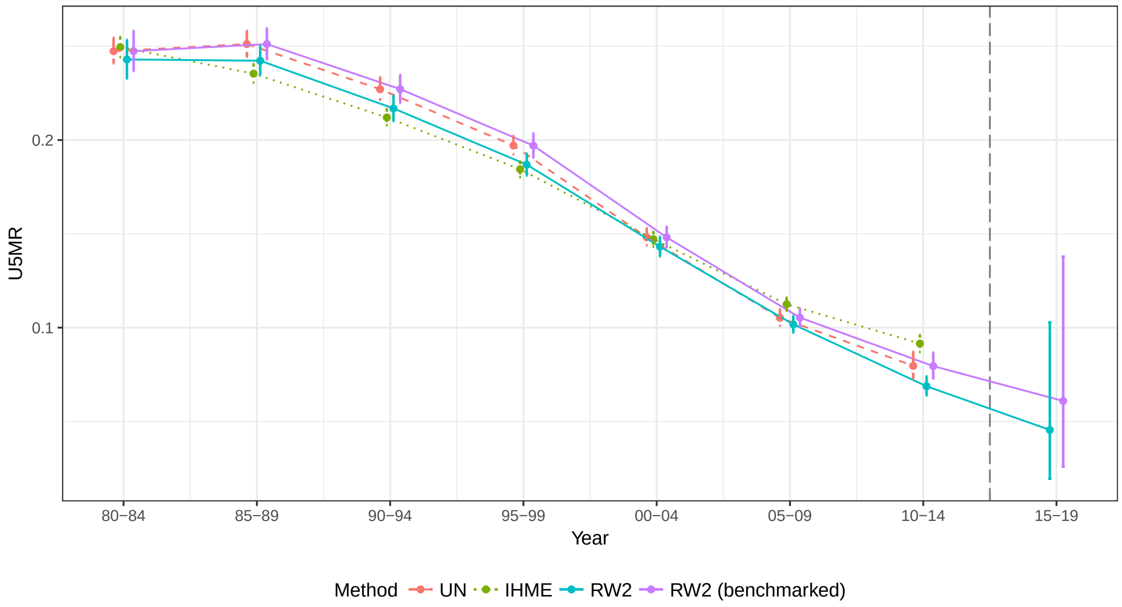

Figure S1.186: Malawi: Temporal national trends along with UN (B3) estimates described in You et al. (2015) and IHME estimates based on GBD 2015 Child Mortality Collaborators (2016). RW2 represents the smoothed national estimates using the original data before benchmarking with UN estimates. RW2-adj represents the smoothed national estimates using the benchmarked data.

We fit the RW2 model to the benchmarked data in each area. We compare the results in Figure S1.187 to S1.191. Figure S1.187 compares the smoothed estimates against the direct estimates. Figure S1.188 and Figure S1.189 show the posterior median estimates of U5MR in each region over time and the reductions from 1990 period respectively. Figure S1.190 shows the smoothed estimates by region over time and Figure S1.191 compares the smoothed estimates with direct estimates from each survey for each region over time.

We further assess the RW2 model by holding out some observations, and compare the projections to the direct estimates in these holdout observations. Figure S1.192 compares the predicted estimates for the out-of-sample observations with the direct estimates by holding out observations from each area in each time period. Figure S1.193 compares the histogram of the bias rescaled by the total variance in the cross validation studies. Figure S1.194 compares the rescaled bias by region and time periods.

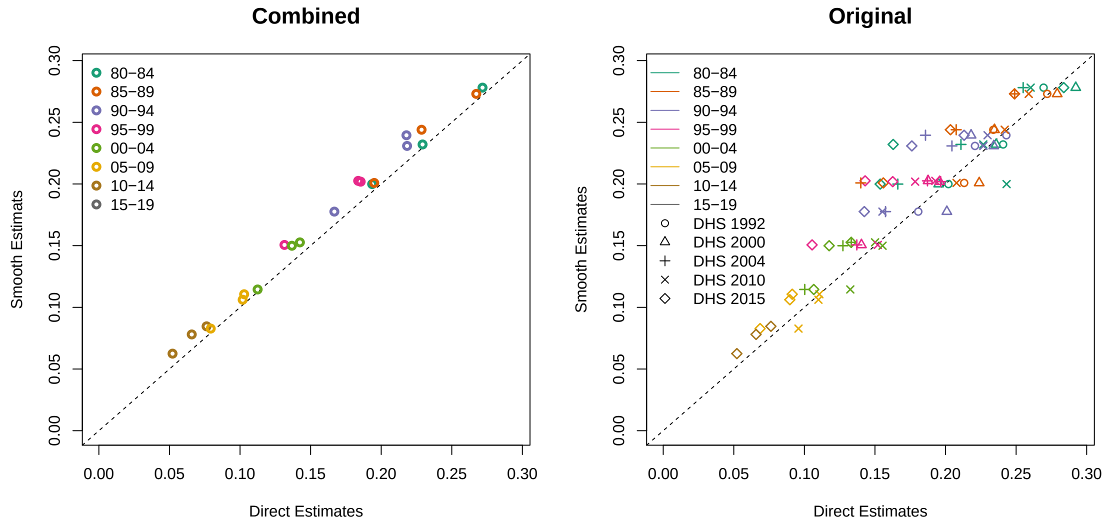

Figure S1.187: Malawi: Smooth versus direct Admin 1 estimates. Left: Combined (meta-analysis) survey estimate against combined direct estimates. Right: Combined (meta-analysis) survey estimate against direct estimates from each survey.

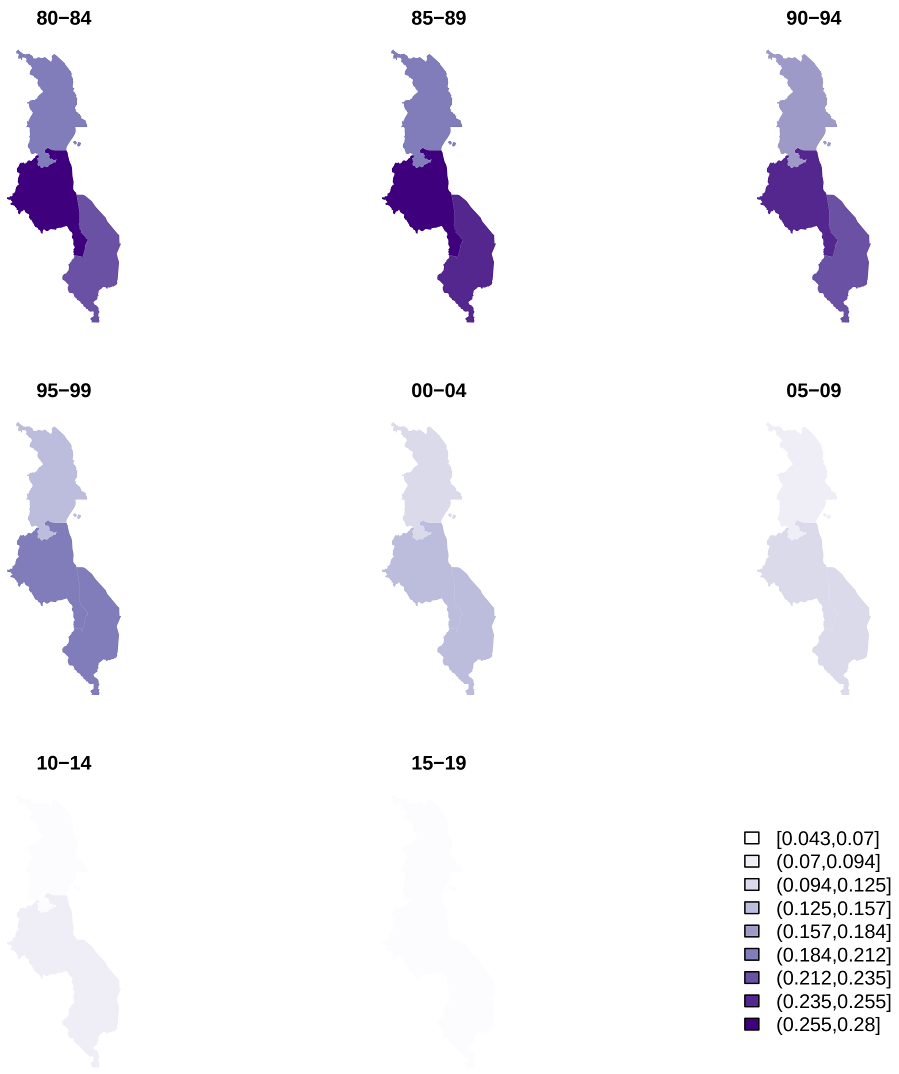

Figure S1.188: Malawi: Maps of posterior medians over time.

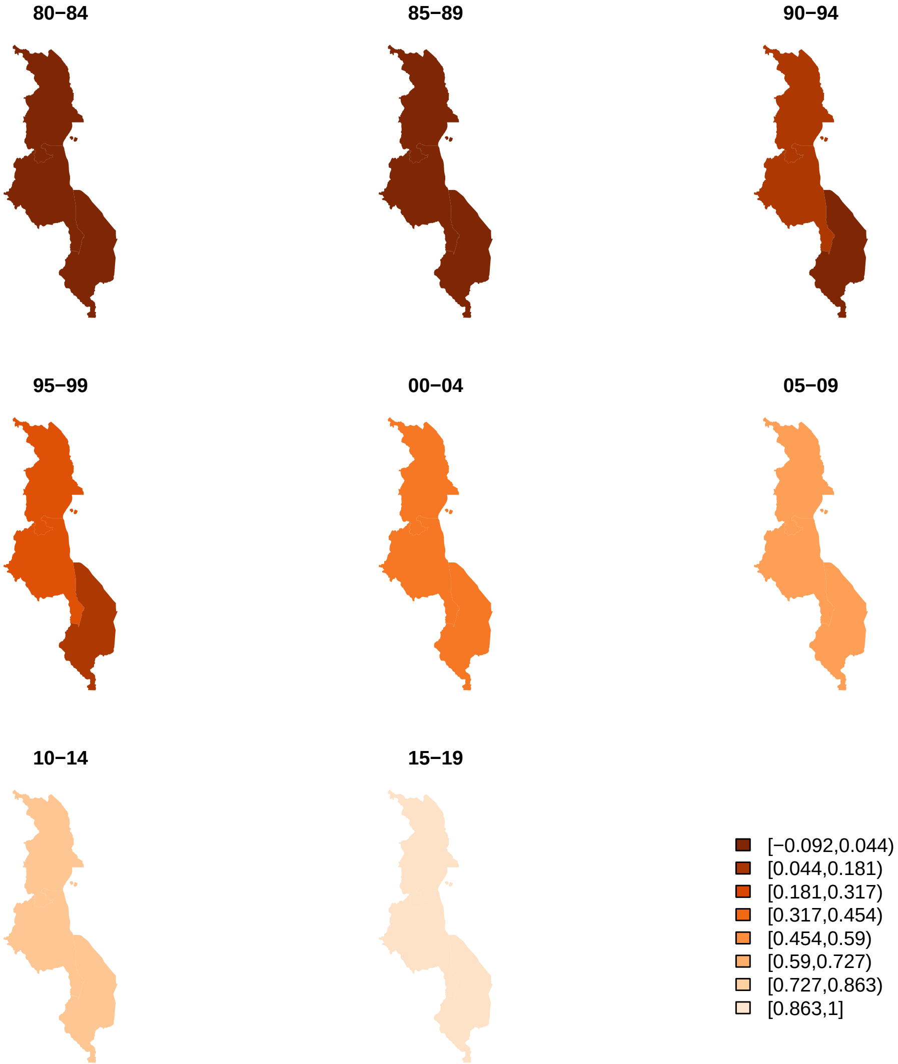

Figure S1.189: Malawi: Maps of reduction of posterior median U5MR in each five-year period compared to 1990 over time.

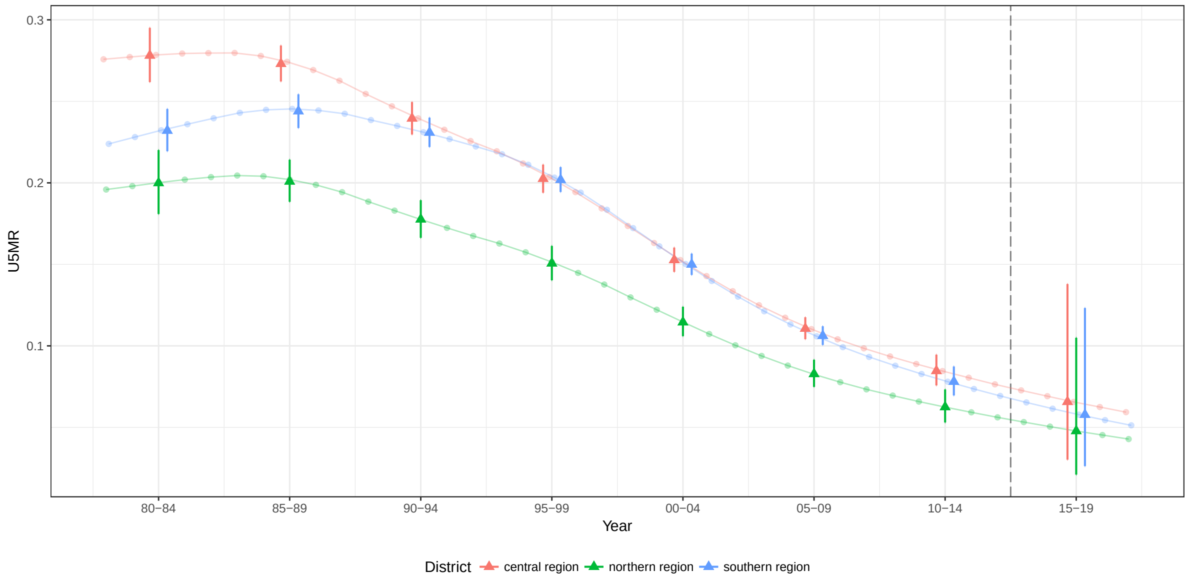

Figure S1.190: Malawi: Smoothed regional estimates over time. The line indicates yearly posterior median estimates and error bars indicate 95 % posterior credible interval at each time period.

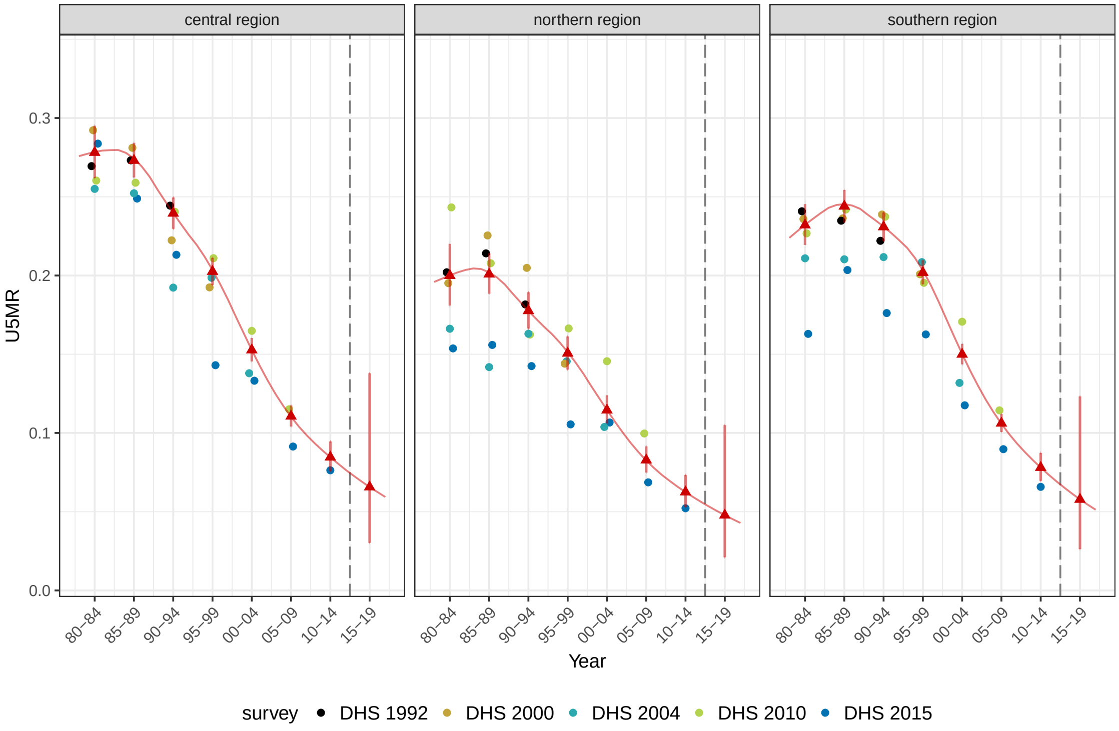

Figure S1.191: Malawi: Smoothed regional estimates over time compared to the direct estimates from each surveys. Direct estimates are not benchmarked with UN estimates. The line indicates posterior median and error bars indicate 95% posterior credible interval.

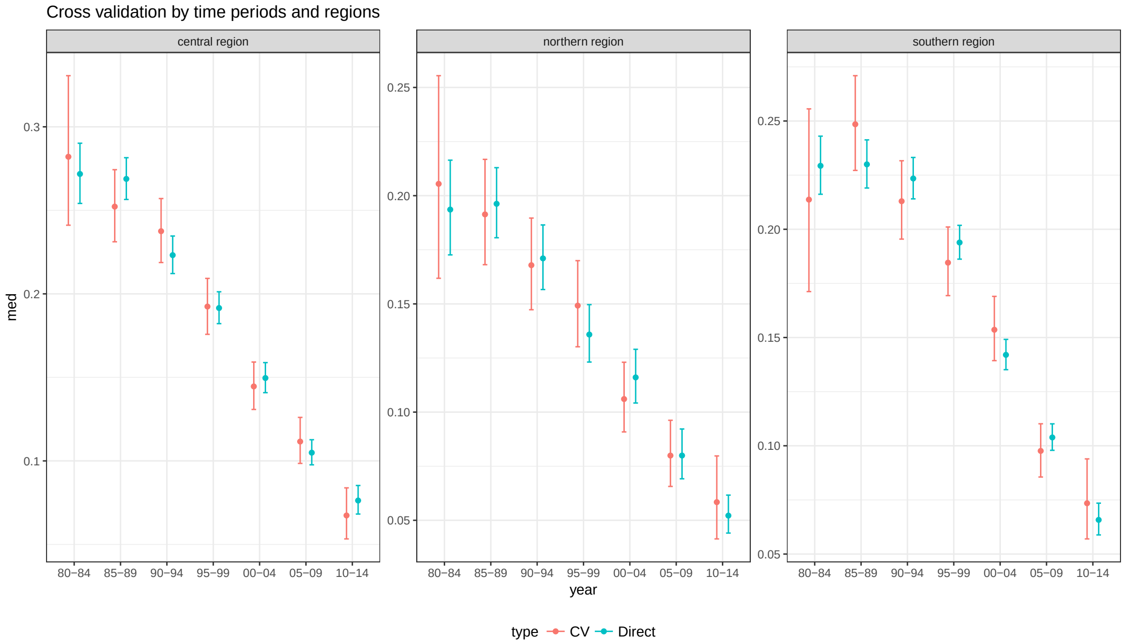

Figure S1.192: Malawi: Out-of-sample predictions along with direct estimates in the cross validation study where data from one region in each time period is held out and predicted using the rest of the data.

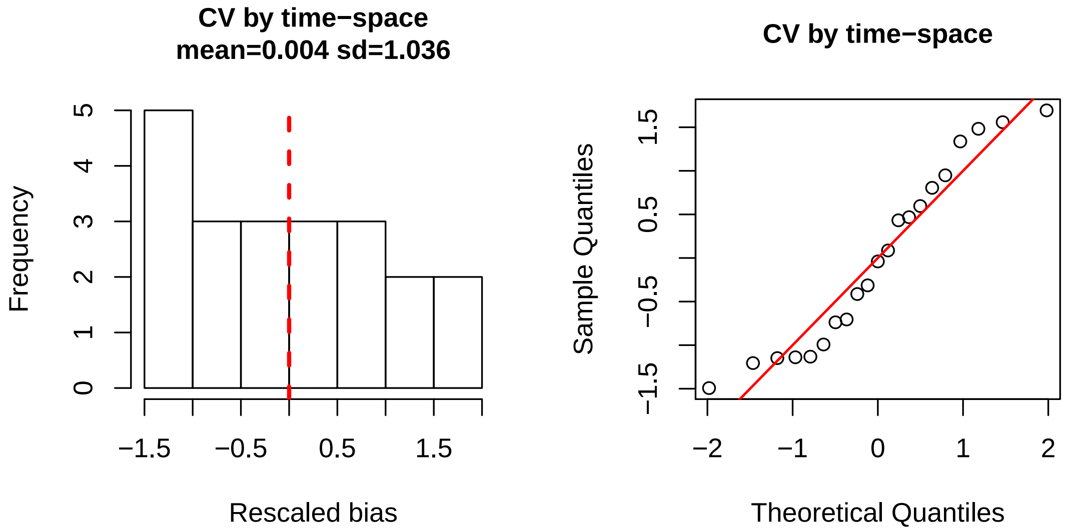

Figure S1.193: Malawi: Histogram and QQ-plot of the rescaled difference between the smoothed estimates and the direct estimates in the cross validation study. The differences between the two estimates are rescaled by the square root of the total variance of the two estimates.

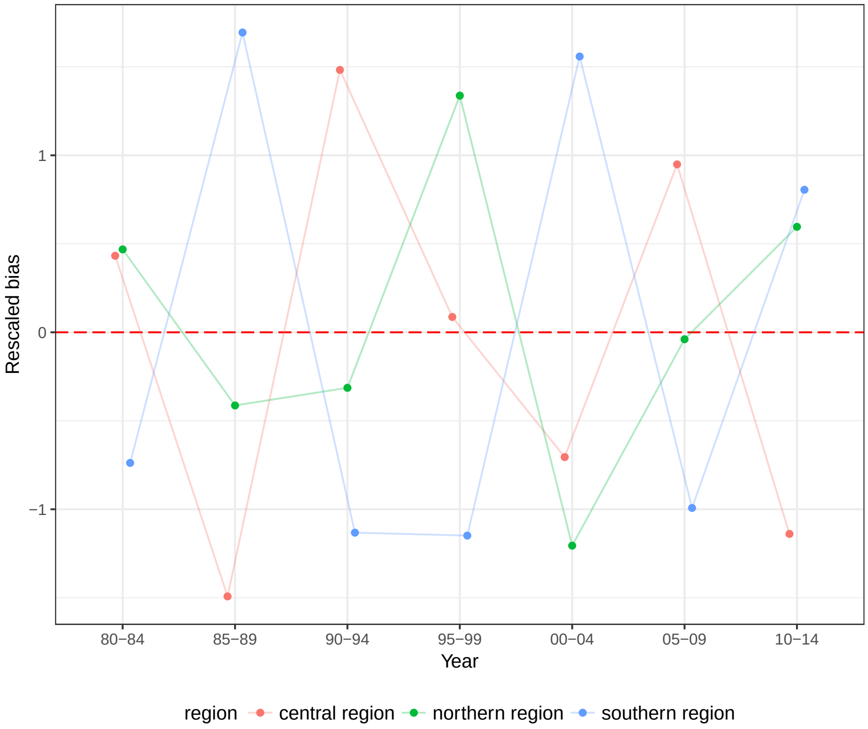

Figure S1.194: Malawi: Line plot of the difference between smoothed estimates and the direct estimates in the cross validation study. The differences between the two estimates are rescaled by the square root of the total variance of the two estimates.

3.5.22 Mali

DHS surveys were conducted in Mali in 1987, 1995, 2001, and 2006.

We fit both the RW2 only model to the combined national data, and compare the time trend at national level with the estimates produced by the UN and IHME in Figure S1.195. We then adjusted the combined national data to the UN estimates of U5MR, and refit the models on the benchmarked data.

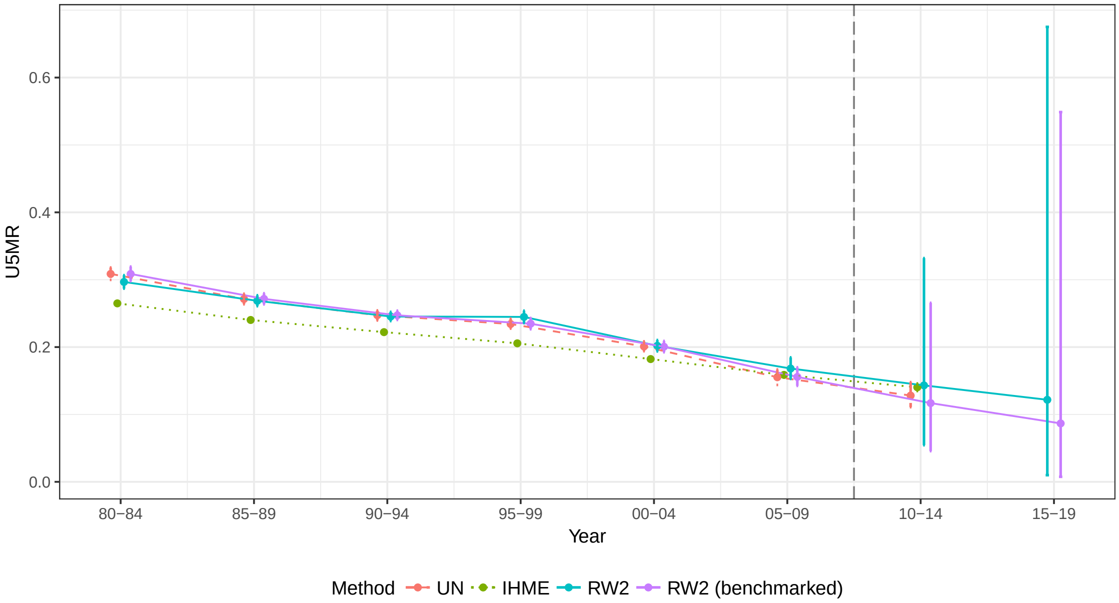

Figure S1.195: Mali: Temporal national trends along with UN (B3) estimates described in You et al. (2015) and IHME estimates based on GBD 2015 Child Mortality Collaborators (2016). RW2 represents the smoothed national estimates using the original data before benchmarking with UN estimates. RW2-adj represents the smoothed national estimates using the benchmarked data.

We fit the RW2 model to the benchmarked data in each area. We compare the results in Figure S1.196 to S1.200. Figure S1.196 compares the smoothed estimates against the direct estimates. Figure S1.197 and Figure S1.198 show the posterior median estimates of U5MR in each region over time and the reductions from 1990 period respectively. Figure S1.199 shows the smoothed estimates by region over time and Figure S1.200 compares the smoothed estimates with direct estimates from each survey for each region over time.

We further assess the RW2 model by holding out some observations, and compare the projections to the direct estimates in these holdout observations. Figure S1.201 compares the predicted estimates for the out-of-sample observations with the direct estimates by holding out observations from each area in each time period. Figure S1.202 compares the histogram of the bias rescaled by the total variance in the cross validation studies. Figure S1.203 compares the rescaled bias by region and time periods.

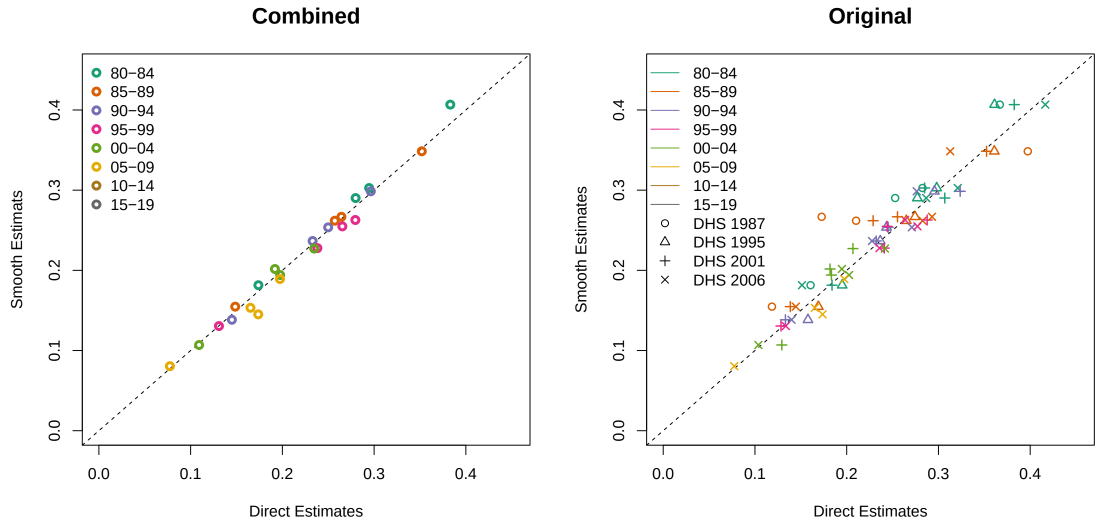

Figure S1.196: Mali: Smooth versus direct Admin 1 estimates. Left: Combined (meta-analysis) survey estimate against combined direct estimates. Right: Combined (meta-analysis) survey estimate against direct estimates from each survey.

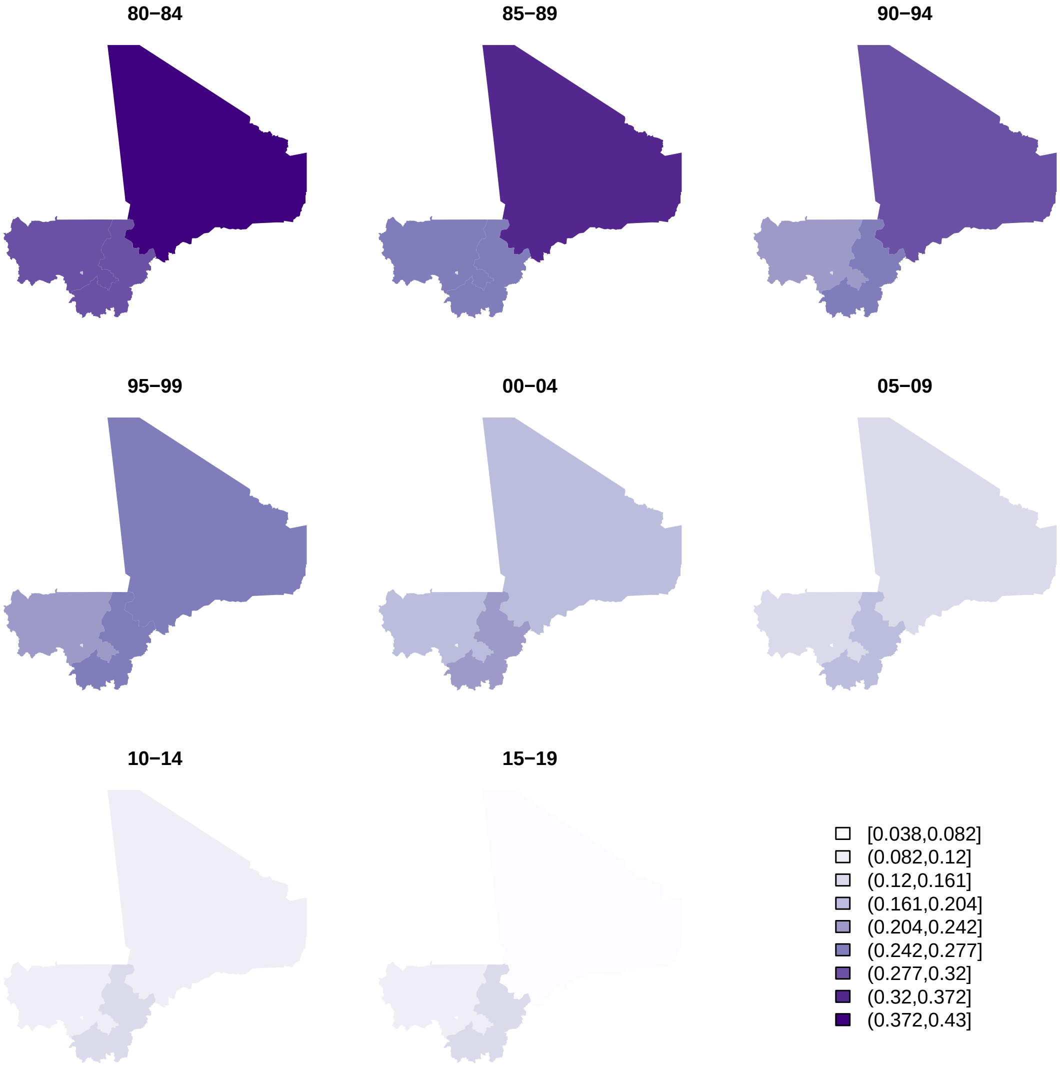

Figure S1.197: Mali: Maps of posterior medians over time.

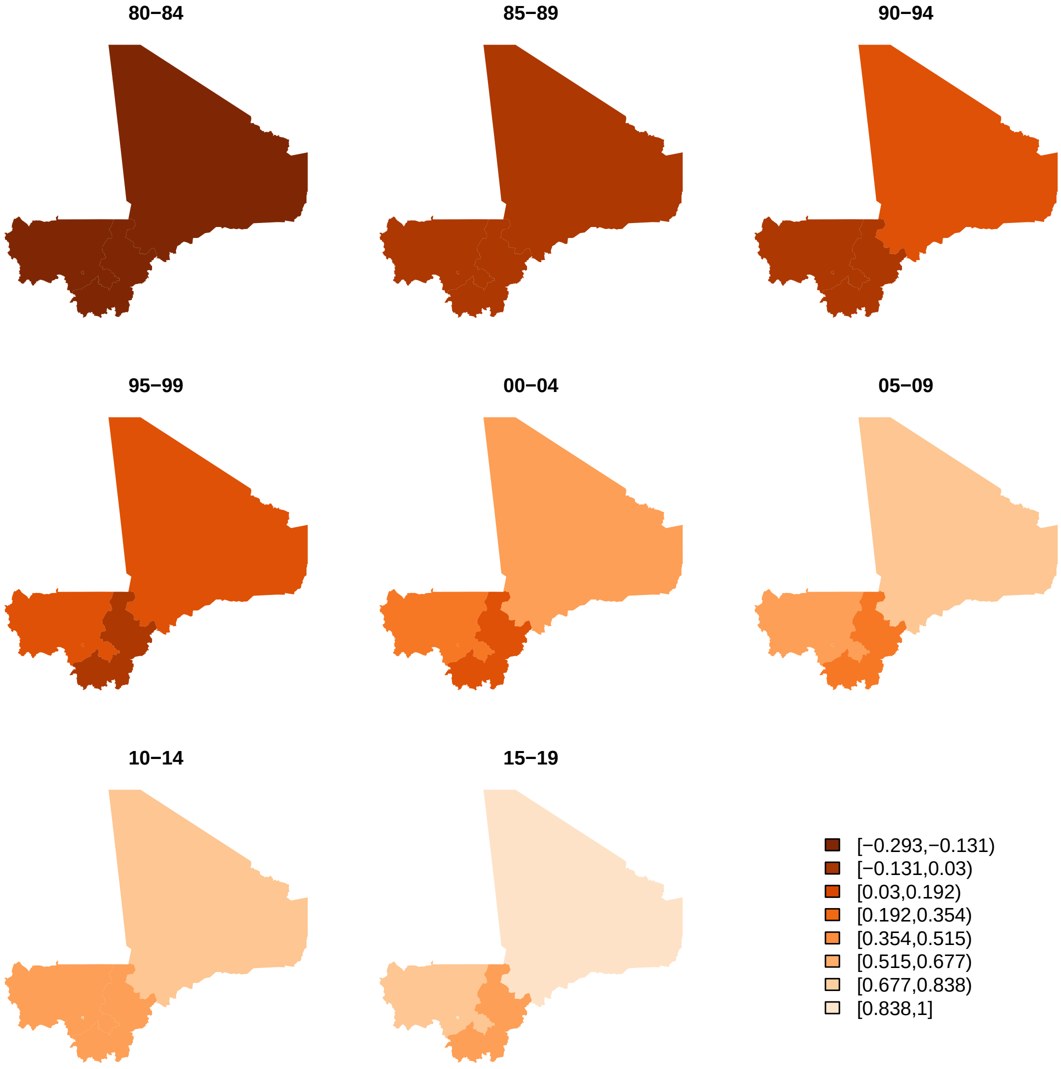

Figure S1.198: Mali: Maps of reduction of posterior median U5MR in each five-year period compared to 1990 over time.

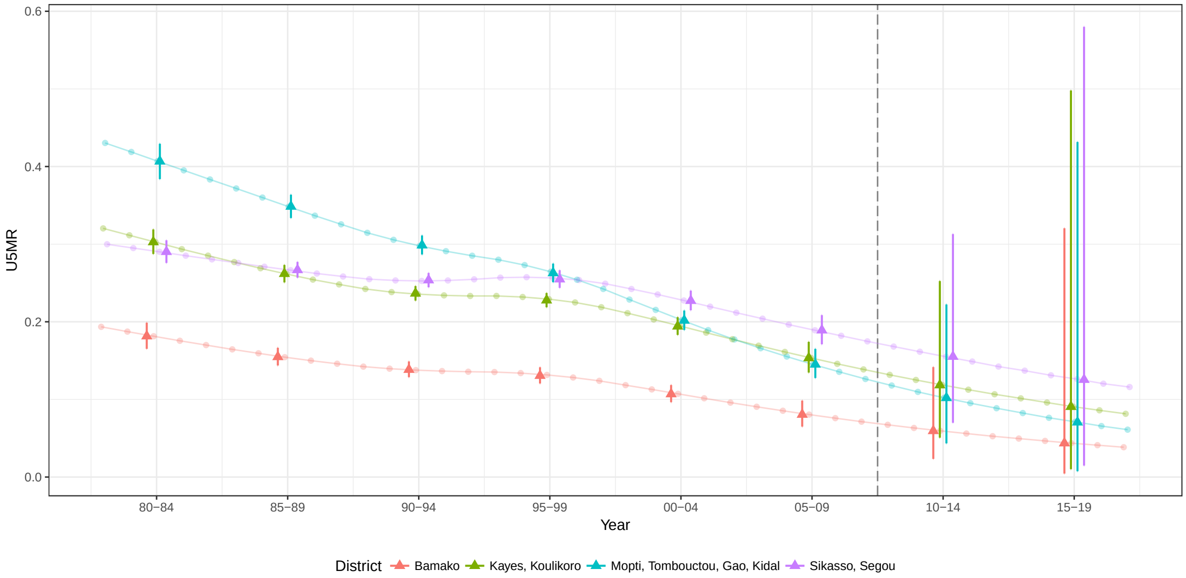

Figure S1.199: Mali: Smoothed regional estimates over time. The line indicates yearly posterior median estimates and error bars indicate 95 % posterior credible interval at each time period.

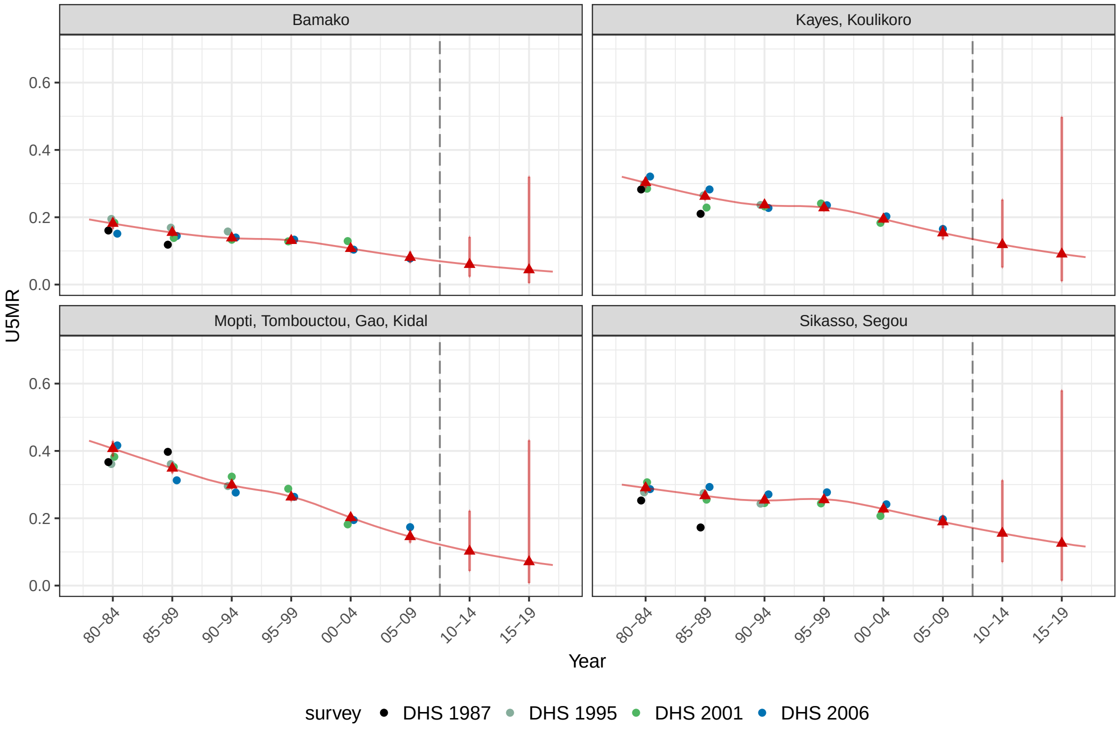

Figure S1.200: Mali: Smoothed regional estimates over time compared to the direct estimates from each surveys. Direct estimates are not benchmarked with UN estimates. The line indicates posterior median and error bars indicate 95% posterior credible interval.

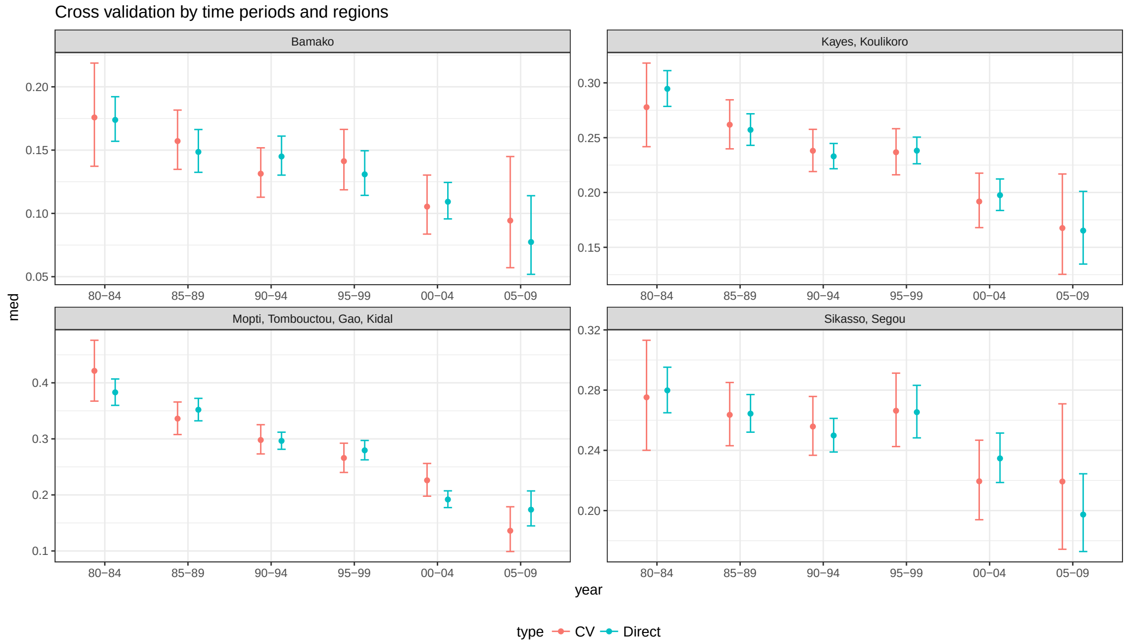

Figure S1.201: Mali: Out-of-sample predictions along with direct estimates in the cross validation study where data from one region in each time period is held out and predicted using the rest of the data.

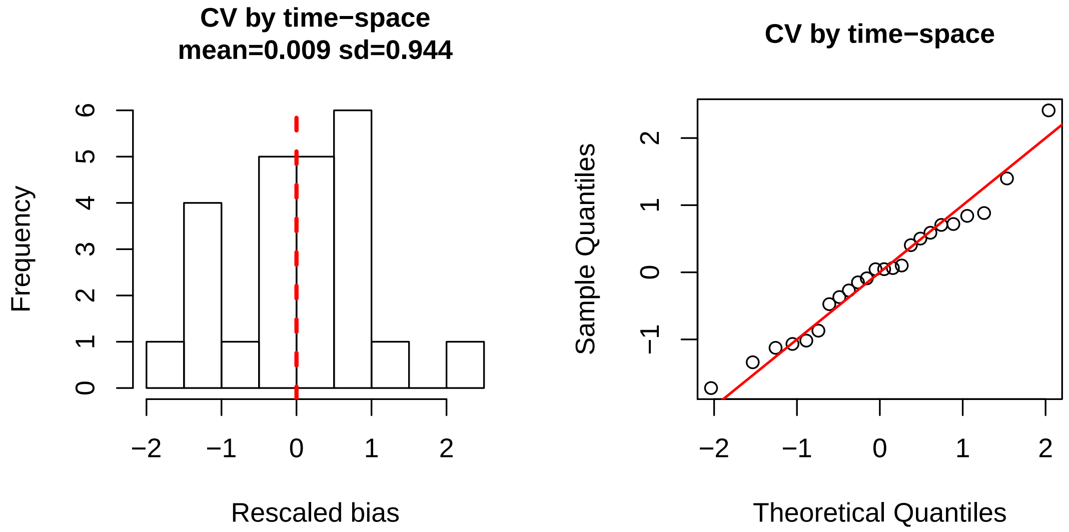

Figure S1.202: Mali: Histogram and QQ-plot of the rescaled difference between the smoothed estimates and the direct estimates in the cross validation study. The differences between the two estimates are rescaled by the square root of the total variance of the two estimates.

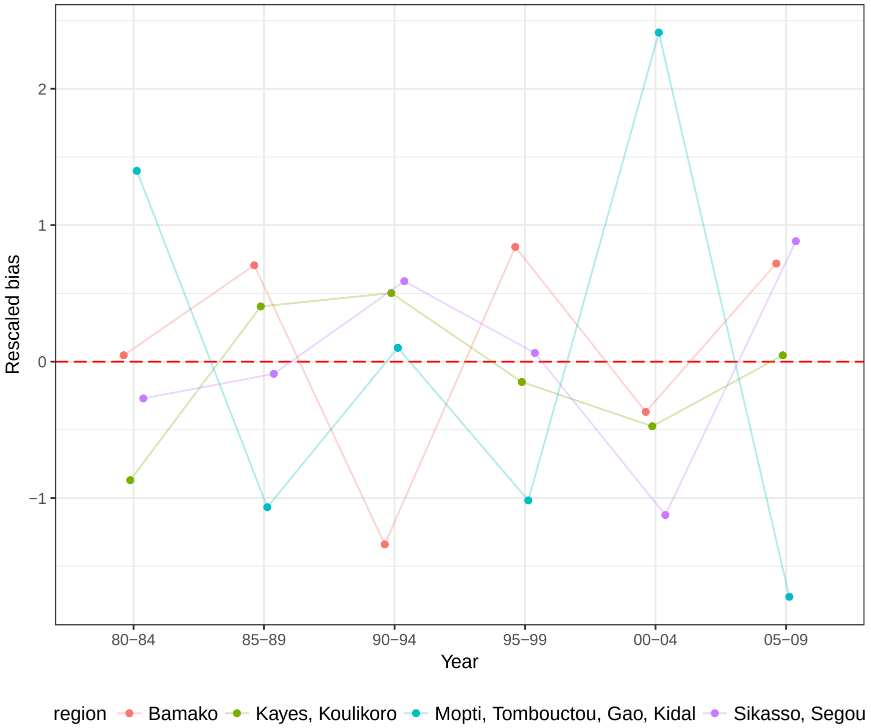

Figure S1.203: Mali: Line plot of the difference between smoothed estimates and the direct estimates in the cross validation study. The differences between the two estimates are rescaled by the square root of the total variance of the two estimates.

3.5.23 Morocco

DHS surveys were conducted in Morocco in 1987, 1992, and 2003.

We fit both the RW2 only model to the combined national data, and compare the time trend at national level with the estimates produced by the UN and IHME in Figure S1.204. We then adjusted the combined national data to the UN estimates of U5MR, and refit the models on the benchmarked data.

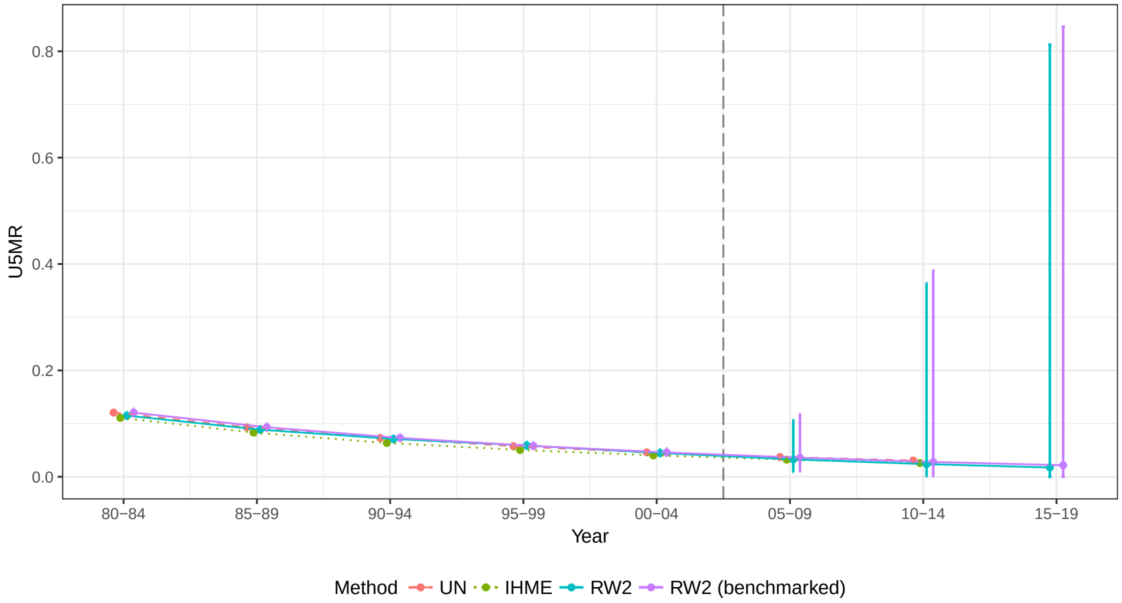

Figure S1.204: Morocco: Temporal national trends along with UN (B3) estimates described in You et al. (2015) and IHME estimates based on GBD 2015 Child Mortality Collaborators (2016). RW2 represents the smoothed national estimates using the original data before benchmarking with UN estimates. RW2-adj represents the smoothed national estimates using the benchmarked data.

We fit the RW2 model to the benchmarked data in each area. We compare the results in Figure S1.205 to S1.209. Figure S1.205 compares the smoothed estimates against the direct estimates. Figure S1.206 and Figure S1.207 show the posterior median estimates of U5MR in each region over time and the reductions from 1990 period respectively. Figure S1.208 shows the smoothed estimates by region over time and Figure S1.209 compares the smoothed estimates with direct estimates from each survey for each region over time.

We further assess the RW2 model by holding out some observations, and compare the projections to the direct estimates in these holdout observations. Figure S1.210 compares the predicted estimates for the out-of-sample observations with the direct estimates by holding out observations from each area in each time period. Figure S1.211 compares the histogram of the bias rescaled by the total variance in the cross validation studies. Figure S1.212 compares the rescaled bias by region and time periods.

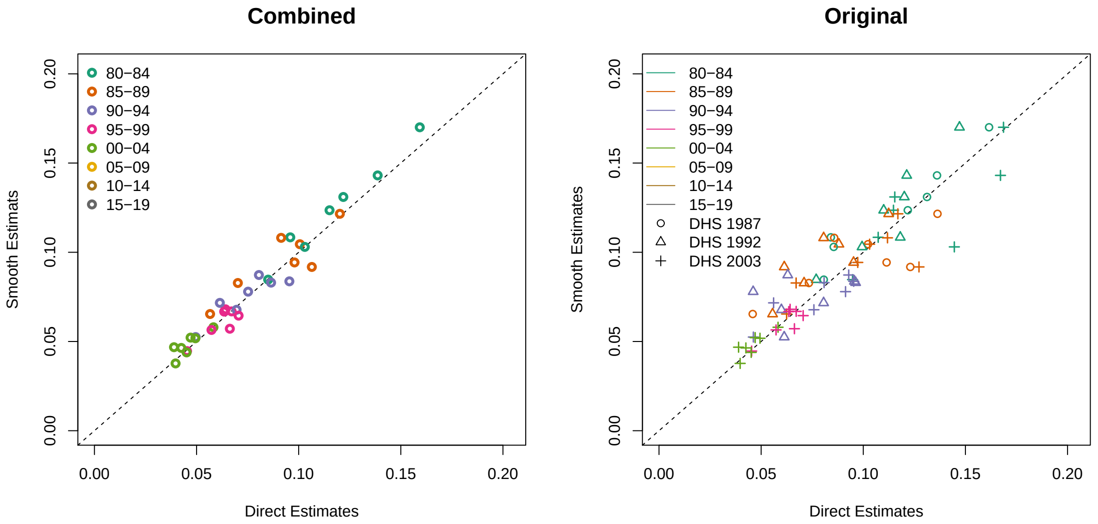

Figure S1.205: Morocco: Smooth versus direct Admin 1 estimates. Left: Combined (meta-analysis) survey estimate against combined direct estimates. Right: Combined (meta-analysis) survey estimate against direct estimates from each survey.

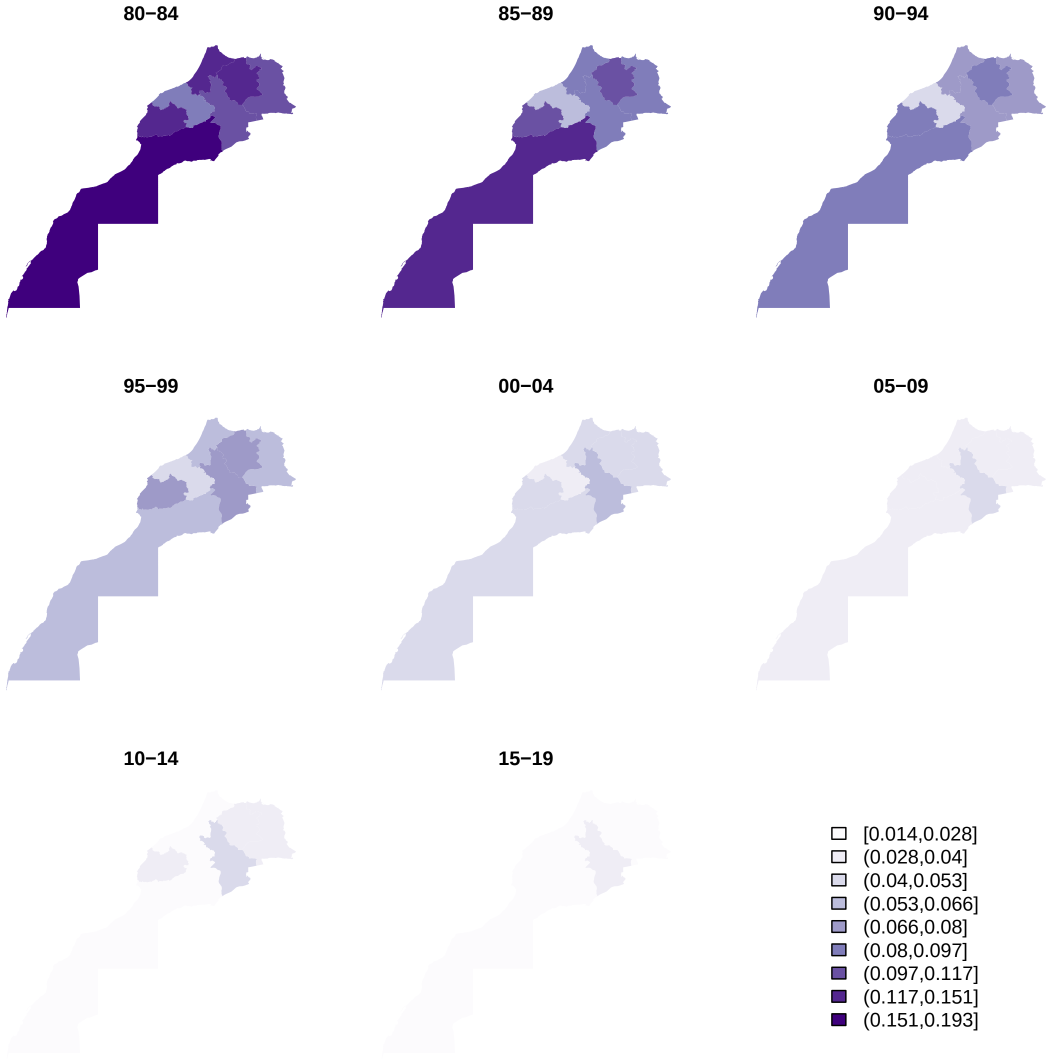

Figure S1.206: Morocco: Maps of posterior medians over time.

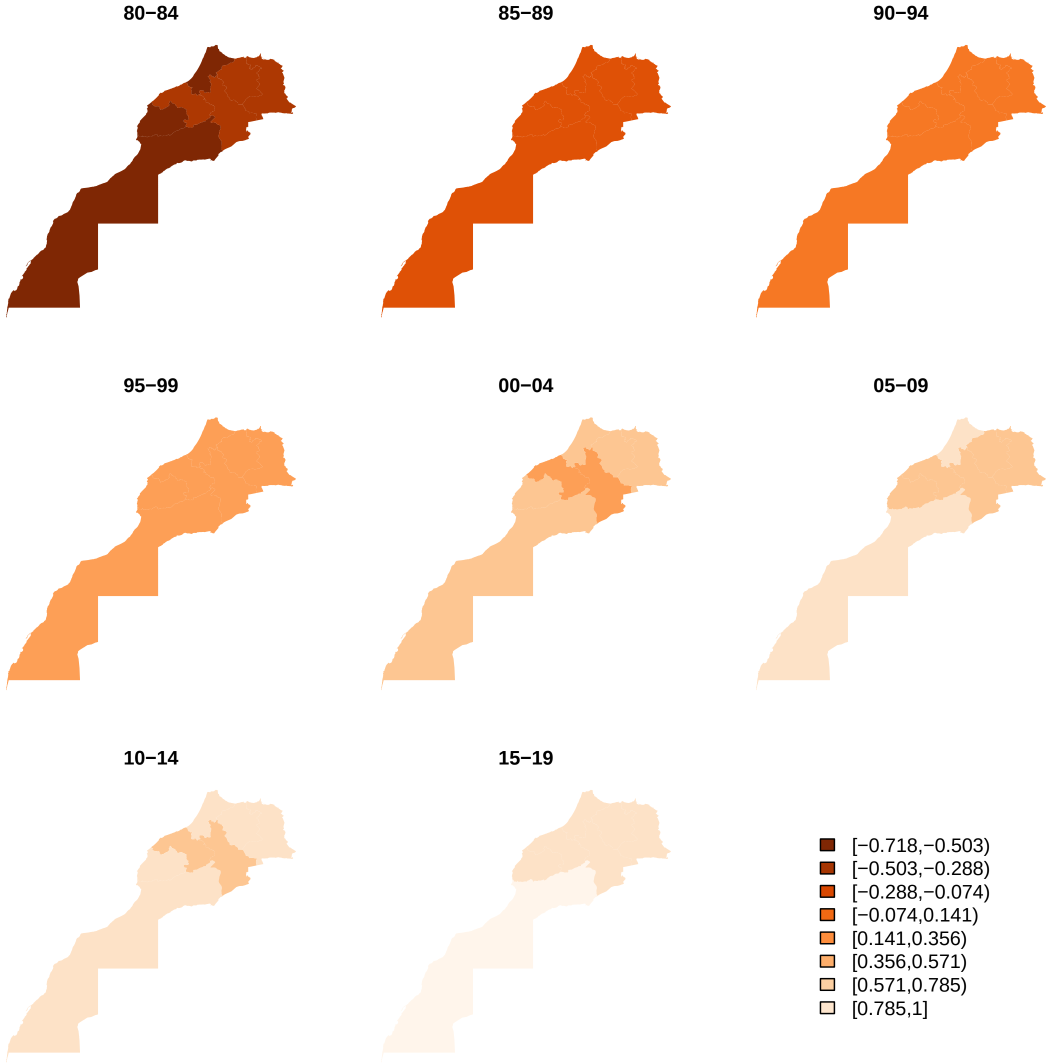

Figure S1.207: Morocco: Maps of reduction of posterior median U5MR in each five-year period compared to 1990 over time.

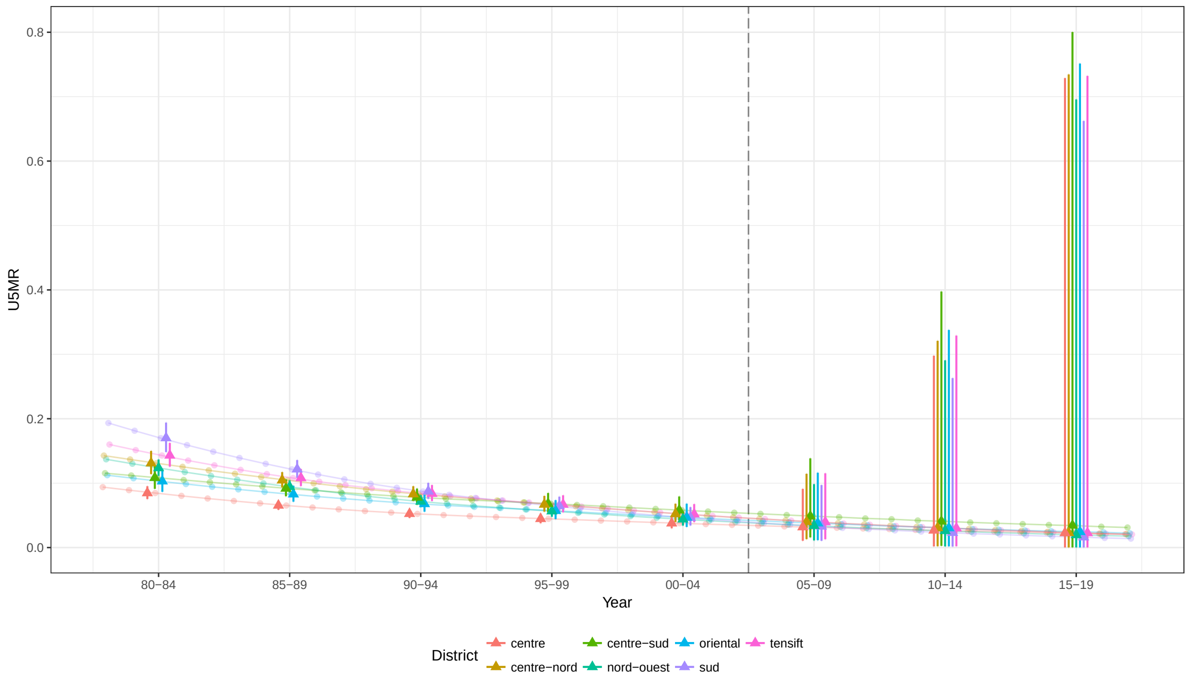

Figure S1.208: Morocco: Smoothed regional estimates over time. The line indicates yearly posterior median estimates and error bars indicate 95 % posterior credible interval at each time period.

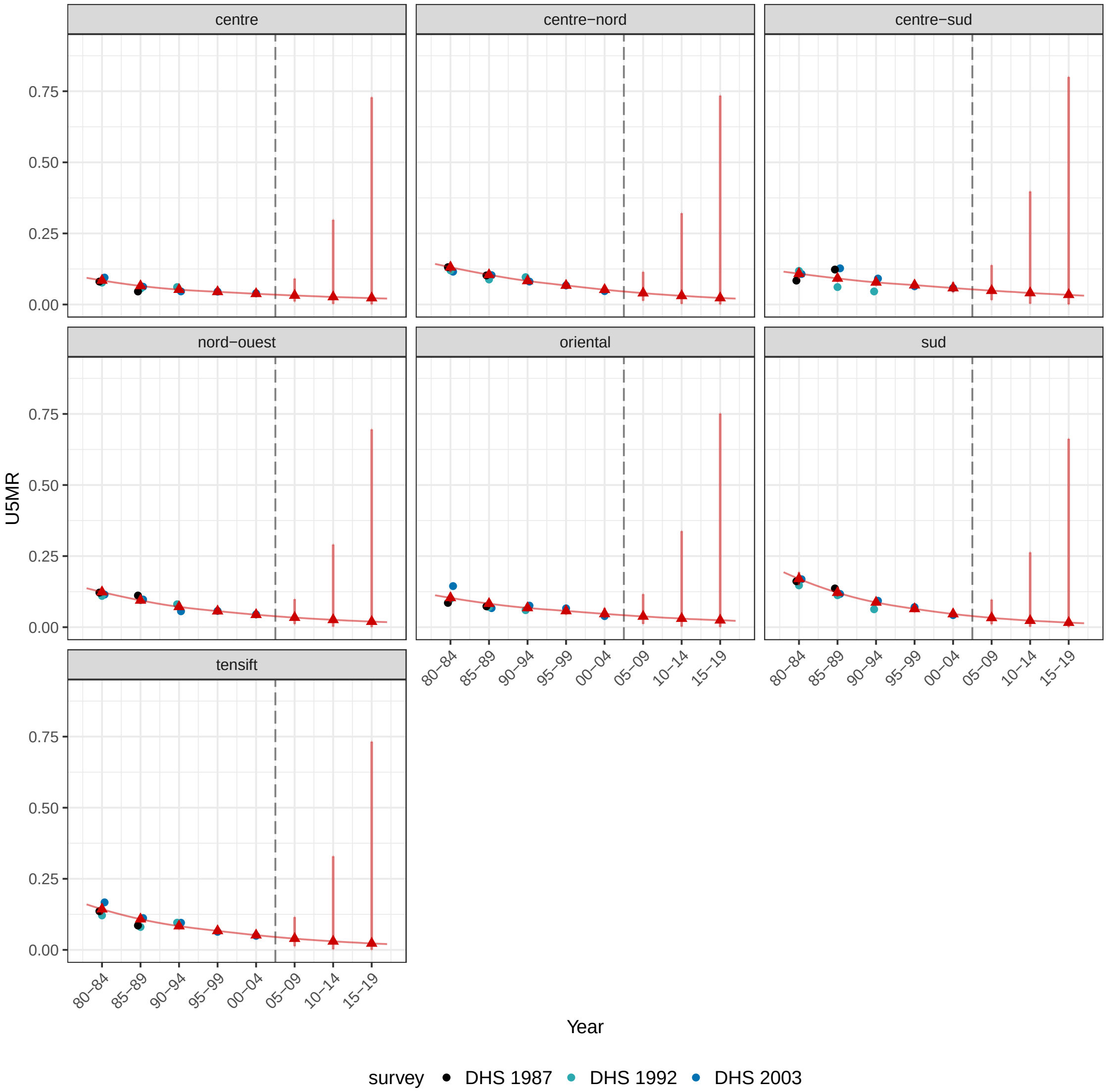

Figure S1.209: Morocco: Smoothed regional estimates over time compared to the direct estimates from each surveys. Direct estimates are not benchmarked with UN estimates. The line indicates posterior median and error bars indicate 95% posterior credible interval.

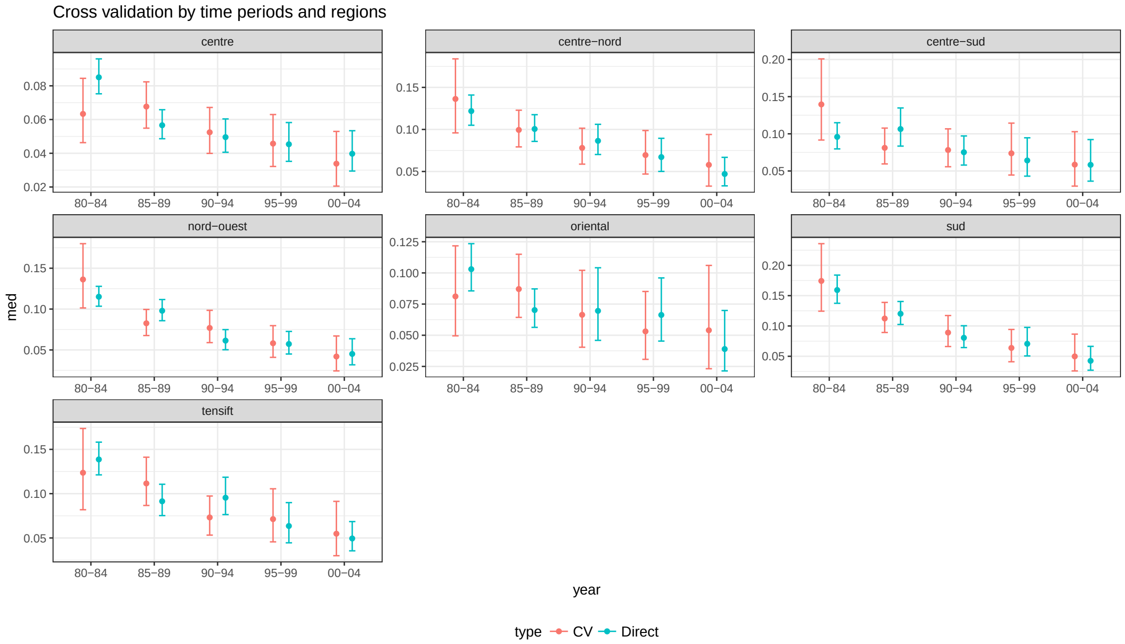

Figure S1.210: Morocco: Out-of-sample predictions along with direct estimates in the cross validation study where data from one region in each time period is held out and predicted using the rest of the data.

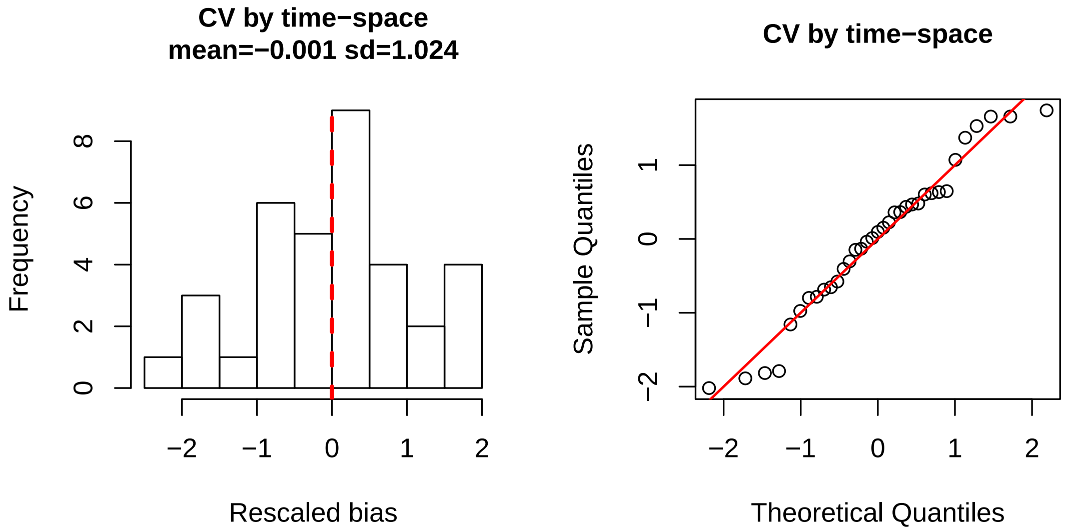

Figure S1.211: Morocco: Histogram and QQ-plot of the rescaled difference between the smoothed estimates and the direct estimates in the cross validation study. The differences between the two estimates are rescaled by the square root of the total variance of the two estimates.

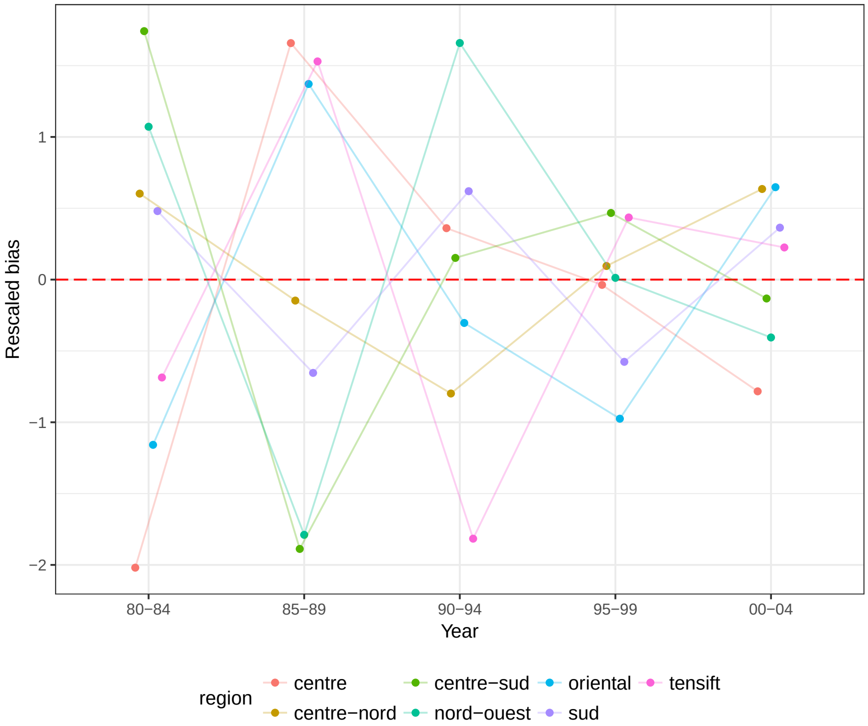

Figure S1.212: Morocco: Line plot of the difference between smoothed estimates and the direct estimates in the cross validation study. The differences between the two estimates are rescaled by the square root of the total variance of the two estimates.

3.5.24 Mozambique

DHS surveys were conducted in Mozambique in 2003, and 2011.

We fit both the RW2 only model to the combined national data, and compare the time trend at national level with the estimates produced by the UN and IHME in Figure S1.213. We then adjusted the combined national data to the UN estimates of U5MR, and refit the models on the benchmarked data.

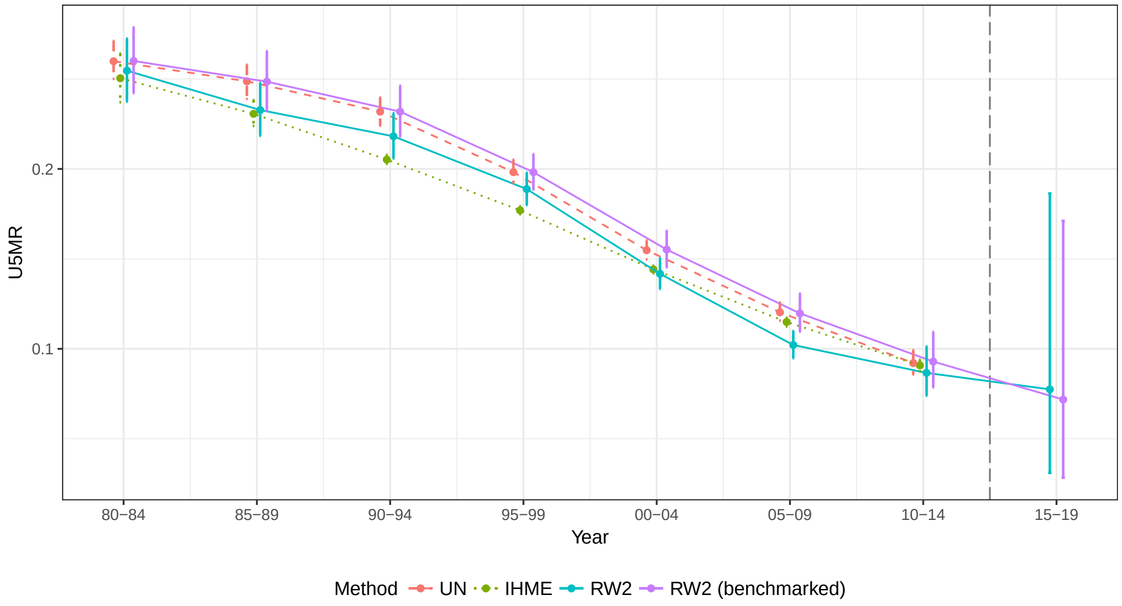

Figure S1.213: Mozambique: Temporal national trends along with UN (B3) estimates described in You et al. (2015) and IHME estimates based on GBD 2015 Child Mortality Collaborators (2016). RW2 represents the smoothed national estimates using the original data before benchmarking with UN estimates. RW2-adj represents the smoothed national estimates using the benchmarked data.

We fit the RW2 model to the benchmarked data in each area. We compare the results in Figure S1.214 to S1.218. Figure S1.214 compares the smoothed estimates against the direct estimates. Figure S1.215 and Figure S1.216 show the posterior median estimates of U5MR in each region over time and the reductions from 1990 period respectively. Figure S1.217 shows the smoothed estimates by region over time and Figure S1.218 compares the smoothed estimates with direct estimates from each survey for each region over time.

We further assess the RW2 model by holding out some observations, and compare the projections to the direct estimates in these holdout observations. Figure S1.219 compares the predicted estimates for the out-of-sample observations with the direct estimates by holding out observations from each area in each time period. Figure S1.220 compares the histogram of the bias rescaled by the total variance in the cross validation studies. Figure S1.221 compares the rescaled bias by region and time periods.

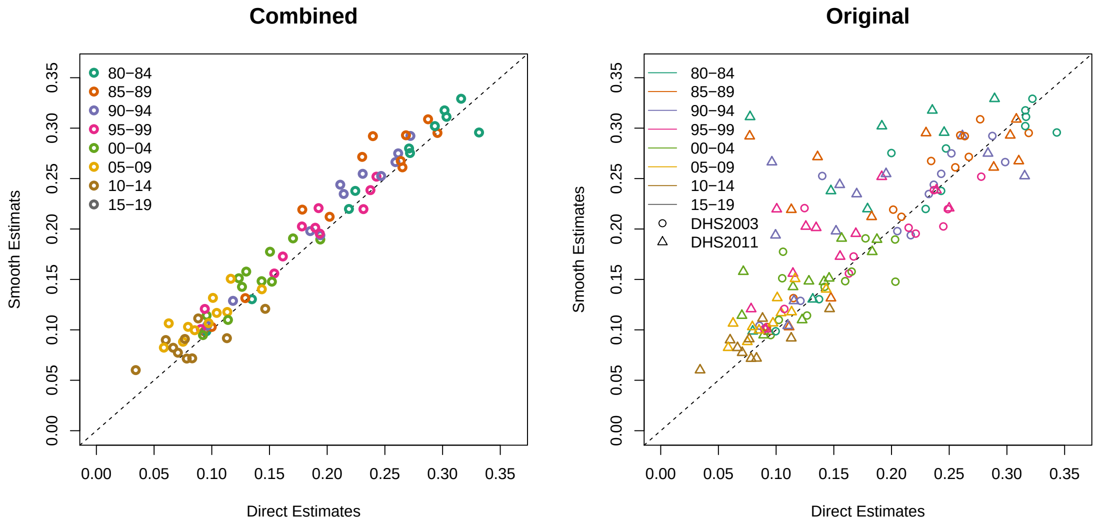

Figure S1.214: Mozambique: Smooth versus direct Admin 1 estimates. Left: Combined (meta-analysis) survey estimate against combined direct estimates. Right: Combined (meta-analysis) survey estimate against direct estimates from each survey.

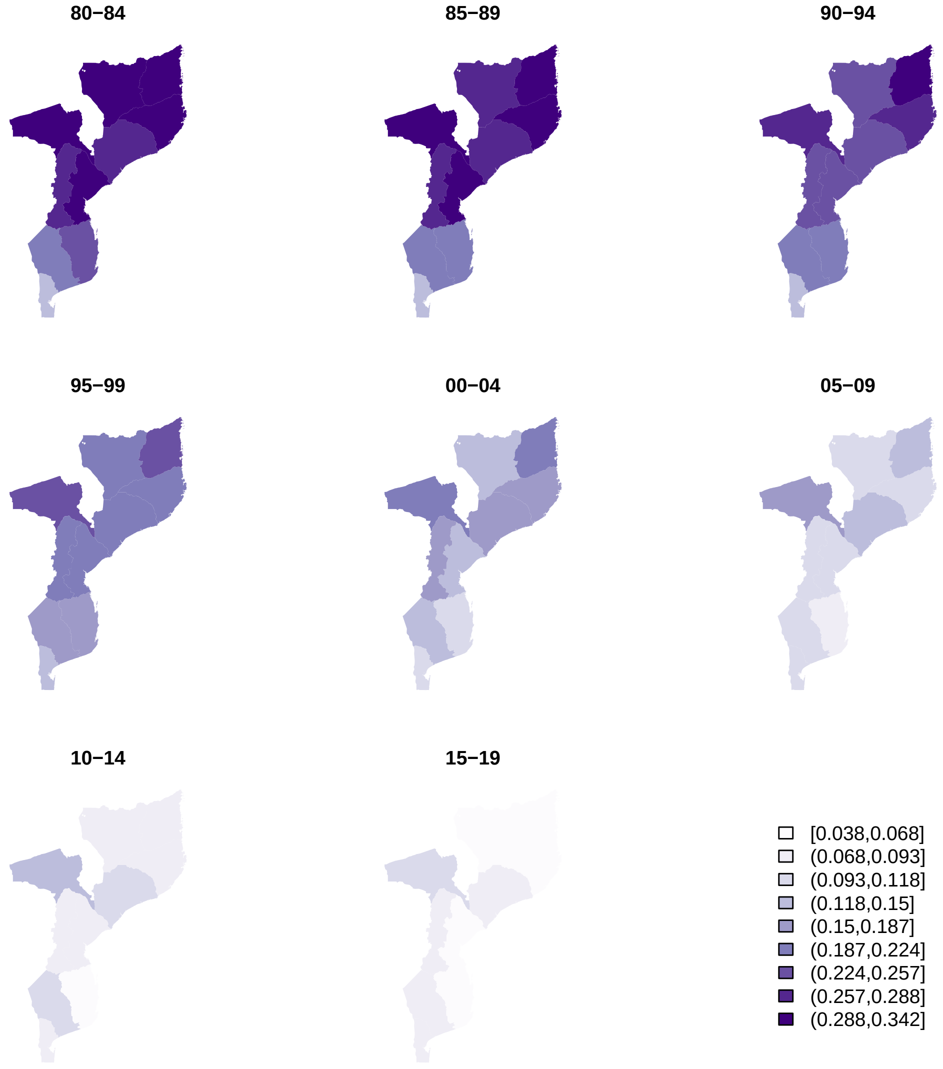

Figure S1.215: Mozambique: Maps of posterior medians over time.

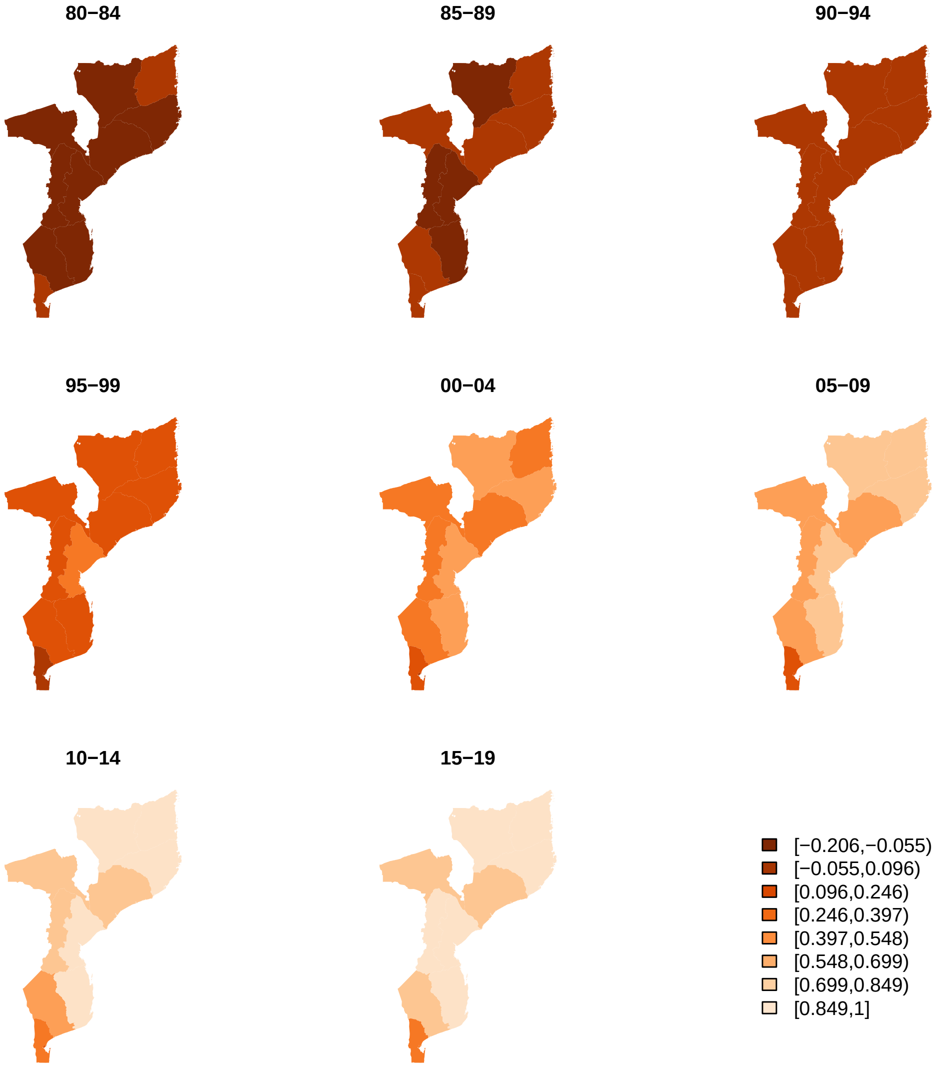

Figure S1.216: Mozambique: Maps of reduction of posterior median U5MR in each five-year period compared to 1990 over time.

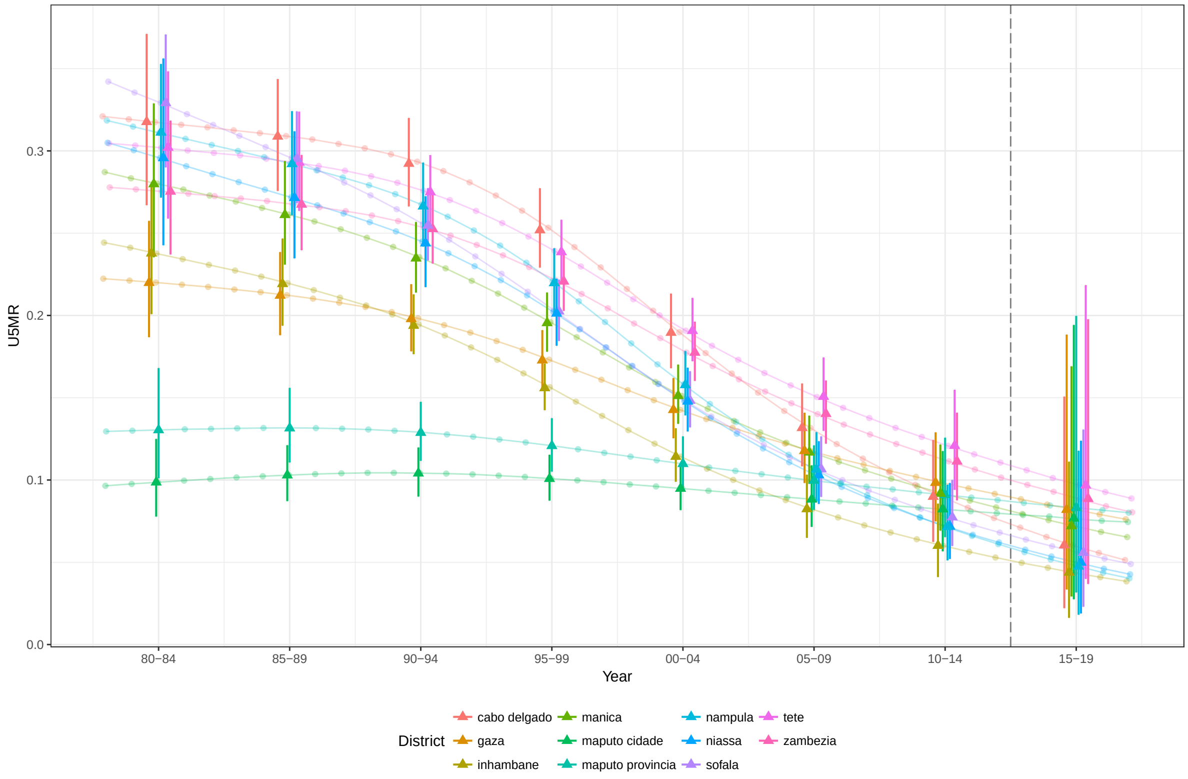

Figure S1.217: Mozambique: Smoothed regional estimates over time. The line indicates yearly posterior median estimates and error bars indicate 95 % posterior credible interval at each time period.

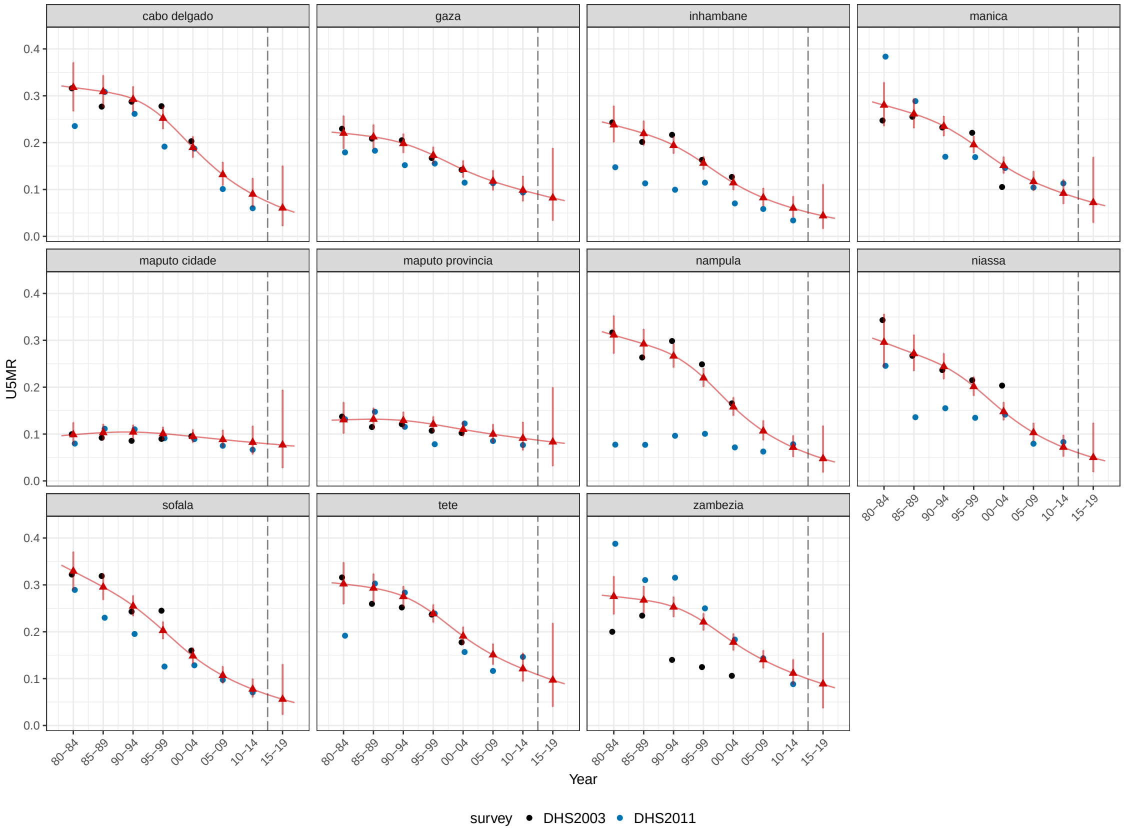

Figure S1.218: Mozambique: Smoothed regional estimates over time compared to the direct estimates from each surveys. Direct estimates are not benchmarked with UN estimates. The line indicates posterior median and error bars indicate 95% posterior credible interval.

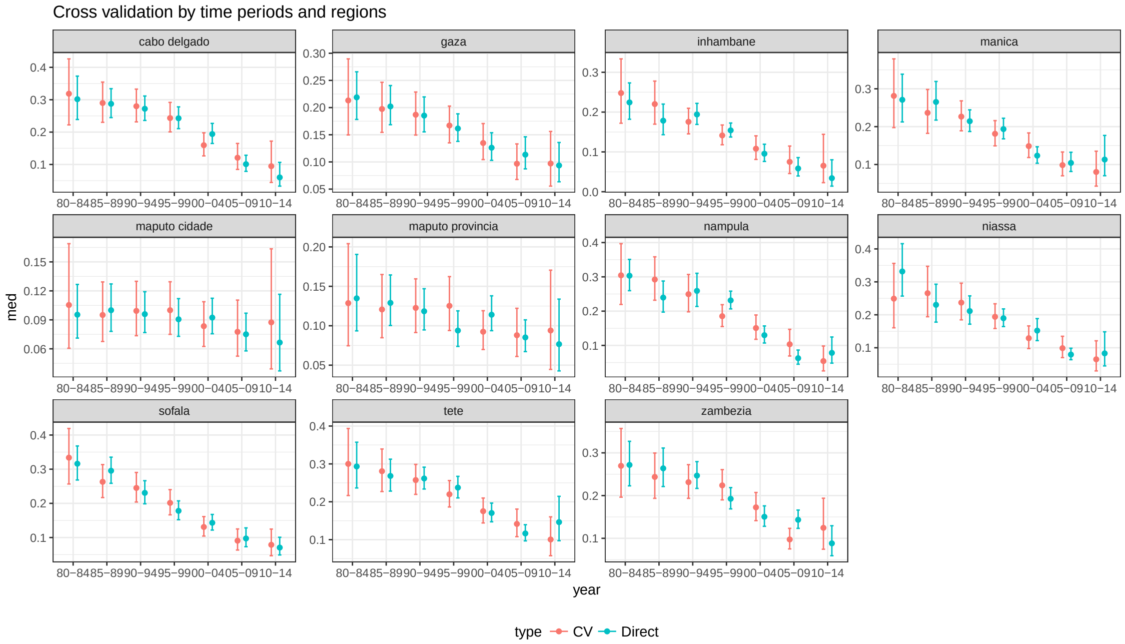

Figure S1.219: Mozambique: Out-of-sample predictions along with direct estimates in the cross validation study where data from one region in each time period is held out and predicted using the rest of the data.

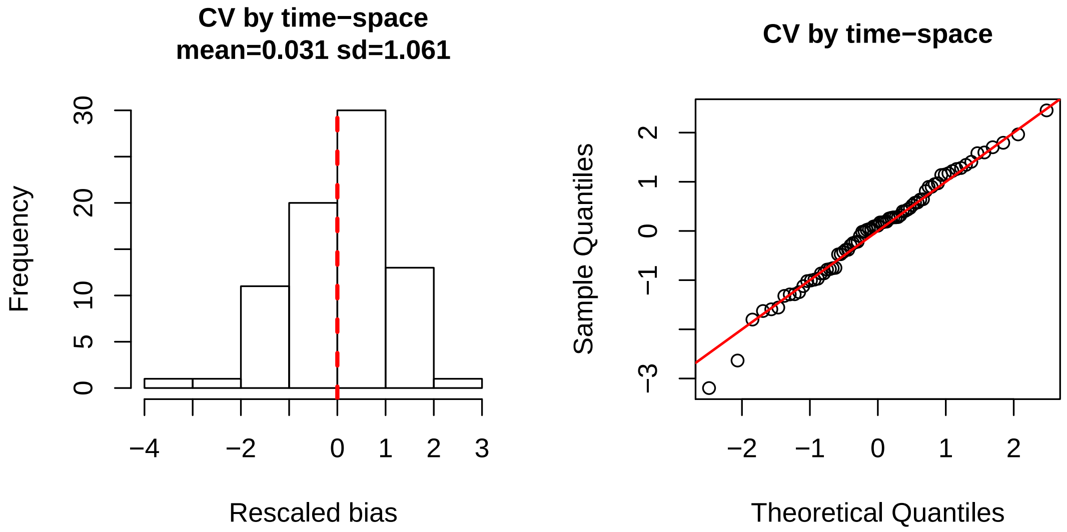

Figure S1.220: Mozambique: Histogram and QQ-plot of the rescaled difference between the smoothed estimates and the direct estimates in the cross validation study. The differences between the two estimates are rescaled by the square root of the total variance of the two estimates.

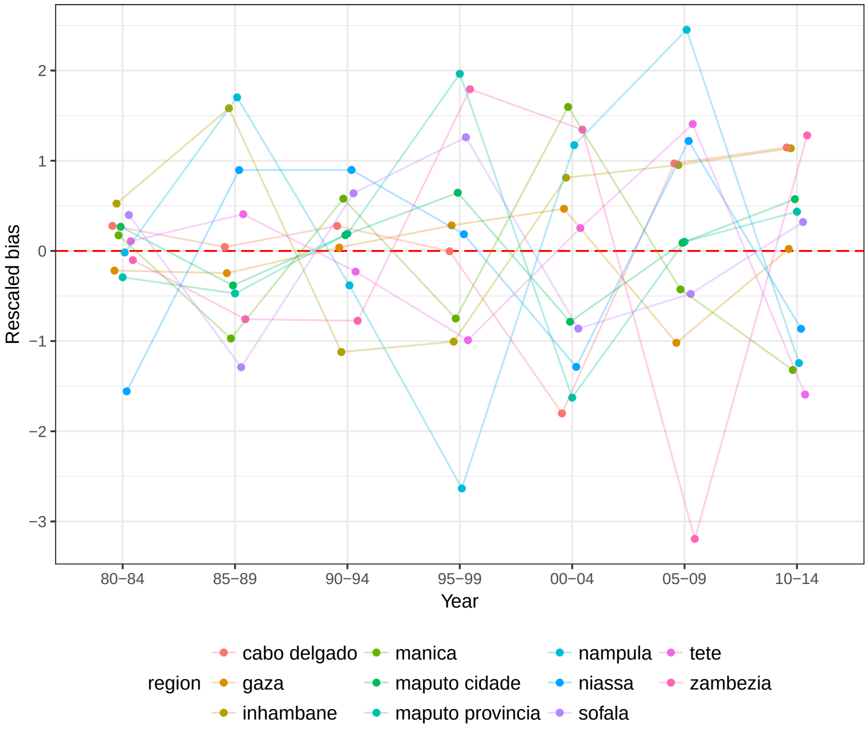

Figure S1.221: Mozambique: Line plot of the difference between smoothed estimates and the direct estimates in the cross validation study. The differences between the two estimates are rescaled by the square root of the total variance of the two estimates.

3.5.25 Namibia

DHS surveys were conducted in Namibia in 2000, 2007, and 2013.

We fit both the RW2 only model to the combined national data, and compare the time trend at national level with the estimates produced by the UN and IHME in Figure S1.222. We then adjusted the combined national data to the UN estimates of U5MR, and refit the models on the benchmarked data.

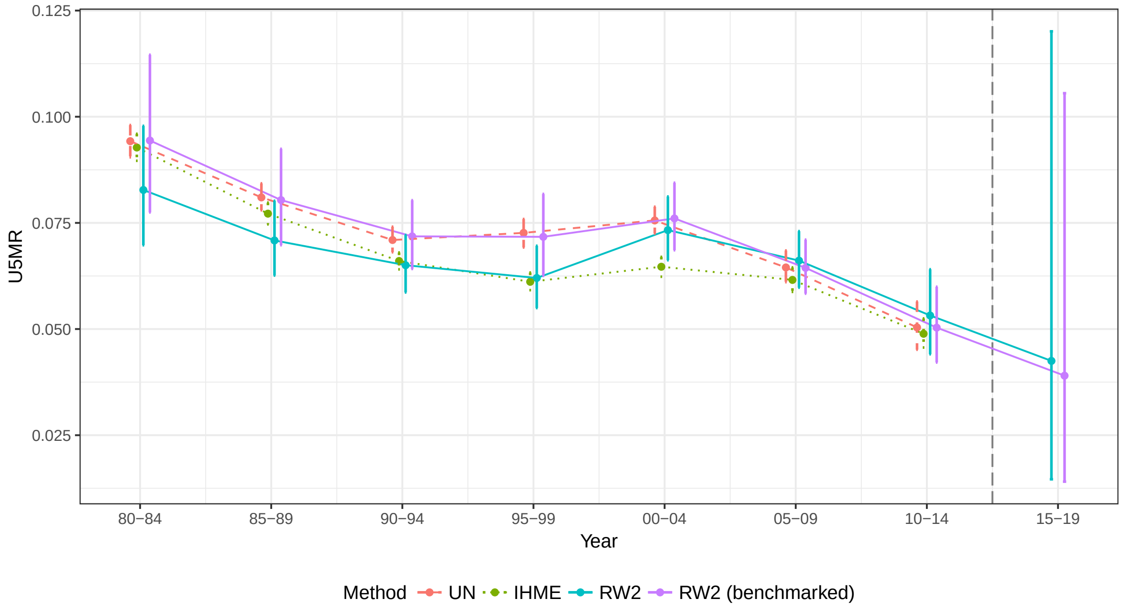

Figure S1.222: Namibia: Temporal national trends along with UN (B3) estimates described in You et al. (2015) and IHME estimates based on GBD 2015 Child Mortality Collaborators (2016). RW2 represents the smoothed national estimates using the original data before benchmarking with UN estimates. RW2-adj represents the smoothed national estimates using the benchmarked data.

We fit the RW2 model to the benchmarked data in each area. We compare the results in Figure S1.223 to S1.227. Figure S1.223 compares the smoothed estimates against the direct estimates. Figure S1.224 and Figure S1.225 show the posterior median estimates of U5MR in each region over time and the reductions from 1990 period respectively. Figure S1.226 shows the smoothed estimates by region over time and Figure S1.227 compares the smoothed estimates with direct estimates from each survey for each region over time.

We further assess the RW2 model by holding out some observations, and compare the projections to the direct estimates in these holdout observations. Figure S1.228 compares the predicted estimates for the out-of-sample observations with the direct estimates by holding out observations from each area in each time period. Figure S1.229 compares the histogram of the bias rescaled by the total variance in the cross validation studies. Figure S1.230 compares the rescaled bias by region and time periods.

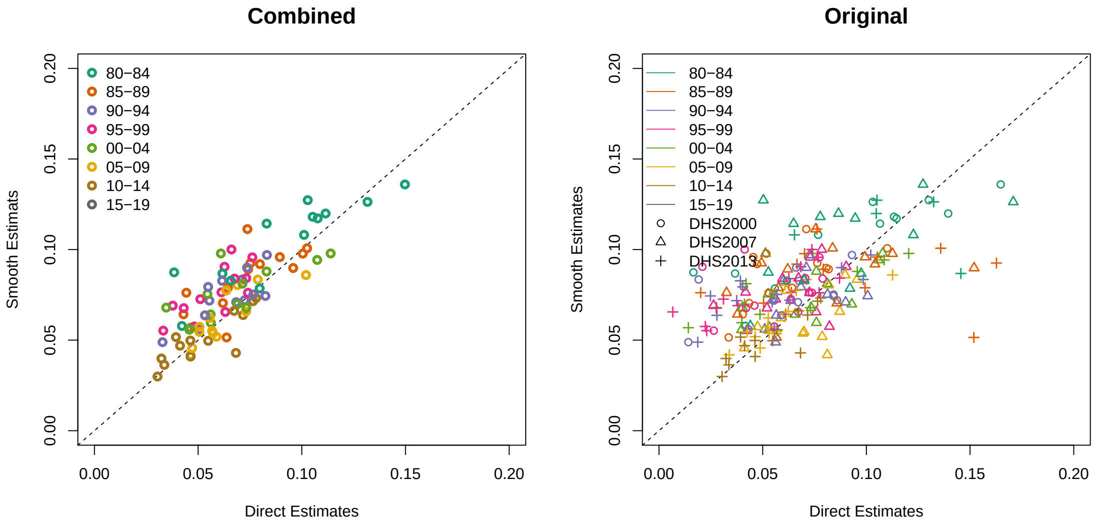

Figure S1.223: Namibia: Smooth versus direct Admin 1 estimates. Left: Combined (meta-analysis) survey estimate against combined direct estimates. Right: Combined (meta-analysis) survey estimate against direct estimates from each survey.

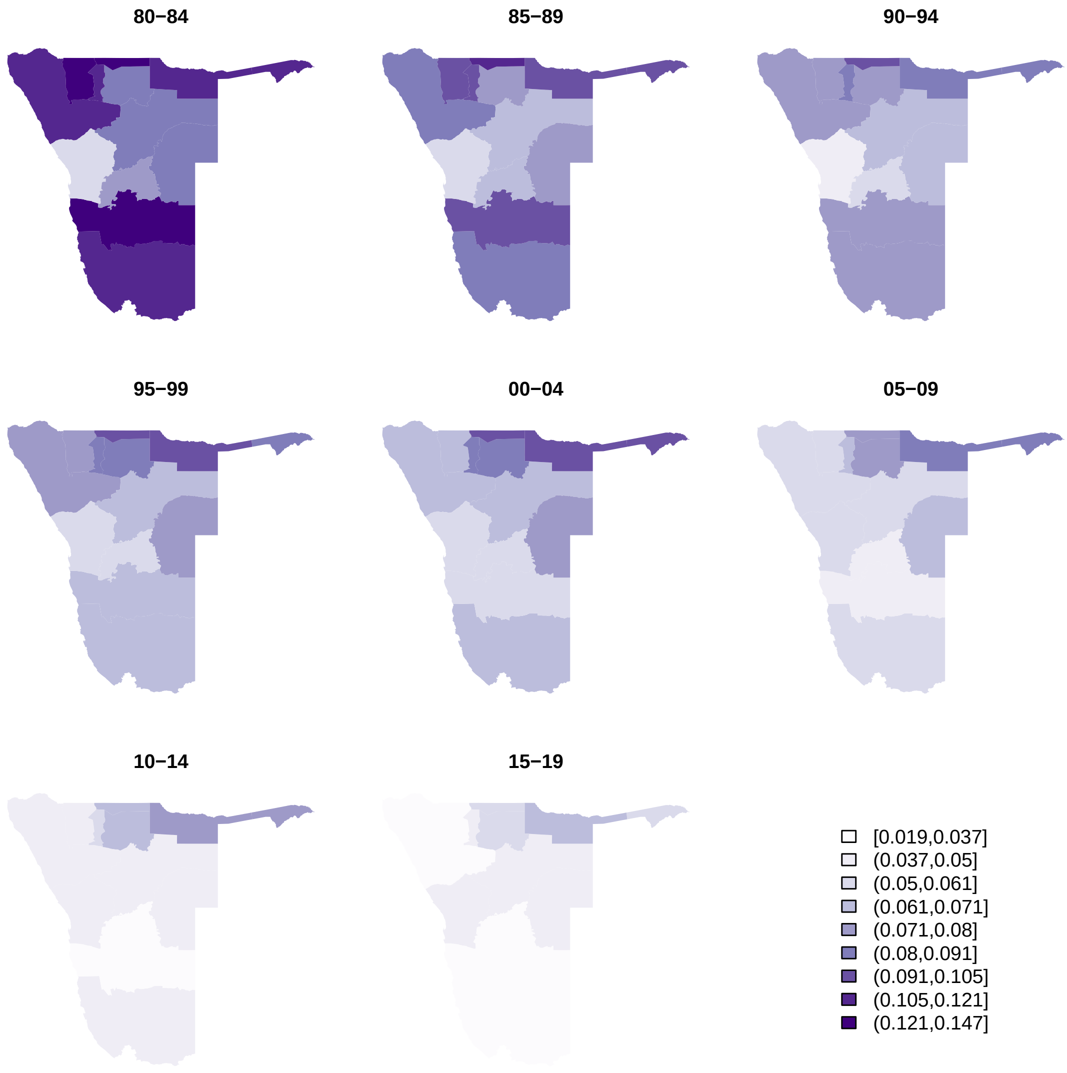

Figure S1.224: Namibia: Maps of posterior medians over time.

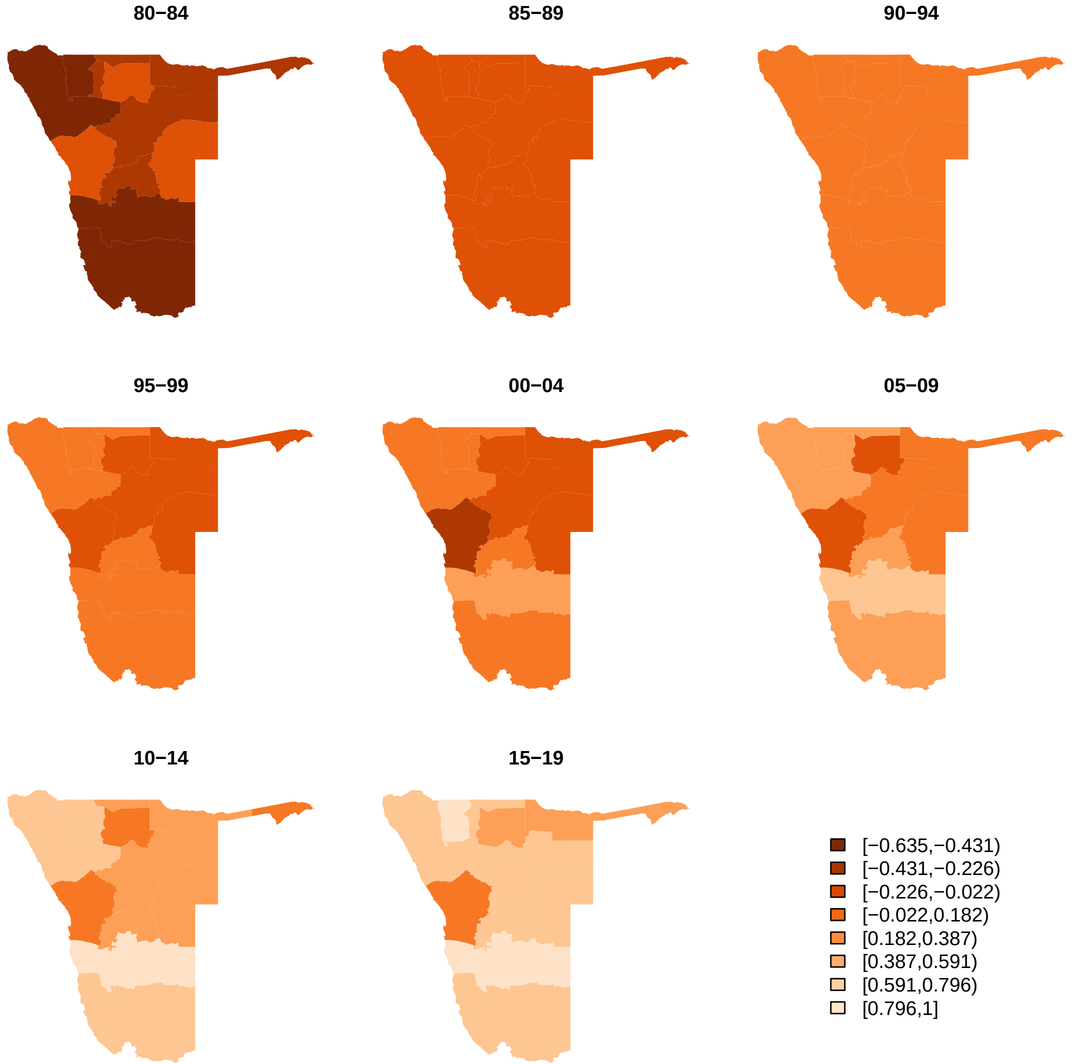

Figure S1.225: Namibia: Maps of reduction of posterior median U5MR in each five-year period compared to 1990 over time.

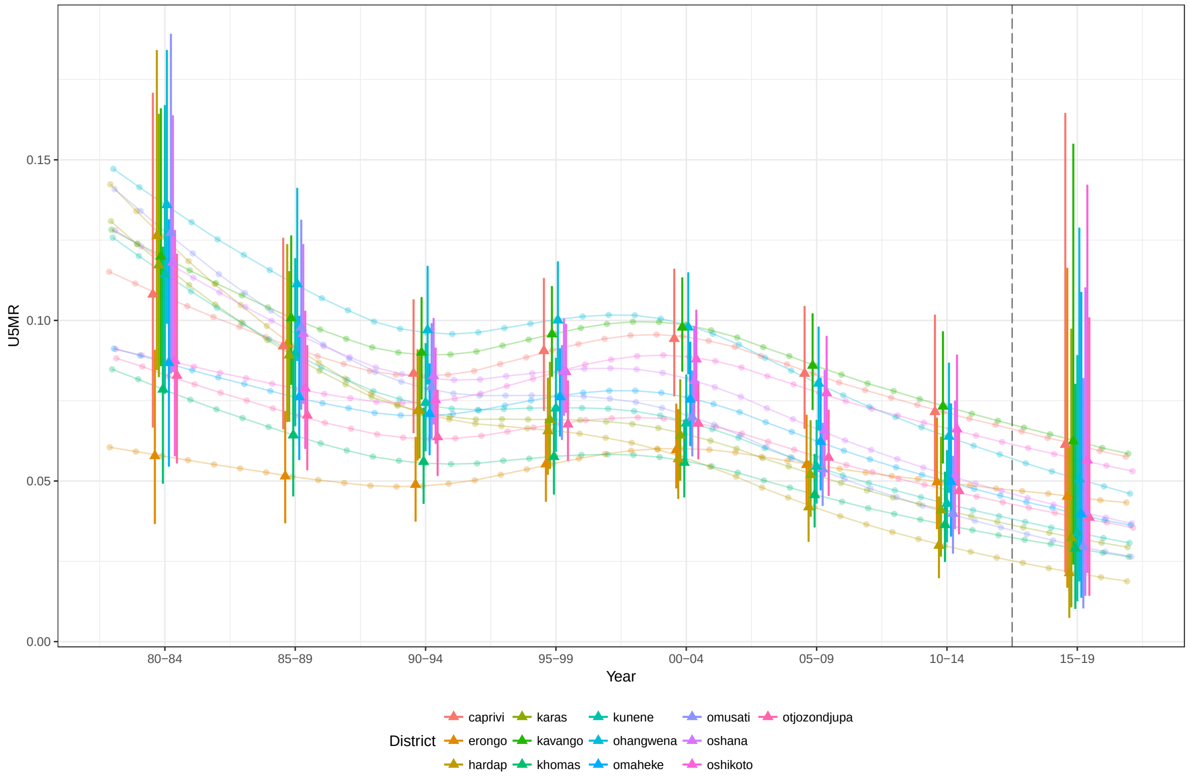

Figure S1.226: Namibia: Smoothed regional estimates over time. The line indicates yearly posterior median estimates and error bars indicate 95 % posterior credible interval at each time period.

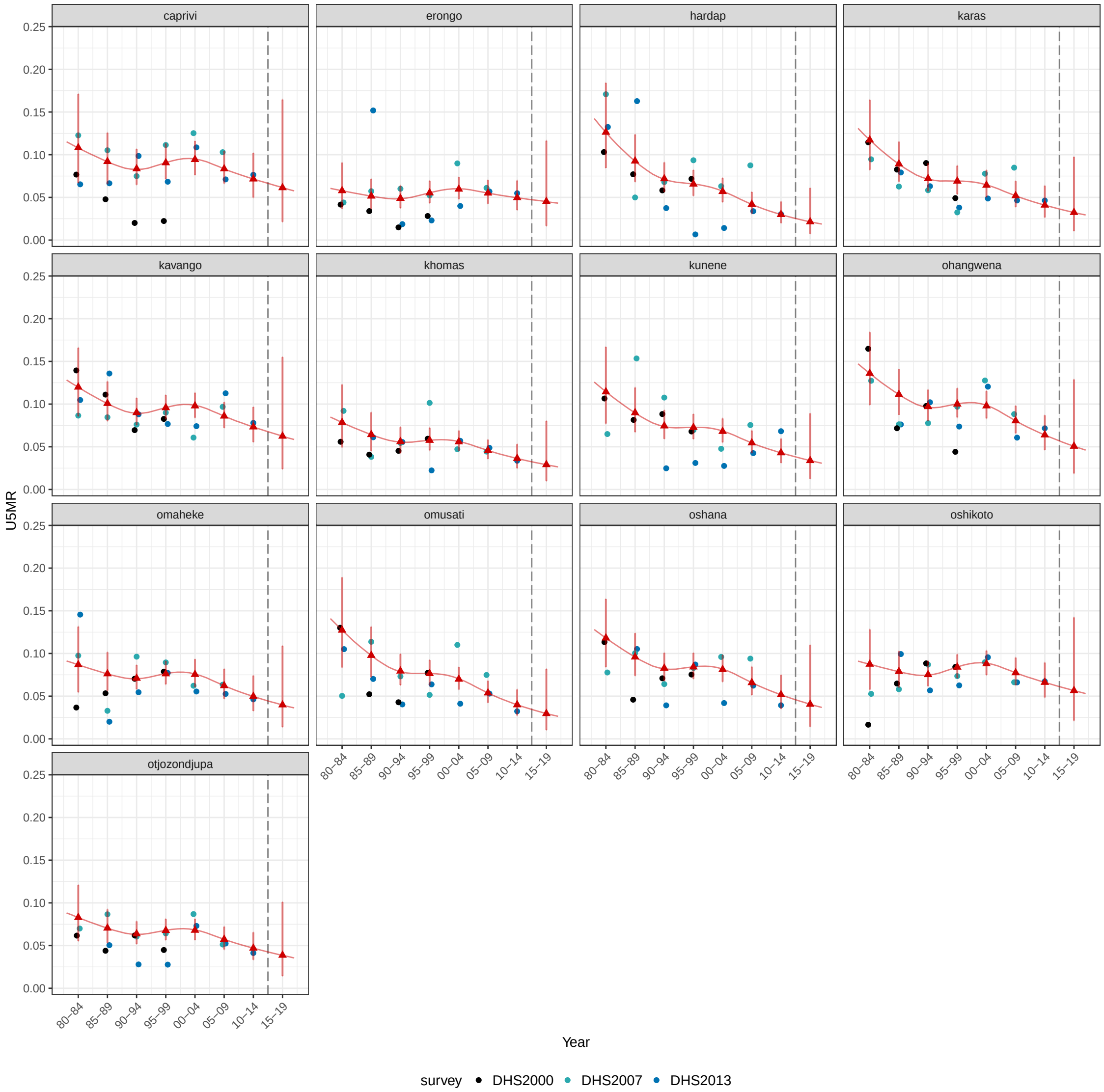

Figure S1.227: Namibia: Smoothed regional estimates over time compared to the direct estimates from each surveys. Direct estimates are not benchmarked with UN estimates. The line indicates posterior median and error bars indicate 95% posterior credible interval.

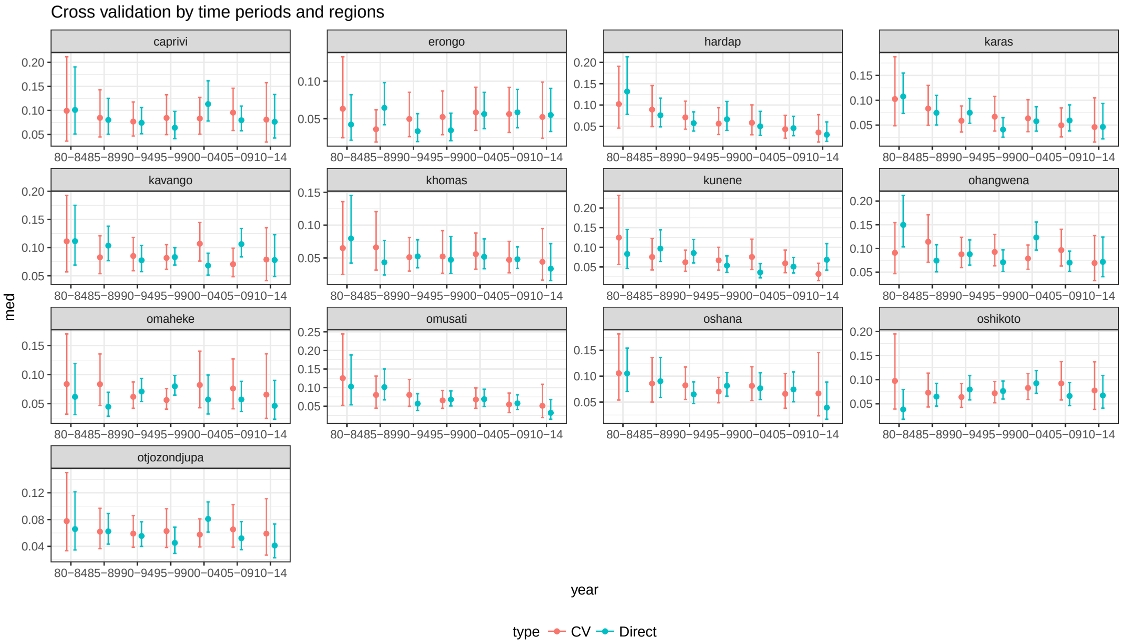

Figure S1.228: Namibia: Out-of-sample predictions along with direct estimates in the cross validation study where data from one region in each time period is held out and predicted using the rest of the data.

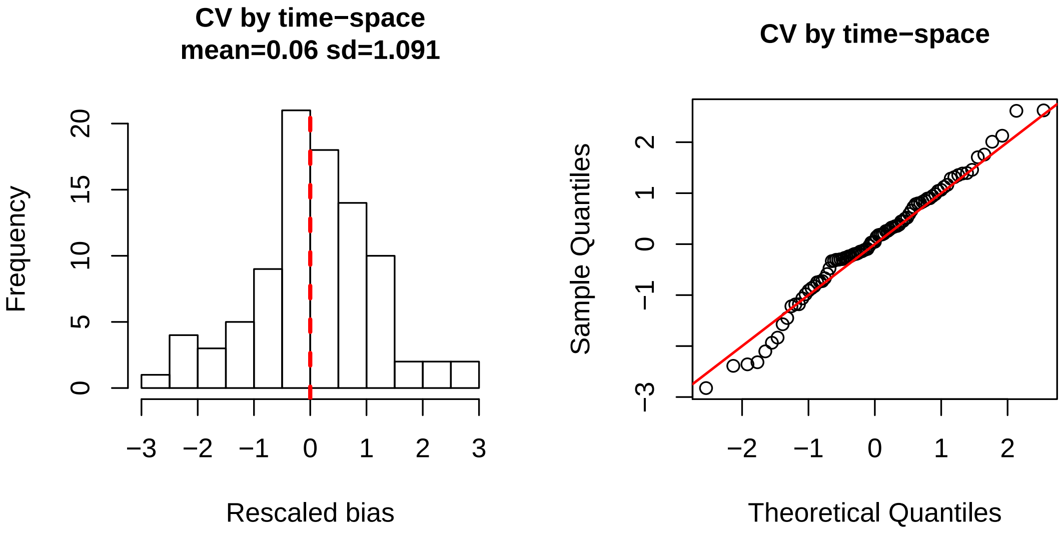

Figure S1.229: Namibia: Histogram and QQ-plot of the rescaled difference between the smoothed estimates and the direct estimates in the cross validation study. The differences between the two estimates are rescaled by the square root of the total variance of the two estimates.

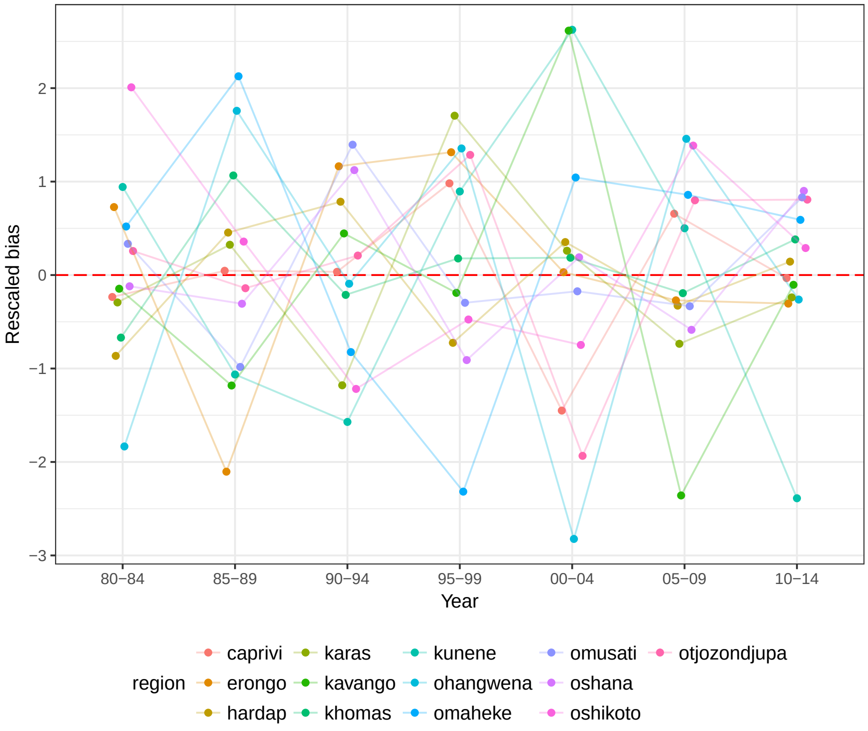

Figure S1.230: Namibia: Line plot of the difference between smoothed estimates and the direct estimates in the cross validation study. The differences between the two estimates are rescaled by the square root of the total variance of the two estimates.

3.5.26 Niger

DHS surveys were conducted in Niger in 1992, 1998, 2006, and 2012.

We fit both the RW2 only model to the combined national data, and compare the time trend at national level with the estimates produced by the UN and IHME in Figure S1.231. We then adjusted the combined national data to the UN estimates of U5MR, and refit the models on the benchmarked data.

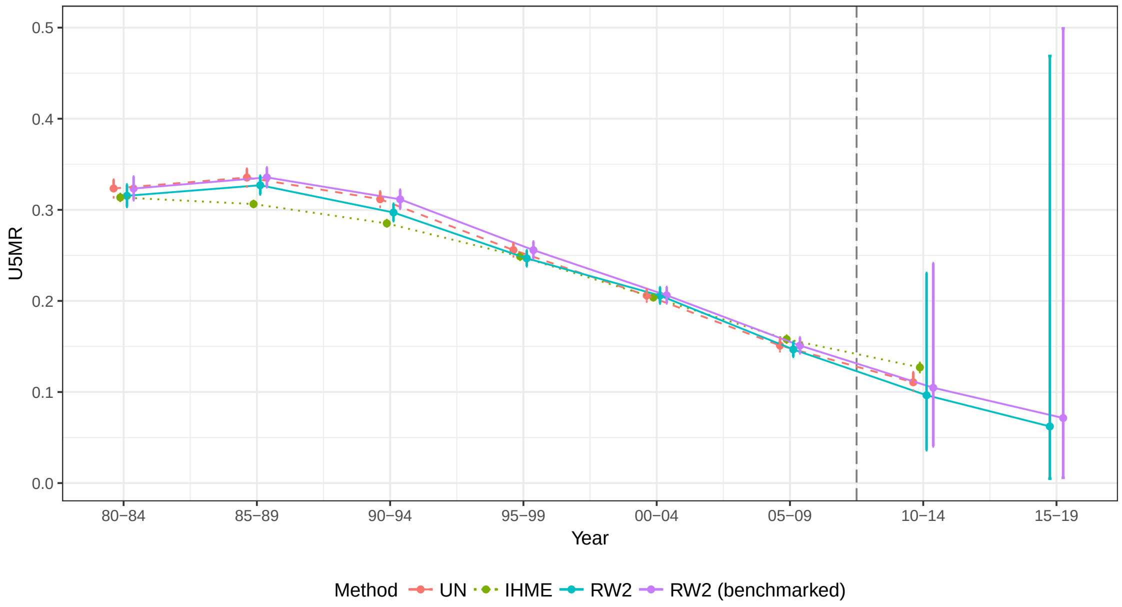

Figure S1.231: Niger: Temporal national trends along with UN (B3) estimates described in You et al. (2015) and IHME estimates based on GBD 2015 Child Mortality Collaborators (2016). RW2 represents the smoothed national estimates using the original data before benchmarking with UN estimates. RW2-adj represents the smoothed national estimates using the benchmarked data.

We fit the RW2 model to the benchmarked data in each area. We compare the results in Figure S1.232 to S1.236. Figure S1.232 compares the smoothed estimates against the direct estimates. Figure S1.233 and Figure S1.234 show the posterior median estimates of U5MR in each region over time and the reductions from 1990 period respectively. Figure S1.235 shows the smoothed estimates by region over time and Figure S1.236 compares the smoothed estimates with direct estimates from each survey for each region over time.

We further assess the RW2 model by holding out some observations, and compare the projections to the direct estimates in these holdout observations. Figure S1.237 compares the predicted estimates for the out-of-sample observations with the direct estimates by holding out observations from each area in each time period. Figure S1.238 compares the histogram of the bias rescaled by the total variance in the cross validation studies. Figure S1.239 compares the rescaled bias by region and time periods.

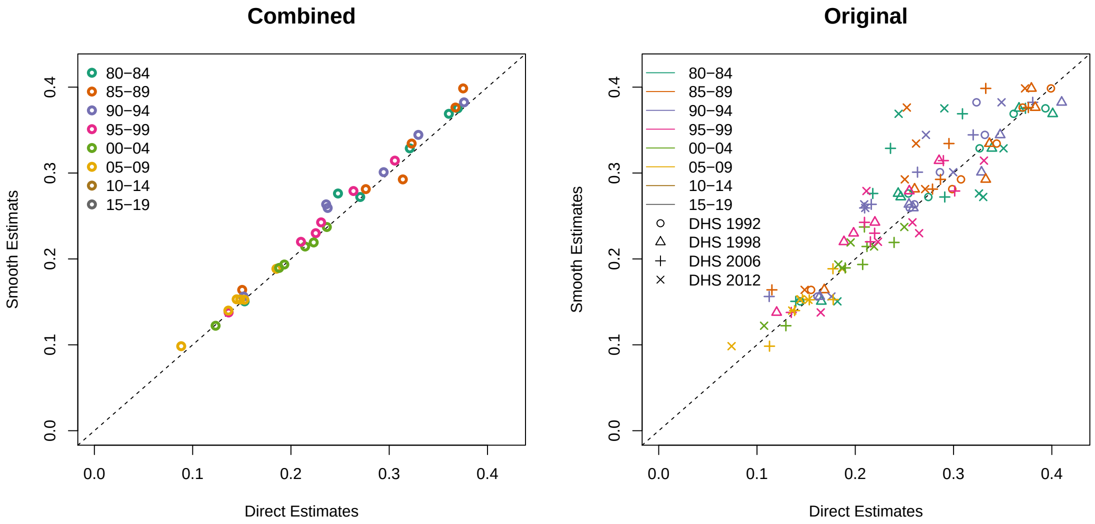

Figure S1.232: Niger: Smooth versus direct Admin 1 estimates. Left: Combined (meta-analysis) survey estimate against combined direct estimates. Right: Combined (meta-analysis) survey estimate against direct estimates from each survey.

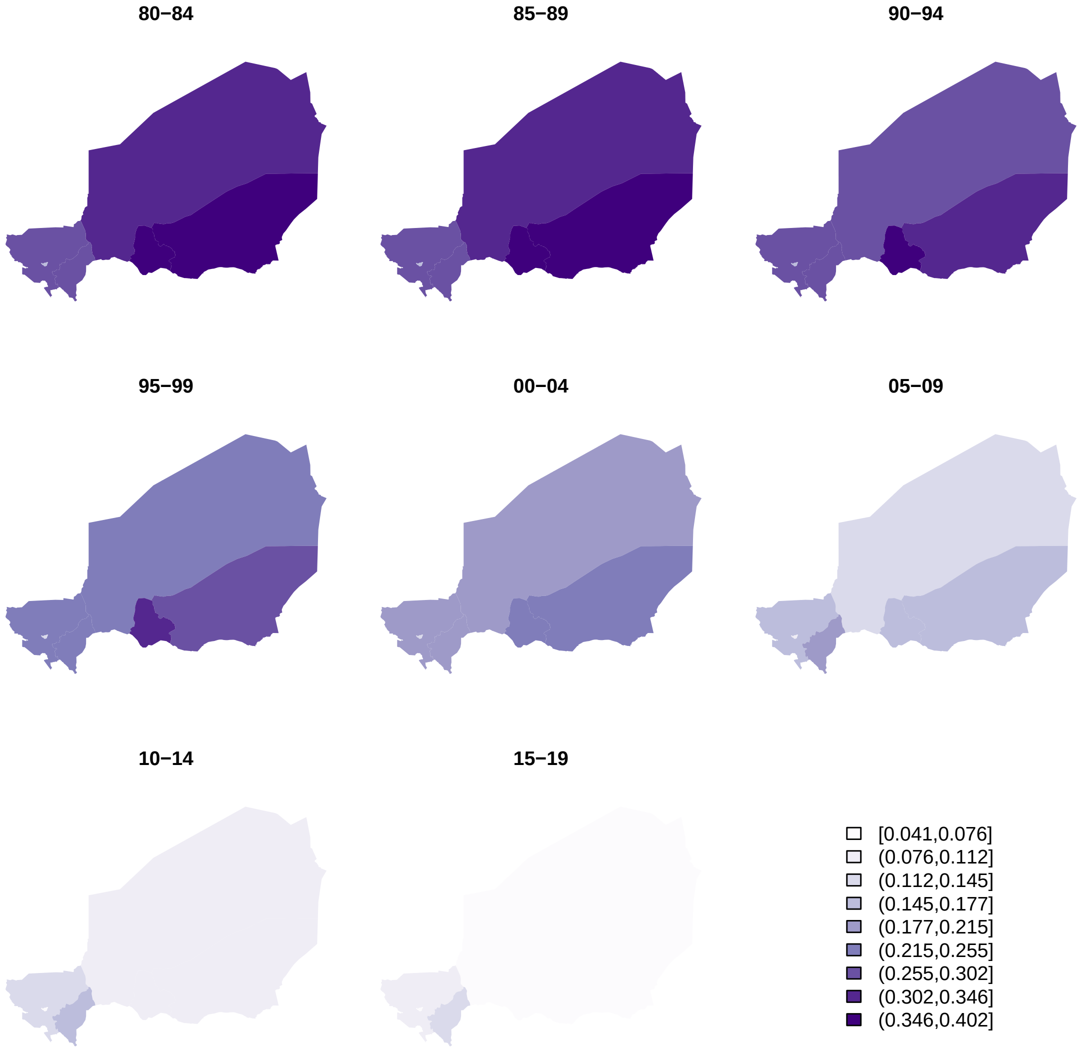

Figure S1.233: Niger: Maps of posterior medians over time.

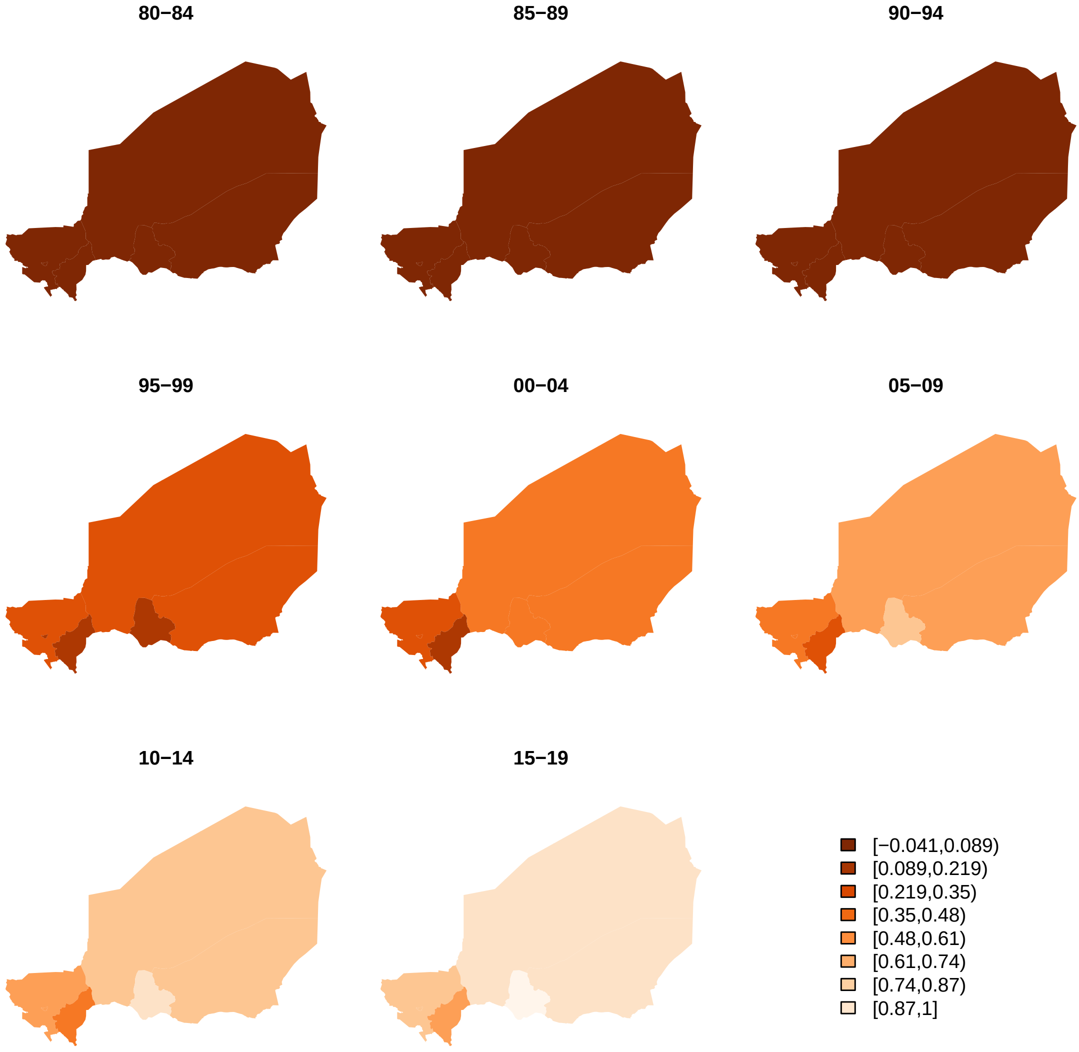

Figure S1.234: Niger: Maps of reduction of posterior median U5MR in each five-year period compared to 1990 over time.

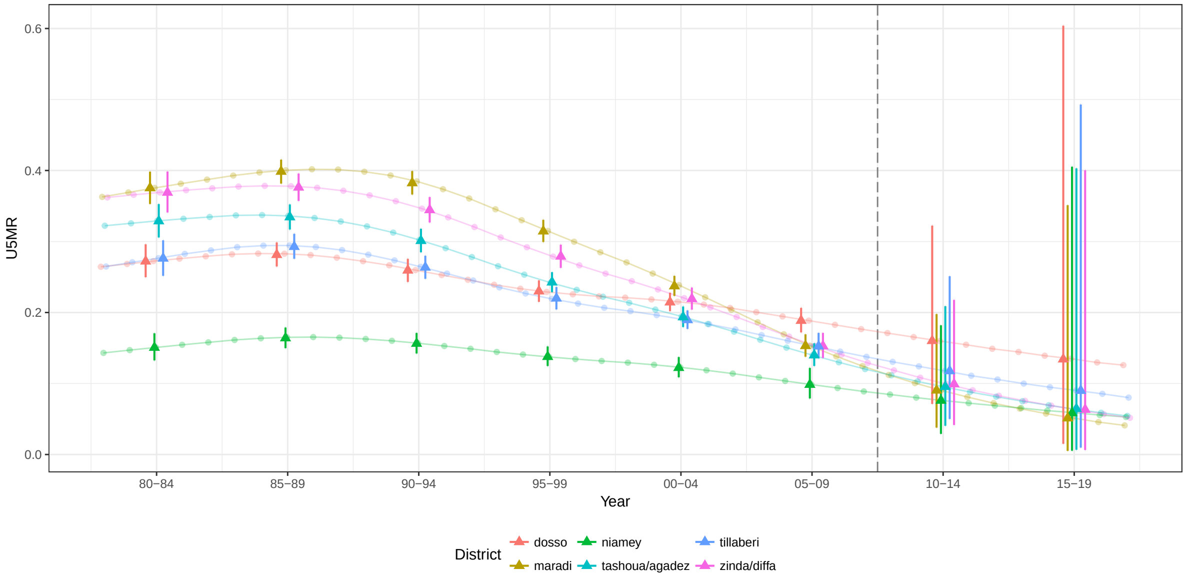

Figure S1.235: Niger: Smoothed regional estimates over time. The line indicates yearly posterior median estimates and error bars indicate 95 % posterior credible interval at each time period.

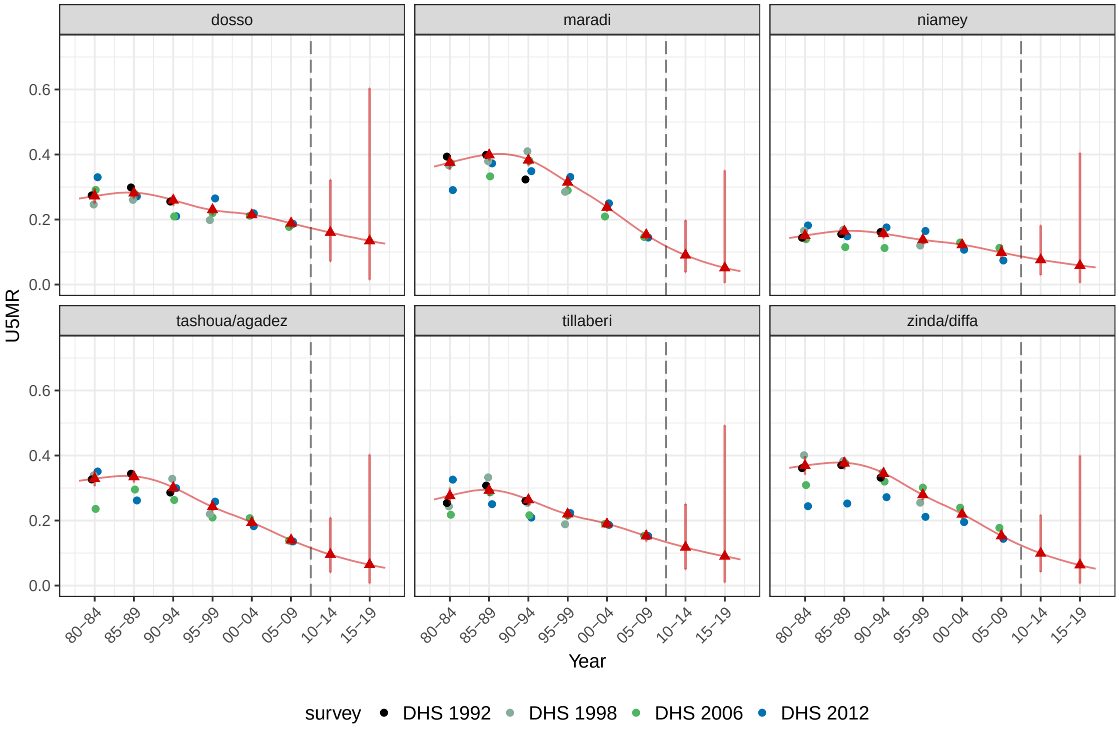

Figure S1.236: Niger: Smoothed regional estimates over time compared to the direct estimates from each surveys. Direct estimates are not benchmarked with UN estimates. The line indicates posterior median and error bars indicate 95% posterior credible interval.

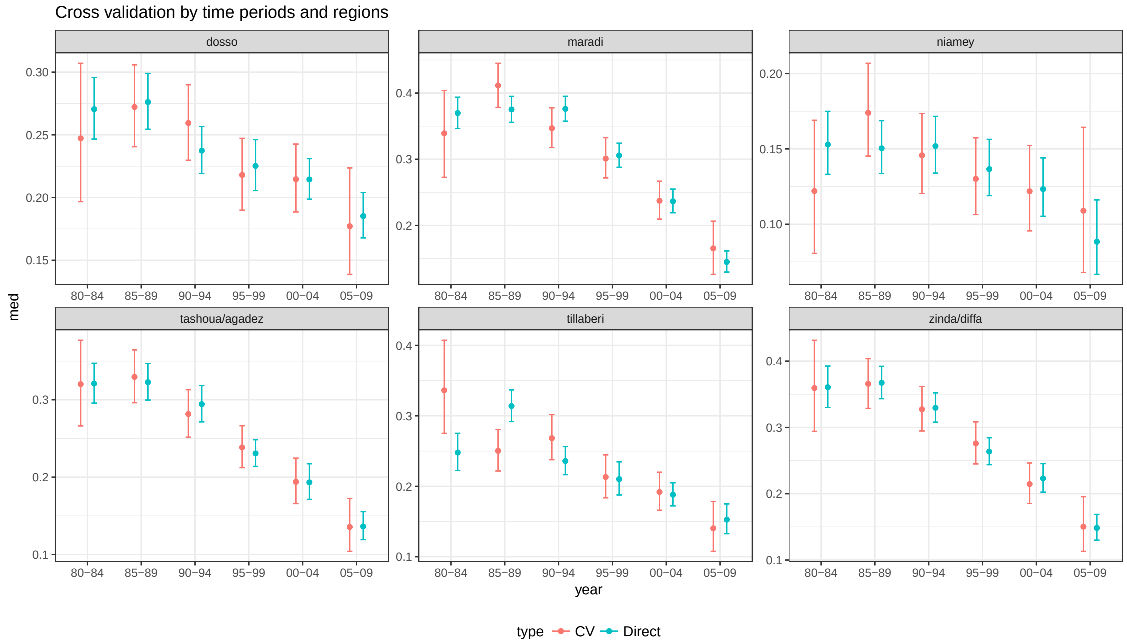

Figure S1.237: Niger: Out-of-sample predictions along with direct estimates in the cross validation study where data from one region in each time period is held out and predicted using the rest of the data.

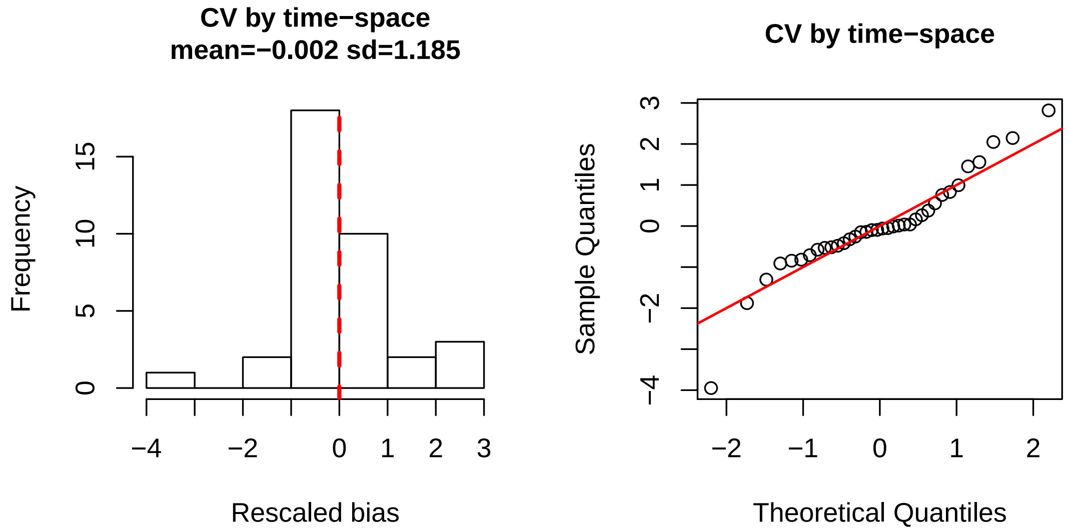

Figure S1.238: Niger: Histogram and QQ-plot of the rescaled difference between the smoothed estimates and the direct estimates in the cross validation study. The differences between the two estimates are rescaled by the square root of the total variance of the two estimates.

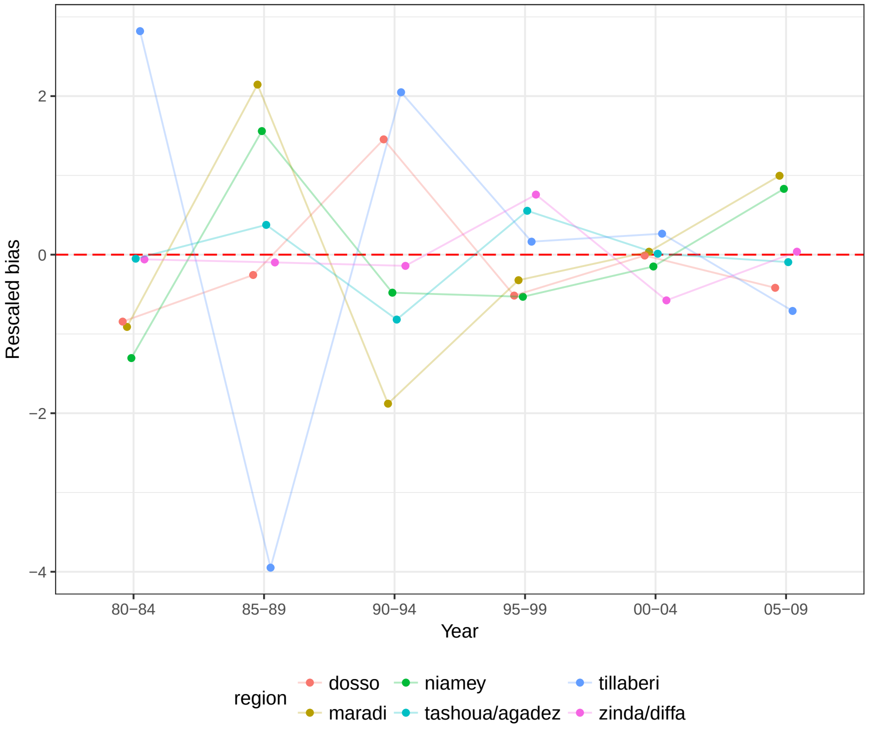

Figure S1.239: Niger: Line plot of the difference between smoothed estimates and the direct estimates in the cross validation study. The differences between the two estimates are rescaled by the square root of the total variance of the two estimates.

3.5.27 Nigeria

DHS surveys were conducted in Nigeria in 1990, 2003, 2008, and 2013.

We fit both the RW2 only model to the combined national data, and compare the time trend at national level with the estimates produced by the UN and IHME in Figure S1.240. We then adjusted the combined national data to the UN estimates of U5MR, and refit the models on the benchmarked data.

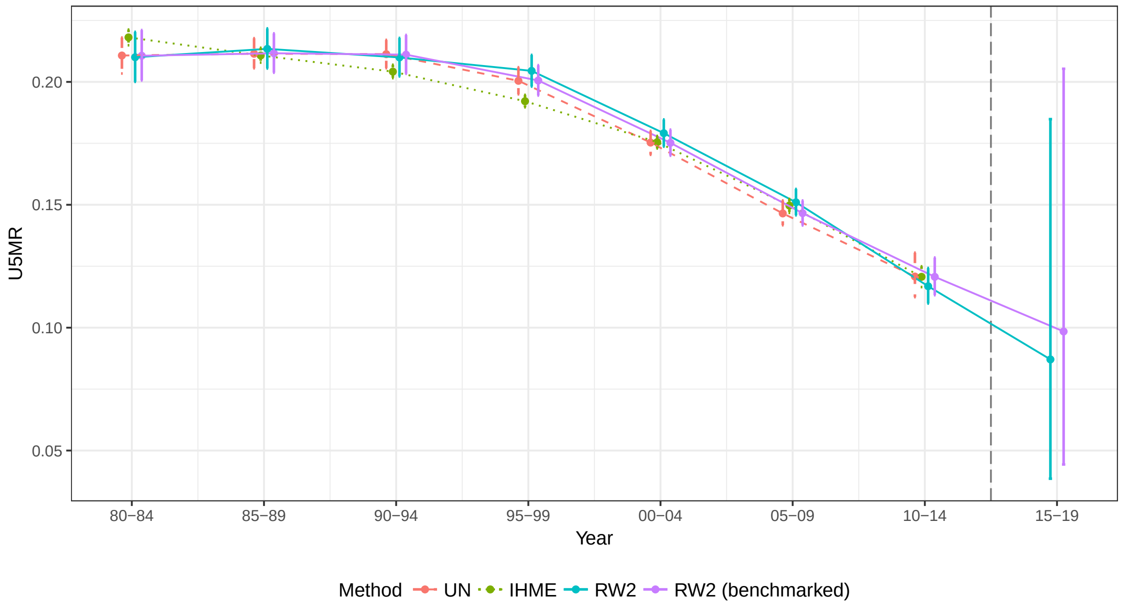

Figure S1.240: Nigeria: Temporal national trends along with UN (B3) estimates described in You et al. (2015) and IHME estimates based on GBD 2015 Child Mortality Collaborators (2016). RW2 represents the smoothed national estimates using the original data before benchmarking with UN estimates. RW2-adj represents the smoothed national estimates using the benchmarked data.

We fit the RW2 model to the benchmarked data in each area. We compare the results in Figure S1.241 to S1.245. Figure S1.241 compares the smoothed estimates against the direct estimates. Figure S1.242 and Figure S1.243 show the posterior median estimates of U5MR in each region over time and the reductions from 1990 period respectively. Figure S1.244 shows the smoothed estimates by region over time and Figure S1.245 compares the smoothed estimates with direct estimates from each survey for each region over time.

We further assess the RW2 model by holding out some observations, and compare the projections to the direct estimates in these holdout observations. Figure S1.246 compares the predicted estimates for the out-of-sample observations with the direct estimates by holding out observations from each area in each time period. Figure S1.247 compares the histogram of the bias rescaled by the total variance in the cross validation studies. Figure S1.248 compares the rescaled bias by region and time periods.

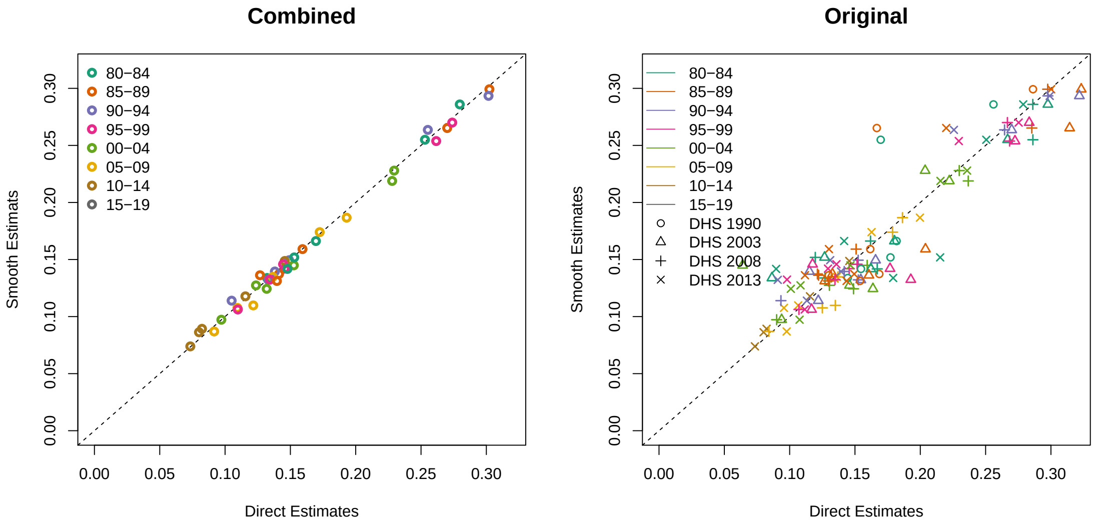

Figure S1.241: Nigeria: Smooth versus direct Admin 1 estimates. Left: Combined (meta-analysis) survey estimate against combined direct estimates. Right: Combined (meta-analysis) survey estimate against direct estimates from each survey.

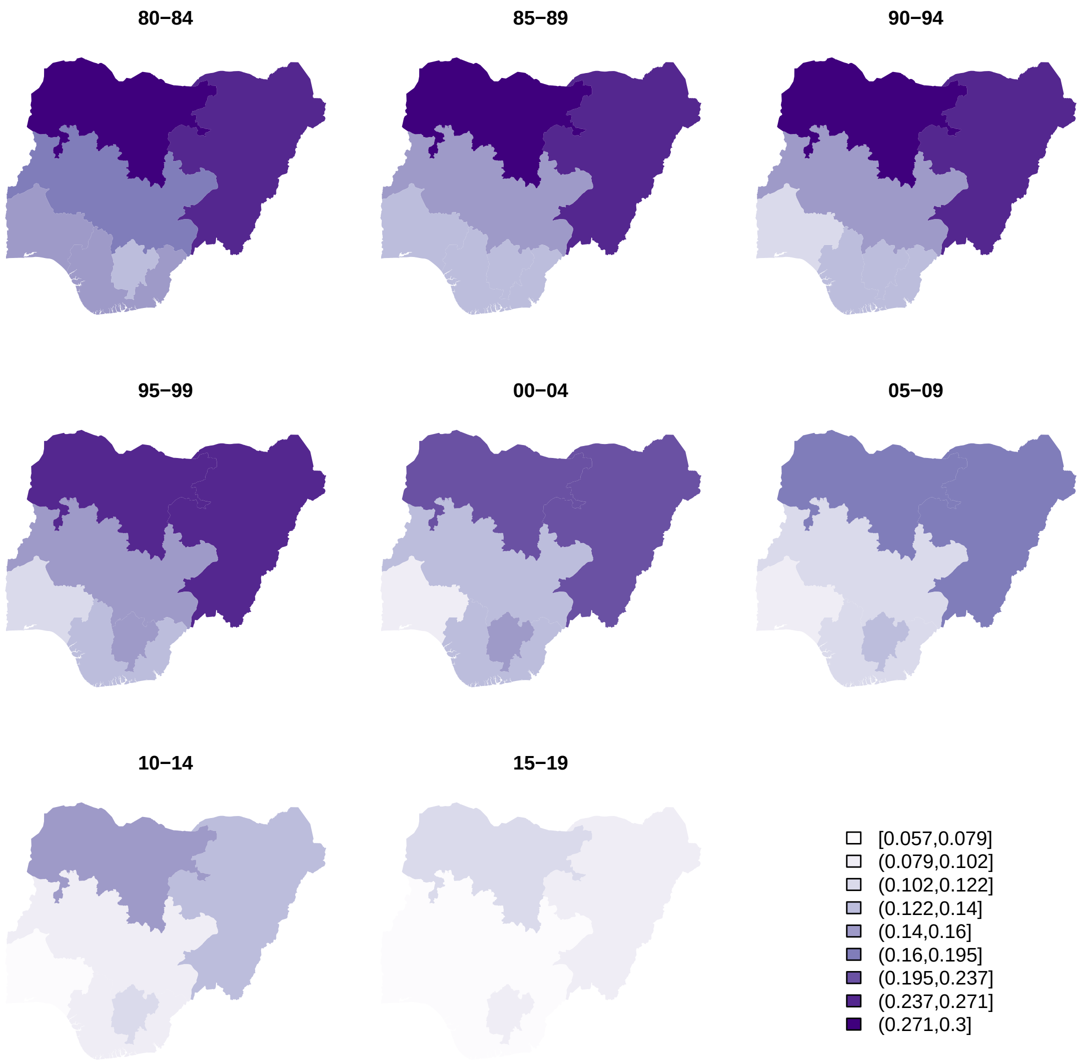

Figure S1.242: Nigeria: Maps of posterior medians over time.

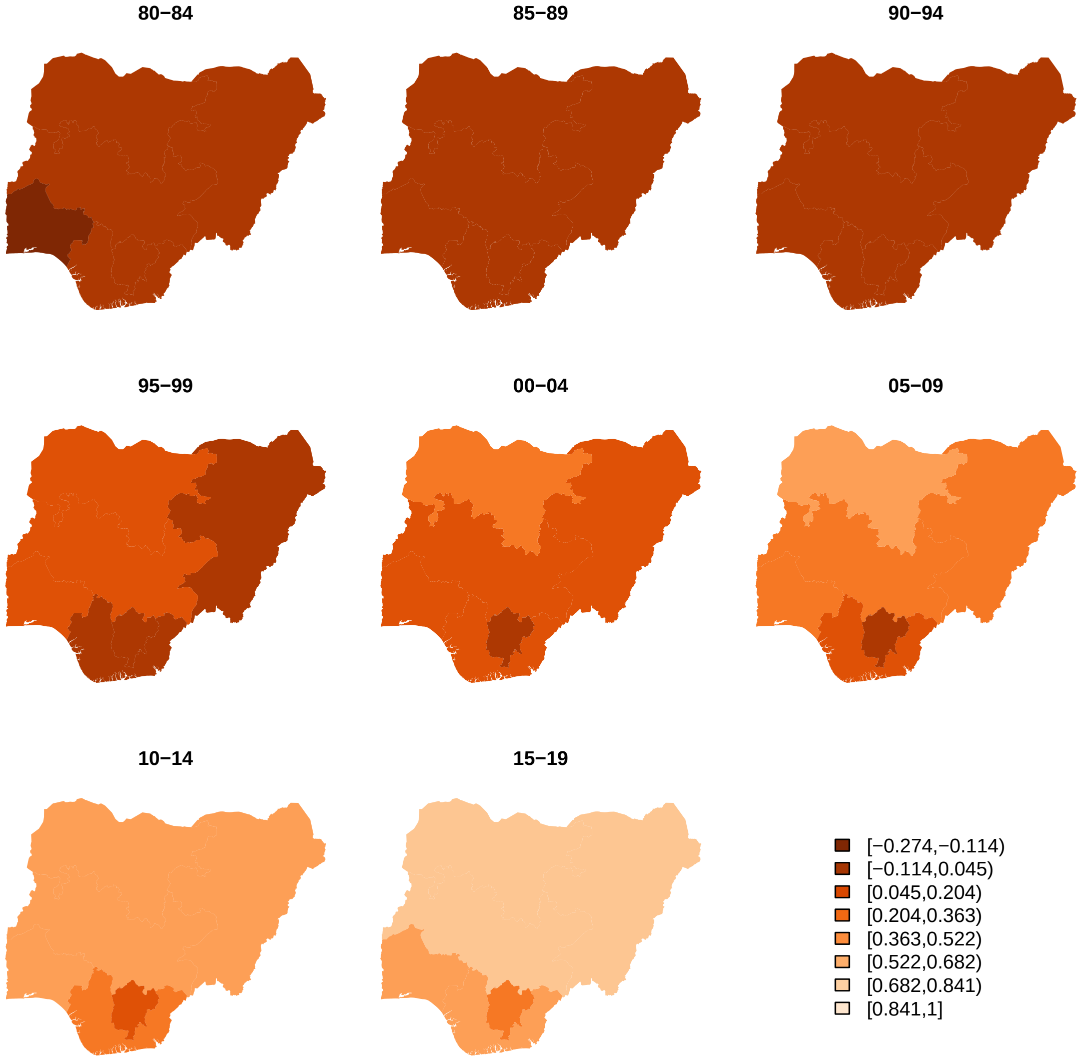

Figure S1.243: Nigeria: Maps of reduction of posterior median U5MR in each five-year period compared to 1990 over time.

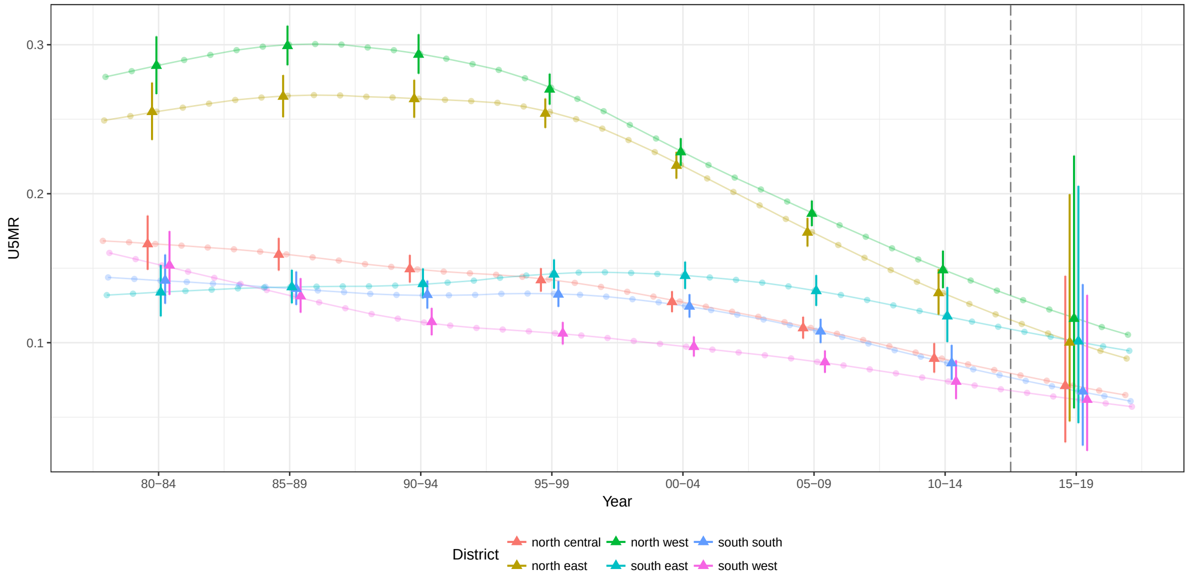

Figure S1.244: Nigeria: Smoothed regional estimates over time. The line indicates yearly posterior median estimates and error bars indicate 95 % posterior credible interval at each time period.

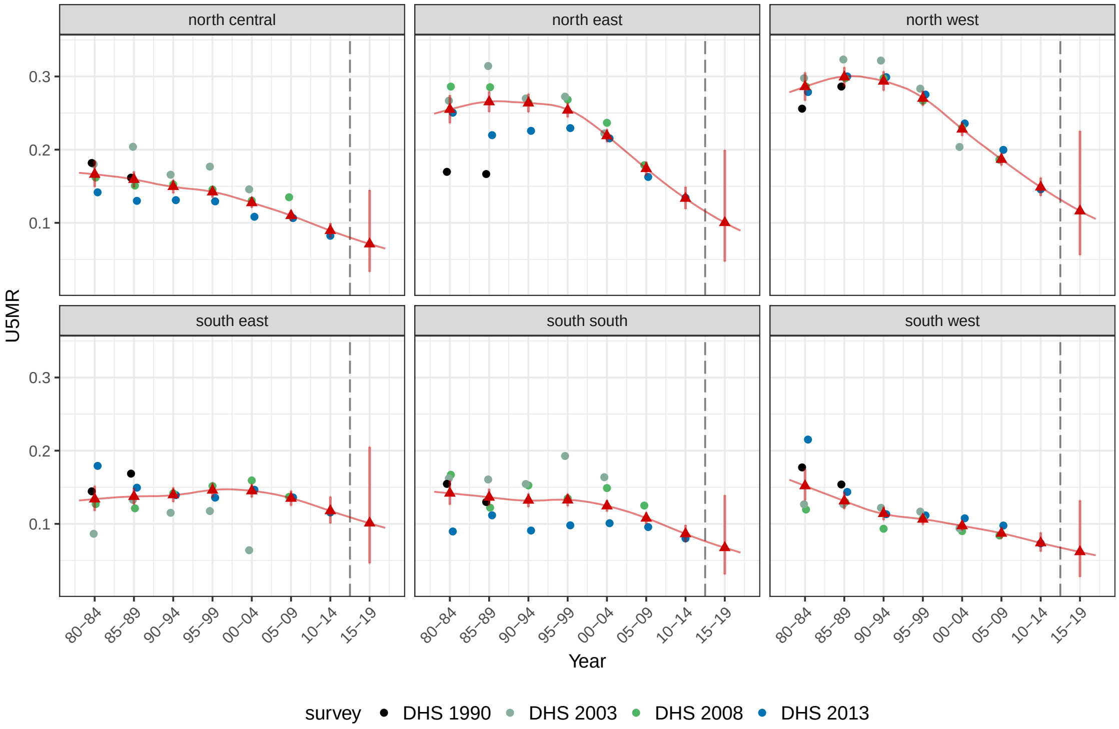

Figure S1.245: Nigeria: Smoothed regional estimates over time compared to the direct estimates from each surveys. Direct estimates are not benchmarked with UN estimates. The line indicates posterior median and error bars indicate 95% posterior credible interval.

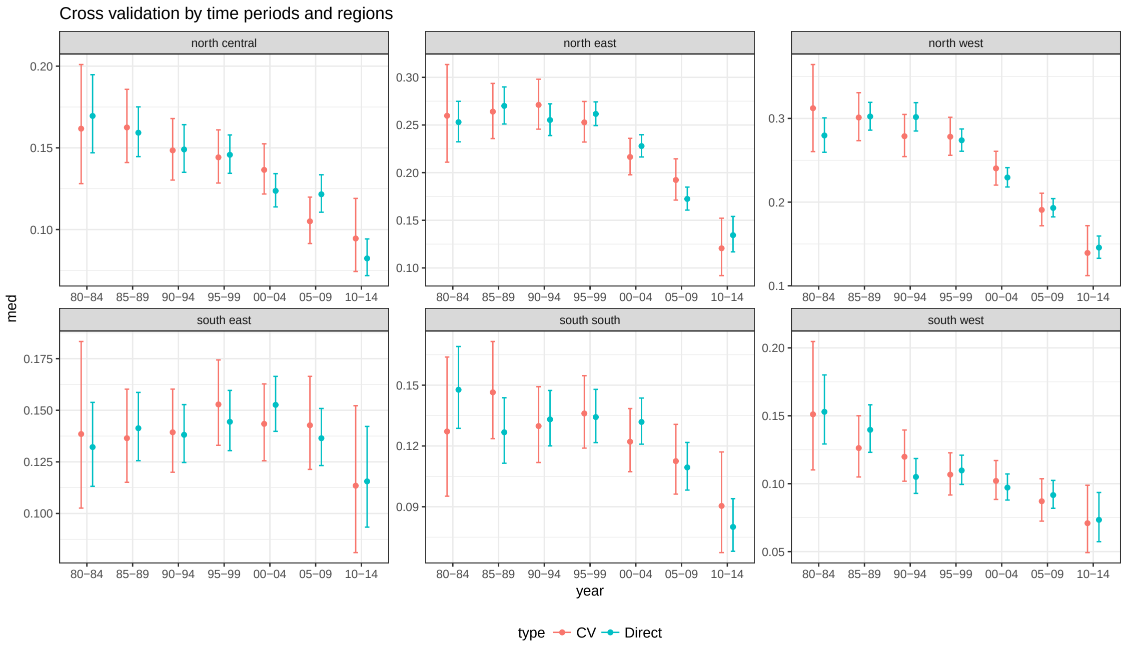

Figure S1.246: Nigeria: Out-of-sample predictions along with direct estimates in the cross validation study where data from one region in each time period is held out and predicted using the rest of the data.

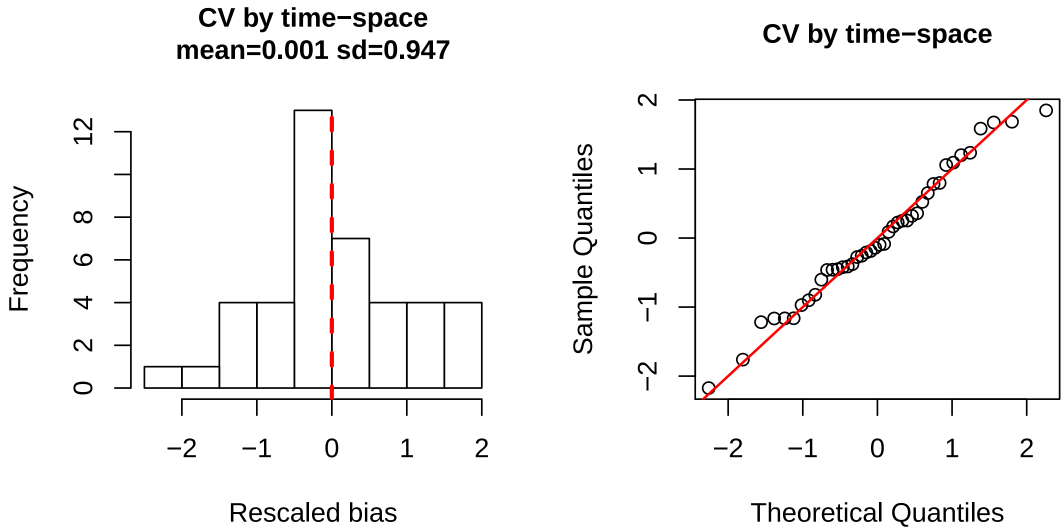

Figure S1.247: Nigeria: Histogram and QQ-plot of the rescaled difference between the smoothed estimates and the direct estimates in the cross validation study. The differences between the two estimates are rescaled by the square root of the total variance of the two estimates.

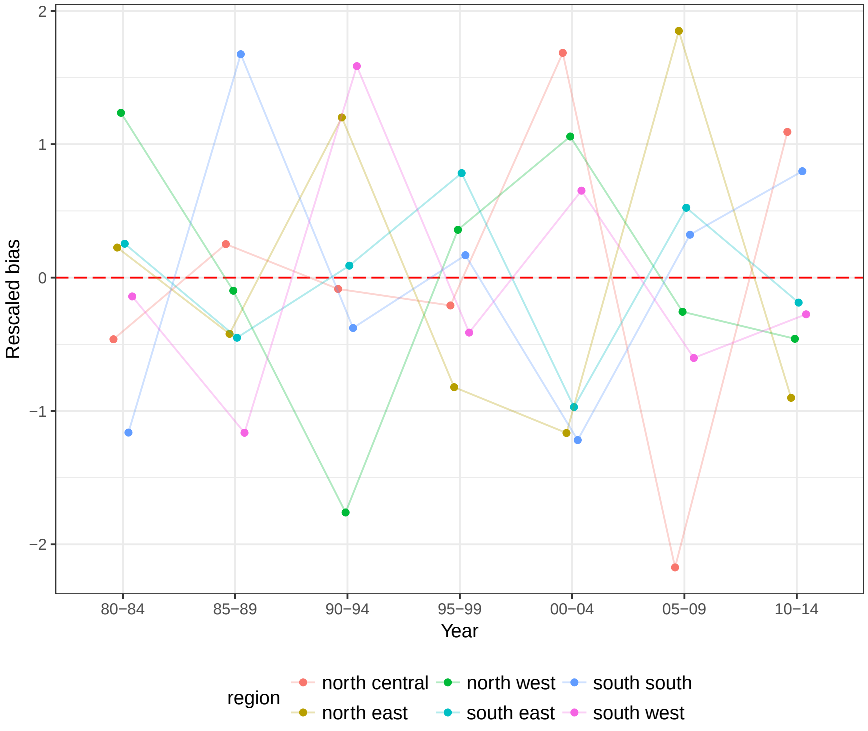

Figure S1.248: Nigeria: Line plot of the difference between smoothed estimates and the direct estimates in the cross validation study. The differences between the two estimates are rescaled by the square root of the total variance of the two estimates.

3.5.28 Rwanda

DHS surveys were conducted in Rwanda in 2000, 2005, 2008, 2010, and 2015.

We fit both the RW2 only model to the combined national data, and compare the time trend at national level with the estimates produced by the UN and IHME in Figure S1.249. We then adjusted the combined national data to the UN estimates of U5MR, and refit the models on the benchmarked data.

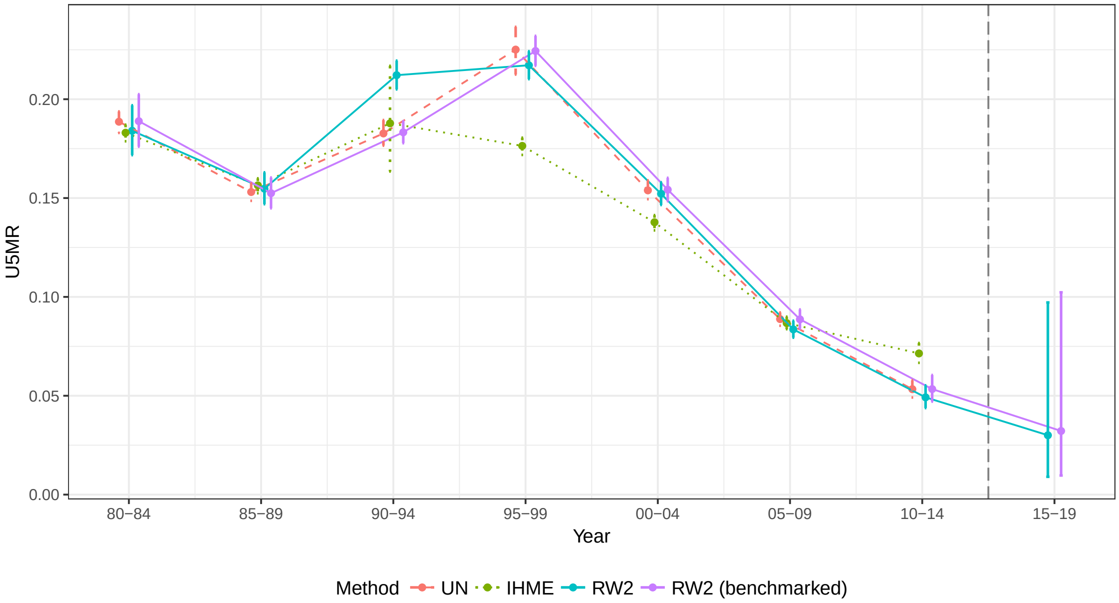

Figure S1.249: Rwanda: Temporal national trends along with UN (B3) estimates described in You et al. (2015) and IHME estimates based on GBD 2015 Child Mortality Collaborators (2016). RW2 represents the smoothed national estimates using the original data before benchmarking with UN estimates. RW2-adj represents the smoothed national estimates using the benchmarked data.

We fit the RW2 model to the benchmarked data in each area. We compare the results in Figure S1.250 to S1.254. Figure S1.250 compares the smoothed estimates against the direct estimates. Figure S1.251 and Figure S1.252 show the posterior median estimates of U5MR in each region over time and the reductions from 1990 period respectively. Figure S1.253 shows the smoothed estimates by region over time and Figure S1.254 compares the smoothed estimates with direct estimates from each survey for each region over time.

We further assess the RW2 model by holding out some observations, and compare the projections to the direct estimates in these holdout observations. Figure S1.255 compares the predicted estimates for the out-of-sample observations with the direct estimates by holding out observations from each area in each time period. Figure S1.256 compares the histogram of the bias rescaled by the total variance in the cross validation studies. Figure S1.257 compares the rescaled bias by region and time periods.

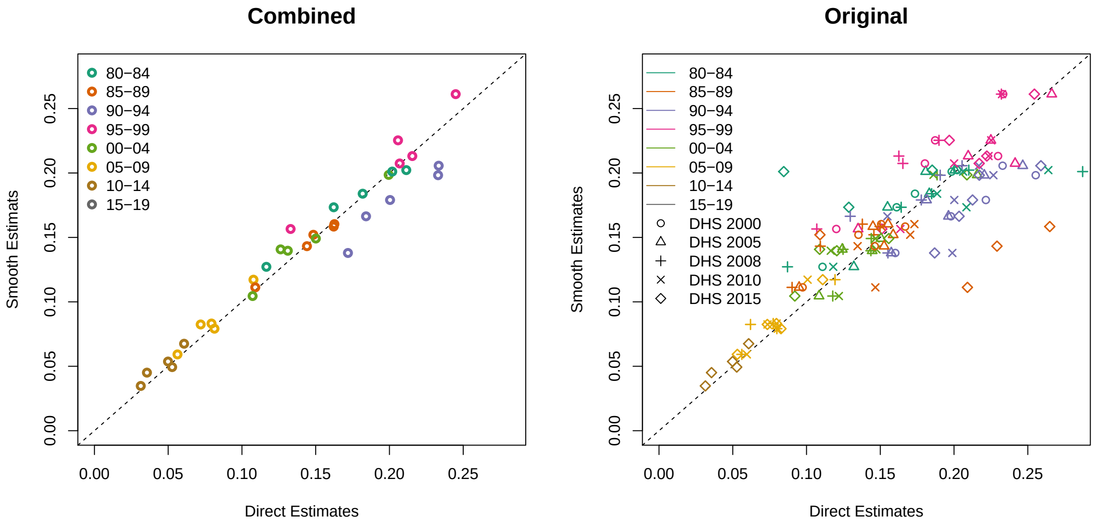

Figure S1.250: Rwanda: Smooth versus direct Admin 1 estimates. Left: Combined (meta-analysis) survey estimate against combined direct estimates. Right: Combined (meta-analysis) survey estimate against direct estimates from each survey.

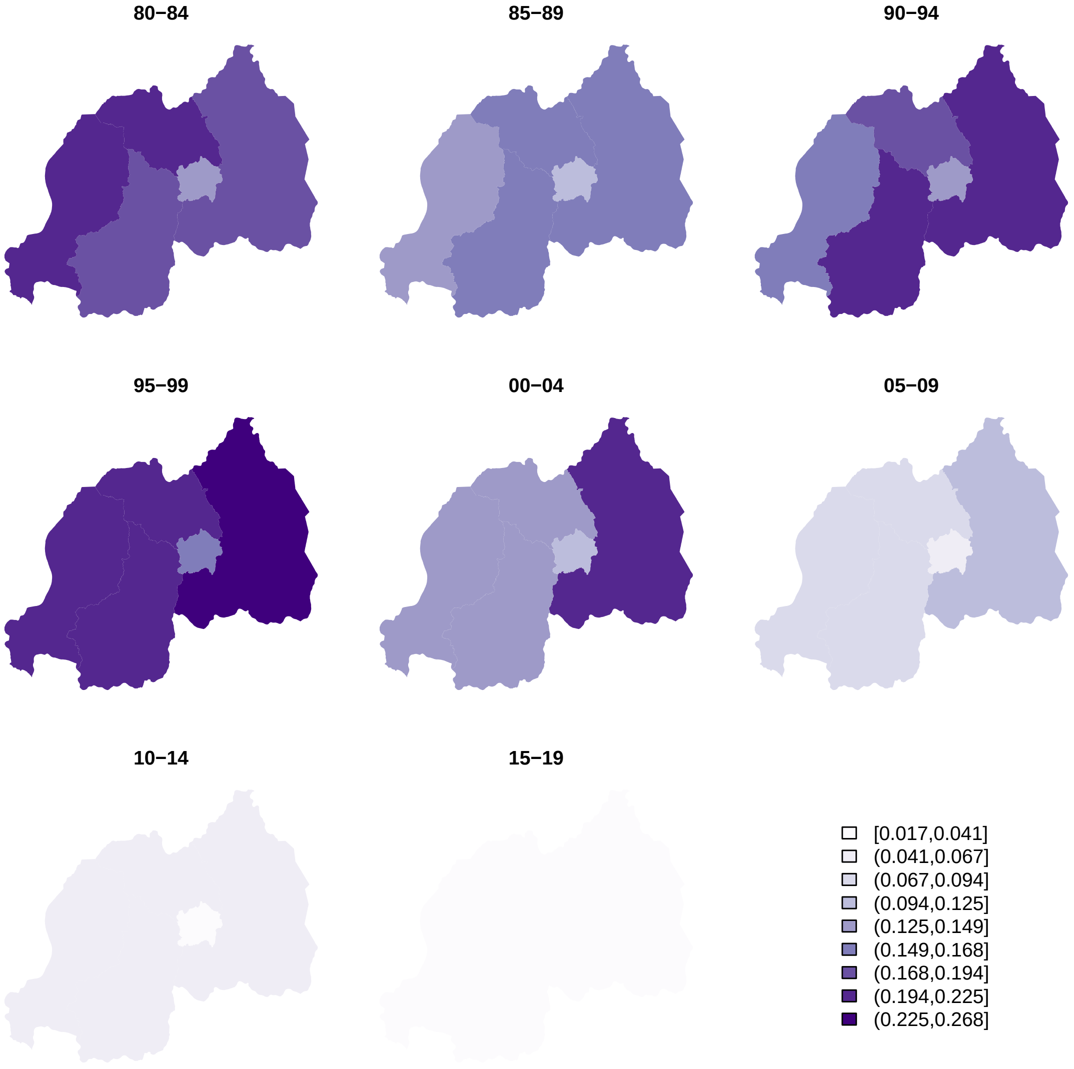

Figure S1.251: Rwanda: Maps of posterior medians over time.

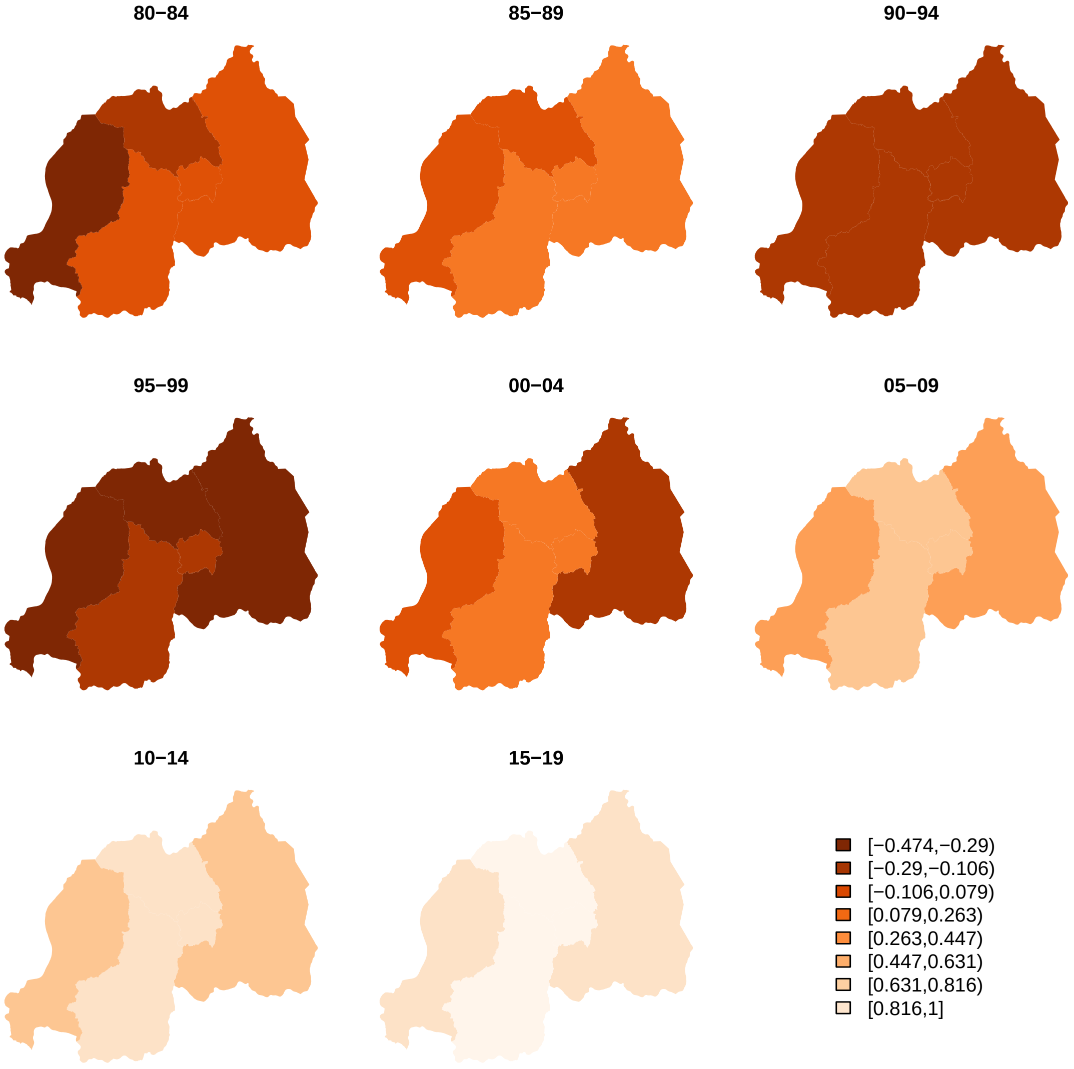

Figure S1.252: Rwanda: Maps of reduction of posterior median U5MR in each five-year period compared to 1990 over time.

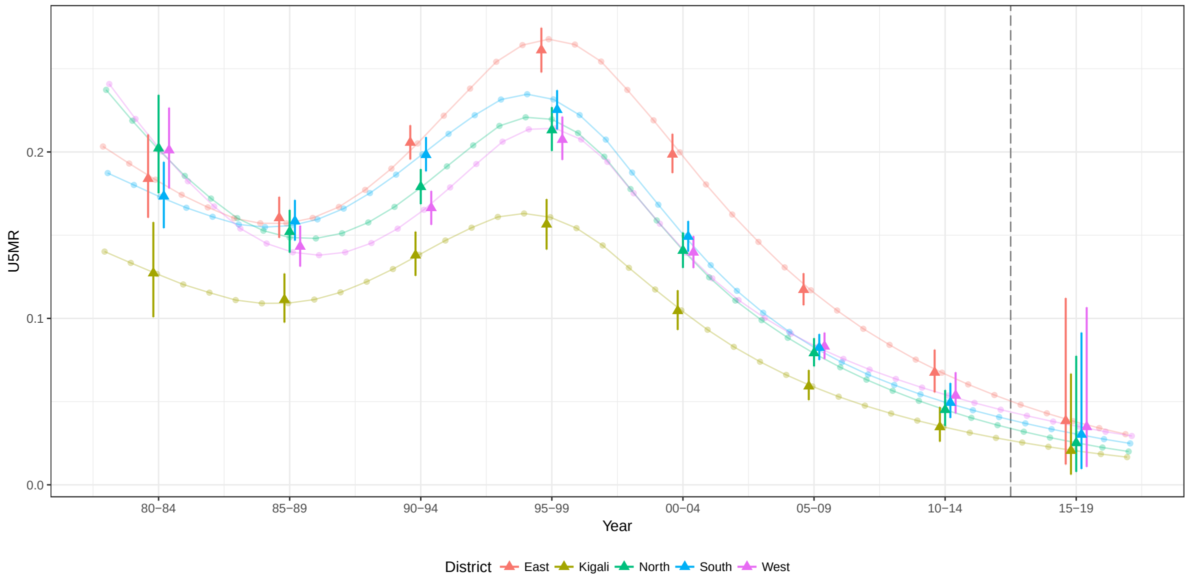

Figure S1.253: Rwanda: Smoothed regional estimates over time. The line indicates yearly posterior median estimates and error bars indicate 95 % posterior credible interval at each time period.

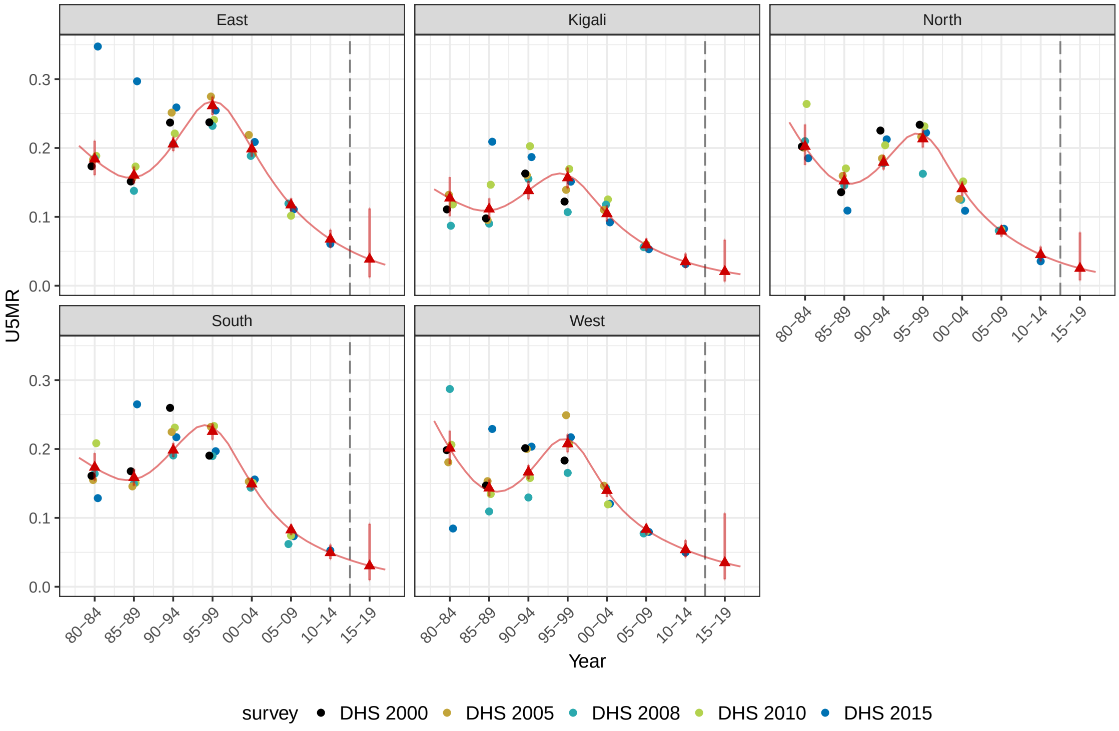

Figure S1.254: Rwanda: Smoothed regional estimates over time compared to the direct estimates from each surveys. Direct estimates are not benchmarked with UN estimates. The line indicates posterior median and error bars indicate 95% posterior credible interval.

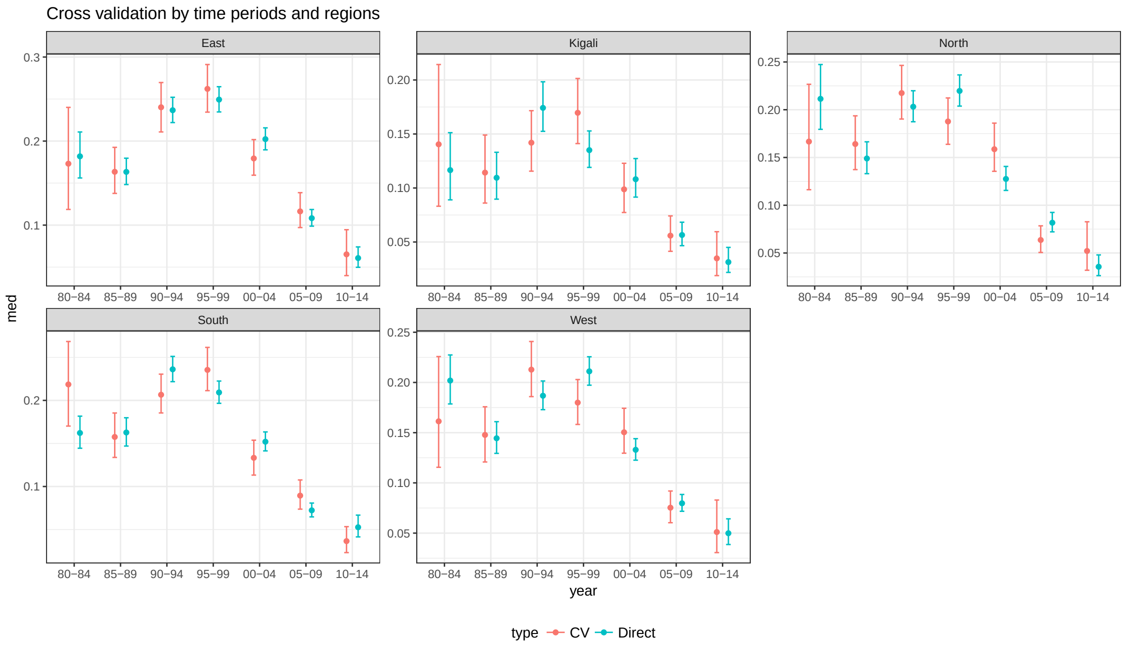

Figure S1.255: Rwanda: Out-of-sample predictions along with direct estimates in the cross validation study where data from one region in each time period is held out and predicted using the rest of the data.

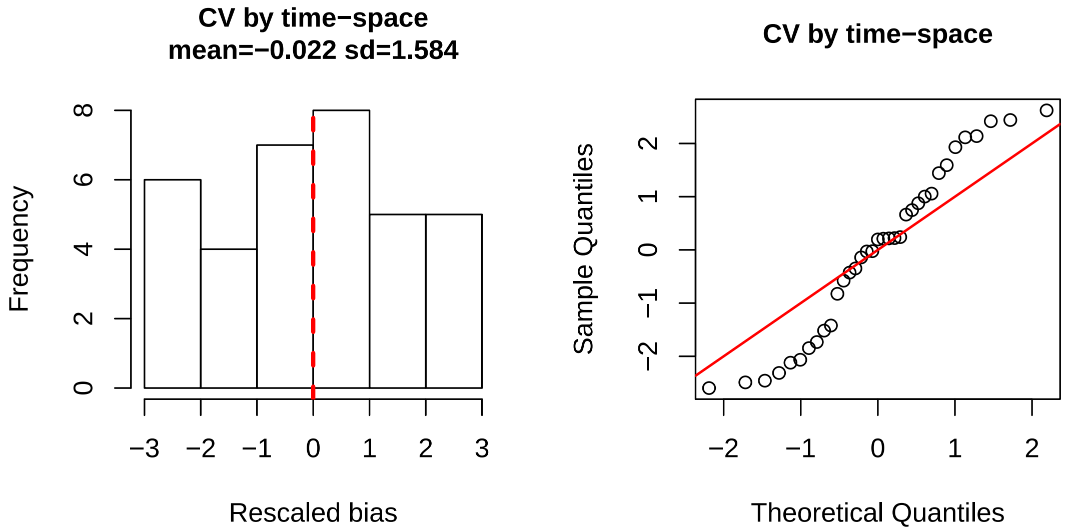

Figure S1.256: Rwanda: Histogram and QQ-plot of the rescaled difference between the smoothed estimates and the direct estimates in the cross validation study. The differences between the two estimates are rescaled by the square root of the total variance of the two estimates.

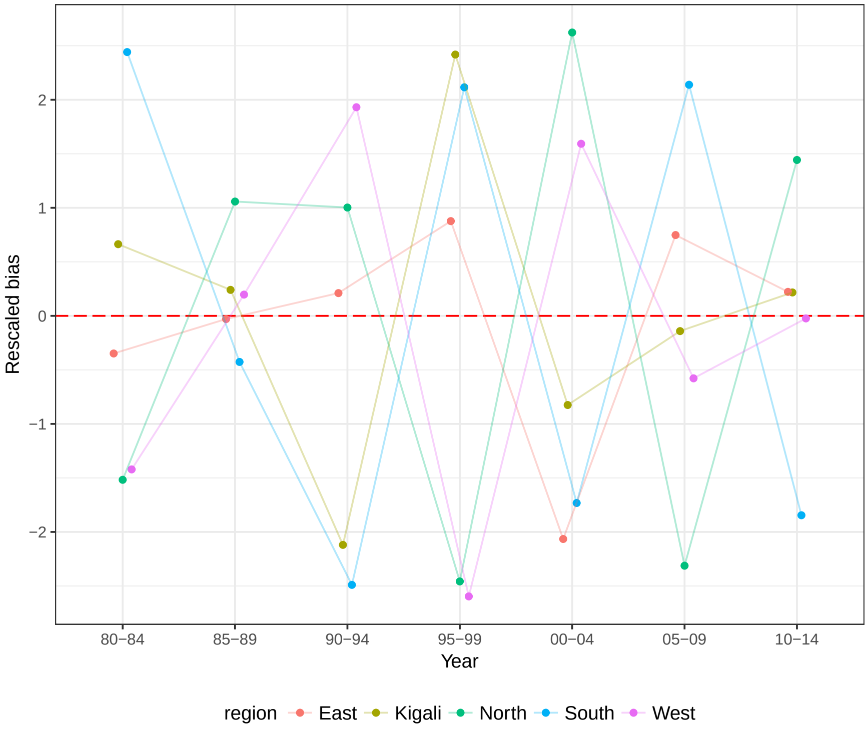

Figure S1.257: Rwanda: Line plot of the difference between smoothed estimates and the direct estimates in the cross validation study. The differences between the two estimates are rescaled by the square root of the total variance of the two estimates.

3.5.29 Senegal

DHS surveys were conducted in Senegal in 1992, 1997, 2005, 2010, 2012, 2014, 2015, and 2016.

We fit both the RW2 only model to the combined national data, and compare the time trend at national level with the estimates produced by the UN and IHME in Figure S1.258. We then adjusted the combined national data to the UN estimates of U5MR, and refit the models on the benchmarked data.

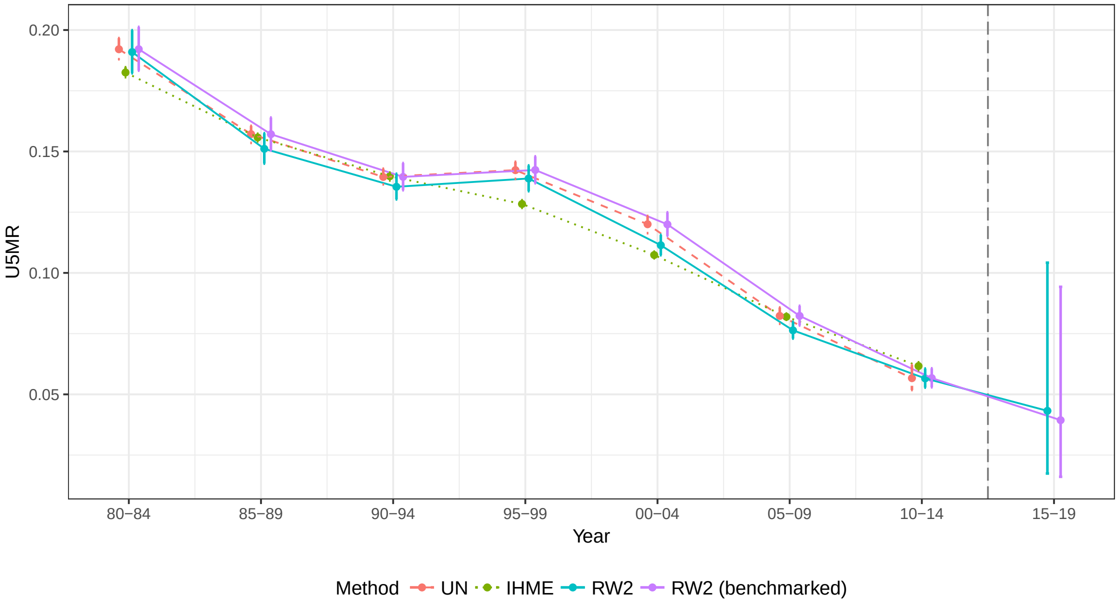

Figure S1.258: Senegal: Temporal national trends along with UN (B3) estimates described in You et al. (2015) and IHME estimates based on GBD 2015 Child Mortality Collaborators (2016). RW2 represents the smoothed national estimates using the original data before benchmarking with UN estimates. RW2-adj represents the smoothed national estimates using the benchmarked data.

We fit the RW2 model to the benchmarked data in each area. We compare the results in Figure S1.259 to S1.263. Figure S1.259 compares the smoothed estimates against the direct estimates. Figure S1.260 and Figure S1.261 show the posterior median estimates of U5MR in each region over time and the reductions from 1990 period respectively. Figure S1.262 shows the smoothed estimates by region over time and Figure S1.263 compares the smoothed estimates with direct estimates from each survey for each region over time.

We further assess the RW2 model by holding out some observations, and compare the projections to the direct estimates in these holdout observations. Figure S1.264 compares the predicted estimates for the out-of-sample observations with the direct estimates by holding out observations from each area in each time period. Figure S1.265 compares the histogram of the bias rescaled by the total variance in the cross validation studies. Figure S1.266 compares the rescaled bias by region and time periods.

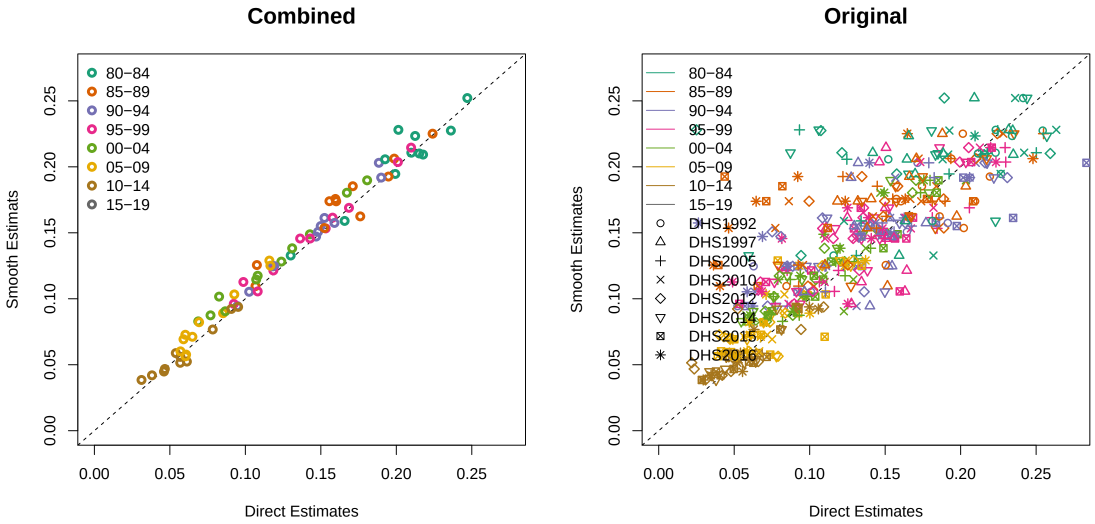

Figure S1.259: Senegal: Smooth versus direct Admin 1 estimates. Left: Combined (meta-analysis) survey estimate against combined direct estimates. Right: Combined (meta-analysis) survey estimate against direct estimates from each survey.

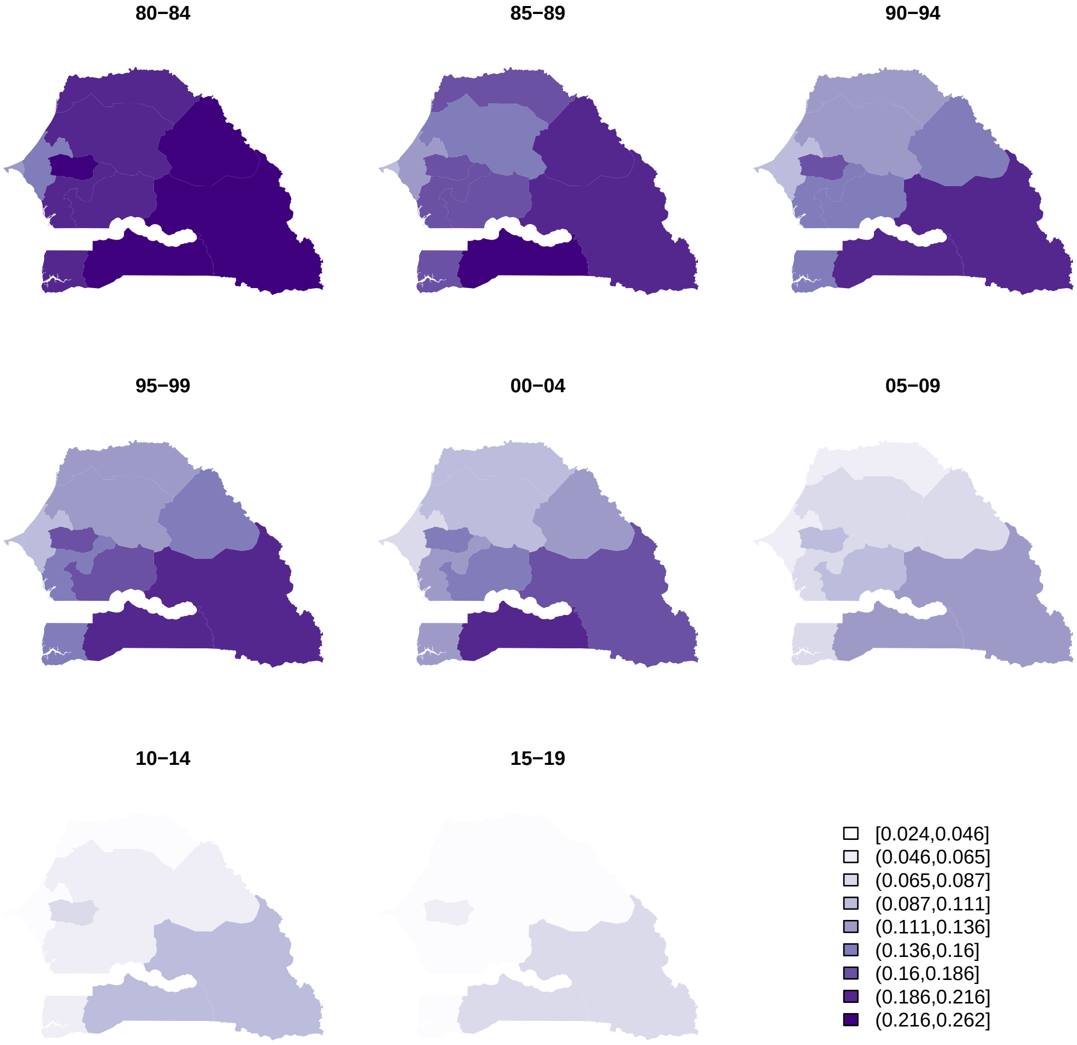

Figure S1.260: Senegal: Maps of posterior medians over time.

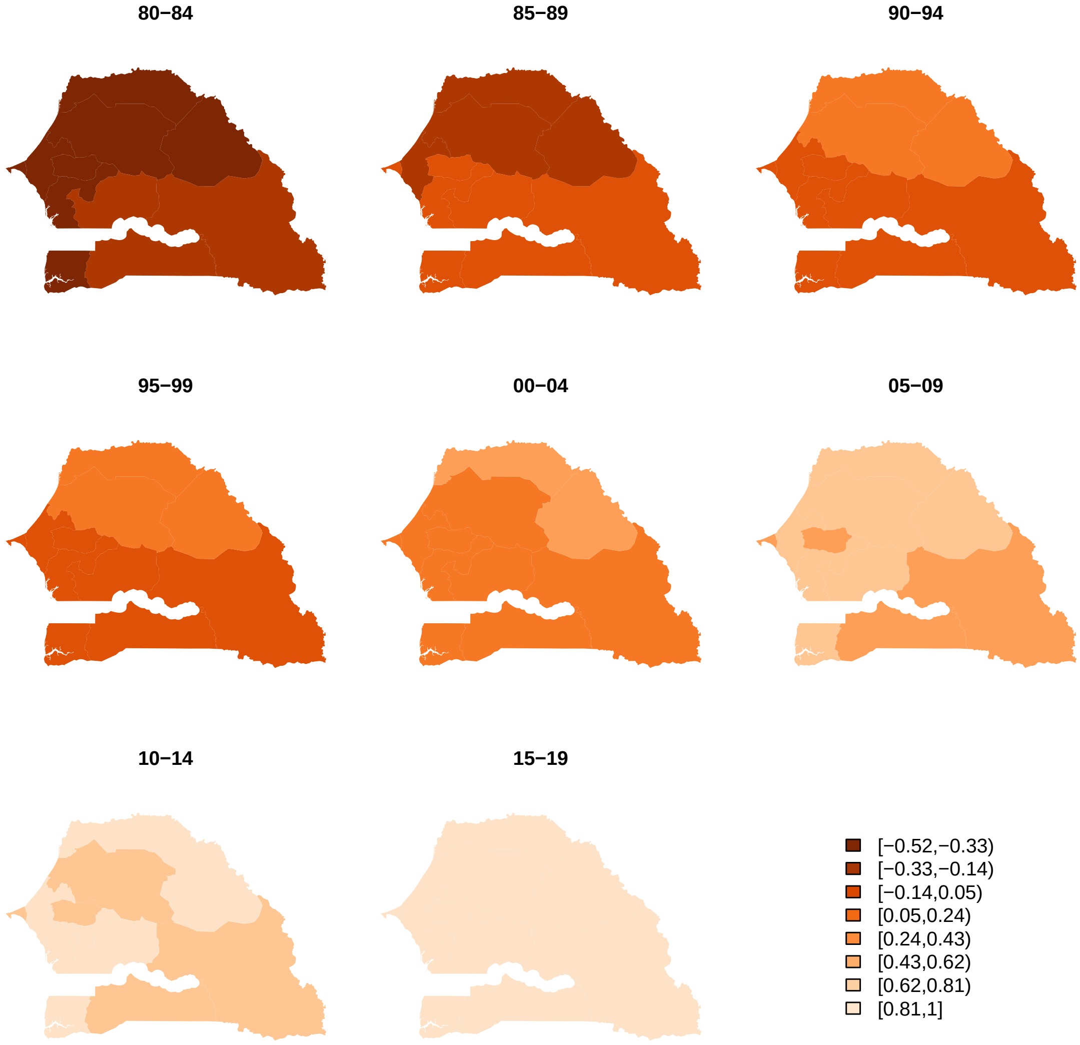

Figure S1.261: Senegal: Maps of reduction of posterior median U5MR in each five-year period compared to 1990 over time.

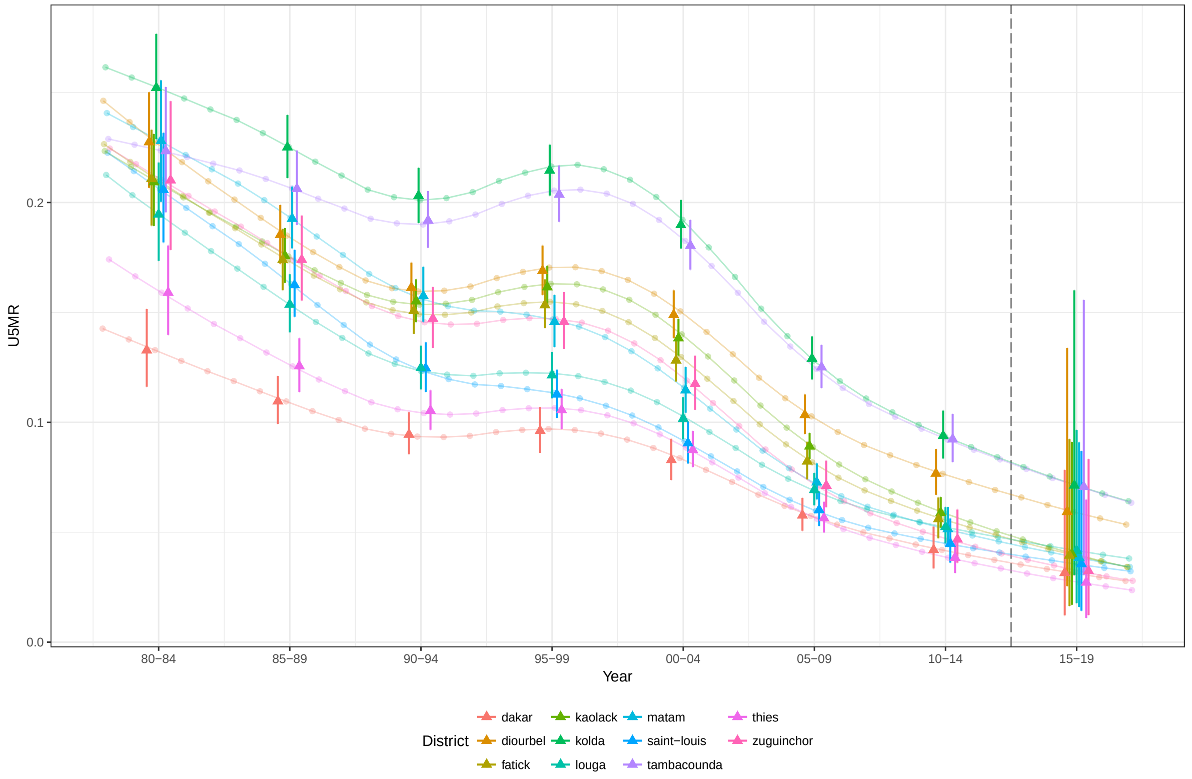

Figure S1.262: Senegal: Smoothed regional estimates over time. The line indicates yearly posterior median estimates and error bars indicate 95 % posterior credible interval at each time period.

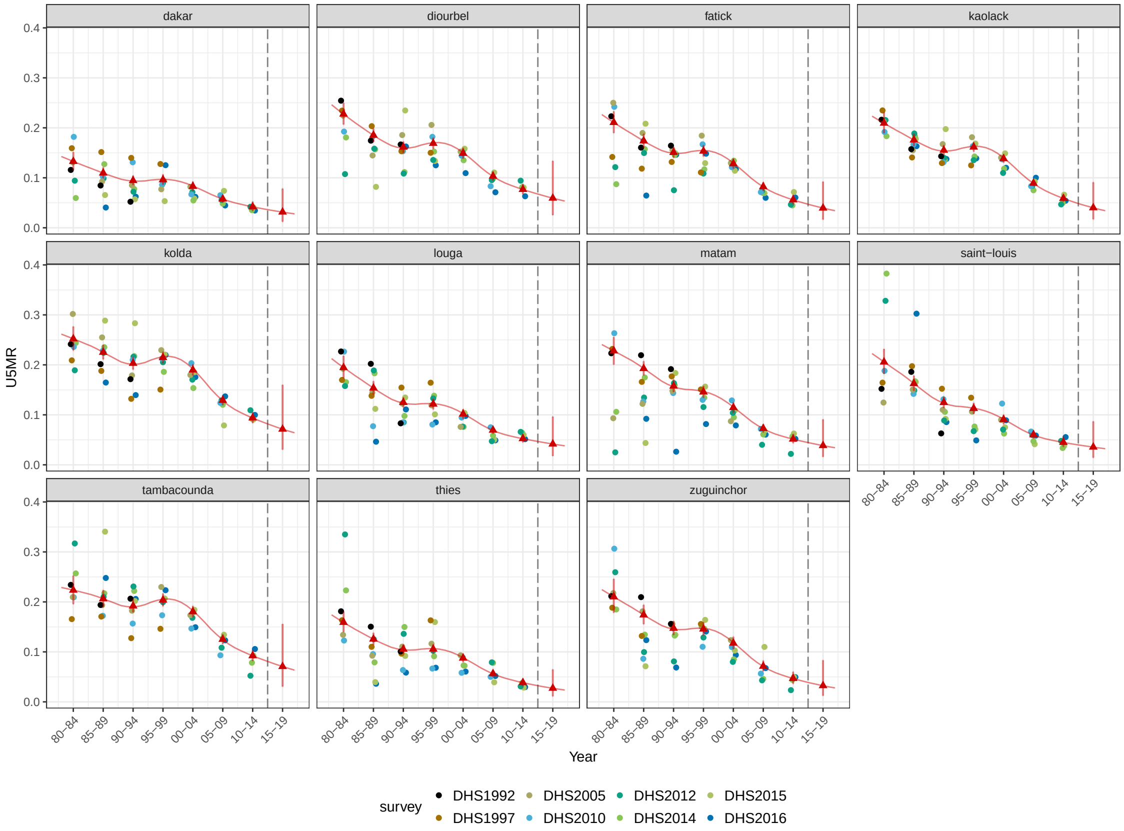

Figure S1.263: Senegal: Smoothed regional estimates over time compared to the direct estimates from each surveys. Direct estimates are not benchmarked with UN estimates. The line indicates posterior median and error bars indicate 95% posterior credible interval.

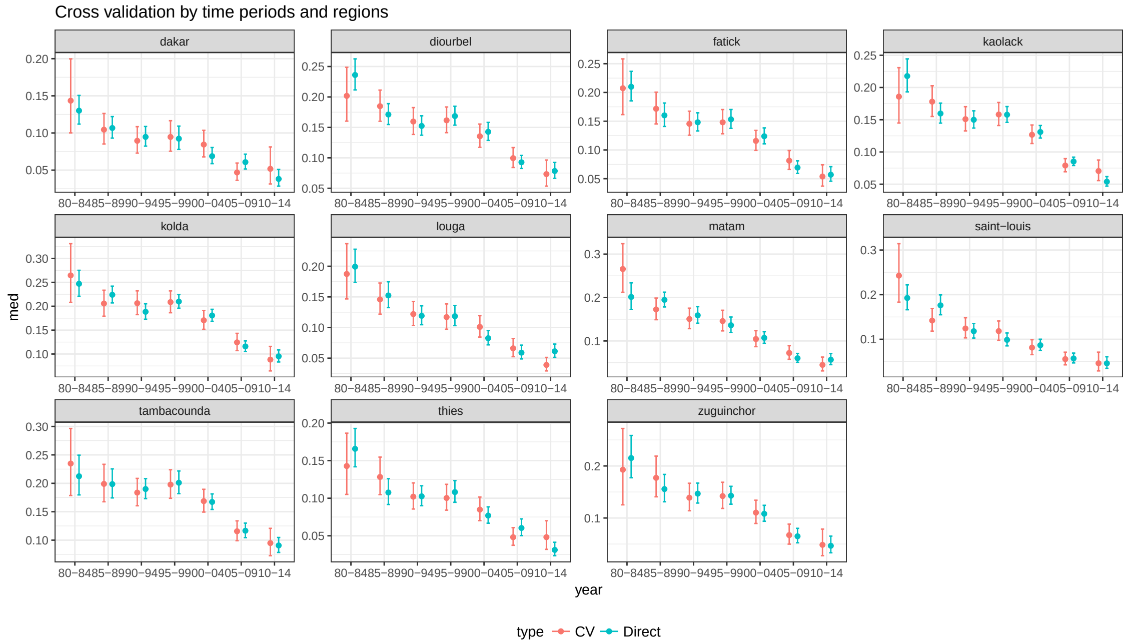

Figure S1.264: Senegal: Out-of-sample predictions along with direct estimates in the cross validation study where data from one region in each time period is held out and predicted using the rest of the data.

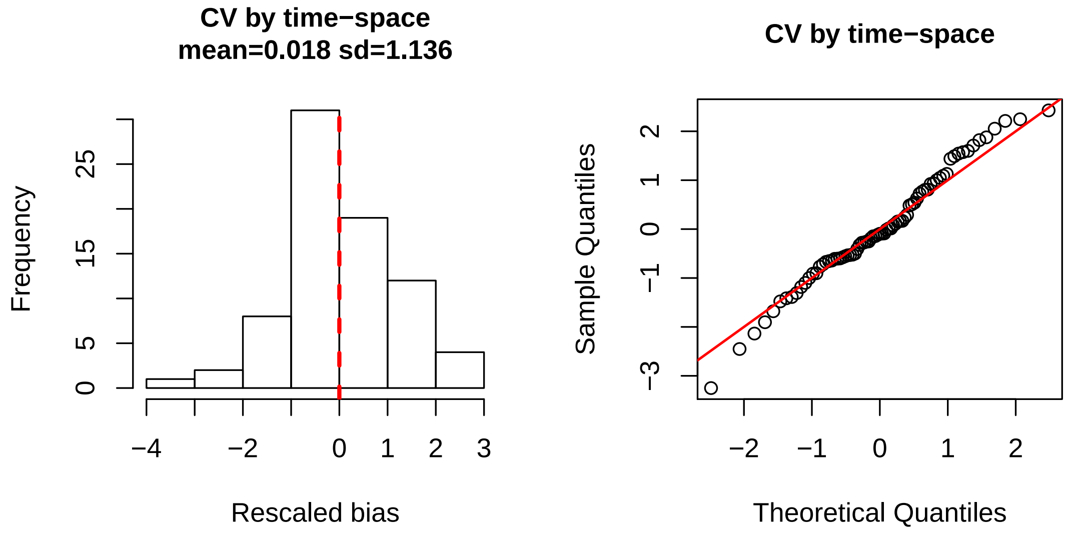

Figure S1.265: Senegal: Histogram and QQ-plot of the rescaled difference between the smoothed estimates and the direct estimates in the cross validation study. The differences between the two estimates are rescaled by the square root of the total variance of the two estimates.

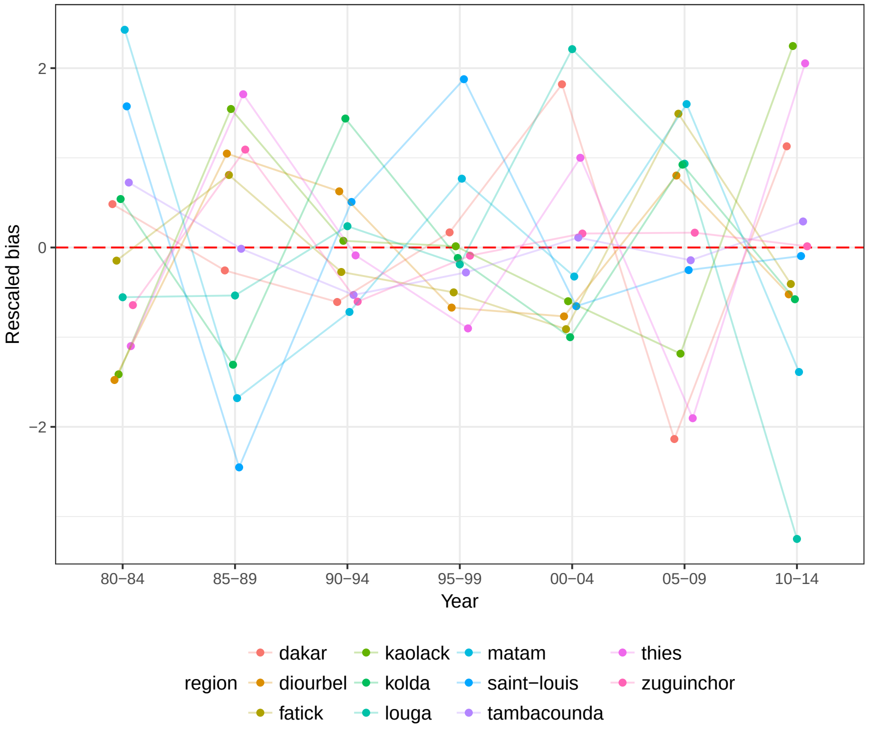

Figure S1.266: Senegal: Line plot of the difference between smoothed estimates and the direct estimates in the cross validation study. The differences between the two estimates are rescaled by the square root of the total variance of the two estimates.

3.5.30 Sierra Leone

DHS surveys were conducted in Sierra Leone in 2013.

We fit both the RW2 only model to the combined national data, and compare the time trend at national level with the estimates produced by the UN and IHME in Figure S1.267. We then adjusted the combined national data to the UN estimates of U5MR, and refit the models on the benchmarked data.

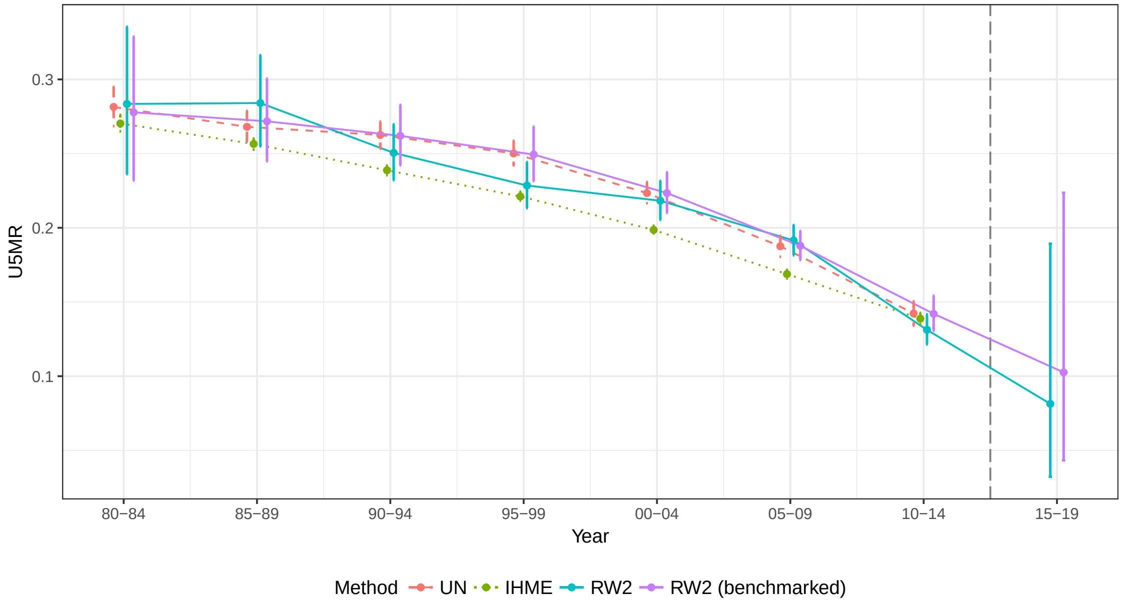

Figure S1.267: Sierra Leone: Temporal national trends along with UN (B3) estimates described in You et al. (2015) and IHME estimates based on GBD 2015 Child Mortality Collaborators (2016). RW2 represents the smoothed national estimates using the original data before benchmarking with UN estimates. RW2-adj represents the smoothed national estimates using the benchmarked data.

We fit the RW2 model to the benchmarked data in each area. We compare the results in Figure S1.268 to S1.272. Figure S1.268 compares the smoothed estimates against the direct estimates. Figure S1.269 and Figure S1.270 show the posterior median estimates of U5MR in each region over time and the reductions from 1990 period respectively. Figure S1.271 shows the smoothed estimates by region over time and Figure S1.272 compares the smoothed estimates with direct estimates from each survey for each region over time.

We further assess the RW2 model by holding out some observations, and compare the projections to the direct estimates in these holdout observations. Figure S1.273 compares the predicted estimates for the out-of-sample observations with the direct estimates by holding out observations from each area in each time period. Figure S1.274 compares the histogram of the bias rescaled by the total variance in the cross validation studies. Figure S1.275 compares the rescaled bias by region and time periods.

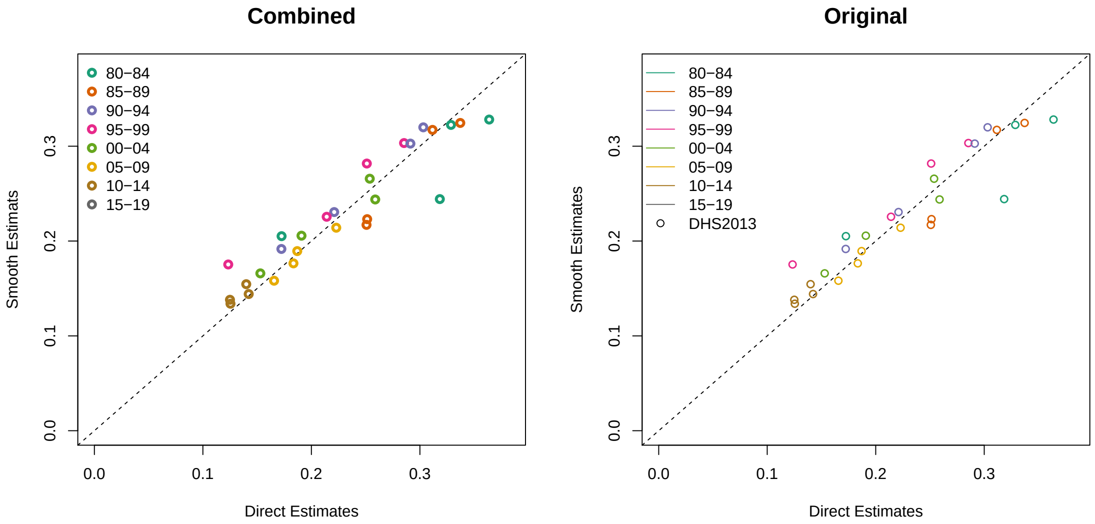

Figure S1.268: Sierra Leone: Smooth versus direct Admin 1 estimates. Left: Combined (meta-analysis) survey estimate against combined direct estimates. Right: Combined (meta-analysis) survey estimate against direct estimates from each survey.

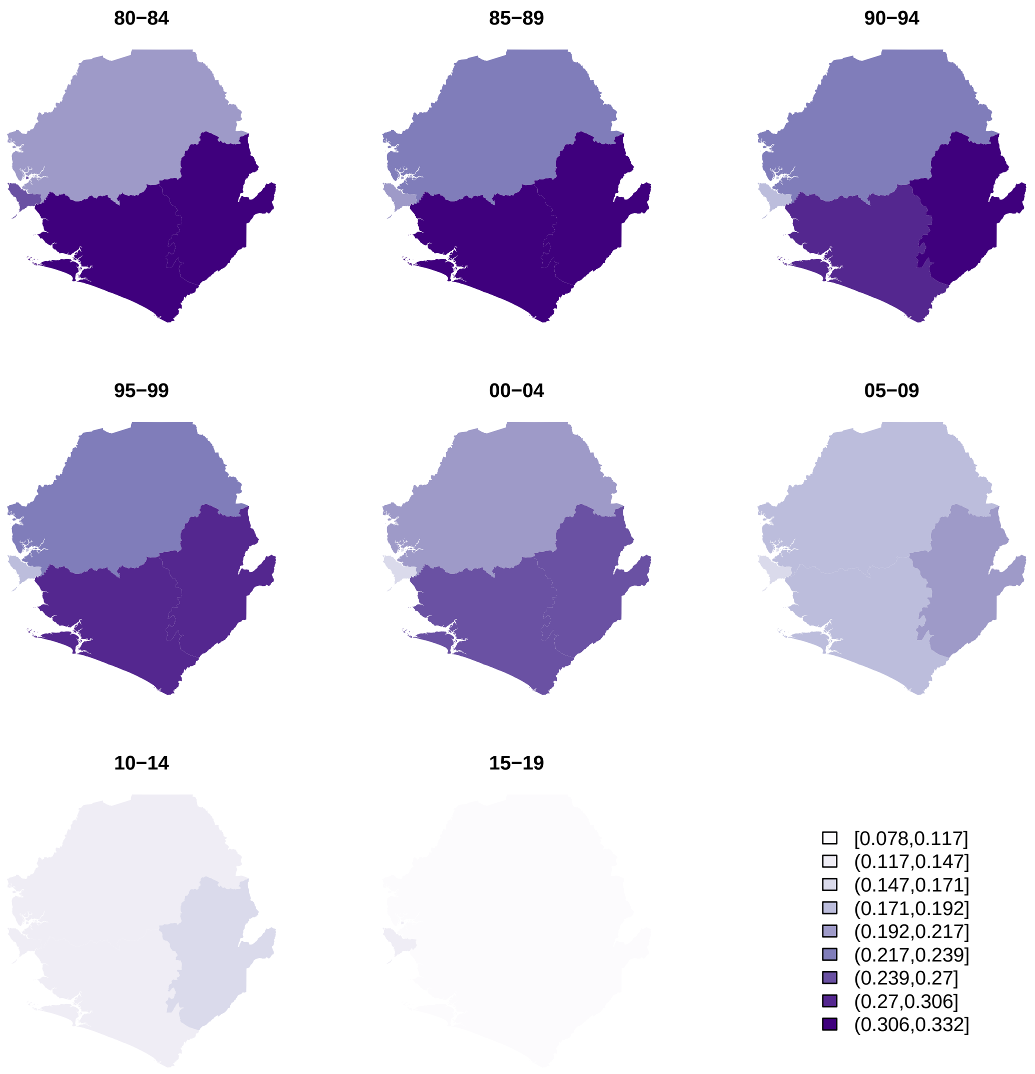

Figure S1.269: Sierra Leone: Maps of posterior medians over time.

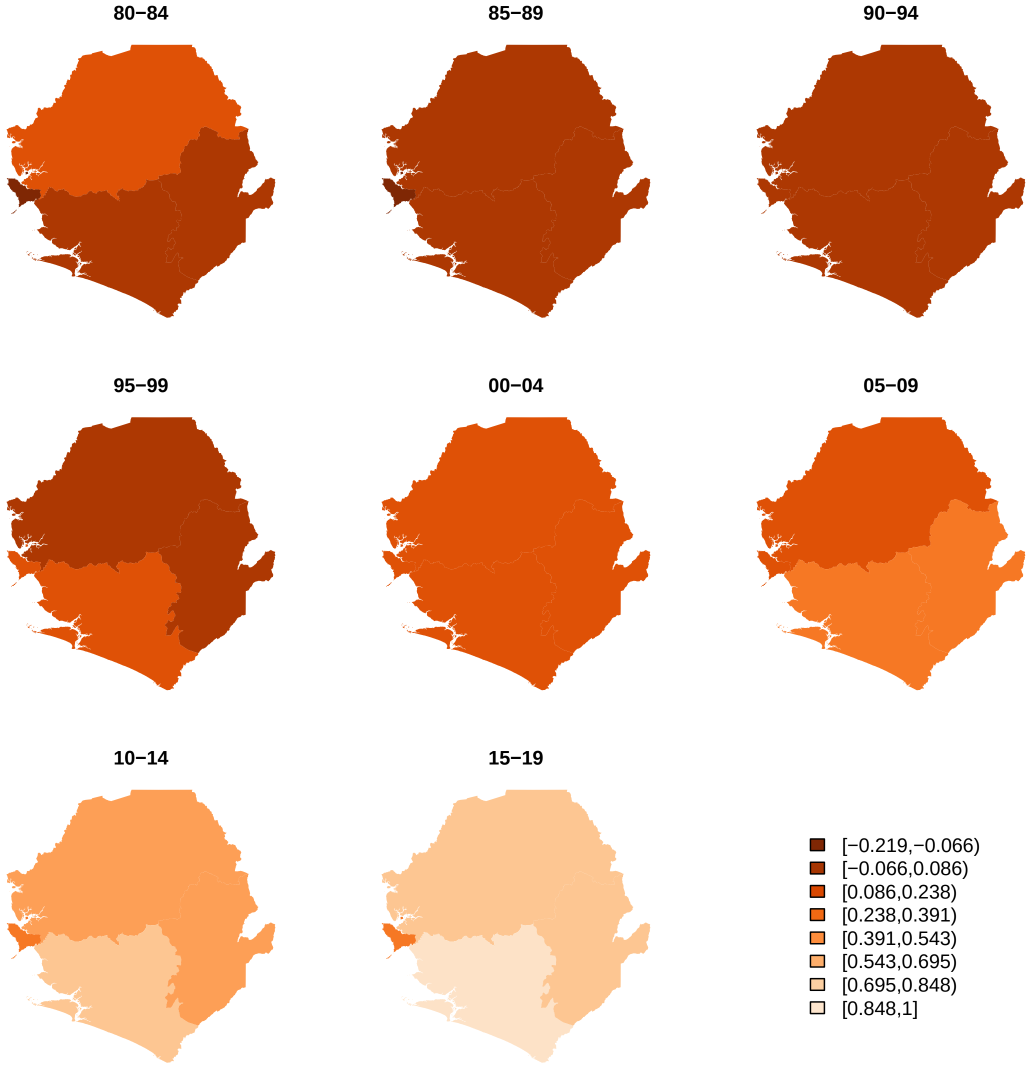

Figure S1.270: Sierra Leone: Maps of reduction of posterior median U5MR in each five-year period compared to 1990 over time.

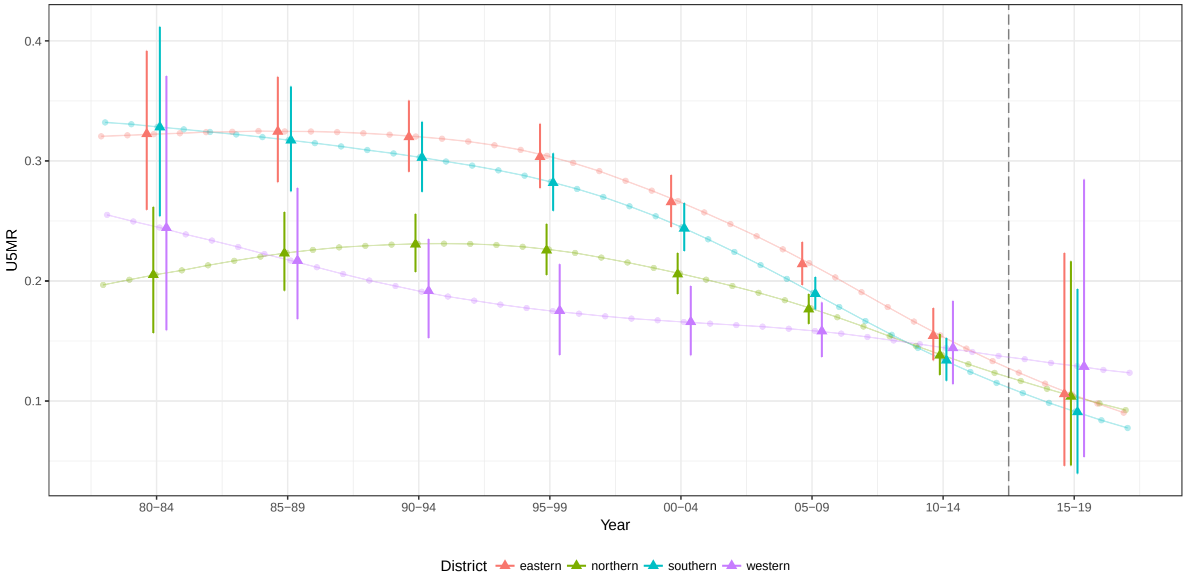

Figure S1.271: Sierra Leone: Smoothed regional estimates over time. The line indicates yearly posterior median estimates and error bars indicate 95 % posterior credible interval at each time period.

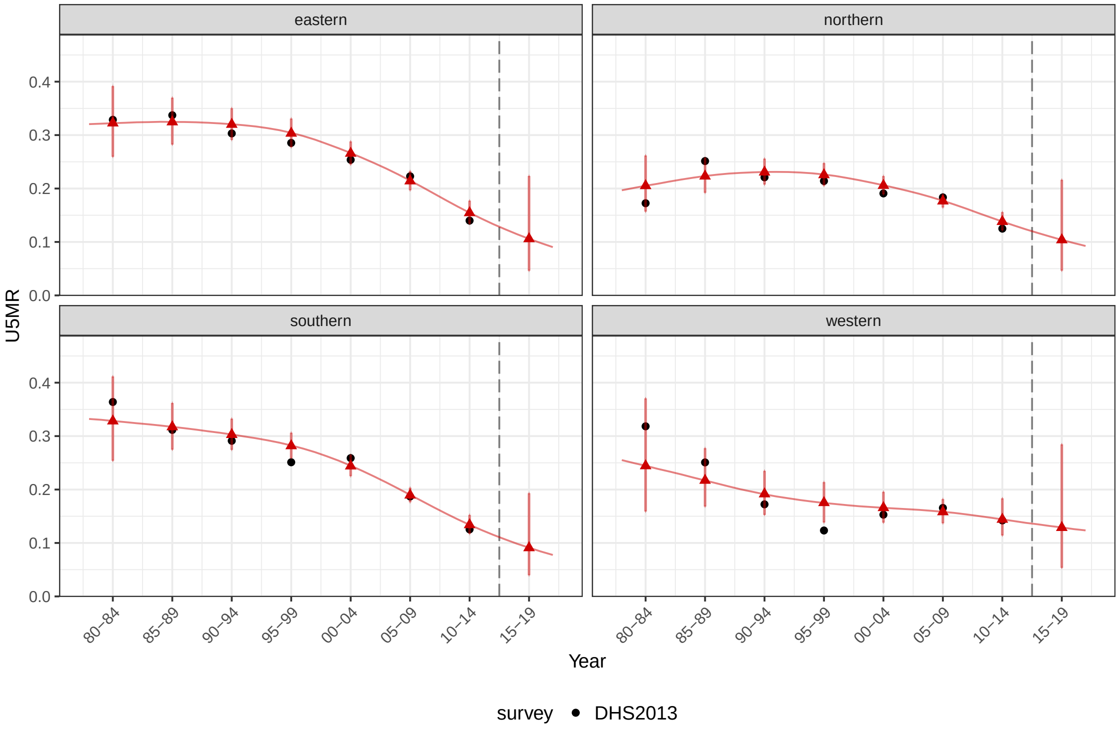

Figure S1.272: Sierra Leone: Smoothed regional estimates over time compared to the direct estimates from each surveys. Direct estimates are not benchmarked with UN estimates. The line indicates posterior median and error bars indicate 95% posterior credible interval.

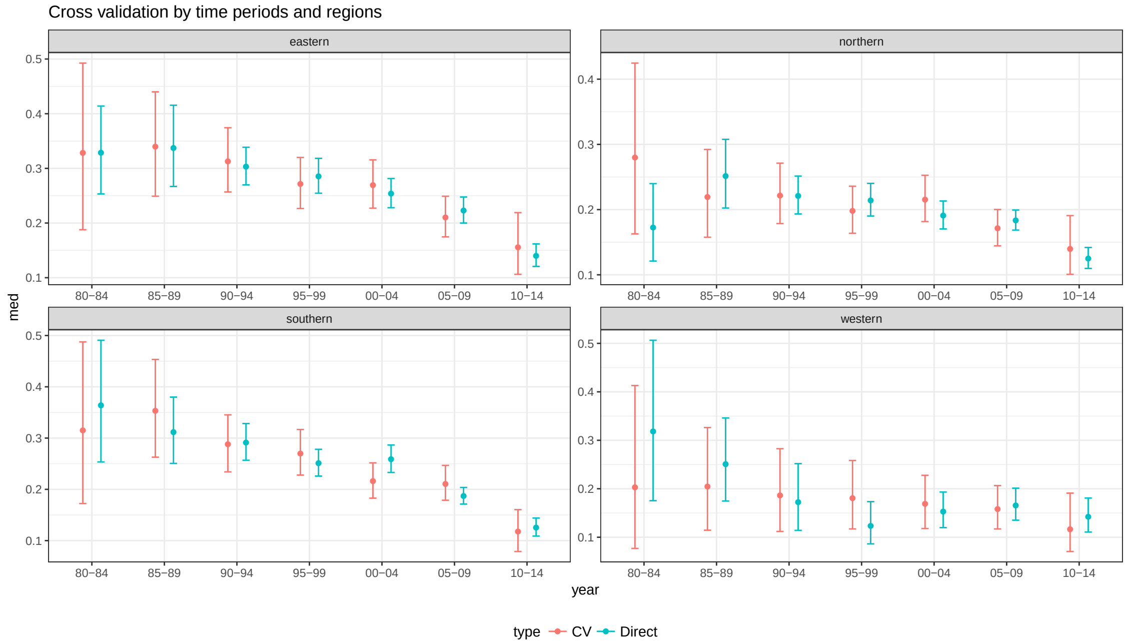

Figure S1.273: Sierra Leone: Out-of-sample predictions along with direct estimates in the cross validation study where data from one region in each time period is held out and predicted using the rest of the data.

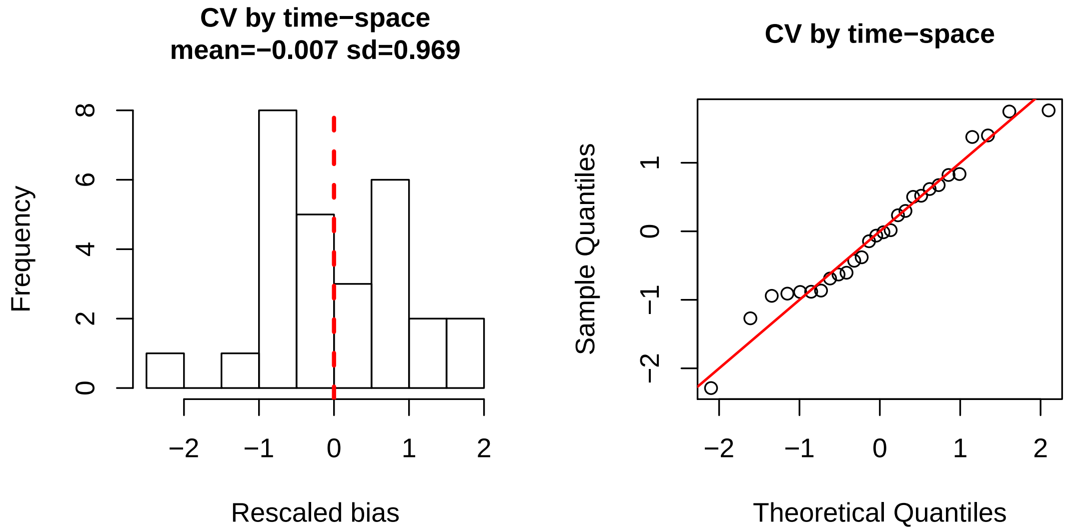

Figure S1.274: Sierra Leone: Histogram and QQ-plot of the rescaled difference between the smoothed estimates and the direct estimates in the cross validation study. The differences between the two estimates are rescaled by the square root of the total variance of the two estimates.

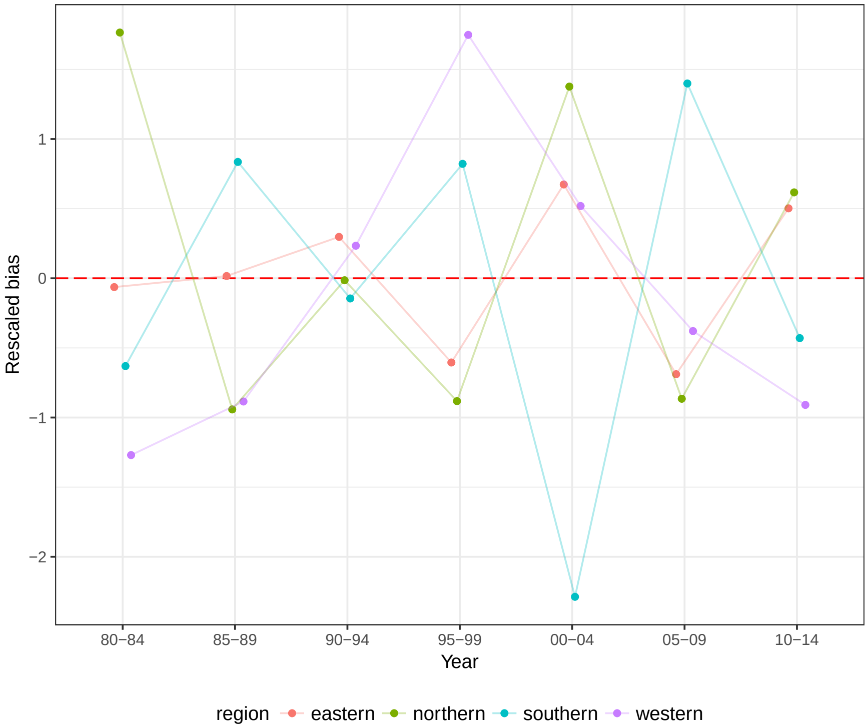

Figure S1.275: Sierra Leone: Line plot of the difference between smoothed estimates and the direct estimates in the cross validation study. The differences between the two estimates are rescaled by the square root of the total variance of the two estimates.

3.5.31 Tanzania

DHS surveys were conducted in Tanzania in 1996, 1999, 2005, 2010, and 2015.

We fit both the RW2 only model to the combined national data, and compare the time trend at national level with the estimates produced by the UN and IHME in Figure S1.276. We then adjusted the combined national data to the UN estimates of U5MR, and refit the models on the benchmarked data.

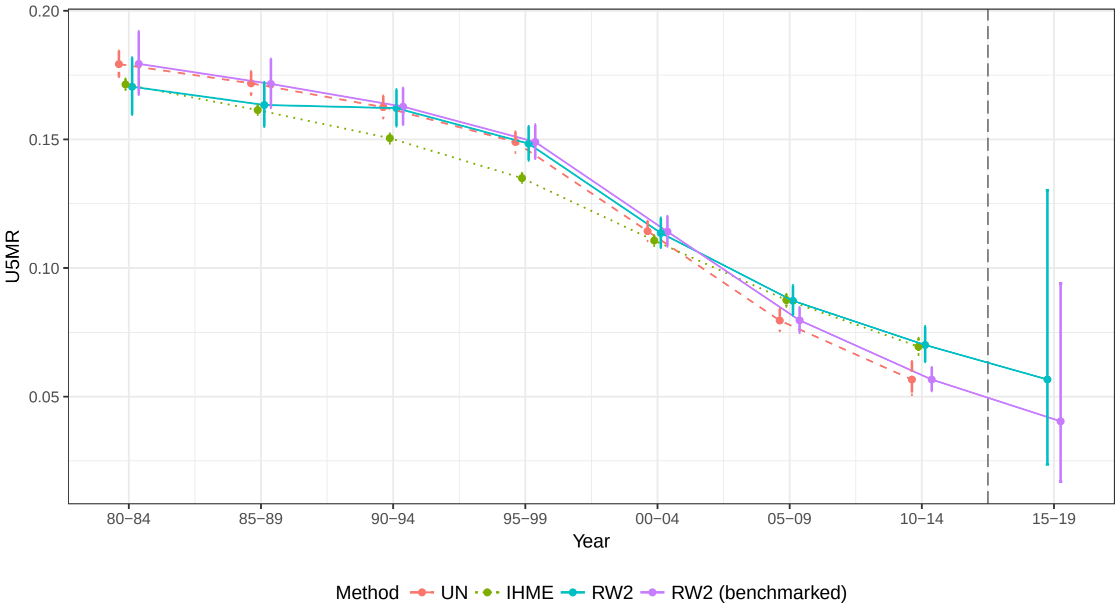

Figure S1.276: Tanzania: Temporal national trends along with UN (B3) estimates described in You et al. (2015) and IHME estimates based on GBD 2015 Child Mortality Collaborators (2016). RW2 represents the smoothed national estimates using the original data before benchmarking with UN estimates. RW2-adj represents the smoothed national estimates using the benchmarked data.

We fit the RW2 model to the benchmarked data in each area. We compare the results in Figure S1.277 to S1.281. Figure S1.277 compares the smoothed estimates against the direct estimates. Figure S1.278 and Figure S1.279 show the posterior median estimates of U5MR in each region over time and the reductions from 1990 period respectively. Figure S1.280 shows the smoothed estimates by region over time and Figure S1.281 compares the smoothed estimates with direct estimates from each survey for each region over time.

We further assess the RW2 model by holding out some observations, and compare the projections to the direct estimates in these holdout observations. Figure S1.282 compares the predicted estimates for the out-of-sample observations with the direct estimates by holding out observations from each area in each time period. Figure S1.283 compares the histogram of the bias rescaled by the total variance in the cross validation studies. Figure S1.284 compares the rescaled bias by region and time periods.

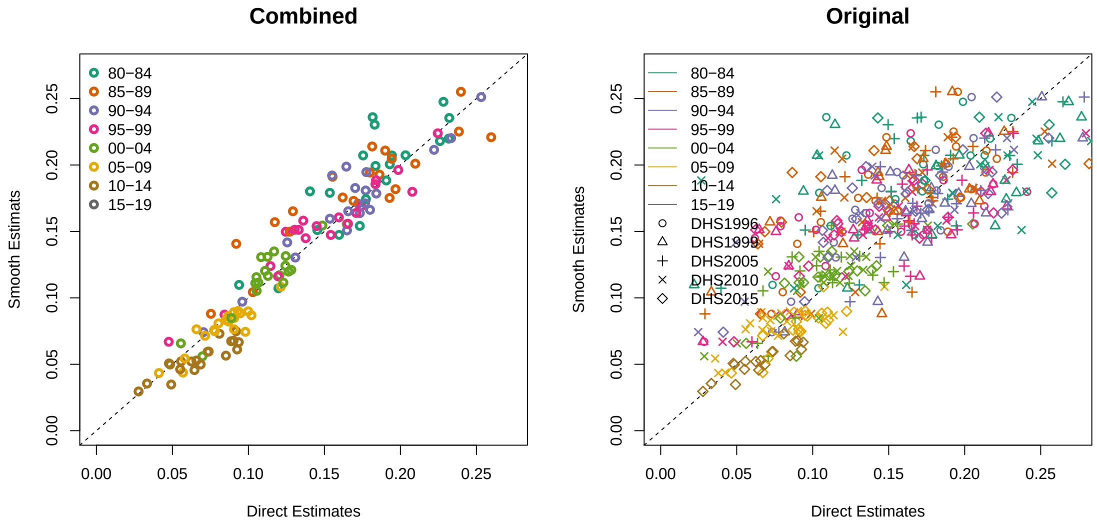

Figure S1.277: Tanzania: Smooth versus direct Admin 1 estimates. Left: Combined (meta-analysis) survey estimate against combined direct estimates. Right: Combined (meta-analysis) survey estimate against direct estimates from each survey.

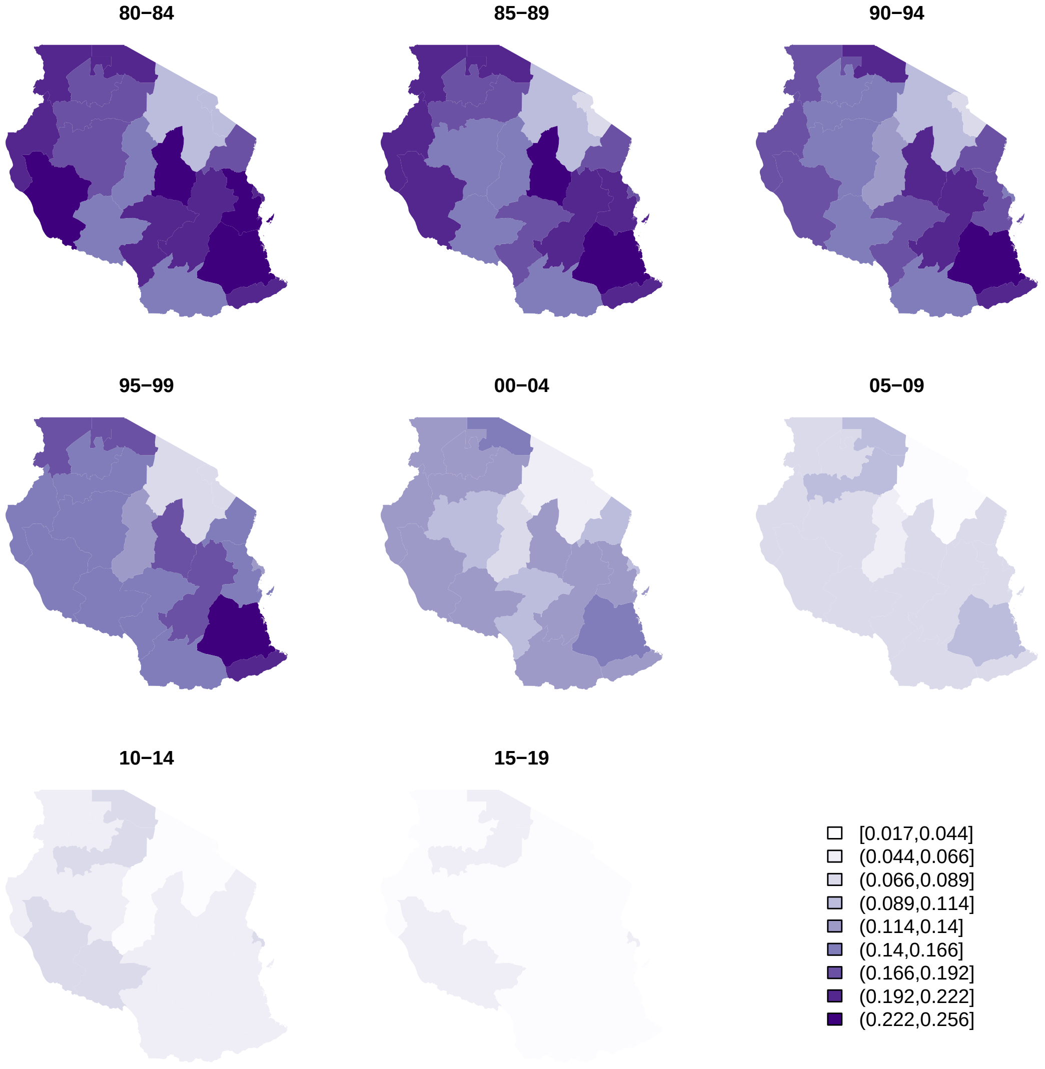

Figure S1.278: Tanzania: Maps of posterior medians over time.

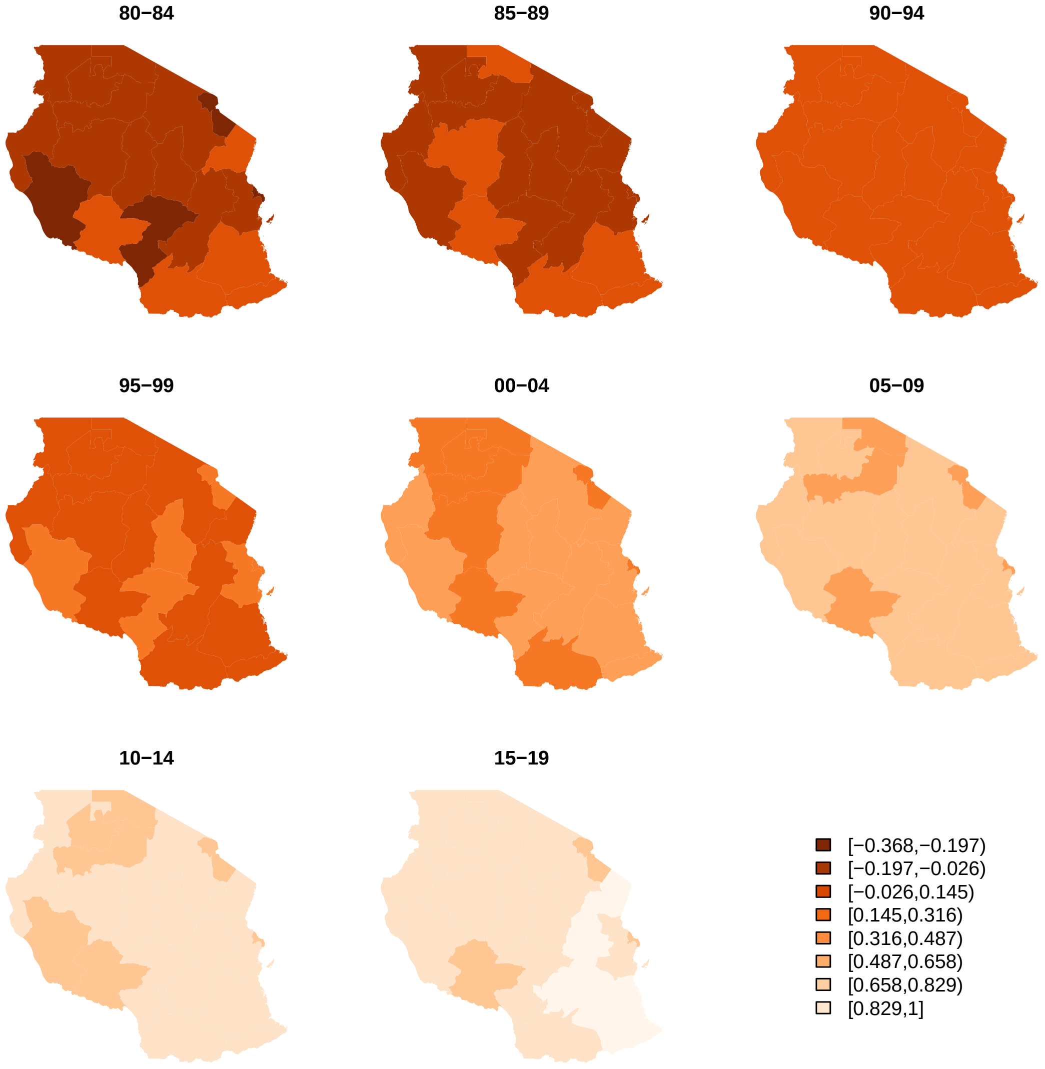

Figure S1.279: Tanzania: Maps of reduction of posterior median U5MR in each five-year period compared to 1990 over time.

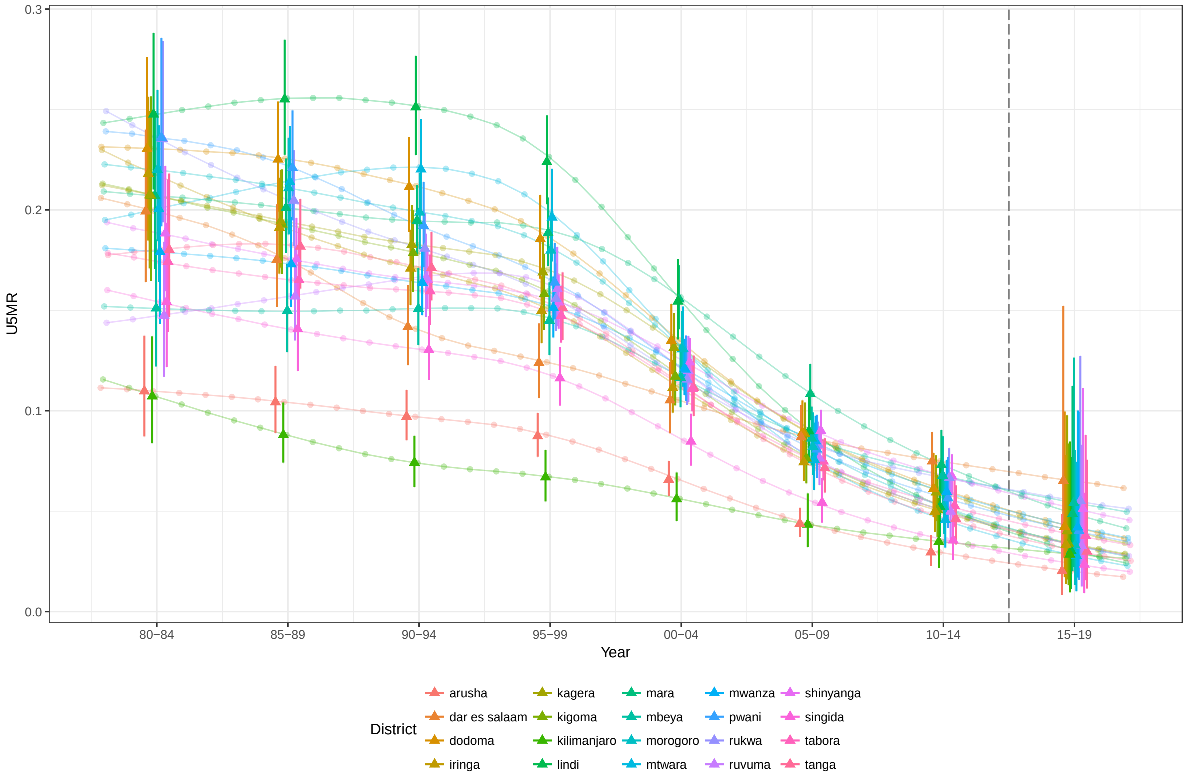

Figure S1.280: Tanzania: Smoothed regional estimates over time. The line indicates yearly posterior median estimates and error bars indicate 95 % posterior credible interval at each time period.

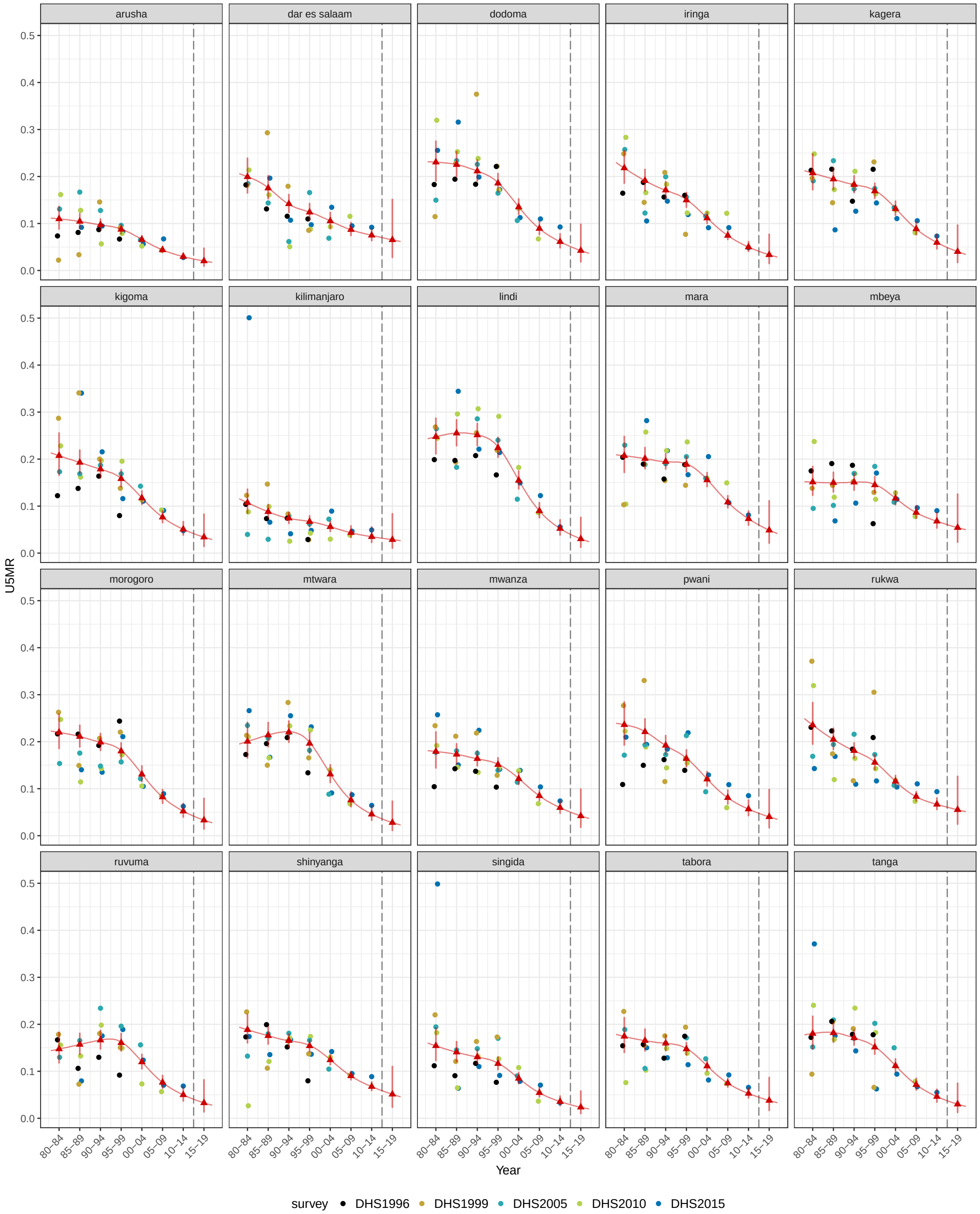

Figure S1.281: Tanzania: Smoothed regional estimates over time compared to the direct estimates from each surveys. Direct estimates are not benchmarked with UN estimates. The line indicates posterior median and error bars indicate 95% posterior credible interval.

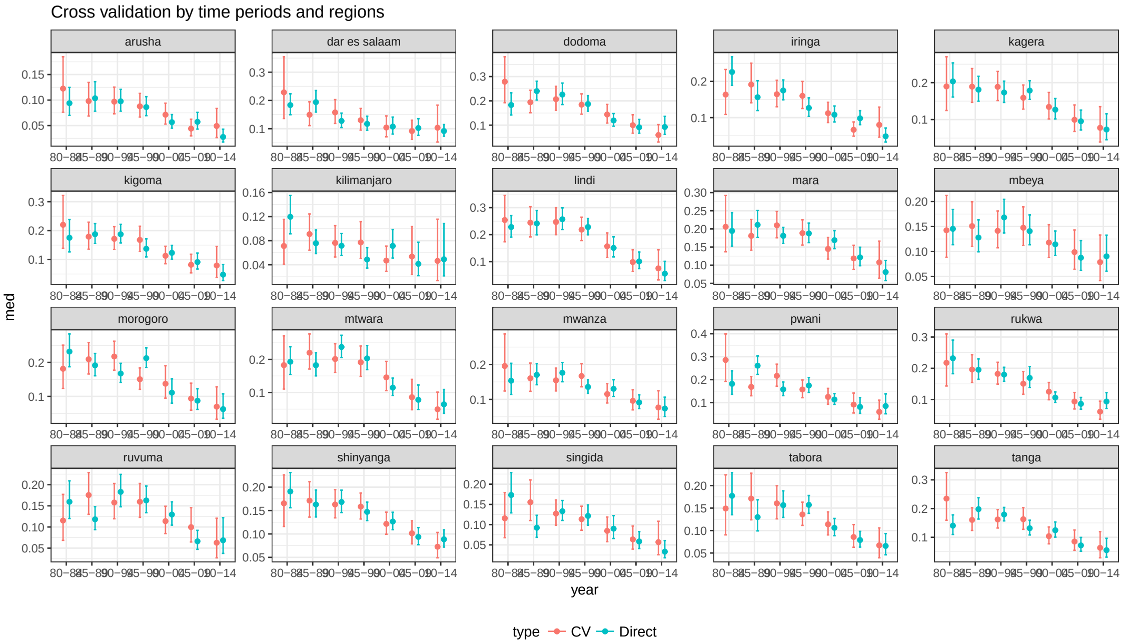

Figure S1.282: Tanzania: Out-of-sample predictions along with direct estimates in the cross validation study where data from one region in each time period is held out and predicted using the rest of the data.

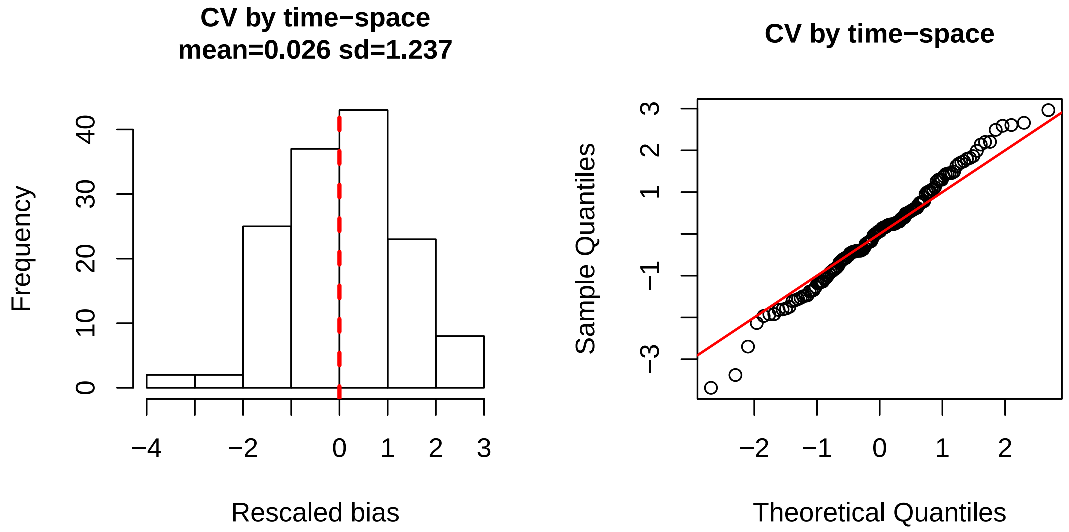

Figure S1.283: Tanzania: Histogram and QQ-plot of the rescaled difference between the smoothed estimates and the direct estimates in the cross validation study. The differences between the two estimates are rescaled by the square root of the total variance of the two estimates.

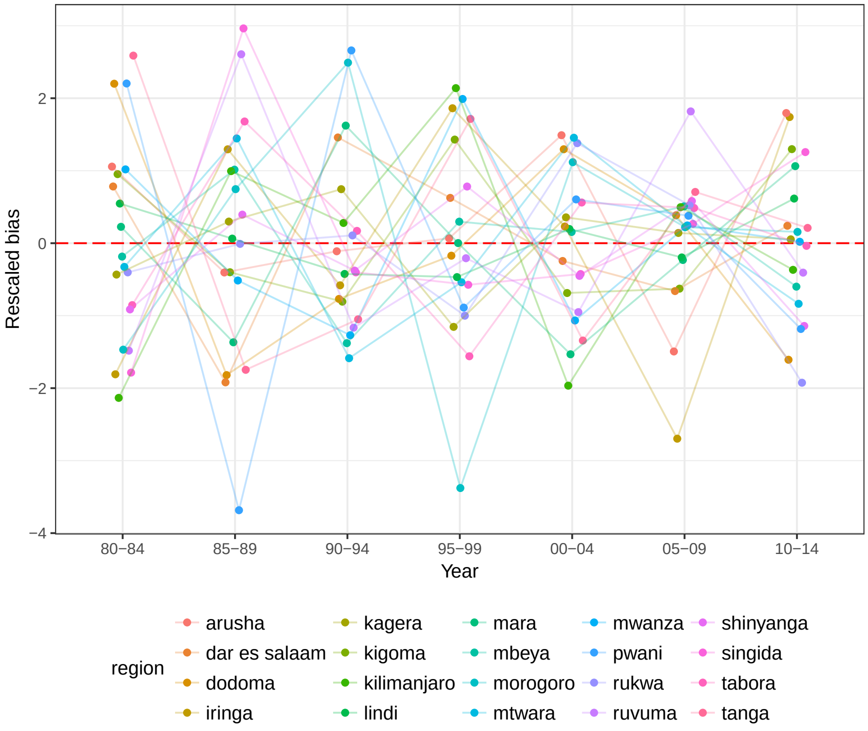

Figure S1.284: Tanzania: Line plot of the difference between smoothed estimates and the direct estimates in the cross validation study. The differences between the two estimates are rescaled by the square root of the total variance of the two estimates.

3.5.32 Togo

DHS surveys were conducted in Togo in 1998, and 2013.

We fit both the RW2 only model to the combined national data, and compare the time trend at national level with the estimates produced by the UN and IHME in Figure S1.285. We then adjusted the combined national data to the UN estimates of U5MR, and refit the models on the benchmarked data.

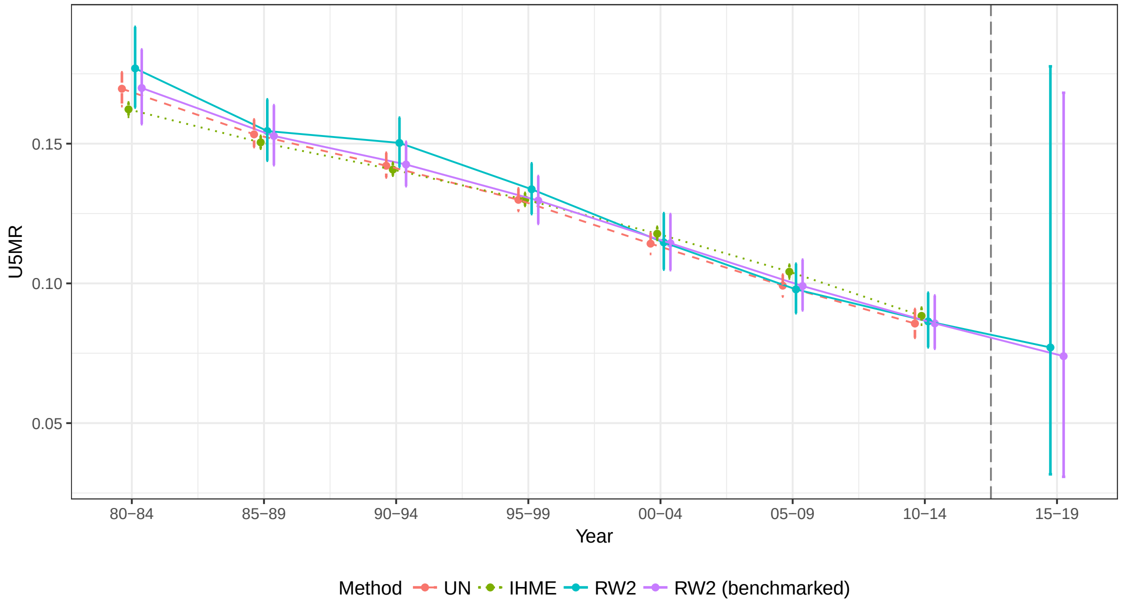

Figure S1.285: Togo: Temporal national trends along with UN (B3) estimates described in You et al. (2015) and IHME estimates based on GBD 2015 Child Mortality Collaborators (2016). RW2 represents the smoothed national estimates using the original data before benchmarking with UN estimates. RW2-adj represents the smoothed national estimates using the benchmarked data.

We fit the RW2 model to the benchmarked data in each area. We compare the results in Figure S1.286 to S1.290. Figure S1.286 compares the smoothed estimates against the direct estimates. Figure S1.287 and Figure S1.288 show the posterior median estimates of U5MR in each region over time and the reductions from 1990 period respectively. Figure S1.289 shows the smoothed estimates by region over time and Figure S1.290 compares the smoothed estimates with direct estimates from each survey for each region over time.

We further assess the RW2 model by holding out some observations, and compare the projections to the direct estimates in these holdout observations. Figure S1.291 compares the predicted estimates for the out-of-sample observations with the direct estimates by holding out observations from each area in each time period. Figure S1.292 compares the histogram of the bias rescaled by the total variance in the cross validation studies. Figure S1.293 compares the rescaled bias by region and time periods.

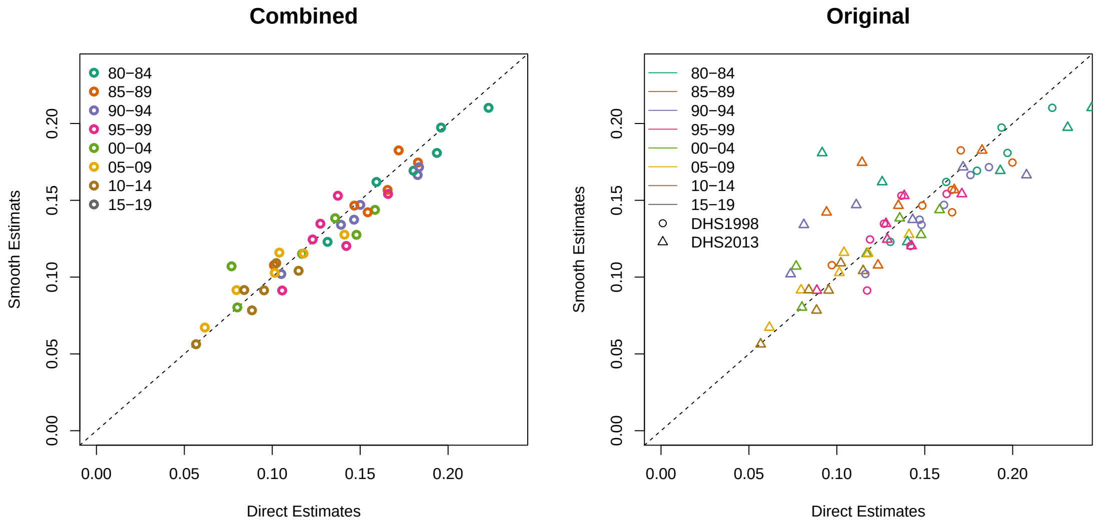

Figure S1.286: Togo: Smooth versus direct Admin 1 estimates. Left: Combined (meta-analysis) survey estimate against combined direct estimates. Right: Combined (meta-analysis) survey estimate against direct estimates from each survey.

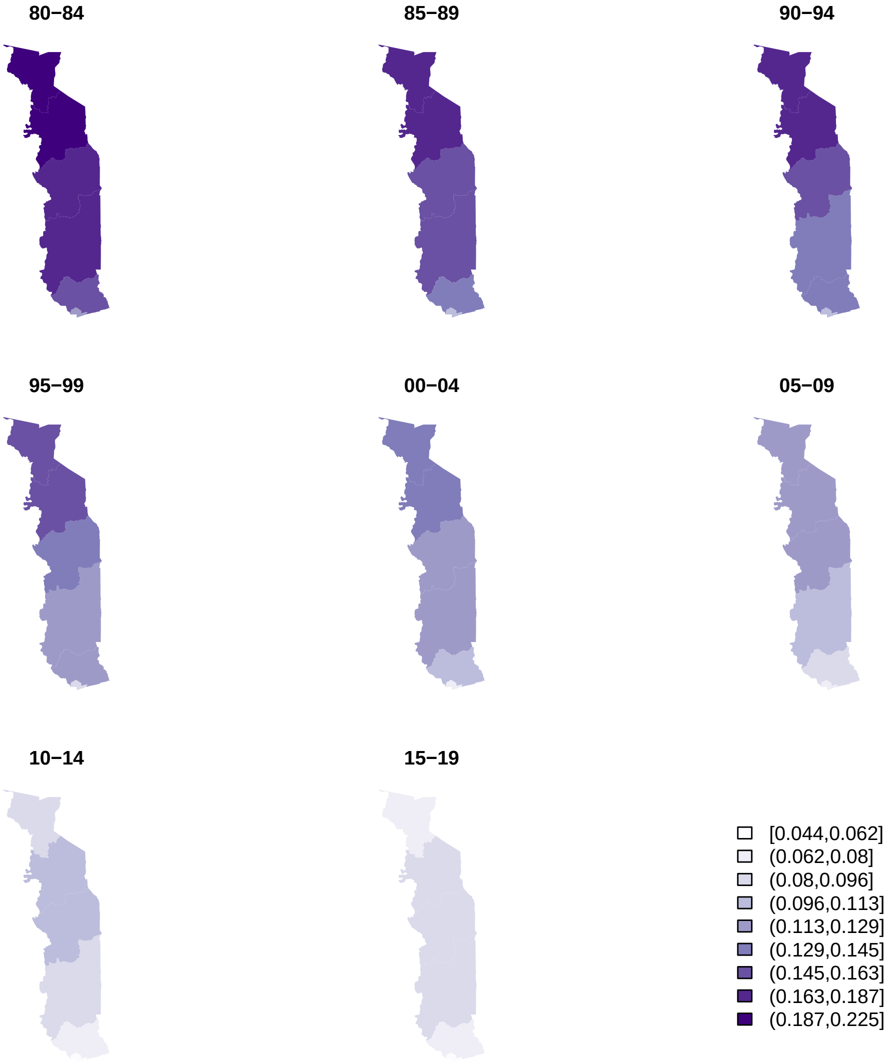

Figure S1.287: Togo: Maps of posterior medians over time.

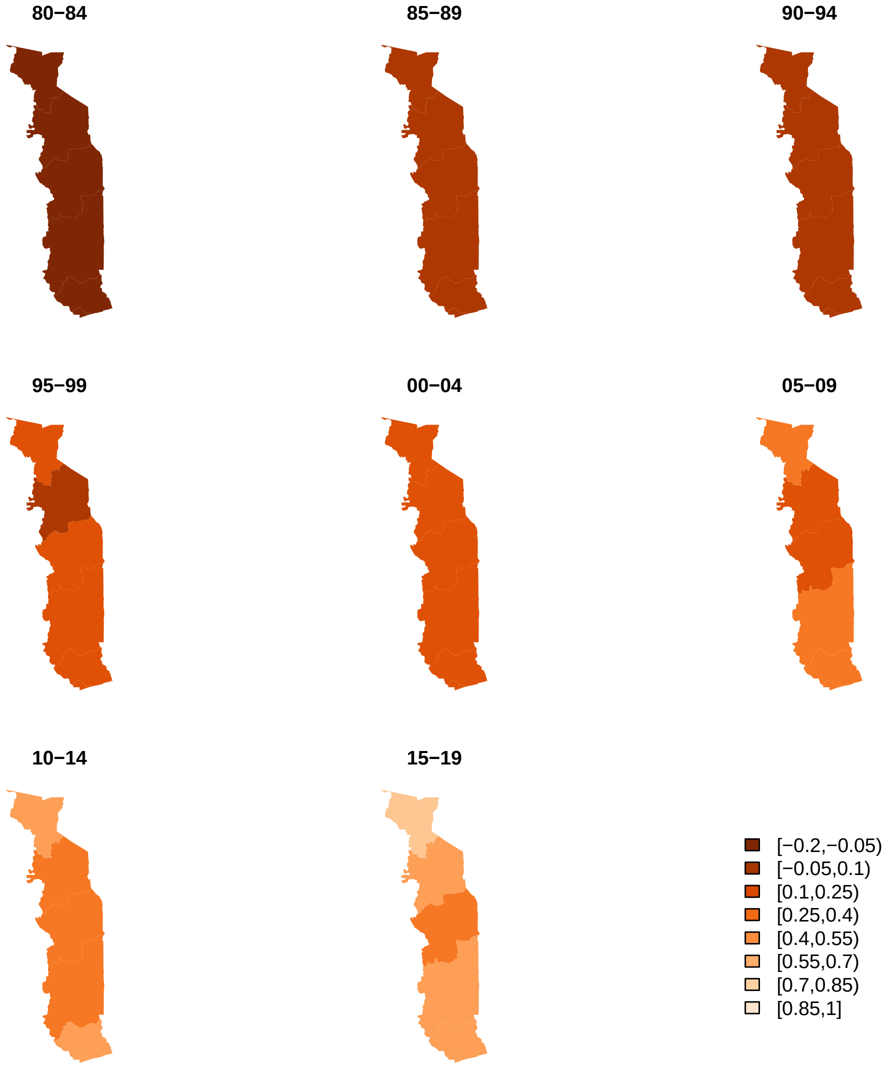

Figure S1.288: Togo: Maps of reduction of posterior median U5MR in each five-year period compared to 1990 over time.

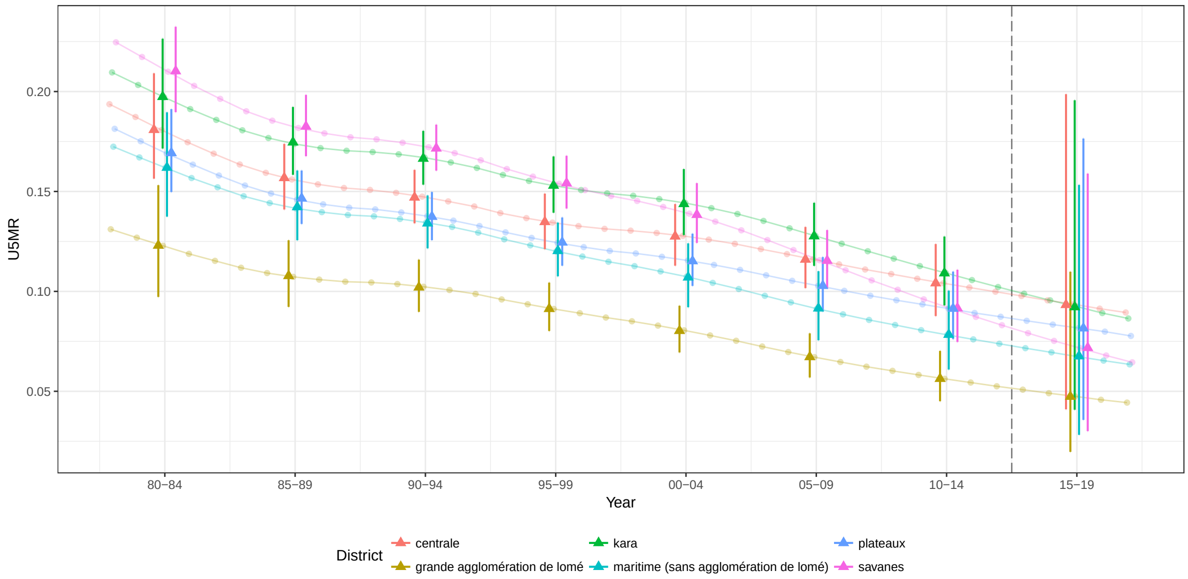

Figure S1.289: Togo: Smoothed regional estimates over time. The line indicates yearly posterior median estimates and error bars indicate 95 % posterior credible interval at each time period.

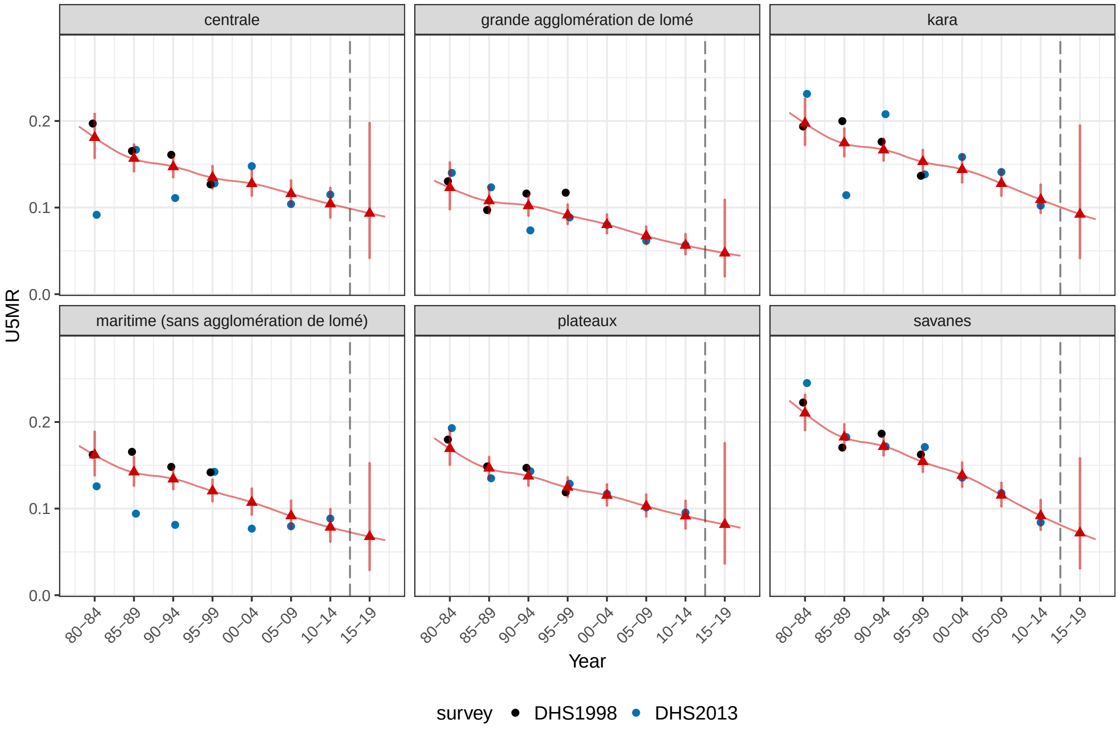

Figure S1.290: Togo: Smoothed regional estimates over time compared to the direct estimates from each surveys. Direct estimates are not benchmarked with UN estimates. The line indicates posterior median and error bars indicate 95% posterior credible interval.

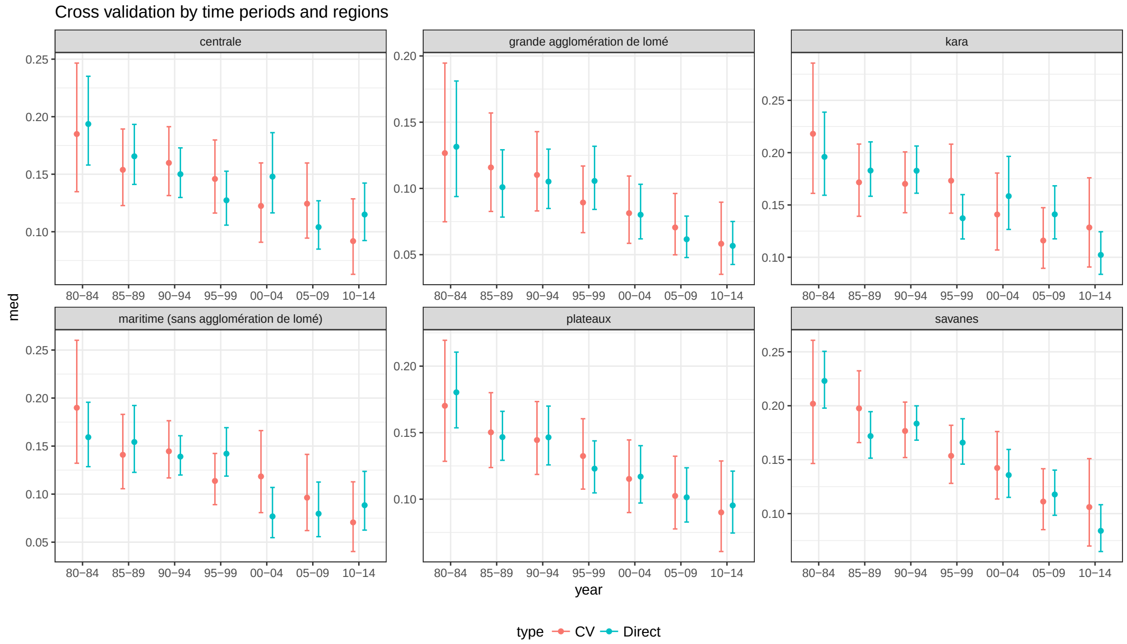

Figure S1.291: Togo: Out-of-sample predictions along with direct estimates in the cross validation study where data from one region in each time period is held out and predicted using the rest of the data.

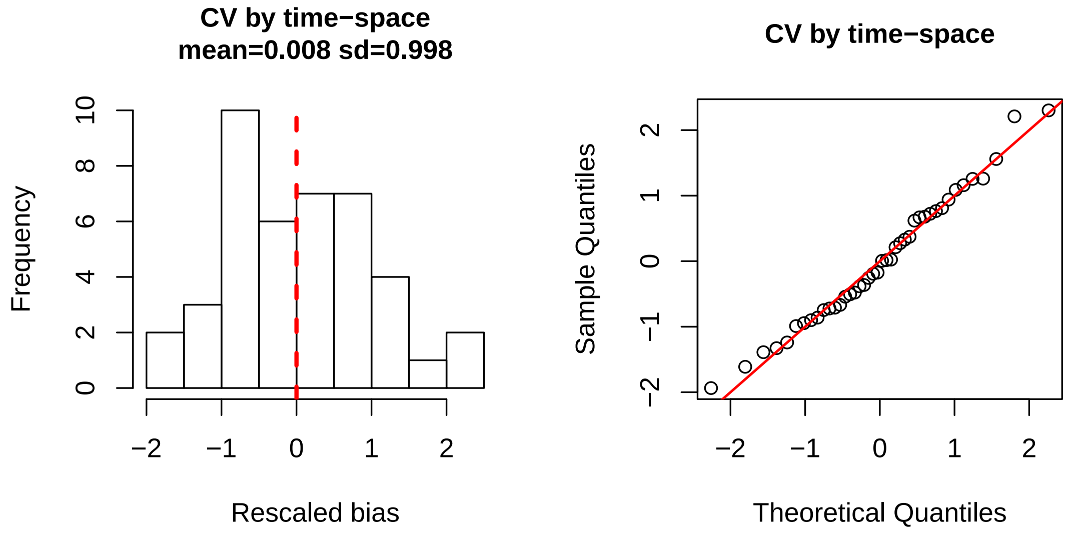

Figure S1.292: Togo: Histogram and QQ-plot of the rescaled difference between the smoothed estimates and the direct estimates in the cross validation study. The differences between the two estimates are rescaled by the square root of the total variance of the two estimates.

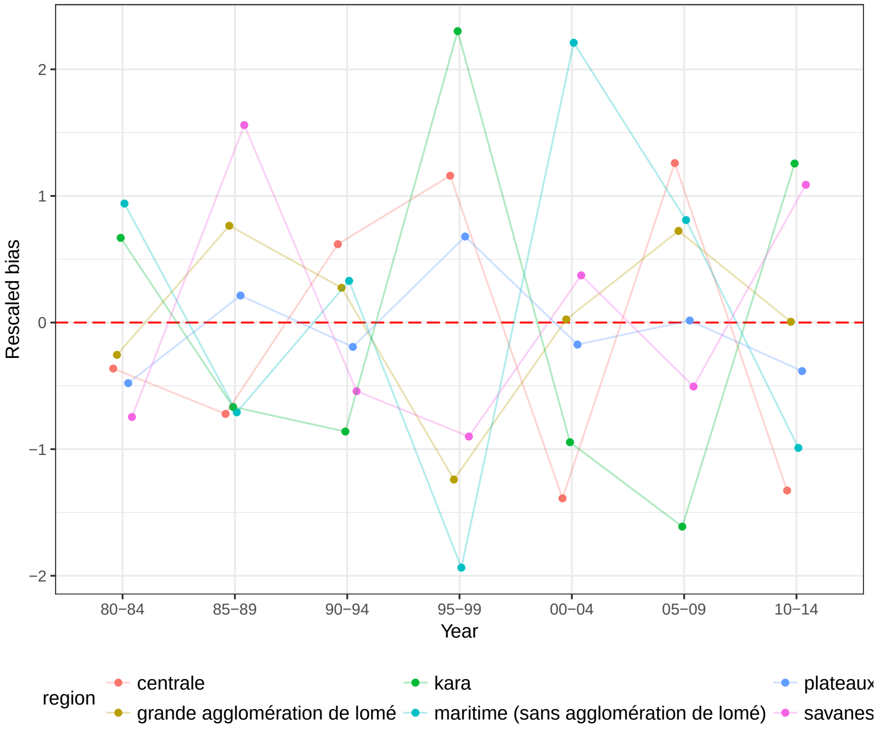

Figure S1.293: Togo: Line plot of the difference between smoothed estimates and the direct estimates in the cross validation study. The differences between the two estimates are rescaled by the square root of the total variance of the two estimates.

3.5.33 Uganda

DHS surveys were conducted in Uganda in 1989, 1995, 2001, 2006, and 2011.

We fit both the RW2 only model to the combined national data, and compare the time trend at national level with the estimates produced by the UN and IHME in Figure S1.294. We then adjusted the combined national data to the UN estimates of U5MR, and refit the models on the benchmarked data.

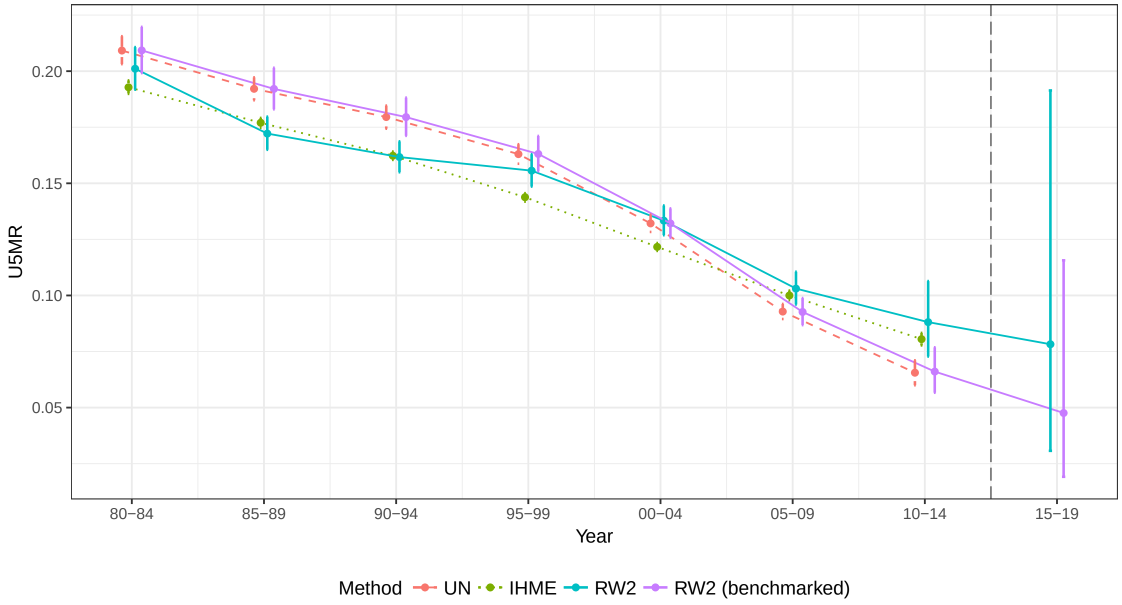

Figure S1.294: Uganda: Temporal national trends along with UN (B3) estimates described in You et al. (2015) and IHME estimates based on GBD 2015 Child Mortality Collaborators (2016). RW2 represents the smoothed national estimates using the original data before benchmarking with UN estimates. RW2-adj represents the smoothed national estimates using the benchmarked data.

We fit the RW2 model to the benchmarked data in each area. We compare the results in Figure S1.295 to S1.299. Figure S1.295 compares the smoothed estimates against the direct estimates. Figure S1.296 and Figure S1.297 show the posterior median estimates of U5MR in each region over time and the reductions from 1990 period respectively. Figure S1.298 shows the smoothed estimates by region over time and Figure S1.299 compares the smoothed estimates with direct estimates from each survey for each region over time.

We further assess the RW2 model by holding out some observations, and compare the projections to the direct estimates in these holdout observations. Figure S1.300 compares the predicted estimates for the out-of-sample observations with the direct estimates by holding out observations from each area in each time period. Figure S1.301 compares the histogram of the bias rescaled by the total variance in the cross validation studies. Figure S1.302 compares the rescaled bias by region and time periods.

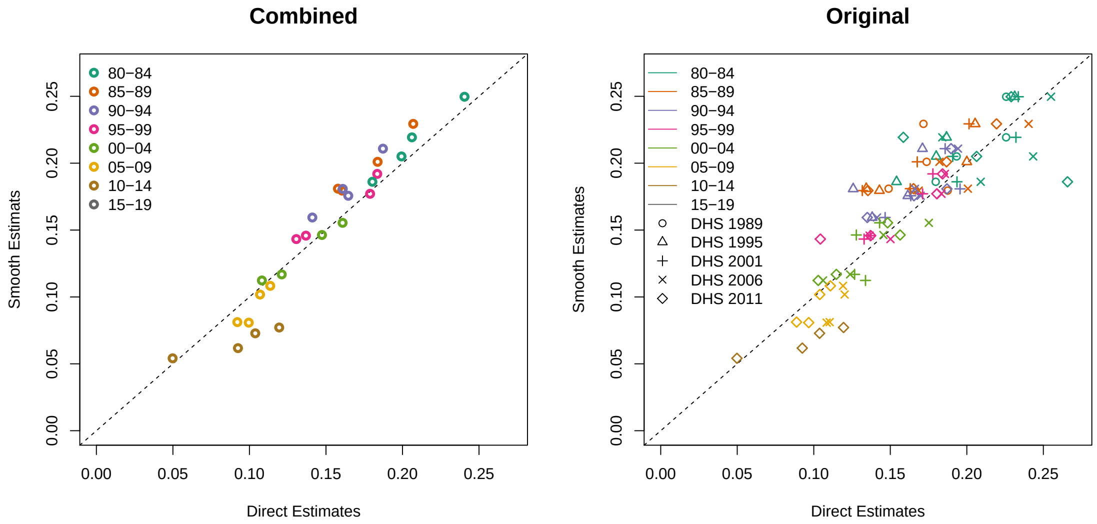

Figure S1.295: Uganda: Smooth versus direct Admin 1 estimates. Left: Combined (meta-analysis) survey estimate against combined direct estimates. Right: Combined (meta-analysis) survey estimate against direct estimates from each survey.

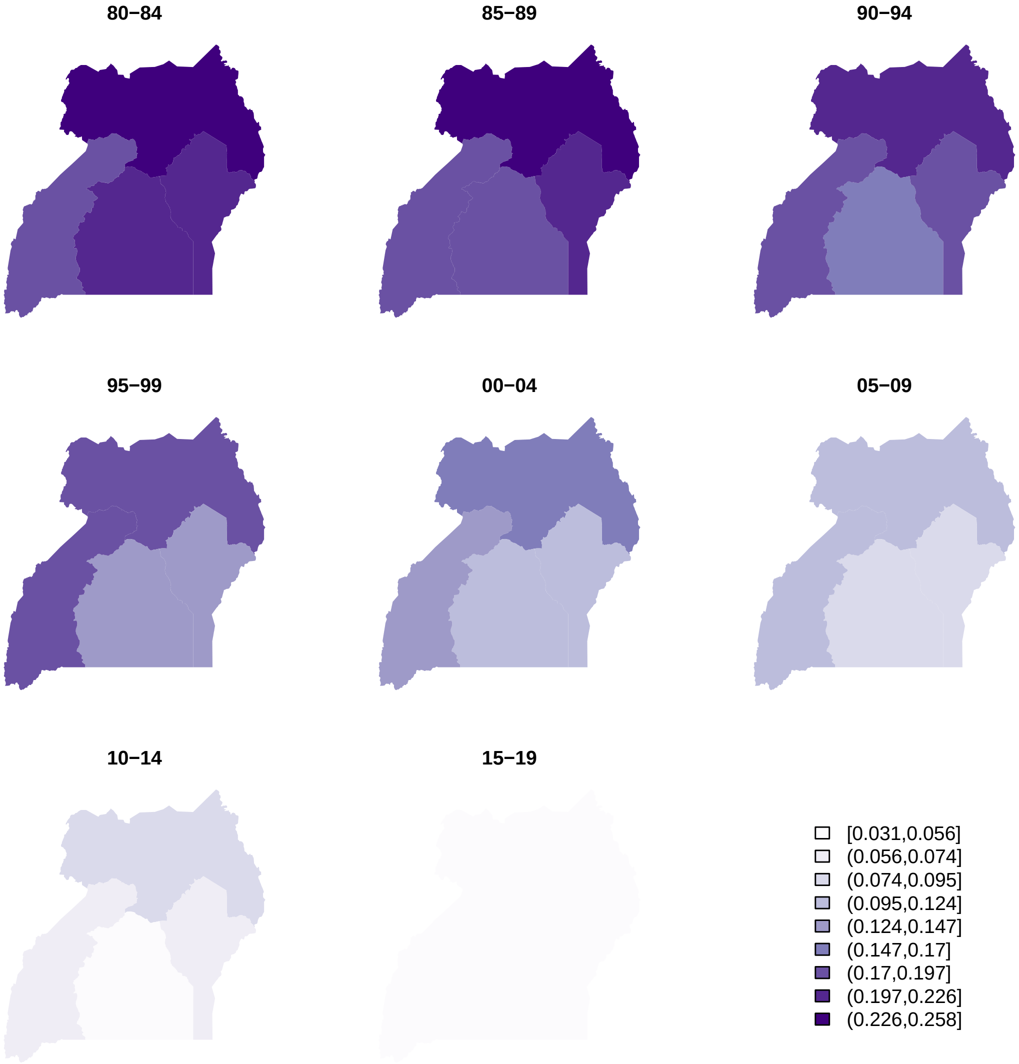

Figure S1.296: Uganda: Maps of posterior medians over time.

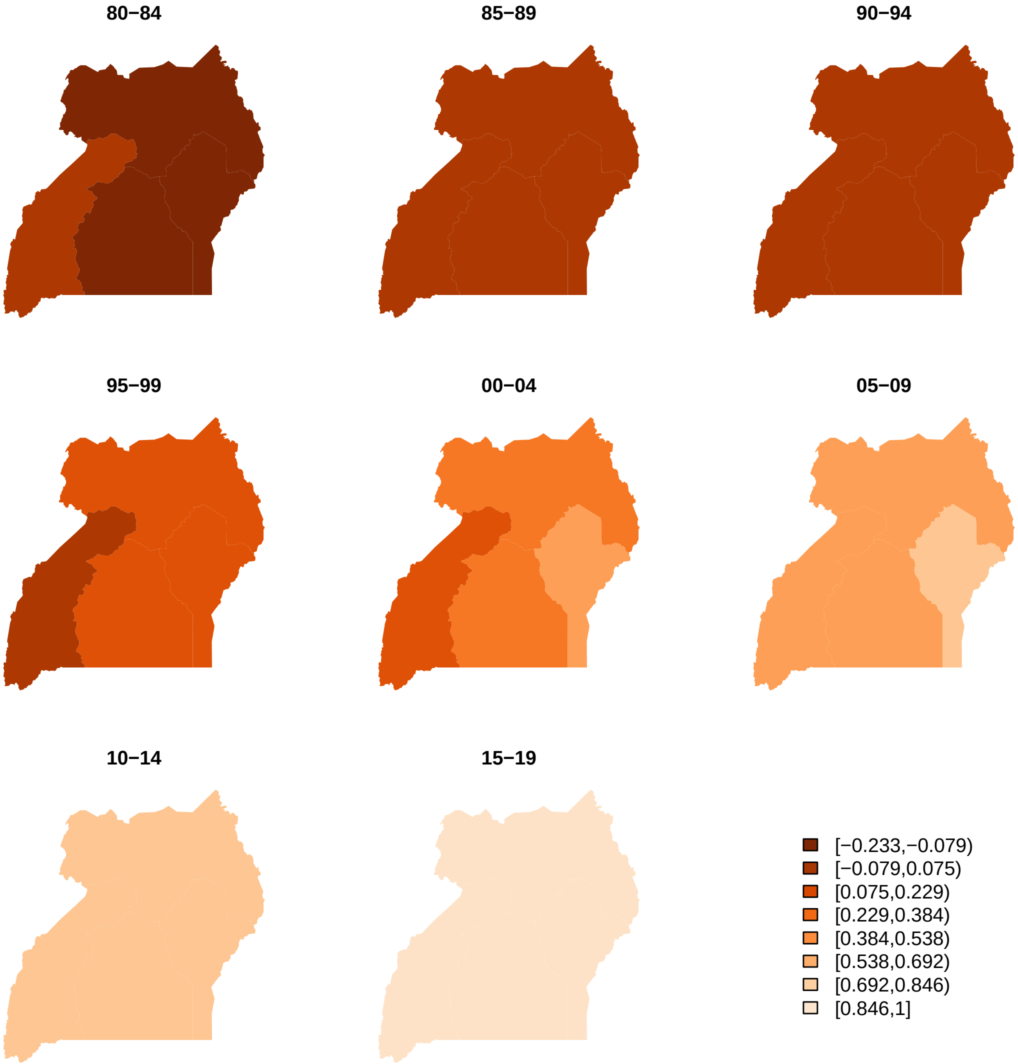

Figure S1.297: Uganda: Maps of reduction of posterior median U5MR in each five-year period compared to 1990 over time.

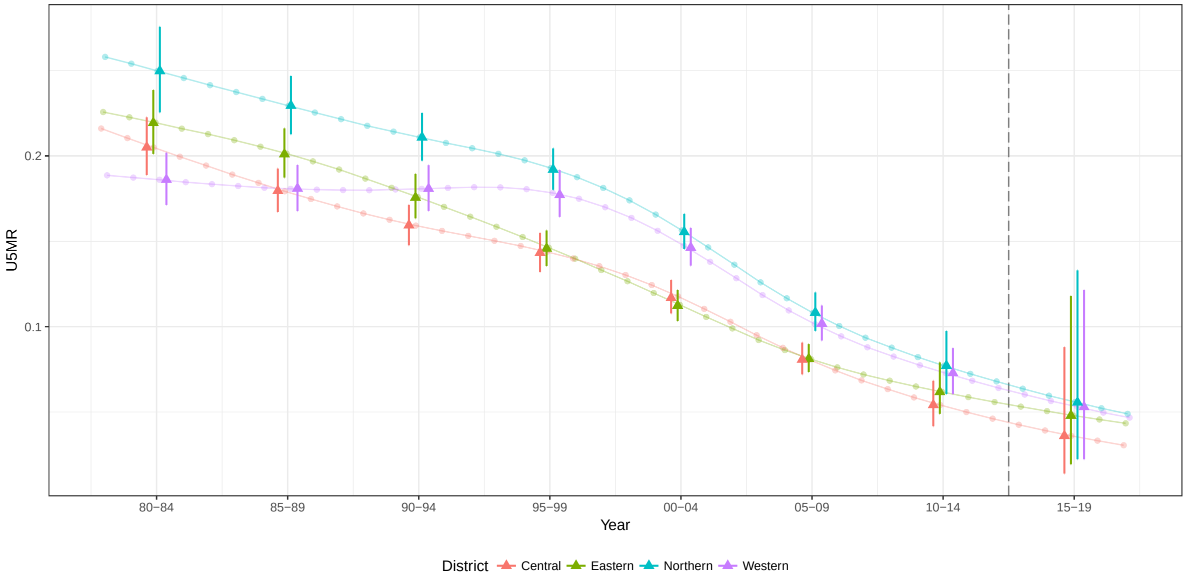

Figure S1.298: Uganda: Smoothed regional estimates over time. The line indicates yearly posterior median estimates and error bars indicate 95 % posterior credible interval at each time period.

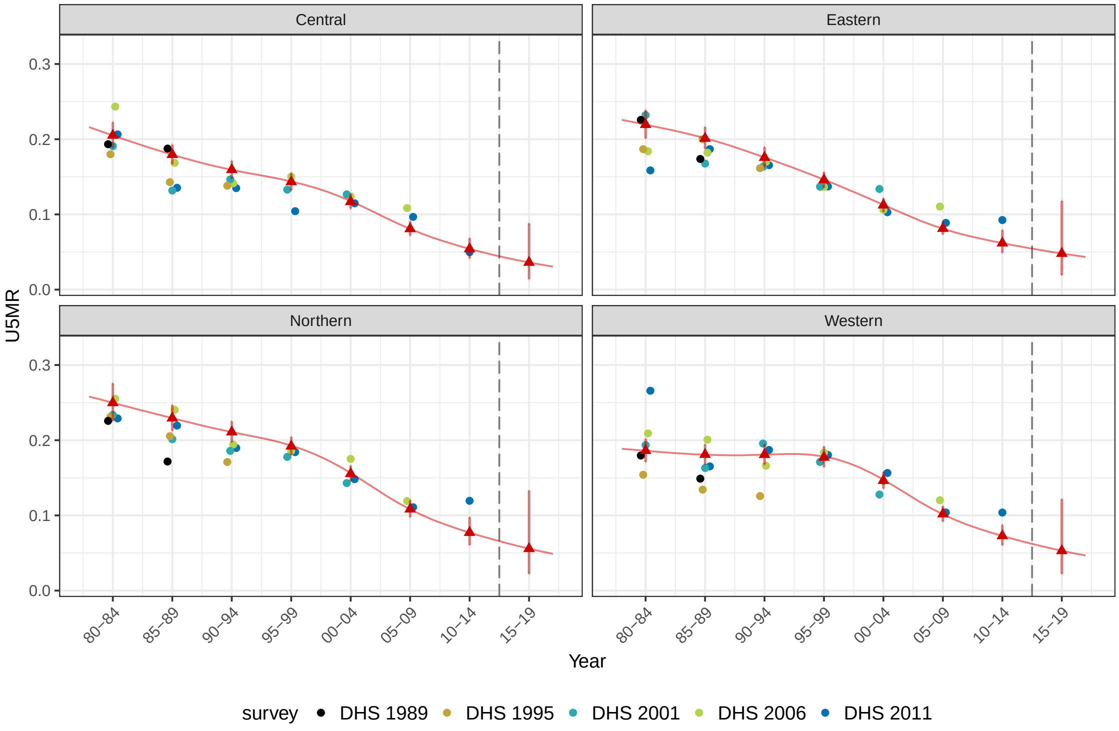

Figure S1.299: Uganda: Smoothed regional estimates over time compared to the direct estimates from each surveys. Direct estimates are not benchmarked with UN estimates. The line indicates posterior median and error bars indicate 95% posterior credible interval.

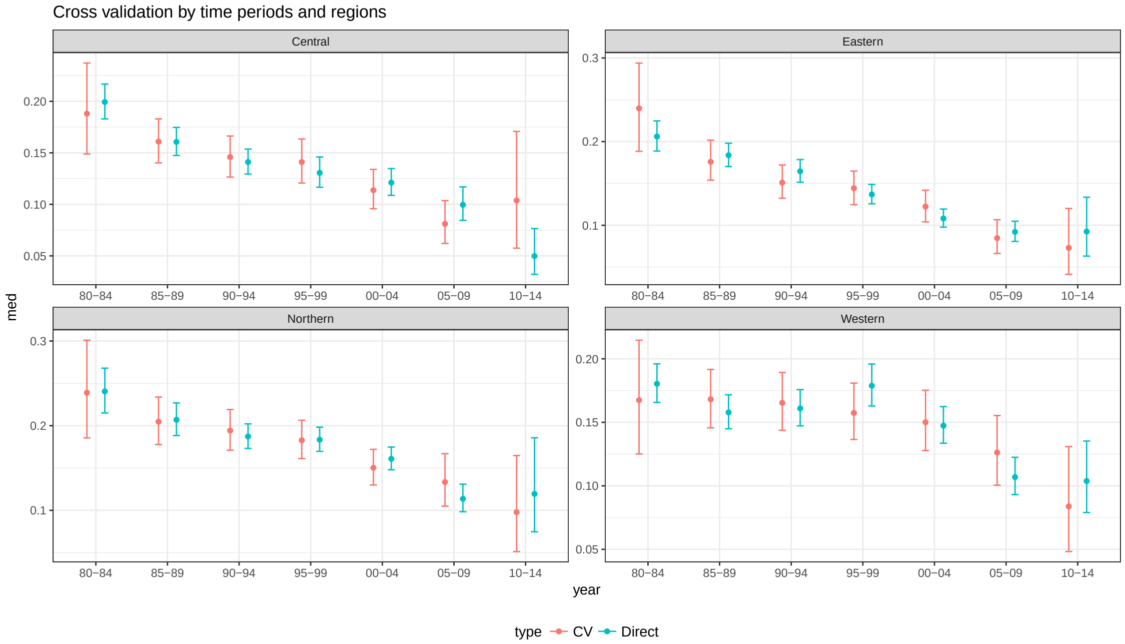

Figure S1.300: Uganda: Out-of-sample predictions along with direct estimates in the cross validation study where data from one region in each time period is held out and predicted using the rest of the data.

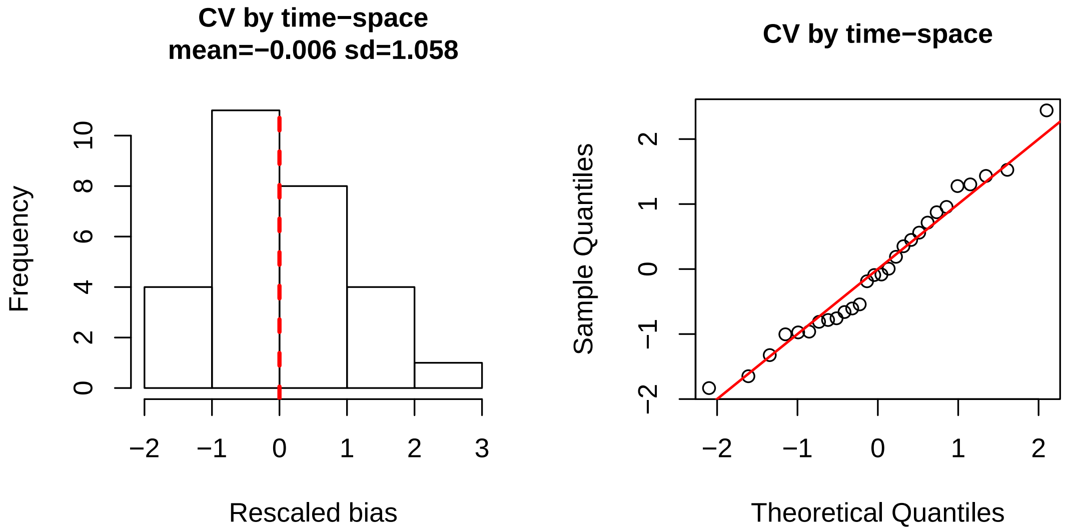

Figure S1.301: Uganda: Histogram and QQ-plot of the rescaled difference between the smoothed estimates and the direct estimates in the cross validation study. The differences between the two estimates are rescaled by the square root of the total variance of the two estimates.

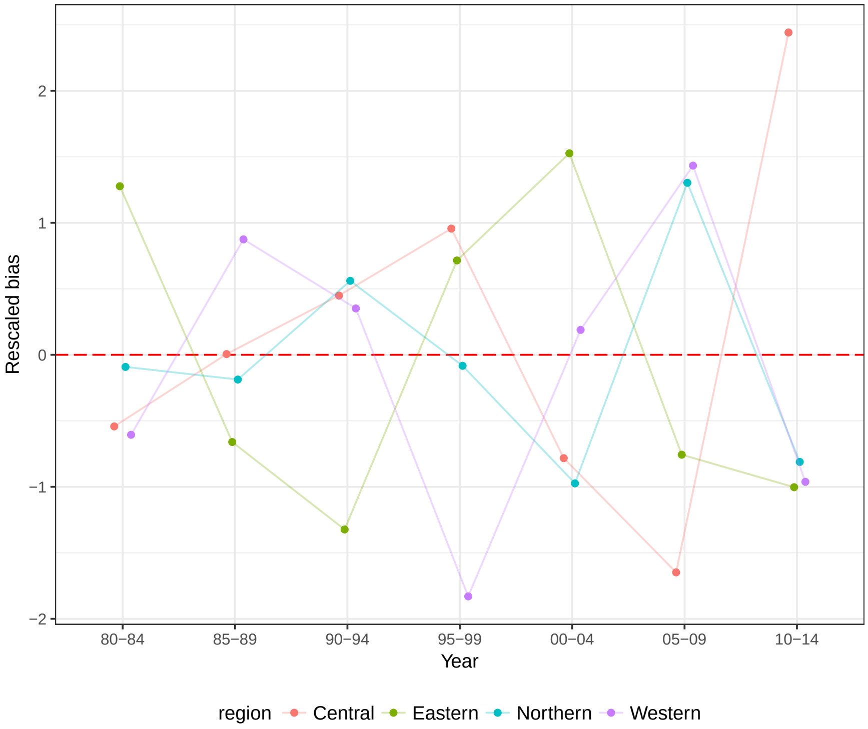

Figure S1.302: Uganda: Line plot of the difference between smoothed estimates and the direct estimates in the cross validation study. The differences between the two estimates are rescaled by the square root of the total variance of the two estimates.

3.5.34 Zambia

DHS surveys were conducted in Zambia in 1992, 1996, 2007, and 2014.

We fit both the RW2 only model to the combined national data, and compare the time trend at national level with the estimates produced by the UN and IHME in Figure S1.303. We then adjusted the combined national data to the UN estimates of U5MR, and refit the models on the benchmarked data.

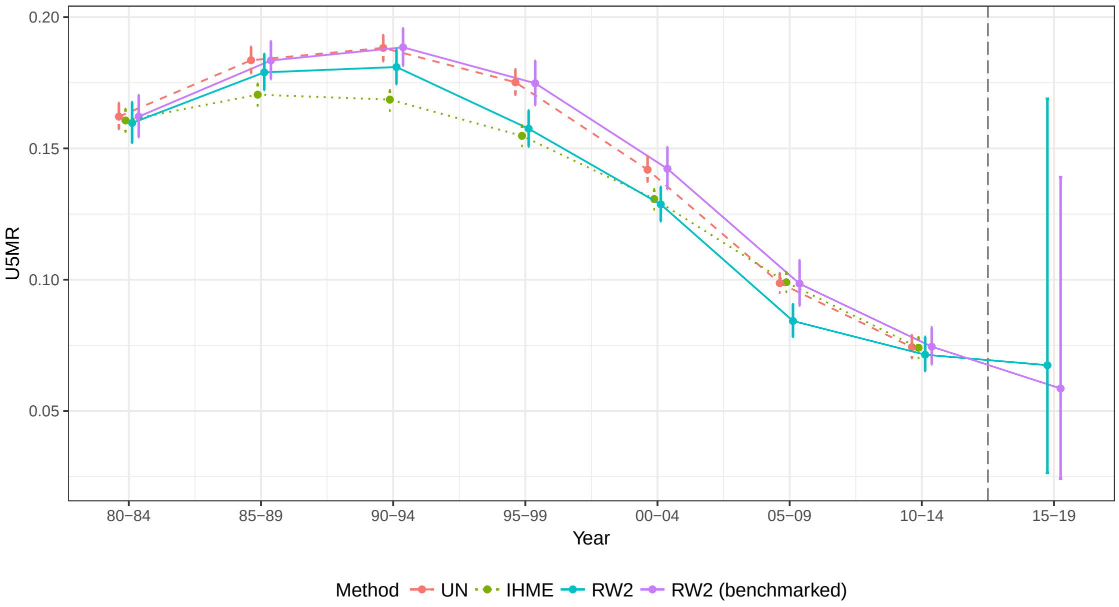

Figure S1.303: Zambia: Temporal national trends along with UN (B3) estimates described in You et al. (2015) and IHME estimates based on GBD 2015 Child Mortality Collaborators (2016). RW2 represents the smoothed national estimates using the original data before benchmarking with UN estimates. RW2-adj represents the smoothed national estimates using the benchmarked data.

We fit the RW2 model to the benchmarked data in each area. We compare the results in Figure S1.304 to S1.308. Figure S1.304 compares the smoothed estimates against the direct estimates. Figure S1.305 and Figure S1.306 show the posterior median estimates of U5MR in each region over time and the reductions from 1990 period respectively. Figure S1.307 shows the smoothed estimates by region over time and Figure S1.308 compares the smoothed estimates with direct estimates from each survey for each region over time.

We further assess the RW2 model by holding out some observations, and compare the projections to the direct estimates in these holdout observations. Figure S1.309 compares the predicted estimates for the out-of-sample observations with the direct estimates by holding out observations from each area in each time period. Figure S1.310 compares the histogram of the bias rescaled by the total variance in the cross validation studies. Figure S1.311 compares the rescaled bias by region and time periods.

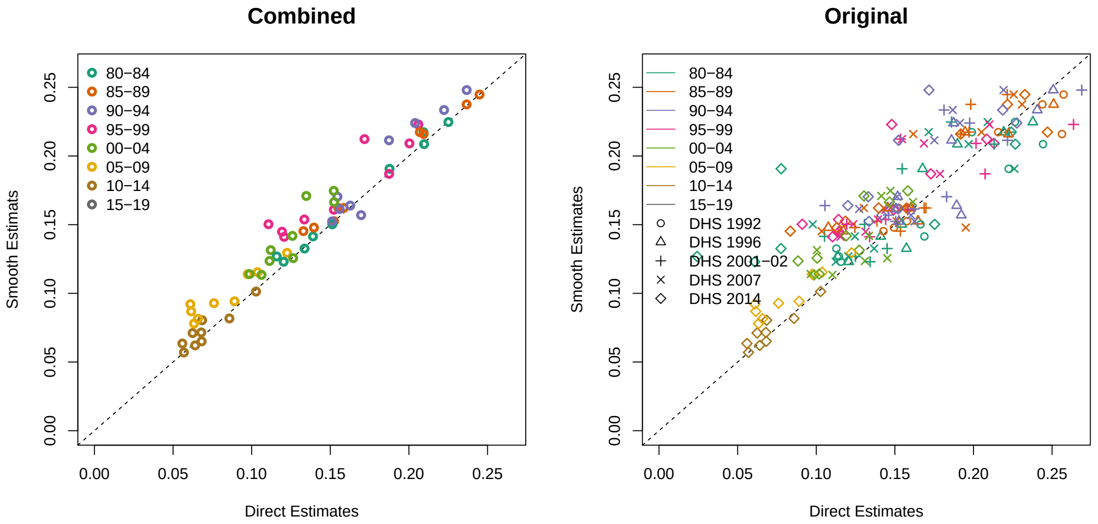

Figure S1.304: Zambia: Smooth versus direct Admin 1 estimates. Left: Combined (meta-analysis) survey estimate against combined direct estimates. Right: Combined (meta-analysis) survey estimate against direct estimates from each survey.

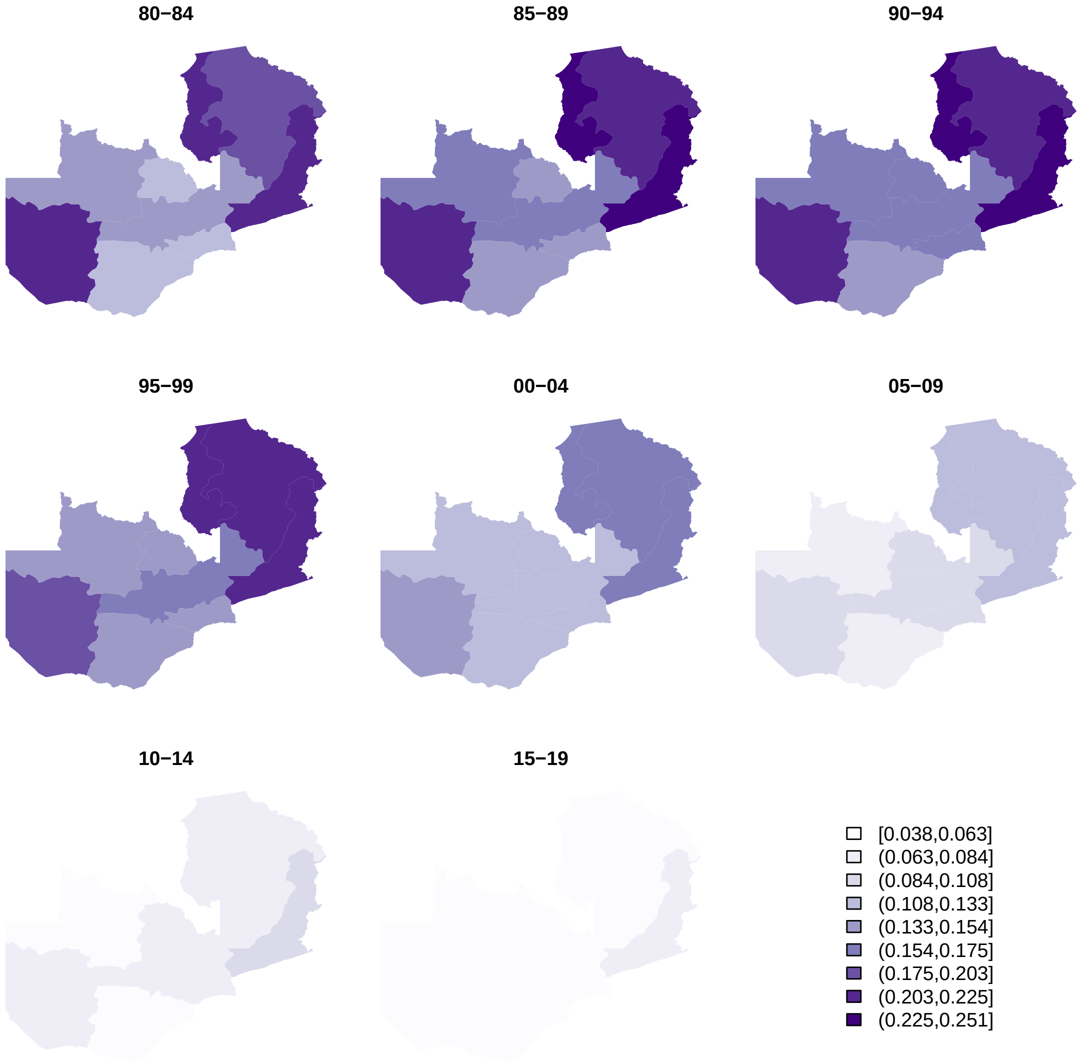

Figure S1.305: Zambia: Maps of posterior medians over time.

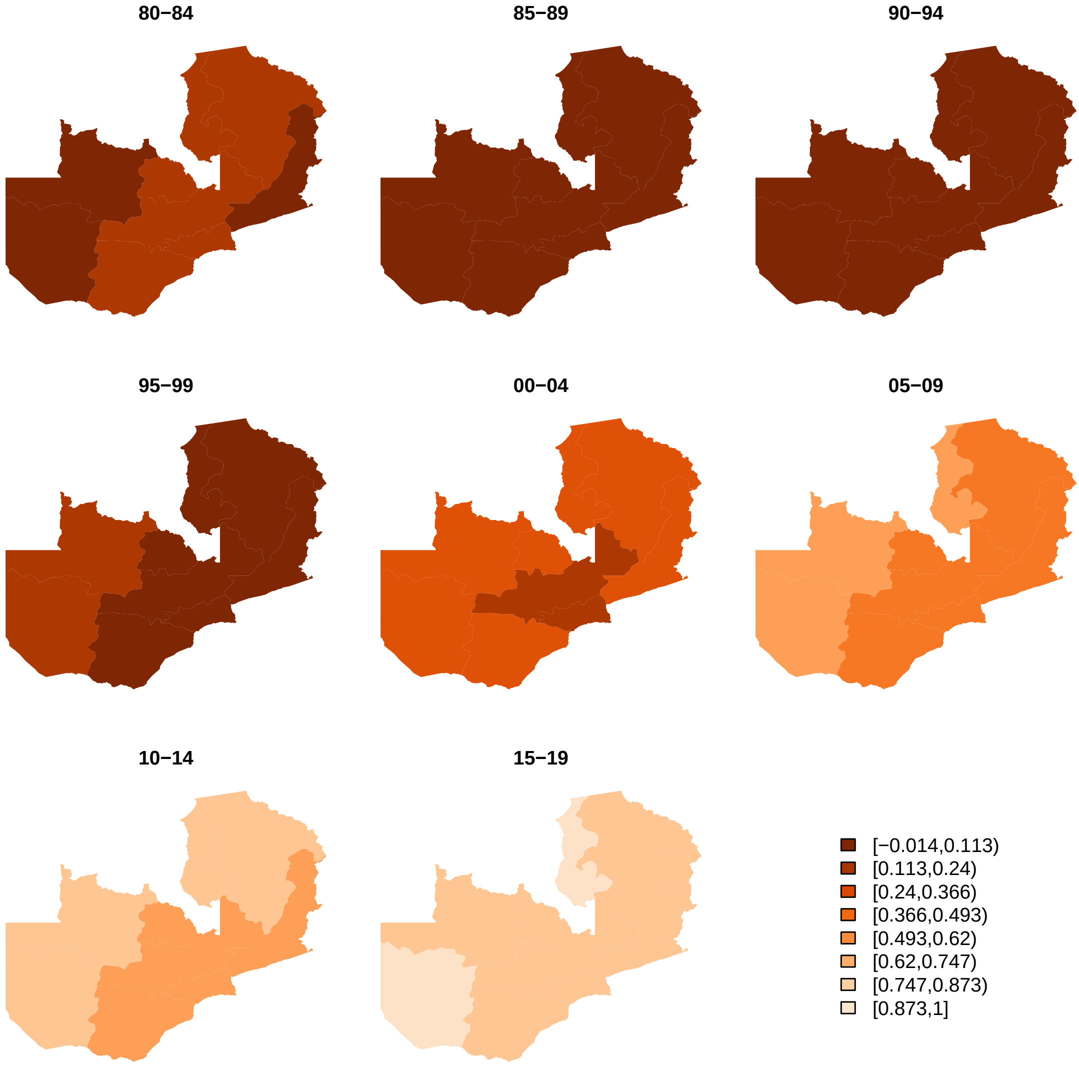

Figure S1.306: Zambia: Maps of reduction of posterior median U5MR in each five-year period compared to 1990 over time.

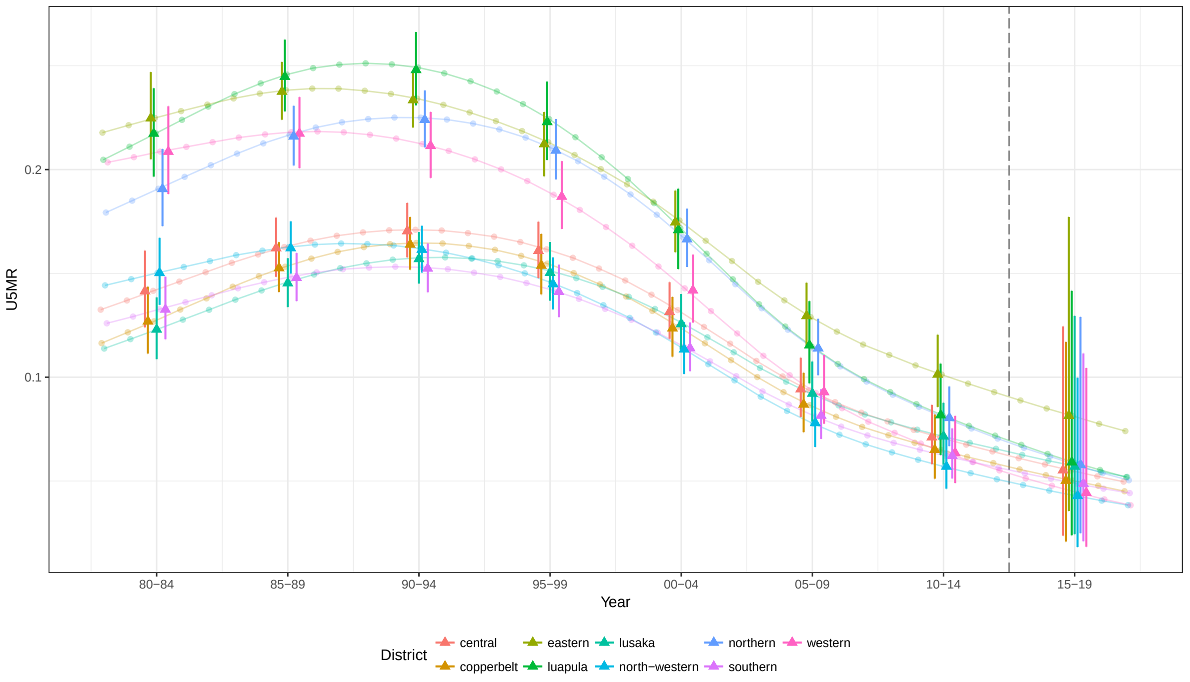

Figure S1.307: Zambia: Smoothed regional estimates over time. The line indicates yearly posterior median estimates and error bars indicate 95 % posterior credible interval at each time period.

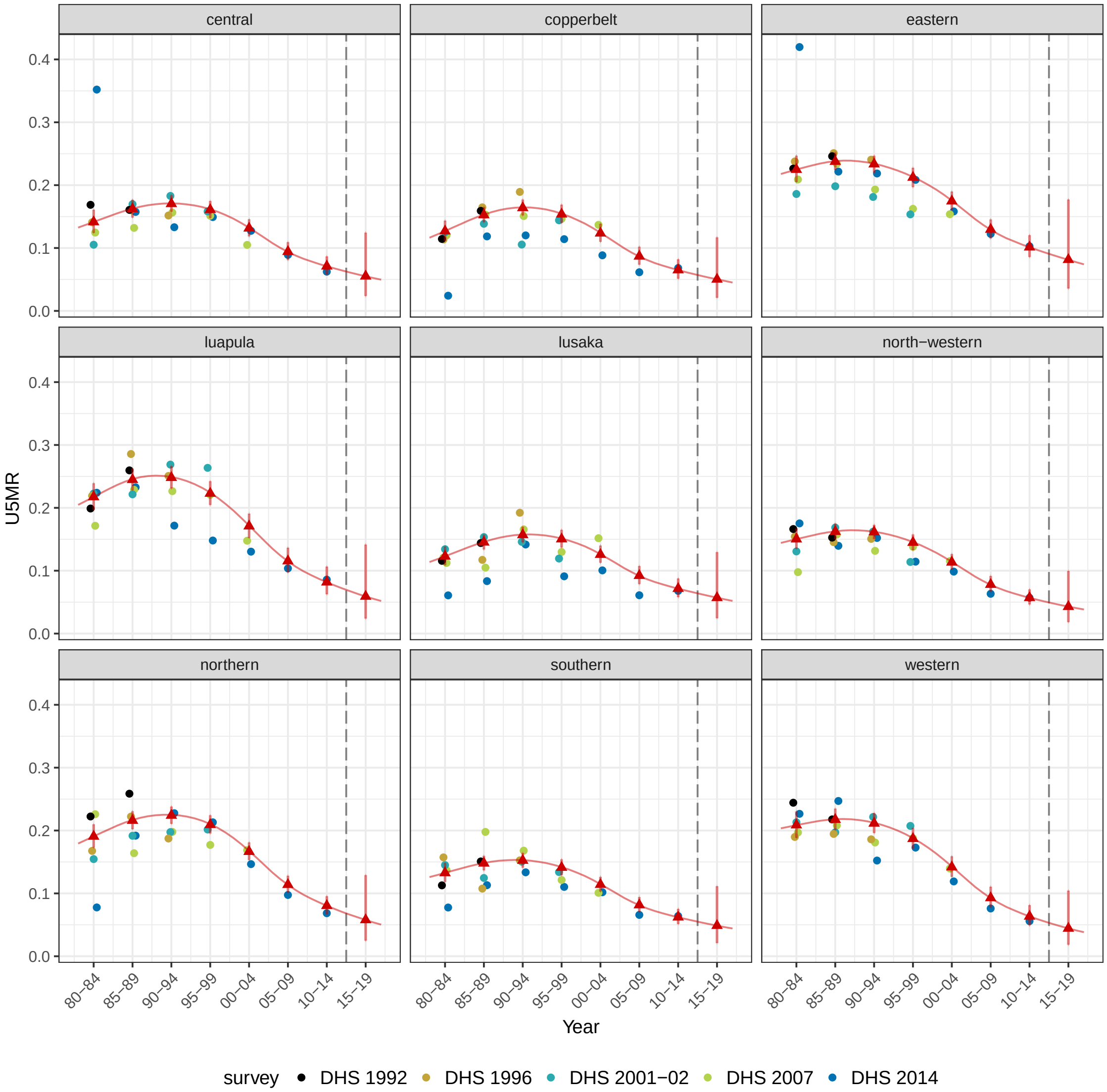

Figure S1.308: Zambia: Smoothed regional estimates over time compared to the direct estimates from each surveys. Direct estimates are not benchmarked with UN estimates. The line indicates posterior median and error bars indicate 95% posterior credible interval.

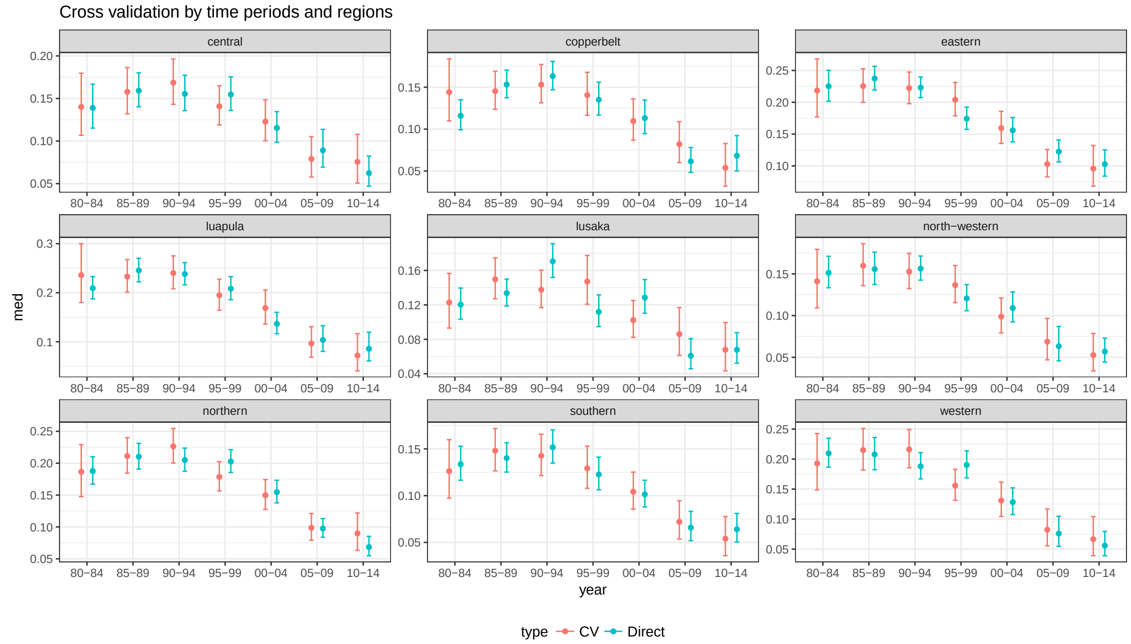

Figure S1.309: Zambia: Out-of-sample predictions along with direct estimates in the cross validation study where data from one region in each time period is held out and predicted using the rest of the data.

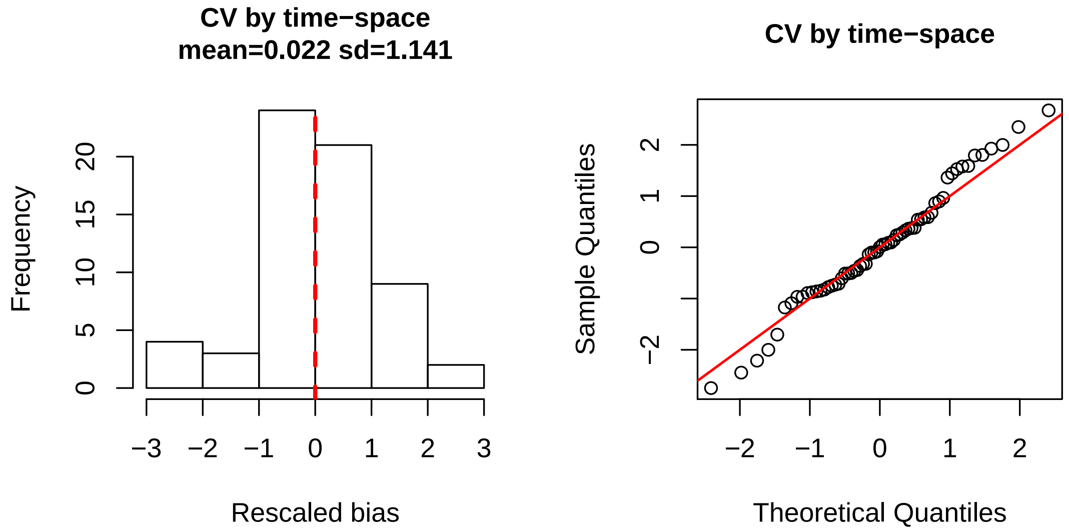

Figure S1.310: Zambia: Histogram and QQ-plot of the rescaled difference between the smoothed estimates and the direct estimates in the cross validation study. The differences between the two estimates are rescaled by the square root of the total variance of the two estimates.

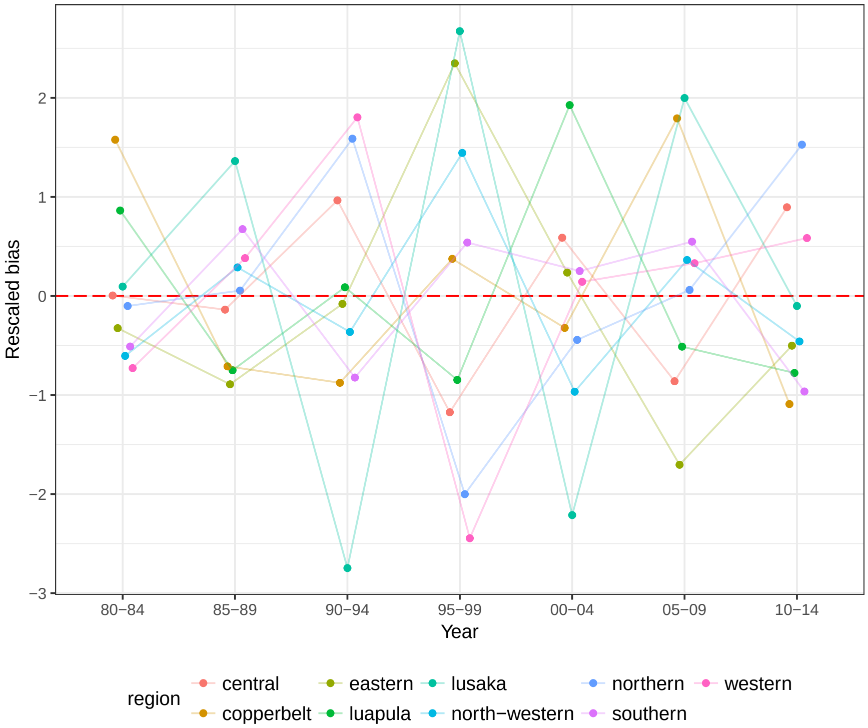

Figure S1.311: Zambia: Line plot of the difference between smoothed estimates and the direct estimates in the cross validation study. The differences between the two estimates are rescaled by the square root of the total variance of the two estimates.

3.5.35 Zimbabwe

DHS surveys were conducted in Zimbabwe in 1994, 1999, 2006, and 2015.

We fit both the RW2 only model to the combined national data, and compare the time trend at national level with the estimates produced by the UN and IHME in Figure S1.312. We then adjusted the combined national data to the UN estimates of U5MR, and refit the models on the benchmarked data.

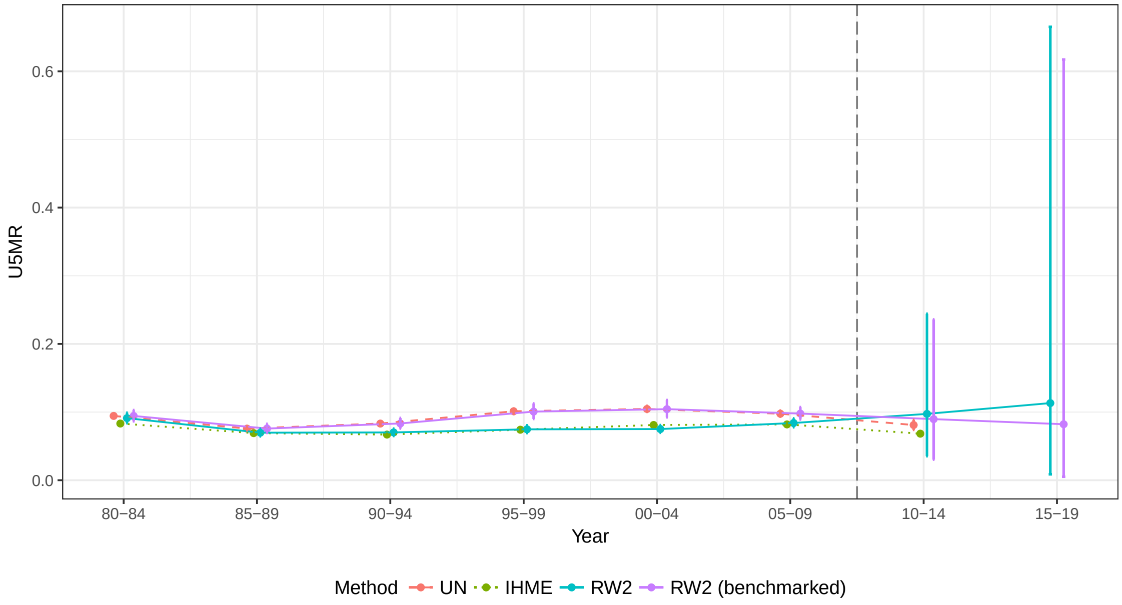

Figure S1.312: Zimbabwe: Temporal national trends along with UN (B3) estimates described in You et al. (2015) and IHME estimates based on GBD 2015 Child Mortality Collaborators (2016). RW2 represents the smoothed national estimates using the original data before benchmarking with UN estimates. RW2-adj represents the smoothed national estimates using the benchmarked data.

We fit the RW2 model to the benchmarked data in each area. We compare the results in Figure S1.313 to S1.317. Figure S1.313 compares the smoothed estimates against the direct estimates. Figure S1.314 and Figure S1.315 show the posterior median estimates of U5MR in each region over time and the reductions from 1990 period respectively. Figure S1.316 shows the smoothed estimates by region over time and Figure S1.317 compares the smoothed estimates with direct estimates from each survey for each region over time.

We further assess the RW2 model by holding out some observations, and compare the projections to the direct estimates in these holdout observations. Figure S1.318 compares the predicted estimates for the out-of-sample observations with the direct estimates by holding out observations from each area in each time period. Figure S1.319 compares the histogram of the bias rescaled by the total variance in the cross validation studies. Figure S1.320 compares the rescaled bias by region and time periods.

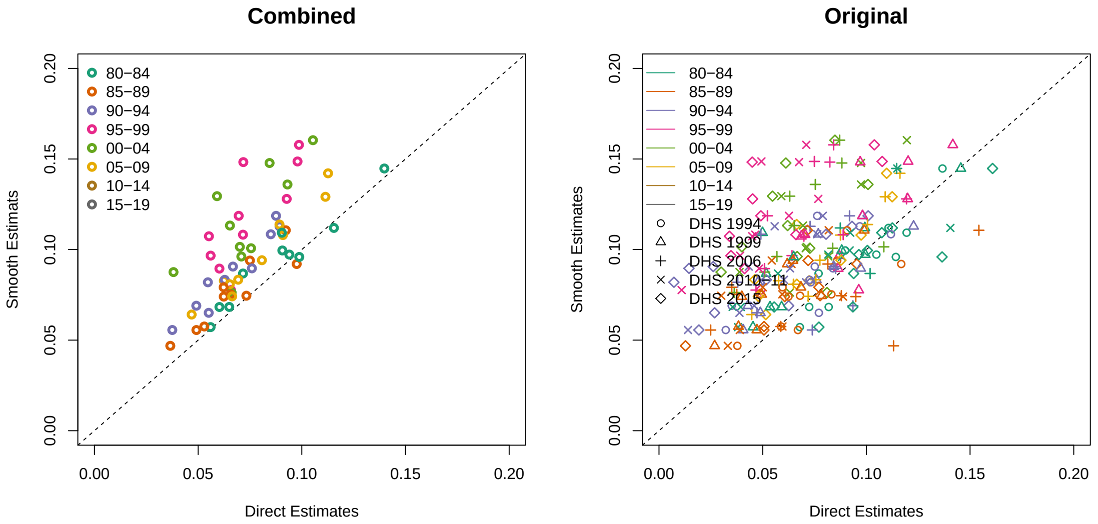

Figure S1.313: Zimbabwe: Smooth versus direct Admin 1 estimates. Left: Combined (meta-analysis) survey estimate against combined direct estimates. Right: Combined (meta-analysis) survey estimate against direct estimates from each survey.

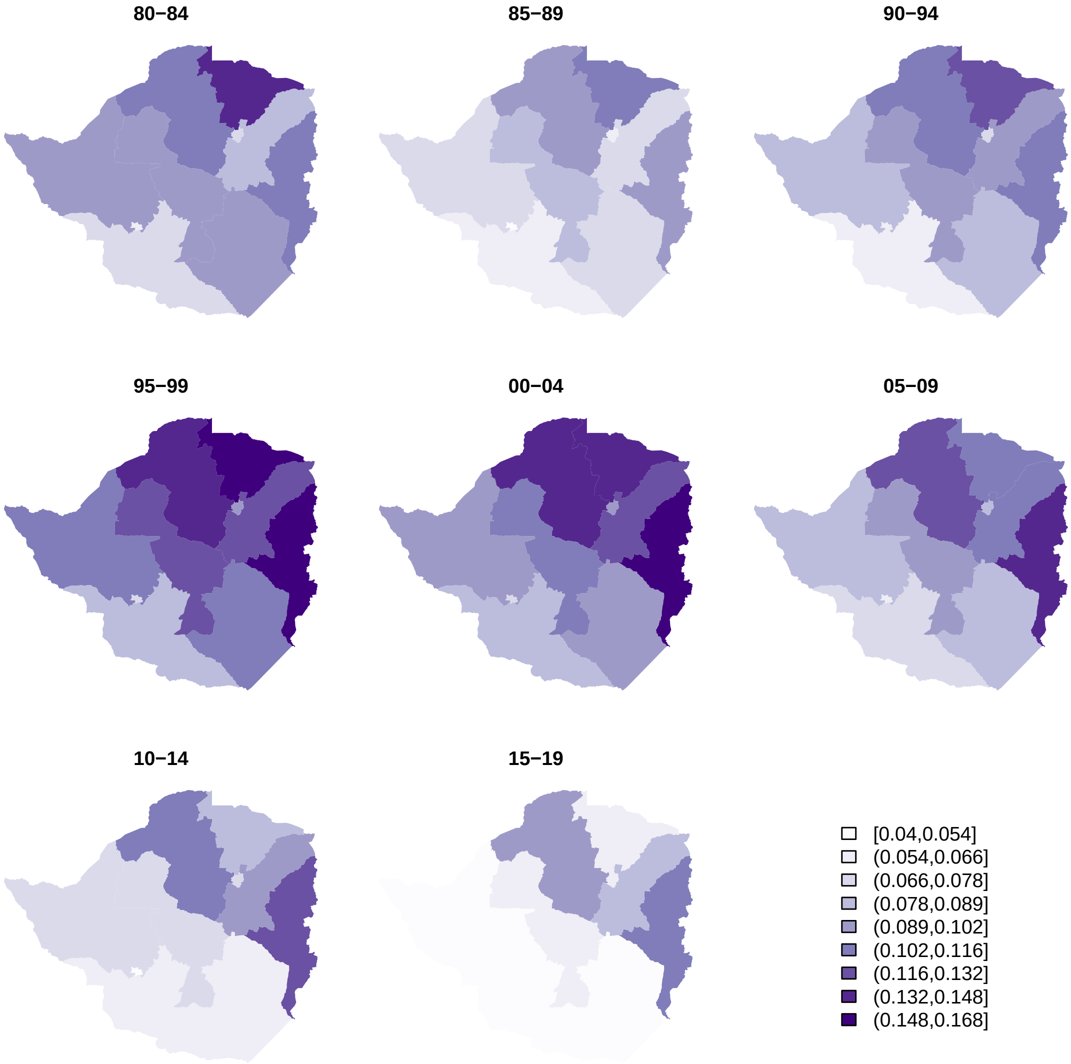

Figure S1.314: Zimbabwe: Maps of posterior medians over time.

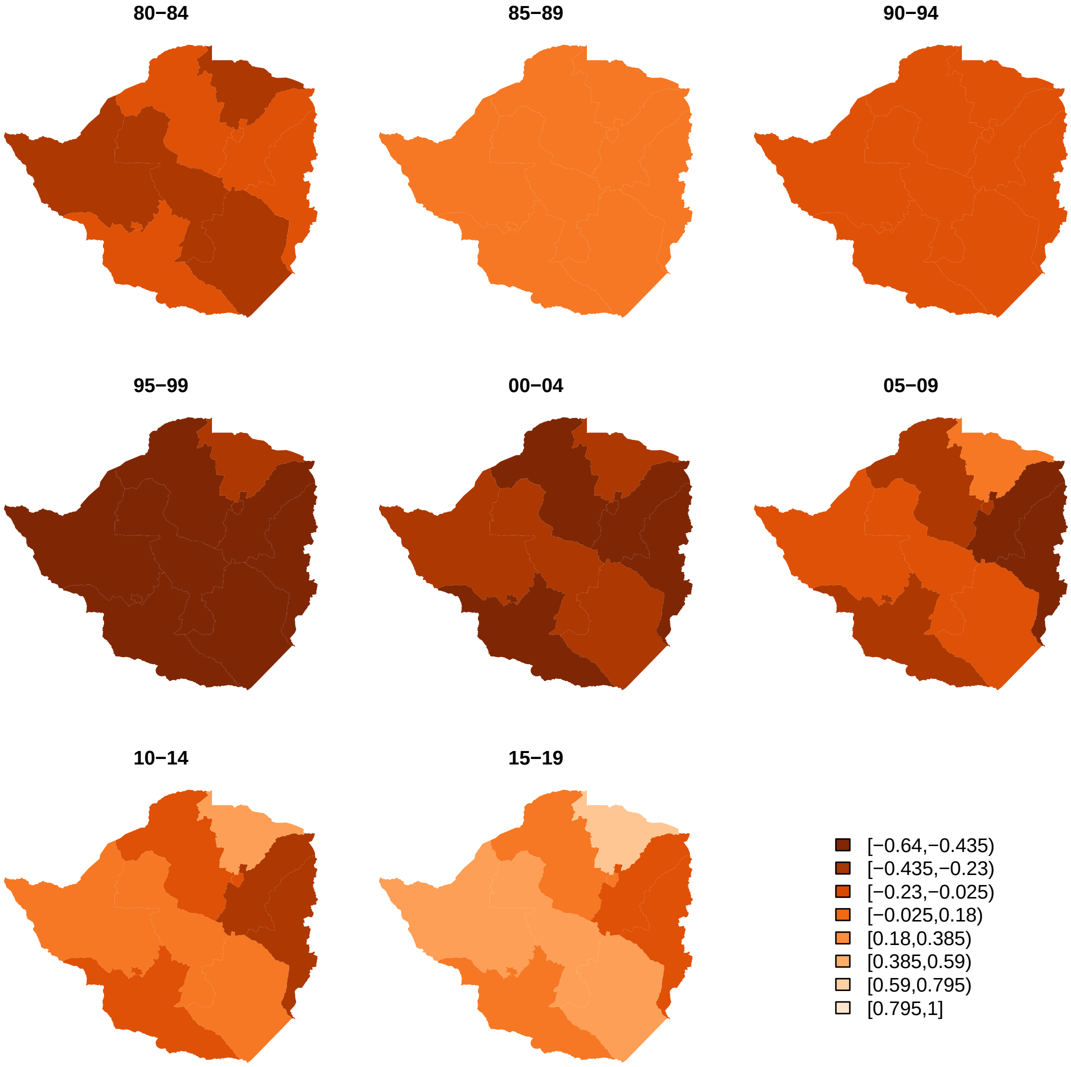

Figure S1.315: Zimbabwe: Maps of reduction of posterior median U5MR in each five-year period compared to 1990 over time.

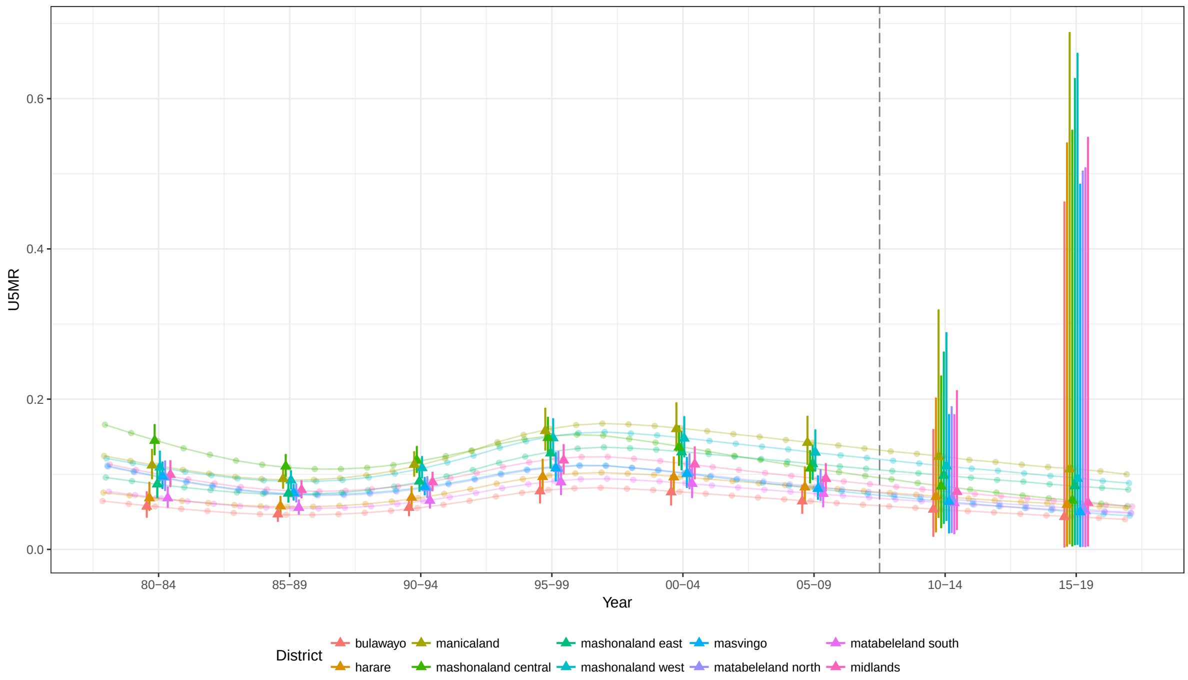

Figure S1.316: Zimbabwe: Smoothed regional estimates over time. The line indicates yearly posterior median estimates and error bars indicate 95 % posterior credible interval at each time period.

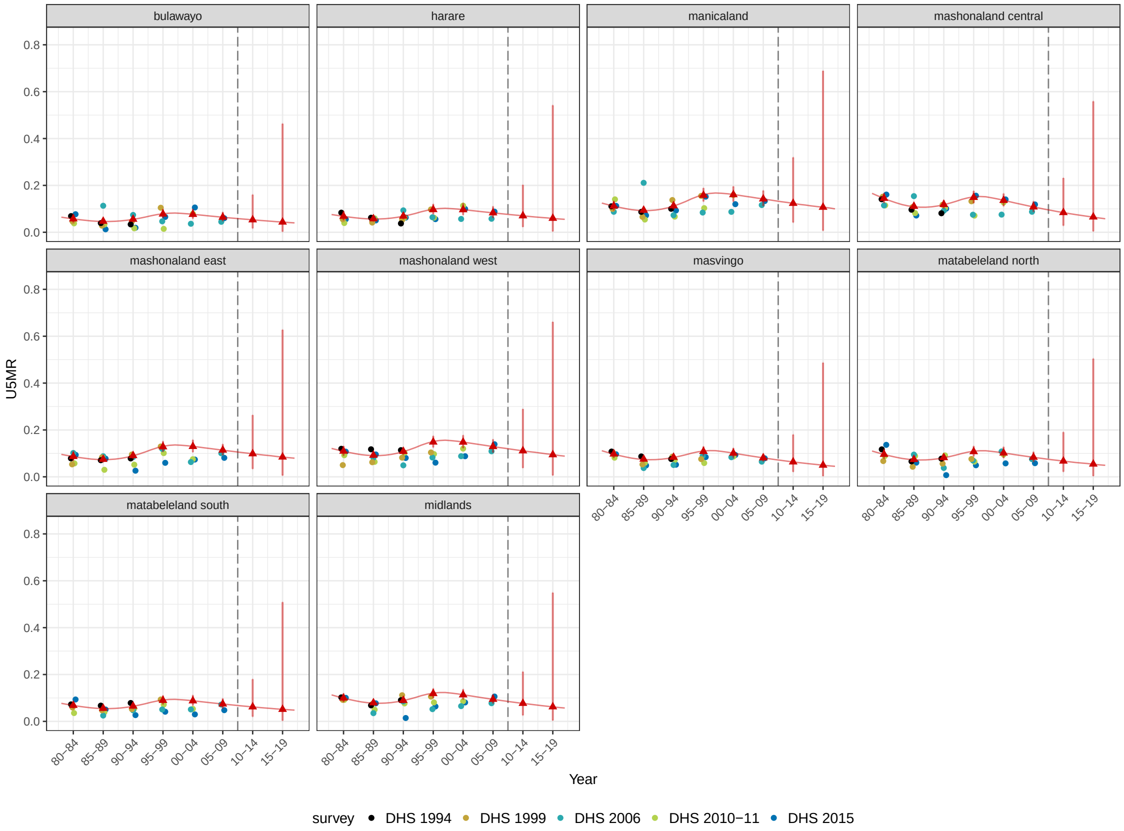

Figure S1.317: Zimbabwe: Smoothed regional estimates over time compared to the direct estimates from each surveys. Direct estimates are not benchmarked with UN estimates. The line indicates posterior median and error bars indicate 95% posterior credible interval.

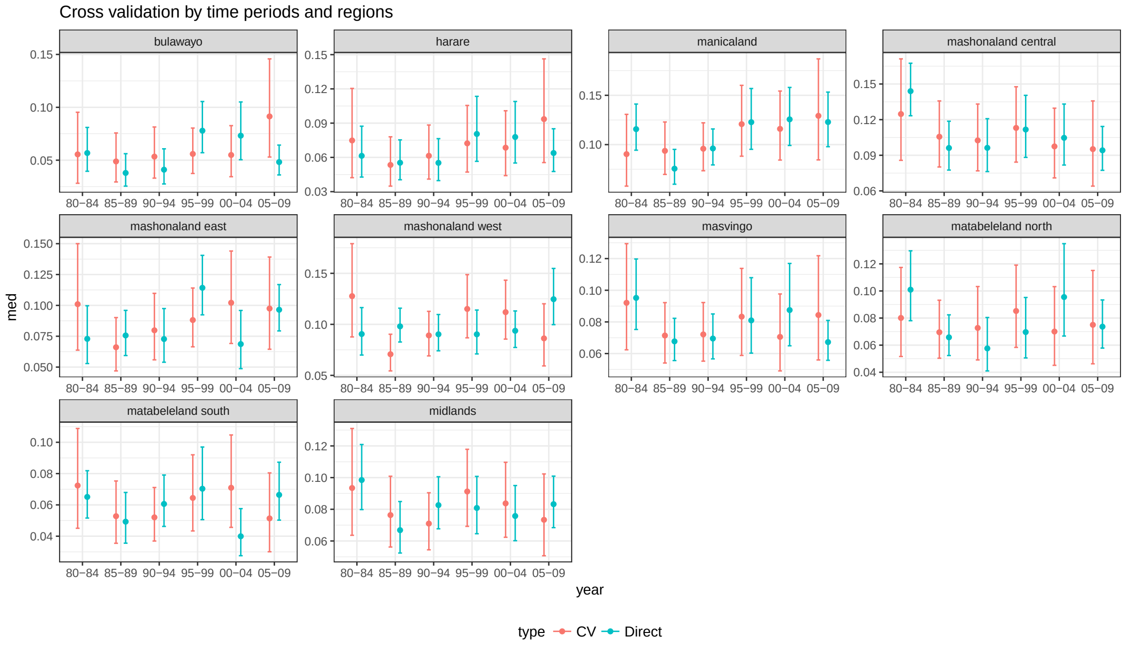

Figure S1.318: Zimbabwe: Out-of-sample predictions along with direct estimates in the cross validation study where data from one region in each time period is held out and predicted using the rest of the data.

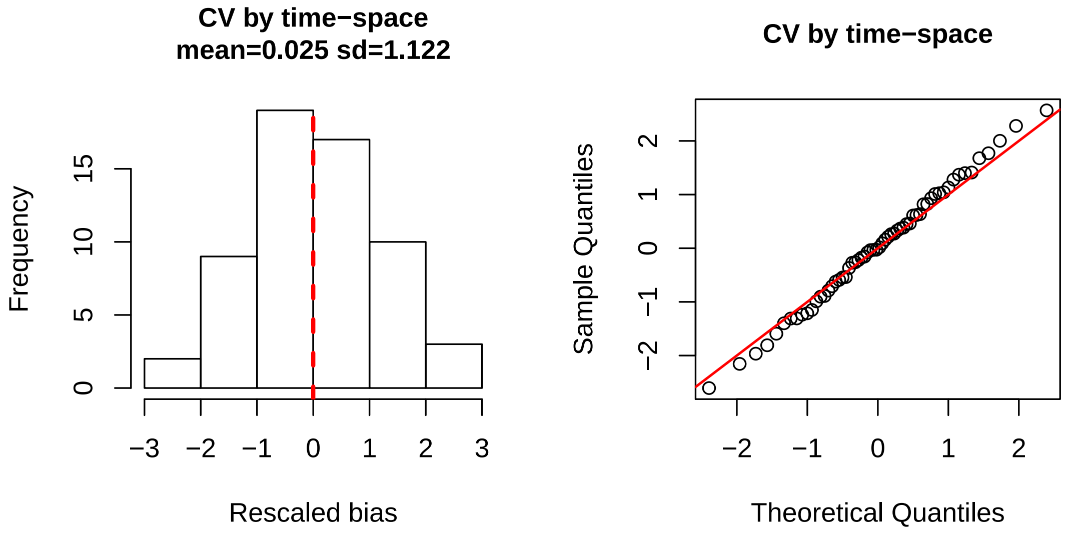

Figure S1.319: Zimbabwe: Histogram and QQ-plot of the rescaled difference between the smoothed estimates and the direct estimates in the cross validation study. The differences between the two estimates are rescaled by the square root of the total variance of the two estimates.

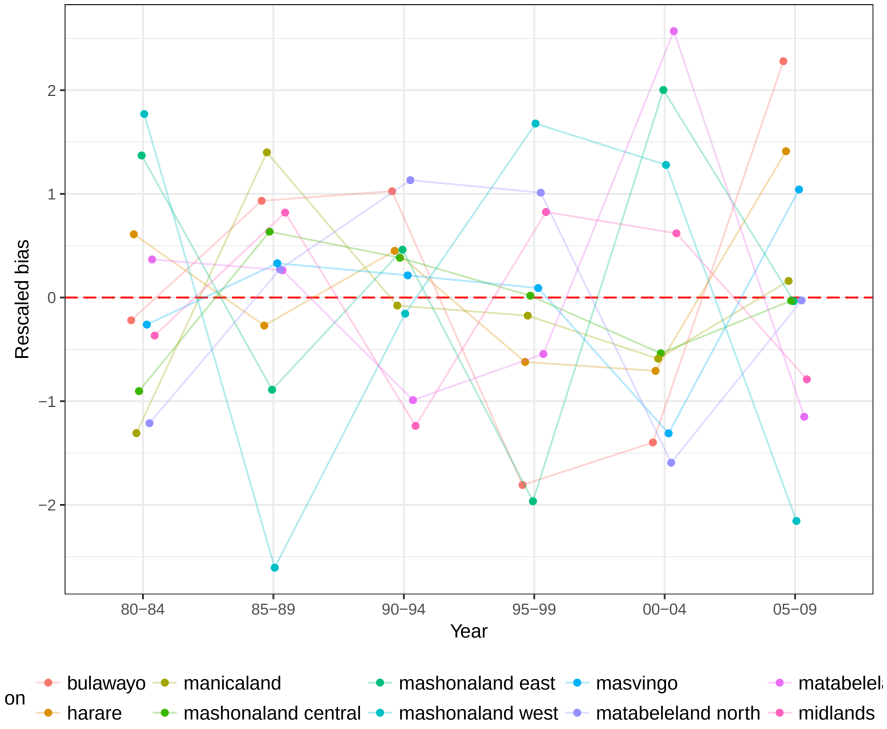

Figure S1.320: Zimbabwe: Line plot of the difference between smoothed estimates and the direct estimates in the cross validation study. The differences between the two estimates are rescaled by the square root of the total variance of the two estimates.

3.6 Table of All Results: 5-year Periods

Table S1.9: Complete Results by 5-Year Period.

Country	Region	Year	Median	Lower	Upper	Method
Angola	ALL	80-84	406.97	547.82	279.93	HT-Direct
Angola	ALL	80-84	189.53	179.33	200.89	IHME
Angola	ALL	80-84	251.28	192.05	322.16	RW2
Angola	ALL	80-84	232.95	214.36	255.00	UN
Angola	ALL	85-89	235.78	283.55	193.88	HT-Direct
Angola	ALL	85-89	187.44	179.24	196.32	IHME
Angola	ALL	85-89	222.56	189.53	258.44	RW2
Angola	ALL	85-89	227.72	214.73	241.69	UN
Angola	ALL	90-94	223.20	253.70	195.41	HT-Direct
Angola	ALL	90-94	182.79	175.12	191.15	IHME
Angola	ALL	90-94	226.02	200.25	254.31	RW2
Angola	ALL	90-94	227.00	215.43	238.75	UN
Angola	ALL	95-99	210.03	231.55	190.01	HT-Direct
Angola	ALL	95-99	161.43	154.52	168.39	IHME
Angola	ALL	95-99	224.40	202.98	247.22	RW2
Angola	ALL	95-99	223.29	211.97	235.65	UN
Angola	ALL	00-04	162.11	178.41	147.03	HT-Direct
Angola	ALL	00-04	144.35	138.13	151.24	IHME
Angola	ALL	00-04	212.56	185.32	242.76	RW2
Angola	ALL	00-04	212.39	198.39	226.68	UN
Angola	ALL	05-09	105.82	117.60	95.09	HT-Direct
Angola	ALL	05-09	113.54	107.89	119.83	IHME
Angola	ALL	05-09	194.23	133.86	273.39	RW2
Angola	ALL	05-09	195.23	177.10	218.26	UN
Angola	ALL	10-14	67.86	75.65	60.83	HT-Direct
Angola	ALL	10-14	91.11	83.25	99.61	IHME
Angola	ALL	10-14	173.94	150.46	200.03	RW2
Angola	ALL	10-14	173.83	144.34	207.56	UN
Angola	BENGO	80-84	109.42	28.82	341.04	RW2
Angola	BENGO	85-89	98.46	430.20	15.55	HT-Direct
Angola	BENGO	85-89	98.18	37.18	232.16	RW2
Angola	BENGO	90-94	37.81	145.36	9.00	HT-Direct
Angola	BENGO	90-94	107.18	54.88	196.73	RW2
Angola	BENGO	95-99	99.96	213.76	43.40	HT-Direct
Angola	BENGO	95-99	110.33	68.70	170.86	RW2
Angola	BENGO	00-04	131.53	220.77	74.90	HT-Direct
Angola	BENGO	00-04	121.38	77.40	184.04	RW2
Angola	BENGO	05-09	66.52	113.31	38.22	HT-Direct
Angola	BENGO	05-09	120.34	61.94	221.01	RW2
Angola	BENGO	10-14	28.58	52.50	15.39	HT-Direct
Angola	BENGO	10-14	109.96	48.26	231.45	RW2
Angola	BENGO	15-19	99.26	19.43	382.74	RW2
Angola	BENGUELA	80-84	528.44	701.44	348.32	HT-Direct
Angola	BENGUELA	80-84	382.03	295.80	472.34	RW2
Angola	BENGUELA	85-89	472.84	609.98	339.67	HT-Direct
Angola	BENGUELA	85-89	371.05	308.05	437.98	RW2
Angola	BENGUELA	90-94	382.04	456.77	312.51	HT-Direct
Angola	BENGUELA	90-94	399.95	350.79	452.10	RW2
Angola	BENGUELA	95-99	396.29	459.02	336.80	HT-Direct
Angola	BENGUELA	95-99	391.60	345.92	441.97	RW2
Angola	BENGUELA	00-04	284.90	347.64	229.50	HT-Direct
Angola	BENGUELA	00-04	388.81	331.42	450.89	RW2
Angola	BENGUELA	05-09	173.61	214.51	139.13	HT-Direct
Angola	BENGUELA	05-09	356.35	259.79	466.03	RW2
Angola	BENGUELA	10-14	117.95	149.51	92.34	HT-Direct
Angola	BENGUELA	10-14	306.23	239.98	380.60	RW2
Angola	BENGUELA	15-19	256.79	87.07	554.63	RW2
Angola	BIÉ	80-84	530.35	747.96	300.55	HT-Direct
Angola	BIÉ	80-84	207.78	140.72	302.01	RW2

Continued on next page

Country	Region	Year	Median	Lower	Upper	Method
Angola	BIÉ	85-89	104.44	232.42	42.98	HT-Direct
Angola	BIÉ	85-89	179.37	131.64	239.28	RW2
Angola	BIÉ	90-94	145.09	247.55	80.50	HT-Direct
Angola	BIÉ	90-94	185.19	147.45	229.35	RW2
Angola	BIÉ	95-99	150.46	183.96	122.14	HT-Direct
Angola	BIÉ	95-99	179.43	150.51	211.60	RW2
Angola	BIÉ	00-04	171.01	217.76	132.60	HT-Direct
Angola	BIÉ	00-04	188.75	153.86	228.77	RW2
Angola	BIÉ	05-09	88.68	116.56	66.97	HT-Direct
Angola	BIÉ	05-09	187.08	127.22	266.30	RW2
Angola	BIÉ	10-14	76.05	98.32	58.50	HT-Direct
Angola	BIÉ	10-14	176.26	129.69	236.48	RW2
Angola	BIÉ	15-19	164.50	51.31	419.47	RW2
Angola	CABINDA	80-84	99.11	32.41	280.32	RW2
Angola	CABINDA	85-89	38.54	228.39	5.40	HT-Direct
Angola	CABINDA	85-89	87.35	39.54	185.43	RW2
Angola	CABINDA	90-94	150.85	295.21	70.06	HT-Direct
Angola	CABINDA	90-94	92.79	53.96	154.23	RW2
Angola	CABINDA	95-99	49.46	96.83	24.64	HT-Direct
Angola	CABINDA	95-99	89.74	59.41	132.96	RW2
Angola	CABINDA	00-04	54.12	94.76	30.32	HT-Direct
Angola	CABINDA	00-04	94.76	62.66	138.82	RW2
Angola	CABINDA	05-09	65.27	112.63	37.00	HT-Direct
Angola	CABINDA	05-09	92.97	51.48	161.67	RW2
Angola	CABINDA	10-14	34.72	54.72	21.85	HT-Direct
Angola	CABINDA	10-14	86.45	44.88	159.87	RW2
Angola	CABINDA	15-19	79.89	18.84	287.17	RW2
Angola	CUANDO CUBANGO	80-84	117.10	34.29	333.57	RW2
Angola	CUANDO CUBANGO	85-89	114.77	46.62	256.40	RW2
Angola	CUANDO CUBANGO	90-94	79.09	200.42	28.58	HT-Direct
Angola	CUANDO CUBANGO	90-94	134.36	73.13	232.00	RW2
Angola	CUANDO CUBANGO	95-99	160.05	274.70	87.48	HT-Direct
Angola	CUANDO CUBANGO	95-99	142.79	92.50	211.75	RW2
Angola	CUANDO CUBANGO	00-04	127.93	227.75	68.00	HT-Direct
Angola	CUANDO CUBANGO	00-04	160.56	111.27	225.42	RW2
Angola	CUANDO CUBANGO	05-09	93.82	141.57	61.03	HT-Direct
Angola	CUANDO CUBANGO	05-09	166.30	100.17	262.85	RW2
Angola	CUANDO CUBANGO	10-14	58.76	85.86	39.84	HT-Direct
Angola	CUANDO CUBANGO	10-14	161.09	96.22	258.20	RW2
Angola	CUANDO CUBANGO	15-19	154.35	41.23	437.49	RW2
Angola	CUANZA NORTE	80-84	192.10	79.26	390.69	RW2
Angola	CUANZA NORTE	85-89	34.74	198.72	5.20	HT-Direct
Angola	CUANZA NORTE	85-89	178.63	97.94	303.21	RW2
Angola	CUANZA NORTE	90-94	208.84	329.17	124.34	HT-Direct
Angola	CUANZA NORTE	90-94	199.01	134.75	283.23	RW2
Angola	CUANZA NORTE	95-99	243.31	376.80	146.04	HT-Direct
Angola	CUANZA NORTE	95-99	203.92	151.92	269.34	RW2
Angola	CUANZA NORTE	00-04	210.17	304.41	139.26	HT-Direct
Angola	CUANZA NORTE	00-04	218.26	161.94	290.73	RW2
Angola	CUANZA NORTE	05-09	115.65	147.23	90.12	HT-Direct
Angola	CUANZA NORTE	05-09	212.26	132.84	324.83	RW2
Angola	CUANZA NORTE	10-14	64.68	96.87	42.69	HT-Direct
Angola	CUANZA NORTE	10-14	192.49	116.23	300.44	RW2
Angola	CUANZA NORTE	15-19	170.96	46.61	466.61	RW2
Angola	CUANZA SUL	80-84	238.79	689.39	42.46	HT-Direct
Angola	CUANZA SUL	80-84	223.42	139.32	338.52	RW2
Angola	CUANZA SUL	85-89	263.21	392.14	165.15	HT-Direct
Angola	CUANZA SUL	85-89	217.14	160.52	286.99	RW2
Angola	CUANZA SUL	90-94	268.58	376.47	182.56	HT-Direct
Angola	CUANZA SUL	90-94	244.80	197.98	298.81	RW2
Angola	CUANZA SUL	95-99	225.53	285.73	174.91	HT-Direct
Angola	CUANZA SUL	95-99	253.65	211.66	300.30	RW2

Continued on next page

Country	Region	Year	Median	Lower	Upper	Method
Angola	CUANZA SUL	00-04	201.75	284.21	138.59	HT-Direct
Angola	CUANZA SUL	00-04	275.07	220.81	337.17	RW2
Angola	CUANZA SUL	05-09	173.43	207.23	144.15	HT-Direct
Angola	CUANZA SUL	05-09	277.19	188.37	388.35	RW2
Angola	CUANZA SUL	10-14	111.11	154.93	78.53	HT-Direct
Angola	CUANZA SUL	10-14	264.54	187.38	359.78	RW2
Angola	CUANZA SUL	15-19	248.55	80.73	557.06	RW2
Angola	CUNENE	80-84	114.44	46.21	244.93	RW2
Angola	CUNENE	85-89	26.46	158.80	3.90	HT-Direct
Angola	CUNENE	85-89	119.82	68.96	196.42	RW2
Angola	CUNENE	90-94	162.50	228.99	112.50	HT-Direct
Angola	CUNENE	90-94	147.94	110.37	195.19	RW2
Angola	CUNENE	95-99	139.97	182.02	106.36	HT-Direct
Angola	CUNENE	95-99	161.94	131.41	197.84	RW2
Angola	CUNENE	00-04	178.73	242.58	128.83	HT-Direct
Angola	CUNENE	00-04	183.52	140.49	237.25	RW2
Angola	CUNENE	05-09	100.54	153.70	64.36	HT-Direct
Angola	CUNENE	05-09	188.07	117.10	288.73	RW2
Angola	CUNENE	10-14	59.55	89.76	39.07	HT-Direct
Angola	CUNENE	10-14	179.08	108.03	282.03	RW2
Angola	CUNENE	15-19	167.24	44.99	460.88	RW2
Angola	HUAMBO	80-84	420.65	626.69	238.98	HT-Direct
Angola	HUAMBO	80-84	301.64	221.49	394.39	RW2
Angola	HUAMBO	85-89	297.44	430.74	191.52	HT-Direct
Angola	HUAMBO	85-89	276.24	218.44	342.88	RW2
Angola	HUAMBO	90-94	338.47	441.04	249.11	HT-Direct
Angola	HUAMBO	90-94	287.52	238.45	342.94	RW2
Angola	HUAMBO	95-99	229.27	301.63	170.05	HT-Direct
Angola	HUAMBO	95-99	270.72	225.77	322.69	RW2
Angola	HUAMBO	00-04	225.22	296.42	167.07	HT-Direct
Angola	HUAMBO	00-04	263.53	211.78	323.65	RW2
Angola	HUAMBO	05-09	132.93	179.51	97.01	HT-Direct
Angola	HUAMBO	05-09	236.06	160.68	333.28	RW2
Angola	HUAMBO	10-14	73.48	96.21	55.78	HT-Direct
Angola	HUAMBO	10-14	197.53	143.12	265.31	RW2
Angola	HUAMBO	15-19	161.33	49.39	412.12	RW2
Angola	HUÍLA	80-84	489.05	924.26	69.83	HT-Direct
Angola	HUÍLA	80-84	246.63	156.09	369.85	RW2
Angola	HUÍLA	85-89	268.10	401.89	166.45	HT-Direct
Angola	HUÍLA	85-89	233.35	174.04	305.37	RW2
Angola	HUÍLA	90-94	248.32	328.09	182.68	HT-Direct
Angola	HUÍLA	90-94	255.43	209.47	307.91	RW2
Angola	HUÍLA	95-99	247.66	322.29	185.58	HT-Direct
Angola	HUÍLA	95-99	254.78	214.39	299.72	RW2
Angola	HUÍLA	00-04	185.56	228.04	149.46	HT-Direct
Angola	HUÍLA	00-04	265.88	219.65	317.35	RW2
Angola	HUÍLA	05-09	140.70	182.53	107.20	HT-Direct
Angola	HUÍLA	05-09	257.80	179.81	356.02	RW2
Angola	HUÍLA	10-14	99.42	129.59	75.66	HT-Direct
Angola	HUÍLA	10-14	236.71	177.01	309.04	RW2
Angola	HUÍLA	15-19	214.04	69.37	498.84	RW2
Angola	LUANDA	80-84	336.21	625.39	133.20	HT-Direct
Angola	LUANDA	80-84	191.71	117.26	294.76	RW2
Angola	LUANDA	85-89	129.35	262.95	58.27	HT-Direct
Angola	LUANDA	85-89	164.54	115.98	226.65	RW2
Angola	LUANDA	90-94	145.78	219.95	93.62	HT-Direct
Angola	LUANDA	90-94	169.20	132.42	213.09	RW2
Angola	LUANDA	95-99	170.36	217.74	131.55	HT-Direct
Angola	LUANDA	95-99	160.13	131.39	194.23	RW2
Angola	LUANDA	00-04	111.93	144.81	85.76	HT-Direct
Angola	LUANDA	00-04	157.62	123.93	199.09	RW2
Angola	LUANDA	05-09	73.93	103.88	52.11	HT-Direct

Continued on next page

Country	Region	Year	Median	Lower	Upper	Method
Angola	LUANDA	05-09	139.66	88.33	213.93	RW2
Angola	LUANDA	10-14	40.39	59.59	27.20	HT-Direct
Angola	LUANDA	10-14	114.17	70.24	179.05	RW2
Angola	LUANDA	15-19	91.90	23.73	293.55	RW2
Angola	LUNDA NORTE	80-84	193.46	83.79	387.91	RW2
Angola	LUNDA NORTE	85-89	171.61	351.28	73.44	HT-Direct
Angola	LUNDA NORTE	85-89	173.83	103.34	278.07	RW2
Angola	LUNDA NORTE	90-94	211.78	348.48	118.92	HT-Direct
Angola	LUNDA NORTE	90-94	183.91	129.87	253.73	RW2
Angola	LUNDA NORTE	95-99	167.23	244.71	110.68	HT-Direct
Angola	LUNDA NORTE	95-99	176.81	132.62	231.84	RW2
Angola	LUNDA NORTE	00-04	115.43	185.27	69.66	HT-Direct
Angola	LUNDA NORTE	00-04	180.85	129.36	245.83	RW2
Angola	LUNDA NORTE	05-09	62.90	107.24	36.15	HT-Direct
Angola	LUNDA NORTE	05-09	173.75	104.84	273.15	RW2
Angola	LUNDA NORTE	10-14	66.14	99.90	43.25	HT-Direct
Angola	LUNDA NORTE	10-14	158.73	93.56	256.28	RW2
Angola	LUNDA NORTE	15-19	143.19	37.82	423.32	RW2
Angola	LUNDA SUL	80-84	109.20	353.84	26.71	HT-Direct
Angola	LUNDA SUL	80-84	108.90	53.92	204.54	RW2
Angola	LUNDA SUL	85-89	15.23	105.87	2.02	HT-Direct
Angola	LUNDA SUL	85-89	105.99	63.36	171.73	RW2
Angola	LUNDA SUL	90-94	193.83	321.41	108.77	HT-Direct
Angola	LUNDA SUL	90-94	123.01	84.78	174.77	RW2
Angola	LUNDA SUL	95-99	143.73	241.71	81.21	HT-Direct
Angola	LUNDA SUL	95-99	127.56	93.35	172.49	RW2
Angola	LUNDA SUL	00-04	82.35	123.86	53.90	HT-Direct
Angola	LUNDA SUL	00-04	139.75	101.44	189.40	RW2
Angola	LUNDA SUL	05-09	46.97	70.00	31.26	HT-Direct
Angola	LUNDA SUL	05-09	141.73	86.66	222.70	RW2
Angola	LUNDA SUL	10-14	48.19	70.85	32.53	HT-Direct
Angola	LUNDA SUL	10-14	136.08	82.95	215.43	RW2
Angola	LUNDA SUL	15-19	129.21	34.99	379.73	RW2
Angola	MALANJE	80-84	579.93	986.73	25.00	HT-Direct
Angola	MALANJE	80-84	264.83	131.24	468.08	RW2
Angola	MALANJE	85-89	274.02	660.60	68.20	HT-Direct
Angola	MALANJE	85-89	216.82	129.29	342.15	RW2
Angola	MALANJE	90-94	198.51	336.01	108.12	HT-Direct
Angola	MALANJE	90-94	208.45	144.93	289.43	RW2
Angola	MALANJE	95-99	168.71	266.33	101.90	HT-Direct
Angola	MALANJE	95-99	185.66	139.92	241.23	RW2
Angola	MALANJE	00-04	125.26	171.96	89.86	HT-Direct
Angola	MALANJE	00-04	177.25	135.58	227.22	RW2
Angola	MALANJE	05-09	64.34	102.49	39.76	HT-Direct
Angola	MALANJE	05-09	157.73	101.88	236.56	RW2
Angola	MALANJE	10-14	54.22	73.86	39.57	HT-Direct
Angola	MALANJE	10-14	132.66	88.06	195.04	RW2
Angola	MALANJE	15-19	109.37	30.90	320.51	RW2
Angola	MOXICO	80-84	420.82	22.26	951.40	RW2
Angola	MOXICO	85-89	284.96	25.53	835.53	RW2
Angola	MOXICO	90-94	214.87	35.74	631.55	RW2
Angola	MOXICO	95-99	144.37	41.52	361.23	RW2
Angola	MOXICO	00-04	8.73	56.86	1.29	HT-Direct
Angola	MOXICO	00-04	100.98	41.93	210.40	RW2
Angola	MOXICO	05-09	1.33	9.89	0.18	HT-Direct
Angola	MOXICO	05-09	64.80	22.69	170.38	RW2
Angola	MOXICO	10-14	18.10	44.66	7.22	HT-Direct
Angola	MOXICO	10-14	39.12	8.51	163.55	RW2
Angola	MOXICO	15-19	23.17	1.88	228.51	RW2
Angola	NAMIBE	80-84	291.47	586.81	106.47	HT-Direct
Angola	NAMIBE	80-84	187.91	112.15	293.95	RW2
Angola	NAMIBE	85-89	150.73	320.17	62.69	HT-Direct
Angola	NAMIBE	85-89	187.73	131.24	261.06	RW2

Continued on next page

Country	Region	Year	Median	Lower	Upper	Method
Angola	NAMIBE	90-94	223.44	324.81	146.82	HT-Direct
Angola	NAMIBE	90-94	217.18	168.81	275.41	RW2
Angola	NAMIBE	95-99	218.53	294.91	157.51	HT-Direct
Angola	NAMIBE	95-99	223.17	178.55	276.85	RW2
Angola	NAMIBE	00-04	183.89	259.15	126.74	HT-Direct
Angola	NAMIBE	00-04	234.44	180.41	299.39	RW2
Angola	NAMIBE	05-09	132.11	187.89	91.03	HT-Direct
Angola	NAMIBE	05-09	222.75	145.23	325.79	RW2
Angola	NAMIBE	10-14	71.44	100.25	50.44	HT-Direct
Angola	NAMIBE	10-14	197.56	131.06	284.00	RW2
Angola	NAMIBE	15-19	170.59	49.46	448.96	RW2
Angola	UÍGE	80-84	694.11	878.49	415.95	HT-Direct
Angola	UÍGE	80-84	390.28	277.44	524.09	RW2
Angola	UÍGE	85-89	431.39	609.64	269.31	HT-Direct
Angola	UÍGE	85-89	293.74	222.37	376.10	RW2
Angola	UÍGE	90-94	182.55	285.37	111.02	HT-Direct
Angola	UÍGE	90-94	254.00	195.77	318.83	RW2
Angola	UÍGE	95-99	157.17	237.29	100.54	HT-Direct
Angola	UÍGE	95-99	210.63	162.59	263.11	RW2
Angola	UÍGE	00-04	155.05	205.94	114.91	HT-Direct
Angola	UÍGE	00-04	190.29	146.06	242.81	RW2
Angola	UÍGE	05-09	100.91	150.54	66.36	HT-Direct
Angola	UÍGE	05-09	160.43	103.89	238.66	RW2
Angola	UÍGE	10-14	58.50	78.62	43.29	HT-Direct
Angola	UÍGE	10-14	126.76	86.46	183.65	RW2
Angola	UÍGE	15-19	97.68	27.61	292.41	RW2
Angola	ZAIRE	80-84	73.36	27.47	191.95	RW2
Angola	ZAIRE	85-89	76.72	204.35	26.18	HT-Direct
Angola	ZAIRE	85-89	70.64	37.55	130.03	RW2
Angola	ZAIRE	90-94	67.85	134.50	32.97	HT-Direct
Angola	ZAIRE	90-94	83.32	54.91	124.25	RW2
Angola	ZAIRE	95-99	85.15	130.15	54.73	HT-Direct
Angola	ZAIRE	95-99	93.36	67.63	127.21	RW2
Angola	ZAIRE	00-04	111.82	175.54	69.29	HT-Direct
Angola	ZAIRE	00-04	113.69	80.46	159.50	RW2
Angola	ZAIRE	05-09	78.01	128.45	46.32	HT-Direct
Angola	ZAIRE	05-09	124.74	74.57	202.46	RW2
Angola	ZAIRE	10-14	44.31	64.82	30.08	HT-Direct
Angola	ZAIRE	10-14	126.81	75.20	205.41	RW2
Angola	ZAIRE	15-19	126.76	32.44	383.78	RW2
Benin	ALL	80-84	219.81	230.57	209.41	HT-Direct
Benin	ALL	80-84	213.87	210.96	216.86	IHME
Benin	ALL	80-84	210.20	200.64	220.10	RW2
Benin	ALL	80-84	210.24	204.33	216.73	UN
Benin	ALL	85-89	195.59	204.27	187.20	HT-Direct
Benin	ALL	85-89	191.79	189.49	194.29	IHME
Benin	ALL	85-89	192.37	184.32	200.65	RW2
Benin	ALL	85-89	192.27	186.66	198.04	UN
Benin	ALL	90-94	169.56	176.23	163.09	HT-Direct
Benin	ALL	90-94	172.46	170.20	174.63	IHME
Benin	ALL	90-94	170.36	163.93	177.00	RW2
Benin	ALL	90-94	170.47	165.82	175.17	UN
Benin	ALL	95-99	157.73	164.11	151.55	HT-Direct
Benin	ALL	95-99	152.56	150.34	154.78	IHME
Benin	ALL	95-99	153.61	147.78	159.58	RW2
Benin	ALL	95-99	153.58	149.23	158.09	UN
Benin	ALL	00-04	137.32	144.44	130.49	HT-Direct
Benin	ALL	00-04	130.49	128.21	132.69	IHME
Benin	ALL	00-04	136.99	130.31	143.99	RW2
Benin	ALL	00-04	136.92	132.18	141.28	UN
Benin	ALL	05-09	104.32	116.61	93.18	HT-Direct
Benin	ALL	05-09	107.75	105.25	110.37	IHME

Continued on next page

Country	Region	Year	Median	Lower	Upper	Method
Benin	ALL	05-09	118.12	103.83	134.08	RW2
Benin	ALL	05-09	118.35	113.03	124.29	UN
Benin	ALL	10-14	88.79	85.44	92.52	IHME
Benin	ALL	10-14	100.31	38.17	235.56	RW2
Benin	ALL	10-14	106.29	98.52	115.37	UN
Benin	ATACORA	80-84	290.00	315.66	265.61	HT-Direct
Benin	ATACORA	80-84	269.43	250.07	289.97	RW2
Benin	ATACORA	85-89	237.50	256.40	219.59	HT-Direct
Benin	ATACORA	85-89	239.27	225.75	252.98	RW2
Benin	ATACORA	90-94	205.36	222.70	189.04	HT-Direct
Benin	ATACORA	90-94	211.46	199.56	223.69	RW2
Benin	ATACORA	95-99	202.99	219.10	187.77	HT-Direct
Benin	ATACORA	95-99	192.08	181.34	203.35	RW2
Benin	ATACORA	00-04	170.83	190.14	153.11	HT-Direct
Benin	ATACORA	00-04	173.59	159.91	188.37	RW2
Benin	ATACORA	05-09	139.52	173.80	111.09	HT-Direct
Benin	ATACORA	05-09	150.79	126.74	178.95	RW2
Benin	ATACORA	10-14	128.39	54.04	274.00	RW2
Benin	ATACORA	15-19	108.71	11.83	546.08	RW2
Benin	ATLANTIQUE	80-84	179.55	201.53	159.48	HT-Direct
Benin	ATLANTIQUE	80-84	171.63	155.85	188.66	RW2
Benin	ATLANTIQUE	85-89	163.21	183.00	145.19	HT-Direct
Benin	ATLANTIQUE	85-89	153.80	142.83	165.58	RW2
Benin	ATLANTIQUE	90-94	126.24	139.82	113.80	HT-Direct
Benin	ATLANTIQUE	90-94	134.31	125.47	143.42	RW2
Benin	ATLANTIQUE	95-99	124.64	136.40	113.77	HT-Direct
Benin	ATLANTIQUE	95-99	121.24	113.57	129.31	RW2
Benin	ATLANTIQUE	00-04	115.02	128.93	102.43	HT-Direct
Benin	ATLANTIQUE	00-04	111.03	101.64	121.36	RW2
Benin	ATLANTIQUE	05-09	80.50	104.68	61.52	HT-Direct
Benin	ATLANTIQUE	05-09	98.12	81.56	118.03	RW2
Benin	ATLANTIQUE	10-14	85.53	35.34	193.84	RW2
Benin	ATLANTIQUE	15-19	73.90	8.03	435.52	RW2
Benin	BORGOU	80-84	213.43	239.38	189.59	HT-Direct
Benin	BORGOU	80-84	207.22	187.95	227.75	RW2
Benin	BORGOU	85-89	194.27	216.02	174.23	HT-Direct
Benin	BORGOU	85-89	195.00	181.62	209.18	RW2
Benin	BORGOU	90-94	184.89	203.83	167.33	HT-Direct
Benin	BORGOU	90-94	179.74	168.23	192.27	RW2
Benin	BORGOU	95-99	174.96	191.97	159.17	HT-Direct
Benin	BORGOU	95-99	166.02	155.09	177.78	RW2
Benin	BORGOU	00-04	141.96	163.68	122.70	HT-Direct
Benin	BORGOU	00-04	150.40	135.61	166.29	RW2
Benin	BORGOU	05-09	110.61	145.48	83.29	HT-Direct
Benin	BORGOU	05-09	130.48	105.74	158.21	RW2
Benin	BORGOU	10-14	111.02	45.50	243.60	RW2
Benin	BORGOU	15-19	93.29	10.08	505.17	RW2
Benin	MONO	80-84	204.65	231.64	180.07	HT-Direct
Benin	MONO	80-84	200.29	180.72	221.16	RW2
Benin	MONO	85-89	182.97	203.22	164.33	HT-Direct
Benin	MONO	85-89	178.87	166.30	192.17	RW2
Benin	MONO	90-94	155.61	170.84	141.52	HT-Direct
Benin	MONO	90-94	154.41	144.56	165.12	RW2
Benin	MONO	95-99	141.13	157.00	126.62	HT-Direct
Benin	MONO	95-99	134.49	125.12	144.42	RW2
Benin	MONO	00-04	112.33	127.30	98.92	HT-Direct
Benin	MONO	00-04	116.72	105.78	128.66	RW2
Benin	MONO	05-09	89.04	121.94	64.37	HT-Direct
Benin	MONO	05-09	97.56	78.95	119.27	RW2
Benin	MONO	10-14	79.99	32.10	185.68	RW2
Benin	MONO	15-19	65.76	6.86	410.35	RW2
Benin	OUEME	80-84	212.06	240.44	186.20	HT-Direct
Benin	OUEME	80-84	204.85	185.25	226.06	RW2

Continued on next page

Country	Region	Year	Median	Lower	Upper	Method
Benin	OUEME	85-89	194.26	213.37	176.48	HT-Direct
Benin	OUEME	85-89	187.77	175.45	200.89	RW2
Benin	OUEME	90-94	165.29	179.90	151.64	HT-Direct
Benin	OUEME	90-94	167.77	157.66	178.20	RW2
Benin	OUEME	95-99	160.48	176.84	145.37	HT-Direct
Benin	OUEME	95-99	154.00	143.99	164.48	RW2
Benin	OUEME	00-04	138.31	154.71	123.40	HT-Direct
Benin	OUEME	00-04	143.47	131.56	156.42	RW2
Benin	OUEME	05-09	130.33	162.18	103.96	HT-Direct
Benin	OUEME	05-09	130.33	109.21	155.32	RW2
Benin	OUEME	10-14	116.77	48.78	253.84	RW2
Benin	OUEME	15-19	104.58	11.75	530.27	RW2
Benin	ZOU	80-84	233.74	260.97	208.55	HT-Direct
Benin	ZOU	80-84	224.20	205.09	244.77	RW2
Benin	ZOU	85-89	201.71	224.15	181.00	HT-Direct
Benin	ZOU	85-89	198.81	185.78	212.65	RW2
Benin	ZOU	90-94	179.20	194.89	164.52	HT-Direct
Benin	ZOU	90-94	172.31	162.59	182.48	RW2
Benin	ZOU	95-99	144.26	156.87	132.50	HT-Direct
Benin	ZOU	95-99	151.86	143.11	160.93	RW2
Benin	ZOU	00-04	147.49	163.21	133.04	HT-Direct
Benin	ZOU	00-04	135.44	124.76	146.93	RW2
Benin	ZOU	05-09	82.36	113.91	58.97	HT-Direct
Benin	ZOU	05-09	115.79	96.90	137.97	RW2
Benin	ZOU	10-14	97.41	40.36	216.81	RW2
Benin	ZOU	15-19	82.14	8.90	460.26	RW2
Burkina Faso	ALL	80-84	237.59	247.59	227.87	HT-Direct
Burkina Faso	ALL	80-84	242.41	239.49	245.15	IHME
Burkina Faso	ALL	80-84	232.08	222.75	241.67	RW2
Burkina Faso	ALL	80-84	232.03	224.90	238.70	UN
Burkina Faso	ALL	85-89	206.30	214.07	198.74	HT-Direct
Burkina Faso	ALL	85-89	218.13	215.86	220.79	IHME
Burkina Faso	ALL	85-89	209.98	202.20	217.93	RW2
Burkina Faso	ALL	85-89	210.07	204.38	216.27	UN
Burkina Faso	ALL	90-94	202.29	208.95	195.79	HT-Direct
Burkina Faso	ALL	90-94	202.65	200.30	204.84	IHME
Burkina Faso	ALL	90-94	201.84	195.42	208.43	RW2
Burkina Faso	ALL	90-94	201.77	196.36	207.54	UN
Burkina Faso	ALL	95-99	194.95	201.74	188.34	HT-Direct
Burkina Faso	ALL	95-99	188.88	186.70	191.42	IHME
Burkina Faso	ALL	95-99	193.74	187.24	200.36	RW2
Burkina Faso	ALL	95-99	193.79	188.27	200.09	UN
Burkina Faso	ALL	00-04	175.49	183.15	168.08	HT-Direct
Burkina Faso	ALL	00-04	168.26	165.68	170.79	IHME
Burkina Faso	ALL	00-04	176.96	169.51	184.71	RW2
Burkina Faso	ALL	00-04	176.96	170.60	183.88	UN
Burkina Faso	ALL	05-09	132.49	140.13	125.21	HT-Direct
Burkina Faso	ALL	05-09	144.04	140.79	147.39	IHME
Burkina Faso	ALL	05-09	139.53	131.41	148.06	RW2
Burkina Faso	ALL	05-09	139.54	133.63	145.72	UN
Burkina Faso	ALL	10-14	122.58	118.41	127.04	IHME
Burkina Faso	ALL	10-14	103.58	40.39	236.89	RW2
Burkina Faso	ALL	10-14	102.06	93.11	111.53	UN
Burkina Faso	CENTRAL/SOUTH	80-84	235.28	252.06	219.28	HT-Direct
Burkina Faso	CENTRAL/SOUTH	80-84	216.67	202.70	231.13	RW2
Burkina Faso	CENTRAL/SOUTH	85-89	187.11	199.35	175.46	HT-Direct
Burkina Faso	CENTRAL/SOUTH	85-89	203.32	193.13	214.06	RW2
Burkina Faso	CENTRAL/SOUTH	90-94	180.67	191.09	170.69	HT-Direct
Burkina Faso	CENTRAL/SOUTH	90-94	201.46	191.83	211.50	RW2
Burkina Faso	CENTRAL/SOUTH	95-99	177.16	187.85	166.95	HT-Direct
Burkina Faso	CENTRAL/SOUTH	95-99	195.31	185.52	205.52	RW2
Burkina Faso	CENTRAL/SOUTH	00-04	145.59	156.55	135.28	HT-Direct
Burkina Faso	CENTRAL/SOUTH	00-04	179.66	168.84	191.14	RW2

Continued on next page

Country	Region	Year	Median	Lower	Upper	Method
Burkina Faso	CENTRAL/SOUTH	05-09	109.25	121.11	98.43	HT-Direct
Burkina Faso	CENTRAL/SOUTH	05-09	142.91	129.38	157.46	RW2
Burkina Faso	CENTRAL/SOUTH	10-14	107.21	47.01	226.55	RW2
Burkina Faso	CENTRAL/SOUTH	15-19	78.90	8.82	443.68	RW2
Burkina Faso	EAST	80-84	263.51	287.21	241.11	HT-Direct
Burkina Faso	EAST	80-84	257.92	239.19	277.88	RW2
Burkina Faso	EAST	85-89	222.35	242.60	203.34	HT-Direct
Burkina Faso	EAST	85-89	226.38	213.11	240.16	RW2
Burkina Faso	EAST	90-94	213.19	228.46	198.68	HT-Direct
Burkina Faso	EAST	90-94	210.81	200.04	221.98	RW2
Burkina Faso	EAST	95-99	193.41	207.86	179.75	HT-Direct
Burkina Faso	EAST	95-99	194.16	183.89	204.83	RW2
Burkina Faso	EAST	00-04	168.41	187.29	151.09	HT-Direct
Burkina Faso	EAST	00-04	170.34	158.43	183.07	RW2
Burkina Faso	EAST	05-09	125.02	142.50	109.40	HT-Direct
Burkina Faso	EAST	05-09	129.97	115.93	145.58	RW2
Burkina Faso	EAST	10-14	93.32	39.90	201.19	RW2
Burkina Faso	EAST	15-19	66.11	7.28	403.03	RW2
Burkina Faso	NORTH	80-84	233.39	257.81	210.63	HT-Direct
Burkina Faso	NORTH	80-84	224.74	211.26	238.88	RW2
Burkina Faso	NORTH	85-89	229.12	246.29	212.81	HT-Direct
Burkina Faso	NORTH	85-89	195.25	185.86	204.99	RW2
Burkina Faso	NORTH	90-94	231.48	248.44	215.35	HT-Direct
Burkina Faso	NORTH	90-94	182.83	174.85	191.00	RW2
Burkina Faso	NORTH	95-99	234.46	251.92	217.86	HT-Direct
Burkina Faso	NORTH	95-99	170.71	163.02	178.72	RW2
Burkina Faso	NORTH	00-04	228.79	248.25	210.42	HT-Direct
Burkina Faso	NORTH	00-04	150.03	141.69	158.90	RW2
Burkina Faso	NORTH	05-09	183.59	203.35	165.35	HT-Direct
Burkina Faso	NORTH	05-09	114.15	104.13	125.00	RW2
Burkina Faso	NORTH	10-14	81.85	35.12	177.19	RW2
Burkina Faso	NORTH	15-19	57.96	6.36	367.23	RW2
Burkina Faso	WEST	80-84	219.42	236.24	203.49	HT-Direct
Burkina Faso	WEST	80-84	233.50	215.16	252.69	RW2
Burkina Faso	WEST	85-89	200.83	214.64	187.69	HT-Direct
Burkina Faso	WEST	85-89	226.80	214.52	239.47	RW2
Burkina Faso	WEST	90-94	205.41	218.87	192.57	HT-Direct
Burkina Faso	WEST	90-94	232.52	221.20	244.37	RW2
Burkina Faso	WEST	95-99	191.78	205.57	178.70	HT-Direct
Burkina Faso	WEST	95-99	235.74	224.02	247.81	RW2
Burkina Faso	WEST	00-04	181.98	197.44	167.48	HT-Direct
Burkina Faso	WEST	00-04	228.14	214.80	242.14	RW2
Burkina Faso	WEST	05-09	133.38	148.88	119.26	HT-Direct
Burkina Faso	WEST	05-09	193.66	176.90	211.83	RW2
Burkina Faso	WEST	10-14	155.58	70.09	308.32	RW2
Burkina Faso	WEST	15-19	123.44	14.09	570.02	RW2
Burundi	ALL	80-84	220.17	273.54	174.70	HT-Direct
Burundi	ALL	80-84	202.90	196.35	208.65	IHME
Burundi	ALL	80-84	202.14	163.91	246.94	RW2
Burundi	ALL	80-84	201.57	193.32	211.06	UN
Burundi	ALL	85-89	177.88	204.32	154.20	HT-Direct
Burundi	ALL	85-89	174.26	169.47	179.73	IHME
Burundi	ALL	85-89	172.52	152.02	194.58	RW2
Burundi	ALL	85-89	172.35	164.06	180.82	UN
Burundi	ALL	90-94	194.77	215.33	175.74	HT-Direct
Burundi	ALL	90-94	174.19	169.16	179.53	IHME
Burundi	ALL	90-94	173.31	158.82	188.95	RW2
Burundi	ALL	90-94	173.74	165.42	182.23	UN
Burundi	ALL	95-99	207.96	226.38	190.67	HT-Direct
Burundi	ALL	95-99	172.33	167.23	177.79	IHME
Burundi	ALL	95-99	164.52	153.42	176.14	RW2
Burundi	ALL	95-99	164.77	156.21	173.93	UN
Burundi	ALL	00-04	181.35	195.11	168.35	HT-Direct

Continued on next page

Country	Region	Year	Median	Lower	Upper	Method
Burundi	ALL	00-04	149.22	144.17	154.66	IHME
Burundi	ALL	00-04	143.59	135.13	152.53	RW2
Burundi	ALL	00-04	142.94	134.94	151.10	UN
Burundi	ALL	05-09	100.90	109.38	93.01	HT-Direct
Burundi	ALL	05-09	99.50	95.67	103.48	IHME
Burundi	ALL	05-09	114.07	103.87	125.14	RW2
Burundi	ALL	05-09	114.34	104.29	124.91	UN
Burundi	ALL	10-14	82.36	74.46	90.07	IHME
Burundi	ALL	10-14	87.32	31.01	218.25	RW2
Burundi	ALL	10-14	92.23	78.38	109.08	UN
Burundi	BUJUMBURA	80-84	237.56	389.25	132.18	HT-Direct
Burundi	BUJUMBURA	80-84	186.14	118.57	288.34	RW2
Burundi	BUJUMBURA	85-89	138.40	240.16	75.47	HT-Direct
Burundi	BUJUMBURA	85-89	133.40	96.33	181.29	RW2
Burundi	BUJUMBURA	90-94	101.43	154.75	65.07	HT-Direct
Burundi	BUJUMBURA	90-94	114.59	87.59	146.46	RW2
Burundi	BUJUMBURA	95-99	148.69	218.95	98.14	HT-Direct
Burundi	BUJUMBURA	95-99	98.61	78.39	122.21	RW2
Burundi	BUJUMBURA	00-04	86.48	122.71	60.22	HT-Direct
Burundi	BUJUMBURA	00-04	77.61	62.65	95.46	RW2
Burundi	BUJUMBURA	05-09	56.89	77.28	41.63	HT-Direct
Burundi	BUJUMBURA	05-09	58.74	43.17	80.34	RW2
Burundi	BUJUMBURA	10-14	44.27	14.32	134.58	RW2
Burundi	BUJUMBURA	15-19	33.49	2.36	358.63	RW2
Burundi	CENTRE-EAST	80-84	313.83	457.10	199.00	HT-Direct
Burundi	CENTRE-EAST	80-84	203.27	146.40	285.79	RW2
Burundi	CENTRE-EAST	85-89	152.09	198.98	114.67	HT-Direct
Burundi	CENTRE-EAST	85-89	161.60	134.30	193.81	RW2
Burundi	CENTRE-EAST	90-94	168.81	200.59	141.17	HT-Direct
Burundi	CENTRE-EAST	90-94	154.14	135.59	173.90	RW2
Burundi	CENTRE-EAST	95-99	180.82	209.82	155.04	HT-Direct
Burundi	CENTRE-EAST	95-99	148.11	133.88	163.11	RW2
Burundi	CENTRE-EAST	00-04	179.53	207.30	154.75	HT-Direct
Burundi	CENTRE-EAST	00-04	134.06	121.55	148.22	RW2
Burundi	CENTRE-EAST	05-09	92.76	108.54	79.08	HT-Direct
Burundi	CENTRE-EAST	05-09	109.01	93.01	127.46	RW2
Burundi	CENTRE-EAST	10-14	84.85	32.72	202.48	RW2
Burundi	CENTRE-EAST	15-19	65.02	5.63	451.28	RW2
Burundi	NORTH	80-84	207.97	314.69	130.54	HT-Direct
Burundi	NORTH	80-84	184.52	130.46	250.53	RW2
Burundi	NORTH	85-89	160.34	213.88	118.18	HT-Direct
Burundi	NORTH	85-89	174.22	142.68	209.40	RW2
Burundi	NORTH	90-94	223.90	265.46	187.18	HT-Direct
Burundi	NORTH	90-94	191.07	168.67	216.47	RW2
Burundi	NORTH	95-99	250.90	295.09	211.34	HT-Direct
Burundi	NORTH	95-99	192.42	173.37	213.87	RW2
Burundi	NORTH	00-04	210.64	239.95	184.04	HT-Direct
Burundi	NORTH	00-04	172.62	157.31	188.95	RW2
Burundi	NORTH	05-09	130.72	148.74	114.59	HT-Direct
Burundi	NORTH	05-09	144.22	125.62	165.16	RW2
Burundi	NORTH	10-14	116.80	45.84	265.72	RW2
Burundi	NORTH	15-19	93.55	8.04	559.64	RW2
Burundi	SOUTH	80-84	203.48	291.21	137.07	HT-Direct
Burundi	SOUTH	80-84	218.88	164.37	287.38	RW2
Burundi	SOUTH	85-89	228.74	292.34	175.55	HT-Direct
Burundi	SOUTH	85-89	173.87	143.34	210.32	RW2
Burundi	SOUTH	90-94	151.94	198.30	114.86	HT-Direct
Burundi	SOUTH	90-94	152.59	130.89	177.25	RW2
Burundi	SOUTH	95-99	164.09	193.32	138.52	HT-Direct
Burundi	SOUTH	95-99	132.11	117.45	147.28	RW2
Burundi	SOUTH	00-04	138.80	158.69	121.04	HT-Direct
Burundi	SOUTH	00-04	110.82	100.65	121.93	RW2
Burundi	SOUTH	05-09	81.50	97.90	67.64	HT-Direct

Continued on next page

Country	Region	Year	Median	Lower	Upper	Method
Burundi	SOUTH	05-09	87.93	73.18	106.22	RW2
Burundi	SOUTH	10-14	68.02	25.20	173.35	RW2
Burundi	SOUTH	15-19	52.71	4.31	413.21	RW2
Burundi	WEST	80-84	166.54	258.40	102.80	HT-Direct
Burundi	WEST	80-84	195.53	135.00	266.75	RW2
Burundi	WEST	85-89	180.68	239.49	133.78	HT-Direct
Burundi	WEST	85-89	187.12	153.64	225.21	RW2
Burundi	WEST	90-94	253.72	309.47	205.03	HT-Direct
Burundi	WEST	90-94	200.39	174.87	229.88	RW2
Burundi	WEST	95-99	245.65	292.03	204.50	HT-Direct
Burundi	WEST	95-99	193.27	172.41	217.85	RW2
Burundi	WEST	00-04	208.71	246.05	175.72	HT-Direct
Burundi	WEST	00-04	161.80	144.84	180.47	RW2
Burundi	WEST	05-09	96.47	118.86	77.93	HT-Direct
Burundi	WEST	05-09	120.06	96.81	146.00	RW2
Burundi	WEST	10-14	84.90	31.35	205.07	RW2
Burundi	WEST	15-19	58.55	4.90	430.22	RW2
Cameroon	ADAM/NORD/EXT-NORD	80-84	219.24	245.15	195.36	HT-Direct
Cameroon	ADAM/NORD/EXT-NORD	80-84	242.28	218.86	267.71	RW2
Cameroon	ADAM/NORD/EXT-NORD	85-89	197.13	214.59	180.76	HT-Direct
Cameroon	ADAM/NORD/EXT-NORD	85-89	198.95	185.03	213.61	RW2
Cameroon	ADAM/NORD/EXT-NORD	90-94	196.96	211.34	183.33	HT-Direct
Cameroon	ADAM/NORD/EXT-NORD	90-94	195.57	184.65	207.00	RW2
Cameroon	ADAM/NORD/EXT-NORD	95-99	198.76	214.58	183.83	HT-Direct
Cameroon	ADAM/NORD/EXT-NORD	95-99	202.45	190.76	214.52	RW2
Cameroon	ADAM/NORD/EXT-NORD	00-04	182.22	195.51	169.64	HT-Direct
Cameroon	ADAM/NORD/EXT-NORD	00-04	185.29	175.08	195.88	RW2
Cameroon	ADAM/NORD/EXT-NORD	05-09	169.03	184.41	154.68	HT-Direct
Cameroon	ADAM/NORD/EXT-NORD	05-09	152.49	142.35	163.26	RW2
Cameroon	ADAM/NORD/EXT-NORD	10-14	149.39	175.90	126.26	HT-Direct
Cameroon	ADAM/NORD/EXT-NORD	10-14	134.00	117.11	153.30	RW2
Cameroon	ADAM/NORD/EXT-NORD	15-19	121.25	53.91	254.20	RW2
Cameroon	ALL	80-84	159.28	171.85	147.47	HT-Direct
Cameroon	ALL	80-84	158.55	156.21	160.95	IHME
Cameroon	ALL	80-84	169.36	155.99	183.63	RW2
Cameroon	ALL	80-84	169.28	163.27	175.03	UN
Cameroon	ALL	85-89	134.49	142.92	126.49	HT-Direct
Cameroon	ALL	85-89	140.84	138.75	142.90	IHME
Cameroon	ALL	85-89	141.89	133.22	150.96	RW2
Cameroon	ALL	85-89	142.18	137.56	146.54	UN
Cameroon	ALL	90-94	144.50	152.09	137.23	HT-Direct
Cameroon	ALL	90-94	138.19	136.10	140.09	IHME
Cameroon	ALL	90-94	143.56	136.57	150.85	RW2
Cameroon	ALL	90-94	143.13	138.46	148.01	UN
Cameroon	ALL	95-99	150.04	157.95	142.46	HT-Direct
Cameroon	ALL	95-99	140.21	137.84	142.57	IHME
Cameroon	ALL	95-99	153.74	145.96	161.82	RW2
Cameroon	ALL	95-99	154.14	148.71	159.73	UN
Cameroon	ALL	00-04	142.83	150.15	135.80	HT-Direct
Cameroon	ALL	00-04	132.62	130.14	135.27	IHME
Cameroon	ALL	00-04	139.93	133.35	146.81	RW2
Cameroon	ALL	00-04	139.95	133.56	146.00	UN
Cameroon	ALL	05-09	127.48	135.69	119.70	HT-Direct
Cameroon	ALL	05-09	117.53	114.49	120.78	IHME
Cameroon	ALL	05-09	116.82	110.32	123.65	RW2
Cameroon	ALL	05-09	116.63	105.17	128.48	UN
Cameroon	ALL	10-14	104.36	118.38	91.84	HT-Direct
Cameroon	ALL	10-14	99.64	95.07	104.52	IHME
Cameroon	ALL	10-14	98.48	87.41	110.70	RW2
Cameroon	ALL	10-14	98.85	80.91	120.50	UN
Cameroon	CENTRE/SUD/EST	80-84	148.34	173.72	126.10	HT-Direct
Cameroon	CENTRE/SUD/EST	80-84	159.13	138.62	181.61	RW2
Cameroon	CENTRE/SUD/EST	85-89	123.21	138.17	109.67	HT-Direct

Continued on next page

Country	Region	Year	Median	Lower	Upper	Method
Cameroon	CENTRE/SUD/EST	85-89	131.98	120.48	144.19	RW2
Cameroon	CENTRE/SUD/EST	90-94	138.00	153.23	124.06	HT-Direct
Cameroon	CENTRE/SUD/EST	90-94	134.73	125.08	145.12	RW2
Cameroon	CENTRE/SUD/EST	95-99	137.79	151.57	125.09	HT-Direct
Cameroon	CENTRE/SUD/EST	95-99	144.35	134.58	154.87	RW2
Cameroon	CENTRE/SUD/EST	00-04	143.06	158.49	128.90	HT-Direct
Cameroon	CENTRE/SUD/EST	00-04	132.44	123.17	142.55	RW2
Cameroon	CENTRE/SUD/EST	05-09	108.10	122.82	94.95	HT-Direct
Cameroon	CENTRE/SUD/EST	05-09	103.32	94.02	113.50	RW2
Cameroon	CENTRE/SUD/EST	10-14	88.00	120.02	63.90	HT-Direct
Cameroon	CENTRE/SUD/EST	10-14	84.67	69.13	102.25	RW2
Cameroon	CENTRE/SUD/EST	15-19	71.07	29.18	162.97	RW2
Cameroon	NORD-OUEST/SUD-OUEST	80-84	121.50	145.99	100.64	HT-Direct
Cameroon	NORD-OUEST/SUD-OUEST	80-84	122.46	104.66	142.71	RW2
Cameroon	NORD-OUEST/SUD-OUEST	85-89	86.39	102.10	72.89	HT-Direct
Cameroon	NORD-OUEST/SUD-OUEST	85-89	99.98	89.33	111.77	RW2
Cameroon	NORD-OUEST/SUD-OUEST	90-94	104.27	119.32	90.92	HT-Direct
Cameroon	NORD-OUEST/SUD-OUEST	90-94	102.30	93.43	111.77	RW2
Cameroon	NORD-OUEST/SUD-OUEST	95-99	107.73	122.55	94.51	HT-Direct
Cameroon	NORD-OUEST/SUD-OUEST	95-99	110.95	101.92	120.83	RW2
Cameroon	NORD-OUEST/SUD-OUEST	00-04	111.34	128.37	96.32	HT-Direct
Cameroon	NORD-OUEST/SUD-OUEST	00-04	102.91	93.94	112.88	RW2
Cameroon	NORD-OUEST/SUD-OUEST	05-09	85.67	102.81	71.17	HT-Direct
Cameroon	NORD-OUEST/SUD-OUEST	05-09	81.60	72.15	92.13	RW2
Cameroon	NORD-OUEST/SUD-OUEST	10-14	67.56	98.00	46.10	HT-Direct
Cameroon	NORD-OUEST/SUD-OUEST	10-14	68.23	54.58	84.37	RW2
Cameroon	NORD-OUEST/SUD-OUEST	15-19	58.52	23.98	136.32	RW2
Cameroon	OUEST/LITTORAL	80-84	120.23	141.80	101.54	HT-Direct
Cameroon	OUEST/LITTORAL	80-84	125.26	107.90	145.22	RW2
Cameroon	OUEST/LITTORAL	85-89	97.43	115.77	81.73	HT-Direct
Cameroon	OUEST/LITTORAL	85-89	104.67	93.42	117.10	RW2
Cameroon	OUEST/LITTORAL	90-94	104.84	122.48	89.49	HT-Direct
Cameroon	OUEST/LITTORAL	90-94	109.46	99.64	119.99	RW2
Cameroon	OUEST/LITTORAL	95-99	123.76	137.69	111.06	HT-Direct
Cameroon	OUEST/LITTORAL	95-99	122.01	112.60	132.17	RW2
Cameroon	OUEST/LITTORAL	00-04	116.38	133.09	101.53	HT-Direct
Cameroon	OUEST/LITTORAL	00-04	116.87	106.81	127.84	RW2
Cameroon	OUEST/LITTORAL	05-09	107.68	132.79	86.85	HT-Direct
Cameroon	OUEST/LITTORAL	05-09	97.29	85.49	110.54	RW2
Cameroon	OUEST/LITTORAL	10-14	88.84	126.66	61.51	HT-Direct
Cameroon	OUEST/LITTORAL	10-14	85.77	68.41	106.58	RW2
Cameroon	OUEST/LITTORAL	15-19	77.59	32.01	177.60	RW2
Cameroon	YAOUNDE/DOUALA	80-84	119.33	156.26	90.19	HT-Direct
Cameroon	YAOUNDE/DOUALA	80-84	119.33	96.39	147.62	RW2
Cameroon	YAOUNDE/DOUALA	85-89	86.58	109.53	68.07	HT-Direct
Cameroon	YAOUNDE/DOUALA	85-89	93.92	80.71	108.93	RW2
Cameroon	YAOUNDE/DOUALA	90-94	93.75	113.08	77.44	HT-Direct
Cameroon	YAOUNDE/DOUALA	90-94	92.88	82.55	104.28	RW2
Cameroon	YAOUNDE/DOUALA	95-99	89.72	106.92	75.06	HT-Direct
Cameroon	YAOUNDE/DOUALA	95-99	98.27	88.27	109.05	RW2
Cameroon	YAOUNDE/DOUALA	00-04	94.35	111.87	79.33	HT-Direct
Cameroon	YAOUNDE/DOUALA	00-04	89.53	80.58	99.55	RW2
Cameroon	YAOUNDE/DOUALA	05-09	78.76	94.65	65.34	HT-Direct
Cameroon	YAOUNDE/DOUALA	05-09	69.76	60.89	79.76	RW2
Cameroon	YAOUNDE/DOUALA	10-14	48.81	84.64	27.69	HT-Direct
Cameroon	YAOUNDE/DOUALA	10-14	57.03	43.91	73.39	RW2
Cameroon	YAOUNDE/DOUALA	15-19	48.07	18.83	117.04	RW2
Chad	ALL	80-84	219.52	246.09	195.08	HT-Direct
Chad	ALL	80-84	229.89	226.29	233.50	IHME
Chad	ALL	80-84	237.13	209.02	267.75	RW2
Chad	ALL	80-84	236.47	227.15	245.73	UN
Chad	ALL	85-89	192.52	211.24	175.10	HT-Direct
Chad	ALL	85-89	212.02	208.56	215.04	IHME

Continued on next page

Country	Region	Year	Median	Lower	Upper	Method
Chad	ALL	85-89	222.45	200.68	245.80	RW2
Chad	ALL	85-89	223.02	215.48	230.64	UN
Chad	ALL	90-94	188.47	202.46	175.23	HT-Direct
Chad	ALL	90-94	195.25	192.48	198.26	IHME
Chad	ALL	90-94	209.21	193.84	225.43	RW2
Chad	ALL	90-94	209.68	202.88	216.80	UN
Chad	ALL	95-99	198.86	211.08	187.17	HT-Direct
Chad	ALL	95-99	186.83	183.80	190.07	IHME
Chad	ALL	95-99	199.03	187.71	210.78	RW2
Chad	ALL	95-99	198.22	191.97	205.09	UN
Chad	ALL	00-04	176.99	188.39	166.13	HT-Direct
Chad	ALL	00-04	178.32	174.29	182.45	IHME
Chad	ALL	00-04	183.50	172.37	195.20	RW2
Chad	ALL	00-04	184.23	176.95	191.97	UN
Chad	ALL	05-09	165.84	180.90	151.80	HT-Direct
Chad	ALL	05-09	159.79	154.39	165.02	IHME
Chad	ALL	05-09	172.08	157.73	187.54	RW2
Chad	ALL	05-09	171.26	160.47	182.07	UN
Chad	ALL	10-14	131.06	138.79	123.71	HT-Direct
Chad	ALL	10-14	141.83	135.05	148.74	IHME
Chad	ALL	10-14	152.93	142.70	163.68	RW2
Chad	ALL	10-14	153.04	134.67	175.78	UN
Chad	ZONE 1	80-84	167.45	224.83	122.40	HT-Direct
Chad	ZONE 1	80-84	190.73	148.14	242.34	RW2
Chad	ZONE 1	85-89	148.35	193.06	112.54	HT-Direct
Chad	ZONE 1	85-89	189.30	159.13	224.28	RW2
Chad	ZONE 1	90-94	193.20	226.56	163.72	HT-Direct
Chad	ZONE 1	90-94	183.50	162.50	207.00	RW2
Chad	ZONE 1	95-99	164.39	187.35	143.75	HT-Direct
Chad	ZONE 1	95-99	171.58	156.07	188.15	RW2
Chad	ZONE 1	00-04	156.96	177.17	138.67	HT-Direct
Chad	ZONE 1	00-04	165.34	150.90	180.71	RW2
Chad	ZONE 1	05-09	157.94	185.07	134.13	HT-Direct
Chad	ZONE 1	05-09	164.45	146.88	183.76	RW2
Chad	ZONE 1	10-14	141.00	171.24	115.35	HT-Direct
Chad	ZONE 1	10-14	158.05	131.74	189.07	RW2
Chad	ZONE 1	15-19	149.13	67.40	298.47	RW2
Chad	ZONE 2	80-84	242.74	306.55	188.60	HT-Direct
Chad	ZONE 2	80-84	254.60	206.28	310.88	RW2
Chad	ZONE 2	85-89	184.89	234.51	143.80	HT-Direct
Chad	ZONE 2	85-89	222.74	188.95	260.52	RW2
Chad	ZONE 2	90-94	171.45	208.28	139.98	HT-Direct
Chad	ZONE 2	90-94	192.84	170.04	218.15	RW2
Chad	ZONE 2	95-99	170.44	196.51	147.19	HT-Direct
Chad	ZONE 2	95-99	163.08	146.99	180.35	RW2
Chad	ZONE 2	00-04	125.39	148.89	105.13	HT-Direct
Chad	ZONE 2	00-04	138.55	124.19	154.08	RW2
Chad	ZONE 2	05-09	110.69	135.88	89.69	HT-Direct
Chad	ZONE 2	05-09	121.67	106.58	138.26	RW2
Chad	ZONE 2	10-14	98.12	119.27	80.38	HT-Direct
Chad	ZONE 2	10-14	105.82	87.59	128.10	RW2
Chad	ZONE 2	15-19	91.60	40.21	195.05	RW2
Chad	ZONE 3	80-84	299.22	372.66	234.84	HT-Direct
Chad	ZONE 3	80-84	296.11	243.13	357.48	RW2
Chad	ZONE 3	85-89	236.85	299.84	183.63	HT-Direct
Chad	ZONE 3	85-89	262.23	224.99	303.66	RW2
Chad	ZONE 3	90-94	191.19	222.18	163.62	HT-Direct
Chad	ZONE 3	90-94	230.30	206.03	256.07	RW2
Chad	ZONE 3	95-99	206.20	235.94	179.34	HT-Direct
Chad	ZONE 3	95-99	199.03	181.51	217.60	RW2
Chad	ZONE 3	00-04	164.77	187.62	144.21	HT-Direct
Chad	ZONE 3	00-04	173.39	158.75	189.05	RW2
Chad	ZONE 3	05-09	146.73	167.58	128.07	HT-Direct

Continued on next page

Country	Region	Year	Median	Lower	Upper	Method
Chad	ZONE 3	05-09	154.59	139.69	170.70	RW2
Chad	ZONE 3	10-14	122.50	145.93	102.38	HT-Direct
Chad	ZONE 3	10-14	134.96	115.01	158.55	RW2
Chad	ZONE 3	15-19	116.53	52.95	237.10	RW2
Chad	ZONE 4	80-84	203.05	272.46	147.73	HT-Direct
Chad	ZONE 4	80-84	211.81	162.77	271.71	RW2
Chad	ZONE 4	85-89	140.08	192.51	100.16	HT-Direct
Chad	ZONE 4	85-89	186.80	153.48	225.67	RW2
Chad	ZONE 4	90-94	147.21	183.84	116.83	HT-Direct
Chad	ZONE 4	90-94	164.02	141.50	189.42	RW2
Chad	ZONE 4	95-99	145.52	173.16	121.64	HT-Direct
Chad	ZONE 4	95-99	140.78	125.15	158.13	RW2
Chad	ZONE 4	00-04	120.58	141.55	102.35	HT-Direct
Chad	ZONE 4	00-04	120.64	108.09	134.36	RW2
Chad	ZONE 4	05-09	93.41	109.41	79.54	HT-Direct
Chad	ZONE 4	05-09	106.11	94.24	119.04	RW2
Chad	ZONE 4	10-14	85.28	102.31	70.86	HT-Direct
Chad	ZONE 4	10-14	93.23	78.00	111.58	RW2
Chad	ZONE 4	15-19	81.80	35.57	176.12	RW2
Chad	ZONE 5	80-84	144.19	207.32	97.90	HT-Direct
Chad	ZONE 5	80-84	189.37	145.88	241.28	RW2
Chad	ZONE 5	85-89	189.24	248.24	141.62	HT-Direct
Chad	ZONE 5	85-89	184.43	152.60	221.26	RW2
Chad	ZONE 5	90-94	162.12	197.39	132.12	HT-Direct
Chad	ZONE 5	90-94	176.27	153.12	202.03	RW2
Chad	ZONE 5	95-99	152.60	192.95	119.43	HT-Direct
Chad	ZONE 5	95-99	163.89	145.64	183.76	RW2
Chad	ZONE 5	00-04	156.70	184.85	132.14	HT-Direct
Chad	ZONE 5	00-04	153.98	138.06	171.11	RW2
Chad	ZONE 5	05-09	123.34	153.27	98.58	HT-Direct
Chad	ZONE 5	05-09	146.67	129.40	165.87	RW2
Chad	ZONE 5	10-14	119.85	141.31	101.26	HT-Direct
Chad	ZONE 5	10-14	134.83	114.50	158.49	RW2
Chad	ZONE 5	15-19	121.95	56.40	244.40	RW2
Chad	ZONE 6	80-84	190.20	266.92	131.57	HT-Direct
Chad	ZONE 6	80-84	175.28	133.38	230.10	RW2
Chad	ZONE 6	85-89	159.45	202.65	124.03	HT-Direct
Chad	ZONE 6	85-89	171.25	141.66	205.18	RW2
Chad	ZONE 6	90-94	153.84	190.64	123.07	HT-Direct
Chad	ZONE 6	90-94	165.42	143.77	189.27	RW2
Chad	ZONE 6	95-99	125.35	151.68	103.04	HT-Direct
Chad	ZONE 6	95-99	158.68	140.89	176.98	RW2
Chad	ZONE 6	00-04	158.13	178.92	139.35	HT-Direct
Chad	ZONE 6	00-04	159.16	145.27	173.91	RW2
Chad	ZONE 6	05-09	165.67	186.22	146.98	HT-Direct
Chad	ZONE 6	05-09	163.04	148.40	178.91	RW2
Chad	ZONE 6	10-14	129.62	151.71	110.33	HT-Direct
Chad	ZONE 6	10-14	158.47	136.73	183.30	RW2
Chad	ZONE 6	15-19	150.81	70.01	295.52	RW2
Chad	ZONE 7	80-84	210.70	276.54	157.13	HT-Direct
Chad	ZONE 7	80-84	238.72	187.39	295.14	RW2
Chad	ZONE 7	85-89	202.04	249.50	161.66	HT-Direct
Chad	ZONE 7	85-89	250.18	214.16	289.37	RW2
Chad	ZONE 7	90-94	221.09	255.93	189.78	HT-Direct
Chad	ZONE 7	90-94	257.58	232.41	284.47	RW2
Chad	ZONE 7	95-99	268.87	294.52	244.67	HT-Direct
Chad	ZONE 7	95-99	255.67	237.31	275.40	RW2
Chad	ZONE 7	00-04	236.21	265.94	208.86	HT-Direct
Chad	ZONE 7	00-04	248.96	229.45	270.85	RW2
Chad	ZONE 7	05-09	247.06	291.14	207.71	HT-Direct
Chad	ZONE 7	05-09	238.38	216.39	261.89	RW2
Chad	ZONE 7	10-14	175.32	195.23	157.05	HT-Direct
Chad	ZONE 7	10-14	214.12	189.32	240.23	RW2

Continued on next page

Country	Region	Year	Median	Lower	Upper	Method
Chad	ZONE 7	15-19	187.90	90.63	345.34	RW2
Chad	ZONE 8	80-84	224.78	300.63	163.59	HT-Direct
Chad	ZONE 8	80-84	261.49	207.35	321.46	RW2
Chad	ZONE 8	85-89	226.53	271.48	187.10	HT-Direct
Chad	ZONE 8	85-89	253.54	217.09	293.07	RW2
Chad	ZONE 8	90-94	216.01	265.49	173.56	HT-Direct
Chad	ZONE 8	90-94	241.89	214.23	271.82	RW2
Chad	ZONE 8	95-99	220.46	252.68	191.30	HT-Direct
Chad	ZONE 8	95-99	223.75	203.33	245.79	RW2
Chad	ZONE 8	00-04	202.47	235.33	173.15	HT-Direct
Chad	ZONE 8	00-04	205.60	187.58	225.59	RW2
Chad	ZONE 8	05-09	186.19	209.14	165.24	HT-Direct
Chad	ZONE 8	05-09	187.45	170.95	205.41	RW2
Chad	ZONE 8	10-14	130.85	150.99	113.04	HT-Direct
Chad	ZONE 8	10-14	161.61	140.20	185.09	RW2
Chad	ZONE 8	15-19	135.92	62.65	269.76	RW2
Comoros	ALL	80-84	154.54	177.06	134.41	HT-Direct
Comoros	ALL	80-84	154.72	150.37	159.08	IHME
Comoros	ALL	80-84	164.34	141.77	189.72	RW2
Comoros	ALL	80-84	164.08	156.29	172.55	UN
Comoros	ALL	85-89	117.34	135.28	101.50	HT-Direct
Comoros	ALL	85-89	131.45	127.70	135.50	IHME
Comoros	ALL	85-89	136.56	116.60	159.15	RW2
Comoros	ALL	85-89	137.66	130.88	145.53	UN
Comoros	ALL	90-94	97.44	110.20	86.02	HT-Direct
Comoros	ALL	90-94	113.33	109.47	116.99	IHME
Comoros	ALL	90-94	117.71	101.57	136.09	RW2
Comoros	ALL	90-94	116.85	110.74	123.03	UN
Comoros	ALL	95-99	70.08	86.12	56.84	HT-Direct
Comoros	ALL	95-99	95.92	90.91	101.18	IHME
Comoros	ALL	95-99	102.93	73.27	142.05	RW2
Comoros	ALL	95-99	101.90	94.26	109.80	UN
Comoros	ALL	00-04	51.26	64.86	40.39	HT-Direct
Comoros	ALL	00-04	77.83	71.47	84.91	IHME
Comoros	ALL	00-04	94.29	42.54	195.48	RW2
Comoros	ALL	00-04	97.73	87.11	108.71	UN
Comoros	ALL	05-09	47.04	59.62	37.01	HT-Direct
Comoros	ALL	05-09	62.98	55.76	71.17	IHME
Comoros	ALL	05-09	89.76	35.41	208.83	RW2
Comoros	ALL	05-09	95.07	79.54	113.51	UN
Comoros	ALL	10-14	57.99	78.48	42.60	HT-Direct
Comoros	ALL	10-14	50.62	43.93	57.81	IHME
Comoros	ALL	10-14	87.45	44.68	163.48	RW2
Comoros	ALL	10-14	85.93	66.84	110.09	UN
Comoros	MOHELI	80-84	164.52	248.69	104.87	HT-Direct
Comoros	MOHELI	80-84	168.02	110.21	247.31	RW2
Comoros	MOHELI	85-89	122.96	207.33	69.89	HT-Direct
Comoros	MOHELI	85-89	129.48	92.23	179.12	RW2
Comoros	MOHELI	90-94	72.72	113.18	45.97	HT-Direct
Comoros	MOHELI	90-94	107.54	75.37	150.51	RW2
Comoros	MOHELI	95-99	66.63	98.47	44.58	HT-Direct
Comoros	MOHELI	95-99	92.97	56.36	149.07	RW2
Comoros	MOHELI	00-04	41.40	62.21	27.35	HT-Direct
Comoros	MOHELI	00-04	84.28	35.11	189.88	RW2
Comoros	MOHELI	05-09	27.35	50.73	14.58	HT-Direct
Comoros	MOHELI	05-09	79.90	26.20	219.10	RW2
Comoros	MOHELI	10-14	70.35	146.95	32.18	HT-Direct
Comoros	MOHELI	10-14	77.37	22.68	234.68	RW2
Comoros	MOHELI	15-19	75.07	8.29	451.39	RW2
Comoros	NDZOUANI	80-84	174.50	221.34	135.85	HT-Direct
Comoros	NDZOUANI	80-84	185.78	147.35	232.28	RW2
Comoros	NDZOUANI	85-89	122.95	149.11	100.83	HT-Direct
Comoros	NDZOUANI	85-89	144.22	120.36	171.61	RW2

Continued on next page

Country	Region	Year	Median	Lower	Upper	Method
Comoros	NDZOUANI	90-94	101.84	122.39	84.41	HT-Direct
Comoros	NDZOUANI	90-94	119.33	98.13	144.33	RW2
Comoros	NDZOUANI	95-99	58.10	81.23	41.27	HT-Direct
Comoros	NDZOUANI	95-99	101.40	69.26	146.00	RW2
Comoros	NDZOUANI	00-04	52.66	76.39	36.01	HT-Direct
Comoros	NDZOUANI	00-04	89.86	41.92	182.45	RW2
Comoros	NDZOUANI	05-09	42.27	63.31	28.01	HT-Direct
Comoros	NDZOUANI	05-09	82.89	33.10	192.37	RW2
Comoros	NDZOUANI	10-14	55.60	88.31	34.55	HT-Direct
Comoros	NDZOUANI	10-14	78.26	32.99	174.79	RW2
Comoros	NDZOUANI	15-19	73.86	11.22	365.63	RW2
Comoros	NGAZIDJA	80-84	137.42	159.67	117.84	HT-Direct
Comoros	NGAZIDJA	80-84	146.63	125.37	170.73	RW2
Comoros	NGAZIDJA	85-89	105.57	129.30	85.77	HT-Direct
Comoros	NGAZIDJA	85-89	124.42	103.95	148.14	RW2
Comoros	NGAZIDJA	90-94	94.66	112.25	79.58	HT-Direct
Comoros	NGAZIDJA	90-94	113.53	94.68	135.93	RW2
Comoros	NGAZIDJA	95-99	80.42	105.43	60.93	HT-Direct
Comoros	NGAZIDJA	95-99	106.48	74.67	149.49	RW2
Comoros	NGAZIDJA	00-04	51.51	69.91	37.75	HT-Direct
Comoros	NGAZIDJA	00-04	102.91	49.45	201.94	RW2
Comoros	NGAZIDJA	05-09	57.07	76.20	42.52	HT-Direct
Comoros	NGAZIDJA	05-09	102.84	42.91	225.84	RW2
Comoros	NGAZIDJA	10-14	59.22	90.94	38.10	HT-Direct
Comoros	NGAZIDJA	10-14	104.23	46.46	215.53	RW2
Comoros	NGAZIDJA	15-19	105.93	16.93	452.55	RW2
Congo	ALL	80-84	102.46	124.52	83.94	HT-Direct
Congo	ALL	80-84	112.06	107.16	117.11	IHME
Congo	ALL	80-84	106.72	87.03	130.28	RW2
Congo	ALL	80-84	107.21	97.73	116.73	UN
Congo	ALL	85-89	93.29	106.80	81.33	HT-Direct
Congo	ALL	85-89	96.31	93.24	99.30	IHME
Congo	ALL	85-89	95.22	83.68	108.10	RW2
Congo	ALL	85-89	94.68	88.56	101.05	UN
Congo	ALL	90-94	95.32	106.80	84.96	HT-Direct
Congo	ALL	90-94	95.27	92.60	97.85	IHME
Congo	ALL	90-94	97.68	87.66	108.59	RW2
Congo	ALL	90-94	98.32	93.04	103.57	UN
Congo	ALL	95-99	126.25	138.06	115.32	HT-Direct
Congo	ALL	95-99	108.34	104.24	112.75	IHME
Congo	ALL	95-99	116.21	107.24	125.76	RW2
Congo	ALL	95-99	115.58	110.27	120.96	UN
Congo	ALL	00-04	112.73	121.45	104.56	HT-Direct
Congo	ALL	00-04	103.83	100.74	106.98	IHME
Congo	ALL	00-04	113.89	105.82	122.58	RW2
Congo	ALL	00-04	113.93	108.87	118.74	UN
Congo	ALL	05-09	70.20	78.63	62.61	HT-Direct
Congo	ALL	05-09	79.42	76.79	81.99	IHME
Congo	ALL	05-09	79.17	70.08	89.16	RW2
Congo	ALL	05-09	79.66	75.41	84.77	UN
Congo	ALL	10-14	66.38	81.82	53.68	HT-Direct
Congo	ALL	10-14	62.66	58.56	67.24	IHME
Congo	ALL	10-14	54.21	45.46	64.47	RW2
Congo	ALL	10-14	53.17	47.00	60.03	UN
Congo	BRAZZAVILLE	80-84	80.37	121.51	52.32	HT-Direct
Congo	BRAZZAVILLE	80-84	75.19	54.51	102.75	RW2
Congo	BRAZZAVILLE	85-89	64.78	86.74	48.08	HT-Direct
Congo	BRAZZAVILLE	85-89	68.99	56.20	84.46	RW2
Congo	BRAZZAVILLE	90-94	61.65	79.69	47.48	HT-Direct
Congo	BRAZZAVILLE	90-94	74.61	63.83	86.99	RW2
Congo	BRAZZAVILLE	95-99	128.71	151.23	109.11	HT-Direct
Congo	BRAZZAVILLE	95-99	103.59	92.41	116.20	RW2
Congo	BRAZZAVILLE	00-04	93.21	110.92	78.08	HT-Direct

Continued on next page

Country	Region	Year	Median	Lower	Upper	Method
Congo	BRAZZAVILLE	00-04	105.74	93.25	119.55	RW2
Congo	BRAZZAVILLE	05-09	68.38	90.44	51.40	HT-Direct
Congo	BRAZZAVILLE	05-09	83.21	68.79	99.98	RW2
Congo	BRAZZAVILLE	10-14	80.14	127.51	49.37	HT-Direct
Congo	BRAZZAVILLE	10-14	55.01	41.03	74.01	RW2
Congo	BRAZZAVILLE	15-19	34.40	11.71	97.51	RW2
Congo	NORD	80-84	119.86	164.48	86.10	HT-Direct
Congo	NORD	80-84	123.68	95.56	159.28	RW2
Congo	NORD	85-89	108.94	133.69	88.30	HT-Direct
Congo	NORD	85-89	104.64	89.35	122.42	RW2
Congo	NORD	90-94	94.11	111.34	79.30	HT-Direct
Congo	NORD	90-94	102.75	90.80	115.70	RW2
Congo	NORD	95-99	142.46	160.16	126.42	HT-Direct
Congo	NORD	95-99	130.48	119.58	142.10	RW2
Congo	NORD	00-04	122.69	133.67	112.49	HT-Direct
Congo	NORD	00-04	126.53	117.51	136.33	RW2
Congo	NORD	05-09	92.19	104.62	81.09	HT-Direct
Congo	NORD	05-09	93.22	83.15	104.51	RW2
Congo	NORD	10-14	60.70	77.28	47.50	HT-Direct
Congo	NORD	10-14	54.59	45.70	64.95	RW2
Congo	NORD	15-19	29.42	10.58	77.40	RW2
Congo	POINTE NOIRE	80-84	93.52	142.88	60.02	HT-Direct
Congo	POINTE NOIRE	80-84	110.51	80.47	151.23	RW2
Congo	POINTE NOIRE	85-89	105.98	142.81	77.79	HT-Direct
Congo	POINTE NOIRE	85-89	90.97	73.50	112.29	RW2
Congo	POINTE NOIRE	90-94	79.97	103.85	61.21	HT-Direct
Congo	POINTE NOIRE	90-94	85.81	72.95	100.87	RW2
Congo	POINTE NOIRE	95-99	115.66	140.18	94.96	HT-Direct
Congo	POINTE NOIRE	95-99	103.98	91.35	117.86	RW2
Congo	POINTE NOIRE	00-04	88.65	108.93	71.84	HT-Direct
Congo	POINTE NOIRE	00-04	96.18	83.30	110.19	RW2
Congo	POINTE NOIRE	05-09	52.13	69.27	39.05	HT-Direct
Congo	POINTE NOIRE	05-09	69.52	57.36	83.91	RW2
Congo	POINTE NOIRE	10-14	68.53	105.75	43.78	HT-Direct
Congo	POINTE NOIRE	10-14	42.53	31.87	58.01	RW2
Congo	POINTE NOIRE	15-19	24.68	8.39	72.50	RW2
Congo	SUD	80-84	131.98	175.90	97.72	HT-Direct
Congo	SUD	80-84	139.28	109.73	174.23	RW2
Congo	SUD	85-89	100.64	126.63	79.50	HT-Direct
Congo	SUD	85-89	115.62	98.43	135.04	RW2
Congo	SUD	90-94	120.06	139.19	103.25	HT-Direct
Congo	SUD	90-94	110.69	98.74	123.93	RW2
Congo	SUD	95-99	133.53	152.73	116.40	HT-Direct
Congo	SUD	95-99	134.12	122.67	146.66	RW2
Congo	SUD	00-04	133.71	150.67	118.39	HT-Direct
Congo	SUD	00-04	122.96	112.37	134.60	RW2
Congo	SUD	05-09	72.61	85.61	61.46	HT-Direct
Congo	SUD	05-09	85.04	74.82	96.63	RW2
Congo	SUD	10-14	55.61	71.04	43.37	HT-Direct
Congo	SUD	10-14	47.91	40.19	56.91	RW2
Congo	SUD	15-19	25.11	9.06	66.07	RW2
Côte d'Ivoire	ALL	80-84	131.42	171.48	99.59	HT-Direct
Côte d'Ivoire	ALL	80-84	155.44	153.04	157.92	IHME
Côte d'Ivoire	ALL	80-84	158.39	114.30	215.37	RW2
Côte d'Ivoire	ALL	80-84	160.36	154.83	165.77	UN
Côte d'Ivoire	ALL	85-89	142.74	171.14	118.38	HT-Direct
Côte d'Ivoire	ALL	85-89	149.63	147.56	151.83	IHME
Côte d'Ivoire	ALL	85-89	155.10	130.26	183.60	RW2
Côte d'Ivoire	ALL	85-89	152.92	148.31	157.65	UN
Côte d'Ivoire	ALL	90-94	138.27	155.75	122.46	HT-Direct
Côte d'Ivoire	ALL	90-94	148.94	146.74	151.09	IHME
Côte d'Ivoire	ALL	90-94	152.43	135.08	171.50	RW2
Côte d'Ivoire	ALL	90-94	153.01	148.26	157.66	UN

Continued on next page

Country	Region	Year	Median	Lower	Upper	Method
Côte d'Ivoire	ALL	95-99	142.64	157.39	129.07	HT-Direct
Côte d'Ivoire	ALL	95-99	145.95	143.33	148.31	IHME
Côte d'Ivoire	ALL	95-99	150.77	136.70	165.94	RW2
Côte d'Ivoire	ALL	95-99	150.89	145.62	156.27	UN
Côte d'Ivoire	ALL	00-04	137.05	150.12	124.95	HT-Direct
Côte d'Ivoire	ALL	00-04	135.89	133.26	138.59	IHME
Côte d'Ivoire	ALL	00-04	139.70	127.98	152.38	RW2
Côte d'Ivoire	ALL	00-04	139.57	133.91	145.15	UN
Côte d'Ivoire	ALL	05-09	118.20	128.24	108.85	HT-Direct
Côte d'Ivoire	ALL	05-09	121.06	117.85	124.21	IHME
Côte d'Ivoire	ALL	05-09	121.06	111.56	131.24	RW2
Côte d'Ivoire	ALL	05-09	121.06	115.65	126.46	UN
Côte d'Ivoire	ALL	10-14	97.02	115.98	80.88	HT-Direct
Côte d'Ivoire	ALL	10-14	104.54	100.31	108.75	IHME
Côte d'Ivoire	ALL	10-14	102.70	85.19	123.18	RW2
Côte d'Ivoire	ALL	10-14	102.89	94.95	110.57	UN
Côte d'Ivoire	CENTRE	80-84	160.59	265.44	91.97	HT-Direct
Côte d'Ivoire	CENTRE	80-84	168.41	106.67	257.22	RW2
Côte d'Ivoire	CENTRE	85-89	119.37	202.67	67.42	HT-Direct
Côte d'Ivoire	CENTRE	85-89	140.18	100.26	193.04	RW2
Côte d'Ivoire	CENTRE	90-94	137.82	210.27	87.56	HT-Direct
Côte d'Ivoire	CENTRE	90-94	139.94	109.68	176.96	RW2
Côte d'Ivoire	CENTRE	95-99	125.98	163.04	96.38	HT-Direct
Côte d'Ivoire	CENTRE	95-99	148.69	125.26	175.23	RW2
Côte d'Ivoire	CENTRE	00-04	158.78	199.53	125.06	HT-Direct
Côte d'Ivoire	CENTRE	00-04	153.45	131.88	177.89	RW2
Côte d'Ivoire	CENTRE	05-09	152.11	193.31	118.41	HT-Direct
Côte d'Ivoire	CENTRE	05-09	141.61	115.94	171.82	RW2
Côte d'Ivoire	CENTRE	10-14	61.13	139.39	25.50	HT-Direct
Côte d'Ivoire	CENTRE	10-14	131.33	92.27	183.38	RW2
Côte d'Ivoire	CENTRE	15-19	123.63	45.03	295.00	RW2
Côte d'Ivoire	CENTRE-EST	80-84	162.46	280.28	88.10	HT-Direct
Côte d'Ivoire	CENTRE-EST	80-84	152.28	92.10	243.73	RW2
Côte d'Ivoire	CENTRE-EST	85-89	106.01	220.16	47.45	HT-Direct
Côte d'Ivoire	CENTRE-EST	85-89	125.17	87.82	175.79	RW2
Côte d'Ivoire	CENTRE-EST	90-94	120.90	162.24	88.97	HT-Direct
Côte d'Ivoire	CENTRE-EST	90-94	123.85	97.65	155.72	RW2
Côte d'Ivoire	CENTRE-EST	95-99	101.71	138.75	73.71	HT-Direct
Côte d'Ivoire	CENTRE-EST	95-99	132.01	109.50	157.48	RW2
Côte d'Ivoire	CENTRE-EST	00-04	130.38	169.98	98.91	HT-Direct
Côte d'Ivoire	CENTRE-EST	00-04	138.68	116.19	164.19	RW2
Côte d'Ivoire	CENTRE-EST	05-09	160.01	214.63	117.21	HT-Direct
Côte d'Ivoire	CENTRE-EST	05-09	131.66	103.54	166.37	RW2
Côte d'Ivoire	CENTRE-EST	10-14	67.70	155.70	27.80	HT-Direct
Côte d'Ivoire	CENTRE-EST	10-14	125.42	84.30	184.69	RW2
Côte d'Ivoire	CENTRE-EST	15-19	120.97	42.98	301.44	RW2
Côte d'Ivoire	CENTRE-NORD	80-84	127.06	189.30	83.19	HT-Direct
Côte d'Ivoire	CENTRE-NORD	80-84	162.55	109.68	233.27	RW2
Côte d'Ivoire	CENTRE-NORD	85-89	116.88	176.47	75.57	HT-Direct
Côte d'Ivoire	CENTRE-NORD	85-89	122.43	89.54	163.77	RW2
Côte d'Ivoire	CENTRE-NORD	90-94	82.78	137.49	48.62	HT-Direct
Côte d'Ivoire	CENTRE-NORD	90-94	110.27	85.08	141.54	RW2
Côte d'Ivoire	CENTRE-NORD	95-99	132.57	218.07	77.27	HT-Direct
Côte d'Ivoire	CENTRE-NORD	95-99	105.61	84.23	131.41	RW2
Côte d'Ivoire	CENTRE-NORD	00-04	71.74	109.00	46.55	HT-Direct
Côte d'Ivoire	CENTRE-NORD	00-04	97.29	77.87	120.85	RW2
Côte d'Ivoire	CENTRE-NORD	05-09	81.70	117.29	56.22	HT-Direct
Côte d'Ivoire	CENTRE-NORD	05-09	79.81	60.68	104.11	RW2
Côte d'Ivoire	CENTRE-NORD	10-14	57.26	112.87	28.17	HT-Direct
Côte d'Ivoire	CENTRE-NORD	10-14	66.34	44.38	98.12	RW2
Côte d'Ivoire	CENTRE-NORD	15-19	55.93	19.71	151.53	RW2
Côte d'Ivoire	CENTRE-OUEST	80-84	53.46	189.78	13.44	HT-Direct
Côte d'Ivoire	CENTRE-OUEST	80-84	174.79	99.11	288.65	RW2

Continued on next page

Country	Region	Year	Median	Lower	Upper	Method
Côte d'Ivoire	CENTRE-OUEST	85-89	95.55	203.46	41.86	HT-Direct
Côte d'Ivoire	CENTRE-OUEST	85-89	135.56	91.25	197.01	RW2
Côte d'Ivoire	CENTRE-OUEST	90-94	143.56	197.48	102.48	HT-Direct
Côte d'Ivoire	CENTRE-OUEST	90-94	124.87	95.59	161.67	RW2
Côte d'Ivoire	CENTRE-OUEST	95-99	92.43	129.04	65.42	HT-Direct
Côte d'Ivoire	CENTRE-OUEST	95-99	120.62	99.04	146.20	RW2
Côte d'Ivoire	CENTRE-OUEST	00-04	119.58	168.48	83.46	HT-Direct
Côte d'Ivoire	CENTRE-OUEST	00-04	111.23	93.04	132.57	RW2
Côte d'Ivoire	CENTRE-OUEST	05-09	84.60	110.52	64.33	HT-Direct
Côte d'Ivoire	CENTRE-OUEST	05-09	90.84	72.46	113.01	RW2
Côte d'Ivoire	CENTRE-OUEST	10-14	70.92	139.11	34.81	HT-Direct
Côte d'Ivoire	CENTRE-OUEST	10-14	75.03	51.53	107.52	RW2
Côte d'Ivoire	CENTRE-OUEST	15-19	63.23	22.53	165.02	RW2
Côte d'Ivoire	NORD	80-84	234.48	394.16	126.03	HT-Direct
Côte d'Ivoire	NORD	80-84	247.92	158.70	364.52	RW2
Côte d'Ivoire	NORD	85-89	185.93	296.60	110.09	HT-Direct
Côte d'Ivoire	NORD	85-89	213.44	156.04	283.80	RW2
Côte d'Ivoire	NORD	90-94	192.67	277.88	128.93	HT-Direct
Côte d'Ivoire	NORD	90-94	217.05	174.60	266.40	RW2
Côte d'Ivoire	NORD	95-99	223.74	273.84	180.52	HT-Direct
Côte d'Ivoire	NORD	95-99	232.72	200.91	267.63	RW2
Côte d'Ivoire	NORD	00-04	244.98	289.95	204.97	HT-Direct
Côte d'Ivoire	NORD	00-04	240.39	214.03	268.99	RW2
Côte d'Ivoire	NORD	05-09	211.03	246.95	179.10	HT-Direct
Côte d'Ivoire	NORD	05-09	223.92	197.19	253.48	RW2
Côte d'Ivoire	NORD	10-14	211.81	274.95	159.96	HT-Direct
Côte d'Ivoire	NORD	10-14	211.57	170.01	260.34	RW2
Côte d'Ivoire	NORD	15-19	202.53	85.46	413.40	RW2
Côte d'Ivoire	NORD-EST	80-84	274.70	461.90	143.19	HT-Direct
Côte d'Ivoire	NORD-EST	80-84	216.17	129.74	340.78	RW2
Côte d'Ivoire	NORD-EST	85-89	117.48	224.25	57.76	HT-Direct
Côte d'Ivoire	NORD-EST	85-89	167.35	115.35	236.99	RW2
Côte d'Ivoire	NORD-EST	90-94	182.93	269.00	119.89	HT-Direct
Côte d'Ivoire	NORD-EST	90-94	153.76	118.15	197.33	RW2
Côte d'Ivoire	NORD-EST	95-99	118.32	157.40	87.93	HT-Direct
Côte d'Ivoire	NORD-EST	95-99	151.15	124.25	182.14	RW2
Côte d'Ivoire	NORD-EST	00-04	145.72	199.23	104.71	HT-Direct
Côte d'Ivoire	NORD-EST	00-04	145.94	120.96	175.15	RW2
Côte d'Ivoire	NORD-EST	05-09	129.59	191.22	85.72	HT-Direct
Côte d'Ivoire	NORD-EST	05-09	127.42	99.84	160.99	RW2
Côte d'Ivoire	NORD-EST	10-14	116.29	179.33	73.42	HT-Direct
Côte d'Ivoire	NORD-EST	10-14	112.60	78.96	158.41	RW2
Côte d'Ivoire	NORD-EST	15-19	100.67	37.67	246.80	RW2
Côte d'Ivoire	NORD-OUEST	80-84	224.51	321.45	150.33	HT-Direct
Côte d'Ivoire	NORD-OUEST	80-84	226.71	158.30	311.51	RW2
Côte d'Ivoire	NORD-OUEST	85-89	168.60	235.20	117.95	HT-Direct
Côte d'Ivoire	NORD-OUEST	85-89	195.99	153.51	246.58	RW2
Côte d'Ivoire	NORD-OUEST	90-94	144.36	194.88	105.22	HT-Direct
Côte d'Ivoire	NORD-OUEST	90-94	200.70	167.86	238.36	RW2
Côte d'Ivoire	NORD-OUEST	95-99	226.21	272.04	186.12	HT-Direct
Côte d'Ivoire	NORD-OUEST	95-99	215.87	190.17	244.69	RW2
Côte d'Ivoire	NORD-OUEST	00-04	223.28	254.77	194.67	HT-Direct
Côte d'Ivoire	NORD-OUEST	00-04	220.26	198.75	243.46	RW2
Côte d'Ivoire	NORD-OUEST	05-09	184.22	219.80	153.28	HT-Direct
Côte d'Ivoire	NORD-OUEST	05-09	200.06	174.52	228.26	RW2
Côte d'Ivoire	NORD-OUEST	10-14	183.99	260.85	125.91	HT-Direct
Côte d'Ivoire	NORD-OUEST	10-14	184.08	142.92	232.85	RW2
Côte d'Ivoire	NORD-OUEST	15-19	171.47	69.78	366.07	RW2
Côte d'Ivoire	OUEST	80-84	135.65	295.00	55.59	HT-Direct
Côte d'Ivoire	OUEST	80-84	319.33	201.30	448.68	RW2
Côte d'Ivoire	OUEST	85-89	226.42	334.19	145.79	HT-Direct
Côte d'Ivoire	OUEST	85-89	253.74	191.66	323.72	RW2
Côte d'Ivoire	OUEST	90-94	207.04	257.76	164.09	HT-Direct

Continued on next page

Country	Region	Year	Median	Lower	Upper	Method
Côte d'Ivoire	OUEST	90-94	231.59	195.31	272.27	RW2
Côte d'Ivoire	OUEST	95-99	220.44	256.47	188.19	HT-Direct
Côte d'Ivoire	OUEST	95-99	217.31	192.95	244.81	RW2
Côte d'Ivoire	OUEST	00-04	193.03	224.33	165.17	HT-Direct
Côte d'Ivoire	OUEST	00-04	190.30	170.03	212.67	RW2
Côte d'Ivoire	OUEST	05-09	123.79	153.40	99.22	HT-Direct
Côte d'Ivoire	OUEST	05-09	146.19	123.99	171.27	RW2
Côte d'Ivoire	OUEST	10-14	132.87	208.99	81.61	HT-Direct
Côte d'Ivoire	OUEST	10-14	113.42	83.08	151.76	RW2
Côte d'Ivoire	OUEST	15-19	88.99	32.88	219.30	RW2
Côte d'Ivoire	SUD SANS ABIDJAN	80-84	93.77	285.60	26.08	HT-Direct
Côte d'Ivoire	SUD SANS ABIDJAN	80-84	192.62	112.24	309.48	RW2
Côte d'Ivoire	SUD SANS ABIDJAN	85-89	138.81	247.04	73.37	HT-Direct
Côte d'Ivoire	SUD SANS ABIDJAN	85-89	152.46	105.12	215.84	RW2
Côte d'Ivoire	SUD SANS ABIDJAN	90-94	126.90	181.23	87.13	HT-Direct
Côte d'Ivoire	SUD SANS ABIDJAN	90-94	142.81	110.56	182.44	RW2
Côte d'Ivoire	SUD SANS ABIDJAN	95-99	137.66	181.86	102.86	HT-Direct
Côte d'Ivoire	SUD SANS ABIDJAN	95-99	141.15	117.83	168.01	RW2
Côte d'Ivoire	SUD SANS ABIDJAN	00-04	139.43	180.57	106.45	HT-Direct
Côte d'Ivoire	SUD SANS ABIDJAN	00-04	134.73	115.82	156.35	RW2
Côte d'Ivoire	SUD SANS ABIDJAN	05-09	113.15	138.47	91.96	HT-Direct
Côte d'Ivoire	SUD SANS ABIDJAN	05-09	114.67	96.08	136.44	RW2
Côte d'Ivoire	SUD SANS ABIDJAN	10-14	70.40	130.08	36.94	HT-Direct
Côte d'Ivoire	SUD SANS ABIDJAN	10-14	98.13	70.65	134.37	RW2
Côte d'Ivoire	SUD SANS ABIDJAN	15-19	84.60	30.86	212.76	RW2
Côte d'Ivoire	SUD-OUEST	80-84	102.08	326.44	25.97	HT-Direct
Côte d'Ivoire	SUD-OUEST	80-84	196.26	107.84	329.72	RW2
Côte d'Ivoire	SUD-OUEST	85-89	152.51	255.09	86.40	HT-Direct
Côte d'Ivoire	SUD-OUEST	85-89	145.75	95.60	216.84	RW2
Côte d'Ivoire	SUD-OUEST	90-94	122.21	189.03	76.77	HT-Direct
Côte d'Ivoire	SUD-OUEST	90-94	127.13	92.40	172.24	RW2
Côte d'Ivoire	SUD-OUEST	95-99	123.36	234.63	60.68	HT-Direct
Côte d'Ivoire	SUD-OUEST	95-99	116.32	89.99	148.95	RW2
Côte d'Ivoire	SUD-OUEST	00-04	65.35	107.39	39.05	HT-Direct
Côte d'Ivoire	SUD-OUEST	00-04	101.58	81.65	125.22	RW2
Côte d'Ivoire	SUD-OUEST	05-09	78.68	101.25	60.80	HT-Direct
Côte d'Ivoire	SUD-OUEST	05-09	79.32	63.23	99.19	RW2
Côte d'Ivoire	SUD-OUEST	10-14	78.02	156.78	37.09	HT-Direct
Côte d'Ivoire	SUD-OUEST	10-14	63.23	42.98	92.49	RW2
Côte d'Ivoire	SUD-OUEST	15-19	51.19	17.60	141.52	RW2
Côte d'Ivoire	VILLE D'ABIDJAN	80-84	125.46	286.42	48.77	HT-Direct
Côte d'Ivoire	VILLE D'ABIDJAN	80-84	151.11	82.69	268.15	RW2
Côte d'Ivoire	VILLE D'ABIDJAN	85-89	133.37	225.15	75.36	HT-Direct
Côte d'Ivoire	VILLE D'ABIDJAN	85-89	116.44	78.37	170.86	RW2
Côte d'Ivoire	VILLE D'ABIDJAN	90-94	88.03	135.03	56.33	HT-Direct
Côte d'Ivoire	VILLE D'ABIDJAN	90-94	107.24	81.50	140.09	RW2
Côte d'Ivoire	VILLE D'ABIDJAN	95-99	103.45	138.84	76.28	HT-Direct
Côte d'Ivoire	VILLE D'ABIDJAN	95-99	106.28	85.81	130.26	RW2
Côte d'Ivoire	VILLE D'ABIDJAN	00-04	83.74	117.33	59.13	HT-Direct
Côte d'Ivoire	VILLE D'ABIDJAN	00-04	104.21	85.62	125.35	RW2
Côte d'Ivoire	VILLE D'ABIDJAN	05-09	99.29	128.09	76.39	HT-Direct
Côte d'Ivoire	VILLE D'ABIDJAN	05-09	93.50	75.83	115.18	RW2
Côte d'Ivoire	VILLE D'ABIDJAN	10-14	80.05	132.59	47.20	HT-Direct
Côte d'Ivoire	VILLE D'ABIDJAN	10-14	85.32	59.22	122.63	RW2
Côte d'Ivoire	VILLE D'ABIDJAN	15-19	78.87	27.29	211.63	RW2
DRC	ALL	80-84	168.47	195.52	144.49	HT-Direct
DRC	ALL	80-84	177.53	170.43	184.58	IHME
DRC	ALL	80-84	206.56	169.75	249.04	RW2
DRC	ALL	80-84	205.54	194.81	217.49	UN
DRC	ALL	85-89	156.77	175.63	139.59	HT-Direct
DRC	ALL	85-89	161.02	156.18	166.64	IHME
DRC	ALL	85-89	190.70	167.27	216.23	RW2
DRC	ALL	85-89	192.63	184.31	202.27	UN

Continued on next page

Country	Region	Year	Median	Lower	Upper	Method
DRC	ALL	90-94	181.11	199.80	163.82	HT-Direct
DRC	ALL	90-94	165.89	160.54	171.62	IHME
DRC	ALL	90-94	183.69	167.32	201.32	RW2
DRC	ALL	90-94	182.13	174.46	190.42	UN
DRC	ALL	95-99	173.76	188.56	159.88	HT-Direct
DRC	ALL	95-99	162.41	157.27	167.95	IHME
DRC	ALL	95-99	171.81	159.01	185.31	RW2
DRC	ALL	95-99	171.66	164.49	179.34	UN
DRC	ALL	00-04	144.53	153.83	135.69	HT-Direct
DRC	ALL	00-04	142.43	137.67	147.57	IHME
DRC	ALL	00-04	151.93	142.57	161.84	RW2
DRC	ALL	00-04	152.22	146.01	158.37	UN
DRC	ALL	05-09	122.01	130.86	113.68	HT-Direct
DRC	ALL	05-09	120.74	116.11	125.82	IHME
DRC	ALL	05-09	129.30	120.20	138.96	RW2
DRC	ALL	05-09	129.24	122.26	136.71	UN
DRC	ALL	10-14	103.96	113.81	94.87	HT-Direct
DRC	ALL	10-14	99.73	94.09	106.03	IHME
DRC	ALL	10-14	107.92	98.21	118.39	RW2
DRC	ALL	10-14	107.92	97.62	119.22	UN
DRC	BANDUNDU	80-84	151.31	208.25	107.82	HT-Direct
DRC	BANDUNDU	80-84	238.22	178.63	309.96	RW2
DRC	BANDUNDU	85-89	153.26	238.72	94.59	HT-Direct
DRC	BANDUNDU	85-89	207.61	166.61	256.08	RW2
DRC	BANDUNDU	90-94	204.52	253.73	162.77	HT-Direct
DRC	BANDUNDU	90-94	177.66	151.75	207.07	RW2
DRC	BANDUNDU	95-99	162.80	210.22	124.39	HT-Direct
DRC	BANDUNDU	95-99	161.33	141.94	182.78	RW2
DRC	BANDUNDU	00-04	117.68	141.98	97.07	HT-Direct
DRC	BANDUNDU	00-04	133.39	118.42	149.94	RW2
DRC	BANDUNDU	05-09	100.27	122.57	81.65	HT-Direct
DRC	BANDUNDU	05-09	101.42	87.91	116.68	RW2
DRC	BANDUNDU	10-14	72.77	95.81	54.93	HT-Direct
DRC	BANDUNDU	10-14	79.50	64.53	97.65	RW2
DRC	BANDUNDU	15-19	63.28	26.72	141.66	RW2
DRC	BAS-CONGO	80-84	183.53	273.26	118.47	HT-Direct
DRC	BAS-CONGO	80-84	258.13	184.46	345.98	RW2
DRC	BAS-CONGO	85-89	186.01	251.34	134.61	HT-Direct
DRC	BAS-CONGO	85-89	235.01	187.22	290.60	RW2
DRC	BAS-CONGO	90-94	226.00	295.06	169.23	HT-Direct
DRC	BAS-CONGO	90-94	210.71	179.20	246.14	RW2
DRC	BAS-CONGO	95-99	210.85	253.40	173.78	HT-Direct
DRC	BAS-CONGO	95-99	200.69	178.06	225.47	RW2
DRC	BAS-CONGO	00-04	164.02	190.52	140.56	HT-Direct
DRC	BAS-CONGO	00-04	175.05	156.16	195.41	RW2
DRC	BAS-CONGO	05-09	131.12	168.66	100.92	HT-Direct
DRC	BAS-CONGO	05-09	141.51	120.41	165.66	RW2
DRC	BAS-CONGO	10-14	117.10	163.64	82.50	HT-Direct
DRC	BAS-CONGO	10-14	118.36	92.14	151.30	RW2
DRC	BAS-CONGO	15-19	100.38	42.18	222.50	RW2
DRC	EQUATEUR	80-84	141.17	208.45	93.05	HT-Direct
DRC	EQUATEUR	80-84	190.05	139.32	253.49	RW2
DRC	EQUATEUR	85-89	160.67	206.67	123.31	HT-Direct
DRC	EQUATEUR	85-89	184.06	147.56	226.12	RW2
DRC	EQUATEUR	90-94	217.44	293.70	156.59	HT-Direct
DRC	EQUATEUR	90-94	175.86	148.75	206.62	RW2
DRC	EQUATEUR	95-99	166.05	217.22	125.01	HT-Direct
DRC	EQUATEUR	95-99	180.37	158.03	204.95	RW2
DRC	EQUATEUR	00-04	148.06	178.74	121.86	HT-Direct
DRC	EQUATEUR	00-04	170.23	151.24	190.91	RW2
DRC	EQUATEUR	05-09	141.63	172.73	115.36	HT-Direct
DRC	EQUATEUR	05-09	149.23	130.25	170.35	RW2
DRC	EQUATEUR	10-14	141.25	178.11	110.99	HT-Direct

Continued on next page

Country	Region	Year	Median	Lower	Upper	Method
DRC	EQUATEUR	10-14	135.07	111.87	162.41	RW2
DRC	EQUATEUR	15-19	123.79	55.20	257.08	RW2
DRC	KASAI-OCCIDENTAL	80-84	251.38	359.52	167.27	HT-Direct
DRC	KASAI-OCCIDENTAL	80-84	258.25	193.80	336.20	RW2
DRC	KASAI-OCCIDENTAL	85-89	197.53	254.22	150.93	HT-Direct
DRC	KASAI-OCCIDENTAL	85-89	236.00	194.24	284.10	RW2
DRC	KASAI-OCCIDENTAL	90-94	189.70	237.17	149.85	HT-Direct
DRC	KASAI-OCCIDENTAL	90-94	212.96	186.35	242.36	RW2
DRC	KASAI-OCCIDENTAL	95-99	210.95	239.83	184.69	HT-Direct
DRC	KASAI-OCCIDENTAL	95-99	205.70	187.10	225.68	RW2
DRC	KASAI-OCCIDENTAL	00-04	177.00	210.87	147.55	HT-Direct
DRC	KASAI-OCCIDENTAL	00-04	181.81	163.43	201.93	RW2
DRC	KASAI-OCCIDENTAL	05-09	145.26	176.94	118.44	HT-Direct
DRC	KASAI-OCCIDENTAL	05-09	147.30	126.76	170.26	RW2
DRC	KASAI-OCCIDENTAL	10-14	105.02	152.58	71.04	HT-Direct
DRC	KASAI-OCCIDENTAL	10-14	121.73	96.17	152.45	RW2
DRC	KASAI-OCCIDENTAL	15-19	101.96	43.76	218.88	RW2
DRC	KASAI-ORIENTAL	80-84	129.05	195.97	82.63	HT-Direct
DRC	KASAI-ORIENTAL	80-84	204.83	155.91	264.46	RW2
DRC	KASAI-ORIENTAL	85-89	194.34	237.70	157.25	HT-Direct
DRC	KASAI-ORIENTAL	85-89	192.61	161.34	228.02	RW2
DRC	KASAI-ORIENTAL	90-94	174.12	209.68	143.49	HT-Direct
DRC	KASAI-ORIENTAL	90-94	178.70	158.42	200.88	RW2
DRC	KASAI-ORIENTAL	95-99	170.51	192.33	150.70	HT-Direct
DRC	KASAI-ORIENTAL	95-99	178.31	163.47	194.01	RW2
DRC	KASAI-ORIENTAL	00-04	161.36	186.33	139.17	HT-Direct
DRC	KASAI-ORIENTAL	00-04	163.00	148.17	179.05	RW2
DRC	KASAI-ORIENTAL	05-09	131.50	158.56	108.46	HT-Direct
DRC	KASAI-ORIENTAL	05-09	136.37	119.80	155.07	RW2
DRC	KASAI-ORIENTAL	10-14	111.25	145.55	84.23	HT-Direct
DRC	KASAI-ORIENTAL	10-14	116.76	96.26	141.12	RW2
DRC	KASAI-ORIENTAL	15-19	101.05	44.28	216.24	RW2
DRC	KATANGA	80-84	144.81	215.69	94.42	HT-Direct
DRC	KATANGA	80-84	209.83	157.40	272.74	RW2
DRC	KATANGA	85-89	180.11	231.00	138.41	HT-Direct
DRC	KATANGA	85-89	200.51	165.63	240.44	RW2
DRC	KATANGA	90-94	193.08	235.59	156.68	HT-Direct
DRC	KATANGA	90-94	189.09	165.59	214.82	RW2
DRC	KATANGA	95-99	184.51	217.86	155.26	HT-Direct
DRC	KATANGA	95-99	190.95	172.51	210.88	RW2
DRC	KATANGA	00-04	173.01	199.14	149.67	HT-Direct
DRC	KATANGA	00-04	175.55	159.87	192.77	RW2
DRC	KATANGA	05-09	130.85	154.29	110.51	HT-Direct
DRC	KATANGA	05-09	147.41	132.73	163.24	RW2
DRC	KATANGA	10-14	124.20	140.95	109.19	HT-Direct
DRC	KATANGA	10-14	126.71	112.48	142.38	RW2
DRC	KATANGA	15-19	109.94	50.36	225.34	RW2
DRC	KINSHASA	80-84	147.77	205.11	104.36	HT-Direct
DRC	KINSHASA	80-84	129.09	94.08	176.80	RW2
DRC	KINSHASA	85-89	87.95	118.44	64.72	HT-Direct
DRC	KINSHASA	85-89	119.38	96.71	147.12	RW2
DRC	KINSHASA	90-94	105.20	129.03	85.34	HT-Direct
DRC	KINSHASA	90-94	110.22	95.95	126.42	RW2
DRC	KINSHASA	95-99	106.92	125.85	90.55	HT-Direct
DRC	KINSHASA	95-99	111.32	99.79	123.89	RW2
DRC	KINSHASA	00-04	103.03	118.45	89.40	HT-Direct
DRC	KINSHASA	00-04	104.33	93.79	115.66	RW2
DRC	KINSHASA	05-09	78.25	98.19	62.07	HT-Direct
DRC	KINSHASA	05-09	91.42	79.68	104.73	RW2
DRC	KINSHASA	10-14	86.87	109.00	68.88	HT-Direct
DRC	KINSHASA	10-14	84.18	69.27	102.46	RW2
DRC	KINSHASA	15-19	79.04	34.00	175.08	RW2
DRC	MANIEMA	80-84	131.24	212.84	77.83	HT-Direct

Continued on next page

Country	Region	Year	Median	Lower	Upper	Method
DRC	MANIEMA	80-84	187.13	134.22	251.84	RW2
DRC	MANIEMA	85-89	163.64	228.40	114.52	HT-Direct
DRC	MANIEMA	85-89	184.65	147.46	227.57	RW2
DRC	MANIEMA	90-94	140.57	174.98	112.01	HT-Direct
DRC	MANIEMA	90-94	180.41	155.86	207.78	RW2
DRC	MANIEMA	95-99	223.91	262.36	189.64	HT-Direct
DRC	MANIEMA	95-99	188.86	169.30	210.80	RW2
DRC	MANIEMA	00-04	196.16	249.20	152.13	HT-Direct
DRC	MANIEMA	00-04	177.16	157.92	199.00	RW2
DRC	MANIEMA	05-09	135.61	162.29	112.73	HT-Direct
DRC	MANIEMA	05-09	150.01	131.02	171.13	RW2
DRC	MANIEMA	10-14	110.82	144.08	84.49	HT-Direct
DRC	MANIEMA	10-14	128.89	105.46	156.15	RW2
DRC	MANIEMA	15-19	111.89	48.97	235.38	RW2
DRC	NORD-KIVU	80-84	239.97	330.11	168.25	HT-Direct
DRC	NORD-KIVU	80-84	266.50	194.62	353.65	RW2
DRC	NORD-KIVU	85-89	127.40	189.88	83.36	HT-Direct
DRC	NORD-KIVU	85-89	217.27	170.83	272.13	RW2
DRC	NORD-KIVU	90-94	186.16	246.78	137.70	HT-Direct
DRC	NORD-KIVU	90-94	173.61	145.03	206.27	RW2
DRC	NORD-KIVU	95-99	144.53	183.51	112.68	HT-Direct
DRC	NORD-KIVU	95-99	147.51	127.73	169.66	RW2
DRC	NORD-KIVU	00-04	90.39	124.57	64.90	HT-Direct
DRC	NORD-KIVU	00-04	112.25	96.80	130.03	RW2
DRC	NORD-KIVU	05-09	80.82	99.85	65.15	HT-Direct
DRC	NORD-KIVU	05-09	76.40	63.83	91.27	RW2
DRC	NORD-KIVU	10-14	36.08	59.54	21.65	HT-Direct
DRC	NORD-KIVU	10-14	52.47	39.46	69.28	RW2
DRC	NORD-KIVU	15-19	36.14	14.40	87.93	RW2
DRC	ORIENTALE	80-84	193.65	312.05	112.80	HT-Direct
DRC	ORIENTALE	80-84	227.06	163.58	306.55	RW2
DRC	ORIENTALE	85-89	189.78	250.12	141.26	HT-Direct
DRC	ORIENTALE	85-89	208.56	166.13	259.10	RW2
DRC	ORIENTALE	90-94	162.38	219.21	118.06	HT-Direct
DRC	ORIENTALE	90-94	189.17	160.27	221.73	RW2
DRC	ORIENTALE	95-99	192.78	234.29	157.11	HT-Direct
DRC	ORIENTALE	95-99	184.36	163.06	207.83	RW2
DRC	ORIENTALE	00-04	154.71	187.77	126.56	HT-Direct
DRC	ORIENTALE	00-04	163.19	145.35	182.85	RW2
DRC	ORIENTALE	05-09	124.07	150.55	101.69	HT-Direct
DRC	ORIENTALE	05-09	131.66	114.85	150.63	RW2
DRC	ORIENTALE	10-14	105.48	135.52	81.47	HT-Direct
DRC	ORIENTALE	10-14	108.41	88.78	131.56	RW2
DRC	ORIENTALE	15-19	90.03	39.07	193.50	RW2
DRC	SUD-KIVU	80-84	252.32	331.34	186.88	HT-Direct
DRC	SUD-KIVU	80-84	267.83	200.97	347.48	RW2
DRC	SUD-KIVU	85-89	137.93	208.20	88.72	HT-Direct
DRC	SUD-KIVU	85-89	247.32	198.86	302.94	RW2
DRC	SUD-KIVU	90-94	226.61	290.05	173.66	HT-Direct
DRC	SUD-KIVU	90-94	226.50	193.71	263.07	RW2
DRC	SUD-KIVU	95-99	242.19	295.45	195.86	HT-Direct
DRC	SUD-KIVU	95-99	221.20	195.76	249.20	RW2
DRC	SUD-KIVU	00-04	178.09	217.00	144.87	HT-Direct
DRC	SUD-KIVU	00-04	195.81	173.74	220.14	RW2
DRC	SUD-KIVU	05-09	145.94	181.41	116.42	HT-Direct
DRC	SUD-KIVU	05-09	157.36	136.20	181.48	RW2
DRC	SUD-KIVU	10-14	129.41	166.65	99.49	HT-Direct
DRC	SUD-KIVU	10-14	128.61	104.39	157.25	RW2
DRC	SUD-KIVU	15-19	105.81	46.06	225.51	RW2
Egypt	ALL	80-84	144.41	148.75	140.17	HT-Direct
Egypt	ALL	80-84	141.99	138.72	145.18	IHME
Egypt	ALL	80-84	147.64	143.17	152.23	RW2
Egypt	ALL	80-84	147.62	144.54	150.81	UN

Continued on next page

Country	Region	Year	Median	Lower	Upper	Method
Egypt	ALL	85-89	98.18	101.15	95.28	HT-Direct
Egypt	ALL	85-89	97.53	95.24	99.97	IHME
Egypt	ALL	85-89	103.18	99.96	106.47	RW2
Egypt	ALL	85-89	103.23	101.14	105.40	UN
Egypt	ALL	90-94	77.83	80.62	75.13	HT-Direct
Egypt	ALL	90-94	74.53	72.62	76.55	IHME
Egypt	ALL	90-94	77.72	75.04	80.49	RW2
Egypt	ALL	90-94	77.64	76.01	79.24	UN
Egypt	ALL	95-99	54.31	56.84	51.88	HT-Direct
Egypt	ALL	95-99	52.51	50.86	54.20	IHME
Egypt	ALL	95-99	56.79	54.17	59.51	RW2
Egypt	ALL	95-99	56.85	55.60	58.34	UN
Egypt	ALL	00-04	38.94	41.38	36.64	HT-Direct
Egypt	ALL	00-04	38.05	36.67	39.38	IHME
Egypt	ALL	00-04	41.53	38.97	44.26	RW2
Egypt	ALL	00-04	41.53	40.30	42.88	UN
Egypt	ALL	05-09	32.00	35.02	29.24	HT-Direct
Egypt	ALL	05-09	31.32	29.78	32.92	IHME
Egypt	ALL	05-09	32.82	29.91	36.01	RW2
Egypt	ALL	05-09	32.82	31.32	34.10	UN
Egypt	ALL	10-14	25.79	24.14	27.47	IHME
Egypt	ALL	10-14	26.43	9.81	68.75	RW2
Egypt	ALL	10-14	26.84	24.98	28.86	UN
Egypt	FRONTIER GOVERNORATES	80-84	98.02	117.07	81.78	HT-Direct
Egypt	FRONTIER GOVERNORATES	80-84	99.33	85.54	115.16	RW2
Egypt	FRONTIER GOVERNORATES	85-89	70.59	82.61	60.20	HT-Direct
Egypt	FRONTIER GOVERNORATES	85-89	72.36	65.10	80.44	RW2
Egypt	FRONTIER GOVERNORATES	90-94	53.94	64.01	45.38	HT-Direct
Egypt	FRONTIER GOVERNORATES	90-94	56.98	51.09	63.27	RW2
Egypt	FRONTIER GOVERNORATES	95-99	44.93	55.25	36.47	HT-Direct
Egypt	FRONTIER GOVERNORATES	95-99	45.38	39.93	51.45	RW2
Egypt	FRONTIER GOVERNORATES	00-04	32.98	42.89	25.30	HT-Direct
Egypt	FRONTIER GOVERNORATES	00-04	36.73	31.05	43.51	RW2
Egypt	FRONTIER GOVERNORATES	05-09	35.12	49.99	24.56	HT-Direct
Egypt	FRONTIER GOVERNORATES	05-09	33.46	25.97	43.34	RW2
Egypt	FRONTIER GOVERNORATES	10-14	31.67	12.53	79.63	RW2
Egypt	FRONTIER GOVERNORATES	15-19	29.83	3.12	236.19	RW2
Egypt	LOWER EGYPT	80-84	122.40	128.61	116.45	HT-Direct
Egypt	LOWER EGYPT	80-84	125.83	119.93	132.05	RW2
Egypt	LOWER EGYPT	85-89	83.34	87.71	79.18	HT-Direct
Egypt	LOWER EGYPT	85-89	86.65	82.87	90.64	RW2
Egypt	LOWER EGYPT	90-94	63.23	67.30	59.40	HT-Direct
Egypt	LOWER EGYPT	90-94	63.14	60.01	66.40	RW2
Egypt	LOWER EGYPT	95-99	42.66	46.44	39.18	HT-Direct
Egypt	LOWER EGYPT	95-99	45.84	42.86	48.97	RW2
Egypt	LOWER EGYPT	00-04	32.65	36.53	29.17	HT-Direct
Egypt	LOWER EGYPT	00-04	33.76	30.90	36.90	RW2
Egypt	LOWER EGYPT	05-09	26.74	31.53	22.66	HT-Direct
Egypt	LOWER EGYPT	05-09	27.74	24.13	31.92	RW2
Egypt	LOWER EGYPT	10-14	23.57	9.64	56.81	RW2
Egypt	LOWER EGYPT	15-19	20.07	2.16	167.70	RW2
Egypt	UPPER EGYPT	80-84	195.32	202.99	187.86	HT-Direct
Egypt	UPPER EGYPT	80-84	198.20	190.74	205.86	RW2
Egypt	UPPER EGYPT	85-89	127.49	132.51	122.64	HT-Direct
Egypt	UPPER EGYPT	85-89	136.80	131.86	141.74	RW2
Egypt	UPPER EGYPT	90-94	103.65	108.25	99.23	HT-Direct
Egypt	UPPER EGYPT	90-94	102.00	98.14	106.06	RW2
Egypt	UPPER EGYPT	95-99	71.69	75.77	67.82	HT-Direct
Egypt	UPPER EGYPT	95-99	74.22	70.57	78.09	RW2
Egypt	UPPER EGYPT	00-04	48.99	52.85	45.40	HT-Direct
Egypt	UPPER EGYPT	00-04	52.71	49.20	56.46	RW2
Egypt	UPPER EGYPT	05-09	39.49	44.37	35.14	HT-Direct
Egypt	UPPER EGYPT	05-09	41.13	36.90	45.82	RW2

Continued on next page

Country	Region	Year	Median	Lower	Upper	Method
Egypt	UPPER EGYPT	10-14	33.01	13.79	77.36	RW2
Egypt	UPPER EGYPT	15-19	26.46	2.81	208.44	RW2
Egypt	URBAN GOVERNORATES	80-84	86.39	94.19	79.19	HT-Direct
Egypt	URBAN GOVERNORATES	80-84	90.03	83.04	97.51	RW2
Egypt	URBAN GOVERNORATES	85-89	66.50	72.91	60.62	HT-Direct
Egypt	URBAN GOVERNORATES	85-89	65.27	61.02	69.98	RW2
Egypt	URBAN GOVERNORATES	90-94	46.74	52.60	41.50	HT-Direct
Egypt	URBAN GOVERNORATES	90-94	50.40	46.41	54.53	RW2
Egypt	URBAN GOVERNORATES	95-99	37.91	44.53	32.24	HT-Direct
Egypt	URBAN GOVERNORATES	95-99	39.56	35.63	43.72	RW2
Egypt	URBAN GOVERNORATES	00-04	29.24	35.49	24.07	HT-Direct
Egypt	URBAN GOVERNORATES	00-04	31.84	27.90	36.38	RW2
Egypt	URBAN GOVERNORATES	05-09	30.64	40.19	23.30	HT-Direct
Egypt	URBAN GOVERNORATES	05-09	28.99	23.64	35.86	RW2
Egypt	URBAN GOVERNORATES	10-14	27.49	10.98	67.89	RW2
Egypt	URBAN GOVERNORATES	15-19	26.12	2.75	213.27	RW2
Ethiopia	ADDIS ABABA	80-84	90.19	117.00	69.04	HT-Direct
Ethiopia	ADDIS ABABA	80-84	89.89	72.59	110.43	RW2
Ethiopia	ADDIS ABABA	85-89	85.55	107.51	67.73	HT-Direct
Ethiopia	ADDIS ABABA	85-89	89.29	77.43	102.73	RW2
Ethiopia	ADDIS ABABA	90-94	85.66	105.45	69.30	HT-Direct
Ethiopia	ADDIS ABABA	90-94	90.05	79.75	101.70	RW2
Ethiopia	ADDIS ABABA	95-99	93.37	111.53	77.90	HT-Direct
Ethiopia	ADDIS ABABA	95-99	79.49	70.09	90.62	RW2
Ethiopia	ADDIS ABABA	00-04	52.84	72.01	38.56	HT-Direct
Ethiopia	ADDIS ABABA	00-04	64.17	54.90	74.96	RW2
Ethiopia	ADDIS ABABA	05-09	46.19	65.19	32.53	HT-Direct
Ethiopia	ADDIS ABABA	05-09	47.08	37.85	58.23	RW2
Ethiopia	ADDIS ABABA	10-14	35.14	63.26	19.27	HT-Direct
Ethiopia	ADDIS ABABA	10-14	34.81	24.26	48.59	RW2
Ethiopia	ADDIS ABABA	15-19	25.95	9.91	64.50	RW2
Ethiopia	AFFAR	80-84	328.98	380.06	281.64	HT-Direct
Ethiopia	AFFAR	80-84	318.85	284.16	357.80	RW2
Ethiopia	AFFAR	85-89	292.96	327.95	260.26	HT-Direct
Ethiopia	AFFAR	85-89	275.80	254.91	297.97	RW2
Ethiopia	AFFAR	90-94	240.69	271.11	212.68	HT-Direct
Ethiopia	AFFAR	90-94	240.11	223.02	257.30	RW2
Ethiopia	AFFAR	95-99	179.55	202.24	158.90	HT-Direct
Ethiopia	AFFAR	95-99	193.45	178.22	208.24	RW2
Ethiopia	AFFAR	00-04	154.54	178.86	132.99	HT-Direct
Ethiopia	AFFAR	00-04	155.07	142.26	168.21	RW2
Ethiopia	AFFAR	05-09	122.21	141.98	104.86	HT-Direct
Ethiopia	AFFAR	05-09	117.85	106.35	130.61	RW2
Ethiopia	AFFAR	10-14	114.45	145.45	89.36	HT-Direct
Ethiopia	AFFAR	10-14	89.92	76.67	106.47	RW2
Ethiopia	AFFAR	15-19	68.89	31.38	146.58	RW2
Ethiopia	ALL	80-84	237.38	251.21	224.10	HT-Direct
Ethiopia	ALL	80-84	254.72	235.08	275.78	IHME
Ethiopia	ALL	80-84	235.97	222.85	249.61	RW2
Ethiopia	ALL	80-84	235.81	227.46	244.60	UN
Ethiopia	ALL	85-89	214.10	223.65	204.86	HT-Direct
Ethiopia	ALL	85-89	211.56	208.67	214.26	IHME
Ethiopia	ALL	85-89	215.41	206.20	224.88	RW2
Ethiopia	ALL	85-89	215.75	208.65	222.99	UN
Ethiopia	ALL	90-94	202.75	211.15	194.59	HT-Direct
Ethiopia	ALL	90-94	191.46	188.83	193.83	IHME
Ethiopia	ALL	90-94	194.59	187.18	202.25	RW2
Ethiopia	ALL	90-94	194.17	188.18	201.32	UN
Ethiopia	ALL	95-99	160.00	166.44	153.76	HT-Direct
Ethiopia	ALL	95-99	162.49	160.11	164.87	IHME
Ethiopia	ALL	95-99	161.77	155.47	168.22	RW2
Ethiopia	ALL	95-99	162.00	156.75	167.62	UN
Ethiopia	ALL	00-04	131.67	138.63	125.00	HT-Direct

Continued on next page

Country	Region	Year	Median	Lower	Upper	Method
Ethiopia	ALL	00-04	129.27	127.17	131.38	IHME
Ethiopia	ALL	00-04	131.82	125.31	138.67	RW2
Ethiopia	ALL	00-04	131.72	126.68	136.42	UN
Ethiopia	ALL	05-09	96.77	103.94	90.05	HT-Direct
Ethiopia	ALL	05-09	97.86	95.47	100.57	IHME
Ethiopia	ALL	05-09	94.28	88.06	100.87	RW2
Ethiopia	ALL	05-09	94.39	89.20	99.54	UN
Ethiopia	ALL	10-14	76.09	85.93	67.30	HT-Direct
Ethiopia	ALL	10-14	72.87	69.72	75.85	IHME
Ethiopia	ALL	10-14	67.46	60.49	75.11	RW2
Ethiopia	ALL	10-14	67.36	60.19	75.41	UN
Ethiopia	AMHARA	80-84	236.38	261.23	213.21	HT-Direct
Ethiopia	AMHARA	80-84	234.19	215.15	254.36	RW2
Ethiopia	AMHARA	85-89	216.11	235.03	198.31	HT-Direct
Ethiopia	AMHARA	85-89	212.94	200.38	225.65	RW2
Ethiopia	AMHARA	90-94	196.04	210.96	181.93	HT-Direct
Ethiopia	AMHARA	90-94	196.03	186.01	206.13	RW2
Ethiopia	AMHARA	95-99	163.13	175.63	151.36	HT-Direct
Ethiopia	AMHARA	95-99	166.19	157.47	175.42	RW2
Ethiopia	AMHARA	00-04	151.21	167.39	136.35	HT-Direct
Ethiopia	AMHARA	00-04	136.62	127.32	147.04	RW2
Ethiopia	AMHARA	05-09	103.66	122.62	87.34	HT-Direct
Ethiopia	AMHARA	05-09	101.01	91.17	111.79	RW2
Ethiopia	AMHARA	10-14	66.28	87.36	50.01	HT-Direct
Ethiopia	AMHARA	10-14	72.58	60.36	85.57	RW2
Ethiopia	AMHARA	15-19	51.66	22.79	112.68	RW2
Ethiopia	BENISHANGUL-GUMUZ	80-84	273.63	323.95	228.49	HT-Direct
Ethiopia	BENISHANGUL-GUMUZ	80-84	270.40	236.35	307.28	RW2
Ethiopia	BENISHANGUL-GUMUZ	85-89	255.95	291.44	223.42	HT-Direct
Ethiopia	BENISHANGUL-GUMUZ	85-89	251.52	230.69	273.31	RW2
Ethiopia	BENISHANGUL-GUMUZ	90-94	240.45	265.79	216.81	HT-Direct
Ethiopia	BENISHANGUL-GUMUZ	90-94	237.28	220.99	253.73	RW2
Ethiopia	BENISHANGUL-GUMUZ	95-99	194.10	219.21	171.24	HT-Direct
Ethiopia	BENISHANGUL-GUMUZ	95-99	206.17	191.52	221.93	RW2
Ethiopia	BENISHANGUL-GUMUZ	00-04	190.56	214.51	168.70	HT-Direct
Ethiopia	BENISHANGUL-GUMUZ	00-04	172.43	158.29	188.83	RW2
Ethiopia	BENISHANGUL-GUMUZ	05-09	135.41	165.59	110.01	HT-Direct
Ethiopia	BENISHANGUL-GUMUZ	05-09	128.00	114.08	143.37	RW2
Ethiopia	BENISHANGUL-GUMUZ	10-14	90.34	116.33	69.70	HT-Direct
Ethiopia	BENISHANGUL-GUMUZ	10-14	90.93	74.90	108.51	RW2
Ethiopia	BENISHANGUL-GUMUZ	15-19	63.66	27.95	137.99	RW2
Ethiopia	DIRE DAWA	80-84	204.66	256.04	161.36	HT-Direct
Ethiopia	DIRE DAWA	80-84	223.81	189.76	260.90	RW2
Ethiopia	DIRE DAWA	85-89	219.10	252.79	188.77	HT-Direct
Ethiopia	DIRE DAWA	85-89	210.13	189.76	231.98	RW2
Ethiopia	DIRE DAWA	90-94	203.24	233.63	175.89	HT-Direct
Ethiopia	DIRE DAWA	90-94	197.41	181.20	215.11	RW2
Ethiopia	DIRE DAWA	95-99	169.82	197.15	145.59	HT-Direct
Ethiopia	DIRE DAWA	95-99	165.01	151.11	180.46	RW2
Ethiopia	DIRE DAWA	00-04	129.26	150.21	110.85	HT-Direct
Ethiopia	DIRE DAWA	00-04	131.23	119.30	144.20	RW2
Ethiopia	DIRE DAWA	05-09	96.29	115.74	79.82	HT-Direct
Ethiopia	DIRE DAWA	05-09	96.36	85.68	108.33	RW2
Ethiopia	DIRE DAWA	10-14	81.13	105.84	61.79	HT-Direct
Ethiopia	DIRE DAWA	10-14	71.09	59.05	85.10	RW2
Ethiopia	DIRE DAWA	15-19	52.69	23.29	115.28	RW2
Ethiopia	GAMBELA	80-84	265.93	312.11	224.35	HT-Direct
Ethiopia	GAMBELA	80-84	273.14	238.32	309.68	RW2
Ethiopia	GAMBELA	85-89	245.68	285.64	209.67	HT-Direct
Ethiopia	GAMBELA	85-89	254.73	232.31	278.42	RW2
Ethiopia	GAMBELA	90-94	261.14	294.62	230.23	HT-Direct
Ethiopia	GAMBELA	90-94	235.05	217.17	254.78	RW2
Ethiopia	GAMBELA	95-99	193.44	219.53	169.77	HT-Direct

Continued on next page

Country	Region	Year	Median	Lower	Upper	Method
Ethiopia	GAMBELA	95-99	189.37	174.51	205.72	RW2
Ethiopia	GAMBELA	00-04	126.24	149.32	106.29	HT-Direct
Ethiopia	GAMBELA	00-04	143.74	130.05	158.09	RW2
Ethiopia	GAMBELA	05-09	106.75	130.40	86.96	HT-Direct
Ethiopia	GAMBELA	05-09	102.65	90.83	115.57	RW2
Ethiopia	GAMBELA	10-14	87.32	111.89	67.73	HT-Direct
Ethiopia	GAMBELA	10-14	74.36	62.20	88.78	RW2
Ethiopia	GAMBELA	15-19	54.28	24.06	118.42	RW2
Ethiopia	HARARI	80-84	265.94	335.32	206.46	HT-Direct
Ethiopia	HARARI	80-84	249.58	208.16	295.88	RW2
Ethiopia	HARARI	85-89	194.98	228.33	165.45	HT-Direct
Ethiopia	HARARI	85-89	219.81	197.45	243.88	RW2
Ethiopia	HARARI	90-94	222.73	251.20	196.63	HT-Direct
Ethiopia	HARARI	90-94	195.38	179.24	213.19	RW2
Ethiopia	HARARI	95-99	151.24	175.09	130.13	HT-Direct
Ethiopia	HARARI	95-99	150.92	137.31	165.39	RW2
Ethiopia	HARARI	00-04	98.46	117.28	82.38	HT-Direct
Ethiopia	HARARI	00-04	111.37	98.88	124.19	RW2
Ethiopia	HARARI	05-09	77.26	96.34	61.71	HT-Direct
Ethiopia	HARARI	05-09	79.87	69.60	91.36	RW2
Ethiopia	HARARI	10-14	78.87	105.46	58.54	HT-Direct
Ethiopia	HARARI	10-14	60.24	48.71	75.24	RW2
Ethiopia	HARARI	15-19	46.20	19.78	106.68	RW2
Ethiopia	OROMIYA	80-84	244.25	268.72	221.34	HT-Direct
Ethiopia	OROMIYA	80-84	235.24	217.63	254.13	RW2
Ethiopia	OROMIYA	85-89	204.02	220.58	188.40	HT-Direct
Ethiopia	OROMIYA	85-89	213.14	201.51	225.33	RW2
Ethiopia	OROMIYA	90-94	207.72	224.96	191.48	HT-Direct
Ethiopia	OROMIYA	90-94	195.54	185.93	205.55	RW2
Ethiopia	OROMIYA	95-99	158.25	170.20	146.99	HT-Direct
Ethiopia	OROMIYA	95-99	160.71	152.72	169.09	RW2
Ethiopia	OROMIYA	00-04	127.96	140.86	116.08	HT-Direct
Ethiopia	OROMIYA	00-04	126.69	119.10	134.74	RW2
Ethiopia	OROMIYA	05-09	93.37	105.08	82.85	HT-Direct
Ethiopia	OROMIYA	05-09	92.57	85.31	100.29	RW2
Ethiopia	OROMIYA	10-14	78.51	96.58	63.58	HT-Direct
Ethiopia	OROMIYA	10-14	67.84	59.62	77.01	RW2
Ethiopia	OROMIYA	15-19	50.09	22.95	105.51	RW2
Ethiopia	SNNP	80-84	237.15	270.34	206.88	HT-Direct
Ethiopia	SNNP	80-84	245.36	219.90	271.79	RW2
Ethiopia	SNNP	85-89	237.31	259.20	216.72	HT-Direct
Ethiopia	SNNP	85-89	229.81	215.19	245.26	RW2
Ethiopia	SNNP	90-94	218.67	235.95	202.33	HT-Direct
Ethiopia	SNNP	90-94	212.86	201.59	224.88	RW2
Ethiopia	SNNP	95-99	173.44	187.58	160.15	HT-Direct
Ethiopia	SNNP	95-99	174.54	164.85	184.81	RW2
Ethiopia	SNNP	00-04	135.26	148.19	123.30	HT-Direct
Ethiopia	SNNP	00-04	136.37	127.38	145.70	RW2
Ethiopia	SNNP	05-09	102.83	118.89	88.72	HT-Direct
Ethiopia	SNNP	05-09	99.72	90.51	109.75	RW2
Ethiopia	SNNP	10-14	83.34	106.93	64.59	HT-Direct
Ethiopia	SNNP	10-14	73.60	62.29	87.04	RW2
Ethiopia	SNNP	15-19	54.61	24.32	119.06	RW2
Ethiopia	SOMALI	80-84	202.00	260.21	154.09	HT-Direct
Ethiopia	SOMALI	80-84	178.96	148.71	214.11	RW2
Ethiopia	SOMALI	85-89	147.16	180.15	119.32	HT-Direct
Ethiopia	SOMALI	85-89	171.42	151.18	193.44	RW2
Ethiopia	SOMALI	90-94	188.28	227.20	154.69	HT-Direct
Ethiopia	SOMALI	90-94	167.32	151.57	184.22	RW2
Ethiopia	SOMALI	95-99	142.12	162.79	123.68	HT-Direct
Ethiopia	SOMALI	95-99	147.61	135.33	160.61	RW2
Ethiopia	SOMALI	00-04	129.97	148.62	113.35	HT-Direct
Ethiopia	SOMALI	00-04	126.73	116.43	137.83	RW2

Continued on next page

Country	Region	Year	Median	Lower	Upper	Method
Ethiopia	SOMALI	05-09	103.87	120.31	89.46	HT-Direct
Ethiopia	SOMALI	05-09	101.56	92.28	111.81	RW2
Ethiopia	SOMALI	10-14	91.15	110.38	74.98	HT-Direct
Ethiopia	SOMALI	10-14	81.86	71.20	94.02	RW2
Ethiopia	SOMALI	15-19	66.41	30.28	140.75	RW2
Ethiopia	TIGRAY	80-84	252.69	285.29	222.67	HT-Direct
Ethiopia	TIGRAY	80-84	259.46	234.14	286.53	RW2
Ethiopia	TIGRAY	85-89	220.93	243.98	199.48	HT-Direct
Ethiopia	TIGRAY	85-89	219.60	204.72	235.61	RW2
Ethiopia	TIGRAY	90-94	197.68	217.23	179.50	HT-Direct
Ethiopia	TIGRAY	90-94	184.43	172.97	196.68	RW2
Ethiopia	TIGRAY	95-99	138.63	152.48	125.84	HT-Direct
Ethiopia	TIGRAY	95-99	138.70	129.32	148.35	RW2
Ethiopia	TIGRAY	00-04	89.39	102.43	77.87	HT-Direct
Ethiopia	TIGRAY	00-04	101.89	93.53	110.65	RW2
Ethiopia	TIGRAY	05-09	77.96	91.18	66.52	HT-Direct
Ethiopia	TIGRAY	05-09	70.68	63.73	78.34	RW2
Ethiopia	TIGRAY	10-14	55.22	69.42	43.78	HT-Direct
Ethiopia	TIGRAY	10-14	48.88	41.56	57.62	RW2
Ethiopia	TIGRAY	15-19	33.96	14.90	75.20	RW2
Gabon	ALL	80-84	105.93	121.81	91.90	HT-Direct
Gabon	ALL	80-84	105.56	102.43	108.52	IHME
Gabon	ALL	80-84	110.48	95.37	127.67	RW2
Gabon	ALL	80-84	109.94	102.25	119.10	UN
Gabon	ALL	85-89	82.19	92.07	73.28	HT-Direct
Gabon	ALL	85-89	89.66	87.53	91.86	IHME
Gabon	ALL	85-89	96.12	84.72	108.69	RW2
Gabon	ALL	85-89	96.92	90.85	103.92	UN
Gabon	ALL	90-94	89.79	99.87	80.62	HT-Direct
Gabon	ALL	90-94	79.90	78.23	81.80	IHME
Gabon	ALL	90-94	92.83	83.69	102.90	RW2
Gabon	ALL	90-94	91.93	86.14	97.99	UN
Gabon	ALL	95-99	77.78	87.16	69.33	HT-Direct
Gabon	ALL	95-99	73.53	71.86	75.24	IHME
Gabon	ALL	95-99	88.84	78.51	100.29	RW2
Gabon	ALL	95-99	88.83	83.31	94.85	UN
Gabon	ALL	00-04	63.12	77.25	51.43	HT-Direct
Gabon	ALL	00-04	69.99	68.33	71.76	IHME
Gabon	ALL	00-04	82.35	66.09	102.83	RW2
Gabon	ALL	00-04	83.16	77.53	89.24	UN
Gabon	ALL	05-09	54.35	63.32	46.59	HT-Direct
Gabon	ALL	05-09	63.23	61.13	64.97	IHME
Gabon	ALL	05-09	70.22	57.95	84.82	RW2
Gabon	ALL	05-09	71.64	66.79	76.89	UN
Gabon	ALL	10-14	81.07	98.47	66.52	HT-Direct
Gabon	ALL	10-14	52.38	49.61	55.02	IHME
Gabon	ALL	10-14	58.55	50.24	68.07	RW2
Gabon	ALL	10-14	57.25	51.45	64.37	UN
Gabon	EAST	80-84	71.07	99.51	50.30	HT-Direct
Gabon	EAST	80-84	75.06	56.14	99.98	RW2
Gabon	EAST	85-89	59.77	81.58	43.52	HT-Direct
Gabon	EAST	85-89	63.53	51.34	78.16	RW2
Gabon	EAST	90-94	53.77	66.88	43.10	HT-Direct
Gabon	EAST	90-94	59.36	50.27	69.84	RW2
Gabon	EAST	95-99	62.94	78.44	50.34	HT-Direct
Gabon	EAST	95-99	70.10	59.01	83.26	RW2
Gabon	EAST	00-04	60.95	81.52	45.32	HT-Direct
Gabon	EAST	00-04	78.85	62.45	100.02	RW2
Gabon	EAST	05-09	53.92	75.62	38.19	HT-Direct
Gabon	EAST	05-09	69.63	54.29	89.38	RW2
Gabon	EAST	10-14	77.36	118.42	49.74	HT-Direct
Gabon	EAST	10-14	56.87	42.26	76.01	RW2
Gabon	EAST	15-19	45.98	16.17	124.15	RW2

Continued on next page

Country	Region	Year	Median	Lower	Upper	Method
Gabon	LIBREVILLE,PORT-GENTIL	80-84	98.38	125.53	76.59	HT-Direct
Gabon	LIBREVILLE,PORT-GENTIL	80-84	113.60	88.89	142.62	RW2
Gabon	LIBREVILLE,PORT-GENTIL	85-89	83.11	100.47	68.52	HT-Direct
Gabon	LIBREVILLE,PORT-GENTIL	85-89	101.20	86.08	118.69	RW2
Gabon	LIBREVILLE,PORT-GENTIL	90-94	104.24	122.98	88.07	HT-Direct
Gabon	LIBREVILLE,PORT-GENTIL	90-94	92.58	80.02	107.94	RW2
Gabon	LIBREVILLE,PORT-GENTIL	95-99	73.79	91.46	59.31	HT-Direct
Gabon	LIBREVILLE,PORT-GENTIL	95-99	93.51	78.65	110.96	RW2
Gabon	LIBREVILLE,PORT-GENTIL	00-04	54.34	79.61	36.78	HT-Direct
Gabon	LIBREVILLE,PORT-GENTIL	00-04	88.14	66.74	113.27	RW2
Gabon	LIBREVILLE,PORT-GENTIL	05-09	47.17	63.14	35.10	HT-Direct
Gabon	LIBREVILLE,PORT-GENTIL	05-09	69.07	52.97	87.86	RW2
Gabon	LIBREVILLE,PORT-GENTIL	10-14	75.53	107.89	52.30	HT-Direct
Gabon	LIBREVILLE,PORT-GENTIL	10-14	53.11	40.84	68.68	RW2
Gabon	LIBREVILLE,PORT-GENTIL	15-19	41.09	14.67	113.80	RW2
Gabon	NORTH	80-84	125.59	166.00	93.90	HT-Direct
Gabon	NORTH	80-84	136.81	107.53	172.54	RW2
Gabon	NORTH	85-89	101.25	123.63	82.54	HT-Direct
Gabon	NORTH	85-89	110.70	93.78	130.39	RW2
Gabon	NORTH	90-94	89.93	108.09	74.56	HT-Direct
Gabon	NORTH	90-94	97.69	84.75	112.04	RW2
Gabon	NORTH	95-99	97.87	117.82	80.98	HT-Direct
Gabon	NORTH	95-99	107.45	92.36	125.18	RW2
Gabon	NORTH	00-04	90.92	123.90	66.05	HT-Direct
Gabon	NORTH	00-04	112.83	91.41	140.24	RW2
Gabon	NORTH	05-09	75.44	90.90	62.44	HT-Direct
Gabon	NORTH	05-09	94.59	78.57	113.70	RW2
Gabon	NORTH	10-14	100.25	126.73	78.81	HT-Direct
Gabon	NORTH	10-14	73.60	61.84	87.44	RW2
Gabon	NORTH	15-19	56.53	21.68	141.29	RW2
Gabon	SOUTH	80-84	120.68	156.22	92.35	HT-Direct
Gabon	SOUTH	80-84	125.02	98.72	156.98	RW2
Gabon	SOUTH	85-89	75.71	97.39	58.54	HT-Direct
Gabon	SOUTH	85-89	98.76	82.51	117.67	RW2
Gabon	SOUTH	90-94	86.45	103.33	72.11	HT-Direct
Gabon	SOUTH	90-94	85.95	74.62	98.70	RW2
Gabon	SOUTH	95-99	78.64	95.31	64.68	HT-Direct
Gabon	SOUTH	95-99	92.22	79.03	108.19	RW2
Gabon	SOUTH	00-04	82.52	107.82	62.74	HT-Direct
Gabon	SOUTH	00-04	93.57	74.92	117.84	RW2
Gabon	SOUTH	05-09	66.64	89.87	49.10	HT-Direct
Gabon	SOUTH	05-09	74.15	59.11	93.09	RW2
Gabon	SOUTH	10-14	68.52	96.32	48.31	HT-Direct
Gabon	SOUTH	10-14	53.66	41.87	68.19	RW2
Gabon	SOUTH	15-19	38.09	13.77	101.42	RW2
Gabon	WEST	80-84	154.47	195.61	120.68	HT-Direct
Gabon	WEST	80-84	142.34	114.10	179.28	RW2
Gabon	WEST	85-89	97.53	118.17	80.17	HT-Direct
Gabon	WEST	85-89	106.86	90.89	125.18	RW2
Gabon	WEST	90-94	67.05	83.30	53.79	HT-Direct
Gabon	WEST	90-94	88.55	75.56	102.65	RW2
Gabon	WEST	95-99	88.25	106.38	72.95	HT-Direct
Gabon	WEST	95-99	93.22	78.36	109.05	RW2
Gabon	WEST	00-04	64.81	106.86	38.59	HT-Direct
Gabon	WEST	00-04	96.37	75.87	121.26	RW2
Gabon	WEST	05-09	57.56	76.43	43.13	HT-Direct
Gabon	WEST	05-09	82.71	66.57	101.99	RW2
Gabon	WEST	10-14	101.09	132.91	76.21	HT-Direct
Gabon	WEST	10-14	68.30	55.55	84.13	RW2
Gabon	WEST	15-19	56.61	21.55	143.38	RW2
Gambia	ALL	80-84	180.05	246.58	128.42	HT-Direct
Gambia	ALL	80-84	135.82	131.90	139.80	IHME
Gambia	ALL	80-84	231.78	145.93	347.78	RW2

Continued on next page

Country	Region	Year	Median	Lower	Upper	Method
Gambia	ALL	80-84	228.47	214.24	242.39	UN
Gambia	ALL	85-89	126.99	156.51	102.36	HT-Direct
Gambia	ALL	85-89	120.48	117.40	123.37	IHME
Gambia	ALL	85-89	190.37	137.48	257.00	RW2
Gambia	ALL	85-89	191.14	180.53	202.28	UN
Gambia	ALL	90-94	105.96	124.30	90.05	HT-Direct
Gambia	ALL	90-94	108.04	105.88	110.37	IHME
Gambia	ALL	90-94	158.19	120.92	204.28	RW2
Gambia	ALL	90-94	158.24	150.73	166.37	UN
Gambia	ALL	95-99	91.88	105.60	79.78	HT-Direct
Gambia	ALL	95-99	94.18	92.22	96.12	IHME
Gambia	ALL	95-99	132.33	106.91	162.34	RW2
Gambia	ALL	95-99	132.66	126.03	139.90	UN
Gambia	ALL	00-04	84.69	96.76	74.00	HT-Direct
Gambia	ALL	00-04	79.35	77.59	81.14	IHME
Gambia	ALL	00-04	111.24	93.35	132.28	RW2
Gambia	ALL	00-04	110.82	104.31	117.45	UN
Gambia	ALL	05-09	66.52	75.79	58.31	HT-Direct
Gambia	ALL	05-09	65.01	63.14	66.77	IHME
Gambia	ALL	05-09	91.10	75.52	109.44	RW2
Gambia	ALL	05-09	90.98	83.48	100.36	UN
Gambia	ALL	10-14	51.06	60.35	43.13	HT-Direct
Gambia	ALL	10-14	51.98	49.99	54.01	IHME
Gambia	ALL	10-14	75.81	54.80	103.78	RW2
Gambia	ALL	10-14	76.27	66.30	88.83	UN
Gambia	BANJUL	80-84	112.43	300.72	35.97	HT-Direct
Gambia	BANJUL	80-84	128.94	50.54	297.19	RW2
Gambia	BANJUL	85-89	86.72	154.53	47.01	HT-Direct
Gambia	BANJUL	85-89	110.04	55.28	206.32	RW2
Gambia	BANJUL	90-94	81.83	130.12	50.42	HT-Direct
Gambia	BANJUL	90-94	97.03	58.20	157.85	RW2
Gambia	BANJUL	95-99	49.26	71.85	33.51	HT-Direct
Gambia	BANJUL	95-99	88.03	59.66	127.26	RW2
Gambia	BANJUL	00-04	53.64	80.84	35.24	HT-Direct
Gambia	BANJUL	00-04	82.55	59.45	112.99	RW2
Gambia	BANJUL	05-09	56.07	75.97	41.15	HT-Direct
Gambia	BANJUL	05-09	75.02	53.23	105.09	RW2
Gambia	BANJUL	10-14	50.91	70.81	36.39	HT-Direct
Gambia	BANJUL	10-14	68.14	40.94	112.05	RW2
Gambia	BANJUL	15-19	62.04	16.83	206.48	RW2
Gambia	CENTRAL RIVER	80-84	145.98	299.41	63.99	HT-Direct
Gambia	CENTRAL RIVER	80-84	223.76	109.53	403.47	RW2
Gambia	CENTRAL RIVER	85-89	111.39	194.41	61.13	HT-Direct
Gambia	CENTRAL RIVER	85-89	182.43	105.68	296.38	RW2
Gambia	CENTRAL RIVER	90-94	84.32	120.50	58.28	HT-Direct
Gambia	CENTRAL RIVER	90-94	152.46	101.06	222.95	RW2
Gambia	CENTRAL RIVER	95-99	107.74	145.43	78.92	HT-Direct
Gambia	CENTRAL RIVER	95-99	127.64	94.47	170.17	RW2
Gambia	CENTRAL RIVER	00-04	78.10	99.95	60.70	HT-Direct
Gambia	CENTRAL RIVER	00-04	106.88	83.35	136.17	RW2
Gambia	CENTRAL RIVER	05-09	57.94	77.17	43.27	HT-Direct
Gambia	CENTRAL RIVER	05-09	83.95	61.95	112.82	RW2
Gambia	CENTRAL RIVER	10-14	41.43	57.43	29.75	HT-Direct
Gambia	CENTRAL RIVER	10-14	64.01	39.87	100.87	RW2
Gambia	CENTRAL RIVER	15-19	48.15	13.62	156.26	RW2
Gambia	LOWER RIVER	80-84	380.22	540.49	242.40	HT-Direct
Gambia	LOWER RIVER	80-84	390.18	225.43	584.05	RW2
Gambia	LOWER RIVER	85-89	167.40	247.12	109.66	HT-Direct
Gambia	LOWER RIVER	85-89	294.69	187.72	431.66	RW2
Gambia	LOWER RIVER	90-94	141.20	190.88	102.81	HT-Direct
Gambia	LOWER RIVER	90-94	221.75	151.52	312.25	RW2
Gambia	LOWER RIVER	95-99	120.08	176.79	79.80	HT-Direct
Gambia	LOWER RIVER	95-99	164.80	119.71	223.19	RW2

Continued on next page

Country	Region	Year	Median	Lower	Upper	Method
Gambia	LOWER RIVER	00-04	83.50	129.15	53.01	HT-Direct
Gambia	LOWER RIVER	00-04	121.84	90.10	163.94	RW2
Gambia	LOWER RIVER	05-09	72.16	96.98	53.32	HT-Direct
Gambia	LOWER RIVER	05-09	84.36	58.61	120.19	RW2
Gambia	LOWER RIVER	10-14	34.17	55.32	20.93	HT-Direct
Gambia	LOWER RIVER	10-14	56.39	32.22	97.25	RW2
Gambia	LOWER RIVER	15-19	37.11	9.73	131.09	RW2
Gambia	NORTH BANK	80-84	206.13	350.18	111.20	HT-Direct
Gambia	NORTH BANK	80-84	279.84	156.63	449.03	RW2
Gambia	NORTH BANK	85-89	148.72	201.16	108.10	HT-Direct
Gambia	NORTH BANK	85-89	219.04	142.40	320.55	RW2
Gambia	NORTH BANK	90-94	101.70	136.95	74.75	HT-Direct
Gambia	NORTH BANK	90-94	174.28	123.85	240.21	RW2
Gambia	NORTH BANK	95-99	96.34	125.47	73.40	HT-Direct
Gambia	NORTH BANK	95-99	138.42	104.91	180.22	RW2
Gambia	NORTH BANK	00-04	93.08	119.46	72.04	HT-Direct
Gambia	NORTH BANK	00-04	109.37	84.74	140.51	RW2
Gambia	NORTH BANK	05-09	55.23	75.04	40.43	HT-Direct
Gambia	NORTH BANK	05-09	80.69	56.96	112.79	RW2
Gambia	NORTH BANK	10-14	28.97	51.08	16.27	HT-Direct
Gambia	NORTH BANK	10-14	57.46	32.18	100.13	RW2
Gambia	NORTH BANK	15-19	40.74	10.24	145.43	RW2
Gambia	UPPER RIVER	80-84	240.89	356.14	154.02	HT-Direct
Gambia	UPPER RIVER	80-84	334.64	199.47	502.82	RW2
Gambia	UPPER RIVER	85-89	166.20	274.60	94.99	HT-Direct
Gambia	UPPER RIVER	85-89	279.53	182.41	402.97	RW2
Gambia	UPPER RIVER	90-94	212.51	265.72	167.53	HT-Direct
Gambia	UPPER RIVER	90-94	237.08	170.44	318.85	RW2
Gambia	UPPER RIVER	95-99	131.26	157.74	108.65	HT-Direct
Gambia	UPPER RIVER	95-99	199.11	155.37	251.94	RW2
Gambia	UPPER RIVER	00-04	121.49	164.61	88.48	HT-Direct
Gambia	UPPER RIVER	00-04	166.42	129.07	213.01	RW2
Gambia	UPPER RIVER	05-09	117.91	158.34	86.74	HT-Direct
Gambia	UPPER RIVER	05-09	130.30	95.51	175.26	RW2
Gambia	UPPER RIVER	10-14	58.91	76.75	45.01	HT-Direct
Gambia	UPPER RIVER	10-14	97.83	63.05	148.30	RW2
Gambia	UPPER RIVER	15-19	72.49	21.34	216.61	RW2
Gambia	WESTERN	80-84	149.61	286.40	71.60	HT-Direct
Gambia	WESTERN	80-84	198.99	99.28	362.69	RW2
Gambia	WESTERN	85-89	123.19	192.34	76.54	HT-Direct
Gambia	WESTERN	85-89	163.43	96.63	263.97	RW2
Gambia	WESTERN	90-94	83.51	121.71	56.53	HT-Direct
Gambia	WESTERN	90-94	138.66	92.07	203.67	RW2
Gambia	WESTERN	95-99	86.41	118.10	62.61	HT-Direct
Gambia	WESTERN	95-99	119.59	87.74	160.30	RW2
Gambia	WESTERN	00-04	85.77	109.98	66.49	HT-Direct
Gambia	WESTERN	00-04	105.00	82.19	133.14	RW2
Gambia	WESTERN	05-09	57.43	71.08	46.27	HT-Direct
Gambia	WESTERN	05-09	88.24	67.94	113.79	RW2
Gambia	WESTERN	10-14	61.64	84.07	44.90	HT-Direct
Gambia	WESTERN	10-14	73.53	47.19	113.42	RW2
Gambia	WESTERN	15-19	61.51	17.46	193.58	RW2
Ghana	ALL	80-84	159.12	167.12	151.43	HT-Direct
Ghana	ALL	80-84	148.92	146.86	151.03	IHME
Ghana	ALL	80-84	162.85	154.80	171.24	RW2
Ghana	ALL	80-84	162.85	158.95	166.77	UN
Ghana	ALL	85-89	138.07	145.08	131.35	HT-Direct
Ghana	ALL	85-89	134.21	132.48	135.87	IHME
Ghana	ALL	85-89	143.76	136.58	151.25	RW2
Ghana	ALL	85-89	143.80	140.49	147.51	UN
Ghana	ALL	90-94	114.61	121.08	108.44	HT-Direct
Ghana	ALL	90-94	116.65	115.07	118.43	IHME
Ghana	ALL	90-94	120.47	113.87	127.37	RW2

Continued on next page

Country	Region	Year	Median	Lower	Upper	Method
Ghana	ALL	90-94	120.40	117.32	123.76	UN
Ghana	ALL	95-99	104.55	111.69	97.81	HT-Direct
Ghana	ALL	95-99	105.14	103.63	106.60	IHME
Ghana	ALL	95-99	108.99	101.93	116.45	RW2
Ghana	ALL	95-99	109.02	106.25	112.08	UN
Ghana	ALL	00-04	89.95	96.67	83.66	HT-Direct
Ghana	ALL	00-04	93.30	91.80	94.80	IHME
Ghana	ALL	00-04	94.18	87.62	101.19	RW2
Ghana	ALL	00-04	94.36	91.46	97.37	UN
Ghana	ALL	05-09	80.90	89.07	73.42	HT-Direct
Ghana	ALL	05-09	79.49	77.85	81.08	IHME
Ghana	ALL	05-09	82.60	75.16	90.73	RW2
Ghana	ALL	05-09	82.20	79.04	85.53	UN
Ghana	ALL	10-14	60.21	69.15	52.36	HT-Direct
Ghana	ALL	10-14	64.38	62.12	66.82	IHME
Ghana	ALL	10-14	68.64	58.60	80.17	RW2
Ghana	ALL	10-14	68.91	64.12	74.35	UN
Ghana	ASHANTI	80-84	132.46	151.34	115.61	HT-Direct
Ghana	ASHANTI	80-84	133.61	118.91	149.69	RW2
Ghana	ASHANTI	85-89	122.88	140.18	107.44	HT-Direct
Ghana	ASHANTI	85-89	122.43	111.55	133.86	RW2
Ghana	ASHANTI	90-94	84.64	98.88	72.28	HT-Direct
Ghana	ASHANTI	90-94	109.15	99.36	119.57	RW2
Ghana	ASHANTI	95-99	115.12	133.15	99.26	HT-Direct
Ghana	ASHANTI	95-99	104.72	94.82	115.65	RW2
Ghana	ASHANTI	00-04	94.63	112.05	79.68	HT-Direct
Ghana	ASHANTI	00-04	98.33	87.72	110.19	RW2
Ghana	ASHANTI	05-09	81.03	104.38	62.53	HT-Direct
Ghana	ASHANTI	05-09	91.21	77.97	106.40	RW2
Ghana	ASHANTI	10-14	74.94	99.86	55.86	HT-Direct
Ghana	ASHANTI	10-14	80.01	63.42	100.48	RW2
Ghana	ASHANTI	15-19	68.57	28.58	154.82	RW2
Ghana	BRONG AHAFO	80-84	132.03	156.16	111.14	HT-Direct
Ghana	BRONG AHAFO	80-84	139.44	121.29	159.89	RW2
Ghana	BRONG AHAFO	85-89	122.97	144.36	104.36	HT-Direct
Ghana	BRONG AHAFO	85-89	122.38	109.82	136.30	RW2
Ghana	BRONG AHAFO	90-94	90.98	112.15	73.47	HT-Direct
Ghana	BRONG AHAFO	90-94	103.67	93.05	115.57	RW2
Ghana	BRONG AHAFO	95-99	90.91	111.74	73.64	HT-Direct
Ghana	BRONG AHAFO	95-99	93.10	82.81	104.77	RW2
Ghana	BRONG AHAFO	00-04	84.62	103.83	68.69	HT-Direct
Ghana	BRONG AHAFO	00-04	81.72	71.46	93.41	RW2
Ghana	BRONG AHAFO	05-09	65.47	89.43	47.60	HT-Direct
Ghana	BRONG AHAFO	05-09	70.45	58.75	84.07	RW2
Ghana	BRONG AHAFO	10-14	44.70	61.67	32.24	HT-Direct
Ghana	BRONG AHAFO	10-14	56.90	43.99	72.81	RW2
Ghana	BRONG AHAFO	15-19	44.99	18.46	103.33	RW2
Ghana	CENTRAL	80-84	184.65	211.69	160.37	HT-Direct
Ghana	CENTRAL	80-84	184.82	164.09	207.91	RW2
Ghana	CENTRAL	85-89	154.43	177.15	134.15	HT-Direct
Ghana	CENTRAL	85-89	155.30	141.18	170.43	RW2
Ghana	CENTRAL	90-94	111.73	130.22	95.57	HT-Direct
Ghana	CENTRAL	90-94	126.57	114.43	139.52	RW2
Ghana	CENTRAL	95-99	102.00	127.72	80.98	HT-Direct
Ghana	CENTRAL	95-99	110.55	97.96	124.16	RW2
Ghana	CENTRAL	00-04	90.56	111.93	72.94	HT-Direct
Ghana	CENTRAL	00-04	95.88	83.48	110.00	RW2
Ghana	CENTRAL	05-09	89.72	118.57	67.35	HT-Direct
Ghana	CENTRAL	05-09	83.13	68.75	100.24	RW2
Ghana	CENTRAL	10-14	68.17	110.51	41.30	HT-Direct
Ghana	CENTRAL	10-14	68.04	50.71	92.22	RW2
Ghana	CENTRAL	15-19	54.70	21.51	131.46	RW2
Ghana	EASTERN	80-84	119.77	136.97	104.47	HT-Direct

Continued on next page

Country	Region	Year	Median	Lower	Upper	Method
Ghana	EASTERN	80-84	124.93	110.83	140.59	RW2
Ghana	EASTERN	85-89	101.64	119.90	85.90	HT-Direct
Ghana	EASTERN	85-89	114.66	103.77	126.69	RW2
Ghana	EASTERN	90-94	117.69	137.92	100.08	HT-Direct
Ghana	EASTERN	90-94	101.75	91.87	112.89	RW2
Ghana	EASTERN	95-99	88.75	110.14	71.18	HT-Direct
Ghana	EASTERN	95-99	94.95	84.27	106.88	RW2
Ghana	EASTERN	00-04	69.93	91.94	52.88	HT-Direct
Ghana	EASTERN	00-04	86.89	75.37	99.90	RW2
Ghana	EASTERN	05-09	69.93	89.68	54.27	HT-Direct
Ghana	EASTERN	05-09	79.20	66.45	94.04	RW2
Ghana	EASTERN	10-14	75.23	107.86	51.89	HT-Direct
Ghana	EASTERN	10-14	68.56	52.76	88.71	RW2
Ghana	EASTERN	15-19	58.26	23.54	134.20	RW2
Ghana	GREATER ACCRA	80-84	123.51	145.58	104.37	HT-Direct
Ghana	GREATER ACCRA	80-84	128.66	111.40	148.28	RW2
Ghana	GREATER ACCRA	85-89	109.33	131.46	90.54	HT-Direct
Ghana	GREATER ACCRA	85-89	108.66	96.61	122.11	RW2
Ghana	GREATER ACCRA	90-94	83.73	104.17	67.00	HT-Direct
Ghana	GREATER ACCRA	90-94	88.61	78.16	100.10	RW2
Ghana	GREATER ACCRA	95-99	73.28	94.28	56.67	HT-Direct
Ghana	GREATER ACCRA	95-99	76.87	66.42	88.68	RW2
Ghana	GREATER ACCRA	00-04	48.57	67.82	34.58	HT-Direct
Ghana	GREATER ACCRA	00-04	66.14	55.53	78.68	RW2
Ghana	GREATER ACCRA	05-09	68.10	93.02	49.50	HT-Direct
Ghana	GREATER ACCRA	05-09	57.18	45.41	71.77	RW2
Ghana	GREATER ACCRA	10-14	30.81	62.30	14.98	HT-Direct
Ghana	GREATER ACCRA	10-14	46.62	32.70	66.76	RW2
Ghana	GREATER ACCRA	15-19	37.24	14.03	95.01	RW2
Ghana	UPPER W,E & NORTHERN	80-84	248.49	270.79	227.45	HT-Direct
Ghana	UPPER W,E & NORTHERN	80-84	253.95	234.53	274.30	RW2
Ghana	UPPER W,E & NORTHERN	85-89	206.91	223.23	191.49	HT-Direct
Ghana	UPPER W,E & NORTHERN	85-89	215.39	202.40	228.85	RW2
Ghana	UPPER W,E & NORTHERN	90-94	171.04	185.98	157.07	HT-Direct
Ghana	UPPER W,E & NORTHERN	90-94	176.59	165.09	188.64	RW2
Ghana	UPPER W,E & NORTHERN	95-99	140.55	154.72	127.48	HT-Direct
Ghana	UPPER W,E & NORTHERN	95-99	152.88	141.69	164.63	RW2
Ghana	UPPER W,E & NORTHERN	00-04	126.60	140.37	114.00	HT-Direct
Ghana	UPPER W,E & NORTHERN	00-04	131.91	121.36	143.22	RW2
Ghana	UPPER W,E & NORTHERN	05-09	117.70	136.86	100.90	HT-Direct
Ghana	UPPER W,E & NORTHERN	05-09	112.93	100.60	126.79	RW2
Ghana	UPPER W,E & NORTHERN	10-14	75.79	93.35	61.31	HT-Direct
Ghana	UPPER W,E & NORTHERN	10-14	90.32	74.71	109.01	RW2
Ghana	UPPER W,E & NORTHERN	15-19	70.15	29.55	156.69	RW2
Ghana	VOLTA	80-84	155.46	177.78	135.48	HT-Direct
Ghana	VOLTA	80-84	153.91	136.94	172.42	RW2
Ghana	VOLTA	85-89	118.64	134.55	104.39	HT-Direct
Ghana	VOLTA	85-89	130.51	119.13	142.80	RW2
Ghana	VOLTA	90-94	107.58	128.13	89.99	HT-Direct
Ghana	VOLTA	90-94	107.46	96.72	119.17	RW2
Ghana	VOLTA	95-99	88.21	114.48	67.51	HT-Direct
Ghana	VOLTA	95-99	93.99	82.52	106.76	RW2
Ghana	VOLTA	00-04	80.22	104.66	61.09	HT-Direct
Ghana	VOLTA	00-04	81.15	69.24	94.69	RW2
Ghana	VOLTA	05-09	64.31	87.12	47.16	HT-Direct
Ghana	VOLTA	05-09	69.67	56.53	85.68	RW2
Ghana	VOLTA	10-14	55.65	97.16	31.26	HT-Direct
Ghana	VOLTA	10-14	56.31	41.16	77.19	RW2
Ghana	VOLTA	15-19	44.45	17.45	108.42	RW2
Ghana	WESTERN	80-84	147.64	175.97	123.19	HT-Direct
Ghana	WESTERN	80-84	155.85	134.45	179.72	RW2
Ghana	WESTERN	85-89	126.72	148.82	107.49	HT-Direct
Ghana	WESTERN	85-89	135.99	122.12	151.25	RW2

Continued on next page

Country	Region	Year	Median	Lower	Upper	Method
Ghana	WESTERN	90-94	117.50	138.05	99.66	HT-Direct
Ghana	WESTERN	90-94	114.39	102.93	127.24	RW2
Ghana	WESTERN	95-99	92.48	114.40	74.40	HT-Direct
Ghana	WESTERN	95-99	101.06	89.55	114.34	RW2
Ghana	WESTERN	00-04	93.60	117.08	74.43	HT-Direct
Ghana	WESTERN	00-04	86.59	75.05	99.85	RW2
Ghana	WESTERN	05-09	61.65	83.35	45.32	HT-Direct
Ghana	WESTERN	05-09	72.27	59.51	87.38	RW2
Ghana	WESTERN	10-14	44.56	67.36	29.23	HT-Direct
Ghana	WESTERN	10-14	56.55	41.79	75.19	RW2
Ghana	WESTERN	15-19	42.90	16.77	103.57	RW2
Guinea	ALL	80-84	271.25	286.80	256.24	HT-Direct
Guinea	ALL	80-84	266.35	262.09	270.46	IHME
Guinea	ALL	80-84	277.54	261.89	293.76	RW2
Guinea	ALL	80-84	277.32	268.92	287.27	UN
Guinea	ALL	85-89	238.17	249.94	226.79	HT-Direct
Guinea	ALL	85-89	243.38	239.92	247.15	IHME
Guinea	ALL	85-89	252.93	240.41	265.82	RW2
Guinea	ALL	85-89	253.38	246.20	260.64	UN
Guinea	ALL	90-94	220.60	229.75	211.71	HT-Direct
Guinea	ALL	90-94	218.40	215.40	221.44	IHME
Guinea	ALL	90-94	225.75	216.66	235.12	RW2
Guinea	ALL	90-94	225.40	219.01	232.15	UN
Guinea	ALL	95-99	186.23	193.90	178.80	HT-Direct
Guinea	ALL	95-99	191.12	188.41	193.91	IHME
Guinea	ALL	95-99	191.68	183.93	199.61	RW2
Guinea	ALL	95-99	191.83	185.91	197.59	UN
Guinea	ALL	00-04	157.68	165.81	149.89	HT-Direct
Guinea	ALL	00-04	164.93	162.06	167.72	IHME
Guinea	ALL	00-04	155.90	148.49	163.64	RW2
Guinea	ALL	00-04	156.13	150.88	161.29	UN
Guinea	ALL	05-09	140.23	153.27	128.13	HT-Direct
Guinea	ALL	05-09	141.98	138.53	145.73	IHME
Guinea	ALL	05-09	127.16	117.49	137.51	RW2
Guinea	ALL	05-09	126.22	120.11	132.37	UN
Guinea	ALL	10-14	106.07	120.46	93.21	HT-Direct
Guinea	ALL	10-14	120.45	116.00	125.13	IHME
Guinea	ALL	10-14	103.98	91.81	117.45	RW2
Guinea	ALL	10-14	104.64	96.21	113.99	UN
Guinea	CENTRAL GUINEA	80-84	263.88	294.91	235.03	HT-Direct
Guinea	CENTRAL GUINEA	80-84	266.06	239.46	294.61	RW2
Guinea	CENTRAL GUINEA	85-89	220.23	243.53	198.57	HT-Direct
Guinea	CENTRAL GUINEA	85-89	237.83	219.77	256.43	RW2
Guinea	CENTRAL GUINEA	90-94	206.07	223.56	189.62	HT-Direct
Guinea	CENTRAL GUINEA	90-94	213.22	199.49	227.47	RW2
Guinea	CENTRAL GUINEA	95-99	182.20	198.39	167.07	HT-Direct
Guinea	CENTRAL GUINEA	95-99	186.64	174.83	199.18	RW2
Guinea	CENTRAL GUINEA	00-04	163.77	176.99	151.35	HT-Direct
Guinea	CENTRAL GUINEA	00-04	159.14	149.08	169.95	RW2
Guinea	CENTRAL GUINEA	05-09	139.19	165.35	116.58	HT-Direct
Guinea	CENTRAL GUINEA	05-09	129.50	115.52	145.02	RW2
Guinea	CENTRAL GUINEA	10-14	98.27	131.01	73.02	HT-Direct
Guinea	CENTRAL GUINEA	10-14	101.78	81.02	126.00	RW2
Guinea	CENTRAL GUINEA	15-19	78.69	32.32	176.51	RW2
Guinea	CONAKRY	80-84	209.49	248.24	175.38	HT-Direct
Guinea	CONAKRY	80-84	215.12	183.40	250.92	RW2
Guinea	CONAKRY	85-89	162.95	190.09	139.02	HT-Direct
Guinea	CONAKRY	85-89	176.29	156.85	197.57	RW2
Guinea	CONAKRY	90-94	147.80	170.04	128.03	HT-Direct
Guinea	CONAKRY	90-94	144.29	129.70	160.50	RW2
Guinea	CONAKRY	95-99	109.77	126.99	94.63	HT-Direct
Guinea	CONAKRY	95-99	112.83	100.18	126.48	RW2
Guinea	CONAKRY	00-04	63.38	86.06	46.38	HT-Direct

Continued on next page

Country	Region	Year	Median	Lower	Upper	Method
Guinea	CONAKRY	00-04	87.36	73.91	102.42	RW2
Guinea	CONAKRY	05-09	88.62	123.66	62.80	HT-Direct
Guinea	CONAKRY	05-09	68.52	54.75	85.52	RW2
Guinea	CONAKRY	10-14	56.44	100.38	31.07	HT-Direct
Guinea	CONAKRY	10-14	51.52	34.64	77.16	RW2
Guinea	CONAKRY	15-19	38.09	12.90	108.01	RW2
Guinea	FOREST GUINEA	80-84	275.95	305.42	248.31	HT-Direct
Guinea	FOREST GUINEA	80-84	288.06	259.96	317.83	RW2
Guinea	FOREST GUINEA	85-89	271.89	298.40	246.92	HT-Direct
Guinea	FOREST GUINEA	85-89	290.70	269.42	313.23	RW2
Guinea	FOREST GUINEA	90-94	270.30	292.17	249.49	HT-Direct
Guinea	FOREST GUINEA	90-94	270.41	252.94	289.05	RW2
Guinea	FOREST GUINEA	95-99	211.77	229.46	195.10	HT-Direct
Guinea	FOREST GUINEA	95-99	217.86	203.69	233.09	RW2
Guinea	FOREST GUINEA	00-04	167.40	185.54	150.70	HT-Direct
Guinea	FOREST GUINEA	00-04	162.06	148.87	175.98	RW2
Guinea	FOREST GUINEA	05-09	112.01	139.83	89.15	HT-Direct
Guinea	FOREST GUINEA	05-09	119.51	103.97	136.41	RW2
Guinea	FOREST GUINEA	10-14	101.43	128.31	79.66	HT-Direct
Guinea	FOREST GUINEA	10-14	93.99	76.40	114.98	RW2
Guinea	FOREST GUINEA	15-19	75.30	31.31	171.68	RW2
Guinea	LOWER GUINEA	80-84	272.29	305.73	241.25	HT-Direct
Guinea	LOWER GUINEA	80-84	276.23	248.00	307.04	RW2
Guinea	LOWER GUINEA	85-89	229.17	250.90	208.79	HT-Direct
Guinea	LOWER GUINEA	85-89	243.17	225.06	261.86	RW2
Guinea	LOWER GUINEA	90-94	206.08	224.88	188.47	HT-Direct
Guinea	LOWER GUINEA	90-94	216.37	201.61	231.60	RW2
Guinea	LOWER GUINEA	95-99	184.50	201.52	168.62	HT-Direct
Guinea	LOWER GUINEA	95-99	188.51	175.72	202.25	RW2
Guinea	LOWER GUINEA	00-04	164.27	181.69	148.21	HT-Direct
Guinea	LOWER GUINEA	00-04	157.21	145.01	170.62	RW2
Guinea	LOWER GUINEA	05-09	135.99	167.08	109.93	HT-Direct
Guinea	LOWER GUINEA	05-09	121.07	105.83	138.14	RW2
Guinea	LOWER GUINEA	10-14	72.30	102.61	50.44	HT-Direct
Guinea	LOWER GUINEA	10-14	85.64	64.66	110.86	RW2
Guinea	LOWER GUINEA	15-19	58.40	22.40	140.62	RW2
Guinea	UPPER GUINEA	80-84	308.94	344.20	275.77	HT-Direct
Guinea	UPPER GUINEA	80-84	314.90	284.19	347.63	RW2
Guinea	UPPER GUINEA	85-89	277.30	303.72	252.34	HT-Direct
Guinea	UPPER GUINEA	85-89	286.40	266.33	307.90	RW2
Guinea	UPPER GUINEA	90-94	245.07	264.09	226.99	HT-Direct
Guinea	UPPER GUINEA	90-94	251.76	236.81	267.15	RW2
Guinea	UPPER GUINEA	95-99	204.03	218.50	190.29	HT-Direct
Guinea	UPPER GUINEA	95-99	212.02	199.72	224.68	RW2
Guinea	UPPER GUINEA	00-04	180.16	197.54	163.99	HT-Direct
Guinea	UPPER GUINEA	00-04	183.84	171.19	197.10	RW2
Guinea	UPPER GUINEA	05-09	194.77	222.05	170.11	HT-Direct
Guinea	UPPER GUINEA	05-09	169.26	153.44	186.47	RW2
Guinea	UPPER GUINEA	10-14	174.31	212.79	141.55	HT-Direct
Guinea	UPPER GUINEA	10-14	163.51	137.31	194.68	RW2
Guinea	UPPER GUINEA	15-19	159.61	72.09	318.82	RW2
Kenya	ALL	80-84	100.25	107.65	93.30	HT-Direct
Kenya	ALL	80-84	101.94	100.33	103.62	IHME
Kenya	ALL	80-84	102.59	95.32	110.36	RW2
Kenya	ALL	80-84	102.57	99.81	105.46	UN
Kenya	ALL	85-89	91.17	96.80	85.84	HT-Direct
Kenya	ALL	85-89	93.67	92.16	95.14	IHME
Kenya	ALL	85-89	97.40	91.44	103.65	RW2
Kenya	ALL	85-89	97.47	94.70	100.12	UN
Kenya	ALL	90-94	102.12	107.95	96.58	HT-Direct
Kenya	ALL	90-94	93.48	91.93	95.02	IHME
Kenya	ALL	90-94	108.35	102.29	114.75	RW2
Kenya	ALL	90-94	108.12	105.07	111.37	UN

Continued on next page

Country	Region	Year	Median	Lower	Upper	Method
Kenya	ALL	95-99	100.52	106.10	95.20	HT-Direct
Kenya	ALL	95-99	93.71	92.03	95.27	IHME
Kenya	ALL	95-99	113.65	106.96	120.65	RW2
Kenya	ALL	95-99	113.99	110.54	117.42	UN
Kenya	ALL	00-04	88.05	93.37	83.01	HT-Direct
Kenya	ALL	00-04	81.88	80.14	83.73	IHME
Kenya	ALL	00-04	100.00	93.65	106.79	RW2
Kenya	ALL	00-04	99.71	96.14	103.53	UN
Kenya	ALL	05-09	62.02	66.78	57.58	HT-Direct
Kenya	ALL	05-09	63.75	62.16	65.45	IHME
Kenya	ALL	05-09	75.16	68.68	82.16	RW2
Kenya	ALL	05-09	75.42	71.54	79.43	UN
Kenya	ALL	10-14	52.20	57.09	47.71	HT-Direct
Kenya	ALL	10-14	52.82	50.68	54.98	IHME
Kenya	ALL	10-14	56.44	51.21	62.13	RW2
Kenya	ALL	10-14	56.31	51.59	61.06	UN
Kenya	CENTRAL	80-84	48.15	61.80	37.40	HT-Direct
Kenya	CENTRAL	80-84	49.10	39.56	60.98	RW2
Kenya	CENTRAL	85-89	42.31	53.90	33.13	HT-Direct
Kenya	CENTRAL	85-89	44.01	37.59	51.41	RW2
Kenya	CENTRAL	90-94	49.57	60.73	40.38	HT-Direct
Kenya	CENTRAL	90-94	53.85	46.56	62.17	RW2
Kenya	CENTRAL	95-99	56.26	71.49	44.12	HT-Direct
Kenya	CENTRAL	95-99	68.02	57.99	79.43	RW2
Kenya	CENTRAL	00-04	60.07	76.93	46.71	HT-Direct
Kenya	CENTRAL	00-04	67.01	56.52	79.36	RW2
Kenya	CENTRAL	05-09	52.10	68.95	39.20	HT-Direct
Kenya	CENTRAL	05-09	56.51	45.88	69.35	RW2
Kenya	CENTRAL	10-14	40.79	57.56	28.75	HT-Direct
Kenya	CENTRAL	10-14	47.71	35.53	64.07	RW2
Kenya	CENTRAL	15-19	40.82	14.93	106.86	RW2
Kenya	COAST	80-84	156.99	181.03	135.62	HT-Direct
Kenya	COAST	80-84	154.42	135.21	176.20	RW2
Kenya	COAST	85-89	105.60	120.65	92.24	HT-Direct
Kenya	COAST	85-89	116.38	104.89	128.81	RW2
Kenya	COAST	90-94	107.92	122.93	94.54	HT-Direct
Kenya	COAST	90-94	120.41	108.61	133.04	RW2
Kenya	COAST	95-99	137.79	163.20	115.79	HT-Direct
Kenya	COAST	95-99	128.56	113.73	145.22	RW2
Kenya	COAST	00-04	80.76	97.76	66.49	HT-Direct
Kenya	COAST	00-04	106.07	92.52	121.30	RW2
Kenya	COAST	05-09	66.19	79.86	54.73	HT-Direct
Kenya	COAST	05-09	76.81	65.06	90.40	RW2
Kenya	COAST	10-14	54.96	74.98	40.06	HT-Direct
Kenya	COAST	10-14	56.99	43.99	74.00	RW2
Kenya	COAST	15-19	42.81	16.01	110.65	RW2
Kenya	EASTERN	80-84	75.07	88.74	63.37	HT-Direct
Kenya	EASTERN	80-84	76.36	65.74	88.73	RW2
Kenya	EASTERN	85-89	62.33	73.67	52.64	HT-Direct
Kenya	EASTERN	85-89	64.77	57.59	72.76	RW2
Kenya	EASTERN	90-94	64.04	74.95	54.62	HT-Direct
Kenya	EASTERN	90-94	75.33	67.43	84.03	RW2
Kenya	EASTERN	95-99	85.41	99.59	73.09	HT-Direct
Kenya	EASTERN	95-99	89.90	79.98	100.96	RW2
Kenya	EASTERN	00-04	76.93	91.16	64.77	HT-Direct
Kenya	EASTERN	00-04	81.91	71.94	93.13	RW2
Kenya	EASTERN	05-09	45.79	58.05	36.01	HT-Direct
Kenya	EASTERN	05-09	63.68	53.82	75.12	RW2
Kenya	EASTERN	10-14	46.60	60.62	35.69	HT-Direct
Kenya	EASTERN	10-14	50.32	39.94	63.33	RW2
Kenya	EASTERN	15-19	40.38	15.48	100.64	RW2
Kenya	NAIROBI	80-84	66.08	97.47	44.31	HT-Direct
Kenya	NAIROBI	80-84	68.51	50.57	92.56	RW2

Continued on next page

Country	Region	Year	Median	Lower	Upper	Method
Kenya	NAIROBI	85-89	60.97	83.52	44.22	HT-Direct
Kenya	NAIROBI	85-89	63.12	51.27	77.58	RW2
Kenya	NAIROBI	90-94	72.91	97.05	54.41	HT-Direct
Kenya	NAIROBI	90-94	78.40	65.23	93.90	RW2
Kenya	NAIROBI	95-99	102.28	140.88	73.35	HT-Direct
Kenya	NAIROBI	95-99	99.07	81.91	119.29	RW2
Kenya	NAIROBI	00-04	72.78	97.93	53.70	HT-Direct
Kenya	NAIROBI	00-04	97.52	79.84	118.40	RW2
Kenya	NAIROBI	05-09	71.39	102.08	49.42	HT-Direct
Kenya	NAIROBI	05-09	83.28	66.06	104.46	RW2
Kenya	NAIROBI	10-14	70.98	99.76	50.04	HT-Direct
Kenya	NAIROBI	10-14	72.15	52.98	97.66	RW2
Kenya	NAIROBI	15-19	62.98	23.32	160.58	RW2
Kenya	NORTHEASTERN	80-84	219.17	312.84	147.53	HT-Direct
Kenya	NORTHEASTERN	80-84	209.80	157.73	271.51	RW2
Kenya	NORTHEASTERN	85-89	117.52	150.44	91.04	HT-Direct
Kenya	NORTHEASTERN	85-89	155.88	130.77	184.21	RW2
Kenya	NORTHEASTERN	90-94	169.72	200.82	142.57	HT-Direct
Kenya	NORTHEASTERN	90-94	153.08	133.77	175.08	RW2
Kenya	NORTHEASTERN	95-99	133.24	162.55	108.54	HT-Direct
Kenya	NORTHEASTERN	95-99	146.68	127.01	169.07	RW2
Kenya	NORTHEASTERN	00-04	77.82	97.41	61.90	HT-Direct
Kenya	NORTHEASTERN	00-04	106.89	90.18	125.51	RW2
Kenya	NORTHEASTERN	05-09	56.48	74.33	42.71	HT-Direct
Kenya	NORTHEASTERN	05-09	69.20	56.38	84.57	RW2
Kenya	NORTHEASTERN	10-14	47.82	65.18	34.90	HT-Direct
Kenya	NORTHEASTERN	10-14	46.84	35.28	62.26	RW2
Kenya	NORTHEASTERN	15-19	32.16	11.68	87.41	RW2
Kenya	NYANZA	80-84	151.29	173.65	131.35	HT-Direct
Kenya	NYANZA	80-84	163.28	142.92	185.21	RW2
Kenya	NYANZA	85-89	169.65	187.37	153.30	HT-Direct
Kenya	NYANZA	85-89	174.04	159.89	189.36	RW2
Kenya	NYANZA	90-94	203.88	226.12	183.32	HT-Direct
Kenya	NYANZA	90-94	217.41	199.74	236.37	RW2
Kenya	NYANZA	95-99	216.33	244.19	190.84	HT-Direct
Kenya	NYANZA	95-99	250.29	227.06	275.19	RW2
Kenya	NYANZA	00-04	205.36	236.89	177.06	HT-Direct
Kenya	NYANZA	00-04	215.27	191.98	241.26	RW2
Kenya	NYANZA	05-09	116.34	135.57	99.53	HT-Direct
Kenya	NYANZA	05-09	139.38	121.77	159.33	RW2
Kenya	NYANZA	10-14	69.00	83.34	56.97	HT-Direct
Kenya	NYANZA	10-14	79.02	65.33	95.20	RW2
Kenya	NYANZA	15-19	42.77	16.47	106.57	RW2
Kenya	RIFT VALLEY	80-84	79.34	93.04	67.50	HT-Direct
Kenya	RIFT VALLEY	80-84	79.03	68.62	90.88	RW2
Kenya	RIFT VALLEY	85-89	61.90	71.65	53.40	HT-Direct
Kenya	RIFT VALLEY	85-89	67.94	61.09	75.30	RW2
Kenya	RIFT VALLEY	90-94	75.98	86.61	66.56	HT-Direct
Kenya	RIFT VALLEY	90-94	77.85	70.61	85.62	RW2
Kenya	RIFT VALLEY	95-99	73.32	85.01	63.13	HT-Direct
Kenya	RIFT VALLEY	95-99	90.23	80.93	100.31	RW2
Kenya	RIFT VALLEY	00-04	71.86	82.30	62.65	HT-Direct
Kenya	RIFT VALLEY	00-04	81.50	73.09	90.70	RW2
Kenya	RIFT VALLEY	05-09	49.53	57.60	42.54	HT-Direct
Kenya	RIFT VALLEY	05-09	62.18	54.74	70.53	RW2
Kenya	RIFT VALLEY	10-14	45.68	53.97	38.61	HT-Direct
Kenya	RIFT VALLEY	10-14	47.21	40.30	55.36	RW2
Kenya	RIFT VALLEY	15-19	35.99	14.43	88.01	RW2
Kenya	WESTERN	80-84	119.11	139.54	101.32	HT-Direct
Kenya	WESTERN	80-84	124.21	107.17	143.54	RW2
Kenya	WESTERN	85-89	118.37	135.87	102.85	HT-Direct
Kenya	WESTERN	85-89	124.33	111.99	138.08	RW2
Kenya	WESTERN	90-94	145.57	163.39	129.40	HT-Direct

Continued on next page

Country	Region	Year	Median	Lower	Upper	Method
Kenya	WESTERN	90-94	152.43	138.58	167.49	RW2
Kenya	WESTERN	95-99	143.68	166.33	123.66	HT-Direct
Kenya	WESTERN	95-99	177.16	158.68	197.25	RW2
Kenya	WESTERN	00-04	148.67	171.88	128.11	HT-Direct
Kenya	WESTERN	00-04	155.27	137.37	175.84	RW2
Kenya	WESTERN	05-09	90.07	112.17	71.97	HT-Direct
Kenya	WESTERN	05-09	103.70	88.49	121.27	RW2
Kenya	WESTERN	10-14	53.89	66.20	43.76	HT-Direct
Kenya	WESTERN	10-14	61.85	50.28	75.48	RW2
Kenya	WESTERN	15-19	35.84	13.69	89.26	RW2
Lesotho	ALL	80-84	90.78	107.31	76.58	HT-Direct
Lesotho	ALL	80-84	98.56	96.09	101.23	IHME
Lesotho	ALL	80-84	109.45	88.41	134.86	RW2
Lesotho	ALL	80-84	109.69	105.00	115.30	UN
Lesotho	ALL	85-89	78.72	88.97	69.57	HT-Direct
Lesotho	ALL	85-89	94.41	92.09	96.83	IHME
Lesotho	ALL	85-89	91.81	80.26	104.69	RW2
Lesotho	ALL	85-89	92.68	88.60	96.91	UN
Lesotho	ALL	90-94	89.11	98.70	80.37	HT-Direct
Lesotho	ALL	90-94	90.80	88.68	92.82	IHME
Lesotho	ALL	90-94	91.73	83.05	101.22	RW2
Lesotho	ALL	90-94	90.01	86.45	93.80	UN
Lesotho	ALL	95-99	87.20	97.23	78.12	HT-Direct
Lesotho	ALL	95-99	97.19	95.08	99.45	IHME
Lesotho	ALL	95-99	104.95	92.86	118.22	RW2
Lesotho	ALL	95-99	107.25	103.63	111.67	UN
Lesotho	ALL	00-04	115.28	125.24	106.02	HT-Direct
Lesotho	ALL	00-04	112.34	109.67	115.04	IHME
Lesotho	ALL	00-04	121.07	111.35	131.59	RW2
Lesotho	ALL	00-04	120.24	115.87	124.57	UN
Lesotho	ALL	05-09	113.15	124.93	102.34	HT-Direct
Lesotho	ALL	05-09	116.61	112.84	120.42	IHME
Lesotho	ALL	05-09	117.67	106.51	129.85	RW2
Lesotho	ALL	05-09	117.97	113.03	123.21	UN
Lesotho	ALL	10-14	86.94	101.03	74.65	HT-Direct
Lesotho	ALL	10-14	99.72	94.23	106.12	IHME
Lesotho	ALL	10-14	95.68	81.15	112.32	RW2
Lesotho	ALL	10-14	95.61	88.80	103.23	UN
Lesotho	BEREA	80-84	156.27	216.69	110.33	HT-Direct
Lesotho	BEREA	80-84	153.18	110.39	208.38	RW2
Lesotho	BEREA	85-89	78.06	112.50	53.53	HT-Direct
Lesotho	BEREA	85-89	113.26	88.62	143.50	RW2
Lesotho	BEREA	90-94	102.41	135.41	76.74	HT-Direct
Lesotho	BEREA	90-94	103.95	86.38	124.33	RW2
Lesotho	BEREA	95-99	84.42	114.54	61.67	HT-Direct
Lesotho	BEREA	95-99	107.93	90.61	127.98	RW2
Lesotho	BEREA	00-04	106.50	133.82	84.21	HT-Direct
Lesotho	BEREA	00-04	109.00	93.36	127.13	RW2
Lesotho	BEREA	05-09	92.54	122.03	69.61	HT-Direct
Lesotho	BEREA	05-09	100.89	83.03	121.89	RW2
Lesotho	BEREA	10-14	75.48	111.98	50.20	HT-Direct
Lesotho	BEREA	10-14	75.59	56.37	100.65	RW2
Lesotho	BEREA	15-19	52.49	19.26	132.79	RW2
Lesotho	BUTHA-BUTHE	80-84	57.90	104.19	31.46	HT-Direct
Lesotho	BUTHA-BUTHE	80-84	91.15	59.08	138.08	RW2
Lesotho	BUTHA-BUTHE	85-89	70.92	105.47	47.09	HT-Direct
Lesotho	BUTHA-BUTHE	85-89	73.80	55.05	98.40	RW2
Lesotho	BUTHA-BUTHE	90-94	71.81	96.96	52.81	HT-Direct
Lesotho	BUTHA-BUTHE	90-94	74.65	60.29	91.88	RW2
Lesotho	BUTHA-BUTHE	95-99	75.18	110.59	50.46	HT-Direct
Lesotho	BUTHA-BUTHE	95-99	84.79	69.42	103.23	RW2
Lesotho	BUTHA-BUTHE	00-04	84.13	110.09	63.86	HT-Direct
Lesotho	BUTHA-BUTHE	00-04	93.41	78.32	111.15	RW2

Continued on next page

Country	Region	Year	Median	Lower	Upper	Method
Lesotho	BUTHA-BUTHE	05-09	90.93	121.32	67.57	HT-Direct
Lesotho	BUTHA-BUTHE	05-09	94.77	76.29	117.09	RW2
Lesotho	BUTHA-BUTHE	10-14	70.91	116.07	42.48	HT-Direct
Lesotho	BUTHA-BUTHE	10-14	78.31	55.74	109.18	RW2
Lesotho	BUTHA-BUTHE	15-19	60.50	21.31	156.43	RW2
Lesotho	LERIBE	80-84	99.07	168.98	56.13	HT-Direct
Lesotho	LERIBE	80-84	102.20	70.07	146.76	RW2
Lesotho	LERIBE	85-89	86.06	117.01	62.72	HT-Direct
Lesotho	LERIBE	85-89	85.57	66.59	109.38	RW2
Lesotho	LERIBE	90-94	73.28	97.22	54.87	HT-Direct
Lesotho	LERIBE	90-94	89.64	74.32	107.65	RW2
Lesotho	LERIBE	95-99	86.06	119.76	61.19	HT-Direct
Lesotho	LERIBE	95-99	106.45	89.35	126.16	RW2
Lesotho	LERIBE	00-04	121.32	152.78	95.62	HT-Direct
Lesotho	LERIBE	00-04	122.65	105.59	141.72	RW2
Lesotho	LERIBE	05-09	130.16	164.10	102.38	HT-Direct
Lesotho	LERIBE	05-09	129.72	108.88	154.00	RW2
Lesotho	LERIBE	10-14	97.98	138.70	68.27	HT-Direct
Lesotho	LERIBE	10-14	112.14	86.24	145.19	RW2
Lesotho	LERIBE	15-19	90.67	34.51	213.69	RW2
Lesotho	MAFETENG	80-84	59.65	107.92	32.19	HT-Direct
Lesotho	MAFETENG	80-84	97.74	64.03	147.47	RW2
Lesotho	MAFETENG	85-89	90.78	128.73	63.21	HT-Direct
Lesotho	MAFETENG	85-89	81.04	60.70	107.68	RW2
Lesotho	MAFETENG	90-94	78.96	110.97	55.60	HT-Direct
Lesotho	MAFETENG	90-94	83.98	67.45	104.10	RW2
Lesotho	MAFETENG	95-99	68.47	103.28	44.80	HT-Direct
Lesotho	MAFETENG	95-99	98.47	80.59	119.35	RW2
Lesotho	MAFETENG	00-04	96.49	125.32	73.74	HT-Direct
Lesotho	MAFETENG	00-04	112.44	95.13	132.30	RW2
Lesotho	MAFETENG	05-09	121.57	151.84	96.64	HT-Direct
Lesotho	MAFETENG	05-09	117.97	97.54	141.97	RW2
Lesotho	MAFETENG	10-14	89.59	155.58	49.93	HT-Direct
Lesotho	MAFETENG	10-14	100.54	72.73	137.91	RW2
Lesotho	MAFETENG	15-19	80.22	28.23	200.48	RW2
Lesotho	MASERU	80-84	50.39	82.60	30.32	HT-Direct
Lesotho	MASERU	80-84	76.38	52.27	109.44	RW2
Lesotho	MASERU	85-89	52.49	77.12	35.42	HT-Direct
Lesotho	MASERU	85-89	68.27	52.47	88.01	RW2
Lesotho	MASERU	90-94	81.30	105.52	62.25	HT-Direct
Lesotho	MASERU	90-94	76.65	63.90	91.68	RW2
Lesotho	MASERU	95-99	74.79	96.62	57.57	HT-Direct
Lesotho	MASERU	95-99	96.44	81.99	113.17	RW2
Lesotho	MASERU	00-04	119.31	144.32	98.14	HT-Direct
Lesotho	MASERU	00-04	116.47	101.39	133.61	RW2
Lesotho	MASERU	05-09	111.96	147.47	84.16	HT-Direct
Lesotho	MASERU	05-09	127.45	106.63	151.96	RW2
Lesotho	MASERU	10-14	96.50	135.63	67.78	HT-Direct
Lesotho	MASERU	10-14	113.38	86.83	146.47	RW2
Lesotho	MASERU	15-19	94.18	35.71	221.44	RW2
Lesotho	MOHALE'S HOEK	80-84	126.37	190.23	81.78	HT-Direct
Lesotho	MOHALE'S HOEK	80-84	132.29	94.49	181.80	RW2
Lesotho	MOHALE'S HOEK	85-89	96.17	128.87	71.09	HT-Direct
Lesotho	MOHALE'S HOEK	85-89	107.06	84.85	134.33	RW2
Lesotho	MOHALE'S HOEK	90-94	87.98	114.58	67.09	HT-Direct
Lesotho	MOHALE'S HOEK	90-94	108.00	91.02	127.84	RW2
Lesotho	MOHALE'S HOEK	95-99	120.89	157.57	91.82	HT-Direct
Lesotho	MOHALE'S HOEK	95-99	122.92	104.65	144.21	RW2
Lesotho	MOHALE'S HOEK	00-04	137.37	173.17	108.00	HT-Direct
Lesotho	MOHALE'S HOEK	00-04	135.22	116.74	156.12	RW2
Lesotho	MOHALE'S HOEK	05-09	133.38	169.68	103.88	HT-Direct
Lesotho	MOHALE'S HOEK	05-09	135.86	113.37	162.11	RW2
Lesotho	MOHALE'S HOEK	10-14	89.62	134.68	58.61	HT-Direct

Continued on next page

Country	Region	Year	Median	Lower	Upper	Method
Lesotho	MOHALE'S HOEK	10-14	110.85	83.63	145.34	RW2
Lesotho	MOHALE'S HOEK	15-19	84.01	31.38	201.11	RW2
Lesotho	MOKHOTLONG	80-84	104.16	180.34	57.89	HT-Direct
Lesotho	MOKHOTLONG	80-84	148.78	101.63	210.85	RW2
Lesotho	MOKHOTLONG	85-89	87.20	124.00	60.56	HT-Direct
Lesotho	MOKHOTLONG	85-89	115.33	89.37	146.62	RW2
Lesotho	MOKHOTLONG	90-94	123.46	154.42	97.98	HT-Direct
Lesotho	MOKHOTLONG	90-94	110.26	92.72	130.62	RW2
Lesotho	MOKHOTLONG	95-99	95.33	128.94	69.78	HT-Direct
Lesotho	MOKHOTLONG	95-99	117.63	99.48	138.82	RW2
Lesotho	MOKHOTLONG	00-04	108.55	134.87	86.86	HT-Direct
Lesotho	MOKHOTLONG	00-04	121.69	104.87	140.83	RW2
Lesotho	MOKHOTLONG	05-09	103.77	137.66	77.47	HT-Direct
Lesotho	MOKHOTLONG	05-09	116.21	96.26	139.59	RW2
Lesotho	MOKHOTLONG	10-14	96.92	136.33	68.00	HT-Direct
Lesotho	MOKHOTLONG	10-14	90.82	68.76	119.13	RW2
Lesotho	MOKHOTLONG	15-19	65.98	24.51	162.84	RW2
Lesotho	QASHA'S NEK	80-84	154.47	243.34	94.02	HT-Direct
Lesotho	QASHA'S NEK	80-84	156.29	109.21	220.38	RW2
Lesotho	QASHA'S NEK	85-89	111.82	155.81	79.09	HT-Direct
Lesotho	QASHA'S NEK	85-89	118.77	92.62	150.84	RW2
Lesotho	QASHA'S NEK	90-94	106.19	134.42	83.32	HT-Direct
Lesotho	QASHA'S NEK	90-94	111.92	93.96	132.73	RW2
Lesotho	QASHA'S NEK	95-99	93.77	122.96	70.95	HT-Direct
Lesotho	QASHA'S NEK	95-99	119.49	100.94	140.96	RW2
Lesotho	QASHA'S NEK	00-04	103.49	136.54	77.73	HT-Direct
Lesotho	QASHA'S NEK	00-04	124.69	105.91	146.14	RW2
Lesotho	QASHA'S NEK	05-09	125.92	162.66	96.53	HT-Direct
Lesotho	QASHA'S NEK	05-09	120.43	98.49	146.36	RW2
Lesotho	QASHA'S NEK	10-14	97.20	154.55	59.64	HT-Direct
Lesotho	QASHA'S NEK	10-14	94.62	69.07	128.79	RW2
Lesotho	QASHA'S NEK	15-19	69.23	24.92	173.64	RW2
Lesotho	QUTHING	80-84	122.26	180.04	81.18	HT-Direct
Lesotho	QUTHING	80-84	150.26	105.49	209.39	RW2
Lesotho	QUTHING	85-89	76.67	113.37	51.17	HT-Direct
Lesotho	QUTHING	85-89	117.51	91.51	149.57	RW2
Lesotho	QUTHING	90-94	123.26	155.31	97.07	HT-Direct
Lesotho	QUTHING	90-94	113.68	95.17	135.25	RW2
Lesotho	QUTHING	95-99	110.08	148.19	80.84	HT-Direct
Lesotho	QUTHING	95-99	122.71	103.01	145.60	RW2
Lesotho	QUTHING	00-04	108.16	141.09	82.19	HT-Direct
Lesotho	QUTHING	00-04	128.08	108.45	150.82	RW2
Lesotho	QUTHING	05-09	125.70	163.64	95.56	HT-Direct
Lesotho	QUTHING	05-09	122.71	99.82	150.12	RW2
Lesotho	QUTHING	10-14	77.80	129.69	45.58	HT-Direct
Lesotho	QUTHING	10-14	95.25	68.05	131.58	RW2
Lesotho	QUTHING	15-19	68.33	24.02	174.77	RW2
Lesotho	THABA-TSEKA	80-84	134.26	218.11	79.38	HT-Direct
Lesotho	THABA-TSEKA	80-84	166.46	115.93	233.67	RW2
Lesotho	THABA-TSEKA	85-89	95.81	135.18	67.02	HT-Direct
Lesotho	THABA-TSEKA	85-89	123.94	95.59	159.51	RW2
Lesotho	THABA-TSEKA	90-94	105.50	142.43	77.29	HT-Direct
Lesotho	THABA-TSEKA	90-94	113.84	94.30	136.96	RW2
Lesotho	THABA-TSEKA	95-99	108.90	140.64	83.62	HT-Direct
Lesotho	THABA-TSEKA	95-99	117.34	99.73	137.79	RW2
Lesotho	THABA-TSEKA	00-04	121.14	148.94	97.92	HT-Direct
Lesotho	THABA-TSEKA	00-04	116.76	101.10	134.66	RW2
Lesotho	THABA-TSEKA	05-09	94.25	121.04	72.90	HT-Direct
Lesotho	THABA-TSEKA	05-09	105.85	87.15	127.73	RW2
Lesotho	THABA-TSEKA	10-14	55.74	105.03	28.84	HT-Direct
Lesotho	THABA-TSEKA	10-14	77.37	56.11	105.10	RW2
Lesotho	THABA-TSEKA	15-19	52.63	18.87	132.93	RW2
Liberia	ALL	80-84	227.15	257.62	199.32	HT-Direct

Continued on next page

Country	Region	Year	Median	Lower	Upper	Method
Liberia	ALL	80-84	236.32	232.47	239.96	IHME
Liberia	ALL	80-84	237.19	207.24	270.04	RW2
Liberia	ALL	80-84	236.87	227.22	245.59	UN
Liberia	ALL	85-89	231.40	251.73	212.25	HT-Direct
Liberia	ALL	85-89	227.05	223.85	230.33	IHME
Liberia	ALL	85-89	242.19	222.41	262.82	RW2
Liberia	ALL	85-89	242.85	234.47	252.21	UN
Liberia	ALL	90-94	264.15	282.64	246.45	HT-Direct
Liberia	ALL	90-94	220.60	216.62	224.48	IHME
Liberia	ALL	90-94	254.77	238.92	271.47	RW2
Liberia	ALL	90-94	253.53	244.13	264.03	UN
Liberia	ALL	95-99	206.41	220.49	193.00	HT-Direct
Liberia	ALL	95-99	195.34	192.46	198.27	IHME
Liberia	ALL	95-99	214.35	200.55	228.66	RW2
Liberia	ALL	95-99	215.18	207.46	223.06	UN
Liberia	ALL	00-04	152.33	163.17	142.09	HT-Direct
Liberia	ALL	00-04	146.95	144.51	149.52	IHME
Liberia	ALL	00-04	157.99	147.43	169.26	RW2
Liberia	ALL	00-04	157.48	151.80	163.44	UN
Liberia	ALL	05-09	100.25	111.64	89.90	HT-Direct
Liberia	ALL	05-09	106.28	104.22	108.45	IHME
Liberia	ALL	05-09	107.67	96.48	119.83	RW2
Liberia	ALL	05-09	108.36	103.63	113.29	UN
Liberia	ALL	10-14	92.88	107.44	80.12	HT-Direct
Liberia	ALL	10-14	83.09	80.24	85.99	IHME
Liberia	ALL	10-14	81.53	71.56	92.67	RW2
Liberia	ALL	10-14	80.99	75.01	87.22	UN
Liberia	NORTH CENTRAL	80-84	232.00	291.44	181.58	HT-Direct
Liberia	NORTH CENTRAL	80-84	230.35	191.17	275.51	RW2
Liberia	NORTH CENTRAL	85-89	227.02	266.10	192.18	HT-Direct
Liberia	NORTH CENTRAL	85-89	238.25	211.52	266.82	RW2
Liberia	NORTH CENTRAL	90-94	246.92	280.07	216.51	HT-Direct
Liberia	NORTH CENTRAL	90-94	252.52	231.31	274.87	RW2
Liberia	NORTH CENTRAL	95-99	207.51	233.97	183.32	HT-Direct
Liberia	NORTH CENTRAL	95-99	205.36	188.49	223.55	RW2
Liberia	NORTH CENTRAL	00-04	139.79	157.90	123.44	HT-Direct
Liberia	NORTH CENTRAL	00-04	144.84	132.22	158.69	RW2
Liberia	NORTH CENTRAL	05-09	96.03	112.78	81.53	HT-Direct
Liberia	NORTH CENTRAL	05-09	101.24	89.51	114.33	RW2
Liberia	NORTH CENTRAL	10-14	75.31	102.44	54.92	HT-Direct
Liberia	NORTH CENTRAL	10-14	70.03	57.15	85.12	RW2
Liberia	NORTH CENTRAL	15-19	47.77	19.01	114.85	RW2
Liberia	NORTH WESTERN	80-84	213.26	300.87	145.84	HT-Direct
Liberia	NORTH WESTERN	80-84	262.90	206.31	324.32	RW2
Liberia	NORTH WESTERN	85-89	273.61	323.02	229.21	HT-Direct
Liberia	NORTH WESTERN	85-89	284.82	249.31	322.32	RW2
Liberia	NORTH WESTERN	90-94	328.52	380.27	280.61	HT-Direct
Liberia	NORTH WESTERN	90-94	311.89	283.41	342.48	RW2
Liberia	NORTH WESTERN	95-99	271.35	308.30	237.31	HT-Direct
Liberia	NORTH WESTERN	95-99	262.51	239.34	287.91	RW2
Liberia	NORTH WESTERN	00-04	172.95	199.87	148.99	HT-Direct
Liberia	NORTH WESTERN	00-04	190.17	172.02	210.08	RW2
Liberia	NORTH WESTERN	05-09	125.63	151.83	103.40	HT-Direct
Liberia	NORTH WESTERN	05-09	135.92	119.87	153.85	RW2
Liberia	NORTH WESTERN	10-14	110.16	134.53	89.74	HT-Direct
Liberia	NORTH WESTERN	10-14	96.36	82.05	112.70	RW2
Liberia	NORTH WESTERN	15-19	67.29	27.42	154.38	RW2
Liberia	SOUTH CENTRAL	80-84	242.85	286.82	203.70	HT-Direct
Liberia	SOUTH CENTRAL	80-84	245.77	211.24	284.01	RW2
Liberia	SOUTH CENTRAL	85-89	240.36	272.63	210.81	HT-Direct
Liberia	SOUTH CENTRAL	85-89	253.02	229.26	278.46	RW2
Liberia	SOUTH CENTRAL	90-94	275.74	305.83	247.56	HT-Direct
Liberia	SOUTH CENTRAL	90-94	267.05	247.24	287.64	RW2

Continued on next page

Country	Region	Year	Median	Lower	Upper	Method
Liberia	SOUTH CENTRAL	95-99	202.88	225.24	182.22	HT-Direct
Liberia	SOUTH CENTRAL	95-99	217.32	201.04	234.54	RW2
Liberia	SOUTH CENTRAL	00-04	156.36	173.81	140.37	HT-Direct
Liberia	SOUTH CENTRAL	00-04	155.26	142.59	169.02	RW2
Liberia	SOUTH CENTRAL	05-09	93.08	113.93	75.72	HT-Direct
Liberia	SOUTH CENTRAL	05-09	110.59	97.25	125.51	RW2
Liberia	SOUTH CENTRAL	10-14	93.64	121.50	71.64	HT-Direct
Liberia	SOUTH CENTRAL	10-14	78.53	65.24	94.38	RW2
Liberia	SOUTH CENTRAL	15-19	55.07	22.33	128.93	RW2
Liberia	SOUTH EASTERN A	80-84	184.28	270.36	121.06	HT-Direct
Liberia	SOUTH EASTERN A	80-84	206.47	160.95	259.81	RW2
Liberia	SOUTH EASTERN A	85-89	204.74	248.18	167.21	HT-Direct
Liberia	SOUTH EASTERN A	85-89	220.12	190.23	253.28	RW2
Liberia	SOUTH EASTERN A	90-94	258.51	305.74	216.30	HT-Direct
Liberia	SOUTH EASTERN A	90-94	239.23	214.50	265.94	RW2
Liberia	SOUTH EASTERN A	95-99	185.08	221.54	153.43	HT-Direct
Liberia	SOUTH EASTERN A	95-99	197.69	177.75	219.61	RW2
Liberia	SOUTH EASTERN A	00-04	139.61	163.62	118.62	HT-Direct
Liberia	SOUTH EASTERN A	00-04	144.60	129.40	161.49	RW2
Liberia	SOUTH EASTERN A	05-09	111.05	135.66	90.44	HT-Direct
Liberia	SOUTH EASTERN A	05-09	107.00	93.03	122.84	RW2
Liberia	SOUTH EASTERN A	10-14	78.28	105.72	57.51	HT-Direct
Liberia	SOUTH EASTERN A	10-14	78.64	63.86	96.05	RW2
Liberia	SOUTH EASTERN A	15-19	56.95	22.77	134.56	RW2
Liberia	SOUTH EASTERN B	80-84	164.40	225.27	117.49	HT-Direct
Liberia	SOUTH EASTERN B	80-84	174.08	135.86	222.32	RW2
Liberia	SOUTH EASTERN B	85-89	191.63	227.89	159.94	HT-Direct
Liberia	SOUTH EASTERN B	85-89	194.06	169.35	221.75	RW2
Liberia	SOUTH EASTERN B	90-94	233.96	263.50	206.80	HT-Direct
Liberia	SOUTH EASTERN B	90-94	219.46	199.96	240.57	RW2
Liberia	SOUTH EASTERN B	95-99	171.96	199.60	147.45	HT-Direct
Liberia	SOUTH EASTERN B	95-99	190.45	170.01	211.17	RW2
Liberia	SOUTH EASTERN B	00-04	136.74	165.64	112.21	HT-Direct
Liberia	SOUTH EASTERN B	00-04	154.88	136.91	173.20	RW2
Liberia	SOUTH EASTERN B	05-09	129.05	142.09	117.05	HT-Direct
Liberia	SOUTH EASTERN B	05-09	136.46	124.59	149.16	RW2
Liberia	SOUTH EASTERN B	10-14	149.05	185.19	118.93	HT-Direct
Liberia	SOUTH EASTERN B	10-14	124.34	105.06	147.78	RW2
Liberia	SOUTH EASTERN B	15-19	113.91	46.90	251.44	RW2
Madagascar	ALL	80-84	187.79	198.41	177.60	HT-Direct
Madagascar	ALL	80-84	165.73	161.52	170.18	IHME
Madagascar	ALL	80-84	180.00	170.61	189.79	RW2
Madagascar	ALL	80-84	180.07	174.50	186.07	UN
Madagascar	ALL	85-89	178.75	187.84	170.01	HT-Direct
Madagascar	ALL	85-89	161.67	157.73	165.74	IHME
Madagascar	ALL	85-89	175.66	167.31	184.31	RW2
Madagascar	ALL	85-89	175.51	170.54	181.16	UN
Madagascar	ALL	90-94	146.51	154.46	138.90	HT-Direct
Madagascar	ALL	90-94	139.44	135.98	142.88	IHME
Madagascar	ALL	90-94	151.48	143.57	159.73	RW2
Madagascar	ALL	90-94	151.62	146.90	156.19	UN
Madagascar	ALL	95-99	122.89	130.62	115.55	HT-Direct
Madagascar	ALL	95-99	119.85	116.48	123.52	IHME
Madagascar	ALL	95-99	127.32	119.66	135.35	RW2
Madagascar	ALL	95-99	127.22	123.04	131.47	UN
Madagascar	ALL	00-04	90.86	97.55	84.59	HT-Direct
Madagascar	ALL	00-04	98.55	95.14	102.11	IHME
Madagascar	ALL	00-04	97.00	90.08	104.43	RW2
Madagascar	ALL	00-04	97.07	92.82	101.48	UN
Madagascar	ALL	05-09	66.11	72.90	59.91	HT-Direct
Madagascar	ALL	05-09	78.84	75.52	82.79	IHME
Madagascar	ALL	05-09	71.38	64.15	79.35	RW2
Madagascar	ALL	05-09	71.36	66.41	76.61	UN

Continued on next page

Country	Region	Year	Median	Lower	Upper	Method
Madagascar	ALL	10-14	71.77	66.33	78.09	IHME
Madagascar	ALL	10-14	51.97	18.99	133.66	RW2
Madagascar	ALL	10-14	55.71	48.63	64.70	UN
Madagascar	ANTANANARIVO	80-84	141.96	158.62	126.78	HT-Direct
Madagascar	ANTANANARIVO	80-84	140.62	126.67	155.59	RW2
Madagascar	ANTANANARIVO	85-89	156.52	174.76	139.86	HT-Direct
Madagascar	ANTANANARIVO	85-89	143.21	130.49	157.71	RW2
Madagascar	ANTANANARIVO	90-94	113.62	129.92	99.14	HT-Direct
Madagascar	ANTANANARIVO	90-94	120.51	108.82	133.49	RW2
Madagascar	ANTANANARIVO	95-99	88.48	102.74	76.03	HT-Direct
Madagascar	ANTANANARIVO	95-99	92.06	81.19	103.57	RW2
Madagascar	ANTANANARIVO	00-04	61.07	72.39	51.42	HT-Direct
Madagascar	ANTANANARIVO	00-04	74.98	64.97	85.89	RW2
Madagascar	ANTANANARIVO	05-09	65.24	81.38	52.13	HT-Direct
Madagascar	ANTANANARIVO	05-09	59.98	48.16	75.34	RW2
Madagascar	ANTANANARIVO	10-14	47.35	17.26	125.17	RW2
Madagascar	ANTANANARIVO	15-19	37.36	3.13	320.63	RW2
Madagascar	ANTSIRANANA	80-84	151.14	176.79	128.63	HT-Direct
Madagascar	ANTSIRANANA	80-84	142.51	123.14	164.36	RW2
Madagascar	ANTSIRANANA	85-89	134.70	156.36	115.63	HT-Direct
Madagascar	ANTSIRANANA	85-89	137.92	122.33	154.87	RW2
Madagascar	ANTSIRANANA	90-94	134.46	158.92	113.26	HT-Direct
Madagascar	ANTSIRANANA	90-94	129.46	113.44	147.54	RW2
Madagascar	ANTSIRANANA	95-99	95.35	115.91	78.12	HT-Direct
Madagascar	ANTSIRANANA	95-99	108.69	93.26	126.17	RW2
Madagascar	ANTSIRANANA	00-04	98.12	126.83	75.35	HT-Direct
Madagascar	ANTSIRANANA	00-04	91.89	74.81	112.86	RW2
Madagascar	ANTSIRANANA	05-09	51.31	84.34	30.79	HT-Direct
Madagascar	ANTSIRANANA	05-09	64.33	42.73	95.70	RW2
Madagascar	ANTSIRANANA	10-14	41.33	12.09	129.80	RW2
Madagascar	ANTSIRANANA	15-19	26.11	1.69	281.80	RW2
Madagascar	FIANARANTSOA	80-84	250.64	278.62	224.60	HT-Direct
Madagascar	FIANARANTSOA	80-84	234.90	212.39	259.50	RW2
Madagascar	FIANARANTSOA	85-89	214.89	236.24	194.97	HT-Direct
Madagascar	FIANARANTSOA	85-89	215.22	198.66	232.31	RW2
Madagascar	FIANARANTSOA	90-94	184.59	202.88	167.59	HT-Direct
Madagascar	FIANARANTSOA	90-94	194.09	178.69	210.00	RW2
Madagascar	FIANARANTSOA	95-99	168.89	188.07	151.31	HT-Direct
Madagascar	FIANARANTSOA	95-99	172.13	157.34	188.49	RW2
Madagascar	FIANARANTSOA	00-04	139.18	155.72	124.14	HT-Direct
Madagascar	FIANARANTSOA	00-04	144.43	130.68	159.77	RW2
Madagascar	FIANARANTSOA	05-09	89.04	102.63	77.09	HT-Direct
Madagascar	FIANARANTSOA	05-09	97.96	84.68	112.99	RW2
Madagascar	FIANARANTSOA	10-14	60.10	22.99	144.54	RW2
Madagascar	FIANARANTSOA	15-19	35.53	3.08	292.81	RW2
Madagascar	MAHAJANGA	80-84	250.23	278.59	223.87	HT-Direct
Madagascar	MAHAJANGA	80-84	233.52	210.75	258.38	RW2
Madagascar	MAHAJANGA	85-89	188.21	209.77	168.40	HT-Direct
Madagascar	MAHAJANGA	85-89	189.91	173.92	206.31	RW2
Madagascar	MAHAJANGA	90-94	146.44	162.32	131.86	HT-Direct
Madagascar	MAHAJANGA	90-94	152.58	139.45	166.50	RW2
Madagascar	MAHAJANGA	95-99	112.95	131.23	96.93	HT-Direct
Madagascar	MAHAJANGA	95-99	121.72	108.61	136.06	RW2
Madagascar	MAHAJANGA	00-04	106.10	126.68	88.53	HT-Direct
Madagascar	MAHAJANGA	00-04	99.07	86.26	114.32	RW2
Madagascar	MAHAJANGA	05-09	57.51	71.56	46.08	HT-Direct
Madagascar	MAHAJANGA	05-09	65.98	53.45	81.19	RW2
Madagascar	MAHAJANGA	10-14	39.96	14.63	103.01	RW2
Madagascar	MAHAJANGA	15-19	24.02	2.02	216.61	RW2
Madagascar	TOAMASINA	80-84	176.87	203.15	153.35	HT-Direct
Madagascar	TOAMASINA	80-84	173.46	152.35	196.62	RW2
Madagascar	TOAMASINA	85-89	185.37	206.17	166.22	HT-Direct
Madagascar	TOAMASINA	85-89	184.68	169.63	201.04	RW2

Continued on next page

Country	Region	Year	Median	Lower	Upper	Method
Madagascar	TOAMASINA	90-94	175.92	196.20	157.32	HT-Direct
Madagascar	TOAMASINA	90-94	170.81	155.19	187.99	RW2
Madagascar	TOAMASINA	95-99	121.03	137.76	106.08	HT-Direct
Madagascar	TOAMASINA	95-99	126.56	113.75	140.57	RW2
Madagascar	TOAMASINA	00-04	72.86	89.03	59.44	HT-Direct
Madagascar	TOAMASINA	00-04	85.90	73.16	100.29	RW2
Madagascar	TOAMASINA	05-09	48.62	67.71	34.71	HT-Direct
Madagascar	TOAMASINA	05-09	50.33	37.58	66.58	RW2
Madagascar	TOAMASINA	10-14	27.76	9.50	76.87	RW2
Madagascar	TOAMASINA	15-19	15.22	1.24	153.91	RW2
Madagascar	TOLIARY	80-84	181.89	212.46	154.86	HT-Direct
Madagascar	TOLIARY	80-84	175.79	152.72	201.63	RW2
Madagascar	TOLIARY	85-89	180.74	201.71	161.52	HT-Direct
Madagascar	TOLIARY	85-89	176.15	160.94	192.55	RW2
Madagascar	TOLIARY	90-94	153.11	172.19	135.81	HT-Direct
Madagascar	TOLIARY	90-94	162.98	148.07	178.96	RW2
Madagascar	TOLIARY	95-99	142.51	162.21	124.85	HT-Direct
Madagascar	TOLIARY	95-99	142.32	128.18	158.18	RW2
Madagascar	TOLIARY	00-04	107.44	121.35	94.96	HT-Direct
Madagascar	TOLIARY	00-04	113.11	101.43	126.13	RW2
Madagascar	TOLIARY	05-09	61.91	75.59	50.57	HT-Direct
Madagascar	TOLIARY	05-09	70.46	57.76	85.25	RW2
Madagascar	TOLIARY	10-14	39.80	14.42	102.53	RW2
Madagascar	TOLIARY	15-19	22.26	1.82	204.12	RW2
Malawi	ALL	80-84	242.81	253.26	232.65	HT-Direct
Malawi	ALL	80-84	249.54	244.17	254.87	IHME
Malawi	ALL	80-84	247.37	236.82	258.23	RW2
Malawi	ALL	80-84	247.38	240.91	254.48	UN
Malawi	ALL	85-89	242.53	250.37	234.86	HT-Direct
Malawi	ALL	85-89	235.41	230.64	240.45	IHME
Malawi	ALL	85-89	251.17	242.98	259.51	RW2
Malawi	ALL	85-89	251.19	244.39	258.13	UN
Malawi	ALL	90-94	216.68	223.62	209.90	HT-Direct
Malawi	ALL	90-94	212.05	207.95	216.48	IHME
Malawi	ALL	90-94	227.16	219.79	234.70	RW2
Malawi	ALL	90-94	227.09	221.44	233.44	UN
Malawi	ALL	95-99	187.01	192.96	181.21	HT-Direct
Malawi	ALL	95-99	184.45	180.19	188.56	IHME
Malawi	ALL	95-99	197.02	190.60	203.56	RW2
Malawi	ALL	95-99	197.10	192.43	202.12	UN
Malawi	ALL	00-04	143.11	148.47	137.91	HT-Direct
Malawi	ALL	00-04	147.19	143.21	151.06	IHME
Malawi	ALL	00-04	148.13	142.64	153.83	RW2
Malawi	ALL	00-04	148.18	143.93	152.99	UN
Malawi	ALL	05-09	101.75	106.32	97.35	HT-Direct
Malawi	ALL	05-09	112.49	109.06	115.99	IHME
Malawi	ALL	05-09	105.34	100.66	110.22	RW2
Malawi	ALL	05-09	105.24	101.01	109.83	UN
Malawi	ALL	10-14	68.74	74.00	63.83	HT-Direct
Malawi	ALL	10-14	91.52	87.03	96.06	IHME
Malawi	ALL	10-14	79.49	72.83	86.66	RW2
Malawi	ALL	10-14	79.65	72.89	87.10	UN
Malawi	CENTRAL REGION	80-84	271.78	290.18	254.14	HT-Direct
Malawi	CENTRAL REGION	80-84	278.09	261.82	295.12	RW2
Malawi	CENTRAL REGION	85-89	268.83	281.51	256.51	HT-Direct
Malawi	CENTRAL REGION	85-89	273.04	262.22	284.25	RW2
Malawi	CENTRAL REGION	90-94	223.18	234.62	212.15	HT-Direct
Malawi	CENTRAL REGION	90-94	239.50	229.67	249.57	RW2
Malawi	CENTRAL REGION	95-99	191.53	201.24	182.17	HT-Direct
Malawi	CENTRAL REGION	95-99	202.53	193.94	211.21	RW2
Malawi	CENTRAL REGION	00-04	149.62	158.84	140.84	HT-Direct
Malawi	CENTRAL REGION	00-04	152.67	145.41	160.30	RW2
Malawi	CENTRAL REGION	05-09	104.93	112.68	97.66	HT-Direct

Continued on next page

Country	Region	Year	Median	Lower	Upper	Method
Malawi	CENTRAL REGION	05-09	110.62	104.09	117.53	RW2
Malawi	CENTRAL REGION	10-14	76.30	85.29	68.19	HT-Direct
Malawi	CENTRAL REGION	10-14	84.62	75.72	94.60	RW2
Malawi	CENTRAL REGION	15-19	65.68	30.19	137.92	RW2
Malawi	NORTHERN REGION	80-84	193.59	216.41	172.65	HT-Direct
Malawi	NORTHERN REGION	80-84	199.91	180.89	220.10	RW2
Malawi	NORTHERN REGION	85-89	196.25	212.94	180.57	HT-Direct
Malawi	NORTHERN REGION	85-89	200.85	188.41	214.20	RW2
Malawi	NORTHERN REGION	90-94	171.04	186.48	156.63	HT-Direct
Malawi	NORTHERN REGION	90-94	177.60	166.31	189.34	RW2
Malawi	NORTHERN REGION	95-99	135.85	149.65	123.13	HT-Direct
Malawi	NORTHERN REGION	95-99	150.60	140.24	161.30	RW2
Malawi	NORTHERN REGION	00-04	116.03	129.02	104.20	HT-Direct
Malawi	NORTHERN REGION	00-04	114.50	105.97	123.97	RW2
Malawi	NORTHERN REGION	05-09	79.94	92.20	69.18	HT-Direct
Malawi	NORTHERN REGION	05-09	82.78	74.87	91.48	RW2
Malawi	NORTHERN REGION	10-14	52.18	61.64	44.11	HT-Direct
Malawi	NORTHERN REGION	10-14	62.47	53.01	73.25	RW2
Malawi	NORTHERN REGION	15-19	47.77	21.03	104.96	RW2
Malawi	SOUTHERN REGION	80-84	229.31	243.00	216.17	HT-Direct
Malawi	SOUTHERN REGION	80-84	232.05	219.40	245.33	RW2
Malawi	SOUTHERN REGION	85-89	229.98	241.28	219.05	HT-Direct
Malawi	SOUTHERN REGION	85-89	243.97	233.64	254.33	RW2
Malawi	SOUTHERN REGION	90-94	223.45	233.16	214.04	HT-Direct
Malawi	SOUTHERN REGION	90-94	230.84	222.01	240.01	RW2
Malawi	SOUTHERN REGION	95-99	193.89	201.82	186.20	HT-Direct
Malawi	SOUTHERN REGION	95-99	201.87	194.41	209.67	RW2
Malawi	SOUTHERN REGION	00-04	142.00	149.15	135.14	HT-Direct
Malawi	SOUTHERN REGION	00-04	149.94	143.53	156.61	RW2
Malawi	SOUTHERN REGION	05-09	103.86	110.13	97.91	HT-Direct
Malawi	SOUTHERN REGION	05-09	106.17	100.59	112.00	RW2
Malawi	SOUTHERN REGION	10-14	65.79	73.50	58.83	HT-Direct
Malawi	SOUTHERN REGION	10-14	78.05	69.58	87.34	RW2
Malawi	SOUTHERN REGION	15-19	57.78	26.21	123.23	RW2
Mali	ALL	80-84	296.64	306.73	286.74	HT-Direct
Mali	ALL	80-84	264.91	261.52	267.92	IHME
Mali	ALL	80-84	308.58	297.83	319.53	RW2
Mali	ALL	80-84	308.66	299.76	318.13	UN
Mali	ALL	85-89	268.90	277.16	260.79	HT-Direct
Mali	ALL	85-89	240.25	237.66	243.28	IHME
Mali	ALL	85-89	271.57	263.33	279.94	RW2
Mali	ALL	85-89	271.40	263.51	279.20	UN
Mali	ALL	90-94	245.08	251.99	238.30	HT-Direct
Mali	ALL	90-94	222.21	219.55	225.14	IHME
Mali	ALL	90-94	247.13	240.27	254.12	RW2
Mali	ALL	90-94	247.33	239.50	254.41	UN
Mali	ALL	95-99	245.50	254.85	236.39	HT-Direct
Mali	ALL	95-99	205.62	202.49	208.44	IHME
Mali	ALL	95-99	234.72	226.41	243.18	RW2
Mali	ALL	95-99	234.26	227.42	242.23	UN
Mali	ALL	00-04	201.49	210.20	193.05	HT-Direct
Mali	ALL	00-04	182.24	179.26	185.52	IHME
Mali	ALL	00-04	200.55	192.30	209.11	RW2
Mali	ALL	00-04	200.96	193.90	208.55	UN
Mali	ALL	05-09	168.55	185.28	153.05	HT-Direct
Mali	ALL	05-09	158.74	155.00	162.52	IHME
Mali	ALL	05-09	155.74	142.41	170.07	RW2
Mali	ALL	05-09	155.29	143.82	166.77	UN
Mali	ALL	10-14	140.35	134.80	145.97	IHME
Mali	ALL	10-14	116.87	45.58	265.70	RW2
Mali	ALL	10-14	128.09	110.80	148.02	UN
Mali	BAMAKO	80-84	173.88	192.18	156.97	HT-Direct
Mali	BAMAKO	80-84	181.48	165.17	198.80	RW2

Continued on next page

Country	Region	Year	Median	Lower	Upper	Method
Mali	BAMAKO	85-89	148.58	166.26	132.47	HT-Direct
Mali	BAMAKO	85-89	154.74	143.71	166.51	RW2
Mali	BAMAKO	90-94	144.98	161.06	130.25	HT-Direct
Mali	BAMAKO	90-94	138.34	128.64	148.89	RW2
Mali	BAMAKO	95-99	130.87	149.49	114.26	HT-Direct
Mali	BAMAKO	95-99	130.56	120.49	141.40	RW2
Mali	BAMAKO	00-04	109.22	124.45	95.66	HT-Direct
Mali	BAMAKO	00-04	106.92	96.40	118.53	RW2
Mali	BAMAKO	05-09	77.40	113.98	51.88	HT-Direct
Mali	BAMAKO	05-09	80.49	64.93	98.49	RW2
Mali	BAMAKO	10-14	59.45	23.40	141.68	RW2
Mali	BAMAKO	15-19	43.63	4.43	320.46	RW2
Mali	KAYES, KOULIKORO	80-84	294.57	311.15	278.52	HT-Direct
Mali	KAYES, KOULIKORO	80-84	302.71	287.34	318.98	RW2
Mali	KAYES, KOULIKORO	85-89	257.13	271.77	243.01	HT-Direct
Mali	KAYES, KOULIKORO	85-89	261.92	250.73	273.23	RW2
Mali	KAYES, KOULIKORO	90-94	232.98	244.69	221.66	HT-Direct
Mali	KAYES, KOULIKORO	90-94	236.60	227.30	246.00	RW2
Mali	KAYES, KOULIKORO	95-99	238.16	250.52	226.22	HT-Direct
Mali	KAYES, KOULIKORO	95-99	227.74	218.69	237.07	RW2
Mali	KAYES, KOULIKORO	00-04	197.55	212.32	183.58	HT-Direct
Mali	KAYES, KOULIKORO	00-04	194.15	183.07	205.95	RW2
Mali	KAYES, KOULIKORO	05-09	165.21	201.03	134.70	HT-Direct
Mali	KAYES, KOULIKORO	05-09	153.29	134.58	174.20	RW2
Mali	KAYES, KOULIKORO	10-14	118.41	50.67	252.50	RW2
Mali	KAYES, KOULIKORO	15-19	90.55	10.11	497.98	RW2
Mali	MOPTI, TOMBOUCTOU, GAO, KIDAL	80-84	383.04	406.82	359.81	HT-Direct
Mali	MOPTI, TOMBOUCTOU, GAO, KIDAL	80-84	406.69	383.81	429.34	RW2
Mali	MOPTI, TOMBOUCTOU, GAO, KIDAL	85-89	351.97	372.29	332.18	HT-Direct
Mali	MOPTI, TOMBOUCTOU, GAO, KIDAL	85-89	348.51	333.65	363.77	RW2
Mali	MOPTI, TOMBOUCTOU, GAO, KIDAL	90-94	296.41	311.90	281.37	HT-Direct
Mali	MOPTI, TOMBOUCTOU, GAO, KIDAL	90-94	298.62	286.69	311.20	RW2
Mali	MOPTI, TOMBOUCTOU, GAO, KIDAL	95-99	279.53	297.16	262.56	HT-Direct
Mali	MOPTI, TOMBOUCTOU, GAO, KIDAL	95-99	262.82	250.97	274.99	RW2
Mali	MOPTI, TOMBOUCTOU, GAO, KIDAL	00-04	191.87	207.24	177.38	HT-Direct
Mali	MOPTI, TOMBOUCTOU, GAO, KIDAL	00-04	201.70	189.43	214.48	RW2
Mali	MOPTI, TOMBOUCTOU, GAO, KIDAL	05-09	173.69	207.09	144.70	HT-Direct
Mali	MOPTI, TOMBOUCTOU, GAO, KIDAL	05-09	145.11	127.56	165.12	RW2
Mali	MOPTI, TOMBOUCTOU, GAO, KIDAL	10-14	101.98	43.37	222.30	RW2
Mali	MOPTI, TOMBOUCTOU, GAO, KIDAL	15-19	70.54	7.48	431.61	RW2
Mali	SIKASSO, SEGOU	80-84	279.83	295.21	264.96	HT-Direct
Mali	SIKASSO, SEGOU	80-84	290.18	275.81	304.97	RW2
Mali	SIKASSO, SEGOU	85-89	264.39	277.07	252.09	HT-Direct
Mali	SIKASSO, SEGOU	85-89	266.79	256.76	277.15	RW2
Mali	SIKASSO, SEGOU	90-94	249.90	261.24	238.90	HT-Direct
Mali	SIKASSO, SEGOU	90-94	253.64	244.43	262.94	RW2
Mali	SIKASSO, SEGOU	95-99	265.37	283.18	248.29	HT-Direct
Mali	SIKASSO, SEGOU	95-99	254.85	243.68	266.42	RW2
Mali	SIKASSO, SEGOU	00-04	234.67	251.49	218.65	HT-Direct
Mali	SIKASSO, SEGOU	00-04	227.15	214.84	240.15	RW2
Mali	SIKASSO, SEGOU	05-09	197.36	224.43	172.82	HT-Direct
Mali	SIKASSO, SEGOU	05-09	189.10	171.01	208.57	RW2
Mali	SIKASSO, SEGOU	10-14	155.15	69.90	313.00	RW2
Mali	SIKASSO, SEGOU	15-19	125.41	14.71	580.00	RW2
Morocco	ALL	80-84	114.95	121.53	108.69	HT-Direct
Morocco	ALL	80-84	110.92	109.27	112.75	IHME
Morocco	ALL	80-84	120.67	113.75	127.96	RW2
Morocco	ALL	80-84	120.68	117.85	123.93	UN
Morocco	ALL	85-89	88.60	95.00	82.59	HT-Direct
Morocco	ALL	85-89	83.10	81.61	84.46	IHME
Morocco	ALL	85-89	93.08	86.59	99.98	RW2
Morocco	ALL	85-89	93.06	90.49	95.68	UN
Morocco	ALL	90-94	71.10	77.68	65.03	HT-Direct

Continued on next page

Country	Region	Year	Median	Lower	Upper	Method
Morocco	ALL	90-94	63.58	62.39	64.93	IHME
Morocco	ALL	90-94	73.04	66.93	79.66	RW2
Morocco	ALL	90-94	73.15	70.89	75.36	UN
Morocco	ALL	95-99	59.09	66.35	52.57	HT-Direct
Morocco	ALL	95-99	50.42	49.10	51.85	IHME
Morocco	ALL	95-99	57.95	51.95	64.54	RW2
Morocco	ALL	95-99	57.73	55.73	59.79	UN
Morocco	ALL	00-04	44.49	51.51	38.39	HT-Direct
Morocco	ALL	00-04	40.28	38.49	42.03	IHME
Morocco	ALL	00-04	45.74	39.40	53.09	RW2
Morocco	ALL	00-04	45.88	44.01	47.82	UN
Morocco	ALL	05-09	32.25	30.20	34.41	IHME
Morocco	ALL	05-09	35.82	10.21	117.54	RW2
Morocco	ALL	05-09	37.16	35.23	39.25	UN
Morocco	ALL	10-14	25.87	23.78	28.26	IHME
Morocco	ALL	10-14	27.81	1.22	388.29	RW2
Morocco	ALL	10-14	30.51	27.87	33.48	UN
Morocco	CENTRE	80-84	85.05	95.98	75.27	HT-Direct
Morocco	CENTRE	80-84	84.67	75.28	95.27	RW2
Morocco	CENTRE	85-89	56.63	65.84	48.64	HT-Direct
Morocco	CENTRE	85-89	65.41	58.52	72.74	RW2
Morocco	CENTRE	90-94	49.56	60.37	40.61	HT-Direct
Morocco	CENTRE	90-94	52.48	45.91	59.71	RW2
Morocco	CENTRE	95-99	45.35	58.21	35.22	HT-Direct
Morocco	CENTRE	95-99	44.62	37.74	52.62	RW2
Morocco	CENTRE	00-04	39.73	53.39	29.46	HT-Direct
Morocco	CENTRE	00-04	37.71	29.85	47.84	RW2
Morocco	CENTRE	05-09	31.80	10.52	91.14	RW2
Morocco	CENTRE	10-14	26.66	1.78	297.88	RW2
Morocco	CENTRE	15-19	22.69	0.19	728.81	RW2
Morocco	CENTRE-NORD	80-84	121.85	140.94	105.02	HT-Direct
Morocco	CENTRE-NORD	80-84	131.00	114.04	150.03	RW2
Morocco	CENTRE-NORD	85-89	100.57	117.58	85.78	HT-Direct
Morocco	CENTRE-NORD	85-89	104.53	93.39	117.13	RW2
Morocco	CENTRE-NORD	90-94	86.48	106.10	70.20	HT-Direct
Morocco	CENTRE-NORD	90-94	82.99	72.41	95.24	RW2
Morocco	CENTRE-NORD	95-99	67.16	89.49	50.10	HT-Direct
Morocco	CENTRE-NORD	95-99	66.87	55.71	80.17	RW2
Morocco	CENTRE-NORD	00-04	47.07	66.85	32.94	HT-Direct
Morocco	CENTRE-NORD	00-04	52.17	39.68	68.06	RW2
Morocco	CENTRE-NORD	05-09	39.99	13.07	114.55	RW2
Morocco	CENTRE-NORD	10-14	30.55	2.01	320.97	RW2
Morocco	CENTRE-NORD	15-19	22.85	0.19	734.55	RW2
Morocco	CENTRE-SUD	80-84	95.88	114.94	79.70	HT-Direct
Morocco	CENTRE-SUD	80-84	108.39	91.22	128.48	RW2
Morocco	CENTRE-SUD	85-89	106.44	134.79	83.47	HT-Direct
Morocco	CENTRE-SUD	85-89	91.78	79.64	105.88	RW2
Morocco	CENTRE-SUD	90-94	75.23	97.13	57.95	HT-Direct
Morocco	CENTRE-SUD	90-94	77.93	66.34	91.61	RW2
Morocco	CENTRE-SUD	95-99	64.17	94.64	43.05	HT-Direct
Morocco	CENTRE-SUD	95-99	68.04	54.50	84.70	RW2
Morocco	CENTRE-SUD	00-04	58.27	92.24	36.31	HT-Direct
Morocco	CENTRE-SUD	00-04	57.98	42.22	79.31	RW2
Morocco	CENTRE-SUD	05-09	48.60	15.72	138.48	RW2
Morocco	CENTRE-SUD	10-14	40.60	2.67	397.51	RW2
Morocco	CENTRE-SUD	15-19	34.38	0.28	800.25	RW2
Morocco	NORD-OUEST	80-84	115.10	127.84	103.49	HT-Direct
Morocco	NORD-OUEST	80-84	123.57	111.44	136.72	RW2
Morocco	NORD-OUEST	85-89	97.96	111.66	85.77	HT-Direct
Morocco	NORD-OUEST	85-89	94.30	85.47	104.19	RW2
Morocco	NORD-OUEST	90-94	61.40	74.85	50.24	HT-Direct
Morocco	NORD-OUEST	90-94	71.65	62.92	81.34	RW2
Morocco	NORD-OUEST	95-99	57.30	72.67	45.02	HT-Direct

Continued on next page

Country	Region	Year	Median	Lower	Upper	Method
Morocco	NORD-OUEST	95-99	56.48	47.81	66.62	RW2
Morocco	NORD-OUEST	00-04	45.13	63.68	31.80	HT-Direct
Morocco	NORD-OUEST	00-04	43.89	34.09	56.53	RW2
Morocco	NORD-OUEST	05-09	33.84	11.04	98.48	RW2
Morocco	NORD-OUEST	10-14	25.95	1.75	290.68	RW2
Morocco	NORD-OUEST	15-19	19.60	0.17	695.69	RW2
Morocco	ORIENTAL	80-84	102.99	123.46	85.59	HT-Direct
Morocco	ORIENTAL	80-84	103.01	86.56	122.28	RW2
Morocco	ORIENTAL	85-89	70.21	87.18	56.34	HT-Direct
Morocco	ORIENTAL	85-89	82.79	71.20	96.04	RW2
Morocco	ORIENTAL	90-94	69.58	104.13	45.90	HT-Direct
Morocco	ORIENTAL	90-94	67.78	55.69	82.07	RW2
Morocco	ORIENTAL	95-99	66.29	95.98	45.32	HT-Direct
Morocco	ORIENTAL	95-99	57.16	44.22	73.62	RW2
Morocco	ORIENTAL	00-04	38.95	69.89	21.40	HT-Direct
Morocco	ORIENTAL	00-04	46.80	31.97	68.14	RW2
Morocco	ORIENTAL	05-09	37.85	11.45	116.21	RW2
Morocco	ORIENTAL	10-14	30.37	1.87	337.87	RW2
Morocco	ORIENTAL	15-19	24.49	0.20	751.42	RW2
Morocco	SUD	80-84	159.28	183.91	137.39	HT-Direct
Morocco	SUD	80-84	170.06	148.23	194.02	RW2
Morocco	SUD	85-89	120.16	140.38	102.50	HT-Direct
Morocco	SUD	85-89	121.56	108.25	135.86	RW2
Morocco	SUD	90-94	80.53	100.37	64.33	HT-Direct
Morocco	SUD	90-94	87.27	75.63	100.50	RW2
Morocco	SUD	95-99	70.57	97.65	50.58	HT-Direct
Morocco	SUD	95-99	64.50	52.58	79.00	RW2
Morocco	SUD	00-04	42.53	66.38	27.00	HT-Direct
Morocco	SUD	00-04	46.40	34.17	62.81	RW2
Morocco	SUD	05-09	32.86	10.47	97.05	RW2
Morocco	SUD	10-14	23.07	1.51	263.13	RW2
Morocco	SUD	15-19	16.08	0.13	662.37	RW2
Morocco	TENSIFT	80-84	138.66	158.16	121.23	HT-Direct
Morocco	TENSIFT	80-84	143.08	125.55	162.42	RW2
Morocco	TENSIFT	85-89	91.41	110.61	75.27	HT-Direct
Morocco	TENSIFT	85-89	108.09	95.41	122.02	RW2
Morocco	TENSIFT	90-94	95.43	118.56	76.43	HT-Direct
Morocco	TENSIFT	90-94	83.72	72.15	97.08	RW2
Morocco	TENSIFT	95-99	63.52	89.90	44.51	HT-Direct
Morocco	TENSIFT	95-99	66.76	54.76	81.18	RW2
Morocco	TENSIFT	00-04	49.43	68.51	35.46	HT-Direct
Morocco	TENSIFT	00-04	51.81	39.45	67.50	RW2
Morocco	TENSIFT	05-09	39.64	12.88	115.30	RW2
Morocco	TENSIFT	10-14	30.01	2.00	329.13	RW2
Morocco	TENSIFT	15-19	22.69	0.20	732.27	RW2
Mozambique	ALL	80-84	254.77	272.82	237.53	HT-Direct
Mozambique	ALL	80-84	250.42	237.09	264.07	IHME
Mozambique	ALL	80-84	260.00	242.14	278.69	RW2
Mozambique	ALL	80-84	259.85	250.08	271.14	UN
Mozambique	ALL	85-89	232.33	247.89	217.46	HT-Direct
Mozambique	ALL	85-89	230.62	223.88	238.24	IHME
Mozambique	ALL	85-89	248.51	232.21	265.48	RW2
Mozambique	ALL	85-89	248.72	238.91	257.88	UN
Mozambique	ALL	90-94	218.30	231.70	205.46	HT-Direct
Mozambique	ALL	90-94	205.17	202.81	207.71	IHME
Mozambique	ALL	90-94	231.83	218.06	246.23	RW2
Mozambique	ALL	90-94	231.85	224.04	239.63	UN
Mozambique	ALL	95-99	188.90	198.28	179.87	HT-Direct
Mozambique	ALL	95-99	176.97	174.80	179.20	IHME
Mozambique	ALL	95-99	198.19	188.48	208.19	RW2
Mozambique	ALL	95-99	198.20	192.02	205.08	UN
Mozambique	ALL	00-04	141.87	150.93	133.26	HT-Direct
Mozambique	ALL	00-04	144.16	141.98	146.21	IHME

Continued on next page

Country	Region	Year	Median	Lower	Upper	Method
Mozambique	ALL	00-04	155.15	145.29	165.59	RW2
Mozambique	ALL	00-04	154.80	149.67	160.17	UN
Mozambique	ALL	05-09	101.60	109.61	94.12	HT-Direct
Mozambique	ALL	05-09	114.91	112.42	117.29	IHME
Mozambique	ALL	05-09	119.68	109.50	130.67	RW2
Mozambique	ALL	05-09	120.29	115.63	125.70	UN
Mozambique	ALL	10-14	87.52	102.60	74.48	HT-Direct
Mozambique	ALL	10-14	90.78	87.44	93.88	IHME
Mozambique	ALL	10-14	92.91	78.59	109.41	RW2
Mozambique	ALL	10-14	92.10	85.76	99.25	UN
Mozambique	CABO DELGADO	80-84	301.69	372.89	238.91	HT-Direct
Mozambique	CABO DELGADO	80-84	317.66	266.98	371.12	RW2
Mozambique	CABO DELGADO	85-89	287.41	334.14	244.81	HT-Direct
Mozambique	CABO DELGADO	85-89	308.80	275.68	343.69	RW2
Mozambique	CABO DELGADO	90-94	272.10	311.44	236.02	HT-Direct
Mozambique	CABO DELGADO	90-94	292.29	266.24	320.04	RW2
Mozambique	CABO DELGADO	95-99	242.70	277.86	210.68	HT-Direct
Mozambique	CABO DELGADO	95-99	251.89	229.06	277.31	RW2
Mozambique	CABO DELGADO	00-04	194.02	226.87	164.92	HT-Direct
Mozambique	CABO DELGADO	00-04	189.58	167.91	213.29	RW2
Mozambique	CABO DELGADO	05-09	100.98	128.53	78.80	HT-Direct
Mozambique	CABO DELGADO	05-09	131.71	108.26	158.67	RW2
Mozambique	CABO DELGADO	10-14	60.13	106.56	33.18	HT-Direct
Mozambique	CABO DELGADO	10-14	89.90	62.39	124.35	RW2
Mozambique	CABO DELGADO	15-19	60.37	22.09	150.69	RW2
Mozambique	GAZA	80-84	218.86	265.84	178.16	HT-Direct
Mozambique	GAZA	80-84	219.87	186.79	257.49	RW2
Mozambique	GAZA	85-89	202.12	240.52	168.49	HT-Direct
Mozambique	GAZA	85-89	212.13	188.00	238.55	RW2
Mozambique	GAZA	90-94	185.32	219.61	155.32	HT-Direct
Mozambique	GAZA	90-94	197.90	178.18	218.99	RW2
Mozambique	GAZA	95-99	161.56	188.50	137.82	HT-Direct
Mozambique	GAZA	95-99	172.77	155.41	191.08	RW2
Mozambique	GAZA	00-04	126.17	153.72	102.96	HT-Direct
Mozambique	GAZA	00-04	142.66	125.36	161.99	RW2
Mozambique	GAZA	05-09	113.40	146.20	87.21	HT-Direct
Mozambique	GAZA	05-09	117.72	98.16	140.87	RW2
Mozambique	GAZA	10-14	93.54	135.74	63.50	HT-Direct
Mozambique	GAZA	10-14	98.35	74.83	128.94	RW2
Mozambique	GAZA	15-19	81.98	33.43	188.36	RW2
Mozambique	INHAMBANE	80-84	224.34	273.20	182.04	HT-Direct
Mozambique	INHAMBANE	80-84	237.78	200.79	278.55	RW2
Mozambique	INHAMBANE	85-89	178.42	220.09	143.19	HT-Direct
Mozambique	INHAMBANE	85-89	219.21	193.75	246.76	RW2
Mozambique	INHAMBANE	90-94	194.09	221.90	169.02	HT-Direct
Mozambique	INHAMBANE	90-94	193.83	176.46	212.83	RW2
Mozambique	INHAMBANE	95-99	154.14	172.51	137.40	HT-Direct
Mozambique	INHAMBANE	95-99	155.92	142.42	170.73	RW2
Mozambique	INHAMBANE	00-04	95.51	119.31	76.05	HT-Direct
Mozambique	INHAMBANE	00-04	114.22	98.92	131.45	RW2
Mozambique	INHAMBANE	05-09	58.48	85.67	39.54	HT-Direct
Mozambique	INHAMBANE	05-09	82.36	64.85	103.23	RW2
Mozambique	INHAMBANE	10-14	34.10	80.20	14.09	HT-Direct
Mozambique	INHAMBANE	10-14	60.11	41.02	85.63	RW2
Mozambique	INHAMBANE	15-19	43.79	16.23	111.15	RW2
Mozambique	MANICA	80-84	270.94	338.61	212.45	HT-Direct
Mozambique	MANICA	80-84	279.87	235.33	328.85	RW2
Mozambique	MANICA	85-89	265.20	319.32	217.33	HT-Direct
Mozambique	MANICA	85-89	261.16	230.89	293.85	RW2
Mozambique	MANICA	90-94	214.34	244.31	187.12	HT-Direct
Mozambique	MANICA	90-94	234.74	213.89	256.79	RW2
Mozambique	MANICA	95-99	193.56	222.21	167.81	HT-Direct
Mozambique	MANICA	95-99	195.46	177.96	213.95	RW2

Continued on next page

Country	Region	Year	Median	Lower	Upper	Method
Mozambique	MANICA	00-04	123.43	146.75	103.37	HT-Direct
Mozambique	MANICA	00-04	151.16	134.12	170.13	RW2
Mozambique	MANICA	05-09	104.27	132.37	81.57	HT-Direct
Mozambique	MANICA	05-09	116.81	97.63	139.10	RW2
Mozambique	MANICA	10-14	113.10	176.77	70.41	HT-Direct
Mozambique	MANICA	10-14	91.76	69.23	121.55	RW2
Mozambique	MANICA	15-19	72.01	29.18	169.07	RW2
Mozambique	MAPUTO CIDADE	80-84	95.34	126.69	71.12	HT-Direct
Mozambique	MAPUTO CIDADE	80-84	98.61	77.76	124.95	RW2
Mozambique	MAPUTO CIDADE	85-89	99.97	127.17	78.07	HT-Direct
Mozambique	MAPUTO CIDADE	85-89	102.89	87.17	121.15	RW2
Mozambique	MAPUTO CIDADE	90-94	95.91	119.13	76.83	HT-Direct
Mozambique	MAPUTO CIDADE	90-94	104.07	89.90	119.77	RW2
Mozambique	MAPUTO CIDADE	95-99	90.57	111.96	72.94	HT-Direct
Mozambique	MAPUTO CIDADE	95-99	100.77	87.45	115.35	RW2
Mozambique	MAPUTO CIDADE	00-04	92.35	112.34	75.62	HT-Direct
Mozambique	MAPUTO CIDADE	00-04	94.80	81.68	109.95	RW2
Mozambique	MAPUTO CIDADE	05-09	75.06	96.89	57.83	HT-Direct
Mozambique	MAPUTO CIDADE	05-09	88.22	71.52	108.91	RW2
Mozambique	MAPUTO CIDADE	10-14	66.54	116.45	37.12	HT-Direct
Mozambique	MAPUTO CIDADE	10-14	82.25	56.65	117.58	RW2
Mozambique	MAPUTO CIDADE	15-19	76.67	27.51	194.23	RW2
Mozambique	MAPUTO PROVINCIA	80-84	134.79	190.48	93.51	HT-Direct
Mozambique	MAPUTO PROVINCIA	80-84	130.37	100.97	168.02	RW2
Mozambique	MAPUTO PROVINCIA	85-89	129.04	164.42	100.37	HT-Direct
Mozambique	MAPUTO PROVINCIA	85-89	131.48	110.64	155.98	RW2
Mozambique	MAPUTO PROVINCIA	90-94	118.37	146.97	94.73	HT-Direct
Mozambique	MAPUTO PROVINCIA	90-94	128.76	111.65	147.50	RW2
Mozambique	MAPUTO PROVINCIA	95-99	93.94	118.88	73.80	HT-Direct
Mozambique	MAPUTO PROVINCIA	95-99	120.75	105.07	137.57	RW2
Mozambique	MAPUTO PROVINCIA	00-04	114.01	137.97	93.77	HT-Direct
Mozambique	MAPUTO PROVINCIA	00-04	109.85	95.19	126.51	RW2
Mozambique	MAPUTO PROVINCIA	05-09	85.20	107.47	67.21	HT-Direct
Mozambique	MAPUTO PROVINCIA	05-09	99.65	81.92	121.03	RW2
Mozambique	MAPUTO PROVINCIA	10-14	76.66	133.78	42.72	HT-Direct
Mozambique	MAPUTO PROVINCIA	10-14	90.96	65.27	125.72	RW2
Mozambique	MAPUTO PROVINCIA	15-19	83.08	31.64	199.73	RW2
Mozambique	NAMPULA	80-84	303.38	350.91	259.70	HT-Direct
Mozambique	NAMPULA	80-84	311.24	271.62	352.85	RW2
Mozambique	NAMPULA	85-89	239.61	287.83	197.23	HT-Direct
Mozambique	NAMPULA	85-89	292.09	260.89	324.15	RW2
Mozambique	NAMPULA	90-94	259.15	310.41	213.73	HT-Direct
Mozambique	NAMPULA	90-94	266.36	241.83	292.92	RW2
Mozambique	NAMPULA	95-99	231.42	258.29	206.56	HT-Direct
Mozambique	NAMPULA	95-99	219.73	200.64	240.83	RW2
Mozambique	NAMPULA	00-04	129.86	156.74	107.00	HT-Direct
Mozambique	NAMPULA	00-04	157.77	139.25	178.36	RW2
Mozambique	NAMPULA	05-09	62.71	86.48	45.14	HT-Direct
Mozambique	NAMPULA	05-09	106.48	86.77	129.10	RW2
Mozambique	NAMPULA	10-14	78.29	124.54	48.27	HT-Direct
Mozambique	NAMPULA	10-14	71.55	51.13	97.12	RW2
Mozambique	NAMPULA	15-19	47.50	18.09	117.77	RW2
Mozambique	NIASSA	80-84	331.59	415.89	256.86	HT-Direct
Mozambique	NIASSA	80-84	295.70	242.64	356.17	RW2
Mozambique	NIASSA	85-89	230.32	292.84	177.80	HT-Direct
Mozambique	NIASSA	85-89	271.51	234.68	311.92	RW2
Mozambique	NIASSA	90-94	211.33	257.42	171.59	HT-Direct
Mozambique	NIASSA	90-94	243.93	217.22	272.44	RW2
Mozambique	NIASSA	95-99	189.65	217.87	164.31	HT-Direct
Mozambique	NIASSA	95-99	201.25	181.60	222.34	RW2
Mozambique	NIASSA	00-04	152.09	188.48	121.68	HT-Direct
Mozambique	NIASSA	00-04	147.78	129.48	168.25	RW2
Mozambique	NIASSA	05-09	79.42	98.47	63.79	HT-Direct

Continued on next page

Country	Region	Year	Median	Lower	Upper	Method
Mozambique	NIASSA	05-09	102.99	85.43	123.57	RW2
Mozambique	NIASSA	10-14	83.02	148.36	44.94	HT-Direct
Mozambique	NIASSA	10-14	71.77	52.01	98.18	RW2
Mozambique	NIASSA	15-19	49.89	18.87	123.87	RW2
Mozambique	SOFALA	80-84	316.07	367.96	268.39	HT-Direct
Mozambique	SOFALA	80-84	329.17	289.94	370.76	RW2
Mozambique	SOFALA	85-89	295.54	335.22	258.73	HT-Direct
Mozambique	SOFALA	85-89	295.24	268.13	324.06	RW2
Mozambique	SOFALA	90-94	230.78	266.29	198.72	HT-Direct
Mozambique	SOFALA	90-94	254.72	233.37	277.37	RW2
Mozambique	SOFALA	95-99	178.11	207.25	152.28	HT-Direct
Mozambique	SOFALA	95-99	202.55	184.46	221.79	RW2
Mozambique	SOFALA	00-04	143.18	167.54	121.84	HT-Direct
Mozambique	SOFALA	00-04	148.27	131.94	166.09	RW2
Mozambique	SOFALA	05-09	97.25	128.19	73.15	HT-Direct
Mozambique	SOFALA	05-09	106.68	89.72	126.52	RW2
Mozambique	SOFALA	10-14	70.63	100.77	49.02	HT-Direct
Mozambique	SOFALA	10-14	77.38	59.86	100.01	RW2
Mozambique	SOFALA	15-19	55.88	22.95	130.68	RW2
Mozambique	TETE	80-84	293.29	357.31	236.51	HT-Direct
Mozambique	TETE	80-84	302.06	258.83	348.35	RW2
Mozambique	TETE	85-89	268.28	312.54	228.20	HT-Direct
Mozambique	TETE	85-89	292.94	263.51	323.85	RW2
Mozambique	TETE	90-94	261.51	291.39	233.70	HT-Direct
Mozambique	TETE	90-94	275.00	253.75	297.43	RW2
Mozambique	TETE	95-99	237.47	267.11	210.18	HT-Direct
Mozambique	TETE	95-99	238.56	219.88	258.15	RW2
Mozambique	TETE	00-04	170.39	196.52	147.11	HT-Direct
Mozambique	TETE	00-04	190.70	172.17	210.67	RW2
Mozambique	TETE	05-09	116.42	139.45	96.77	HT-Direct
Mozambique	TETE	05-09	150.66	129.83	174.48	RW2
Mozambique	TETE	10-14	146.31	214.37	97.18	HT-Direct
Mozambique	TETE	10-14	120.86	94.00	154.84	RW2
Mozambique	TETE	15-19	96.78	39.92	218.44	RW2
Mozambique	ZAMBEZIA	80-84	271.78	326.99	222.81	HT-Direct
Mozambique	ZAMBEZIA	80-84	275.33	237.04	318.43	RW2
Mozambique	ZAMBEZIA	85-89	263.78	311.27	221.20	HT-Direct
Mozambique	ZAMBEZIA	85-89	267.44	239.63	297.54	RW2
Mozambique	ZAMBEZIA	90-94	246.76	279.44	216.75	HT-Direct
Mozambique	ZAMBEZIA	90-94	252.59	231.52	274.83	RW2
Mozambique	ZAMBEZIA	95-99	192.54	218.65	168.87	HT-Direct
Mozambique	ZAMBEZIA	95-99	220.71	202.74	239.46	RW2
Mozambique	ZAMBEZIA	00-04	150.44	175.90	128.09	HT-Direct
Mozambique	ZAMBEZIA	00-04	177.47	160.21	196.21	RW2
Mozambique	ZAMBEZIA	05-09	143.37	166.09	123.30	HT-Direct
Mozambique	ZAMBEZIA	05-09	140.24	122.06	160.49	RW2
Mozambique	ZAMBEZIA	10-14	88.24	129.42	59.27	HT-Direct
Mozambique	ZAMBEZIA	10-14	111.34	87.59	140.93	RW2
Mozambique	ZAMBEZIA	15-19	88.62	36.80	197.67	RW2
Namibia	ALL	80-84	83.11	98.70	69.80	HT-Direct
Namibia	ALL	80-84	92.76	89.63	95.98	IHME
Namibia	ALL	80-84	94.40	77.46	114.60	RW2
Namibia	ALL	80-84	94.25	90.45	98.04	UN
Namibia	ALL	85-89	70.39	80.42	61.53	HT-Direct
Namibia	ALL	85-89	77.18	74.67	79.77	IHME
Namibia	ALL	85-89	80.38	69.75	92.45	RW2
Namibia	ALL	85-89	81.00	77.97	84.32	UN
Namibia	ALL	90-94	65.80	73.48	58.87	HT-Direct
Namibia	ALL	90-94	66.03	64.06	68.03	IHME
Namibia	ALL	90-94	71.83	64.15	80.34	RW2
Namibia	ALL	90-94	70.97	68.09	74.11	UN
Namibia	ALL	95-99	60.22	68.37	52.98	HT-Direct
Namibia	ALL	95-99	61.14	59.16	63.31	IHME

Continued on next page

Country	Region	Year	Median	Lower	Upper	Method
Namibia	ALL	95-99	71.72	62.53	81.83	RW2
Namibia	ALL	95-99	72.65	69.21	75.92	UN
Namibia	ALL	00-04	74.81	83.32	67.10	HT-Direct
Namibia	ALL	00-04	64.68	62.33	67.00	IHME
Namibia	ALL	00-04	76.05	68.56	84.46	RW2
Namibia	ALL	00-04	75.58	72.49	78.81	UN
Namibia	ALL	05-09	65.73	73.01	59.14	HT-Direct
Namibia	ALL	05-09	61.58	58.75	64.51	IHME
Namibia	ALL	05-09	64.42	58.34	71.06	RW2
Namibia	ALL	05-09	64.51	61.04	68.51	UN
Namibia	ALL	10-14	53.16	64.30	43.85	HT-Direct
Namibia	ALL	10-14	48.89	45.65	52.49	IHME
Namibia	ALL	10-14	50.33	42.12	59.97	RW2
Namibia	ALL	10-14	50.39	45.14	56.48	UN
Namibia	CAPRIVI	80-84	101.08	190.45	51.00	HT-Direct
Namibia	CAPRIVI	80-84	108.11	66.66	170.86	RW2
Namibia	CAPRIVI	85-89	80.31	125.07	50.64	HT-Direct
Namibia	CAPRIVI	85-89	91.98	66.08	125.71	RW2
Namibia	CAPRIVI	90-94	74.33	106.00	51.58	HT-Direct
Namibia	CAPRIVI	90-94	83.48	64.89	106.54	RW2
Namibia	CAPRIVI	95-99	64.11	97.96	41.42	HT-Direct
Namibia	CAPRIVI	95-99	90.52	71.82	113.18	RW2
Namibia	CAPRIVI	00-04	113.30	161.55	78.11	HT-Direct
Namibia	CAPRIVI	00-04	94.29	76.36	116.09	RW2
Namibia	CAPRIVI	05-09	79.73	109.06	57.78	HT-Direct
Namibia	CAPRIVI	05-09	83.44	66.28	104.49	RW2
Namibia	CAPRIVI	10-14	76.48	133.14	42.74	HT-Direct
Namibia	CAPRIVI	10-14	71.54	49.75	101.78	RW2
Namibia	CAPRIVI	15-19	61.32	21.45	164.58	RW2
Namibia	ERONGO	80-84	42.21	81.94	21.29	HT-Direct
Namibia	ERONGO	80-84	57.82	36.62	90.84	RW2
Namibia	ERONGO	85-89	64.51	98.04	41.91	HT-Direct
Namibia	ERONGO	85-89	51.50	36.83	71.78	RW2
Namibia	ERONGO	90-94	33.31	56.63	19.39	HT-Direct
Namibia	ERONGO	90-94	48.88	37.35	63.73	RW2
Namibia	ERONGO	95-99	34.55	57.34	20.62	HT-Direct
Namibia	ERONGO	95-99	55.24	43.54	69.44	RW2
Namibia	ERONGO	00-04	56.20	85.17	36.69	HT-Direct
Namibia	ERONGO	00-04	59.64	47.70	74.11	RW2
Namibia	ERONGO	05-09	58.43	88.99	37.93	HT-Direct
Namibia	ERONGO	05-09	55.05	42.61	70.64	RW2
Namibia	ERONGO	10-14	54.86	90.32	32.82	HT-Direct
Namibia	ERONGO	10-14	49.62	35.01	69.77	RW2
Namibia	ERONGO	15-19	45.20	16.77	116.35	RW2
Namibia	HARDAP	80-84	131.68	213.15	78.26	HT-Direct
Namibia	HARDAP	80-84	126.32	84.53	184.16	RW2
Namibia	HARDAP	85-89	76.08	116.26	49.02	HT-Direct
Namibia	HARDAP	85-89	92.56	68.38	123.74	RW2
Namibia	HARDAP	90-94	57.58	83.96	39.13	HT-Direct
Namibia	HARDAP	90-94	71.81	56.36	91.00	RW2
Namibia	HARDAP	95-99	66.81	108.39	40.46	HT-Direct
Namibia	HARDAP	95-99	65.56	51.92	82.14	RW2
Namibia	HARDAP	00-04	50.20	85.41	29.04	HT-Direct
Namibia	HARDAP	00-04	56.86	44.44	72.39	RW2
Namibia	HARDAP	05-09	45.87	73.59	28.27	HT-Direct
Namibia	HARDAP	05-09	41.87	31.09	56.34	RW2
Namibia	HARDAP	10-14	30.41	60.43	15.07	HT-Direct
Namibia	HARDAP	10-14	29.91	19.75	45.17	RW2
Namibia	HARDAP	15-19	21.39	7.40	61.15	RW2
Namibia	KARAS	80-84	107.64	154.83	73.57	HT-Direct
Namibia	KARAS	80-84	117.21	82.36	164.30	RW2
Namibia	KARAS	85-89	74.77	109.87	50.25	HT-Direct
Namibia	KARAS	85-89	89.16	68.45	115.35	RW2

Continued on next page

Country	Region	Year	Median	Lower	Upper	Method
Namibia	KARAS	90-94	74.91	103.52	53.74	HT-Direct
Namibia	KARAS	90-94	72.02	57.02	90.14	RW2
Namibia	KARAS	95-99	41.06	64.96	25.72	HT-Direct
Namibia	KARAS	95-99	69.05	53.77	87.08	RW2
Namibia	KARAS	00-04	57.75	87.00	37.93	HT-Direct
Namibia	KARAS	00-04	64.22	50.10	81.67	RW2
Namibia	KARAS	05-09	59.32	90.66	38.36	HT-Direct
Namibia	KARAS	05-09	51.91	38.87	68.92	RW2
Namibia	KARAS	10-14	46.36	93.53	22.39	HT-Direct
Namibia	KARAS	10-14	40.94	26.46	63.81	RW2
Namibia	KARAS	15-19	32.32	10.70	97.43	RW2
Namibia	KAVANGO	80-84	111.48	175.10	69.04	HT-Direct
Namibia	KAVANGO	80-84	119.90	85.77	166.03	RW2
Namibia	KAVANGO	85-89	103.51	138.03	76.85	HT-Direct
Namibia	KAVANGO	85-89	100.73	79.95	126.45	RW2
Namibia	KAVANGO	90-94	77.45	103.94	57.27	HT-Direct
Namibia	KAVANGO	90-94	90.03	75.37	107.24	RW2
Namibia	KAVANGO	95-99	83.17	99.83	69.08	HT-Direct
Namibia	KAVANGO	95-99	95.72	82.46	110.67	RW2
Namibia	KAVANGO	00-04	68.11	90.04	51.21	HT-Direct
Namibia	KAVANGO	00-04	97.85	84.06	113.39	RW2
Namibia	KAVANGO	05-09	106.12	133.89	83.56	HT-Direct
Namibia	KAVANGO	05-09	85.95	72.12	102.20	RW2
Namibia	KAVANGO	10-14	77.96	122.95	48.53	HT-Direct
Namibia	KAVANGO	10-14	73.23	55.45	96.60	RW2
Namibia	KAVANGO	15-19	62.41	23.98	155.00	RW2
Namibia	KHOMAS	80-84	79.70	145.33	42.25	HT-Direct
Namibia	KHOMAS	80-84	78.53	49.17	122.95	RW2
Namibia	KHOMAS	85-89	43.35	76.73	24.12	HT-Direct
Namibia	KHOMAS	85-89	64.27	45.21	90.31	RW2
Namibia	KHOMAS	90-94	52.37	77.59	35.04	HT-Direct
Namibia	KHOMAS	90-94	56.01	42.89	72.80	RW2
Namibia	KHOMAS	95-99	47.14	82.78	26.40	HT-Direct
Namibia	KHOMAS	95-99	57.54	45.80	72.27	RW2
Namibia	KHOMAS	00-04	51.76	79.01	33.56	HT-Direct
Namibia	KHOMAS	00-04	55.74	44.89	69.05	RW2
Namibia	KHOMAS	05-09	47.98	66.85	34.24	HT-Direct
Namibia	KHOMAS	05-09	45.67	35.54	58.42	RW2
Namibia	KHOMAS	10-14	33.69	71.75	15.49	HT-Direct
Namibia	KHOMAS	10-14	36.34	24.80	52.90	RW2
Namibia	KHOMAS	15-19	29.06	10.19	80.25	RW2
Namibia	KUNENE	80-84	83.05	145.22	46.06	HT-Direct
Namibia	KUNENE	80-84	114.32	77.19	167.01	RW2
Namibia	KUNENE	85-89	96.75	144.44	63.63	HT-Direct
Namibia	KUNENE	85-89	89.82	67.09	119.37	RW2
Namibia	KUNENE	90-94	85.35	119.76	60.15	HT-Direct
Namibia	KUNENE	90-94	74.40	59.27	92.91	RW2
Namibia	KUNENE	95-99	53.44	78.09	36.26	HT-Direct
Namibia	KUNENE	95-99	72.64	59.08	88.36	RW2
Namibia	KUNENE	00-04	36.18	58.36	22.24	HT-Direct
Namibia	KUNENE	00-04	67.97	55.12	83.12	RW2
Namibia	KUNENE	05-09	50.84	74.05	34.64	HT-Direct
Namibia	KUNENE	05-09	54.53	42.98	69.02	RW2
Namibia	KUNENE	10-14	68.24	109.19	41.92	HT-Direct
Namibia	KUNENE	10-14	42.94	30.92	59.60	RW2
Namibia	KUNENE	15-19	33.83	12.59	89.20	RW2
Namibia	OHANGWENA	80-84	149.73	211.99	103.37	HT-Direct
Namibia	OHANGWENA	80-84	135.95	98.94	184.19	RW2
Namibia	OHANGWENA	85-89	74.38	107.77	50.74	HT-Direct
Namibia	OHANGWENA	85-89	111.31	87.34	141.25	RW2
Namibia	OHANGWENA	90-94	87.90	117.98	64.93	HT-Direct
Namibia	OHANGWENA	90-94	97.01	80.03	116.95	RW2
Namibia	OHANGWENA	95-99	71.00	96.98	51.58	HT-Direct

Continued on next page

Country	Region	Year	Median	Lower	Upper	Method
Namibia	OHANGWENA	95-99	100.04	84.37	118.38	RW2
Namibia	OHANGWENA	00-04	123.21	155.85	96.63	HT-Direct
Namibia	OHANGWENA	00-04	97.87	83.23	114.97	RW2
Namibia	OHANGWENA	05-09	70.09	94.42	51.67	HT-Direct
Namibia	OHANGWENA	05-09	80.39	65.76	98.05	RW2
Namibia	OHANGWENA	10-14	71.68	124.08	40.39	HT-Direct
Namibia	OHANGWENA	10-14	63.86	46.35	86.84	RW2
Namibia	OHANGWENA	15-19	50.62	18.71	128.87	RW2
Namibia	OMAHEKE	80-84	61.73	118.97	31.06	HT-Direct
Namibia	OMAHEKE	80-84	86.79	54.47	131.47	RW2
Namibia	OMAHEKE	85-89	44.68	69.65	28.39	HT-Direct
Namibia	OMAHEKE	85-89	76.17	56.52	101.42	RW2
Namibia	OMAHEKE	90-94	70.93	93.48	53.50	HT-Direct
Namibia	OMAHEKE	90-94	70.96	58.03	86.63	RW2
Namibia	OMAHEKE	95-99	80.01	98.54	64.72	HT-Direct
Namibia	OMAHEKE	95-99	76.15	63.86	91.26	RW2
Namibia	OMAHEKE	00-04	57.08	99.30	32.17	HT-Direct
Namibia	OMAHEKE	00-04	75.42	60.88	93.34	RW2
Namibia	OMAHEKE	05-09	57.20	88.46	36.54	HT-Direct
Namibia	OMAHEKE	05-09	62.26	47.08	82.18	RW2
Namibia	OMAHEKE	10-14	46.25	90.12	23.20	HT-Direct
Namibia	OMAHEKE	10-14	49.78	32.70	74.17	RW2
Namibia	OMAHEKE	15-19	39.77	13.67	108.86	RW2
Namibia	OMUSATI	80-84	102.85	187.78	53.79	HT-Direct
Namibia	OMUSATI	80-84	127.27	83.51	189.21	RW2
Namibia	OMUSATI	85-89	101.30	150.06	67.13	HT-Direct
Namibia	OMUSATI	85-89	97.76	72.19	131.35	RW2
Namibia	OMUSATI	90-94	57.06	83.48	38.65	HT-Direct
Namibia	OMUSATI	90-94	79.38	63.11	99.16	RW2
Namibia	OMUSATI	95-99	68.22	91.13	50.75	HT-Direct
Namibia	OMUSATI	95-99	76.39	62.78	92.30	RW2
Namibia	OMUSATI	00-04	69.06	95.77	49.39	HT-Direct
Namibia	OMUSATI	00-04	69.86	57.67	84.39	RW2
Namibia	OMUSATI	05-09	57.76	80.55	41.12	HT-Direct
Namibia	OMUSATI	05-09	53.77	42.27	68.11	RW2
Namibia	OMUSATI	10-14	32.32	67.73	15.12	HT-Direct
Namibia	OMUSATI	10-14	39.86	27.37	57.80	RW2
Namibia	OMUSATI	15-19	29.57	10.35	82.04	RW2
Namibia	OSHANA	80-84	105.26	154.18	70.57	HT-Direct
Namibia	OSHANA	80-84	118.10	83.91	163.85	RW2
Namibia	OSHANA	85-89	90.19	135.89	58.82	HT-Direct
Namibia	OSHANA	85-89	95.87	74.02	123.76	RW2
Namibia	OSHANA	90-94	64.90	88.85	47.06	HT-Direct
Namibia	OSHANA	90-94	82.76	67.61	100.76	RW2
Namibia	OSHANA	95-99	81.35	107.09	61.37	HT-Direct
Namibia	OSHANA	95-99	84.22	70.33	100.64	RW2
Namibia	OSHANA	00-04	76.67	106.52	54.67	HT-Direct
Namibia	OSHANA	00-04	81.17	66.94	98.28	RW2
Namibia	OSHANA	05-09	74.36	108.05	50.58	HT-Direct
Namibia	OSHANA	05-09	65.81	51.29	84.57	RW2
Namibia	OSHANA	10-14	39.32	88.60	16.94	HT-Direct
Namibia	OSHANA	10-14	51.68	35.03	75.04	RW2
Namibia	OSHANA	15-19	40.50	14.27	110.29	RW2
Namibia	OSHIKOTO	80-84	38.42	79.53	18.14	HT-Direct
Namibia	OSHIKOTO	80-84	87.42	57.73	128.14	RW2
Namibia	OSHIKOTO	85-89	64.98	92.35	45.32	HT-Direct
Namibia	OSHIKOTO	85-89	78.89	60.01	103.00	RW2
Namibia	OSHIKOTO	90-94	79.69	108.33	58.12	HT-Direct
Namibia	OSHIKOTO	90-94	75.20	61.60	91.54	RW2
Namibia	OSHIKOTO	95-99	76.50	97.17	59.94	HT-Direct
Namibia	OSHIKOTO	95-99	83.98	71.21	98.93	RW2
Namibia	OSHIKOTO	00-04	92.63	118.77	71.78	HT-Direct
Namibia	OSHIKOTO	00-04	87.92	74.81	103.29	RW2

Continued on next page

Country	Region	Year	Median	Lower	Upper	Method
Namibia	OSHIKOTO	05-09	66.24	94.08	46.22	HT-Direct
Namibia	OSHIKOTO	05-09	77.40	62.78	95.17	RW2
Namibia	OSHIKOTO	10-14	67.35	108.58	41.06	HT-Direct
Namibia	OSHIKOTO	10-14	66.15	48.45	89.35	RW2
Namibia	OSHIKOTO	15-19	56.47	21.38	142.25	RW2
Namibia	OTJOZONDJUPA	80-84	65.78	121.48	34.62	HT-Direct
Namibia	OTJOZONDJUPA	80-84	82.84	55.47	120.75	RW2
Namibia	OTJOZONDJUPA	85-89	62.43	89.12	43.35	HT-Direct
Namibia	OTJOZONDJUPA	85-89	70.44	53.33	92.29	RW2
Namibia	OTJOZONDJUPA	90-94	55.50	76.66	39.93	HT-Direct
Namibia	OTJOZONDJUPA	90-94	63.69	51.56	78.36	RW2
Namibia	OTJOZONDJUPA	95-99	45.17	68.64	29.48	HT-Direct
Namibia	OTJOZONDJUPA	95-99	67.66	56.23	81.30	RW2
Namibia	OTJOZONDJUPA	00-04	81.00	106.34	61.28	HT-Direct
Namibia	OTJOZONDJUPA	00-04	67.91	56.75	81.12	RW2
Namibia	OTJOZONDJUPA	05-09	52.05	76.85	34.96	HT-Direct
Namibia	OTJOZONDJUPA	05-09	57.32	45.38	72.19	RW2
Namibia	OTJOZONDJUPA	10-14	41.29	73.38	22.89	HT-Direct
Namibia	OTJOZONDJUPA	10-14	46.92	33.40	65.46	RW2
Namibia	OTJOZONDJUPA	15-19	38.65	14.27	100.95	RW2
Niger	ALL	80-84	315.24	327.65	303.10	HT-Direct
Niger	ALL	80-84	313.59	309.88	317.33	IHME
Niger	ALL	80-84	323.38	310.60	336.43	RW2
Niger	ALL	80-84	323.45	313.75	332.99	UN
Niger	ALL	85-89	327.20	337.64	316.93	HT-Direct
Niger	ALL	85-89	306.35	303.06	309.70	IHME
Niger	ALL	85-89	335.58	324.87	346.43	RW2
Niger	ALL	85-89	335.45	325.61	344.98	UN
Niger	ALL	90-94	297.18	306.91	287.63	HT-Direct
Niger	ALL	90-94	285.11	281.84	288.73	IHME
Niger	ALL	90-94	311.54	301.20	322.09	RW2
Niger	ALL	90-94	311.64	303.32	320.12	UN
Niger	ALL	95-99	246.40	255.27	237.73	HT-Direct
Niger	ALL	95-99	248.85	245.15	252.33	IHME
Niger	ALL	95-99	255.79	246.55	265.17	RW2
Niger	ALL	95-99	255.87	248.96	263.81	UN
Niger	ALL	00-04	206.27	215.43	197.40	HT-Direct
Niger	ALL	00-04	203.87	200.76	207.10	IHME
Niger	ALL	00-04	206.12	197.38	215.21	RW2
Niger	ALL	00-04	205.98	199.46	212.56	UN
Niger	ALL	05-09	146.56	154.99	138.51	HT-Direct
Niger	ALL	05-09	158.01	154.33	161.76	IHME
Niger	ALL	05-09	151.01	142.43	160.00	RW2
Niger	ALL	05-09	151.08	144.77	157.85	UN
Niger	ALL	10-14	127.02	121.93	131.92	IHME
Niger	ALL	10-14	104.71	40.31	241.38	RW2
Niger	ALL	10-14	110.69	101.57	121.65	UN
Niger	DOSSO	80-84	270.51	295.75	246.66	HT-Direct
Niger	DOSSO	80-84	272.11	249.69	296.03	RW2
Niger	DOSSO	85-89	276.17	298.99	254.46	HT-Direct
Niger	DOSSO	85-89	281.29	264.79	298.71	RW2
Niger	DOSSO	90-94	237.39	256.62	219.18	HT-Direct
Niger	DOSSO	90-94	259.36	243.02	275.58	RW2
Niger	DOSSO	95-99	225.22	246.18	205.56	HT-Direct
Niger	DOSSO	95-99	229.97	215.00	245.26	RW2
Niger	DOSSO	00-04	214.44	231.03	198.74	HT-Direct
Niger	DOSSO	00-04	214.48	201.91	227.82	RW2
Niger	DOSSO	05-09	185.22	203.99	167.81	HT-Direct
Niger	DOSSO	05-09	188.53	171.96	206.53	RW2
Niger	DOSSO	10-14	160.11	71.27	322.36	RW2
Niger	DOSSO	15-19	134.17	15.05	603.94	RW2
Niger	MARADI	80-84	369.76	393.72	346.42	HT-Direct
Niger	MARADI	80-84	375.33	352.86	398.35	RW2

Continued on next page

Country	Region	Year	Median	Lower	Upper	Method
Niger	MARADI	85-89	375.22	395.04	355.80	HT-Direct
Niger	MARADI	85-89	398.54	381.57	415.32	RW2
Niger	MARADI	90-94	376.13	395.11	357.51	HT-Direct
Niger	MARADI	90-94	382.33	366.17	399.22	RW2
Niger	MARADI	95-99	305.75	324.28	287.82	HT-Direct
Niger	MARADI	95-99	314.46	299.29	330.54	RW2
Niger	MARADI	00-04	236.53	254.91	219.08	HT-Direct
Niger	MARADI	00-04	237.16	223.37	251.57	RW2
Niger	MARADI	05-09	144.75	161.41	129.55	HT-Direct
Niger	MARADI	05-09	153.00	137.78	169.23	RW2
Niger	MARADI	10-14	90.32	37.86	197.77	RW2
Niger	MARADI	15-19	51.11	5.17	351.10	RW2
Niger	NIAMEY	80-84	152.87	174.93	133.14	HT-Direct
Niger	NIAMEY	80-84	150.60	132.52	170.68	RW2
Niger	NIAMEY	85-89	150.39	168.75	133.71	HT-Direct
Niger	NIAMEY	85-89	163.96	149.81	178.84	RW2
Niger	NIAMEY	90-94	151.82	171.61	133.94	HT-Direct
Niger	NIAMEY	90-94	156.24	142.28	171.19	RW2
Niger	NIAMEY	95-99	136.59	156.34	118.98	HT-Direct
Niger	NIAMEY	95-99	137.68	124.62	152.15	RW2
Niger	NIAMEY	00-04	123.33	144.00	105.27	HT-Direct
Niger	NIAMEY	00-04	122.21	108.74	137.27	RW2
Niger	NIAMEY	05-09	88.35	116.13	66.71	HT-Direct
Niger	NIAMEY	05-09	98.41	78.89	121.94	RW2
Niger	NIAMEY	10-14	76.00	29.03	181.98	RW2
Niger	NIAMEY	15-19	58.40	5.33	405.34	RW2
Niger	TASHOUA/AGADEZ	80-84	320.93	347.19	295.75	HT-Direct
Niger	TASHOUA/AGADEZ	80-84	328.74	305.67	352.76	RW2
Niger	TASHOUA/AGADEZ	85-89	322.83	346.89	299.67	HT-Direct
Niger	TASHOUA/AGADEZ	85-89	334.35	316.88	352.25	RW2
Niger	TASHOUA/AGADEZ	90-94	294.34	318.36	271.42	HT-Direct
Niger	TASHOUA/AGADEZ	90-94	301.01	284.76	318.01	RW2
Niger	TASHOUA/AGADEZ	95-99	230.70	248.34	213.96	HT-Direct
Niger	TASHOUA/AGADEZ	95-99	242.58	228.70	256.81	RW2
Niger	TASHOUA/AGADEZ	00-04	193.26	217.28	171.32	HT-Direct
Niger	TASHOUA/AGADEZ	00-04	193.51	179.50	208.45	RW2
Niger	TASHOUA/AGADEZ	05-09	136.33	155.42	119.25	HT-Direct
Niger	TASHOUA/AGADEZ	05-09	139.86	124.65	156.53	RW2
Niger	TASHOUA/AGADEZ	10-14	95.44	40.48	208.86	RW2
Niger	TASHOUA/AGADEZ	15-19	64.34	6.69	403.03	RW2
Niger	TILLABERI	80-84	247.86	275.21	222.39	HT-Direct
Niger	TILLABERI	80-84	276.19	251.62	301.68	RW2
Niger	TILLABERI	85-89	313.85	336.75	291.82	HT-Direct
Niger	TILLABERI	85-89	292.60	275.76	310.81	RW2
Niger	TILLABERI	90-94	235.83	256.34	216.48	HT-Direct
Niger	TILLABERI	90-94	263.56	247.50	279.90	RW2
Niger	TILLABERI	95-99	210.20	234.65	187.67	HT-Direct
Niger	TILLABERI	95-99	219.95	204.28	235.88	RW2
Niger	TILLABERI	00-04	187.99	204.92	172.16	HT-Direct
Niger	TILLABERI	00-04	189.53	176.87	202.94	RW2
Niger	TILLABERI	05-09	152.63	174.92	132.73	HT-Direct
Niger	TILLABERI	05-09	152.42	135.48	171.32	RW2
Niger	TILLABERI	10-14	117.59	50.01	251.08	RW2
Niger	TILLABERI	15-19	89.90	9.66	492.96	RW2
Niger	ZINDA/DIFFA	80-84	360.69	392.59	329.98	HT-Direct
Niger	ZINDA/DIFFA	80-84	368.98	340.90	398.49	RW2
Niger	ZINDA/DIFFA	85-89	367.41	392.18	343.33	HT-Direct
Niger	ZINDA/DIFFA	85-89	376.26	357.19	395.77	RW2
Niger	ZINDA/DIFFA	90-94	329.65	352.07	307.99	HT-Direct
Niger	ZINDA/DIFFA	90-94	344.43	326.77	362.58	RW2
Niger	ZINDA/DIFFA	95-99	263.60	284.43	243.78	HT-Direct
Niger	ZINDA/DIFFA	95-99	279.07	262.87	295.55	RW2
Niger	ZINDA/DIFFA	00-04	223.15	245.37	202.41	HT-Direct

Continued on next page

Country	Region	Year	Median	Lower	Upper	Method
Niger	ZINDA/DIFFA	00-04	219.19	204.06	235.18	RW2
Niger	ZINDA/DIFFA	05-09	148.38	168.86	130.00	HT-Direct
Niger	ZINDA/DIFFA	05-09	152.73	135.91	171.28	RW2
Niger	ZINDA/DIFFA	10-14	99.14	41.55	217.74	RW2
Niger	ZINDA/DIFFA	15-19	63.22	6.40	400.32	RW2
Nigeria	ALL	80-84	209.97	220.34	199.97	HT-Direct
Nigeria	ALL	80-84	218.11	214.92	221.28	IHME
Nigeria	ALL	80-84	210.64	200.57	221.08	RW2
Nigeria	ALL	80-84	210.74	203.42	218.05	UN
Nigeria	ALL	85-89	213.59	221.98	205.44	HT-Direct
Nigeria	ALL	85-89	210.74	207.86	213.88	IHME
Nigeria	ALL	85-89	211.64	203.72	219.76	RW2
Nigeria	ALL	85-89	211.46	205.53	217.77	UN
Nigeria	ALL	90-94	209.73	217.79	201.89	HT-Direct
Nigeria	ALL	90-94	204.18	201.54	206.97	IHME
Nigeria	ALL	90-94	211.08	203.29	219.09	RW2
Nigeria	ALL	90-94	211.30	205.57	217.09	UN
Nigeria	ALL	95-99	204.68	211.23	198.29	HT-Direct
Nigeria	ALL	95-99	192.15	189.68	194.71	IHME
Nigeria	ALL	95-99	200.57	194.44	206.80	RW2
Nigeria	ALL	95-99	200.45	194.94	205.96	UN
Nigeria	ALL	00-04	178.99	184.63	173.49	HT-Direct
Nigeria	ALL	00-04	175.49	172.92	178.00	IHME
Nigeria	ALL	00-04	175.20	169.96	180.59	RW2
Nigeria	ALL	00-04	175.32	170.33	180.06	UN
Nigeria	ALL	05-09	151.13	156.59	145.82	HT-Direct
Nigeria	ALL	05-09	149.69	146.71	152.37	IHME
Nigeria	ALL	05-09	146.59	141.58	151.74	RW2
Nigeria	ALL	05-09	146.49	141.68	151.76	UN
Nigeria	ALL	10-14	116.78	124.19	109.75	HT-Direct
Nigeria	ALL	10-14	120.69	116.52	124.95	IHME
Nigeria	ALL	10-14	120.69	113.21	128.53	RW2
Nigeria	ALL	10-14	120.81	112.42	130.51	UN
Nigeria	NORTH CENTRAL	80-84	169.54	194.77	146.98	HT-Direct
Nigeria	NORTH CENTRAL	80-84	166.20	148.97	185.36	RW2
Nigeria	NORTH CENTRAL	85-89	159.27	175.08	144.64	HT-Direct
Nigeria	NORTH CENTRAL	85-89	159.12	148.43	170.30	RW2
Nigeria	NORTH CENTRAL	90-94	149.02	164.20	135.02	HT-Direct
Nigeria	NORTH CENTRAL	90-94	149.42	140.36	158.93	RW2
Nigeria	NORTH CENTRAL	95-99	145.76	157.89	134.41	HT-Direct
Nigeria	NORTH CENTRAL	95-99	142.03	134.28	150.02	RW2
Nigeria	NORTH CENTRAL	00-04	123.67	134.21	113.85	HT-Direct
Nigeria	NORTH CENTRAL	00-04	127.37	120.50	134.57	RW2
Nigeria	NORTH CENTRAL	05-09	121.60	133.53	110.60	HT-Direct
Nigeria	NORTH CENTRAL	05-09	109.80	102.75	117.41	RW2
Nigeria	NORTH CENTRAL	10-14	82.33	94.25	71.80	HT-Direct
Nigeria	NORTH CENTRAL	10-14	89.29	79.92	99.71	RW2
Nigeria	NORTH CENTRAL	15-19	71.04	33.09	144.88	RW2
Nigeria	NORTH EAST	80-84	253.01	274.84	232.36	HT-Direct
Nigeria	NORTH EAST	80-84	254.96	236.08	274.61	RW2
Nigeria	NORTH EAST	85-89	270.05	289.94	251.04	HT-Direct
Nigeria	NORTH EAST	85-89	265.23	251.31	279.63	RW2
Nigeria	NORTH EAST	90-94	255.21	272.22	238.92	HT-Direct
Nigeria	NORTH EAST	90-94	263.60	251.02	276.45	RW2
Nigeria	NORTH EAST	95-99	261.63	274.25	249.39	HT-Direct
Nigeria	NORTH EAST	95-99	253.81	244.14	263.88	RW2
Nigeria	NORTH EAST	00-04	227.90	239.77	216.44	HT-Direct
Nigeria	NORTH EAST	00-04	218.86	210.13	228.06	RW2
Nigeria	NORTH EAST	05-09	172.43	184.84	160.69	HT-Direct
Nigeria	NORTH EAST	05-09	173.99	164.55	183.73	RW2
Nigeria	NORTH EAST	10-14	134.37	154.03	116.87	HT-Direct
Nigeria	NORTH EAST	10-14	133.35	118.82	149.33	RW2
Nigeria	NORTH EAST	15-19	100.16	47.14	199.66	RW2

Continued on next page

Country	Region	Year	Median	Lower	Upper	Method
Nigeria	NORTH WEST	80-84	279.66	300.69	259.56	HT-Direct
Nigeria	NORTH WEST	80-84	285.94	266.87	305.61	RW2
Nigeria	NORTH WEST	85-89	302.35	319.22	285.98	HT-Direct
Nigeria	NORTH WEST	85-89	299.24	286.24	312.77	RW2
Nigeria	NORTH WEST	90-94	301.66	318.89	284.97	HT-Direct
Nigeria	NORTH WEST	90-94	293.41	280.54	307.03	RW2
Nigeria	NORTH WEST	95-99	273.90	287.43	260.78	HT-Direct
Nigeria	NORTH WEST	95-99	270.01	259.80	280.56	RW2
Nigeria	NORTH WEST	00-04	229.44	241.20	218.08	HT-Direct
Nigeria	NORTH WEST	00-04	227.94	218.73	237.25	RW2
Nigeria	NORTH WEST	05-09	193.05	204.21	182.36	HT-Direct
Nigeria	NORTH WEST	05-09	186.68	178.24	195.41	RW2
Nigeria	NORTH WEST	10-14	145.66	159.50	132.83	HT-Direct
Nigeria	NORTH WEST	10-14	148.72	136.64	161.77	RW2
Nigeria	NORTH WEST	15-19	116.12	55.93	225.60	RW2
Nigeria	SOUTH EAST	80-84	132.17	153.81	113.16	HT-Direct
Nigeria	SOUTH EAST	80-84	133.83	117.67	152.11	RW2
Nigeria	SOUTH EAST	85-89	141.31	158.64	125.59	HT-Direct
Nigeria	SOUTH EAST	85-89	137.33	126.43	148.92	RW2
Nigeria	SOUTH EAST	90-94	138.12	152.75	124.69	HT-Direct
Nigeria	SOUTH EAST	90-94	139.52	129.77	149.82	RW2
Nigeria	SOUTH EAST	95-99	144.42	159.61	130.45	HT-Direct
Nigeria	SOUTH EAST	95-99	145.87	136.26	155.87	RW2
Nigeria	SOUTH EAST	00-04	152.62	166.41	139.78	HT-Direct
Nigeria	SOUTH EAST	00-04	144.92	136.09	154.42	RW2
Nigeria	SOUTH EAST	05-09	136.46	150.87	123.22	HT-Direct
Nigeria	SOUTH EAST	05-09	134.75	124.73	145.38	RW2
Nigeria	SOUTH EAST	10-14	115.56	142.17	93.39	HT-Direct
Nigeria	SOUTH EAST	10-14	117.76	100.44	137.33	RW2
Nigeria	SOUTH EAST	15-19	100.99	46.03	205.26	RW2
Nigeria	SOUTH SOUTH	80-84	147.74	169.07	128.69	HT-Direct
Nigeria	SOUTH SOUTH	80-84	141.78	126.17	159.21	RW2
Nigeria	SOUTH SOUTH	85-89	126.75	143.78	111.48	HT-Direct
Nigeria	SOUTH SOUTH	85-89	136.24	125.33	147.79	RW2
Nigeria	SOUTH SOUTH	90-94	133.12	147.38	120.05	HT-Direct
Nigeria	SOUTH SOUTH	90-94	132.17	122.92	141.68	RW2
Nigeria	SOUTH SOUTH	95-99	134.25	147.92	121.67	HT-Direct
Nigeria	SOUTH SOUTH	95-99	132.40	124.07	141.31	RW2
Nigeria	SOUTH SOUTH	00-04	131.85	143.64	120.89	HT-Direct
Nigeria	SOUTH SOUTH	00-04	124.34	116.86	132.52	RW2
Nigeria	SOUTH SOUTH	05-09	109.43	121.73	98.23	HT-Direct
Nigeria	SOUTH SOUTH	05-09	107.62	99.78	116.00	RW2
Nigeria	SOUTH SOUTH	10-14	80.06	93.99	68.04	HT-Direct
Nigeria	SOUTH SOUTH	10-14	86.29	75.12	98.50	RW2
Nigeria	SOUTH SOUTH	15-19	67.36	30.93	139.27	RW2
Nigeria	SOUTH WEST	80-84	152.94	180.09	129.24	HT-Direct
Nigeria	SOUTH WEST	80-84	151.87	132.13	174.91	RW2
Nigeria	SOUTH WEST	85-89	139.67	158.09	123.09	HT-Direct
Nigeria	SOUTH WEST	85-89	131.22	120.17	143.29	RW2
Nigeria	SOUTH WEST	90-94	105.02	118.55	92.87	HT-Direct
Nigeria	SOUTH WEST	90-94	113.96	104.97	123.33	RW2
Nigeria	SOUTH WEST	95-99	109.77	121.01	99.46	HT-Direct
Nigeria	SOUTH WEST	95-99	106.23	98.81	113.89	RW2
Nigeria	SOUTH WEST	00-04	97.15	107.19	87.97	HT-Direct
Nigeria	SOUTH WEST	00-04	97.26	90.73	104.17	RW2
Nigeria	SOUTH WEST	05-09	91.64	102.42	81.90	HT-Direct
Nigeria	SOUTH WEST	05-09	86.97	79.80	94.78	RW2
Nigeria	SOUTH WEST	10-14	73.40	93.53	57.33	HT-Direct
Nigeria	SOUTH WEST	10-14	73.92	62.15	88.19	RW2
Nigeria	SOUTH WEST	15-19	61.79	27.42	132.06	RW2
Rwanda	ALL	80-84	184.82	197.83	172.49	HT-Direct
Rwanda	ALL	80-84	183.05	178.72	187.12	IHME
Rwanda	ALL	80-84	188.89	175.97	202.53	RW2

Continued on next page

Country	Region	Year	Median	Lower	Upper	Method
Rwanda	ALL	80-84	188.63	182.96	193.81	UN
Rwanda	ALL	85-89	153.79	162.00	145.92	HT-Direct
Rwanda	ALL	85-89	156.43	152.45	159.97	IHME
Rwanda	ALL	85-89	152.46	144.80	160.41	RW2
Rwanda	ALL	85-89	153.07	148.61	157.43	UN
Rwanda	ALL	90-94	212.65	220.05	205.44	HT-Direct
Rwanda	ALL	90-94	187.82	163.45	216.87	IHME
Rwanda	ALL	90-94	183.25	177.74	188.89	RW2
Rwanda	ALL	90-94	182.69	176.51	189.45	UN
Rwanda	ALL	95-99	217.09	224.24	210.11	HT-Direct
Rwanda	ALL	95-99	176.38	171.82	180.55	IHME
Rwanda	ALL	95-99	224.40	216.90	232.04	RW2
Rwanda	ALL	95-99	225.09	212.46	236.58	UN
Rwanda	ALL	00-04	152.26	158.15	146.55	HT-Direct
Rwanda	ALL	00-04	137.76	133.65	141.35	IHME
Rwanda	ALL	00-04	154.22	148.40	160.24	RW2
Rwanda	ALL	00-04	153.90	149.44	158.95	UN
Rwanda	ALL	05-09	83.45	87.87	79.23	HT-Direct
Rwanda	ALL	05-09	86.74	83.70	89.98	IHME
Rwanda	ALL	05-09	88.66	83.90	93.66	RW2
Rwanda	ALL	05-09	88.79	85.42	92.16	UN
Rwanda	ALL	10-14	49.34	55.42	43.89	HT-Direct
Rwanda	ALL	10-14	71.42	66.66	76.65	IHME
Rwanda	ALL	10-14	53.39	47.04	60.49	RW2
Rwanda	ALL	10-14	53.34	49.12	57.82	UN
Rwanda	EAST	80-84	181.85	210.81	156.09	HT-Direct
Rwanda	EAST	80-84	183.93	160.50	210.53	RW2
Rwanda	EAST	85-89	163.34	179.61	148.28	HT-Direct
Rwanda	EAST	85-89	160.32	148.41	173.05	RW2
Rwanda	EAST	90-94	236.73	252.10	222.02	HT-Direct
Rwanda	EAST	90-94	205.65	195.50	216.05	RW2
Rwanda	EAST	95-99	249.38	264.70	234.66	HT-Direct
Rwanda	EAST	95-99	261.20	247.79	274.60	RW2
Rwanda	EAST	00-04	202.32	215.71	189.57	HT-Direct
Rwanda	EAST	00-04	198.54	187.33	210.89	RW2
Rwanda	EAST	05-09	108.27	118.56	98.77	HT-Direct
Rwanda	EAST	05-09	117.21	107.88	127.10	RW2
Rwanda	EAST	10-14	60.79	74.03	49.79	HT-Direct
Rwanda	EAST	10-14	67.50	55.53	81.22	RW2
Rwanda	EAST	15-19	38.44	12.23	112.22	RW2
Rwanda	KIGALI	80-84	116.56	151.21	89.01	HT-Direct
Rwanda	KIGALI	80-84	127.18	100.77	157.85	RW2
Rwanda	KIGALI	85-89	109.49	133.06	89.67	HT-Direct
Rwanda	KIGALI	85-89	111.23	97.52	126.95	RW2
Rwanda	KIGALI	90-94	174.20	198.31	152.46	HT-Direct
Rwanda	KIGALI	90-94	137.94	125.59	152.13	RW2
Rwanda	KIGALI	95-99	135.05	152.81	119.07	HT-Direct
Rwanda	KIGALI	95-99	156.54	141.39	171.67	RW2
Rwanda	KIGALI	00-04	108.13	127.26	91.57	HT-Direct
Rwanda	KIGALI	00-04	104.51	93.09	116.87	RW2
Rwanda	KIGALI	05-09	56.50	68.32	46.62	HT-Direct
Rwanda	KIGALI	05-09	59.23	50.92	68.98	RW2
Rwanda	KIGALI	10-14	31.42	44.99	21.85	HT-Direct
Rwanda	KIGALI	10-14	34.75	25.95	46.74	RW2
Rwanda	KIGALI	15-19	20.52	6.15	66.80	RW2
Rwanda	NORTH	80-84	211.41	247.33	179.46	HT-Direct
Rwanda	NORTH	80-84	202.24	175.19	234.28	RW2
Rwanda	NORTH	85-89	148.97	166.40	133.07	HT-Direct
Rwanda	NORTH	85-89	152.04	139.51	165.18	RW2
Rwanda	NORTH	90-94	203.16	219.86	187.42	HT-Direct
Rwanda	NORTH	90-94	179.05	168.64	189.69	RW2
Rwanda	NORTH	95-99	219.69	236.44	203.81	HT-Direct
Rwanda	NORTH	95-99	213.14	200.60	226.91	RW2

Continued on next page

Country	Region	Year	Median	Lower	Upper	Method
Rwanda	NORTH	00-04	127.54	140.62	115.52	HT-Direct
Rwanda	NORTH	00-04	140.73	130.36	151.61	RW2
Rwanda	NORTH	05-09	81.73	92.47	72.13	HT-Direct
Rwanda	NORTH	05-09	79.17	71.18	88.09	RW2
Rwanda	NORTH	10-14	35.60	47.97	26.33	HT-Direct
Rwanda	NORTH	10-14	45.08	35.44	56.94	RW2
Rwanda	NORTH	15-19	25.17	7.80	77.46	RW2
Rwanda	SOUTH	80-84	162.21	181.70	144.45	HT-Direct
Rwanda	SOUTH	80-84	173.41	154.23	194.05	RW2
Rwanda	SOUTH	85-89	162.75	179.84	146.98	HT-Direct
Rwanda	SOUTH	85-89	158.42	146.74	171.15	RW2
Rwanda	SOUTH	90-94	236.13	251.10	221.78	HT-Direct
Rwanda	SOUTH	90-94	198.28	188.36	208.91	RW2
Rwanda	SOUTH	95-99	209.25	222.49	196.60	HT-Direct
Rwanda	SOUTH	95-99	225.35	213.52	237.10	RW2
Rwanda	SOUTH	00-04	152.05	163.39	141.36	HT-Direct
Rwanda	SOUTH	00-04	149.14	140.13	158.50	RW2
Rwanda	SOUTH	05-09	72.34	80.77	64.72	HT-Direct
Rwanda	SOUTH	05-09	82.45	74.99	90.60	RW2
Rwanda	SOUTH	10-14	52.69	66.76	41.46	HT-Direct
Rwanda	SOUTH	10-14	49.37	40.20	61.16	RW2
Rwanda	SOUTH	15-19	30.22	9.55	91.48	RW2
Rwanda	WEST	80-84	201.90	227.39	178.60	HT-Direct
Rwanda	WEST	80-84	201.09	178.07	226.61	RW2
Rwanda	WEST	85-89	144.45	160.95	129.38	HT-Direct
Rwanda	WEST	85-89	143.21	131.15	155.76	RW2
Rwanda	WEST	90-94	186.78	201.47	172.94	HT-Direct
Rwanda	WEST	90-94	166.39	156.23	176.59	RW2
Rwanda	WEST	95-99	211.09	225.58	197.29	HT-Direct
Rwanda	WEST	95-99	207.40	195.23	221.24	RW2
Rwanda	WEST	00-04	132.96	144.04	122.62	HT-Direct
Rwanda	WEST	00-04	139.67	130.25	149.46	RW2
Rwanda	WEST	05-09	79.71	88.47	71.74	HT-Direct
Rwanda	WEST	05-09	83.18	75.59	91.46	RW2
Rwanda	WEST	10-14	49.89	64.21	38.63	HT-Direct
Rwanda	WEST	10-14	53.68	42.91	67.61	RW2
Rwanda	WEST	15-19	34.80	10.79	106.65	RW2
Senegal	ALL	80-84	191.01	200.05	182.27	HT-Direct
Senegal	ALL	80-84	182.52	180.58	184.68	IHME
Senegal	ALL	80-84	192.12	183.26	201.29	RW2
Senegal	ALL	80-84	192.10	187.98	196.67	UN
Senegal	ALL	85-89	151.16	157.61	144.93	HT-Direct
Senegal	ALL	85-89	155.77	154.15	157.31	IHME
Senegal	ALL	85-89	157.11	150.44	163.99	RW2
Senegal	ALL	85-89	157.15	153.53	160.55	UN
Senegal	ALL	90-94	135.19	140.62	129.95	HT-Direct
Senegal	ALL	90-94	139.81	138.06	141.32	IHME
Senegal	ALL	90-94	139.54	134.03	145.23	RW2
Senegal	ALL	90-94	139.52	136.45	142.90	UN
Senegal	ALL	95-99	139.17	144.62	133.89	HT-Direct
Senegal	ALL	95-99	128.38	126.78	129.94	IHME
Senegal	ALL	95-99	142.37	136.89	148.01	RW2
Senegal	ALL	95-99	142.32	138.72	145.75	UN
Senegal	ALL	00-04	111.41	115.73	107.23	HT-Direct
Senegal	ALL	00-04	107.37	106.02	108.76	IHME
Senegal	ALL	00-04	119.97	115.18	124.97	RW2
Senegal	ALL	00-04	120.05	116.36	123.45	UN
Senegal	ALL	05-09	76.27	79.84	72.84	HT-Direct
Senegal	ALL	05-09	81.94	80.70	83.26	IHME
Senegal	ALL	05-09	82.31	78.33	86.47	RW2
Senegal	ALL	05-09	82.31	79.05	85.76	UN
Senegal	ALL	10-14	56.64	60.72	52.83	HT-Direct
Senegal	ALL	10-14	61.65	59.91	63.20	IHME

Continued on next page

Country	Region	Year	Median	Lower	Upper	Method
Senegal	ALL	10-14	56.69	52.88	60.73	RW2
Senegal	ALL	10-14	56.68	51.79	62.09	UN
Senegal	DAKAR	80-84	130.04	150.57	111.95	HT-Direct
Senegal	DAKAR	80-84	132.79	116.22	151.53	RW2
Senegal	DAKAR	85-89	106.71	122.05	93.09	HT-Direct
Senegal	DAKAR	85-89	109.60	99.28	120.97	RW2
Senegal	DAKAR	90-94	94.71	108.77	82.31	HT-Direct
Senegal	DAKAR	90-94	94.47	85.42	104.55	RW2
Senegal	DAKAR	95-99	92.34	109.25	77.82	HT-Direct
Senegal	DAKAR	95-99	96.15	86.12	106.86	RW2
Senegal	DAKAR	00-04	68.79	80.44	58.72	HT-Direct
Senegal	DAKAR	00-04	82.87	73.82	92.55	RW2
Senegal	DAKAR	05-09	60.75	71.50	51.52	HT-Direct
Senegal	DAKAR	05-09	57.68	50.70	65.67	RW2
Senegal	DAKAR	10-14	38.15	50.98	28.46	HT-Direct
Senegal	DAKAR	10-14	41.98	33.50	52.56	RW2
Senegal	DAKAR	15-19	31.52	12.15	78.43	RW2
Senegal	DIOURBEL	80-84	236.15	262.57	211.62	HT-Direct
Senegal	DIOURBEL	80-84	227.48	206.68	250.16	RW2
Senegal	DIOURBEL	85-89	171.17	188.97	154.73	HT-Direct
Senegal	DIOURBEL	85-89	185.31	172.20	198.81	RW2
Senegal	DIOURBEL	90-94	152.41	169.02	137.16	HT-Direct
Senegal	DIOURBEL	90-94	161.28	150.10	172.68	RW2
Senegal	DIOURBEL	95-99	168.65	184.77	153.66	HT-Direct
Senegal	DIOURBEL	95-99	168.94	158.04	180.43	RW2
Senegal	DIOURBEL	00-04	142.76	158.26	128.54	HT-Direct
Senegal	DIOURBEL	00-04	148.89	138.38	160.03	RW2
Senegal	DIOURBEL	05-09	92.69	103.97	82.52	HT-Direct
Senegal	DIOURBEL	05-09	103.31	94.70	112.68	RW2
Senegal	DIOURBEL	10-14	78.40	92.43	66.34	HT-Direct
Senegal	DIOURBEL	10-14	76.73	67.05	87.85	RW2
Senegal	DIOURBEL	15-19	59.26	25.40	133.82	RW2
Senegal	FATICK	80-84	209.94	236.79	185.40	HT-Direct
Senegal	FATICK	80-84	210.72	189.52	233.11	RW2
Senegal	FATICK	85-89	160.18	181.64	140.82	HT-Direct
Senegal	FATICK	85-89	173.90	160.05	187.99	RW2
Senegal	FATICK	90-94	148.13	164.58	133.07	HT-Direct
Senegal	FATICK	90-94	150.71	140.27	161.86	RW2
Senegal	FATICK	95-99	153.21	170.33	137.53	HT-Direct
Senegal	FATICK	95-99	153.33	142.79	165.04	RW2
Senegal	FATICK	00-04	123.84	138.37	110.64	HT-Direct
Senegal	FATICK	00-04	128.18	118.54	138.72	RW2
Senegal	FATICK	05-09	69.35	81.04	59.24	HT-Direct
Senegal	FATICK	05-09	82.25	74.01	91.21	RW2
Senegal	FATICK	10-14	56.96	70.94	45.59	HT-Direct
Senegal	FATICK	10-14	55.97	47.19	65.81	RW2
Senegal	FATICK	15-19	39.43	16.46	92.27	RW2
Senegal	KAOLACK	80-84	217.72	244.39	193.22	HT-Direct
Senegal	KAOLACK	80-84	209.29	189.28	231.19	RW2
Senegal	KAOLACK	85-89	159.66	175.79	144.75	HT-Direct
Senegal	KAOLACK	85-89	175.68	163.48	188.36	RW2
Senegal	KAOLACK	90-94	149.94	163.79	137.07	HT-Direct
Senegal	KAOLACK	90-94	155.12	145.50	165.05	RW2
Senegal	KAOLACK	95-99	157.77	170.41	145.90	HT-Direct
Senegal	KAOLACK	95-99	161.49	152.48	171.16	RW2
Senegal	KAOLACK	00-04	131.01	141.06	121.58	HT-Direct
Senegal	KAOLACK	00-04	138.30	130.37	147.04	RW2
Senegal	KAOLACK	05-09	85.24	91.98	78.95	HT-Direct
Senegal	KAOLACK	05-09	89.09	83.39	95.10	RW2
Senegal	KAOLACK	10-14	53.93	61.78	47.03	HT-Direct
Senegal	KAOLACK	10-14	58.83	52.06	65.95	RW2
Senegal	KAOLACK	15-19	40.08	17.01	91.12	RW2
Senegal	KOLDA	80-84	247.03	275.21	220.85	HT-Direct

Continued on next page

Country	Region	Year	Median	Lower	Upper	Method
Senegal	KOLDA	80-84	252.15	228.88	276.71	RW2
Senegal	KOLDA	85-89	224.04	242.06	206.99	HT-Direct
Senegal	KOLDA	85-89	225.14	211.10	239.75	RW2
Senegal	KOLDA	90-94	188.44	205.14	172.80	HT-Direct
Senegal	KOLDA	90-94	203.05	190.66	215.75	RW2
Senegal	KOLDA	95-99	209.79	224.41	195.87	HT-Direct
Senegal	KOLDA	95-99	214.57	203.17	226.35	RW2
Senegal	KOLDA	00-04	180.67	193.60	168.42	HT-Direct
Senegal	KOLDA	00-04	189.80	179.05	201.22	RW2
Senegal	KOLDA	05-09	115.91	127.45	105.28	HT-Direct
Senegal	KOLDA	05-09	128.97	119.48	139.09	RW2
Senegal	KOLDA	10-14	95.21	108.59	83.32	HT-Direct
Senegal	KOLDA	10-14	93.85	83.52	105.36	RW2
Senegal	KOLDA	15-19	71.32	30.48	160.04	RW2
Senegal	LOUGA	80-84	199.32	227.80	173.59	HT-Direct
Senegal	LOUGA	80-84	194.62	173.50	218.22	RW2
Senegal	LOUGA	85-89	152.39	174.65	132.51	HT-Direct
Senegal	LOUGA	85-89	153.63	140.91	167.35	RW2
Senegal	LOUGA	90-94	119.16	135.43	104.60	HT-Direct
Senegal	LOUGA	90-94	124.77	114.96	134.91	RW2
Senegal	LOUGA	95-99	118.54	135.93	103.10	HT-Direct
Senegal	LOUGA	95-99	121.45	110.89	132.04	RW2
Senegal	LOUGA	00-04	82.62	95.00	71.72	HT-Direct
Senegal	LOUGA	00-04	101.64	92.24	111.38	RW2
Senegal	LOUGA	05-09	59.03	71.27	48.78	HT-Direct
Senegal	LOUGA	05-09	69.30	62.21	77.05	RW2
Senegal	LOUGA	10-14	61.26	73.01	51.30	HT-Direct
Senegal	LOUGA	10-14	52.40	45.00	61.35	RW2
Senegal	LOUGA	15-19	41.67	17.70	96.51	RW2
Senegal	MATAM	80-84	201.37	233.88	172.36	HT-Direct
Senegal	MATAM	80-84	228.04	200.38	255.59	RW2
Senegal	MATAM	85-89	194.80	212.37	178.35	HT-Direct
Senegal	MATAM	85-89	192.64	179.12	207.27	RW2
Senegal	MATAM	90-94	159.03	178.99	140.91	HT-Direct
Senegal	MATAM	90-94	157.47	145.81	170.81	RW2
Senegal	MATAM	95-99	136.28	155.16	119.38	HT-Direct
Senegal	MATAM	95-99	145.67	134.22	157.82	RW2
Senegal	MATAM	00-04	107.19	121.34	94.51	HT-Direct
Senegal	MATAM	00-04	114.60	104.39	125.10	RW2
Senegal	MATAM	05-09	60.24	71.25	50.84	HT-Direct
Senegal	MATAM	05-09	72.73	64.90	81.31	RW2
Senegal	MATAM	10-14	56.86	71.00	45.39	HT-Direct
Senegal	MATAM	10-14	51.48	43.30	61.51	RW2
Senegal	MATAM	15-19	38.45	16.01	90.88	RW2
Senegal	SAINT-LOUIS	80-84	192.57	221.99	166.22	HT-Direct
Senegal	SAINT-LOUIS	80-84	205.73	181.84	231.75	RW2
Senegal	SAINT-LOUIS	85-89	176.11	199.47	154.95	HT-Direct
Senegal	SAINT-LOUIS	85-89	162.46	148.07	178.50	RW2
Senegal	SAINT-LOUIS	90-94	118.09	135.36	102.75	HT-Direct
Senegal	SAINT-LOUIS	90-94	124.61	113.82	136.38	RW2
Senegal	SAINT-LOUIS	95-99	98.69	114.09	85.18	HT-Direct
Senegal	SAINT-LOUIS	95-99	112.73	101.87	124.00	RW2
Senegal	SAINT-LOUIS	00-04	86.69	100.09	74.93	HT-Direct
Senegal	SAINT-LOUIS	00-04	90.53	81.23	100.36	RW2
Senegal	SAINT-LOUIS	05-09	57.03	69.04	47.00	HT-Direct
Senegal	SAINT-LOUIS	05-09	60.16	52.78	68.56	RW2
Senegal	SAINT-LOUIS	10-14	46.09	60.77	34.83	HT-Direct
Senegal	SAINT-LOUIS	10-14	44.86	36.16	56.25	RW2
Senegal	SAINT-LOUIS	15-19	35.51	14.29	86.98	RW2
Senegal	TAMBACOUNDA	80-84	212.46	249.52	179.59	HT-Direct
Senegal	TAMBACOUNDA	80-84	223.42	195.51	252.55	RW2
Senegal	TAMBACOUNDA	85-89	198.62	225.39	174.32	HT-Direct
Senegal	TAMBACOUNDA	85-89	206.24	189.77	223.70	RW2

Continued on next page

Country	Region	Year	Median	Lower	Upper	Method
Senegal	TAMBACOUNDA	90-94	189.97	208.21	172.98	HT-Direct
Senegal	TAMBACOUNDA	90-94	191.84	179.44	205.17	RW2
Senegal	TAMBACOUNDA	95-99	201.03	221.68	181.86	HT-Direct
Senegal	TAMBACOUNDA	95-99	203.63	191.27	216.85	RW2
Senegal	TAMBACOUNDA	00-04	167.25	181.35	154.05	HT-Direct
Senegal	TAMBACOUNDA	00-04	180.39	169.50	191.96	RW2
Senegal	TAMBACOUNDA	05-09	116.59	129.97	104.43	HT-Direct
Senegal	TAMBACOUNDA	05-09	125.08	115.59	135.22	RW2
Senegal	TAMBACOUNDA	10-14	90.71	104.72	78.42	HT-Direct
Senegal	TAMBACOUNDA	10-14	92.31	81.82	103.80	RW2
Senegal	TAMBACOUNDA	15-19	70.67	30.65	155.70	RW2
Senegal	THIES	80-84	165.76	192.82	141.84	HT-Direct
Senegal	THIES	80-84	159.01	139.86	180.45	RW2
Senegal	THIES	85-89	107.64	126.01	91.66	HT-Direct
Senegal	THIES	85-89	125.57	113.88	138.21	RW2
Senegal	THIES	90-94	102.53	116.56	90.02	HT-Direct
Senegal	THIES	90-94	105.30	96.66	114.54	RW2
Senegal	THIES	95-99	108.17	123.43	94.59	HT-Direct
Senegal	THIES	95-99	105.58	97.00	115.03	RW2
Senegal	THIES	00-04	76.93	88.51	66.75	HT-Direct
Senegal	THIES	00-04	87.46	79.57	96.14	RW2
Senegal	THIES	05-09	60.40	72.47	50.24	HT-Direct
Senegal	THIES	05-09	56.43	49.84	63.91	RW2
Senegal	THIES	10-14	31.22	41.39	23.49	HT-Direct
Senegal	THIES	10-14	38.47	31.43	46.69	RW2
Senegal	THIES	15-19	27.17	11.06	64.87	RW2
Senegal	ZUGUINCHOR	80-84	215.24	258.63	177.38	HT-Direct
Senegal	ZUGUINCHOR	80-84	210.13	178.39	246.09	RW2
Senegal	ZUGUINCHOR	85-89	155.69	183.78	131.21	HT-Direct
Senegal	ZUGUINCHOR	85-89	173.96	155.40	194.10	RW2
Senegal	ZUGUINCHOR	90-94	146.86	167.08	128.72	HT-Direct
Senegal	ZUGUINCHOR	90-94	147.17	133.75	161.66	RW2
Senegal	ZUGUINCHOR	95-99	142.88	160.92	126.55	HT-Direct
Senegal	ZUGUINCHOR	95-99	145.68	133.27	159.23	RW2
Senegal	ZUGUINCHOR	00-04	108.17	124.43	93.80	HT-Direct
Senegal	ZUGUINCHOR	00-04	117.46	105.73	130.36	RW2
Senegal	ZUGUINCHOR	05-09	64.92	80.18	52.40	HT-Direct
Senegal	ZUGUINCHOR	05-09	71.20	61.30	82.60	RW2
Senegal	ZUGUINCHOR	10-14	46.67	65.19	33.22	HT-Direct
Senegal	ZUGUINCHOR	10-14	46.79	36.15	60.32	RW2
Senegal	ZUGUINCHOR	15-19	32.37	12.32	83.21	RW2
Sierra Leone	ALL	80-84	279.46	333.49	231.15	HT-Direct
Sierra Leone	ALL	80-84	270.31	265.06	276.10	IHME
Sierra Leone	ALL	80-84	277.79	231.92	328.82	RW2
Sierra Leone	ALL	80-84	281.41	268.54	294.89	UN
Sierra Leone	ALL	85-89	288.50	323.94	255.47	HT-Direct
Sierra Leone	ALL	85-89	256.50	252.65	260.16	IHME
Sierra Leone	ALL	85-89	271.75	244.82	300.40	RW2
Sierra Leone	ALL	85-89	268.02	258.06	278.78	UN
Sierra Leone	ALL	90-94	249.46	269.94	230.05	HT-Direct
Sierra Leone	ALL	90-94	238.75	235.46	241.96	IHME
Sierra Leone	ALL	90-94	261.95	242.13	282.77	RW2
Sierra Leone	ALL	90-94	262.55	253.37	271.50	UN
Sierra Leone	ALL	95-99	228.30	245.06	212.36	HT-Direct
Sierra Leone	ALL	95-99	221.12	218.35	224.35	IHME
Sierra Leone	ALL	95-99	249.36	231.48	268.06	RW2
Sierra Leone	ALL	95-99	250.10	242.01	258.61	UN
Sierra Leone	ALL	00-04	217.99	232.00	204.61	HT-Direct
Sierra Leone	ALL	00-04	198.59	196.17	201.45	IHME
Sierra Leone	ALL	00-04	223.42	210.05	237.42	RW2
Sierra Leone	ALL	00-04	223.39	216.57	230.81	UN
Sierra Leone	ALL	05-09	192.05	202.60	181.93	HT-Direct
Sierra Leone	ALL	05-09	168.80	165.82	171.71	IHME

Continued on next page

Country	Region	Year	Median	Lower	Upper	Method
Sierra Leone	ALL	05-09	187.88	178.30	197.86	RW2
Sierra Leone	ALL	05-09	187.57	180.45	194.59	UN
Sierra Leone	ALL	10-14	130.86	141.31	121.08	HT-Direct
Sierra Leone	ALL	10-14	138.76	135.08	142.62	IHME
Sierra Leone	ALL	10-14	142.03	130.56	154.24	RW2
Sierra Leone	ALL	10-14	142.26	134.04	150.47	UN
Sierra Leone	EASTERN	80-84	328.65	414.01	253.28	HT-Direct
Sierra Leone	EASTERN	80-84	322.49	259.38	391.71	RW2
Sierra Leone	EASTERN	85-89	337.21	415.51	266.94	HT-Direct
Sierra Leone	EASTERN	85-89	324.56	282.23	370.03	RW2
Sierra Leone	EASTERN	90-94	303.14	338.63	269.86	HT-Direct
Sierra Leone	EASTERN	90-94	319.95	290.98	350.38	RW2
Sierra Leone	EASTERN	95-99	285.39	318.36	254.56	HT-Direct
Sierra Leone	EASTERN	95-99	303.36	277.27	331.01	RW2
Sierra Leone	EASTERN	00-04	253.79	281.40	228.03	HT-Direct
Sierra Leone	EASTERN	00-04	265.75	244.82	288.18	RW2
Sierra Leone	EASTERN	05-09	222.90	247.65	199.97	HT-Direct
Sierra Leone	EASTERN	05-09	214.07	196.72	232.58	RW2
Sierra Leone	EASTERN	10-14	139.96	161.80	120.64	HT-Direct
Sierra Leone	EASTERN	10-14	154.52	133.82	177.30	RW2
Sierra Leone	EASTERN	15-19	105.86	46.08	223.52	RW2
Sierra Leone	NORTHERN	80-84	172.52	239.84	121.08	HT-Direct
Sierra Leone	NORTHERN	80-84	205.16	156.77	261.83	RW2
Sierra Leone	NORTHERN	85-89	251.41	307.73	202.38	HT-Direct
Sierra Leone	NORTHERN	85-89	223.12	191.99	257.35	RW2
Sierra Leone	NORTHERN	90-94	221.01	251.41	193.34	HT-Direct
Sierra Leone	NORTHERN	90-94	230.58	207.44	255.92	RW2
Sierra Leone	NORTHERN	95-99	214.10	240.19	190.13	HT-Direct
Sierra Leone	NORTHERN	95-99	225.61	205.22	247.67	RW2
Sierra Leone	NORTHERN	00-04	190.87	213.22	170.35	HT-Direct
Sierra Leone	NORTHERN	00-04	205.66	189.03	223.37	RW2
Sierra Leone	NORTHERN	05-09	183.47	199.37	168.57	HT-Direct
Sierra Leone	NORTHERN	05-09	176.48	164.40	189.29	RW2
Sierra Leone	NORTHERN	10-14	124.95	141.90	109.77	HT-Direct
Sierra Leone	NORTHERN	10-14	138.08	121.87	155.92	RW2
Sierra Leone	NORTHERN	15-19	103.85	46.29	216.34	RW2
Sierra Leone	SOUTHERN	80-84	363.86	490.77	253.43	HT-Direct
Sierra Leone	SOUTHERN	80-84	328.15	253.86	411.73	RW2
Sierra Leone	SOUTHERN	85-89	311.62	379.99	250.58	HT-Direct
Sierra Leone	SOUTHERN	85-89	317.25	274.63	362.03	RW2
Sierra Leone	SOUTHERN	90-94	291.24	328.34	256.73	HT-Direct
Sierra Leone	SOUTHERN	90-94	302.78	274.29	332.66	RW2
Sierra Leone	SOUTHERN	95-99	251.08	278.09	225.87	HT-Direct
Sierra Leone	SOUTHERN	95-99	281.74	258.59	306.34	RW2
Sierra Leone	SOUTHERN	00-04	258.84	286.52	232.96	HT-Direct
Sierra Leone	SOUTHERN	00-04	243.86	224.97	264.87	RW2
Sierra Leone	SOUTHERN	05-09	186.96	203.68	171.32	HT-Direct
Sierra Leone	SOUTHERN	05-09	189.42	176.28	203.42	RW2
Sierra Leone	SOUTHERN	10-14	125.42	144.05	108.90	HT-Direct
Sierra Leone	SOUTHERN	10-14	133.93	116.97	152.52	RW2
Sierra Leone	SOUTHERN	15-19	90.86	39.57	193.15	RW2
Sierra Leone	WESTERN	80-84	318.37	506.39	175.35	HT-Direct
Sierra Leone	WESTERN	80-84	244.26	158.87	370.67	RW2
Sierra Leone	WESTERN	85-89	250.80	346.08	174.75	HT-Direct
Sierra Leone	WESTERN	85-89	217.07	168.09	277.45	RW2
Sierra Leone	WESTERN	90-94	172.34	251.84	114.11	HT-Direct
Sierra Leone	WESTERN	90-94	191.67	152.50	234.99	RW2
Sierra Leone	WESTERN	95-99	123.36	173.41	86.26	HT-Direct
Sierra Leone	WESTERN	95-99	175.31	138.35	213.90	RW2
Sierra Leone	WESTERN	00-04	152.96	193.32	119.78	HT-Direct
Sierra Leone	WESTERN	00-04	165.93	138.02	195.69	RW2
Sierra Leone	WESTERN	05-09	165.60	201.26	135.18	HT-Direct
Sierra Leone	WESTERN	05-09	158.17	136.82	182.13	RW2

Continued on next page

Country	Region	Year	Median	Lower	Upper	Method
Sierra Leone	WESTERN	10-14	142.23	180.87	110.72	HT-Direct
Sierra Leone	WESTERN	10-14	144.12	113.92	183.57	RW2
Sierra Leone	WESTERN	15-19	128.75	53.45	284.57	RW2
Tanzania	ALL	80-84	170.57	182.00	159.72	HT-Direct
Tanzania	ALL	80-84	171.36	169.32	173.54	IHME
Tanzania	ALL	80-84	179.37	167.40	192.00	RW2
Tanzania	ALL	80-84	179.27	174.32	184.40	UN
Tanzania	ALL	85-89	163.24	172.25	154.60	HT-Direct
Tanzania	ALL	85-89	161.41	159.64	163.27	IHME
Tanzania	ALL	85-89	171.57	162.25	181.27	RW2
Tanzania	ALL	85-89	171.80	167.30	176.39	UN
Tanzania	ALL	90-94	162.13	169.55	154.97	HT-Direct
Tanzania	ALL	90-94	150.46	148.53	152.19	IHME
Tanzania	ALL	90-94	162.73	155.68	170.03	RW2
Tanzania	ALL	90-94	162.57	158.29	166.92	UN
Tanzania	ALL	95-99	148.63	155.48	142.03	HT-Direct
Tanzania	ALL	95-99	134.95	133.30	136.87	IHME
Tanzania	ALL	95-99	149.01	142.48	155.75	RW2
Tanzania	ALL	95-99	149.00	144.89	152.97	UN
Tanzania	ALL	00-04	113.41	119.51	107.58	HT-Direct
Tanzania	ALL	00-04	110.67	108.67	112.55	IHME
Tanzania	ALL	00-04	114.16	108.43	120.18	RW2
Tanzania	ALL	00-04	114.33	110.51	118.20	UN
Tanzania	ALL	05-09	87.26	93.37	81.52	HT-Direct
Tanzania	ALL	05-09	87.55	85.11	89.87	IHME
Tanzania	ALL	05-09	79.67	74.92	84.69	RW2
Tanzania	ALL	05-09	79.59	75.50	84.15	UN
Tanzania	ALL	10-14	70.14	77.35	63.55	HT-Direct
Tanzania	ALL	10-14	69.44	66.37	72.79	IHME
Tanzania	ALL	10-14	56.63	52.16	61.43	RW2
Tanzania	ALL	10-14	56.64	50.70	63.71	UN
Tanzania	ARUSHA	80-84	93.90	124.83	70.02	HT-Direct
Tanzania	ARUSHA	80-84	109.75	87.20	137.40	RW2
Tanzania	ARUSHA	85-89	103.58	136.04	78.17	HT-Direct
Tanzania	ARUSHA	85-89	104.30	88.79	122.15	RW2
Tanzania	ARUSHA	90-94	97.58	121.12	78.21	HT-Direct
Tanzania	ARUSHA	90-94	97.11	85.27	110.45	RW2
Tanzania	ARUSHA	95-99	86.27	106.67	69.47	HT-Direct
Tanzania	ARUSHA	95-99	87.40	77.15	98.84	RW2
Tanzania	ARUSHA	00-04	56.77	71.58	44.87	HT-Direct
Tanzania	ARUSHA	00-04	65.75	57.52	75.08	RW2
Tanzania	ARUSHA	05-09	57.41	75.99	43.16	HT-Direct
Tanzania	ARUSHA	05-09	43.77	37.06	51.75	RW2
Tanzania	ARUSHA	10-14	27.76	43.03	17.81	HT-Direct
Tanzania	ARUSHA	10-14	29.57	22.82	38.12	RW2
Tanzania	ARUSHA	15-19	20.21	8.29	48.48	RW2
Tanzania	DAR ES SALAAM	80-84	183.66	223.51	149.54	HT-Direct
Tanzania	DAR ES SALAAM	80-84	199.32	164.11	240.00	RW2
Tanzania	DAR ES SALAAM	85-89	194.14	235.18	158.77	HT-Direct
Tanzania	DAR ES SALAAM	85-89	175.21	151.66	202.78	RW2
Tanzania	DAR ES SALAAM	90-94	127.47	155.26	104.04	HT-Direct
Tanzania	DAR ES SALAAM	90-94	141.76	122.63	162.60	RW2
Tanzania	DAR ES SALAAM	95-99	117.17	144.47	94.46	HT-Direct
Tanzania	DAR ES SALAAM	95-99	123.94	106.22	143.51	RW2
Tanzania	DAR ES SALAAM	00-04	107.46	141.23	81.01	HT-Direct
Tanzania	DAR ES SALAAM	00-04	105.29	88.73	124.39	RW2
Tanzania	DAR ES SALAAM	05-09	102.43	135.42	76.77	HT-Direct
Tanzania	DAR ES SALAAM	05-09	86.90	73.42	102.98	RW2
Tanzania	DAR ES SALAAM	10-14	91.88	114.80	73.17	HT-Direct
Tanzania	DAR ES SALAAM	10-14	74.88	62.54	89.42	RW2
Tanzania	DAR ES SALAAM	15-19	65.16	26.87	152.11	RW2
Tanzania	DODOMA	80-84	183.08	233.02	141.86	HT-Direct
Tanzania	DODOMA	80-84	230.37	189.48	276.22	RW2

Continued on next page

Country	Region	Year	Median	Lower	Upper	Method
Tanzania	DODOMA	85-89	240.05	280.97	203.41	HT-Direct
Tanzania	DODOMA	85-89	225.18	199.16	254.00	RW2
Tanzania	DODOMA	90-94	225.95	273.26	184.74	HT-Direct
Tanzania	DODOMA	90-94	211.45	189.07	236.33	RW2
Tanzania	DODOMA	95-99	187.08	221.43	156.99	HT-Direct
Tanzania	DODOMA	95-99	185.60	165.77	207.35	RW2
Tanzania	DODOMA	00-04	119.29	147.69	95.74	HT-Direct
Tanzania	DODOMA	00-04	134.97	118.29	153.29	RW2
Tanzania	DODOMA	05-09	91.26	123.75	66.64	HT-Direct
Tanzania	DODOMA	05-09	89.20	75.50	105.14	RW2
Tanzania	DODOMA	10-14	92.59	136.52	61.78	HT-Direct
Tanzania	DODOMA	10-14	61.17	47.16	79.02	RW2
Tanzania	DODOMA	15-19	42.32	17.31	99.64	RW2
Tanzania	IRINGA	80-84	226.04	267.96	188.99	HT-Direct
Tanzania	IRINGA	80-84	218.04	184.75	256.35	RW2
Tanzania	IRINGA	85-89	156.36	201.94	119.52	HT-Direct
Tanzania	IRINGA	85-89	191.20	168.28	216.02	RW2
Tanzania	IRINGA	90-94	174.93	204.04	149.20	HT-Direct
Tanzania	IRINGA	90-94	170.88	152.72	190.14	RW2
Tanzania	IRINGA	95-99	126.82	154.57	103.44	HT-Direct
Tanzania	IRINGA	95-99	149.78	133.59	166.93	RW2
Tanzania	IRINGA	00-04	108.16	132.12	88.10	HT-Direct
Tanzania	IRINGA	00-04	111.44	99.07	125.22	RW2
Tanzania	IRINGA	05-09	98.20	119.20	80.56	HT-Direct
Tanzania	IRINGA	05-09	74.43	64.96	85.15	RW2
Tanzania	IRINGA	10-14	48.49	71.36	32.69	HT-Direct
Tanzania	IRINGA	10-14	49.85	39.76	62.51	RW2
Tanzania	IRINGA	15-19	33.37	13.80	78.08	RW2
Tanzania	KAGERA	80-84	203.38	253.49	161.04	HT-Direct
Tanzania	KAGERA	80-84	207.34	170.86	249.16	RW2
Tanzania	KAGERA	85-89	181.10	217.29	149.78	HT-Direct
Tanzania	KAGERA	85-89	194.37	171.28	219.78	RW2
Tanzania	KAGERA	90-94	173.29	204.11	146.28	HT-Direct
Tanzania	KAGERA	90-94	182.71	164.59	202.50	RW2
Tanzania	KAGERA	95-99	178.56	205.23	154.68	HT-Direct
Tanzania	KAGERA	95-99	168.98	152.69	186.73	RW2
Tanzania	KAGERA	00-04	126.88	156.97	101.87	HT-Direct
Tanzania	KAGERA	00-04	131.47	116.34	148.75	RW2
Tanzania	KAGERA	05-09	95.49	125.77	71.90	HT-Direct
Tanzania	KAGERA	05-09	88.45	74.99	104.14	RW2
Tanzania	KAGERA	10-14	73.16	115.66	45.47	HT-Direct
Tanzania	KAGERA	10-14	59.42	44.95	77.82	RW2
Tanzania	KAGERA	15-19	40.08	15.99	97.72	RW2
Tanzania	KIGOMA	80-84	175.48	238.27	126.48	HT-Direct
Tanzania	KIGOMA	80-84	207.22	164.67	256.53	RW2
Tanzania	KIGOMA	85-89	187.69	224.26	155.88	HT-Direct
Tanzania	KIGOMA	85-89	192.80	168.20	220.18	RW2
Tanzania	KIGOMA	90-94	187.56	221.91	157.45	HT-Direct
Tanzania	KIGOMA	90-94	178.54	159.24	199.79	RW2
Tanzania	KIGOMA	95-99	137.33	171.51	109.07	HT-Direct
Tanzania	KIGOMA	95-99	158.12	140.28	178.21	RW2
Tanzania	KIGOMA	00-04	123.11	149.15	101.08	HT-Direct
Tanzania	KIGOMA	00-04	117.09	102.64	133.35	RW2
Tanzania	KIGOMA	05-09	91.19	122.40	67.33	HT-Direct
Tanzania	KIGOMA	05-09	76.34	63.84	91.04	RW2
Tanzania	KIGOMA	10-14	47.76	82.77	27.12	HT-Direct
Tanzania	KIGOMA	10-14	50.64	37.29	68.00	RW2
Tanzania	KIGOMA	15-19	33.95	13.24	83.70	RW2
Tanzania	KILIMANJARO	80-84	119.76	155.32	91.45	HT-Direct
Tanzania	KILIMANJARO	80-84	107.24	83.79	137.03	RW2
Tanzania	KILIMANJARO	85-89	75.89	97.84	58.54	HT-Direct
Tanzania	KILIMANJARO	85-89	87.98	74.19	104.11	RW2
Tanzania	KILIMANJARO	90-94	71.65	91.95	55.56	HT-Direct

Continued on next page

Country	Region	Year	Median	Lower	Upper	Method
Tanzania	KILIMANJARO	90-94	74.21	62.13	87.58	RW2
Tanzania	KILIMANJARO	95-99	48.75	68.41	34.52	HT-Direct
Tanzania	KILIMANJARO	95-99	66.94	54.85	80.49	RW2
Tanzania	KILIMANJARO	00-04	71.32	98.50	51.22	HT-Direct
Tanzania	KILIMANJARO	00-04	56.03	45.21	69.27	RW2
Tanzania	KILIMANJARO	05-09	41.55	77.52	21.88	HT-Direct
Tanzania	KILIMANJARO	05-09	43.41	32.06	58.84	RW2
Tanzania	KILIMANJARO	10-14	49.22	108.66	21.51	HT-Direct
Tanzania	KILIMANJARO	10-14	34.77	21.72	56.64	RW2
Tanzania	KILIMANJARO	15-19	28.48	9.56	84.70	RW2
Tanzania	LINDI	80-84	228.32	270.63	190.89	HT-Direct
Tanzania	LINDI	80-84	247.58	210.16	288.06	RW2
Tanzania	LINDI	85-89	241.50	289.26	199.42	HT-Direct
Tanzania	LINDI	85-89	255.11	227.49	284.76	RW2
Tanzania	LINDI	90-94	257.03	299.28	218.88	HT-Direct
Tanzania	LINDI	90-94	251.15	227.45	276.77	RW2
Tanzania	LINDI	95-99	228.34	260.03	199.47	HT-Direct
Tanzania	LINDI	95-99	223.88	202.77	247.08	RW2
Tanzania	LINDI	00-04	151.11	191.98	117.67	HT-Direct
Tanzania	LINDI	00-04	154.45	135.49	175.54	RW2
Tanzania	LINDI	05-09	100.97	135.81	74.30	HT-Direct
Tanzania	LINDI	05-09	89.85	74.16	108.20	RW2
Tanzania	LINDI	10-14	55.71	101.48	29.89	HT-Direct
Tanzania	LINDI	10-14	52.18	37.29	71.82	RW2
Tanzania	LINDI	15-19	30.21	11.38	77.02	RW2
Tanzania	MARA	80-84	194.42	244.65	152.42	HT-Direct
Tanzania	MARA	80-84	207.16	170.67	249.27	RW2
Tanzania	MARA	85-89	211.38	250.79	176.71	HT-Direct
Tanzania	MARA	85-89	201.00	178.51	225.60	RW2
Tanzania	MARA	90-94	180.99	204.33	159.78	HT-Direct
Tanzania	MARA	90-94	194.69	177.89	212.40	RW2
Tanzania	MARA	95-99	187.63	216.05	162.17	HT-Direct
Tanzania	MARA	95-99	188.54	172.15	206.19	RW2
Tanzania	MARA	00-04	168.89	195.15	145.52	HT-Direct
Tanzania	MARA	00-04	155.57	140.53	172.42	RW2
Tanzania	MARA	05-09	121.70	149.45	98.51	HT-Direct
Tanzania	MARA	05-09	108.38	95.27	123.19	RW2
Tanzania	MARA	10-14	80.97	112.97	57.45	HT-Direct
Tanzania	MARA	10-14	73.00	58.34	90.47	RW2
Tanzania	MARA	15-19	48.64	20.09	112.28	RW2
Tanzania	MBEYA	80-84	145.34	184.21	113.54	HT-Direct
Tanzania	MBEYA	80-84	151.04	122.02	185.29	RW2
Tanzania	MBEYA	85-89	127.93	163.27	99.32	HT-Direct
Tanzania	MBEYA	85-89	149.79	129.11	173.03	RW2
Tanzania	MBEYA	90-94	168.16	204.57	137.12	HT-Direct
Tanzania	MBEYA	90-94	150.80	132.79	170.97	RW2
Tanzania	MBEYA	95-99	140.71	173.24	113.46	HT-Direct
Tanzania	MBEYA	95-99	144.93	127.84	163.91	RW2
Tanzania	MBEYA	00-04	114.27	141.01	92.06	HT-Direct
Tanzania	MBEYA	00-04	116.52	101.71	133.06	RW2
Tanzania	MBEYA	05-09	87.43	121.73	62.11	HT-Direct
Tanzania	MBEYA	05-09	86.27	72.78	102.09	RW2
Tanzania	MBEYA	10-14	90.23	132.68	60.41	HT-Direct
Tanzania	MBEYA	10-14	67.71	52.26	87.27	RW2
Tanzania	MBEYA	15-19	54.13	22.22	126.50	RW2
Tanzania	MOROGORO	80-84	231.80	283.50	187.07	HT-Direct
Tanzania	MOROGORO	80-84	219.87	184.45	259.66	RW2
Tanzania	MOROGORO	85-89	191.27	226.30	160.53	HT-Direct
Tanzania	MOROGORO	85-89	210.86	187.75	235.96	RW2
Tanzania	MOROGORO	90-94	167.27	197.22	141.08	HT-Direct
Tanzania	MOROGORO	90-94	198.78	180.36	218.52	RW2
Tanzania	MOROGORO	95-99	212.36	242.61	184.96	HT-Direct
Tanzania	MOROGORO	95-99	179.86	162.56	198.65	RW2

Continued on next page

Country	Region	Year	Median	Lower	Upper	Method
Tanzania	MOROGORO	00-04	110.90	151.60	80.09	HT-Direct
Tanzania	MOROGORO	00-04	130.67	114.05	149.55	RW2
Tanzania	MOROGORO	05-09	87.42	122.40	61.74	HT-Direct
Tanzania	MOROGORO	05-09	82.21	67.96	99.19	RW2
Tanzania	MOROGORO	10-14	62.52	107.36	35.67	HT-Direct
Tanzania	MOROGORO	10-14	52.10	38.57	69.65	RW2
Tanzania	MOROGORO	15-19	33.01	13.21	80.37	RW2
Tanzania	MTWARA	80-84	193.03	238.22	154.67	HT-Direct
Tanzania	MTWARA	80-84	200.55	164.21	242.11	RW2
Tanzania	MTWARA	85-89	182.64	220.22	150.23	HT-Direct
Tanzania	MTWARA	85-89	213.97	188.17	241.87	RW2
Tanzania	MTWARA	90-94	237.53	272.91	205.44	HT-Direct
Tanzania	MTWARA	90-94	220.25	197.63	245.20	RW2
Tanzania	MTWARA	95-99	203.11	242.02	169.05	HT-Direct
Tanzania	MTWARA	95-99	196.27	174.30	220.53	RW2
Tanzania	MTWARA	00-04	114.76	143.66	91.05	HT-Direct
Tanzania	MTWARA	00-04	130.87	112.00	151.93	RW2
Tanzania	MTWARA	05-09	78.09	122.69	48.80	HT-Direct
Tanzania	MTWARA	05-09	75.74	60.47	94.12	RW2
Tanzania	MTWARA	10-14	64.52	109.40	37.27	HT-Direct
Tanzania	MTWARA	10-14	45.62	31.88	65.01	RW2
Tanzania	MTWARA	15-19	27.83	10.09	74.49	RW2
Tanzania	MWANZA	80-84	153.58	203.55	114.11	HT-Direct
Tanzania	MWANZA	80-84	179.07	143.07	221.77	RW2
Tanzania	MWANZA	85-89	170.33	202.90	142.06	HT-Direct
Tanzania	MWANZA	85-89	173.00	151.58	196.60	RW2
Tanzania	MWANZA	90-94	176.48	205.67	150.65	HT-Direct
Tanzania	MWANZA	90-94	163.78	147.53	181.55	RW2
Tanzania	MWANZA	95-99	135.66	156.48	117.21	HT-Direct
Tanzania	MWANZA	95-99	151.20	136.46	166.86	RW2
Tanzania	MWANZA	00-04	130.47	156.30	108.36	HT-Direct
Tanzania	MWANZA	00-04	121.07	107.96	135.72	RW2
Tanzania	MWANZA	05-09	91.34	113.06	73.45	HT-Direct
Tanzania	MWANZA	05-09	84.88	73.80	97.55	RW2
Tanzania	MWANZA	10-14	73.88	106.42	50.73	HT-Direct
Tanzania	MWANZA	10-14	59.60	46.94	75.37	RW2
Tanzania	MWANZA	15-19	42.03	16.97	100.13	RW2
Tanzania	PWANI	80-84	181.74	238.36	136.17	HT-Direct
Tanzania	PWANI	80-84	236.01	191.82	285.58	RW2
Tanzania	PWANI	85-89	261.14	303.19	223.06	HT-Direct
Tanzania	PWANI	85-89	220.93	194.78	249.53	RW2
Tanzania	PWANI	90-94	157.87	189.81	130.44	HT-Direct
Tanzania	PWANI	90-94	192.11	171.70	214.02	RW2
Tanzania	PWANI	95-99	174.53	209.38	144.41	HT-Direct
Tanzania	PWANI	95-99	163.94	145.59	183.63	RW2
Tanzania	PWANI	00-04	113.70	139.14	92.42	HT-Direct
Tanzania	PWANI	00-04	120.28	104.90	137.49	RW2
Tanzania	PWANI	05-09	81.29	121.76	53.46	HT-Direct
Tanzania	PWANI	05-09	80.82	66.52	98.05	RW2
Tanzania	PWANI	10-14	85.21	137.96	51.43	HT-Direct
Tanzania	PWANI	10-14	56.50	41.61	76.85	RW2
Tanzania	PWANI	15-19	40.17	15.78	99.52	RW2
Tanzania	RUKWA	80-84	232.14	290.25	182.67	HT-Direct
Tanzania	RUKWA	80-84	235.44	193.79	284.17	RW2
Tanzania	RUKWA	85-89	195.34	229.90	164.87	HT-Direct
Tanzania	RUKWA	85-89	204.70	182.09	229.68	RW2
Tanzania	RUKWA	90-94	179.72	203.26	158.36	HT-Direct
Tanzania	RUKWA	90-94	180.83	164.38	198.56	RW2
Tanzania	RUKWA	95-99	169.25	205.57	138.24	HT-Direct
Tanzania	RUKWA	95-99	155.79	139.59	173.36	RW2
Tanzania	RUKWA	00-04	106.35	124.23	90.77	HT-Direct
Tanzania	RUKWA	00-04	115.63	102.85	129.19	RW2
Tanzania	RUKWA	05-09	86.17	106.89	69.15	HT-Direct

Continued on next page

Country	Region	Year	Median	Lower	Upper	Method
Tanzania	RUKWA	05-09	83.24	73.05	94.61	RW2
Tanzania	RUKWA	10-14	93.70	122.01	71.42	HT-Direct
Tanzania	RUKWA	10-14	66.63	54.73	81.31	RW2
Tanzania	RUKWA	15-19	55.23	23.51	127.35	RW2
Tanzania	RUVUMA	80-84	159.66	209.12	120.13	HT-Direct
Tanzania	RUVUMA	80-84	147.41	116.94	184.18	RW2
Tanzania	RUVUMA	85-89	118.21	148.04	93.73	HT-Direct
Tanzania	RUVUMA	85-89	157.00	134.96	181.88	RW2
Tanzania	RUVUMA	90-94	183.01	224.41	147.80	HT-Direct
Tanzania	RUVUMA	90-94	166.26	146.64	188.20	RW2
Tanzania	RUVUMA	95-99	162.84	196.78	133.77	HT-Direct
Tanzania	RUVUMA	95-99	160.62	142.16	181.54	RW2
Tanzania	RUVUMA	00-04	129.48	159.50	104.41	HT-Direct
Tanzania	RUVUMA	00-04	119.77	104.20	137.16	RW2
Tanzania	RUVUMA	05-09	66.21	92.09	47.22	HT-Direct
Tanzania	RUVUMA	05-09	76.35	62.98	92.14	RW2
Tanzania	RUVUMA	10-14	68.63	121.95	37.62	HT-Direct
Tanzania	RUVUMA	10-14	49.89	35.80	68.20	RW2
Tanzania	RUVUMA	15-19	32.71	12.50	83.02	RW2
Tanzania	SHINYANGA	80-84	190.72	231.11	155.96	HT-Direct
Tanzania	SHINYANGA	80-84	188.39	159.50	221.84	RW2
Tanzania	SHINYANGA	85-89	162.95	193.74	136.22	HT-Direct
Tanzania	SHINYANGA	85-89	175.55	156.94	196.02	RW2
Tanzania	SHINYANGA	90-94	168.27	193.54	145.71	HT-Direct
Tanzania	SHINYANGA	90-94	165.21	150.62	180.61	RW2
Tanzania	SHINYANGA	95-99	147.13	167.96	128.48	HT-Direct
Tanzania	SHINYANGA	95-99	154.02	140.87	167.85	RW2
Tanzania	SHINYANGA	00-04	126.54	146.34	109.07	HT-Direct
Tanzania	SHINYANGA	00-04	124.18	113.03	136.30	RW2
Tanzania	SHINYANGA	05-09	93.72	113.56	77.04	HT-Direct
Tanzania	SHINYANGA	05-09	90.11	80.75	100.54	RW2
Tanzania	SHINYANGA	10-14	88.66	108.92	71.86	HT-Direct
Tanzania	SHINYANGA	10-14	67.39	57.98	78.31	RW2
Tanzania	SHINYANGA	15-19	51.09	22.59	111.27	RW2
Tanzania	SINGIDA	80-84	173.27	228.70	129.03	HT-Direct
Tanzania	SINGIDA	80-84	154.18	121.77	193.58	RW2
Tanzania	SINGIDA	85-89	92.39	123.10	68.74	HT-Direct
Tanzania	SINGIDA	85-89	140.68	119.82	164.12	RW2
Tanzania	SINGIDA	90-94	133.16	159.69	110.46	HT-Direct
Tanzania	SINGIDA	90-94	130.39	115.25	147.08	RW2
Tanzania	SINGIDA	95-99	121.56	148.91	98.64	HT-Direct
Tanzania	SINGIDA	95-99	116.24	102.55	131.66	RW2
Tanzania	SINGIDA	00-04	90.11	122.18	65.83	HT-Direct
Tanzania	SINGIDA	00-04	84.75	72.67	98.58	RW2
Tanzania	SINGIDA	05-09	58.54	82.86	41.04	HT-Direct
Tanzania	SINGIDA	05-09	54.32	44.28	66.53	RW2
Tanzania	SINGIDA	10-14	33.32	60.69	18.06	HT-Direct
Tanzania	SINGIDA	10-14	35.52	25.82	48.42	RW2
Tanzania	SINGIDA	15-19	23.51	9.22	59.03	RW2
Tanzania	TABORA	80-84	177.31	229.12	135.16	HT-Direct
Tanzania	TABORA	80-84	174.31	139.19	215.18	RW2
Tanzania	TABORA	85-89	129.93	168.42	99.19	HT-Direct
Tanzania	TABORA	85-89	165.19	142.16	190.87	RW2
Tanzania	TABORA	90-94	156.58	188.39	129.29	HT-Direct
Tanzania	TABORA	90-94	159.56	142.77	177.80	RW2
Tanzania	TABORA	95-99	157.20	178.14	138.32	HT-Direct
Tanzania	TABORA	95-99	147.37	133.92	162.04	RW2
Tanzania	TABORA	00-04	106.21	126.98	88.50	HT-Direct
Tanzania	TABORA	00-04	111.05	99.69	123.48	RW2
Tanzania	TABORA	05-09	78.88	98.17	63.11	HT-Direct
Tanzania	TABORA	05-09	74.86	65.31	85.71	RW2
Tanzania	TABORA	10-14	65.78	93.23	46.00	HT-Direct
Tanzania	TABORA	10-14	52.80	42.28	65.61	RW2

Continued on next page

Country	Region	Year	Median	Lower	Upper	Method
Tanzania	TABORA	15-19	37.82	15.76	87.85	RW2
Tanzania	TANGA	80-84	140.51	178.03	109.84	HT-Direct
Tanzania	TANGA	80-84	180.18	146.70	218.11	RW2
Tanzania	TANGA	85-89	197.98	237.71	163.47	HT-Direct
Tanzania	TANGA	85-89	181.83	160.82	205.39	RW2
Tanzania	TANGA	90-94	179.36	204.06	157.06	HT-Direct
Tanzania	TANGA	90-94	171.19	154.93	188.92	RW2
Tanzania	TANGA	95-99	131.61	159.45	108.01	HT-Direct
Tanzania	TANGA	95-99	151.25	135.28	168.88	RW2
Tanzania	TANGA	00-04	124.57	153.27	100.60	HT-Direct
Tanzania	TANGA	00-04	111.51	97.45	127.49	RW2
Tanzania	TANGA	05-09	71.88	99.55	51.46	HT-Direct
Tanzania	TANGA	05-09	71.48	59.27	86.28	RW2
Tanzania	TANGA	10-14	55.12	96.47	30.88	HT-Direct
Tanzania	TANGA	10-14	46.23	33.39	62.86	RW2
Tanzania	TANGA	15-19	29.98	11.47	75.66	RW2
Togo	ALL	80-84	177.38	192.44	163.26	HT-Direct
Togo	ALL	80-84	162.29	159.60	164.74	IHME
Togo	ALL	80-84	169.92	156.97	183.70	RW2
Togo	ALL	80-84	169.65	163.43	175.54	UN
Togo	ALL	85-89	153.65	165.35	142.64	HT-Direct
Togo	ALL	85-89	150.47	148.21	152.78	IHME
Togo	ALL	85-89	152.75	142.29	163.77	RW2
Togo	ALL	85-89	153.33	148.78	158.66	UN
Togo	ALL	90-94	150.87	160.27	141.92	HT-Direct
Togo	ALL	90-94	140.73	138.55	143.15	IHME
Togo	ALL	90-94	142.54	134.78	150.69	RW2
Togo	ALL	90-94	142.10	137.90	146.75	UN
Togo	ALL	95-99	133.40	143.17	124.21	HT-Direct
Togo	ALL	95-99	130.01	127.73	132.37	IHME
Togo	ALL	95-99	129.65	121.30	138.41	RW2
Togo	ALL	95-99	129.92	125.92	134.00	UN
Togo	ALL	00-04	114.83	126.25	104.32	HT-Direct
Togo	ALL	00-04	117.78	115.57	120.26	IHME
Togo	ALL	00-04	114.36	104.74	124.80	RW2
Togo	ALL	00-04	114.23	110.54	118.01	UN
Togo	ALL	05-09	97.69	107.39	88.78	HT-Direct
Togo	ALL	05-09	104.17	101.75	106.69	IHME
Togo	ALL	05-09	99.05	90.33	108.52	RW2
Togo	ALL	05-09	99.20	95.31	103.13	UN
Togo	ALL	10-14	86.51	96.89	77.15	HT-Direct
Togo	ALL	10-14	88.39	85.23	91.33	IHME
Togo	ALL	10-14	85.70	76.61	95.67	RW2
Togo	ALL	10-14	85.64	80.54	90.83	UN
Togo	CENTRALE	80-84	193.64	235.13	157.97	HT-Direct
Togo	CENTRALE	80-84	180.86	156.40	209.14	RW2
Togo	CENTRALE	85-89	165.58	193.29	141.14	HT-Direct
Togo	CENTRALE	85-89	156.78	140.99	173.81	RW2
Togo	CENTRALE	90-94	150.06	172.90	129.76	HT-Direct
Togo	CENTRALE	90-94	147.04	134.06	160.82	RW2
Togo	CENTRALE	95-99	127.34	152.62	105.74	HT-Direct
Togo	CENTRALE	95-99	134.78	121.12	148.94	RW2
Togo	CENTRALE	00-04	147.89	186.12	116.39	HT-Direct
Togo	CENTRALE	00-04	127.54	112.86	143.72	RW2
Togo	CENTRALE	05-09	104.04	126.91	84.89	HT-Direct
Togo	CENTRALE	05-09	115.96	101.64	132.25	RW2
Togo	CENTRALE	10-14	114.98	142.30	92.34	HT-Direct
Togo	CENTRALE	10-14	104.12	87.65	123.71	RW2
Togo	CENTRALE	15-19	93.28	40.99	198.68	RW2
Togo	GRANDE AGGLOMÉRATION DE LOMÉ	80-84	131.45	181.08	93.87	HT-Direct
Togo	GRANDE AGGLOMÉRATION DE LOMÉ	80-84	122.97	97.23	153.14	RW2
Togo	GRANDE AGGLOMÉRATION DE LOMÉ	85-89	100.95	129.16	78.35	HT-Direct
Togo	GRANDE AGGLOMÉRATION DE LOMÉ	85-89	107.76	92.25	125.57	RW2

Continued on next page

Country	Region	Year	Median	Lower	Upper	Method
Togo	GRANDE AGGLOMÉRATION DE LOMÉ	90-94	105.17	129.66	84.85	HT-Direct
Togo	GRANDE AGGLOMÉRATION DE LOMÉ	90-94	102.03	89.75	115.91	RW2
Togo	GRANDE AGGLOMÉRATION DE LOMÉ	95-99	105.65	131.83	84.16	HT-Direct
Togo	GRANDE AGGLOMÉRATION DE LOMÉ	95-99	91.29	80.18	104.41	RW2
Togo	GRANDE AGGLOMÉRATION DE LOMÉ	00-04	80.15	103.13	61.93	HT-Direct
Togo	GRANDE AGGLOMÉRATION DE LOMÉ	00-04	80.29	69.44	92.84	RW2
Togo	GRANDE AGGLOMÉRATION DE LOMÉ	05-09	61.64	79.11	47.82	HT-Direct
Togo	GRANDE AGGLOMÉRATION DE LOMÉ	05-09	67.19	56.91	79.00	RW2
Togo	GRANDE AGGLOMÉRATION DE LOMÉ	10-14	56.67	75.03	42.59	HT-Direct
Togo	GRANDE AGGLOMÉRATION DE LOMÉ	10-14	56.31	45.04	70.22	RW2
Togo	GRANDE AGGLOMÉRATION DE LOMÉ	15-19	47.35	19.71	109.85	RW2
Togo	KARA	80-84	195.98	238.72	159.29	HT-Direct
Togo	KARA	80-84	197.38	171.50	226.44	RW2
Togo	KARA	85-89	182.88	210.30	158.32	HT-Direct
Togo	KARA	85-89	174.61	158.34	192.28	RW2
Togo	KARA	90-94	182.71	206.33	161.24	HT-Direct
Togo	KARA	90-94	166.46	153.41	180.33	RW2
Togo	KARA	95-99	137.33	159.88	117.51	HT-Direct
Togo	KARA	95-99	152.98	139.37	167.54	RW2
Togo	KARA	00-04	158.44	196.44	126.63	HT-Direct
Togo	KARA	00-04	143.79	128.20	161.20	RW2
Togo	KARA	05-09	141.04	168.28	117.59	HT-Direct
Togo	KARA	05-09	127.65	112.77	144.34	RW2
Togo	KARA	10-14	102.27	124.39	83.70	HT-Direct
Togo	KARA	10-14	109.12	92.99	127.45	RW2
Togo	KARA	15-19	92.15	40.78	195.64	RW2
Togo	MARITIME (SANS AGGLOMÉRATION DE LOMÉ)	80-84	159.26	195.63	128.58	HT-Direct
Togo	MARITIME (SANS AGGLOMÉRATION DE LOMÉ)	80-84	161.95	137.42	189.61	RW2
Togo	MARITIME (SANS AGGLOMÉRATION DE LOMÉ)	85-89	154.28	192.27	122.66	HT-Direct
Togo	MARITIME (SANS AGGLOMÉRATION DE LOMÉ)	85-89	142.21	125.59	160.50	RW2
Togo	MARITIME (SANS AGGLOMÉRATION DE LOMÉ)	90-94	139.09	160.78	119.90	HT-Direct
Togo	MARITIME (SANS AGGLOMÉRATION DE LOMÉ)	90-94	134.08	121.53	148.01	RW2
Togo	MARITIME (SANS AGGLOMÉRATION DE LOMÉ)	95-99	142.11	169.17	118.75	HT-Direct
Togo	MARITIME (SANS AGGLOMÉRATION DE LOMÉ)	95-99	120.29	107.53	134.43	RW2
Togo	MARITIME (SANS AGGLOMÉRATION DE LOMÉ)	00-04	76.84	106.96	54.69	HT-Direct
Togo	MARITIME (SANS AGGLOMÉRATION DE LOMÉ)	00-04	107.04	92.06	124.03	RW2
Togo	MARITIME (SANS AGGLOMÉRATION DE LOMÉ)	05-09	79.65	112.58	55.74	HT-Direct
Togo	MARITIME (SANS AGGLOMÉRATION DE LOMÉ)	05-09	91.54	75.55	110.08	RW2
Togo	MARITIME (SANS AGGLOMÉRATION DE LOMÉ)	10-14	88.47	123.68	62.56	HT-Direct
Togo	MARITIME (SANS AGGLOMÉRATION DE LOMÉ)	10-14	78.37	60.89	100.40	RW2
Togo	MARITIME (SANS AGGLOMÉRATION DE LOMÉ)	15-19	67.60	28.23	153.30	RW2
Togo	PLATEAUX	80-84	180.33	210.53	153.63	HT-Direct
Togo	PLATEAUX	80-84	169.28	149.72	191.19	RW2
Togo	PLATEAUX	85-89	146.69	166.04	129.24	HT-Direct
Togo	PLATEAUX	85-89	146.56	133.74	160.48	RW2
Togo	PLATEAUX	90-94	146.52	169.98	125.81	HT-Direct
Togo	PLATEAUX	90-94	137.39	125.64	149.74	RW2
Togo	PLATEAUX	95-99	122.96	143.85	104.74	HT-Direct
Togo	PLATEAUX	95-99	124.53	112.85	137.00	RW2
Togo	PLATEAUX	00-04	116.97	140.22	97.13	HT-Direct
Togo	PLATEAUX	00-04	115.17	102.74	128.89	RW2
Togo	PLATEAUX	05-09	101.42	123.60	82.84	HT-Direct
Togo	PLATEAUX	05-09	102.82	89.98	117.20	RW2
Togo	PLATEAUX	10-14	95.34	121.10	74.59	HT-Direct
Togo	PLATEAUX	10-14	91.39	76.11	109.92	RW2
Togo	PLATEAUX	15-19	81.55	35.70	176.49	RW2
Togo	SAVANES	80-84	223.03	250.47	197.79	HT-Direct
Togo	SAVANES	80-84	210.24	189.68	232.44	RW2
Togo	SAVANES	85-89	171.90	194.50	151.44	HT-Direct
Togo	SAVANES	85-89	182.51	167.64	198.35	RW2
Togo	SAVANES	90-94	183.47	199.91	168.10	HT-Direct

Continued on next page

Country	Region	Year	Median	Lower	Upper	Method
Togo	SAVANES	90-94	171.59	160.42	183.46	RW2
Togo	SAVANES	95-99	165.85	187.96	145.88	HT-Direct
Togo	SAVANES	95-99	154.15	141.46	167.88	RW2
Togo	SAVANES	00-04	135.77	159.50	115.08	HT-Direct
Togo	SAVANES	00-04	138.35	124.24	154.18	RW2
Togo	SAVANES	05-09	117.79	140.33	98.46	HT-Direct
Togo	SAVANES	05-09	115.39	101.63	130.70	RW2
Togo	SAVANES	10-14	84.06	108.25	64.88	HT-Direct
Togo	SAVANES	10-14	91.60	74.68	110.83	RW2
Togo	SAVANES	15-19	71.78	30.07	158.96	RW2
Uganda	ALL	80-84	201.20	210.87	191.86	HT-Direct
Uganda	ALL	80-84	192.81	189.88	195.87	IHME
Uganda	ALL	80-84	209.25	199.14	219.74	RW2
Uganda	ALL	80-84	209.20	203.14	215.58	UN
Uganda	ALL	85-89	172.06	179.68	164.70	HT-Direct
Uganda	ALL	85-89	177.00	174.76	179.15	IHME
Uganda	ALL	85-89	192.10	182.98	201.52	RW2
Uganda	ALL	85-89	192.14	187.02	197.24	UN
Uganda	ALL	90-94	161.67	168.81	154.77	HT-Direct
Uganda	ALL	90-94	162.28	160.44	164.15	IHME
Uganda	ALL	90-94	179.53	171.17	188.21	RW2
Uganda	ALL	90-94	179.55	174.34	184.65	UN
Uganda	ALL	95-99	155.74	163.26	148.50	HT-Direct
Uganda	ALL	95-99	143.81	141.82	145.60	IHME
Uganda	ALL	95-99	163.10	155.34	171.11	RW2
Uganda	ALL	95-99	163.03	158.73	167.42	UN
Uganda	ALL	00-04	133.56	140.53	126.89	HT-Direct
Uganda	ALL	00-04	121.66	119.87	123.38	IHME
Uganda	ALL	00-04	132.13	125.76	138.81	RW2
Uganda	ALL	00-04	132.14	128.32	136.05	UN
Uganda	ALL	05-09	102.60	110.35	95.34	HT-Direct
Uganda	ALL	05-09	99.98	97.90	102.17	IHME
Uganda	ALL	05-09	92.64	86.77	98.85	RW2
Uganda	ALL	05-09	92.82	89.49	96.21	UN
Uganda	ALL	10-14	89.18	108.33	73.14	HT-Direct
Uganda	ALL	10-14	80.52	77.72	83.31	IHME
Uganda	ALL	10-14	66.06	56.63	76.87	RW2
Uganda	ALL	10-14	65.56	59.96	71.10	UN
Uganda	CENTRAL	80-84	199.34	216.78	182.98	HT-Direct
Uganda	CENTRAL	80-84	205.05	188.71	222.59	RW2
Uganda	CENTRAL	85-89	160.57	174.67	147.41	HT-Direct
Uganda	CENTRAL	85-89	179.52	167.05	192.61	RW2
Uganda	CENTRAL	90-94	141.10	153.63	129.44	HT-Direct
Uganda	CENTRAL	90-94	159.40	147.61	171.34	RW2
Uganda	CENTRAL	95-99	130.62	146.02	116.62	HT-Direct
Uganda	CENTRAL	95-99	143.28	132.09	154.87	RW2
Uganda	CENTRAL	00-04	121.10	134.73	108.68	HT-Direct
Uganda	CENTRAL	00-04	116.86	107.73	127.25	RW2
Uganda	CENTRAL	05-09	99.55	116.96	84.49	HT-Direct
Uganda	CENTRAL	05-09	80.79	72.01	90.69	RW2
Uganda	CENTRAL	10-14	49.81	76.54	32.10	HT-Direct
Uganda	CENTRAL	10-14	54.13	41.62	68.35	RW2
Uganda	CENTRAL	15-19	36.07	13.96	87.93	RW2
Uganda	EASTERN	80-84	206.16	224.77	188.72	HT-Direct
Uganda	EASTERN	80-84	219.33	201.13	238.52	RW2
Uganda	EASTERN	85-89	183.74	198.11	170.20	HT-Direct
Uganda	EASTERN	85-89	201.02	187.34	216.11	RW2
Uganda	EASTERN	90-94	164.58	178.53	151.51	HT-Direct
Uganda	EASTERN	90-94	175.65	163.41	189.44	RW2
Uganda	EASTERN	95-99	136.88	148.83	125.75	HT-Direct
Uganda	EASTERN	95-99	145.83	135.58	156.33	RW2
Uganda	EASTERN	00-04	108.13	119.40	97.81	HT-Direct
Uganda	EASTERN	00-04	112.33	103.32	121.50	RW2

Continued on next page

Country	Region	Year	Median	Lower	Upper	Method
Uganda	EASTERN	05-09	92.10	104.86	80.75	HT-Direct
Uganda	EASTERN	05-09	81.19	73.51	89.75	RW2
Uganda	EASTERN	10-14	92.51	133.51	63.18	HT-Direct
Uganda	EASTERN	10-14	61.75	48.99	79.03	RW2
Uganda	EASTERN	15-19	47.94	19.37	117.89	RW2
Uganda	NORTHERN	80-84	240.53	267.97	215.08	HT-Direct
Uganda	NORTHERN	80-84	249.69	225.48	275.59	RW2
Uganda	NORTHERN	85-89	207.01	226.98	188.36	HT-Direct
Uganda	NORTHERN	85-89	229.40	212.64	246.74	RW2
Uganda	NORTHERN	90-94	187.23	202.19	173.14	HT-Direct
Uganda	NORTHERN	90-94	210.86	197.25	225.08	RW2
Uganda	NORTHERN	95-99	183.51	198.25	169.62	HT-Direct
Uganda	NORTHERN	95-99	191.99	180.23	204.40	RW2
Uganda	NORTHERN	00-04	160.86	174.74	147.88	HT-Direct
Uganda	NORTHERN	00-04	155.41	145.46	166.10	RW2
Uganda	NORTHERN	05-09	113.60	130.90	98.32	HT-Direct
Uganda	NORTHERN	05-09	108.29	97.62	120.03	RW2
Uganda	NORTHERN	10-14	119.45	185.76	74.64	HT-Direct
Uganda	NORTHERN	10-14	77.15	60.67	97.50	RW2
Uganda	NORTHERN	15-19	55.68	22.33	132.93	RW2
Uganda	WESTERN	80-84	180.34	196.02	165.64	HT-Direct
Uganda	WESTERN	80-84	186.09	171.22	201.88	RW2
Uganda	WESTERN	85-89	157.84	171.68	144.92	HT-Direct
Uganda	WESTERN	85-89	180.95	167.60	194.57	RW2
Uganda	WESTERN	90-94	160.98	175.76	147.22	HT-Direct
Uganda	WESTERN	90-94	180.79	167.74	194.52	RW2
Uganda	WESTERN	95-99	178.83	195.93	162.91	HT-Direct
Uganda	WESTERN	95-99	177.12	164.32	191.62	RW2
Uganda	WESTERN	00-04	147.38	162.37	133.56	HT-Direct
Uganda	WESTERN	00-04	146.32	135.77	157.89	RW2
Uganda	WESTERN	05-09	106.91	122.47	93.13	HT-Direct
Uganda	WESTERN	05-09	101.81	91.88	112.39	RW2
Uganda	WESTERN	10-14	103.80	135.27	78.98	HT-Direct
Uganda	WESTERN	10-14	72.82	60.48	87.38	RW2
Uganda	WESTERN	15-19	52.94	22.33	121.53	RW2
Zambia	ALL	80-84	159.63	167.50	152.06	HT-Direct
Zambia	ALL	80-84	160.63	156.43	164.93	IHME
Zambia	ALL	80-84	162.11	154.29	170.24	RW2
Zambia	ALL	80-84	162.09	157.42	167.22	UN
Zambia	ALL	85-89	178.94	185.94	172.16	HT-Direct
Zambia	ALL	85-89	170.45	166.39	174.58	IHME
Zambia	ALL	85-89	183.49	176.41	190.76	RW2
Zambia	ALL	85-89	183.57	178.67	188.63	UN
Zambia	ALL	90-94	181.18	187.93	174.62	HT-Direct
Zambia	ALL	90-94	168.58	164.39	172.06	IHME
Zambia	ALL	90-94	188.48	181.48	195.69	RW2
Zambia	ALL	90-94	188.27	183.24	193.10	UN
Zambia	ALL	95-99	157.06	164.07	150.29	HT-Direct
Zambia	ALL	95-99	154.77	150.93	158.70	IHME
Zambia	ALL	95-99	174.79	166.50	183.32	RW2
Zambia	ALL	95-99	175.17	170.41	180.08	UN
Zambia	ALL	00-04	129.42	136.26	122.88	HT-Direct
Zambia	ALL	00-04	130.72	126.83	134.32	IHME
Zambia	ALL	00-04	142.23	134.50	150.41	RW2
Zambia	ALL	00-04	141.89	137.34	147.01	UN
Zambia	ALL	05-09	83.31	89.82	77.24	HT-Direct
Zambia	ALL	05-09	98.98	95.39	102.72	IHME
Zambia	ALL	05-09	98.44	90.12	107.34	RW2
Zambia	ALL	05-09	98.69	95.12	102.47	UN
Zambia	ALL	10-14	71.80	78.63	65.53	HT-Direct
Zambia	ALL	10-14	74.07	70.17	78.06	IHME
Zambia	ALL	10-14	74.47	67.75	81.74	RW2
Zambia	ALL	10-14	74.30	70.13	78.89	UN

Continued on next page

Country	Region	Year	Median	Lower	Upper	Method
Zambia	CENTRAL	80-84	139.03	166.84	115.21	HT-Direct
Zambia	CENTRAL	80-84	141.44	123.97	160.98	RW2
Zambia	CENTRAL	85-89	159.22	180.17	140.30	HT-Direct
Zambia	CENTRAL	85-89	162.15	148.34	176.89	RW2
Zambia	CENTRAL	90-94	155.41	177.30	135.77	HT-Direct
Zambia	CENTRAL	90-94	170.36	157.73	183.94	RW2
Zambia	CENTRAL	95-99	154.71	175.45	136.01	HT-Direct
Zambia	CENTRAL	95-99	160.88	147.78	174.88	RW2
Zambia	CENTRAL	00-04	115.39	134.57	98.63	HT-Direct
Zambia	CENTRAL	00-04	131.47	118.55	145.74	RW2
Zambia	CENTRAL	05-09	89.09	113.77	69.35	HT-Direct
Zambia	CENTRAL	05-09	94.23	80.87	109.38	RW2
Zambia	CENTRAL	10-14	62.44	82.39	47.07	HT-Direct
Zambia	CENTRAL	10-14	71.10	58.09	86.68	RW2
Zambia	CENTRAL	15-19	55.20	23.65	124.47	RW2
Zambia	COPPERBELT	80-84	115.91	135.07	99.16	HT-Direct
Zambia	COPPERBELT	80-84	126.88	111.37	143.62	RW2
Zambia	COPPERBELT	85-89	153.23	170.44	137.47	HT-Direct
Zambia	COPPERBELT	85-89	152.56	140.97	165.04	RW2
Zambia	COPPERBELT	90-94	163.17	180.78	146.97	HT-Direct
Zambia	COPPERBELT	90-94	163.91	151.80	177.13	RW2
Zambia	COPPERBELT	95-99	135.13	155.96	116.69	HT-Direct
Zambia	COPPERBELT	95-99	153.78	139.90	169.01	RW2
Zambia	COPPERBELT	00-04	113.06	134.66	94.54	HT-Direct
Zambia	COPPERBELT	00-04	123.56	109.89	138.65	RW2
Zambia	COPPERBELT	05-09	61.52	78.03	48.33	HT-Direct
Zambia	COPPERBELT	05-09	86.87	73.52	102.11	RW2
Zambia	COPPERBELT	10-14	68.20	92.24	50.09	HT-Direct
Zambia	COPPERBELT	10-14	65.05	51.13	82.02	RW2
Zambia	COPPERBELT	15-19	50.17	20.84	117.01	RW2
Zambia	EASTERN	80-84	225.13	250.32	201.80	HT-Direct
Zambia	EASTERN	80-84	224.73	204.93	246.95	RW2
Zambia	EASTERN	85-89	237.44	256.59	219.29	HT-Direct
Zambia	EASTERN	85-89	237.56	224.00	251.88	RW2
Zambia	EASTERN	90-94	223.14	239.77	207.36	HT-Direct
Zambia	EASTERN	90-94	233.48	220.22	246.87	RW2
Zambia	EASTERN	95-99	174.24	192.45	157.42	HT-Direct
Zambia	EASTERN	95-99	212.28	196.76	227.70	RW2
Zambia	EASTERN	00-04	155.98	175.99	137.86	HT-Direct
Zambia	EASTERN	00-04	174.63	160.20	189.88	RW2
Zambia	EASTERN	05-09	122.48	140.91	106.16	HT-Direct
Zambia	EASTERN	05-09	129.50	115.13	145.49	RW2
Zambia	EASTERN	10-14	102.80	125.31	83.94	HT-Direct
Zambia	EASTERN	10-14	101.32	85.70	120.46	RW2
Zambia	EASTERN	15-19	81.46	35.55	177.18	RW2
Zambia	LUAPULA	80-84	209.28	232.85	187.51	HT-Direct
Zambia	LUAPULA	80-84	217.36	196.60	239.18	RW2
Zambia	LUAPULA	85-89	245.51	270.35	222.27	HT-Direct
Zambia	LUAPULA	85-89	244.74	227.99	262.60	RW2
Zambia	LUAPULA	90-94	238.09	261.43	216.22	HT-Direct
Zambia	LUAPULA	90-94	248.00	230.90	266.23	RW2
Zambia	LUAPULA	95-99	208.33	232.79	185.81	HT-Direct
Zambia	LUAPULA	95-99	222.94	204.56	242.41	RW2
Zambia	LUAPULA	00-04	136.74	160.11	116.31	HT-Direct
Zambia	LUAPULA	00-04	170.91	152.08	190.78	RW2
Zambia	LUAPULA	05-09	103.77	132.77	80.51	HT-Direct
Zambia	LUAPULA	05-09	115.40	97.04	136.61	RW2
Zambia	LUAPULA	10-14	85.84	119.46	61.03	HT-Direct
Zambia	LUAPULA	10-14	81.73	62.58	106.53	RW2
Zambia	LUAPULA	15-19	59.06	23.86	141.61	RW2
Zambia	LUSAKA	80-84	120.34	139.56	103.45	HT-Direct
Zambia	LUSAKA	80-84	122.99	108.67	138.45	RW2
Zambia	LUSAKA	85-89	133.64	150.01	118.82	HT-Direct

Continued on next page

Country	Region	Year	Median	Lower	Upper	Method
Zambia	LUSAKA	85-89	145.29	133.75	157.32	RW2
Zambia	LUSAKA	90-94	170.62	191.12	151.91	HT-Direct
Zambia	LUSAKA	90-94	156.96	145.08	169.90	RW2
Zambia	LUSAKA	95-99	111.96	131.64	94.91	HT-Direct
Zambia	LUSAKA	95-99	150.31	136.84	165.11	RW2
Zambia	LUSAKA	00-04	128.59	149.42	110.29	HT-Direct
Zambia	LUSAKA	00-04	125.63	112.60	140.08	RW2
Zambia	LUSAKA	05-09	60.99	80.81	45.79	HT-Direct
Zambia	LUSAKA	05-09	92.13	78.62	107.55	RW2
Zambia	LUSAKA	10-14	67.92	87.77	52.31	HT-Direct
Zambia	LUSAKA	10-14	71.54	57.96	87.67	RW2
Zambia	LUSAKA	15-19	56.98	24.38	129.54	RW2
Zambia	NORTH-WESTERN	80-84	151.29	171.09	133.40	HT-Direct
Zambia	NORTH-WESTERN	80-84	150.24	134.84	167.14	RW2
Zambia	NORTH-WESTERN	85-89	155.72	176.06	137.34	HT-Direct
Zambia	NORTH-WESTERN	85-89	162.18	149.90	175.08	RW2
Zambia	NORTH-WESTERN	90-94	156.39	171.39	142.47	HT-Direct
Zambia	NORTH-WESTERN	90-94	161.43	150.37	172.97	RW2
Zambia	NORTH-WESTERN	95-99	120.63	137.15	105.86	HT-Direct
Zambia	NORTH-WESTERN	95-99	144.85	132.62	157.72	RW2
Zambia	NORTH-WESTERN	00-04	109.08	128.30	92.43	HT-Direct
Zambia	NORTH-WESTERN	00-04	113.42	101.48	126.59	RW2
Zambia	NORTH-WESTERN	05-09	63.32	86.99	45.76	HT-Direct
Zambia	NORTH-WESTERN	05-09	77.99	66.34	91.71	RW2
Zambia	NORTH-WESTERN	10-14	56.94	72.97	44.26	HT-Direct
Zambia	NORTH-WESTERN	10-14	56.98	46.30	70.27	RW2
Zambia	NORTH-WESTERN	15-19	42.85	18.19	99.74	RW2
Zambia	NORTHERN	80-84	187.68	210.16	167.10	HT-Direct
Zambia	NORTHERN	80-84	190.68	172.76	209.89	RW2
Zambia	NORTHERN	85-89	210.22	231.13	190.73	HT-Direct
Zambia	NORTHERN	85-89	215.98	201.95	230.74	RW2
Zambia	NORTHERN	90-94	204.84	223.59	187.29	HT-Direct
Zambia	NORTHERN	90-94	224.00	210.66	238.15	RW2
Zambia	NORTHERN	95-99	202.68	221.12	185.41	HT-Direct
Zambia	NORTHERN	95-99	209.18	195.22	224.38	RW2
Zambia	NORTHERN	00-04	154.61	173.16	137.71	HT-Direct
Zambia	NORTHERN	00-04	166.47	153.05	181.21	RW2
Zambia	NORTHERN	05-09	97.49	113.13	83.80	HT-Direct
Zambia	NORTHERN	05-09	113.94	100.90	128.16	RW2
Zambia	NORTHERN	10-14	68.49	85.18	54.88	HT-Direct
Zambia	NORTHERN	10-14	80.42	66.77	95.55	RW2
Zambia	NORTHERN	15-19	57.84	24.84	129.04	RW2
Zambia	SOUTHERN	80-84	133.64	152.85	116.51	HT-Direct
Zambia	SOUTHERN	80-84	132.61	118.27	148.39	RW2
Zambia	SOUTHERN	85-89	140.17	156.59	125.21	HT-Direct
Zambia	SOUTHERN	85-89	147.88	136.65	159.79	RW2
Zambia	SOUTHERN	90-94	151.73	170.32	134.84	HT-Direct
Zambia	SOUTHERN	90-94	152.38	140.89	164.43	RW2
Zambia	SOUTHERN	95-99	122.63	141.13	106.25	HT-Direct
Zambia	SOUTHERN	95-99	141.17	128.88	154.25	RW2
Zambia	SOUTHERN	00-04	101.32	116.45	87.96	HT-Direct
Zambia	SOUTHERN	00-04	114.03	102.84	126.39	RW2
Zambia	SOUTHERN	05-09	65.83	83.22	51.88	HT-Direct
Zambia	SOUTHERN	05-09	81.51	70.28	94.23	RW2
Zambia	SOUTHERN	10-14	64.05	80.96	50.48	HT-Direct
Zambia	SOUTHERN	10-14	62.11	51.18	75.36	RW2
Zambia	SOUTHERN	15-19	48.71	20.96	111.45	RW2
Zambia	WESTERN	80-84	209.67	234.78	186.59	HT-Direct
Zambia	WESTERN	80-84	208.63	188.23	230.49	RW2
Zambia	WESTERN	85-89	207.78	235.99	182.14	HT-Direct
Zambia	WESTERN	85-89	217.38	200.70	234.89	RW2
Zambia	WESTERN	90-94	187.88	210.81	166.92	HT-Direct
Zambia	WESTERN	90-94	211.43	196.01	227.72	RW2

Continued on next page

Country	Region	Year	Median	Lower	Upper	Method
Zambia	WESTERN	95-99	190.19	213.64	168.77	HT-Direct
Zambia	WESTERN	95-99	186.99	171.42	204.08	RW2
Zambia	WESTERN	00-04	128.18	152.08	107.57	HT-Direct
Zambia	WESTERN	00-04	141.85	126.27	159.09	RW2
Zambia	WESTERN	05-09	76.02	104.75	54.69	HT-Direct
Zambia	WESTERN	05-09	92.84	77.56	110.72	RW2
Zambia	WESTERN	10-14	55.93	79.49	39.05	HT-Direct
Zambia	WESTERN	10-14	63.51	48.98	81.41	RW2
Zambia	WESTERN	15-19	44.33	18.33	104.42	RW2
Zimbabwe	ALL	80-84	91.40	99.43	83.96	HT-Direct
Zimbabwe	ALL	80-84	83.19	81.66	84.71	IHME
Zimbabwe	ALL	80-84	94.31	86.38	102.91	RW2
Zimbabwe	ALL	80-84	94.36	91.13	97.64	UN
Zimbabwe	ALL	85-89	69.33	75.28	63.82	HT-Direct
Zimbabwe	ALL	85-89	69.14	68.10	70.27	IHME
Zimbabwe	ALL	85-89	75.82	69.42	82.69	RW2
Zimbabwe	ALL	85-89	75.76	73.21	78.66	UN
Zimbabwe	ALL	90-94	70.25	76.09	64.83	HT-Direct
Zimbabwe	ALL	90-94	66.99	65.87	68.08	IHME
Zimbabwe	ALL	90-94	83.27	75.84	91.36	RW2
Zimbabwe	ALL	90-94	83.14	80.33	86.01	UN
Zimbabwe	ALL	95-99	74.96	81.04	69.29	HT-Direct
Zimbabwe	ALL	95-99	74.12	72.87	75.48	IHME
Zimbabwe	ALL	95-99	100.70	89.87	112.71	RW2
Zimbabwe	ALL	95-99	101.16	97.31	105.10	UN
Zimbabwe	ALL	00-04	74.80	80.79	69.22	HT-Direct
Zimbabwe	ALL	00-04	81.03	79.51	82.40	IHME
Zimbabwe	ALL	00-04	104.27	92.34	117.61	RW2
Zimbabwe	ALL	00-04	104.55	100.33	109.08	UN
Zimbabwe	ALL	05-09	84.10	90.87	77.79	HT-Direct
Zimbabwe	ALL	05-09	81.90	79.93	83.63	IHME
Zimbabwe	ALL	05-09	97.79	89.06	107.28	RW2
Zimbabwe	ALL	05-09	97.60	93.32	102.22	UN
Zimbabwe	ALL	10-14	68.31	65.57	71.12	IHME
Zimbabwe	ALL	10-14	89.65	30.17	235.98	RW2
Zimbabwe	ALL	10-14	81.03	73.89	88.62	UN
Zimbabwe	BULAWAYO	80-84	56.78	80.95	39.52	HT-Direct
Zimbabwe	BULAWAYO	80-84	57.19	42.20	77.19	RW2
Zimbabwe	BULAWAYO	85-89	37.93	56.14	25.46	HT-Direct
Zimbabwe	BULAWAYO	85-89	46.86	36.77	59.54	RW2
Zimbabwe	BULAWAYO	90-94	41.02	60.73	27.52	HT-Direct
Zimbabwe	BULAWAYO	90-94	55.60	44.28	69.69	RW2
Zimbabwe	BULAWAYO	95-99	77.88	105.46	57.06	HT-Direct
Zimbabwe	BULAWAYO	95-99	77.64	61.27	98.21	RW2
Zimbabwe	BULAWAYO	00-04	73.18	105.05	50.44	HT-Direct
Zimbabwe	BULAWAYO	00-04	76.29	58.43	99.21	RW2
Zimbabwe	BULAWAYO	05-09	48.23	64.27	36.04	HT-Direct
Zimbabwe	BULAWAYO	05-09	64.13	47.44	86.20	RW2
Zimbabwe	BULAWAYO	10-14	53.07	16.78	160.27	RW2
Zimbabwe	BULAWAYO	15-19	43.35	2.57	463.19	RW2
Zimbabwe	HARARE	80-84	61.37	87.32	42.76	HT-Direct
Zimbabwe	HARARE	80-84	68.29	51.50	89.95	RW2
Zimbabwe	HARARE	85-89	55.35	75.34	40.43	HT-Direct
Zimbabwe	HARARE	85-89	57.53	46.29	71.19	RW2
Zimbabwe	HARARE	90-94	55.23	76.41	39.66	HT-Direct
Zimbabwe	HARARE	90-94	68.99	56.38	84.34	RW2
Zimbabwe	HARARE	95-99	80.53	113.45	56.56	HT-Direct
Zimbabwe	HARARE	95-99	96.65	77.48	120.57	RW2
Zimbabwe	HARARE	00-04	77.85	108.96	55.08	HT-Direct
Zimbabwe	HARARE	00-04	96.16	74.29	123.64	RW2
Zimbabwe	HARARE	05-09	63.85	85.08	47.64	HT-Direct
Zimbabwe	HARARE	05-09	82.78	61.47	110.32	RW2
Zimbabwe	HARARE	10-14	70.20	22.76	202.06	RW2

Continued on next page

Country	Region	Year	Median	Lower	Upper	Method
Zimbabwe	HARARE	15-19	59.55	3.63	541.77	RW2
Zimbabwe	MANICALAND	80-84	115.70	141.13	94.34	HT-Direct
Zimbabwe	MANICALAND	80-84	111.94	93.48	133.71	RW2
Zimbabwe	MANICALAND	85-89	75.61	95.13	59.83	HT-Direct
Zimbabwe	MANICALAND	85-89	93.95	80.70	109.12	RW2
Zimbabwe	MANICALAND	90-94	96.11	115.87	79.41	HT-Direct
Zimbabwe	MANICALAND	90-94	112.84	96.86	130.65	RW2
Zimbabwe	MANICALAND	95-99	122.78	156.90	95.24	HT-Direct
Zimbabwe	MANICALAND	95-99	157.80	131.55	188.56	RW2
Zimbabwe	MANICALAND	00-04	125.63	157.98	99.12	HT-Direct
Zimbabwe	MANICALAND	00-04	160.39	130.72	195.71	RW2
Zimbabwe	MANICALAND	05-09	122.91	153.24	97.89	HT-Direct
Zimbabwe	MANICALAND	05-09	142.10	112.54	177.86	RW2
Zimbabwe	MANICALAND	10-14	123.37	41.90	319.44	RW2
Zimbabwe	MANICALAND	15-19	106.67	6.88	688.63	RW2
Zimbabwe	MASHONALAND CENTRAL	80-84	143.98	167.49	123.28	HT-Direct
Zimbabwe	MASHONALAND CENTRAL	80-84	144.76	125.40	166.79	RW2
Zimbabwe	MASHONALAND CENTRAL	85-89	96.14	118.66	77.52	HT-Direct
Zimbabwe	MASHONALAND CENTRAL	85-89	110.65	96.27	126.98	RW2
Zimbabwe	MASHONALAND CENTRAL	90-94	96.26	120.80	76.28	HT-Direct
Zimbabwe	MASHONALAND CENTRAL	90-94	118.62	101.74	137.69	RW2
Zimbabwe	MASHONALAND CENTRAL	95-99	111.69	140.45	88.21	HT-Direct
Zimbabwe	MASHONALAND CENTRAL	95-99	148.64	124.45	176.39	RW2
Zimbabwe	MASHONALAND CENTRAL	00-04	104.70	133.12	81.77	HT-Direct
Zimbabwe	MASHONALAND CENTRAL	00-04	135.94	111.26	165.03	RW2
Zimbabwe	MASHONALAND CENTRAL	05-09	94.18	114.24	77.34	HT-Direct
Zimbabwe	MASHONALAND CENTRAL	05-09	108.00	87.77	132.19	RW2
Zimbabwe	MASHONALAND CENTRAL	10-14	83.94	28.27	231.48	RW2
Zimbabwe	MASHONALAND CENTRAL	15-19	65.22	4.00	558.63	RW2
Zimbabwe	MASHONALAND EAST	80-84	72.89	99.69	52.87	HT-Direct
Zimbabwe	MASHONALAND EAST	80-84	86.79	68.40	109.15	RW2
Zimbabwe	MASHONALAND EAST	85-89	75.65	95.97	59.35	HT-Direct
Zimbabwe	MASHONALAND EAST	85-89	74.52	62.27	88.99	RW2
Zimbabwe	MASHONALAND EAST	90-94	72.74	97.42	53.94	HT-Direct
Zimbabwe	MASHONALAND EAST	90-94	90.61	76.77	106.79	RW2
Zimbabwe	MASHONALAND EAST	95-99	114.25	140.56	92.34	HT-Direct
Zimbabwe	MASHONALAND EAST	95-99	127.98	107.46	152.02	RW2
Zimbabwe	MASHONALAND EAST	00-04	68.65	95.90	48.73	HT-Direct
Zimbabwe	MASHONALAND EAST	00-04	129.48	105.37	157.67	RW2
Zimbabwe	MASHONALAND EAST	05-09	96.47	116.86	79.32	HT-Direct
Zimbabwe	MASHONALAND EAST	05-09	113.82	92.45	139.48	RW2
Zimbabwe	MASHONALAND EAST	10-14	98.29	33.97	263.36	RW2
Zimbabwe	MASHONALAND EAST	15-19	84.35	5.55	627.45	RW2
Zimbabwe	MASHONALAND WEST	80-84	90.50	116.39	69.92	HT-Direct
Zimbabwe	MASHONALAND WEST	80-84	109.32	90.15	131.52	RW2
Zimbabwe	MASHONALAND WEST	85-89	98.02	115.83	82.70	HT-Direct
Zimbabwe	MASHONALAND WEST	85-89	92.03	79.95	105.35	RW2
Zimbabwe	MASHONALAND WEST	90-94	90.27	109.62	74.05	HT-Direct
Zimbabwe	MASHONALAND WEST	90-94	108.46	94.43	124.26	RW2
Zimbabwe	MASHONALAND WEST	95-99	90.24	114.00	71.03	HT-Direct
Zimbabwe	MASHONALAND WEST	95-99	148.28	125.64	174.55	RW2
Zimbabwe	MASHONALAND WEST	00-04	93.71	113.10	77.35	HT-Direct
Zimbabwe	MASHONALAND WEST	00-04	147.78	122.51	177.38	RW2
Zimbabwe	MASHONALAND WEST	05-09	124.64	154.78	99.68	HT-Direct
Zimbabwe	MASHONALAND WEST	05-09	129.14	103.69	159.74	RW2
Zimbabwe	MASHONALAND WEST	10-14	110.83	37.94	289.14	RW2
Zimbabwe	MASHONALAND WEST	15-19	94.23	6.08	661.02	RW2
Zimbabwe	MASVINGO	80-84	95.18	119.74	75.23	HT-Direct
Zimbabwe	MASVINGO	80-84	97.23	80.40	116.95	RW2
Zimbabwe	MASVINGO	85-89	67.72	82.28	55.57	HT-Direct
Zimbabwe	MASVINGO	85-89	75.27	64.95	87.13	RW2
Zimbabwe	MASVINGO	90-94	69.50	85.02	56.64	HT-Direct
Zimbabwe	MASVINGO	90-94	83.29	71.93	96.37	RW2

Continued on next page

Country	Region	Year	Median	Lower	Upper	Method
Zimbabwe	MASVINGO	95-99	80.97	107.94	60.29	HT-Direct
Zimbabwe	MASVINGO	95-99	108.23	90.50	129.47	RW2
Zimbabwe	MASVINGO	00-04	87.49	116.94	64.91	HT-Direct
Zimbabwe	MASVINGO	00-04	100.73	81.96	123.09	RW2
Zimbabwe	MASVINGO	05-09	67.26	80.95	55.74	HT-Direct
Zimbabwe	MASVINGO	05-09	80.86	66.06	98.65	RW2
Zimbabwe	MASVINGO	10-14	63.58	21.33	180.15	RW2
Zimbabwe	MASVINGO	15-19	49.57	3.12	486.81	RW2
Zimbabwe	MATABELELAND NORTH	80-84	100.95	129.63	78.04	HT-Direct
Zimbabwe	MATABELELAND NORTH	80-84	95.93	77.69	118.28	RW2
Zimbabwe	MATABELELAND NORTH	85-89	65.79	82.38	52.35	HT-Direct
Zimbabwe	MATABELELAND NORTH	85-89	74.03	62.50	87.59	RW2
Zimbabwe	MATABELELAND NORTH	90-94	57.62	80.42	40.99	HT-Direct
Zimbabwe	MATABELELAND NORTH	90-94	81.95	68.44	97.49	RW2
Zimbabwe	MATABELELAND NORTH	95-99	69.66	95.20	50.59	HT-Direct
Zimbabwe	MATABELELAND NORTH	95-99	107.35	87.37	130.98	RW2
Zimbabwe	MATABELELAND NORTH	00-04	95.51	134.91	66.74	HT-Direct
Zimbabwe	MATABELELAND NORTH	00-04	101.50	80.18	127.79	RW2
Zimbabwe	MATABELELAND NORTH	05-09	73.65	93.36	57.84	HT-Direct
Zimbabwe	MATABELELAND NORTH	05-09	83.37	64.54	106.81	RW2
Zimbabwe	MATABELELAND NORTH	10-14	67.05	21.78	190.59	RW2
Zimbabwe	MATABELELAND NORTH	15-19	54.33	3.39	504.17	RW2
Zimbabwe	MATABELELAND SOUTH	80-84	65.10	81.82	51.60	HT-Direct
Zimbabwe	MATABELELAND SOUTH	80-84	68.33	55.45	83.79	RW2
Zimbabwe	MATABELELAND SOUTH	85-89	49.28	67.95	35.55	HT-Direct
Zimbabwe	MATABELELAND SOUTH	85-89	55.58	46.28	66.47	RW2
Zimbabwe	MATABELELAND SOUTH	90-94	60.62	79.08	46.26	HT-Direct
Zimbabwe	MATABELELAND SOUTH	90-94	65.09	54.37	77.75	RW2
Zimbabwe	MATABELELAND SOUTH	95-99	70.33	96.97	50.61	HT-Direct
Zimbabwe	MATABELELAND SOUTH	95-99	89.49	72.42	110.10	RW2
Zimbabwe	MATABELELAND SOUTH	00-04	39.95	57.61	27.54	HT-Direct
Zimbabwe	MATABELELAND SOUTH	00-04	87.56	68.27	111.61	RW2
Zimbabwe	MATABELELAND SOUTH	05-09	66.41	87.27	50.27	HT-Direct
Zimbabwe	MATABELELAND SOUTH	05-09	74.18	56.12	97.70	RW2
Zimbabwe	MATABELELAND SOUTH	10-14	61.83	20.22	179.94	RW2
Zimbabwe	MATABELELAND SOUTH	15-19	51.27	3.12	508.54	RW2
Zimbabwe	MIDLANDS	80-84	98.47	120.91	79.82	HT-Direct
Zimbabwe	MIDLANDS	80-84	99.51	82.92	118.72	RW2
Zimbabwe	MIDLANDS	85-89	66.85	84.91	52.41	HT-Direct
Zimbabwe	MIDLANDS	85-89	79.19	67.94	92.05	RW2
Zimbabwe	MIDLANDS	90-94	82.64	100.54	67.69	HT-Direct
Zimbabwe	MIDLANDS	90-94	89.63	77.53	103.26	RW2
Zimbabwe	MIDLANDS	95-99	80.85	100.77	64.59	HT-Direct
Zimbabwe	MIDLANDS	95-99	118.64	100.19	140.11	RW2
Zimbabwe	MIDLANDS	00-04	75.77	95.00	60.18	HT-Direct
Zimbabwe	MIDLANDS	00-04	113.26	93.30	137.10	RW2
Zimbabwe	MIDLANDS	05-09	83.25	100.92	68.43	HT-Direct
Zimbabwe	MIDLANDS	05-09	94.13	76.82	114.67	RW2
Zimbabwe	MIDLANDS	10-14	76.81	25.89	211.80	RW2
Zimbabwe	MIDLANDS	15-19	62.08	3.97	549.10	RW2

3.7 Table of All Results: 1-year Periods

Table S1.10: Complete Results by 1-year Period.

Country	Region	Year	Median	Lower	Upper	Method
Angola	ALL	1980	192.05	166.46	218.22	IHME
Angola	ALL	1980	267.19	161.54	414.02	RW2
Angola	ALL	1980	234.10	187.40	290.20	UN
Angola	ALL	1981	190.62	166.49	216.75	IHME
Angola	ALL	1981	258.88	173.81	369.41	RW2
Angola	ALL	1981	232.80	190.00	282.90	UN
Angola	ALL	1982	190.06	166.87	215.42	IHME
Angola	ALL	1982	250.84	179.92	340.18	RW2
Angola	ALL	1982	231.50	192.70	276.80	UN
Angola	ALL	1983	188.67	166.89	214.52	IHME
Angola	ALL	1983	242.50	178.35	323.38	RW2
Angola	ALL	1983	230.20	194.30	271.50	UN
Angola	ALL	1984	186.39	165.28	208.88	IHME
Angola	ALL	1984	235.80	173.17	313.87	RW2
Angola	ALL	1984	229.10	196.00	267.40	UN
Angola	ALL	1985	184.66	163.76	206.17	IHME
Angola	ALL	1985	228.29	169.87	296.79	RW2
Angola	ALL	1985	228.30	197.40	264.00	UN
Angola	ALL	1986	185.06	167.54	205.99	IHME
Angola	ALL	1986	223.80	168.72	287.44	RW2
Angola	ALL	1986	227.50	198.40	260.90	UN
Angola	ALL	1987	187.09	169.97	207.37	IHME
Angola	ALL	1987	221.17	168.73	282.04	RW2
Angola	ALL	1987	226.90	199.80	258.60	UN
Angola	ALL	1988	189.57	171.66	208.66	IHME
Angola	ALL	1988	220.03	166.98	280.41	RW2
Angola	ALL	1988	226.50	200.60	257.30	UN
Angola	ALL	1989	190.63	172.40	210.19	IHME
Angola	ALL	1989	220.42	166.82	281.28	RW2
Angola	ALL	1989	226.20	201.10	256.10	UN
Angola	ALL	1990	190.46	172.22	210.74	IHME
Angola	ALL	1990	222.95	172.25	285.58	RW2
Angola	ALL	1990	226.00	201.60	255.30	UN
Angola	ALL	1991	187.49	169.26	207.69	IHME
Angola	ALL	1991	224.71	175.71	283.55	RW2
Angola	ALL	1991	225.90	201.90	254.40	UN
Angola	ALL	1992	183.58	165.23	202.16	IHME
Angola	ALL	1992	226.33	177.82	284.13	RW2
Angola	ALL	1992	226.00	202.30	253.60	UN
Angola	ALL	1993	180.97	163.00	198.26	IHME
Angola	ALL	1993	227.46	178.37	286.60	RW2
Angola	ALL	1993	225.80	202.10	253.10	UN
Angola	ALL	1994	173.34	157.40	190.41	IHME
Angola	ALL	1994	227.72	177.26	290.12	RW2
Angola	ALL	1994	225.50	201.90	252.80	UN
Angola	ALL	1995	166.87	151.71	184.23	IHME
Angola	ALL	1995	227.10	177.26	285.88	RW2
Angola	ALL	1995	224.80	201.10	252.50	UN
Angola	ALL	1996	162.48	147.40	179.06	IHME
Angola	ALL	1996	226.08	178.44	282.72	RW2
Angola	ALL	1996	224.00	199.90	251.70	UN
Angola	ALL	1997	160.06	145.68	176.16	IHME
Angola	ALL	1997	224.62	178.48	278.69	RW2
Angola	ALL	1997	222.60	198.20	250.40	UN
Angola	ALL	1998	159.19	143.73	174.19	IHME
Angola	ALL	1998	222.95	175.85	279.68	RW2
Angola	ALL	1998	220.80	195.90	249.20	UN
Angola	ALL	1999	158.04	143.43	174.54	IHME
Angola	ALL	1999	220.85	171.50	278.76	RW2
Angola	ALL	1999	218.90	193.30	247.90	UN

Continued on next page

Country	Region	Year	Median	Lower	Upper	Method
Angola	ALL	2000	155.77	141.10	172.43	IHME
Angola	ALL	2000	218.43	168.38	277.79	RW2
Angola	ALL	2000	216.70	190.40	246.60	UN
Angola	ALL	2001	152.29	137.14	169.36	IHME
Angola	ALL	2001	215.76	167.13	273.55	RW2
Angola	ALL	2001	214.10	186.90	245.40	UN
Angola	ALL	2002	145.79	131.78	161.52	IHME
Angola	ALL	2002	212.74	165.51	269.31	RW2
Angola	ALL	2002	211.70	182.50	244.70	UN
Angola	ALL	2003	138.24	124.62	153.25	IHME
Angola	ALL	2003	209.68	160.49	269.22	RW2
Angola	ALL	2003	209.20	178.30	244.80	UN
Angola	ALL	2004	130.47	117.15	144.56	IHME
Angola	ALL	2004	205.83	150.29	275.03	RW2
Angola	ALL	2004	206.70	172.70	245.10	UN
Angola	ALL	2005	123.26	109.99	137.53	IHME
Angola	ALL	2005	202.32	136.46	289.66	RW2
Angola	ALL	2005	203.90	166.40	245.40	UN
Angola	ALL	2006	117.63	104.59	131.06	IHME
Angola	ALL	2006	198.22	127.65	294.62	RW2
Angola	ALL	2006	200.50	159.90	245.20	UN
Angola	ALL	2007	113.34	100.96	127.82	IHME
Angola	ALL	2007	194.18	122.07	294.94	RW2
Angola	ALL	2007	196.40	153.20	245.60	UN
Angola	ALL	2008	109.20	97.08	123.16	IHME
Angola	ALL	2008	190.60	119.57	290.51	RW2
Angola	ALL	2008	192.00	145.70	245.80	UN
Angola	ALL	2009	105.19	92.21	119.21	IHME
Angola	ALL	2009	186.12	119.92	279.04	RW2
Angola	ALL	2009	187.30	137.80	246.80	UN
Angola	ALL	2010	100.82	86.82	117.10	IHME
Angola	ALL	2010	181.94	125.91	257.36	RW2
Angola	ALL	2010	182.50	130.10	248.30	UN
Angola	ALL	2011	96.34	79.62	113.74	IHME
Angola	ALL	2011	177.99	131.12	237.04	RW2
Angola	ALL	2011	177.30	122.10	249.50	UN
Angola	ALL	2012	91.75	72.10	111.10	IHME
Angola	ALL	2012	173.89	133.54	223.05	RW2
Angola	ALL	2012	172.20	114.90	250.80	UN
Angola	ALL	2013	87.22	66.86	108.42	IHME
Angola	ALL	2013	170.00	124.33	227.55	RW2
Angola	ALL	2013	167.10	107.70	252.20	UN
Angola	ALL	2014	82.88	64.29	105.43	IHME
Angola	ALL	2014	165.97	104.06	253.64	RW2
Angola	ALL	2014	162.20	101.10	253.60	UN
Angola	ALL	2015	78.70	60.29	100.90	IHME
Angola	ALL	2015	161.83	80.27	299.23	RW2
Angola	ALL	2015	156.90	94.80	253.70	UN
Angola	ALL	2016	158.45	61.59	352.80	RW2
Angola	ALL	2017	154.25	45.15	415.77	RW2
Angola	ALL	2018	150.51	32.82	490.34	RW2
Angola	ALL	2019	146.61	21.99	561.79	RW2
Angola	ALL	15-19	154.26	46.12	408.89	RW2
Angola	BENGO	1980	117.65	24.86	414.58	RW2
Angola	BENGO	1981	113.45	26.83	376.56	RW2
Angola	BENGO	1982	108.93	28.17	346.39	RW2
Angola	BENGO	1983	105.14	29.17	311.47	RW2
Angola	BENGO	1984	101.61	30.71	288.88	RW2
Angola	BENGO	1985	98.63	31.89	265.29	RW2
Angola	BENGO	1986	96.75	33.56	247.32	RW2
Angola	BENGO	1987	96.57	36.02	233.35	RW2
Angola	BENGO	1988	97.74	38.53	225.09	RW2
Angola	BENGO	1989	99.66	41.91	218.55	RW2

Continued on next page

Country	Region	Year	Median	Lower	Upper	Method
Angola	BENGO	1990	103.59	46.22	215.33	RW2
Angola	BENGO	1991	105.82	49.88	209.25	RW2
Angola	BENGO	1992	107.82	53.83	204.15	RW2
Angola	BENGO	1993	109.05	56.69	198.08	RW2
Angola	BENGO	1994	109.47	59.17	193.26	RW2
Angola	BENGO	1995	108.50	60.33	185.24	RW2
Angola	BENGO	1996	108.75	63.02	180.31	RW2
Angola	BENGO	1997	109.66	65.22	177.03	RW2
Angola	BENGO	1998	111.22	67.41	176.78	RW2
Angola	BENGO	1999	113.70	69.35	180.07	RW2
Angola	BENGO	2000	117.07	71.99	185.02	RW2
Angola	BENGO	2001	119.74	74.05	188.35	RW2
Angola	BENGO	2002	121.86	74.75	192.11	RW2
Angola	BENGO	2003	123.45	73.92	198.22	RW2
Angola	BENGO	2004	123.77	71.65	207.08	RW2
Angola	BENGO	2005	123.09	66.24	216.73	RW2
Angola	BENGO	2006	121.95	62.10	223.87	RW2
Angola	BENGO	2007	120.76	58.80	229.46	RW2
Angola	BENGO	2008	118.81	56.14	232.94	RW2
Angola	BENGO	2009	116.59	53.38	234.12	RW2
Angola	BENGO	2010	114.67	52.09	233.14	RW2
Angola	BENGO	2011	112.48	50.00	233.39	RW2
Angola	BENGO	2012	109.92	47.05	236.63	RW2
Angola	BENGO	2013	107.87	42.79	245.12	RW2
Angola	BENGO	2014	105.98	37.30	262.87	RW2
Angola	BENGO	2015	103.01	31.03	293.42	RW2
Angola	BENGO	2016	101.27	24.91	331.19	RW2
Angola	BENGO	2017	99.53	19.68	379.67	RW2
Angola	BENGO	2018	96.58	14.34	436.41	RW2
Angola	BENGO	2019	94.92	10.71	510.07	RW2
Angola	BENGUELA	1980	393.18	260.51	542.13	RW2
Angola	BENGUELA	1981	387.58	277.18	506.85	RW2
Angola	BENGUELA	1982	381.10	285.08	485.87	RW2
Angola	BENGUELA	1983	375.53	284.66	474.54	RW2
Angola	BENGUELA	1984	370.74	282.33	469.15	RW2
Angola	BENGUELA	1985	366.96	279.96	457.98	RW2
Angola	BENGUELA	1986	366.13	283.66	453.38	RW2
Angola	BENGUELA	1987	368.73	289.42	451.78	RW2
Angola	BENGUELA	1988	374.17	296.00	455.72	RW2
Angola	BENGUELA	1989	381.27	303.53	462.02	RW2
Angola	BENGUELA	1990	391.28	319.01	470.46	RW2
Angola	BENGUELA	1991	398.03	328.86	473.85	RW2
Angola	BENGUELA	1992	402.24	334.86	474.48	RW2
Angola	BENGUELA	1993	403.65	336.49	477.83	RW2
Angola	BENGUELA	1994	401.86	333.26	478.89	RW2
Angola	BENGUELA	1995	396.90	327.15	470.28	RW2
Angola	BENGUELA	1996	393.30	326.38	463.76	RW2
Angola	BENGUELA	1997	390.71	325.85	460.66	RW2
Angola	BENGUELA	1998	389.44	322.87	462.35	RW2
Angola	BENGUELA	1999	389.23	318.57	465.83	RW2
Angola	BENGUELA	2000	391.45	318.62	471.98	RW2
Angola	BENGUELA	2001	391.47	319.78	471.56	RW2
Angola	BENGUELA	2002	390.37	317.36	470.68	RW2
Angola	BENGUELA	2003	387.45	309.95	472.57	RW2
Angola	BENGUELA	2004	382.05	295.74	478.05	RW2
Angola	BENGUELA	2005	374.56	274.37	486.47	RW2
Angola	BENGUELA	2006	366.73	258.36	488.41	RW2
Angola	BENGUELA	2007	356.81	245.10	484.93	RW2
Angola	BENGUELA	2008	347.59	236.68	475.32	RW2
Angola	BENGUELA	2009	337.53	232.73	459.52	RW2
Angola	BENGUELA	2010	326.83	233.77	436.14	RW2
Angola	BENGUELA	2011	316.68	233.62	412.06	RW2
Angola	BENGUELA	2012	306.31	229.34	394.07	RW2

Continued on next page

Country	Region	Year	Median	Lower	Upper	Method
Angola	BENGUELA	2013	295.78	212.29	394.02	RW2
Angola	BENGUELA	2014	285.97	183.15	416.63	RW2
Angola	BENGUELA	2015	275.54	146.56	457.45	RW2
Angola	BENGUELA	2016	265.59	115.08	503.97	RW2
Angola	BENGUELA	2017	255.94	86.15	563.33	RW2
Angola	BENGUELA	2018	246.45	62.92	618.42	RW2
Angola	BENGUELA	2019	237.81	44.29	675.96	RW2
Angola	BIÉ	1980	225.17	129.27	375.26	RW2
Angola	BIÉ	1981	216.26	135.33	335.51	RW2
Angola	BIÉ	1982	206.94	134.90	310.02	RW2
Angola	BIÉ	1983	198.51	131.24	293.14	RW2
Angola	BIÉ	1984	191.08	126.95	279.75	RW2
Angola	BIÉ	1985	184.03	123.50	264.91	RW2
Angola	BIÉ	1986	179.91	122.40	254.26	RW2
Angola	BIÉ	1987	177.77	123.15	248.64	RW2
Angola	BIÉ	1988	177.62	124.29	244.90	RW2
Angola	BIÉ	1989	179.04	127.23	244.06	RW2
Angola	BIÉ	1990	183.24	133.14	247.49	RW2
Angola	BIÉ	1991	185.32	137.27	245.77	RW2
Angola	BIÉ	1992	186.25	139.73	244.48	RW2
Angola	BIÉ	1993	185.78	140.72	242.56	RW2
Angola	BIÉ	1994	184.14	140.34	240.16	RW2
Angola	BIÉ	1995	180.76	137.30	231.44	RW2
Angola	BIÉ	1996	178.90	138.96	226.32	RW2
Angola	BIÉ	1997	178.28	139.42	223.16	RW2
Angola	BIÉ	1998	178.64	139.04	224.51	RW2
Angola	BIÉ	1999	180.52	139.05	229.67	RW2
Angola	BIÉ	2000	184.17	140.58	237.15	RW2
Angola	BIÉ	2001	187.07	143.70	240.35	RW2
Angola	BIÉ	2002	189.40	145.53	243.15	RW2
Angola	BIÉ	2003	190.92	144.16	248.95	RW2
Angola	BIÉ	2004	191.43	138.79	258.34	RW2
Angola	BIÉ	2005	190.34	129.72	270.33	RW2
Angola	BIÉ	2006	189.33	123.84	278.01	RW2
Angola	BIÉ	2007	187.67	119.50	282.45	RW2
Angola	BIÉ	2008	185.73	117.54	280.43	RW2
Angola	BIÉ	2009	183.37	117.05	275.36	RW2
Angola	BIÉ	2010	181.22	120.14	263.14	RW2
Angola	BIÉ	2011	178.85	123.28	252.25	RW2
Angola	BIÉ	2012	176.23	123.02	247.32	RW2
Angola	BIÉ	2013	173.59	116.27	253.49	RW2
Angola	BIÉ	2014	171.01	102.20	275.23	RW2
Angola	BIÉ	2015	168.68	82.57	315.63	RW2
Angola	BIÉ	2016	166.65	66.12	363.76	RW2
Angola	BIÉ	2017	163.59	50.55	425.82	RW2
Angola	BIÉ	2018	161.45	37.90	490.22	RW2
Angola	BIÉ	2019	159.31	27.75	568.46	RW2
Angola	CABINDA	1980	107.50	28.37	351.19	RW2
Angola	CABINDA	1981	103.08	30.65	312.78	RW2
Angola	CABINDA	1982	99.12	31.62	284.14	RW2
Angola	CABINDA	1983	95.03	32.71	258.42	RW2
Angola	CABINDA	1984	91.94	33.35	235.58	RW2
Angola	CABINDA	1985	88.51	34.37	214.93	RW2
Angola	CABINDA	1986	87.29	36.06	200.09	RW2
Angola	CABINDA	1987	86.67	37.78	187.16	RW2
Angola	CABINDA	1988	87.26	40.17	178.66	RW2
Angola	CABINDA	1989	88.29	42.75	172.45	RW2
Angola	CABINDA	1990	91.07	46.68	170.26	RW2
Angola	CABINDA	1991	92.53	49.93	165.76	RW2
Angola	CABINDA	1992	93.37	52.36	159.95	RW2

Continued on next page

Country	Region	Year	Median	Lower	Upper	Method
Angola	CABINDA	1993	93.26	53.92	156.61	RW2
Angola	CABINDA	1994	92.54	54.90	152.47	RW2
Angola	CABINDA	1995	90.41	54.66	144.99	RW2
Angola	CABINDA	1996	89.60	55.80	140.24	RW2
Angola	CABINDA	1997	89.23	56.29	136.80	RW2
Angola	CABINDA	1998	89.44	56.59	136.85	RW2
Angola	CABINDA	1999	90.35	57.33	137.99	RW2
Angola	CABINDA	2000	92.32	58.42	142.17	RW2
Angola	CABINDA	2001	93.81	59.57	143.37	RW2
Angola	CABINDA	2002	94.92	60.25	145.95	RW2
Angola	CABINDA	2003	95.58	59.55	148.59	RW2
Angola	CABINDA	2004	95.84	57.47	154.50	RW2
Angola	CABINDA	2005	95.12	53.83	160.73	RW2
Angola	CABINDA	2006	94.30	51.12	166.23	RW2
Angola	CABINDA	2007	92.97	48.76	168.95	RW2
Angola	CABINDA	2008	92.04	47.08	170.90	RW2
Angola	CABINDA	2009	90.49	46.13	168.65	RW2
Angola	CABINDA	2010	89.16	45.84	165.53	RW2
Angola	CABINDA	2011	87.80	45.07	163.98	RW2
Angola	CABINDA	2012	86.29	43.64	163.65	RW2
Angola	CABINDA	2013	85.06	40.59	170.48	RW2
Angola	CABINDA	2014	83.79	35.70	185.56	RW2
Angola	CABINDA	2015	82.54	29.34	209.66	RW2
Angola	CABINDA	2016	80.85	23.47	245.29	RW2
Angola	CABINDA	2017	79.52	18.30	287.91	RW2
Angola	CABINDA	2018	77.93	13.78	345.11	RW2
Angola	CABINDA	2019	77.31	10.05	406.32	RW2
Angola	CUANDO CUBANGO	1980	120.89	28.77	397.30	RW2
Angola	CUANDO CUBANGO	1981	119.20	31.58	363.43	RW2
Angola	CUANDO CUBANGO	1982	116.72	33.42	338.95	RW2
Angola	CUANDO CUBANGO	1983	115.08	35.04	319.43	RW2
Angola	CUANDO CUBANGO	1984	112.96	36.94	299.00	RW2
Angola	CUANDO CUBANGO	1985	111.32	38.88	280.22	RW2
Angola	CUANDO CUBANGO	1986	112.23	41.49	268.75	RW2
Angola	CUANDO CUBANGO	1987	113.94	45.21	258.08	RW2
Angola	CUANDO CUBANGO	1988	116.50	49.03	251.08	RW2
Angola	CUANDO CUBANGO	1989	120.87	53.87	248.13	RW2
Angola	CUANDO CUBANGO	1990	127.07	60.59	248.47	RW2
Angola	CUANDO CUBANGO	1991	131.78	66.00	245.98	RW2
Angola	CUANDO CUBANGO	1992	135.65	71.15	240.01	RW2
Angola	CUANDO CUBANGO	1993	137.95	75.72	237.47	RW2
Angola	CUANDO CUBANGO	1994	139.68	79.78	233.15	RW2
Angola	CUANDO CUBANGO	1995	139.09	81.29	225.27	RW2
Angola	CUANDO CUBANGO	1996	140.36	85.33	220.99	RW2
Angola	CUANDO CUBANGO	1997	141.85	88.49	217.39	RW2
Angola	CUANDO CUBANGO	1998	144.67	92.26	218.91	RW2
Angola	CUANDO CUBANGO	1999	148.05	95.30	221.89	RW2
Angola	CUANDO CUBANGO	2000	153.34	100.01	228.33	RW2
Angola	CUANDO CUBANGO	2001	157.41	103.73	231.98	RW2
Angola	CUANDO CUBANGO	2002	161.15	106.89	234.88	RW2
Angola	CUANDO CUBANGO	2003	164.17	107.67	242.32	RW2
Angola	CUANDO CUBANGO	2004	165.92	106.45	250.38	RW2
Angola	CUANDO CUBANGO	2005	166.57	101.47	261.63	RW2
Angola	CUANDO CUBANGO	2006	166.81	97.47	269.71	RW2
Angola	CUANDO CUBANGO	2007	166.54	95.12	275.12	RW2
Angola	CUANDO CUBANGO	2008	166.04	93.13	276.13	RW2
Angola	CUANDO CUBANGO	2009	165.02	92.97	274.21	RW2
Angola	CUANDO CUBANGO	2010	164.23	93.96	270.54	RW2
Angola	CUANDO CUBANGO	2011	162.76	94.00	265.77	RW2
Angola	CUANDO CUBANGO	2012	161.19	92.80	265.31	RW2
Angola	CUANDO CUBANGO	2013	160.00	88.00	274.55	RW2
Angola	CUANDO CUBANGO	2014	159.12	77.96	298.36	RW2
Angola	CUANDO CUBANGO	2015	157.65	65.15	335.15	RW2

Continued on next page

Country	Region	Year	Median	Lower	Upper	Method
Angola	CUANDO CUBANGO	2016	156.29	52.41	384.14	RW2
Angola	CUANDO CUBANGO	2017	154.74	41.13	440.17	RW2
Angola	CUANDO CUBANGO	2018	153.86	31.27	508.11	RW2
Angola	CUANDO CUBANGO	2019	153.11	22.97	578.46	RW2
Angola	CUANZA NORTE	1980	202.27	68.63	457.30	RW2
Angola	CUANZA NORTE	1981	197.15	73.36	423.99	RW2
Angola	CUANZA NORTE	1982	191.53	76.78	392.50	RW2
Angola	CUANZA NORTE	1983	186.64	79.90	372.25	RW2
Angola	CUANZA NORTE	1984	182.64	82.29	353.24	RW2
Angola	CUANZA NORTE	1985	177.77	84.16	330.48	RW2
Angola	CUANZA NORTE	1986	176.63	88.64	318.16	RW2
Angola	CUANZA NORTE	1987	177.16	93.59	308.29	RW2
Angola	CUANZA NORTE	1988	179.94	99.07	301.12	RW2
Angola	CUANZA NORTE	1989	184.38	106.54	299.12	RW2
Angola	CUANZA NORTE	1990	191.06	116.08	298.98	RW2
Angola	CUANZA NORTE	1991	196.20	123.93	296.54	RW2
Angola	CUANZA NORTE	1992	199.75	130.64	294.30	RW2
Angola	CUANZA NORTE	1993	202.20	134.79	292.89	RW2
Angola	CUANZA NORTE	1994	203.10	137.63	289.78	RW2
Angola	CUANZA NORTE	1995	201.73	138.19	283.07	RW2
Angola	CUANZA NORTE	1996	201.85	141.22	280.22	RW2
Angola	CUANZA NORTE	1997	202.82	142.84	278.40	RW2
Angola	CUANZA NORTE	1998	204.87	145.86	281.29	RW2
Angola	CUANZA NORTE	1999	208.00	147.09	286.49	RW2
Angola	CUANZA NORTE	2000	213.07	151.18	295.78	RW2
Angola	CUANZA NORTE	2001	216.65	154.03	300.14	RW2
Angola	CUANZA NORTE	2002	219.29	155.85	302.91	RW2
Angola	CUANZA NORTE	2003	220.44	153.55	309.38	RW2
Angola	CUANZA NORTE	2004	220.71	148.18	316.66	RW2
Angola	CUANZA NORTE	2005	218.69	138.07	327.84	RW2
Angola	CUANZA NORTE	2006	216.44	131.39	333.88	RW2
Angola	CUANZA NORTE	2007	213.41	125.92	338.05	RW2
Angola	CUANZA NORTE	2008	209.57	121.39	337.16	RW2
Angola	CUANZA NORTE	2009	205.88	119.13	331.43	RW2
Angola	CUANZA NORTE	2010	201.19	117.64	321.52	RW2
Angola	CUANZA NORTE	2011	196.90	116.49	312.22	RW2
Angola	CUANZA NORTE	2012	192.59	112.56	310.06	RW2
Angola	CUANZA NORTE	2013	187.86	104.08	315.78	RW2
Angola	CUANZA NORTE	2014	183.56	91.48	335.36	RW2
Angola	CUANZA NORTE	2015	179.50	75.59	369.06	RW2
Angola	CUANZA NORTE	2016	175.60	60.53	417.14	RW2
Angola	CUANZA NORTE	2017	170.70	46.33	470.95	RW2
Angola	CUANZA NORTE	2018	167.00	34.42	535.18	RW2
Angola	CUANZA NORTE	2019	163.42	25.01	601.65	RW2
Angola	CUANZA SUL	1980	231.39	120.67	403.55	RW2
Angola	CUANZA SUL	1981	226.85	129.34	371.02	RW2
Angola	CUANZA SUL	1982	223.06	135.27	347.06	RW2
Angola	CUANZA SUL	1983	218.99	137.17	331.57	RW2
Angola	CUANZA SUL	1984	215.52	139.51	318.89	RW2
Angola	CUANZA SUL	1985	212.46	141.85	305.57	RW2
Angola	CUANZA SUL	1986	212.52	145.10	298.74	RW2
Angola	CUANZA SUL	1987	214.90	151.35	294.82	RW2
Angola	CUANZA SUL	1988	219.40	156.97	295.97	RW2
Angola	CUANZA SUL	1989	225.52	163.08	300.84	RW2
Angola	CUANZA SUL	1990	234.70	174.50	310.37	RW2
Angola	CUANZA SUL	1991	241.23	182.18	313.06	RW2
Angola	CUANZA SUL	1992	246.37	189.19	315.60	RW2
Angola	CUANZA SUL	1993	249.76	192.43	319.88	RW2
Angola	CUANZA SUL	1994	251.21	193.58	321.78	RW2
Angola	CUANZA SUL	1995	249.90	192.93	316.02	RW2
Angola	CUANZA SUL	1996	250.48	196.09	313.78	RW2
Angola	CUANZA SUL	1997	252.23	198.35	314.28	RW2
Angola	CUANZA SUL	1998	255.56	199.43	320.32	RW2

Continued on next page

Country	Region	Year	Median	Lower	Upper	Method
Angola	CUANZA SUL	1999	259.72	200.62	329.24	RW2
Angola	CUANZA SUL	2000	266.36	204.26	340.89	RW2
Angola	CUANZA SUL	2001	271.72	208.61	346.79	RW2
Angola	CUANZA SUL	2002	276.25	210.87	353.44	RW2
Angola	CUANZA SUL	2003	279.41	209.10	362.25	RW2
Angola	CUANZA SUL	2004	280.92	203.35	373.91	RW2
Angola	CUANZA SUL	2005	280.23	190.66	389.41	RW2
Angola	CUANZA SUL	2006	279.79	183.87	401.49	RW2
Angola	CUANZA SUL	2007	277.71	177.66	405.02	RW2
Angola	CUANZA SUL	2008	275.69	174.61	405.04	RW2
Angola	CUANZA SUL	2009	273.41	174.38	399.45	RW2
Angola	CUANZA SUL	2010	270.97	178.38	386.47	RW2
Angola	CUANZA SUL	2011	267.55	181.04	375.60	RW2
Angola	CUANZA SUL	2012	264.86	180.85	371.32	RW2
Angola	CUANZA SUL	2013	261.55	170.48	377.47	RW2
Angola	CUANZA SUL	2014	258.19	152.54	403.48	RW2
Angola	CUANZA SUL	2015	255.22	125.85	446.57	RW2
Angola	CUANZA SUL	2016	252.20	102.14	500.15	RW2
Angola	CUANZA SUL	2017	248.89	79.32	561.66	RW2
Angola	CUANZA SUL	2018	245.81	59.38	622.16	RW2
Angola	CUANZA SUL	2019	242.63	44.00	687.84	RW2
Angola	CUNENE	1980	115.71	36.65	293.92	RW2
Angola	CUNENE	1981	115.15	41.49	268.84	RW2
Angola	CUNENE	1982	114.61	44.84	248.19	RW2
Angola	CUNENE	1983	113.93	48.23	235.04	RW2
Angola	CUNENE	1984	113.90	51.88	224.89	RW2
Angola	CUNENE	1985	113.54	54.92	212.56	RW2
Angola	CUNENE	1986	115.35	59.85	206.09	RW2
Angola	CUNENE	1987	118.66	65.60	201.70	RW2
Angola	CUNENE	1988	123.25	71.93	199.17	RW2
Angola	CUNENE	1989	129.04	79.55	200.43	RW2
Angola	CUNENE	1990	137.04	89.44	203.36	RW2
Angola	CUNENE	1991	143.65	98.37	205.22	RW2
Angola	CUNENE	1992	148.84	105.75	205.06	RW2
Angola	CUNENE	1993	153.00	111.30	207.64	RW2
Angola	CUNENE	1994	156.02	114.88	209.60	RW2
Angola	CUNENE	1995	156.59	115.72	206.93	RW2
Angola	CUNENE	1996	158.84	119.65	206.38	RW2
Angola	CUNENE	1997	161.24	122.44	208.92	RW2
Angola	CUNENE	1998	164.65	124.22	214.51	RW2
Angola	CUNENE	1999	169.37	125.85	223.94	RW2
Angola	CUNENE	2000	175.40	129.29	235.24	RW2
Angola	CUNENE	2001	180.26	132.88	242.16	RW2
Angola	CUNENE	2002	184.64	133.81	250.10	RW2
Angola	CUNENE	2003	187.75	132.71	260.41	RW2
Angola	CUNENE	2004	189.80	128.46	271.81	RW2
Angola	CUNENE	2005	189.65	120.42	285.67	RW2
Angola	CUNENE	2006	189.83	115.25	296.75	RW2
Angola	CUNENE	2007	188.49	110.52	300.54	RW2
Angola	CUNENE	2008	186.75	107.61	304.09	RW2
Angola	CUNENE	2009	185.42	106.44	302.07	RW2
Angola	CUNENE	2010	183.39	107.29	295.39	RW2
Angola	CUNENE	2011	181.38	106.95	289.63	RW2
Angola	CUNENE	2012	178.89	104.30	289.60	RW2
Angola	CUNENE	2013	176.72	97.78	298.22	RW2
Angola	CUNENE	2014	174.51	86.28	319.32	RW2
Angola	CUNENE	2015	172.22	71.71	359.63	RW2
Angola	CUNENE	2016	169.54	57.93	407.44	RW2
Angola	CUNENE	2017	167.83	44.43	465.45	RW2
Angola	CUNENE	2018	165.07	33.30	528.74	RW2
Angola	CUNENE	2019	162.65	24.71	600.64	RW2
Angola	HUAMBO	1980	318.62	198.99	468.20	RW2
Angola	HUAMBO	1981	309.85	208.59	430.39	RW2

Continued on next page

Country	Region	Year	Median	Lower	Upper	Method
Angola	HUAMBO	1982	301.04	213.03	406.46	RW2
Angola	HUAMBO	1983	292.74	210.22	391.16	RW2
Angola	HUAMBO	1984	285.20	205.47	381.36	RW2
Angola	HUAMBO	1985	278.29	201.71	367.19	RW2
Angola	HUAMBO	1986	275.09	202.56	358.39	RW2
Angola	HUAMBO	1987	274.41	204.15	354.12	RW2
Angola	HUAMBO	1988	275.98	207.10	353.77	RW2
Angola	HUAMBO	1989	279.17	210.59	357.02	RW2
Angola	HUAMBO	1990	285.20	220.17	363.03	RW2
Angola	HUAMBO	1991	288.18	225.38	362.58	RW2
Angola	HUAMBO	1992	289.60	227.45	362.10	RW2
Angola	HUAMBO	1993	287.97	226.10	362.31	RW2
Angola	HUAMBO	1994	284.74	223.06	359.84	RW2
Angola	HUAMBO	1995	277.86	216.46	349.66	RW2
Angola	HUAMBO	1996	273.35	214.03	342.12	RW2
Angola	HUAMBO	1997	269.56	211.76	336.60	RW2
Angola	HUAMBO	1998	266.99	207.78	336.43	RW2
Angola	HUAMBO	1999	265.80	204.63	337.79	RW2
Angola	HUAMBO	2000	266.87	203.68	343.07	RW2
Angola	HUAMBO	2001	266.13	204.31	341.52	RW2
Angola	HUAMBO	2002	264.76	202.38	339.68	RW2
Angola	HUAMBO	2003	261.66	196.35	340.70	RW2
Angola	HUAMBO	2004	257.31	185.95	345.82	RW2
Angola	HUAMBO	2005	250.94	170.75	352.30	RW2
Angola	HUAMBO	2006	244.24	160.55	354.20	RW2
Angola	HUAMBO	2007	236.67	151.17	348.68	RW2
Angola	HUAMBO	2008	229.03	145.09	341.69	RW2
Angola	HUAMBO	2009	221.10	141.71	327.22	RW2
Angola	HUAMBO	2010	213.28	141.65	308.65	RW2
Angola	HUAMBO	2011	205.12	140.81	288.68	RW2
Angola	HUAMBO	2012	197.46	136.82	275.85	RW2
Angola	HUAMBO	2013	189.88	125.54	276.45	RW2
Angola	HUAMBO	2014	182.52	107.43	292.89	RW2
Angola	HUAMBO	2015	174.88	84.63	325.55	RW2
Angola	HUAMBO	2016	167.89	65.63	367.17	RW2
Angola	HUAMBO	2017	161.37	49.27	418.30	RW2
Angola	HUAMBO	2018	154.88	36.13	479.28	RW2
Angola	HUAMBO	2019	148.37	24.68	547.60	RW2
Angola	HUÍLA	1980	257.55	135.88	440.12	RW2
Angola	HUÍLA	1981	252.10	145.29	403.65	RW2
Angola	HUÍLA	1982	246.01	150.87	378.45	RW2
Angola	HUÍLA	1983	240.57	152.59	359.53	RW2
Angola	HUÍLA	1984	236.03	153.19	346.63	RW2
Angola	HUÍLA	1985	231.55	154.55	330.20	RW2
Angola	HUÍLA	1986	230.22	159.84	320.41	RW2
Angola	HUÍLA	1987	231.23	164.31	314.55	RW2
Angola	HUÍLA	1988	234.88	169.01	314.39	RW2
Angola	HUÍLA	1989	240.14	176.02	316.68	RW2
Angola	HUÍLA	1990	248.09	187.05	323.05	RW2
Angola	HUÍLA	1991	253.25	194.26	324.23	RW2
Angola	HUÍLA	1992	256.76	199.37	325.69	RW2
Angola	HUÍLA	1993	258.66	201.14	327.36	RW2
Angola	HUÍLA	1994	258.65	201.21	328.74	RW2
Angola	HUÍLA	1995	255.15	197.36	320.98	RW2
Angola	HUÍLA	1996	254.11	199.65	315.99	RW2
Angola	HUÍLA	1997	253.47	200.20	314.98	RW2
Angola	HUÍLA	1998	254.69	200.16	317.11	RW2
Angola	HUÍLA	1999	257.15	199.94	322.63	RW2
Angola	HUÍLA	2000	261.39	203.24	330.81	RW2
Angola	HUÍLA	2001	264.59	207.04	332.85	RW2
Angola	HUÍLA	2002	266.87	208.33	334.91	RW2

Continued on next page

Country	Region	Year	Median	Lower	Upper	Method
Angola	HUÍLA	2003	267.76	205.43	340.31	RW2
Angola	HUÍLA	2004	267.53	198.97	349.55	RW2
Angola	HUÍLA	2005	264.93	184.47	364.47	RW2
Angola	HUÍLA	2006	262.23	176.27	369.88	RW2
Angola	HUÍLA	2007	258.57	169.74	371.91	RW2
Angola	HUÍLA	2008	254.63	164.81	369.40	RW2
Angola	HUÍLA	2009	250.32	164.54	360.17	RW2
Angola	HUÍLA	2010	245.62	167.65	345.15	RW2
Angola	HUÍLA	2011	241.23	169.85	329.98	RW2
Angola	HUÍLA	2012	236.60	169.03	320.54	RW2
Angola	HUÍLA	2013	231.84	158.61	325.50	RW2
Angola	HUÍLA	2014	227.05	138.50	349.35	RW2
Angola	HUÍLA	2015	222.44	113.04	393.06	RW2
Angola	HUÍLA	2016	218.58	88.19	443.06	RW2
Angola	HUÍLA	2017	213.07	68.17	502.79	RW2
Angola	HUÍLA	2018	208.55	50.84	565.88	RW2
Angola	HUÍLA	2019	205.15	36.35	633.60	RW2
Angola	LUANDA	1980	208.38	107.19	367.85	RW2
Angola	LUANDA	1981	200.13	112.65	330.10	RW2
Angola	LUANDA	1982	190.68	113.66	302.51	RW2
Angola	LUANDA	1983	182.91	111.75	283.84	RW2
Angola	LUANDA	1984	175.69	109.70	269.87	RW2
Angola	LUANDA	1985	168.94	107.71	253.80	RW2
Angola	LUANDA	1986	164.90	108.16	242.05	RW2
Angola	LUANDA	1987	163.10	109.04	234.93	RW2
Angola	LUANDA	1988	162.71	110.61	230.28	RW2
Angola	LUANDA	1989	164.29	113.23	229.48	RW2
Angola	LUANDA	1990	167.86	118.90	231.29	RW2
Angola	LUANDA	1991	169.45	123.18	228.35	RW2
Angola	LUANDA	1992	170.05	126.24	226.18	RW2
Angola	LUANDA	1993	169.48	126.53	224.87	RW2
Angola	LUANDA	1994	167.83	125.73	222.87	RW2
Angola	LUANDA	1995	163.81	122.83	213.60	RW2
Angola	LUANDA	1996	161.27	122.52	208.33	RW2
Angola	LUANDA	1997	159.32	122.19	205.21	RW2
Angola	LUANDA	1998	158.47	120.61	205.11	RW2
Angola	LUANDA	1999	158.09	118.66	207.46	RW2
Angola	LUANDA	2000	159.26	119.10	212.02	RW2
Angola	LUANDA	2001	159.23	118.69	212.00	RW2
Angola	LUANDA	2002	158.28	117.39	211.18	RW2
Angola	LUANDA	2003	156.78	113.52	214.57	RW2
Angola	LUANDA	2004	153.79	106.50	218.10	RW2
Angola	LUANDA	2005	149.49	96.83	223.81	RW2
Angola	LUANDA	2006	144.96	89.45	226.45	RW2
Angola	LUANDA	2007	140.10	83.19	226.39	RW2
Angola	LUANDA	2008	134.95	79.13	220.84	RW2
Angola	LUANDA	2009	129.81	75.58	213.32	RW2
Angola	LUANDA	2010	124.42	73.95	200.89	RW2
Angola	LUANDA	2011	119.42	71.62	191.27	RW2
Angola	LUANDA	2012	114.55	67.59	185.63	RW2
Angola	LUANDA	2013	109.41	60.87	187.14	RW2
Angola	LUANDA	2014	104.65	51.75	198.87	RW2
Angola	LUANDA	2015	99.99	40.47	222.30	RW2
Angola	LUANDA	2016	95.56	31.57	252.67	RW2
Angola	LUANDA	2017	91.08	23.29	291.45	RW2
Angola	LUANDA	2018	87.61	16.70	345.13	RW2
Angola	LUANDA	2019	83.23	11.93	410.49	RW2
Angola	LUNDA NORTE	1980	207.09	71.89	473.43	RW2
Angola	LUNDA NORTE	1981	200.22	77.95	427.42	RW2
Angola	LUNDA NORTE	1982	193.53	82.54	392.62	RW2
Angola	LUNDA NORTE	1983	186.32	85.66	364.04	RW2
Angola	LUNDA NORTE	1984	181.31	88.24	340.22	RW2

Continued on next page

Country	Region	Year	Median	Lower	Upper	Method
Angola	LUNDA NORTE	1985	174.85	90.06	312.69	RW2
Angola	LUNDA NORTE	1986	172.55	93.83	294.95	RW2
Angola	LUNDA NORTE	1987	171.99	98.26	283.49	RW2
Angola	LUNDA NORTE	1988	173.13	103.51	274.68	RW2
Angola	LUNDA NORTE	1989	175.83	107.86	271.18	RW2
Angola	LUNDA NORTE	1990	181.17	115.99	273.02	RW2
Angola	LUNDA NORTE	1991	183.49	121.06	269.46	RW2
Angola	LUNDA NORTE	1992	184.97	125.28	265.69	RW2
Angola	LUNDA NORTE	1993	184.85	127.25	262.11	RW2
Angola	LUNDA NORTE	1994	183.42	127.14	258.72	RW2
Angola	LUNDA NORTE	1995	179.40	125.19	250.52	RW2
Angola	LUNDA NORTE	1996	177.08	124.95	244.11	RW2
Angola	LUNDA NORTE	1997	175.73	125.17	241.63	RW2
Angola	LUNDA NORTE	1998	175.79	123.41	242.27	RW2
Angola	LUNDA NORTE	1999	176.28	122.22	244.59	RW2
Angola	LUNDA NORTE	2000	178.62	123.67	252.29	RW2
Angola	LUNDA NORTE	2001	180.71	124.08	254.28	RW2
Angola	LUNDA NORTE	2002	181.63	123.67	257.13	RW2
Angola	LUNDA NORTE	2003	181.76	121.60	261.49	RW2
Angola	LUNDA NORTE	2004	180.83	116.89	269.11	RW2
Angola	LUNDA NORTE	2005	178.96	109.02	277.20	RW2
Angola	LUNDA NORTE	2006	176.30	103.14	284.04	RW2
Angola	LUNDA NORTE	2007	174.10	99.01	287.33	RW2
Angola	LUNDA NORTE	2008	170.98	95.70	285.36	RW2
Angola	LUNDA NORTE	2009	167.97	93.99	280.53	RW2
Angola	LUNDA NORTE	2010	165.08	94.60	272.52	RW2
Angola	LUNDA NORTE	2011	161.99	93.91	265.49	RW2
Angola	LUNDA NORTE	2012	159.05	90.54	263.14	RW2
Angola	LUNDA NORTE	2013	155.67	83.97	270.55	RW2
Angola	LUNDA NORTE	2014	152.52	73.78	290.48	RW2
Angola	LUNDA NORTE	2015	149.50	60.94	325.93	RW2
Angola	LUNDA NORTE	2016	145.99	47.89	368.83	RW2
Angola	LUNDA NORTE	2017	143.23	37.15	423.92	RW2
Angola	LUNDA NORTE	2018	140.69	26.91	489.65	RW2
Angola	LUNDA NORTE	2019	136.91	19.64	563.14	RW2
Angola	LUNDA SUL	1980	113.38	47.29	247.28	RW2
Angola	LUNDA SUL	1981	110.76	50.55	225.04	RW2
Angola	LUNDA SUL	1982	108.36	52.52	210.33	RW2
Angola	LUNDA SUL	1983	106.43	53.43	199.52	RW2
Angola	LUNDA SUL	1984	104.64	54.54	191.50	RW2
Angola	LUNDA SUL	1985	103.14	55.31	183.71	RW2
Angola	LUNDA SUL	1986	103.30	56.88	178.30	RW2
Angola	LUNDA SUL	1987	104.93	59.89	175.88	RW2
Angola	LUNDA SUL	1988	107.48	63.18	176.14	RW2
Angola	LUNDA SUL	1989	111.58	67.01	178.22	RW2
Angola	LUNDA SUL	1990	117.02	73.05	182.04	RW2
Angola	LUNDA SUL	1991	120.83	77.70	182.82	RW2
Angola	LUNDA SUL	1992	123.79	81.45	184.29	RW2
Angola	LUNDA SUL	1993	126.08	84.06	184.33	RW2
Angola	LUNDA SUL	1994	126.94	85.76	185.48	RW2
Angola	LUNDA SUL	1995	125.74	85.01	181.83	RW2
Angola	LUNDA SUL	1996	126.22	86.73	179.76	RW2
Angola	LUNDA SUL	1997	126.95	87.71	180.20	RW2
Angola	LUNDA SUL	1998	128.53	89.07	181.72	RW2
Angola	LUNDA SUL	1999	130.99	90.19	186.37	RW2
Angola	LUNDA SUL	2000	134.81	92.66	193.06	RW2
Angola	LUNDA SUL	2001	137.76	95.31	195.81	RW2
Angola	LUNDA SUL	2002	140.29	96.92	199.87	RW2
Angola	LUNDA SUL	2003	142.45	96.68	204.96	RW2
Angola	LUNDA SUL	2004	143.50	94.39	212.70	RW2
Angola	LUNDA SUL	2005	142.89	88.64	221.50	RW2
Angola	LUNDA SUL	2006	142.71	84.87	229.81	RW2
Angola	LUNDA SUL	2007	142.20	82.03	233.89	RW2

Continued on next page

Country	Region	Year	Median	Lower	Upper	Method
Angola	LUNDA SUL	2008	141.02	80.59	235.36	RW2
Angola	LUNDA SUL	2009	140.16	80.25	233.99	RW2
Angola	LUNDA SUL	2010	138.89	81.06	226.15	RW2
Angola	LUNDA SUL	2011	137.46	81.57	222.11	RW2
Angola	LUNDA SUL	2012	136.26	80.31	221.94	RW2
Angola	LUNDA SUL	2013	134.65	75.22	228.36	RW2
Angola	LUNDA SUL	2014	133.26	66.61	247.64	RW2
Angola	LUNDA SUL	2015	132.18	55.17	282.92	RW2
Angola	LUNDA SUL	2016	130.96	44.64	328.31	RW2
Angola	LUNDA SUL	2017	128.90	34.36	384.36	RW2
Angola	LUNDA SUL	2018	127.91	26.25	448.53	RW2
Angola	LUNDA SUL	2019	126.55	19.48	520.23	RW2
Angola	MALANJE	1980	293.22	125.86	555.11	RW2
Angola	MALANJE	1981	278.35	128.38	510.52	RW2
Angola	MALANJE	1982	263.70	128.28	475.68	RW2
Angola	MALANJE	1983	250.60	126.89	442.47	RW2
Angola	MALANJE	1984	238.88	123.55	414.95	RW2
Angola	MALANJE	1985	227.86	121.55	385.93	RW2
Angola	MALANJE	1986	220.29	121.83	363.56	RW2
Angola	MALANJE	1987	214.98	123.40	347.56	RW2
Angola	MALANJE	1988	211.89	125.78	332.95	RW2
Angola	MALANJE	1989	210.85	128.18	325.64	RW2
Angola	MALANJE	1990	211.90	134.06	319.96	RW2
Angola	MALANJE	1991	211.50	138.22	310.58	RW2
Angola	MALANJE	1992	209.55	139.84	301.89	RW2
Angola	MALANJE	1993	205.93	139.95	293.51	RW2
Angola	MALANJE	1994	200.86	138.11	283.48	RW2
Angola	MALANJE	1995	193.89	134.20	269.31	RW2
Angola	MALANJE	1996	188.36	133.18	259.00	RW2
Angola	MALANJE	1997	184.42	131.96	249.99	RW2
Angola	MALANJE	1998	181.62	130.76	246.06	RW2
Angola	MALANJE	1999	180.01	129.03	244.36	RW2
Angola	MALANJE	2000	180.27	129.76	244.97	RW2
Angola	MALANJE	2001	179.25	130.10	241.88	RW2
Angola	MALANJE	2002	177.82	129.60	239.53	RW2
Angola	MALANJE	2003	175.50	125.95	239.48	RW2
Angola	MALANJE	2004	172.34	119.33	243.67	RW2
Angola	MALANJE	2005	167.90	109.17	248.03	RW2
Angola	MALANJE	2006	163.21	101.64	250.07	RW2
Angola	MALANJE	2007	158.34	95.21	249.68	RW2
Angola	MALANJE	2008	152.92	91.11	242.81	RW2
Angola	MALANJE	2009	147.83	88.96	234.38	RW2
Angola	MALANJE	2010	142.56	88.25	221.79	RW2
Angola	MALANJE	2011	137.41	87.25	209.15	RW2
Angola	MALANJE	2012	132.57	84.09	202.19	RW2
Angola	MALANJE	2013	127.48	77.35	204.65	RW2
Angola	MALANJE	2014	122.98	66.40	216.52	RW2
Angola	MALANJE	2015	118.04	53.20	244.90	RW2
Angola	MALANJE	2016	113.85	41.15	279.42	RW2
Angola	MALANJE	2017	109.09	30.54	323.28	RW2
Angola	MALANJE	2018	105.34	22.29	378.66	RW2
Angola	MALANJE	2019	101.22	15.50	442.90	RW2
Angola	MOXICO	1980	488.52	20.71	973.37	RW2
Angola	MOXICO	1981	451.90	20.53	964.30	RW2
Angola	MOXICO	1982	423.52	21.88	952.06	RW2
Angola	MOXICO	1983	388.32	21.99	936.07	RW2
Angola	MOXICO	1984	356.02	23.09	917.95	RW2
Angola	MOXICO	1985	328.45	22.64	894.16	RW2
Angola	MOXICO	1986	306.10	24.48	867.04	RW2
Angola	MOXICO	1987	285.68	25.55	834.17	RW2
Angola	MOXICO	1988	266.11	27.60	799.30	RW2
Angola	MOXICO	1989	252.99	29.40	764.87	RW2
Angola	MOXICO	1990	242.58	31.85	727.45	RW2

Continued on next page

Country	Region	Year	Median	Lower	Upper	Method
Angola	MOXICO	1991	229.93	34.01	683.90	RW2
Angola	MOXICO	1992	216.28	36.09	634.40	RW2
Angola	MOXICO	1993	202.11	37.32	585.47	RW2
Angola	MOXICO	1994	186.22	39.00	528.86	RW2
Angola	MOXICO	1995	168.91	39.17	468.67	RW2
Angola	MOXICO	1996	155.52	40.04	416.01	RW2
Angola	MOXICO	1997	143.58	40.76	364.14	RW2
Angola	MOXICO	1998	132.73	41.25	324.96	RW2
Angola	MOXICO	1999	123.49	41.77	289.59	RW2
Angola	MOXICO	2000	116.53	42.64	261.80	RW2
Angola	MOXICO	2001	109.41	42.45	238.25	RW2
Angola	MOXICO	2002	101.44	41.36	215.64	RW2
Angola	MOXICO	2003	94.13	38.83	201.04	RW2
Angola	MOXICO	2004	86.70	35.44	190.20	RW2
Angola	MOXICO	2005	78.74	30.30	184.51	RW2
Angola	MOXICO	2006	72.17	26.29	180.09	RW2
Angola	MOXICO	2007	65.18	22.24	176.55	RW2
Angola	MOXICO	2008	59.18	18.52	171.86	RW2
Angola	MOXICO	2009	53.36	15.58	168.98	RW2
Angola	MOXICO	2010	48.20	12.91	165.35	RW2
Angola	MOXICO	2011	43.59	10.49	164.12	RW2
Angola	MOXICO	2012	39.37	8.39	165.34	RW2
Angola	MOXICO	2013	35.31	6.64	169.03	RW2
Angola	MOXICO	2014	32.05	5.10	175.80	RW2
Angola	MOXICO	2015	28.54	3.70	187.61	RW2
Angola	MOXICO	2016	25.64	2.69	206.47	RW2
Angola	MOXICO	2017	23.13	1.87	231.22	RW2
Angola	MOXICO	2018	20.77	1.29	260.34	RW2
Angola	MOXICO	2019	18.72	0.86	300.06	RW2
Angola	NAMIBE	1980	193.05	95.58	347.43	RW2
Angola	NAMIBE	1981	190.14	102.65	318.65	RW2
Angola	NAMIBE	1982	187.61	108.28	300.18	RW2
Angola	NAMIBE	1983	185.23	110.84	291.18	RW2
Angola	NAMIBE	1984	183.31	112.53	283.47	RW2
Angola	NAMIBE	1985	182.05	114.38	274.02	RW2
Angola	NAMIBE	1986	182.89	118.57	269.71	RW2
Angola	NAMIBE	1987	186.21	123.82	268.31	RW2
Angola	NAMIBE	1988	191.25	129.72	270.79	RW2
Angola	NAMIBE	1989	197.62	136.27	275.20	RW2
Angola	NAMIBE	1990	206.81	147.27	284.05	RW2
Angola	NAMIBE	1991	213.73	155.49	287.21	RW2
Angola	NAMIBE	1992	218.95	161.32	290.33	RW2
Angola	NAMIBE	1993	222.04	164.88	293.50	RW2
Angola	NAMIBE	1994	223.30	166.24	295.36	RW2
Angola	NAMIBE	1995	221.48	165.20	291.21	RW2
Angola	NAMIBE	1996	221.71	166.99	289.02	RW2
Angola	NAMIBE	1997	221.96	167.93	288.52	RW2
Angola	NAMIBE	1998	223.77	168.21	293.04	RW2
Angola	NAMIBE	1999	226.39	167.34	298.29	RW2
Angola	NAMIBE	2000	230.59	170.59	306.81	RW2
Angola	NAMIBE	2001	233.56	172.56	310.90	RW2
Angola	NAMIBE	2002	235.15	172.91	313.69	RW2
Angola	NAMIBE	2003	235.51	169.79	318.66	RW2
Angola	NAMIBE	2004	234.59	163.87	325.78	RW2
Angola	NAMIBE	2005	231.34	151.56	335.42	RW2
Angola	NAMIBE	2006	227.83	143.12	341.32	RW2
Angola	NAMIBE	2007	223.54	137.37	342.02	RW2
Angola	NAMIBE	2008	218.73	132.97	338.80	RW2
Angola	NAMIBE	2009	213.48	130.32	328.57	RW2
Angola	NAMIBE	2010	208.31	131.05	313.86	RW2
Angola	NAMIBE	2011	202.70	129.88	301.00	RW2
Angola	NAMIBE	2012	197.15	126.44	293.44	RW2
Angola	NAMIBE	2013	191.75	117.05	298.26	RW2

Continued on next page

Country	Region	Year	Median	Lower	Upper	Method
Angola	NAMIBE	2014	186.24	101.35	316.00	RW2
Angola	NAMIBE	2015	181.60	82.66	354.88	RW2
Angola	NAMIBE	2016	176.54	64.13	397.99	RW2
Angola	NAMIBE	2017	170.93	49.19	452.11	RW2
Angola	NAMIBE	2018	166.00	35.69	520.79	RW2
Angola	NAMIBE	2019	161.68	25.63	580.42	RW2
Angola	UÍGE	1980	440.20	275.17	632.91	RW2
Angola	UÍGE	1981	413.77	275.62	578.65	RW2
Angola	UÍGE	1982	389.45	268.15	533.96	RW2
Angola	UÍGE	1983	365.42	254.93	496.99	RW2
Angola	UÍGE	1984	343.06	240.61	466.86	RW2
Angola	UÍGE	1985	321.70	225.79	432.20	RW2
Angola	UÍGE	1986	304.32	217.25	406.70	RW2
Angola	UÍGE	1987	291.18	209.18	386.21	RW2
Angola	UÍGE	1988	280.55	202.91	371.88	RW2
Angola	UÍGE	1989	272.97	197.48	361.76	RW2
Angola	UÍGE	1990	268.72	196.03	355.47	RW2
Angola	UÍGE	1991	261.98	192.44	344.78	RW2
Angola	UÍGE	1992	254.78	187.34	333.95	RW2
Angola	UÍGE	1993	246.38	180.29	325.58	RW2
Angola	UÍGE	1994	236.47	171.60	313.95	RW2
Angola	UÍGE	1995	225.25	161.95	297.75	RW2
Angola	UÍGE	1996	216.28	157.06	284.76	RW2
Angola	UÍGE	1997	209.09	152.88	274.20	RW2
Angola	UÍGE	1998	203.64	148.06	268.74	RW2
Angola	UÍGE	1999	199.46	144.04	265.06	RW2
Angola	UÍGE	2000	197.72	143.07	265.47	RW2
Angola	UÍGE	2001	194.47	141.23	260.00	RW2
Angola	UÍGE	2002	190.90	138.94	255.38	RW2
Angola	UÍGE	2003	186.69	134.29	253.43	RW2
Angola	UÍGE	2004	181.15	125.46	254.57	RW2
Angola	UÍGE	2005	174.41	113.83	257.41	RW2
Angola	UÍGE	2006	167.86	104.74	255.91	RW2
Angola	UÍGE	2007	160.57	97.93	251.69	RW2
Angola	UÍGE	2008	153.88	92.78	243.86	RW2
Angola	UÍGE	2009	146.93	89.10	231.79	RW2
Angola	UÍGE	2010	139.86	88.16	214.93	RW2
Angola	UÍGE	2011	132.95	85.96	200.54	RW2
Angola	UÍGE	2012	126.53	82.09	190.05	RW2
Angola	UÍGE	2013	120.33	74.41	189.80	RW2
Angola	UÍGE	2014	114.24	62.83	202.12	RW2
Angola	UÍGE	2015	108.69	48.95	225.83	RW2
Angola	UÍGE	2016	103.00	37.35	257.21	RW2
Angola	UÍGE	2017	98.03	27.18	298.12	RW2
Angola	UÍGE	2018	92.73	19.55	349.21	RW2
Angola	UÍGE	2019	88.06	13.48	410.62	RW2
Angola	ZAIRE	1980	77.01	22.86	249.93	RW2
Angola	ZAIRE	1981	74.93	25.11	218.97	RW2
Angola	ZAIRE	1982	73.06	27.01	193.88	RW2
Angola	ZAIRE	1983	71.33	28.32	177.14	RW2
Angola	ZAIRE	1984	70.27	29.85	162.21	RW2
Angola	ZAIRE	1985	68.75	31.49	147.03	RW2
Angola	ZAIRE	1986	68.74	33.29	138.54	RW2
Angola	ZAIRE	1987	69.54	35.69	131.98	RW2
Angola	ZAIRE	1988	71.28	38.37	128.80	RW2
Angola	ZAIRE	1989	74.00	41.39	127.59	RW2
Angola	ZAIRE	1990	77.70	45.57	130.02	RW2
Angola	ZAIRE	1991	81.04	49.60	129.87	RW2
Angola	ZAIRE	1992	83.77	52.80	130.58	RW2
Angola	ZAIRE	1993	85.84	55.30	131.03	RW2

Continued on next page

Country	Region	Year	Median	Lower	Upper	Method
Angola	ZAIRE	1994	87.73	57.63	132.64	RW2
Angola	ZAIRE	1995	88.61	58.81	130.76	RW2
Angola	ZAIRE	1996	90.41	61.36	130.66	RW2
Angola	ZAIRE	1997	92.78	63.74	133.30	RW2
Angola	ZAIRE	1998	96.02	65.71	138.02	RW2
Angola	ZAIRE	1999	100.14	67.92	144.97	RW2
Angola	ZAIRE	2000	105.12	71.26	154.37	RW2
Angola	ZAIRE	2001	109.76	74.01	161.59	RW2
Angola	ZAIRE	2002	114.30	76.78	167.84	RW2
Angola	ZAIRE	2003	117.73	77.70	176.44	RW2
Angola	ZAIRE	2004	120.90	77.10	185.95	RW2
Angola	ZAIRE	2005	122.54	73.52	198.10	RW2
Angola	ZAIRE	2006	124.23	71.49	205.65	RW2
Angola	ZAIRE	2007	125.31	69.82	212.85	RW2
Angola	ZAIRE	2008	126.09	69.70	217.34	RW2
Angola	ZAIRE	2009	126.29	70.10	215.61	RW2
Angola	ZAIRE	2010	126.73	71.71	212.55	RW2
Angola	ZAIRE	2011	126.81	73.07	209.80	RW2
Angola	ZAIRE	2012	126.84	72.52	212.04	RW2
Angola	ZAIRE	2013	126.87	68.66	222.84	RW2
Angola	ZAIRE	2014	126.93	61.52	244.59	RW2
Angola	ZAIRE	2015	126.42	51.27	281.03	RW2
Angola	ZAIRE	2016	126.81	41.32	327.70	RW2
Angola	ZAIRE	2017	126.52	31.92	383.88	RW2
Angola	ZAIRE	2018	127.50	24.59	456.30	RW2
Angola	ZAIRE	2019	127.39	17.97	533.64	RW2
Benin	ALL	1980	223.93	217.05	230.95	IHME
Benin	ALL	1980	216.37	162.37	281.94	RW2
Benin	ALL	1980	217.40	203.60	232.00	UN
Benin	ALL	1981	218.77	212.00	225.59	IHME
Benin	ALL	1981	213.27	174.73	257.40	RW2
Benin	ALL	1981	213.70	200.60	227.80	UN
Benin	ALL	1982	213.78	207.45	220.20	IHME
Benin	ALL	1982	210.26	175.10	250.22	RW2
Benin	ALL	1982	210.20	197.60	224.00	UN
Benin	ALL	1983	209.44	203.18	215.64	IHME
Benin	ALL	1983	206.95	168.16	251.47	RW2
Benin	ALL	1983	206.80	194.40	220.40	UN
Benin	ALL	1984	205.04	199.05	211.06	IHME
Benin	ALL	1984	203.90	161.62	252.56	RW2
Benin	ALL	1984	203.30	191.00	216.60	UN
Benin	ALL	1985	200.55	194.56	206.46	IHME
Benin	ALL	1985	200.44	162.65	244.66	RW2
Benin	ALL	1985	199.70	187.50	213.00	UN
Benin	ALL	1986	196.11	190.30	201.82	IHME
Benin	ALL	1986	196.70	162.27	236.16	RW2
Benin	ALL	1986	196.10	183.80	209.30	UN
Benin	ALL	1987	192.01	186.55	197.23	IHME
Benin	ALL	1987	192.68	160.61	229.98	RW2
Benin	ALL	1987	192.20	180.20	205.30	UN
Benin	ALL	1988	187.91	182.84	193.22	IHME
Benin	ALL	1988	188.22	155.09	226.67	RW2
Benin	ALL	1988	188.10	176.40	201.10	UN
Benin	ALL	1989	184.16	178.89	189.64	IHME
Benin	ALL	1989	183.60	149.37	224.22	RW2
Benin	ALL	1989	183.90	172.60	196.30	UN
Benin	ALL	1990	180.25	175.05	185.80	IHME
Benin	ALL	1990	178.71	145.13	217.92	RW2
Benin	ALL	1990	179.50	168.40	191.40	UN
Benin	ALL	1991	176.26	171.23	181.69	IHME
Benin	ALL	1991	174.25	143.40	209.41	RW2
Benin	ALL	1991	175.00	164.30	186.30	UN
Benin	ALL	1992	172.37	167.57	177.99	IHME

Continued on next page

Country	Region	Year	Median	Lower	Upper	Method
Benin	ALL	1992	170.07	140.75	203.65	RW2
Benin	ALL	1992	170.40	159.90	181.40	UN
Benin	ALL	1993	168.45	163.67	173.86	IHME
Benin	ALL	1993	166.28	136.76	200.73	RW2
Benin	ALL	1993	166.20	155.60	177.00	UN
Benin	ALL	1994	164.77	159.82	170.12	IHME
Benin	ALL	1994	162.75	131.84	199.86	RW2
Benin	ALL	1994	162.40	152.10	173.10	UN
Benin	ALL	1995	160.98	156.09	166.22	IHME
Benin	ALL	1995	159.76	129.68	195.71	RW2
Benin	ALL	1995	159.10	149.00	169.70	UN
Benin	ALL	1996	157.01	152.05	162.11	IHME
Benin	ALL	1996	156.63	128.66	190.23	RW2
Benin	ALL	1996	156.20	146.30	166.70	UN
Benin	ALL	1997	152.93	147.92	157.98	IHME
Benin	ALL	1997	153.58	126.95	184.71	RW2
Benin	ALL	1997	153.60	143.80	164.10	UN
Benin	ALL	1998	148.78	143.88	153.72	IHME
Benin	ALL	1998	150.56	123.38	183.11	RW2
Benin	ALL	1998	150.90	141.10	161.80	UN
Benin	ALL	1999	144.45	139.48	149.58	IHME
Benin	ALL	1999	147.40	118.79	180.73	RW2
Benin	ALL	1999	147.90	138.00	159.30	UN
Benin	ALL	2000	140.00	135.08	145.22	IHME
Benin	ALL	2000	144.12	116.03	177.43	RW2
Benin	ALL	2000	144.70	134.60	155.70	UN
Benin	ALL	2001	135.42	130.46	140.41	IHME
Benin	ALL	2001	140.71	114.48	171.58	RW2
Benin	ALL	2001	141.10	131.10	151.70	UN
Benin	ALL	2002	130.72	125.83	135.62	IHME
Benin	ALL	2002	137.12	112.63	166.01	RW2
Benin	ALL	2002	137.10	127.40	147.60	UN
Benin	ALL	2003	126.07	121.13	130.81	IHME
Benin	ALL	2003	133.50	108.96	162.72	RW2
Benin	ALL	2003	133.10	123.30	143.70	UN
Benin	ALL	2004	121.39	116.45	126.45	IHME
Benin	ALL	2004	129.57	103.02	161.51	RW2
Benin	ALL	2004	129.20	118.70	140.10	UN
Benin	ALL	2005	116.84	111.73	122.01	IHME
Benin	ALL	2005	125.75	97.47	160.53	RW2
Benin	ALL	2005	125.50	114.00	136.90	UN
Benin	ALL	2006	112.41	106.94	117.66	IHME
Benin	ALL	2006	121.85	95.38	154.06	RW2
Benin	ALL	2006	122.10	109.50	134.20	UN
Benin	ALL	2007	108.01	102.17	113.64	IHME
Benin	ALL	2007	118.06	93.65	147.70	RW2
Benin	ALL	2007	119.00	105.60	131.90	UN
Benin	ALL	2008	103.73	97.81	109.78	IHME
Benin	ALL	2008	114.52	88.12	147.62	RW2
Benin	ALL	2008	116.30	102.00	129.70	UN
Benin	ALL	2009	99.63	93.34	105.90	IHME
Benin	ALL	2009	110.75	77.03	157.33	RW2
Benin	ALL	2009	113.90	98.70	128.20	UN
Benin	ALL	2010	95.78	89.20	102.94	IHME
Benin	ALL	2010	107.15	62.14	180.23	RW2
Benin	ALL	2010	111.60	95.40	127.10	UN
Benin	ALL	2011	92.12	85.19	99.97	IHME
Benin	ALL	2011	103.83	48.94	206.73	RW2
Benin	ALL	2011	109.30	92.10	126.60	UN
Benin	ALL	2012	88.65	81.20	97.16	IHME
Benin	ALL	2012	100.38	37.26	242.48	RW2
Benin	ALL	2012	107.00	88.70	126.60	UN
Benin	ALL	2013	85.00	77.16	94.39	IHME

Continued on next page

Country	Region	Year	Median	Lower	Upper	Method
Benin	ALL	2013	97.43	28.01	285.54	RW2
Benin	ALL	2013	104.80	85.40	126.50	UN
Benin	ALL	2014	81.41	73.17	91.20	IHME
Benin	ALL	2014	94.18	20.48	338.63	RW2
Benin	ALL	2014	102.10	81.60	126.70	UN
Benin	ALL	2015	77.88	69.51	88.30	IHME
Benin	ALL	2015	90.77	14.90	396.73	RW2
Benin	ALL	2015	99.50	78.30	126.90	UN
Benin	ALL	2016	88.43	10.69	470.34	RW2
Benin	ALL	2017	85.05	7.31	545.59	RW2
Benin	ALL	2018	82.24	5.09	627.02	RW2
Benin	ALL	2019	79.29	3.17	692.07	RW2
Benin	ALL	15-19	85.05	7.37	540.55	RW2
Benin	ATACORA	1980	281.56	222.81	349.24	RW2
Benin	ATACORA	1981	275.58	234.62	321.39	RW2
Benin	ATACORA	1982	269.41	232.77	309.38	RW2
Benin	ATACORA	1983	263.34	223.39	307.40	RW2
Benin	ATACORA	1984	257.25	214.53	304.30	RW2
Benin	ATACORA	1985	251.44	213.38	293.87	RW2
Benin	ATACORA	1986	245.43	211.59	282.11	RW2
Benin	ATACORA	1987	239.36	207.92	273.77	RW2
Benin	ATACORA	1988	233.34	200.81	269.33	RW2
Benin	ATACORA	1989	227.40	193.40	265.52	RW2
Benin	ATACORA	1990	221.44	188.09	258.40	RW2
Benin	ATACORA	1991	216.18	186.08	249.51	RW2
Benin	ATACORA	1992	211.19	182.55	242.67	RW2
Benin	ATACORA	1993	206.55	177.46	238.95	RW2
Benin	ATACORA	1994	202.57	171.49	237.10	RW2
Benin	ATACORA	1995	198.82	168.46	233.17	RW2
Benin	ATACORA	1996	195.36	167.50	226.48	RW2
Benin	ATACORA	1997	191.95	165.73	221.58	RW2
Benin	ATACORA	1998	188.60	161.94	218.83	RW2
Benin	ATACORA	1999	185.21	155.66	217.72	RW2
Benin	ATACORA	2000	181.67	152.19	215.01	RW2
Benin	ATACORA	2001	177.95	150.50	208.73	RW2
Benin	ATACORA	2002	173.92	147.98	203.27	RW2
Benin	ATACORA	2003	169.55	142.71	200.70	RW2
Benin	ATACORA	2004	165.08	135.43	199.67	RW2
Benin	ATACORA	2005	160.27	128.44	198.21	RW2
Benin	ATACORA	2006	155.62	124.77	192.59	RW2
Benin	ATACORA	2007	150.85	120.04	187.50	RW2
Benin	ATACORA	2008	146.30	112.14	188.47	RW2
Benin	ATACORA	2009	141.56	99.47	197.48	RW2
Benin	ATACORA	2010	137.27	83.10	219.01	RW2
Benin	ATACORA	2011	132.75	67.65	244.80	RW2
Benin	ATACORA	2012	128.54	53.78	277.01	RW2
Benin	ATACORA	2013	124.64	41.78	320.49	RW2
Benin	ATACORA	2014	120.87	31.55	365.12	RW2
Benin	ATACORA	2015	117.02	23.49	418.85	RW2
Benin	ATACORA	2016	112.84	17.12	477.36	RW2
Benin	ATACORA	2017	109.60	12.40	544.09	RW2
Benin	ATACORA	2018	105.45	8.67	613.24	RW2
Benin	ATACORA	2019	102.19	6.03	684.48	RW2
Benin	ATLANTIQUE	1980	178.33	136.08	230.88	RW2
Benin	ATLANTIQUE	1981	174.97	144.88	209.92	RW2
Benin	ATLANTIQUE	1982	171.69	144.53	202.13	RW2
Benin	ATLANTIQUE	1983	168.22	139.24	202.00	RW2
Benin	ATLANTIQUE	1984	164.90	134.63	200.06	RW2
Benin	ATLANTIQUE	1985	161.51	133.60	193.11	RW2
Benin	ATLANTIQUE	1986	157.74	133.32	185.94	RW2
Benin	ATLANTIQUE	1987	153.92	131.37	179.56	RW2
Benin	ATLANTIQUE	1988	149.89	126.86	176.33	RW2
Benin	ATLANTIQUE	1989	145.70	121.69	174.04	RW2

Continued on next page

Country	Region	Year	Median	Lower	Upper	Method
Benin	ATLANTIQUE	1990	141.49	118.06	168.60	RW2
Benin	ATLANTIQUE	1991	137.72	116.45	161.46	RW2
Benin	ATLANTIQUE	1992	134.05	114.27	156.41	RW2
Benin	ATLANTIQUE	1993	130.82	111.02	154.01	RW2
Benin	ATLANTIQUE	1994	128.02	106.86	152.88	RW2
Benin	ATLANTIQUE	1995	125.45	104.65	150.07	RW2
Benin	ATLANTIQUE	1996	123.15	104.24	144.74	RW2
Benin	ATLANTIQUE	1997	121.07	103.23	141.89	RW2
Benin	ATLANTIQUE	1998	119.20	100.75	140.57	RW2
Benin	ATLANTIQUE	1999	117.24	97.44	140.12	RW2
Benin	ATLANTIQUE	2000	115.38	95.69	138.88	RW2
Benin	ATLANTIQUE	2001	113.40	94.96	135.01	RW2
Benin	ATLANTIQUE	2002	111.19	93.70	131.71	RW2
Benin	ATLANTIQUE	2003	108.80	90.44	130.41	RW2
Benin	ATLANTIQUE	2004	106.29	85.99	130.27	RW2
Benin	ATLANTIQUE	2005	103.53	81.62	130.25	RW2
Benin	ATLANTIQUE	2006	100.74	79.68	127.07	RW2
Benin	ATLANTIQUE	2007	98.11	77.35	124.29	RW2
Benin	ATLANTIQUE	2008	95.50	72.41	125.40	RW2
Benin	ATLANTIQUE	2009	92.77	64.29	132.31	RW2
Benin	ATLANTIQUE	2010	90.34	53.42	148.82	RW2
Benin	ATLANTIQUE	2011	87.85	43.80	168.91	RW2
Benin	ATLANTIQUE	2012	85.41	34.73	195.04	RW2
Benin	ATLANTIQUE	2013	83.07	27.01	227.68	RW2
Benin	ATLANTIQUE	2014	80.48	20.12	266.72	RW2
Benin	ATLANTIQUE	2015	78.58	15.19	317.32	RW2
Benin	ATLANTIQUE	2016	76.16	10.99	372.91	RW2
Benin	ATLANTIQUE	2017	74.38	8.01	439.20	RW2
Benin	ATLANTIQUE	2018	71.87	5.73	506.42	RW2
Benin	ATLANTIQUE	2019	70.30	4.10	580.09	RW2
Benin	BORGOU	1980	211.49	162.17	270.50	RW2
Benin	BORGOU	1981	209.36	174.48	250.00	RW2
Benin	BORGOU	1982	207.22	175.34	243.09	RW2
Benin	BORGOU	1983	204.97	170.31	244.35	RW2
Benin	BORGOU	1984	202.70	166.53	244.01	RW2
Benin	BORGOU	1985	200.48	167.63	238.36	RW2
Benin	BORGOU	1986	197.98	168.32	231.25	RW2
Benin	BORGOU	1987	195.21	167.50	226.16	RW2
Benin	BORGOU	1988	192.20	163.72	224.61	RW2
Benin	BORGOU	1989	189.18	159.41	223.77	RW2
Benin	BORGOU	1990	185.67	156.47	219.22	RW2
Benin	BORGOU	1991	182.66	155.47	212.74	RW2
Benin	BORGOU	1992	179.63	154.10	208.30	RW2
Benin	BORGOU	1993	176.65	150.60	206.57	RW2
Benin	BORGOU	1994	173.93	146.29	206.11	RW2
Benin	BORGOU	1995	171.40	144.38	202.75	RW2
Benin	BORGOU	1996	168.66	143.50	197.27	RW2
Benin	BORGOU	1997	166.10	142.60	193.14	RW2
Benin	BORGOU	1998	163.38	139.01	191.56	RW2
Benin	BORGOU	1999	160.58	134.02	190.73	RW2
Benin	BORGOU	2000	157.58	130.77	188.74	RW2
Benin	BORGOU	2001	154.28	129.29	183.30	RW2
Benin	BORGOU	2002	150.77	126.53	178.26	RW2
Benin	BORGOU	2003	146.88	121.52	176.38	RW2
Benin	BORGOU	2004	142.94	115.26	176.05	RW2
Benin	BORGOU	2005	138.84	108.84	174.76	RW2
Benin	BORGOU	2006	134.65	105.01	169.91	RW2
Benin	BORGOU	2007	130.45	100.82	165.70	RW2
Benin	BORGOU	2008	126.30	93.69	166.15	RW2
Benin	BORGOU	2009	122.37	83.34	174.20	RW2
Benin	BORGOU	2010	118.45	69.48	192.87	RW2
Benin	BORGOU	2011	114.56	56.53	216.45	RW2
Benin	BORGOU	2012	111.11	44.70	247.09	RW2

Continued on next page

Country	Region	Year	Median	Lower	Upper	Method
Benin	BORGOU	2013	107.54	35.42	284.68	RW2
Benin	BORGOU	2014	103.84	26.64	328.24	RW2
Benin	BORGOU	2015	100.47	19.65	384.41	RW2
Benin	BORGOU	2016	97.00	14.03	441.38	RW2
Benin	BORGOU	2017	94.10	10.22	506.99	RW2
Benin	BORGOU	2018	90.41	7.17	572.40	RW2
Benin	BORGOU	2019	87.99	4.95	649.75	RW2
Benin	MONO	1980	208.27	159.34	267.93	RW2
Benin	MONO	1981	204.30	169.07	245.02	RW2
Benin	MONO	1982	200.42	168.87	235.91	RW2
Benin	MONO	1983	196.31	162.76	234.50	RW2
Benin	MONO	1984	192.25	157.37	232.95	RW2
Benin	MONO	1985	188.22	156.63	224.35	RW2
Benin	MONO	1986	183.79	156.15	215.21	RW2
Benin	MONO	1987	179.10	153.20	208.22	RW2
Benin	MONO	1988	174.23	148.04	203.99	RW2
Benin	MONO	1989	169.16	141.79	200.58	RW2
Benin	MONO	1990	163.93	137.26	193.70	RW2
Benin	MONO	1991	158.96	134.85	186.14	RW2
Benin	MONO	1992	154.13	131.99	179.82	RW2
Benin	MONO	1993	149.70	127.35	175.91	RW2
Benin	MONO	1994	145.40	121.27	172.75	RW2
Benin	MONO	1995	141.61	118.31	168.60	RW2
Benin	MONO	1996	137.95	116.74	162.74	RW2
Benin	MONO	1997	134.33	114.50	157.20	RW2
Benin	MONO	1998	131.01	110.46	154.49	RW2
Benin	MONO	1999	127.53	105.69	152.39	RW2
Benin	MONO	2000	124.15	102.75	149.17	RW2
Benin	MONO	2001	120.56	100.72	143.87	RW2
Benin	MONO	2002	117.01	98.12	138.93	RW2
Benin	MONO	2003	113.11	93.31	136.04	RW2
Benin	MONO	2004	109.29	87.75	135.08	RW2
Benin	MONO	2005	105.30	82.26	133.58	RW2
Benin	MONO	2006	101.31	78.67	129.12	RW2
Benin	MONO	2007	97.40	74.89	124.94	RW2
Benin	MONO	2008	93.81	69.25	124.92	RW2
Benin	MONO	2009	90.14	60.68	131.16	RW2
Benin	MONO	2010	86.73	50.38	145.48	RW2
Benin	MONO	2011	83.04	40.29	163.12	RW2
Benin	MONO	2012	79.78	31.69	185.76	RW2
Benin	MONO	2013	76.69	24.31	216.07	RW2
Benin	MONO	2014	73.81	18.19	252.40	RW2
Benin	MONO	2015	70.78	13.53	298.68	RW2
Benin	MONO	2016	68.05	9.58	346.73	RW2
Benin	MONO	2017	65.21	6.85	407.30	RW2
Benin	MONO	2018	62.61	4.90	477.75	RW2
Benin	MONO	2019	60.26	3.24	544.36	RW2
Benin	OUEME	1980	211.11	161.53	271.36	RW2
Benin	OUEME	1981	208.01	172.49	249.17	RW2
Benin	OUEME	1982	204.77	173.01	241.09	RW2
Benin	OUEME	1983	201.91	167.56	240.72	RW2
Benin	OUEME	1984	198.57	162.50	239.29	RW2
Benin	OUEME	1985	195.45	162.90	232.09	RW2
Benin	OUEME	1986	191.80	163.02	223.69	RW2
Benin	OUEME	1987	187.96	161.18	217.93	RW2
Benin	OUEME	1988	183.84	156.78	214.07	RW2
Benin	OUEME	1989	179.67	151.40	212.60	RW2
Benin	OUEME	1990	175.34	147.63	206.95	RW2
Benin	OUEME	1991	171.23	146.14	199.72	RW2
Benin	OUEME	1992	167.41	143.77	193.88	RW2
Benin	OUEME	1993	164.04	139.87	191.45	RW2
Benin	OUEME	1994	160.94	135.12	190.60	RW2
Benin	OUEME	1995	158.42	132.96	187.63	RW2

Continued on next page

Country	Region	Year	Median	Lower	Upper	Method
Benin	OUEME	1996	155.99	132.56	182.86	RW2
Benin	OUEME	1997	153.81	131.39	179.23	RW2
Benin	OUEME	1998	151.76	128.87	177.93	RW2
Benin	OUEME	1999	149.73	124.78	178.14	RW2
Benin	OUEME	2000	147.97	123.22	176.79	RW2
Benin	OUEME	2001	145.94	122.79	172.82	RW2
Benin	OUEME	2002	143.76	121.67	169.46	RW2
Benin	OUEME	2003	141.33	118.02	168.36	RW2
Benin	OUEME	2004	138.72	113.35	169.07	RW2
Benin	OUEME	2005	135.97	108.26	168.97	RW2
Benin	OUEME	2006	133.02	106.02	165.91	RW2
Benin	OUEME	2007	130.41	103.59	162.89	RW2
Benin	OUEME	2008	127.60	97.88	165.80	RW2
Benin	OUEME	2009	124.71	87.43	175.71	RW2
Benin	OUEME	2010	121.91	73.20	196.97	RW2
Benin	OUEME	2011	119.57	60.45	222.02	RW2
Benin	OUEME	2012	116.96	48.30	255.62	RW2
Benin	OUEME	2013	113.67	37.62	293.17	RW2
Benin	OUEME	2014	111.65	29.12	346.33	RW2
Benin	OUEME	2015	108.99	21.92	396.99	RW2
Benin	OUEME	2016	106.74	16.00	468.85	RW2
Benin	OUEME	2017	104.95	11.71	535.12	RW2
Benin	OUEME	2018	102.68	8.38	603.73	RW2
Benin	OUEME	2019	99.51	5.75	680.64	RW2
Benin	ZOU	1980	233.86	181.09	296.27	RW2
Benin	ZOU	1981	229.10	192.24	270.56	RW2
Benin	ZOU	1982	224.23	190.90	261.26	RW2
Benin	ZOU	1983	219.28	183.18	260.06	RW2
Benin	ZOU	1984	214.35	176.97	256.68	RW2
Benin	ZOU	1985	209.46	175.23	247.78	RW2
Benin	ZOU	1986	204.35	174.09	237.73	RW2
Benin	ZOU	1987	199.03	171.45	229.54	RW2
Benin	ZOU	1988	193.53	165.18	225.18	RW2
Benin	ZOU	1989	187.96	158.46	221.54	RW2
Benin	ZOU	1990	182.22	153.77	214.50	RW2
Benin	ZOU	1991	176.97	151.35	206.07	RW2
Benin	ZOU	1992	171.97	147.81	199.12	RW2
Benin	ZOU	1993	167.27	143.04	194.69	RW2
Benin	ZOU	1994	162.92	137.17	192.51	RW2
Benin	ZOU	1995	159.01	133.82	187.88	RW2
Benin	ZOU	1996	155.37	132.23	181.57	RW2
Benin	ZOU	1997	151.94	130.28	176.23	RW2
Benin	ZOU	1998	148.66	126.63	174.15	RW2
Benin	ZOU	1999	145.47	121.74	173.01	RW2
Benin	ZOU	2000	142.31	118.37	169.60	RW2
Benin	ZOU	2001	139.01	117.35	163.91	RW2
Benin	ZOU	2002	135.56	115.01	159.17	RW2
Benin	ZOU	2003	131.79	110.51	156.66	RW2
Benin	ZOU	2004	127.95	104.61	155.58	RW2
Benin	ZOU	2005	123.95	98.38	154.86	RW2
Benin	ZOU	2006	119.93	95.11	150.15	RW2
Benin	ZOU	2007	115.91	91.63	145.59	RW2
Benin	ZOU	2008	112.07	85.50	145.68	RW2
Benin	ZOU	2009	108.14	75.71	152.47	RW2
Benin	ZOU	2010	104.69	62.27	169.96	RW2
Benin	ZOU	2011	100.80	50.77	190.53	RW2
Benin	ZOU	2012	97.54	40.05	217.37	RW2
Benin	ZOU	2013	93.94	30.62	252.25	RW2
Benin	ZOU	2014	90.72	23.03	289.72	RW2
Benin	ZOU	2015	87.66	17.27	345.17	RW2
Benin	ZOU	2016	84.52	12.70	401.53	RW2
Benin	ZOU	2017	81.71	9.01	469.82	RW2
Benin	ZOU	2018	78.35	6.11	536.34	RW2

Continued on next page

Country	Region	Year	Median	Lower	Upper	Method
Benin	ZOU	2019	75.43	4.37	603.78	RW2
Burkina Faso	ALL	1980	254.55	247.90	261.61	IHME
Burkina Faso	ALL	1980	243.08	183.37	313.56	RW2
Burkina Faso	ALL	1980	241.20	225.20	258.50	UN
Burkina Faso	ALL	1981	248.24	241.57	254.92	IHME
Burkina Faso	ALL	1981	237.43	195.11	284.41	RW2
Burkina Faso	ALL	1981	236.10	220.60	252.70	UN
Burkina Faso	ALL	1982	242.20	235.74	248.59	IHME
Burkina Faso	ALL	1982	231.88	193.90	274.87	RW2
Burkina Faso	ALL	1982	231.80	216.50	247.90	UN
Burkina Faso	ALL	1983	236.96	230.65	243.04	IHME
Burkina Faso	ALL	1983	226.29	184.82	274.51	RW2
Burkina Faso	ALL	1983	227.80	212.90	243.40	UN
Burkina Faso	ALL	1984	232.18	226.11	238.02	IHME
Burkina Faso	ALL	1984	221.45	176.60	274.27	RW2
Burkina Faso	ALL	1984	223.70	209.00	238.90	UN
Burkina Faso	ALL	1985	227.01	221.24	232.86	IHME
Burkina Faso	ALL	1985	216.27	175.78	263.05	RW2
Burkina Faso	ALL	1985	219.20	205.20	234.10	UN
Burkina Faso	ALL	1986	221.98	216.28	227.82	IHME
Burkina Faso	ALL	1986	212.36	175.69	254.28	RW2
Burkina Faso	ALL	1986	214.40	200.80	228.60	UN
Burkina Faso	ALL	1987	217.78	212.35	223.53	IHME
Burkina Faso	ALL	1987	209.28	174.96	249.08	RW2
Burkina Faso	ALL	1987	209.60	196.40	223.50	UN
Burkina Faso	ALL	1988	213.85	208.59	219.25	IHME
Burkina Faso	ALL	1988	206.82	170.74	247.96	RW2
Burkina Faso	ALL	1988	205.80	192.70	219.30	UN
Burkina Faso	ALL	1989	210.51	205.31	215.92	IHME
Burkina Faso	ALL	1989	205.04	166.96	248.77	RW2
Burkina Faso	ALL	1989	203.40	190.50	216.60	UN
Burkina Faso	ALL	1990	207.73	202.61	213.20	IHME
Burkina Faso	ALL	1990	204.10	166.81	248.00	RW2
Burkina Faso	ALL	1990	202.20	189.40	215.10	UN
Burkina Faso	ALL	1991	204.88	199.72	210.36	IHME
Burkina Faso	ALL	1991	203.00	168.02	242.62	RW2
Burkina Faso	ALL	1991	201.80	189.30	215.00	UN
Burkina Faso	ALL	1992	202.45	197.27	207.88	IHME
Burkina Faso	ALL	1992	201.88	167.97	240.03	RW2
Burkina Faso	ALL	1992	202.10	189.40	215.30	UN
Burkina Faso	ALL	1993	200.11	194.99	205.50	IHME
Burkina Faso	ALL	1993	200.66	166.05	240.38	RW2
Burkina Faso	ALL	1993	202.00	189.10	215.50	UN
Burkina Faso	ALL	1994	198.03	192.96	203.64	IHME
Burkina Faso	ALL	1994	199.17	162.53	242.67	RW2
Burkina Faso	ALL	1994	201.10	188.20	214.60	UN
Burkina Faso	ALL	1995	195.71	190.49	201.18	IHME
Burkina Faso	ALL	1995	197.38	161.15	239.54	RW2
Burkina Faso	ALL	1995	199.40	186.70	212.80	UN
Burkina Faso	ALL	1996	192.73	187.45	198.05	IHME
Burkina Faso	ALL	1996	195.64	161.80	235.57	RW2
Burkina Faso	ALL	1996	197.00	184.20	210.30	UN
Burkina Faso	ALL	1997	189.38	184.26	194.96	IHME
Burkina Faso	ALL	1997	193.86	161.43	231.27	RW2
Burkina Faso	ALL	1997	194.00	181.10	207.50	UN
Burkina Faso	ALL	1998	185.65	180.29	191.19	IHME
Burkina Faso	ALL	1998	191.99	158.47	231.32	RW2
Burkina Faso	ALL	1998	191.30	178.00	204.80	UN
Burkina Faso	ALL	1999	181.60	176.12	187.31	IHME
Burkina Faso	ALL	1999	189.67	153.88	230.05	RW2
Burkina Faso	ALL	1999	188.50	175.20	202.30	UN
Burkina Faso	ALL	2000	177.59	171.86	183.32	IHME
Burkina Faso	ALL	2000	187.18	152.28	228.43	RW2

Continued on next page

Country	Region	Year	Median	Lower	Upper	Method
Burkina Faso	ALL	2000	185.70	172.20	199.90	UN
Burkina Faso	ALL	2001	173.31	167.63	179.09	IHME
Burkina Faso	ALL	2001	183.24	150.46	221.36	RW2
Burkina Faso	ALL	2001	182.40	168.70	196.90	UN
Burkina Faso	ALL	2002	168.85	163.03	174.32	IHME
Burkina Faso	ALL	2002	178.10	147.41	213.57	RW2
Burkina Faso	ALL	2002	178.30	164.30	193.20	UN
Burkina Faso	ALL	2003	164.11	158.27	169.71	IHME
Burkina Faso	ALL	2003	171.90	141.34	207.60	RW2
Burkina Faso	ALL	2003	173.10	159.00	188.10	UN
Burkina Faso	ALL	2004	159.32	153.10	165.32	IHME
Burkina Faso	ALL	2004	164.44	131.92	203.14	RW2
Burkina Faso	ALL	2004	166.30	152.40	181.10	UN
Burkina Faso	ALL	2005	154.28	147.87	160.50	IHME
Burkina Faso	ALL	2005	156.10	121.87	196.28	RW2
Burkina Faso	ALL	2005	158.30	144.90	172.90	UN
Burkina Faso	ALL	2006	149.25	142.44	155.85	IHME
Burkina Faso	ALL	2006	147.82	117.37	183.33	RW2
Burkina Faso	ALL	2006	149.40	136.50	163.40	UN
Burkina Faso	ALL	2007	144.32	137.05	151.37	IHME
Burkina Faso	ALL	2007	139.63	113.88	169.77	RW2
Burkina Faso	ALL	2007	139.90	127.20	153.40	UN
Burkina Faso	ALL	2008	139.55	131.99	147.18	IHME
Burkina Faso	ALL	2008	131.79	105.98	163.49	RW2
Burkina Faso	ALL	2008	130.40	117.90	144.30	UN
Burkina Faso	ALL	2009	134.99	127.18	142.96	IHME
Burkina Faso	ALL	2009	124.07	90.29	169.30	RW2
Burkina Faso	ALL	2009	121.40	108.30	136.10	UN
Burkina Faso	ALL	2010	130.57	122.28	139.57	IHME
Burkina Faso	ALL	2010	116.91	70.45	190.46	RW2
Burkina Faso	ALL	2010	113.50	98.90	129.70	UN
Burkina Faso	ALL	2011	126.42	117.84	136.33	IHME
Burkina Faso	ALL	2011	110.19	53.65	213.17	RW2
Burkina Faso	ALL	2011	106.90	90.50	124.80	UN
Burkina Faso	ALL	2012	122.35	113.11	132.86	IHME
Burkina Faso	ALL	2012	103.61	39.43	244.32	RW2
Burkina Faso	ALL	2012	101.40	82.80	121.90	UN
Burkina Faso	ALL	2013	118.34	108.63	129.85	IHME
Burkina Faso	ALL	2013	97.75	28.60	281.67	RW2
Burkina Faso	ALL	2013	96.60	76.20	120.10	UN
Burkina Faso	ALL	2014	114.25	104.03	126.19	IHME
Burkina Faso	ALL	2014	91.83	20.17	327.98	RW2
Burkina Faso	ALL	2014	92.40	70.20	119.60	UN
Burkina Faso	ALL	2015	110.38	100.10	123.03	IHME
Burkina Faso	ALL	2015	86.01	14.14	378.47	RW2
Burkina Faso	ALL	2015	88.60	64.90	119.10	UN
Burkina Faso	ALL	2016	81.38	9.78	444.22	RW2
Burkina Faso	ALL	2017	76.01	6.43	512.31	RW2
Burkina Faso	ALL	2018	71.36	4.32	588.53	RW2
Burkina Faso	ALL	2019	66.78	2.58	650.17	RW2
Burkina Faso	ALL	15-19	76.03	6.49	506.97	RW2
Burkina Faso	CENTRAL/SOUTH	1980	223.95	174.76	282.54	RW2
Burkina Faso	CENTRAL/SOUTH	1981	220.23	186.39	257.77	RW2
Burkina Faso	CENTRAL/SOUTH	1982	216.55	186.18	249.65	RW2
Burkina Faso	CENTRAL/SOUTH	1983	212.84	179.64	251.09	RW2
Burkina Faso	CENTRAL/SOUTH	1984	209.63	174.43	250.34	RW2
Burkina Faso	CENTRAL/SOUTH	1985	206.48	173.57	242.58	RW2
Burkina Faso	CENTRAL/SOUTH	1986	204.13	175.41	236.58	RW2
Burkina Faso	CENTRAL/SOUTH	1987	202.65	175.80	232.54	RW2
Burkina Faso	CENTRAL/SOUTH	1988	201.79	173.39	233.36	RW2
Burkina Faso	CENTRAL/SOUTH	1989	201.39	170.60	236.14	RW2
Burkina Faso	CENTRAL/SOUTH	1990	201.71	171.33	236.52	RW2
Burkina Faso	CENTRAL/SOUTH	1991	201.87	173.67	232.75	RW2

Continued on next page

Country	Region	Year	Median	Lower	Upper	Method
Burkina Faso	CENTRAL/SOUTH	1992	201.57	174.72	231.10	RW2
Burkina Faso	CENTRAL/SOUTH	1993	201.03	173.50	232.43	RW2
Burkina Faso	CENTRAL/SOUTH	1994	200.21	170.09	234.71	RW2
Burkina Faso	CENTRAL/SOUTH	1995	198.60	168.36	233.01	RW2
Burkina Faso	CENTRAL/SOUTH	1996	197.16	169.54	227.67	RW2
Burkina Faso	CENTRAL/SOUTH	1997	195.59	169.47	225.29	RW2
Burkina Faso	CENTRAL/SOUTH	1998	193.96	166.67	224.62	RW2
Burkina Faso	CENTRAL/SOUTH	1999	191.77	162.15	224.64	RW2
Burkina Faso	CENTRAL/SOUTH	2000	189.41	160.27	223.39	RW2
Burkina Faso	CENTRAL/SOUTH	2001	185.73	158.77	216.44	RW2
Burkina Faso	CENTRAL/SOUTH	2002	180.80	155.67	209.25	RW2
Burkina Faso	CENTRAL/SOUTH	2003	174.66	148.67	204.11	RW2
Burkina Faso	CENTRAL/SOUTH	2004	167.55	139.34	199.66	RW2
Burkina Faso	CENTRAL/SOUTH	2005	159.30	129.56	193.23	RW2
Burkina Faso	CENTRAL/SOUTH	2006	151.09	124.81	181.68	RW2
Burkina Faso	CENTRAL/SOUTH	2007	143.05	120.18	169.64	RW2
Burkina Faso	CENTRAL/SOUTH	2008	135.17	110.97	163.93	RW2
Burkina Faso	CENTRAL/SOUTH	2009	127.46	95.48	168.12	RW2
Burkina Faso	CENTRAL/SOUTH	2010	120.44	76.45	184.79	RW2
Burkina Faso	CENTRAL/SOUTH	2011	113.58	60.47	203.66	RW2
Burkina Faso	CENTRAL/SOUTH	2012	107.05	46.22	228.11	RW2
Burkina Faso	CENTRAL/SOUTH	2013	100.88	34.64	258.25	RW2
Burkina Faso	CENTRAL/SOUTH	2014	94.70	24.85	293.64	RW2
Burkina Faso	CENTRAL/SOUTH	2015	89.51	18.05	339.45	RW2
Burkina Faso	CENTRAL/SOUTH	2016	84.01	12.56	388.70	RW2
Burkina Faso	CENTRAL/SOUTH	2017	79.39	8.80	447.50	RW2
Burkina Faso	CENTRAL/SOUTH	2018	74.23	6.06	506.46	RW2
Burkina Faso	CENTRAL/SOUTH	2019	70.23	4.16	571.98	RW2
Burkina Faso	EAST	1980	273.38	214.87	339.74	RW2
Burkina Faso	EAST	1981	265.54	225.26	309.19	RW2
Burkina Faso	EAST	1982	257.73	221.78	297.28	RW2
Burkina Faso	EAST	1983	249.88	211.45	293.65	RW2
Burkina Faso	EAST	1984	242.89	202.75	288.64	RW2
Burkina Faso	EAST	1985	235.99	199.39	277.06	RW2
Burkina Faso	EAST	1986	230.27	197.96	266.20	RW2
Burkina Faso	EAST	1987	225.56	195.14	259.21	RW2
Burkina Faso	EAST	1988	221.59	190.16	255.80	RW2
Burkina Faso	EAST	1989	218.46	185.32	255.13	RW2
Burkina Faso	EAST	1990	216.03	183.66	252.09	RW2
Burkina Faso	EAST	1991	213.46	184.30	246.03	RW2
Burkina Faso	EAST	1992	210.92	183.17	241.47	RW2
Burkina Faso	EAST	1993	208.01	178.93	240.48	RW2
Burkina Faso	EAST	1994	204.95	174.03	240.03	RW2
Burkina Faso	EAST	1995	201.23	171.05	235.34	RW2
Burkina Faso	EAST	1996	197.87	170.02	228.73	RW2
Burkina Faso	EAST	1997	194.39	168.28	223.71	RW2
Burkina Faso	EAST	1998	190.86	163.67	221.26	RW2
Burkina Faso	EAST	1999	186.80	157.09	219.81	RW2
Burkina Faso	EAST	2000	182.90	153.85	215.86	RW2
Burkina Faso	EAST	2001	177.63	151.11	207.60	RW2
Burkina Faso	EAST	2002	171.50	146.85	199.46	RW2
Burkina Faso	EAST	2003	164.25	138.65	192.99	RW2
Burkina Faso	EAST	2004	156.29	129.05	188.32	RW2
Burkina Faso	EAST	2005	147.33	119.06	180.30	RW2
Burkina Faso	EAST	2006	138.70	113.05	168.32	RW2
Burkina Faso	EAST	2007	130.21	107.94	155.98	RW2
Burkina Faso	EAST	2008	121.91	99.03	149.85	RW2
Burkina Faso	EAST	2009	114.16	84.51	152.45	RW2
Burkina Faso	EAST	2010	106.86	66.73	166.59	RW2
Burkina Faso	EAST	2011	100.01	52.52	182.81	RW2
Burkina Faso	EAST	2012	93.51	39.87	202.38	RW2
Burkina Faso	EAST	2013	87.32	29.50	229.34	RW2
Burkina Faso	EAST	2014	81.50	21.35	262.14	RW2

Continued on next page

Country	Region	Year	Median	Lower	Upper	Method
Burkina Faso	EAST	2015	76.28	15.07	302.38	RW2
Burkina Faso	EAST	2016	71.11	10.55	347.90	RW2
Burkina Faso	EAST	2017	66.14	7.38	397.13	RW2
Burkina Faso	EAST	2018	61.78	5.06	457.30	RW2
Burkina Faso	EAST	2019	57.65	3.32	520.44	RW2
Burkina Faso	NORTH	1980	239.53	188.86	299.55	RW2
Burkina Faso	NORTH	1981	231.85	197.33	270.23	RW2
Burkina Faso	NORTH	1982	224.37	193.82	258.64	RW2
Burkina Faso	NORTH	1983	217.17	183.59	255.75	RW2
Burkina Faso	NORTH	1984	210.56	175.11	251.87	RW2
Burkina Faso	NORTH	1985	204.03	171.90	240.16	RW2
Burkina Faso	NORTH	1986	198.90	170.98	229.76	RW2
Burkina Faso	NORTH	1987	194.46	168.39	223.76	RW2
Burkina Faso	NORTH	1988	191.12	163.61	221.22	RW2
Burkina Faso	NORTH	1989	188.49	159.47	221.05	RW2
Burkina Faso	NORTH	1990	186.74	158.60	218.99	RW2
Burkina Faso	NORTH	1991	184.88	159.15	214.25	RW2
Burkina Faso	NORTH	1992	183.00	158.43	210.30	RW2
Burkina Faso	NORTH	1993	180.99	155.89	209.40	RW2
Burkina Faso	NORTH	1994	178.80	151.60	210.27	RW2
Burkina Faso	NORTH	1995	176.00	149.10	206.40	RW2
Burkina Faso	NORTH	1996	173.48	149.04	201.29	RW2
Burkina Faso	NORTH	1997	170.75	147.81	196.64	RW2
Burkina Faso	NORTH	1998	167.87	143.90	194.69	RW2
Burkina Faso	NORTH	1999	164.65	138.87	194.10	RW2
Burkina Faso	NORTH	2000	161.10	135.53	190.76	RW2
Burkina Faso	NORTH	2001	156.58	133.88	183.08	RW2
Burkina Faso	NORTH	2002	151.13	129.61	175.35	RW2
Burkina Faso	NORTH	2003	144.66	122.57	169.42	RW2
Burkina Faso	NORTH	2004	137.51	114.06	164.82	RW2
Burkina Faso	NORTH	2005	129.57	105.02	157.69	RW2
Burkina Faso	NORTH	2006	121.86	100.08	146.75	RW2
Burkina Faso	NORTH	2007	114.26	95.89	136.10	RW2
Burkina Faso	NORTH	2008	107.00	87.78	129.77	RW2
Burkina Faso	NORTH	2009	100.04	74.81	132.81	RW2
Burkina Faso	NORTH	2010	93.75	59.12	145.29	RW2
Burkina Faso	NORTH	2011	87.58	46.07	160.08	RW2
Burkina Faso	NORTH	2012	81.94	35.36	179.13	RW2
Burkina Faso	NORTH	2013	76.57	25.75	201.48	RW2
Burkina Faso	NORTH	2014	71.37	18.50	234.57	RW2
Burkina Faso	NORTH	2015	66.79	13.27	272.02	RW2
Burkina Faso	NORTH	2016	62.26	9.33	311.93	RW2
Burkina Faso	NORTH	2017	57.94	6.43	361.98	RW2
Burkina Faso	NORTH	2018	54.10	4.33	421.85	RW2
Burkina Faso	NORTH	2019	50.23	2.91	486.62	RW2
Burkina Faso	WEST	1980	238.16	185.68	299.68	RW2
Burkina Faso	WEST	1981	235.94	198.73	277.44	RW2
Burkina Faso	WEST	1982	233.42	200.09	270.26	RW2
Burkina Faso	WEST	1983	231.10	194.75	272.36	RW2
Burkina Faso	WEST	1984	229.01	190.02	273.52	RW2
Burkina Faso	WEST	1985	227.14	191.75	266.82	RW2
Burkina Faso	WEST	1986	226.20	194.61	260.79	RW2
Burkina Faso	WEST	1987	225.96	196.29	258.77	RW2
Burkina Faso	WEST	1988	226.50	194.97	261.27	RW2
Burkina Faso	WEST	1989	227.64	193.68	265.11	RW2
Burkina Faso	WEST	1990	229.52	195.96	267.30	RW2
Burkina Faso	WEST	1991	231.30	200.30	265.75	RW2
Burkina Faso	WEST	1992	232.79	202.57	265.76	RW2
Burkina Faso	WEST	1993	233.90	202.44	268.57	RW2
Burkina Faso	WEST	1994	234.93	200.54	272.78	RW2
Burkina Faso	WEST	1995	235.17	200.76	273.18	RW2
Burkina Faso	WEST	1996	235.70	203.79	270.65	RW2
Burkina Faso	WEST	1997	236.02	205.69	269.71	RW2

Continued on next page

Country	Region	Year	Median	Lower	Upper	Method
Burkina Faso	WEST	1998	236.16	204.87	270.88	RW2
Burkina Faso	WEST	1999	235.89	200.62	273.54	RW2
Burkina Faso	WEST	2000	235.24	200.14	274.33	RW2
Burkina Faso	WEST	2001	233.11	200.47	268.89	RW2
Burkina Faso	WEST	2002	229.49	198.84	263.17	RW2
Burkina Faso	WEST	2003	224.32	192.73	259.89	RW2
Burkina Faso	WEST	2004	217.94	183.26	257.25	RW2
Burkina Faso	WEST	2005	210.02	173.20	251.50	RW2
Burkina Faso	WEST	2006	202.13	168.94	239.65	RW2
Burkina Faso	WEST	2007	193.90	164.25	226.84	RW2
Burkina Faso	WEST	2008	185.84	154.32	222.20	RW2
Burkina Faso	WEST	2009	177.76	135.31	230.05	RW2
Burkina Faso	WEST	2010	170.37	111.15	253.43	RW2
Burkina Faso	WEST	2011	162.81	89.14	279.57	RW2
Burkina Faso	WEST	2012	155.70	69.78	311.81	RW2
Burkina Faso	WEST	2013	149.06	53.35	355.03	RW2
Burkina Faso	WEST	2014	142.70	39.63	398.29	RW2
Burkina Faso	WEST	2015	136.37	29.01	449.87	RW2
Burkina Faso	WEST	2016	129.81	20.77	505.33	RW2
Burkina Faso	WEST	2017	124.39	14.78	568.17	RW2
Burkina Faso	WEST	2018	118.10	10.15	632.76	RW2
Burkina Faso	WEST	2019	112.88	6.92	699.12	RW2
Burundi	ALL	1980	221.51	207.20	236.97	IHME
Burundi	ALL	1980	219.20	141.59	327.50	RW2
Burundi	ALL	1980	221.80	200.80	244.60	UN
Burundi	ALL	1981	212.72	198.89	227.79	IHME
Burundi	ALL	1981	210.29	150.29	287.51	RW2
Burundi	ALL	1981	212.30	192.20	233.70	UN
Burundi	ALL	1982	203.03	189.47	217.37	IHME
Burundi	ALL	1982	201.67	152.14	263.92	RW2
Burundi	ALL	1982	202.30	182.60	222.70	UN
Burundi	ALL	1983	193.26	181.47	206.81	IHME
Burundi	ALL	1983	193.10	147.01	251.83	RW2
Burundi	ALL	1983	192.60	173.30	213.00	UN
Burundi	ALL	1984	184.52	173.35	197.30	IHME
Burundi	ALL	1984	186.03	140.00	244.46	RW2
Burundi	ALL	1984	184.20	164.80	204.60	UN
Burundi	ALL	1985	178.26	167.38	190.07	IHME
Burundi	ALL	1985	178.57	136.08	228.69	RW2
Burundi	ALL	1985	177.50	158.00	198.10	UN
Burundi	ALL	1986	174.55	163.44	186.04	IHME
Burundi	ALL	1986	173.94	134.71	219.59	RW2
Burundi	ALL	1986	172.90	154.00	193.40	UN
Burundi	ALL	1987	172.97	161.55	184.31	IHME
Burundi	ALL	1987	171.13	134.15	214.41	RW2
Burundi	ALL	1987	170.60	152.60	190.20	UN
Burundi	ALL	1988	172.75	161.35	184.64	IHME
Burundi	ALL	1988	169.86	131.93	213.15	RW2
Burundi	ALL	1988	170.10	153.00	188.70	UN
Burundi	ALL	1989	173.03	161.52	185.51	IHME
Burundi	ALL	1989	169.98	131.01	214.29	RW2
Burundi	ALL	1989	170.70	154.00	189.10	UN
Burundi	ALL	1990	173.41	161.55	184.97	IHME
Burundi	ALL	1990	172.03	135.41	218.01	RW2
Burundi	ALL	1990	171.80	155.40	190.30	UN
Burundi	ALL	1991	174.16	162.26	184.60	IHME
Burundi	ALL	1991	173.16	138.21	215.40	RW2
Burundi	ALL	1991	172.90	156.20	192.00	UN
Burundi	ALL	1992	174.70	163.07	185.33	IHME
Burundi	ALL	1992	173.89	139.47	214.90	RW2
Burundi	ALL	1992	173.50	156.60	193.60	UN
Burundi	ALL	1993	175.20	163.59	187.01	IHME
Burundi	ALL	1993	173.86	138.80	216.27	RW2

Continued on next page

Country	Region	Year	Median	Lower	Upper	Method
Burundi	ALL	1993	173.50	155.90	194.20	UN
Burundi	ALL	1994	174.88	164.09	186.85	IHME
Burundi	ALL	1994	172.77	136.37	218.34	RW2
Burundi	ALL	1994	172.50	154.20	193.70	UN
Burundi	ALL	1995	174.67	163.04	186.67	IHME
Burundi	ALL	1995	170.50	134.63	213.05	RW2
Burundi	ALL	1995	170.50	152.40	192.20	UN
Burundi	ALL	1996	174.29	163.47	186.27	IHME
Burundi	ALL	1996	167.91	134.36	208.14	RW2
Burundi	ALL	1996	167.70	149.60	189.40	UN
Burundi	ALL	1997	172.49	161.65	184.79	IHME
Burundi	ALL	1997	164.86	132.96	202.63	RW2
Burundi	ALL	1997	164.40	146.30	185.70	UN
Burundi	ALL	1998	170.90	159.54	183.32	IHME
Burundi	ALL	1998	161.54	129.20	200.99	RW2
Burundi	ALL	1998	160.50	142.80	181.40	UN
Burundi	ALL	1999	168.72	156.97	181.43	IHME
Burundi	ALL	1999	157.80	124.16	197.64	RW2
Burundi	ALL	1999	156.40	139.00	176.70	UN
Burundi	ALL	2000	165.89	153.86	178.38	IHME
Burundi	ALL	2000	153.84	121.35	193.01	RW2
Burundi	ALL	2000	152.20	134.90	172.00	UN
Burundi	ALL	2001	159.91	148.29	172.13	IHME
Burundi	ALL	2001	149.23	119.05	185.39	RW2
Burundi	ALL	2001	147.80	130.70	167.20	UN
Burundi	ALL	2002	151.93	140.61	163.13	IHME
Burundi	ALL	2002	144.10	116.01	177.75	RW2
Burundi	ALL	2002	143.20	126.30	162.10	UN
Burundi	ALL	2003	141.58	130.71	151.96	IHME
Burundi	ALL	2003	138.61	110.81	172.28	RW2
Burundi	ALL	2003	138.30	121.30	157.30	UN
Burundi	ALL	2004	129.91	119.94	139.74	IHME
Burundi	ALL	2004	132.53	103.11	168.71	RW2
Burundi	ALL	2004	133.00	115.60	152.30	UN
Burundi	ALL	2005	118.19	109.03	127.49	IHME
Burundi	ALL	2005	126.31	95.79	164.23	RW2
Burundi	ALL	2005	127.10	109.10	147.10	UN
Burundi	ALL	2006	107.22	98.85	115.76	IHME
Burundi	ALL	2006	120.10	92.59	153.78	RW2
Burundi	ALL	2006	120.90	102.00	142.10	UN
Burundi	ALL	2007	97.85	89.49	105.94	IHME
Burundi	ALL	2007	114.07	90.16	143.12	RW2
Burundi	ALL	2007	114.50	94.20	138.40	UN
Burundi	ALL	2008	91.37	83.35	99.77	IHME
Burundi	ALL	2008	108.37	83.84	139.26	RW2
Burundi	ALL	2008	108.60	86.80	135.80	UN
Burundi	ALL	2009	87.46	78.74	96.72	IHME
Burundi	ALL	2009	102.64	71.16	146.48	RW2
Burundi	ALL	2009	103.50	79.30	133.70	UN
Burundi	ALL	2010	85.12	74.92	95.74	IHME
Burundi	ALL	2010	97.29	55.00	168.19	RW2
Burundi	ALL	2010	98.80	72.80	132.30	UN
Burundi	ALL	2011	83.92	71.22	97.84	IHME
Burundi	ALL	2011	92.31	41.49	192.43	RW2
Burundi	ALL	2011	94.90	66.90	131.50	UN
Burundi	ALL	2012	81.92	67.16	99.21	IHME
Burundi	ALL	2012	87.37	30.15	225.85	RW2
Burundi	ALL	2012	91.20	61.90	130.50	UN
Burundi	ALL	2013	80.12	63.79	101.44	IHME
Burundi	ALL	2013	83.02	21.63	266.56	RW2
Burundi	ALL	2013	87.80	57.40	130.50	UN
Burundi	ALL	2014	78.40	60.28	103.46	IHME
Burundi	ALL	2014	78.53	15.06	317.64	RW2

Continued on next page

Country	Region	Year	Median	Lower	Upper	Method
Burundi	ALL	2014	84.60	53.20	130.70	UN
Burundi	ALL	2015	76.81	57.35	104.34	IHME
Burundi	ALL	2015	74.05	10.43	374.13	RW2
Burundi	ALL	2015	81.70	49.70	130.30	UN
Burundi	ALL	2016	70.62	7.12	447.68	RW2
Burundi	ALL	2017	66.41	4.61	523.94	RW2
Burundi	ALL	2018	62.81	3.05	608.17	RW2
Burundi	ALL	2019	59.22	1.78	675.47	RW2
Burundi	ALL	15-19	66.42	4.65	518.18	RW2
Burundi	BUJUMBURA	1980	215.37	113.59	383.89	RW2
Burundi	BUJUMBURA	1981	200.31	116.02	332.05	RW2
Burundi	BUJUMBURA	1982	185.87	113.99	294.17	RW2
Burundi	BUJUMBURA	1983	171.69	109.42	263.95	RW2
Burundi	BUJUMBURA	1984	159.72	103.90	239.49	RW2
Burundi	BUJUMBURA	1985	148.12	98.36	217.71	RW2
Burundi	BUJUMBURA	1986	139.06	94.19	200.62	RW2
Burundi	BUJUMBURA	1987	131.99	90.46	187.80	RW2
Burundi	BUJUMBURA	1988	126.34	87.30	177.35	RW2
Burundi	BUJUMBURA	1989	122.25	85.08	170.32	RW2
Burundi	BUJUMBURA	1990	119.56	84.37	164.86	RW2
Burundi	BUJUMBURA	1991	116.91	84.04	158.82	RW2
Burundi	BUJUMBURA	1992	114.62	83.14	153.94	RW2
Burundi	BUJUMBURA	1993	112.07	81.33	150.78	RW2
Burundi	BUJUMBURA	1994	109.43	79.48	147.47	RW2
Burundi	BUJUMBURA	1995	106.08	77.90	141.83	RW2
Burundi	BUJUMBURA	1996	102.76	76.25	135.73	RW2
Burundi	BUJUMBURA	1997	99.02	74.18	130.17	RW2
Burundi	BUJUMBURA	1998	95.04	71.01	125.27	RW2
Burundi	BUJUMBURA	1999	90.60	67.00	120.52	RW2
Burundi	BUJUMBURA	2000	86.38	64.09	114.20	RW2
Burundi	BUJUMBURA	2001	81.98	61.55	107.49	RW2
Burundi	BUJUMBURA	2002	77.78	58.78	101.66	RW2
Burundi	BUJUMBURA	2003	73.57	54.94	97.13	RW2
Burundi	BUJUMBURA	2004	69.64	51.00	94.31	RW2
Burundi	BUJUMBURA	2005	65.74	47.16	91.00	RW2
Burundi	BUJUMBURA	2006	62.19	44.15	86.89	RW2
Burundi	BUJUMBURA	2007	58.82	41.13	83.89	RW2
Burundi	BUJUMBURA	2008	55.51	36.92	84.53	RW2
Burundi	BUJUMBURA	2009	52.48	31.25	89.19	RW2
Burundi	BUJUMBURA	2010	49.60	24.54	100.99	RW2
Burundi	BUJUMBURA	2011	46.95	19.13	116.13	RW2
Burundi	BUJUMBURA	2012	44.41	14.32	135.01	RW2
Burundi	BUJUMBURA	2013	41.98	10.42	161.79	RW2
Burundi	BUJUMBURA	2014	39.67	7.40	195.98	RW2
Burundi	BUJUMBURA	2015	37.66	5.12	239.95	RW2
Burundi	BUJUMBURA	2016	35.57	3.51	292.37	RW2
Burundi	BUJUMBURA	2017	33.52	2.40	351.83	RW2
Burundi	BUJUMBURA	2018	31.77	1.61	426.12	RW2
Burundi	BUJUMBURA	2019	30.08	1.02	505.83	RW2
Burundi	CENTRE-EAST	1980	227.34	136.15	373.80	RW2
Burundi	CENTRE-EAST	1981	214.78	140.90	327.13	RW2
Burundi	CENTRE-EAST	1982	203.06	140.83	292.33	RW2
Burundi	CENTRE-EAST	1983	191.68	137.25	268.44	RW2
Burundi	CENTRE-EAST	1984	181.80	132.52	247.93	RW2
Burundi	CENTRE-EAST	1985	172.33	128.00	227.35	RW2
Burundi	CENTRE-EAST	1986	165.12	126.34	213.29	RW2
Burundi	CENTRE-EAST	1987	160.05	124.85	202.38	RW2
Burundi	CENTRE-EAST	1988	156.68	122.84	196.57	RW2
Burundi	CENTRE-EAST	1989	154.71	121.37	193.96	RW2
Burundi	CENTRE-EAST	1990	154.55	123.20	192.56	RW2
Burundi	CENTRE-EAST	1991	154.45	125.02	188.64	RW2
Burundi	CENTRE-EAST	1992	154.14	125.96	186.89	RW2
Burundi	CENTRE-EAST	1993	153.72	125.56	187.32	RW2

Continued on next page

Country	Region	Year	Median	Lower	Upper	Method
Burundi	CENTRE-EAST	1994	153.03	123.67	188.17	RW2
Burundi	CENTRE-EAST	1995	151.51	122.79	185.41	RW2
Burundi	CENTRE-EAST	1996	150.02	123.56	180.20	RW2
Burundi	CENTRE-EAST	1997	148.30	123.29	177.62	RW2
Burundi	CENTRE-EAST	1998	146.49	121.02	176.37	RW2
Burundi	CENTRE-EAST	1999	144.21	117.51	175.56	RW2
Burundi	CENTRE-EAST	2000	141.63	115.52	172.96	RW2
Burundi	CENTRE-EAST	2001	138.52	114.33	167.23	RW2
Burundi	CENTRE-EAST	2002	134.70	112.04	161.73	RW2
Burundi	CENTRE-EAST	2003	130.26	106.92	158.11	RW2
Burundi	CENTRE-EAST	2004	125.34	100.27	155.24	RW2
Burundi	CENTRE-EAST	2005	119.92	93.88	151.67	RW2
Burundi	CENTRE-EAST	2006	114.40	90.66	143.75	RW2
Burundi	CENTRE-EAST	2007	109.06	87.07	136.10	RW2
Burundi	CENTRE-EAST	2008	103.83	79.85	133.95	RW2
Burundi	CENTRE-EAST	2009	98.62	68.43	139.73	RW2
Burundi	CENTRE-EAST	2010	93.87	54.34	156.73	RW2
Burundi	CENTRE-EAST	2011	89.19	42.55	177.16	RW2
Burundi	CENTRE-EAST	2012	84.72	32.11	203.91	RW2
Burundi	CENTRE-EAST	2013	80.48	23.73	237.42	RW2
Burundi	CENTRE-EAST	2014	76.12	16.72	277.51	RW2
Burundi	CENTRE-EAST	2015	72.59	11.96	329.84	RW2
Burundi	CENTRE-EAST	2016	68.66	8.16	387.03	RW2
Burundi	CENTRE-EAST	2017	65.48	5.62	455.36	RW2
Burundi	CENTRE-EAST	2018	61.71	3.79	523.98	RW2
Burundi	CENTRE-EAST	2019	58.94	2.55	598.93	RW2
Burundi	NORTH	1980	193.02	112.74	308.35	RW2
Burundi	NORTH	1981	188.91	121.40	278.60	RW2
Burundi	NORTH	1982	184.24	126.08	258.09	RW2
Burundi	NORTH	1983	180.01	127.32	246.66	RW2
Burundi	NORTH	1984	176.40	126.74	238.80	RW2
Burundi	NORTH	1985	173.26	127.80	228.45	RW2
Burundi	NORTH	1986	172.23	130.18	221.90	RW2
Burundi	NORTH	1987	172.76	133.28	219.34	RW2
Burundi	NORTH	1988	174.99	136.09	220.37	RW2
Burundi	NORTH	1989	178.54	139.85	223.31	RW2
Burundi	NORTH	1990	183.63	146.81	227.79	RW2
Burundi	NORTH	1991	188.25	153.48	229.55	RW2
Burundi	NORTH	1992	192.10	157.83	232.27	RW2
Burundi	NORTH	1993	194.77	159.78	236.11	RW2
Burundi	NORTH	1994	196.44	159.62	239.65	RW2
Burundi	NORTH	1995	196.24	159.99	238.60	RW2
Burundi	NORTH	1996	195.31	161.28	234.48	RW2
Burundi	NORTH	1997	193.28	160.97	230.81	RW2
Burundi	NORTH	1998	190.47	158.23	227.97	RW2
Burundi	NORTH	1999	186.97	152.64	225.72	RW2
Burundi	NORTH	2000	182.79	149.48	220.74	RW2
Burundi	NORTH	2001	178.20	147.71	212.58	RW2
Burundi	NORTH	2002	173.10	144.79	205.28	RW2
Burundi	NORTH	2003	167.57	138.92	201.05	RW2
Burundi	NORTH	2004	161.91	131.12	198.24	RW2
Burundi	NORTH	2005	155.94	124.03	194.28	RW2
Burundi	NORTH	2006	150.14	120.87	185.23	RW2
Burundi	NORTH	2007	144.27	116.93	176.35	RW2
Burundi	NORTH	2008	138.62	108.63	175.11	RW2
Burundi	NORTH	2009	132.84	94.03	184.34	RW2
Burundi	NORTH	2010	127.55	75.88	207.45	RW2
Burundi	NORTH	2011	122.08	59.60	234.75	RW2
Burundi	NORTH	2012	116.96	45.61	268.99	RW2
Burundi	NORTH	2013	112.21	34.04	315.55	RW2
Burundi	NORTH	2014	107.65	24.64	363.62	RW2
Burundi	NORTH	2015	103.08	17.56	421.77	RW2
Burundi	NORTH	2016	98.25	12.23	485.22	RW2

Continued on next page

Country	Region	Year	Median	Lower	Upper	Method
Burundi	NORTH	2017	94.40	8.46	557.43	RW2
Burundi	NORTH	2018	89.74	5.63	631.56	RW2
Burundi	NORTH	2019	86.00	3.72	706.68	RW2
Burundi	SOUTH	1980	242.02	151.66	367.27	RW2
Burundi	SOUTH	1981	230.14	158.04	325.49	RW2
Burundi	SOUTH	1982	218.82	158.21	296.39	RW2
Burundi	SOUTH	1983	207.65	153.22	277.24	RW2
Burundi	SOUTH	1984	197.52	146.74	263.07	RW2
Burundi	SOUTH	1985	187.64	141.14	244.81	RW2
Burundi	SOUTH	1986	179.61	137.82	231.27	RW2
Burundi	SOUTH	1987	172.86	133.90	220.84	RW2
Burundi	SOUTH	1988	167.45	130.03	213.19	RW2
Burundi	SOUTH	1989	162.96	126.20	207.38	RW2
Burundi	SOUTH	1990	159.84	125.25	202.29	RW2
Burundi	SOUTH	1991	156.29	124.03	195.63	RW2
Burundi	SOUTH	1992	152.61	122.33	189.68	RW2
Burundi	SOUTH	1993	148.94	119.22	185.14	RW2
Burundi	SOUTH	1994	144.75	114.64	180.43	RW2
Burundi	SOUTH	1995	140.48	112.28	173.66	RW2
Burundi	SOUTH	1996	136.31	110.81	166.36	RW2
Burundi	SOUTH	1997	132.01	108.54	159.45	RW2
Burundi	SOUTH	1998	128.02	104.53	155.37	RW2
Burundi	SOUTH	1999	123.83	99.87	151.72	RW2
Burundi	SOUTH	2000	119.72	96.98	146.58	RW2
Burundi	SOUTH	2001	115.43	94.96	139.79	RW2
Burundi	SOUTH	2002	111.15	92.20	133.47	RW2
Burundi	SOUTH	2003	106.51	87.04	129.39	RW2
Burundi	SOUTH	2004	101.92	81.14	127.30	RW2
Burundi	SOUTH	2005	97.18	75.48	124.32	RW2
Burundi	SOUTH	2006	92.46	71.98	118.16	RW2
Burundi	SOUTH	2007	87.86	68.39	112.46	RW2
Burundi	SOUTH	2008	83.59	62.47	111.71	RW2
Burundi	SOUTH	2009	79.39	53.30	117.84	RW2
Burundi	SOUTH	2010	75.52	42.68	132.36	RW2
Burundi	SOUTH	2011	71.45	32.85	150.25	RW2
Burundi	SOUTH	2012	67.82	24.85	173.40	RW2
Burundi	SOUTH	2013	64.43	18.30	204.80	RW2
Burundi	SOUTH	2014	61.29	13.12	242.96	RW2
Burundi	SOUTH	2015	58.06	9.35	292.27	RW2
Burundi	SOUTH	2016	55.16	6.31	344.01	RW2
Burundi	SOUTH	2017	52.22	4.31	409.81	RW2
Burundi	SOUTH	2018	49.54	2.94	486.64	RW2
Burundi	SOUTH	2019	47.12	1.84	559.00	RW2
Burundi	WEST	1980	202.19	114.68	321.66	RW2
Burundi	WEST	1981	198.59	125.18	293.35	RW2
Burundi	WEST	1982	194.99	130.52	274.59	RW2
Burundi	WEST	1983	191.46	132.90	264.16	RW2
Burundi	WEST	1984	188.61	134.71	255.85	RW2
Burundi	WEST	1985	185.96	136.83	245.73	RW2
Burundi	WEST	1986	185.30	140.11	239.09	RW2
Burundi	WEST	1987	185.95	143.85	235.58	RW2
Burundi	WEST	1988	187.89	146.77	236.22	RW2
Burundi	WEST	1989	191.12	150.08	239.17	RW2
Burundi	WEST	1990	195.27	156.03	243.10	RW2
Burundi	WEST	1991	198.89	160.79	243.73	RW2
Burundi	WEST	1992	201.54	164.38	245.57	RW2
Burundi	WEST	1993	202.81	165.11	248.28	RW2
Burundi	WEST	1994	202.84	163.95	250.17	RW2
Burundi	WEST	1995	201.18	163.35	246.20	RW2
Burundi	WEST	1996	198.23	162.72	239.88	RW2
Burundi	WEST	1997	194.48	161.35	233.80	RW2
Burundi	WEST	1998	189.66	156.55	229.16	RW2
Burundi	WEST	1999	184.07	149.78	224.08	RW2

Continued on next page

Country	Region	Year	Median	Lower	Upper	Method
Burundi	WEST	2000	177.60	144.45	216.55	RW2
Burundi	WEST	2001	170.37	140.57	205.44	RW2
Burundi	WEST	2002	162.59	134.73	194.65	RW2
Burundi	WEST	2003	154.17	126.06	187.42	RW2
Burundi	WEST	2004	145.61	115.99	181.83	RW2
Burundi	WEST	2005	136.95	106.04	174.89	RW2
Burundi	WEST	2006	128.36	99.03	164.14	RW2
Burundi	WEST	2007	120.07	91.76	154.29	RW2
Burundi	WEST	2008	112.15	81.62	149.74	RW2
Burundi	WEST	2009	104.77	68.93	153.12	RW2
Burundi	WEST	2010	97.71	54.20	166.87	RW2
Burundi	WEST	2011	91.00	41.47	184.62	RW2
Burundi	WEST	2012	84.97	30.75	208.57	RW2
Burundi	WEST	2013	79.13	22.86	238.69	RW2
Burundi	WEST	2014	73.47	16.05	274.28	RW2
Burundi	WEST	2015	68.34	11.04	322.47	RW2
Burundi	WEST	2016	63.40	7.33	372.15	RW2
Burundi	WEST	2017	59.12	4.97	432.22	RW2
Burundi	WEST	2018	54.52	3.24	494.37	RW2
Burundi	WEST	2019	51.01	2.08	573.57	RW2
Cameroon	ADAM/NORD/EXT-NORD	1980	266.62	206.52	336.03	RW2
Cameroon	ADAM/NORD/EXT-NORD	1981	254.14	211.49	301.11	RW2
Cameroon	ADAM/NORD/EXT-NORD	1982	241.91	205.53	282.58	RW2
Cameroon	ADAM/NORD/EXT-NORD	1983	230.06	193.73	272.37	RW2
Cameroon	ADAM/NORD/EXT-NORD	1984	219.68	183.01	262.55	RW2
Cameroon	ADAM/NORD/EXT-NORD	1985	209.77	176.22	247.32	RW2
Cameroon	ADAM/NORD/EXT-NORD	1986	202.36	172.75	235.36	RW2
Cameroon	ADAM/NORD/EXT-NORD	1987	196.98	169.33	227.87	RW2
Cameroon	ADAM/NORD/EXT-NORD	1988	193.47	165.39	224.40	RW2
Cameroon	ADAM/NORD/EXT-NORD	1989	191.88	162.56	224.58	RW2
Cameroon	ADAM/NORD/EXT-NORD	1990	192.12	163.55	224.69	RW2
Cameroon	ADAM/NORD/EXT-NORD	1991	193.18	166.88	223.14	RW2
Cameroon	ADAM/NORD/EXT-NORD	1992	195.12	169.41	223.69	RW2
Cameroon	ADAM/NORD/EXT-NORD	1993	197.35	169.80	227.82	RW2
Cameroon	ADAM/NORD/EXT-NORD	1994	199.72	169.85	232.78	RW2
Cameroon	ADAM/NORD/EXT-NORD	1995	201.78	172.47	235.29	RW2
Cameroon	ADAM/NORD/EXT-NORD	1996	203.17	175.21	234.16	RW2
Cameroon	ADAM/NORD/EXT-NORD	1997	203.53	176.61	233.43	RW2
Cameroon	ADAM/NORD/EXT-NORD	1998	202.76	174.71	233.82	RW2
Cameroon	ADAM/NORD/EXT-NORD	1999	200.43	170.19	233.87	RW2
Cameroon	ADAM/NORD/EXT-NORD	2000	197.11	167.61	229.30	RW2
Cameroon	ADAM/NORD/EXT-NORD	2001	192.29	165.48	221.64	RW2
Cameroon	ADAM/NORD/EXT-NORD	2002	186.44	161.64	214.13	RW2
Cameroon	ADAM/NORD/EXT-NORD	2003	179.59	154.32	207.83	RW2
Cameroon	ADAM/NORD/EXT-NORD	2004	172.38	146.24	202.99	RW2
Cameroon	ADAM/NORD/EXT-NORD	2005	164.53	138.55	193.60	RW2
Cameroon	ADAM/NORD/EXT-NORD	2006	157.79	134.14	184.04	RW2
Cameroon	ADAM/NORD/EXT-NORD	2007	151.83	130.13	176.12	RW2
Cameroon	ADAM/NORD/EXT-NORD	2008	146.69	124.67	172.14	RW2
Cameroon	ADAM/NORD/EXT-NORD	2009	142.54	118.44	170.26	RW2
Cameroon	ADAM/NORD/EXT-NORD	2010	139.42	113.60	170.54	RW2
Cameroon	ADAM/NORD/EXT-NORD	2011	136.49	112.09	166.15	RW2
Cameroon	ADAM/NORD/EXT-NORD	2012	133.84	110.23	161.94	RW2
Cameroon	ADAM/NORD/EXT-NORD	2013	131.32	104.68	163.74	RW2
Cameroon	ADAM/NORD/EXT-NORD	2014	128.82	94.14	174.26	RW2
Cameroon	ADAM/NORD/EXT-NORD	2015	126.36	79.16	195.95	RW2
Cameroon	ADAM/NORD/EXT-NORD	2016	123.87	65.90	221.71	RW2
Cameroon	ADAM/NORD/EXT-NORD	2017	121.31	53.72	253.70	RW2
Cameroon	ADAM/NORD/EXT-NORD	2018	119.00	42.80	294.50	RW2
Cameroon	ADAM/NORD/EXT-NORD	2019	116.71	33.02	342.47	RW2
Cameroon	ALL	1980	167.34	161.53	173.39	IHME
Cameroon	ALL	1980	185.05	137.86	243.34	RW2
Cameroon	ALL	1980	181.60	167.20	197.50	UN

Continued on next page

Country	Region	Year	Median	Lower	Upper	Method
Cameroon	ALL	1981	163.07	157.41	169.09	IHME
Cameroon	ALL	1981	176.89	143.26	215.61	RW2
Cameroon	ALL	1981	176.30	162.90	191.30	UN
Cameroon	ALL	1982	158.57	153.13	164.17	IHME
Cameroon	ALL	1982	169.05	140.19	202.74	RW2
Cameroon	ALL	1982	170.10	157.60	184.20	UN
Cameroon	ALL	1983	154.54	149.26	159.79	IHME
Cameroon	ALL	1983	161.47	131.73	197.45	RW2
Cameroon	ALL	1983	163.40	151.70	176.20	UN
Cameroon	ALL	1984	150.57	145.53	155.71	IHME
Cameroon	ALL	1984	154.94	123.80	193.26	RW2
Cameroon	ALL	1984	156.60	145.60	168.40	UN
Cameroon	ALL	1985	146.38	141.57	151.31	IHME
Cameroon	ALL	1985	148.40	120.08	181.31	RW2
Cameroon	ALL	1985	150.20	139.90	161.20	UN
Cameroon	ALL	1986	143.28	138.78	148.09	IHME
Cameroon	ALL	1986	143.81	118.27	173.32	RW2
Cameroon	ALL	1986	144.70	135.00	155.20	UN
Cameroon	ALL	1987	140.07	135.65	144.70	IHME
Cameroon	ALL	1987	140.61	116.90	168.60	RW2
Cameroon	ALL	1987	140.70	131.10	150.80	UN
Cameroon	ALL	1988	138.25	133.80	142.65	IHME
Cameroon	ALL	1988	138.69	114.19	167.39	RW2
Cameroon	ALL	1988	138.10	128.60	148.00	UN
Cameroon	ALL	1989	137.09	132.59	141.42	IHME
Cameroon	ALL	1989	138.14	112.48	168.40	RW2
Cameroon	ALL	1989	137.20	127.70	147.00	UN
Cameroon	ALL	1990	136.78	132.33	141.33	IHME
Cameroon	ALL	1990	139.03	113.89	169.79	RW2
Cameroon	ALL	1990	138.00	128.40	147.70	UN
Cameroon	ALL	1991	137.02	132.72	141.58	IHME
Cameroon	ALL	1991	140.69	116.64	168.97	RW2
Cameroon	ALL	1991	140.00	130.30	150.10	UN
Cameroon	ALL	1992	137.81	133.58	142.26	IHME
Cameroon	ALL	1992	143.12	119.11	170.67	RW2
Cameroon	ALL	1992	142.80	132.70	153.20	UN
Cameroon	ALL	1993	138.94	134.62	143.52	IHME
Cameroon	ALL	1993	146.06	120.76	174.79	RW2
Cameroon	ALL	1993	146.00	135.60	156.60	UN
Cameroon	ALL	1994	139.90	135.31	144.70	IHME
Cameroon	ALL	1994	149.02	121.54	180.59	RW2
Cameroon	ALL	1994	149.00	138.10	160.30	UN
Cameroon	ALL	1995	140.65	135.94	145.43	IHME
Cameroon	ALL	1995	152.29	125.23	184.73	RW2
Cameroon	ALL	1995	151.80	140.80	163.60	UN
Cameroon	ALL	1996	141.03	136.11	146.04	IHME
Cameroon	ALL	1996	154.16	128.05	185.30	RW2
Cameroon	ALL	1996	154.00	142.60	166.40	UN
Cameroon	ALL	1997	140.83	135.72	145.96	IHME
Cameroon	ALL	1997	154.90	129.39	184.49	RW2
Cameroon	ALL	1997	155.30	143.20	168.20	UN
Cameroon	ALL	1998	140.05	134.75	145.34	IHME
Cameroon	ALL	1998	154.34	128.17	185.62	RW2
Cameroon	ALL	1998	155.30	142.90	168.90	UN
Cameroon	ALL	1999	138.82	133.46	144.25	IHME
Cameroon	ALL	1999	152.38	125.05	184.50	RW2
Cameroon	ALL	1999	153.80	140.90	167.70	UN
Cameroon	ALL	2000	137.49	132.05	143.00	IHME
Cameroon	ALL	2000	148.80	121.75	179.46	RW2
Cameroon	ALL	2000	150.40	137.50	164.20	UN
Cameroon	ALL	2001	135.69	129.98	141.29	IHME
Cameroon	ALL	2001	144.85	119.73	173.50	RW2
Cameroon	ALL	2001	145.70	132.90	159.00	UN

Continued on next page

Country	Region	Year	Median	Lower	Upper	Method
Cameroon	ALL	2002	133.32	127.66	139.18	IHME
Cameroon	ALL	2002	140.31	116.96	167.52	RW2
Cameroon	ALL	2002	140.20	127.30	154.00	UN
Cameroon	ALL	2003	130.55	124.53	136.46	IHME
Cameroon	ALL	2003	135.57	112.61	162.83	RW2
Cameroon	ALL	2003	134.70	121.00	149.60	UN
Cameroon	ALL	2004	127.59	121.27	133.59	IHME
Cameroon	ALL	2004	130.55	106.66	159.13	RW2
Cameroon	ALL	2004	129.60	114.20	146.80	UN
Cameroon	ALL	2005	124.64	118.27	131.03	IHME
Cameroon	ALL	2005	125.74	102.20	153.76	RW2
Cameroon	ALL	2005	125.10	107.30	144.90	UN
Cameroon	ALL	2006	121.33	114.53	128.00	IHME
Cameroon	ALL	2006	121.03	99.49	146.34	RW2
Cameroon	ALL	2006	120.70	100.20	143.90	UN
Cameroon	ALL	2007	117.91	110.78	124.89	IHME
Cameroon	ALL	2007	116.62	96.63	140.06	RW2
Cameroon	ALL	2007	116.60	93.00	144.70	UN
Cameroon	ALL	2008	114.28	107.09	121.87	IHME
Cameroon	ALL	2008	112.60	92.49	136.47	RW2
Cameroon	ALL	2008	112.80	85.40	146.10	UN
Cameroon	ALL	2009	110.69	102.94	118.75	IHME
Cameroon	ALL	2009	108.64	87.48	134.42	RW2
Cameroon	ALL	2009	109.00	78.60	147.40	UN
Cameroon	ALL	2010	106.82	98.45	115.54	IHME
Cameroon	ALL	2010	105.10	83.22	132.90	RW2
Cameroon	ALL	2010	104.80	72.10	148.30	UN
Cameroon	ALL	2011	103.29	94.10	113.02	IHME
Cameroon	ALL	2011	101.72	81.02	127.20	RW2
Cameroon	ALL	2011	101.00	66.60	149.10	UN
Cameroon	ALL	2012	99.86	89.90	110.81	IHME
Cameroon	ALL	2012	98.42	79.00	121.95	RW2
Cameroon	ALL	2012	97.40	61.90	149.20	UN
Cameroon	ALL	2013	96.26	85.56	107.79	IHME
Cameroon	ALL	2013	95.31	74.33	121.18	RW2
Cameroon	ALL	2013	93.90	57.30	148.60	UN
Cameroon	ALL	2014	93.17	81.58	105.53	IHME
Cameroon	ALL	2014	92.18	65.29	128.48	RW2
Cameroon	ALL	2014	90.60	53.50	148.20	UN
Cameroon	ALL	2015	89.76	77.87	103.40	IHME
Cameroon	ALL	2015	89.03	53.07	145.74	RW2
Cameroon	ALL	2015	87.90	50.30	147.90	UN
Cameroon	ALL	2016	86.30	43.00	166.82	RW2
Cameroon	ALL	2017	83.26	33.54	193.94	RW2
Cameroon	ALL	2018	80.48	25.87	229.60	RW2
Cameroon	ALL	2019	77.67	18.78	268.82	RW2
Cameroon	ALL	15-19	83.25	34.06	190.98	RW2
Cameroon	CENTRE/SUD/EST	1980	175.36	130.52	231.36	RW2
Cameroon	CENTRE/SUD/EST	1981	167.08	134.01	205.95	RW2
Cameroon	CENTRE/SUD/EST	1982	158.85	130.83	191.35	RW2
Cameroon	CENTRE/SUD/EST	1983	151.22	123.76	184.03	RW2
Cameroon	CENTRE/SUD/EST	1984	144.36	116.90	177.58	RW2
Cameroon	CENTRE/SUD/EST	1985	138.10	113.46	166.61	RW2
Cameroon	CENTRE/SUD/EST	1986	133.65	111.87	158.34	RW2
Cameroon	CENTRE/SUD/EST	1987	130.62	110.53	153.66	RW2
Cameroon	CENTRE/SUD/EST	1988	129.09	108.53	152.68	RW2
Cameroon	CENTRE/SUD/EST	1989	128.90	107.36	153.62	RW2
Cameroon	CENTRE/SUD/EST	1990	130.14	109.03	155.17	RW2
Cameroon	CENTRE/SUD/EST	1991	132.06	112.17	155.24	RW2
Cameroon	CENTRE/SUD/EST	1992	134.46	114.70	156.90	RW2
Cameroon	CENTRE/SUD/EST	1993	137.09	116.31	160.61	RW2
Cameroon	CENTRE/SUD/EST	1994	139.89	117.12	165.39	RW2
Cameroon	CENTRE/SUD/EST	1995	142.42	119.79	168.76	RW2

Continued on next page

Country	Region	Year	Median	Lower	Upper	Method
Cameroon	CENTRE/SUD/EST	1996	144.35	122.82	168.90	RW2
Cameroon	CENTRE/SUD/EST	1997	145.38	124.58	169.24	RW2
Cameroon	CENTRE/SUD/EST	1998	145.41	124.13	169.87	RW2
Cameroon	CENTRE/SUD/EST	1999	144.26	120.98	170.32	RW2
Cameroon	CENTRE/SUD/EST	2000	141.83	118.91	167.74	RW2
Cameroon	CENTRE/SUD/EST	2001	138.19	117.12	161.85	RW2
Cameroon	CENTRE/SUD/EST	2002	133.35	113.87	155.63	RW2
Cameroon	CENTRE/SUD/EST	2003	127.56	108.34	150.35	RW2
Cameroon	CENTRE/SUD/EST	2004	121.32	101.50	144.98	RW2
Cameroon	CENTRE/SUD/EST	2005	114.53	95.12	136.91	RW2
Cameroon	CENTRE/SUD/EST	2006	108.49	91.20	128.55	RW2
Cameroon	CENTRE/SUD/EST	2007	102.95	86.64	121.64	RW2
Cameroon	CENTRE/SUD/EST	2008	98.18	81.44	117.73	RW2
Cameroon	CENTRE/SUD/EST	2009	93.96	75.98	115.37	RW2
Cameroon	CENTRE/SUD/EST	2010	90.67	71.77	114.41	RW2
Cameroon	CENTRE/SUD/EST	2011	87.45	68.76	110.65	RW2
Cameroon	CENTRE/SUD/EST	2012	84.55	65.79	107.42	RW2
Cameroon	CENTRE/SUD/EST	2013	81.82	61.13	108.00	RW2
Cameroon	CENTRE/SUD/EST	2014	79.14	54.09	113.37	RW2
Cameroon	CENTRE/SUD/EST	2015	76.43	44.98	126.12	RW2
Cameroon	CENTRE/SUD/EST	2016	73.74	36.82	141.86	RW2
Cameroon	CENTRE/SUD/EST	2017	71.35	29.44	163.42	RW2
Cameroon	CENTRE/SUD/EST	2018	68.73	22.84	191.13	RW2
Cameroon	CENTRE/SUD/EST	2019	66.44	17.42	226.90	RW2
Cameroon	NORD-OUEST/SUD-OUEST	1980	136.06	99.24	184.36	RW2
Cameroon	NORD-OUEST/SUD-OUEST	1981	128.95	101.55	162.25	RW2
Cameroon	NORD-OUEST/SUD-OUEST	1982	122.20	98.68	150.01	RW2
Cameroon	NORD-OUEST/SUD-OUEST	1983	115.79	93.07	143.87	RW2
Cameroon	NORD-OUEST/SUD-OUEST	1984	110.22	87.96	137.84	RW2
Cameroon	NORD-OUEST/SUD-OUEST	1985	105.05	84.52	128.83	RW2
Cameroon	NORD-OUEST/SUD-OUEST	1986	101.32	83.36	122.61	RW2
Cameroon	NORD-OUEST/SUD-OUEST	1987	98.90	82.34	118.25	RW2
Cameroon	NORD-OUEST/SUD-OUEST	1988	97.62	80.90	117.16	RW2
Cameroon	NORD-OUEST/SUD-OUEST	1989	97.41	79.98	118.05	RW2
Cameroon	NORD-OUEST/SUD-OUEST	1990	98.42	81.33	119.12	RW2
Cameroon	NORD-OUEST/SUD-OUEST	1991	100.02	83.74	118.85	RW2
Cameroon	NORD-OUEST/SUD-OUEST	1992	101.97	86.04	120.30	RW2
Cameroon	NORD-OUEST/SUD-OUEST	1993	104.27	87.68	123.71	RW2
Cameroon	NORD-OUEST/SUD-OUEST	1994	106.68	88.44	127.95	RW2
Cameroon	NORD-OUEST/SUD-OUEST	1995	108.89	90.52	130.93	RW2
Cameroon	NORD-OUEST/SUD-OUEST	1996	110.61	93.16	130.78	RW2
Cameroon	NORD-OUEST/SUD-OUEST	1997	111.69	94.70	131.70	RW2
Cameroon	NORD-OUEST/SUD-OUEST	1998	112.06	94.34	132.75	RW2
Cameroon	NORD-OUEST/SUD-OUEST	1999	111.36	92.54	133.29	RW2
Cameroon	NORD-OUEST/SUD-OUEST	2000	109.75	91.23	131.67	RW2
Cameroon	NORD-OUEST/SUD-OUEST	2001	107.17	89.93	127.29	RW2
Cameroon	NORD-OUEST/SUD-OUEST	2002	103.64	87.52	122.62	RW2
Cameroon	NORD-OUEST/SUD-OUEST	2003	99.41	83.23	118.67	RW2
Cameroon	NORD-OUEST/SUD-OUEST	2004	94.80	78.10	114.72	RW2
Cameroon	NORD-OUEST/SUD-OUEST	2005	89.75	73.24	109.04	RW2
Cameroon	NORD-OUEST/SUD-OUEST	2006	85.22	70.37	102.96	RW2
Cameroon	NORD-OUEST/SUD-OUEST	2007	81.24	67.25	98.05	RW2
Cameroon	NORD-OUEST/SUD-OUEST	2008	77.78	63.31	95.19	RW2
Cameroon	NORD-OUEST/SUD-OUEST	2009	74.72	59.30	93.53	RW2
Cameroon	NORD-OUEST/SUD-OUEST	2010	72.42	56.11	93.13	RW2
Cameroon	NORD-OUEST/SUD-OUEST	2011	70.16	54.10	90.51	RW2
Cameroon	NORD-OUEST/SUD-OUEST	2012	68.10	51.93	88.33	RW2
Cameroon	NORD-OUEST/SUD-OUEST	2013	66.15	48.53	88.75	RW2
Cameroon	NORD-OUEST/SUD-OUEST	2014	64.15	43.05	93.47	RW2
Cameroon	NORD-OUEST/SUD-OUEST	2015	62.30	36.10	104.72	RW2
Cameroon	NORD-OUEST/SUD-OUEST	2016	60.38	29.59	118.58	RW2
Cameroon	NORD-OUEST/SUD-OUEST	2017	58.70	23.77	137.70	RW2
Cameroon	NORD-OUEST/SUD-OUEST	2018	56.79	18.65	161.36	RW2

Continued on next page

Country	Region	Year	Median	Lower	Upper	Method
Cameroon	NORD-OUEST/SUD-OUEST	2019	55.27	14.43	191.98	RW2
Cameroon	OUEST/LITTORAL	1980	137.91	101.12	185.33	RW2
Cameroon	OUEST/LITTORAL	1981	131.29	104.49	164.46	RW2
Cameroon	OUEST/LITTORAL	1982	124.96	101.65	152.99	RW2
Cameroon	OUEST/LITTORAL	1983	119.03	95.86	147.40	RW2
Cameroon	OUEST/LITTORAL	1984	113.77	90.94	142.08	RW2
Cameroon	OUEST/LITTORAL	1985	108.95	88.09	133.83	RW2
Cameroon	OUEST/LITTORAL	1986	105.66	86.87	127.67	RW2
Cameroon	OUEST/LITTORAL	1987	103.53	86.03	123.97	RW2
Cameroon	OUEST/LITTORAL	1988	102.61	84.87	123.47	RW2
Cameroon	OUEST/LITTORAL	1989	102.94	84.48	124.79	RW2
Cameroon	OUEST/LITTORAL	1990	104.37	86.25	126.40	RW2
Cameroon	OUEST/LITTORAL	1991	106.55	88.96	126.86	RW2
Cameroon	OUEST/LITTORAL	1992	109.22	91.95	129.02	RW2
Cameroon	OUEST/LITTORAL	1993	112.17	94.05	133.11	RW2
Cameroon	OUEST/LITTORAL	1994	115.31	95.68	138.16	RW2
Cameroon	OUEST/LITTORAL	1995	118.44	98.91	141.56	RW2
Cameroon	OUEST/LITTORAL	1996	120.89	102.05	142.65	RW2
Cameroon	OUEST/LITTORAL	1997	122.83	104.68	143.91	RW2
Cameroon	OUEST/LITTORAL	1998	123.85	104.87	146.00	RW2
Cameroon	OUEST/LITTORAL	1999	123.90	103.33	147.53	RW2
Cameroon	OUEST/LITTORAL	2000	122.87	102.26	146.71	RW2
Cameroon	OUEST/LITTORAL	2001	120.75	101.72	142.83	RW2
Cameroon	OUEST/LITTORAL	2002	117.70	99.56	138.48	RW2
Cameroon	OUEST/LITTORAL	2003	113.81	95.51	135.54	RW2
Cameroon	OUEST/LITTORAL	2004	109.57	90.61	132.78	RW2
Cameroon	OUEST/LITTORAL	2005	104.86	85.72	127.32	RW2
Cameroon	OUEST/LITTORAL	2006	100.64	82.87	121.54	RW2
Cameroon	OUEST/LITTORAL	2007	96.86	79.83	117.09	RW2
Cameroon	OUEST/LITTORAL	2008	93.64	75.86	114.90	RW2
Cameroon	OUEST/LITTORAL	2009	91.06	72.01	114.29	RW2
Cameroon	OUEST/LITTORAL	2010	89.14	69.00	114.69	RW2
Cameroon	OUEST/LITTORAL	2011	87.26	67.09	112.85	RW2
Cameroon	OUEST/LITTORAL	2012	85.67	65.16	111.49	RW2
Cameroon	OUEST/LITTORAL	2013	84.11	61.96	113.19	RW2
Cameroon	OUEST/LITTORAL	2014	82.51	55.86	120.13	RW2
Cameroon	OUEST/LITTORAL	2015	80.91	47.27	135.48	RW2
Cameroon	OUEST/LITTORAL	2016	79.31	39.17	153.92	RW2
Cameroon	OUEST/LITTORAL	2017	77.90	31.93	178.90	RW2
Cameroon	OUEST/LITTORAL	2018	76.18	25.23	209.72	RW2
Cameroon	OUEST/LITTORAL	2019	74.96	19.53	251.78	RW2
Cameroon	YAOUNDE/DOUALA	1980	134.53	94.11	190.39	RW2
Cameroon	YAOUNDE/DOUALA	1981	126.58	95.11	167.73	RW2
Cameroon	YAOUNDE/DOUALA	1982	119.15	91.93	153.63	RW2
Cameroon	YAOUNDE/DOUALA	1983	112.09	86.52	144.64	RW2
Cameroon	YAOUNDE/DOUALA	1984	105.86	81.59	137.35	RW2
Cameroon	YAOUNDE/DOUALA	1985	100.14	78.28	126.86	RW2
Cameroon	YAOUNDE/DOUALA	1986	95.90	76.73	119.22	RW2
Cameroon	YAOUNDE/DOUALA	1987	92.85	75.14	114.12	RW2
Cameroon	YAOUNDE/DOUALA	1988	91.00	73.67	111.72	RW2
Cameroon	YAOUNDE/DOUALA	1989	90.19	72.53	111.19	RW2
Cameroon	YAOUNDE/DOUALA	1990	90.52	73.33	111.05	RW2
Cameroon	YAOUNDE/DOUALA	1991	91.31	75.00	110.65	RW2
Cameroon	YAOUNDE/DOUALA	1992	92.52	76.78	111.28	RW2
Cameroon	YAOUNDE/DOUALA	1993	94.09	77.80	113.43	RW2
Cameroon	YAOUNDE/DOUALA	1994	95.59	77.85	116.00	RW2
Cameroon	YAOUNDE/DOUALA	1995	97.22	79.68	118.06	RW2
Cameroon	YAOUNDE/DOUALA	1996	98.36	81.64	118.23	RW2
Cameroon	YAOUNDE/DOUALA	1997	98.87	82.72	117.79	RW2
Cameroon	YAOUNDE/DOUALA	1998	98.86	82.09	118.34	RW2
Cameroon	YAOUNDE/DOUALA	1999	97.89	80.33	118.29	RW2
Cameroon	YAOUNDE/DOUALA	2000	96.15	79.07	116.23	RW2
Cameroon	YAOUNDE/DOUALA	2001	93.51	77.72	112.21	RW2

Continued on next page

Country	Region	Year	Median	Lower	Upper	Method
Cameroon	YAOUNDE/DOUALA	2002	90.23	75.42	107.71	RW2
Cameroon	YAOUNDE/DOUALA	2003	86.19	71.41	103.74	RW2
Cameroon	YAOUNDE/DOUALA	2004	81.95	66.97	100.29	RW2
Cameroon	YAOUNDE/DOUALA	2005	77.33	62.71	94.81	RW2
Cameroon	YAOUNDE/DOUALA	2006	73.16	59.77	89.16	RW2
Cameroon	YAOUNDE/DOUALA	2007	69.39	56.73	84.45	RW2
Cameroon	YAOUNDE/DOUALA	2008	66.20	53.21	81.88	RW2
Cameroon	YAOUNDE/DOUALA	2009	63.37	49.45	80.75	RW2
Cameroon	YAOUNDE/DOUALA	2010	61.14	46.62	80.27	RW2
Cameroon	YAOUNDE/DOUALA	2011	58.88	44.21	78.10	RW2
Cameroon	YAOUNDE/DOUALA	2012	56.90	42.01	76.32	RW2
Cameroon	YAOUNDE/DOUALA	2013	55.03	38.84	76.88	RW2
Cameroon	YAOUNDE/DOUALA	2014	53.23	34.38	80.81	RW2
Cameroon	YAOUNDE/DOUALA	2015	51.36	28.74	90.09	RW2
Cameroon	YAOUNDE/DOUALA	2016	49.63	23.40	101.26	RW2
Cameroon	YAOUNDE/DOUALA	2017	47.89	18.69	117.17	RW2
Cameroon	YAOUNDE/DOUALA	2018	46.25	14.67	138.57	RW2
Cameroon	YAOUNDE/DOUALA	2019	44.72	10.99	164.01	RW2
Chad	ALL	1980	233.65	225.33	242.15	IHME
Chad	ALL	1980	242.81	176.54	323.55	RW2
Chad	ALL	1980	240.10	219.40	263.20	UN
Chad	ALL	1981	233.09	224.78	242.09	IHME
Chad	ALL	1981	239.91	189.81	297.91	RW2
Chad	ALL	1981	238.10	218.00	260.10	UN
Chad	ALL	1982	230.47	222.69	238.69	IHME
Chad	ALL	1982	237.16	194.05	286.31	RW2
Chad	ALL	1982	236.00	216.50	257.30	UN
Chad	ALL	1983	227.54	219.54	236.00	IHME
Chad	ALL	1983	234.04	190.39	284.18	RW2
Chad	ALL	1983	233.50	214.50	254.20	UN
Chad	ALL	1984	224.46	216.88	231.97	IHME
Chad	ALL	1984	231.41	185.12	284.54	RW2
Chad	ALL	1984	230.90	212.70	250.80	UN
Chad	ALL	1985	219.46	211.96	227.49	IHME
Chad	ALL	1985	228.24	185.00	278.53	RW2
Chad	ALL	1985	228.20	210.50	247.50	UN
Chad	ALL	1986	215.10	208.41	222.22	IHME
Chad	ALL	1986	225.30	184.37	272.40	RW2
Chad	ALL	1986	225.50	208.40	244.30	UN
Chad	ALL	1987	214.03	206.54	222.32	IHME
Chad	ALL	1987	222.44	183.56	267.58	RW2
Chad	ALL	1987	222.80	206.10	241.00	UN
Chad	ALL	1988	207.38	200.78	214.08	IHME
Chad	ALL	1988	219.39	180.17	264.64	RW2
Chad	ALL	1988	220.10	203.80	237.80	UN
Chad	ALL	1989	204.04	197.68	210.65	IHME
Chad	ALL	1989	216.48	176.97	262.45	RW2
Chad	ALL	1989	217.30	201.50	234.60	UN
Chad	ALL	1990	201.29	194.65	207.57	IHME
Chad	ALL	1990	213.60	175.31	257.70	RW2
Chad	ALL	1990	214.60	199.00	231.50	UN
Chad	ALL	1991	197.18	190.91	203.45	IHME
Chad	ALL	1991	211.15	175.05	251.68	RW2
Chad	ALL	1991	212.00	196.60	228.60	UN
Chad	ALL	1992	194.98	188.54	201.32	IHME
Chad	ALL	1992	208.99	173.91	248.22	RW2
Chad	ALL	1992	209.40	194.40	225.80	UN
Chad	ALL	1993	192.85	186.46	199.32	IHME
Chad	ALL	1993	207.14	171.64	247.10	RW2
Chad	ALL	1993	207.00	192.30	223.10	UN
Chad	ALL	1994	191.16	184.66	197.65	IHME
Chad	ALL	1994	205.32	168.53	247.55	RW2
Chad	ALL	1994	204.80	190.10	220.50	UN

Continued on next page

Country	Region	Year	Median	Lower	Upper	Method
Chad	ALL	1995	189.56	182.74	196.16	IHME
Chad	ALL	1995	204.00	168.67	244.90	RW2
Chad	ALL	1995	202.70	188.10	218.30	UN
Chad	ALL	1996	188.40	181.36	195.33	IHME
Chad	ALL	1996	201.83	168.55	240.64	RW2
Chad	ALL	1996	200.60	186.10	216.20	UN
Chad	ALL	1997	187.30	180.08	194.51	IHME
Chad	ALL	1997	199.29	167.32	235.97	RW2
Chad	ALL	1997	198.30	183.70	213.90	UN
Chad	ALL	1998	185.84	178.54	193.20	IHME
Chad	ALL	1998	196.39	163.96	234.80	RW2
Chad	ALL	1998	195.80	181.10	211.60	UN
Chad	ALL	1999	184.35	176.66	192.12	IHME
Chad	ALL	1999	193.10	159.39	232.28	RW2
Chad	ALL	1999	193.00	178.10	209.00	UN
Chad	ALL	2000	183.24	175.62	191.24	IHME
Chad	ALL	2000	189.17	155.60	227.00	RW2
Chad	ALL	2000	190.20	175.00	206.30	UN
Chad	ALL	2001	181.22	173.12	189.81	IHME
Chad	ALL	2001	185.99	154.21	221.95	RW2
Chad	ALL	2001	187.30	171.90	203.90	UN
Chad	ALL	2002	179.27	170.45	188.22	IHME
Chad	ALL	2002	183.14	152.86	217.72	RW2
Chad	ALL	2002	184.60	168.60	201.50	UN
Chad	ALL	2003	176.81	167.47	186.10	IHME
Chad	ALL	2003	180.83	150.31	215.69	RW2
Chad	ALL	2003	181.90	165.20	199.50	UN
Chad	ALL	2004	172.44	162.57	182.25	IHME
Chad	ALL	2004	178.57	146.10	215.49	RW2
Chad	ALL	2004	179.40	161.30	198.30	UN
Chad	ALL	2005	167.91	157.72	178.33	IHME
Chad	ALL	2005	177.23	144.94	216.06	RW2
Chad	ALL	2005	177.00	157.90	197.50	UN
Chad	ALL	2006	164.11	153.38	175.20	IHME
Chad	ALL	2006	174.98	144.01	211.58	RW2
Chad	ALL	2006	174.60	154.00	196.40	UN
Chad	ALL	2007	159.84	148.54	171.48	IHME
Chad	ALL	2007	172.42	142.48	207.53	RW2
Chad	ALL	2007	171.50	149.40	195.50	UN
Chad	ALL	2008	156.33	144.42	169.18	IHME
Chad	ALL	2008	169.52	139.03	205.64	RW2
Chad	ALL	2008	168.00	143.80	195.40	UN
Chad	ALL	2009	152.64	140.59	166.63	IHME
Chad	ALL	2009	165.77	134.21	204.19	RW2
Chad	ALL	2009	164.00	137.30	196.00	UN
Chad	ALL	2010	148.24	135.62	162.93	IHME
Chad	ALL	2010	161.42	129.21	200.33	RW2
Chad	ALL	2010	160.10	129.80	196.70	UN
Chad	ALL	2011	144.54	131.49	159.96	IHME
Chad	ALL	2011	157.30	127.64	191.98	RW2
Chad	ALL	2011	156.00	122.00	198.20	UN
Chad	ALL	2012	140.78	127.16	157.21	IHME
Chad	ALL	2012	152.98	126.34	183.75	RW2
Chad	ALL	2012	151.60	114.30	199.90	UN
Chad	ALL	2013	137.30	123.14	154.24	IHME
Chad	ALL	2013	148.75	120.80	181.75	RW2
Chad	ALL	2013	147.10	107.60	201.30	UN
Chad	ALL	2014	134.16	119.68	151.86	IHME
Chad	ALL	2014	144.54	107.09	191.83	RW2
Chad	ALL	2014	142.90	100.70	202.30	UN
Chad	ALL	2015	130.50	115.86	148.82	IHME
Chad	ALL	2015	140.37	87.81	216.55	RW2
Chad	ALL	2015	138.70	94.00	202.80	UN

Continued on next page

Country	Region	Year	Median	Lower	Upper	Method
Chad	ALL	2016	136.67	71.78	244.76	RW2
Chad	ALL	2017	132.52	56.54	279.90	RW2
Chad	ALL	2018	128.70	44.01	324.35	RW2
Chad	ALL	2019	124.81	32.27	371.24	RW2
Chad	ALL	15-19	132.55	57.38	275.67	RW2
Chad	ZONE 1	1980	190.83	127.72	274.43	RW2
Chad	ZONE 1	1981	190.76	138.33	258.26	RW2
Chad	ZONE 1	1982	190.70	143.14	249.53	RW2
Chad	ZONE 1	1983	190.52	144.58	246.96	RW2
Chad	ZONE 1	1984	190.35	145.51	245.16	RW2
Chad	ZONE 1	1985	190.17	147.80	241.96	RW2
Chad	ZONE 1	1986	189.94	149.77	238.05	RW2
Chad	ZONE 1	1987	189.51	151.49	234.71	RW2
Chad	ZONE 1	1988	188.86	151.94	232.90	RW2
Chad	ZONE 1	1989	188.20	152.08	231.29	RW2
Chad	ZONE 1	1990	186.98	152.84	227.81	RW2
Chad	ZONE 1	1991	185.68	153.40	222.82	RW2
Chad	ZONE 1	1992	183.82	153.31	219.00	RW2
Chad	ZONE 1	1993	181.47	151.43	216.53	RW2
Chad	ZONE 1	1994	179.01	148.68	214.61	RW2
Chad	ZONE 1	1995	176.35	147.29	209.71	RW2
Chad	ZONE 1	1996	173.64	146.48	204.33	RW2
Chad	ZONE 1	1997	171.37	145.84	200.55	RW2
Chad	ZONE 1	1998	169.33	143.45	199.20	RW2
Chad	ZONE 1	1999	167.72	140.40	198.75	RW2
Chad	ZONE 1	2000	166.38	139.30	197.32	RW2
Chad	ZONE 1	2001	165.53	140.23	194.50	RW2
Chad	ZONE 1	2002	165.10	140.46	192.69	RW2
Chad	ZONE 1	2003	164.84	139.38	193.86	RW2
Chad	ZONE 1	2004	164.92	137.85	196.25	RW2
Chad	ZONE 1	2005	165.28	137.73	197.41	RW2
Chad	ZONE 1	2006	165.15	138.58	195.79	RW2
Chad	ZONE 1	2007	164.72	138.41	195.02	RW2
Chad	ZONE 1	2008	163.93	135.86	196.36	RW2
Chad	ZONE 1	2009	162.92	132.47	198.79	RW2
Chad	ZONE 1	2010	161.36	128.70	200.22	RW2
Chad	ZONE 1	2011	159.74	127.28	198.71	RW2
Chad	ZONE 1	2012	158.14	125.56	197.50	RW2
Chad	ZONE 1	2013	156.39	121.20	200.96	RW2
Chad	ZONE 1	2014	154.58	111.01	212.40	RW2
Chad	ZONE 1	2015	152.91	95.78	236.94	RW2
Chad	ZONE 1	2016	151.12	80.91	264.70	RW2
Chad	ZONE 1	2017	149.62	67.24	300.63	RW2
Chad	ZONE 1	2018	147.56	54.24	342.79	RW2
Chad	ZONE 1	2019	146.32	42.85	396.69	RW2
Chad	ZONE 2	1980	267.95	190.93	364.30	RW2
Chad	ZONE 2	1981	261.20	198.64	337.09	RW2
Chad	ZONE 2	1982	254.74	199.22	320.10	RW2
Chad	ZONE 2	1983	247.97	194.79	310.41	RW2
Chad	ZONE 2	1984	241.50	189.44	303.38	RW2
Chad	ZONE 2	1985	235.18	186.26	292.28	RW2
Chad	ZONE 2	1986	228.89	183.81	281.54	RW2
Chad	ZONE 2	1987	222.71	180.14	271.98	RW2
Chad	ZONE 2	1988	216.74	175.92	263.85	RW2
Chad	ZONE 2	1989	210.76	170.98	256.54	RW2
Chad	ZONE 2	1990	204.94	167.44	247.61	RW2
Chad	ZONE 2	1991	198.97	164.36	238.53	RW2
Chad	ZONE 2	1992	192.82	160.75	230.25	RW2
Chad	ZONE 2	1993	186.85	155.64	223.44	RW2
Chad	ZONE 2	1994	180.60	149.00	216.72	RW2
Chad	ZONE 2	1995	174.60	144.91	208.53	RW2
Chad	ZONE 2	1996	168.72	141.71	199.90	RW2
Chad	ZONE 2	1997	162.83	137.73	191.73	RW2

Continued on next page

Country	Region	Year	Median	Lower	Upper	Method
Chad	ZONE 2	1998	157.44	132.19	186.33	RW2
Chad	ZONE 2	1999	152.06	126.20	181.58	RW2
Chad	ZONE 2	2000	147.03	122.11	175.77	RW2
Chad	ZONE 2	2001	142.40	119.16	169.41	RW2
Chad	ZONE 2	2002	138.37	116.07	163.92	RW2
Chad	ZONE 2	2003	134.45	111.66	160.31	RW2
Chad	ZONE 2	2004	131.04	107.44	158.31	RW2
Chad	ZONE 2	2005	127.97	104.74	155.51	RW2
Chad	ZONE 2	2006	124.75	102.67	150.63	RW2
Chad	ZONE 2	2007	121.58	100.16	146.34	RW2
Chad	ZONE 2	2008	118.58	96.61	144.31	RW2
Chad	ZONE 2	2009	115.39	92.16	143.51	RW2
Chad	ZONE 2	2010	112.14	88.27	141.73	RW2
Chad	ZONE 2	2011	108.84	85.62	137.64	RW2
Chad	ZONE 2	2012	105.74	83.28	133.85	RW2
Chad	ZONE 2	2013	102.69	78.71	133.95	RW2
Chad	ZONE 2	2014	99.77	70.73	139.87	RW2
Chad	ZONE 2	2015	96.86	59.82	154.89	RW2
Chad	ZONE 2	2016	94.09	49.34	171.98	RW2
Chad	ZONE 2	2017	91.29	39.90	195.60	RW2
Chad	ZONE 2	2018	88.63	31.74	226.24	RW2
Chad	ZONE 2	2019	86.13	24.16	261.25	RW2
Chad	ZONE 3	1980	310.43	225.28	413.26	RW2
Chad	ZONE 3	1981	303.22	234.40	384.93	RW2
Chad	ZONE 3	1982	295.81	234.90	367.52	RW2
Chad	ZONE 3	1983	289.39	229.90	357.48	RW2
Chad	ZONE 3	1984	282.22	223.56	349.00	RW2
Chad	ZONE 3	1985	275.61	220.52	337.92	RW2
Chad	ZONE 3	1986	268.78	217.73	326.26	RW2
Chad	ZONE 3	1987	262.22	214.44	316.31	RW2
Chad	ZONE 3	1988	255.68	210.24	306.62	RW2
Chad	ZONE 3	1989	249.39	205.41	299.61	RW2
Chad	ZONE 3	1990	243.24	202.04	290.00	RW2
Chad	ZONE 3	1991	236.78	199.05	279.36	RW2
Chad	ZONE 3	1992	230.31	194.82	269.87	RW2
Chad	ZONE 3	1993	223.97	189.08	263.13	RW2
Chad	ZONE 3	1994	217.48	182.29	257.41	RW2
Chad	ZONE 3	1995	211.19	177.46	248.82	RW2
Chad	ZONE 3	1996	204.92	174.03	239.55	RW2
Chad	ZONE 3	1997	198.86	169.67	231.57	RW2
Chad	ZONE 3	1998	193.01	164.20	225.74	RW2
Chad	ZONE 3	1999	187.39	157.41	221.37	RW2
Chad	ZONE 3	2000	182.31	153.41	215.21	RW2
Chad	ZONE 3	2001	177.55	150.74	208.12	RW2
Chad	ZONE 3	2002	173.20	147.78	202.24	RW2
Chad	ZONE 3	2003	169.13	143.17	198.41	RW2
Chad	ZONE 3	2004	165.32	138.60	195.97	RW2
Chad	ZONE 3	2005	161.97	135.51	192.31	RW2
Chad	ZONE 3	2006	158.26	133.56	186.75	RW2
Chad	ZONE 3	2007	154.79	130.95	181.69	RW2
Chad	ZONE 3	2008	151.01	126.44	179.87	RW2
Chad	ZONE 3	2009	147.03	120.55	178.49	RW2
Chad	ZONE 3	2010	142.87	114.83	176.40	RW2
Chad	ZONE 3	2011	139.02	112.09	171.02	RW2
Chad	ZONE 3	2012	135.04	109.06	166.38	RW2
Chad	ZONE 3	2013	130.91	102.99	165.68	RW2
Chad	ZONE 3	2014	127.25	92.71	173.52	RW2
Chad	ZONE 3	2015	123.51	78.22	190.18	RW2
Chad	ZONE 3	2016	119.96	64.76	212.86	RW2
Chad	ZONE 3	2017	116.69	52.50	239.79	RW2
Chad	ZONE 3	2018	113.30	41.54	273.02	RW2
Chad	ZONE 3	2019	109.52	31.86	316.51	RW2
Chad	ZONE 4	1980	222.05	148.48	318.73	RW2

Continued on next page

Country	Region	Year	Median	Lower	Upper	Method
Chad	ZONE 4	1981	217.00	156.04	293.43	RW2
Chad	ZONE 4	1982	211.77	157.38	278.57	RW2
Chad	ZONE 4	1983	206.48	154.95	269.76	RW2
Chad	ZONE 4	1984	201.37	152.08	261.51	RW2
Chad	ZONE 4	1985	196.33	149.85	252.31	RW2
Chad	ZONE 4	1986	191.56	148.36	243.04	RW2
Chad	ZONE 4	1987	186.88	146.83	234.40	RW2
Chad	ZONE 4	1988	182.25	143.74	227.78	RW2
Chad	ZONE 4	1989	177.70	140.64	221.62	RW2
Chad	ZONE 4	1990	173.20	138.67	214.43	RW2
Chad	ZONE 4	1991	168.77	136.88	206.62	RW2
Chad	ZONE 4	1992	164.24	134.06	199.58	RW2
Chad	ZONE 4	1993	159.55	130.57	193.65	RW2
Chad	ZONE 4	1994	154.81	126.09	188.99	RW2
Chad	ZONE 4	1995	150.01	122.78	181.91	RW2
Chad	ZONE 4	1996	145.36	120.30	174.54	RW2
Chad	ZONE 4	1997	140.75	117.44	167.85	RW2
Chad	ZONE 4	1998	136.24	113.46	163.47	RW2
Chad	ZONE 4	1999	131.92	108.72	159.69	RW2
Chad	ZONE 4	2000	127.74	105.06	154.04	RW2
Chad	ZONE 4	2001	123.93	103.33	148.01	RW2
Chad	ZONE 4	2002	120.45	100.88	143.18	RW2
Chad	ZONE 4	2003	117.12	97.57	139.95	RW2
Chad	ZONE 4	2004	114.15	93.97	137.60	RW2
Chad	ZONE 4	2005	111.54	91.45	135.36	RW2
Chad	ZONE 4	2006	108.83	89.97	130.99	RW2
Chad	ZONE 4	2007	106.17	88.07	127.19	RW2
Chad	ZONE 4	2008	103.60	85.15	125.34	RW2
Chad	ZONE 4	2009	100.92	81.60	124.33	RW2
Chad	ZONE 4	2010	98.34	77.70	123.31	RW2
Chad	ZONE 4	2011	95.69	76.01	119.91	RW2
Chad	ZONE 4	2012	93.25	74.01	117.02	RW2
Chad	ZONE 4	2013	90.72	69.80	117.76	RW2
Chad	ZONE 4	2014	88.34	62.60	123.47	RW2
Chad	ZONE 4	2015	86.07	52.87	138.43	RW2
Chad	ZONE 4	2016	83.77	44.00	155.59	RW2
Chad	ZONE 4	2017	81.62	35.46	179.21	RW2
Chad	ZONE 4	2018	79.25	27.62	207.57	RW2
Chad	ZONE 4	2019	77.06	21.63	242.00	RW2
Chad	ZONE 5	1980	191.26	128.15	274.00	RW2
Chad	ZONE 5	1981	190.23	136.70	257.29	RW2
Chad	ZONE 5	1982	189.23	141.12	249.39	RW2
Chad	ZONE 5	1983	188.42	142.02	246.11	RW2
Chad	ZONE 5	1984	187.61	141.41	243.65	RW2
Chad	ZONE 5	1985	186.56	143.24	239.79	RW2
Chad	ZONE 5	1986	185.56	144.59	234.96	RW2
Chad	ZONE 5	1987	184.51	145.81	230.85	RW2
Chad	ZONE 5	1988	183.32	145.48	227.98	RW2
Chad	ZONE 5	1989	181.71	145.22	225.78	RW2
Chad	ZONE 5	1990	180.38	145.60	221.11	RW2
Chad	ZONE 5	1991	178.54	145.84	216.76	RW2
Chad	ZONE 5	1992	176.55	145.60	212.28	RW2
Chad	ZONE 5	1993	174.14	143.27	210.21	RW2
Chad	ZONE 5	1994	171.58	140.55	207.93	RW2
Chad	ZONE 5	1995	168.83	138.62	203.68	RW2
Chad	ZONE 5	1996	166.28	138.54	198.88	RW2
Chad	ZONE 5	1997	163.83	136.88	194.46	RW2
Chad	ZONE 5	1998	161.27	134.51	192.36	RW2
Chad	ZONE 5	1999	159.09	131.37	190.75	RW2
Chad	ZONE 5	2000	157.05	129.97	188.43	RW2
Chad	ZONE 5	2001	155.23	129.99	184.50	RW2
Chad	ZONE 5	2002	153.77	129.51	181.91	RW2
Chad	ZONE 5	2003	152.45	127.52	180.68	RW2

Continued on next page

Country	Region	Year	Median	Lower	Upper	Method
Chad	ZONE 5	2004	151.12	124.77	181.35	RW2
Chad	ZONE 5	2005	150.11	123.85	181.18	RW2
Chad	ZONE 5	2006	148.61	123.29	177.97	RW2
Chad	ZONE 5	2007	146.95	122.30	175.90	RW2
Chad	ZONE 5	2008	144.86	119.41	175.21	RW2
Chad	ZONE 5	2009	142.69	115.14	175.48	RW2
Chad	ZONE 5	2010	140.04	111.49	174.52	RW2
Chad	ZONE 5	2011	137.52	110.04	170.10	RW2
Chad	ZONE 5	2012	134.87	108.89	166.48	RW2
Chad	ZONE 5	2013	132.18	104.11	166.22	RW2
Chad	ZONE 5	2014	129.53	95.04	174.69	RW2
Chad	ZONE 5	2015	127.13	80.65	194.80	RW2
Chad	ZONE 5	2016	124.38	68.28	216.88	RW2
Chad	ZONE 5	2017	121.82	55.19	246.77	RW2
Chad	ZONE 5	2018	119.51	44.28	281.69	RW2
Chad	ZONE 5	2019	117.18	34.50	322.77	RW2
Chad	ZONE 6	1980	176.59	116.13	264.08	RW2
Chad	ZONE 6	1981	175.82	124.90	246.43	RW2
Chad	ZONE 6	1982	174.98	128.43	236.59	RW2
Chad	ZONE 6	1983	174.40	130.20	232.01	RW2
Chad	ZONE 6	1984	173.69	130.49	228.94	RW2
Chad	ZONE 6	1985	172.99	132.36	223.92	RW2
Chad	ZONE 6	1986	172.15	133.98	218.43	RW2
Chad	ZONE 6	1987	171.26	135.38	214.72	RW2
Chad	ZONE 6	1988	170.24	135.66	211.69	RW2
Chad	ZONE 6	1989	169.21	135.09	209.67	RW2
Chad	ZONE 6	1990	168.19	135.59	206.82	RW2
Chad	ZONE 6	1991	166.95	136.34	203.39	RW2
Chad	ZONE 6	1992	165.47	135.98	199.34	RW2
Chad	ZONE 6	1993	163.84	134.46	197.66	RW2
Chad	ZONE 6	1994	162.20	132.22	196.92	RW2
Chad	ZONE 6	1995	160.66	131.57	193.78	RW2
Chad	ZONE 6	1996	159.32	131.88	189.87	RW2
Chad	ZONE 6	1997	158.42	132.22	187.22	RW2
Chad	ZONE 6	1998	157.67	131.55	186.95	RW2
Chad	ZONE 6	1999	157.45	130.25	188.74	RW2
Chad	ZONE 6	2000	157.56	130.90	187.84	RW2
Chad	ZONE 6	2001	158.06	133.21	185.86	RW2
Chad	ZONE 6	2002	158.93	135.01	185.89	RW2
Chad	ZONE 6	2003	159.92	135.53	187.45	RW2
Chad	ZONE 6	2004	161.14	135.14	190.68	RW2
Chad	ZONE 6	2005	162.34	135.97	192.52	RW2
Chad	ZONE 6	2006	163.17	138.10	191.88	RW2
Chad	ZONE 6	2007	163.35	138.63	191.58	RW2
Chad	ZONE 6	2008	163.11	137.31	192.72	RW2
Chad	ZONE 6	2009	162.65	134.43	195.67	RW2
Chad	ZONE 6	2010	161.37	130.64	196.84	RW2
Chad	ZONE 6	2011	160.04	130.72	194.78	RW2
Chad	ZONE 6	2012	158.57	129.68	193.01	RW2
Chad	ZONE 6	2013	157.06	124.84	195.65	RW2
Chad	ZONE 6	2014	155.69	114.38	207.71	RW2
Chad	ZONE 6	2015	154.16	99.26	232.97	RW2
Chad	ZONE 6	2016	152.58	83.76	260.86	RW2
Chad	ZONE 6	2017	151.37	69.44	296.71	RW2
Chad	ZONE 6	2018	149.15	56.32	341.17	RW2
Chad	ZONE 6	2019	147.70	44.47	390.61	RW2
Chad	ZONE 7	1980	233.44	158.75	325.25	RW2
Chad	ZONE 7	1981	236.12	172.75	309.26	RW2
Chad	ZONE 7	1982	238.63	181.59	302.95	RW2
Chad	ZONE 7	1983	241.06	186.34	303.75	RW2
Chad	ZONE 7	1984	243.40	189.39	305.30	RW2
Chad	ZONE 7	1985	245.86	194.57	304.26	RW2
Chad	ZONE 7	1986	248.27	199.55	302.42	RW2

Continued on next page

Country	Region	Year	Median	Lower	Upper	Method
Chad	ZONE 7	1987	250.23	204.23	301.39	RW2
Chad	ZONE 7	1988	252.55	207.32	302.48	RW2
Chad	ZONE 7	1989	254.41	209.71	304.24	RW2
Chad	ZONE 7	1990	256.14	213.74	303.26	RW2
Chad	ZONE 7	1991	257.27	217.66	301.62	RW2
Chad	ZONE 7	1992	258.04	220.01	300.01	RW2
Chad	ZONE 7	1993	258.39	220.53	300.34	RW2
Chad	ZONE 7	1994	258.14	219.16	301.49	RW2
Chad	ZONE 7	1995	257.50	219.43	299.04	RW2
Chad	ZONE 7	1996	256.68	221.28	294.91	RW2
Chad	ZONE 7	1997	255.62	221.60	292.96	RW2
Chad	ZONE 7	1998	254.45	220.22	292.81	RW2
Chad	ZONE 7	1999	253.25	216.43	294.40	RW2
Chad	ZONE 7	2000	251.75	214.67	292.64	RW2
Chad	ZONE 7	2001	250.50	215.66	289.53	RW2
Chad	ZONE 7	2002	249.03	215.29	286.69	RW2
Chad	ZONE 7	2003	247.92	212.80	287.71	RW2
Chad	ZONE 7	2004	246.22	208.73	288.38	RW2
Chad	ZONE 7	2005	244.59	207.25	286.91	RW2
Chad	ZONE 7	2006	242.09	206.54	282.00	RW2
Chad	ZONE 7	2007	239.05	204.33	277.49	RW2
Chad	ZONE 7	2008	235.10	199.46	274.90	RW2
Chad	ZONE 7	2009	230.52	192.68	273.33	RW2
Chad	ZONE 7	2010	225.05	185.75	269.93	RW2
Chad	ZONE 7	2011	219.84	182.14	260.98	RW2
Chad	ZONE 7	2012	214.21	179.42	253.46	RW2
Chad	ZONE 7	2013	208.73	171.14	251.85	RW2
Chad	ZONE 7	2014	203.10	154.20	261.75	RW2
Chad	ZONE 7	2015	197.98	131.11	287.61	RW2
Chad	ZONE 7	2016	193.00	109.78	315.94	RW2
Chad	ZONE 7	2017	187.49	89.22	349.28	RW2
Chad	ZONE 7	2018	181.99	71.40	390.03	RW2
Chad	ZONE 7	2019	177.34	55.59	441.25	RW2
Chad	ZONE 8	1980	263.83	182.12	363.60	RW2
Chad	ZONE 8	1981	262.60	194.80	341.96	RW2
Chad	ZONE 8	1982	261.29	200.50	331.11	RW2
Chad	ZONE 8	1983	259.67	202.38	325.72	RW2
Chad	ZONE 8	1984	258.23	202.50	322.80	RW2
Chad	ZONE 8	1985	256.99	204.37	316.14	RW2
Chad	ZONE 8	1986	255.21	206.72	309.94	RW2
Chad	ZONE 8	1987	253.46	207.53	305.20	RW2
Chad	ZONE 8	1988	251.74	206.75	302.40	RW2
Chad	ZONE 8	1989	249.74	204.82	300.01	RW2
Chad	ZONE 8	1990	247.50	204.98	296.50	RW2
Chad	ZONE 8	1991	245.07	204.24	291.09	RW2
Chad	ZONE 8	1992	242.08	203.11	285.79	RW2
Chad	ZONE 8	1993	238.89	200.42	282.43	RW2
Chad	ZONE 8	1994	235.28	196.39	279.30	RW2
Chad	ZONE 8	1995	231.39	194.63	272.92	RW2
Chad	ZONE 8	1996	227.51	193.42	265.75	RW2
Chad	ZONE 8	1997	223.71	191.45	259.92	RW2
Chad	ZONE 8	1998	219.90	186.93	256.67	RW2
Chad	ZONE 8	1999	216.04	182.04	254.21	RW2
Chad	ZONE 8	2000	212.40	179.19	250.02	RW2
Chad	ZONE 8	2001	208.88	177.47	244.44	RW2
Chad	ZONE 8	2002	205.68	175.89	239.68	RW2
Chad	ZONE 8	2003	202.32	171.72	236.68	RW2
Chad	ZONE 8	2004	199.07	167.52	234.87	RW2
Chad	ZONE 8	2005	195.77	164.68	231.44	RW2
Chad	ZONE 8	2006	192.03	163.22	224.90	RW2
Chad	ZONE 8	2007	187.81	160.08	219.14	RW2
Chad	ZONE 8	2008	183.17	154.66	215.44	RW2
Chad	ZONE 8	2009	178.13	147.66	213.17	RW2

Continued on next page

Country	Region	Year	Median	Lower	Upper	Method
Chad	ZONE 8	2010	172.49	140.57	209.64	RW2
Chad	ZONE 8	2011	167.15	136.65	202.39	RW2
Chad	ZONE 8	2012	161.63	132.84	195.02	RW2
Chad	ZONE 8	2013	156.24	125.01	193.52	RW2
Chad	ZONE 8	2014	150.91	111.82	200.03	RW2
Chad	ZONE 8	2015	145.91	93.74	219.43	RW2
Chad	ZONE 8	2016	140.76	77.11	241.77	RW2
Chad	ZONE 8	2017	136.02	61.98	273.92	RW2
Chad	ZONE 8	2018	131.39	48.70	307.44	RW2
Chad	ZONE 8	2019	127.03	38.01	346.53	RW2
Comoros	ALL	1980	165.11	155.04	175.33	IHME
Comoros	ALL	1980	177.18	111.68	269.23	RW2
Comoros	ALL	1980	175.30	157.10	197.90	UN
Comoros	ALL	1981	160.33	150.36	170.52	IHME
Comoros	ALL	1981	170.59	121.83	232.97	RW2
Comoros	ALL	1981	169.40	151.80	191.20	UN
Comoros	ALL	1982	155.16	145.18	165.51	IHME
Comoros	ALL	1982	164.25	122.01	217.64	RW2
Comoros	ALL	1982	163.60	146.30	184.30	UN
Comoros	ALL	1983	150.29	140.39	160.46	IHME
Comoros	ALL	1983	157.82	114.43	213.88	RW2
Comoros	ALL	1983	157.90	140.90	177.70	UN
Comoros	ALL	1984	144.93	135.22	154.46	IHME
Comoros	ALL	1984	152.15	106.46	212.21	RW2
Comoros	ALL	1984	152.40	135.40	171.40	UN
Comoros	ALL	1985	140.05	130.92	149.56	IHME
Comoros	ALL	1985	146.13	103.77	201.39	RW2
Comoros	ALL	1985	147.20	130.70	165.40	UN
Comoros	ALL	1986	135.34	126.46	144.77	IHME
Comoros	ALL	1986	140.95	101.63	191.59	RW2
Comoros	ALL	1986	142.20	126.10	159.60	UN
Comoros	ALL	1987	131.37	122.67	140.81	IHME
Comoros	ALL	1987	136.31	99.64	184.06	RW2
Comoros	ALL	1987	137.60	122.00	154.30	UN
Comoros	ALL	1988	127.32	119.08	136.23	IHME
Comoros	ALL	1988	131.92	95.20	179.34	RW2
Comoros	ALL	1988	133.20	118.40	149.30	UN
Comoros	ALL	1989	123.98	116.13	132.47	IHME
Comoros	ALL	1989	127.93	91.27	175.98	RW2
Comoros	ALL	1989	129.10	114.70	144.20	UN
Comoros	ALL	1990	120.20	112.10	128.60	IHME
Comoros	ALL	1990	124.46	89.14	172.18	RW2
Comoros	ALL	1990	125.10	111.30	139.70	UN
Comoros	ALL	1991	116.70	108.59	125.21	IHME
Comoros	ALL	1991	120.98	87.83	164.18	RW2
Comoros	ALL	1991	121.20	107.50	135.50	UN
Comoros	ALL	1992	113.00	104.84	121.89	IHME
Comoros	ALL	1992	117.61	85.81	158.84	RW2
Comoros	ALL	1992	117.20	103.50	131.70	UN
Comoros	ALL	1993	109.36	101.34	118.52	IHME
Comoros	ALL	1993	114.38	82.53	156.58	RW2
Comoros	ALL	1993	113.60	99.00	128.30	UN
Comoros	ALL	1994	106.08	97.59	116.15	IHME
Comoros	ALL	1994	111.16	77.78	157.41	RW2
Comoros	ALL	1994	110.50	94.70	125.40	UN
Comoros	ALL	1995	102.70	93.77	113.09	IHME
Comoros	ALL	1995	108.10	72.48	157.29	RW2
Comoros	ALL	1995	107.80	90.90	123.00	UN
Comoros	ALL	1996	99.82	89.87	111.18	IHME
Comoros	ALL	1996	105.21	69.07	157.03	RW2
Comoros	ALL	1996	105.60	88.00	121.00	UN
Comoros	ALL	1997	96.14	84.77	108.43	IHME
Comoros	ALL	1997	102.61	65.46	156.83	RW2

Continued on next page

Country	Region	Year	Median	Lower	Upper	Method
Comoros	ALL	1997	104.00	85.70	119.70	UN
Comoros	ALL	1998	92.70	80.14	105.80	IHME
Comoros	ALL	1998	100.48	60.68	162.11	RW2
Comoros	ALL	1998	102.70	83.80	118.90	UN
Comoros	ALL	1999	88.89	75.51	102.87	IHME
Comoros	ALL	1999	98.53	54.86	168.71	RW2
Comoros	ALL	1999	101.80	82.40	118.70	UN
Comoros	ALL	2000	85.41	71.38	99.61	IHME
Comoros	ALL	2000	96.87	48.06	183.93	RW2
Comoros	ALL	2000	101.10	80.90	119.10	UN
Comoros	ALL	2001	81.91	67.26	96.94	IHME
Comoros	ALL	2001	95.43	42.56	198.23	RW2
Comoros	ALL	2001	100.80	78.70	119.80	UN
Comoros	ALL	2002	78.46	63.37	94.41	IHME
Comoros	ALL	2002	94.07	38.55	210.67	RW2
Comoros	ALL	2002	100.80	76.40	121.80	UN
Comoros	ALL	2003	75.05	59.58	92.22	IHME
Comoros	ALL	2003	93.24	35.83	219.85	RW2
Comoros	ALL	2003	100.40	73.70	125.20	UN
Comoros	ALL	2004	71.85	56.39	90.08	IHME
Comoros	ALL	2004	91.74	33.22	226.59	RW2
Comoros	ALL	2004	99.40	70.90	129.60	UN
Comoros	ALL	2005	68.79	53.04	87.68	IHME
Comoros	ALL	2005	91.20	32.09	232.02	RW2
Comoros	ALL	2005	97.80	68.10	133.80	UN
Comoros	ALL	2006	65.81	49.97	84.90	IHME
Comoros	ALL	2006	90.20	31.53	230.59	RW2
Comoros	ALL	2006	95.70	65.30	137.00	UN
Comoros	ALL	2007	63.01	47.33	82.07	IHME
Comoros	ALL	2007	89.52	32.31	224.22	RW2
Comoros	ALL	2007	93.50	62.20	139.00	UN
Comoros	ALL	2008	60.12	44.66	79.21	IHME
Comoros	ALL	2008	89.47	34.07	215.11	RW2
Comoros	ALL	2008	91.20	59.30	139.90	UN
Comoros	ALL	2009	57.91	42.55	77.15	IHME
Comoros	ALL	2009	88.58	36.50	201.76	RW2
Comoros	ALL	2009	88.50	56.10	141.90	UN
Comoros	ALL	2010	55.06	39.80	74.18	IHME
Comoros	ALL	2010	88.09	40.35	184.39	RW2
Comoros	ALL	2010	86.00	52.80	142.10	UN
Comoros	ALL	2011	52.94	38.06	72.08	IHME
Comoros	ALL	2011	87.87	42.18	174.46	RW2
Comoros	ALL	2011	83.30	50.20	143.30	UN
Comoros	ALL	2012	50.48	35.91	69.24	IHME
Comoros	ALL	2012	87.42	41.62	174.49	RW2
Comoros	ALL	2012	80.90	47.30	143.30	UN
Comoros	ALL	2013	48.25	34.33	66.85	IHME
Comoros	ALL	2013	87.23	37.71	188.52	RW2
Comoros	ALL	2013	78.30	44.50	143.90	UN
Comoros	ALL	2014	46.05	32.70	63.80	IHME
Comoros	ALL	2014	86.71	31.01	220.31	RW2
Comoros	ALL	2014	75.90	42.00	144.10	UN
Comoros	ALL	2015	44.02	30.70	61.97	IHME
Comoros	ALL	2015	85.96	23.35	272.72	RW2
Comoros	ALL	2015	73.50	39.40	144.60	UN
Comoros	ALL	2016	85.99	17.19	343.20	RW2
Comoros	ALL	2017	85.11	11.90	428.65	RW2
Comoros	ALL	2018	84.61	8.19	530.50	RW2
Comoros	ALL	2019	83.91	5.05	624.13	RW2
Comoros	ALL	15-19	85.10	12.19	420.77	RW2
Comoros	MOHELI	1980	187.03	103.42	317.17	RW2
Comoros	MOHELI	1981	177.08	106.57	280.26	RW2
Comoros	MOHELI	1982	167.66	105.57	256.48	RW2

Continued on next page

Country	Region	Year	Median	Lower	Upper	Method
Comoros	MOHELI	1983	158.60	101.26	240.42	RW2
Comoros	MOHELI	1984	150.39	96.36	227.24	RW2
Comoros	MOHELI	1985	142.06	92.40	211.72	RW2
Comoros	MOHELI	1986	135.28	89.66	198.05	RW2
Comoros	MOHELI	1987	128.77	86.33	188.12	RW2
Comoros	MOHELI	1988	123.50	82.48	179.66	RW2
Comoros	MOHELI	1989	118.76	79.20	173.82	RW2
Comoros	MOHELI	1990	114.82	76.43	168.73	RW2
Comoros	MOHELI	1991	110.97	73.94	163.36	RW2
Comoros	MOHELI	1992	107.39	71.08	158.42	RW2
Comoros	MOHELI	1993	104.10	68.05	155.71	RW2
Comoros	MOHELI	1994	101.05	64.14	155.54	RW2
Comoros	MOHELI	1995	97.80	60.28	154.22	RW2
Comoros	MOHELI	1996	95.20	57.15	154.24	RW2
Comoros	MOHELI	1997	92.69	53.81	154.83	RW2
Comoros	MOHELI	1998	90.49	49.83	158.32	RW2
Comoros	MOHELI	1999	88.60	45.53	165.70	RW2
Comoros	MOHELI	2000	86.60	40.39	175.52	RW2
Comoros	MOHELI	2001	85.35	36.97	186.02	RW2
Comoros	MOHELI	2002	84.33	33.15	196.68	RW2
Comoros	MOHELI	2003	83.04	30.16	205.56	RW2
Comoros	MOHELI	2004	82.16	28.12	214.58	RW2
Comoros	MOHELI	2005	81.26	26.55	220.72	RW2
Comoros	MOHELI	2006	80.52	25.42	224.90	RW2
Comoros	MOHELI	2007	79.75	25.04	230.59	RW2
Comoros	MOHELI	2008	79.36	24.47	227.34	RW2
Comoros	MOHELI	2009	78.61	24.50	227.01	RW2
Comoros	MOHELI	2010	78.47	24.36	225.34	RW2
Comoros	MOHELI	2011	77.84	23.68	229.60	RW2
Comoros	MOHELI	2012	77.56	22.60	238.69	RW2
Comoros	MOHELI	2013	77.14	20.04	254.34	RW2
Comoros	MOHELI	2014	76.51	17.09	286.40	RW2
Comoros	MOHELI	2015	76.31	13.96	330.40	RW2
Comoros	MOHELI	2016	75.70	11.00	381.33	RW2
Comoros	MOHELI	2017	75.05	8.33	447.43	RW2
Comoros	MOHELI	2018	74.71	6.07	526.09	RW2
Comoros	MOHELI	2019	73.95	4.34	609.49	RW2
Comoros	NDZOUANI	1980	206.07	132.09	306.67	RW2
Comoros	NDZOUANI	1981	195.74	139.31	267.28	RW2
Comoros	NDZOUANI	1982	185.62	138.08	244.94	RW2
Comoros	NDZOUANI	1983	175.53	131.31	231.73	RW2
Comoros	NDZOUANI	1984	166.64	123.78	220.65	RW2
Comoros	NDZOUANI	1985	157.94	118.46	207.18	RW2
Comoros	NDZOUANI	1986	150.30	114.60	194.32	RW2
Comoros	NDZOUANI	1987	143.62	110.40	184.33	RW2
Comoros	NDZOUANI	1988	137.55	105.40	176.62	RW2
Comoros	NDZOUANI	1989	132.31	100.55	171.65	RW2
Comoros	NDZOUANI	1990	127.64	96.78	166.33	RW2
Comoros	NDZOUANI	1991	123.21	94.38	159.73	RW2
Comoros	NDZOUANI	1992	119.18	90.99	154.67	RW2
Comoros	NDZOUANI	1993	115.17	85.91	152.89	RW2
Comoros	NDZOUANI	1994	111.47	80.37	152.72	RW2
Comoros	NDZOUANI	1995	107.64	74.70	152.74	RW2
Comoros	NDZOUANI	1996	104.36	69.87	152.35	RW2
Comoros	NDZOUANI	1997	101.27	65.30	153.65	RW2
Comoros	NDZOUANI	1998	98.59	60.08	157.14	RW2
Comoros	NDZOUANI	1999	95.71	53.91	163.40	RW2
Comoros	NDZOUANI	2000	93.71	48.08	172.37	RW2
Comoros	NDZOUANI	2001	91.62	43.29	181.81	RW2
Comoros	NDZOUANI	2002	90.09	39.51	191.46	RW2
Comoros	NDZOUANI	2003	88.33	35.85	198.15	RW2
Comoros	NDZOUANI	2004	87.04	33.58	205.86	RW2
Comoros	NDZOUANI	2005	85.46	32.14	207.76	RW2

Continued on next page

Country	Region	Year	Median	Lower	Upper	Method
Comoros	NDZOUANI	2006	84.28	31.03	207.08	RW2
Comoros	NDZOUANI	2007	83.24	31.13	202.34	RW2
Comoros	NDZOUANI	2008	81.92	31.69	197.06	RW2
Comoros	NDZOUANI	2009	81.05	32.33	188.27	RW2
Comoros	NDZOUANI	2010	80.08	33.09	180.90	RW2
Comoros	NDZOUANI	2011	79.31	33.56	177.27	RW2
Comoros	NDZOUANI	2012	78.46	32.27	178.49	RW2
Comoros	NDZOUANI	2013	77.56	29.23	190.53	RW2
Comoros	NDZOUANI	2014	76.66	24.83	213.89	RW2
Comoros	NDZOUANI	2015	75.93	19.62	252.42	RW2
Comoros	NDZOUANI	2016	74.97	15.06	301.74	RW2
Comoros	NDZOUANI	2017	73.91	11.26	362.51	RW2
Comoros	NDZOUANI	2018	73.16	8.14	439.48	RW2
Comoros	NDZOUANI	2019	72.39	5.59	524.74	RW2
Comoros	NGAZIDJA	1980	157.54	106.70	227.81	RW2
Comoros	NGAZIDJA	1981	152.02	115.87	197.55	RW2
Comoros	NGAZIDJA	1982	146.52	115.49	184.27	RW2
Comoros	NGAZIDJA	1983	141.15	109.16	181.30	RW2
Comoros	NGAZIDJA	1984	136.09	102.32	178.01	RW2
Comoros	NGAZIDJA	1985	131.38	98.96	172.12	RW2
Comoros	NGAZIDJA	1986	127.41	96.70	165.33	RW2
Comoros	NGAZIDJA	1987	123.90	95.52	159.37	RW2
Comoros	NGAZIDJA	1988	121.13	92.68	156.49	RW2
Comoros	NGAZIDJA	1989	118.67	89.76	154.36	RW2
Comoros	NGAZIDJA	1990	116.80	88.54	152.62	RW2
Comoros	NGAZIDJA	1991	114.98	88.30	148.82	RW2
Comoros	NGAZIDJA	1992	113.39	87.35	146.45	RW2
Comoros	NGAZIDJA	1993	111.87	84.57	146.62	RW2
Comoros	NGAZIDJA	1994	110.27	80.89	149.22	RW2
Comoros	NGAZIDJA	1995	108.75	76.57	152.39	RW2
Comoros	NGAZIDJA	1996	107.46	73.67	154.41	RW2
Comoros	NGAZIDJA	1997	106.19	70.32	157.25	RW2
Comoros	NGAZIDJA	1998	105.19	65.86	163.38	RW2
Comoros	NGAZIDJA	1999	104.43	60.98	172.63	RW2
Comoros	NGAZIDJA	2000	103.70	55.25	186.42	RW2
Comoros	NGAZIDJA	2001	103.55	50.65	198.91	RW2
Comoros	NGAZIDJA	2002	102.73	46.49	211.03	RW2
Comoros	NGAZIDJA	2003	102.73	43.50	223.51	RW2
Comoros	NGAZIDJA	2004	102.45	41.55	232.50	RW2
Comoros	NGAZIDJA	2005	102.41	39.88	237.49	RW2
Comoros	NGAZIDJA	2006	102.78	39.66	237.21	RW2
Comoros	NGAZIDJA	2007	102.79	40.43	236.55	RW2
Comoros	NGAZIDJA	2008	102.74	41.62	233.14	RW2
Comoros	NGAZIDJA	2009	103.28	42.95	226.94	RW2
Comoros	NGAZIDJA	2010	103.47	45.26	218.75	RW2
Comoros	NGAZIDJA	2011	104.00	46.47	215.97	RW2
Comoros	NGAZIDJA	2012	104.46	45.20	221.54	RW2
Comoros	NGAZIDJA	2013	104.97	41.58	240.35	RW2
Comoros	NGAZIDJA	2014	104.54	35.42	270.39	RW2
Comoros	NGAZIDJA	2015	105.30	28.83	317.78	RW2
Comoros	NGAZIDJA	2016	105.67	22.22	383.84	RW2
Comoros	NGAZIDJA	2017	105.64	16.78	454.35	RW2
Comoros	NGAZIDJA	2018	106.48	12.24	536.06	RW2
Comoros	NGAZIDJA	2019	106.63	8.44	627.24	RW2
Congo	ALL	1980	121.14	105.35	137.76	IHME
Congo	ALL	1980	112.71	71.97	172.45	RW2
Congo	ALL	1980	111.90	89.00	142.90	UN
Congo	ALL	1981	116.44	103.66	129.86	IHME
Congo	ALL	1981	109.60	78.12	151.55	RW2
Congo	ALL	1981	109.10	88.50	135.70	UN
Congo	ALL	1982	111.97	102.21	123.06	IHME
Congo	ALL	1982	106.64	80.18	140.59	RW2
Congo	ALL	1982	106.20	87.90	128.80	UN

Continued on next page

Country	Region	Year	Median	Lower	Upper	Method
Congo	ALL	1983	107.73	99.41	116.90	IHME
Congo	ALL	1983	103.55	78.28	136.25	RW2
Congo	ALL	1983	103.20	86.80	123.30	UN
Congo	ALL	1984	103.61	95.89	111.62	IHME
Congo	ALL	1984	100.98	75.39	134.22	RW2
Congo	ALL	1984	100.20	85.60	118.10	UN
Congo	ALL	1985	100.42	93.09	107.69	IHME
Congo	ALL	1985	98.24	74.77	127.99	RW2
Congo	ALL	1985	97.50	84.00	113.80	UN
Congo	ALL	1986	97.87	91.00	104.95	IHME
Congo	ALL	1986	96.19	74.42	123.35	RW2
Congo	ALL	1986	95.30	82.60	110.40	UN
Congo	ALL	1987	95.92	89.35	102.92	IHME
Congo	ALL	1987	94.66	74.20	120.49	RW2
Congo	ALL	1987	93.70	81.60	107.80	UN
Congo	ALL	1988	94.43	88.36	101.16	IHME
Congo	ALL	1988	93.59	72.89	119.72	RW2
Congo	ALL	1988	92.80	81.10	106.10	UN
Congo	ALL	1989	93.44	87.32	99.89	IHME
Congo	ALL	1989	93.21	72.09	120.26	RW2
Congo	ALL	1989	92.80	81.30	105.80	UN
Congo	ALL	1990	93.35	87.29	99.37	IHME
Congo	ALL	1990	93.39	72.21	119.58	RW2
Congo	ALL	1990	93.60	82.40	106.40	UN
Congo	ALL	1991	93.82	87.70	99.83	IHME
Congo	ALL	1991	94.68	74.18	119.37	RW2
Congo	ALL	1991	95.20	84.00	107.60	UN
Congo	ALL	1992	94.67	88.50	100.78	IHME
Congo	ALL	1992	96.91	76.38	121.51	RW2
Congo	ALL	1992	97.40	86.50	109.70	UN
Congo	ALL	1993	96.37	90.11	102.51	IHME
Congo	ALL	1993	100.08	78.54	125.90	RW2
Congo	ALL	1993	100.20	89.40	112.30	UN
Congo	ALL	1994	98.31	91.74	105.07	IHME
Congo	ALL	1994	103.94	80.68	132.10	RW2
Congo	ALL	1994	103.80	93.00	115.80	UN
Congo	ALL	1995	100.37	93.35	107.96	IHME
Congo	ALL	1995	108.81	85.97	138.08	RW2
Congo	ALL	1995	107.70	96.70	119.90	UN
Congo	ALL	1996	102.79	95.19	111.15	IHME
Congo	ALL	1996	113.16	90.58	141.83	RW2
Congo	ALL	1996	111.90	100.70	124.20	UN
Congo	ALL	1997	115.77	103.91	129.70	IHME
Congo	ALL	1997	117.11	94.24	144.75	RW2
Congo	ALL	1997	116.00	104.60	128.50	UN
Congo	ALL	1998	110.97	102.53	120.36	IHME
Congo	ALL	1998	120.21	95.84	149.66	RW2
Congo	ALL	1998	119.30	107.80	132.00	UN
Congo	ALL	1999	109.84	101.56	118.87	IHME
Congo	ALL	1999	121.85	95.73	152.35	RW2
Congo	ALL	1999	121.40	109.60	134.40	UN
Congo	ALL	2000	109.38	101.70	118.22	IHME
Congo	ALL	2000	122.08	96.75	153.08	RW2
Congo	ALL	2000	121.60	109.80	134.80	UN
Congo	ALL	2001	107.56	100.29	115.81	IHME
Congo	ALL	2001	119.94	95.91	148.87	RW2
Congo	ALL	2001	119.70	108.00	132.50	UN
Congo	ALL	2002	104.95	98.62	111.97	IHME
Congo	ALL	2002	115.69	93.30	142.85	RW2
Congo	ALL	2002	115.60	104.60	127.90	UN
Congo	ALL	2003	101.02	95.14	107.61	IHME
Congo	ALL	2003	109.72	88.21	136.62	RW2
Congo	ALL	2003	109.80	99.30	121.40	UN

Continued on next page

Country	Region	Year	Median	Lower	Upper	Method
Congo	ALL	2004	95.77	90.00	102.06	IHME
Congo	ALL	2004	102.32	80.71	130.06	RW2
Congo	ALL	2004	102.90	92.80	113.90	UN
Congo	ALL	2005	90.04	84.01	96.37	IHME
Congo	ALL	2005	93.82	72.11	119.04	RW2
Congo	ALL	2005	95.30	85.30	105.90	UN
Congo	ALL	2006	84.32	78.20	90.52	IHME
Congo	ALL	2006	86.09	66.78	108.82	RW2
Congo	ALL	2006	87.40	77.80	98.10	UN
Congo	ALL	2007	79.33	73.07	85.46	IHME
Congo	ALL	2007	78.97	61.71	99.83	RW2
Congo	ALL	2007	79.90	70.30	90.50	UN
Congo	ALL	2008	74.72	68.71	80.79	IHME
Congo	ALL	2008	72.74	56.27	93.10	RW2
Congo	ALL	2008	72.60	62.90	83.80	UN
Congo	ALL	2009	71.09	64.96	77.65	IHME
Congo	ALL	2009	67.11	50.92	87.69	RW2
Congo	ALL	2009	66.10	55.80	78.10	UN
Congo	ALL	2010	67.99	61.02	75.35	IHME
Congo	ALL	2010	62.48	46.97	83.61	RW2
Congo	ALL	2010	60.60	49.50	73.90	UN
Congo	ALL	2011	65.07	57.13	73.58	IHME
Congo	ALL	2011	58.12	43.99	76.88	RW2
Congo	ALL	2011	56.10	44.20	70.80	UN
Congo	ALL	2012	62.80	53.72	72.67	IHME
Congo	ALL	2012	54.11	41.04	71.26	RW2
Congo	ALL	2012	52.60	39.40	69.00	UN
Congo	ALL	2013	60.16	49.94	71.68	IHME
Congo	ALL	2013	50.47	36.61	69.17	RW2
Congo	ALL	2013	49.60	35.60	67.80	UN
Congo	ALL	2014	58.15	47.10	71.48	IHME
Congo	ALL	2014	46.97	30.36	72.35	RW2
Congo	ALL	2014	47.10	32.30	67.00	UN
Congo	ALL	2015	56.13	44.35	70.14	IHME
Congo	ALL	2015	43.60	23.17	81.75	RW2
Congo	ALL	2015	45.00	29.60	66.50	UN
Congo	ALL	2016	40.66	17.60	93.89	RW2
Congo	ALL	2017	37.69	12.82	110.17	RW2
Congo	ALL	2018	35.01	9.23	132.76	RW2
Congo	ALL	2019	32.46	6.21	158.38	RW2
Congo	ALL	15-19	37.68	13.06	108.16	RW2
Congo	BRAZZAVILLE	1980	78.24	46.77	128.88	RW2
Congo	BRAZZAVILLE	1981	76.62	50.50	114.85	RW2
Congo	BRAZZAVILLE	1982	75.09	52.23	106.99	RW2
Congo	BRAZZAVILLE	1983	73.55	52.19	102.89	RW2
Congo	BRAZZAVILLE	1984	72.17	51.69	100.05	RW2
Congo	BRAZZAVILLE	1985	70.74	51.89	95.74	RW2
Congo	BRAZZAVILLE	1986	69.61	52.33	91.80	RW2
Congo	BRAZZAVILLE	1987	68.51	52.38	89.47	RW2
Congo	BRAZZAVILLE	1988	67.93	52.07	88.23	RW2
Congo	BRAZZAVILLE	1989	67.88	52.17	88.39	RW2
Congo	BRAZZAVILLE	1990	68.23	52.62	87.48	RW2
Congo	BRAZZAVILLE	1991	70.17	55.08	88.78	RW2
Congo	BRAZZAVILLE	1992	73.51	58.28	91.87	RW2
Congo	BRAZZAVILLE	1993	78.37	62.15	97.64	RW2
Congo	BRAZZAVILLE	1994	84.56	66.42	105.81	RW2
Congo	BRAZZAVILLE	1995	92.38	74.25	115.45	RW2
Congo	BRAZZAVILLE	1996	99.34	81.22	121.92	RW2
Congo	BRAZZAVILLE	1997	105.26	86.78	127.55	RW2
Congo	BRAZZAVILLE	1998	109.45	89.60	133.33	RW2
Congo	BRAZZAVILLE	1999	111.54	90.24	138.01	RW2
Congo	BRAZZAVILLE	2000	110.87	88.89	136.46	RW2
Congo	BRAZZAVILLE	2001	109.41	89.16	133.35	RW2

Continued on next page

Country	Region	Year	Median	Lower	Upper	Method
Congo	BRAZZAVILLE	2002	106.78	87.04	129.81	RW2
Congo	BRAZZAVILLE	2003	103.19	82.99	126.88	RW2
Congo	BRAZZAVILLE	2004	99.05	78.09	124.27	RW2
Congo	BRAZZAVILLE	2005	94.51	73.60	120.01	RW2
Congo	BRAZZAVILLE	2006	89.35	69.56	113.46	RW2
Congo	BRAZZAVILLE	2007	83.77	65.07	107.49	RW2
Congo	BRAZZAVILLE	2008	77.98	59.33	101.47	RW2
Congo	BRAZZAVILLE	2009	71.98	53.51	96.38	RW2
Congo	BRAZZAVILLE	2010	66.07	47.76	90.43	RW2
Congo	BRAZZAVILLE	2011	60.40	43.43	83.59	RW2
Congo	BRAZZAVILLE	2012	55.11	39.35	77.48	RW2
Congo	BRAZZAVILLE	2013	50.17	34.11	73.82	RW2
Congo	BRAZZAVILLE	2014	45.63	28.00	74.48	RW2
Congo	BRAZZAVILLE	2015	41.62	21.53	80.03	RW2
Congo	BRAZZAVILLE	2016	37.85	16.13	86.82	RW2
Congo	BRAZZAVILLE	2017	34.39	11.68	97.30	RW2
Congo	BRAZZAVILLE	2018	31.30	8.21	112.02	RW2
Congo	BRAZZAVILLE	2019	28.38	5.67	131.06	RW2
Congo	NORD	1980	132.78	84.06	204.92	RW2
Congo	NORD	1981	128.12	89.73	180.52	RW2
Congo	NORD	1982	123.71	91.07	165.78	RW2
Congo	NORD	1983	119.20	89.32	158.25	RW2
Congo	NORD	1984	115.09	86.75	151.69	RW2
Congo	NORD	1985	111.17	85.16	143.49	RW2
Congo	NORD	1986	107.35	84.29	136.38	RW2
Congo	NORD	1987	104.04	82.95	130.19	RW2
Congo	NORD	1988	101.21	80.76	126.84	RW2
Congo	NORD	1989	99.15	78.75	125.28	RW2
Congo	NORD	1990	97.71	77.52	121.76	RW2
Congo	NORD	1991	98.60	79.38	120.67	RW2
Congo	NORD	1992	101.14	82.22	122.88	RW2
Congo	NORD	1993	105.66	85.68	128.57	RW2
Congo	NORD	1994	111.84	89.51	136.72	RW2
Congo	NORD	1995	119.91	97.98	147.45	RW2
Congo	NORD	1996	126.75	105.36	152.12	RW2
Congo	NORD	1997	132.38	110.96	157.72	RW2
Congo	NORD	1998	136.02	113.42	162.76	RW2
Congo	NORD	1999	137.07	113.03	165.86	RW2
Congo	NORD	2000	135.15	111.07	162.73	RW2
Congo	NORD	2001	132.20	110.24	157.18	RW2
Congo	NORD	2002	127.79	107.69	151.22	RW2
Congo	NORD	2003	122.37	102.13	146.23	RW2
Congo	NORD	2004	116.21	95.04	141.06	RW2
Congo	NORD	2005	109.48	88.85	134.52	RW2
Congo	NORD	2006	101.91	83.62	124.48	RW2
Congo	NORD	2007	93.98	77.29	114.47	RW2
Congo	NORD	2008	85.74	69.41	105.80	RW2
Congo	NORD	2009	77.35	61.21	97.51	RW2
Congo	NORD	2010	69.20	53.52	88.84	RW2
Congo	NORD	2011	61.62	48.07	78.61	RW2
Congo	NORD	2012	54.66	42.74	69.47	RW2
Congo	NORD	2013	48.38	36.27	63.98	RW2
Congo	NORD	2014	42.72	28.52	62.79	RW2
Congo	NORD	2015	37.83	20.98	66.59	RW2
Congo	NORD	2016	33.36	15.00	71.17	RW2
Congo	NORD	2017	29.51	10.49	78.45	RW2
Congo	NORD	2018	25.94	7.16	87.65	RW2
Congo	NORD	2019	22.96	4.81	100.21	RW2
Congo	POINTE NOIRE	1980	119.63	71.60	192.52	RW2
Congo	POINTE NOIRE	1981	115.03	76.02	170.15	RW2
Congo	POINTE NOIRE	1982	110.52	76.78	156.90	RW2
Congo	POINTE NOIRE	1983	105.82	75.28	147.94	RW2
Congo	POINTE NOIRE	1984	101.75	73.12	140.47	RW2

Continued on next page

Country	Region	Year	Median	Lower	Upper	Method
Congo	POINTE NOIRE	1985	97.72	71.75	132.44	RW2
Congo	POINTE NOIRE	1986	93.89	70.45	124.68	RW2
Congo	POINTE NOIRE	1987	90.45	68.84	118.60	RW2
Congo	POINTE NOIRE	1988	87.35	66.92	113.85	RW2
Congo	POINTE NOIRE	1989	85.05	65.29	111.00	RW2
Congo	POINTE NOIRE	1990	83.06	63.92	106.78	RW2
Congo	POINTE NOIRE	1991	83.01	65.17	105.20	RW2
Congo	POINTE NOIRE	1992	84.54	66.98	105.95	RW2
Congo	POINTE NOIRE	1993	87.47	68.91	109.68	RW2
Congo	POINTE NOIRE	1994	91.70	71.69	115.08	RW2
Congo	POINTE NOIRE	1995	97.43	77.93	122.26	RW2
Congo	POINTE NOIRE	1996	102.07	82.65	125.66	RW2
Congo	POINTE NOIRE	1997	105.58	86.08	128.83	RW2
Congo	POINTE NOIRE	1998	107.40	86.89	131.88	RW2
Congo	POINTE NOIRE	1999	107.05	85.26	133.48	RW2
Congo	POINTE NOIRE	2000	104.56	82.78	129.54	RW2
Congo	POINTE NOIRE	2001	101.24	81.05	124.47	RW2
Congo	POINTE NOIRE	2002	97.09	78.15	119.25	RW2
Congo	POINTE NOIRE	2003	92.22	73.21	114.53	RW2
Congo	POINTE NOIRE	2004	87.04	67.84	110.50	RW2
Congo	POINTE NOIRE	2005	81.62	63.09	104.73	RW2
Congo	POINTE NOIRE	2006	75.91	58.68	97.23	RW2
Congo	POINTE NOIRE	2007	70.06	54.14	89.91	RW2
Congo	POINTE NOIRE	2008	64.11	48.88	83.95	RW2
Congo	POINTE NOIRE	2009	58.34	43.32	78.28	RW2
Congo	POINTE NOIRE	2010	52.67	37.98	72.65	RW2
Congo	POINTE NOIRE	2011	47.46	34.19	66.28	RW2
Congo	POINTE NOIRE	2012	42.62	30.42	60.60	RW2
Congo	POINTE NOIRE	2013	38.20	26.10	57.44	RW2
Congo	POINTE NOIRE	2014	34.24	21.12	57.08	RW2
Congo	POINTE NOIRE	2015	30.78	15.89	60.67	RW2
Congo	POINTE NOIRE	2016	27.58	11.69	65.46	RW2
Congo	POINTE NOIRE	2017	24.68	8.38	72.27	RW2
Congo	POINTE NOIRE	2018	22.14	5.85	82.31	RW2
Congo	POINTE NOIRE	2019	19.84	3.93	95.26	RW2
Congo	SUD	1980	150.51	98.47	222.33	RW2
Congo	SUD	1981	144.99	104.16	197.43	RW2
Congo	SUD	1982	139.30	104.65	182.04	RW2
Congo	SUD	1983	133.85	101.49	174.22	RW2
Congo	SUD	1984	128.60	97.25	168.06	RW2
Congo	SUD	1985	123.80	95.47	159.29	RW2
Congo	SUD	1986	119.23	93.62	150.51	RW2
Congo	SUD	1987	115.02	91.64	143.88	RW2
Congo	SUD	1988	111.45	88.92	139.68	RW2
Congo	SUD	1989	108.78	86.69	136.72	RW2
Congo	SUD	1990	106.66	85.18	132.08	RW2
Congo	SUD	1991	106.99	86.99	130.47	RW2
Congo	SUD	1992	109.09	89.31	131.90	RW2
Congo	SUD	1993	112.99	92.06	136.66	RW2
Congo	SUD	1994	118.57	95.32	144.00	RW2
Congo	SUD	1995	125.94	103.39	154.13	RW2
Congo	SUD	1996	131.87	109.77	158.52	RW2
Congo	SUD	1997	136.28	114.25	162.51	RW2
Congo	SUD	1998	138.45	115.66	165.80	RW2
Congo	SUD	1999	138.06	113.25	167.41	RW2
Congo	SUD	2000	134.50	109.68	162.52	RW2
Congo	SUD	2001	129.96	107.45	155.39	RW2
Congo	SUD	2002	124.06	103.56	147.87	RW2
Congo	SUD	2003	117.23	97.12	141.34	RW2
Congo	SUD	2004	109.88	89.24	134.64	RW2
Congo	SUD	2005	102.21	82.45	126.47	RW2
Congo	SUD	2006	94.10	76.52	115.59	RW2
Congo	SUD	2007	85.74	69.57	105.07	RW2

Continued on next page

Country	Region	Year	Median	Lower	Upper	Method
Congo	SUD	2008	77.46	61.97	96.36	RW2
Congo	SUD	2009	69.28	54.29	88.11	RW2
Congo	SUD	2010	61.53	47.40	79.50	RW2
Congo	SUD	2011	54.41	42.27	69.72	RW2
Congo	SUD	2012	47.99	37.60	60.97	RW2
Congo	SUD	2013	42.24	31.92	55.82	RW2
Congo	SUD	2014	37.16	25.15	54.30	RW2
Congo	SUD	2015	32.69	18.38	57.08	RW2
Congo	SUD	2016	28.66	13.15	60.60	RW2
Congo	SUD	2017	25.20	9.17	66.40	RW2
Congo	SUD	2018	22.05	6.19	74.46	RW2
Congo	SUD	2019	19.35	4.10	85.69	RW2
Côte d'Ivoire	ALL	1980	160.49	154.91	166.29	IHME
Côte d'Ivoire	ALL	1980	159.38	90.08	265.92	RW2
Côte d'Ivoire	ALL	1980	166.60	153.90	180.30	UN
Côte d'Ivoire	ALL	1981	157.08	151.78	162.96	IHME
Côte d'Ivoire	ALL	1981	158.81	100.89	240.69	RW2
Côte d'Ivoire	ALL	1981	162.80	150.30	176.00	UN
Côte d'Ivoire	ALL	1982	154.31	149.18	160.08	IHME
Côte d'Ivoire	ALL	1982	158.46	109.07	224.40	RW2
Côte d'Ivoire	ALL	1982	159.70	147.50	172.40	UN
Côte d'Ivoire	ALL	1983	152.90	147.98	158.54	IHME
Côte d'Ivoire	ALL	1983	157.60	113.07	215.53	RW2
Côte d'Ivoire	ALL	1983	157.30	145.40	169.70	UN
Côte d'Ivoire	ALL	1984	151.80	146.87	157.20	IHME
Côte d'Ivoire	ALL	1984	157.30	114.48	211.58	RW2
Côte d'Ivoire	ALL	1984	155.50	143.80	167.70	UN
Côte d'Ivoire	ALL	1985	150.82	145.79	156.12	IHME
Côte d'Ivoire	ALL	1985	156.37	117.20	205.96	RW2
Côte d'Ivoire	ALL	1985	154.20	142.80	166.20	UN
Côte d'Ivoire	ALL	1986	149.99	145.25	155.15	IHME
Côte d'Ivoire	ALL	1986	155.70	118.53	202.00	RW2
Côte d'Ivoire	ALL	1986	153.30	142.00	165.10	UN
Côte d'Ivoire	ALL	1987	149.48	144.72	154.46	IHME
Côte d'Ivoire	ALL	1987	155.11	119.66	199.30	RW2
Côte d'Ivoire	ALL	1987	152.80	141.70	164.40	UN
Côte d'Ivoire	ALL	1988	149.16	144.36	154.11	IHME
Côte d'Ivoire	ALL	1988	154.33	119.16	197.70	RW2
Côte d'Ivoire	ALL	1988	152.50	141.70	163.90	UN
Côte d'Ivoire	ALL	1989	148.92	144.14	153.73	IHME
Côte d'Ivoire	ALL	1989	153.65	118.98	196.47	RW2
Côte d'Ivoire	ALL	1989	152.50	141.80	163.70	UN
Côte d'Ivoire	ALL	1990	148.88	144.20	153.65	IHME
Côte d'Ivoire	ALL	1990	152.97	119.76	193.23	RW2
Côte d'Ivoire	ALL	1990	152.60	142.10	163.60	UN
Côte d'Ivoire	ALL	1991	148.87	144.14	154.02	IHME
Côte d'Ivoire	ALL	1991	152.55	120.94	189.83	RW2
Côte d'Ivoire	ALL	1991	152.70	142.40	163.60	UN
Côte d'Ivoire	ALL	1992	148.95	144.14	154.15	IHME
Côte d'Ivoire	ALL	1992	152.30	121.43	188.56	RW2
Côte d'Ivoire	ALL	1992	152.70	142.40	163.80	UN
Côte d'Ivoire	ALL	1993	148.98	143.78	154.11	IHME
Côte d'Ivoire	ALL	1993	152.25	121.14	189.01	RW2
Côte d'Ivoire	ALL	1993	152.80	142.40	164.10	UN
Côte d'Ivoire	ALL	1994	148.99	143.70	154.10	IHME
Côte d'Ivoire	ALL	1994	152.14	120.23	190.61	RW2
Côte d'Ivoire	ALL	1994	152.90	142.20	164.30	UN
Côte d'Ivoire	ALL	1995	148.69	143.30	153.93	IHME
Côte d'Ivoire	ALL	1995	152.33	121.56	189.60	RW2
Côte d'Ivoire	ALL	1995	152.70	142.00	164.30	UN
Côte d'Ivoire	ALL	1996	147.77	142.39	153.22	IHME
Côte d'Ivoire	ALL	1996	151.89	122.47	187.78	RW2
Côte d'Ivoire	ALL	1996	152.20	141.20	164.20	UN

Continued on next page

Country	Region	Year	Median	Lower	Upper	Method
Côte d'Ivoire	ALL	1997	146.46	141.00	151.90	IHME
Côte d'Ivoire	ALL	1997	151.11	122.51	185.31	RW2
Côte d'Ivoire	ALL	1997	151.30	140.10	163.50	UN
Côte d'Ivoire	ALL	1998	144.80	139.25	150.39	IHME
Côte d'Ivoire	ALL	1998	149.95	120.91	185.41	RW2
Côte d'Ivoire	ALL	1998	149.80	138.40	162.40	UN
Côte d'Ivoire	ALL	1999	142.81	137.13	148.73	IHME
Côte d'Ivoire	ALL	1999	148.20	118.31	183.79	RW2
Côte d'Ivoire	ALL	1999	147.90	136.20	160.80	UN
Côte d'Ivoire	ALL	2000	140.78	134.98	146.61	IHME
Côte d'Ivoire	ALL	2000	145.86	116.83	180.39	RW2
Côte d'Ivoire	ALL	2000	145.60	133.80	158.60	UN
Côte d'Ivoire	ALL	2001	138.48	132.81	144.28	IHME
Côte d'Ivoire	ALL	2001	143.17	115.54	175.86	RW2
Côte d'Ivoire	ALL	2001	142.80	130.80	156.00	UN
Côte d'Ivoire	ALL	2002	136.26	130.21	142.21	IHME
Côte d'Ivoire	ALL	2002	140.02	113.74	171.30	RW2
Côte d'Ivoire	ALL	2002	139.70	127.80	152.70	UN
Côte d'Ivoire	ALL	2003	133.74	127.53	139.81	IHME
Côte d'Ivoire	ALL	2003	136.65	110.65	167.81	RW2
Côte d'Ivoire	ALL	2003	136.30	124.50	149.10	UN
Côte d'Ivoire	ALL	2004	131.06	124.80	137.40	IHME
Côte d'Ivoire	ALL	2004	132.80	106.02	165.02	RW2
Côte d'Ivoire	ALL	2004	132.70	121.10	145.50	UN
Côte d'Ivoire	ALL	2005	128.05	121.53	134.56	IHME
Côte d'Ivoire	ALL	2005	128.95	102.83	160.37	RW2
Côte d'Ivoire	ALL	2005	128.80	117.50	141.50	UN
Côte d'Ivoire	ALL	2006	124.82	118.13	131.44	IHME
Côte d'Ivoire	ALL	2006	124.96	100.78	153.68	RW2
Côte d'Ivoire	ALL	2006	125.10	113.70	137.80	UN
Côte d'Ivoire	ALL	2007	121.45	114.75	128.31	IHME
Côte d'Ivoire	ALL	2007	121.03	98.36	147.98	RW2
Côte d'Ivoire	ALL	2007	121.10	109.60	134.20	UN
Côte d'Ivoire	ALL	2008	117.73	110.50	125.14	IHME
Côte d'Ivoire	ALL	2008	117.29	94.48	144.85	RW2
Côte d'Ivoire	ALL	2008	116.50	104.70	129.80	UN
Côte d'Ivoire	ALL	2009	114.16	106.60	122.19	IHME
Côte d'Ivoire	ALL	2009	113.40	89.52	143.11	RW2
Côte d'Ivoire	ALL	2009	112.70	100.40	126.60	UN
Côte d'Ivoire	ALL	2010	110.66	102.85	119.56	IHME
Côte d'Ivoire	ALL	2010	109.71	84.76	141.69	RW2
Côte d'Ivoire	ALL	2010	109.10	95.80	124.10	UN
Côte d'Ivoire	ALL	2011	107.72	99.30	117.31	IHME
Côte d'Ivoire	ALL	2011	106.19	81.76	136.89	RW2
Côte d'Ivoire	ALL	2011	105.90	91.70	122.60	UN
Côte d'Ivoire	ALL	2012	104.40	95.74	114.71	IHME
Côte d'Ivoire	ALL	2012	102.67	78.35	133.41	RW2
Côte d'Ivoire	ALL	2012	102.50	87.00	120.90	UN
Côte d'Ivoire	ALL	2013	100.83	91.72	111.51	IHME
Côte d'Ivoire	ALL	2013	99.37	72.41	134.66	RW2
Côte d'Ivoire	ALL	2013	99.00	82.00	119.50	UN
Côte d'Ivoire	ALL	2014	97.26	87.95	108.81	IHME
Côte d'Ivoire	ALL	2014	96.04	63.08	143.39	RW2
Côte d'Ivoire	ALL	2014	95.50	76.80	119.20	UN
Côte d'Ivoire	ALL	2015	93.49	83.69	105.67	IHME
Côte d'Ivoire	ALL	2015	92.70	51.15	162.46	RW2
Côte d'Ivoire	ALL	2015	92.60	72.30	118.80	UN
Côte d'Ivoire	ALL	2016	89.83	41.18	186.09	RW2
Côte d'Ivoire	ALL	2017	86.59	31.87	216.39	RW2
Côte d'Ivoire	ALL	2018	83.63	24.37	256.13	RW2
Côte d'Ivoire	ALL	2019	80.65	17.50	299.39	RW2
Côte d'Ivoire	ALL	15-19	86.59	32.38	212.92	RW2
Côte d'Ivoire	CENTRE	1980	184.17	99.04	319.01	RW2

Continued on next page

Country	Region	Year	Median	Lower	Upper	Method
Côte d'Ivoire	CENTRE	1981	176.16	103.25	284.73	RW2
Côte d'Ivoire	CENTRE	1982	168.08	103.61	261.23	RW2
Côte d'Ivoire	CENTRE	1983	160.33	101.70	244.47	RW2
Côte d'Ivoire	CENTRE	1984	153.43	99.49	229.66	RW2
Côte d'Ivoire	CENTRE	1985	146.86	96.62	215.80	RW2
Côte d'Ivoire	CENTRE	1986	142.30	95.55	205.49	RW2
Côte d'Ivoire	CENTRE	1987	139.05	95.51	197.29	RW2
Côte d'Ivoire	CENTRE	1988	137.11	95.34	192.28	RW2
Côte d'Ivoire	CENTRE	1989	136.42	96.23	188.97	RW2
Côte d'Ivoire	CENTRE	1990	137.12	99.01	187.35	RW2
Côte d'Ivoire	CENTRE	1991	138.25	101.87	185.50	RW2
Côte d'Ivoire	CENTRE	1992	139.76	104.43	184.58	RW2
Côte d'Ivoire	CENTRE	1993	141.37	107.06	184.49	RW2
Côte d'Ivoire	CENTRE	1994	143.09	109.00	185.85	RW2
Côte d'Ivoire	CENTRE	1995	144.76	111.91	184.97	RW2
Côte d'Ivoire	CENTRE	1996	146.76	115.32	184.49	RW2
Côte d'Ivoire	CENTRE	1997	148.78	118.46	184.65	RW2
Côte d'Ivoire	CENTRE	1998	150.74	120.47	187.10	RW2
Côte d'Ivoire	CENTRE	1999	152.51	121.21	189.92	RW2
Côte d'Ivoire	CENTRE	2000	154.13	122.93	190.95	RW2
Côte d'Ivoire	CENTRE	2001	154.69	125.08	189.88	RW2
Côte d'Ivoire	CENTRE	2002	154.36	125.20	189.02	RW2
Côte d'Ivoire	CENTRE	2003	152.84	123.17	188.74	RW2
Côte d'Ivoire	CENTRE	2004	150.64	119.75	188.27	RW2
Côte d'Ivoire	CENTRE	2005	147.43	115.59	185.78	RW2
Côte d'Ivoire	CENTRE	2006	144.42	113.16	182.39	RW2
Côte d'Ivoire	CENTRE	2007	141.44	110.00	179.92	RW2
Côte d'Ivoire	CENTRE	2008	138.87	105.83	180.12	RW2
Côte d'Ivoire	CENTRE	2009	136.40	101.16	181.70	RW2
Côte d'Ivoire	CENTRE	2010	134.85	96.34	185.39	RW2
Côte d'Ivoire	CENTRE	2011	132.81	93.13	186.36	RW2
Côte d'Ivoire	CENTRE	2012	131.32	89.30	188.48	RW2
Côte d'Ivoire	CENTRE	2013	129.60	83.58	194.84	RW2
Côte d'Ivoire	CENTRE	2014	128.05	75.64	206.51	RW2
Côte d'Ivoire	CENTRE	2015	126.47	65.21	231.41	RW2
Côte d'Ivoire	CENTRE	2016	124.80	55.11	260.43	RW2
Côte d'Ivoire	CENTRE	2017	123.32	44.94	299.63	RW2
Côte d'Ivoire	CENTRE	2018	121.33	35.25	345.25	RW2
Côte d'Ivoire	CENTRE	2019	119.61	27.79	398.41	RW2
Côte d'Ivoire	CENTRE-EST	1980	168.30	86.26	306.63	RW2
Côte d'Ivoire	CENTRE-EST	1981	159.84	88.96	273.52	RW2
Côte d'Ivoire	CENTRE-EST	1982	151.81	89.80	249.72	RW2
Côte d'Ivoire	CENTRE-EST	1983	144.58	88.49	231.07	RW2
Côte d'Ivoire	CENTRE-EST	1984	138.11	85.89	215.44	RW2
Côte d'Ivoire	CENTRE-EST	1985	131.76	84.28	201.27	RW2
Côte d'Ivoire	CENTRE-EST	1986	127.25	83.33	189.70	RW2
Côte d'Ivoire	CENTRE-EST	1987	124.06	83.41	180.66	RW2
Côte d'Ivoire	CENTRE-EST	1988	122.10	83.58	174.15	RW2
Côte d'Ivoire	CENTRE-EST	1989	120.99	84.98	169.88	RW2
Côte d'Ivoire	CENTRE-EST	1990	121.66	87.83	165.98	RW2
Côte d'Ivoire	CENTRE-EST	1991	122.38	90.39	163.75	RW2
Côte d'Ivoire	CENTRE-EST	1992	123.62	93.15	162.09	RW2
Côte d'Ivoire	CENTRE-EST	1993	124.93	94.76	163.05	RW2
Côte d'Ivoire	CENTRE-EST	1994	126.45	96.29	164.23	RW2
Côte d'Ivoire	CENTRE-EST	1995	127.96	98.13	164.54	RW2
Côte d'Ivoire	CENTRE-EST	1996	129.92	101.33	165.10	RW2
Côte d'Ivoire	CENTRE-EST	1997	132.05	103.32	165.96	RW2
Côte d'Ivoire	CENTRE-EST	1998	133.98	104.98	168.64	RW2
Côte d'Ivoire	CENTRE-EST	1999	136.02	105.83	171.40	RW2
Côte d'Ivoire	CENTRE-EST	2000	138.03	108.13	174.11	RW2
Côte d'Ivoire	CENTRE-EST	2001	139.03	110.09	174.02	RW2
Côte d'Ivoire	CENTRE-EST	2002	139.41	110.75	174.28	RW2
Côte d'Ivoire	CENTRE-EST	2003	138.92	109.30	174.62	RW2

Continued on next page

Country	Region	Year	Median	Lower	Upper	Method
Côte d'Ivoire	CENTRE-EST	2004	137.54	106.46	176.01	RW2
Côte d'Ivoire	CENTRE-EST	2005	135.40	103.61	174.89	RW2
Côte d'Ivoire	CENTRE-EST	2006	133.32	101.41	173.00	RW2
Côte d'Ivoire	CENTRE-EST	2007	131.42	99.00	172.95	RW2
Côte d'Ivoire	CENTRE-EST	2008	129.53	95.41	174.59	RW2
Côte d'Ivoire	CENTRE-EST	2009	128.21	90.94	178.03	RW2
Côte d'Ivoire	CENTRE-EST	2010	127.20	87.57	182.54	RW2
Côte d'Ivoire	CENTRE-EST	2011	126.29	84.69	184.82	RW2
Côte d'Ivoire	CENTRE-EST	2012	125.40	81.92	189.56	RW2
Côte d'Ivoire	CENTRE-EST	2013	124.50	76.87	196.05	RW2
Côte d'Ivoire	CENTRE-EST	2014	123.65	70.37	210.58	RW2
Côte d'Ivoire	CENTRE-EST	2015	122.97	60.38	235.95	RW2
Côte d'Ivoire	CENTRE-EST	2016	121.77	51.73	264.70	RW2
Côte d'Ivoire	CENTRE-EST	2017	120.82	41.98	303.85	RW2
Côte d'Ivoire	CENTRE-EST	2018	120.14	33.83	349.27	RW2
Côte d'Ivoire	CENTRE-EST	2019	119.37	26.35	402.10	RW2
Côte d'Ivoire	CENTRE-NORD	1980	184.79	106.76	303.16	RW2
Côte d'Ivoire	CENTRE-NORD	1981	173.08	108.67	265.46	RW2
Côte d'Ivoire	CENTRE-NORD	1982	161.81	105.81	238.87	RW2
Côte d'Ivoire	CENTRE-NORD	1983	151.61	101.82	220.20	RW2
Côte d'Ivoire	CENTRE-NORD	1984	142.26	96.38	205.36	RW2
Côte d'Ivoire	CENTRE-NORD	1985	133.60	91.49	190.23	RW2
Côte d'Ivoire	CENTRE-NORD	1986	126.74	87.76	178.14	RW2
Côte d'Ivoire	CENTRE-NORD	1987	121.27	85.03	169.46	RW2
Côte d'Ivoire	CENTRE-NORD	1988	117.08	82.79	162.41	RW2
Côte d'Ivoire	CENTRE-NORD	1989	114.14	81.03	157.50	RW2
Côte d'Ivoire	CENTRE-NORD	1990	112.54	81.02	154.30	RW2
Côte d'Ivoire	CENTRE-NORD	1991	111.16	81.12	151.20	RW2
Côte d'Ivoire	CENTRE-NORD	1992	110.04	80.95	147.60	RW2
Côte d'Ivoire	CENTRE-NORD	1993	109.05	80.51	145.94	RW2
Côte d'Ivoire	CENTRE-NORD	1994	108.16	79.76	145.02	RW2
Côte d'Ivoire	CENTRE-NORD	1995	107.27	79.85	142.38	RW2
Côte d'Ivoire	CENTRE-NORD	1996	106.43	80.09	139.58	RW2
Côte d'Ivoire	CENTRE-NORD	1997	105.75	80.16	137.44	RW2
Côte d'Ivoire	CENTRE-NORD	1998	104.75	79.40	136.47	RW2
Côte d'Ivoire	CENTRE-NORD	1999	103.69	77.82	136.50	RW2
Côte d'Ivoire	CENTRE-NORD	2000	102.51	77.20	134.58	RW2
Côte d'Ivoire	CENTRE-NORD	2001	100.49	76.27	130.79	RW2
Côte d'Ivoire	CENTRE-NORD	2002	97.92	74.40	127.66	RW2
Côte d'Ivoire	CENTRE-NORD	2003	94.69	71.51	124.36	RW2
Côte d'Ivoire	CENTRE-NORD	2004	91.18	67.89	121.50	RW2
Côte d'Ivoire	CENTRE-NORD	2005	87.00	63.90	116.67	RW2
Côte d'Ivoire	CENTRE-NORD	2006	83.31	61.09	112.22	RW2
Côte d'Ivoire	CENTRE-NORD	2007	79.58	57.82	108.48	RW2
Côte d'Ivoire	CENTRE-NORD	2008	76.26	54.48	105.74	RW2
Côte d'Ivoire	CENTRE-NORD	2009	73.53	51.10	104.68	RW2
Côte d'Ivoire	CENTRE-NORD	2010	70.98	47.78	103.85	RW2
Côte d'Ivoire	CENTRE-NORD	2011	68.56	45.60	102.31	RW2
Côte d'Ivoire	CENTRE-NORD	2012	66.29	42.90	101.58	RW2
Côte d'Ivoire	CENTRE-NORD	2013	64.15	39.51	102.27	RW2
Côte d'Ivoire	CENTRE-NORD	2014	62.19	35.16	107.15	RW2
Côte d'Ivoire	CENTRE-NORD	2015	60.06	29.89	118.59	RW2
Côte d'Ivoire	CENTRE-NORD	2016	58.02	24.41	132.39	RW2
Côte d'Ivoire	CENTRE-NORD	2017	56.25	19.54	151.96	RW2
Côte d'Ivoire	CENTRE-NORD	2018	53.99	15.25	178.52	RW2
Côte d'Ivoire	CENTRE-NORD	2019	52.20	11.54	210.37	RW2
Côte d'Ivoire	CENTRE-OUEST	1980	196.39	95.02	361.15	RW2
Côte d'Ivoire	CENTRE-OUEST	1981	185.29	97.18	321.64	RW2
Côte d'Ivoire	CENTRE-OUEST	1982	174.53	97.13	291.20	RW2
Côte d'Ivoire	CENTRE-OUEST	1983	164.44	95.23	268.85	RW2
Côte d'Ivoire	CENTRE-OUEST	1984	155.11	92.24	248.72	RW2
Côte d'Ivoire	CENTRE-OUEST	1985	146.51	89.61	229.31	RW2
Côte d'Ivoire	CENTRE-OUEST	1986	139.91	87.81	213.49	RW2

Continued on next page

Country	Region	Year	Median	Lower	Upper	Method
Côte d'Ivoire	CENTRE-OUEST	1987	134.29	86.97	200.57	RW2
Côte d'Ivoire	CENTRE-OUEST	1988	130.69	86.54	191.10	RW2
Côte d'Ivoire	CENTRE-OUEST	1989	128.00	86.82	183.86	RW2
Côte d'Ivoire	CENTRE-OUEST	1990	126.73	88.78	177.56	RW2
Côte d'Ivoire	CENTRE-OUEST	1991	125.50	90.29	172.40	RW2
Côte d'Ivoire	CENTRE-OUEST	1992	124.68	91.42	167.87	RW2
Côte d'Ivoire	CENTRE-OUEST	1993	124.02	92.20	164.93	RW2
Côte d'Ivoire	CENTRE-OUEST	1994	123.23	92.13	163.02	RW2
Côte d'Ivoire	CENTRE-OUEST	1995	122.34	92.44	159.87	RW2
Côte d'Ivoire	CENTRE-OUEST	1996	121.58	93.38	156.30	RW2
Côte d'Ivoire	CENTRE-OUEST	1997	120.76	93.72	154.11	RW2
Côte d'Ivoire	CENTRE-OUEST	1998	119.83	93.53	152.43	RW2
Côte d'Ivoire	CENTRE-OUEST	1999	118.68	91.96	151.52	RW2
Côte d'Ivoire	CENTRE-OUEST	2000	117.22	91.28	149.09	RW2
Côte d'Ivoire	CENTRE-OUEST	2001	114.98	90.53	145.11	RW2
Côte d'Ivoire	CENTRE-OUEST	2002	111.84	88.46	140.55	RW2
Côte d'Ivoire	CENTRE-OUEST	2003	108.33	84.97	137.67	RW2
Côte d'Ivoire	CENTRE-OUEST	2004	104.00	80.42	133.99	RW2
Côte d'Ivoire	CENTRE-OUEST	2005	99.19	76.06	128.03	RW2
Côte d'Ivoire	CENTRE-OUEST	2006	94.76	72.62	122.52	RW2
Côte d'Ivoire	CENTRE-OUEST	2007	90.69	68.86	118.21	RW2
Côte d'Ivoire	CENTRE-OUEST	2008	86.85	64.65	115.61	RW2
Côte d'Ivoire	CENTRE-OUEST	2009	83.43	60.27	114.32	RW2
Côte d'Ivoire	CENTRE-OUEST	2010	80.40	56.35	113.96	RW2
Côte d'Ivoire	CENTRE-OUEST	2011	77.70	52.91	111.69	RW2
Côte d'Ivoire	CENTRE-OUEST	2012	74.95	49.90	111.38	RW2
Côte d'Ivoire	CENTRE-OUEST	2013	72.45	45.94	112.88	RW2
Côte d'Ivoire	CENTRE-OUEST	2014	69.88	40.45	118.29	RW2
Côte d'Ivoire	CENTRE-OUEST	2015	67.64	33.96	131.24	RW2
Côte d'Ivoire	CENTRE-OUEST	2016	65.48	27.88	147.20	RW2
Côte d'Ivoire	CENTRE-OUEST	2017	63.03	22.14	167.38	RW2
Côte d'Ivoire	CENTRE-OUEST	2018	60.63	17.30	194.44	RW2
Côte d'Ivoire	CENTRE-OUEST	2019	58.67	13.11	232.18	RW2
Côte d'Ivoire	NORD	1980	266.98	146.69	437.69	RW2
Côte d'Ivoire	NORD	1981	256.99	153.07	399.21	RW2
Côte d'Ivoire	NORD	1982	247.18	154.44	371.62	RW2
Côte d'Ivoire	NORD	1983	237.48	153.73	349.30	RW2
Côte d'Ivoire	NORD	1984	229.00	151.41	332.25	RW2
Côte d'Ivoire	NORD	1985	221.29	148.90	313.33	RW2
Côte d'Ivoire	NORD	1986	215.32	148.84	299.83	RW2
Côte d'Ivoire	NORD	1987	211.40	149.00	290.14	RW2
Côte d'Ivoire	NORD	1988	209.65	150.05	284.00	RW2
Côte d'Ivoire	NORD	1989	209.51	151.87	280.13	RW2
Côte d'Ivoire	NORD	1990	211.30	157.30	279.20	RW2
Côte d'Ivoire	NORD	1991	213.75	161.51	277.57	RW2
Côte d'Ivoire	NORD	1992	216.48	166.45	276.77	RW2
Côte d'Ivoire	NORD	1993	219.73	170.94	278.27	RW2
Côte d'Ivoire	NORD	1994	222.91	174.51	280.41	RW2
Côte d'Ivoire	NORD	1995	225.97	180.02	280.05	RW2
Côte d'Ivoire	NORD	1996	229.34	185.70	279.84	RW2
Côte d'Ivoire	NORD	1997	232.84	190.83	280.84	RW2
Côte d'Ivoire	NORD	1998	236.13	193.38	284.01	RW2
Côte d'Ivoire	NORD	1999	238.79	195.16	287.46	RW2
Côte d'Ivoire	NORD	2000	241.38	199.15	289.47	RW2
Côte d'Ivoire	NORD	2001	242.17	201.70	287.94	RW2
Côte d'Ivoire	NORD	2002	241.79	202.98	285.89	RW2
Côte d'Ivoire	NORD	2003	239.63	199.92	284.54	RW2
Côte d'Ivoire	NORD	2004	236.43	195.72	283.47	RW2
Côte d'Ivoire	NORD	2005	231.65	190.47	277.87	RW2
Côte d'Ivoire	NORD	2006	227.49	188.58	271.44	RW2
Côte d'Ivoire	NORD	2007	223.48	185.31	266.78	RW2
Côte d'Ivoire	NORD	2008	220.03	180.25	265.40	RW2
Côte d'Ivoire	NORD	2009	217.17	174.16	266.89	RW2

Continued on next page

Country	Region	Year	Median	Lower	Upper	Method
Côte d'Ivoire	NORD	2010	214.98	169.11	269.83	RW2
Côte d'Ivoire	NORD	2011	213.17	166.23	269.26	RW2
Côte d'Ivoire	NORD	2012	211.30	162.99	269.92	RW2
Côte d'Ivoire	NORD	2013	209.64	155.27	277.86	RW2
Côte d'Ivoire	NORD	2014	207.87	141.92	294.70	RW2
Côte d'Ivoire	NORD	2015	206.30	122.17	327.90	RW2
Côte d'Ivoire	NORD	2016	204.29	102.87	366.59	RW2
Côte d'Ivoire	NORD	2017	202.74	84.50	418.63	RW2
Côte d'Ivoire	NORD	2018	201.13	67.69	470.92	RW2
Côte d'Ivoire	NORD	2019	199.77	53.80	528.11	RW2
Côte d'Ivoire	NORD-EST	1980	243.90	127.08	422.77	RW2
Côte d'Ivoire	NORD-EST	1981	229.25	128.80	381.51	RW2
Côte d'Ivoire	NORD-EST	1982	216.30	126.79	347.37	RW2
Côte d'Ivoire	NORD-EST	1983	203.51	123.83	321.13	RW2
Côte d'Ivoire	NORD-EST	1984	191.73	118.85	296.91	RW2
Côte d'Ivoire	NORD-EST	1985	180.93	114.62	274.34	RW2
Côte d'Ivoire	NORD-EST	1986	172.33	112.12	256.60	RW2
Côte d'Ivoire	NORD-EST	1987	165.74	109.93	241.57	RW2
Côte d'Ivoire	NORD-EST	1988	160.95	109.16	230.80	RW2
Côte d'Ivoire	NORD-EST	1989	157.47	108.37	222.71	RW2
Côte d'Ivoire	NORD-EST	1990	155.55	109.99	215.72	RW2
Côte d'Ivoire	NORD-EST	1991	154.26	111.90	209.48	RW2
Côte d'Ivoire	NORD-EST	1992	153.29	112.74	204.71	RW2
Côte d'Ivoire	NORD-EST	1993	152.72	114.10	201.72	RW2
Côte d'Ivoire	NORD-EST	1994	151.99	114.02	199.52	RW2
Côte d'Ivoire	NORD-EST	1995	151.64	115.25	196.32	RW2
Côte d'Ivoire	NORD-EST	1996	151.27	117.05	192.87	RW2
Côte d'Ivoire	NORD-EST	1997	151.17	117.86	190.83	RW2
Côte d'Ivoire	NORD-EST	1998	151.02	117.91	190.81	RW2
Côte d'Ivoire	NORD-EST	1999	150.71	117.20	190.77	RW2
Côte d'Ivoire	NORD-EST	2000	150.46	117.34	190.52	RW2
Côte d'Ivoire	NORD-EST	2001	149.11	117.14	187.57	RW2
Côte d'Ivoire	NORD-EST	2002	146.79	115.82	184.66	RW2
Côte d'Ivoire	NORD-EST	2003	143.75	111.82	182.97	RW2
Côte d'Ivoire	NORD-EST	2004	140.05	107.78	180.70	RW2
Côte d'Ivoire	NORD-EST	2005	135.28	102.56	176.14	RW2
Côte d'Ivoire	NORD-EST	2006	131.18	99.30	171.54	RW2
Côte d'Ivoire	NORD-EST	2007	127.21	95.45	167.79	RW2
Côte d'Ivoire	NORD-EST	2008	123.49	90.83	165.73	RW2
Côte d'Ivoire	NORD-EST	2009	120.34	86.21	165.46	RW2
Côte d'Ivoire	NORD-EST	2010	117.48	82.28	165.93	RW2
Côte d'Ivoire	NORD-EST	2011	114.93	79.28	163.78	RW2
Côte d'Ivoire	NORD-EST	2012	112.40	76.05	163.45	RW2
Côte d'Ivoire	NORD-EST	2013	110.15	71.38	166.25	RW2
Côte d'Ivoire	NORD-EST	2014	107.76	64.55	175.48	RW2
Côte d'Ivoire	NORD-EST	2015	105.29	54.94	193.73	RW2
Côte d'Ivoire	NORD-EST	2016	102.97	45.80	216.57	RW2
Côte d'Ivoire	NORD-EST	2017	100.57	37.24	247.60	RW2
Côte d'Ivoire	NORD-EST	2018	98.26	29.06	287.95	RW2
Côte d'Ivoire	NORD-EST	2019	96.26	22.36	336.25	RW2
Côte d'Ivoire	NORD-OUEST	1980	243.85	144.88	381.07	RW2
Côte d'Ivoire	NORD-OUEST	1981	234.63	151.60	343.59	RW2
Côte d'Ivoire	NORD-OUEST	1982	226.17	153.64	318.68	RW2
Côte d'Ivoire	NORD-OUEST	1983	217.32	152.14	300.72	RW2
Côte d'Ivoire	NORD-OUEST	1984	209.74	149.84	285.96	RW2
Côte d'Ivoire	NORD-OUEST	1985	202.56	146.63	271.61	RW2
Côte d'Ivoire	NORD-OUEST	1986	197.52	145.34	261.14	RW2
Côte d'Ivoire	NORD-OUEST	1987	194.47	145.74	254.52	RW2
Côte d'Ivoire	NORD-OUEST	1988	192.97	145.46	250.67	RW2
Côte d'Ivoire	NORD-OUEST	1989	193.10	146.44	248.84	RW2
Côte d'Ivoire	NORD-OUEST	1990	195.23	150.98	248.65	RW2
Côte d'Ivoire	NORD-OUEST	1991	197.63	155.13	248.91	RW2
Côte d'Ivoire	NORD-OUEST	1992	200.47	159.45	249.63	RW2

Continued on next page

Country	Region	Year	Median	Lower	Upper	Method
Côte d'Ivoire	NORD-OUEST	1993	203.64	162.18	252.91	RW2
Côte d'Ivoire	NORD-OUEST	1994	206.89	165.47	255.74	RW2
Côte d'Ivoire	NORD-OUEST	1995	209.89	169.58	256.91	RW2
Côte d'Ivoire	NORD-OUEST	1996	213.17	175.21	257.56	RW2
Côte d'Ivoire	NORD-OUEST	1997	216.12	179.41	258.62	RW2
Côte d'Ivoire	NORD-OUEST	1998	219.01	181.61	261.54	RW2
Côte d'Ivoire	NORD-OUEST	1999	221.29	182.54	265.32	RW2
Côte d'Ivoire	NORD-OUEST	2000	222.89	185.30	266.36	RW2
Côte d'Ivoire	NORD-OUEST	2001	222.90	187.29	263.52	RW2
Côte d'Ivoire	NORD-OUEST	2002	221.60	187.36	260.84	RW2
Côte d'Ivoire	NORD-OUEST	2003	218.63	183.35	258.47	RW2
Côte d'Ivoire	NORD-OUEST	2004	214.66	177.84	257.25	RW2
Côte d'Ivoire	NORD-OUEST	2005	209.24	171.73	251.69	RW2
Côte d'Ivoire	NORD-OUEST	2006	204.52	168.41	245.05	RW2
Côte d'Ivoire	NORD-OUEST	2007	199.66	164.36	240.37	RW2
Côte d'Ivoire	NORD-OUEST	2008	195.62	158.21	238.73	RW2
Côte d'Ivoire	NORD-OUEST	2009	191.87	151.26	239.99	RW2
Côte d'Ivoire	NORD-OUEST	2010	189.28	145.45	242.54	RW2
Côte d'Ivoire	NORD-OUEST	2011	186.39	141.74	240.29	RW2
Côte d'Ivoire	NORD-OUEST	2012	183.85	137.73	241.51	RW2
Côte d'Ivoire	NORD-OUEST	2013	181.52	129.71	247.17	RW2
Côte d'Ivoire	NORD-OUEST	2014	179.09	117.29	262.31	RW2
Côte d'Ivoire	NORD-OUEST	2015	176.61	100.83	290.21	RW2
Côte d'Ivoire	NORD-OUEST	2016	174.19	84.21	325.76	RW2
Côte d'Ivoire	NORD-OUEST	2017	171.63	69.06	369.29	RW2
Côte d'Ivoire	NORD-OUEST	2018	168.71	55.65	417.86	RW2
Côte d'Ivoire	NORD-OUEST	2019	166.63	43.04	476.51	RW2
Côte d'Ivoire	OUEST	1980	351.80	189.95	540.58	RW2
Côte d'Ivoire	OUEST	1981	335.46	196.79	492.09	RW2
Côte d'Ivoire	OUEST	1982	318.31	196.87	454.51	RW2
Côte d'Ivoire	OUEST	1983	302.11	194.34	424.49	RW2
Côte d'Ivoire	OUEST	1984	287.02	190.70	398.14	RW2
Côte d'Ivoire	OUEST	1985	272.85	187.42	371.92	RW2
Côte d'Ivoire	OUEST	1986	261.42	184.66	349.44	RW2
Côte d'Ivoire	OUEST	1987	252.03	183.31	332.54	RW2
Côte d'Ivoire	OUEST	1988	244.79	181.75	318.03	RW2
Côte d'Ivoire	OUEST	1989	239.50	181.74	307.19	RW2
Côte d'Ivoire	OUEST	1990	236.71	183.30	299.52	RW2
Côte d'Ivoire	OUEST	1991	233.92	184.86	291.13	RW2
Côte d'Ivoire	OUEST	1992	231.44	185.39	285.30	RW2
Côte d'Ivoire	OUEST	1993	229.05	184.97	280.85	RW2
Côte d'Ivoire	OUEST	1994	226.56	183.73	277.31	RW2
Côte d'Ivoire	OUEST	1995	223.86	182.27	271.30	RW2
Côte d'Ivoire	OUEST	1996	220.83	182.98	265.03	RW2
Côte d'Ivoire	OUEST	1997	217.76	181.34	259.02	RW2
Côte d'Ivoire	OUEST	1998	213.96	177.59	255.08	RW2
Côte d'Ivoire	OUEST	1999	209.78	173.15	251.97	RW2
Côte d'Ivoire	OUEST	2000	205.09	169.14	245.97	RW2
Côte d'Ivoire	OUEST	2001	198.93	165.81	237.15	RW2
Côte d'Ivoire	OUEST	2002	191.47	160.24	227.71	RW2
Côte d'Ivoire	OUEST	2003	182.99	151.87	219.54	RW2
Côte d'Ivoire	OUEST	2004	173.81	141.68	211.74	RW2
Côte d'Ivoire	OUEST	2005	163.77	132.07	200.71	RW2
Côte d'Ivoire	OUEST	2006	154.62	124.70	189.64	RW2
Côte d'Ivoire	OUEST	2007	145.97	116.99	180.60	RW2
Côte d'Ivoire	OUEST	2008	138.10	108.55	173.88	RW2
Côte d'Ivoire	OUEST	2009	130.91	99.64	169.89	RW2
Côte d'Ivoire	OUEST	2010	124.78	91.87	166.69	RW2
Côte d'Ivoire	OUEST	2011	118.89	85.93	161.41	RW2
Côte d'Ivoire	OUEST	2012	113.28	79.61	157.68	RW2
Côte d'Ivoire	OUEST	2013	107.93	72.24	157.11	RW2
Côte d'Ivoire	OUEST	2014	102.80	62.99	162.54	RW2
Côte d'Ivoire	OUEST	2015	97.91	51.40	177.09	RW2

Continued on next page

Country	Region	Year	Median	Lower	Upper	Method
Côte d'Ivoire	OUEST	2016	93.41	41.70	195.63	RW2
Côte d'Ivoire	OUEST	2017	88.55	32.51	222.32	RW2
Côte d'Ivoire	OUEST	2018	84.35	24.86	252.53	RW2
Côte d'Ivoire	OUEST	2019	80.32	18.60	294.67	RW2
Côte d'Ivoire	SUD SANS ABIDJAN	1980	215.08	106.20	385.92	RW2
Côte d'Ivoire	SUD SANS ABIDJAN	1981	203.70	109.93	346.09	RW2
Côte d'Ivoire	SUD SANS ABIDJAN	1982	192.76	109.49	313.45	RW2
Côte d'Ivoire	SUD SANS ABIDJAN	1983	182.15	107.77	289.56	RW2
Côte d'Ivoire	SUD SANS ABIDJAN	1984	172.64	105.58	269.71	RW2
Côte d'Ivoire	SUD SANS ABIDJAN	1985	163.42	102.62	249.03	RW2
Côte d'Ivoire	SUD SANS ABIDJAN	1986	156.46	101.38	233.83	RW2
Côte d'Ivoire	SUD SANS ABIDJAN	1987	151.15	100.55	221.18	RW2
Côte d'Ivoire	SUD SANS ABIDJAN	1988	147.19	99.90	210.83	RW2
Côte d'Ivoire	SUD SANS ABIDJAN	1989	144.56	100.33	204.14	RW2
Côte d'Ivoire	SUD SANS ABIDJAN	1990	143.60	101.99	198.12	RW2
Côte d'Ivoire	SUD SANS ABIDJAN	1991	142.97	104.04	193.60	RW2
Côte d'Ivoire	SUD SANS ABIDJAN	1992	142.31	105.58	188.78	RW2
Côte d'Ivoire	SUD SANS ABIDJAN	1993	142.08	106.71	187.04	RW2
Côte d'Ivoire	SUD SANS ABIDJAN	1994	142.02	107.45	185.45	RW2
Côte d'Ivoire	SUD SANS ABIDJAN	1995	141.56	108.35	182.63	RW2
Côte d'Ivoire	SUD SANS ABIDJAN	1996	141.59	110.40	179.27	RW2
Côte d'Ivoire	SUD SANS ABIDJAN	1997	141.25	111.40	177.27	RW2
Côte d'Ivoire	SUD SANS ABIDJAN	1998	140.89	111.39	176.23	RW2
Côte d'Ivoire	SUD SANS ABIDJAN	1999	140.54	110.89	176.34	RW2
Côte d'Ivoire	SUD SANS ABIDJAN	2000	139.84	111.13	174.59	RW2
Côte d'Ivoire	SUD SANS ABIDJAN	2001	138.08	111.34	170.47	RW2
Côte d'Ivoire	SUD SANS ABIDJAN	2002	135.56	109.47	166.67	RW2
Côte d'Ivoire	SUD SANS ABIDJAN	2003	132.14	106.29	163.75	RW2
Côte d'Ivoire	SUD SANS ABIDJAN	2004	128.15	101.91	160.28	RW2
Côte d'Ivoire	SUD SANS ABIDJAN	2005	123.19	97.43	154.30	RW2
Côte d'Ivoire	SUD SANS ABIDJAN	2006	118.85	94.42	148.59	RW2
Côte d'Ivoire	SUD SANS ABIDJAN	2007	114.45	90.59	143.16	RW2
Côte d'Ivoire	SUD SANS ABIDJAN	2008	110.41	85.79	141.18	RW2
Côte d'Ivoire	SUD SANS ABIDJAN	2009	106.95	80.58	140.56	RW2
Côte d'Ivoire	SUD SANS ABIDJAN	2010	103.88	75.82	141.08	RW2
Côte d'Ivoire	SUD SANS ABIDJAN	2011	100.93	72.07	139.33	RW2
Côte d'Ivoire	SUD SANS ABIDJAN	2012	98.00	68.04	138.95	RW2
Côte d'Ivoire	SUD SANS ABIDJAN	2013	95.31	62.81	141.53	RW2
Côte d'Ivoire	SUD SANS ABIDJAN	2014	92.65	55.55	149.29	RW2
Côte d'Ivoire	SUD SANS ABIDJAN	2015	89.96	46.66	166.28	RW2
Côte d'Ivoire	SUD SANS ABIDJAN	2016	87.19	38.51	187.28	RW2
Côte d'Ivoire	SUD SANS ABIDJAN	2017	84.87	30.53	214.95	RW2
Côte d'Ivoire	SUD SANS ABIDJAN	2018	82.16	23.74	248.44	RW2
Côte d'Ivoire	SUD SANS ABIDJAN	2019	79.66	18.28	291.81	RW2
Côte d'Ivoire	SUD-OUEST	1980	224.17	105.08	414.28	RW2
Côte d'Ivoire	SUD-OUEST	1981	209.77	105.66	370.70	RW2
Côte d'Ivoire	SUD-OUEST	1982	196.08	105.67	335.12	RW2
Côte d'Ivoire	SUD-OUEST	1983	183.24	102.72	304.57	RW2
Côte d'Ivoire	SUD-OUEST	1984	171.16	99.06	280.09	RW2
Côte d'Ivoire	SUD-OUEST	1985	160.32	95.78	255.51	RW2
Côte d'Ivoire	SUD-OUEST	1986	151.69	93.56	235.42	RW2
Côte d'Ivoire	SUD-OUEST	1987	144.67	91.38	219.79	RW2
Côte d'Ivoire	SUD-OUEST	1988	139.03	90.00	207.53	RW2
Côte d'Ivoire	SUD-OUEST	1989	134.66	88.86	198.66	RW2
Côte d'Ivoire	SUD-OUEST	1990	131.78	89.13	191.18	RW2
Côte d'Ivoire	SUD-OUEST	1991	129.11	89.10	183.84	RW2
Côte d'Ivoire	SUD-OUEST	1992	127.06	88.73	178.22	RW2
Côte d'Ivoire	SUD-OUEST	1993	124.72	88.03	174.30	RW2
Côte d'Ivoire	SUD-OUEST	1994	122.78	87.49	170.14	RW2
Côte d'Ivoire	SUD-OUEST	1995	120.44	86.85	165.31	RW2
Côte d'Ivoire	SUD-OUEST	1996	118.47	86.42	160.13	RW2
Côte d'Ivoire	SUD-OUEST	1997	116.36	85.84	155.24	RW2
Côte d'Ivoire	SUD-OUEST	1998	114.08	84.38	151.98	RW2

Continued on next page

Country	Region	Year	Median	Lower	Upper	Method
Côte d'Ivoire	SUD-OUEST	1999	111.67	82.91	148.27	RW2
Côte d'Ivoire	SUD-OUEST	2000	109.27	81.45	144.27	RW2
Côte d'Ivoire	SUD-OUEST	2001	105.97	80.37	138.32	RW2
Côte d'Ivoire	SUD-OUEST	2002	102.21	78.13	132.27	RW2
Côte d'Ivoire	SUD-OUEST	2003	97.80	74.72	126.98	RW2
Côte d'Ivoire	SUD-OUEST	2004	93.15	70.60	122.23	RW2
Côte d'Ivoire	SUD-OUEST	2005	88.06	66.56	115.38	RW2
Côte d'Ivoire	SUD-OUEST	2006	83.45	63.57	109.12	RW2
Côte d'Ivoire	SUD-OUEST	2007	79.11	59.99	103.49	RW2
Côte d'Ivoire	SUD-OUEST	2008	75.24	56.01	100.61	RW2
Côte d'Ivoire	SUD-OUEST	2009	71.74	51.84	98.70	RW2
Côte d'Ivoire	SUD-OUEST	2010	68.77	47.95	98.47	RW2
Côte d'Ivoire	SUD-OUEST	2011	65.80	44.74	96.37	RW2
Côte d'Ivoire	SUD-OUEST	2012	63.14	41.52	95.48	RW2
Côte d'Ivoire	SUD-OUEST	2013	60.60	37.63	97.01	RW2
Côte d'Ivoire	SUD-OUEST	2014	58.18	32.96	101.90	RW2
Côte d'Ivoire	SUD-OUEST	2015	55.62	27.13	111.61	RW2
Côte d'Ivoire	SUD-OUEST	2016	53.33	22.14	125.19	RW2
Côte d'Ivoire	SUD-OUEST	2017	51.22	17.57	143.59	RW2
Côte d'Ivoire	SUD-OUEST	2018	49.12	13.65	168.29	RW2
Côte d'Ivoire	SUD-OUEST	2019	47.03	10.03	200.27	RW2
Côte d'Ivoire	VILLE D'ABIDJAN	1980	170.32	77.28	346.89	RW2
Côte d'Ivoire	VILLE D'ABIDJAN	1981	160.61	79.98	305.05	RW2
Côte d'Ivoire	VILLE D'ABIDJAN	1982	150.85	80.86	272.29	RW2
Côte d'Ivoire	VILLE D'ABIDJAN	1983	141.92	79.76	244.95	RW2
Côte d'Ivoire	VILLE D'ABIDJAN	1984	133.78	77.94	223.93	RW2
Côte d'Ivoire	VILLE D'ABIDJAN	1985	126.14	76.39	203.26	RW2
Côte d'Ivoire	VILLE D'ABIDJAN	1986	120.08	76.09	187.36	RW2
Côte d'Ivoire	VILLE D'ABIDJAN	1987	115.29	75.07	174.60	RW2
Côte d'Ivoire	VILLE D'ABIDJAN	1988	111.97	74.21	165.87	RW2
Côte d'Ivoire	VILLE D'ABIDJAN	1989	109.66	74.56	158.98	RW2
Côte d'Ivoire	VILLE D'ABIDJAN	1990	108.51	75.71	153.84	RW2
Côte d'Ivoire	VILLE D'ABIDJAN	1991	107.48	76.59	149.13	RW2
Côte d'Ivoire	VILLE D'ABIDJAN	1992	106.80	77.48	145.96	RW2
Côte d'Ivoire	VILLE D'ABIDJAN	1993	106.61	77.95	143.99	RW2
Côte d'Ivoire	VILLE D'ABIDJAN	1994	106.51	78.52	143.05	RW2
Côte d'Ivoire	VILLE D'ABIDJAN	1995	106.12	79.01	140.54	RW2
Côte d'Ivoire	VILLE D'ABIDJAN	1996	106.22	80.55	138.14	RW2
Côte d'Ivoire	VILLE D'ABIDJAN	1997	106.16	81.11	137.28	RW2
Côte d'Ivoire	VILLE D'ABIDJAN	1998	106.36	81.20	137.17	RW2
Côte d'Ivoire	VILLE D'ABIDJAN	1999	106.45	80.89	137.73	RW2
Côte d'Ivoire	VILLE D'ABIDJAN	2000	106.41	81.19	137.45	RW2
Côte d'Ivoire	VILLE D'ABIDJAN	2001	105.84	81.63	135.42	RW2
Côte d'Ivoire	VILLE D'ABIDJAN	2002	104.67	81.08	133.25	RW2
Côte d'Ivoire	VILLE D'ABIDJAN	2003	102.92	79.55	131.58	RW2
Côte d'Ivoire	VILLE D'ABIDJAN	2004	100.86	77.73	129.94	RW2
Côte d'Ivoire	VILLE D'ABIDJAN	2005	98.07	75.05	127.11	RW2
Côte d'Ivoire	VILLE D'ABIDJAN	2006	95.68	73.79	123.10	RW2
Côte d'Ivoire	VILLE D'ABIDJAN	2007	93.35	71.91	120.57	RW2
Côte d'Ivoire	VILLE D'ABIDJAN	2008	91.28	68.84	120.24	RW2
Côte d'Ivoire	VILLE D'ABIDJAN	2009	89.46	65.68	121.18	RW2
Côte d'Ivoire	VILLE D'ABIDJAN	2010	87.93	62.50	123.70	RW2
Côte d'Ivoire	VILLE D'ABIDJAN	2011	86.55	59.86	124.62	RW2
Côte d'Ivoire	VILLE D'ABIDJAN	2012	85.21	57.15	126.41	RW2
Côte d'Ivoire	VILLE D'ABIDJAN	2013	83.86	53.12	131.27	RW2
Côte d'Ivoire	VILLE D'ABIDJAN	2014	82.52	47.46	141.62	RW2
Côte d'Ivoire	VILLE D'ABIDJAN	2015	81.18	40.33	159.93	RW2
Côte d'Ivoire	VILLE D'ABIDJAN	2016	80.18	33.09	182.73	RW2
Côte d'Ivoire	VILLE D'ABIDJAN	2017	78.50	26.94	213.21	RW2
Côte d'Ivoire	VILLE D'ABIDJAN	2018	77.22	21.26	250.19	RW2
Côte d'Ivoire	VILLE D'ABIDJAN	2019	76.37	16.22	296.78	RW2
DRC	ALL	1980	185.30	167.38	206.56	IHME
DRC	ALL	1980	214.12	143.93	307.10	RW2

Continued on next page

Country	Region	Year	Median	Lower	Upper	Method
DRC	ALL	1980	212.00	184.00	242.60	UN
DRC	ALL	1981	182.37	164.97	201.67	IHME
DRC	ALL	1981	210.21	154.92	278.80	RW2
DRC	ALL	1981	208.80	182.30	237.40	UN
DRC	ALL	1982	178.65	163.24	196.21	IHME
DRC	ALL	1982	206.47	159.95	262.56	RW2
DRC	ALL	1982	205.70	180.50	232.50	UN
DRC	ALL	1983	173.80	158.27	188.44	IHME
DRC	ALL	1983	202.42	158.58	255.21	RW2
DRC	ALL	1983	202.80	178.90	228.20	UN
DRC	ALL	1984	166.76	153.41	180.69	IHME
DRC	ALL	1984	199.14	154.80	252.02	RW2
DRC	ALL	1984	200.10	177.20	223.90	UN
DRC	ALL	1985	162.34	150.44	176.52	IHME
DRC	ALL	1985	195.28	153.84	244.07	RW2
DRC	ALL	1985	197.70	175.70	220.30	UN
DRC	ALL	1986	159.41	147.86	172.17	IHME
DRC	ALL	1986	192.49	153.20	238.22	RW2
DRC	ALL	1986	195.30	174.20	216.70	UN
DRC	ALL	1987	159.06	147.29	171.15	IHME
DRC	ALL	1987	190.32	152.91	234.07	RW2
DRC	ALL	1987	193.00	172.90	213.30	UN
DRC	ALL	1988	160.82	149.39	172.78	IHME
DRC	ALL	1988	188.46	150.92	231.83	RW2
DRC	ALL	1988	190.80	171.70	210.40	UN
DRC	ALL	1989	163.58	151.82	176.31	IHME
DRC	ALL	1989	187.07	149.52	230.55	RW2
DRC	ALL	1989	188.50	170.40	207.60	UN
DRC	ALL	1990	166.62	154.87	178.44	IHME
DRC	ALL	1990	186.30	150.62	228.72	RW2
DRC	ALL	1990	186.50	169.10	204.70	UN
DRC	ALL	1991	167.40	155.46	179.60	IHME
DRC	ALL	1991	185.19	151.37	224.35	RW2
DRC	ALL	1991	184.30	167.30	202.00	UN
DRC	ALL	1992	166.23	154.71	178.44	IHME
DRC	ALL	1992	183.92	150.98	221.90	RW2
DRC	ALL	1992	182.30	165.90	199.50	UN
DRC	ALL	1993	165.32	153.73	177.38	IHME
DRC	ALL	1993	182.39	149.27	221.04	RW2
DRC	ALL	1993	180.30	164.50	197.30	UN
DRC	ALL	1994	164.57	153.03	176.94	IHME
DRC	ALL	1994	180.38	146.42	221.02	RW2
DRC	ALL	1994	178.30	162.90	195.30	UN
DRC	ALL	1995	164.58	152.23	176.41	IHME
DRC	ALL	1995	178.07	145.14	216.57	RW2
DRC	ALL	1995	176.40	161.20	193.30	UN
DRC	ALL	1996	166.48	154.69	178.94	IHME
DRC	ALL	1996	175.25	144.24	211.87	RW2
DRC	ALL	1996	174.10	159.00	191.10	UN
DRC	ALL	1997	164.22	152.62	175.57	IHME
DRC	ALL	1997	172.09	142.39	206.67	RW2
DRC	ALL	1997	171.50	156.50	188.70	UN
DRC	ALL	1998	161.33	150.49	172.82	IHME
DRC	ALL	1998	168.67	138.74	204.39	RW2
DRC	ALL	1998	168.40	153.50	185.70	UN
DRC	ALL	1999	157.09	145.78	168.19	IHME
DRC	ALL	1999	164.87	134.17	200.63	RW2
DRC	ALL	1999	164.90	150.20	181.90	UN
DRC	ALL	2000	151.83	140.18	163.69	IHME
DRC	ALL	2000	160.75	131.23	195.10	RW2
DRC	ALL	2000	161.00	146.50	177.80	UN
DRC	ALL	2001	147.58	136.66	159.57	IHME
DRC	ALL	2001	156.51	128.85	188.60	RW2

Continued on next page

Country	Region	Year	Median	Lower	Upper	Method
DRC	ALL	2001	156.80	142.50	173.10	UN
DRC	ALL	2002	143.83	133.08	155.57	IHME
DRC	ALL	2002	152.08	126.02	182.49	RW2
DRC	ALL	2002	152.40	138.30	168.20	UN
DRC	ALL	2003	138.43	128.24	149.22	IHME
DRC	ALL	2003	147.66	121.83	177.95	RW2
DRC	ALL	2003	147.80	133.90	163.10	UN
DRC	ALL	2004	132.63	122.08	142.97	IHME
DRC	ALL	2004	142.94	116.09	174.53	RW2
DRC	ALL	2004	143.20	129.30	158.50	UN
DRC	ALL	2005	128.84	118.02	139.01	IHME
DRC	ALL	2005	138.45	112.14	169.80	RW2
DRC	ALL	2005	138.40	124.40	153.90	UN
DRC	ALL	2006	125.91	115.74	137.62	IHME
DRC	ALL	2006	133.81	109.44	162.47	RW2
DRC	ALL	2006	133.50	119.40	149.80	UN
DRC	ALL	2007	121.97	111.49	133.14	IHME
DRC	ALL	2007	129.27	106.42	156.11	RW2
DRC	ALL	2007	128.90	114.00	146.30	UN
DRC	ALL	2008	116.64	106.11	128.71	IHME
DRC	ALL	2008	124.93	102.01	152.26	RW2
DRC	ALL	2008	124.50	108.20	143.00	UN
DRC	ALL	2009	111.73	101.21	122.96	IHME
DRC	ALL	2009	120.42	96.66	149.52	RW2
DRC	ALL	2009	120.10	102.70	140.10	UN
DRC	ALL	2010	107.20	96.35	118.74	IHME
DRC	ALL	2010	116.09	91.87	146.38	RW2
DRC	ALL	2010	116.10	96.80	137.60	UN
DRC	ALL	2011	103.57	93.48	114.68	IHME
DRC	ALL	2011	111.97	89.52	139.17	RW2
DRC	ALL	2011	112.20	91.40	135.40	UN
DRC	ALL	2012	100.15	89.88	112.15	IHME
DRC	ALL	2012	107.88	87.45	132.28	RW2
DRC	ALL	2012	108.50	86.10	133.00	UN
DRC	ALL	2013	96.70	84.40	109.85	IHME
DRC	ALL	2013	104.00	82.46	130.16	RW2
DRC	ALL	2013	104.80	81.00	131.30	UN
DRC	ALL	2014	92.43	77.08	109.25	IHME
DRC	ALL	2014	100.15	72.24	137.07	RW2
DRC	ALL	2014	101.70	76.10	130.30	UN
DRC	ALL	2015	88.04	70.19	108.92	IHME
DRC	ALL	2015	96.32	58.41	154.94	RW2
DRC	ALL	2015	98.30	71.30	129.80	UN
DRC	ALL	2016	92.94	47.14	176.12	RW2
DRC	ALL	2017	89.27	36.65	203.26	RW2
DRC	ALL	2018	85.90	28.16	238.81	RW2
DRC	ALL	2019	82.53	20.38	277.54	RW2
DRC	ALL	15-19	89.28	37.24	199.95	RW2
DRC	BANDUNDU	1980	249.40	163.10	359.84	RW2
DRC	BANDUNDU	1981	243.97	171.48	333.15	RW2
DRC	BANDUNDU	1982	238.31	173.10	317.26	RW2
DRC	BANDUNDU	1983	232.43	170.68	307.65	RW2
DRC	BANDUNDU	1984	226.56	167.64	298.32	RW2
DRC	BANDUNDU	1985	220.80	165.14	288.37	RW2
DRC	BANDUNDU	1986	214.55	162.74	277.05	RW2
DRC	BANDUNDU	1987	207.93	160.15	265.63	RW2
DRC	BANDUNDU	1988	201.06	155.83	255.85	RW2
DRC	BANDUNDU	1989	194.24	151.61	246.26	RW2
DRC	BANDUNDU	1990	187.03	147.82	233.92	RW2
DRC	BANDUNDU	1991	181.52	145.77	224.04	RW2
DRC	BANDUNDU	1992	176.92	143.26	216.34	RW2
DRC	BANDUNDU	1993	173.16	140.60	211.10	RW2
DRC	BANDUNDU	1994	170.12	137.33	208.32	RW2

Continued on next page

Country	Region	Year	Median	Lower	Upper	Method
DRC	BANDUNDU	1995	168.19	136.94	205.54	RW2
DRC	BANDUNDU	1996	165.42	136.04	199.99	RW2
DRC	BANDUNDU	1997	162.08	134.26	194.36	RW2
DRC	BANDUNDU	1998	157.92	130.59	190.35	RW2
DRC	BANDUNDU	1999	152.91	125.21	185.89	RW2
DRC	BANDUNDU	2000	146.93	120.02	177.84	RW2
DRC	BANDUNDU	2001	140.59	116.42	168.66	RW2
DRC	BANDUNDU	2002	133.89	111.39	160.17	RW2
DRC	BANDUNDU	2003	126.87	104.98	152.97	RW2
DRC	BANDUNDU	2004	120.01	98.03	146.39	RW2
DRC	BANDUNDU	2005	113.20	91.51	139.02	RW2
DRC	BANDUNDU	2006	106.95	86.95	130.84	RW2
DRC	BANDUNDU	2007	101.17	82.27	123.72	RW2
DRC	BANDUNDU	2008	96.01	77.11	118.86	RW2
DRC	BANDUNDU	2009	91.23	71.90	115.12	RW2
DRC	BANDUNDU	2010	87.19	67.20	112.34	RW2
DRC	BANDUNDU	2011	83.12	64.30	107.06	RW2
DRC	BANDUNDU	2012	79.47	61.33	102.29	RW2
DRC	BANDUNDU	2013	75.89	56.80	100.60	RW2
DRC	BANDUNDU	2014	72.51	50.09	103.28	RW2
DRC	BANDUNDU	2015	69.25	41.44	113.88	RW2
DRC	BANDUNDU	2016	66.10	33.77	126.39	RW2
DRC	BANDUNDU	2017	63.15	26.61	144.30	RW2
DRC	BANDUNDU	2018	60.11	20.23	166.09	RW2
DRC	BANDUNDU	2019	57.29	15.47	193.01	RW2
DRC	BAS-CONGO	1980	266.73	165.19	396.33	RW2
DRC	BAS-CONGO	1981	262.27	174.53	370.85	RW2
DRC	BAS-CONGO	1982	257.97	179.85	354.67	RW2
DRC	BAS-CONGO	1983	253.95	181.12	343.42	RW2
DRC	BAS-CONGO	1984	249.80	179.95	333.56	RW2
DRC	BAS-CONGO	1985	245.46	181.48	323.60	RW2
DRC	BAS-CONGO	1986	240.53	181.10	312.05	RW2
DRC	BAS-CONGO	1987	235.25	180.27	301.29	RW2
DRC	BAS-CONGO	1988	229.65	177.42	291.94	RW2
DRC	BAS-CONGO	1989	223.68	174.81	283.69	RW2
DRC	BAS-CONGO	1990	217.79	171.78	271.41	RW2
DRC	BAS-CONGO	1991	213.28	170.68	262.98	RW2
DRC	BAS-CONGO	1992	209.92	170.13	255.78	RW2
DRC	BAS-CONGO	1993	207.35	168.15	252.66	RW2
DRC	BAS-CONGO	1994	205.64	166.51	250.40	RW2
DRC	BAS-CONGO	1995	205.14	167.86	248.78	RW2
DRC	BAS-CONGO	1996	203.70	169.47	244.20	RW2
DRC	BAS-CONGO	1997	201.61	168.44	239.18	RW2
DRC	BAS-CONGO	1998	198.25	165.46	235.90	RW2
DRC	BAS-CONGO	1999	194.00	160.32	232.00	RW2
DRC	BAS-CONGO	2000	188.48	155.80	225.70	RW2
DRC	BAS-CONGO	2001	182.26	152.21	216.62	RW2
DRC	BAS-CONGO	2002	175.57	147.22	208.37	RW2
DRC	BAS-CONGO	2003	168.49	139.98	201.13	RW2
DRC	BAS-CONGO	2004	161.19	131.75	195.75	RW2
DRC	BAS-CONGO	2005	153.96	124.67	188.48	RW2
DRC	BAS-CONGO	2006	147.22	119.17	180.12	RW2
DRC	BAS-CONGO	2007	141.12	113.84	173.88	RW2
DRC	BAS-CONGO	2008	135.46	107.53	169.74	RW2
DRC	BAS-CONGO	2009	130.59	100.74	167.31	RW2
DRC	BAS-CONGO	2010	126.27	95.67	165.37	RW2
DRC	BAS-CONGO	2011	122.20	91.92	160.47	RW2
DRC	BAS-CONGO	2012	118.30	88.49	157.13	RW2
DRC	BAS-CONGO	2013	114.52	82.56	156.35	RW2
DRC	BAS-CONGO	2014	110.85	74.27	162.90	RW2
DRC	BAS-CONGO	2015	107.40	62.19	179.35	RW2
DRC	BAS-CONGO	2016	103.71	51.92	198.13	RW2
DRC	BAS-CONGO	2017	100.28	41.23	224.56	RW2

Continued on next page

Country	Region	Year	Median	Lower	Upper	Method
DRC	BAS-CONGO	2018	97.13	32.47	255.93	RW2
DRC	BAS-CONGO	2019	94.01	24.78	293.48	RW2
DRC	EQUATEUR	1980	191.16	120.63	289.49	RW2
DRC	EQUATEUR	1981	190.53	130.27	270.81	RW2
DRC	EQUATEUR	1982	189.79	134.47	260.35	RW2
DRC	EQUATEUR	1983	189.25	137.04	255.51	RW2
DRC	EQUATEUR	1984	188.42	137.75	252.26	RW2
DRC	EQUATEUR	1985	187.70	140.03	247.25	RW2
DRC	EQUATEUR	1986	186.16	141.16	240.94	RW2
DRC	EQUATEUR	1987	184.20	141.80	236.16	RW2
DRC	EQUATEUR	1988	181.87	141.21	231.64	RW2
DRC	EQUATEUR	1989	179.55	139.85	228.03	RW2
DRC	EQUATEUR	1990	176.85	138.76	222.25	RW2
DRC	EQUATEUR	1991	175.53	139.73	218.56	RW2
DRC	EQUATEUR	1992	174.98	140.48	215.18	RW2
DRC	EQUATEUR	1993	175.32	140.91	215.29	RW2
DRC	EQUATEUR	1994	176.46	141.21	217.25	RW2
DRC	EQUATEUR	1995	178.88	144.94	219.09	RW2
DRC	EQUATEUR	1996	180.27	147.92	217.69	RW2
DRC	EQUATEUR	1997	181.25	150.01	216.60	RW2
DRC	EQUATEUR	1998	181.07	149.73	216.82	RW2
DRC	EQUATEUR	1999	179.98	147.37	217.85	RW2
DRC	EQUATEUR	2000	177.63	145.56	214.13	RW2
DRC	EQUATEUR	2001	174.55	144.67	208.10	RW2
DRC	EQUATEUR	2002	170.82	142.43	203.17	RW2
DRC	EQUATEUR	2003	166.49	138.37	198.98	RW2
DRC	EQUATEUR	2004	162.08	133.18	196.02	RW2
DRC	EQUATEUR	2005	157.22	128.21	190.53	RW2
DRC	EQUATEUR	2006	152.97	125.66	184.65	RW2
DRC	EQUATEUR	2007	148.82	122.24	179.92	RW2
DRC	EQUATEUR	2008	145.20	118.09	177.22	RW2
DRC	EQUATEUR	2009	142.31	113.53	176.88	RW2
DRC	EQUATEUR	2010	139.73	109.43	176.46	RW2
DRC	EQUATEUR	2011	137.26	108.26	173.12	RW2
DRC	EQUATEUR	2012	134.95	106.36	170.33	RW2
DRC	EQUATEUR	2013	132.75	101.73	171.20	RW2
DRC	EQUATEUR	2014	130.71	92.77	180.47	RW2
DRC	EQUATEUR	2015	128.43	79.84	201.70	RW2
DRC	EQUATEUR	2016	126.19	66.70	225.99	RW2
DRC	EQUATEUR	2017	124.31	54.67	258.07	RW2
DRC	EQUATEUR	2018	121.56	43.80	298.81	RW2
DRC	EQUATEUR	2019	119.51	34.12	345.04	RW2
DRC	KASAI-OCCIDENTAL	1980	265.89	173.36	384.63	RW2
DRC	KASAI-OCCIDENTAL	1981	262.14	183.67	359.06	RW2
DRC	KASAI-OCCIDENTAL	1982	258.35	188.48	342.82	RW2
DRC	KASAI-OCCIDENTAL	1983	254.47	189.08	333.79	RW2
DRC	KASAI-OCCIDENTAL	1984	250.31	187.66	325.59	RW2
DRC	KASAI-OCCIDENTAL	1985	246.37	188.23	315.91	RW2
DRC	KASAI-OCCIDENTAL	1986	241.67	187.44	304.99	RW2
DRC	KASAI-OCCIDENTAL	1987	236.12	186.04	294.80	RW2
DRC	KASAI-OCCIDENTAL	1988	230.87	183.16	286.58	RW2
DRC	KASAI-OCCIDENTAL	1989	225.34	179.89	279.14	RW2
DRC	KASAI-OCCIDENTAL	1990	219.48	177.09	267.97	RW2
DRC	KASAI-OCCIDENTAL	1991	215.17	176.38	259.87	RW2
DRC	KASAI-OCCIDENTAL	1992	212.05	175.49	253.49	RW2
DRC	KASAI-OCCIDENTAL	1993	210.10	174.24	250.43	RW2
DRC	KASAI-OCCIDENTAL	1994	208.90	172.37	249.57	RW2
DRC	KASAI-OCCIDENTAL	1995	209.04	174.28	248.90	RW2
DRC	KASAI-OCCIDENTAL	1996	208.15	175.81	244.48	RW2
DRC	KASAI-OCCIDENTAL	1997	206.57	175.51	241.42	RW2
DRC	KASAI-OCCIDENTAL	1998	203.97	173.03	239.13	RW2
DRC	KASAI-OCCIDENTAL	1999	200.23	167.57	237.39	RW2
DRC	KASAI-OCCIDENTAL	2000	194.98	162.43	231.29	RW2

Continued on next page

Country	Region	Year	Median	Lower	Upper	Method
DRC	KASAI-OCCIDENTAL	2001	189.14	158.93	223.33	RW2
DRC	KASAI-OCCIDENTAL	2002	182.36	153.70	214.96	RW2
DRC	KASAI-OCCIDENTAL	2003	175.45	146.49	209.43	RW2
DRC	KASAI-OCCIDENTAL	2004	167.98	138.07	203.42	RW2
DRC	KASAI-OCCIDENTAL	2005	160.42	130.86	194.78	RW2
DRC	KASAI-OCCIDENTAL	2006	153.42	125.45	186.08	RW2
DRC	KASAI-OCCIDENTAL	2007	147.01	119.63	179.01	RW2
DRC	KASAI-OCCIDENTAL	2008	140.99	112.98	174.40	RW2
DRC	KASAI-OCCIDENTAL	2009	135.54	106.10	171.43	RW2
DRC	KASAI-OCCIDENTAL	2010	130.63	100.28	169.05	RW2
DRC	KASAI-OCCIDENTAL	2011	126.15	95.87	163.13	RW2
DRC	KASAI-OCCIDENTAL	2012	121.64	92.20	159.04	RW2
DRC	KASAI-OCCIDENTAL	2013	117.46	86.44	157.95	RW2
DRC	KASAI-OCCIDENTAL	2014	113.24	77.12	163.20	RW2
DRC	KASAI-OCCIDENTAL	2015	109.39	64.96	179.04	RW2
DRC	KASAI-OCCIDENTAL	2016	105.71	53.71	198.07	RW2
DRC	KASAI-OCCIDENTAL	2017	101.71	43.00	221.78	RW2
DRC	KASAI-OCCIDENTAL	2018	97.78	33.88	252.61	RW2
DRC	KASAI-OCCIDENTAL	2019	94.45	25.96	294.14	RW2
DRC	KASAI-ORIENTAL	1980	208.34	135.63	306.19	RW2
DRC	KASAI-ORIENTAL	1981	206.56	145.87	284.73	RW2
DRC	KASAI-ORIENTAL	1982	204.80	150.55	272.72	RW2
DRC	KASAI-ORIENTAL	1983	202.71	152.13	265.39	RW2
DRC	KASAI-ORIENTAL	1984	200.63	152.05	260.56	RW2
DRC	KASAI-ORIENTAL	1985	198.80	153.38	253.10	RW2
DRC	KASAI-ORIENTAL	1986	195.84	154.33	245.41	RW2
DRC	KASAI-ORIENTAL	1987	192.59	153.82	238.72	RW2
DRC	KASAI-ORIENTAL	1988	189.20	152.07	233.51	RW2
DRC	KASAI-ORIENTAL	1989	185.69	149.57	228.67	RW2
DRC	KASAI-ORIENTAL	1990	181.73	148.03	221.44	RW2
DRC	KASAI-ORIENTAL	1991	179.36	147.66	215.78	RW2
DRC	KASAI-ORIENTAL	1992	177.77	147.82	211.84	RW2
DRC	KASAI-ORIENTAL	1993	177.26	147.41	211.12	RW2
DRC	KASAI-ORIENTAL	1994	177.44	146.55	211.95	RW2
DRC	KASAI-ORIENTAL	1995	178.76	149.75	212.86	RW2
DRC	KASAI-ORIENTAL	1996	179.22	151.93	210.63	RW2
DRC	KASAI-ORIENTAL	1997	179.15	152.86	208.88	RW2
DRC	KASAI-ORIENTAL	1998	178.12	150.73	208.65	RW2
DRC	KASAI-ORIENTAL	1999	175.89	147.31	208.07	RW2
DRC	KASAI-ORIENTAL	2000	172.54	144.42	204.26	RW2
DRC	KASAI-ORIENTAL	2001	168.39	141.82	198.32	RW2
DRC	KASAI-ORIENTAL	2002	163.65	138.58	192.44	RW2
DRC	KASAI-ORIENTAL	2003	158.19	132.60	187.66	RW2
DRC	KASAI-ORIENTAL	2004	152.57	126.32	183.59	RW2
DRC	KASAI-ORIENTAL	2005	146.57	120.27	177.11	RW2
DRC	KASAI-ORIENTAL	2006	141.14	116.57	169.89	RW2
DRC	KASAI-ORIENTAL	2007	136.03	112.14	164.08	RW2
DRC	KASAI-ORIENTAL	2008	131.41	106.82	160.47	RW2
DRC	KASAI-ORIENTAL	2009	127.22	101.19	158.48	RW2
DRC	KASAI-ORIENTAL	2010	123.48	96.63	156.89	RW2
DRC	KASAI-ORIENTAL	2011	120.04	93.90	152.32	RW2
DRC	KASAI-ORIENTAL	2012	116.63	91.48	147.88	RW2
DRC	KASAI-ORIENTAL	2013	113.40	86.57	147.73	RW2
DRC	KASAI-ORIENTAL	2014	110.21	78.06	153.59	RW2
DRC	KASAI-ORIENTAL	2015	107.17	65.73	170.16	RW2
DRC	KASAI-ORIENTAL	2016	104.02	54.27	190.29	RW2
DRC	KASAI-ORIENTAL	2017	101.15	43.75	219.82	RW2
DRC	KASAI-ORIENTAL	2018	98.33	34.45	251.56	RW2
DRC	KASAI-ORIENTAL	2019	95.69	26.96	289.49	RW2
DRC	KATANGA	1980	212.80	137.43	312.52	RW2
DRC	KATANGA	1981	211.19	147.52	293.02	RW2
DRC	KATANGA	1982	210.20	152.45	281.12	RW2
DRC	KATANGA	1983	208.72	154.82	275.53	RW2

Continued on next page

Country	Region	Year	Median	Lower	Upper	Method
DRC	KATANGA	1984	207.02	154.86	270.48	RW2
DRC	KATANGA	1985	205.53	156.86	264.42	RW2
DRC	KATANGA	1986	203.19	158.18	257.58	RW2
DRC	KATANGA	1987	200.59	158.09	250.88	RW2
DRC	KATANGA	1988	197.72	157.40	246.06	RW2
DRC	KATANGA	1989	194.67	155.41	241.93	RW2
DRC	KATANGA	1990	191.08	154.10	233.98	RW2
DRC	KATANGA	1991	189.19	155.23	228.53	RW2
DRC	KATANGA	1992	188.13	155.28	225.32	RW2
DRC	KATANGA	1993	188.16	155.69	225.17	RW2
DRC	KATANGA	1994	188.75	154.90	226.62	RW2
DRC	KATANGA	1995	190.83	158.23	228.67	RW2
DRC	KATANGA	1996	191.64	161.18	226.73	RW2
DRC	KATANGA	1997	191.89	162.17	225.29	RW2
DRC	KATANGA	1998	191.03	160.97	225.36	RW2
DRC	KATANGA	1999	188.96	157.94	224.47	RW2
DRC	KATANGA	2000	185.60	154.92	220.45	RW2
DRC	KATANGA	2001	181.37	152.97	213.52	RW2
DRC	KATANGA	2002	176.22	149.86	206.69	RW2
DRC	KATANGA	2003	170.58	143.64	201.75	RW2
DRC	KATANGA	2004	164.65	137.52	196.78	RW2
DRC	KATANGA	2005	158.27	131.16	189.50	RW2
DRC	KATANGA	2006	152.54	127.70	181.39	RW2
DRC	KATANGA	2007	147.14	123.63	174.42	RW2
DRC	KATANGA	2008	142.17	118.22	169.87	RW2
DRC	KATANGA	2009	137.77	112.56	167.33	RW2
DRC	KATANGA	2010	133.80	108.18	165.02	RW2
DRC	KATANGA	2011	130.11	106.37	158.09	RW2
DRC	KATANGA	2012	126.55	104.81	151.93	RW2
DRC	KATANGA	2013	123.20	99.81	150.72	RW2
DRC	KATANGA	2014	119.82	89.45	158.71	RW2
DRC	KATANGA	2015	116.40	74.58	177.17	RW2
DRC	KATANGA	2016	113.14	61.55	198.43	RW2
DRC	KATANGA	2017	109.87	49.73	226.42	RW2
DRC	KATANGA	2018	106.70	38.76	262.21	RW2
DRC	KATANGA	2019	103.84	29.86	304.82	RW2
DRC	KINSHASA	1980	132.20	81.86	209.23	RW2
DRC	KINSHASA	1981	130.46	87.95	191.93	RW2
DRC	KINSHASA	1982	129.08	90.84	182.26	RW2
DRC	KINSHASA	1983	127.22	91.43	176.23	RW2
DRC	KINSHASA	1984	125.51	91.57	171.06	RW2
DRC	KINSHASA	1985	123.85	92.04	165.74	RW2
DRC	KINSHASA	1986	121.70	92.18	159.66	RW2
DRC	KINSHASA	1987	119.51	92.35	154.62	RW2
DRC	KINSHASA	1988	117.07	91.10	150.26	RW2
DRC	KINSHASA	1989	114.69	89.77	146.20	RW2
DRC	KINSHASA	1990	112.17	89.04	140.16	RW2
DRC	KINSHASA	1991	110.60	89.30	136.29	RW2
DRC	KINSHASA	1992	109.64	89.64	133.54	RW2
DRC	KINSHASA	1993	109.40	89.03	133.34	RW2
DRC	KINSHASA	1994	109.72	88.90	133.84	RW2
DRC	KINSHASA	1995	110.84	90.66	135.17	RW2
DRC	KINSHASA	1996	111.50	92.54	134.28	RW2
DRC	KINSHASA	1997	111.76	93.32	133.45	RW2
DRC	KINSHASA	1998	111.60	92.64	133.62	RW2
DRC	KINSHASA	1999	110.77	91.02	134.08	RW2
DRC	KINSHASA	2000	109.08	89.64	131.84	RW2
DRC	KINSHASA	2001	107.05	88.83	128.13	RW2
DRC	KINSHASA	2002	104.65	87.41	124.91	RW2
DRC	KINSHASA	2003	101.86	84.31	122.33	RW2
DRC	KINSHASA	2004	99.03	80.88	120.83	RW2
DRC	KINSHASA	2005	96.03	77.78	117.73	RW2
DRC	KINSHASA	2006	93.52	76.16	113.95	RW2

Continued on next page

Country	Region	Year	Median	Lower	Upper	Method
DRC	KINSHASA	2007	91.12	74.42	111.26	RW2
DRC	KINSHASA	2008	89.23	71.84	110.15	RW2
DRC	KINSHASA	2009	87.53	69.04	110.43	RW2
DRC	KINSHASA	2010	86.40	67.01	111.05	RW2
DRC	KINSHASA	2011	85.16	66.35	108.73	RW2
DRC	KINSHASA	2012	84.08	65.80	107.79	RW2
DRC	KINSHASA	2013	83.10	63.02	109.45	RW2
DRC	KINSHASA	2014	82.11	57.33	116.77	RW2
DRC	KINSHASA	2015	81.11	49.03	131.86	RW2
DRC	KINSHASA	2016	80.14	40.94	151.55	RW2
DRC	KINSHASA	2017	79.12	33.62	177.09	RW2
DRC	KINSHASA	2018	77.92	27.19	207.67	RW2
DRC	KINSHASA	2019	77.11	21.18	247.78	RW2
DRC	MANIEMA	1980	186.41	115.09	285.23	RW2
DRC	MANIEMA	1981	186.92	125.07	267.21	RW2
DRC	MANIEMA	1982	186.94	130.17	258.21	RW2
DRC	MANIEMA	1983	187.00	132.77	254.43	RW2
DRC	MANIEMA	1984	186.87	134.51	251.71	RW2
DRC	MANIEMA	1985	186.92	137.79	247.86	RW2
DRC	MANIEMA	1986	186.17	139.79	242.01	RW2
DRC	MANIEMA	1987	184.91	141.57	237.72	RW2
DRC	MANIEMA	1988	183.33	142.01	233.25	RW2
DRC	MANIEMA	1989	181.70	142.64	229.55	RW2
DRC	MANIEMA	1990	179.91	142.60	223.72	RW2
DRC	MANIEMA	1991	179.38	144.79	219.53	RW2
DRC	MANIEMA	1992	179.69	146.68	218.00	RW2
DRC	MANIEMA	1993	180.94	148.25	218.73	RW2
DRC	MANIEMA	1994	182.97	149.79	221.25	RW2
DRC	MANIEMA	1995	186.29	153.47	224.36	RW2
DRC	MANIEMA	1996	188.33	157.77	224.37	RW2
DRC	MANIEMA	1997	189.78	159.63	224.05	RW2
DRC	MANIEMA	1998	189.83	158.92	225.06	RW2
DRC	MANIEMA	1999	188.75	156.82	226.22	RW2
DRC	MANIEMA	2000	186.09	153.47	222.98	RW2
DRC	MANIEMA	2001	182.54	152.08	217.79	RW2
DRC	MANIEMA	2002	177.93	149.05	211.81	RW2
DRC	MANIEMA	2003	172.67	143.83	206.84	RW2
DRC	MANIEMA	2004	167.01	137.19	202.27	RW2
DRC	MANIEMA	2005	160.85	131.61	195.07	RW2
DRC	MANIEMA	2006	155.22	127.77	187.22	RW2
DRC	MANIEMA	2007	149.82	123.44	180.96	RW2
DRC	MANIEMA	2008	144.86	117.97	176.74	RW2
DRC	MANIEMA	2009	140.25	111.65	174.88	RW2
DRC	MANIEMA	2010	136.38	106.39	173.19	RW2
DRC	MANIEMA	2011	132.53	103.44	168.17	RW2
DRC	MANIEMA	2012	128.81	100.18	163.80	RW2
DRC	MANIEMA	2013	125.21	94.79	163.07	RW2
DRC	MANIEMA	2014	121.68	85.54	169.82	RW2
DRC	MANIEMA	2015	118.24	71.84	187.32	RW2
DRC	MANIEMA	2016	115.07	60.06	208.76	RW2
DRC	MANIEMA	2017	111.45	48.39	238.56	RW2
DRC	MANIEMA	2018	108.36	38.29	272.25	RW2
DRC	MANIEMA	2019	105.34	29.69	317.68	RW2
DRC	NORD-KIVU	1980	286.79	184.32	416.21	RW2
DRC	NORD-KIVU	1981	276.74	190.46	384.37	RW2
DRC	NORD-KIVU	1982	266.95	189.16	360.61	RW2
DRC	NORD-KIVU	1983	256.99	184.74	344.46	RW2
DRC	NORD-KIVU	1984	247.25	179.46	330.43	RW2
DRC	NORD-KIVU	1985	237.58	174.77	314.27	RW2
DRC	NORD-KIVU	1986	227.53	170.09	298.26	RW2
DRC	NORD-KIVU	1987	217.45	164.59	282.50	RW2
DRC	NORD-KIVU	1988	207.26	157.95	267.36	RW2
DRC	NORD-KIVU	1989	197.37	151.69	254.29	RW2

Continued on next page

Country	Region	Year	Median	Lower	Upper	Method
DRC	NORD-KIVU	1990	187.55	145.29	237.77	RW2
DRC	NORD-KIVU	1991	179.64	141.42	225.29	RW2
DRC	NORD-KIVU	1992	172.52	137.32	213.75	RW2
DRC	NORD-KIVU	1993	166.76	133.05	206.61	RW2
DRC	NORD-KIVU	1994	161.96	128.84	200.70	RW2
DRC	NORD-KIVU	1995	158.05	126.77	195.62	RW2
DRC	NORD-KIVU	1996	153.59	124.76	187.53	RW2
DRC	NORD-KIVU	1997	148.31	121.14	180.33	RW2
DRC	NORD-KIVU	1998	142.38	115.82	173.71	RW2
DRC	NORD-KIVU	1999	135.92	109.61	167.65	RW2
DRC	NORD-KIVU	2000	128.48	103.29	158.49	RW2
DRC	NORD-KIVU	2001	120.72	98.03	148.02	RW2
DRC	NORD-KIVU	2002	112.81	91.46	138.30	RW2
DRC	NORD-KIVU	2003	104.80	84.44	129.86	RW2
DRC	NORD-KIVU	2004	97.07	77.16	121.61	RW2
DRC	NORD-KIVU	2005	89.40	70.67	112.41	RW2
DRC	NORD-KIVU	2006	82.60	65.52	103.74	RW2
DRC	NORD-KIVU	2007	76.20	60.22	95.70	RW2
DRC	NORD-KIVU	2008	70.41	54.72	90.27	RW2
DRC	NORD-KIVU	2009	65.31	49.40	85.79	RW2
DRC	NORD-KIVU	2010	60.71	44.82	81.92	RW2
DRC	NORD-KIVU	2011	56.43	41.23	76.62	RW2
DRC	NORD-KIVU	2012	52.41	37.82	72.07	RW2
DRC	NORD-KIVU	2013	48.74	33.93	69.27	RW2
DRC	NORD-KIVU	2014	45.30	29.06	69.34	RW2
DRC	NORD-KIVU	2015	42.05	23.47	74.01	RW2
DRC	NORD-KIVU	2016	38.97	18.64	80.14	RW2
DRC	NORD-KIVU	2017	36.25	14.24	88.97	RW2
DRC	NORD-KIVU	2018	33.55	10.67	100.08	RW2
DRC	NORD-KIVU	2019	31.08	7.92	115.44	RW2
DRC	ORIENTALE	1980	233.57	146.13	350.85	RW2
DRC	ORIENTALE	1981	230.35	154.12	328.51	RW2
DRC	ORIENTALE	1982	227.23	159.14	314.11	RW2
DRC	ORIENTALE	1983	224.01	159.80	304.03	RW2
DRC	ORIENTALE	1984	220.34	159.16	296.76	RW2
DRC	ORIENTALE	1985	217.16	160.26	287.50	RW2
DRC	ORIENTALE	1986	213.23	160.55	277.11	RW2
DRC	ORIENTALE	1987	208.88	159.44	268.01	RW2
DRC	ORIENTALE	1988	204.15	157.62	260.14	RW2
DRC	ORIENTALE	1989	199.38	155.03	253.60	RW2
DRC	ORIENTALE	1990	194.24	152.63	243.81	RW2
DRC	ORIENTALE	1991	190.70	152.17	236.06	RW2
DRC	ORIENTALE	1992	188.40	151.58	230.88	RW2
DRC	ORIENTALE	1993	186.76	150.65	228.87	RW2
DRC	ORIENTALE	1994	186.26	150.32	227.81	RW2
DRC	ORIENTALE	1995	186.70	152.51	227.61	RW2
DRC	ORIENTALE	1996	186.32	153.94	223.97	RW2
DRC	ORIENTALE	1997	185.17	154.25	220.35	RW2
DRC	ORIENTALE	1998	182.94	151.85	218.62	RW2
DRC	ORIENTALE	1999	179.63	148.31	216.01	RW2
DRC	ORIENTALE	2000	175.12	143.92	210.58	RW2
DRC	ORIENTALE	2001	169.78	141.37	202.65	RW2
DRC	ORIENTALE	2002	163.84	137.02	194.83	RW2
DRC	ORIENTALE	2003	157.28	130.69	188.52	RW2
DRC	ORIENTALE	2004	150.63	123.42	183.20	RW2
DRC	ORIENTALE	2005	143.74	117.04	175.14	RW2
DRC	ORIENTALE	2006	137.38	112.81	166.74	RW2
DRC	ORIENTALE	2007	131.37	107.84	158.94	RW2
DRC	ORIENTALE	2008	125.93	102.10	154.60	RW2
DRC	ORIENTALE	2009	120.94	96.11	151.25	RW2
DRC	ORIENTALE	2010	116.60	90.96	149.12	RW2
DRC	ORIENTALE	2011	112.27	87.63	143.05	RW2
DRC	ORIENTALE	2012	108.30	84.41	137.90	RW2

Continued on next page

Country	Region	Year	Median	Lower	Upper	Method
DRC	ORIENTALE	2013	104.47	79.07	136.69	RW2
DRC	ORIENTALE	2014	100.76	70.60	141.83	RW2
DRC	ORIENTALE	2015	96.92	58.67	155.34	RW2
DRC	ORIENTALE	2016	93.38	48.42	172.91	RW2
DRC	ORIENTALE	2017	90.07	38.91	196.18	RW2
DRC	ORIENTALE	2018	86.79	30.68	226.37	RW2
DRC	ORIENTALE	2019	83.53	22.97	264.12	RW2
DRC	SUD-KIVU	1980	274.56	180.72	393.76	RW2
DRC	SUD-KIVU	1981	271.44	190.94	369.89	RW2
DRC	SUD-KIVU	1982	267.86	195.26	355.80	RW2
DRC	SUD-KIVU	1983	264.32	194.80	346.96	RW2
DRC	SUD-KIVU	1984	260.58	192.88	341.17	RW2
DRC	SUD-KIVU	1985	256.99	193.15	333.00	RW2
DRC	SUD-KIVU	1986	252.39	193.62	323.42	RW2
DRC	SUD-KIVU	1987	247.39	191.69	313.91	RW2
DRC	SUD-KIVU	1988	242.43	188.56	306.39	RW2
DRC	SUD-KIVU	1989	237.41	186.46	298.70	RW2
DRC	SUD-KIVU	1990	231.88	184.09	287.44	RW2
DRC	SUD-KIVU	1991	228.01	183.61	279.00	RW2
DRC	SUD-KIVU	1992	225.25	183.37	273.44	RW2
DRC	SUD-KIVU	1993	223.97	182.36	270.99	RW2
DRC	SUD-KIVU	1994	223.56	181.82	270.94	RW2
DRC	SUD-KIVU	1995	224.07	183.72	270.73	RW2
DRC	SUD-KIVU	1996	223.70	186.15	266.68	RW2
DRC	SUD-KIVU	1997	222.14	185.86	263.84	RW2
DRC	SUD-KIVU	1998	219.68	183.18	261.36	RW2
DRC	SUD-KIVU	1999	215.79	178.54	258.75	RW2
DRC	SUD-KIVU	2000	210.14	173.59	252.00	RW2
DRC	SUD-KIVU	2001	203.83	169.89	242.85	RW2
DRC	SUD-KIVU	2002	196.55	164.17	233.65	RW2
DRC	SUD-KIVU	2003	188.64	156.45	225.83	RW2
DRC	SUD-KIVU	2004	180.64	148.42	218.57	RW2
DRC	SUD-KIVU	2005	172.19	140.06	210.05	RW2
DRC	SUD-KIVU	2006	164.44	134.64	199.26	RW2
DRC	SUD-KIVU	2007	157.09	128.70	190.57	RW2
DRC	SUD-KIVU	2008	150.37	121.29	184.88	RW2
DRC	SUD-KIVU	2009	144.20	114.17	180.64	RW2
DRC	SUD-KIVU	2010	138.65	107.85	177.48	RW2
DRC	SUD-KIVU	2011	133.45	103.47	170.66	RW2
DRC	SUD-KIVU	2012	128.46	99.50	164.15	RW2
DRC	SUD-KIVU	2013	123.61	93.07	161.85	RW2
DRC	SUD-KIVU	2014	118.88	82.91	167.20	RW2
DRC	SUD-KIVU	2015	114.27	69.44	182.76	RW2
DRC	SUD-KIVU	2016	110.13	56.48	201.76	RW2
DRC	SUD-KIVU	2017	105.44	45.43	227.39	RW2
DRC	SUD-KIVU	2018	101.28	35.51	258.09	RW2
DRC	SUD-KIVU	2019	97.68	26.87	296.36	RW2
Egypt	ALL	1980	160.16	152.80	169.05	IHME
Egypt	ALL	1980	171.28	126.90	225.78	RW2
Egypt	ALL	1980	167.60	160.00	175.50	UN
Egypt	ALL	1981	151.88	144.32	159.82	IHME
Egypt	ALL	1981	159.01	128.76	193.51	RW2
Egypt	ALL	1981	158.10	150.90	165.60	UN
Egypt	ALL	1982	142.96	135.26	150.54	IHME
Egypt	ALL	1982	147.46	121.47	177.97	RW2
Egypt	ALL	1982	148.40	141.70	155.30	UN
Egypt	ALL	1983	133.18	126.87	140.14	IHME
Egypt	ALL	1983	136.57	109.54	169.61	RW2
Egypt	ALL	1983	138.50	132.20	144.90	UN
Egypt	ALL	1984	123.58	117.31	129.78	IHME
Egypt	ALL	1984	126.83	99.05	161.62	RW2
Egypt	ALL	1984	128.50	122.80	134.30	UN
Egypt	ALL	1985	113.82	108.22	119.91	IHME

Continued on next page

Country	Region	Year	Median	Lower	Upper	Method
Egypt	ALL	1985	117.43	93.52	146.54	RW2
Egypt	ALL	1985	118.80	113.60	124.20	UN
Egypt	ALL	1986	104.22	98.87	110.25	IHME
Egypt	ALL	1986	109.54	88.90	134.37	RW2
Egypt	ALL	1986	110.10	105.10	115.20	UN
Egypt	ALL	1987	96.12	91.06	101.40	IHME
Egypt	ALL	1987	102.67	84.22	125.18	RW2
Egypt	ALL	1987	102.50	97.70	107.30	UN
Egypt	ALL	1988	89.59	84.80	94.71	IHME
Egypt	ALL	1988	96.63	78.05	118.86	RW2
Egypt	ALL	1988	96.00	91.50	100.60	UN
Egypt	ALL	1989	85.69	80.95	90.53	IHME
Egypt	ALL	1989	91.31	72.50	113.95	RW2
Egypt	ALL	1989	90.60	86.50	94.90	UN
Egypt	ALL	1990	82.12	77.46	86.68	IHME
Egypt	ALL	1990	86.80	69.30	109.01	RW2
Egypt	ALL	1990	85.90	82.10	90.00	UN
Egypt	ALL	1991	79.13	74.55	83.71	IHME
Egypt	ALL	1991	82.26	66.49	101.31	RW2
Egypt	ALL	1991	81.70	78.00	85.60	UN
Egypt	ALL	1992	75.30	70.68	79.69	IHME
Egypt	ALL	1992	77.84	63.16	95.25	RW2
Egypt	ALL	1992	77.50	73.90	81.40	UN
Egypt	ALL	1993	70.35	66.19	74.81	IHME
Egypt	ALL	1993	73.49	59.17	90.70	RW2
Egypt	ALL	1993	73.30	69.70	77.10	UN
Egypt	ALL	1994	65.89	61.57	70.40	IHME
Egypt	ALL	1994	69.12	54.75	87.11	RW2
Egypt	ALL	1994	68.90	65.50	72.70	UN
Egypt	ALL	1995	61.06	57.05	65.29	IHME
Egypt	ALL	1995	64.81	51.22	81.33	RW2
Egypt	ALL	1995	64.60	61.40	68.20	UN
Egypt	ALL	1996	55.24	51.48	59.02	IHME
Egypt	ALL	1996	60.67	48.62	75.53	RW2
Egypt	ALL	1996	60.40	57.40	63.80	UN
Egypt	ALL	1997	50.84	47.39	54.82	IHME
Egypt	ALL	1997	56.74	45.88	70.02	RW2
Egypt	ALL	1997	56.50	53.50	59.70	UN
Egypt	ALL	1998	48.54	45.14	52.24	IHME
Egypt	ALL	1998	53.08	42.61	66.49	RW2
Egypt	ALL	1998	52.80	49.90	56.00	UN
Egypt	ALL	1999	46.51	43.20	50.05	IHME
Egypt	ALL	1999	49.69	39.21	62.88	RW2
Egypt	ALL	1999	49.50	46.60	52.60	UN
Egypt	ALL	2000	43.25	39.93	46.83	IHME
Egypt	ALL	2000	46.53	36.52	58.93	RW2
Egypt	ALL	2000	46.50	43.60	49.50	UN
Egypt	ALL	2001	39.57	36.34	42.55	IHME
Egypt	ALL	2001	43.78	34.77	54.90	RW2
Egypt	ALL	2001	43.80	41.00	46.80	UN
Egypt	ALL	2002	37.03	34.04	40.15	IHME
Egypt	ALL	2002	41.34	33.18	51.44	RW2
Egypt	ALL	2002	41.40	38.60	44.30	UN
Egypt	ALL	2003	35.94	32.92	39.18	IHME
Egypt	ALL	2003	39.23	31.26	49.13	RW2
Egypt	ALL	2003	39.20	36.50	42.20	UN
Egypt	ALL	2004	35.26	32.09	38.43	IHME
Egypt	ALL	2004	37.31	28.93	47.79	RW2
Egypt	ALL	2004	37.30	34.60	40.30	UN
Egypt	ALL	2005	34.06	30.66	37.25	IHME
Egypt	ALL	2005	35.73	27.21	47.04	RW2
Egypt	ALL	2005	35.60	32.80	38.60	UN
Egypt	ALL	2006	32.60	29.41	36.22	IHME

Continued on next page

Country	Region	Year	Median	Lower	Upper	Method
Egypt	ALL	2006	34.18	26.47	44.14	RW2
Egypt	ALL	2006	34.10	31.20	37.10	UN
Egypt	ALL	2007	31.19	27.87	35.06	IHME
Egypt	ALL	2007	32.77	25.99	41.26	RW2
Egypt	ALL	2007	32.70	29.80	35.90	UN
Egypt	ALL	2008	30.04	26.49	33.66	IHME
Egypt	ALL	2008	31.47	24.40	40.47	RW2
Egypt	ALL	2008	31.40	28.40	34.80	UN
Egypt	ALL	2009	29.20	25.84	32.95	IHME
Egypt	ALL	2009	30.13	21.03	43.19	RW2
Egypt	ALL	2009	30.20	27.00	33.70	UN
Egypt	ALL	2010	28.46	25.21	32.15	IHME
Egypt	ALL	2010	28.83	16.52	50.34	RW2
Egypt	ALL	2010	29.00	25.60	32.80	UN
Egypt	ALL	2011	27.45	24.21	30.95	IHME
Egypt	ALL	2011	27.65	12.77	58.94	RW2
Egypt	ALL	2011	27.90	24.20	32.00	UN
Egypt	ALL	2012	26.07	22.94	29.63	IHME
Egypt	ALL	2012	26.46	9.55	71.22	RW2
Egypt	ALL	2012	26.80	22.90	31.40	UN
Egypt	ALL	2013	24.45	21.13	28.41	IHME
Egypt	ALL	2013	25.43	7.07	87.08	RW2
Egypt	ALL	2013	25.80	21.60	30.90	UN
Egypt	ALL	2014	22.82	18.44	28.28	IHME
Egypt	ALL	2014	24.33	5.10	108.54	RW2
Egypt	ALL	2014	24.80	20.30	30.50	UN
Egypt	ALL	2015	21.54	16.27	28.31	IHME
Egypt	ALL	2015	23.22	3.66	134.77	RW2
Egypt	ALL	2015	24.00	19.10	30.30	UN
Egypt	ALL	2016	22.41	2.60	173.27	RW2
Egypt	ALL	2017	21.34	1.76	220.17	RW2
Egypt	ALL	2018	20.43	1.21	282.60	RW2
Egypt	ALL	2019	19.51	0.75	344.02	RW2
Egypt	ALL	15-19	21.33	1.77	216.74	RW2
Egypt	FRONTIER GOVERNORATES	1980	113.53	82.70	154.40	RW2
Egypt	FRONTIER GOVERNORATES	1981	106.16	83.86	133.59	RW2
Egypt	FRONTIER GOVERNORATES	1982	99.26	80.08	122.13	RW2
Egypt	FRONTIER GOVERNORATES	1983	92.68	74.01	115.93	RW2
Egypt	FRONTIER GOVERNORATES	1984	86.75	68.64	109.33	RW2
Egypt	FRONTIER GOVERNORATES	1985	81.17	65.11	100.23	RW2
Egypt	FRONTIER GOVERNORATES	1986	76.26	62.73	92.64	RW2
Egypt	FRONTIER GOVERNORATES	1987	72.03	59.92	86.41	RW2
Egypt	FRONTIER GOVERNORATES	1988	68.29	56.29	82.59	RW2
Egypt	FRONTIER GOVERNORATES	1989	64.97	52.74	79.80	RW2
Egypt	FRONTIER GOVERNORATES	1990	62.14	50.52	76.45	RW2
Egypt	FRONTIER GOVERNORATES	1991	59.51	48.94	71.91	RW2
Egypt	FRONTIER GOVERNORATES	1992	56.94	47.11	68.43	RW2
Egypt	FRONTIER GOVERNORATES	1993	54.52	44.81	66.18	RW2
Egypt	FRONTIER GOVERNORATES	1994	52.20	42.10	64.41	RW2
Egypt	FRONTIER GOVERNORATES	1995	49.85	40.05	61.81	RW2
Egypt	FRONTIER GOVERNORATES	1996	47.57	38.67	58.13	RW2
Egypt	FRONTIER GOVERNORATES	1997	45.35	37.06	55.47	RW2
Egypt	FRONTIER GOVERNORATES	1998	43.26	35.02	53.43	RW2
Egypt	FRONTIER GOVERNORATES	1999	41.24	32.81	51.77	RW2
Egypt	FRONTIER GOVERNORATES	2000	39.34	31.04	49.75	RW2
Egypt	FRONTIER GOVERNORATES	2001	37.81	29.96	47.59	RW2
Egypt	FRONTIER GOVERNORATES	2002	36.52	28.97	46.04	RW2
Egypt	FRONTIER GOVERNORATES	2003	35.50	27.68	45.45	RW2
Egypt	FRONTIER GOVERNORATES	2004	34.74	26.34	45.48	RW2
Egypt	FRONTIER GOVERNORATES	2005	34.24	25.42	46.13	RW2
Egypt	FRONTIER GOVERNORATES	2006	33.74	25.11	45.63	RW2
Egypt	FRONTIER GOVERNORATES	2007	33.39	24.76	45.33	RW2
Egypt	FRONTIER GOVERNORATES	2008	33.06	23.67	46.39	RW2

Continued on next page

Country	Region	Year	Median	Lower	Upper	Method
Egypt	FRONTIER GOVERNORATES	2009	32.67	21.52	49.72	RW2
Egypt	FRONTIER GOVERNORATES	2010	32.33	18.23	57.15	RW2
Egypt	FRONTIER GOVERNORATES	2011	31.98	15.24	66.86	RW2
Egypt	FRONTIER GOVERNORATES	2012	31.63	12.30	80.08	RW2
Egypt	FRONTIER GOVERNORATES	2013	31.29	9.74	97.55	RW2
Egypt	FRONTIER GOVERNORATES	2014	30.84	7.39	119.90	RW2
Egypt	FRONTIER GOVERNORATES	2015	30.64	5.68	151.09	RW2
Egypt	FRONTIER GOVERNORATES	2016	30.22	4.19	188.86	RW2
Egypt	FRONTIER GOVERNORATES	2017	30.04	3.11	238.81	RW2
Egypt	FRONTIER GOVERNORATES	2018	29.52	2.27	296.03	RW2
Egypt	FRONTIER GOVERNORATES	2019	29.40	1.65	366.90	RW2
Egypt	LOWER EGYPT	1980	146.55	112.97	186.97	RW2
Egypt	LOWER EGYPT	1981	135.86	114.36	159.94	RW2
Egypt	LOWER EGYPT	1982	125.76	107.22	146.99	RW2
Egypt	LOWER EGYPT	1983	116.23	96.79	139.54	RW2
Egypt	LOWER EGYPT	1984	107.65	88.07	131.33	RW2
Egypt	LOWER EGYPT	1985	99.60	82.63	119.69	RW2
Egypt	LOWER EGYPT	1986	92.53	78.31	109.12	RW2
Egypt	LOWER EGYPT	1987	86.27	73.49	101.12	RW2
Egypt	LOWER EGYPT	1988	80.66	67.99	95.18	RW2
Egypt	LOWER EGYPT	1989	75.69	62.84	90.74	RW2
Egypt	LOWER EGYPT	1990	71.25	59.24	85.52	RW2
Egypt	LOWER EGYPT	1991	67.06	56.70	79.31	RW2
Egypt	LOWER EGYPT	1992	63.15	53.69	74.08	RW2
Egypt	LOWER EGYPT	1993	59.38	49.85	70.46	RW2
Egypt	LOWER EGYPT	1994	55.79	46.08	67.29	RW2
Egypt	LOWER EGYPT	1995	52.30	43.22	63.12	RW2
Egypt	LOWER EGYPT	1996	49.01	40.95	58.34	RW2
Egypt	LOWER EGYPT	1997	45.85	38.64	54.33	RW2
Egypt	LOWER EGYPT	1998	42.90	35.84	51.35	RW2
Egypt	LOWER EGYPT	1999	40.11	32.86	48.94	RW2
Egypt	LOWER EGYPT	2000	37.58	30.61	45.73	RW2
Egypt	LOWER EGYPT	2001	35.41	29.26	42.65	RW2
Egypt	LOWER EGYPT	2002	33.57	27.99	40.25	RW2
Egypt	LOWER EGYPT	2003	31.99	26.28	38.75	RW2
Egypt	LOWER EGYPT	2004	30.69	24.61	38.15	RW2
Egypt	LOWER EGYPT	2005	29.61	23.39	37.63	RW2
Egypt	LOWER EGYPT	2006	28.62	22.79	35.95	RW2
Egypt	LOWER EGYPT	2007	27.72	22.38	34.31	RW2
Egypt	LOWER EGYPT	2008	26.85	21.09	34.24	RW2
Egypt	LOWER EGYPT	2009	26.03	18.54	36.43	RW2
Egypt	LOWER EGYPT	2010	25.18	15.02	41.82	RW2
Egypt	LOWER EGYPT	2011	24.40	12.23	48.58	RW2
Egypt	LOWER EGYPT	2012	23.64	9.62	57.10	RW2
Egypt	LOWER EGYPT	2013	22.88	7.39	69.13	RW2
Egypt	LOWER EGYPT	2014	22.14	5.56	84.83	RW2
Egypt	LOWER EGYPT	2015	21.51	4.09	105.74	RW2
Egypt	LOWER EGYPT	2016	20.80	2.99	132.05	RW2
Egypt	LOWER EGYPT	2017	20.09	2.18	164.11	RW2
Egypt	LOWER EGYPT	2018	19.48	1.57	207.94	RW2
Egypt	LOWER EGYPT	2019	18.89	1.07	261.09	RW2
Egypt	UPPER EGYPT	1980	230.29	182.73	285.50	RW2
Egypt	UPPER EGYPT	1981	213.76	183.18	247.75	RW2
Egypt	UPPER EGYPT	1982	197.89	171.45	227.21	RW2
Egypt	UPPER EGYPT	1983	183.12	154.64	215.91	RW2
Egypt	UPPER EGYPT	1984	169.46	140.01	203.87	RW2
Egypt	UPPER EGYPT	1985	156.91	131.68	185.88	RW2
Egypt	UPPER EGYPT	1986	145.95	124.73	169.71	RW2
Egypt	UPPER EGYPT	1987	136.31	117.43	157.71	RW2
Egypt	UPPER EGYPT	1988	127.89	108.85	149.60	RW2
Egypt	UPPER EGYPT	1989	120.48	100.97	142.88	RW2
Egypt	UPPER EGYPT	1990	114.00	95.76	135.59	RW2
Egypt	UPPER EGYPT	1991	107.89	91.97	126.47	RW2

Continued on next page

Country	Region	Year	Median	Lower	Upper	Method
Egypt	UPPER EGYPT	1992	102.00	87.26	118.76	RW2
Egypt	UPPER EGYPT	1993	96.22	81.63	112.88	RW2
Egypt	UPPER EGYPT	1994	90.64	75.51	107.96	RW2
Egypt	UPPER EGYPT	1995	85.06	70.77	101.70	RW2
Egypt	UPPER EGYPT	1996	79.62	67.12	93.98	RW2
Egypt	UPPER EGYPT	1997	74.28	63.18	87.32	RW2
Egypt	UPPER EGYPT	1998	69.16	58.57	81.88	RW2
Egypt	UPPER EGYPT	1999	64.36	53.28	77.44	RW2
Egypt	UPPER EGYPT	2000	59.78	49.12	72.13	RW2
Egypt	UPPER EGYPT	2001	55.87	46.54	66.65	RW2
Egypt	UPPER EGYPT	2002	52.44	44.11	62.19	RW2
Egypt	UPPER EGYPT	2003	49.47	41.22	59.35	RW2
Egypt	UPPER EGYPT	2004	46.95	38.15	57.49	RW2
Egypt	UPPER EGYPT	2005	44.85	35.93	56.15	RW2
Egypt	UPPER EGYPT	2006	42.90	34.89	52.88	RW2
Egypt	UPPER EGYPT	2007	41.08	33.81	49.71	RW2
Egypt	UPPER EGYPT	2008	39.40	31.54	48.95	RW2
Egypt	UPPER EGYPT	2009	37.71	27.37	51.67	RW2
Egypt	UPPER EGYPT	2010	36.11	22.13	58.62	RW2
Egypt	UPPER EGYPT	2011	34.53	17.60	67.18	RW2
Egypt	UPPER EGYPT	2012	33.06	13.70	78.39	RW2
Egypt	UPPER EGYPT	2013	31.71	10.45	94.63	RW2
Egypt	UPPER EGYPT	2014	30.41	7.76	112.76	RW2
Egypt	UPPER EGYPT	2015	29.12	5.70	137.01	RW2
Egypt	UPPER EGYPT	2016	27.78	4.10	167.00	RW2
Egypt	UPPER EGYPT	2017	26.70	2.94	207.00	RW2
Egypt	UPPER EGYPT	2018	25.41	2.03	256.81	RW2
Egypt	UPPER EGYPT	2019	24.37	1.40	320.23	RW2
Egypt	URBAN GOVERNORATES	1980	103.01	78.04	135.03	RW2
Egypt	URBAN GOVERNORATES	1981	96.29	79.55	115.92	RW2
Egypt	URBAN GOVERNORATES	1982	89.96	75.64	106.85	RW2
Egypt	URBAN GOVERNORATES	1983	84.04	69.04	102.31	RW2
Egypt	URBAN GOVERNORATES	1984	78.63	63.46	97.47	RW2
Egypt	URBAN GOVERNORATES	1985	73.44	60.19	89.29	RW2
Egypt	URBAN GOVERNORATES	1986	68.99	57.83	82.07	RW2
Egypt	URBAN GOVERNORATES	1987	64.93	54.87	76.94	RW2
Egypt	URBAN GOVERNORATES	1988	61.40	51.19	73.30	RW2
Egypt	URBAN GOVERNORATES	1989	58.23	47.86	70.63	RW2
Egypt	URBAN GOVERNORATES	1990	55.49	45.73	67.42	RW2
Egypt	URBAN GOVERNORATES	1991	52.86	44.12	63.43	RW2
Egypt	URBAN GOVERNORATES	1992	50.40	42.23	59.93	RW2
Egypt	URBAN GOVERNORATES	1993	48.07	39.95	57.62	RW2
Egypt	URBAN GOVERNORATES	1994	45.84	37.37	56.02	RW2
Egypt	URBAN GOVERNORATES	1995	43.64	35.49	53.34	RW2
Egypt	URBAN GOVERNORATES	1996	41.56	34.20	50.28	RW2
Egypt	URBAN GOVERNORATES	1997	39.52	32.75	47.52	RW2
Egypt	URBAN GOVERNORATES	1998	37.60	30.87	45.69	RW2
Egypt	URBAN GOVERNORATES	1999	35.82	28.95	44.43	RW2
Egypt	URBAN GOVERNORATES	2000	34.10	27.28	42.37	RW2
Egypt	URBAN GOVERNORATES	2001	32.75	26.59	40.36	RW2
Egypt	URBAN GOVERNORATES	2002	31.65	25.70	38.89	RW2
Egypt	URBAN GOVERNORATES	2003	30.76	24.59	38.28	RW2
Egypt	URBAN GOVERNORATES	2004	30.10	23.48	38.41	RW2
Egypt	URBAN GOVERNORATES	2005	29.67	22.70	38.80	RW2
Egypt	URBAN GOVERNORATES	2006	29.28	22.45	38.22	RW2
Egypt	URBAN GOVERNORATES	2007	28.94	22.30	38.00	RW2
Egypt	URBAN GOVERNORATES	2008	28.66	21.33	38.70	RW2
Egypt	URBAN GOVERNORATES	2009	28.35	19.36	41.88	RW2
Egypt	URBAN GOVERNORATES	2010	28.09	16.28	48.36	RW2
Egypt	URBAN GOVERNORATES	2011	27.78	13.50	57.00	RW2
Egypt	URBAN GOVERNORATES	2012	27.54	11.04	68.57	RW2
Egypt	URBAN GOVERNORATES	2013	27.27	8.56	83.15	RW2
Egypt	URBAN GOVERNORATES	2014	26.94	6.56	105.15	RW2

Continued on next page

Country	Region	Year	Median	Lower	Upper	Method
Egypt	URBAN GOVERNORATES	2015	26.74	5.02	132.63	RW2
Egypt	URBAN GOVERNORATES	2016	26.44	3.77	165.29	RW2
Egypt	URBAN GOVERNORATES	2017	26.11	2.77	209.25	RW2
Egypt	URBAN GOVERNORATES	2018	25.88	2.00	266.60	RW2
Egypt	URBAN GOVERNORATES	2019	25.52	1.44	335.29	RW2
Ethiopia	ADDIS ABABA	1980	90.78	63.18	127.92	RW2
Ethiopia	ADDIS ABABA	1981	90.31	67.40	119.03	RW2
Ethiopia	ADDIS ABABA	1982	89.83	69.36	115.43	RW2
Ethiopia	ADDIS ABABA	1983	89.38	69.23	114.45	RW2
Ethiopia	ADDIS ABABA	1984	88.96	68.95	114.20	RW2
Ethiopia	ADDIS ABABA	1985	88.67	70.07	111.49	RW2
Ethiopia	ADDIS ABABA	1986	88.78	71.67	109.22	RW2
Ethiopia	ADDIS ABABA	1987	89.16	72.68	108.38	RW2
Ethiopia	ADDIS ABABA	1988	89.70	73.07	109.11	RW2
Ethiopia	ADDIS ABABA	1989	90.29	73.04	110.74	RW2
Ethiopia	ADDIS ABABA	1990	91.11	74.29	111.52	RW2
Ethiopia	ADDIS ABABA	1991	91.18	75.24	110.18	RW2
Ethiopia	ADDIS ABABA	1992	90.75	75.22	108.95	RW2
Ethiopia	ADDIS ABABA	1993	89.47	73.86	108.42	RW2
Ethiopia	ADDIS ABABA	1994	87.70	71.82	107.34	RW2
Ethiopia	ADDIS ABABA	1995	84.98	69.35	104.09	RW2
Ethiopia	ADDIS ABABA	1996	82.38	67.74	100.18	RW2
Ethiopia	ADDIS ABABA	1997	79.60	65.74	96.42	RW2
Ethiopia	ADDIS ABABA	1998	76.72	62.78	93.85	RW2
Ethiopia	ADDIS ABABA	1999	73.81	59.71	91.21	RW2
Ethiopia	ADDIS ABABA	2000	71.06	57.08	88.33	RW2
Ethiopia	ADDIS ABABA	2001	67.85	54.94	83.90	RW2
Ethiopia	ADDIS ABABA	2002	64.50	52.13	79.64	RW2
Ethiopia	ADDIS ABABA	2003	60.92	48.65	76.08	RW2
Ethiopia	ADDIS ABABA	2004	57.32	44.90	72.94	RW2
Ethiopia	ADDIS ABABA	2005	53.61	41.52	68.77	RW2
Ethiopia	ADDIS ABABA	2006	50.21	38.89	64.60	RW2
Ethiopia	ADDIS ABABA	2007	47.00	36.06	60.73	RW2
Ethiopia	ADDIS ABABA	2008	44.09	33.14	58.30	RW2
Ethiopia	ADDIS ABABA	2009	41.43	30.25	56.26	RW2
Ethiopia	ADDIS ABABA	2010	39.08	27.62	54.83	RW2
Ethiopia	ADDIS ABABA	2011	36.81	25.50	52.29	RW2
Ethiopia	ADDIS ABABA	2012	34.77	23.48	50.23	RW2
Ethiopia	ADDIS ABABA	2013	32.83	21.19	49.35	RW2
Ethiopia	ADDIS ABABA	2014	31.00	18.52	50.19	RW2
Ethiopia	ADDIS ABABA	2015	29.16	15.24	53.50	RW2
Ethiopia	ADDIS ABABA	2016	27.49	12.45	58.43	RW2
Ethiopia	ADDIS ABABA	2017	25.96	9.89	65.43	RW2
Ethiopia	ADDIS ABABA	2018	24.48	7.71	75.09	RW2
Ethiopia	ADDIS ABABA	2019	23.06	5.71	87.92	RW2
Ethiopia	AFFAR	1980	338.93	268.27	418.62	RW2
Ethiopia	AFFAR	1981	328.63	275.04	388.49	RW2
Ethiopia	AFFAR	1982	318.60	271.43	372.14	RW2
Ethiopia	AFFAR	1983	309.04	261.49	362.84	RW2
Ethiopia	AFFAR	1984	299.86	251.34	353.45	RW2
Ethiopia	AFFAR	1985	290.59	247.35	338.81	RW2
Ethiopia	AFFAR	1986	282.55	244.04	324.92	RW2
Ethiopia	AFFAR	1987	275.26	239.75	314.22	RW2
Ethiopia	AFFAR	1988	268.56	232.17	307.61	RW2
Ethiopia	AFFAR	1989	261.92	224.63	302.91	RW2
Ethiopia	AFFAR	1990	256.29	220.46	295.79	RW2
Ethiopia	AFFAR	1991	249.12	215.79	285.52	RW2
Ethiopia	AFFAR	1992	241.15	209.88	274.79	RW2
Ethiopia	AFFAR	1993	232.08	200.32	267.04	RW2
Ethiopia	AFFAR	1994	222.35	189.77	258.87	RW2
Ethiopia	AFFAR	1995	211.67	179.56	246.39	RW2
Ethiopia	AFFAR	1996	202.11	173.57	234.02	RW2
Ethiopia	AFFAR	1997	193.16	166.05	222.63	RW2

Continued on next page

Country	Region	Year	Median	Lower	Upper	Method
Ethiopia	AFFAR	1998	184.68	157.91	214.50	RW2
Ethiopia	AFFAR	1999	177.00	149.19	207.42	RW2
Ethiopia	AFFAR	2000	170.15	143.48	200.68	RW2
Ethiopia	AFFAR	2001	162.81	138.63	190.34	RW2
Ethiopia	AFFAR	2002	155.44	132.96	181.01	RW2
Ethiopia	AFFAR	2003	147.83	125.50	172.82	RW2
Ethiopia	AFFAR	2004	140.00	117.24	166.18	RW2
Ethiopia	AFFAR	2005	132.08	110.15	157.43	RW2
Ethiopia	AFFAR	2006	124.62	104.70	147.43	RW2
Ethiopia	AFFAR	2007	117.67	99.30	139.27	RW2
Ethiopia	AFFAR	2008	111.14	92.72	133.23	RW2
Ethiopia	AFFAR	2009	105.25	85.59	128.84	RW2
Ethiopia	AFFAR	2010	99.85	79.88	124.73	RW2
Ethiopia	AFFAR	2011	94.75	76.00	117.85	RW2
Ethiopia	AFFAR	2012	89.89	72.59	112.00	RW2
Ethiopia	AFFAR	2013	85.27	66.97	108.69	RW2
Ethiopia	AFFAR	2014	80.88	58.95	111.24	RW2
Ethiopia	AFFAR	2015	76.79	48.15	121.15	RW2
Ethiopia	AFFAR	2016	72.67	39.33	132.15	RW2
Ethiopia	AFFAR	2017	68.83	30.69	148.05	RW2
Ethiopia	AFFAR	2018	65.30	23.78	167.12	RW2
Ethiopia	AFFAR	2019	61.89	17.91	190.41	RW2
Ethiopia	ALL	1980	234.70	226.49	242.99	IHME
Ethiopia	ALL	1980	244.89	189.68	309.51	RW2
Ethiopia	ALL	1980	241.60	222.40	263.00	UN
Ethiopia	ALL	1981	231.96	224.28	239.72	IHME
Ethiopia	ALL	1981	240.35	200.48	284.57	RW2
Ethiopia	ALL	1981	239.30	220.80	260.10	UN
Ethiopia	ALL	1982	229.35	221.93	237.11	IHME
Ethiopia	ALL	1982	235.88	200.23	275.81	RW2
Ethiopia	ALL	1982	236.00	218.10	255.90	UN
Ethiopia	ALL	1983	282.13	221.71	366.04	IHME
Ethiopia	ALL	1983	231.26	192.90	275.00	RW2
Ethiopia	ALL	1983	232.00	214.70	251.00	UN
Ethiopia	ALL	1984	276.95	218.69	356.99	IHME
Ethiopia	ALL	1984	227.19	185.77	274.62	RW2
Ethiopia	ALL	1984	227.60	210.80	245.80	UN
Ethiopia	ALL	1985	219.13	212.03	225.49	IHME
Ethiopia	ALL	1985	222.63	185.29	265.39	RW2
Ethiopia	ALL	1985	223.40	206.90	241.00	UN
Ethiopia	ALL	1986	215.46	208.72	221.66	IHME
Ethiopia	ALL	1986	218.82	184.66	257.50	RW2
Ethiopia	ALL	1986	219.30	203.20	236.50	UN
Ethiopia	ALL	1987	212.19	205.78	218.43	IHME
Ethiopia	ALL	1987	215.35	183.15	252.00	RW2
Ethiopia	ALL	1987	215.50	200.00	232.60	UN
Ethiopia	ALL	1988	208.26	201.59	214.06	IHME
Ethiopia	ALL	1988	211.91	178.28	249.25	RW2
Ethiopia	ALL	1988	212.00	196.90	228.50	UN
Ethiopia	ALL	1989	205.09	198.72	211.53	IHME
Ethiopia	ALL	1989	208.43	173.28	247.53	RW2
Ethiopia	ALL	1989	208.40	193.90	224.70	UN
Ethiopia	ALL	1990	202.24	195.85	208.88	IHME
Ethiopia	ALL	1990	205.23	171.29	244.40	RW2
Ethiopia	ALL	1990	204.60	190.20	220.60	UN
Ethiopia	ALL	1991	196.20	190.36	202.28	IHME
Ethiopia	ALL	1991	200.75	169.11	235.98	RW2
Ethiopia	ALL	1991	200.20	186.10	215.90	UN
Ethiopia	ALL	1992	191.69	186.07	197.46	IHME
Ethiopia	ALL	1992	195.40	165.23	229.05	RW2
Ethiopia	ALL	1992	195.00	181.10	210.40	UN
Ethiopia	ALL	1993	186.88	181.35	192.60	IHME
Ethiopia	ALL	1993	189.24	159.38	223.69	RW2

Continued on next page

Country	Region	Year	Median	Lower	Upper	Method
Ethiopia	ALL	1993	188.90	175.40	203.60	UN
Ethiopia	ALL	1994	181.58	176.20	187.06	IHME
Ethiopia	ALL	1994	182.40	151.90	219.25	RW2
Ethiopia	ALL	1994	182.00	169.20	196.10	UN
Ethiopia	ALL	1995	175.72	170.40	181.23	IHME
Ethiopia	ALL	1995	174.69	144.78	208.63	RW2
Ethiopia	ALL	1995	175.00	162.50	188.40	UN
Ethiopia	ALL	1996	169.09	163.83	174.58	IHME
Ethiopia	ALL	1996	167.84	140.63	199.59	RW2
Ethiopia	ALL	1996	168.30	156.10	181.00	UN
Ethiopia	ALL	1997	162.37	157.30	167.72	IHME
Ethiopia	ALL	1997	161.49	136.05	190.88	RW2
Ethiopia	ALL	1997	162.00	150.20	174.20	UN
Ethiopia	ALL	1998	156.20	151.15	161.29	IHME
Ethiopia	ALL	1998	155.71	130.07	185.72	RW2
Ethiopia	ALL	1998	156.20	144.90	168.10	UN
Ethiopia	ALL	1999	151.30	146.11	156.53	IHME
Ethiopia	ALL	1999	150.17	123.50	180.09	RW2
Ethiopia	ALL	1999	150.80	139.60	162.70	UN
Ethiopia	ALL	2000	143.12	138.13	147.83	IHME
Ethiopia	ALL	2000	145.37	120.23	175.71	RW2
Ethiopia	ALL	2000	145.10	134.10	157.20	UN
Ethiopia	ALL	2001	136.23	131.23	140.75	IHME
Ethiopia	ALL	2001	139.35	115.89	167.06	RW2
Ethiopia	ALL	2001	139.00	128.10	151.20	UN
Ethiopia	ALL	2002	129.44	124.59	134.04	IHME
Ethiopia	ALL	2002	132.64	110.79	158.17	RW2
Ethiopia	ALL	2002	132.20	121.70	144.20	UN
Ethiopia	ALL	2003	123.07	118.11	127.65	IHME
Ethiopia	ALL	2003	125.32	104.19	150.23	RW2
Ethiopia	ALL	2003	124.80	114.70	136.30	UN
Ethiopia	ALL	2004	116.70	111.86	121.51	IHME
Ethiopia	ALL	2004	117.35	96.03	142.89	RW2
Ethiopia	ALL	2004	117.00	107.20	128.10	UN
Ethiopia	ALL	2005	110.17	104.90	115.19	IHME
Ethiopia	ALL	2005	108.92	88.16	132.79	RW2
Ethiopia	ALL	2005	109.10	99.60	119.90	UN
Ethiopia	ALL	2006	103.72	98.39	108.99	IHME
Ethiopia	ALL	2006	101.21	82.80	122.41	RW2
Ethiopia	ALL	2006	101.30	91.30	112.30	UN
Ethiopia	ALL	2007	97.51	91.90	103.18	IHME
Ethiopia	ALL	2007	94.07	77.60	113.39	RW2
Ethiopia	ALL	2007	93.80	83.10	105.60	UN
Ethiopia	ALL	2008	91.82	86.06	97.92	IHME
Ethiopia	ALL	2008	87.67	71.76	106.81	RW2
Ethiopia	ALL	2008	86.90	75.00	99.80	UN
Ethiopia	ALL	2009	86.48	80.60	92.94	IHME
Ethiopia	ALL	2009	81.76	65.70	101.58	RW2
Ethiopia	ALL	2009	80.80	67.80	94.90	UN
Ethiopia	ALL	2010	81.63	75.47	88.41	IHME
Ethiopia	ALL	2010	76.66	60.74	97.19	RW2
Ethiopia	ALL	2010	75.70	61.50	90.80	UN
Ethiopia	ALL	2011	76.99	70.63	84.07	IHME
Ethiopia	ALL	2011	71.86	57.39	89.95	RW2
Ethiopia	ALL	2011	71.30	56.10	87.90	UN
Ethiopia	ALL	2012	72.56	66.10	80.04	IHME
Ethiopia	ALL	2012	67.38	54.34	83.35	RW2
Ethiopia	ALL	2012	67.70	51.40	85.90	UN
Ethiopia	ALL	2013	68.29	61.63	76.01	IHME
Ethiopia	ALL	2013	63.24	49.66	80.07	RW2
Ethiopia	ALL	2013	64.60	47.20	84.50	UN
Ethiopia	ALL	2014	64.20	57.48	72.16	IHME
Ethiopia	ALL	2014	59.27	42.36	82.38	RW2

Continued on next page

Country	Region	Year	Median	Lower	Upper	Method
Ethiopia	ALL	2014	61.80	43.80	83.30	UN
Ethiopia	ALL	2015	60.27	53.72	68.47	IHME
Ethiopia	ALL	2015	55.44	33.42	91.02	RW2
Ethiopia	ALL	2015	59.20	40.60	83.00	UN
Ethiopia	ALL	2016	52.05	26.34	101.62	RW2
Ethiopia	ALL	2017	48.62	20.02	115.60	RW2
Ethiopia	ALL	2018	45.50	15.06	134.58	RW2
Ethiopia	ALL	2019	42.51	10.69	155.58	RW2
Ethiopia	ALL	15-19	48.61	20.34	113.73	RW2
Ethiopia	AMHARA	1980	244.29	193.42	303.66	RW2
Ethiopia	AMHARA	1981	239.11	202.97	279.57	RW2
Ethiopia	AMHARA	1982	233.89	201.05	269.76	RW2
Ethiopia	AMHARA	1983	228.97	194.65	267.70	RW2
Ethiopia	AMHARA	1984	224.26	188.41	265.28	RW2
Ethiopia	AMHARA	1985	219.64	186.97	256.33	RW2
Ethiopia	AMHARA	1986	215.81	186.50	247.90	RW2
Ethiopia	AMHARA	1987	212.51	185.11	242.78	RW2
Ethiopia	AMHARA	1988	209.62	181.42	240.31	RW2
Ethiopia	AMHARA	1989	206.93	176.82	239.37	RW2
Ethiopia	AMHARA	1990	204.77	175.88	237.35	RW2
Ethiopia	AMHARA	1991	201.37	174.86	231.40	RW2
Ethiopia	AMHARA	1992	197.04	171.85	224.33	RW2
Ethiopia	AMHARA	1993	191.74	166.31	219.86	RW2
Ethiopia	AMHARA	1994	185.70	159.08	216.04	RW2
Ethiopia	AMHARA	1995	178.71	152.61	207.41	RW2
Ethiopia	AMHARA	1996	172.26	148.69	198.15	RW2
Ethiopia	AMHARA	1997	166.15	144.37	190.25	RW2
Ethiopia	AMHARA	1998	160.24	138.31	184.91	RW2
Ethiopia	AMHARA	1999	154.66	131.22	181.47	RW2
Ethiopia	AMHARA	2000	149.48	126.76	175.90	RW2
Ethiopia	AMHARA	2001	143.54	122.82	167.10	RW2
Ethiopia	AMHARA	2002	137.17	117.72	159.41	RW2
Ethiopia	AMHARA	2003	130.23	110.98	152.36	RW2
Ethiopia	AMHARA	2004	123.04	103.31	146.10	RW2
Ethiopia	AMHARA	2005	115.32	95.96	137.42	RW2
Ethiopia	AMHARA	2006	108.08	90.72	128.10	RW2
Ethiopia	AMHARA	2007	100.98	84.85	119.75	RW2
Ethiopia	AMHARA	2008	94.36	78.39	113.13	RW2
Ethiopia	AMHARA	2009	88.36	71.72	108.30	RW2
Ethiopia	AMHARA	2010	82.74	65.57	103.39	RW2
Ethiopia	AMHARA	2011	77.45	61.58	96.68	RW2
Ethiopia	AMHARA	2012	72.53	57.32	90.47	RW2
Ethiopia	AMHARA	2013	67.92	51.88	86.68	RW2
Ethiopia	AMHARA	2014	63.58	44.80	87.56	RW2
Ethiopia	AMHARA	2015	59.37	36.57	94.37	RW2
Ethiopia	AMHARA	2016	55.43	29.00	102.09	RW2
Ethiopia	AMHARA	2017	51.88	22.59	113.17	RW2
Ethiopia	AMHARA	2018	48.18	17.22	128.20	RW2
Ethiopia	AMHARA	2019	44.98	12.78	145.95	RW2
Ethiopia	BENISHANGUL-GUMUZ	1980	279.93	215.62	354.51	RW2
Ethiopia	BENISHANGUL-GUMUZ	1981	274.94	225.34	331.55	RW2
Ethiopia	BENISHANGUL-GUMUZ	1982	270.42	225.60	320.25	RW2
Ethiopia	BENISHANGUL-GUMUZ	1983	265.78	221.50	316.44	RW2
Ethiopia	BENISHANGUL-GUMUZ	1984	261.36	216.60	311.97	RW2
Ethiopia	BENISHANGUL-GUMUZ	1985	257.09	216.26	302.38	RW2
Ethiopia	BENISHANGUL-GUMUZ	1986	253.68	217.36	294.06	RW2
Ethiopia	BENISHANGUL-GUMUZ	1987	251.00	216.64	288.12	RW2
Ethiopia	BENISHANGUL-GUMUZ	1988	248.84	214.19	286.47	RW2
Ethiopia	BENISHANGUL-GUMUZ	1989	246.78	210.08	286.38	RW2
Ethiopia	BENISHANGUL-GUMUZ	1990	245.17	210.06	284.10	RW2
Ethiopia	BENISHANGUL-GUMUZ	1991	242.31	210.08	277.74	RW2
Ethiopia	BENISHANGUL-GUMUZ	1992	238.30	207.00	271.70	RW2
Ethiopia	BENISHANGUL-GUMUZ	1993	233.22	202.13	267.52	RW2

Continued on next page

Country	Region	Year	Median	Lower	Upper	Method
Ethiopia	BENISHANGUL-GUMUZ	1994	226.96	194.15	263.47	RW2
Ethiopia	BENISHANGUL-GUMUZ	1995	219.70	187.15	254.93	RW2
Ethiopia	BENISHANGUL-GUMUZ	1996	212.80	183.35	245.23	RW2
Ethiopia	BENISHANGUL-GUMUZ	1997	206.20	178.24	237.06	RW2
Ethiopia	BENISHANGUL-GUMUZ	1998	199.83	171.70	231.71	RW2
Ethiopia	BENISHANGUL-GUMUZ	1999	193.64	164.34	226.74	RW2
Ethiopia	BENISHANGUL-GUMUZ	2000	187.88	159.26	221.20	RW2
Ethiopia	BENISHANGUL-GUMUZ	2001	181.03	154.60	211.61	RW2
Ethiopia	BENISHANGUL-GUMUZ	2002	173.33	148.76	202.13	RW2
Ethiopia	BENISHANGUL-GUMUZ	2003	164.96	139.80	194.20	RW2
Ethiopia	BENISHANGUL-GUMUZ	2004	155.99	130.80	185.89	RW2
Ethiopia	BENISHANGUL-GUMUZ	2005	146.23	121.47	175.11	RW2
Ethiopia	BENISHANGUL-GUMUZ	2006	137.02	114.71	163.25	RW2
Ethiopia	BENISHANGUL-GUMUZ	2007	128.06	107.30	152.47	RW2
Ethiopia	BENISHANGUL-GUMUZ	2008	119.52	98.73	143.96	RW2
Ethiopia	BENISHANGUL-GUMUZ	2009	111.61	90.09	137.32	RW2
Ethiopia	BENISHANGUL-GUMUZ	2010	104.20	82.46	131.06	RW2
Ethiopia	BENISHANGUL-GUMUZ	2011	97.32	76.78	121.96	RW2
Ethiopia	BENISHANGUL-GUMUZ	2012	90.84	71.24	114.02	RW2
Ethiopia	BENISHANGUL-GUMUZ	2013	84.83	64.28	109.26	RW2
Ethiopia	BENISHANGUL-GUMUZ	2014	79.06	55.38	109.97	RW2
Ethiopia	BENISHANGUL-GUMUZ	2015	73.55	44.69	117.15	RW2
Ethiopia	BENISHANGUL-GUMUZ	2016	68.44	35.53	125.93	RW2
Ethiopia	BENISHANGUL-GUMUZ	2017	63.61	27.64	138.63	RW2
Ethiopia	BENISHANGUL-GUMUZ	2018	59.11	20.75	155.83	RW2
Ethiopia	BENISHANGUL-GUMUZ	2019	55.04	15.39	177.06	RW2
Ethiopia	DIRE DAWA	1980	230.30	171.06	301.31	RW2
Ethiopia	DIRE DAWA	1981	227.13	179.84	280.85	RW2
Ethiopia	DIRE DAWA	1982	223.65	181.78	271.45	RW2
Ethiopia	DIRE DAWA	1983	220.34	179.12	267.43	RW2
Ethiopia	DIRE DAWA	1984	217.24	176.26	264.64	RW2
Ethiopia	DIRE DAWA	1985	214.15	176.92	256.51	RW2
Ethiopia	DIRE DAWA	1986	211.73	179.09	249.43	RW2
Ethiopia	DIRE DAWA	1987	209.75	178.94	244.59	RW2
Ethiopia	DIRE DAWA	1988	208.24	176.42	243.62	RW2
Ethiopia	DIRE DAWA	1989	206.72	174.05	243.38	RW2
Ethiopia	DIRE DAWA	1990	205.44	173.93	241.56	RW2
Ethiopia	DIRE DAWA	1991	202.56	173.02	235.70	RW2
Ethiopia	DIRE DAWA	1992	198.48	170.40	230.06	RW2
Ethiopia	DIRE DAWA	1993	193.36	164.91	225.41	RW2
Ethiopia	DIRE DAWA	1994	187.15	158.16	220.94	RW2
Ethiopia	DIRE DAWA	1995	179.37	150.67	211.52	RW2
Ethiopia	DIRE DAWA	1996	172.23	146.56	201.29	RW2
Ethiopia	DIRE DAWA	1997	164.97	140.92	192.80	RW2
Ethiopia	DIRE DAWA	1998	158.17	134.18	185.83	RW2
Ethiopia	DIRE DAWA	1999	151.58	127.02	180.01	RW2
Ethiopia	DIRE DAWA	2000	145.35	121.73	173.21	RW2
Ethiopia	DIRE DAWA	2001	138.68	117.21	163.81	RW2
Ethiopia	DIRE DAWA	2002	131.70	111.50	154.87	RW2
Ethiopia	DIRE DAWA	2003	124.45	104.50	147.36	RW2
Ethiopia	DIRE DAWA	2004	117.18	97.37	140.44	RW2
Ethiopia	DIRE DAWA	2005	109.64	90.26	132.51	RW2
Ethiopia	DIRE DAWA	2006	102.74	85.39	122.97	RW2
Ethiopia	DIRE DAWA	2007	96.24	80.28	115.03	RW2
Ethiopia	DIRE DAWA	2008	90.28	74.23	109.38	RW2
Ethiopia	DIRE DAWA	2009	84.83	68.39	104.90	RW2
Ethiopia	DIRE DAWA	2010	79.89	63.13	101.10	RW2
Ethiopia	DIRE DAWA	2011	75.32	59.41	95.09	RW2
Ethiopia	DIRE DAWA	2012	71.01	56.04	89.43	RW2
Ethiopia	DIRE DAWA	2013	66.93	51.32	86.47	RW2
Ethiopia	DIRE DAWA	2014	63.03	44.67	87.99	RW2
Ethiopia	DIRE DAWA	2015	59.34	36.55	95.16	RW2
Ethiopia	DIRE DAWA	2016	56.00	29.13	103.96	RW2

Continued on next page

Country	Region	Year	Median	Lower	Upper	Method
Ethiopia	DIRE DAWA	2017	52.51	22.99	116.30	RW2
Ethiopia	DIRE DAWA	2018	49.39	17.67	131.49	RW2
Ethiopia	DIRE DAWA	2019	46.65	13.18	151.21	RW2
Ethiopia	GAMBELA	1980	281.25	215.98	356.22	RW2
Ethiopia	GAMBELA	1981	277.25	227.01	332.36	RW2
Ethiopia	GAMBELA	1982	272.82	227.81	322.13	RW2
Ethiopia	GAMBELA	1983	268.62	223.41	319.08	RW2
Ethiopia	GAMBELA	1984	264.65	219.16	315.92	RW2
Ethiopia	GAMBELA	1985	260.72	219.20	307.27	RW2
Ethiopia	GAMBELA	1986	257.52	219.70	298.79	RW2
Ethiopia	GAMBELA	1987	254.67	219.28	293.90	RW2
Ethiopia	GAMBELA	1988	252.05	215.89	291.37	RW2
Ethiopia	GAMBELA	1989	249.33	212.23	290.13	RW2
Ethiopia	GAMBELA	1990	246.99	210.72	287.70	RW2
Ethiopia	GAMBELA	1991	242.58	208.94	279.93	RW2
Ethiopia	GAMBELA	1992	236.56	204.52	272.34	RW2
Ethiopia	GAMBELA	1993	228.94	197.16	265.02	RW2
Ethiopia	GAMBELA	1994	219.97	187.81	257.31	RW2
Ethiopia	GAMBELA	1995	209.56	177.41	244.78	RW2
Ethiopia	GAMBELA	1996	199.35	171.01	231.65	RW2
Ethiopia	GAMBELA	1997	189.45	162.86	219.08	RW2
Ethiopia	GAMBELA	1998	179.69	153.25	209.22	RW2
Ethiopia	GAMBELA	1999	170.49	143.70	201.07	RW2
Ethiopia	GAMBELA	2000	161.96	135.64	191.67	RW2
Ethiopia	GAMBELA	2001	153.11	129.25	180.23	RW2
Ethiopia	GAMBELA	2002	144.17	122.08	169.23	RW2
Ethiopia	GAMBELA	2003	135.26	113.64	160.04	RW2
Ethiopia	GAMBELA	2004	126.50	104.60	151.80	RW2
Ethiopia	GAMBELA	2005	117.72	96.85	141.90	RW2
Ethiopia	GAMBELA	2006	109.82	90.99	131.61	RW2
Ethiopia	GAMBELA	2007	102.47	85.14	122.87	RW2
Ethiopia	GAMBELA	2008	95.77	78.78	115.99	RW2
Ethiopia	GAMBELA	2009	89.64	72.14	111.01	RW2
Ethiopia	GAMBELA	2010	84.23	66.42	106.24	RW2
Ethiopia	GAMBELA	2011	79.11	62.59	99.56	RW2
Ethiopia	GAMBELA	2012	74.29	58.81	93.58	RW2
Ethiopia	GAMBELA	2013	69.77	53.85	90.15	RW2
Ethiopia	GAMBELA	2014	65.50	46.86	91.22	RW2
Ethiopia	GAMBELA	2015	61.50	37.90	98.24	RW2
Ethiopia	GAMBELA	2016	57.81	30.56	107.04	RW2
Ethiopia	GAMBELA	2017	54.07	23.79	120.14	RW2
Ethiopia	GAMBELA	2018	50.76	18.22	135.21	RW2
Ethiopia	GAMBELA	2019	47.64	13.69	156.97	RW2
Ethiopia	HARARI	1980	263.93	193.26	348.42	RW2
Ethiopia	HARARI	1981	256.70	200.82	321.82	RW2
Ethiopia	HARARI	1982	249.56	199.98	305.38	RW2
Ethiopia	HARARI	1983	242.49	195.37	296.30	RW2
Ethiopia	HARARI	1984	235.92	190.84	288.38	RW2
Ethiopia	HARARI	1985	229.39	188.74	275.54	RW2
Ethiopia	HARARI	1986	224.04	188.17	264.94	RW2
Ethiopia	HARARI	1987	219.47	186.42	256.88	RW2
Ethiopia	HARARI	1988	215.37	182.33	251.95	RW2
Ethiopia	HARARI	1989	211.46	177.89	249.32	RW2
Ethiopia	HARARI	1990	208.01	175.69	244.55	RW2
Ethiopia	HARARI	1991	203.04	173.40	236.77	RW2
Ethiopia	HARARI	1992	196.51	168.66	227.52	RW2
Ethiopia	HARARI	1993	188.78	161.17	220.48	RW2
Ethiopia	HARARI	1994	180.02	151.94	212.63	RW2
Ethiopia	HARARI	1995	169.80	142.33	200.77	RW2
Ethiopia	HARARI	1996	160.32	135.73	187.99	RW2
Ethiopia	HARARI	1997	150.90	128.10	176.85	RW2
Ethiopia	HARARI	1998	142.03	119.53	167.58	RW2
Ethiopia	HARARI	1999	133.90	111.11	160.15	RW2

Continued on next page

Country	Region	Year	Median	Lower	Upper	Method
Ethiopia	HARARI	2000	126.44	104.60	151.79	RW2
Ethiopia	HARARI	2001	118.88	99.10	141.61	RW2
Ethiopia	HARARI	2002	111.58	92.70	132.70	RW2
Ethiopia	HARARI	2003	104.41	86.06	125.51	RW2
Ethiopia	HARARI	2004	97.58	79.34	118.75	RW2
Ethiopia	HARARI	2005	90.82	73.52	111.03	RW2
Ethiopia	HARARI	2006	84.99	69.35	103.42	RW2
Ethiopia	HARARI	2007	79.58	65.12	96.44	RW2
Ethiopia	HARARI	2008	74.78	60.55	92.07	RW2
Ethiopia	HARARI	2009	70.62	55.95	88.80	RW2
Ethiopia	HARARI	2010	66.91	51.95	86.09	RW2
Ethiopia	HARARI	2011	63.46	49.12	81.89	RW2
Ethiopia	HARARI	2012	60.16	46.37	78.51	RW2
Ethiopia	HARARI	2013	57.11	42.66	77.24	RW2
Ethiopia	HARARI	2014	54.22	37.32	79.30	RW2
Ethiopia	HARARI	2015	51.45	30.74	86.58	RW2
Ethiopia	HARARI	2016	48.74	24.98	95.58	RW2
Ethiopia	HARARI	2017	46.33	19.57	107.83	RW2
Ethiopia	HARARI	2018	43.84	15.07	122.97	RW2
Ethiopia	HARARI	2019	41.53	11.51	143.31	RW2
Ethiopia	OROMIYA	1980	245.61	195.61	303.62	RW2
Ethiopia	OROMIYA	1981	240.32	204.85	279.44	RW2
Ethiopia	OROMIYA	1982	235.00	203.54	269.72	RW2
Ethiopia	OROMIYA	1983	229.84	196.36	268.12	RW2
Ethiopia	OROMIYA	1984	224.93	189.71	265.37	RW2
Ethiopia	OROMIYA	1985	220.17	187.99	256.33	RW2
Ethiopia	OROMIYA	1986	216.31	187.29	248.07	RW2
Ethiopia	OROMIYA	1987	212.85	185.63	242.41	RW2
Ethiopia	OROMIYA	1988	210.10	181.69	240.57	RW2
Ethiopia	OROMIYA	1989	207.37	177.26	239.98	RW2
Ethiopia	OROMIYA	1990	205.07	176.44	237.29	RW2
Ethiopia	OROMIYA	1991	201.31	175.02	230.86	RW2
Ethiopia	OROMIYA	1992	196.54	171.57	223.99	RW2
Ethiopia	OROMIYA	1993	190.65	165.67	218.62	RW2
Ethiopia	OROMIYA	1994	183.71	157.60	213.56	RW2
Ethiopia	OROMIYA	1995	175.65	149.80	203.87	RW2
Ethiopia	OROMIYA	1996	168.08	144.97	193.32	RW2
Ethiopia	OROMIYA	1997	160.69	139.35	184.64	RW2
Ethiopia	OROMIYA	1998	153.66	132.78	177.55	RW2
Ethiopia	OROMIYA	1999	146.96	124.89	171.99	RW2
Ethiopia	OROMIYA	2000	140.69	119.45	165.12	RW2
Ethiopia	OROMIYA	2001	134.03	115.01	155.93	RW2
Ethiopia	OROMIYA	2002	127.06	109.51	146.87	RW2
Ethiopia	OROMIYA	2003	120.08	102.65	140.34	RW2
Ethiopia	OROMIYA	2004	112.82	95.01	133.70	RW2
Ethiopia	OROMIYA	2005	105.51	88.41	125.05	RW2
Ethiopia	OROMIYA	2006	98.74	83.55	116.07	RW2
Ethiopia	OROMIYA	2007	92.47	78.54	108.38	RW2
Ethiopia	OROMIYA	2008	86.64	72.87	102.69	RW2
Ethiopia	OROMIYA	2009	81.33	67.01	98.35	RW2
Ethiopia	OROMIYA	2010	76.49	61.98	94.34	RW2
Ethiopia	OROMIYA	2011	72.04	58.56	87.81	RW2
Ethiopia	OROMIYA	2012	67.78	55.81	82.23	RW2
Ethiopia	OROMIYA	2013	63.83	51.39	79.18	RW2
Ethiopia	OROMIYA	2014	60.02	44.45	80.60	RW2
Ethiopia	OROMIYA	2015	56.53	36.07	87.96	RW2
Ethiopia	OROMIYA	2016	53.26	28.94	96.41	RW2
Ethiopia	OROMIYA	2017	49.98	22.56	107.06	RW2
Ethiopia	OROMIYA	2018	46.86	17.36	121.31	RW2
Ethiopia	OROMIYA	2019	44.12	13.02	141.41	RW2
Ethiopia	SNNP	1980	252.39	196.23	317.16	RW2
Ethiopia	SNNP	1981	248.72	206.99	294.61	RW2
Ethiopia	SNNP	1982	245.23	208.18	286.06	RW2

Continued on next page

Country	Region	Year	Median	Lower	Upper	Method
Ethiopia	SNNP	1983	241.47	203.62	284.39	RW2
Ethiopia	SNNP	1984	238.10	199.87	281.94	RW2
Ethiopia	SNNP	1985	234.66	199.46	274.01	RW2
Ethiopia	SNNP	1986	231.88	200.31	266.71	RW2
Ethiopia	SNNP	1987	229.60	200.43	262.46	RW2
Ethiopia	SNNP	1988	227.42	196.80	261.14	RW2
Ethiopia	SNNP	1989	225.14	192.47	260.50	RW2
Ethiopia	SNNP	1990	223.11	192.13	257.92	RW2
Ethiopia	SNNP	1991	219.36	190.74	251.35	RW2
Ethiopia	SNNP	1992	214.24	187.47	243.96	RW2
Ethiopia	SNNP	1993	207.80	180.23	238.56	RW2
Ethiopia	SNNP	1994	200.23	171.99	232.20	RW2
Ethiopia	SNNP	1995	191.29	163.47	221.79	RW2
Ethiopia	SNNP	1996	182.88	158.27	210.63	RW2
Ethiopia	SNNP	1997	174.52	151.81	200.28	RW2
Ethiopia	SNNP	1998	166.63	143.75	192.36	RW2
Ethiopia	SNNP	1999	159.04	135.24	185.98	RW2
Ethiopia	SNNP	2000	151.91	129.19	178.41	RW2
Ethiopia	SNNP	2001	144.39	123.72	168.00	RW2
Ethiopia	SNNP	2002	136.82	117.73	158.62	RW2
Ethiopia	SNNP	2003	128.99	109.86	150.42	RW2
Ethiopia	SNNP	2004	121.25	101.85	143.68	RW2
Ethiopia	SNNP	2005	113.38	94.55	134.99	RW2
Ethiopia	SNNP	2006	106.28	89.32	125.55	RW2
Ethiopia	SNNP	2007	99.52	84.03	117.61	RW2
Ethiopia	SNNP	2008	93.44	77.80	111.73	RW2
Ethiopia	SNNP	2009	87.78	71.43	107.52	RW2
Ethiopia	SNNP	2010	82.82	66.01	103.65	RW2
Ethiopia	SNNP	2011	78.00	62.38	97.10	RW2
Ethiopia	SNNP	2012	73.54	59.05	91.87	RW2
Ethiopia	SNNP	2013	69.37	53.98	89.02	RW2
Ethiopia	SNNP	2014	65.40	46.87	90.67	RW2
Ethiopia	SNNP	2015	61.63	38.30	97.88	RW2
Ethiopia	SNNP	2016	58.07	30.61	107.55	RW2
Ethiopia	SNNP	2017	54.65	24.07	120.43	RW2
Ethiopia	SNNP	2018	51.32	18.65	135.83	RW2
Ethiopia	SNNP	2019	48.39	13.94	156.73	RW2
Ethiopia	SOMALI	1980	182.94	133.38	246.41	RW2
Ethiopia	SOMALI	1981	180.82	140.82	229.83	RW2
Ethiopia	SOMALI	1982	178.72	142.11	222.84	RW2
Ethiopia	SOMALI	1983	176.55	140.65	219.97	RW2
Ethiopia	SOMALI	1984	174.69	139.03	217.99	RW2
Ethiopia	SOMALI	1985	172.99	139.83	211.39	RW2
Ethiopia	SOMALI	1986	171.74	141.91	206.20	RW2
Ethiopia	SOMALI	1987	171.05	142.88	203.28	RW2
Ethiopia	SOMALI	1988	170.84	142.39	203.17	RW2
Ethiopia	SOMALI	1989	170.72	141.14	203.95	RW2
Ethiopia	SOMALI	1990	170.94	142.81	204.24	RW2
Ethiopia	SOMALI	1991	170.07	143.25	200.73	RW2
Ethiopia	SOMALI	1992	168.13	142.72	196.82	RW2
Ethiopia	SOMALI	1993	165.25	139.98	194.25	RW2
Ethiopia	SOMALI	1994	161.39	135.40	191.57	RW2
Ethiopia	SOMALI	1995	156.42	131.34	184.95	RW2
Ethiopia	SOMALI	1996	151.87	128.93	177.93	RW2
Ethiopia	SOMALI	1997	147.51	126.13	171.91	RW2
Ethiopia	SOMALI	1998	143.37	121.59	167.96	RW2
Ethiopia	SOMALI	1999	139.33	116.85	164.74	RW2
Ethiopia	SOMALI	2000	135.82	114.27	160.99	RW2
Ethiopia	SOMALI	2001	131.64	111.58	154.76	RW2
Ethiopia	SOMALI	2002	127.22	108.53	148.79	RW2
Ethiopia	SOMALI	2003	122.26	103.35	143.88	RW2
Ethiopia	SOMALI	2004	117.08	97.92	139.58	RW2
Ethiopia	SOMALI	2005	111.48	92.64	133.22	RW2

Continued on next page

Country	Region	Year	Median	Lower	Upper	Method
Ethiopia	SOMALI	2006	106.33	89.35	125.99	RW2
Ethiopia	SOMALI	2007	101.40	85.53	119.83	RW2
Ethiopia	SOMALI	2008	96.85	80.88	115.50	RW2
Ethiopia	SOMALI	2009	92.65	75.85	112.59	RW2
Ethiopia	SOMALI	2010	88.81	71.58	109.90	RW2
Ethiopia	SOMALI	2011	85.24	69.09	104.79	RW2
Ethiopia	SOMALI	2012	81.77	66.92	99.73	RW2
Ethiopia	SOMALI	2013	78.50	62.71	98.10	RW2
Ethiopia	SOMALI	2014	75.30	55.65	101.26	RW2
Ethiopia	SOMALI	2015	72.28	46.06	111.81	RW2
Ethiopia	SOMALI	2016	69.25	37.54	124.38	RW2
Ethiopia	SOMALI	2017	66.47	29.93	143.18	RW2
Ethiopia	SOMALI	2018	63.77	23.36	163.56	RW2
Ethiopia	SOMALI	2019	61.25	18.14	188.46	RW2
Ethiopia	TIGRAY	1980	277.45	217.68	345.51	RW2
Ethiopia	TIGRAY	1981	268.43	225.16	315.68	RW2
Ethiopia	TIGRAY	1982	259.36	221.49	300.87	RW2
Ethiopia	TIGRAY	1983	250.48	211.76	294.02	RW2
Ethiopia	TIGRAY	1984	242.01	203.12	286.13	RW2
Ethiopia	TIGRAY	1985	233.64	198.14	272.88	RW2
Ethiopia	TIGRAY	1986	226.26	194.91	260.79	RW2
Ethiopia	TIGRAY	1987	219.41	190.68	251.06	RW2
Ethiopia	TIGRAY	1988	212.93	183.40	245.18	RW2
Ethiopia	TIGRAY	1989	206.58	175.83	240.21	RW2
Ethiopia	TIGRAY	1990	200.60	171.59	233.90	RW2
Ethiopia	TIGRAY	1991	193.45	167.04	223.41	RW2
Ethiopia	TIGRAY	1992	185.41	160.51	213.13	RW2
Ethiopia	TIGRAY	1993	176.46	152.10	203.98	RW2
Ethiopia	TIGRAY	1994	166.92	142.01	195.82	RW2
Ethiopia	TIGRAY	1995	156.68	132.63	183.42	RW2
Ethiopia	TIGRAY	1996	147.32	125.87	171.28	RW2
Ethiopia	TIGRAY	1997	138.49	119.03	160.37	RW2
Ethiopia	TIGRAY	1998	130.31	111.29	152.38	RW2
Ethiopia	TIGRAY	1999	122.75	103.16	145.44	RW2
Ethiopia	TIGRAY	2000	115.95	97.10	137.67	RW2
Ethiopia	TIGRAY	2001	109.04	92.37	128.29	RW2
Ethiopia	TIGRAY	2002	102.26	86.87	119.89	RW2
Ethiopia	TIGRAY	2003	95.44	80.51	112.82	RW2
Ethiopia	TIGRAY	2004	88.82	73.92	106.35	RW2
Ethiopia	TIGRAY	2005	82.25	67.86	99.12	RW2
Ethiopia	TIGRAY	2006	76.21	63.47	91.18	RW2
Ethiopia	TIGRAY	2007	70.59	59.04	84.16	RW2
Ethiopia	TIGRAY	2008	65.47	54.20	78.93	RW2
Ethiopia	TIGRAY	2009	60.73	49.33	74.66	RW2
Ethiopia	TIGRAY	2010	56.55	44.90	71.00	RW2
Ethiopia	TIGRAY	2011	52.51	42.00	65.70	RW2
Ethiopia	TIGRAY	2012	48.87	39.19	60.87	RW2
Ethiopia	TIGRAY	2013	45.43	35.39	58.23	RW2
Ethiopia	TIGRAY	2014	42.24	30.30	58.42	RW2
Ethiopia	TIGRAY	2015	39.26	24.33	63.10	RW2
Ethiopia	TIGRAY	2016	36.46	19.30	68.52	RW2
Ethiopia	TIGRAY	2017	33.89	14.84	76.62	RW2
Ethiopia	TIGRAY	2018	31.39	11.04	86.55	RW2
Ethiopia	TIGRAY	2019	29.11	8.26	98.99	RW2
Gabon	ALL	1980	113.20	106.00	120.94	IHME
Gabon	ALL	1980	118.13	80.31	170.99	RW2
Gabon	ALL	1980	117.30	97.50	141.50	UN
Gabon	ALL	1981	109.24	102.74	116.37	IHME
Gabon	ALL	1981	114.16	85.72	150.32	RW2
Gabon	ALL	1981	113.20	95.10	134.60	UN
Gabon	ALL	1982	105.77	99.66	112.18	IHME
Gabon	ALL	1982	110.34	86.11	140.62	RW2
Gabon	ALL	1982	109.30	92.50	128.90	UN

Continued on next page

Country	Region	Year	Median	Lower	Upper	Method
Gabon	ALL	1983	102.17	96.15	107.99	IHME
Gabon	ALL	1983	106.51	82.33	137.29	RW2
Gabon	ALL	1983	106.00	90.10	124.40	UN
Gabon	ALL	1984	98.45	92.95	104.03	IHME
Gabon	ALL	1984	103.29	78.06	135.64	RW2
Gabon	ALL	1984	103.00	88.00	120.40	UN
Gabon	ALL	1985	94.98	89.57	100.25	IHME
Gabon	ALL	1985	99.83	76.22	128.97	RW2
Gabon	ALL	1985	100.40	85.90	117.20	UN
Gabon	ALL	1986	91.78	86.84	96.95	IHME
Gabon	ALL	1986	97.41	75.34	124.37	RW2
Gabon	ALL	1986	98.20	84.30	114.40	UN
Gabon	ALL	1987	89.43	84.72	94.33	IHME
Gabon	ALL	1987	95.64	74.80	121.32	RW2
Gabon	ALL	1987	96.50	82.90	112.10	UN
Gabon	ALL	1988	87.47	83.10	92.21	IHME
Gabon	ALL	1988	94.37	73.23	120.03	RW2
Gabon	ALL	1988	95.10	82.10	110.10	UN
Gabon	ALL	1989	85.34	81.13	89.90	IHME
Gabon	ALL	1989	93.61	72.11	119.82	RW2
Gabon	ALL	1989	93.90	81.50	108.70	UN
Gabon	ALL	1990	83.27	79.32	87.80	IHME
Gabon	ALL	1990	93.54	72.84	120.20	RW2
Gabon	ALL	1990	92.90	80.80	107.50	UN
Gabon	ALL	1991	81.41	77.53	85.91	IHME
Gabon	ALL	1991	93.24	73.40	117.89	RW2
Gabon	ALL	1991	92.00	80.10	106.60	UN
Gabon	ALL	1992	79.64	75.80	83.77	IHME
Gabon	ALL	1992	92.90	73.43	116.87	RW2
Gabon	ALL	1992	91.30	79.60	105.70	UN
Gabon	ALL	1993	78.13	74.33	82.16	IHME
Gabon	ALL	1993	92.43	72.72	117.02	RW2
Gabon	ALL	1993	90.60	79.10	104.90	UN
Gabon	ALL	1994	76.74	72.99	80.71	IHME
Gabon	ALL	1994	91.72	71.26	118.03	RW2
Gabon	ALL	1994	89.80	78.50	104.20	UN
Gabon	ALL	1995	75.36	71.71	79.30	IHME
Gabon	ALL	1995	90.75	70.02	116.31	RW2
Gabon	ALL	1995	89.10	77.90	103.70	UN
Gabon	ALL	1996	74.63	71.00	78.45	IHME
Gabon	ALL	1996	89.80	69.90	114.69	RW2
Gabon	ALL	1996	88.50	77.30	103.10	UN
Gabon	ALL	1997	73.34	69.78	77.19	IHME
Gabon	ALL	1997	88.85	69.53	112.75	RW2
Gabon	ALL	1997	87.90	76.60	102.30	UN
Gabon	ALL	1998	72.43	68.75	76.37	IHME
Gabon	ALL	1998	87.93	68.16	113.13	RW2
Gabon	ALL	1998	87.20	76.00	101.50	UN
Gabon	ALL	1999	71.78	68.13	75.69	IHME
Gabon	ALL	1999	86.87	65.96	113.34	RW2
Gabon	ALL	1999	86.30	75.20	100.70	UN
Gabon	ALL	2000	71.39	67.71	75.29	IHME
Gabon	ALL	2000	85.85	63.70	115.69	RW2
Gabon	ALL	2000	85.30	74.10	100.00	UN
Gabon	ALL	2001	70.97	67.20	75.08	IHME
Gabon	ALL	2001	84.40	61.79	115.30	RW2
Gabon	ALL	2001	84.00	72.90	98.80	UN
Gabon	ALL	2002	70.22	66.39	74.40	IHME
Gabon	ALL	2002	82.61	60.22	113.53	RW2
Gabon	ALL	2002	82.50	71.60	97.10	UN
Gabon	ALL	2003	69.14	65.13	73.28	IHME
Gabon	ALL	2003	80.62	58.72	110.73	RW2
Gabon	ALL	2003	80.80	69.90	95.00	UN

Continued on next page

Country	Region	Year	Median	Lower	Upper	Method
Gabon	ALL	2004	68.06	63.89	72.19	IHME
Gabon	ALL	2004	78.10	56.76	107.33	RW2
Gabon	ALL	2004	78.80	68.00	92.40	UN
Gabon	ALL	2005	66.78	62.57	70.99	IHME
Gabon	ALL	2005	75.48	55.41	102.08	RW2
Gabon	ALL	2005	76.70	66.00	89.60	UN
Gabon	ALL	2006	65.45	61.12	69.91	IHME
Gabon	ALL	2006	72.81	53.99	97.32	RW2
Gabon	ALL	2006	74.50	63.90	86.70	UN
Gabon	ALL	2007	63.63	59.18	68.35	IHME
Gabon	ALL	2007	70.19	52.37	93.31	RW2
Gabon	ALL	2007	71.90	61.50	83.50	UN
Gabon	ALL	2008	61.32	56.73	66.11	IHME
Gabon	ALL	2008	67.77	50.31	90.50	RW2
Gabon	ALL	2008	68.90	58.70	80.70	UN
Gabon	ALL	2009	59.39	54.78	64.53	IHME
Gabon	ALL	2009	65.24	48.14	87.86	RW2
Gabon	ALL	2009	66.10	55.60	78.10	UN
Gabon	ALL	2010	57.50	52.58	62.80	IHME
Gabon	ALL	2010	62.92	46.72	84.61	RW2
Gabon	ALL	2010	63.30	52.30	76.00	UN
Gabon	ALL	2011	55.08	49.94	60.55	IHME
Gabon	ALL	2011	60.71	45.94	79.77	RW2
Gabon	ALL	2011	60.60	48.80	74.20	UN
Gabon	ALL	2012	52.33	46.96	58.09	IHME
Gabon	ALL	2012	58.53	44.87	75.93	RW2
Gabon	ALL	2012	57.70	45.10	72.30	UN
Gabon	ALL	2013	49.87	44.42	55.98	IHME
Gabon	ALL	2013	56.48	41.74	75.86	RW2
Gabon	ALL	2013	54.80	41.50	70.60	UN
Gabon	ALL	2014	47.53	41.85	53.83	IHME
Gabon	ALL	2014	54.42	35.86	81.81	RW2
Gabon	ALL	2014	52.30	38.40	69.50	UN
Gabon	ALL	2015	46.11	40.23	52.69	IHME
Gabon	ALL	2015	52.36	28.38	95.15	RW2
Gabon	ALL	2015	50.80	35.90	70.10	UN
Gabon	ALL	2016	50.59	22.41	111.76	RW2
Gabon	ALL	2017	48.61	17.01	133.64	RW2
Gabon	ALL	2018	46.82	12.78	163.40	RW2
Gabon	ALL	2019	45.01	8.99	197.28	RW2
Gabon	ALL	15-19	48.61	17.33	130.99	RW2
Gabon	EAST	1980	80.46	50.50	126.53	RW2
Gabon	EAST	1981	77.85	53.36	112.83	RW2
Gabon	EAST	1982	75.11	53.74	104.24	RW2
Gabon	EAST	1983	72.51	52.40	99.73	RW2
Gabon	EAST	1984	69.98	50.51	96.19	RW2
Gabon	EAST	1985	67.66	49.83	91.42	RW2
Gabon	EAST	1986	65.41	49.04	86.59	RW2
Gabon	EAST	1987	63.30	48.18	82.87	RW2
Gabon	EAST	1988	61.45	46.93	80.25	RW2
Gabon	EAST	1989	59.94	45.88	78.18	RW2
Gabon	EAST	1990	58.67	45.15	75.57	RW2
Gabon	EAST	1991	58.35	45.66	73.99	RW2
Gabon	EAST	1992	58.68	46.23	73.86	RW2
Gabon	EAST	1993	59.75	46.93	75.45	RW2
Gabon	EAST	1994	61.67	47.78	78.51	RW2
Gabon	EAST	1995	64.33	49.95	82.61	RW2
Gabon	EAST	1996	67.23	52.60	85.62	RW2
Gabon	EAST	1997	70.26	55.23	89.33	RW2
Gabon	EAST	1998	73.25	57.28	93.66	RW2
Gabon	EAST	1999	75.91	57.96	98.68	RW2
Gabon	EAST	2000	78.06	58.70	103.88	RW2
Gabon	EAST	2001	79.41	59.22	106.46	RW2

Continued on next page

Country	Region	Year	Median	Lower	Upper	Method
Gabon	EAST	2002	79.75	59.27	107.57	RW2
Gabon	EAST	2003	79.06	58.56	107.39	RW2
Gabon	EAST	2004	77.60	57.09	105.67	RW2
Gabon	EAST	2005	75.12	55.25	101.66	RW2
Gabon	EAST	2006	72.59	53.64	97.81	RW2
Gabon	EAST	2007	69.81	51.19	94.28	RW2
Gabon	EAST	2008	67.09	48.56	91.72	RW2
Gabon	EAST	2009	64.29	45.93	89.02	RW2
Gabon	EAST	2010	61.82	43.95	86.33	RW2
Gabon	EAST	2011	59.26	42.28	82.57	RW2
Gabon	EAST	2012	56.87	40.40	79.50	RW2
Gabon	EAST	2013	54.60	37.21	79.59	RW2
Gabon	EAST	2014	52.41	32.40	83.37	RW2
Gabon	EAST	2015	50.27	26.42	93.40	RW2
Gabon	EAST	2016	48.10	21.05	106.29	RW2
Gabon	EAST	2017	46.19	16.35	124.58	RW2
Gabon	EAST	2018	44.12	12.28	148.81	RW2
Gabon	EAST	2019	42.31	9.06	181.16	RW2
Gabon	LIBREVILLE,PORT-GENTIL	1980	118.66	75.62	176.84	RW2
Gabon	LIBREVILLE,PORT-GENTIL	1981	116.15	81.99	158.76	RW2
Gabon	LIBREVILLE,PORT-GENTIL	1982	113.59	84.27	149.60	RW2
Gabon	LIBREVILLE,PORT-GENTIL	1983	110.78	83.64	144.93	RW2
Gabon	LIBREVILLE,PORT-GENTIL	1984	108.39	82.13	141.31	RW2
Gabon	LIBREVILLE,PORT-GENTIL	1985	105.93	81.70	136.33	RW2
Gabon	LIBREVILLE,PORT-GENTIL	1986	103.45	81.23	131.01	RW2
Gabon	LIBREVILLE,PORT-GENTIL	1987	101.07	80.13	127.10	RW2
Gabon	LIBREVILLE,PORT-GENTIL	1988	98.65	78.26	124.21	RW2
Gabon	LIBREVILLE,PORT-GENTIL	1989	96.51	76.27	122.62	RW2
Gabon	LIBREVILLE,PORT-GENTIL	1990	94.25	74.34	119.01	RW2
Gabon	LIBREVILLE,PORT-GENTIL	1991	92.83	74.29	116.29	RW2
Gabon	LIBREVILLE,PORT-GENTIL	1992	92.04	73.96	114.78	RW2
Gabon	LIBREVILLE,PORT-GENTIL	1993	91.71	72.95	115.30	RW2
Gabon	LIBREVILLE,PORT-GENTIL	1994	91.94	72.16	116.63	RW2
Gabon	LIBREVILLE,PORT-GENTIL	1995	92.66	72.87	118.20	RW2
Gabon	LIBREVILLE,PORT-GENTIL	1996	93.30	73.55	118.09	RW2
Gabon	LIBREVILLE,PORT-GENTIL	1997	93.82	73.85	118.69	RW2
Gabon	LIBREVILLE,PORT-GENTIL	1998	94.12	72.77	120.37	RW2
Gabon	LIBREVILLE,PORT-GENTIL	1999	93.69	70.30	122.66	RW2
Gabon	LIBREVILLE,PORT-GENTIL	2000	93.07	68.11	124.46	RW2
Gabon	LIBREVILLE,PORT-GENTIL	2001	91.36	65.86	123.67	RW2
Gabon	LIBREVILLE,PORT-GENTIL	2002	88.98	63.48	121.36	RW2
Gabon	LIBREVILLE,PORT-GENTIL	2003	85.67	60.39	117.33	RW2
Gabon	LIBREVILLE,PORT-GENTIL	2004	81.89	57.57	112.84	RW2
Gabon	LIBREVILLE,PORT-GENTIL	2005	77.33	54.72	105.27	RW2
Gabon	LIBREVILLE,PORT-GENTIL	2006	73.14	52.19	98.74	RW2
Gabon	LIBREVILLE,PORT-GENTIL	2007	69.11	49.85	92.89	RW2
Gabon	LIBREVILLE,PORT-GENTIL	2008	65.26	47.26	88.43	RW2
Gabon	LIBREVILLE,PORT-GENTIL	2009	61.83	44.56	84.22	RW2
Gabon	LIBREVILLE,PORT-GENTIL	2010	58.72	42.25	80.53	RW2
Gabon	LIBREVILLE,PORT-GENTIL	2011	55.83	40.85	75.87	RW2
Gabon	LIBREVILLE,PORT-GENTIL	2012	53.10	38.86	72.01	RW2
Gabon	LIBREVILLE,PORT-GENTIL	2013	50.50	35.42	71.54	RW2
Gabon	LIBREVILLE,PORT-GENTIL	2014	47.99	30.41	75.41	RW2
Gabon	LIBREVILLE,PORT-GENTIL	2015	45.66	24.30	85.07	RW2
Gabon	LIBREVILLE,PORT-GENTIL	2016	43.35	19.08	97.31	RW2
Gabon	LIBREVILLE,PORT-GENTIL	2017	41.10	14.63	113.42	RW2
Gabon	LIBREVILLE,PORT-GENTIL	2018	39.06	10.95	135.58	RW2
Gabon	LIBREVILLE,PORT-GENTIL	2019	37.11	7.90	163.68	RW2
Gabon	NORTH	1980	148.95	97.96	220.77	RW2
Gabon	NORTH	1981	142.75	102.63	195.27	RW2
Gabon	NORTH	1982	136.85	102.45	179.78	RW2
Gabon	NORTH	1983	130.91	99.07	171.46	RW2
Gabon	NORTH	1984	125.44	95.02	163.86	RW2

Continued on next page

Country	Region	Year	Median	Lower	Upper	Method
Gabon	NORTH	1985	120.19	92.16	154.54	RW2
Gabon	NORTH	1986	115.01	90.08	146.10	RW2
Gabon	NORTH	1987	110.33	87.53	138.31	RW2
Gabon	NORTH	1988	106.01	84.11	133.08	RW2
Gabon	NORTH	1989	102.23	80.76	129.35	RW2
Gabon	NORTH	1990	98.97	78.18	124.15	RW2
Gabon	NORTH	1991	97.25	77.75	120.08	RW2
Gabon	NORTH	1992	96.51	77.77	118.71	RW2
Gabon	NORTH	1993	97.06	77.97	120.18	RW2
Gabon	NORTH	1994	98.78	78.15	123.65	RW2
Gabon	NORTH	1995	101.54	80.38	128.20	RW2
Gabon	NORTH	1996	104.51	83.53	130.27	RW2
Gabon	NORTH	1997	107.64	86.39	134.31	RW2
Gabon	NORTH	1998	110.69	87.97	139.13	RW2
Gabon	NORTH	1999	112.97	88.15	144.44	RW2
Gabon	NORTH	2000	114.61	88.04	150.19	RW2
Gabon	NORTH	2001	115.04	87.63	151.68	RW2
Gabon	NORTH	2002	114.07	86.74	150.81	RW2
Gabon	NORTH	2003	111.81	84.80	147.92	RW2
Gabon	NORTH	2004	108.51	82.22	142.96	RW2
Gabon	NORTH	2005	103.96	79.33	135.21	RW2
Gabon	NORTH	2006	99.31	76.95	127.77	RW2
Gabon	NORTH	2007	94.69	73.73	121.15	RW2
Gabon	NORTH	2008	90.12	69.58	115.81	RW2
Gabon	NORTH	2009	85.58	65.45	110.69	RW2
Gabon	NORTH	2010	81.48	62.21	105.76	RW2
Gabon	NORTH	2011	77.42	60.30	98.94	RW2
Gabon	NORTH	2012	73.55	57.87	93.07	RW2
Gabon	NORTH	2013	69.88	53.00	91.56	RW2
Gabon	NORTH	2014	66.28	45.14	96.07	RW2
Gabon	NORTH	2015	62.98	36.03	108.28	RW2
Gabon	NORTH	2016	59.67	28.09	122.86	RW2
Gabon	NORTH	2017	56.71	21.46	143.03	RW2
Gabon	NORTH	2018	53.61	16.02	167.85	RW2
Gabon	NORTH	2019	51.00	11.77	200.10	RW2
Gabon	SOUTH	1980	137.58	91.13	201.83	RW2
Gabon	SOUTH	1981	131.12	95.37	178.43	RW2
Gabon	SOUTH	1982	124.93	93.97	164.06	RW2
Gabon	SOUTH	1983	118.93	89.58	156.12	RW2
Gabon	SOUTH	1984	113.26	85.17	149.07	RW2
Gabon	SOUTH	1985	108.00	82.32	140.74	RW2
Gabon	SOUTH	1986	103.04	79.59	132.23	RW2
Gabon	SOUTH	1987	98.42	76.94	125.00	RW2
Gabon	SOUTH	1988	94.24	73.85	119.84	RW2
Gabon	SOUTH	1989	90.80	71.16	115.77	RW2
Gabon	SOUTH	1990	87.59	69.00	110.44	RW2
Gabon	SOUTH	1991	85.86	68.42	106.47	RW2
Gabon	SOUTH	1992	85.03	68.41	104.85	RW2
Gabon	SOUTH	1993	85.13	68.17	105.68	RW2
Gabon	SOUTH	1994	86.21	68.13	108.32	RW2
Gabon	SOUTH	1995	88.29	69.86	111.59	RW2
Gabon	SOUTH	1996	90.29	71.82	113.44	RW2
Gabon	SOUTH	1997	92.54	74.04	116.00	RW2
Gabon	SOUTH	1998	94.49	74.87	119.69	RW2
Gabon	SOUTH	1999	95.96	74.40	123.60	RW2
Gabon	SOUTH	2000	96.69	73.65	127.71	RW2
Gabon	SOUTH	2001	96.25	72.89	128.17	RW2
Gabon	SOUTH	2002	94.71	71.26	126.42	RW2
Gabon	SOUTH	2003	91.91	68.85	123.72	RW2
Gabon	SOUTH	2004	88.32	65.94	119.47	RW2
Gabon	SOUTH	2005	83.77	62.43	112.19	RW2
Gabon	SOUTH	2006	79.09	59.28	105.20	RW2
Gabon	SOUTH	2007	74.33	55.70	98.80	RW2

Continued on next page

Country	Region	Year	Median	Lower	Upper	Method
Gabon	SOUTH	2008	69.69	51.62	93.26	RW2
Gabon	SOUTH	2009	65.35	47.90	88.14	RW2
Gabon	SOUTH	2010	61.25	44.81	82.92	RW2
Gabon	SOUTH	2011	57.30	42.33	76.96	RW2
Gabon	SOUTH	2012	53.66	39.62	71.94	RW2
Gabon	SOUTH	2013	50.21	35.79	69.84	RW2
Gabon	SOUTH	2014	46.91	30.19	71.84	RW2
Gabon	SOUTH	2015	43.84	23.75	79.63	RW2
Gabon	SOUTH	2016	40.91	18.21	89.02	RW2
Gabon	SOUTH	2017	38.26	13.74	102.40	RW2
Gabon	SOUTH	2018	35.58	10.03	119.35	RW2
Gabon	SOUTH	2019	33.33	7.16	144.02	RW2
Gabon	WEST	1980	159.75	107.71	235.30	RW2
Gabon	WEST	1981	150.79	110.83	206.10	RW2
Gabon	WEST	1982	142.44	108.46	187.05	RW2
Gabon	WEST	1983	134.25	102.56	174.93	RW2
Gabon	WEST	1984	126.58	96.46	165.45	RW2
Gabon	WEST	1985	119.43	92.39	153.26	RW2
Gabon	WEST	1986	112.65	88.86	142.14	RW2
Gabon	WEST	1987	106.40	84.62	133.09	RW2
Gabon	WEST	1988	100.84	80.10	126.40	RW2
Gabon	WEST	1989	96.04	75.61	121.33	RW2
Gabon	WEST	1990	91.83	71.96	115.20	RW2
Gabon	WEST	1991	89.08	70.39	111.08	RW2
Gabon	WEST	1992	87.41	69.53	108.83	RW2
Gabon	WEST	1993	87.00	68.77	108.87	RW2
Gabon	WEST	1994	87.45	67.91	110.26	RW2
Gabon	WEST	1995	89.21	69.62	113.11	RW2
Gabon	WEST	1996	91.13	71.74	114.77	RW2
Gabon	WEST	1997	93.13	73.62	116.52	RW2
Gabon	WEST	1998	95.27	74.33	120.16	RW2
Gabon	WEST	1999	96.78	74.00	124.32	RW2
Gabon	WEST	2000	97.89	73.58	128.97	RW2
Gabon	WEST	2001	97.95	72.95	130.64	RW2
Gabon	WEST	2002	97.30	72.07	130.14	RW2
Gabon	WEST	2003	95.39	70.31	127.92	RW2
Gabon	WEST	2004	92.93	68.54	124.87	RW2
Gabon	WEST	2005	89.50	66.55	118.70	RW2
Gabon	WEST	2006	86.04	64.68	113.04	RW2
Gabon	WEST	2007	82.59	62.40	107.91	RW2
Gabon	WEST	2008	79.45	59.74	104.29	RW2
Gabon	WEST	2009	76.36	56.88	101.37	RW2
Gabon	WEST	2010	73.62	55.11	97.87	RW2
Gabon	WEST	2011	70.78	53.71	92.89	RW2
Gabon	WEST	2012	68.20	52.29	88.74	RW2
Gabon	WEST	2013	65.71	48.73	88.63	RW2
Gabon	WEST	2014	63.31	42.56	94.02	RW2
Gabon	WEST	2015	60.92	34.68	107.01	RW2
Gabon	WEST	2016	58.66	27.49	122.27	RW2
Gabon	WEST	2017	56.39	21.35	143.71	RW2
Gabon	WEST	2018	54.26	16.30	172.38	RW2
Gabon	WEST	2019	52.27	11.85	206.24	RW2
Gambia	ALL	1980	142.42	132.90	152.19	IHME
Gambia	ALL	1980	249.93	119.90	449.68	RW2
Gambia	ALL	1980	238.50	208.60	279.20	UN
Gambia	ALL	1981	142.11	132.03	152.49	IHME
Gambia	ALL	1981	240.58	131.55	398.47	RW2
Gambia	ALL	1981	231.90	203.20	271.50	UN
Gambia	ALL	1982	135.26	126.66	143.69	IHME
Gambia	ALL	1982	231.87	139.45	359.91	RW2
Gambia	ALL	1982	225.30	197.70	263.00	UN
Gambia	ALL	1983	131.71	123.76	139.78	IHME
Gambia	ALL	1983	222.45	140.85	333.46	RW2

Continued on next page

Country	Region	Year	Median	Lower	Upper	Method
Gambia	ALL	1983	218.30	192.00	254.50	UN
Gambia	ALL	1984	128.79	121.31	136.75	IHME
Gambia	ALL	1984	214.46	137.70	316.83	RW2
Gambia	ALL	1984	211.20	186.40	245.10	UN
Gambia	ALL	1985	126.10	119.08	133.51	IHME
Gambia	ALL	1985	205.45	134.33	301.06	RW2
Gambia	ALL	1985	204.00	180.60	235.40	UN
Gambia	ALL	1986	123.16	116.45	130.24	IHME
Gambia	ALL	1986	197.57	129.95	288.06	RW2
Gambia	ALL	1986	196.80	174.90	225.30	UN
Gambia	ALL	1987	120.38	113.97	127.42	IHME
Gambia	ALL	1987	190.30	126.41	276.27	RW2
Gambia	ALL	1987	189.90	168.90	216.20	UN
Gambia	ALL	1988	117.64	111.48	124.38	IHME
Gambia	ALL	1988	183.05	121.84	264.92	RW2
Gambia	ALL	1988	183.10	163.00	207.00	UN
Gambia	ALL	1989	115.16	109.29	121.60	IHME
Gambia	ALL	1989	176.29	118.38	253.90	RW2
Gambia	ALL	1989	176.60	157.40	198.50	UN
Gambia	ALL	1990	112.71	107.01	118.98	IHME
Gambia	ALL	1990	170.09	115.81	243.11	RW2
Gambia	ALL	1990	170.20	152.10	190.50	UN
Gambia	ALL	1991	110.54	105.12	116.33	IHME
Gambia	ALL	1991	163.98	112.86	231.49	RW2
Gambia	ALL	1991	164.20	146.90	182.90	UN
Gambia	ALL	1992	108.50	103.17	113.93	IHME
Gambia	ALL	1992	158.10	109.68	222.00	RW2
Gambia	ALL	1992	158.50	142.10	176.30	UN
Gambia	ALL	1993	105.84	100.76	111.04	IHME
Gambia	ALL	1993	152.52	106.59	213.45	RW2
Gambia	ALL	1993	152.90	137.20	170.00	UN
Gambia	ALL	1994	103.33	98.20	108.09	IHME
Gambia	ALL	1994	147.00	103.21	206.03	RW2
Gambia	ALL	1994	147.50	132.30	164.10	UN
Gambia	ALL	1995	100.67	95.64	105.20	IHME
Gambia	ALL	1995	141.84	100.97	195.18	RW2
Gambia	ALL	1995	142.40	127.50	158.60	UN
Gambia	ALL	1996	97.70	92.87	102.22	IHME
Gambia	ALL	1996	136.83	98.49	187.32	RW2
Gambia	ALL	1996	137.40	122.80	153.40	UN
Gambia	ALL	1997	94.66	90.02	98.99	IHME
Gambia	ALL	1997	132.14	95.86	179.25	RW2
Gambia	ALL	1997	132.50	118.20	148.60	UN
Gambia	ALL	1998	91.55	87.16	95.68	IHME
Gambia	ALL	1998	127.84	92.68	174.20	RW2
Gambia	ALL	1998	127.90	113.70	144.00	UN
Gambia	ALL	1999	88.56	84.20	92.68	IHME
Gambia	ALL	1999	123.61	89.35	167.57	RW2
Gambia	ALL	1999	123.20	109.20	139.50	UN
Gambia	ALL	2000	85.34	81.09	89.56	IHME
Gambia	ALL	2000	119.62	87.77	161.25	RW2
Gambia	ALL	2000	118.80	104.90	135.20	UN
Gambia	ALL	2001	82.37	78.33	86.46	IHME
Gambia	ALL	2001	115.51	85.37	154.78	RW2
Gambia	ALL	2001	114.50	100.60	131.10	UN
Gambia	ALL	2002	79.62	75.54	83.75	IHME
Gambia	ALL	2002	111.31	82.83	148.56	RW2
Gambia	ALL	2002	110.20	96.20	126.80	UN
Gambia	ALL	2003	76.80	72.75	80.88	IHME
Gambia	ALL	2003	107.21	79.63	143.39	RW2
Gambia	ALL	2003	106.00	91.80	123.30	UN
Gambia	ALL	2004	73.82	69.90	77.76	IHME
Gambia	ALL	2004	102.83	75.37	139.06	RW2

Continued on next page

Country	Region	Year	Median	Lower	Upper	Method
Gambia	ALL	2004	101.90	87.40	119.90	UN
Gambia	ALL	2005	70.92	66.79	74.87	IHME
Gambia	ALL	2005	98.66	71.91	133.54	RW2
Gambia	ALL	2005	97.90	82.70	116.70	UN
Gambia	ALL	2006	68.10	63.90	72.03	IHME
Gambia	ALL	2006	94.64	69.36	127.35	RW2
Gambia	ALL	2006	94.20	78.20	113.80	UN
Gambia	ALL	2007	65.33	61.18	69.35	IHME
Gambia	ALL	2007	90.92	66.85	122.17	RW2
Gambia	ALL	2007	90.60	74.00	111.00	UN
Gambia	ALL	2008	62.47	58.35	66.50	IHME
Gambia	ALL	2008	87.61	63.72	119.08	RW2
Gambia	ALL	2008	87.40	70.00	108.60	UN
Gambia	ALL	2009	59.70	55.60	63.67	IHME
Gambia	ALL	2009	84.26	60.15	116.99	RW2
Gambia	ALL	2009	84.30	65.90	106.80	UN
Gambia	ALL	2010	56.96	52.79	61.08	IHME
Gambia	ALL	2010	81.29	56.98	115.63	RW2
Gambia	ALL	2010	81.40	62.00	105.60	UN
Gambia	ALL	2011	54.44	50.19	58.59	IHME
Gambia	ALL	2011	78.50	54.11	112.77	RW2
Gambia	ALL	2011	78.60	58.20	104.80	UN
Gambia	ALL	2012	51.97	47.65	56.38	IHME
Gambia	ALL	2012	75.75	50.45	112.32	RW2
Gambia	ALL	2012	76.10	54.60	104.30	UN
Gambia	ALL	2013	49.68	45.29	54.27	IHME
Gambia	ALL	2013	73.24	45.20	116.40	RW2
Gambia	ALL	2013	73.60	51.00	103.90	UN
Gambia	ALL	2014	47.55	43.18	52.41	IHME
Gambia	ALL	2014	70.65	38.19	127.26	RW2
Gambia	ALL	2014	71.10	47.90	104.20	UN
Gambia	ALL	2015	45.39	40.89	50.31	IHME
Gambia	ALL	2015	68.00	29.99	148.03	RW2
Gambia	ALL	2015	68.90	44.70	104.10	UN
Gambia	ALL	2016	65.85	23.35	175.00	RW2
Gambia	ALL	2017	63.30	17.37	210.03	RW2
Gambia	ALL	2018	61.03	12.79	256.94	RW2
Gambia	ALL	2019	58.72	8.73	308.51	RW2
Gambia	ALL	15-19	63.29	17.74	205.75	RW2
Gambia	BANJUL	1980	137.94	43.66	369.53	RW2
Gambia	BANJUL	1981	134.03	47.22	334.08	RW2
Gambia	BANJUL	1982	129.30	49.87	300.98	RW2
Gambia	BANJUL	1983	124.96	51.46	277.43	RW2
Gambia	BANJUL	1984	120.68	51.81	258.25	RW2
Gambia	BANJUL	1985	116.78	52.79	240.66	RW2
Gambia	BANJUL	1986	113.22	53.08	224.87	RW2
Gambia	BANJUL	1987	109.84	53.59	212.35	RW2
Gambia	BANJUL	1988	106.82	53.85	200.86	RW2
Gambia	BANJUL	1989	104.06	54.50	189.52	RW2
Gambia	BANJUL	1990	101.57	55.05	180.22	RW2
Gambia	BANJUL	1991	99.42	55.73	171.23	RW2
Gambia	BANJUL	1992	97.08	55.78	163.31	RW2
Gambia	BANJUL	1993	94.70	56.01	155.84	RW2
Gambia	BANJUL	1994	92.92	55.97	149.41	RW2
Gambia	BANJUL	1995	90.83	56.19	142.85	RW2
Gambia	BANJUL	1996	89.29	56.46	137.27	RW2
Gambia	BANJUL	1997	87.86	56.68	133.09	RW2
Gambia	BANJUL	1998	86.68	56.90	129.22	RW2
Gambia	BANJUL	1999	85.68	56.16	126.40	RW2
Gambia	BANJUL	2000	84.79	56.45	124.16	RW2
Gambia	BANJUL	2001	83.84	56.47	121.42	RW2
Gambia	BANJUL	2002	82.69	56.27	119.11	RW2
Gambia	BANJUL	2003	81.28	55.53	117.41	RW2

Continued on next page

Country	Region	Year	Median	Lower	Upper	Method
Gambia	BANJUL	2004	79.86	54.24	115.70	RW2
Gambia	BANJUL	2005	78.15	53.10	113.44	RW2
Gambia	BANJUL	2006	76.61	52.15	111.21	RW2
Gambia	BANJUL	2007	74.99	50.41	109.69	RW2
Gambia	BANJUL	2008	73.57	48.48	109.83	RW2
Gambia	BANJUL	2009	72.01	46.30	110.33	RW2
Gambia	BANJUL	2010	70.77	44.26	111.78	RW2
Gambia	BANJUL	2011	69.40	42.11	113.05	RW2
Gambia	BANJUL	2012	68.15	39.66	115.67	RW2
Gambia	BANJUL	2013	67.00	36.31	122.43	RW2
Gambia	BANJUL	2014	65.89	31.89	132.71	RW2
Gambia	BANJUL	2015	64.70	26.54	151.21	RW2
Gambia	BANJUL	2016	63.38	21.56	174.66	RW2
Gambia	BANJUL	2017	62.39	17.07	206.99	RW2
Gambia	BANJUL	2018	61.02	13.04	248.67	RW2
Gambia	BANJUL	2019	59.99	9.76	302.40	RW2
Gambia	CENTRAL RIVER	1980	242.60	98.11	484.18	RW2
Gambia	CENTRAL RIVER	1981	232.90	105.10	443.39	RW2
Gambia	CENTRAL RIVER	1982	223.61	107.19	408.12	RW2
Gambia	CENTRAL RIVER	1983	214.32	106.97	382.44	RW2
Gambia	CENTRAL RIVER	1984	205.55	106.20	359.54	RW2
Gambia	CENTRAL RIVER	1985	197.08	104.53	340.67	RW2
Gambia	CENTRAL RIVER	1986	189.44	102.42	322.45	RW2
Gambia	CENTRAL RIVER	1987	182.17	101.11	305.01	RW2
Gambia	CENTRAL RIVER	1988	175.30	99.83	289.31	RW2
Gambia	CENTRAL RIVER	1989	169.40	99.28	273.97	RW2
Gambia	CENTRAL RIVER	1990	163.22	98.70	258.95	RW2
Gambia	CENTRAL RIVER	1991	158.00	97.47	244.10	RW2
Gambia	CENTRAL RIVER	1992	152.57	96.65	231.57	RW2
Gambia	CENTRAL RIVER	1993	147.00	95.35	220.10	RW2
Gambia	CENTRAL RIVER	1994	141.94	93.75	209.56	RW2
Gambia	CENTRAL RIVER	1995	136.92	92.50	197.44	RW2
Gambia	CENTRAL RIVER	1996	131.94	90.78	187.17	RW2
Gambia	CENTRAL RIVER	1997	127.67	89.72	178.47	RW2
Gambia	CENTRAL RIVER	1998	123.46	87.73	171.17	RW2
Gambia	CENTRAL RIVER	1999	119.50	85.19	164.21	RW2
Gambia	CENTRAL RIVER	2000	115.64	83.63	157.91	RW2
Gambia	CENTRAL RIVER	2001	111.51	81.76	150.82	RW2
Gambia	CENTRAL RIVER	2002	107.26	78.89	144.15	RW2
Gambia	CENTRAL RIVER	2003	102.65	75.11	139.27	RW2
Gambia	CENTRAL RIVER	2004	98.08	70.93	134.85	RW2
Gambia	CENTRAL RIVER	2005	93.36	66.60	129.32	RW2
Gambia	CENTRAL RIVER	2006	88.63	62.76	123.57	RW2
Gambia	CENTRAL RIVER	2007	83.98	58.70	118.71	RW2
Gambia	CENTRAL RIVER	2008	79.51	54.17	114.94	RW2
Gambia	CENTRAL RIVER	2009	75.37	49.96	111.89	RW2
Gambia	CENTRAL RIVER	2010	71.38	45.93	109.09	RW2
Gambia	CENTRAL RIVER	2011	67.53	42.21	106.22	RW2
Gambia	CENTRAL RIVER	2012	64.01	38.31	104.58	RW2
Gambia	CENTRAL RIVER	2013	60.58	34.15	105.63	RW2
Gambia	CENTRAL RIVER	2014	57.24	28.84	110.48	RW2
Gambia	CENTRAL RIVER	2015	54.14	23.01	122.57	RW2
Gambia	CENTRAL RIVER	2016	51.12	17.83	137.09	RW2
Gambia	CENTRAL RIVER	2017	48.42	13.60	157.77	RW2
Gambia	CENTRAL RIVER	2018	45.56	10.00	183.75	RW2
Gambia	CENTRAL RIVER	2019	43.24	7.18	221.23	RW2
Gambia	LOWER RIVER	1980	432.20	216.24	680.43	RW2
Gambia	LOWER RIVER	1981	410.87	221.20	632.01	RW2
Gambia	LOWER RIVER	1982	390.31	220.67	588.98	RW2
Gambia	LOWER RIVER	1983	368.91	215.65	555.93	RW2
Gambia	LOWER RIVER	1984	349.33	207.44	523.84	RW2
Gambia	LOWER RIVER	1985	329.86	196.48	494.20	RW2
Gambia	LOWER RIVER	1986	311.01	188.28	468.67	RW2

Continued on next page

Country	Region	Year	Median	Lower	Upper	Method
Gambia	LOWER RIVER	1987	294.17	180.39	440.64	RW2
Gambia	LOWER RIVER	1988	277.95	172.32	415.28	RW2
Gambia	LOWER RIVER	1989	262.29	164.83	391.43	RW2
Gambia	LOWER RIVER	1990	248.07	158.37	367.57	RW2
Gambia	LOWER RIVER	1991	234.85	151.99	342.95	RW2
Gambia	LOWER RIVER	1992	221.24	145.46	321.78	RW2
Gambia	LOWER RIVER	1993	208.70	139.27	302.56	RW2
Gambia	LOWER RIVER	1994	197.07	131.95	284.56	RW2
Gambia	LOWER RIVER	1995	185.20	125.25	265.72	RW2
Gambia	LOWER RIVER	1996	174.51	119.62	246.99	RW2
Gambia	LOWER RIVER	1997	164.56	113.83	233.04	RW2
Gambia	LOWER RIVER	1998	155.56	107.83	219.53	RW2
Gambia	LOWER RIVER	1999	146.64	101.61	206.84	RW2
Gambia	LOWER RIVER	2000	138.47	96.81	195.59	RW2
Gambia	LOWER RIVER	2001	130.38	91.38	183.57	RW2
Gambia	LOWER RIVER	2002	122.24	85.79	172.36	RW2
Gambia	LOWER RIVER	2003	114.17	79.43	162.15	RW2
Gambia	LOWER RIVER	2004	106.34	72.91	152.33	RW2
Gambia	LOWER RIVER	2005	98.49	66.89	142.72	RW2
Gambia	LOWER RIVER	2006	91.05	61.63	133.34	RW2
Gambia	LOWER RIVER	2007	84.27	56.14	125.22	RW2
Gambia	LOWER RIVER	2008	77.89	50.38	118.70	RW2
Gambia	LOWER RIVER	2009	71.74	44.89	112.78	RW2
Gambia	LOWER RIVER	2010	66.27	39.80	108.32	RW2
Gambia	LOWER RIVER	2011	61.09	35.48	103.53	RW2
Gambia	LOWER RIVER	2012	56.30	31.10	99.92	RW2
Gambia	LOWER RIVER	2013	51.87	26.63	98.65	RW2
Gambia	LOWER RIVER	2014	47.68	21.76	100.55	RW2
Gambia	LOWER RIVER	2015	44.01	17.10	108.22	RW2
Gambia	LOWER RIVER	2016	40.40	12.97	117.98	RW2
Gambia	LOWER RIVER	2017	37.28	9.64	132.77	RW2
Gambia	LOWER RIVER	2018	34.13	6.97	151.24	RW2
Gambia	LOWER RIVER	2019	31.52	4.96	176.13	RW2
Gambia	NORTH BANK	1980	307.61	142.53	546.40	RW2
Gambia	NORTH BANK	1981	293.49	149.91	497.20	RW2
Gambia	NORTH BANK	1982	280.24	153.24	455.33	RW2
Gambia	NORTH BANK	1983	266.28	152.31	422.74	RW2
Gambia	NORTH BANK	1984	253.44	149.20	397.90	RW2
Gambia	NORTH BANK	1985	241.10	145.00	372.13	RW2
Gambia	NORTH BANK	1986	229.46	141.27	350.28	RW2
Gambia	NORTH BANK	1987	218.59	136.48	330.59	RW2
Gambia	NORTH BANK	1988	208.68	132.59	312.00	RW2
Gambia	NORTH BANK	1989	199.26	128.43	294.93	RW2
Gambia	NORTH BANK	1990	190.65	124.87	278.59	RW2
Gambia	NORTH BANK	1991	182.21	121.25	264.18	RW2
Gambia	NORTH BANK	1992	173.84	117.88	250.40	RW2
Gambia	NORTH BANK	1993	166.22	114.08	237.27	RW2
Gambia	NORTH BANK	1994	158.40	108.96	224.01	RW2
Gambia	NORTH BANK	1995	151.28	105.76	211.19	RW2
Gambia	NORTH BANK	1996	144.63	102.62	200.21	RW2
Gambia	NORTH BANK	1997	138.10	99.24	189.25	RW2
Gambia	NORTH BANK	1998	132.41	95.23	180.63	RW2
Gambia	NORTH BANK	1999	126.52	91.02	172.24	RW2
Gambia	NORTH BANK	2000	121.09	88.18	164.30	RW2
Gambia	NORTH BANK	2001	115.34	84.45	156.42	RW2
Gambia	NORTH BANK	2002	109.80	80.23	148.82	RW2
Gambia	NORTH BANK	2003	103.75	74.80	142.06	RW2
Gambia	NORTH BANK	2004	97.95	69.41	136.54	RW2
Gambia	NORTH BANK	2005	91.96	64.18	130.08	RW2
Gambia	NORTH BANK	2006	86.11	59.18	123.56	RW2
Gambia	NORTH BANK	2007	80.50	54.11	117.68	RW2
Gambia	NORTH BANK	2008	75.46	49.05	113.65	RW2
Gambia	NORTH BANK	2009	70.48	43.94	110.80	RW2

Continued on next page

Country	Region	Year	Median	Lower	Upper	Method
Gambia	NORTH BANK	2010	65.93	39.60	107.90	RW2
Gambia	NORTH BANK	2011	61.38	35.18	104.75	RW2
Gambia	NORTH BANK	2012	57.33	31.15	102.54	RW2
Gambia	NORTH BANK	2013	53.55	26.87	103.16	RW2
Gambia	NORTH BANK	2014	50.06	22.33	106.98	RW2
Gambia	NORTH BANK	2015	46.65	17.72	116.72	RW2
Gambia	NORTH BANK	2016	43.52	13.54	128.16	RW2
Gambia	NORTH BANK	2017	40.50	10.16	145.54	RW2
Gambia	NORTH BANK	2018	37.75	7.48	169.54	RW2
Gambia	NORTH BANK	2019	35.23	5.20	197.53	RW2
Gambia	UPPER RIVER	1980	359.35	180.65	587.20	RW2
Gambia	UPPER RIVER	1981	346.95	190.92	544.28	RW2
Gambia	UPPER RIVER	1982	333.84	194.56	510.29	RW2
Gambia	UPPER RIVER	1983	323.20	192.96	485.32	RW2
Gambia	UPPER RIVER	1984	310.92	187.91	465.20	RW2
Gambia	UPPER RIVER	1985	300.03	182.84	447.49	RW2
Gambia	UPPER RIVER	1986	289.13	178.12	430.08	RW2
Gambia	UPPER RIVER	1987	279.23	174.11	414.65	RW2
Gambia	UPPER RIVER	1988	269.77	171.30	395.93	RW2
Gambia	UPPER RIVER	1989	261.25	168.91	381.40	RW2
Gambia	UPPER RIVER	1990	253.32	167.35	364.54	RW2
Gambia	UPPER RIVER	1991	244.91	165.47	347.47	RW2
Gambia	UPPER RIVER	1992	236.68	163.04	330.69	RW2
Gambia	UPPER RIVER	1993	228.92	160.41	316.06	RW2
Gambia	UPPER RIVER	1994	220.90	156.81	302.54	RW2
Gambia	UPPER RIVER	1995	213.39	154.09	287.10	RW2
Gambia	UPPER RIVER	1996	206.01	151.10	274.49	RW2
Gambia	UPPER RIVER	1997	199.05	147.12	263.69	RW2
Gambia	UPPER RIVER	1998	192.39	142.70	254.73	RW2
Gambia	UPPER RIVER	1999	185.85	136.65	247.55	RW2
Gambia	UPPER RIVER	2000	180.13	132.77	240.70	RW2
Gambia	UPPER RIVER	2001	173.73	127.89	232.93	RW2
Gambia	UPPER RIVER	2002	167.08	122.47	225.30	RW2
Gambia	UPPER RIVER	2003	160.01	115.75	217.80	RW2
Gambia	UPPER RIVER	2004	152.64	109.27	210.49	RW2
Gambia	UPPER RIVER	2005	145.14	102.60	201.04	RW2
Gambia	UPPER RIVER	2006	137.45	96.76	192.11	RW2
Gambia	UPPER RIVER	2007	130.49	90.92	183.17	RW2
Gambia	UPPER RIVER	2008	123.38	84.67	177.03	RW2
Gambia	UPPER RIVER	2009	116.38	78.05	170.36	RW2
Gambia	UPPER RIVER	2010	109.81	72.00	164.17	RW2
Gambia	UPPER RIVER	2011	103.83	66.73	157.41	RW2
Gambia	UPPER RIVER	2012	97.90	60.77	153.21	RW2
Gambia	UPPER RIVER	2013	91.95	53.59	152.27	RW2
Gambia	UPPER RIVER	2014	86.84	45.46	159.17	RW2
Gambia	UPPER RIVER	2015	81.69	36.21	172.45	RW2
Gambia	UPPER RIVER	2016	76.95	28.00	193.92	RW2
Gambia	UPPER RIVER	2017	72.65	21.14	219.57	RW2
Gambia	UPPER RIVER	2018	68.37	15.48	252.46	RW2
Gambia	UPPER RIVER	2019	63.90	10.91	297.79	RW2
Gambia	WESTERN	1980	214.64	86.60	443.79	RW2
Gambia	WESTERN	1981	206.95	93.54	399.01	RW2
Gambia	WESTERN	1982	198.95	96.78	366.20	RW2
Gambia	WESTERN	1983	190.87	97.40	341.60	RW2
Gambia	WESTERN	1984	183.26	97.11	319.32	RW2
Gambia	WESTERN	1985	175.87	94.99	301.46	RW2
Gambia	WESTERN	1986	169.38	93.62	285.57	RW2
Gambia	WESTERN	1987	163.31	92.89	270.28	RW2
Gambia	WESTERN	1988	157.62	91.29	257.39	RW2
Gambia	WESTERN	1989	152.37	90.32	244.93	RW2
Gambia	WESTERN	1990	147.50	89.87	233.39	RW2
Gambia	WESTERN	1991	143.07	89.17	222.38	RW2
Gambia	WESTERN	1992	138.74	87.95	211.88	RW2

Continued on next page

Country	Region	Year	Median	Lower	Upper	Method
Gambia	WESTERN	1993	134.38	87.14	201.58	RW2
Gambia	WESTERN	1994	130.21	85.63	193.27	RW2
Gambia	WESTERN	1995	126.19	84.59	183.40	RW2
Gambia	WESTERN	1996	122.67	83.70	175.36	RW2
Gambia	WESTERN	1997	119.42	82.98	167.95	RW2
Gambia	WESTERN	1998	116.38	82.02	162.63	RW2
Gambia	WESTERN	1999	113.57	80.50	157.63	RW2
Gambia	WESTERN	2000	110.98	79.65	151.82	RW2
Gambia	WESTERN	2001	108.17	79.16	146.13	RW2
Gambia	WESTERN	2002	105.26	77.64	141.25	RW2
Gambia	WESTERN	2003	101.94	75.33	136.99	RW2
Gambia	WESTERN	2004	98.63	72.49	132.99	RW2
Gambia	WESTERN	2005	95.13	69.43	128.89	RW2
Gambia	WESTERN	2006	91.68	66.91	124.32	RW2
Gambia	WESTERN	2007	88.28	63.95	120.47	RW2
Gambia	WESTERN	2008	85.11	60.49	118.34	RW2
Gambia	WESTERN	2009	81.92	56.75	117.00	RW2
Gambia	WESTERN	2010	79.14	52.65	116.97	RW2
Gambia	WESTERN	2011	76.15	49.38	116.15	RW2
Gambia	WESTERN	2012	73.54	45.50	116.94	RW2
Gambia	WESTERN	2013	70.83	40.57	121.31	RW2
Gambia	WESTERN	2014	68.32	34.80	129.37	RW2
Gambia	WESTERN	2015	65.92	28.40	147.23	RW2
Gambia	WESTERN	2016	63.52	22.68	168.19	RW2
Gambia	WESTERN	2017	61.30	17.43	197.73	RW2
Gambia	WESTERN	2018	58.84	12.84	233.36	RW2
Gambia	WESTERN	2019	56.62	9.56	277.07	RW2
Ghana	ALL	1980	152.49	147.24	157.26	IHME
Ghana	ALL	1980	169.14	128.15	219.74	RW2
Ghana	ALL	1980	166.20	157.60	175.10	UN
Ghana	ALL	1981	150.80	145.97	155.41	IHME
Ghana	ALL	1981	165.99	136.55	200.47	RW2
Ghana	ALL	1981	164.60	155.80	173.70	UN
Ghana	ALL	1982	149.26	144.87	153.79	IHME
Ghana	ALL	1982	162.99	136.07	193.81	RW2
Ghana	ALL	1982	163.10	154.60	172.20	UN
Ghana	ALL	1983	147.70	143.54	152.23	IHME
Ghana	ALL	1983	159.69	130.37	193.37	RW2
Ghana	ALL	1983	161.30	152.90	170.40	UN
Ghana	ALL	1984	145.24	141.23	149.57	IHME
Ghana	ALL	1984	156.49	124.71	193.00	RW2
Ghana	ALL	1984	158.50	150.30	167.30	UN
Ghana	ALL	1985	141.92	137.83	146.09	IHME
Ghana	ALL	1985	153.04	124.77	186.54	RW2
Ghana	ALL	1985	154.60	146.60	163.20	UN
Ghana	ALL	1986	138.17	134.42	142.19	IHME
Ghana	ALL	1986	148.86	123.02	178.76	RW2
Ghana	ALL	1986	149.70	142.00	158.00	UN
Ghana	ALL	1987	134.43	130.59	138.39	IHME
Ghana	ALL	1987	144.21	120.34	172.34	RW2
Ghana	ALL	1987	144.10	136.70	152.20	UN
Ghana	ALL	1988	130.38	126.68	134.38	IHME
Ghana	ALL	1988	139.04	115.03	167.69	RW2
Ghana	ALL	1988	138.20	131.10	146.10	UN
Ghana	ALL	1989	126.41	123.06	130.40	IHME
Ghana	ALL	1989	133.76	109.49	163.63	RW2
Ghana	ALL	1989	132.60	125.50	140.00	UN
Ghana	ALL	1990	122.71	119.50	126.55	IHME
Ghana	ALL	1990	128.03	104.00	155.94	RW2
Ghana	ALL	1990	127.40	120.50	134.60	UN
Ghana	ALL	1991	119.16	115.92	122.90	IHME
Ghana	ALL	1991	123.54	101.56	148.77	RW2
Ghana	ALL	1991	123.10	116.20	130.20	UN

Continued on next page

Country	Region	Year	Median	Lower	Upper	Method
Ghana	ALL	1992	116.09	112.75	119.81	IHME
Ghana	ALL	1992	119.83	99.00	143.91	RW2
Ghana	ALL	1992	119.70	113.00	126.60	UN
Ghana	ALL	1993	113.54	110.21	117.01	IHME
Ghana	ALL	1993	116.96	96.04	141.27	RW2
Ghana	ALL	1993	117.10	110.50	124.00	UN
Ghana	ALL	1994	111.64	108.30	115.11	IHME
Ghana	ALL	1994	114.66	92.83	140.45	RW2
Ghana	ALL	1994	115.10	108.70	122.10	UN
Ghana	ALL	1995	109.31	105.98	112.80	IHME
Ghana	ALL	1995	113.48	92.50	139.66	RW2
Ghana	ALL	1995	113.40	107.00	120.30	UN
Ghana	ALL	1996	107.36	104.09	110.87	IHME
Ghana	ALL	1996	111.51	91.60	136.20	RW2
Ghana	ALL	1996	111.60	105.20	118.50	UN
Ghana	ALL	1997	105.28	102.09	108.71	IHME
Ghana	ALL	1997	109.26	90.12	132.27	RW2
Ghana	ALL	1997	109.50	103.20	116.40	UN
Ghana	ALL	1998	103.09	99.82	106.42	IHME
Ghana	ALL	1998	106.66	87.41	130.40	RW2
Ghana	ALL	1998	106.90	100.70	113.80	UN
Ghana	ALL	1999	100.75	97.58	104.06	IHME
Ghana	ALL	1999	103.64	83.94	127.53	RW2
Ghana	ALL	1999	104.00	97.80	110.60	UN
Ghana	ALL	2000	98.36	95.08	101.70	IHME
Ghana	ALL	2000	99.96	80.51	122.53	RW2
Ghana	ALL	2000	100.70	94.60	107.30	UN
Ghana	ALL	2001	96.04	92.83	99.34	IHME
Ghana	ALL	2001	96.87	78.70	118.04	RW2
Ghana	ALL	2001	97.30	91.30	104.00	UN
Ghana	ALL	2002	93.49	90.37	96.80	IHME
Ghana	ALL	2002	93.98	76.95	114.13	RW2
Ghana	ALL	2002	94.20	88.00	100.80	UN
Ghana	ALL	2003	90.84	87.59	94.26	IHME
Ghana	ALL	2003	91.48	74.60	111.53	RW2
Ghana	ALL	2003	91.30	85.10	98.00	UN
Ghana	ALL	2004	88.10	84.82	91.48	IHME
Ghana	ALL	2004	89.08	71.46	110.03	RW2
Ghana	ALL	2004	88.70	82.50	95.50	UN
Ghana	ALL	2005	85.37	82.05	88.79	IHME
Ghana	ALL	2005	87.30	70.02	109.02	RW2
Ghana	ALL	2005	86.50	80.10	93.50	UN
Ghana	ALL	2006	82.57	79.31	85.90	IHME
Ghana	ALL	2006	85.07	68.76	105.20	RW2
Ghana	ALL	2006	84.40	77.80	91.80	UN
Ghana	ALL	2007	79.70	76.38	83.19	IHME
Ghana	ALL	2007	82.75	67.20	101.73	RW2
Ghana	ALL	2007	82.20	75.40	90.00	UN
Ghana	ALL	2008	76.79	73.15	80.36	IHME
Ghana	ALL	2008	80.32	64.66	99.61	RW2
Ghana	ALL	2008	79.90	72.50	88.10	UN
Ghana	ALL	2009	74.06	70.16	77.83	IHME
Ghana	ALL	2009	77.50	61.38	97.97	RW2
Ghana	ALL	2009	77.40	69.50	86.10	UN
Ghana	ALL	2010	70.99	66.90	75.16	IHME
Ghana	ALL	2010	74.46	57.88	95.51	RW2
Ghana	ALL	2010	74.70	66.00	84.10	UN
Ghana	ALL	2011	67.40	63.10	72.03	IHME
Ghana	ALL	2011	71.59	55.83	91.11	RW2
Ghana	ALL	2011	71.90	62.30	82.50	UN
Ghana	ALL	2012	63.86	59.28	69.14	IHME
Ghana	ALL	2012	68.68	53.58	87.41	RW2
Ghana	ALL	2012	69.20	58.50	81.10	UN

Continued on next page

Country	Region	Year	Median	Lower	Upper	Method
Ghana	ALL	2013	60.95	55.92	66.83	IHME
Ghana	ALL	2013	65.91	49.51	86.98	RW2
Ghana	ALL	2013	66.50	54.80	80.00	UN
Ghana	ALL	2014	58.32	52.95	65.02	IHME
Ghana	ALL	2014	63.18	42.92	91.64	RW2
Ghana	ALL	2014	64.00	51.30	78.90	UN
Ghana	ALL	2015	55.52	49.76	62.70	IHME
Ghana	ALL	2015	60.52	34.62	103.23	RW2
Ghana	ALL	2015	61.60	48.10	78.20	UN
Ghana	ALL	2016	58.18	27.75	117.44	RW2
Ghana	ALL	2017	55.64	21.44	136.00	RW2
Ghana	ALL	2018	53.32	16.38	160.95	RW2
Ghana	ALL	2019	51.02	11.80	188.89	RW2
Ghana	ALL	15-19	55.65	21.75	133.68	RW2
Ghana	ASHANTI	1980	137.24	104.05	179.10	RW2
Ghana	ASHANTI	1981	135.41	111.01	164.57	RW2
Ghana	ASHANTI	1982	133.55	111.06	159.25	RW2
Ghana	ASHANTI	1983	131.72	108.32	159.00	RW2
Ghana	ASHANTI	1984	129.75	105.36	158.39	RW2
Ghana	ASHANTI	1985	127.82	105.51	154.09	RW2
Ghana	ASHANTI	1986	125.36	105.09	148.39	RW2
Ghana	ASHANTI	1987	122.60	103.75	144.29	RW2
Ghana	ASHANTI	1988	119.57	100.73	141.50	RW2
Ghana	ASHANTI	1989	116.50	96.97	139.50	RW2
Ghana	ASHANTI	1990	113.26	93.96	135.43	RW2
Ghana	ASHANTI	1991	110.77	92.96	131.63	RW2
Ghana	ASHANTI	1992	108.76	91.75	128.07	RW2
Ghana	ASHANTI	1993	107.29	90.00	127.07	RW2
Ghana	ASHANTI	1994	106.33	88.03	127.49	RW2
Ghana	ASHANTI	1995	106.06	88.05	127.57	RW2
Ghana	ASHANTI	1996	105.47	88.28	125.58	RW2
Ghana	ASHANTI	1997	104.84	88.20	124.04	RW2
Ghana	ASHANTI	1998	103.85	86.92	123.70	RW2
Ghana	ASHANTI	1999	102.68	84.71	124.27	RW2
Ghana	ASHANTI	2000	101.11	83.05	122.19	RW2
Ghana	ASHANTI	2001	99.68	82.61	119.38	RW2
Ghana	ASHANTI	2002	98.32	81.72	117.73	RW2
Ghana	ASHANTI	2003	96.97	80.04	116.88	RW2
Ghana	ASHANTI	2004	95.77	77.87	117.05	RW2
Ghana	ASHANTI	2005	94.62	76.31	116.69	RW2
Ghana	ASHANTI	2006	93.22	75.38	114.82	RW2
Ghana	ASHANTI	2007	91.42	73.64	113.04	RW2
Ghana	ASHANTI	2008	89.44	71.26	111.75	RW2
Ghana	ASHANTI	2009	87.41	68.35	111.30	RW2
Ghana	ASHANTI	2010	84.90	64.93	109.72	RW2
Ghana	ASHANTI	2011	82.48	63.21	107.06	RW2
Ghana	ASHANTI	2012	80.04	60.70	105.05	RW2
Ghana	ASHANTI	2013	77.65	56.65	105.27	RW2
Ghana	ASHANTI	2014	75.40	50.55	110.28	RW2
Ghana	ASHANTI	2015	73.12	42.88	122.43	RW2
Ghana	ASHANTI	2016	70.86	35.18	136.39	RW2
Ghana	ASHANTI	2017	68.87	28.35	155.48	RW2
Ghana	ASHANTI	2018	66.39	22.35	180.71	RW2
Ghana	ASHANTI	2019	64.38	17.16	210.47	RW2
Ghana	BRONG AHAFO	1980	145.60	109.21	191.45	RW2
Ghana	BRONG AHAFO	1981	142.56	114.91	175.38	RW2
Ghana	BRONG AHAFO	1982	139.51	114.68	168.43	RW2
Ghana	BRONG AHAFO	1983	136.43	111.05	166.64	RW2
Ghana	BRONG AHAFO	1984	133.23	107.27	164.08	RW2
Ghana	BRONG AHAFO	1985	130.13	106.43	158.26	RW2
Ghana	BRONG AHAFO	1986	126.56	105.02	151.26	RW2
Ghana	BRONG AHAFO	1987	122.56	102.70	145.36	RW2
Ghana	BRONG AHAFO	1988	118.56	98.77	141.51	RW2

Continued on next page

Country	Region	Year	Median	Lower	Upper	Method
Ghana	BRONG AHAFO	1989	114.40	94.39	138.31	RW2
Ghana	BRONG AHAFO	1990	110.08	90.69	132.59	RW2
Ghana	BRONG AHAFO	1991	106.42	88.63	127.44	RW2
Ghana	BRONG AHAFO	1992	103.30	86.43	123.08	RW2
Ghana	BRONG AHAFO	1993	100.73	83.85	120.58	RW2
Ghana	BRONG AHAFO	1994	98.53	80.91	119.33	RW2
Ghana	BRONG AHAFO	1995	96.96	79.53	117.96	RW2
Ghana	BRONG AHAFO	1996	95.14	78.65	114.69	RW2
Ghana	BRONG AHAFO	1997	93.23	77.31	112.22	RW2
Ghana	BRONG AHAFO	1998	91.16	75.41	110.25	RW2
Ghana	BRONG AHAFO	1999	88.95	72.54	108.84	RW2
Ghana	BRONG AHAFO	2000	86.34	70.05	105.59	RW2
Ghana	BRONG AHAFO	2001	84.02	68.76	102.24	RW2
Ghana	BRONG AHAFO	2002	81.65	67.01	99.11	RW2
Ghana	BRONG AHAFO	2003	79.55	64.63	97.72	RW2
Ghana	BRONG AHAFO	2004	77.32	61.80	96.32	RW2
Ghana	BRONG AHAFO	2005	75.31	59.81	94.56	RW2
Ghana	BRONG AHAFO	2006	73.04	57.99	91.65	RW2
Ghana	BRONG AHAFO	2007	70.68	55.80	88.97	RW2
Ghana	BRONG AHAFO	2008	68.08	53.09	86.83	RW2
Ghana	BRONG AHAFO	2009	65.36	50.04	84.94	RW2
Ghana	BRONG AHAFO	2010	62.41	46.94	82.48	RW2
Ghana	BRONG AHAFO	2011	59.71	44.51	78.74	RW2
Ghana	BRONG AHAFO	2012	56.93	42.26	76.10	RW2
Ghana	BRONG AHAFO	2013	54.30	38.96	75.12	RW2
Ghana	BRONG AHAFO	2014	51.70	34.12	77.24	RW2
Ghana	BRONG AHAFO	2015	49.39	28.31	84.64	RW2
Ghana	BRONG AHAFO	2016	47.18	23.03	93.54	RW2
Ghana	BRONG AHAFO	2017	44.85	18.16	104.89	RW2
Ghana	BRONG AHAFO	2018	42.62	14.12	120.21	RW2
Ghana	BRONG AHAFO	2019	40.69	10.69	141.88	RW2
Ghana	CENTRAL	1980	196.37	150.91	252.60	RW2
Ghana	CENTRAL	1981	190.55	156.91	230.53	RW2
Ghana	CENTRAL	1982	184.96	154.79	219.57	RW2
Ghana	CENTRAL	1983	179.18	148.03	214.98	RW2
Ghana	CENTRAL	1984	173.47	141.71	210.68	RW2
Ghana	CENTRAL	1985	167.92	139.17	201.18	RW2
Ghana	CENTRAL	1986	161.83	136.46	190.97	RW2
Ghana	CENTRAL	1987	155.49	131.88	182.36	RW2
Ghana	CENTRAL	1988	149.07	125.81	175.89	RW2
Ghana	CENTRAL	1989	142.68	118.99	170.32	RW2
Ghana	CENTRAL	1990	136.21	113.03	162.14	RW2
Ghana	CENTRAL	1991	130.74	109.47	154.80	RW2
Ghana	CENTRAL	1992	125.93	106.13	148.90	RW2
Ghana	CENTRAL	1993	121.99	102.14	145.15	RW2
Ghana	CENTRAL	1994	118.54	97.49	142.43	RW2
Ghana	CENTRAL	1995	116.07	95.54	140.36	RW2
Ghana	CENTRAL	1996	113.38	93.95	136.47	RW2
Ghana	CENTRAL	1997	110.56	91.93	132.49	RW2
Ghana	CENTRAL	1998	107.83	88.85	130.07	RW2
Ghana	CENTRAL	1999	104.78	85.30	127.69	RW2
Ghana	CENTRAL	2000	101.52	82.48	123.95	RW2
Ghana	CENTRAL	2001	98.53	80.53	120.05	RW2
Ghana	CENTRAL	2002	95.86	78.43	116.58	RW2
Ghana	CENTRAL	2003	93.14	75.38	114.24	RW2
Ghana	CENTRAL	2004	90.73	72.34	113.00	RW2
Ghana	CENTRAL	2005	88.49	70.10	111.44	RW2
Ghana	CENTRAL	2006	85.91	67.93	108.31	RW2
Ghana	CENTRAL	2007	83.20	65.35	105.34	RW2
Ghana	CENTRAL	2008	80.48	62.14	103.60	RW2
Ghana	CENTRAL	2009	77.46	58.39	102.45	RW2
Ghana	CENTRAL	2010	74.30	54.88	100.57	RW2
Ghana	CENTRAL	2011	71.06	51.79	97.67	RW2

Continued on next page

Country	Region	Year	Median	Lower	Upper	Method
Ghana	CENTRAL	2012	68.01	48.82	95.16	RW2
Ghana	CENTRAL	2013	65.07	44.84	95.01	RW2
Ghana	CENTRAL	2014	62.29	39.51	98.03	RW2
Ghana	CENTRAL	2015	59.58	33.04	106.72	RW2
Ghana	CENTRAL	2016	57.02	26.83	116.89	RW2
Ghana	CENTRAL	2017	54.48	21.36	131.73	RW2
Ghana	CENTRAL	2018	52.10	16.73	151.69	RW2
Ghana	CENTRAL	2019	49.88	12.51	175.00	RW2
Ghana	EASTERN	1980	128.08	96.64	167.64	RW2
Ghana	EASTERN	1981	126.59	103.36	153.99	RW2
Ghana	EASTERN	1982	124.99	103.83	149.50	RW2
Ghana	EASTERN	1983	123.29	100.90	149.61	RW2
Ghana	EASTERN	1984	121.50	98.46	148.66	RW2
Ghana	EASTERN	1985	119.71	98.25	144.82	RW2
Ghana	EASTERN	1986	117.50	98.02	139.89	RW2
Ghana	EASTERN	1987	114.97	96.94	135.73	RW2
Ghana	EASTERN	1988	112.16	93.91	133.49	RW2
Ghana	EASTERN	1989	109.23	90.51	131.60	RW2
Ghana	EASTERN	1990	106.01	87.65	127.58	RW2
Ghana	EASTERN	1991	103.48	86.47	123.60	RW2
Ghana	EASTERN	1992	101.34	84.96	120.57	RW2
Ghana	EASTERN	1993	99.60	83.12	118.99	RW2
Ghana	EASTERN	1994	98.23	80.82	118.87	RW2
Ghana	EASTERN	1995	97.40	80.10	118.33	RW2
Ghana	EASTERN	1996	96.32	79.65	116.22	RW2
Ghana	EASTERN	1997	95.11	78.92	114.24	RW2
Ghana	EASTERN	1998	93.68	77.30	113.57	RW2
Ghana	EASTERN	1999	92.08	74.93	113.01	RW2
Ghana	EASTERN	2000	90.14	72.75	110.55	RW2
Ghana	EASTERN	2001	88.44	72.08	107.91	RW2
Ghana	EASTERN	2002	86.87	70.88	105.93	RW2
Ghana	EASTERN	2003	85.29	69.10	104.85	RW2
Ghana	EASTERN	2004	83.89	67.07	104.23	RW2
Ghana	EASTERN	2005	82.68	65.61	103.84	RW2
Ghana	EASTERN	2006	81.16	64.53	101.69	RW2
Ghana	EASTERN	2007	79.44	63.02	99.59	RW2
Ghana	EASTERN	2008	77.59	60.82	98.42	RW2
Ghana	EASTERN	2009	75.44	58.14	97.47	RW2
Ghana	EASTERN	2010	73.21	54.95	96.43	RW2
Ghana	EASTERN	2011	70.83	53.02	93.99	RW2
Ghana	EASTERN	2012	68.62	50.69	92.05	RW2
Ghana	EASTERN	2013	66.32	47.09	92.56	RW2
Ghana	EASTERN	2014	64.15	41.93	96.23	RW2
Ghana	EASTERN	2015	62.10	35.38	106.78	RW2
Ghana	EASTERN	2016	60.04	29.31	119.13	RW2
Ghana	EASTERN	2017	58.11	23.48	136.65	RW2
Ghana	EASTERN	2018	56.03	18.17	158.01	RW2
Ghana	EASTERN	2019	54.11	14.14	184.47	RW2
Ghana	GREATER ACCRA	1980	136.52	101.82	180.96	RW2
Ghana	GREATER ACCRA	1981	132.59	106.40	164.52	RW2
Ghana	GREATER ACCRA	1982	128.63	105.22	156.68	RW2
Ghana	GREATER ACCRA	1983	125.02	100.90	153.45	RW2
Ghana	GREATER ACCRA	1984	121.06	96.60	149.92	RW2
Ghana	GREATER ACCRA	1985	117.38	95.20	143.52	RW2
Ghana	GREATER ACCRA	1986	113.19	93.33	136.25	RW2
Ghana	GREATER ACCRA	1987	108.86	90.49	130.40	RW2
Ghana	GREATER ACCRA	1988	104.38	86.44	125.46	RW2
Ghana	GREATER ACCRA	1989	99.98	81.94	122.01	RW2
Ghana	GREATER ACCRA	1990	95.47	77.88	116.34	RW2
Ghana	GREATER ACCRA	1991	91.58	75.40	110.78	RW2
Ghana	GREATER ACCRA	1992	88.18	72.85	106.22	RW2
Ghana	GREATER ACCRA	1993	85.36	70.01	103.52	RW2
Ghana	GREATER ACCRA	1994	82.89	67.04	101.84	RW2

Continued on next page

Country	Region	Year	Median	Lower	Upper	Method
Ghana	GREATER ACCRA	1995	81.08	65.49	100.04	RW2
Ghana	GREATER ACCRA	1996	79.02	64.17	97.03	RW2
Ghana	GREATER ACCRA	1997	76.96	62.51	94.36	RW2
Ghana	GREATER ACCRA	1998	74.80	60.40	92.36	RW2
Ghana	GREATER ACCRA	1999	72.53	57.65	90.79	RW2
Ghana	GREATER ACCRA	2000	70.23	55.55	88.20	RW2
Ghana	GREATER ACCRA	2001	68.12	54.02	85.48	RW2
Ghana	GREATER ACCRA	2002	66.14	52.44	83.17	RW2
Ghana	GREATER ACCRA	2003	64.27	50.37	81.53	RW2
Ghana	GREATER ACCRA	2004	62.49	48.39	80.40	RW2
Ghana	GREATER ACCRA	2005	60.95	46.78	78.98	RW2
Ghana	GREATER ACCRA	2006	59.11	45.29	76.98	RW2
Ghana	GREATER ACCRA	2007	57.36	43.55	75.02	RW2
Ghana	GREATER ACCRA	2008	55.36	41.30	74.18	RW2
Ghana	GREATER ACCRA	2009	53.20	38.63	73.19	RW2
Ghana	GREATER ACCRA	2010	50.92	35.92	72.03	RW2
Ghana	GREATER ACCRA	2011	48.84	33.86	70.14	RW2
Ghana	GREATER ACCRA	2012	46.69	31.65	68.84	RW2
Ghana	GREATER ACCRA	2013	44.49	28.90	68.52	RW2
Ghana	GREATER ACCRA	2014	42.61	25.56	71.22	RW2
Ghana	GREATER ACCRA	2015	40.70	21.37	76.68	RW2
Ghana	GREATER ACCRA	2016	38.93	17.44	85.44	RW2
Ghana	GREATER ACCRA	2017	37.31	13.93	96.19	RW2
Ghana	GREATER ACCRA	2018	35.67	10.84	110.34	RW2
Ghana	GREATER ACCRA	2019	33.92	8.17	130.45	RW2
Ghana	UPPER W,E & NORTHERN	1980	268.70	213.56	332.08	RW2
Ghana	UPPER W,E & NORTHERN	1981	261.29	222.74	304.25	RW2
Ghana	UPPER W,E & NORTHERN	1982	253.97	219.37	291.92	RW2
Ghana	UPPER W,E & NORTHERN	1983	246.53	209.99	286.91	RW2
Ghana	UPPER W,E & NORTHERN	1984	239.12	200.95	281.75	RW2
Ghana	UPPER W,E & NORTHERN	1985	232.00	197.25	270.06	RW2
Ghana	UPPER W,E & NORTHERN	1986	223.96	193.52	257.26	RW2
Ghana	UPPER W,E & NORTHERN	1987	215.65	187.41	246.62	RW2
Ghana	UPPER W,E & NORTHERN	1988	207.18	178.81	238.99	RW2
Ghana	UPPER W,E & NORTHERN	1989	198.69	169.25	232.04	RW2
Ghana	UPPER W,E & NORTHERN	1990	189.94	161.65	221.97	RW2
Ghana	UPPER W,E & NORTHERN	1991	182.52	156.29	211.79	RW2
Ghana	UPPER W,E & NORTHERN	1992	175.83	151.43	203.06	RW2
Ghana	UPPER W,E & NORTHERN	1993	170.13	145.72	197.61	RW2
Ghana	UPPER W,E & NORTHERN	1994	165.14	139.51	193.86	RW2
Ghana	UPPER W,E & NORTHERN	1995	161.18	136.64	189.95	RW2
Ghana	UPPER W,E & NORTHERN	1996	157.03	134.07	183.48	RW2
Ghana	UPPER W,E & NORTHERN	1997	152.98	131.16	177.87	RW2
Ghana	UPPER W,E & NORTHERN	1998	148.82	126.48	174.07	RW2
Ghana	UPPER W,E & NORTHERN	1999	144.47	121.50	170.71	RW2
Ghana	UPPER W,E & NORTHERN	2000	139.94	117.51	165.33	RW2
Ghana	UPPER W,E & NORTHERN	2001	135.76	114.79	159.47	RW2
Ghana	UPPER W,E & NORTHERN	2002	131.89	112.22	154.46	RW2
Ghana	UPPER W,E & NORTHERN	2003	128.06	107.89	151.00	RW2
Ghana	UPPER W,E & NORTHERN	2004	124.44	103.58	148.65	RW2
Ghana	UPPER W,E & NORTHERN	2005	120.97	100.13	145.78	RW2
Ghana	UPPER W,E & NORTHERN	2006	117.22	97.58	140.56	RW2
Ghana	UPPER W,E & NORTHERN	2007	113.19	94.09	135.77	RW2
Ghana	UPPER W,E & NORTHERN	2008	108.91	89.48	132.01	RW2
Ghana	UPPER W,E & NORTHERN	2009	104.40	84.17	128.91	RW2
Ghana	UPPER W,E & NORTHERN	2010	99.53	78.78	124.94	RW2
Ghana	UPPER W,E & NORTHERN	2011	94.96	75.01	119.36	RW2
Ghana	UPPER W,E & NORTHERN	2012	90.35	71.08	114.37	RW2
Ghana	UPPER W,E & NORTHERN	2013	85.93	65.07	113.15	RW2
Ghana	UPPER W,E & NORTHERN	2014	81.67	56.71	116.26	RW2
Ghana	UPPER W,E & NORTHERN	2015	77.75	46.45	127.01	RW2
Ghana	UPPER W,E & NORTHERN	2016	73.81	37.27	139.89	RW2
Ghana	UPPER W,E & NORTHERN	2017	70.21	29.24	159.43	RW2

Continued on next page

Country	Region	Year	Median	Lower	Upper	Method
Ghana	UPPER W,E & NORTHERN	2018	66.75	22.45	180.29	RW2
Ghana	UPPER W,E & NORTHERN	2019	63.53	17.14	205.71	RW2
Ghana	VOLTA	1980	163.11	124.04	210.87	RW2
Ghana	VOLTA	1981	158.42	129.70	191.83	RW2
Ghana	VOLTA	1982	153.87	128.58	183.35	RW2
Ghana	VOLTA	1983	149.43	123.33	180.04	RW2
Ghana	VOLTA	1984	144.97	117.88	176.11	RW2
Ghana	VOLTA	1985	140.55	116.46	169.01	RW2
Ghana	VOLTA	1986	135.76	114.18	160.60	RW2
Ghana	VOLTA	1987	130.78	110.96	153.62	RW2
Ghana	VOLTA	1988	125.65	105.71	148.74	RW2
Ghana	VOLTA	1989	120.43	100.39	144.75	RW2
Ghana	VOLTA	1990	115.28	95.55	137.88	RW2
Ghana	VOLTA	1991	110.86	92.58	132.00	RW2
Ghana	VOLTA	1992	107.05	89.87	126.86	RW2
Ghana	VOLTA	1993	103.73	86.21	124.24	RW2
Ghana	VOLTA	1994	100.92	82.75	122.29	RW2
Ghana	VOLTA	1995	98.75	80.73	120.44	RW2
Ghana	VOLTA	1996	96.45	79.55	117.10	RW2
Ghana	VOLTA	1997	94.12	77.42	113.80	RW2
Ghana	VOLTA	1998	91.54	74.80	111.71	RW2
Ghana	VOLTA	1999	88.95	71.62	109.63	RW2
Ghana	VOLTA	2000	86.11	68.96	106.76	RW2
Ghana	VOLTA	2001	83.49	67.27	103.15	RW2
Ghana	VOLTA	2002	81.08	65.37	100.25	RW2
Ghana	VOLTA	2003	78.79	62.85	97.95	RW2
Ghana	VOLTA	2004	76.52	60.07	96.71	RW2
Ghana	VOLTA	2005	74.54	58.18	95.28	RW2
Ghana	VOLTA	2006	72.23	56.21	92.34	RW2
Ghana	VOLTA	2007	69.85	54.07	90.03	RW2
Ghana	VOLTA	2008	67.23	51.23	88.17	RW2
Ghana	VOLTA	2009	64.62	47.87	86.70	RW2
Ghana	VOLTA	2010	61.75	44.75	84.78	RW2
Ghana	VOLTA	2011	59.06	42.24	81.85	RW2
Ghana	VOLTA	2012	56.36	39.80	79.86	RW2
Ghana	VOLTA	2013	53.74	36.30	78.93	RW2
Ghana	VOLTA	2014	51.23	32.05	81.46	RW2
Ghana	VOLTA	2015	48.97	26.50	88.78	RW2
Ghana	VOLTA	2016	46.58	21.81	97.13	RW2
Ghana	VOLTA	2017	44.39	17.08	109.54	RW2
Ghana	VOLTA	2018	42.38	13.28	124.75	RW2
Ghana	VOLTA	2019	40.43	10.02	143.77	RW2
Ghana	WESTERN	1980	163.11	121.57	214.67	RW2
Ghana	WESTERN	1981	159.48	128.05	197.44	RW2
Ghana	WESTERN	1982	155.90	127.46	189.07	RW2
Ghana	WESTERN	1983	152.24	123.30	186.20	RW2
Ghana	WESTERN	1984	148.52	119.56	182.64	RW2
Ghana	WESTERN	1985	144.90	118.70	176.09	RW2
Ghana	WESTERN	1986	140.77	117.09	168.23	RW2
Ghana	WESTERN	1987	136.30	114.47	161.51	RW2
Ghana	WESTERN	1988	131.55	110.02	156.96	RW2
Ghana	WESTERN	1989	126.86	105.19	152.98	RW2
Ghana	WESTERN	1990	121.75	100.71	146.58	RW2
Ghana	WESTERN	1991	117.65	98.00	140.16	RW2
Ghana	WESTERN	1992	113.98	95.51	135.47	RW2
Ghana	WESTERN	1993	110.80	92.30	132.72	RW2
Ghana	WESTERN	1994	108.18	88.97	131.17	RW2
Ghana	WESTERN	1995	106.18	87.37	129.15	RW2
Ghana	WESTERN	1996	103.74	85.76	125.40	RW2
Ghana	WESTERN	1997	101.34	84.20	122.16	RW2
Ghana	WESTERN	1998	98.67	81.39	119.83	RW2
Ghana	WESTERN	1999	95.83	77.89	117.48	RW2
Ghana	WESTERN	2000	92.58	74.84	113.92	RW2

Continued on next page

Country	Region	Year	Median	Lower	Upper	Method
Ghana	WESTERN	2001	89.55	72.94	109.64	RW2
Ghana	WESTERN	2002	86.62	70.58	105.73	RW2
Ghana	WESTERN	2003	83.69	67.59	103.29	RW2
Ghana	WESTERN	2004	80.94	64.42	101.40	RW2
Ghana	WESTERN	2005	78.38	61.82	99.11	RW2
Ghana	WESTERN	2006	75.49	59.46	95.44	RW2
Ghana	WESTERN	2007	72.47	56.72	92.14	RW2
Ghana	WESTERN	2008	69.33	53.24	89.56	RW2
Ghana	WESTERN	2009	66.17	49.71	87.30	RW2
Ghana	WESTERN	2010	62.83	46.07	84.61	RW2
Ghana	WESTERN	2011	59.63	43.16	81.18	RW2
Ghana	WESTERN	2012	56.59	40.17	78.18	RW2
Ghana	WESTERN	2013	53.61	36.75	77.00	RW2
Ghana	WESTERN	2014	50.72	32.02	78.64	RW2
Ghana	WESTERN	2015	48.04	26.37	85.25	RW2
Ghana	WESTERN	2016	45.44	21.18	93.12	RW2
Ghana	WESTERN	2017	43.07	16.75	104.48	RW2
Ghana	WESTERN	2018	40.63	12.84	118.79	RW2
Ghana	WESTERN	2019	38.57	9.64	139.38	RW2
Guinea	ALL	1980	275.01	264.87	286.04	IHME
Guinea	ALL	1980	287.24	225.44	357.62	RW2
Guinea	ALL	1980	286.90	266.40	309.10	UN
Guinea	ALL	1981	270.62	261.22	281.23	IHME
Guinea	ALL	1981	282.34	237.92	330.90	RW2
Guinea	ALL	1981	282.20	262.20	303.40	UN
Guinea	ALL	1982	266.27	257.31	276.33	IHME
Guinea	ALL	1982	277.54	237.82	321.07	RW2
Guinea	ALL	1982	277.40	258.00	297.60	UN
Guinea	ALL	1983	261.93	253.03	271.47	IHME
Guinea	ALL	1983	272.48	229.60	320.00	RW2
Guinea	ALL	1983	272.60	253.70	292.40	UN
Guinea	ALL	1984	257.35	248.57	266.68	IHME
Guinea	ALL	1984	267.88	221.39	319.43	RW2
Guinea	ALL	1984	267.80	249.70	287.00	UN
Guinea	ALL	1985	252.77	244.34	261.53	IHME
Guinea	ALL	1985	262.75	220.68	309.87	RW2
Guinea	ALL	1985	263.10	245.60	281.60	UN
Guinea	ALL	1986	248.32	240.17	256.37	IHME
Guinea	ALL	1986	257.89	219.23	300.69	RW2
Guinea	ALL	1986	258.40	241.40	276.60	UN
Guinea	ALL	1987	243.83	235.90	251.72	IHME
Guinea	ALL	1987	253.08	216.78	293.75	RW2
Guinea	ALL	1987	253.60	237.20	271.30	UN
Guinea	ALL	1988	239.07	231.73	246.74	IHME
Guinea	ALL	1988	248.02	210.53	289.53	RW2
Guinea	ALL	1988	248.80	232.90	266.00	UN
Guinea	ALL	1989	234.15	227.00	241.63	IHME
Guinea	ALL	1989	242.86	204.09	286.22	RW2
Guinea	ALL	1989	243.70	228.00	260.60	UN
Guinea	ALL	1990	229.11	222.30	236.52	IHME
Guinea	ALL	1990	237.67	200.11	280.31	RW2
Guinea	ALL	1990	238.20	223.10	254.80	UN
Guinea	ALL	1991	223.98	217.18	231.18	IHME
Guinea	ALL	1991	232.06	197.21	270.57	RW2
Guinea	ALL	1991	232.40	217.70	248.50	UN
Guinea	ALL	1992	219.01	212.18	226.18	IHME
Guinea	ALL	1992	226.14	192.78	262.92	RW2
Guinea	ALL	1992	226.20	211.80	241.90	UN
Guinea	ALL	1993	213.79	207.06	220.79	IHME
Guinea	ALL	1993	219.92	186.45	257.36	RW2
Guinea	ALL	1993	219.70	205.70	234.80	UN
Guinea	ALL	1994	208.42	201.89	215.20	IHME
Guinea	ALL	1994	213.27	178.63	253.13	RW2

Continued on next page

Country	Region	Year	Median	Lower	Upper	Method
Guinea	ALL	1994	212.90	199.30	227.60	UN
Guinea	ALL	1995	202.81	196.53	209.34	IHME
Guinea	ALL	1995	206.46	172.90	244.46	RW2
Guinea	ALL	1995	205.90	192.80	220.30	UN
Guinea	ALL	1996	196.88	190.79	203.22	IHME
Guinea	ALL	1996	199.24	168.36	234.71	RW2
Guinea	ALL	1996	199.00	186.10	212.80	UN
Guinea	ALL	1997	191.16	185.11	197.50	IHME
Guinea	ALL	1997	191.87	162.89	224.75	RW2
Guinea	ALL	1997	192.00	179.50	205.40	UN
Guinea	ALL	1998	185.70	179.57	192.00	IHME
Guinea	ALL	1998	184.48	155.54	218.35	RW2
Guinea	ALL	1998	184.80	172.70	197.80	UN
Guinea	ALL	1999	180.22	173.98	186.66	IHME
Guinea	ALL	1999	177.03	147.31	211.09	RW2
Guinea	ALL	1999	177.50	165.80	190.30	UN
Guinea	ALL	2000	175.08	168.89	181.33	IHME
Guinea	ALL	2000	169.56	140.79	202.41	RW2
Guinea	ALL	2000	170.20	158.60	182.70	UN
Guinea	ALL	2001	169.94	163.71	176.28	IHME
Guinea	ALL	2001	162.49	135.93	192.85	RW2
Guinea	ALL	2001	162.90	151.40	175.30	UN
Guinea	ALL	2002	164.76	158.48	171.12	IHME
Guinea	ALL	2002	155.72	131.04	184.13	RW2
Guinea	ALL	2002	155.80	144.20	168.20	UN
Guinea	ALL	2003	159.97	153.56	166.57	IHME
Guinea	ALL	2003	149.40	125.12	177.51	RW2
Guinea	ALL	2003	149.00	137.50	161.40	UN
Guinea	ALL	2004	155.43	148.79	162.32	IHME
Guinea	ALL	2004	143.23	117.93	172.57	RW2
Guinea	ALL	2004	142.70	131.00	155.30	UN
Guinea	ALL	2005	151.05	144.27	158.14	IHME
Guinea	ALL	2005	137.70	112.75	167.36	RW2
Guinea	ALL	2005	136.70	124.90	149.60	UN
Guinea	ALL	2006	146.68	139.40	153.93	IHME
Guinea	ALL	2006	132.22	109.04	159.42	RW2
Guinea	ALL	2006	131.20	118.90	144.50	UN
Guinea	ALL	2007	142.22	134.66	149.79	IHME
Guinea	ALL	2007	127.03	105.29	152.50	RW2
Guinea	ALL	2007	125.90	113.20	140.10	UN
Guinea	ALL	2008	137.63	129.59	145.73	IHME
Guinea	ALL	2008	122.19	100.43	147.98	RW2
Guinea	ALL	2008	121.00	107.60	136.20	UN
Guinea	ALL	2009	133.35	125.11	141.98	IHME
Guinea	ALL	2009	117.29	94.84	144.61	RW2
Guinea	ALL	2009	116.30	102.00	133.00	UN
Guinea	ALL	2010	129.00	120.10	138.56	IHME
Guinea	ALL	2010	112.64	89.76	141.12	RW2
Guinea	ALL	2010	111.90	96.60	129.80	UN
Guinea	ALL	2011	124.62	115.19	134.41	IHME
Guinea	ALL	2011	108.25	86.77	134.27	RW2
Guinea	ALL	2011	107.70	91.60	126.80	UN
Guinea	ALL	2012	120.40	110.57	130.95	IHME
Guinea	ALL	2012	103.94	83.79	128.17	RW2
Guinea	ALL	2012	104.00	86.90	124.70	UN
Guinea	ALL	2013	116.21	105.76	127.21	IHME
Guinea	ALL	2013	99.87	78.10	126.66	RW2
Guinea	ALL	2013	100.40	81.80	123.40	UN
Guinea	ALL	2014	112.71	101.75	124.64	IHME
Guinea	ALL	2014	95.84	68.28	132.77	RW2
Guinea	ALL	2014	97.00	76.70	122.60	UN
Guinea	ALL	2015	108.75	97.10	121.41	IHME
Guinea	ALL	2015	91.85	55.56	148.22	RW2

Continued on next page

Country	Region	Year	Median	Lower	Upper	Method
Guinea	ALL	2015	93.70	71.80	122.00	UN
Guinea	ALL	2016	88.33	45.01	166.99	RW2
Guinea	ALL	2017	84.56	35.17	191.05	RW2
Guinea	ALL	2018	81.08	27.18	222.59	RW2
Guinea	ALL	2019	77.63	19.83	256.93	RW2
Guinea	ALL	15-19	84.56	35.67	188.23	RW2
Guinea	CENTRAL GUINEA	1980	278.21	218.37	347.67	RW2
Guinea	CENTRAL GUINEA	1981	272.27	228.00	322.00	RW2
Guinea	CENTRAL GUINEA	1982	266.05	227.44	308.58	RW2
Guinea	CENTRAL GUINEA	1983	260.02	220.40	304.06	RW2
Guinea	CENTRAL GUINEA	1984	254.08	212.84	299.85	RW2
Guinea	CENTRAL GUINEA	1985	248.38	211.00	290.01	RW2
Guinea	CENTRAL GUINEA	1986	242.94	209.06	279.84	RW2
Guinea	CENTRAL GUINEA	1987	237.70	205.97	272.47	RW2
Guinea	CENTRAL GUINEA	1988	232.71	200.37	268.13	RW2
Guinea	CENTRAL GUINEA	1989	227.84	194.60	264.39	RW2
Guinea	CENTRAL GUINEA	1990	223.19	191.14	258.86	RW2
Guinea	CENTRAL GUINEA	1991	218.46	189.20	250.80	RW2
Guinea	CENTRAL GUINEA	1992	213.47	185.52	244.03	RW2
Guinea	CENTRAL GUINEA	1993	208.23	180.12	239.36	RW2
Guinea	CENTRAL GUINEA	1994	203.09	173.57	235.78	RW2
Guinea	CENTRAL GUINEA	1995	197.49	168.63	229.54	RW2
Guinea	CENTRAL GUINEA	1996	192.15	165.66	221.42	RW2
Guinea	CENTRAL GUINEA	1997	186.73	161.98	214.63	RW2
Guinea	CENTRAL GUINEA	1998	181.35	156.79	209.30	RW2
Guinea	CENTRAL GUINEA	1999	175.98	149.69	205.09	RW2
Guinea	CENTRAL GUINEA	2000	170.52	145.04	199.13	RW2
Guinea	CENTRAL GUINEA	2001	165.04	141.73	191.01	RW2
Guinea	CENTRAL GUINEA	2002	159.37	137.68	183.75	RW2
Guinea	CENTRAL GUINEA	2003	153.51	131.71	178.52	RW2
Guinea	CENTRAL GUINEA	2004	147.62	124.54	174.02	RW2
Guinea	CENTRAL GUINEA	2005	141.61	118.52	168.56	RW2
Guinea	CENTRAL GUINEA	2006	135.66	113.97	160.94	RW2
Guinea	CENTRAL GUINEA	2007	129.65	108.36	154.07	RW2
Guinea	CENTRAL GUINEA	2008	123.82	101.89	149.41	RW2
Guinea	CENTRAL GUINEA	2009	117.96	94.92	145.44	RW2
Guinea	CENTRAL GUINEA	2010	112.42	88.47	141.76	RW2
Guinea	CENTRAL GUINEA	2011	106.96	83.25	136.07	RW2
Guinea	CENTRAL GUINEA	2012	101.79	77.86	131.22	RW2
Guinea	CENTRAL GUINEA	2013	96.86	70.77	130.42	RW2
Guinea	CENTRAL GUINEA	2014	92.13	61.60	134.02	RW2
Guinea	CENTRAL GUINEA	2015	87.54	50.81	144.96	RW2
Guinea	CENTRAL GUINEA	2016	83.03	41.18	158.38	RW2
Guinea	CENTRAL GUINEA	2017	78.98	32.63	177.04	RW2
Guinea	CENTRAL GUINEA	2018	74.78	25.13	200.90	RW2
Guinea	CENTRAL GUINEA	2019	71.04	19.03	231.52	RW2
Guinea	CONAKRY	1980	232.66	171.89	308.12	RW2
Guinea	CONAKRY	1981	223.73	177.47	278.83	RW2
Guinea	CONAKRY	1982	215.15	175.45	261.02	RW2
Guinea	CONAKRY	1983	206.56	168.31	250.88	RW2
Guinea	CONAKRY	1984	198.41	160.84	242.78	RW2
Guinea	CONAKRY	1985	190.61	156.43	230.12	RW2
Guinea	CONAKRY	1986	183.17	152.71	218.41	RW2
Guinea	CONAKRY	1987	176.14	147.62	208.85	RW2
Guinea	CONAKRY	1988	169.52	141.53	201.69	RW2
Guinea	CONAKRY	1989	163.11	134.95	195.57	RW2
Guinea	CONAKRY	1990	157.02	130.13	187.90	RW2
Guinea	CONAKRY	1991	150.79	126.16	179.21	RW2
Guinea	CONAKRY	1992	144.40	121.70	171.16	RW2
Guinea	CONAKRY	1993	138.08	115.79	164.52	RW2
Guinea	CONAKRY	1994	131.53	108.61	158.07	RW2
Guinea	CONAKRY	1995	125.10	103.25	150.47	RW2
Guinea	CONAKRY	1996	118.87	98.90	142.29	RW2

Continued on next page

Country	Region	Year	Median	Lower	Upper	Method
Guinea	CONAKRY	1997	112.73	94.03	134.54	RW2
Guinea	CONAKRY	1998	107.06	88.11	129.02	RW2
Guinea	CONAKRY	1999	101.52	82.02	124.11	RW2
Guinea	CONAKRY	2000	96.41	77.27	118.89	RW2
Guinea	CONAKRY	2001	91.61	73.35	113.23	RW2
Guinea	CONAKRY	2002	87.33	69.69	108.09	RW2
Guinea	CONAKRY	2003	83.13	65.59	104.03	RW2
Guinea	CONAKRY	2004	79.36	61.76	101.03	RW2
Guinea	CONAKRY	2005	75.73	58.51	97.55	RW2
Guinea	CONAKRY	2006	72.10	55.52	93.31	RW2
Guinea	CONAKRY	2007	68.51	52.25	89.40	RW2
Guinea	CONAKRY	2008	65.09	48.57	86.76	RW2
Guinea	CONAKRY	2009	61.56	44.49	84.92	RW2
Guinea	CONAKRY	2010	58.14	40.77	83.05	RW2
Guinea	CONAKRY	2011	54.66	37.11	80.67	RW2
Guinea	CONAKRY	2012	51.46	33.64	78.92	RW2
Guinea	CONAKRY	2013	48.45	29.75	79.18	RW2
Guinea	CONAKRY	2014	45.62	25.40	81.74	RW2
Guinea	CONAKRY	2015	42.89	20.76	88.41	RW2
Guinea	CONAKRY	2016	40.37	16.46	96.27	RW2
Guinea	CONAKRY	2017	37.91	12.83	107.95	RW2
Guinea	CONAKRY	2018	35.65	9.84	123.89	RW2
Guinea	CONAKRY	2019	33.57	7.20	142.46	RW2
Guinea	FOREST GUINEA	1980	285.04	221.53	356.84	RW2
Guinea	FOREST GUINEA	1981	286.60	240.38	338.31	RW2
Guinea	FOREST GUINEA	1982	288.14	247.14	332.85	RW2
Guinea	FOREST GUINEA	1983	289.52	246.26	336.77	RW2
Guinea	FOREST GUINEA	1984	290.73	245.54	340.20	RW2
Guinea	FOREST GUINEA	1985	291.59	249.32	338.10	RW2
Guinea	FOREST GUINEA	1986	292.00	252.72	334.54	RW2
Guinea	FOREST GUINEA	1987	291.63	254.25	332.10	RW2
Guinea	FOREST GUINEA	1988	290.31	252.00	332.16	RW2
Guinea	FOREST GUINEA	1989	288.05	248.31	331.85	RW2
Guinea	FOREST GUINEA	1990	284.28	245.80	327.27	RW2
Guinea	FOREST GUINEA	1991	279.10	242.95	317.96	RW2
Guinea	FOREST GUINEA	1992	272.06	237.99	308.99	RW2
Guinea	FOREST GUINEA	1993	263.26	229.11	301.13	RW2
Guinea	FOREST GUINEA	1994	253.24	217.99	292.96	RW2
Guinea	FOREST GUINEA	1995	242.01	208.09	279.67	RW2
Guinea	FOREST GUINEA	1996	230.17	199.24	264.23	RW2
Guinea	FOREST GUINEA	1997	218.34	190.19	250.01	RW2
Guinea	FOREST GUINEA	1998	206.46	178.50	238.32	RW2
Guinea	FOREST GUINEA	1999	194.87	165.84	227.40	RW2
Guinea	FOREST GUINEA	2000	183.53	155.54	215.33	RW2
Guinea	FOREST GUINEA	2001	172.54	147.44	201.14	RW2
Guinea	FOREST GUINEA	2002	162.02	138.61	188.16	RW2
Guinea	FOREST GUINEA	2003	151.91	128.59	178.47	RW2
Guinea	FOREST GUINEA	2004	142.55	118.57	170.43	RW2
Guinea	FOREST GUINEA	2005	133.98	110.00	161.81	RW2
Guinea	FOREST GUINEA	2006	126.10	103.51	152.14	RW2
Guinea	FOREST GUINEA	2007	119.05	97.46	144.03	RW2
Guinea	FOREST GUINEA	2008	112.81	91.12	138.14	RW2
Guinea	FOREST GUINEA	2009	107.38	85.38	133.71	RW2
Guinea	FOREST GUINEA	2010	102.48	80.29	129.63	RW2
Guinea	FOREST GUINEA	2011	98.00	76.66	124.29	RW2
Guinea	FOREST GUINEA	2012	93.91	72.80	120.08	RW2
Guinea	FOREST GUINEA	2013	89.96	67.36	119.66	RW2
Guinea	FOREST GUINEA	2014	86.12	59.00	124.59	RW2
Guinea	FOREST GUINEA	2015	82.46	48.69	137.45	RW2
Guinea	FOREST GUINEA	2016	78.88	39.32	152.47	RW2
Guinea	FOREST GUINEA	2017	75.60	31.27	172.91	RW2
Guinea	FOREST GUINEA	2018	72.12	24.14	197.91	RW2
Guinea	FOREST GUINEA	2019	69.23	18.27	232.23	RW2

Continued on next page

Country	Region	Year	Median	Lower	Upper	Method
Guinea	LOWER GUINEA	1980	290.62	226.63	363.88	RW2
Guinea	LOWER GUINEA	1981	283.39	236.35	335.40	RW2
Guinea	LOWER GUINEA	1982	276.16	235.26	321.24	RW2
Guinea	LOWER GUINEA	1983	268.69	227.77	314.59	RW2
Guinea	LOWER GUINEA	1984	261.91	220.25	308.20	RW2
Guinea	LOWER GUINEA	1985	255.10	217.06	297.51	RW2
Guinea	LOWER GUINEA	1986	248.73	214.41	286.54	RW2
Guinea	LOWER GUINEA	1987	242.84	210.33	278.34	RW2
Guinea	LOWER GUINEA	1988	237.13	204.31	272.55	RW2
Guinea	LOWER GUINEA	1989	231.82	198.09	268.82	RW2
Guinea	LOWER GUINEA	1990	226.74	193.93	262.74	RW2
Guinea	LOWER GUINEA	1991	221.60	191.78	254.57	RW2
Guinea	LOWER GUINEA	1992	216.55	188.17	247.64	RW2
Guinea	LOWER GUINEA	1993	211.24	182.16	243.57	RW2
Guinea	LOWER GUINEA	1994	205.93	175.79	239.91	RW2
Guinea	LOWER GUINEA	1995	200.12	170.96	232.88	RW2
Guinea	LOWER GUINEA	1996	194.53	167.49	224.46	RW2
Guinea	LOWER GUINEA	1997	188.76	163.43	217.28	RW2
Guinea	LOWER GUINEA	1998	182.92	157.26	211.97	RW2
Guinea	LOWER GUINEA	1999	176.70	149.68	207.34	RW2
Guinea	LOWER GUINEA	2000	170.64	144.46	200.10	RW2
Guinea	LOWER GUINEA	2001	164.21	140.27	191.29	RW2
Guinea	LOWER GUINEA	2002	157.67	135.26	183.29	RW2
Guinea	LOWER GUINEA	2003	150.78	127.85	176.66	RW2
Guinea	LOWER GUINEA	2004	143.77	119.97	171.49	RW2
Guinea	LOWER GUINEA	2005	136.44	112.81	164.36	RW2
Guinea	LOWER GUINEA	2006	129.04	106.53	155.34	RW2
Guinea	LOWER GUINEA	2007	121.55	100.04	146.68	RW2
Guinea	LOWER GUINEA	2008	113.94	92.37	139.89	RW2
Guinea	LOWER GUINEA	2009	106.52	84.01	133.68	RW2
Guinea	LOWER GUINEA	2010	99.22	75.78	128.13	RW2
Guinea	LOWER GUINEA	2011	92.31	69.39	121.07	RW2
Guinea	LOWER GUINEA	2012	85.73	62.56	114.76	RW2
Guinea	LOWER GUINEA	2013	79.53	54.81	111.84	RW2
Guinea	LOWER GUINEA	2014	73.69	46.19	112.94	RW2
Guinea	LOWER GUINEA	2015	68.32	36.89	119.77	RW2
Guinea	LOWER GUINEA	2016	63.22	29.02	128.52	RW2
Guinea	LOWER GUINEA	2017	58.41	22.38	140.26	RW2
Guinea	LOWER GUINEA	2018	54.07	16.86	156.58	RW2
Guinea	LOWER GUINEA	2019	50.02	12.31	176.70	RW2
Guinea	UPPER GUINEA	1980	325.72	257.77	403.27	RW2
Guinea	UPPER GUINEA	1981	320.29	269.97	375.66	RW2
Guinea	UPPER GUINEA	1982	314.95	270.74	362.29	RW2
Guinea	UPPER GUINEA	1983	309.31	264.34	358.88	RW2
Guinea	UPPER GUINEA	1984	303.93	257.73	354.56	RW2
Guinea	UPPER GUINEA	1985	298.30	255.15	344.47	RW2
Guinea	UPPER GUINEA	1986	292.45	254.02	334.76	RW2
Guinea	UPPER GUINEA	1987	286.65	250.77	325.65	RW2
Guinea	UPPER GUINEA	1988	280.50	244.07	320.07	RW2
Guinea	UPPER GUINEA	1989	273.92	236.18	315.46	RW2
Guinea	UPPER GUINEA	1990	267.23	230.83	307.56	RW2
Guinea	UPPER GUINEA	1991	260.06	226.67	295.78	RW2
Guinea	UPPER GUINEA	1992	252.12	220.89	285.89	RW2
Guinea	UPPER GUINEA	1993	243.97	212.93	278.61	RW2
Guinea	UPPER GUINEA	1994	235.75	203.07	272.27	RW2
Guinea	UPPER GUINEA	1995	227.14	195.18	262.58	RW2
Guinea	UPPER GUINEA	1996	219.10	190.26	250.37	RW2
Guinea	UPPER GUINEA	1997	211.56	184.64	241.69	RW2
Guinea	UPPER GUINEA	1998	204.75	177.44	235.27	RW2
Guinea	UPPER GUINEA	1999	198.41	169.69	230.37	RW2
Guinea	UPPER GUINEA	2000	192.83	164.65	224.85	RW2
Guinea	UPPER GUINEA	2001	187.90	161.62	217.34	RW2
Guinea	UPPER GUINEA	2002	183.44	158.60	211.32	RW2

Continued on next page

Country	Region	Year	Median	Lower	Upper	Method
Guinea	UPPER GUINEA	2003	179.50	153.99	208.03	RW2
Guinea	UPPER GUINEA	2004	176.11	148.93	206.26	RW2
Guinea	UPPER GUINEA	2005	173.16	145.68	204.38	RW2
Guinea	UPPER GUINEA	2006	170.63	144.84	200.41	RW2
Guinea	UPPER GUINEA	2007	168.72	143.51	197.82	RW2
Guinea	UPPER GUINEA	2008	167.20	140.50	198.03	RW2
Guinea	UPPER GUINEA	2009	165.83	136.82	199.73	RW2
Guinea	UPPER GUINEA	2010	164.92	133.48	202.29	RW2
Guinea	UPPER GUINEA	2011	164.10	132.74	201.85	RW2
Guinea	UPPER GUINEA	2012	163.37	131.06	202.60	RW2
Guinea	UPPER GUINEA	2013	162.68	125.87	208.84	RW2
Guinea	UPPER GUINEA	2014	161.82	115.16	223.41	RW2
Guinea	UPPER GUINEA	2015	161.32	100.29	250.49	RW2
Guinea	UPPER GUINEA	2016	160.44	85.52	281.69	RW2
Guinea	UPPER GUINEA	2017	160.02	71.56	321.40	RW2
Guinea	UPPER GUINEA	2018	158.92	58.59	367.00	RW2
Guinea	UPPER GUINEA	2019	158.65	47.28	420.67	RW2
Kenya	ALL	1980	107.20	103.15	111.60	IHME
Kenya	ALL	1980	106.95	78.24	144.05	RW2
Kenya	ALL	1980	108.70	102.10	115.60	UN
Kenya	ALL	1981	104.26	100.38	108.30	IHME
Kenya	ALL	1981	104.65	83.81	129.17	RW2
Kenya	ALL	1981	105.30	98.90	111.90	UN
Kenya	ALL	1982	101.70	97.96	105.39	IHME
Kenya	ALL	1982	102.39	84.05	124.39	RW2
Kenya	ALL	1982	102.30	96.00	108.80	UN
Kenya	ALL	1983	99.56	95.89	103.19	IHME
Kenya	ALL	1983	100.18	80.72	124.48	RW2
Kenya	ALL	1983	99.80	93.50	106.20	UN
Kenya	ALL	1984	97.65	94.27	101.08	IHME
Kenya	ALL	1984	98.53	77.66	125.14	RW2
Kenya	ALL	1984	97.90	91.70	104.30	UN
Kenya	ALL	1985	95.93	92.69	99.53	IHME
Kenya	ALL	1985	96.69	77.30	119.90	RW2
Kenya	ALL	1985	96.80	90.60	103.10	UN
Kenya	ALL	1986	94.43	91.20	97.82	IHME
Kenya	ALL	1986	96.19	78.30	117.43	RW2
Kenya	ALL	1986	96.30	90.30	102.60	UN
Kenya	ALL	1987	93.32	90.06	96.61	IHME
Kenya	ALL	1987	96.59	79.54	117.09	RW2
Kenya	ALL	1987	96.70	90.70	103.00	UN
Kenya	ALL	1988	92.58	89.36	96.00	IHME
Kenya	ALL	1988	97.82	79.66	119.18	RW2
Kenya	ALL	1988	97.90	91.90	104.20	UN
Kenya	ALL	1989	92.32	89.04	95.64	IHME
Kenya	ALL	1989	99.85	80.30	122.83	RW2
Kenya	ALL	1989	99.80	93.70	106.20	UN
Kenya	ALL	1990	92.29	88.88	95.40	IHME
Kenya	ALL	1990	102.96	83.55	127.41	RW2
Kenya	ALL	1990	102.30	96.10	108.90	UN
Kenya	ALL	1991	92.75	89.38	95.99	IHME
Kenya	ALL	1991	105.85	86.86	128.62	RW2
Kenya	ALL	1991	105.20	98.80	112.20	UN
Kenya	ALL	1992	93.50	90.16	96.86	IHME
Kenya	ALL	1992	108.67	89.53	131.08	RW2
Kenya	ALL	1992	108.20	101.50	115.40	UN
Kenya	ALL	1993	94.48	91.03	97.84	IHME
Kenya	ALL	1993	111.17	91.09	134.76	RW2
Kenya	ALL	1993	111.10	104.10	118.60	UN
Kenya	ALL	1994	95.22	91.66	98.53	IHME
Kenya	ALL	1994	113.06	91.49	139.08	RW2
Kenya	ALL	1994	113.40	106.20	121.10	UN
Kenya	ALL	1995	95.04	91.50	98.54	IHME

Continued on next page

Country	Region	Year	Median	Lower	Upper	Method
Kenya	ALL	1995	114.26	92.63	140.06	RW2
Kenya	ALL	1995	114.80	107.40	122.70	UN
Kenya	ALL	1996	94.94	91.33	98.74	IHME
Kenya	ALL	1996	114.72	94.07	139.55	RW2
Kenya	ALL	1996	115.40	107.80	123.40	UN
Kenya	ALL	1997	94.40	90.79	98.26	IHME
Kenya	ALL	1997	114.40	94.43	138.00	RW2
Kenya	ALL	1997	114.90	107.30	123.10	UN
Kenya	ALL	1998	93.15	89.55	96.99	IHME
Kenya	ALL	1998	113.32	92.91	138.06	RW2
Kenya	ALL	1998	113.40	105.70	121.90	UN
Kenya	ALL	1999	91.22	87.39	95.18	IHME
Kenya	ALL	1999	111.35	90.08	136.52	RW2
Kenya	ALL	1999	111.00	103.30	119.60	UN
Kenya	ALL	2000	88.56	84.78	92.67	IHME
Kenya	ALL	2000	108.61	87.90	133.32	RW2
Kenya	ALL	2000	107.90	100.20	116.70	UN
Kenya	ALL	2001	85.47	81.53	89.39	IHME
Kenya	ALL	2001	105.01	85.69	127.92	RW2
Kenya	ALL	2001	104.10	96.40	113.00	UN
Kenya	ALL	2002	82.07	78.30	86.03	IHME
Kenya	ALL	2002	100.66	82.74	122.08	RW2
Kenya	ALL	2002	99.90	92.10	108.90	UN
Kenya	ALL	2003	78.56	74.89	82.35	IHME
Kenya	ALL	2003	95.83	78.46	116.90	RW2
Kenya	ALL	2003	95.40	87.40	104.50	UN
Kenya	ALL	2004	75.10	71.46	79.13	IHME
Kenya	ALL	2004	90.51	72.88	112.19	RW2
Kenya	ALL	2004	90.60	82.50	99.80	UN
Kenya	ALL	2005	71.15	67.40	75.04	IHME
Kenya	ALL	2005	85.02	67.75	105.66	RW2
Kenya	ALL	2005	85.50	77.00	94.60	UN
Kenya	ALL	2006	67.26	63.61	70.95	IHME
Kenya	ALL	2006	79.84	64.24	98.40	RW2
Kenya	ALL	2006	80.70	72.10	89.80	UN
Kenya	ALL	2007	63.82	60.27	67.45	IHME
Kenya	ALL	2007	75.02	60.78	92.08	RW2
Kenya	ALL	2007	76.10	67.40	85.50	UN
Kenya	ALL	2008	60.16	56.60	63.80	IHME
Kenya	ALL	2008	70.67	56.75	87.61	RW2
Kenya	ALL	2008	70.10	61.40	79.50	UN
Kenya	ALL	2009	57.60	53.95	61.28	IHME
Kenya	ALL	2009	66.56	52.53	84.07	RW2
Kenya	ALL	2009	65.80	56.80	75.50	UN
Kenya	ALL	2010	55.40	51.50	59.31	IHME
Kenya	ALL	2010	62.98	49.22	80.91	RW2
Kenya	ALL	2010	62.10	52.90	72.30	UN
Kenya	ALL	2011	53.35	49.33	57.62	IHME
Kenya	ALL	2011	59.59	47.16	75.21	RW2
Kenya	ALL	2011	58.50	49.20	69.40	UN
Kenya	ALL	2012	52.21	48.02	56.73	IHME
Kenya	ALL	2012	56.38	45.32	69.94	RW2
Kenya	ALL	2012	55.60	45.80	67.20	UN
Kenya	ALL	2013	51.75	47.24	57.06	IHME
Kenya	ALL	2013	53.41	41.91	67.66	RW2
Kenya	ALL	2013	53.40	43.10	65.90	UN
Kenya	ALL	2014	50.85	45.87	56.87	IHME
Kenya	ALL	2014	50.51	35.86	70.71	RW2
Kenya	ALL	2014	51.30	40.30	64.60	UN
Kenya	ALL	2015	50.83	45.37	57.64	IHME
Kenya	ALL	2015	47.70	28.23	79.77	RW2
Kenya	ALL	2015	49.40	38.00	64.00	UN
Kenya	ALL	2016	45.20	22.22	90.89	RW2

Continued on next page

Country	Region	Year	Median	Lower	Upper	Method
Kenya	ALL	2017	42.63	16.85	105.59	RW2
Kenya	ALL	2018	40.28	12.64	125.63	RW2
Kenya	ALL	2019	38.00	8.93	148.25	RW2
Kenya	ALL	15-19	42.62	17.13	103.78	RW2
Kenya	CENTRAL	1980	53.30	36.05	78.60	RW2
Kenya	CENTRAL	1981	51.07	37.70	69.10	RW2
Kenya	CENTRAL	1982	48.98	37.36	64.10	RW2
Kenya	CENTRAL	1983	47.00	35.82	61.73	RW2
Kenya	CENTRAL	1984	45.36	34.46	60.13	RW2
Kenya	CENTRAL	1985	43.90	33.81	56.58	RW2
Kenya	CENTRAL	1986	43.27	34.16	54.64	RW2
Kenya	CENTRAL	1987	43.31	34.55	54.13	RW2
Kenya	CENTRAL	1988	44.09	35.05	55.22	RW2
Kenya	CENTRAL	1989	45.58	35.82	57.53	RW2
Kenya	CENTRAL	1990	47.93	37.89	60.46	RW2
Kenya	CENTRAL	1991	50.64	40.50	63.14	RW2
Kenya	CENTRAL	1992	53.71	43.33	66.55	RW2
Kenya	CENTRAL	1993	57.10	45.76	71.04	RW2
Kenya	CENTRAL	1994	60.43	47.52	75.88	RW2
Kenya	CENTRAL	1995	63.84	50.34	80.60	RW2
Kenya	CENTRAL	1996	66.67	53.03	83.61	RW2
Kenya	CENTRAL	1997	68.76	55.00	85.69	RW2
Kenya	CENTRAL	1998	70.19	55.67	88.01	RW2
Kenya	CENTRAL	1999	70.60	55.34	89.42	RW2
Kenya	CENTRAL	2000	70.07	54.83	88.76	RW2
Kenya	CENTRAL	2001	68.98	54.41	87.09	RW2
Kenya	CENTRAL	2002	67.48	53.39	84.92	RW2
Kenya	CENTRAL	2003	65.42	51.26	83.03	RW2
Kenya	CENTRAL	2004	63.27	48.89	81.57	RW2
Kenya	CENTRAL	2005	60.89	46.71	78.98	RW2
Kenya	CENTRAL	2006	58.55	45.02	75.81	RW2
Kenya	CENTRAL	2007	56.33	43.18	73.02	RW2
Kenya	CENTRAL	2008	54.39	41.03	71.52	RW2
Kenya	CENTRAL	2009	52.50	38.66	70.82	RW2
Kenya	CENTRAL	2010	50.87	36.91	70.09	RW2
Kenya	CENTRAL	2011	49.15	35.38	68.16	RW2
Kenya	CENTRAL	2012	47.62	33.97	66.58	RW2
Kenya	CENTRAL	2013	46.16	31.64	67.17	RW2
Kenya	CENTRAL	2014	44.77	28.02	70.95	RW2
Kenya	CENTRAL	2015	43.34	23.30	79.98	RW2
Kenya	CENTRAL	2016	42.01	18.78	91.04	RW2
Kenya	CENTRAL	2017	40.66	14.81	106.99	RW2
Kenya	CENTRAL	2018	39.39	11.47	128.77	RW2
Kenya	CENTRAL	2019	38.20	8.45	155.14	RW2
Kenya	COAST	1980	179.02	131.83	239.05	RW2
Kenya	COAST	1981	166.07	133.06	205.73	RW2
Kenya	COAST	1982	153.78	126.47	186.54	RW2
Kenya	COAST	1983	142.94	115.93	175.57	RW2
Kenya	COAST	1984	133.12	106.55	165.82	RW2
Kenya	COAST	1985	124.53	100.63	152.00	RW2
Kenya	COAST	1986	118.45	97.63	142.25	RW2
Kenya	COAST	1987	114.51	95.25	136.82	RW2
Kenya	COAST	1988	112.59	93.09	134.92	RW2
Kenya	COAST	1989	112.56	91.92	136.84	RW2
Kenya	COAST	1990	114.49	94.01	139.58	RW2
Kenya	COAST	1991	117.02	97.29	140.66	RW2
Kenya	COAST	1992	120.21	100.42	143.12	RW2
Kenya	COAST	1993	123.67	102.47	148.11	RW2
Kenya	COAST	1994	126.75	103.37	153.96	RW2
Kenya	COAST	1995	129.48	105.59	158.03	RW2
Kenya	COAST	1996	130.58	107.34	158.30	RW2
Kenya	COAST	1997	130.11	107.25	157.24	RW2
Kenya	COAST	1998	127.92	104.94	155.55	RW2

Continued on next page

Country	Region	Year	Median	Lower	Upper	Method
Kenya	COAST	1999	124.07	100.22	152.86	RW2
Kenya	COAST	2000	118.89	95.70	146.42	RW2
Kenya	COAST	2001	113.00	91.63	138.46	RW2
Kenya	COAST	2002	106.65	86.82	130.48	RW2
Kenya	COAST	2003	100.10	80.72	123.44	RW2
Kenya	COAST	2004	93.66	74.70	117.14	RW2
Kenya	COAST	2005	87.49	69.21	109.55	RW2
Kenya	COAST	2006	81.72	65.08	102.13	RW2
Kenya	COAST	2007	76.64	60.98	95.62	RW2
Kenya	COAST	2008	71.93	56.41	91.56	RW2
Kenya	COAST	2009	67.61	51.68	88.12	RW2
Kenya	COAST	2010	63.80	47.70	85.20	RW2
Kenya	COAST	2011	60.32	44.87	80.79	RW2
Kenya	COAST	2012	56.98	41.92	77.31	RW2
Kenya	COAST	2013	53.71	37.93	75.96	RW2
Kenya	COAST	2014	50.82	32.70	79.08	RW2
Kenya	COAST	2015	47.96	26.32	86.46	RW2
Kenya	COAST	2016	45.31	20.68	97.88	RW2
Kenya	COAST	2017	42.90	15.87	112.05	RW2
Kenya	COAST	2018	40.51	11.86	130.64	RW2
Kenya	COAST	2019	38.04	8.57	157.00	RW2
Kenya	EASTERN	1980	84.68	60.32	117.44	RW2
Kenya	EASTERN	1981	80.33	62.88	101.78	RW2
Kenya	EASTERN	1982	76.11	61.24	94.25	RW2
Kenya	EASTERN	1983	72.24	57.46	90.91	RW2
Kenya	EASTERN	1984	68.93	54.37	87.63	RW2
Kenya	EASTERN	1985	65.97	52.43	82.08	RW2
Kenya	EASTERN	1986	64.34	52.24	78.60	RW2
Kenya	EASTERN	1987	63.74	52.45	77.08	RW2
Kenya	EASTERN	1988	64.22	52.36	78.21	RW2
Kenya	EASTERN	1989	65.72	52.92	80.92	RW2
Kenya	EASTERN	1990	68.40	55.51	84.53	RW2
Kenya	EASTERN	1991	71.61	58.88	87.23	RW2
Kenya	EASTERN	1992	75.31	62.19	90.85	RW2
Kenya	EASTERN	1993	79.21	65.09	95.78	RW2
Kenya	EASTERN	1994	83.03	67.22	101.76	RW2
Kenya	EASTERN	1995	86.65	70.31	106.50	RW2
Kenya	EASTERN	1996	89.38	73.18	108.73	RW2
Kenya	EASTERN	1997	91.03	75.06	109.97	RW2
Kenya	EASTERN	1998	91.45	75.11	111.48	RW2
Kenya	EASTERN	1999	90.64	73.44	111.93	RW2
Kenya	EASTERN	2000	88.51	71.15	108.90	RW2
Kenya	EASTERN	2001	85.75	69.88	104.66	RW2
Kenya	EASTERN	2002	82.41	67.39	100.42	RW2
Kenya	EASTERN	2003	78.60	63.76	96.77	RW2
Kenya	EASTERN	2004	74.72	59.65	93.30	RW2
Kenya	EASTERN	2005	70.79	55.78	89.25	RW2
Kenya	EASTERN	2006	67.07	53.07	84.33	RW2
Kenya	EASTERN	2007	63.59	50.28	79.98	RW2
Kenya	EASTERN	2008	60.46	47.19	77.03	RW2
Kenya	EASTERN	2009	57.53	44.09	74.72	RW2
Kenya	EASTERN	2010	55.05	41.32	72.89	RW2
Kenya	EASTERN	2011	52.54	39.67	69.44	RW2
Kenya	EASTERN	2012	50.30	37.90	66.45	RW2
Kenya	EASTERN	2013	48.09	34.96	65.84	RW2
Kenya	EASTERN	2014	46.02	30.51	68.54	RW2
Kenya	EASTERN	2015	44.03	24.90	77.15	RW2
Kenya	EASTERN	2016	42.09	19.96	87.58	RW2
Kenya	EASTERN	2017	40.29	15.44	102.64	RW2
Kenya	EASTERN	2018	38.40	11.50	121.42	RW2
Kenya	EASTERN	2019	36.66	8.62	145.29	RW2
Kenya	NAIROBI	1980	73.47	46.09	115.15	RW2
Kenya	NAIROBI	1981	70.81	48.50	103.12	RW2

Continued on next page

Country	Region	Year	Median	Lower	Upper	Method
Kenya	NAIROBI	1982	68.29	48.39	95.85	RW2
Kenya	NAIROBI	1983	65.96	47.17	91.95	RW2
Kenya	NAIROBI	1984	64.04	46.26	88.61	RW2
Kenya	NAIROBI	1985	62.32	45.97	83.98	RW2
Kenya	NAIROBI	1986	61.80	46.64	81.36	RW2
Kenya	NAIROBI	1987	62.17	47.73	80.61	RW2
Kenya	NAIROBI	1988	63.53	48.83	82.32	RW2
Kenya	NAIROBI	1989	65.98	50.53	85.71	RW2
Kenya	NAIROBI	1990	69.50	53.68	90.23	RW2
Kenya	NAIROBI	1991	73.69	57.36	94.05	RW2
Kenya	NAIROBI	1992	78.37	61.42	99.30	RW2
Kenya	NAIROBI	1993	83.29	65.00	106.01	RW2
Kenya	NAIROBI	1994	88.33	68.18	113.48	RW2
Kenya	NAIROBI	1995	93.28	72.18	119.91	RW2
Kenya	NAIROBI	1996	97.19	75.58	124.00	RW2
Kenya	NAIROBI	1997	100.31	78.54	127.42	RW2
Kenya	NAIROBI	1998	102.15	79.60	130.49	RW2
Kenya	NAIROBI	1999	102.78	79.11	132.22	RW2
Kenya	NAIROBI	2000	101.92	78.12	131.34	RW2
Kenya	NAIROBI	2001	100.37	77.64	128.60	RW2
Kenya	NAIROBI	2002	98.13	76.06	125.24	RW2
Kenya	NAIROBI	2003	95.28	73.39	122.91	RW2
Kenya	NAIROBI	2004	92.29	70.25	120.74	RW2
Kenya	NAIROBI	2005	89.14	67.20	117.10	RW2
Kenya	NAIROBI	2006	86.04	65.03	112.84	RW2
Kenya	NAIROBI	2007	83.11	62.66	109.42	RW2
Kenya	NAIROBI	2008	80.42	59.67	107.37	RW2
Kenya	NAIROBI	2009	78.09	56.82	106.23	RW2
Kenya	NAIROBI	2010	76.01	54.37	105.40	RW2
Kenya	NAIROBI	2011	73.96	52.59	103.23	RW2
Kenya	NAIROBI	2012	72.12	50.71	101.61	RW2
Kenya	NAIROBI	2013	70.27	47.93	102.67	RW2
Kenya	NAIROBI	2014	68.41	42.81	108.28	RW2
Kenya	NAIROBI	2015	66.64	35.82	121.85	RW2
Kenya	NAIROBI	2016	64.85	29.14	138.56	RW2
Kenya	NAIROBI	2017	63.26	23.28	161.82	RW2
Kenya	NAIROBI	2018	61.39	17.97	191.07	RW2
Kenya	NAIROBI	2019	60.00	13.55	231.90	RW2
Kenya	NORTHEASTERN	1980	242.68	158.21	351.45	RW2
Kenya	NORTHEASTERN	1981	225.37	158.07	309.00	RW2
Kenya	NORTHEASTERN	1982	208.99	151.63	279.77	RW2
Kenya	NORTHEASTERN	1983	193.69	142.96	257.26	RW2
Kenya	NORTHEASTERN	1984	180.32	134.43	238.72	RW2
Kenya	NORTHEASTERN	1985	168.36	127.76	216.71	RW2
Kenya	NORTHEASTERN	1986	159.54	124.71	201.06	RW2
Kenya	NORTHEASTERN	1987	153.49	122.12	190.76	RW2
Kenya	NORTHEASTERN	1988	150.10	119.81	186.03	RW2
Kenya	NORTHEASTERN	1989	148.87	118.27	184.85	RW2
Kenya	NORTHEASTERN	1990	149.82	120.74	186.10	RW2
Kenya	NORTHEASTERN	1991	151.39	123.14	185.54	RW2
Kenya	NORTHEASTERN	1992	153.14	125.56	185.97	RW2
Kenya	NORTHEASTERN	1993	154.74	126.28	188.60	RW2
Kenya	NORTHEASTERN	1994	155.39	125.13	191.24	RW2
Kenya	NORTHEASTERN	1995	155.05	125.25	191.52	RW2
Kenya	NORTHEASTERN	1996	152.69	124.08	187.26	RW2
Kenya	NORTHEASTERN	1997	148.52	121.06	181.42	RW2
Kenya	NORTHEASTERN	1998	142.46	114.89	175.19	RW2
Kenya	NORTHEASTERN	1999	134.71	107.31	167.54	RW2
Kenya	NORTHEASTERN	2000	125.76	99.64	156.70	RW2
Kenya	NORTHEASTERN	2001	116.50	92.42	144.98	RW2
Kenya	NORTHEASTERN	2002	107.36	85.25	133.72	RW2
Kenya	NORTHEASTERN	2003	98.32	77.05	123.81	RW2
Kenya	NORTHEASTERN	2004	89.93	69.56	115.07	RW2

Continued on next page

Country	Region	Year	Median	Lower	Upper	Method
Kenya	NORTHEASTERN	2005	82.05	62.82	105.76	RW2
Kenya	NORTHEASTERN	2006	75.13	57.79	96.68	RW2
Kenya	NORTHEASTERN	2007	68.93	52.89	89.03	RW2
Kenya	NORTHEASTERN	2008	63.45	48.07	83.02	RW2
Kenya	NORTHEASTERN	2009	58.60	43.49	78.29	RW2
Kenya	NORTHEASTERN	2010	54.28	39.62	74.10	RW2
Kenya	NORTHEASTERN	2011	50.41	36.57	69.17	RW2
Kenya	NORTHEASTERN	2012	46.76	33.68	64.94	RW2
Kenya	NORTHEASTERN	2013	43.42	29.99	63.17	RW2
Kenya	NORTHEASTERN	2014	40.29	25.38	64.04	RW2
Kenya	NORTHEASTERN	2015	37.44	20.03	69.67	RW2
Kenya	NORTHEASTERN	2016	34.68	15.41	77.05	RW2
Kenya	NORTHEASTERN	2017	32.21	11.55	89.08	RW2
Kenya	NORTHEASTERN	2018	29.89	8.45	102.22	RW2
Kenya	NORTHEASTERN	2019	27.78	6.14	118.93	RW2
Kenya	NYANZA	1980	162.97	118.03	218.45	RW2
Kenya	NYANZA	1981	162.92	129.31	201.46	RW2
Kenya	NYANZA	1982	162.87	133.99	196.82	RW2
Kenya	NYANZA	1983	163.18	133.51	199.20	RW2
Kenya	NYANZA	1984	164.04	132.74	201.89	RW2
Kenya	NYANZA	1985	164.87	135.48	198.81	RW2
Kenya	NYANZA	1986	167.84	140.83	198.70	RW2
Kenya	NYANZA	1987	172.45	146.48	202.34	RW2
Kenya	NYANZA	1988	178.82	150.54	210.86	RW2
Kenya	NYANZA	1989	186.71	155.76	222.60	RW2
Kenya	NYANZA	1990	196.95	165.28	234.15	RW2
Kenya	NYANZA	1991	207.34	175.94	243.62	RW2
Kenya	NYANZA	1992	218.13	186.53	253.35	RW2
Kenya	NYANZA	1993	228.40	193.69	266.79	RW2
Kenya	NYANZA	1994	237.65	199.22	279.86	RW2
Kenya	NYANZA	1995	245.58	206.02	290.13	RW2
Kenya	NYANZA	1996	250.93	213.02	294.22	RW2
Kenya	NYANZA	1997	253.44	215.29	295.39	RW2
Kenya	NYANZA	1998	252.40	213.41	296.47	RW2
Kenya	NYANZA	1999	248.30	207.10	294.47	RW2
Kenya	NYANZA	2000	240.53	199.45	286.71	RW2
Kenya	NYANZA	2001	230.20	192.35	273.51	RW2
Kenya	NYANZA	2002	217.62	182.34	258.83	RW2
Kenya	NYANZA	2003	203.18	168.67	243.14	RW2
Kenya	NYANZA	2004	187.41	153.23	227.86	RW2
Kenya	NYANZA	2005	171.17	139.14	209.23	RW2
Kenya	NYANZA	2006	155.08	126.63	188.56	RW2
Kenya	NYANZA	2007	139.75	114.40	170.12	RW2
Kenya	NYANZA	2008	125.25	101.30	154.32	RW2
Kenya	NYANZA	2009	112.03	88.33	140.72	RW2
Kenya	NYANZA	2010	99.97	77.78	127.90	RW2
Kenya	NYANZA	2011	88.99	69.44	113.11	RW2
Kenya	NYANZA	2012	79.05	61.83	100.71	RW2
Kenya	NYANZA	2013	70.09	52.47	92.30	RW2
Kenya	NYANZA	2014	62.05	42.12	90.19	RW2
Kenya	NYANZA	2015	54.95	31.14	94.45	RW2
Kenya	NYANZA	2016	48.44	22.98	99.27	RW2
Kenya	NYANZA	2017	42.72	16.08	107.70	RW2
Kenya	NYANZA	2018	37.74	11.18	118.06	RW2
Kenya	NYANZA	2019	33.29	7.52	131.16	RW2
Kenya	RIFT VALLEY	1980	86.93	62.61	119.53	RW2
Kenya	RIFT VALLEY	1981	82.74	65.39	104.20	RW2
Kenya	RIFT VALLEY	1982	78.70	63.61	96.87	RW2
Kenya	RIFT VALLEY	1983	75.08	60.12	93.88	RW2
Kenya	RIFT VALLEY	1984	71.93	56.92	91.30	RW2
Kenya	RIFT VALLEY	1985	69.12	55.32	85.67	RW2
Kenya	RIFT VALLEY	1986	67.54	55.23	81.95	RW2
Kenya	RIFT VALLEY	1987	66.95	55.47	80.64	RW2

Continued on next page

Country	Region	Year	Median	Lower	Upper	Method
Kenya	RIFT VALLEY	1988	67.40	55.50	81.50	RW2
Kenya	RIFT VALLEY	1989	68.85	55.88	84.12	RW2
Kenya	RIFT VALLEY	1990	71.44	58.42	87.62	RW2
Kenya	RIFT VALLEY	1991	74.43	61.72	90.15	RW2
Kenya	RIFT VALLEY	1992	77.78	64.84	92.82	RW2
Kenya	RIFT VALLEY	1993	81.26	67.19	97.49	RW2
Kenya	RIFT VALLEY	1994	84.61	68.82	102.99	RW2
Kenya	RIFT VALLEY	1995	87.77	71.58	107.26	RW2
Kenya	RIFT VALLEY	1996	89.98	74.10	108.64	RW2
Kenya	RIFT VALLEY	1997	91.32	75.71	109.38	RW2
Kenya	RIFT VALLEY	1998	91.43	75.46	110.31	RW2
Kenya	RIFT VALLEY	1999	90.49	73.60	111.09	RW2
Kenya	RIFT VALLEY	2000	88.33	71.57	107.96	RW2
Kenya	RIFT VALLEY	2001	85.49	70.20	103.19	RW2
Kenya	RIFT VALLEY	2002	82.06	67.85	98.78	RW2
Kenya	RIFT VALLEY	2003	78.15	64.32	94.69	RW2
Kenya	RIFT VALLEY	2004	74.12	60.14	91.15	RW2
Kenya	RIFT VALLEY	2005	69.90	56.23	86.17	RW2
Kenya	RIFT VALLEY	2006	65.95	53.58	80.77	RW2
Kenya	RIFT VALLEY	2007	62.10	50.57	75.99	RW2
Kenya	RIFT VALLEY	2008	58.57	47.25	72.32	RW2
Kenya	RIFT VALLEY	2009	55.44	43.86	69.79	RW2
Kenya	RIFT VALLEY	2010	52.52	40.89	67.05	RW2
Kenya	RIFT VALLEY	2011	49.76	39.38	62.92	RW2
Kenya	RIFT VALLEY	2012	47.17	37.64	59.19	RW2
Kenya	RIFT VALLEY	2013	44.73	34.53	57.70	RW2
Kenya	RIFT VALLEY	2014	42.46	29.66	60.22	RW2
Kenya	RIFT VALLEY	2015	40.22	23.87	67.67	RW2
Kenya	RIFT VALLEY	2016	38.08	18.64	76.31	RW2
Kenya	RIFT VALLEY	2017	36.17	14.30	88.37	RW2
Kenya	RIFT VALLEY	2018	34.05	10.72	104.82	RW2
Kenya	RIFT VALLEY	2019	32.26	7.81	124.86	RW2
Kenya	WESTERN	1980	127.50	91.32	174.51	RW2
Kenya	WESTERN	1981	125.73	98.72	157.98	RW2
Kenya	WESTERN	1982	123.91	100.55	151.68	RW2
Kenya	WESTERN	1983	122.32	98.63	151.78	RW2
Kenya	WESTERN	1984	121.18	96.74	152.02	RW2
Kenya	WESTERN	1985	120.22	97.03	147.59	RW2
Kenya	WESTERN	1986	120.99	99.54	145.72	RW2
Kenya	WESTERN	1987	122.95	102.41	146.78	RW2
Kenya	WESTERN	1988	126.60	104.69	151.87	RW2
Kenya	WESTERN	1989	131.57	107.62	159.41	RW2
Kenya	WESTERN	1990	138.28	114.20	167.32	RW2
Kenya	WESTERN	1991	145.28	121.66	173.53	RW2
Kenya	WESTERN	1992	152.74	128.61	180.43	RW2
Kenya	WESTERN	1993	160.15	134.02	189.71	RW2
Kenya	WESTERN	1994	166.86	137.52	199.99	RW2
Kenya	WESTERN	1995	172.88	142.36	208.16	RW2
Kenya	WESTERN	1996	177.06	147.05	211.04	RW2
Kenya	WESTERN	1997	179.30	149.54	213.25	RW2
Kenya	WESTERN	1998	179.33	149.31	214.41	RW2
Kenya	WESTERN	1999	177.08	145.41	214.40	RW2
Kenya	WESTERN	2000	172.07	140.50	208.20	RW2
Kenya	WESTERN	2001	165.47	136.46	199.39	RW2
Kenya	WESTERN	2002	156.90	129.89	188.85	RW2
Kenya	WESTERN	2003	147.34	120.72	179.85	RW2
Kenya	WESTERN	2004	136.57	110.01	169.38	RW2
Kenya	WESTERN	2005	125.40	100.12	156.19	RW2
Kenya	WESTERN	2006	114.44	91.69	142.21	RW2
Kenya	WESTERN	2007	104.00	83.15	129.31	RW2
Kenya	WESTERN	2008	94.07	74.34	118.30	RW2
Kenya	WESTERN	2009	84.89	65.82	108.66	RW2
Kenya	WESTERN	2010	76.49	58.58	99.54	RW2

Continued on next page

Country	Region	Year	Median	Lower	Upper	Method
Kenya	WESTERN	2011	68.88	52.76	88.71	RW2
Kenya	WESTERN	2012	61.84	47.61	79.90	RW2
Kenya	WESTERN	2013	55.53	41.21	74.26	RW2
Kenya	WESTERN	2014	49.73	33.34	73.13	RW2
Kenya	WESTERN	2015	44.62	25.23	77.58	RW2
Kenya	WESTERN	2016	40.04	18.73	83.19	RW2
Kenya	WESTERN	2017	35.74	13.45	90.64	RW2
Kenya	WESTERN	2018	31.85	9.51	101.23	RW2
Kenya	WESTERN	2019	28.54	6.54	117.04	RW2
Lesotho	ALL	1980	99.98	93.97	106.25	IHME
Lesotho	ALL	1980	119.18	76.50	182.26	RW2
Lesotho	ALL	1980	120.50	109.00	133.40	UN
Lesotho	ALL	1981	99.25	93.58	105.31	IHME
Lesotho	ALL	1981	114.11	81.23	158.29	RW2
Lesotho	ALL	1981	114.90	103.50	127.10	UN
Lesotho	ALL	1982	98.60	92.83	104.73	IHME
Lesotho	ALL	1982	109.28	82.17	144.36	RW2
Lesotho	ALL	1982	109.60	98.60	121.30	UN
Lesotho	ALL	1983	98.17	92.47	103.97	IHME
Lesotho	ALL	1983	104.48	79.36	137.15	RW2
Lesotho	ALL	1983	105.00	94.30	116.20	UN
Lesotho	ALL	1984	97.41	91.79	102.98	IHME
Lesotho	ALL	1984	100.42	75.53	132.73	RW2
Lesotho	ALL	1984	100.90	90.80	111.60	UN
Lesotho	ALL	1985	96.39	91.11	101.71	IHME
Lesotho	ALL	1985	96.28	73.57	124.61	RW2
Lesotho	ALL	1985	97.40	87.80	107.70	UN
Lesotho	ALL	1986	95.57	90.37	100.64	IHME
Lesotho	ALL	1986	93.28	72.33	119.02	RW2
Lesotho	ALL	1986	94.40	85.30	104.20	UN
Lesotho	ALL	1987	94.71	89.55	99.71	IHME
Lesotho	ALL	1987	91.10	71.47	115.37	RW2
Lesotho	ALL	1987	92.10	83.40	101.40	UN
Lesotho	ALL	1988	93.63	88.65	98.39	IHME
Lesotho	ALL	1988	89.62	69.89	113.78	RW2
Lesotho	ALL	1988	90.20	81.80	99.20	UN
Lesotho	ALL	1989	92.51	87.54	97.44	IHME
Lesotho	ALL	1989	88.95	69.01	113.54	RW2
Lesotho	ALL	1989	88.90	80.80	97.50	UN
Lesotho	ALL	1990	91.58	86.94	96.68	IHME
Lesotho	ALL	1990	89.17	69.83	113.42	RW2
Lesotho	ALL	1990	88.10	80.20	96.40	UN
Lesotho	ALL	1991	90.83	86.17	95.88	IHME
Lesotho	ALL	1991	89.89	71.38	112.41	RW2
Lesotho	ALL	1991	87.90	80.30	96.10	UN
Lesotho	ALL	1992	90.23	85.61	95.11	IHME
Lesotho	ALL	1992	91.20	72.84	113.39	RW2
Lesotho	ALL	1992	88.70	81.10	96.90	UN
Lesotho	ALL	1993	90.26	85.71	95.01	IHME
Lesotho	ALL	1993	93.06	73.98	116.42	RW2
Lesotho	ALL	1993	90.80	83.10	99.20	UN
Lesotho	ALL	1994	90.92	86.45	95.56	IHME
Lesotho	ALL	1994	95.35	74.81	121.18	RW2
Lesotho	ALL	1994	94.30	86.50	102.90	UN
Lesotho	ALL	1995	92.62	88.15	97.08	IHME
Lesotho	ALL	1995	98.14	76.69	124.66	RW2
Lesotho	ALL	1995	98.90	90.80	107.80	UN
Lesotho	ALL	1996	94.44	89.87	99.15	IHME
Lesotho	ALL	1996	101.27	79.68	128.16	RW2
Lesotho	ALL	1996	103.70	95.10	112.80	UN
Lesotho	ALL	1997	96.78	92.23	101.67	IHME
Lesotho	ALL	1997	104.78	82.87	131.52	RW2
Lesotho	ALL	1997	107.90	99.10	117.20	UN

Continued on next page

Country	Region	Year	Median	Lower	Upper	Method
Lesotho	ALL	1998	99.45	94.77	104.68	IHME
Lesotho	ALL	1998	108.61	85.55	137.06	RW2
Lesotho	ALL	1998	111.40	102.50	120.70	UN
Lesotho	ALL	1999	102.15	97.35	107.35	IHME
Lesotho	ALL	1999	112.44	87.96	141.58	RW2
Lesotho	ALL	1999	114.30	105.50	123.60	UN
Lesotho	ALL	2000	106.34	101.16	111.92	IHME
Lesotho	ALL	2000	116.32	92.43	145.71	RW2
Lesotho	ALL	2000	116.80	108.00	126.20	UN
Lesotho	ALL	2001	110.11	104.76	116.03	IHME
Lesotho	ALL	2001	119.51	96.08	147.92	RW2
Lesotho	ALL	2001	118.60	109.80	128.10	UN
Lesotho	ALL	2002	112.79	107.27	118.88	IHME
Lesotho	ALL	2002	121.94	98.82	149.76	RW2
Lesotho	ALL	2002	120.40	111.50	130.10	UN
Lesotho	ALL	2003	114.97	109.16	121.27	IHME
Lesotho	ALL	2003	123.51	99.70	152.16	RW2
Lesotho	ALL	2003	122.00	112.90	132.00	UN
Lesotho	ALL	2004	116.97	110.73	123.56	IHME
Lesotho	ALL	2004	123.78	98.33	154.54	RW2
Lesotho	ALL	2004	123.00	113.80	133.30	UN
Lesotho	ALL	2005	119.61	112.74	126.78	IHME
Lesotho	ALL	2005	123.12	97.46	154.39	RW2
Lesotho	ALL	2005	123.40	113.80	134.20	UN
Lesotho	ALL	2006	120.46	113.06	128.57	IHME
Lesotho	ALL	2006	121.27	96.91	150.53	RW2
Lesotho	ALL	2006	123.20	113.10	134.60	UN
Lesotho	ALL	2007	117.42	109.53	126.29	IHME
Lesotho	ALL	2007	118.47	95.33	146.34	RW2
Lesotho	ALL	2007	119.30	108.80	131.20	UN
Lesotho	ALL	2008	114.75	106.16	124.25	IHME
Lesotho	ALL	2008	114.96	91.73	143.55	RW2
Lesotho	ALL	2008	116.50	105.20	129.20	UN
Lesotho	ALL	2009	110.40	101.16	120.67	IHME
Lesotho	ALL	2009	110.55	86.64	140.96	RW2
Lesotho	ALL	2009	106.50	95.10	119.30	UN
Lesotho	ALL	2010	104.63	94.58	116.38	IHME
Lesotho	ALL	2010	105.56	80.94	136.42	RW2
Lesotho	ALL	2010	100.70	88.80	114.60	UN
Lesotho	ALL	2011	97.41	86.76	109.66	IHME
Lesotho	ALL	2011	100.72	77.57	129.19	RW2
Lesotho	ALL	2011	96.80	83.70	112.00	UN
Lesotho	ALL	2012	95.67	84.35	108.98	IHME
Lesotho	ALL	2012	95.79	73.92	122.91	RW2
Lesotho	ALL	2012	94.10	79.40	111.30	UN
Lesotho	ALL	2013	98.87	85.59	113.95	IHME
Lesotho	ALL	2013	91.08	67.51	121.44	RW2
Lesotho	ALL	2013	93.60	77.00	113.30	UN
Lesotho	ALL	2014	100.84	86.18	117.34	IHME
Lesotho	ALL	2014	86.49	57.44	127.54	RW2
Lesotho	ALL	2014	92.00	73.60	114.10	UN
Lesotho	ALL	2015	83.67	70.34	99.44	IHME
Lesotho	ALL	2015	82.14	45.32	143.83	RW2
Lesotho	ALL	2015	90.20	70.20	115.00	UN
Lesotho	ALL	2016	78.22	35.40	163.54	RW2
Lesotho	ALL	2017	74.07	26.54	189.19	RW2
Lesotho	ALL	2018	70.28	19.66	223.41	RW2
Lesotho	ALL	2019	66.57	13.64	260.96	RW2
Lesotho	ALL	15-19	74.10	26.93	185.85	RW2
Lesotho	BEREA	1980	176.42	109.38	272.98	RW2
Lesotho	BEREA	1981	164.14	110.05	239.18	RW2
Lesotho	BEREA	1982	153.07	106.38	215.22	RW2
Lesotho	BEREA	1983	142.50	101.00	198.69	RW2

Continued on next page

Country	Region	Year	Median	Lower	Upper	Method
Lesotho	BEREA	1984	132.95	94.59	184.12	RW2
Lesotho	BEREA	1985	124.28	89.52	169.21	RW2
Lesotho	BEREA	1986	117.36	86.17	157.69	RW2
Lesotho	BEREA	1987	112.02	83.13	148.54	RW2
Lesotho	BEREA	1988	108.12	80.93	142.64	RW2
Lesotho	BEREA	1989	105.41	79.00	138.77	RW2
Lesotho	BEREA	1990	103.94	79.23	135.17	RW2
Lesotho	BEREA	1991	103.33	80.35	132.13	RW2
Lesotho	BEREA	1992	103.35	80.94	130.64	RW2
Lesotho	BEREA	1993	103.96	81.71	131.30	RW2
Lesotho	BEREA	1994	104.73	81.48	133.19	RW2
Lesotho	BEREA	1995	106.08	82.51	135.21	RW2
Lesotho	BEREA	1996	107.10	84.13	135.47	RW2
Lesotho	BEREA	1997	108.09	85.20	136.01	RW2
Lesotho	BEREA	1998	108.81	85.83	137.37	RW2
Lesotho	BEREA	1999	109.29	86.00	138.16	RW2
Lesotho	BEREA	2000	109.38	86.44	137.31	RW2
Lesotho	BEREA	2001	109.47	87.57	135.93	RW2
Lesotho	BEREA	2002	109.31	88.09	135.20	RW2
Lesotho	BEREA	2003	108.99	86.64	136.02	RW2
Lesotho	BEREA	2004	108.29	85.18	136.67	RW2
Lesotho	BEREA	2005	107.23	83.88	136.71	RW2
Lesotho	BEREA	2006	105.09	82.43	133.65	RW2
Lesotho	BEREA	2007	101.90	79.53	130.00	RW2
Lesotho	BEREA	2008	97.67	74.91	126.55	RW2
Lesotho	BEREA	2009	92.76	69.41	123.22	RW2
Lesotho	BEREA	2010	86.80	63.27	117.86	RW2
Lesotho	BEREA	2011	81.25	58.70	110.86	RW2
Lesotho	BEREA	2012	75.68	53.97	105.01	RW2
Lesotho	BEREA	2013	70.43	48.13	101.75	RW2
Lesotho	BEREA	2014	65.43	40.88	103.04	RW2
Lesotho	BEREA	2015	60.84	32.49	110.55	RW2
Lesotho	BEREA	2016	56.51	25.20	119.84	RW2
Lesotho	BEREA	2017	52.42	19.06	133.54	RW2
Lesotho	BEREA	2018	48.62	13.83	152.47	RW2
Lesotho	BEREA	2019	45.22	9.91	176.24	RW2
Lesotho	BUTHA-BUTHE	1980	101.59	55.42	179.56	RW2
Lesotho	BUTHA-BUTHE	1981	96.15	57.30	157.29	RW2
Lesotho	BUTHA-BUTHE	1982	90.79	57.14	141.94	RW2
Lesotho	BUTHA-BUTHE	1983	86.35	55.70	131.20	RW2
Lesotho	BUTHA-BUTHE	1984	81.90	53.89	122.37	RW2
Lesotho	BUTHA-BUTHE	1985	78.03	52.72	113.20	RW2
Lesotho	BUTHA-BUTHE	1986	75.07	52.16	106.33	RW2
Lesotho	BUTHA-BUTHE	1987	73.02	51.85	101.74	RW2
Lesotho	BUTHA-BUTHE	1988	71.78	51.79	98.29	RW2
Lesotho	BUTHA-BUTHE	1989	71.43	52.05	97.29	RW2
Lesotho	BUTHA-BUTHE	1990	71.94	53.32	96.59	RW2
Lesotho	BUTHA-BUTHE	1991	72.81	55.05	95.94	RW2
Lesotho	BUTHA-BUTHE	1992	74.19	56.78	96.35	RW2
Lesotho	BUTHA-BUTHE	1993	76.04	58.28	98.58	RW2
Lesotho	BUTHA-BUTHE	1994	78.07	59.42	101.92	RW2
Lesotho	BUTHA-BUTHE	1995	80.51	61.27	105.12	RW2
Lesotho	BUTHA-BUTHE	1996	82.76	63.48	107.41	RW2
Lesotho	BUTHA-BUTHE	1997	84.95	65.48	109.69	RW2
Lesotho	BUTHA-BUTHE	1998	86.98	67.25	112.18	RW2
Lesotho	BUTHA-BUTHE	1999	88.81	68.27	114.85	RW2
Lesotho	BUTHA-BUTHE	2000	90.47	70.14	115.81	RW2
Lesotho	BUTHA-BUTHE	2001	92.13	72.24	116.81	RW2
Lesotho	BUTHA-BUTHE	2002	93.71	73.86	118.31	RW2
Lesotho	BUTHA-BUTHE	2003	95.07	74.25	120.65	RW2
Lesotho	BUTHA-BUTHE	2004	96.11	74.25	123.41	RW2
Lesotho	BUTHA-BUTHE	2005	97.09	74.67	125.40	RW2
Lesotho	BUTHA-BUTHE	2006	96.73	74.51	125.07	RW2

Continued on next page

Country	Region	Year	Median	Lower	Upper	Method
Lesotho	BUTHA-BUTHE	2007	95.74	73.25	123.93	RW2
Lesotho	BUTHA-BUTHE	2008	93.58	70.37	123.96	RW2
Lesotho	BUTHA-BUTHE	2009	90.46	66.19	122.97	RW2
Lesotho	BUTHA-BUTHE	2010	86.40	61.23	120.30	RW2
Lesotho	BUTHA-BUTHE	2011	82.59	57.69	116.38	RW2
Lesotho	BUTHA-BUTHE	2012	78.51	53.72	113.21	RW2
Lesotho	BUTHA-BUTHE	2013	74.30	48.62	111.99	RW2
Lesotho	BUTHA-BUTHE	2014	70.62	42.25	116.13	RW2
Lesotho	BUTHA-BUTHE	2015	67.06	34.51	125.71	RW2
Lesotho	BUTHA-BUTHE	2016	63.68	27.35	140.45	RW2
Lesotho	BUTHA-BUTHE	2017	60.60	21.16	158.54	RW2
Lesotho	BUTHA-BUTHE	2018	57.52	15.90	182.10	RW2
Lesotho	BUTHA-BUTHE	2019	54.28	11.53	215.07	RW2
Lesotho	LERIBE	1980	112.47	64.84	188.73	RW2
Lesotho	LERIBE	1981	107.02	67.25	166.31	RW2
Lesotho	LERIBE	1982	101.87	67.70	151.78	RW2
Lesotho	LERIBE	1983	97.22	66.36	141.51	RW2
Lesotho	LERIBE	1984	93.09	64.26	133.08	RW2
Lesotho	LERIBE	1985	89.12	63.19	124.43	RW2
Lesotho	LERIBE	1986	86.41	62.64	117.92	RW2
Lesotho	LERIBE	1987	84.65	62.58	113.53	RW2
Lesotho	LERIBE	1988	83.84	62.36	111.41	RW2
Lesotho	LERIBE	1989	83.81	62.94	111.07	RW2
Lesotho	LERIBE	1990	85.06	64.75	110.89	RW2
Lesotho	LERIBE	1991	86.78	67.12	111.60	RW2
Lesotho	LERIBE	1992	89.23	69.91	113.08	RW2
Lesotho	LERIBE	1993	92.09	71.99	117.07	RW2
Lesotho	LERIBE	1994	95.38	74.11	121.84	RW2
Lesotho	LERIBE	1995	99.06	76.91	126.75	RW2
Lesotho	LERIBE	1996	102.85	80.85	130.67	RW2
Lesotho	LERIBE	1997	106.64	84.00	134.26	RW2
Lesotho	LERIBE	1998	110.11	86.95	138.85	RW2
Lesotho	LERIBE	1999	113.58	89.28	143.11	RW2
Lesotho	LERIBE	2000	116.64	92.33	146.03	RW2
Lesotho	LERIBE	2001	119.77	96.22	148.13	RW2
Lesotho	LERIBE	2002	122.92	99.40	151.20	RW2
Lesotho	LERIBE	2003	125.86	100.99	155.01	RW2
Lesotho	LERIBE	2004	128.28	101.51	160.08	RW2
Lesotho	LERIBE	2005	130.65	103.50	164.34	RW2
Lesotho	LERIBE	2006	131.37	104.37	164.21	RW2
Lesotho	LERIBE	2007	130.91	103.99	164.02	RW2
Lesotho	LERIBE	2008	128.95	101.13	163.87	RW2
Lesotho	LERIBE	2009	126.01	96.24	163.56	RW2
Lesotho	LERIBE	2010	121.52	90.71	160.50	RW2
Lesotho	LERIBE	2011	117.11	86.84	155.25	RW2
Lesotho	LERIBE	2012	112.39	82.81	151.34	RW2
Lesotho	LERIBE	2013	107.63	75.94	150.08	RW2
Lesotho	LERIBE	2014	103.04	66.65	156.11	RW2
Lesotho	LERIBE	2015	99.00	54.36	172.40	RW2
Lesotho	LERIBE	2016	94.60	43.99	190.38	RW2
Lesotho	LERIBE	2017	90.53	33.72	216.02	RW2
Lesotho	LERIBE	2018	86.81	25.64	246.62	RW2
Lesotho	LERIBE	2019	83.16	18.85	283.56	RW2
Lesotho	MAFETENG	1980	107.51	59.17	188.87	RW2
Lesotho	MAFETENG	1981	102.49	61.77	165.73	RW2
Lesotho	MAFETENG	1982	97.54	61.87	150.77	RW2
Lesotho	MAFETENG	1983	92.82	60.56	140.43	RW2
Lesotho	MAFETENG	1984	88.64	59.19	131.26	RW2
Lesotho	MAFETENG	1985	84.74	57.76	122.17	RW2
Lesotho	MAFETENG	1986	82.06	57.36	115.56	RW2
Lesotho	MAFETENG	1987	80.22	57.42	110.73	RW2
Lesotho	MAFETENG	1988	79.26	57.25	108.39	RW2
Lesotho	MAFETENG	1989	79.18	57.64	107.49	RW2

Continued on next page

Country	Region	Year	Median	Lower	Upper	Method
Lesotho	MAFETENG	1990	80.04	59.28	107.56	RW2
Lesotho	MAFETENG	1991	81.53	61.43	107.75	RW2
Lesotho	MAFETENG	1992	83.60	63.55	109.11	RW2
Lesotho	MAFETENG	1993	86.08	65.72	111.82	RW2
Lesotho	MAFETENG	1994	88.91	67.47	116.19	RW2
Lesotho	MAFETENG	1995	92.14	70.03	120.21	RW2
Lesotho	MAFETENG	1996	95.40	73.03	123.41	RW2
Lesotho	MAFETENG	1997	98.61	76.15	126.47	RW2
Lesotho	MAFETENG	1998	101.70	78.86	130.61	RW2
Lesotho	MAFETENG	1999	104.67	80.95	134.60	RW2
Lesotho	MAFETENG	2000	107.25	83.33	136.09	RW2
Lesotho	MAFETENG	2001	110.02	87.04	137.83	RW2
Lesotho	MAFETENG	2002	112.76	89.80	140.40	RW2
Lesotho	MAFETENG	2003	115.16	91.37	144.00	RW2
Lesotho	MAFETENG	2004	117.34	92.08	147.83	RW2
Lesotho	MAFETENG	2005	119.35	93.38	151.73	RW2
Lesotho	MAFETENG	2006	119.88	94.11	151.79	RW2
Lesotho	MAFETENG	2007	119.17	93.20	151.12	RW2
Lesotho	MAFETENG	2008	117.30	90.21	151.22	RW2
Lesotho	MAFETENG	2009	114.11	85.64	150.94	RW2
Lesotho	MAFETENG	2010	109.89	79.31	149.24	RW2
Lesotho	MAFETENG	2011	105.29	75.07	145.68	RW2
Lesotho	MAFETENG	2012	100.80	70.15	142.65	RW2
Lesotho	MAFETENG	2013	96.13	63.57	143.01	RW2
Lesotho	MAFETENG	2014	91.75	55.20	147.52	RW2
Lesotho	MAFETENG	2015	87.75	45.43	162.50	RW2
Lesotho	MAFETENG	2016	83.72	36.48	179.66	RW2
Lesotho	MAFETENG	2017	79.98	28.21	204.26	RW2
Lesotho	MAFETENG	2018	76.05	20.96	233.87	RW2
Lesotho	MAFETENG	2019	72.45	15.67	270.02	RW2
Lesotho	MASERU	1980	81.64	47.11	137.81	RW2
Lesotho	MASERU	1981	78.84	49.72	122.38	RW2
Lesotho	MASERU	1982	76.12	50.26	113.30	RW2
Lesotho	MASERU	1983	73.46	49.82	107.17	RW2
Lesotho	MASERU	1984	71.22	48.97	102.85	RW2
Lesotho	MASERU	1985	69.26	48.41	97.14	RW2
Lesotho	MASERU	1986	67.98	48.82	93.42	RW2
Lesotho	MASERU	1987	67.46	49.32	91.31	RW2
Lesotho	MASERU	1988	67.78	50.04	90.87	RW2
Lesotho	MASERU	1989	68.84	51.12	91.52	RW2
Lesotho	MASERU	1990	70.73	53.76	92.99	RW2
Lesotho	MASERU	1991	73.22	56.53	94.41	RW2
Lesotho	MASERU	1992	76.20	59.73	96.73	RW2
Lesotho	MASERU	1993	79.74	62.70	100.95	RW2
Lesotho	MASERU	1994	83.59	65.31	106.34	RW2
Lesotho	MASERU	1995	87.86	68.98	111.73	RW2
Lesotho	MASERU	1996	92.18	72.98	116.16	RW2
Lesotho	MASERU	1997	96.59	77.05	120.82	RW2
Lesotho	MASERU	1998	100.92	80.27	126.25	RW2
Lesotho	MASERU	1999	105.04	83.38	131.62	RW2
Lesotho	MASERU	2000	108.93	87.22	135.15	RW2
Lesotho	MASERU	2001	112.87	91.34	138.68	RW2
Lesotho	MASERU	2002	116.86	95.20	142.86	RW2
Lesotho	MASERU	2003	120.47	97.01	148.26	RW2
Lesotho	MASERU	2004	123.78	98.37	154.32	RW2
Lesotho	MASERU	2005	126.76	100.27	159.59	RW2
Lesotho	MASERU	2006	128.37	101.88	161.03	RW2
Lesotho	MASERU	2007	128.66	101.61	161.82	RW2
Lesotho	MASERU	2008	127.61	99.31	162.61	RW2
Lesotho	MASERU	2009	125.31	95.41	163.08	RW2
Lesotho	MASERU	2010	121.42	90.30	160.75	RW2
Lesotho	MASERU	2011	117.71	86.90	156.71	RW2
Lesotho	MASERU	2012	113.52	83.22	152.72	RW2

Continued on next page

Country	Region	Year	Median	Lower	Upper	Method
Lesotho	MASERU	2013	109.35	77.13	152.98	RW2
Lesotho	MASERU	2014	105.26	68.08	158.51	RW2
Lesotho	MASERU	2015	101.64	56.28	175.20	RW2
Lesotho	MASERU	2016	97.76	45.19	195.31	RW2
Lesotho	MASERU	2017	94.25	35.34	225.47	RW2
Lesotho	MASERU	2018	90.82	26.94	257.80	RW2
Lesotho	MASERU	2019	87.62	20.40	296.72	RW2
Lesotho	MOHALE'S HOEK	1980	146.72	89.12	233.24	RW2
Lesotho	MOHALE'S HOEK	1981	139.03	91.82	205.19	RW2
Lesotho	MOHALE'S HOEK	1982	131.96	91.03	187.54	RW2
Lesotho	MOHALE'S HOEK	1983	124.89	88.03	175.11	RW2
Lesotho	MOHALE'S HOEK	1984	118.78	84.98	164.53	RW2
Lesotho	MOHALE'S HOEK	1985	113.11	82.09	153.47	RW2
Lesotho	MOHALE'S HOEK	1986	108.88	80.55	145.09	RW2
Lesotho	MOHALE'S HOEK	1987	105.98	79.93	139.59	RW2
Lesotho	MOHALE'S HOEK	1988	104.18	78.85	136.39	RW2
Lesotho	MOHALE'S HOEK	1989	103.55	78.55	134.83	RW2
Lesotho	MOHALE'S HOEK	1990	104.21	80.43	134.01	RW2
Lesotho	MOHALE'S HOEK	1991	105.52	82.81	133.86	RW2
Lesotho	MOHALE'S HOEK	1992	107.51	85.46	134.68	RW2
Lesotho	MOHALE'S HOEK	1993	110.11	87.15	138.13	RW2
Lesotho	MOHALE'S HOEK	1994	113.11	89.11	142.36	RW2
Lesotho	MOHALE'S HOEK	1995	116.50	91.78	147.06	RW2
Lesotho	MOHALE'S HOEK	1996	119.89	95.43	150.28	RW2
Lesotho	MOHALE'S HOEK	1997	123.07	98.51	153.35	RW2
Lesotho	MOHALE'S HOEK	1998	126.19	100.78	157.25	RW2
Lesotho	MOHALE'S HOEK	1999	129.02	102.56	161.58	RW2
Lesotho	MOHALE'S HOEK	2000	131.16	104.75	163.09	RW2
Lesotho	MOHALE'S HOEK	2001	133.44	107.67	164.25	RW2
Lesotho	MOHALE'S HOEK	2002	135.63	109.99	166.40	RW2
Lesotho	MOHALE'S HOEK	2003	137.36	110.23	169.22	RW2
Lesotho	MOHALE'S HOEK	2004	138.72	109.85	173.12	RW2
Lesotho	MOHALE'S HOEK	2005	139.70	110.44	175.81	RW2
Lesotho	MOHALE'S HOEK	2006	139.26	110.28	174.28	RW2
Lesotho	MOHALE'S HOEK	2007	137.12	108.48	172.31	RW2
Lesotho	MOHALE'S HOEK	2008	133.85	104.07	170.45	RW2
Lesotho	MOHALE'S HOEK	2009	129.10	98.03	168.67	RW2
Lesotho	MOHALE'S HOEK	2010	123.23	90.90	164.18	RW2
Lesotho	MOHALE'S HOEK	2011	117.14	85.83	156.87	RW2
Lesotho	MOHALE'S HOEK	2012	111.02	80.57	151.56	RW2
Lesotho	MOHALE'S HOEK	2013	105.09	72.91	148.95	RW2
Lesotho	MOHALE'S HOEK	2014	99.38	62.83	152.44	RW2
Lesotho	MOHALE'S HOEK	2015	94.12	51.24	164.75	RW2
Lesotho	MOHALE'S HOEK	2016	89.00	40.30	181.43	RW2
Lesotho	MOHALE'S HOEK	2017	84.06	31.09	203.61	RW2
Lesotho	MOHALE'S HOEK	2018	79.16	23.56	229.95	RW2
Lesotho	MOHALE'S HOEK	2019	74.93	17.11	265.20	RW2
Lesotho	MOKHOTLONG	1980	167.71	97.59	273.12	RW2
Lesotho	MOKHOTLONG	1981	157.74	99.90	239.73	RW2
Lesotho	MOKHOTLONG	1982	148.13	97.84	216.51	RW2
Lesotho	MOKHOTLONG	1983	139.43	94.93	200.12	RW2
Lesotho	MOKHOTLONG	1984	131.49	90.96	186.64	RW2
Lesotho	MOKHOTLONG	1985	124.18	87.85	172.10	RW2
Lesotho	MOKHOTLONG	1986	118.50	85.77	160.45	RW2
Lesotho	MOKHOTLONG	1987	114.14	84.40	152.33	RW2
Lesotho	MOKHOTLONG	1988	111.05	83.19	146.42	RW2
Lesotho	MOKHOTLONG	1989	109.24	82.36	142.90	RW2
Lesotho	MOKHOTLONG	1990	108.76	83.49	140.57	RW2
Lesotho	MOKHOTLONG	1991	108.92	85.31	138.70	RW2
Lesotho	MOKHOTLONG	1992	109.70	86.91	137.34	RW2
Lesotho	MOKHOTLONG	1993	110.93	87.89	138.94	RW2
Lesotho	MOKHOTLONG	1994	112.51	88.32	142.24	RW2
Lesotho	MOKHOTLONG	1995	114.47	89.85	145.08	RW2

Continued on next page

Country	Region	Year	Median	Lower	Upper	Method
Lesotho	MOKHOTLONG	1996	116.19	91.76	146.14	RW2
Lesotho	MOKHOTLONG	1997	117.89	93.63	147.29	RW2
Lesotho	MOKHOTLONG	1998	119.13	94.64	149.20	RW2
Lesotho	MOKHOTLONG	1999	120.24	94.83	151.87	RW2
Lesotho	MOKHOTLONG	2000	120.83	95.82	150.92	RW2
Lesotho	MOKHOTLONG	2001	121.46	97.59	149.68	RW2
Lesotho	MOKHOTLONG	2002	121.99	98.45	149.99	RW2
Lesotho	MOKHOTLONG	2003	122.22	97.95	151.11	RW2
Lesotho	MOKHOTLONG	2004	122.21	96.45	153.14	RW2
Lesotho	MOKHOTLONG	2005	121.85	95.62	154.12	RW2
Lesotho	MOKHOTLONG	2006	120.24	94.56	151.86	RW2
Lesotho	MOKHOTLONG	2007	117.25	91.75	148.82	RW2
Lesotho	MOKHOTLONG	2008	113.24	87.49	145.60	RW2
Lesotho	MOKHOTLONG	2009	108.57	82.04	142.77	RW2
Lesotho	MOKHOTLONG	2010	102.56	75.29	137.08	RW2
Lesotho	MOKHOTLONG	2011	96.77	71.03	130.48	RW2
Lesotho	MOKHOTLONG	2012	90.97	65.89	124.58	RW2
Lesotho	MOKHOTLONG	2013	85.37	59.26	121.26	RW2
Lesotho	MOKHOTLONG	2014	80.19	50.72	123.52	RW2
Lesotho	MOKHOTLONG	2015	75.27	41.12	134.14	RW2
Lesotho	MOKHOTLONG	2016	70.54	31.96	146.21	RW2
Lesotho	MOKHOTLONG	2017	66.29	24.32	163.65	RW2
Lesotho	MOKHOTLONG	2018	61.72	18.05	187.36	RW2
Lesotho	MOKHOTLONG	2019	57.84	12.99	215.34	RW2
Lesotho	QASHA'S NEK	1980	177.29	105.95	286.35	RW2
Lesotho	QASHA'S NEK	1981	166.47	108.06	250.28	RW2
Lesotho	QASHA'S NEK	1982	155.77	105.47	225.82	RW2
Lesotho	QASHA'S NEK	1983	145.97	100.82	208.19	RW2
Lesotho	QASHA'S NEK	1984	137.11	95.92	193.28	RW2
Lesotho	QASHA'S NEK	1985	129.00	92.10	177.92	RW2
Lesotho	QASHA'S NEK	1986	122.62	89.25	165.33	RW2
Lesotho	QASHA'S NEK	1987	117.60	87.27	156.60	RW2
Lesotho	QASHA'S NEK	1988	113.97	85.34	150.02	RW2
Lesotho	QASHA'S NEK	1989	111.67	84.55	145.89	RW2
Lesotho	QASHA'S NEK	1990	110.91	85.08	143.24	RW2
Lesotho	QASHA'S NEK	1991	110.78	86.57	140.56	RW2
Lesotho	QASHA'S NEK	1992	111.35	87.95	139.98	RW2
Lesotho	QASHA'S NEK	1993	112.48	89.00	141.22	RW2
Lesotho	QASHA'S NEK	1994	114.02	89.79	143.95	RW2
Lesotho	QASHA'S NEK	1995	116.06	90.81	146.73	RW2
Lesotho	QASHA'S NEK	1996	117.83	93.26	148.23	RW2
Lesotho	QASHA'S NEK	1997	119.65	94.81	149.45	RW2
Lesotho	QASHA'S NEK	1998	121.05	95.69	151.62	RW2
Lesotho	QASHA'S NEK	1999	122.39	96.51	154.30	RW2
Lesotho	QASHA'S NEK	2000	123.22	96.89	154.22	RW2
Lesotho	QASHA'S NEK	2001	124.23	98.86	154.50	RW2
Lesotho	QASHA'S NEK	2002	125.00	99.98	155.06	RW2
Lesotho	QASHA'S NEK	2003	125.59	99.69	156.88	RW2
Lesotho	QASHA'S NEK	2004	125.81	98.23	159.05	RW2
Lesotho	QASHA'S NEK	2005	125.73	98.11	160.27	RW2
Lesotho	QASHA'S NEK	2006	124.31	97.08	158.07	RW2
Lesotho	QASHA'S NEK	2007	121.63	94.52	155.55	RW2
Lesotho	QASHA'S NEK	2008	117.76	90.07	152.82	RW2
Lesotho	QASHA'S NEK	2009	112.76	83.93	150.52	RW2
Lesotho	QASHA'S NEK	2010	106.75	76.85	145.64	RW2
Lesotho	QASHA'S NEK	2011	100.84	71.79	139.51	RW2
Lesotho	QASHA'S NEK	2012	94.79	66.22	134.18	RW2
Lesotho	QASHA'S NEK	2013	88.91	59.59	131.31	RW2
Lesotho	QASHA'S NEK	2014	83.37	51.18	133.41	RW2
Lesotho	QASHA'S NEK	2015	78.38	41.01	143.69	RW2
Lesotho	QASHA'S NEK	2016	73.70	32.48	156.70	RW2
Lesotho	QASHA'S NEK	2017	68.85	24.67	176.39	RW2
Lesotho	QASHA'S NEK	2018	64.64	18.37	199.01	RW2

Continued on next page

Country	Region	Year	Median	Lower	Upper	Method
Lesotho	QASHA'S NEK	2019	60.65	13.38	231.75	RW2
Lesotho	QUTHING	1980	169.18	101.06	269.52	RW2
Lesotho	QUTHING	1981	159.46	103.71	237.55	RW2
Lesotho	QUTHING	1982	150.18	101.74	214.76	RW2
Lesotho	QUTHING	1983	141.36	97.85	199.56	RW2
Lesotho	QUTHING	1984	133.51	93.73	187.29	RW2
Lesotho	QUTHING	1985	126.10	89.84	173.37	RW2
Lesotho	QUTHING	1986	120.50	87.78	163.20	RW2
Lesotho	QUTHING	1987	116.31	86.16	155.31	RW2
Lesotho	QUTHING	1988	113.39	84.67	149.71	RW2
Lesotho	QUTHING	1989	111.74	84.19	146.88	RW2
Lesotho	QUTHING	1990	111.49	85.32	144.10	RW2
Lesotho	QUTHING	1991	112.00	87.43	142.54	RW2
Lesotho	QUTHING	1992	112.98	89.27	141.62	RW2
Lesotho	QUTHING	1993	114.62	90.57	144.06	RW2
Lesotho	QUTHING	1994	116.68	91.46	147.57	RW2
Lesotho	QUTHING	1995	118.88	92.80	151.19	RW2
Lesotho	QUTHING	1996	121.14	95.24	152.81	RW2
Lesotho	QUTHING	1997	122.95	96.96	154.93	RW2
Lesotho	QUTHING	1998	124.53	98.02	157.17	RW2
Lesotho	QUTHING	1999	126.06	98.85	159.99	RW2
Lesotho	QUTHING	2000	126.87	99.83	159.70	RW2
Lesotho	QUTHING	2001	127.72	101.84	159.24	RW2
Lesotho	QUTHING	2002	128.49	102.30	159.84	RW2
Lesotho	QUTHING	2003	128.90	101.85	161.83	RW2
Lesotho	QUTHING	2004	128.97	100.40	163.51	RW2
Lesotho	QUTHING	2005	128.59	99.94	164.50	RW2
Lesotho	QUTHING	2006	126.99	98.73	162.38	RW2
Lesotho	QUTHING	2007	123.86	95.67	158.51	RW2
Lesotho	QUTHING	2008	119.53	90.69	156.32	RW2
Lesotho	QUTHING	2009	114.42	84.44	153.46	RW2
Lesotho	QUTHING	2010	107.97	77.16	148.49	RW2
Lesotho	QUTHING	2011	101.76	71.51	141.93	RW2
Lesotho	QUTHING	2012	95.38	65.61	136.32	RW2
Lesotho	QUTHING	2013	89.34	58.62	133.46	RW2
Lesotho	QUTHING	2014	83.61	49.87	135.35	RW2
Lesotho	QUTHING	2015	78.32	40.17	145.87	RW2
Lesotho	QUTHING	2016	73.10	31.59	158.82	RW2
Lesotho	QUTHING	2017	68.52	23.79	176.86	RW2
Lesotho	QUTHING	2018	63.85	17.55	199.14	RW2
Lesotho	QUTHING	2019	59.58	12.82	229.13	RW2
Lesotho	THABA-TSEKA	1980	190.40	114.40	301.20	RW2
Lesotho	THABA-TSEKA	1981	177.98	115.23	264.43	RW2
Lesotho	THABA-TSEKA	1982	166.12	112.36	238.50	RW2
Lesotho	THABA-TSEKA	1983	155.07	107.06	220.42	RW2
Lesotho	THABA-TSEKA	1984	145.02	101.09	204.48	RW2
Lesotho	THABA-TSEKA	1985	135.82	96.29	187.82	RW2
Lesotho	THABA-TSEKA	1986	128.60	92.75	174.55	RW2
Lesotho	THABA-TSEKA	1987	122.61	90.06	164.29	RW2
Lesotho	THABA-TSEKA	1988	118.53	87.68	157.51	RW2
Lesotho	THABA-TSEKA	1989	115.62	86.13	152.97	RW2
Lesotho	THABA-TSEKA	1990	114.15	86.59	149.00	RW2
Lesotho	THABA-TSEKA	1991	113.32	87.64	145.77	RW2
Lesotho	THABA-TSEKA	1992	113.30	88.71	143.63	RW2
Lesotho	THABA-TSEKA	1993	113.84	89.50	143.72	RW2
Lesotho	THABA-TSEKA	1994	114.62	89.61	145.39	RW2
Lesotho	THABA-TSEKA	1995	115.75	90.61	146.79	RW2
Lesotho	THABA-TSEKA	1996	116.73	92.28	146.47	RW2
Lesotho	THABA-TSEKA	1997	117.51	93.54	146.89	RW2
Lesotho	THABA-TSEKA	1998	118.04	94.37	147.40	RW2
Lesotho	THABA-TSEKA	1999	118.29	93.93	148.35	RW2
Lesotho	THABA-TSEKA	2000	117.89	93.95	146.46	RW2
Lesotho	THABA-TSEKA	2001	117.63	94.99	144.78	RW2

Continued on next page

Country	Region	Year	Median	Lower	Upper	Method
Lesotho	THABA-TSEKA	2002	117.00	94.92	143.30	RW2
Lesotho	THABA-TSEKA	2003	116.38	93.40	144.14	RW2
Lesotho	THABA-TSEKA	2004	115.03	90.74	144.44	RW2
Lesotho	THABA-TSEKA	2005	113.54	89.33	143.63	RW2
Lesotho	THABA-TSEKA	2006	110.71	87.07	139.92	RW2
Lesotho	THABA-TSEKA	2007	106.95	83.40	135.91	RW2
Lesotho	THABA-TSEKA	2008	102.06	78.14	132.23	RW2
Lesotho	THABA-TSEKA	2009	96.42	71.83	128.39	RW2
Lesotho	THABA-TSEKA	2010	89.81	64.87	122.64	RW2
Lesotho	THABA-TSEKA	2011	83.71	59.26	115.29	RW2
Lesotho	THABA-TSEKA	2012	77.52	54.12	109.59	RW2
Lesotho	THABA-TSEKA	2013	71.73	47.93	105.99	RW2
Lesotho	THABA-TSEKA	2014	66.22	40.24	106.36	RW2
Lesotho	THABA-TSEKA	2015	61.44	32.03	113.94	RW2
Lesotho	THABA-TSEKA	2016	56.92	24.81	123.07	RW2
Lesotho	THABA-TSEKA	2017	52.44	18.55	135.09	RW2
Lesotho	THABA-TSEKA	2018	48.27	13.65	151.90	RW2
Lesotho	THABA-TSEKA	2019	44.67	9.73	176.36	RW2
Liberia	ALL	1980	243.13	233.47	252.25	IHME
Liberia	ALL	1980	237.64	169.20	324.10	RW2
Liberia	ALL	1980	241.10	220.10	264.40	UN
Liberia	ALL	1981	239.34	230.37	248.02	IHME
Liberia	ALL	1981	237.24	184.57	299.36	RW2
Liberia	ALL	1981	238.00	217.50	260.30	UN
Liberia	ALL	1982	236.32	227.61	244.77	IHME
Liberia	ALL	1982	236.87	191.40	290.15	RW2
Liberia	ALL	1982	235.60	215.80	257.00	UN
Liberia	ALL	1983	233.41	225.51	241.36	IHME
Liberia	ALL	1983	236.32	190.34	290.96	RW2
Liberia	ALL	1983	234.00	214.80	254.90	UN
Liberia	ALL	1984	231.63	224.02	239.08	IHME
Liberia	ALL	1984	236.77	187.95	294.61	RW2
Liberia	ALL	1984	233.70	215.00	253.90	UN
Liberia	ALL	1985	230.15	222.87	237.75	IHME
Liberia	ALL	1985	236.37	190.35	288.38	RW2
Liberia	ALL	1985	235.00	216.40	255.00	UN
Liberia	ALL	1986	228.87	221.40	236.68	IHME
Liberia	ALL	1986	238.29	194.85	286.89	RW2
Liberia	ALL	1986	237.80	219.10	257.70	UN
Liberia	ALL	1987	227.51	219.94	235.21	IHME
Liberia	ALL	1987	241.53	199.52	288.49	RW2
Liberia	ALL	1987	242.10	223.40	262.20	UN
Liberia	ALL	1988	225.42	217.75	232.94	IHME
Liberia	ALL	1988	245.61	201.12	293.49	RW2
Liberia	ALL	1988	246.90	227.60	267.90	UN
Liberia	ALL	1989	223.01	215.21	230.78	IHME
Liberia	ALL	1989	250.10	203.01	300.09	RW2
Liberia	ALL	1989	251.50	231.40	273.70	UN
Liberia	ALL	1990	225.87	216.95	235.55	IHME
Liberia	ALL	1990	255.91	211.43	309.16	RW2
Liberia	ALL	1990	255.00	234.20	278.30	UN
Liberia	ALL	1991	220.40	212.85	228.28	IHME
Liberia	ALL	1991	258.08	215.27	307.05	RW2
Liberia	ALL	1991	256.70	235.20	280.20	UN
Liberia	ALL	1992	218.11	210.67	225.54	IHME
Liberia	ALL	1992	257.54	215.45	304.91	RW2
Liberia	ALL	1992	255.40	234.00	278.90	UN
Liberia	ALL	1993	218.39	210.68	227.29	IHME
Liberia	ALL	1993	253.87	211.53	302.59	RW2
Liberia	ALL	1993	251.60	231.00	274.50	UN
Liberia	ALL	1994	217.16	207.91	228.23	IHME
Liberia	ALL	1994	247.07	203.72	299.10	RW2
Liberia	ALL	1994	245.50	225.60	267.40	UN

Continued on next page

Country	Region	Year	Median	Lower	Upper	Method
Liberia	ALL	1995	210.56	203.09	218.84	IHME
Liberia	ALL	1995	236.75	193.44	284.13	RW2
Liberia	ALL	1995	237.40	218.30	258.20	UN
Liberia	ALL	1996	207.53	199.62	215.90	IHME
Liberia	ALL	1996	226.20	186.54	270.87	RW2
Liberia	ALL	1996	227.70	210.10	247.40	UN
Liberia	ALL	1997	197.05	190.82	203.75	IHME
Liberia	ALL	1997	215.00	178.21	256.65	RW2
Liberia	ALL	1997	217.10	200.50	235.50	UN
Liberia	ALL	1998	188.78	182.66	195.16	IHME
Liberia	ALL	1998	203.74	167.52	246.09	RW2
Liberia	ALL	1998	205.80	190.30	223.20	UN
Liberia	ALL	1999	178.78	173.22	184.66	IHME
Liberia	ALL	1999	192.40	155.88	234.00	RW2
Liberia	ALL	1999	193.90	179.30	210.40	UN
Liberia	ALL	2000	167.18	161.91	172.69	IHME
Liberia	ALL	2000	181.64	147.55	222.13	RW2
Liberia	ALL	2000	181.80	167.80	197.50	UN
Liberia	ALL	2001	156.16	150.92	161.52	IHME
Liberia	ALL	2001	170.12	138.98	206.94	RW2
Liberia	ALL	2001	169.70	156.30	184.20	UN
Liberia	ALL	2002	146.90	141.40	152.93	IHME
Liberia	ALL	2002	158.45	130.02	192.16	RW2
Liberia	ALL	2002	157.60	145.10	171.10	UN
Liberia	ALL	2003	138.97	133.17	145.12	IHME
Liberia	ALL	2003	146.93	119.89	179.45	RW2
Liberia	ALL	2003	145.90	134.00	158.40	UN
Liberia	ALL	2004	127.87	122.97	132.59	IHME
Liberia	ALL	2004	135.53	108.53	168.47	RW2
Liberia	ALL	2004	134.80	123.50	146.70	UN
Liberia	ALL	2005	119.91	115.13	124.58	IHME
Liberia	ALL	2005	124.54	98.08	155.48	RW2
Liberia	ALL	2005	124.70	113.80	136.20	UN
Liberia	ALL	2006	112.75	107.96	117.34	IHME
Liberia	ALL	2006	115.08	91.21	143.04	RW2
Liberia	ALL	2006	115.70	105.30	126.90	UN
Liberia	ALL	2007	105.92	101.08	110.54	IHME
Liberia	ALL	2007	106.92	85.17	132.75	RW2
Liberia	ALL	2007	107.80	97.60	118.70	UN
Liberia	ALL	2008	100.20	95.27	104.92	IHME
Liberia	ALL	2008	100.15	78.99	125.62	RW2
Liberia	ALL	2008	100.90	91.00	111.60	UN
Liberia	ALL	2009	95.53	90.28	100.82	IHME
Liberia	ALL	2009	94.24	73.06	120.28	RW2
Liberia	ALL	2009	94.70	84.80	105.70	UN
Liberia	ALL	2010	91.28	85.86	97.11	IHME
Liberia	ALL	2010	89.64	69.16	116.76	RW2
Liberia	ALL	2010	89.30	78.90	101.20	UN
Liberia	ALL	2011	86.90	81.23	93.16	IHME
Liberia	ALL	2011	85.32	66.53	109.41	RW2
Liberia	ALL	2011	84.50	73.30	97.70	UN
Liberia	ALL	2012	82.85	76.96	89.73	IHME
Liberia	ALL	2012	81.35	64.03	103.05	RW2
Liberia	ALL	2012	80.30	67.90	95.20	UN
Liberia	ALL	2013	78.45	72.25	85.93	IHME
Liberia	ALL	2013	77.72	59.20	101.25	RW2
Liberia	ALL	2013	76.30	62.90	93.50	UN
Liberia	ALL	2014	75.72	69.00	83.58	IHME
Liberia	ALL	2014	74.12	50.88	107.18	RW2
Liberia	ALL	2014	72.90	58.20	92.40	UN
Liberia	ALL	2015	71.39	64.64	79.71	IHME
Liberia	ALL	2015	70.47	40.17	121.88	RW2
Liberia	ALL	2015	69.90	53.90	91.80	UN

Continued on next page

Country	Region	Year	Median	Lower	Upper	Method
Liberia	ALL	2016	67.33	31.75	140.37	RW2
Liberia	ALL	2017	63.99	24.12	164.51	RW2
Liberia	ALL	2018	60.94	18.11	196.87	RW2
Liberia	ALL	2019	57.93	12.76	232.86	RW2
Liberia	ALL	15-19	63.96	24.56	161.81	RW2
Liberia	NORTH CENTRAL	1980	229.99	162.59	314.89	RW2
Liberia	NORTH CENTRAL	1981	230.09	176.00	294.23	RW2
Liberia	NORTH CENTRAL	1982	230.03	182.12	286.36	RW2
Liberia	NORTH CENTRAL	1983	229.71	183.51	284.90	RW2
Liberia	NORTH CENTRAL	1984	230.48	184.29	284.82	RW2
Liberia	NORTH CENTRAL	1985	230.90	187.30	280.64	RW2
Liberia	NORTH CENTRAL	1986	233.40	192.87	279.09	RW2
Liberia	NORTH CENTRAL	1987	237.38	198.12	280.89	RW2
Liberia	NORTH CENTRAL	1988	242.25	202.07	285.35	RW2
Liberia	NORTH CENTRAL	1989	247.66	205.80	292.25	RW2
Liberia	NORTH CENTRAL	1990	253.89	213.86	299.60	RW2
Liberia	NORTH CENTRAL	1991	256.41	219.03	298.77	RW2
Liberia	NORTH CENTRAL	1992	255.93	219.66	296.05	RW2
Liberia	NORTH CENTRAL	1993	251.56	214.49	293.21	RW2
Liberia	NORTH CENTRAL	1994	243.79	205.98	287.29	RW2
Liberia	NORTH CENTRAL	1995	231.83	194.86	272.10	RW2
Liberia	NORTH CENTRAL	1996	219.47	185.87	255.91	RW2
Liberia	NORTH CENTRAL	1997	206.16	175.66	240.47	RW2
Liberia	NORTH CENTRAL	1998	192.80	163.07	226.92	RW2
Liberia	NORTH CENTRAL	1999	179.54	149.47	214.35	RW2
Liberia	NORTH CENTRAL	2000	167.24	139.03	199.31	RW2
Liberia	NORTH CENTRAL	2001	155.52	130.58	184.17	RW2
Liberia	NORTH CENTRAL	2002	144.74	122.10	171.00	RW2
Liberia	NORTH CENTRAL	2003	134.64	112.18	160.23	RW2
Liberia	NORTH CENTRAL	2004	125.38	102.83	151.83	RW2
Liberia	NORTH CENTRAL	2005	116.82	95.51	142.52	RW2
Liberia	NORTH CENTRAL	2006	108.79	89.26	131.88	RW2
Liberia	NORTH CENTRAL	2007	101.28	83.24	122.51	RW2
Liberia	NORTH CENTRAL	2008	94.15	76.41	115.70	RW2
Liberia	NORTH CENTRAL	2009	87.54	69.29	109.81	RW2
Liberia	NORTH CENTRAL	2010	81.27	62.79	104.31	RW2
Liberia	NORTH CENTRAL	2011	75.49	58.55	96.80	RW2
Liberia	NORTH CENTRAL	2012	70.06	54.18	89.71	RW2
Liberia	NORTH CENTRAL	2013	64.97	48.41	86.18	RW2
Liberia	NORTH CENTRAL	2014	60.21	40.93	87.22	RW2
Liberia	NORTH CENTRAL	2015	55.83	32.22	94.22	RW2
Liberia	NORTH CENTRAL	2016	51.68	24.99	102.98	RW2
Liberia	NORTH CENTRAL	2017	47.79	18.95	114.70	RW2
Liberia	NORTH CENTRAL	2018	44.26	14.02	130.98	RW2
Liberia	NORTH CENTRAL	2019	40.97	10.03	151.35	RW2
Liberia	NORTH WESTERN	1980	257.37	173.68	360.06	RW2
Liberia	NORTH WESTERN	1981	260.35	189.34	342.45	RW2
Liberia	NORTH WESTERN	1982	262.67	199.45	334.08	RW2
Liberia	NORTH WESTERN	1983	265.32	205.32	333.97	RW2
Liberia	NORTH WESTERN	1984	268.46	209.66	335.82	RW2
Liberia	NORTH WESTERN	1985	271.69	217.51	332.39	RW2
Liberia	NORTH WESTERN	1986	277.17	227.02	332.23	RW2
Liberia	NORTH WESTERN	1987	284.02	236.45	336.23	RW2
Liberia	NORTH WESTERN	1988	292.10	243.65	344.24	RW2
Liberia	NORTH WESTERN	1989	300.47	250.36	353.49	RW2
Liberia	NORTH WESTERN	1990	309.81	261.72	364.35	RW2
Liberia	NORTH WESTERN	1991	314.93	269.29	365.78	RW2
Liberia	NORTH WESTERN	1992	316.01	271.31	364.88	RW2
Liberia	NORTH WESTERN	1993	312.49	267.65	362.24	RW2
Liberia	NORTH WESTERN	1994	305.04	258.79	356.62	RW2
Liberia	NORTH WESTERN	1995	292.33	246.61	341.42	RW2
Liberia	NORTH WESTERN	1996	278.73	237.03	323.87	RW2
Liberia	NORTH WESTERN	1997	263.60	225.31	306.61	RW2

Continued on next page

Country	Region	Year	Median	Lower	Upper	Method
Liberia	NORTH WESTERN	1998	248.03	211.13	290.31	RW2
Liberia	NORTH WESTERN	1999	232.59	194.55	275.26	RW2
Liberia	NORTH WESTERN	2000	217.54	181.38	258.34	RW2
Liberia	NORTH WESTERN	2001	203.37	170.72	240.21	RW2
Liberia	NORTH WESTERN	2002	190.00	160.04	224.10	RW2
Liberia	NORTH WESTERN	2003	177.46	148.25	211.26	RW2
Liberia	NORTH WESTERN	2004	165.93	136.32	200.04	RW2
Liberia	NORTH WESTERN	2005	155.33	127.16	188.82	RW2
Liberia	NORTH WESTERN	2006	145.33	119.76	175.56	RW2
Liberia	NORTH WESTERN	2007	135.86	111.71	163.80	RW2
Liberia	NORTH WESTERN	2008	127.04	103.23	155.07	RW2
Liberia	NORTH WESTERN	2009	118.56	94.67	147.33	RW2
Liberia	NORTH WESTERN	2010	110.73	87.33	139.65	RW2
Liberia	NORTH WESTERN	2011	103.28	82.08	129.32	RW2
Liberia	NORTH WESTERN	2012	96.34	77.16	119.66	RW2
Liberia	NORTH WESTERN	2013	89.85	69.84	115.12	RW2
Liberia	NORTH WESTERN	2014	83.75	59.29	116.70	RW2
Liberia	NORTH WESTERN	2015	77.98	46.88	126.44	RW2
Liberia	NORTH WESTERN	2016	72.48	36.46	138.24	RW2
Liberia	NORTH WESTERN	2017	67.54	27.67	155.06	RW2
Liberia	NORTH WESTERN	2018	62.64	20.35	176.92	RW2
Liberia	NORTH WESTERN	2019	58.28	14.70	205.52	RW2
Liberia	SOUTH CENTRAL	1980	245.97	180.09	328.49	RW2
Liberia	SOUTH CENTRAL	1981	245.70	194.54	305.60	RW2
Liberia	SOUTH CENTRAL	1982	245.44	200.79	296.18	RW2
Liberia	SOUTH CENTRAL	1983	245.11	201.03	296.77	RW2
Liberia	SOUTH CENTRAL	1984	245.62	200.64	297.79	RW2
Liberia	SOUTH CENTRAL	1985	245.97	202.59	293.17	RW2
Liberia	SOUTH CENTRAL	1986	248.27	208.56	292.59	RW2
Liberia	SOUTH CENTRAL	1987	252.21	214.06	293.85	RW2
Liberia	SOUTH CENTRAL	1988	257.20	217.41	299.62	RW2
Liberia	SOUTH CENTRAL	1989	262.44	220.15	307.28	RW2
Liberia	SOUTH CENTRAL	1990	268.76	228.51	315.38	RW2
Liberia	SOUTH CENTRAL	1991	271.39	233.13	313.50	RW2
Liberia	SOUTH CENTRAL	1992	270.37	233.54	310.69	RW2
Liberia	SOUTH CENTRAL	1993	265.68	228.85	307.43	RW2
Liberia	SOUTH CENTRAL	1994	257.48	219.06	301.63	RW2
Liberia	SOUTH CENTRAL	1995	244.94	206.51	286.44	RW2
Liberia	SOUTH CENTRAL	1996	231.92	197.60	268.63	RW2
Liberia	SOUTH CENTRAL	1997	218.08	186.85	253.25	RW2
Liberia	SOUTH CENTRAL	1998	204.34	173.86	239.18	RW2
Liberia	SOUTH CENTRAL	1999	190.80	160.16	225.96	RW2
Liberia	SOUTH CENTRAL	2000	178.04	149.19	211.52	RW2
Liberia	SOUTH CENTRAL	2001	166.13	140.27	195.94	RW2
Liberia	SOUTH CENTRAL	2002	154.97	131.36	182.30	RW2
Liberia	SOUTH CENTRAL	2003	144.64	121.19	171.64	RW2
Liberia	SOUTH CENTRAL	2004	135.13	111.10	162.59	RW2
Liberia	SOUTH CENTRAL	2005	126.42	103.28	154.01	RW2
Liberia	SOUTH CENTRAL	2006	118.12	97.18	143.41	RW2
Liberia	SOUTH CENTRAL	2007	110.50	90.82	134.18	RW2
Liberia	SOUTH CENTRAL	2008	103.32	83.56	127.20	RW2
Liberia	SOUTH CENTRAL	2009	96.44	76.36	121.08	RW2
Liberia	SOUTH CENTRAL	2010	90.10	70.02	115.21	RW2
Liberia	SOUTH CENTRAL	2011	84.10	65.77	107.13	RW2
Liberia	SOUTH CENTRAL	2012	78.47	61.50	99.70	RW2
Liberia	SOUTH CENTRAL	2013	73.20	55.59	95.86	RW2
Liberia	SOUTH CENTRAL	2014	68.17	47.14	97.23	RW2
Liberia	SOUTH CENTRAL	2015	63.61	37.48	105.66	RW2
Liberia	SOUTH CENTRAL	2016	59.19	29.10	115.86	RW2
Liberia	SOUTH CENTRAL	2017	55.23	22.12	130.49	RW2
Liberia	SOUTH CENTRAL	2018	51.27	16.42	148.53	RW2
Liberia	SOUTH CENTRAL	2019	47.87	12.01	172.13	RW2
Liberia	SOUTH EASTERN A	1980	203.72	135.57	293.29	RW2

Continued on next page

Country	Region	Year	Median	Lower	Upper	Method
Liberia	SOUTH EASTERN A	1981	204.93	148.32	276.27	RW2
Liberia	SOUTH EASTERN A	1982	206.11	154.88	268.22	RW2
Liberia	SOUTH EASTERN A	1983	207.37	158.01	267.28	RW2
Liberia	SOUTH EASTERN A	1984	209.14	161.19	267.22	RW2
Liberia	SOUTH EASTERN A	1985	210.89	166.12	263.72	RW2
Liberia	SOUTH EASTERN A	1986	214.58	172.68	262.94	RW2
Liberia	SOUTH EASTERN A	1987	219.44	179.42	264.97	RW2
Liberia	SOUTH EASTERN A	1988	225.26	184.61	270.93	RW2
Liberia	SOUTH EASTERN A	1989	231.64	189.55	278.35	RW2
Liberia	SOUTH EASTERN A	1990	238.58	198.16	286.57	RW2
Liberia	SOUTH EASTERN A	1991	242.24	203.02	286.45	RW2
Liberia	SOUTH EASTERN A	1992	242.58	204.70	285.03	RW2
Liberia	SOUTH EASTERN A	1993	239.09	201.22	282.54	RW2
Liberia	SOUTH EASTERN A	1994	232.32	193.80	277.56	RW2
Liberia	SOUTH EASTERN A	1995	221.72	183.96	263.66	RW2
Liberia	SOUTH EASTERN A	1996	210.31	175.75	248.76	RW2
Liberia	SOUTH EASTERN A	1997	198.44	167.13	234.45	RW2
Liberia	SOUTH EASTERN A	1998	186.43	156.07	222.18	RW2
Liberia	SOUTH EASTERN A	1999	174.92	144.33	210.56	RW2
Liberia	SOUTH EASTERN A	2000	163.95	134.87	197.96	RW2
Liberia	SOUTH EASTERN A	2001	153.75	127.80	184.36	RW2
Liberia	SOUTH EASTERN A	2002	144.37	120.33	172.21	RW2
Liberia	SOUTH EASTERN A	2003	135.63	112.08	163.39	RW2
Liberia	SOUTH EASTERN A	2004	127.70	104.03	156.09	RW2
Liberia	SOUTH EASTERN A	2005	120.54	97.66	148.28	RW2
Liberia	SOUTH EASTERN A	2006	113.55	92.50	138.82	RW2
Liberia	SOUTH EASTERN A	2007	106.93	87.09	130.73	RW2
Liberia	SOUTH EASTERN A	2008	100.64	80.65	124.72	RW2
Liberia	SOUTH EASTERN A	2009	94.74	74.34	119.84	RW2
Liberia	SOUTH EASTERN A	2010	89.05	68.58	114.74	RW2
Liberia	SOUTH EASTERN A	2011	83.67	64.45	107.77	RW2
Liberia	SOUTH EASTERN A	2012	78.66	60.42	101.32	RW2
Liberia	SOUTH EASTERN A	2013	73.86	55.15	98.18	RW2
Liberia	SOUTH EASTERN A	2014	69.28	47.30	100.07	RW2
Liberia	SOUTH EASTERN A	2015	64.99	37.83	109.34	RW2
Liberia	SOUTH EASTERN A	2016	60.90	29.56	120.28	RW2
Liberia	SOUTH EASTERN A	2017	57.17	22.71	135.83	RW2
Liberia	SOUTH EASTERN A	2018	53.42	16.89	155.26	RW2
Liberia	SOUTH EASTERN A	2019	50.22	12.30	182.99	RW2
Liberia	SOUTH EASTERN B	1980	168.78	110.94	251.81	RW2
Liberia	SOUTH EASTERN B	1981	171.30	122.73	236.68	RW2
Liberia	SOUTH EASTERN B	1982	173.99	130.48	229.99	RW2
Liberia	SOUTH EASTERN B	1983	176.57	134.82	229.59	RW2
Liberia	SOUTH EASTERN B	1984	179.71	138.62	231.87	RW2
Liberia	SOUTH EASTERN B	1985	182.81	144.06	229.09	RW2
Liberia	SOUTH EASTERN B	1986	187.55	151.80	229.79	RW2
Liberia	SOUTH EASTERN B	1987	193.43	158.80	233.39	RW2
Liberia	SOUTH EASTERN B	1988	200.35	164.96	240.14	RW2
Liberia	SOUTH EASTERN B	1989	207.57	170.19	248.53	RW2
Liberia	SOUTH EASTERN B	1990	215.73	179.76	257.52	RW2
Liberia	SOUTH EASTERN B	1991	220.49	186.14	259.88	RW2
Liberia	SOUTH EASTERN B	1992	222.26	189.21	260.28	RW2
Liberia	SOUTH EASTERN B	1993	220.88	187.18	259.87	RW2
Liberia	SOUTH EASTERN B	1994	216.03	180.17	256.71	RW2
Liberia	SOUTH EASTERN B	1995	208.02	172.08	246.93	RW2
Liberia	SOUTH EASTERN B	1996	199.61	166.34	236.34	RW2
Liberia	SOUTH EASTERN B	1997	190.52	159.27	225.33	RW2
Liberia	SOUTH EASTERN B	1998	181.94	150.43	217.17	RW2
Liberia	SOUTH EASTERN B	1999	173.65	141.52	209.67	RW2
Liberia	SOUTH EASTERN B	2000	166.28	135.23	201.26	RW2
Liberia	SOUTH EASTERN B	2001	159.71	130.95	192.37	RW2
Liberia	SOUTH EASTERN B	2002	154.27	127.24	184.55	RW2
Liberia	SOUTH EASTERN B	2003	149.31	122.66	179.02	RW2

Continued on next page

Country	Region	Year	Median	Lower	Upper	Method
Liberia	SOUTH EASTERN B	2004	145.33	118.70	175.67	RW2
Liberia	SOUTH EASTERN B	2005	142.04	116.99	171.54	RW2
Liberia	SOUTH EASTERN B	2006	138.92	116.07	165.44	RW2
Liberia	SOUTH EASTERN B	2007	136.13	114.54	160.62	RW2
Liberia	SOUTH EASTERN B	2008	133.70	111.34	159.34	RW2
Liberia	SOUTH EASTERN B	2009	131.25	106.77	160.26	RW2
Liberia	SOUTH EASTERN B	2010	128.91	103.06	160.72	RW2
Liberia	SOUTH EASTERN B	2011	126.47	100.99	157.78	RW2
Liberia	SOUTH EASTERN B	2012	124.23	99.10	155.42	RW2
Liberia	SOUTH EASTERN B	2013	122.04	93.79	158.57	RW2
Liberia	SOUTH EASTERN B	2014	119.94	83.97	169.54	RW2
Liberia	SOUTH EASTERN B	2015	117.74	70.59	192.00	RW2
Liberia	SOUTH EASTERN B	2016	115.68	57.88	217.64	RW2
Liberia	SOUTH EASTERN B	2017	113.52	46.52	252.01	RW2
Liberia	SOUTH EASTERN B	2018	111.47	36.75	295.65	RW2
Liberia	SOUTH EASTERN B	2019	109.59	27.67	344.70	RW2
Madagascar	ALL	1980	160.07	151.09	170.34	IHME
Madagascar	ALL	1980	178.53	130.95	238.60	RW2
Madagascar	ALL	1980	174.60	162.00	188.70	UN
Madagascar	ALL	1981	163.44	154.09	173.64	IHME
Madagascar	ALL	1981	179.33	144.76	220.31	RW2
Madagascar	ALL	1981	177.60	165.00	191.50	UN
Madagascar	ALL	1982	166.85	157.19	176.99	IHME
Madagascar	ALL	1982	180.23	147.99	217.40	RW2
Madagascar	ALL	1982	180.30	167.80	194.20	UN
Madagascar	ALL	1983	168.11	158.71	177.25	IHME
Madagascar	ALL	1983	180.67	144.52	222.31	RW2
Madagascar	ALL	1983	182.40	169.80	196.40	UN
Madagascar	ALL	1984	168.48	159.72	177.99	IHME
Madagascar	ALL	1984	181.03	141.04	226.96	RW2
Madagascar	ALL	1984	183.10	170.50	197.00	UN
Madagascar	ALL	1985	168.91	159.76	178.34	IHME
Madagascar	ALL	1985	180.89	145.05	223.78	RW2
Madagascar	ALL	1985	182.40	170.00	196.20	UN
Madagascar	ALL	1986	168.34	158.97	177.94	IHME
Madagascar	ALL	1986	179.38	146.29	217.82	RW2
Madagascar	ALL	1986	180.10	168.00	193.60	UN
Madagascar	ALL	1987	164.68	155.80	174.59	IHME
Madagascar	ALL	1987	176.74	145.77	213.21	RW2
Madagascar	ALL	1987	176.50	164.80	189.40	UN
Madagascar	ALL	1988	157.40	148.97	166.62	IHME
Madagascar	ALL	1988	172.80	140.97	210.55	RW2
Madagascar	ALL	1988	171.70	160.40	184.10	UN
Madagascar	ALL	1989	149.03	140.58	157.60	IHME
Madagascar	ALL	1989	167.98	135.32	207.91	RW2
Madagascar	ALL	1989	166.20	155.30	177.90	UN
Madagascar	ALL	1990	143.12	135.08	151.04	IHME
Madagascar	ALL	1990	161.96	129.57	199.74	RW2
Madagascar	ALL	1990	160.70	150.00	172.10	UN
Madagascar	ALL	1991	140.43	133.04	147.86	IHME
Madagascar	ALL	1991	156.55	127.00	190.48	RW2
Madagascar	ALL	1991	155.80	145.40	166.80	UN
Madagascar	ALL	1992	139.35	131.67	147.58	IHME
Madagascar	ALL	1992	151.34	123.52	183.62	RW2
Madagascar	ALL	1992	151.30	141.40	161.90	UN
Madagascar	ALL	1993	138.32	130.24	146.87	IHME
Madagascar	ALL	1993	146.52	118.75	179.22	RW2
Madagascar	ALL	1993	147.10	137.60	157.50	UN
Madagascar	ALL	1994	135.94	128.17	143.82	IHME
Madagascar	ALL	1994	141.88	113.08	176.59	RW2
Madagascar	ALL	1994	142.90	133.60	153.40	UN
Madagascar	ALL	1995	131.40	123.08	139.22	IHME
Madagascar	ALL	1995	137.93	110.44	172.04	RW2

Continued on next page

Country	Region	Year	Median	Lower	Upper	Method
Madagascar	ALL	1995	138.40	129.20	149.00	UN
Madagascar	ALL	1996	125.57	117.93	133.17	IHME
Madagascar	ALL	1996	133.07	107.64	164.60	RW2
Madagascar	ALL	1996	133.40	124.30	143.90	UN
Madagascar	ALL	1997	119.78	112.01	127.10	IHME
Madagascar	ALL	1997	127.83	103.92	156.53	RW2
Madagascar	ALL	1997	127.70	118.60	138.10	UN
Madagascar	ALL	1998	114.59	107.02	122.33	IHME
Madagascar	ALL	1998	122.15	98.40	151.47	RW2
Madagascar	ALL	1998	121.70	112.60	132.10	UN
Madagascar	ALL	1999	110.50	103.16	118.96	IHME
Madagascar	ALL	1999	116.03	91.93	145.23	RW2
Madagascar	ALL	1999	115.50	106.20	125.70	UN
Madagascar	ALL	2000	107.08	99.82	115.23	IHME
Madagascar	ALL	2000	109.41	86.21	137.11	RW2
Madagascar	ALL	2000	109.20	99.90	119.40	UN
Madagascar	ALL	2001	103.41	96.18	111.31	IHME
Madagascar	ALL	2001	103.13	82.10	128.26	RW2
Madagascar	ALL	2001	103.10	93.90	112.90	UN
Madagascar	ALL	2002	99.34	91.95	107.49	IHME
Madagascar	ALL	2002	97.01	77.98	120.05	RW2
Madagascar	ALL	2002	97.20	88.10	106.90	UN
Madagascar	ALL	2003	94.18	86.60	102.21	IHME
Madagascar	ALL	2003	91.27	72.84	113.87	RW2
Madagascar	ALL	2003	91.50	82.40	101.00	UN
Madagascar	ALL	2004	88.63	80.84	96.94	IHME
Madagascar	ALL	2004	85.70	66.62	109.38	RW2
Madagascar	ALL	2004	85.90	76.80	95.60	UN
Madagascar	ALL	2005	83.56	75.85	91.52	IHME
Madagascar	ALL	2005	80.71	61.46	105.43	RW2
Madagascar	ALL	2005	80.60	71.20	90.50	UN
Madagascar	ALL	2006	80.08	72.74	87.61	IHME
Madagascar	ALL	2006	75.84	58.66	97.50	RW2
Madagascar	ALL	2006	75.40	65.60	86.20	UN
Madagascar	ALL	2007	78.13	70.67	85.92	IHME
Madagascar	ALL	2007	71.30	56.37	89.84	RW2
Madagascar	ALL	2007	70.80	60.10	82.80	UN
Madagascar	ALL	2008	77.01	69.18	85.40	IHME
Madagascar	ALL	2008	67.12	51.72	86.73	RW2
Madagascar	ALL	2008	66.70	55.30	79.90	UN
Madagascar	ALL	2009	76.61	67.13	86.62	IHME
Madagascar	ALL	2009	62.96	43.60	90.63	RW2
Madagascar	ALL	2009	63.30	50.80	78.00	UN
Madagascar	ALL	2010	75.84	64.58	87.00	IHME
Madagascar	ALL	2010	59.07	33.60	103.17	RW2
Madagascar	ALL	2010	60.30	46.70	76.60	UN
Madagascar	ALL	2011	74.39	62.34	86.91	IHME
Madagascar	ALL	2011	55.50	25.36	117.72	RW2
Madagascar	ALL	2011	57.80	43.20	75.80	UN
Madagascar	ALL	2012	72.24	60.15	86.02	IHME
Madagascar	ALL	2012	52.02	18.49	138.29	RW2
Madagascar	ALL	2012	55.50	40.00	75.40	UN
Madagascar	ALL	2013	69.66	56.94	84.42	IHME
Madagascar	ALL	2013	48.95	13.33	164.05	RW2
Madagascar	ALL	2013	53.40	37.10	75.40	UN
Madagascar	ALL	2014	66.92	54.66	83.01	IHME
Madagascar	ALL	2014	45.86	9.34	197.67	RW2
Madagascar	ALL	2014	51.50	34.40	74.90	UN
Madagascar	ALL	2015	64.20	51.93	80.64	IHME
Madagascar	ALL	2015	42.83	6.52	236.65	RW2
Madagascar	ALL	2015	49.60	32.00	74.90	UN
Madagascar	ALL	2016	40.46	4.49	291.19	RW2
Madagascar	ALL	2017	37.70	2.95	352.75	RW2

Continued on next page

Country	Region	Year	Median	Lower	Upper	Method
Madagascar	ALL	2018	35.32	1.97	428.43	RW2
Madagascar	ALL	2019	32.99	1.17	495.57	RW2
Madagascar	ALL	15-19	37.70	2.97	347.99	RW2
Madagascar	ANTANANARIVO	1980	136.31	100.04	182.18	RW2
Madagascar	ANTANANARIVO	1981	138.72	112.36	169.78	RW2
Madagascar	ANTANANARIVO	1982	140.90	117.37	167.96	RW2
Madagascar	ANTANANARIVO	1983	142.97	117.00	173.43	RW2
Madagascar	ANTANANARIVO	1984	144.62	116.09	178.24	RW2
Madagascar	ANTANANARIVO	1985	145.74	119.30	177.51	RW2
Madagascar	ANTANANARIVO	1986	145.73	121.34	174.40	RW2
Madagascar	ANTANANARIVO	1987	144.43	121.21	171.97	RW2
Madagascar	ANTANANARIVO	1988	141.84	117.74	170.43	RW2
Madagascar	ANTANANARIVO	1989	137.94	113.01	167.66	RW2
Madagascar	ANTANANARIVO	1990	132.99	108.85	161.82	RW2
Madagascar	ANTANANARIVO	1991	127.29	105.51	153.04	RW2
Madagascar	ANTANANARIVO	1992	121.00	100.61	144.83	RW2
Madagascar	ANTANANARIVO	1993	114.46	94.47	138.19	RW2
Madagascar	ANTANANARIVO	1994	108.20	87.77	132.53	RW2
Madagascar	ANTANANARIVO	1995	101.84	82.11	125.01	RW2
Madagascar	ANTANANARIVO	1996	96.45	78.42	117.40	RW2
Madagascar	ANTANANARIVO	1997	91.64	74.81	111.40	RW2
Madagascar	ANTANANARIVO	1998	87.49	70.86	107.04	RW2
Madagascar	ANTANANARIVO	1999	83.90	66.33	104.03	RW2
Madagascar	ANTANANARIVO	2000	81.01	64.05	101.49	RW2
Madagascar	ANTANANARIVO	2001	78.03	62.19	96.76	RW2
Madagascar	ANTANANARIVO	2002	75.08	60.25	92.59	RW2
Madagascar	ANTANANARIVO	2003	72.06	57.48	89.89	RW2
Madagascar	ANTANANARIVO	2004	69.06	53.99	87.97	RW2
Madagascar	ANTANANARIVO	2005	65.81	50.34	85.41	RW2
Madagascar	ANTANANARIVO	2006	62.90	48.21	81.81	RW2
Madagascar	ANTANANARIVO	2007	59.99	45.46	79.10	RW2
Madagascar	ANTANANARIVO	2008	57.25	41.37	79.83	RW2
Madagascar	ANTANANARIVO	2009	54.51	35.51	84.48	RW2
Madagascar	ANTANANARIVO	2010	52.13	28.66	95.54	RW2
Madagascar	ANTANANARIVO	2011	49.67	22.47	109.15	RW2
Madagascar	ANTANANARIVO	2012	47.40	17.19	126.91	RW2
Madagascar	ANTANANARIVO	2013	45.33	12.86	152.49	RW2
Madagascar	ANTANANARIVO	2014	43.37	9.35	180.65	RW2
Madagascar	ANTANANARIVO	2015	41.41	6.71	217.59	RW2
Madagascar	ANTANANARIVO	2016	39.35	4.71	262.02	RW2
Madagascar	ANTANANARIVO	2017	37.73	3.29	318.84	RW2
Madagascar	ANTANANARIVO	2018	35.77	2.22	385.78	RW2
Madagascar	ANTANANARIVO	2019	34.20	1.49	464.97	RW2
Madagascar	ANTSIRANANA	1980	144.76	102.64	201.98	RW2
Madagascar	ANTSIRANANA	1981	143.60	112.39	182.14	RW2
Madagascar	ANTSIRANANA	1982	142.47	115.26	174.29	RW2
Madagascar	ANTSIRANANA	1983	141.18	113.19	174.85	RW2
Madagascar	ANTSIRANANA	1984	140.16	110.91	175.06	RW2
Madagascar	ANTSIRANANA	1985	139.31	111.41	171.70	RW2
Madagascar	ANTSIRANANA	1986	138.44	112.93	168.46	RW2
Madagascar	ANTSIRANANA	1987	137.90	113.48	166.09	RW2
Madagascar	ANTSIRANANA	1988	137.32	112.06	166.77	RW2
Madagascar	ANTSIRANANA	1989	136.48	109.94	168.36	RW2
Madagascar	ANTSIRANANA	1990	135.32	108.81	167.34	RW2
Madagascar	ANTSIRANANA	1991	133.40	108.25	162.96	RW2
Madagascar	ANTSIRANANA	1992	130.24	106.29	158.91	RW2
Madagascar	ANTSIRANANA	1993	126.29	102.55	155.66	RW2
Madagascar	ANTSIRANANA	1994	121.81	97.23	152.30	RW2
Madagascar	ANTSIRANANA	1995	116.57	92.35	146.11	RW2
Madagascar	ANTSIRANANA	1996	112.09	89.54	138.93	RW2
Madagascar	ANTSIRANANA	1997	108.23	86.65	134.47	RW2
Madagascar	ANTSIRANANA	1998	105.11	83.21	131.64	RW2
Madagascar	ANTSIRANANA	1999	102.23	79.51	129.89	RW2

Continued on next page

Country	Region	Year	Median	Lower	Upper	Method
Madagascar	ANTSIRANANA	2000	99.95	77.45	128.93	RW2
Madagascar	ANTSIRANANA	2001	96.82	74.84	124.90	RW2
Madagascar	ANTSIRANANA	2002	92.84	71.28	120.64	RW2
Madagascar	ANTSIRANANA	2003	88.01	65.95	116.87	RW2
Madagascar	ANTSIRANANA	2004	82.51	59.56	113.01	RW2
Madagascar	ANTSIRANANA	2005	76.21	52.71	108.55	RW2
Madagascar	ANTSIRANANA	2006	70.10	47.23	103.19	RW2
Madagascar	ANTSIRANANA	2007	64.37	41.50	98.85	RW2
Madagascar	ANTSIRANANA	2008	58.98	35.14	97.21	RW2
Madagascar	ANTSIRANANA	2009	53.83	28.52	98.70	RW2
Madagascar	ANTSIRANANA	2010	49.36	21.88	106.42	RW2
Madagascar	ANTSIRANANA	2011	45.11	16.44	116.47	RW2
Madagascar	ANTSIRANANA	2012	41.23	11.89	130.40	RW2
Madagascar	ANTSIRANANA	2013	37.70	8.42	148.60	RW2
Madagascar	ANTSIRANANA	2014	34.27	5.69	171.13	RW2
Madagascar	ANTSIRANANA	2015	31.50	3.90	202.51	RW2
Madagascar	ANTSIRANANA	2016	28.65	2.55	238.40	RW2
Madagascar	ANTSIRANANA	2017	26.33	1.68	284.89	RW2
Madagascar	ANTSIRANANA	2018	23.85	1.09	335.41	RW2
Madagascar	ANTSIRANANA	2019	21.95	0.70	397.14	RW2
Madagascar	FIANARANTSOA	1980	242.47	183.11	314.83	RW2
Madagascar	FIANARANTSOA	1981	238.63	197.37	287.42	RW2
Madagascar	FIANARANTSOA	1982	234.81	198.32	276.08	RW2
Madagascar	FIANARANTSOA	1983	230.89	191.57	275.46	RW2
Madagascar	FIANARANTSOA	1984	227.00	185.94	273.65	RW2
Madagascar	FIANARANTSOA	1985	223.26	185.86	266.32	RW2
Madagascar	FIANARANTSOA	1986	219.37	185.46	257.30	RW2
Madagascar	FIANARANTSOA	1987	215.35	183.46	250.60	RW2
Madagascar	FIANARANTSOA	1988	211.19	178.24	247.95	RW2
Madagascar	FIANARANTSOA	1989	207.09	172.63	246.20	RW2
Madagascar	FIANARANTSOA	1990	202.74	169.05	241.14	RW2
Madagascar	FIANARANTSOA	1991	198.57	167.05	233.02	RW2
Madagascar	FIANARANTSOA	1992	194.19	164.69	226.91	RW2
Madagascar	FIANARANTSOA	1993	189.67	160.02	223.77	RW2
Madagascar	FIANARANTSOA	1994	185.34	154.33	222.00	RW2
Madagascar	FIANARANTSOA	1995	180.65	149.90	215.88	RW2
Madagascar	FIANARANTSOA	1996	176.36	147.68	209.10	RW2
Madagascar	FIANARANTSOA	1997	172.38	145.59	203.60	RW2
Madagascar	FIANARANTSOA	1998	168.27	140.86	200.29	RW2
Madagascar	FIANARANTSOA	1999	163.88	134.68	197.17	RW2
Madagascar	FIANARANTSOA	2000	159.36	131.12	193.55	RW2
Madagascar	FIANARANTSOA	2001	153.17	127.49	184.01	RW2
Madagascar	FIANARANTSOA	2002	145.83	121.83	173.73	RW2
Madagascar	FIANARANTSOA	2003	137.21	113.40	165.55	RW2
Madagascar	FIANARANTSOA	2004	127.84	103.53	157.75	RW2
Madagascar	FIANARANTSOA	2005	117.65	93.07	147.08	RW2
Madagascar	FIANARANTSOA	2006	107.77	86.16	133.50	RW2
Madagascar	FIANARANTSOA	2007	98.19	79.25	120.89	RW2
Madagascar	FIANARANTSOA	2008	89.17	69.27	113.88	RW2
Madagascar	FIANARANTSOA	2009	80.94	56.52	114.10	RW2
Madagascar	FIANARANTSOA	2010	73.40	42.80	122.29	RW2
Madagascar	FIANARANTSOA	2011	66.38	31.57	132.67	RW2
Madagascar	FIANARANTSOA	2012	60.15	22.59	147.11	RW2
Madagascar	FIANARANTSOA	2013	54.35	16.23	165.60	RW2
Madagascar	FIANARANTSOA	2014	48.94	11.04	187.78	RW2
Madagascar	FIANARANTSOA	2015	44.14	7.36	219.12	RW2
Madagascar	FIANARANTSOA	2016	39.69	4.74	252.18	RW2
Madagascar	FIANARANTSOA	2017	35.88	3.12	294.44	RW2
Madagascar	FIANARANTSOA	2018	32.07	1.98	340.70	RW2
Madagascar	FIANARANTSOA	2019	29.08	1.23	405.71	RW2
Madagascar	MAHAJANGA	1980	252.80	192.28	327.32	RW2
Madagascar	MAHAJANGA	1981	242.99	200.68	292.51	RW2
Madagascar	MAHAJANGA	1982	233.49	196.94	274.73	RW2

Continued on next page

Country	Region	Year	Median	Lower	Upper	Method
Madagascar	MAHAJANGA	1983	223.99	185.70	267.43	RW2
Madagascar	MAHAJANGA	1984	214.91	175.37	260.80	RW2
Madagascar	MAHAJANGA	1985	206.32	170.64	247.13	RW2
Madagascar	MAHAJANGA	1986	197.90	166.62	233.38	RW2
Madagascar	MAHAJANGA	1987	189.83	160.49	222.50	RW2
Madagascar	MAHAJANGA	1988	182.09	152.61	215.09	RW2
Madagascar	MAHAJANGA	1989	174.54	144.11	208.93	RW2
Madagascar	MAHAJANGA	1990	167.26	137.97	200.03	RW2
Madagascar	MAHAJANGA	1991	159.93	133.50	189.68	RW2
Madagascar	MAHAJANGA	1992	152.55	128.48	180.47	RW2
Madagascar	MAHAJANGA	1993	145.44	121.61	173.68	RW2
Madagascar	MAHAJANGA	1994	138.43	113.35	167.64	RW2
Madagascar	MAHAJANGA	1995	131.78	107.30	160.03	RW2
Madagascar	MAHAJANGA	1996	126.18	103.78	152.58	RW2
Madagascar	MAHAJANGA	1997	121.22	100.24	145.98	RW2
Madagascar	MAHAJANGA	1998	117.14	95.70	142.31	RW2
Madagascar	MAHAJANGA	1999	113.19	90.88	139.40	RW2
Madagascar	MAHAJANGA	2000	109.75	88.26	136.61	RW2
Madagascar	MAHAJANGA	2001	105.23	85.17	130.55	RW2
Madagascar	MAHAJANGA	2002	100.11	81.13	123.72	RW2
Madagascar	MAHAJANGA	2003	93.92	75.01	117.34	RW2
Madagascar	MAHAJANGA	2004	87.25	68.20	111.45	RW2
Madagascar	MAHAJANGA	2005	79.88	61.08	103.69	RW2
Madagascar	MAHAJANGA	2006	72.79	55.79	94.30	RW2
Madagascar	MAHAJANGA	2007	66.01	50.48	85.70	RW2
Madagascar	MAHAJANGA	2008	59.84	43.85	81.01	RW2
Madagascar	MAHAJANGA	2009	54.10	35.63	81.11	RW2
Madagascar	MAHAJANGA	2010	49.01	27.29	86.63	RW2
Madagascar	MAHAJANGA	2011	44.11	20.03	93.60	RW2
Madagascar	MAHAJANGA	2012	39.83	14.45	103.13	RW2
Madagascar	MAHAJANGA	2013	35.98	10.16	116.83	RW2
Madagascar	MAHAJANGA	2014	32.54	6.97	133.74	RW2
Madagascar	MAHAJANGA	2015	29.30	4.75	156.65	RW2
Madagascar	MAHAJANGA	2016	26.45	3.08	180.86	RW2
Madagascar	MAHAJANGA	2017	23.79	2.02	214.49	RW2
Madagascar	MAHAJANGA	2018	21.44	1.32	257.96	RW2
Madagascar	MAHAJANGA	2019	19.37	0.80	302.70	RW2
Madagascar	TOAMASINA	1980	167.43	121.41	225.66	RW2
Madagascar	TOAMASINA	1981	170.50	136.05	211.38	RW2
Madagascar	TOAMASINA	1982	173.44	142.77	209.28	RW2
Madagascar	TOAMASINA	1983	176.76	143.69	214.75	RW2
Madagascar	TOAMASINA	1984	179.52	144.25	219.85	RW2
Madagascar	TOAMASINA	1985	182.32	149.55	219.69	RW2
Madagascar	TOAMASINA	1986	184.31	154.46	217.84	RW2
Madagascar	TOAMASINA	1987	185.66	157.07	218.02	RW2
Madagascar	TOAMASINA	1988	185.98	156.36	219.39	RW2
Madagascar	TOAMASINA	1989	185.14	153.60	222.13	RW2
Madagascar	TOAMASINA	1990	182.88	151.44	219.71	RW2
Madagascar	TOAMASINA	1991	178.56	149.45	212.54	RW2
Madagascar	TOAMASINA	1992	172.39	144.99	204.14	RW2
Madagascar	TOAMASINA	1993	164.67	137.58	196.72	RW2
Madagascar	TOAMASINA	1994	155.59	128.12	188.78	RW2
Madagascar	TOAMASINA	1995	145.65	119.30	176.44	RW2
Madagascar	TOAMASINA	1996	135.95	112.53	163.51	RW2
Madagascar	TOAMASINA	1997	126.65	105.08	151.85	RW2
Madagascar	TOAMASINA	1998	117.87	96.78	142.54	RW2
Madagascar	TOAMASINA	1999	109.54	87.77	134.85	RW2
Madagascar	TOAMASINA	2000	102.17	81.54	127.42	RW2
Madagascar	TOAMASINA	2001	94.34	75.30	117.46	RW2
Madagascar	TOAMASINA	2002	86.53	68.80	108.08	RW2
Madagascar	TOAMASINA	2003	78.68	61.33	100.02	RW2
Madagascar	TOAMASINA	2004	70.97	53.96	92.86	RW2
Madagascar	TOAMASINA	2005	63.44	46.65	84.82	RW2

Continued on next page

Country	Region	Year	Median	Lower	Upper	Method
Madagascar	TOAMASINA	2006	56.53	41.15	76.68	RW2
Madagascar	TOAMASINA	2007	50.42	35.98	69.42	RW2
Madagascar	TOAMASINA	2008	44.78	30.37	65.38	RW2
Madagascar	TOAMASINA	2009	39.69	24.21	64.06	RW2
Madagascar	TOAMASINA	2010	35.21	18.12	66.96	RW2
Madagascar	TOAMASINA	2011	31.36	13.30	70.86	RW2
Madagascar	TOAMASINA	2012	27.82	9.42	77.52	RW2
Madagascar	TOAMASINA	2013	24.47	6.49	85.11	RW2
Madagascar	TOAMASINA	2014	21.81	4.45	98.50	RW2
Madagascar	TOAMASINA	2015	19.29	2.96	110.95	RW2
Madagascar	TOAMASINA	2016	17.13	1.91	133.71	RW2
Madagascar	TOAMASINA	2017	15.28	1.24	156.55	RW2
Madagascar	TOAMASINA	2018	13.55	0.78	184.87	RW2
Madagascar	TOAMASINA	2019	11.87	0.47	226.93	RW2
Madagascar	TOLIARY	1980	173.56	124.15	236.79	RW2
Madagascar	TOLIARY	1981	174.82	138.03	218.49	RW2
Madagascar	TOLIARY	1982	175.88	143.48	213.42	RW2
Madagascar	TOLIARY	1983	176.74	143.04	216.17	RW2
Madagascar	TOLIARY	1984	177.43	142.69	218.00	RW2
Madagascar	TOLIARY	1985	177.85	145.43	215.28	RW2
Madagascar	TOLIARY	1986	177.68	148.33	210.99	RW2
Madagascar	TOLIARY	1987	176.83	149.37	207.81	RW2
Madagascar	TOLIARY	1988	175.23	146.49	207.67	RW2
Madagascar	TOLIARY	1989	172.94	142.59	207.68	RW2
Madagascar	TOLIARY	1990	170.10	140.33	204.59	RW2
Madagascar	TOLIARY	1991	166.88	139.24	198.70	RW2
Madagascar	TOLIARY	1992	163.31	136.85	193.48	RW2
Madagascar	TOLIARY	1993	159.43	133.05	190.13	RW2
Madagascar	TOLIARY	1994	155.42	127.84	188.41	RW2
Madagascar	TOLIARY	1995	150.97	123.54	182.86	RW2
Madagascar	TOLIARY	1996	146.90	121.38	176.67	RW2
Madagascar	TOLIARY	1997	142.69	118.71	170.73	RW2
Madagascar	TOLIARY	1998	138.24	114.30	166.90	RW2
Madagascar	TOLIARY	1999	133.37	108.48	162.96	RW2
Madagascar	TOLIARY	2000	128.32	104.38	157.22	RW2
Madagascar	TOLIARY	2001	121.81	100.56	147.42	RW2
Madagascar	TOLIARY	2002	114.36	94.78	137.60	RW2
Madagascar	TOLIARY	2003	105.91	86.74	128.99	RW2
Madagascar	TOLIARY	2004	97.02	77.49	120.89	RW2
Madagascar	TOLIARY	2005	87.69	67.85	111.88	RW2
Madagascar	TOLIARY	2006	78.91	61.10	100.76	RW2
Madagascar	TOLIARY	2007	70.64	54.46	90.59	RW2
Madagascar	TOLIARY	2008	63.13	46.51	84.56	RW2
Madagascar	TOLIARY	2009	56.24	37.32	83.20	RW2
Madagascar	TOLIARY	2010	50.31	27.66	88.15	RW2
Madagascar	TOLIARY	2011	44.66	20.26	94.09	RW2
Madagascar	TOLIARY	2012	39.85	14.32	102.87	RW2
Madagascar	TOLIARY	2013	35.36	9.80	115.41	RW2
Madagascar	TOLIARY	2014	31.46	6.59	128.77	RW2
Madagascar	TOLIARY	2015	28.00	4.42	152.14	RW2
Madagascar	TOLIARY	2016	24.85	2.91	176.71	RW2
Madagascar	TOLIARY	2017	22.12	1.84	210.63	RW2
Madagascar	TOLIARY	2018	19.49	1.11	246.99	RW2
Madagascar	TOLIARY	2019	17.26	0.71	289.38	RW2
Malawi	ALL	1980	258.10	244.90	271.86	IHME
Malawi	ALL	1980	241.77	188.53	304.58	RW2
Malawi	ALL	1980	256.70	241.00	272.40	UN
Malawi	ALL	1981	253.00	241.01	265.60	IHME
Malawi	ALL	1981	244.66	206.00	288.36	RW2
Malawi	ALL	1981	249.20	234.00	264.70	UN
Malawi	ALL	1982	248.57	237.06	260.79	IHME
Malawi	ALL	1982	247.67	211.43	287.52	RW2
Malawi	ALL	1982	244.40	229.40	259.70	UN

Continued on next page

Country	Region	Year	Median	Lower	Upper	Method
Malawi	ALL	1983	244.80	233.54	257.50	IHME
Malawi	ALL	1983	250.15	209.35	294.60	RW2
Malawi	ALL	1983	242.70	228.00	258.30	UN
Malawi	ALL	1984	242.80	231.12	254.44	IHME
Malawi	ALL	1984	252.50	207.06	301.42	RW2
Malawi	ALL	1984	244.60	230.30	260.10	UN
Malawi	ALL	1985	241.29	230.06	252.83	IHME
Malawi	ALL	1985	254.24	213.50	299.99	RW2
Malawi	ALL	1985	248.40	234.30	264.40	UN
Malawi	ALL	1986	239.43	228.72	250.57	IHME
Malawi	ALL	1986	254.19	216.36	295.61	RW2
Malawi	ALL	1986	252.00	237.40	268.30	UN
Malawi	ALL	1987	236.73	225.95	247.75	IHME
Malawi	ALL	1987	252.62	216.96	292.36	RW2
Malawi	ALL	1987	253.10	238.70	268.90	UN
Malawi	ALL	1988	233.05	221.99	244.01	IHME
Malawi	ALL	1988	249.31	212.43	290.81	RW2
Malawi	ALL	1988	251.80	237.40	267.10	UN
Malawi	ALL	1989	228.63	218.19	238.98	IHME
Malawi	ALL	1989	244.73	206.52	289.04	RW2
Malawi	ALL	1989	248.10	234.10	262.70	UN
Malawi	ALL	1990	224.22	214.23	234.60	IHME
Malawi	ALL	1990	238.35	200.17	280.62	RW2
Malawi	ALL	1990	242.40	229.10	256.90	UN
Malawi	ALL	1991	218.71	209.51	228.54	IHME
Malawi	ALL	1991	232.67	197.61	271.17	RW2
Malawi	ALL	1991	235.00	221.90	249.10	UN
Malawi	ALL	1992	212.71	203.55	222.62	IHME
Malawi	ALL	1992	227.10	193.73	264.12	RW2
Malawi	ALL	1992	226.80	213.90	239.90	UN
Malawi	ALL	1993	205.74	195.98	216.09	IHME
Malawi	ALL	1993	221.82	188.07	259.52	RW2
Malawi	ALL	1993	219.00	206.40	231.80	UN
Malawi	ALL	1994	199.18	190.01	208.21	IHME
Malawi	ALL	1994	216.53	181.03	256.61	RW2
Malawi	ALL	1994	212.60	200.60	225.30	UN
Malawi	ALL	1995	193.54	184.48	202.51	IHME
Malawi	ALL	1995	212.00	178.19	251.73	RW2
Malawi	ALL	1995	207.50	195.60	219.80	UN
Malawi	ALL	1996	189.35	180.81	198.67	IHME
Malawi	ALL	1996	205.63	174.17	242.37	RW2
Malawi	ALL	1996	203.00	191.60	215.00	UN
Malawi	ALL	1997	184.87	175.48	194.29	IHME
Malawi	ALL	1997	198.23	168.37	231.84	RW2
Malawi	ALL	1997	198.70	187.60	210.20	UN
Malawi	ALL	1998	180.13	171.47	189.74	IHME
Malawi	ALL	1998	189.70	159.82	224.10	RW2
Malawi	ALL	1998	193.10	182.00	204.70	UN
Malawi	ALL	1999	174.30	165.82	183.87	IHME
Malawi	ALL	1999	180.08	149.65	214.52	RW2
Malawi	ALL	1999	185.00	174.00	196.60	UN
Malawi	ALL	2000	166.84	158.71	175.22	IHME
Malawi	ALL	2000	169.34	140.23	201.77	RW2
Malawi	ALL	2000	174.40	163.70	185.70	UN
Malawi	ALL	2001	157.76	149.94	166.57	IHME
Malawi	ALL	2001	158.78	132.58	188.24	RW2
Malawi	ALL	2001	161.70	151.50	172.70	UN
Malawi	ALL	2002	148.08	139.18	157.36	IHME
Malawi	ALL	2002	148.28	124.71	175.50	RW2
Malawi	ALL	2002	148.00	138.30	158.40	UN
Malawi	ALL	2003	137.14	128.58	145.70	IHME
Malawi	ALL	2003	138.26	115.82	164.83	RW2
Malawi	ALL	2003	135.20	125.70	145.60	UN

Continued on next page

Country	Region	Year	Median	Lower	Upper	Method
Malawi	ALL	2004	128.13	120.31	136.12	IHME
Malawi	ALL	2004	128.67	105.98	155.82	RW2
Malawi	ALL	2004	124.60	115.10	134.70	UN
Malawi	ALL	2005	121.08	113.11	128.84	IHME
Malawi	ALL	2005	119.88	98.11	145.65	RW2
Malawi	ALL	2005	116.20	107.00	126.00	UN
Malawi	ALL	2006	116.15	108.35	123.23	IHME
Malawi	ALL	2006	111.94	92.63	134.57	RW2
Malawi	ALL	2006	109.90	101.10	119.30	UN
Malawi	ALL	2007	112.53	105.22	119.91	IHME
Malawi	ALL	2007	104.88	87.45	125.30	RW2
Malawi	ALL	2007	104.90	96.10	114.60	UN
Malawi	ALL	2008	109.33	102.30	117.08	IHME
Malawi	ALL	2008	98.71	81.58	119.01	RW2
Malawi	ALL	2008	100.00	90.50	110.70	UN
Malawi	ALL	2009	105.56	97.93	113.73	IHME
Malawi	ALL	2009	93.05	75.39	114.48	RW2
Malawi	ALL	2009	95.40	84.90	107.10	UN
Malawi	ALL	2010	100.88	93.46	109.28	IHME
Malawi	ALL	2010	88.21	70.43	111.00	RW2
Malawi	ALL	2010	90.90	79.40	104.40	UN
Malawi	ALL	2011	95.21	87.09	104.12	IHME
Malawi	ALL	2011	83.66	67.37	103.79	RW2
Malawi	ALL	2011	84.50	72.60	100.40	UN
Malawi	ALL	2012	90.01	81.40	99.35	IHME
Malawi	ALL	2012	79.39	64.77	96.98	RW2
Malawi	ALL	2012	77.30	64.80	95.70	UN
Malawi	ALL	2013	86.65	76.14	97.34	IHME
Malawi	ALL	2013	75.42	60.21	93.82	RW2
Malawi	ALL	2013	71.30	57.80	92.40	UN
Malawi	ALL	2014	84.78	73.18	97.84	IHME
Malawi	ALL	2014	71.56	52.09	97.39	RW2
Malawi	ALL	2014	66.90	51.60	90.60	UN
Malawi	ALL	2015	83.35	70.24	98.43	IHME
Malawi	ALL	2015	67.76	41.53	108.60	RW2
Malawi	ALL	2015	64.00	47.10	90.80	UN
Malawi	ALL	2016	64.40	33.14	122.24	RW2
Malawi	ALL	2017	60.93	25.50	140.05	RW2
Malawi	ALL	2018	57.74	19.41	163.94	RW2
Malawi	ALL	2019	54.64	13.94	190.38	RW2
Malawi	ALL	15-19	60.92	25.90	137.85	RW2
Malawi	CENTRAL REGION	1980	275.80	222.85	336.74	RW2
Malawi	CENTRAL REGION	1981	277.18	240.48	318.18	RW2
Malawi	CENTRAL REGION	1982	278.46	244.56	314.96	RW2
Malawi	CENTRAL REGION	1983	279.34	241.71	320.10	RW2
Malawi	CENTRAL REGION	1984	279.61	238.59	322.69	RW2
Malawi	CENTRAL REGION	1985	279.69	242.92	320.30	RW2
Malawi	CENTRAL REGION	1986	277.83	244.30	313.71	RW2
Malawi	CENTRAL REGION	1987	274.26	243.87	307.36	RW2
Malawi	CENTRAL REGION	1988	269.20	237.56	303.99	RW2
Malawi	CENTRAL REGION	1989	262.66	228.98	299.78	RW2
Malawi	CENTRAL REGION	1990	254.54	221.04	290.27	RW2
Malawi	CENTRAL REGION	1991	246.95	217.03	279.40	RW2
Malawi	CENTRAL REGION	1992	239.60	211.75	270.23	RW2
Malawi	CENTRAL REGION	1993	232.57	203.74	263.82	RW2
Malawi	CENTRAL REGION	1994	225.65	195.25	259.05	RW2
Malawi	CENTRAL REGION	1995	219.37	190.08	252.85	RW2
Malawi	CENTRAL REGION	1996	211.89	185.43	241.70	RW2
Malawi	CENTRAL REGION	1997	203.53	178.85	230.60	RW2
Malawi	CENTRAL REGION	1998	194.31	169.19	221.78	RW2
Malawi	CENTRAL REGION	1999	184.34	158.60	213.22	RW2
Malawi	CENTRAL REGION	2000	173.54	148.82	201.04	RW2
Malawi	CENTRAL REGION	2001	163.10	141.27	187.27	RW2

Continued on next page

Country	Region	Year	Median	Lower	Upper	Method
Malawi	CENTRAL REGION	2002	152.64	132.84	174.81	RW2
Malawi	CENTRAL REGION	2003	142.82	123.37	165.24	RW2
Malawi	CENTRAL REGION	2004	133.53	113.88	156.79	RW2
Malawi	CENTRAL REGION	2005	124.94	105.75	146.98	RW2
Malawi	CENTRAL REGION	2006	117.26	100.35	136.19	RW2
Malawi	CENTRAL REGION	2007	110.29	95.08	127.54	RW2
Malawi	CENTRAL REGION	2008	104.02	88.79	121.65	RW2
Malawi	CENTRAL REGION	2009	98.48	82.09	117.46	RW2
Malawi	CENTRAL REGION	2010	93.51	76.69	113.76	RW2
Malawi	CENTRAL REGION	2011	88.89	73.62	107.16	RW2
Malawi	CENTRAL REGION	2012	84.56	70.61	101.00	RW2
Malawi	CENTRAL REGION	2013	80.47	65.49	98.75	RW2
Malawi	CENTRAL REGION	2014	76.40	57.05	101.74	RW2
Malawi	CENTRAL REGION	2015	72.70	46.81	111.30	RW2
Malawi	CENTRAL REGION	2016	69.13	37.76	123.96	RW2
Malawi	CENTRAL REGION	2017	65.61	29.92	138.82	RW2
Malawi	CENTRAL REGION	2018	62.49	23.18	158.09	RW2
Malawi	CENTRAL REGION	2019	59.33	17.40	183.61	RW2
Malawi	NORTHERN REGION	1980	195.90	152.19	249.05	RW2
Malawi	NORTHERN REGION	1981	198.00	165.29	235.50	RW2
Malawi	NORTHERN REGION	1982	200.12	170.52	233.40	RW2
Malawi	NORTHERN REGION	1983	201.99	170.15	237.72	RW2
Malawi	NORTHERN REGION	1984	203.52	169.65	241.35	RW2
Malawi	NORTHERN REGION	1985	204.49	173.61	239.34	RW2
Malawi	NORTHERN REGION	1986	204.07	176.28	234.61	RW2
Malawi	NORTHERN REGION	1987	202.00	175.78	231.39	RW2
Malawi	NORTHERN REGION	1988	198.76	171.63	228.94	RW2
Malawi	NORTHERN REGION	1989	194.29	166.33	226.59	RW2
Malawi	NORTHERN REGION	1990	188.47	160.94	219.12	RW2
Malawi	NORTHERN REGION	1991	182.97	157.99	211.18	RW2
Malawi	NORTHERN REGION	1992	177.55	153.96	203.85	RW2
Malawi	NORTHERN REGION	1993	172.38	148.63	199.03	RW2
Malawi	NORTHERN REGION	1994	167.42	142.21	195.85	RW2
Malawi	NORTHERN REGION	1995	162.78	138.59	190.70	RW2
Malawi	NORTHERN REGION	1996	157.40	135.25	182.71	RW2
Malawi	NORTHERN REGION	1997	151.40	130.66	174.65	RW2
Malawi	NORTHERN REGION	1998	144.78	123.95	168.20	RW2
Malawi	NORTHERN REGION	1999	137.62	116.40	162.34	RW2
Malawi	NORTHERN REGION	2000	129.74	109.17	153.02	RW2
Malawi	NORTHERN REGION	2001	122.16	104.23	142.93	RW2
Malawi	NORTHERN REGION	2002	114.64	98.00	133.74	RW2
Malawi	NORTHERN REGION	2003	107.28	90.90	126.16	RW2
Malawi	NORTHERN REGION	2004	100.36	83.88	119.79	RW2
Malawi	NORTHERN REGION	2005	93.82	77.95	112.38	RW2
Malawi	NORTHERN REGION	2006	87.90	73.64	104.47	RW2
Malawi	NORTHERN REGION	2007	82.52	69.52	98.15	RW2
Malawi	NORTHERN REGION	2008	77.72	64.57	93.06	RW2
Malawi	NORTHERN REGION	2009	73.32	59.73	89.67	RW2
Malawi	NORTHERN REGION	2010	69.51	55.54	86.64	RW2
Malawi	NORTHERN REGION	2011	65.83	52.80	81.84	RW2
Malawi	NORTHERN REGION	2012	62.45	50.29	77.33	RW2
Malawi	NORTHERN REGION	2013	59.24	46.11	75.24	RW2
Malawi	NORTHERN REGION	2014	56.15	40.19	77.52	RW2
Malawi	NORTHERN REGION	2015	53.25	32.91	84.67	RW2
Malawi	NORTHERN REGION	2016	50.46	26.61	93.06	RW2
Malawi	NORTHERN REGION	2017	47.77	20.95	104.86	RW2
Malawi	NORTHERN REGION	2018	45.29	16.10	120.61	RW2
Malawi	NORTHERN REGION	2019	42.83	12.21	140.41	RW2
Malawi	SOUTHERN REGION	1980	223.89	178.89	276.02	RW2
Malawi	SOUTHERN REGION	1981	228.09	196.57	263.03	RW2
Malawi	SOUTHERN REGION	1982	232.25	202.77	264.38	RW2
Malawi	SOUTHERN REGION	1983	235.96	202.78	272.39	RW2
Malawi	SOUTHERN REGION	1984	239.62	203.35	278.83	RW2

Continued on next page

Country	Region	Year	Median	Lower	Upper	Method
Malawi	SOUTHERN REGION	1985	242.98	209.64	280.06	RW2
Malawi	SOUTHERN REGION	1986	244.78	214.34	277.82	RW2
Malawi	SOUTHERN REGION	1987	245.36	216.13	276.93	RW2
Malawi	SOUTHERN REGION	1988	244.44	214.27	277.19	RW2
Malawi	SOUTHERN REGION	1989	242.38	210.66	278.02	RW2
Malawi	SOUTHERN REGION	1990	238.54	206.65	272.49	RW2
Malawi	SOUTHERN REGION	1991	234.91	206.36	266.03	RW2
Malawi	SOUTHERN REGION	1992	231.07	204.04	260.55	RW2
Malawi	SOUTHERN REGION	1993	226.86	198.53	257.84	RW2
Malawi	SOUTHERN REGION	1994	222.33	192.19	255.24	RW2
Malawi	SOUTHERN REGION	1995	217.55	188.94	250.23	RW2
Malawi	SOUTHERN REGION	1996	211.10	184.75	240.19	RW2
Malawi	SOUTHERN REGION	1997	203.22	178.69	230.26	RW2
Malawi	SOUTHERN REGION	1998	194.02	169.23	221.38	RW2
Malawi	SOUTHERN REGION	1999	183.49	157.52	212.35	RW2
Malawi	SOUTHERN REGION	2000	172.24	147.46	198.94	RW2
Malawi	SOUTHERN REGION	2001	161.05	139.43	184.66	RW2
Malawi	SOUTHERN REGION	2002	150.20	130.93	171.86	RW2
Malawi	SOUTHERN REGION	2003	139.81	120.66	161.23	RW2
Malawi	SOUTHERN REGION	2004	130.20	110.81	152.76	RW2
Malawi	SOUTHERN REGION	2005	121.22	102.78	142.51	RW2
Malawi	SOUTHERN REGION	2006	113.16	96.85	131.65	RW2
Malawi	SOUTHERN REGION	2007	105.86	91.31	122.27	RW2
Malawi	SOUTHERN REGION	2008	99.20	84.83	115.86	RW2
Malawi	SOUTHERN REGION	2009	93.21	77.93	110.88	RW2
Malawi	SOUTHERN REGION	2010	87.81	71.91	106.89	RW2
Malawi	SOUTHERN REGION	2011	82.76	68.46	100.00	RW2
Malawi	SOUTHERN REGION	2012	78.02	65.04	93.25	RW2
Malawi	SOUTHERN REGION	2013	73.55	59.60	90.29	RW2
Malawi	SOUTHERN REGION	2014	69.30	51.51	92.49	RW2
Malawi	SOUTHERN REGION	2015	65.30	41.58	100.70	RW2
Malawi	SOUTHERN REGION	2016	61.47	33.29	110.43	RW2
Malawi	SOUTHERN REGION	2017	57.80	26.13	123.06	RW2
Malawi	SOUTHERN REGION	2018	54.44	20.08	140.08	RW2
Malawi	SOUTHERN REGION	2019	51.25	14.98	161.04	RW2
Mali	ALL	1980	276.41	268.72	284.06	IHME
Mali	ALL	1980	324.53	251.88	405.82	RW2
Mali	ALL	1980	324.70	303.10	347.70	UN
Mali	ALL	1981	270.49	263.12	277.90	IHME
Mali	ALL	1981	316.41	265.38	371.52	RW2
Mali	ALL	1981	317.00	295.90	339.10	UN
Mali	ALL	1982	264.51	257.34	272.00	IHME
Mali	ALL	1982	308.49	262.28	358.80	RW2
Mali	ALL	1982	308.90	288.80	329.80	UN
Mali	ALL	1983	259.20	252.36	266.72	IHME
Mali	ALL	1983	300.38	249.52	356.60	RW2
Mali	ALL	1983	300.70	281.40	321.00	UN
Mali	ALL	1984	254.20	247.42	261.56	IHME
Mali	ALL	1984	292.94	237.74	354.30	RW2
Mali	ALL	1984	292.60	274.00	312.30	UN
Mali	ALL	1985	249.07	242.75	256.48	IHME
Mali	ALL	1985	285.13	235.65	339.89	RW2
Mali	ALL	1985	285.00	267.00	303.90	UN
Mali	ALL	1986	243.91	237.74	251.17	IHME
Mali	ALL	1986	277.93	233.26	326.83	RW2
Mali	ALL	1986	277.90	260.30	296.50	UN
Mali	ALL	1987	239.35	233.09	246.43	IHME
Mali	ALL	1987	271.15	229.79	317.65	RW2
Mali	ALL	1987	271.30	254.30	289.50	UN
Mali	ALL	1988	235.93	229.59	242.67	IHME
Mali	ALL	1988	264.67	221.83	312.96	RW2
Mali	ALL	1988	265.20	248.80	283.10	UN
Mali	ALL	1989	232.35	226.11	238.91	IHME

Continued on next page

Country	Region	Year	Median	Lower	Upper	Method
Mali	ALL	1989	258.86	214.24	310.25	RW2
Mali	ALL	1989	259.50	243.20	277.00	UN
Mali	ALL	1990	228.96	222.75	235.13	IHME
Mali	ALL	1990	253.48	209.53	303.67	RW2
Mali	ALL	1990	254.40	238.20	271.20	UN
Mali	ALL	1991	225.37	218.99	231.70	IHME
Mali	ALL	1991	249.42	208.79	294.77	RW2
Mali	ALL	1991	250.00	233.80	266.60	UN
Mali	ALL	1992	222.29	216.15	228.50	IHME
Mali	ALL	1992	246.33	207.16	289.87	RW2
Mali	ALL	1992	246.60	230.60	262.90	UN
Mali	ALL	1993	219.13	212.96	225.54	IHME
Mali	ALL	1993	244.14	203.86	288.96	RW2
Mali	ALL	1993	243.90	228.10	260.30	UN
Mali	ALL	1994	216.28	210.04	222.83	IHME
Mali	ALL	1994	242.33	199.21	290.95	RW2
Mali	ALL	1994	242.00	226.10	258.20	UN
Mali	ALL	1995	212.94	206.88	219.18	IHME
Mali	ALL	1995	241.48	199.65	290.39	RW2
Mali	ALL	1995	240.00	224.30	256.60	UN
Mali	ALL	1996	209.54	203.00	216.12	IHME
Mali	ALL	1996	239.13	199.68	285.06	RW2
Mali	ALL	1996	237.90	221.90	255.10	UN
Mali	ALL	1997	205.67	199.19	212.06	IHME
Mali	ALL	1997	235.81	197.87	278.49	RW2
Mali	ALL	1997	235.30	219.20	253.00	UN
Mali	ALL	1998	203.82	196.61	211.64	IHME
Mali	ALL	1998	231.28	192.45	276.03	RW2
Mali	ALL	1998	231.70	215.30	249.60	UN
Mali	ALL	1999	196.54	190.04	202.93	IHME
Mali	ALL	1999	225.34	184.62	271.25	RW2
Mali	ALL	1999	226.40	210.00	244.50	UN
Mali	ALL	2000	192.60	186.09	199.06	IHME
Mali	ALL	2000	217.84	177.78	262.78	RW2
Mali	ALL	2000	219.60	203.30	237.30	UN
Mali	ALL	2001	187.45	180.84	194.17	IHME
Mali	ALL	2001	209.84	172.98	251.35	RW2
Mali	ALL	2001	211.20	195.30	228.70	UN
Mali	ALL	2002	182.45	175.78	189.41	IHME
Mali	ALL	2002	201.15	167.35	239.90	RW2
Mali	ALL	2002	201.80	186.10	219.00	UN
Mali	ALL	2003	177.36	170.73	184.63	IHME
Mali	ALL	2003	192.20	158.90	231.15	RW2
Mali	ALL	2003	191.80	175.80	209.20	UN
Mali	ALL	2004	172.36	165.51	179.73	IHME
Mali	ALL	2004	182.82	147.29	224.82	RW2
Mali	ALL	2004	181.70	164.90	200.00	UN
Mali	ALL	2005	167.49	160.44	174.87	IHME
Mali	ALL	2005	173.63	136.40	218.10	RW2
Mali	ALL	2005	171.80	153.50	192.20	UN
Mali	ALL	2006	162.97	155.54	170.61	IHME
Mali	ALL	2006	164.49	130.86	204.29	RW2
Mali	ALL	2006	162.90	142.40	185.60	UN
Mali	ALL	2007	158.92	150.94	166.90	IHME
Mali	ALL	2007	155.70	126.35	190.27	RW2
Mali	ALL	2007	155.10	131.90	179.80	UN
Mali	ALL	2008	155.00	146.59	163.15	IHME
Mali	ALL	2008	147.41	117.02	184.25	RW2
Mali	ALL	2008	148.30	122.30	176.70	UN
Mali	ALL	2009	151.15	141.70	160.63	IHME
Mali	ALL	2009	139.13	100.01	191.15	RW2
Mali	ALL	2009	142.00	113.30	174.80	UN
Mali	ALL	2010	147.35	137.15	157.84	IHME

Continued on next page

Country	Region	Year	Median	Lower	Upper	Method
Mali	ALL	2010	131.35	78.52	213.99	RW2
Mali	ALL	2010	136.60	105.30	174.30	UN
Mali	ALL	2011	143.32	132.27	155.08	IHME
Mali	ALL	2011	124.09	60.15	239.19	RW2
Mali	ALL	2011	131.70	97.50	173.80	UN
Mali	ALL	2012	139.95	128.24	153.13	IHME
Mali	ALL	2012	116.94	44.47	273.41	RW2
Mali	ALL	2012	127.00	90.90	174.20	UN
Mali	ALL	2013	136.92	123.74	152.15	IHME
Mali	ALL	2013	110.57	32.43	314.03	RW2
Mali	ALL	2013	122.70	84.90	174.50	UN
Mali	ALL	2014	133.52	119.54	150.69	IHME
Mali	ALL	2014	104.11	22.97	363.69	RW2
Mali	ALL	2014	118.30	78.60	175.30	UN
Mali	ALL	2015	130.20	115.37	148.88	IHME
Mali	ALL	2015	97.72	16.18	417.03	RW2
Mali	ALL	2015	114.70	73.00	175.90	UN
Mali	ALL	2016	92.66	11.24	485.06	RW2
Mali	ALL	2017	86.74	7.42	553.96	RW2
Mali	ALL	2018	81.60	5.00	629.15	RW2
Mali	ALL	2019	76.53	3.00	688.57	RW2
Mali	ALL	15-19	86.74	7.49	548.82	RW2
Mali	BAMAKO	1980	193.50	148.44	248.90	RW2
Mali	BAMAKO	1981	187.34	155.61	223.88	RW2
Mali	BAMAKO	1982	181.38	153.23	213.52	RW2
Mali	BAMAKO	1983	175.53	145.40	210.52	RW2
Mali	BAMAKO	1984	169.99	138.63	206.77	RW2
Mali	BAMAKO	1985	164.46	136.17	196.86	RW2
Mali	BAMAKO	1986	159.40	134.67	187.03	RW2
Mali	BAMAKO	1987	154.39	131.54	180.57	RW2
Mali	BAMAKO	1988	149.94	126.47	176.76	RW2
Mali	BAMAKO	1989	145.93	121.78	174.67	RW2
Mali	BAMAKO	1990	142.25	118.68	169.78	RW2
Mali	BAMAKO	1991	139.59	118.16	164.88	RW2
Mali	BAMAKO	1992	137.70	117.25	161.32	RW2
Mali	BAMAKO	1993	136.48	115.38	160.77	RW2
Mali	BAMAKO	1994	135.65	112.63	162.18	RW2
Mali	BAMAKO	1995	135.24	112.81	162.11	RW2
Mali	BAMAKO	1996	133.87	112.97	158.51	RW2
Mali	BAMAKO	1997	131.56	111.65	154.47	RW2
Mali	BAMAKO	1998	128.22	107.77	151.83	RW2
Mali	BAMAKO	1999	123.91	102.56	149.48	RW2
Mali	BAMAKO	2000	118.34	97.03	142.88	RW2
Mali	BAMAKO	2001	112.87	94.03	135.06	RW2
Mali	BAMAKO	2002	107.20	89.42	127.90	RW2
Mali	BAMAKO	2003	101.43	83.27	122.74	RW2
Mali	BAMAKO	2004	95.87	76.54	119.26	RW2
Mali	BAMAKO	2005	90.51	70.21	115.53	RW2
Mali	BAMAKO	2006	85.37	65.85	109.33	RW2
Mali	BAMAKO	2007	80.45	61.71	104.10	RW2
Mali	BAMAKO	2008	75.85	55.72	101.20	RW2
Mali	BAMAKO	2009	71.34	47.98	104.01	RW2
Mali	BAMAKO	2010	67.27	38.52	113.69	RW2
Mali	BAMAKO	2011	63.23	30.36	126.37	RW2
Mali	BAMAKO	2012	59.56	23.56	143.09	RW2
Mali	BAMAKO	2013	56.03	17.31	163.17	RW2
Mali	BAMAKO	2014	52.57	12.56	193.27	RW2
Mali	BAMAKO	2015	49.57	9.08	228.21	RW2
Mali	BAMAKO	2016	46.53	6.44	266.43	RW2
Mali	BAMAKO	2017	43.62	4.47	315.31	RW2
Mali	BAMAKO	2018	41.04	3.04	375.14	RW2
Mali	BAMAKO	2019	38.39	2.06	441.57	RW2
Mali	KAYES, KOULIKORO	1980	320.30	257.43	389.54	RW2

Continued on next page

Country	Region	Year	Median	Lower	Upper	Method
Mali	KAYES, KOULIKORO	1981	311.41	269.42	356.37	RW2
Mali	KAYES, KOULIKORO	1982	302.57	264.50	343.53	RW2
Mali	KAYES, KOULIKORO	1983	293.57	251.70	339.68	RW2
Mali	KAYES, KOULIKORO	1984	285.19	240.88	333.79	RW2
Mali	KAYES, KOULIKORO	1985	276.91	236.80	320.77	RW2
Mali	KAYES, KOULIKORO	1986	268.94	233.67	306.97	RW2
Mali	KAYES, KOULIKORO	1987	261.44	228.49	297.17	RW2
Mali	KAYES, KOULIKORO	1988	254.34	220.65	291.10	RW2
Mali	KAYES, KOULIKORO	1989	248.12	212.83	287.93	RW2
Mali	KAYES, KOULIKORO	1990	242.27	207.32	280.36	RW2
Mali	KAYES, KOULIKORO	1991	238.22	207.12	272.72	RW2
Mali	KAYES, KOULIKORO	1992	235.60	206.04	268.01	RW2
Mali	KAYES, KOULIKORO	1993	234.01	202.43	268.30	RW2
Mali	KAYES, KOULIKORO	1994	233.25	198.90	270.29	RW2
Mali	KAYES, KOULIKORO	1995	233.28	200.38	271.13	RW2
Mali	KAYES, KOULIKORO	1996	231.91	201.08	265.93	RW2
Mali	KAYES, KOULIKORO	1997	229.19	199.96	261.29	RW2
Mali	KAYES, KOULIKORO	1998	224.90	194.59	258.37	RW2
Mali	KAYES, KOULIKORO	1999	218.76	185.92	255.39	RW2
Mali	KAYES, KOULIKORO	2000	211.07	178.34	246.11	RW2
Mali	KAYES, KOULIKORO	2001	202.98	173.76	234.81	RW2
Mali	KAYES, KOULIKORO	2002	194.59	168.01	224.48	RW2
Mali	KAYES, KOULIKORO	2003	185.95	158.31	216.91	RW2
Mali	KAYES, KOULIKORO	2004	177.53	147.55	212.44	RW2
Mali	KAYES, KOULIKORO	2005	169.21	137.65	206.58	RW2
Mali	KAYES, KOULIKORO	2006	161.17	131.54	195.79	RW2
Mali	KAYES, KOULIKORO	2007	153.42	126.21	185.03	RW2
Mali	KAYES, KOULIKORO	2008	145.82	116.62	181.17	RW2
Mali	KAYES, KOULIKORO	2009	138.63	101.16	186.78	RW2
Mali	KAYES, KOULIKORO	2010	131.63	81.41	205.48	RW2
Mali	KAYES, KOULIKORO	2011	125.05	65.34	227.48	RW2
Mali	KAYES, KOULIKORO	2012	118.69	50.59	253.66	RW2
Mali	KAYES, KOULIKORO	2013	112.53	38.15	288.70	RW2
Mali	KAYES, KOULIKORO	2014	106.63	28.13	330.41	RW2
Mali	KAYES, KOULIKORO	2015	101.34	20.22	380.12	RW2
Mali	KAYES, KOULIKORO	2016	95.93	14.40	434.66	RW2
Mali	KAYES, KOULIKORO	2017	90.62	10.24	491.57	RW2
Mali	KAYES, KOULIKORO	2018	85.96	7.14	557.57	RW2
Mali	KAYES, KOULIKORO	2019	81.46	4.76	623.17	RW2
Mali	MOPTI, TOMBOUCTOU, GAO, KIDAL	1980	430.33	355.60	507.66	RW2
Mali	MOPTI, TOMBOUCTOU, GAO, KIDAL	1981	418.74	367.57	471.54	RW2
Mali	MOPTI, TOMBOUCTOU, GAO, KIDAL	1982	406.85	361.13	453.69	RW2
Mali	MOPTI, TOMBOUCTOU, GAO, KIDAL	1983	395.09	344.70	447.60	RW2
Mali	MOPTI, TOMBOUCTOU, GAO, KIDAL	1984	383.32	329.10	439.86	RW2
Mali	MOPTI, TOMBOUCTOU, GAO, KIDAL	1985	371.74	323.35	422.48	RW2
Mali	MOPTI, TOMBOUCTOU, GAO, KIDAL	1986	360.04	317.40	403.99	RW2
Mali	MOPTI, TOMBOUCTOU, GAO, KIDAL	1987	348.26	309.03	389.81	RW2
Mali	MOPTI, TOMBOUCTOU, GAO, KIDAL	1988	336.69	296.10	380.61	RW2
Mali	MOPTI, TOMBOUCTOU, GAO, KIDAL	1989	325.56	282.99	372.37	RW2
Mali	MOPTI, TOMBOUCTOU, GAO, KIDAL	1990	314.60	272.86	359.74	RW2
Mali	MOPTI, TOMBOUCTOU, GAO, KIDAL	1991	305.59	268.35	346.21	RW2
Mali	MOPTI, TOMBOUCTOU, GAO, KIDAL	1992	297.73	262.28	335.89	RW2
Mali	MOPTI, TOMBOUCTOU, GAO, KIDAL	1993	290.93	254.26	329.97	RW2
Mali	MOPTI, TOMBOUCTOU, GAO, KIDAL	1994	285.08	245.10	326.71	RW2
Mali	MOPTI, TOMBOUCTOU, GAO, KIDAL	1995	279.94	241.63	322.86	RW2
Mali	MOPTI, TOMBOUCTOU, GAO, KIDAL	1996	273.02	237.80	311.44	RW2
Mali	MOPTI, TOMBOUCTOU, GAO, KIDAL	1997	264.46	231.45	300.47	RW2
Mali	MOPTI, TOMBOUCTOU, GAO, KIDAL	1998	254.15	221.15	290.56	RW2
Mali	MOPTI, TOMBOUCTOU, GAO, KIDAL	1999	242.28	206.53	281.03	RW2
Mali	MOPTI, TOMBOUCTOU, GAO, KIDAL	2000	228.60	193.35	266.25	RW2
Mali	MOPTI, TOMBOUCTOU, GAO, KIDAL	2001	215.31	184.03	248.96	RW2
Mali	MOPTI, TOMBOUCTOU, GAO, KIDAL	2002	202.07	174.04	233.14	RW2
Mali	MOPTI, TOMBOUCTOU, GAO, KIDAL	2003	189.25	161.36	221.33	RW2

Continued on next page

Country	Region	Year	Median	Lower	Upper	Method
Mali	MOPTI, TOMBOUCTOU, GAO, KIDAL	2004	177.21	147.28	211.83	RW2
Mali	MOPTI, TOMBOUCTOU, GAO, KIDAL	2005	165.89	134.99	202.54	RW2
Mali	MOPTI, TOMBOUCTOU, GAO, KIDAL	2006	155.22	127.22	188.49	RW2
Mali	MOPTI, TOMBOUCTOU, GAO, KIDAL	2007	145.03	119.27	175.15	RW2
Mali	MOPTI, TOMBOUCTOU, GAO, KIDAL	2008	135.52	108.04	168.81	RW2
Mali	MOPTI, TOMBOUCTOU, GAO, KIDAL	2009	126.31	91.92	171.52	RW2
Mali	MOPTI, TOMBOUCTOU, GAO, KIDAL	2010	117.88	73.20	185.80	RW2
Mali	MOPTI, TOMBOUCTOU, GAO, KIDAL	2011	109.67	56.88	202.88	RW2
Mali	MOPTI, TOMBOUCTOU, GAO, KIDAL	2012	102.10	43.14	224.90	RW2
Mali	MOPTI, TOMBOUCTOU, GAO, KIDAL	2013	95.15	31.96	256.24	RW2
Mali	MOPTI, TOMBOUCTOU, GAO, KIDAL	2014	88.65	23.01	288.22	RW2
Mali	MOPTI, TOMBOUCTOU, GAO, KIDAL	2015	82.43	16.33	328.47	RW2
Mali	MOPTI, TOMBOUCTOU, GAO, KIDAL	2016	76.30	11.35	374.05	RW2
Mali	MOPTI, TOMBOUCTOU, GAO, KIDAL	2017	71.14	7.84	429.54	RW2
Mali	MOPTI, TOMBOUCTOU, GAO, KIDAL	2018	65.65	5.23	491.06	RW2
Mali	MOPTI, TOMBOUCTOU, GAO, KIDAL	2019	61.04	3.46	560.20	RW2
Mali	SIKASSO, SEGOU	1980	299.87	240.15	367.88	RW2
Mali	SIKASSO, SEGOU	1981	294.96	254.66	338.79	RW2
Mali	SIKASSO, SEGOU	1982	290.14	253.46	328.85	RW2
Mali	SIKASSO, SEGOU	1983	285.13	244.30	330.09	RW2
Mali	SIKASSO, SEGOU	1984	280.41	236.83	328.34	RW2
Mali	SIKASSO, SEGOU	1985	275.72	235.31	318.45	RW2
Mali	SIKASSO, SEGOU	1986	270.93	235.86	309.02	RW2
Mali	SIKASSO, SEGOU	1987	266.45	234.07	301.53	RW2
Mali	SIKASSO, SEGOU	1988	262.17	228.45	299.51	RW2
Mali	SIKASSO, SEGOU	1989	258.33	222.19	299.59	RW2
Mali	SIKASSO, SEGOU	1990	254.88	218.88	294.88	RW2
Mali	SIKASSO, SEGOU	1991	253.24	220.34	288.76	RW2
Mali	SIKASSO, SEGOU	1992	252.60	221.35	286.56	RW2
Mali	SIKASSO, SEGOU	1993	253.22	220.65	289.11	RW2
Mali	SIKASSO, SEGOU	1994	254.67	218.19	294.06	RW2
Mali	SIKASSO, SEGOU	1995	256.87	220.86	298.25	RW2
Mali	SIKASSO, SEGOU	1996	257.40	223.95	293.97	RW2
Mali	SIKASSO, SEGOU	1997	256.46	224.41	291.91	RW2
Mali	SIKASSO, SEGOU	1998	253.79	220.44	290.58	RW2
Mali	SIKASSO, SEGOU	1999	249.05	213.24	288.72	RW2
Mali	SIKASSO, SEGOU	2000	242.23	206.26	281.59	RW2
Mali	SIKASSO, SEGOU	2001	235.15	202.42	270.90	RW2
Mali	SIKASSO, SEGOU	2002	227.42	197.45	260.86	RW2
Mali	SIKASSO, SEGOU	2003	219.50	188.58	254.26	RW2
Mali	SIKASSO, SEGOU	2004	211.67	177.68	249.69	RW2
Mali	SIKASSO, SEGOU	2005	203.99	167.95	245.40	RW2
Mali	SIKASSO, SEGOU	2006	196.32	163.71	234.36	RW2
Mali	SIKASSO, SEGOU	2007	189.05	159.64	223.01	RW2
Mali	SIKASSO, SEGOU	2008	181.95	149.68	219.42	RW2
Mali	SIKASSO, SEGOU	2009	174.76	131.66	228.21	RW2
Mali	SIKASSO, SEGOU	2010	168.08	108.01	252.41	RW2
Mali	SIKASSO, SEGOU	2011	161.43	87.62	280.07	RW2
Mali	SIKASSO, SEGOU	2012	154.97	68.68	314.69	RW2
Mali	SIKASSO, SEGOU	2013	148.77	52.75	355.96	RW2
Mali	SIKASSO, SEGOU	2014	142.29	38.76	402.69	RW2
Mali	SIKASSO, SEGOU	2015	137.02	28.80	459.95	RW2
Mali	SIKASSO, SEGOU	2016	131.04	20.49	518.50	RW2
Mali	SIKASSO, SEGOU	2017	126.17	14.66	583.67	RW2
Mali	SIKASSO, SEGOU	2018	120.25	10.30	644.77	RW2
Mali	SIKASSO, SEGOU	2019	115.92	7.21	707.26	RW2
Morocco	ALL	1980	124.28	120.41	128.36	IHME
Morocco	ALL	1980	133.68	90.47	192.39	RW2
Morocco	ALL	1980	133.80	126.40	141.80	UN
Morocco	ALL	1981	117.60	113.73	121.58	IHME
Morocco	ALL	1981	126.99	97.01	163.87	RW2
Morocco	ALL	1981	127.20	120.00	134.80	UN
Morocco	ALL	1982	110.84	107.13	114.74	IHME

Continued on next page

Country	Region	Year	Median	Lower	Upper	Method
Morocco	ALL	1982	120.62	94.35	152.98	RW2
Morocco	ALL	1982	120.80	114.00	127.90	UN
Morocco	ALL	1983	104.53	100.97	108.22	IHME
Morocco	ALL	1983	114.36	86.30	150.05	RW2
Morocco	ALL	1983	114.60	108.10	121.40	UN
Morocco	ALL	1984	98.56	95.07	102.21	IHME
Morocco	ALL	1984	108.68	79.20	146.99	RW2
Morocco	ALL	1984	108.60	102.40	115.20	UN
Morocco	ALL	1985	92.89	89.67	96.34	IHME
Morocco	ALL	1985	102.96	77.07	136.31	RW2
Morocco	ALL	1985	103.00	96.90	109.30	UN
Morocco	ALL	1986	87.53	84.50	90.90	IHME
Morocco	ALL	1986	97.78	75.00	126.41	RW2
Morocco	ALL	1986	97.70	91.80	103.80	UN
Morocco	ALL	1987	82.78	79.73	85.92	IHME
Morocco	ALL	1987	92.97	72.44	119.07	RW2
Morocco	ALL	1987	92.70	87.10	98.60	UN
Morocco	ALL	1988	78.14	75.08	81.12	IHME
Morocco	ALL	1988	88.39	67.64	114.65	RW2
Morocco	ALL	1988	88.10	82.70	93.90	UN
Morocco	ALL	1989	73.96	70.99	76.81	IHME
Morocco	ALL	1989	84.13	63.14	111.30	RW2
Morocco	ALL	1989	84.00	78.60	89.50	UN
Morocco	ALL	1990	70.10	67.20	72.97	IHME
Morocco	ALL	1990	80.20	60.09	106.56	RW2
Morocco	ALL	1990	80.10	74.90	85.50	UN
Morocco	ALL	1991	66.51	63.65	69.47	IHME
Morocco	ALL	1991	76.47	58.26	99.44	RW2
Morocco	ALL	1991	76.50	71.40	81.70	UN
Morocco	ALL	1992	63.33	60.54	66.32	IHME
Morocco	ALL	1992	72.96	56.01	94.21	RW2
Morocco	ALL	1992	73.00	68.20	78.10	UN
Morocco	ALL	1993	60.45	57.62	63.38	IHME
Morocco	ALL	1993	69.67	53.00	91.02	RW2
Morocco	ALL	1993	69.70	64.90	74.60	UN
Morocco	ALL	1994	57.60	54.63	60.51	IHME
Morocco	ALL	1994	66.49	49.51	89.16	RW2
Morocco	ALL	1994	66.40	61.80	71.30	UN
Morocco	ALL	1995	55.20	52.26	58.11	IHME
Morocco	ALL	1995	63.55	47.17	85.17	RW2
Morocco	ALL	1995	63.30	58.70	68.10	UN
Morocco	ALL	1996	52.54	49.53	55.59	IHME
Morocco	ALL	1996	60.66	45.72	80.49	RW2
Morocco	ALL	1996	60.30	55.90	65.00	UN
Morocco	ALL	1997	50.24	47.17	53.50	IHME
Morocco	ALL	1997	57.90	44.10	75.77	RW2
Morocco	ALL	1997	57.50	53.10	62.10	UN
Morocco	ALL	1998	47.94	44.93	51.24	IHME
Morocco	ALL	1998	55.29	41.53	73.76	RW2
Morocco	ALL	1998	54.80	50.40	59.40	UN
Morocco	ALL	1999	45.84	42.66	49.39	IHME
Morocco	ALL	1999	52.75	38.47	71.68	RW2
Morocco	ALL	1999	52.30	48.00	56.80	UN
Morocco	ALL	2000	43.85	40.54	47.50	IHME
Morocco	ALL	2000	50.29	35.90	69.88	RW2
Morocco	ALL	2000	50.00	45.70	54.50	UN
Morocco	ALL	2001	41.97	38.49	45.66	IHME
Morocco	ALL	2001	47.97	34.78	65.69	RW2
Morocco	ALL	2001	47.90	43.60	52.20	UN
Morocco	ALL	2002	40.23	36.65	44.09	IHME
Morocco	ALL	2002	45.72	34.05	61.16	RW2
Morocco	ALL	2002	45.80	41.60	50.20	UN
Morocco	ALL	2003	38.48	34.48	42.67	IHME

Continued on next page

Country	Region	Year	Median	Lower	Upper	Method
Morocco	ALL	2003	43.62	31.47	60.19	RW2
Morocco	ALL	2003	43.90	39.70	48.20	UN
Morocco	ALL	2004	36.88	32.69	41.38	IHME
Morocco	ALL	2004	41.47	25.96	65.41	RW2
Morocco	ALL	2004	42.10	38.00	46.50	UN
Morocco	ALL	2005	35.24	30.74	40.01	IHME
Morocco	ALL	2005	39.57	19.32	79.33	RW2
Morocco	ALL	2005	40.40	36.20	44.80	UN
Morocco	ALL	2006	33.70	29.09	38.60	IHME
Morocco	ALL	2006	37.59	14.15	95.69	RW2
Morocco	ALL	2006	38.80	34.50	43.10	UN
Morocco	ALL	2007	32.24	27.56	37.27	IHME
Morocco	ALL	2007	35.75	10.10	118.46	RW2
Morocco	ALL	2007	37.30	32.80	41.70	UN
Morocco	ALL	2008	30.89	26.23	36.15	IHME
Morocco	ALL	2008	34.29	7.04	151.41	RW2
Morocco	ALL	2008	35.80	31.20	40.50	UN
Morocco	ALL	2009	29.56	24.77	34.99	IHME
Morocco	ALL	2009	32.40	4.75	194.23	RW2
Morocco	ALL	2009	34.50	29.60	39.50	UN
Morocco	ALL	2010	28.33	23.61	33.86	IHME
Morocco	ALL	2010	30.75	3.19	247.72	RW2
Morocco	ALL	2010	33.10	27.90	38.50	UN
Morocco	ALL	2011	27.17	22.42	32.73	IHME
Morocco	ALL	2011	29.39	1.96	318.08	RW2
Morocco	ALL	2011	31.90	26.40	37.80	UN
Morocco	ALL	2012	25.91	21.24	31.42	IHME
Morocco	ALL	2012	27.88	1.17	407.63	RW2
Morocco	ALL	2012	30.70	25.00	37.10	UN
Morocco	ALL	2013	24.68	20.15	30.02	IHME
Morocco	ALL	2013	26.75	0.73	501.11	RW2
Morocco	ALL	2013	29.70	23.50	36.70	UN
Morocco	ALL	2014	23.54	19.04	28.91	IHME
Morocco	ALL	2014	25.38	0.44	602.14	RW2
Morocco	ALL	2014	28.60	22.00	36.50	UN
Morocco	ALL	2015	22.44	18.00	27.92	IHME
Morocco	ALL	2015	23.94	0.27	689.10	RW2
Morocco	ALL	2015	27.60	20.70	36.60	UN
Morocco	ALL	2016	23.15	0.17	782.55	RW2
Morocco	ALL	2017	21.75	0.09	850.67	RW2
Morocco	ALL	2018	20.68	0.06	905.44	RW2
Morocco	ALL	2019	19.56	0.03	934.19	RW2
Morocco	ALL	15-19	21.74	0.09	845.96	RW2
Morocco	CENTRE	1980	93.82	66.57	131.46	RW2
Morocco	CENTRE	1981	89.10	70.54	112.56	RW2
Morocco	CENTRE	1982	84.63	68.46	104.27	RW2
Morocco	CENTRE	1983	80.26	63.12	101.50	RW2
Morocco	CENTRE	1984	76.16	58.68	98.65	RW2
Morocco	CENTRE	1985	72.32	56.67	91.73	RW2
Morocco	CENTRE	1986	68.69	55.27	85.03	RW2
Morocco	CENTRE	1987	65.30	52.95	80.02	RW2
Morocco	CENTRE	1988	62.19	49.86	77.05	RW2
Morocco	CENTRE	1989	59.31	46.59	74.89	RW2
Morocco	CENTRE	1990	56.67	44.25	71.61	RW2
Morocco	CENTRE	1991	54.35	42.98	68.04	RW2
Morocco	CENTRE	1992	52.26	41.75	65.17	RW2
Morocco	CENTRE	1993	50.46	39.96	63.56	RW2
Morocco	CENTRE	1994	48.78	37.67	62.42	RW2
Morocco	CENTRE	1995	47.40	36.53	61.24	RW2
Morocco	CENTRE	1996	46.01	35.82	59.12	RW2
Morocco	CENTRE	1997	44.59	34.88	56.99	RW2
Morocco	CENTRE	1998	43.28	33.26	56.12	RW2
Morocco	CENTRE	1999	41.86	31.32	55.57	RW2

Continued on next page

Country	Region	Year	Median	Lower	Upper	Method
Morocco	CENTRE	2000	40.42	29.71	54.72	RW2
Morocco	CENTRE	2001	39.03	28.84	52.80	RW2
Morocco	CENTRE	2002	37.75	27.97	50.94	RW2
Morocco	CENTRE	2003	36.38	25.80	51.18	RW2
Morocco	CENTRE	2004	35.18	22.27	55.26	RW2
Morocco	CENTRE	2005	34.02	17.76	64.45	RW2
Morocco	CENTRE	2006	32.78	13.76	76.04	RW2
Morocco	CENTRE	2007	31.57	10.30	91.18	RW2
Morocco	CENTRE	2008	30.69	7.58	113.45	RW2
Morocco	CENTRE	2009	29.61	5.37	145.31	RW2
Morocco	CENTRE	2010	28.68	3.92	184.03	RW2
Morocco	CENTRE	2011	27.39	2.57	233.88	RW2
Morocco	CENTRE	2012	26.47	1.75	292.53	RW2
Morocco	CENTRE	2013	25.62	1.16	369.74	RW2
Morocco	CENTRE	2014	24.87	0.75	453.67	RW2
Morocco	CENTRE	2015	23.94	0.50	551.62	RW2
Morocco	CENTRE	2016	23.18	0.30	632.80	RW2
Morocco	CENTRE	2017	22.30	0.19	721.65	RW2
Morocco	CENTRE	2018	21.55	0.12	802.76	RW2
Morocco	CENTRE	2019	20.92	0.07	857.65	RW2
Morocco	CENTRE-NORD	1980	142.78	100.77	197.59	RW2
Morocco	CENTRE-NORD	1981	136.78	107.60	172.95	RW2
Morocco	CENTRE-NORD	1982	130.97	105.30	161.70	RW2
Morocco	CENTRE-NORD	1983	125.30	98.42	158.20	RW2
Morocco	CENTRE-NORD	1984	119.85	92.75	153.49	RW2
Morocco	CENTRE-NORD	1985	114.61	90.39	144.67	RW2
Morocco	CENTRE-NORD	1986	109.52	88.25	135.20	RW2
Morocco	CENTRE-NORD	1987	104.53	85.25	127.79	RW2
Morocco	CENTRE-NORD	1988	99.69	80.39	123.49	RW2
Morocco	CENTRE-NORD	1989	95.16	75.45	120.02	RW2
Morocco	CENTRE-NORD	1990	90.63	71.51	114.73	RW2
Morocco	CENTRE-NORD	1991	86.65	68.89	108.22	RW2
Morocco	CENTRE-NORD	1992	82.89	66.31	103.19	RW2
Morocco	CENTRE-NORD	1993	79.34	62.79	100.03	RW2
Morocco	CENTRE-NORD	1994	76.10	59.02	97.84	RW2
Morocco	CENTRE-NORD	1995	73.12	56.38	94.64	RW2
Morocco	CENTRE-NORD	1996	69.98	54.12	90.13	RW2
Morocco	CENTRE-NORD	1997	67.02	51.97	86.32	RW2
Morocco	CENTRE-NORD	1998	63.99	48.77	83.89	RW2
Morocco	CENTRE-NORD	1999	61.00	45.07	81.90	RW2
Morocco	CENTRE-NORD	2000	57.93	41.75	79.68	RW2
Morocco	CENTRE-NORD	2001	55.01	39.74	75.64	RW2
Morocco	CENTRE-NORD	2002	52.23	37.49	71.83	RW2
Morocco	CENTRE-NORD	2003	49.47	33.99	71.21	RW2
Morocco	CENTRE-NORD	2004	46.95	28.95	75.09	RW2
Morocco	CENTRE-NORD	2005	44.63	22.74	85.09	RW2
Morocco	CENTRE-NORD	2006	42.28	17.46	97.74	RW2
Morocco	CENTRE-NORD	2007	39.98	12.94	115.55	RW2
Morocco	CENTRE-NORD	2008	37.78	9.17	139.52	RW2
Morocco	CENTRE-NORD	2009	35.91	6.51	170.80	RW2
Morocco	CENTRE-NORD	2010	33.99	4.49	210.39	RW2
Morocco	CENTRE-NORD	2011	32.12	2.97	263.89	RW2
Morocco	CENTRE-NORD	2012	30.63	1.94	328.51	RW2
Morocco	CENTRE-NORD	2013	29.01	1.36	402.32	RW2
Morocco	CENTRE-NORD	2014	27.34	0.85	479.72	RW2
Morocco	CENTRE-NORD	2015	25.90	0.52	576.33	RW2
Morocco	CENTRE-NORD	2016	24.42	0.31	655.72	RW2
Morocco	CENTRE-NORD	2017	23.27	0.19	736.43	RW2
Morocco	CENTRE-NORD	2018	21.74	0.12	801.11	RW2
Morocco	CENTRE-NORD	2019	20.85	0.07	867.39	RW2
Morocco	CENTRE-SUD	1980	115.43	79.28	164.60	RW2
Morocco	CENTRE-SUD	1981	111.93	85.44	144.99	RW2
Morocco	CENTRE-SUD	1982	108.39	84.80	137.35	RW2

Continued on next page

Country	Region	Year	Median	Lower	Upper	Method
Morocco	CENTRE-SUD	1983	104.88	80.55	135.50	RW2
Morocco	CENTRE-SUD	1984	101.48	77.07	132.43	RW2
Morocco	CENTRE-SUD	1985	98.16	75.64	126.06	RW2
Morocco	CENTRE-SUD	1986	94.94	74.94	119.29	RW2
Morocco	CENTRE-SUD	1987	91.76	73.56	113.95	RW2
Morocco	CENTRE-SUD	1988	88.65	70.27	111.32	RW2
Morocco	CENTRE-SUD	1989	85.66	66.79	109.39	RW2
Morocco	CENTRE-SUD	1990	82.74	64.34	106.05	RW2
Morocco	CENTRE-SUD	1991	80.18	63.02	101.87	RW2
Morocco	CENTRE-SUD	1992	77.83	61.24	98.54	RW2
Morocco	CENTRE-SUD	1993	75.65	58.93	96.67	RW2
Morocco	CENTRE-SUD	1994	73.63	56.06	96.21	RW2
Morocco	CENTRE-SUD	1995	71.85	54.12	94.91	RW2
Morocco	CENTRE-SUD	1996	70.01	52.70	92.48	RW2
Morocco	CENTRE-SUD	1997	68.13	51.12	90.27	RW2
Morocco	CENTRE-SUD	1998	66.17	48.84	89.63	RW2
Morocco	CENTRE-SUD	1999	64.16	45.96	89.35	RW2
Morocco	CENTRE-SUD	2000	62.03	43.18	87.94	RW2
Morocco	CENTRE-SUD	2001	59.99	41.88	85.34	RW2
Morocco	CENTRE-SUD	2002	58.03	40.19	83.19	RW2
Morocco	CENTRE-SUD	2003	55.97	37.27	83.52	RW2
Morocco	CENTRE-SUD	2004	54.09	32.53	88.43	RW2
Morocco	CENTRE-SUD	2005	52.34	26.09	101.87	RW2
Morocco	CENTRE-SUD	2006	50.54	20.46	118.89	RW2
Morocco	CENTRE-SUD	2007	48.72	15.47	141.36	RW2
Morocco	CENTRE-SUD	2008	47.16	11.48	172.03	RW2
Morocco	CENTRE-SUD	2009	45.26	8.36	209.09	RW2
Morocco	CENTRE-SUD	2010	43.98	5.72	261.59	RW2
Morocco	CENTRE-SUD	2011	41.99	3.99	322.17	RW2
Morocco	CENTRE-SUD	2012	40.77	2.67	393.41	RW2
Morocco	CENTRE-SUD	2013	39.07	1.73	478.79	RW2
Morocco	CENTRE-SUD	2014	37.73	1.12	552.71	RW2
Morocco	CENTRE-SUD	2015	36.49	0.74	658.08	RW2
Morocco	CENTRE-SUD	2016	35.12	0.48	736.75	RW2
Morocco	CENTRE-SUD	2017	34.01	0.29	812.84	RW2
Morocco	CENTRE-SUD	2018	32.39	0.17	865.48	RW2
Morocco	CENTRE-SUD	2019	31.12	0.11	905.52	RW2
Morocco	NORD-OUEST	1980	137.03	98.68	187.52	RW2
Morocco	NORD-OUEST	1981	130.16	104.34	161.09	RW2
Morocco	NORD-OUEST	1982	123.61	101.15	149.59	RW2
Morocco	NORD-OUEST	1983	117.18	93.43	146.28	RW2
Morocco	NORD-OUEST	1984	111.16	86.85	141.18	RW2
Morocco	NORD-OUEST	1985	105.32	83.28	131.62	RW2
Morocco	NORD-OUEST	1986	99.62	80.91	122.39	RW2
Morocco	NORD-OUEST	1987	94.25	77.45	114.38	RW2
Morocco	NORD-OUEST	1988	89.07	72.22	109.52	RW2
Morocco	NORD-OUEST	1989	84.11	66.80	105.77	RW2
Morocco	NORD-OUEST	1990	79.46	62.66	100.31	RW2
Morocco	NORD-OUEST	1991	75.34	60.07	93.58	RW2
Morocco	NORD-OUEST	1992	71.44	57.34	88.46	RW2
Morocco	NORD-OUEST	1993	67.98	54.12	85.25	RW2
Morocco	NORD-OUEST	1994	64.89	50.49	82.98	RW2
Morocco	NORD-OUEST	1995	61.98	47.93	80.09	RW2
Morocco	NORD-OUEST	1996	59.19	46.22	75.41	RW2
Morocco	NORD-OUEST	1997	56.49	44.22	72.20	RW2
Morocco	NORD-OUEST	1998	53.91	41.49	69.91	RW2
Morocco	NORD-OUEST	1999	51.27	38.37	68.20	RW2
Morocco	NORD-OUEST	2000	48.69	35.62	66.37	RW2
Morocco	NORD-OUEST	2001	46.28	33.83	63.00	RW2
Morocco	NORD-OUEST	2002	43.91	32.06	59.99	RW2
Morocco	NORD-OUEST	2003	41.65	29.04	59.42	RW2
Morocco	NORD-OUEST	2004	39.53	24.56	62.52	RW2
Morocco	NORD-OUEST	2005	37.49	19.20	71.54	RW2

Continued on next page

Country	Region	Year	Median	Lower	Upper	Method
Morocco	NORD-OUEST	2006	35.47	14.83	82.94	RW2
Morocco	NORD-OUEST	2007	33.73	11.04	98.92	RW2
Morocco	NORD-OUEST	2008	32.09	7.89	120.75	RW2
Morocco	NORD-OUEST	2009	30.26	5.53	146.81	RW2
Morocco	NORD-OUEST	2010	28.79	3.79	184.71	RW2
Morocco	NORD-OUEST	2011	27.28	2.59	232.35	RW2
Morocco	NORD-OUEST	2012	25.84	1.69	290.10	RW2
Morocco	NORD-OUEST	2013	24.51	1.11	358.03	RW2
Morocco	NORD-OUEST	2014	23.03	0.67	432.67	RW2
Morocco	NORD-OUEST	2015	22.05	0.44	526.06	RW2
Morocco	NORD-OUEST	2016	20.76	0.26	611.96	RW2
Morocco	NORD-OUEST	2017	19.87	0.16	701.42	RW2
Morocco	NORD-OUEST	2018	18.60	0.10	772.42	RW2
Morocco	NORD-OUEST	2019	17.85	0.06	836.79	RW2
Morocco	ORIENTAL	1980	112.31	77.02	161.28	RW2
Morocco	ORIENTAL	1981	107.56	81.86	140.52	RW2
Morocco	ORIENTAL	1982	102.86	80.48	130.97	RW2
Morocco	ORIENTAL	1983	98.65	75.65	127.38	RW2
Morocco	ORIENTAL	1984	94.31	71.13	123.49	RW2
Morocco	ORIENTAL	1985	90.34	69.42	116.26	RW2
Morocco	ORIENTAL	1986	86.42	67.92	108.89	RW2
Morocco	ORIENTAL	1987	82.74	65.58	103.71	RW2
Morocco	ORIENTAL	1988	79.23	62.14	100.17	RW2
Morocco	ORIENTAL	1989	76.00	58.45	98.48	RW2
Morocco	ORIENTAL	1990	72.95	55.54	95.20	RW2
Morocco	ORIENTAL	1991	70.13	53.73	91.08	RW2
Morocco	ORIENTAL	1992	67.57	51.74	87.66	RW2
Morocco	ORIENTAL	1993	65.30	49.35	85.85	RW2
Morocco	ORIENTAL	1994	63.13	46.70	84.77	RW2
Morocco	ORIENTAL	1995	61.28	44.92	83.04	RW2
Morocco	ORIENTAL	1996	59.27	43.47	80.44	RW2
Morocco	ORIENTAL	1997	57.25	41.68	78.20	RW2
Morocco	ORIENTAL	1998	55.17	39.47	76.81	RW2
Morocco	ORIENTAL	1999	53.01	36.61	76.14	RW2
Morocco	ORIENTAL	2000	50.95	34.23	75.12	RW2
Morocco	ORIENTAL	2001	48.89	32.40	73.17	RW2
Morocco	ORIENTAL	2002	46.90	30.53	71.49	RW2
Morocco	ORIENTAL	2003	44.92	27.74	71.70	RW2
Morocco	ORIENTAL	2004	43.00	24.07	75.66	RW2
Morocco	ORIENTAL	2005	41.28	19.32	85.02	RW2
Morocco	ORIENTAL	2006	39.35	15.09	98.42	RW2
Morocco	ORIENTAL	2007	37.96	11.44	115.95	RW2
Morocco	ORIENTAL	2008	36.34	8.41	144.51	RW2
Morocco	ORIENTAL	2009	34.61	5.91	177.16	RW2
Morocco	ORIENTAL	2010	33.01	4.08	219.46	RW2
Morocco	ORIENTAL	2011	31.90	2.80	270.71	RW2
Morocco	ORIENTAL	2012	30.53	1.85	338.53	RW2
Morocco	ORIENTAL	2013	28.70	1.20	404.07	RW2
Morocco	ORIENTAL	2014	27.81	0.80	503.73	RW2
Morocco	ORIENTAL	2015	26.51	0.51	577.22	RW2
Morocco	ORIENTAL	2016	25.48	0.31	684.27	RW2
Morocco	ORIENTAL	2017	24.68	0.20	758.36	RW2
Morocco	ORIENTAL	2018	23.65	0.12	822.16	RW2
Morocco	ORIENTAL	2019	22.23	0.07	883.05	RW2
Morocco	SUD	1980	193.37	138.69	263.02	RW2
Morocco	SUD	1981	181.36	143.67	226.55	RW2
Morocco	SUD	1982	169.86	136.90	207.99	RW2
Morocco	SUD	1983	159.06	125.91	198.87	RW2
Morocco	SUD	1984	148.79	115.64	189.37	RW2
Morocco	SUD	1985	139.15	109.98	174.56	RW2
Morocco	SUD	1986	130.01	104.92	159.40	RW2
Morocco	SUD	1987	121.41	99.13	147.91	RW2
Morocco	SUD	1988	113.35	91.60	139.51	RW2

Continued on next page

Country	Region	Year	Median	Lower	Upper	Method
Morocco	SUD	1989	105.91	83.83	132.82	RW2
Morocco	SUD	1990	99.01	77.93	124.87	RW2
Morocco	SUD	1991	92.79	73.85	116.42	RW2
Morocco	SUD	1992	87.08	69.48	108.35	RW2
Morocco	SUD	1993	81.85	64.46	103.22	RW2
Morocco	SUD	1994	77.07	59.27	99.48	RW2
Morocco	SUD	1995	72.78	55.50	95.03	RW2
Morocco	SUD	1996	68.55	52.34	89.25	RW2
Morocco	SUD	1997	64.59	49.15	84.24	RW2
Morocco	SUD	1998	60.62	45.29	80.72	RW2
Morocco	SUD	1999	56.86	41.06	78.44	RW2
Morocco	SUD	2000	53.17	37.40	74.90	RW2
Morocco	SUD	2001	49.69	34.87	70.05	RW2
Morocco	SUD	2002	46.44	32.32	66.21	RW2
Morocco	SUD	2003	43.32	28.92	64.32	RW2
Morocco	SUD	2004	40.51	24.29	66.55	RW2
Morocco	SUD	2005	37.80	18.76	73.84	RW2
Morocco	SUD	2006	35.38	14.26	84.20	RW2
Morocco	SUD	2007	32.81	10.28	98.72	RW2
Morocco	SUD	2008	30.47	7.34	116.25	RW2
Morocco	SUD	2009	28.67	5.14	142.85	RW2
Morocco	SUD	2010	26.61	3.38	172.43	RW2
Morocco	SUD	2011	24.78	2.35	215.49	RW2
Morocco	SUD	2012	23.08	1.49	272.83	RW2
Morocco	SUD	2013	21.55	0.93	327.80	RW2
Morocco	SUD	2014	20.29	0.58	402.75	RW2
Morocco	SUD	2015	18.82	0.39	495.86	RW2
Morocco	SUD	2016	17.46	0.22	572.82	RW2
Morocco	SUD	2017	16.44	0.13	657.18	RW2
Morocco	SUD	2018	14.91	0.08	741.97	RW2
Morocco	SUD	2019	13.92	0.05	805.30	RW2
Morocco	TENSIFT	1980	159.98	113.86	219.23	RW2
Morocco	TENSIFT	1981	151.24	119.31	189.34	RW2
Morocco	TENSIFT	1982	142.92	115.65	175.71	RW2
Morocco	TENSIFT	1983	135.08	106.81	169.88	RW2
Morocco	TENSIFT	1984	127.68	98.55	163.15	RW2
Morocco	TENSIFT	1985	120.63	94.88	152.44	RW2
Morocco	TENSIFT	1986	114.06	91.44	141.19	RW2
Morocco	TENSIFT	1987	107.95	87.45	132.47	RW2
Morocco	TENSIFT	1988	102.26	81.68	126.98	RW2
Morocco	TENSIFT	1989	96.86	76.29	122.86	RW2
Morocco	TENSIFT	1990	92.03	72.09	116.46	RW2
Morocco	TENSIFT	1991	87.58	69.26	110.18	RW2
Morocco	TENSIFT	1992	83.57	66.54	104.47	RW2
Morocco	TENSIFT	1993	79.79	62.60	101.35	RW2
Morocco	TENSIFT	1994	76.29	58.66	98.70	RW2
Morocco	TENSIFT	1995	73.08	55.59	95.66	RW2
Morocco	TENSIFT	1996	69.94	53.67	91.38	RW2
Morocco	TENSIFT	1997	66.87	50.98	87.16	RW2
Morocco	TENSIFT	1998	63.67	47.89	84.44	RW2
Morocco	TENSIFT	1999	60.62	44.34	82.04	RW2
Morocco	TENSIFT	2000	57.55	41.26	79.65	RW2
Morocco	TENSIFT	2001	54.58	39.34	75.35	RW2
Morocco	TENSIFT	2002	51.81	37.33	71.64	RW2
Morocco	TENSIFT	2003	49.14	33.91	70.30	RW2
Morocco	TENSIFT	2004	46.52	28.73	74.01	RW2
Morocco	TENSIFT	2005	44.18	22.67	84.26	RW2
Morocco	TENSIFT	2006	41.78	17.25	96.67	RW2
Morocco	TENSIFT	2007	39.61	12.86	115.68	RW2
Morocco	TENSIFT	2008	37.31	9.24	139.96	RW2
Morocco	TENSIFT	2009	35.45	6.29	172.42	RW2
Morocco	TENSIFT	2010	33.47	4.40	212.43	RW2
Morocco	TENSIFT	2011	31.80	2.94	259.17	RW2

Continued on next page

Country	Region	Year	Median	Lower	Upper	Method
Morocco	TENSIFT	2012	30.09	2.02	329.46	RW2
Morocco	TENSIFT	2013	28.40	1.26	390.32	RW2
Morocco	TENSIFT	2014	26.81	0.83	480.87	RW2
Morocco	TENSIFT	2015	25.60	0.48	580.53	RW2
Morocco	TENSIFT	2016	23.92	0.32	654.29	RW2
Morocco	TENSIFT	2017	22.57	0.18	736.66	RW2
Morocco	TENSIFT	2018	21.49	0.11	798.58	RW2
Morocco	TENSIFT	2019	20.38	0.06	849.98	RW2
Mozambique	ALL	1980	227.57	219.89	235.60	IHME
Mozambique	ALL	1980	264.22	203.01	335.64	RW2
Mozambique	ALL	1980	261.80	235.90	289.80	UN
Mozambique	ALL	1981	252.26	223.19	292.18	IHME
Mozambique	ALL	1981	262.08	217.50	311.59	RW2
Mozambique	ALL	1981	261.00	236.40	287.70	UN
Mozambique	ALL	1982	251.16	222.83	290.16	IHME
Mozambique	ALL	1982	259.99	220.25	304.10	RW2
Mozambique	ALL	1982	259.80	236.50	285.80	UN
Mozambique	ALL	1983	250.51	223.07	286.46	IHME
Mozambique	ALL	1983	257.60	214.81	305.64	RW2
Mozambique	ALL	1983	258.30	235.90	283.30	UN
Mozambique	ALL	1984	254.05	225.32	291.23	IHME
Mozambique	ALL	1984	255.66	209.00	308.06	RW2
Mozambique	ALL	1984	256.20	234.60	280.50	UN
Mozambique	ALL	1985	253.46	225.24	290.83	IHME
Mozambique	ALL	1985	253.09	209.98	301.75	RW2
Mozambique	ALL	1985	253.80	232.50	277.10	UN
Mozambique	ALL	1986	228.91	219.91	239.58	IHME
Mozambique	ALL	1986	250.87	210.66	295.75	RW2
Mozambique	ALL	1986	251.20	230.50	273.90	UN
Mozambique	ALL	1987	227.57	218.92	237.53	IHME
Mozambique	ALL	1987	248.69	210.57	291.62	RW2
Mozambique	ALL	1987	248.50	228.50	270.20	UN
Mozambique	ALL	1988	220.79	214.88	227.24	IHME
Mozambique	ALL	1988	246.21	206.81	289.99	RW2
Mozambique	ALL	1988	245.70	226.20	266.70	UN
Mozambique	ALL	1989	217.72	211.96	224.06	IHME
Mozambique	ALL	1989	243.51	202.71	289.16	RW2
Mozambique	ALL	1989	242.80	224.10	262.90	UN
Mozambique	ALL	1990	214.22	208.68	220.13	IHME
Mozambique	ALL	1990	240.70	200.93	286.30	RW2
Mozambique	ALL	1990	239.70	221.80	259.10	UN
Mozambique	ALL	1991	210.03	204.58	215.93	IHME
Mozambique	ALL	1991	237.03	199.56	278.81	RW2
Mozambique	ALL	1991	236.40	219.00	255.40	UN
Mozambique	ALL	1992	205.87	200.37	211.37	IHME
Mozambique	ALL	1992	232.63	196.41	272.81	RW2
Mozambique	ALL	1992	232.40	215.60	251.00	UN
Mozambique	ALL	1993	201.13	195.86	206.50	IHME
Mozambique	ALL	1993	227.46	191.10	268.29	RW2
Mozambique	ALL	1993	227.70	211.30	245.60	UN
Mozambique	ALL	1994	196.05	190.79	201.32	IHME
Mozambique	ALL	1994	221.34	183.99	264.43	RW2
Mozambique	ALL	1994	222.00	206.00	239.30	UN
Mozambique	ALL	1995	190.73	185.64	195.76	IHME
Mozambique	ALL	1995	214.58	178.67	255.31	RW2
Mozambique	ALL	1995	215.20	199.80	232.20	UN
Mozambique	ALL	1996	184.30	179.41	189.45	IHME
Mozambique	ALL	1996	206.95	173.91	244.93	RW2
Mozambique	ALL	1996	207.40	192.60	223.70	UN
Mozambique	ALL	1997	177.59	172.75	182.57	IHME
Mozambique	ALL	1997	198.75	167.81	233.96	RW2
Mozambique	ALL	1997	198.80	184.50	214.20	UN
Mozambique	ALL	1998	170.60	165.88	175.59	IHME

Continued on next page

Country	Region	Year	Median	Lower	Upper	Method
Mozambique	ALL	1998	190.21	159.45	226.37	RW2
Mozambique	ALL	1998	189.50	175.90	204.30	UN
Mozambique	ALL	1999	163.72	158.98	168.63	IHME
Mozambique	ALL	1999	181.34	149.95	217.55	RW2
Mozambique	ALL	1999	180.10	167.20	194.20	UN
Mozambique	ALL	2000	157.46	152.70	162.31	IHME
Mozambique	ALL	2000	172.21	141.77	207.00	RW2
Mozambique	ALL	2000	171.10	158.60	184.60	UN
Mozambique	ALL	2001	150.85	146.21	155.53	IHME
Mozambique	ALL	2001	163.49	135.42	195.68	RW2
Mozambique	ALL	2001	162.50	150.40	175.60	UN
Mozambique	ALL	2002	144.27	139.73	148.79	IHME
Mozambique	ALL	2002	155.06	129.13	185.10	RW2
Mozambique	ALL	2002	154.50	142.90	167.10	UN
Mozambique	ALL	2003	138.07	133.48	142.69	IHME
Mozambique	ALL	2003	147.14	121.94	176.55	RW2
Mozambique	ALL	2003	147.10	135.80	159.30	UN
Mozambique	ALL	2004	132.19	127.58	136.82	IHME
Mozambique	ALL	2004	139.46	113.67	169.66	RW2
Mozambique	ALL	2004	140.20	129.20	152.30	UN
Mozambique	ALL	2005	126.33	121.44	131.12	IHME
Mozambique	ALL	2005	132.52	107.47	162.53	RW2
Mozambique	ALL	2005	133.80	123.10	145.60	UN
Mozambique	ALL	2006	120.54	115.40	125.56	IHME
Mozambique	ALL	2006	125.79	102.74	153.09	RW2
Mozambique	ALL	2006	127.80	117.20	139.10	UN
Mozambique	ALL	2007	114.94	109.50	120.22	IHME
Mozambique	ALL	2007	119.51	98.07	144.86	RW2
Mozambique	ALL	2007	120.10	109.80	131.20	UN
Mozambique	ALL	2008	109.78	104.03	115.48	IHME
Mozambique	ALL	2008	113.71	92.46	139.16	RW2
Mozambique	ALL	2008	113.60	103.30	124.70	UN
Mozambique	ALL	2009	104.80	98.91	111.03	IHME
Mozambique	ALL	2009	108.00	86.26	134.76	RW2
Mozambique	ALL	2009	107.60	97.00	119.30	UN
Mozambique	ALL	2010	99.66	93.41	106.32	IHME
Mozambique	ALL	2010	102.69	80.62	130.68	RW2
Mozambique	ALL	2010	102.80	91.40	115.80	UN
Mozambique	ALL	2011	95.10	88.58	102.07	IHME
Mozambique	ALL	2011	97.70	76.67	123.79	RW2
Mozambique	ALL	2011	97.50	85.40	112.10	UN
Mozambique	ALL	2012	90.59	83.75	98.26	IHME
Mozambique	ALL	2012	92.87	72.51	118.17	RW2
Mozambique	ALL	2012	90.90	77.90	106.70	UN
Mozambique	ALL	2013	86.26	79.20	94.50	IHME
Mozambique	ALL	2013	88.36	66.17	116.85	RW2
Mozambique	ALL	2013	85.60	71.40	103.10	UN
Mozambique	ALL	2014	81.97	74.54	90.72	IHME
Mozambique	ALL	2014	83.94	56.94	121.97	RW2
Mozambique	ALL	2014	81.20	65.80	101.20	UN
Mozambique	ALL	2015	77.93	70.40	87.26	IHME
Mozambique	ALL	2015	79.61	45.70	135.32	RW2
Mozambique	ALL	2015	78.50	61.50	100.80	UN
Mozambique	ALL	2016	75.80	36.44	152.03	RW2
Mozambique	ALL	2017	71.80	27.99	173.68	RW2
Mozambique	ALL	2018	68.14	21.26	202.47	RW2
Mozambique	ALL	2019	64.56	15.22	233.92	RW2
Mozambique	ALL	15-19	71.80	28.39	171.09	RW2
Mozambique	CABO DELGADO	1980	321.00	237.97	414.35	RW2
Mozambique	CABO DELGADO	1981	319.21	251.80	392.81	RW2
Mozambique	CABO DELGADO	1982	317.44	257.72	383.36	RW2
Mozambique	CABO DELGADO	1983	315.87	257.71	380.34	RW2
Mozambique	CABO DELGADO	1984	314.32	255.93	377.74	RW2

Continued on next page

Country	Region	Year	Median	Lower	Upper	Method
Mozambique	CABO DELGADO	1985	312.52	259.17	371.50	RW2
Mozambique	CABO DELGADO	1986	310.79	261.62	364.29	RW2
Mozambique	CABO DELGADO	1987	309.00	262.98	358.94	RW2
Mozambique	CABO DELGADO	1988	306.96	260.81	356.53	RW2
Mozambique	CABO DELGADO	1989	304.26	258.11	355.40	RW2
Mozambique	CABO DELGADO	1990	301.60	256.68	350.35	RW2
Mozambique	CABO DELGADO	1991	297.92	255.64	344.01	RW2
Mozambique	CABO DELGADO	1992	293.45	253.33	336.99	RW2
Mozambique	CABO DELGADO	1993	287.72	246.87	332.78	RW2
Mozambique	CABO DELGADO	1994	280.89	238.84	327.53	RW2
Mozambique	CABO DELGADO	1995	272.88	231.63	318.69	RW2
Mozambique	CABO DELGADO	1996	263.68	226.07	306.77	RW2
Mozambique	CABO DELGADO	1997	253.30	216.99	293.85	RW2
Mozambique	CABO DELGADO	1998	241.55	205.62	282.68	RW2
Mozambique	CABO DELGADO	1999	229.20	192.22	270.91	RW2
Mozambique	CABO DELGADO	2000	216.09	180.12	257.20	RW2
Mozambique	CABO DELGADO	2001	202.85	169.93	240.83	RW2
Mozambique	CABO DELGADO	2002	189.86	159.01	225.72	RW2
Mozambique	CABO DELGADO	2003	177.12	146.54	212.07	RW2
Mozambique	CABO DELGADO	2004	164.70	133.69	200.88	RW2
Mozambique	CABO DELGADO	2005	153.11	122.75	189.39	RW2
Mozambique	CABO DELGADO	2006	141.95	112.85	176.50	RW2
Mozambique	CABO DELGADO	2007	131.62	103.38	165.79	RW2
Mozambique	CABO DELGADO	2008	121.84	93.44	156.87	RW2
Mozambique	CABO DELGADO	2009	112.98	83.43	149.52	RW2
Mozambique	CABO DELGADO	2010	104.66	74.88	142.66	RW2
Mozambique	CABO DELGADO	2011	97.03	67.46	134.42	RW2
Mozambique	CABO DELGADO	2012	89.87	60.64	128.14	RW2
Mozambique	CABO DELGADO	2013	83.14	52.83	123.67	RW2
Mozambique	CABO DELGADO	2014	76.84	44.89	124.35	RW2
Mozambique	CABO DELGADO	2015	71.08	35.83	131.30	RW2
Mozambique	CABO DELGADO	2016	65.41	28.55	139.24	RW2
Mozambique	CABO DELGADO	2017	60.29	21.60	152.07	RW2
Mozambique	CABO DELGADO	2018	55.68	16.25	167.62	RW2
Mozambique	CABO DELGADO	2019	51.36	11.84	186.93	RW2
Mozambique	GAZA	1980	222.33	164.42	293.59	RW2
Mozambique	GAZA	1981	221.20	175.62	275.55	RW2
Mozambique	GAZA	1982	220.03	178.40	267.62	RW2
Mozambique	GAZA	1983	218.67	177.00	266.68	RW2
Mozambique	GAZA	1984	217.35	175.51	266.29	RW2
Mozambique	GAZA	1985	215.78	176.44	261.00	RW2
Mozambique	GAZA	1986	214.14	178.00	255.83	RW2
Mozambique	GAZA	1987	212.35	178.12	251.65	RW2
Mozambique	GAZA	1988	210.22	175.82	249.09	RW2
Mozambique	GAZA	1989	207.77	172.96	247.98	RW2
Mozambique	GAZA	1990	205.13	171.07	243.59	RW2
Mozambique	GAZA	1991	202.07	170.34	238.12	RW2
Mozambique	GAZA	1992	198.25	167.96	231.80	RW2
Mozambique	GAZA	1993	194.10	163.68	228.42	RW2
Mozambique	GAZA	1994	189.56	158.27	224.93	RW2
Mozambique	GAZA	1995	184.42	153.64	219.18	RW2
Mozambique	GAZA	1996	179.09	150.60	210.85	RW2
Mozambique	GAZA	1997	173.12	146.02	203.62	RW2
Mozambique	GAZA	1998	166.97	139.89	197.72	RW2
Mozambique	GAZA	1999	160.93	133.28	193.19	RW2
Mozambique	GAZA	2000	154.58	127.31	186.03	RW2
Mozambique	GAZA	2001	148.47	123.23	177.94	RW2
Mozambique	GAZA	2002	142.65	117.97	171.23	RW2
Mozambique	GAZA	2003	137.02	112.24	166.51	RW2
Mozambique	GAZA	2004	131.78	106.13	162.35	RW2
Mozambique	GAZA	2005	126.70	101.06	157.88	RW2
Mozambique	GAZA	2006	122.12	97.25	152.61	RW2
Mozambique	GAZA	2007	117.54	93.06	147.16	RW2

Continued on next page

Country	Region	Year	Median	Lower	Upper	Method
Mozambique	GAZA	2008	113.23	88.39	144.30	RW2
Mozambique	GAZA	2009	109.35	83.56	142.02	RW2
Mozambique	GAZA	2010	105.54	79.09	139.97	RW2
Mozambique	GAZA	2011	101.91	75.69	136.09	RW2
Mozambique	GAZA	2012	98.28	72.02	133.39	RW2
Mozambique	GAZA	2013	94.88	66.91	133.55	RW2
Mozambique	GAZA	2014	91.58	59.46	138.59	RW2
Mozambique	GAZA	2015	88.33	50.09	151.92	RW2
Mozambique	GAZA	2016	85.06	41.50	168.38	RW2
Mozambique	GAZA	2017	82.21	33.10	190.25	RW2
Mozambique	GAZA	2018	79.07	25.92	216.76	RW2
Mozambique	GAZA	2019	76.15	20.12	251.27	RW2
Mozambique	INHAMBANE	1980	244.28	181.04	320.79	RW2
Mozambique	INHAMBANE	1981	241.17	191.14	298.83	RW2
Mozambique	INHAMBANE	1982	237.63	192.38	289.12	RW2
Mozambique	INHAMBANE	1983	234.20	189.15	285.85	RW2
Mozambique	INHAMBANE	1984	230.73	185.65	282.61	RW2
Mozambique	INHAMBANE	1985	227.29	185.63	275.34	RW2
Mozambique	INHAMBANE	1986	223.61	185.16	266.65	RW2
Mozambique	INHAMBANE	1987	219.65	183.91	260.25	RW2
Mozambique	INHAMBANE	1988	215.39	180.16	255.00	RW2
Mozambique	INHAMBANE	1989	210.80	176.03	250.57	RW2
Mozambique	INHAMBANE	1990	206.05	172.50	244.28	RW2
Mozambique	INHAMBANE	1991	200.59	169.88	235.37	RW2
Mozambique	INHAMBANE	1992	194.50	165.60	227.42	RW2
Mozambique	INHAMBANE	1993	187.82	159.44	220.35	RW2
Mozambique	INHAMBANE	1994	180.58	152.12	213.61	RW2
Mozambique	INHAMBANE	1995	173.03	145.15	204.47	RW2
Mozambique	INHAMBANE	1996	164.80	140.05	193.48	RW2
Mozambique	INHAMBANE	1997	156.40	133.03	182.73	RW2
Mozambique	INHAMBANE	1998	147.62	124.38	174.07	RW2
Mozambique	INHAMBANE	1999	138.96	115.47	166.66	RW2
Mozambique	INHAMBANE	2000	130.28	106.67	157.26	RW2
Mozambique	INHAMBANE	2001	122.08	100.21	147.70	RW2
Mozambique	INHAMBANE	2002	114.21	93.43	138.92	RW2
Mozambique	INHAMBANE	2003	106.86	86.08	131.92	RW2
Mozambique	INHAMBANE	2004	100.02	78.62	126.04	RW2
Mozambique	INHAMBANE	2005	93.64	72.47	119.96	RW2
Mozambique	INHAMBANE	2006	87.79	67.22	113.39	RW2
Mozambique	INHAMBANE	2007	82.31	62.10	107.87	RW2
Mozambique	INHAMBANE	2008	77.23	56.97	103.17	RW2
Mozambique	INHAMBANE	2009	72.44	51.76	99.73	RW2
Mozambique	INHAMBANE	2010	68.10	47.07	96.27	RW2
Mozambique	INHAMBANE	2011	63.99	43.36	92.04	RW2
Mozambique	INHAMBANE	2012	60.07	39.58	88.62	RW2
Mozambique	INHAMBANE	2013	56.36	35.59	86.63	RW2
Mozambique	INHAMBANE	2014	52.89	30.93	87.61	RW2
Mozambique	INHAMBANE	2015	49.64	25.29	93.26	RW2
Mozambique	INHAMBANE	2016	46.65	20.55	100.87	RW2
Mozambique	INHAMBANE	2017	43.57	16.06	112.76	RW2
Mozambique	INHAMBANE	2018	40.90	12.34	126.52	RW2
Mozambique	INHAMBANE	2019	38.38	9.28	146.93	RW2
Mozambique	MANICA	1980	287.13	214.16	372.57	RW2
Mozambique	MANICA	1981	283.35	224.66	351.26	RW2
Mozambique	MANICA	1982	280.12	226.34	340.58	RW2
Mozambique	MANICA	1983	276.53	224.05	336.85	RW2
Mozambique	MANICA	1984	272.85	220.26	332.39	RW2
Mozambique	MANICA	1985	269.22	220.44	324.01	RW2
Mozambique	MANICA	1986	265.30	221.01	315.17	RW2
Mozambique	MANICA	1987	261.32	219.64	307.24	RW2
Mozambique	MANICA	1988	257.10	216.59	302.30	RW2
Mozambique	MANICA	1989	252.38	211.42	298.17	RW2
Mozambique	MANICA	1990	247.20	208.44	290.61	RW2

Continued on next page

Country	Region	Year	Median	Lower	Upper	Method
Mozambique	MANICA	1991	241.64	206.42	281.15	RW2
Mozambique	MANICA	1992	235.38	201.64	272.38	RW2
Mozambique	MANICA	1993	228.62	195.47	265.64	RW2
Mozambique	MANICA	1994	221.03	186.57	259.30	RW2
Mozambique	MANICA	1995	213.28	179.86	250.52	RW2
Mozambique	MANICA	1996	204.73	174.27	238.77	RW2
Mozambique	MANICA	1997	195.91	166.96	227.96	RW2
Mozambique	MANICA	1998	186.77	158.10	219.42	RW2
Mozambique	MANICA	1999	177.55	148.43	210.99	RW2
Mozambique	MANICA	2000	168.38	139.79	201.16	RW2
Mozambique	MANICA	2001	159.59	133.16	189.94	RW2
Mozambique	MANICA	2002	151.12	126.45	180.01	RW2
Mozambique	MANICA	2003	143.24	118.07	172.70	RW2
Mozambique	MANICA	2004	135.90	110.55	166.27	RW2
Mozambique	MANICA	2005	128.95	103.51	159.69	RW2
Mozambique	MANICA	2006	122.62	98.31	152.30	RW2
Mozambique	MANICA	2007	116.66	92.84	145.90	RW2
Mozambique	MANICA	2008	111.02	86.76	141.11	RW2
Mozambique	MANICA	2009	105.84	80.76	137.71	RW2
Mozambique	MANICA	2010	100.78	75.27	134.54	RW2
Mozambique	MANICA	2011	96.16	70.98	129.40	RW2
Mozambique	MANICA	2012	91.66	66.54	125.78	RW2
Mozambique	MANICA	2013	87.48	60.90	124.82	RW2
Mozambique	MANICA	2014	83.37	53.60	128.66	RW2
Mozambique	MANICA	2015	79.38	44.48	138.89	RW2
Mozambique	MANICA	2016	75.61	36.24	151.72	RW2
Mozambique	MANICA	2017	71.94	28.88	169.70	RW2
Mozambique	MANICA	2018	68.45	22.17	193.70	RW2
Mozambique	MANICA	2019	65.30	16.81	223.13	RW2
Mozambique	MAPUTO CIDADE	1980	96.47	65.00	141.88	RW2
Mozambique	MAPUTO CIDADE	1981	97.62	71.14	133.11	RW2
Mozambique	MAPUTO CIDADE	1982	98.61	74.56	130.02	RW2
Mozambique	MAPUTO CIDADE	1983	99.63	75.80	129.94	RW2
Mozambique	MAPUTO CIDADE	1984	100.61	76.60	131.01	RW2
Mozambique	MAPUTO CIDADE	1985	101.53	78.82	129.86	RW2
Mozambique	MAPUTO CIDADE	1986	102.30	81.37	128.55	RW2
Mozambique	MAPUTO CIDADE	1987	102.96	82.66	127.89	RW2
Mozambique	MAPUTO CIDADE	1988	103.59	82.82	128.81	RW2
Mozambique	MAPUTO CIDADE	1989	104.05	83.03	129.82	RW2
Mozambique	MAPUTO CIDADE	1990	104.35	83.64	129.47	RW2
Mozambique	MAPUTO CIDADE	1991	104.36	84.39	128.06	RW2
Mozambique	MAPUTO CIDADE	1992	104.15	84.67	127.18	RW2
Mozambique	MAPUTO CIDADE	1993	103.89	83.95	127.19	RW2
Mozambique	MAPUTO CIDADE	1994	103.43	82.98	127.76	RW2
Mozambique	MAPUTO CIDADE	1995	102.64	82.22	126.65	RW2
Mozambique	MAPUTO CIDADE	1996	101.86	82.65	124.26	RW2
Mozambique	MAPUTO CIDADE	1997	100.79	82.17	122.79	RW2
Mozambique	MAPUTO CIDADE	1998	99.77	81.05	122.03	RW2
Mozambique	MAPUTO CIDADE	1999	98.67	79.49	121.90	RW2
Mozambique	MAPUTO CIDADE	2000	97.30	78.20	120.41	RW2
Mozambique	MAPUTO CIDADE	2001	96.09	77.88	118.28	RW2
Mozambique	MAPUTO CIDADE	2002	94.80	76.86	116.59	RW2
Mozambique	MAPUTO CIDADE	2003	93.45	75.02	115.97	RW2
Mozambique	MAPUTO CIDADE	2004	92.20	73.07	115.95	RW2
Mozambique	MAPUTO CIDADE	2005	90.85	70.98	116.14	RW2
Mozambique	MAPUTO CIDADE	2006	89.56	69.85	114.41	RW2
Mozambique	MAPUTO CIDADE	2007	88.22	68.26	113.66	RW2
Mozambique	MAPUTO CIDADE	2008	86.94	65.70	114.38	RW2
Mozambique	MAPUTO CIDADE	2009	85.69	63.04	115.83	RW2
Mozambique	MAPUTO CIDADE	2010	84.41	60.10	118.11	RW2
Mozambique	MAPUTO CIDADE	2011	83.30	57.59	119.15	RW2
Mozambique	MAPUTO CIDADE	2012	82.18	54.96	120.82	RW2
Mozambique	MAPUTO CIDADE	2013	81.01	51.17	125.09	RW2

Continued on next page

Country	Region	Year	Median	Lower	Upper	Method
Mozambique	MAPUTO CIDADE	2014	79.84	46.00	134.08	RW2
Mozambique	MAPUTO CIDADE	2015	78.69	39.57	149.94	RW2
Mozambique	MAPUTO CIDADE	2016	77.83	32.91	169.51	RW2
Mozambique	MAPUTO CIDADE	2017	76.32	27.20	195.61	RW2
Mozambique	MAPUTO CIDADE	2018	75.19	21.84	227.27	RW2
Mozambique	MAPUTO CIDADE	2019	74.47	16.98	267.22	RW2
Mozambique	MAPUTO PROVINCIA	1980	129.52	87.01	189.91	RW2
Mozambique	MAPUTO PROVINCIA	1981	129.96	93.42	178.91	RW2
Mozambique	MAPUTO PROVINCIA	1982	130.42	97.24	174.09	RW2
Mozambique	MAPUTO PROVINCIA	1983	130.83	98.32	172.42	RW2
Mozambique	MAPUTO PROVINCIA	1984	131.04	98.96	171.99	RW2
Mozambique	MAPUTO PROVINCIA	1985	131.38	101.29	168.95	RW2
Mozambique	MAPUTO PROVINCIA	1986	131.60	103.67	165.53	RW2
Mozambique	MAPUTO PROVINCIA	1987	131.68	105.05	163.34	RW2
Mozambique	MAPUTO PROVINCIA	1988	131.54	105.44	162.60	RW2
Mozambique	MAPUTO PROVINCIA	1989	131.18	104.98	162.78	RW2
Mozambique	MAPUTO PROVINCIA	1990	130.68	105.33	161.22	RW2
Mozambique	MAPUTO PROVINCIA	1991	129.92	105.88	158.31	RW2
Mozambique	MAPUTO PROVINCIA	1992	129.05	105.52	156.13	RW2
Mozambique	MAPUTO PROVINCIA	1993	127.69	103.98	155.45	RW2
Mozambique	MAPUTO PROVINCIA	1994	126.31	102.16	154.70	RW2
Mozambique	MAPUTO PROVINCIA	1995	124.61	100.78	152.76	RW2
Mozambique	MAPUTO PROVINCIA	1996	122.83	99.98	149.17	RW2
Mozambique	MAPUTO PROVINCIA	1997	120.84	98.92	145.83	RW2
Mozambique	MAPUTO PROVINCIA	1998	118.67	96.72	144.27	RW2
Mozambique	MAPUTO PROVINCIA	1999	116.47	94.42	142.76	RW2
Mozambique	MAPUTO PROVINCIA	2000	114.21	92.07	140.20	RW2
Mozambique	MAPUTO PROVINCIA	2001	111.97	91.34	136.68	RW2
Mozambique	MAPUTO PROVINCIA	2002	109.82	89.81	133.76	RW2
Mozambique	MAPUTO PROVINCIA	2003	107.61	87.22	132.30	RW2
Mozambique	MAPUTO PROVINCIA	2004	105.53	84.08	131.93	RW2
Mozambique	MAPUTO PROVINCIA	2005	103.52	81.67	130.63	RW2
Mozambique	MAPUTO PROVINCIA	2006	101.55	80.13	128.53	RW2
Mozambique	MAPUTO PROVINCIA	2007	99.58	77.92	126.47	RW2
Mozambique	MAPUTO PROVINCIA	2008	97.75	75.16	126.67	RW2
Mozambique	MAPUTO PROVINCIA	2009	95.94	71.95	127.32	RW2
Mozambique	MAPUTO PROVINCIA	2010	94.28	68.66	129.07	RW2
Mozambique	MAPUTO PROVINCIA	2011	92.49	66.10	128.66	RW2
Mozambique	MAPUTO PROVINCIA	2012	90.91	63.19	129.58	RW2
Mozambique	MAPUTO PROVINCIA	2013	89.33	59.01	133.55	RW2
Mozambique	MAPUTO PROVINCIA	2014	87.82	53.28	141.96	RW2
Mozambique	MAPUTO PROVINCIA	2015	86.04	45.41	156.79	RW2
Mozambique	MAPUTO PROVINCIA	2016	84.50	38.37	176.47	RW2
Mozambique	MAPUTO PROVINCIA	2017	83.12	31.60	202.25	RW2
Mozambique	MAPUTO PROVINCIA	2018	81.66	25.53	235.59	RW2
Mozambique	MAPUTO PROVINCIA	2019	80.12	19.58	277.16	RW2
Mozambique	NAMPULA	1980	318.48	245.82	400.76	RW2
Mozambique	NAMPULA	1981	314.77	258.67	376.65	RW2
Mozambique	NAMPULA	1982	310.92	259.58	366.16	RW2
Mozambique	NAMPULA	1983	307.30	255.76	363.84	RW2
Mozambique	NAMPULA	1984	303.60	250.75	361.92	RW2
Mozambique	NAMPULA	1985	299.96	250.32	354.57	RW2
Mozambique	NAMPULA	1986	296.20	249.88	346.00	RW2
Mozambique	NAMPULA	1987	292.33	248.51	340.00	RW2
Mozambique	NAMPULA	1988	288.20	244.62	335.71	RW2
Mozambique	NAMPULA	1989	283.84	239.27	332.47	RW2
Mozambique	NAMPULA	1990	279.16	236.07	326.85	RW2
Mozambique	NAMPULA	1991	273.73	233.75	318.78	RW2
Mozambique	NAMPULA	1992	267.36	229.33	309.12	RW2
Mozambique	NAMPULA	1993	260.00	222.13	301.99	RW2
Mozambique	NAMPULA	1994	251.66	212.87	295.16	RW2
Mozambique	NAMPULA	1995	242.46	205.42	284.11	RW2
Mozambique	NAMPULA	1996	232.02	198.13	269.81	RW2

Continued on next page

Country	Region	Year	Median	Lower	Upper	Method
Mozambique	NAMPULA	1997	220.80	189.31	255.81	RW2
Mozambique	NAMPULA	1998	208.57	177.58	243.69	RW2
Mozambique	NAMPULA	1999	195.99	164.04	233.04	RW2
Mozambique	NAMPULA	2000	183.03	151.95	218.53	RW2
Mozambique	NAMPULA	2001	170.30	141.87	202.62	RW2
Mozambique	NAMPULA	2002	158.02	131.31	188.88	RW2
Mozambique	NAMPULA	2003	146.21	120.04	176.75	RW2
Mozambique	NAMPULA	2004	135.24	108.80	166.56	RW2
Mozambique	NAMPULA	2005	124.88	98.77	155.96	RW2
Mozambique	NAMPULA	2006	115.37	90.66	145.05	RW2
Mozambique	NAMPULA	2007	106.34	82.49	135.30	RW2
Mozambique	NAMPULA	2008	98.09	74.68	126.92	RW2
Mozambique	NAMPULA	2009	90.75	67.24	120.33	RW2
Mozambique	NAMPULA	2010	83.79	60.21	113.74	RW2
Mozambique	NAMPULA	2011	77.40	54.95	106.67	RW2
Mozambique	NAMPULA	2012	71.49	49.40	100.70	RW2
Mozambique	NAMPULA	2013	66.01	43.42	96.64	RW2
Mozambique	NAMPULA	2014	61.01	36.85	96.54	RW2
Mozambique	NAMPULA	2015	56.17	29.90	101.80	RW2
Mozambique	NAMPULA	2016	51.71	23.38	108.21	RW2
Mozambique	NAMPULA	2017	47.74	17.96	118.15	RW2
Mozambique	NAMPULA	2018	43.67	13.47	132.12	RW2
Mozambique	NAMPULA	2019	40.21	9.82	148.65	RW2
Mozambique	NIASSA	1980	304.94	222.48	403.14	RW2
Mozambique	NIASSA	1981	300.40	232.88	378.38	RW2
Mozambique	NIASSA	1982	295.63	234.26	365.50	RW2
Mozambique	NIASSA	1983	290.71	230.78	359.07	RW2
Mozambique	NIASSA	1984	285.89	227.49	352.22	RW2
Mozambique	NIASSA	1985	281.06	226.30	342.46	RW2
Mozambique	NIASSA	1986	276.42	225.96	332.50	RW2
Mozambique	NIASSA	1987	271.74	224.91	323.69	RW2
Mozambique	NIASSA	1988	266.93	220.87	317.92	RW2
Mozambique	NIASSA	1989	261.95	216.26	312.80	RW2
Mozambique	NIASSA	1990	256.69	213.23	305.68	RW2
Mozambique	NIASSA	1991	251.08	210.79	296.26	RW2
Mozambique	NIASSA	1992	244.83	206.54	287.12	RW2
Mozambique	NIASSA	1993	237.76	200.55	279.08	RW2
Mozambique	NIASSA	1994	229.94	192.41	272.16	RW2
Mozambique	NIASSA	1995	221.44	185.61	261.60	RW2
Mozambique	NIASSA	1996	212.17	179.27	248.89	RW2
Mozambique	NIASSA	1997	202.16	171.64	236.29	RW2
Mozambique	NIASSA	1998	191.56	161.71	226.13	RW2
Mozambique	NIASSA	1999	180.62	150.17	216.24	RW2
Mozambique	NIASSA	2000	169.37	139.38	203.51	RW2
Mozambique	NIASSA	2001	158.45	131.25	190.04	RW2
Mozambique	NIASSA	2002	147.91	122.34	177.82	RW2
Mozambique	NIASSA	2003	137.66	112.75	167.31	RW2
Mozambique	NIASSA	2004	128.09	103.23	157.85	RW2
Mozambique	NIASSA	2005	119.18	94.80	148.83	RW2
Mozambique	NIASSA	2006	110.77	87.90	138.73	RW2
Mozambique	NIASSA	2007	102.94	81.09	129.72	RW2
Mozambique	NIASSA	2008	95.78	74.10	122.79	RW2
Mozambique	NIASSA	2009	89.00	67.24	116.93	RW2
Mozambique	NIASSA	2010	82.96	60.58	112.20	RW2
Mozambique	NIASSA	2011	77.06	55.45	106.19	RW2
Mozambique	NIASSA	2012	71.78	50.27	101.16	RW2
Mozambique	NIASSA	2013	66.70	44.41	98.72	RW2
Mozambique	NIASSA	2014	62.04	37.87	99.12	RW2
Mozambique	NIASSA	2015	57.65	30.73	105.77	RW2
Mozambique	NIASSA	2016	53.53	24.47	113.79	RW2
Mozambique	NIASSA	2017	49.76	18.84	126.09	RW2
Mozambique	NIASSA	2018	46.04	13.99	141.09	RW2
Mozambique	NIASSA	2019	42.68	10.45	159.82	RW2

Continued on next page

Country	Region	Year	Median	Lower	Upper	Method
Mozambique	SOFALA	1980	342.12	267.31	425.64	RW2
Mozambique	SOFALA	1981	335.46	278.17	398.00	RW2
Mozambique	SOFALA	1982	329.15	277.75	384.93	RW2
Mozambique	SOFALA	1983	322.34	270.67	379.16	RW2
Mozambique	SOFALA	1984	315.78	264.08	372.87	RW2
Mozambique	SOFALA	1985	309.16	260.96	361.94	RW2
Mozambique	SOFALA	1986	302.33	258.29	350.06	RW2
Mozambique	SOFALA	1987	295.53	254.89	340.58	RW2
Mozambique	SOFALA	1988	288.31	247.23	333.54	RW2
Mozambique	SOFALA	1989	280.76	238.65	326.88	RW2
Mozambique	SOFALA	1990	272.98	232.91	317.19	RW2
Mozambique	SOFALA	1991	264.56	227.46	305.77	RW2
Mozambique	SOFALA	1992	255.52	220.69	294.14	RW2
Mozambique	SOFALA	1993	245.97	210.43	285.17	RW2
Mozambique	SOFALA	1994	235.87	199.88	275.66	RW2
Mozambique	SOFALA	1995	225.33	190.55	264.08	RW2
Mozambique	SOFALA	1996	214.44	182.96	249.97	RW2
Mozambique	SOFALA	1997	203.11	173.74	236.23	RW2
Mozambique	SOFALA	1998	191.81	162.68	224.66	RW2
Mozambique	SOFALA	1999	180.50	151.02	214.40	RW2
Mozambique	SOFALA	2000	169.10	140.73	201.70	RW2
Mozambique	SOFALA	2001	158.34	132.34	188.17	RW2
Mozambique	SOFALA	2002	148.22	124.05	176.37	RW2
Mozambique	SOFALA	2003	138.55	114.49	166.32	RW2
Mozambique	SOFALA	2004	129.65	105.35	158.44	RW2
Mozambique	SOFALA	2005	121.34	97.42	150.24	RW2
Mozambique	SOFALA	2006	113.78	91.08	140.95	RW2
Mozambique	SOFALA	2007	106.50	84.97	132.96	RW2
Mozambique	SOFALA	2008	99.93	78.41	126.43	RW2
Mozambique	SOFALA	2009	93.64	71.97	121.21	RW2
Mozambique	SOFALA	2010	87.97	66.28	116.17	RW2
Mozambique	SOFALA	2011	82.45	61.88	109.17	RW2
Mozambique	SOFALA	2012	77.33	57.61	104.09	RW2
Mozambique	SOFALA	2013	72.56	51.91	101.14	RW2
Mozambique	SOFALA	2014	68.03	44.76	102.41	RW2
Mozambique	SOFALA	2015	63.77	36.58	109.17	RW2
Mozambique	SOFALA	2016	59.76	29.08	118.94	RW2
Mozambique	SOFALA	2017	55.93	22.72	132.17	RW2
Mozambique	SOFALA	2018	52.21	17.48	148.03	RW2
Mozambique	SOFALA	2019	48.97	12.95	169.78	RW2
Mozambique	TETE	1980	304.61	230.52	389.97	RW2
Mozambique	TETE	1981	303.30	244.46	369.08	RW2
Mozambique	TETE	1982	301.92	248.46	361.07	RW2
Mozambique	TETE	1983	300.27	247.47	359.10	RW2
Mozambique	TETE	1984	298.72	245.60	358.23	RW2
Mozambique	TETE	1985	297.29	247.42	351.40	RW2
Mozambique	TETE	1986	295.26	250.14	344.40	RW2
Mozambique	TETE	1987	293.11	250.66	339.25	RW2
Mozambique	TETE	1988	290.78	248.49	336.83	RW2
Mozambique	TETE	1989	287.95	244.64	334.86	RW2
Mozambique	TETE	1990	284.58	243.69	330.53	RW2
Mozambique	TETE	1991	280.65	242.00	323.07	RW2
Mozambique	TETE	1992	275.74	239.16	315.64	RW2
Mozambique	TETE	1993	270.12	233.38	310.54	RW2
Mozambique	TETE	1994	263.52	225.20	305.62	RW2
Mozambique	TETE	1995	256.16	219.26	297.18	RW2
Mozambique	TETE	1996	247.97	213.70	285.80	RW2
Mozambique	TETE	1997	239.16	206.82	274.90	RW2
Mozambique	TETE	1998	229.77	196.77	266.04	RW2
Mozambique	TETE	1999	219.88	186.03	257.51	RW2
Mozambique	TETE	2000	209.87	176.93	246.65	RW2
Mozambique	TETE	2001	200.05	169.23	234.55	RW2
Mozambique	TETE	2002	190.72	161.81	223.55	RW2

Continued on next page

Country	Region	Year	Median	Lower	Upper	Method
Mozambique	TETE	2003	181.58	152.29	214.74	RW2
Mozambique	TETE	2004	173.06	143.18	207.68	RW2
Mozambique	TETE	2005	164.99	135.11	199.93	RW2
Mozambique	TETE	2006	157.51	129.14	190.88	RW2
Mozambique	TETE	2007	150.44	122.63	183.26	RW2
Mozambique	TETE	2008	143.85	115.46	177.67	RW2
Mozambique	TETE	2009	137.64	108.00	173.68	RW2
Mozambique	TETE	2010	131.67	101.13	170.07	RW2
Mozambique	TETE	2011	126.18	95.76	164.76	RW2
Mozambique	TETE	2012	120.74	90.38	160.31	RW2
Mozambique	TETE	2013	115.60	83.00	160.11	RW2
Mozambique	TETE	2014	110.60	73.25	164.57	RW2
Mozambique	TETE	2015	105.94	60.98	178.57	RW2
Mozambique	TETE	2016	101.20	49.66	195.81	RW2
Mozambique	TETE	2017	96.88	39.51	221.84	RW2
Mozambique	TETE	2018	92.69	30.71	249.22	RW2
Mozambique	TETE	2019	88.78	23.72	282.02	RW2
Mozambique	ZAMBEZIA	1980	277.84	210.63	357.80	RW2
Mozambique	ZAMBEZIA	1981	276.63	223.44	337.63	RW2
Mozambique	ZAMBEZIA	1982	275.33	227.35	329.55	RW2
Mozambique	ZAMBEZIA	1983	273.99	225.69	329.15	RW2
Mozambique	ZAMBEZIA	1984	272.53	223.24	328.40	RW2
Mozambique	ZAMBEZIA	1985	271.08	224.89	322.98	RW2
Mozambique	ZAMBEZIA	1986	269.54	226.54	316.61	RW2
Mozambique	ZAMBEZIA	1987	267.53	227.18	311.72	RW2
Mozambique	ZAMBEZIA	1988	265.73	225.23	309.84	RW2
Mozambique	ZAMBEZIA	1989	263.39	222.24	308.78	RW2
Mozambique	ZAMBEZIA	1990	260.74	221.37	304.25	RW2
Mozambique	ZAMBEZIA	1991	257.33	220.85	297.96	RW2
Mozambique	ZAMBEZIA	1992	253.30	218.40	291.51	RW2
Mozambique	ZAMBEZIA	1993	248.55	213.55	286.99	RW2
Mozambique	ZAMBEZIA	1994	242.86	206.40	283.10	RW2
Mozambique	ZAMBEZIA	1995	236.52	200.71	275.82	RW2
Mozambique	ZAMBEZIA	1996	229.35	196.26	265.26	RW2
Mozambique	ZAMBEZIA	1997	221.49	190.16	255.96	RW2
Mozambique	ZAMBEZIA	1998	213.08	182.33	247.74	RW2
Mozambique	ZAMBEZIA	1999	204.33	172.24	240.68	RW2
Mozambique	ZAMBEZIA	2000	195.10	163.42	230.37	RW2
Mozambique	ZAMBEZIA	2001	186.24	157.21	219.06	RW2
Mozambique	ZAMBEZIA	2002	177.42	150.24	208.18	RW2
Mozambique	ZAMBEZIA	2003	169.27	142.12	200.79	RW2
Mozambique	ZAMBEZIA	2004	161.22	133.36	193.73	RW2
Mozambique	ZAMBEZIA	2005	153.75	126.44	185.69	RW2
Mozambique	ZAMBEZIA	2006	146.64	120.87	176.76	RW2
Mozambique	ZAMBEZIA	2007	140.04	115.02	169.21	RW2
Mozambique	ZAMBEZIA	2008	133.67	108.37	163.78	RW2
Mozambique	ZAMBEZIA	2009	127.68	101.30	159.81	RW2
Mozambique	ZAMBEZIA	2010	121.91	94.67	156.29	RW2
Mozambique	ZAMBEZIA	2011	116.62	89.28	150.29	RW2
Mozambique	ZAMBEZIA	2012	111.33	84.24	146.61	RW2
Mozambique	ZAMBEZIA	2013	106.38	77.23	145.96	RW2
Mozambique	ZAMBEZIA	2014	101.48	67.51	150.63	RW2
Mozambique	ZAMBEZIA	2015	97.06	56.10	164.17	RW2
Mozambique	ZAMBEZIA	2016	92.84	45.76	180.27	RW2
Mozambique	ZAMBEZIA	2017	88.38	36.21	200.22	RW2
Mozambique	ZAMBEZIA	2018	84.07	28.24	226.37	RW2
Mozambique	ZAMBEZIA	2019	80.36	21.44	262.03	RW2
Namibia	ALL	1980	98.34	90.95	106.46	IHME
Namibia	ALL	1980	100.82	65.17	152.69	RW2
Namibia	ALL	1980	97.70	88.70	107.30	UN
Namibia	ALL	1981	96.78	89.88	104.23	IHME
Namibia	ALL	1981	97.52	70.09	133.96	RW2
Namibia	ALL	1981	96.50	87.70	105.80	UN

Continued on next page

Country	Region	Year	Median	Lower	Upper	Method
Namibia	ALL	1982	93.68	87.01	100.73	IHME
Namibia	ALL	1982	94.39	71.36	123.84	RW2
Namibia	ALL	1982	94.70	86.20	103.70	UN
Namibia	ALL	1983	89.37	83.04	96.34	IHME
Namibia	ALL	1983	91.15	69.11	119.41	RW2
Namibia	ALL	1983	92.50	84.30	101.20	UN
Namibia	ALL	1984	85.18	78.91	92.17	IHME
Namibia	ALL	1984	88.31	65.94	116.99	RW2
Namibia	ALL	1984	89.80	81.90	98.20	UN
Namibia	ALL	1985	81.54	75.60	88.17	IHME
Namibia	ALL	1985	85.30	64.82	111.59	RW2
Namibia	ALL	1985	87.00	79.50	94.90	UN
Namibia	ALL	1986	78.81	73.28	85.12	IHME
Namibia	ALL	1986	82.64	63.58	106.73	RW2
Namibia	ALL	1986	83.90	76.90	91.50	UN
Namibia	ALL	1987	76.74	70.96	82.59	IHME
Namibia	ALL	1987	80.22	62.40	102.88	RW2
Namibia	ALL	1987	81.10	74.30	88.30	UN
Namibia	ALL	1988	75.49	70.15	80.69	IHME
Namibia	ALL	1988	77.92	60.29	100.23	RW2
Namibia	ALL	1988	78.30	71.90	85.30	UN
Namibia	ALL	1989	73.97	68.95	78.96	IHME
Namibia	ALL	1989	75.89	58.46	98.23	RW2
Namibia	ALL	1989	75.70	69.60	82.50	UN
Namibia	ALL	1990	71.65	67.01	76.28	IHME
Namibia	ALL	1990	74.12	57.33	95.39	RW2
Namibia	ALL	1990	73.50	67.40	80.20	UN
Namibia	ALL	1991	68.73	64.26	73.50	IHME
Namibia	ALL	1991	72.65	56.88	92.07	RW2
Namibia	ALL	1991	71.70	65.50	78.30	UN
Namibia	ALL	1992	65.69	61.26	70.36	IHME
Namibia	ALL	1992	71.48	56.28	90.24	RW2
Namibia	ALL	1992	70.40	64.10	77.20	UN
Namibia	ALL	1993	63.20	59.05	67.91	IHME
Namibia	ALL	1993	70.68	55.40	89.86	RW2
Namibia	ALL	1993	69.70	63.10	76.60	UN
Namibia	ALL	1994	61.15	56.88	65.93	IHME
Namibia	ALL	1994	70.21	54.30	90.72	RW2
Namibia	ALL	1994	69.70	62.80	76.80	UN
Namibia	ALL	1995	60.18	55.92	65.18	IHME
Namibia	ALL	1995	70.06	53.72	90.19	RW2
Namibia	ALL	1995	70.20	63.20	77.50	UN
Namibia	ALL	1996	59.98	55.64	65.01	IHME
Namibia	ALL	1996	70.49	54.36	90.54	RW2
Namibia	ALL	1996	71.20	64.00	78.70	UN
Namibia	ALL	1997	60.53	56.00	65.48	IHME
Namibia	ALL	1997	71.40	55.29	91.00	RW2
Namibia	ALL	1997	72.40	65.20	80.10	UN
Namibia	ALL	1998	61.58	57.18	66.49	IHME
Namibia	ALL	1998	72.76	56.03	93.18	RW2
Namibia	ALL	1998	73.70	66.70	81.40	UN
Namibia	ALL	1999	62.46	57.96	67.82	IHME
Namibia	ALL	1999	74.23	56.69	94.83	RW2
Namibia	ALL	1999	74.90	68.10	82.60	UN
Namibia	ALL	2000	63.71	59.28	68.93	IHME
Namibia	ALL	2000	76.13	59.58	97.71	RW2
Namibia	ALL	2000	75.70	69.10	83.40	UN
Namibia	ALL	2001	64.31	59.69	69.65	IHME
Namibia	ALL	2001	76.94	60.76	97.69	RW2
Namibia	ALL	2001	76.00	69.40	83.60	UN
Namibia	ALL	2002	64.49	59.40	70.08	IHME
Namibia	ALL	2002	76.88	61.12	96.94	RW2
Namibia	ALL	2002	75.80	69.20	83.40	UN

Continued on next page

Country	Region	Year	Median	Lower	Upper	Method
Namibia	ALL	2003	64.74	59.51	70.21	IHME
Namibia	ALL	2003	75.89	60.21	96.08	RW2
Namibia	ALL	2003	75.20	68.50	82.80	UN
Namibia	ALL	2004	65.36	60.07	71.14	IHME
Namibia	ALL	2004	73.82	57.82	94.75	RW2
Namibia	ALL	2004	73.90	67.10	81.60	UN
Namibia	ALL	2005	64.26	59.13	70.31	IHME
Namibia	ALL	2005	70.77	54.86	90.08	RW2
Namibia	ALL	2005	71.70	64.70	79.70	UN
Namibia	ALL	2006	62.31	56.69	68.48	IHME
Namibia	ALL	2006	67.70	53.16	85.18	RW2
Namibia	ALL	2006	68.40	61.20	76.60	UN
Namibia	ALL	2007	61.35	55.60	68.03	IHME
Namibia	ALL	2007	64.51	51.15	80.81	RW2
Namibia	ALL	2007	64.00	56.60	72.40	UN
Namibia	ALL	2008	60.22	54.00	67.42	IHME
Namibia	ALL	2008	61.45	48.32	77.88	RW2
Namibia	ALL	2008	60.10	52.60	69.20	UN
Namibia	ALL	2009	58.72	52.47	65.92	IHME
Namibia	ALL	2009	58.38	44.99	75.63	RW2
Namibia	ALL	2009	56.70	48.70	66.70	UN
Namibia	ALL	2010	55.26	49.10	62.06	IHME
Namibia	ALL	2010	55.59	42.07	73.55	RW2
Namibia	ALL	2010	53.80	45.10	64.80	UN
Namibia	ALL	2011	51.27	45.14	58.20	IHME
Namibia	ALL	2011	52.90	40.17	69.46	RW2
Namibia	ALL	2011	51.60	42.30	64.20	UN
Namibia	ALL	2012	48.75	42.16	56.08	IHME
Namibia	ALL	2012	50.31	38.17	66.08	RW2
Namibia	ALL	2012	49.90	39.80	63.80	UN
Namibia	ALL	2013	46.09	38.89	53.89	IHME
Namibia	ALL	2013	47.89	34.82	65.45	RW2
Namibia	ALL	2013	47.70	37.00	62.60	UN
Namibia	ALL	2014	44.73	36.10	54.41	IHME
Namibia	ALL	2014	45.52	29.68	69.26	RW2
Namibia	ALL	2014	46.40	34.80	62.80	UN
Namibia	ALL	2015	40.52	31.39	51.56	IHME
Namibia	ALL	2015	43.22	23.40	79.02	RW2
Namibia	ALL	2015	45.40	33.00	63.00	UN
Namibia	ALL	2016	41.18	18.35	91.28	RW2
Namibia	ALL	2017	39.02	13.82	107.61	RW2
Namibia	ALL	2018	37.05	10.30	130.13	RW2
Namibia	ALL	2019	35.13	7.19	155.87	RW2
Namibia	ALL	15-19	39.02	14.06	105.54	RW2
Namibia	CAPRIVI	1980	115.16	60.05	211.72	RW2
Namibia	CAPRIVI	1981	111.49	63.65	190.06	RW2
Namibia	CAPRIVI	1982	107.78	64.46	174.93	RW2
Namibia	CAPRIVI	1983	104.44	64.93	164.45	RW2
Namibia	CAPRIVI	1984	101.09	64.36	155.73	RW2
Namibia	CAPRIVI	1985	97.97	64.36	146.65	RW2
Namibia	CAPRIVI	1986	94.78	63.97	137.76	RW2
Namibia	CAPRIVI	1987	91.73	63.52	130.88	RW2
Namibia	CAPRIVI	1988	88.87	62.64	124.86	RW2
Namibia	CAPRIVI	1989	86.44	61.50	120.31	RW2
Namibia	CAPRIVI	1990	84.24	60.62	115.61	RW2
Namibia	CAPRIVI	1991	83.10	60.82	112.77	RW2
Namibia	CAPRIVI	1992	82.70	61.12	110.44	RW2
Namibia	CAPRIVI	1993	83.09	61.54	110.72	RW2
Namibia	CAPRIVI	1994	84.25	62.07	112.71	RW2
Namibia	CAPRIVI	1995	86.42	64.11	115.44	RW2
Namibia	CAPRIVI	1996	88.49	66.16	116.97	RW2
Namibia	CAPRIVI	1997	90.76	68.29	118.89	RW2
Namibia	CAPRIVI	1998	92.64	69.81	121.49	RW2

Continued on next page

Country	Region	Year	Median	Lower	Upper	Method
Namibia	CAPRIVI	1999	94.25	70.53	124.67	RW2
Namibia	CAPRIVI	2000	95.34	71.74	125.46	RW2
Namibia	CAPRIVI	2001	95.57	72.71	124.33	RW2
Namibia	CAPRIVI	2002	95.04	72.74	123.34	RW2
Namibia	CAPRIVI	2003	93.65	71.66	121.73	RW2
Namibia	CAPRIVI	2004	91.74	69.65	120.21	RW2
Namibia	CAPRIVI	2005	88.94	67.09	116.46	RW2
Namibia	CAPRIVI	2006	86.31	65.39	112.85	RW2
Namibia	CAPRIVI	2007	83.41	62.88	109.83	RW2
Namibia	CAPRIVI	2008	80.67	59.94	107.78	RW2
Namibia	CAPRIVI	2009	78.30	56.65	107.31	RW2
Namibia	CAPRIVI	2010	75.96	53.24	106.90	RW2
Namibia	CAPRIVI	2011	73.68	50.98	105.77	RW2
Namibia	CAPRIVI	2012	71.51	47.99	105.62	RW2
Namibia	CAPRIVI	2013	69.43	44.05	107.28	RW2
Namibia	CAPRIVI	2014	67.50	38.94	113.55	RW2
Namibia	CAPRIVI	2015	65.43	32.90	127.02	RW2
Namibia	CAPRIVI	2016	63.41	26.71	142.94	RW2
Namibia	CAPRIVI	2017	61.67	21.28	165.05	RW2
Namibia	CAPRIVI	2018	59.38	16.55	194.65	RW2
Namibia	CAPRIVI	2019	57.59	12.49	229.87	RW2
Namibia	ERONGO	1980	60.52	32.73	109.76	RW2
Namibia	ERONGO	1981	59.16	34.70	99.31	RW2
Namibia	ERONGO	1982	57.82	35.59	92.71	RW2
Namibia	ERONGO	1983	56.49	35.66	88.85	RW2
Namibia	ERONGO	1984	55.15	35.33	85.28	RW2
Namibia	ERONGO	1985	53.92	35.41	81.39	RW2
Namibia	ERONGO	1986	52.68	35.30	77.54	RW2
Namibia	ERONGO	1987	51.34	35.18	74.25	RW2
Namibia	ERONGO	1988	50.32	34.84	71.95	RW2
Namibia	ERONGO	1989	49.38	34.53	70.24	RW2
Namibia	ERONGO	1990	48.54	34.36	67.88	RW2
Namibia	ERONGO	1991	48.25	34.73	66.69	RW2
Namibia	ERONGO	1992	48.45	35.24	66.10	RW2
Namibia	ERONGO	1993	49.16	35.93	66.64	RW2
Namibia	ERONGO	1994	50.24	36.60	68.16	RW2
Namibia	ERONGO	1995	51.94	38.06	70.25	RW2
Namibia	ERONGO	1996	53.62	39.70	71.56	RW2
Namibia	ERONGO	1997	55.35	41.24	73.45	RW2
Namibia	ERONGO	1998	56.99	42.69	75.38	RW2
Namibia	ERONGO	1999	58.41	43.43	77.54	RW2
Namibia	ERONGO	2000	59.44	44.37	78.58	RW2
Namibia	ERONGO	2001	60.05	45.30	78.84	RW2
Namibia	ERONGO	2002	60.05	45.54	78.52	RW2
Namibia	ERONGO	2003	59.76	45.04	78.96	RW2
Namibia	ERONGO	2004	58.87	43.88	78.67	RW2
Namibia	ERONGO	2005	57.56	42.64	76.96	RW2
Namibia	ERONGO	2006	56.29	41.74	75.29	RW2
Namibia	ERONGO	2007	55.06	40.56	74.04	RW2
Namibia	ERONGO	2008	53.79	39.08	73.48	RW2
Namibia	ERONGO	2009	52.63	37.43	73.45	RW2
Namibia	ERONGO	2010	51.55	35.98	73.59	RW2
Namibia	ERONGO	2011	50.62	34.78	72.48	RW2
Namibia	ERONGO	2012	49.59	33.78	72.57	RW2
Namibia	ERONGO	2013	48.67	31.94	74.02	RW2
Namibia	ERONGO	2014	47.67	28.74	78.48	RW2
Namibia	ERONGO	2015	46.86	24.52	88.77	RW2
Namibia	ERONGO	2016	46.07	20.45	101.60	RW2
Namibia	ERONGO	2017	45.06	16.48	118.13	RW2
Namibia	ERONGO	2018	44.04	13.07	140.54	RW2
Namibia	ERONGO	2019	43.29	10.06	172.28	RW2
Namibia	HARDAP	1980	142.37	82.33	235.11	RW2
Namibia	HARDAP	1981	134.03	83.73	208.01	RW2

Continued on next page

Country	Region	Year	Median	Lower	Upper	Method
Namibia	HARDAP	1982	126.14	81.76	189.71	RW2
Namibia	HARDAP	1983	118.44	78.47	175.27	RW2
Namibia	HARDAP	1984	111.30	74.58	163.38	RW2
Namibia	HARDAP	1985	104.80	71.40	150.42	RW2
Namibia	HARDAP	1986	98.26	68.61	138.63	RW2
Namibia	HARDAP	1987	92.23	65.45	128.48	RW2
Namibia	HARDAP	1988	86.80	62.18	120.03	RW2
Namibia	HARDAP	1989	81.87	58.96	112.62	RW2
Namibia	HARDAP	1990	77.25	56.43	105.22	RW2
Namibia	HARDAP	1991	73.85	54.44	99.31	RW2
Namibia	HARDAP	1992	71.12	53.00	94.66	RW2
Namibia	HARDAP	1993	69.20	51.68	91.97	RW2
Namibia	HARDAP	1994	67.82	50.32	90.43	RW2
Namibia	HARDAP	1995	67.15	50.19	89.62	RW2
Namibia	HARDAP	1996	66.39	49.86	88.01	RW2
Namibia	HARDAP	1997	65.68	49.45	86.68	RW2
Namibia	HARDAP	1998	64.76	48.33	85.77	RW2
Namibia	HARDAP	1999	63.45	47.02	84.58	RW2
Namibia	HARDAP	2000	61.89	45.97	82.67	RW2
Namibia	HARDAP	2001	59.78	44.42	79.85	RW2
Namibia	HARDAP	2002	57.36	42.65	76.74	RW2
Namibia	HARDAP	2003	54.44	40.00	73.58	RW2
Namibia	HARDAP	2004	51.36	37.31	70.44	RW2
Namibia	HARDAP	2005	47.96	34.43	66.26	RW2
Namibia	HARDAP	2006	44.84	32.14	62.21	RW2
Namibia	HARDAP	2007	41.84	29.66	58.69	RW2
Namibia	HARDAP	2008	39.06	27.16	55.78	RW2
Namibia	HARDAP	2009	36.49	24.72	53.44	RW2
Namibia	HARDAP	2010	34.10	22.57	51.34	RW2
Namibia	HARDAP	2011	31.95	20.75	48.87	RW2
Namibia	HARDAP	2012	29.87	19.07	46.66	RW2
Namibia	HARDAP	2013	27.96	17.10	45.74	RW2
Namibia	HARDAP	2014	26.15	14.77	46.09	RW2
Namibia	HARDAP	2015	24.50	12.00	49.56	RW2
Namibia	HARDAP	2016	22.87	9.49	54.30	RW2
Namibia	HARDAP	2017	21.42	7.32	62.38	RW2
Namibia	HARDAP	2018	20.04	5.50	71.23	RW2
Namibia	HARDAP	2019	18.78	4.10	82.69	RW2
Namibia	KARAS	1980	130.88	78.38	211.15	RW2
Namibia	KARAS	1981	123.74	80.64	186.94	RW2
Namibia	KARAS	1982	117.37	79.54	169.96	RW2
Namibia	KARAS	1983	111.02	76.96	158.58	RW2
Namibia	KARAS	1984	104.94	73.21	148.36	RW2
Namibia	KARAS	1985	99.35	70.49	138.30	RW2
Namibia	KARAS	1986	93.90	67.90	128.87	RW2
Namibia	KARAS	1987	88.89	64.93	120.41	RW2
Namibia	KARAS	1988	84.30	62.06	113.87	RW2
Namibia	KARAS	1989	80.13	58.91	108.43	RW2
Namibia	KARAS	1990	76.19	56.36	101.93	RW2
Namibia	KARAS	1991	73.42	55.01	97.23	RW2
Namibia	KARAS	1992	71.34	53.44	93.99	RW2
Namibia	KARAS	1993	70.03	52.40	92.48	RW2
Namibia	KARAS	1994	69.19	51.04	92.10	RW2
Namibia	KARAS	1995	69.28	51.03	92.69	RW2
Namibia	KARAS	1996	69.16	51.16	92.06	RW2
Namibia	KARAS	1997	69.15	51.00	91.79	RW2
Namibia	KARAS	1998	68.96	50.69	92.08	RW2
Namibia	KARAS	1999	68.49	50.09	91.82	RW2
Namibia	KARAS	2000	67.77	49.64	90.98	RW2
Namibia	KARAS	2001	66.50	49.07	88.76	RW2
Namibia	KARAS	2002	64.69	48.10	86.31	RW2
Namibia	KARAS	2003	62.54	46.03	84.23	RW2
Namibia	KARAS	2004	60.09	44.03	81.74	RW2

Continued on next page

Country	Region	Year	Median	Lower	Upper	Method
Namibia	KARAS	2005	57.16	41.45	78.22	RW2
Namibia	KARAS	2006	54.52	39.53	74.89	RW2
Namibia	KARAS	2007	51.91	37.26	72.06	RW2
Namibia	KARAS	2008	49.38	34.64	70.06	RW2
Namibia	KARAS	2009	47.09	32.02	68.95	RW2
Namibia	KARAS	2010	44.88	29.64	68.17	RW2
Namibia	KARAS	2011	42.86	27.62	66.47	RW2
Namibia	KARAS	2012	40.88	25.55	65.80	RW2
Namibia	KARAS	2013	39.07	23.12	66.44	RW2
Namibia	KARAS	2014	37.28	20.20	69.68	RW2
Namibia	KARAS	2015	35.53	16.65	76.47	RW2
Namibia	KARAS	2016	33.89	13.43	85.19	RW2
Namibia	KARAS	2017	32.28	10.58	97.71	RW2
Namibia	KARAS	2018	30.76	8.00	115.08	RW2
Namibia	KARAS	2019	29.41	5.97	137.31	RW2
Namibia	KAVANGO	1980	128.25	78.03	204.93	RW2
Namibia	KAVANGO	1981	123.84	81.98	184.06	RW2
Namibia	KAVANGO	1982	119.85	82.72	171.42	RW2
Namibia	KAVANGO	1983	115.55	81.37	162.65	RW2
Namibia	KAVANGO	1984	111.58	79.76	154.83	RW2
Namibia	KAVANGO	1985	107.82	78.51	146.76	RW2
Namibia	KAVANGO	1986	104.03	77.23	138.83	RW2
Namibia	KAVANGO	1987	100.54	76.11	132.50	RW2
Namibia	KAVANGO	1988	97.20	73.89	127.43	RW2
Namibia	KAVANGO	1989	94.26	71.77	123.20	RW2
Namibia	KAVANGO	1990	91.60	70.32	118.07	RW2
Namibia	KAVANGO	1991	90.02	70.08	114.80	RW2
Namibia	KAVANGO	1992	89.25	70.34	112.55	RW2
Namibia	KAVANGO	1993	89.39	70.24	112.65	RW2
Namibia	KAVANGO	1994	90.30	70.82	113.65	RW2
Namibia	KAVANGO	1995	92.14	72.90	115.88	RW2
Namibia	KAVANGO	1996	94.02	75.41	116.91	RW2
Namibia	KAVANGO	1997	95.87	77.36	118.22	RW2
Namibia	KAVANGO	1998	97.62	78.40	120.52	RW2
Namibia	KAVANGO	1999	98.92	78.73	123.23	RW2
Namibia	KAVANGO	2000	99.58	79.34	124.29	RW2
Namibia	KAVANGO	2001	99.48	79.75	123.31	RW2
Namibia	KAVANGO	2002	98.66	79.41	122.13	RW2
Namibia	KAVANGO	2003	96.94	77.41	120.60	RW2
Namibia	KAVANGO	2004	94.69	74.87	119.33	RW2
Namibia	KAVANGO	2005	91.69	72.12	115.60	RW2
Namibia	KAVANGO	2006	88.90	70.25	111.45	RW2
Namibia	KAVANGO	2007	85.87	67.93	108.17	RW2
Namibia	KAVANGO	2008	83.12	64.63	106.16	RW2
Namibia	KAVANGO	2009	80.36	60.98	105.36	RW2
Namibia	KAVANGO	2010	78.01	57.78	104.85	RW2
Namibia	KAVANGO	2011	75.51	55.56	101.99	RW2
Namibia	KAVANGO	2012	73.17	53.25	100.83	RW2
Namibia	KAVANGO	2013	70.96	49.31	101.72	RW2
Namibia	KAVANGO	2014	68.76	43.62	107.19	RW2
Namibia	KAVANGO	2015	66.63	36.54	119.17	RW2
Namibia	KAVANGO	2016	64.56	29.70	135.43	RW2
Namibia	KAVANGO	2017	62.48	23.73	156.85	RW2
Namibia	KAVANGO	2018	60.30	18.64	182.69	RW2
Namibia	KAVANGO	2019	58.51	14.07	217.41	RW2
Namibia	KHOMAS	1980	84.77	45.68	152.43	RW2
Namibia	KHOMAS	1981	81.68	47.75	136.20	RW2
Namibia	KHOMAS	1982	78.38	47.70	125.93	RW2
Namibia	KHOMAS	1983	75.30	46.78	118.84	RW2
Namibia	KHOMAS	1984	72.33	45.66	112.54	RW2
Namibia	KHOMAS	1985	69.60	45.07	106.12	RW2
Namibia	KHOMAS	1986	66.85	44.18	99.35	RW2
Namibia	KHOMAS	1987	64.18	43.41	94.03	RW2

Continued on next page

Country	Region	Year	Median	Lower	Upper	Method
Namibia	KHOMAS	1988	61.70	42.34	88.99	RW2
Namibia	KHOMAS	1989	59.51	41.56	84.89	RW2
Namibia	KHOMAS	1990	57.58	40.60	80.74	RW2
Namibia	KHOMAS	1991	56.31	40.48	77.57	RW2
Namibia	KHOMAS	1992	55.53	40.45	75.74	RW2
Namibia	KHOMAS	1993	55.27	40.58	74.89	RW2
Namibia	KHOMAS	1994	55.48	40.85	75.02	RW2
Namibia	KHOMAS	1995	56.33	41.53	75.92	RW2
Namibia	KHOMAS	1996	56.98	42.70	76.11	RW2
Namibia	KHOMAS	1997	57.70	43.40	76.17	RW2
Namibia	KHOMAS	1998	58.10	43.71	76.65	RW2
Namibia	KHOMAS	1999	58.30	43.82	77.27	RW2
Namibia	KHOMAS	2000	58.11	43.60	76.69	RW2
Namibia	KHOMAS	2001	57.43	43.55	75.35	RW2
Namibia	KHOMAS	2002	56.20	42.88	73.50	RW2
Namibia	KHOMAS	2003	54.56	41.45	71.72	RW2
Namibia	KHOMAS	2004	52.57	39.43	69.86	RW2
Namibia	KHOMAS	2005	50.17	37.44	66.82	RW2
Namibia	KHOMAS	2006	47.93	35.77	63.84	RW2
Namibia	KHOMAS	2007	45.69	33.88	61.43	RW2
Namibia	KHOMAS	2008	43.57	31.73	59.57	RW2
Namibia	KHOMAS	2009	41.54	29.35	58.56	RW2
Namibia	KHOMAS	2010	39.76	27.21	57.64	RW2
Namibia	KHOMAS	2011	38.01	25.57	56.07	RW2
Namibia	KHOMAS	2012	36.32	23.81	54.99	RW2
Namibia	KHOMAS	2013	34.70	21.76	54.91	RW2
Namibia	KHOMAS	2014	33.16	19.12	57.00	RW2
Namibia	KHOMAS	2015	31.71	15.72	62.69	RW2
Namibia	KHOMAS	2016	30.38	12.85	70.22	RW2
Namibia	KHOMAS	2017	28.90	10.08	81.55	RW2
Namibia	KHOMAS	2018	27.65	7.76	95.06	RW2
Namibia	KHOMAS	2019	26.45	5.85	115.14	RW2
Namibia	KUNENE	1980	125.81	72.90	208.87	RW2
Namibia	KUNENE	1981	120.03	75.48	186.92	RW2
Namibia	KUNENE	1982	114.52	74.67	171.22	RW2
Namibia	KUNENE	1983	109.07	72.53	160.76	RW2
Namibia	KUNENE	1984	103.93	70.14	151.88	RW2
Namibia	KUNENE	1985	98.94	68.04	141.84	RW2
Namibia	KUNENE	1986	94.14	66.22	132.75	RW2
Namibia	KUNENE	1987	89.63	64.15	124.46	RW2
Namibia	KUNENE	1988	85.35	61.66	117.15	RW2
Namibia	KUNENE	1989	81.50	59.46	111.55	RW2
Namibia	KUNENE	1990	77.97	57.28	104.77	RW2
Namibia	KUNENE	1991	75.51	56.43	100.23	RW2
Namibia	KUNENE	1992	73.60	55.66	96.30	RW2
Namibia	KUNENE	1993	72.54	55.00	94.88	RW2
Namibia	KUNENE	1994	72.16	54.48	94.41	RW2
Namibia	KUNENE	1995	72.38	54.84	94.84	RW2
Namibia	KUNENE	1996	72.69	55.58	94.04	RW2
Namibia	KUNENE	1997	72.80	55.84	93.85	RW2
Namibia	KUNENE	1998	72.74	55.61	93.89	RW2
Namibia	KUNENE	1999	72.50	55.07	94.28	RW2
Namibia	KUNENE	2000	71.74	54.56	93.18	RW2
Namibia	KUNENE	2001	70.38	54.03	90.82	RW2
Namibia	KUNENE	2002	68.53	52.43	88.47	RW2
Namibia	KUNENE	2003	66.14	50.35	86.34	RW2
Namibia	KUNENE	2004	63.45	47.76	83.68	RW2
Namibia	KUNENE	2005	60.25	45.11	79.79	RW2
Namibia	KUNENE	2006	57.40	43.07	76.06	RW2
Namibia	KUNENE	2007	54.48	40.74	72.22	RW2
Namibia	KUNENE	2008	51.76	38.17	69.93	RW2
Namibia	KUNENE	2009	49.35	35.58	68.04	RW2
Namibia	KUNENE	2010	47.11	33.28	66.42	RW2

Continued on next page

Country	Region	Year	Median	Lower	Upper	Method
Namibia	KUNENE	2011	44.97	31.50	63.81	RW2
Namibia	KUNENE	2012	42.88	29.67	61.85	RW2
Namibia	KUNENE	2013	40.94	27.27	61.36	RW2
Namibia	KUNENE	2014	39.08	23.89	63.45	RW2
Namibia	KUNENE	2015	37.28	19.74	70.05	RW2
Namibia	KUNENE	2016	35.49	16.00	78.52	RW2
Namibia	KUNENE	2017	33.93	12.45	90.27	RW2
Namibia	KUNENE	2018	32.27	9.49	105.14	RW2
Namibia	KUNENE	2019	30.73	7.18	125.57	RW2
Namibia	OHANGWENA	1980	147.17	92.09	226.57	RW2
Namibia	OHANGWENA	1981	141.46	95.16	204.52	RW2
Namibia	OHANGWENA	1982	135.94	95.45	190.41	RW2
Namibia	OHANGWENA	1983	130.58	92.68	180.63	RW2
Namibia	OHANGWENA	1984	125.23	89.29	173.13	RW2
Namibia	OHANGWENA	1985	120.41	87.18	164.14	RW2
Namibia	OHANGWENA	1986	115.67	85.21	154.94	RW2
Namibia	OHANGWENA	1987	111.16	82.80	147.19	RW2
Namibia	OHANGWENA	1988	106.92	80.33	140.90	RW2
Namibia	OHANGWENA	1989	103.13	77.81	136.01	RW2
Namibia	OHANGWENA	1990	99.63	75.83	129.68	RW2
Namibia	OHANGWENA	1991	97.39	75.21	124.99	RW2
Namibia	OHANGWENA	1992	96.22	74.83	122.28	RW2
Namibia	OHANGWENA	1993	95.74	74.52	121.98	RW2
Namibia	OHANGWENA	1994	96.27	74.76	122.78	RW2
Namibia	OHANGWENA	1995	97.61	76.26	124.82	RW2
Namibia	OHANGWENA	1996	98.98	77.89	125.22	RW2
Namibia	OHANGWENA	1997	100.27	79.41	125.75	RW2
Namibia	OHANGWENA	1998	101.21	79.94	127.40	RW2
Namibia	OHANGWENA	1999	101.68	80.13	128.40	RW2
Namibia	OHANGWENA	2000	101.60	80.05	128.14	RW2
Namibia	OHANGWENA	2001	100.52	80.22	125.77	RW2
Namibia	OHANGWENA	2002	98.64	78.93	122.94	RW2
Namibia	OHANGWENA	2003	95.83	76.12	120.43	RW2
Namibia	OHANGWENA	2004	92.45	72.35	117.97	RW2
Namibia	OHANGWENA	2005	88.36	68.51	113.05	RW2
Namibia	OHANGWENA	2006	84.39	65.70	108.04	RW2
Namibia	OHANGWENA	2007	80.41	62.21	103.15	RW2
Namibia	OHANGWENA	2008	76.66	58.30	100.36	RW2
Namibia	OHANGWENA	2009	73.12	54.24	97.97	RW2
Namibia	OHANGWENA	2010	69.93	50.50	96.44	RW2
Namibia	OHANGWENA	2011	66.73	47.57	92.84	RW2
Namibia	OHANGWENA	2012	63.80	44.55	90.24	RW2
Namibia	OHANGWENA	2013	60.99	40.62	90.12	RW2
Namibia	OHANGWENA	2014	58.29	35.56	93.64	RW2
Namibia	OHANGWENA	2015	55.52	29.14	102.35	RW2
Namibia	OHANGWENA	2016	52.99	23.65	114.38	RW2
Namibia	OHANGWENA	2017	50.65	18.66	130.83	RW2
Namibia	OHANGWENA	2018	48.34	14.43	152.98	RW2
Namibia	OHANGWENA	2019	46.08	10.55	181.75	RW2
Namibia	OMAHEKE	1980	91.23	48.26	159.93	RW2
Namibia	OMAHEKE	1981	89.10	51.29	144.87	RW2
Namibia	OMAHEKE	1982	86.72	52.90	135.22	RW2
Namibia	OMAHEKE	1983	84.46	53.19	128.26	RW2
Namibia	OMAHEKE	1984	82.25	53.05	123.10	RW2
Namibia	OMAHEKE	1985	80.19	53.56	116.94	RW2
Namibia	OMAHEKE	1986	78.03	54.27	110.94	RW2
Namibia	OMAHEKE	1987	75.98	54.14	105.60	RW2
Namibia	OMAHEKE	1988	74.21	53.60	101.84	RW2
Namibia	OMAHEKE	1989	72.66	53.42	98.62	RW2
Namibia	OMAHEKE	1990	71.18	53.10	94.64	RW2
Namibia	OMAHEKE	1991	70.46	53.55	91.99	RW2
Namibia	OMAHEKE	1992	70.31	54.23	90.78	RW2
Namibia	OMAHEKE	1993	70.88	54.77	91.11	RW2

Continued on next page

Country	Region	Year	Median	Lower	Upper	Method
Namibia	OMAHEKE	1994	71.97	55.55	92.81	RW2
Namibia	OMAHEKE	1995	73.53	57.08	94.82	RW2
Namibia	OMAHEKE	1996	75.07	59.04	95.70	RW2
Namibia	OMAHEKE	1997	76.36	60.18	97.37	RW2
Namibia	OMAHEKE	1998	77.49	60.63	99.22	RW2
Namibia	OMAHEKE	1999	78.12	60.39	101.10	RW2
Namibia	OMAHEKE	2000	78.11	60.01	101.77	RW2
Namibia	OMAHEKE	2001	77.45	59.53	100.85	RW2
Namibia	OMAHEKE	2002	76.03	58.02	99.38	RW2
Namibia	OMAHEKE	2003	73.94	55.67	97.77	RW2
Namibia	OMAHEKE	2004	71.46	53.12	95.75	RW2
Namibia	OMAHEKE	2005	68.30	49.98	92.71	RW2
Namibia	OMAHEKE	2006	65.31	47.71	88.67	RW2
Namibia	OMAHEKE	2007	62.30	45.12	85.46	RW2
Namibia	OMAHEKE	2008	59.45	42.00	83.36	RW2
Namibia	OMAHEKE	2009	56.76	39.08	81.71	RW2
Namibia	OMAHEKE	2010	54.25	36.26	80.66	RW2
Namibia	OMAHEKE	2011	51.94	33.87	78.44	RW2
Namibia	OMAHEKE	2012	49.72	31.63	76.48	RW2
Namibia	OMAHEKE	2013	47.55	28.82	76.18	RW2
Namibia	OMAHEKE	2014	45.45	25.29	78.99	RW2
Namibia	OMAHEKE	2015	43.43	21.09	86.37	RW2
Namibia	OMAHEKE	2016	41.65	16.94	96.03	RW2
Namibia	OMAHEKE	2017	39.58	13.49	109.84	RW2
Namibia	OMAHEKE	2018	37.79	10.41	127.29	RW2
Namibia	OMAHEKE	2019	36.28	7.75	150.52	RW2
Namibia	OMUSATI	1980	140.87	79.37	238.36	RW2
Namibia	OMUSATI	1981	134.07	81.78	212.08	RW2
Namibia	OMUSATI	1982	127.20	80.95	194.44	RW2
Namibia	OMUSATI	1983	120.85	78.49	181.50	RW2
Namibia	OMUSATI	1984	114.40	75.76	169.34	RW2
Namibia	OMUSATI	1985	108.54	73.75	157.26	RW2
Namibia	OMUSATI	1986	103.03	71.49	146.34	RW2
Namibia	OMUSATI	1987	97.64	69.28	136.13	RW2
Namibia	OMUSATI	1988	92.49	66.42	127.70	RW2
Namibia	OMUSATI	1989	88.07	63.79	120.93	RW2
Namibia	OMUSATI	1990	83.92	61.53	113.64	RW2
Namibia	OMUSATI	1991	80.92	60.17	108.01	RW2
Namibia	OMUSATI	1992	78.75	59.21	103.25	RW2
Namibia	OMUSATI	1993	77.27	58.43	101.20	RW2
Namibia	OMUSATI	1994	76.59	58.02	100.12	RW2
Namibia	OMUSATI	1995	76.61	58.32	99.92	RW2
Namibia	OMUSATI	1996	76.65	59.16	98.86	RW2
Namibia	OMUSATI	1997	76.55	59.39	97.74	RW2
Namibia	OMUSATI	1998	76.33	59.27	97.80	RW2
Namibia	OMUSATI	1999	75.62	58.36	97.43	RW2
Namibia	OMUSATI	2000	74.55	57.66	96.03	RW2
Namibia	OMUSATI	2001	72.75	56.53	93.26	RW2
Namibia	OMUSATI	2002	70.39	54.80	89.88	RW2
Namibia	OMUSATI	2003	67.53	52.09	87.39	RW2
Namibia	OMUSATI	2004	64.23	49.03	84.20	RW2
Namibia	OMUSATI	2005	60.59	45.82	79.79	RW2
Namibia	OMUSATI	2006	57.11	43.00	75.34	RW2
Namibia	OMUSATI	2007	53.73	40.23	71.51	RW2
Namibia	OMUSATI	2008	50.58	37.01	68.53	RW2
Namibia	OMUSATI	2009	47.60	33.90	66.29	RW2
Namibia	OMUSATI	2010	44.96	30.98	64.83	RW2
Namibia	OMUSATI	2011	42.34	28.58	62.10	RW2
Namibia	OMUSATI	2012	39.87	26.38	59.79	RW2
Namibia	OMUSATI	2013	37.62	23.81	59.06	RW2
Namibia	OMUSATI	2014	35.54	20.45	61.04	RW2
Namibia	OMUSATI	2015	33.46	16.69	65.90	RW2
Namibia	OMUSATI	2016	31.52	13.26	73.21	RW2

Continued on next page

Country	Region	Year	Median	Lower	Upper	Method
Namibia	OMUSATI	2017	29.64	10.32	82.62	RW2
Namibia	OMUSATI	2018	27.98	7.82	96.02	RW2
Namibia	OMUSATI	2019	26.43	5.77	112.50	RW2
Namibia	OSHANA	1980	128.01	78.05	202.15	RW2
Namibia	OSHANA	1981	123.03	80.99	182.16	RW2
Namibia	OSHANA	1982	118.03	80.75	168.24	RW2
Namibia	OSHANA	1983	113.27	78.77	160.22	RW2
Namibia	OSHANA	1984	108.71	76.08	153.02	RW2
Namibia	OSHANA	1985	104.15	74.05	144.14	RW2
Namibia	OSHANA	1986	99.89	72.44	136.50	RW2
Namibia	OSHANA	1987	95.73	70.43	129.37	RW2
Namibia	OSHANA	1988	91.93	68.02	123.22	RW2
Namibia	OSHANA	1989	88.57	66.07	118.59	RW2
Namibia	OSHANA	1990	85.32	64.19	112.42	RW2
Namibia	OSHANA	1991	83.29	63.62	108.09	RW2
Namibia	OSHANA	1992	81.93	63.35	105.37	RW2
Namibia	OSHANA	1993	81.45	62.89	104.65	RW2
Namibia	OSHANA	1994	81.62	62.77	104.82	RW2
Namibia	OSHANA	1995	82.67	64.03	106.20	RW2
Namibia	OSHANA	1996	83.53	65.38	106.54	RW2
Namibia	OSHANA	1997	84.38	66.02	106.75	RW2
Namibia	OSHANA	1998	84.97	66.63	107.91	RW2
Namibia	OSHANA	1999	85.12	66.11	108.86	RW2
Namibia	OSHANA	2000	84.78	65.93	108.85	RW2
Namibia	OSHANA	2001	83.67	65.27	107.05	RW2
Namibia	OSHANA	2002	81.80	63.89	104.45	RW2
Namibia	OSHANA	2003	79.21	61.15	102.52	RW2
Namibia	OSHANA	2004	76.26	57.89	99.87	RW2
Namibia	OSHANA	2005	72.66	54.44	95.99	RW2
Namibia	OSHANA	2006	69.26	51.77	91.74	RW2
Namibia	OSHANA	2007	65.90	48.87	88.37	RW2
Namibia	OSHANA	2008	62.64	45.46	85.87	RW2
Namibia	OSHANA	2009	59.68	42.13	83.93	RW2
Namibia	OSHANA	2010	56.84	38.81	82.40	RW2
Namibia	OSHANA	2011	54.19	36.41	79.64	RW2
Namibia	OSHANA	2012	51.68	33.87	77.93	RW2
Namibia	OSHANA	2013	49.20	30.73	77.56	RW2
Namibia	OSHANA	2014	46.90	26.91	80.33	RW2
Namibia	OSHANA	2015	44.72	22.32	87.11	RW2
Namibia	OSHANA	2016	42.65	18.12	97.88	RW2
Namibia	OSHANA	2017	40.46	14.19	111.48	RW2
Namibia	OSHANA	2018	38.58	10.84	130.35	RW2
Namibia	OSHANA	2019	36.81	8.13	153.71	RW2
Namibia	OSHIKOTO	1980	91.05	51.10	154.81	RW2
Namibia	OSHIKOTO	1981	89.11	54.34	141.20	RW2
Namibia	OSHIKOTO	1982	87.47	56.15	132.09	RW2
Namibia	OSHIKOTO	1983	85.61	56.26	126.52	RW2
Namibia	OSHIKOTO	1984	83.69	56.44	121.56	RW2
Namibia	OSHIKOTO	1985	82.02	57.07	116.51	RW2
Namibia	OSHIKOTO	1986	80.28	56.97	111.62	RW2
Namibia	OSHIKOTO	1987	78.63	57.29	107.05	RW2
Namibia	OSHIKOTO	1988	77.10	56.83	103.87	RW2
Namibia	OSHIKOTO	1989	75.79	56.22	101.67	RW2
Namibia	OSHIKOTO	1990	74.66	55.99	98.78	RW2
Namibia	OSHIKOTO	1991	74.30	56.54	96.58	RW2
Namibia	OSHIKOTO	1992	74.60	57.71	95.73	RW2
Namibia	OSHIKOTO	1993	75.62	58.57	96.98	RW2
Namibia	OSHIKOTO	1994	77.20	59.59	99.00	RW2
Namibia	OSHIKOTO	1995	79.57	62.07	101.84	RW2
Namibia	OSHIKOTO	1996	81.82	64.69	103.55	RW2
Namibia	OSHIKOTO	1997	84.15	66.91	105.49	RW2
Namibia	OSHIKOTO	1998	86.28	68.41	108.37	RW2
Namibia	OSHIKOTO	1999	87.81	69.42	111.01	RW2

Continued on next page

Country	Region	Year	Median	Lower	Upper	Method
Namibia	OSHIKOTO	2000	88.89	70.31	112.03	RW2
Namibia	OSHIKOTO	2001	89.18	71.13	111.44	RW2
Namibia	OSHIKOTO	2002	88.65	70.79	110.67	RW2
Namibia	OSHIKOTO	2003	87.33	69.08	109.89	RW2
Namibia	OSHIKOTO	2004	85.35	66.63	108.76	RW2
Namibia	OSHIKOTO	2005	82.64	63.64	106.08	RW2
Namibia	OSHIKOTO	2006	80.12	61.81	103.31	RW2
Namibia	OSHIKOTO	2007	77.39	59.35	100.23	RW2
Namibia	OSHIKOTO	2008	74.86	56.43	98.61	RW2
Namibia	OSHIKOTO	2009	72.50	53.34	97.64	RW2
Namibia	OSHIKOTO	2010	70.40	50.65	96.64	RW2
Namibia	OSHIKOTO	2011	68.15	48.64	94.47	RW2
Namibia	OSHIKOTO	2012	66.19	46.66	93.05	RW2
Namibia	OSHIKOTO	2013	64.16	43.19	93.31	RW2
Namibia	OSHIKOTO	2014	62.16	38.60	98.37	RW2
Namibia	OSHIKOTO	2015	60.27	32.13	109.01	RW2
Namibia	OSHIKOTO	2016	58.43	26.50	123.81	RW2
Namibia	OSHIKOTO	2017	56.56	21.07	143.83	RW2
Namibia	OSHIKOTO	2018	54.80	16.28	167.53	RW2
Namibia	OSHIKOTO	2019	53.05	12.46	199.56	RW2
Namibia	OTJOZONDJUPA	1980	88.20	50.47	148.89	RW2
Namibia	OTJOZONDJUPA	1981	85.63	53.25	134.26	RW2
Namibia	OTJOZONDJUPA	1982	82.63	53.89	124.47	RW2
Namibia	OTJOZONDJUPA	1983	80.10	53.19	118.25	RW2
Namibia	OTJOZONDJUPA	1984	77.47	52.38	113.31	RW2
Namibia	OTJOZONDJUPA	1985	75.08	52.09	107.47	RW2
Namibia	OTJOZONDJUPA	1986	72.62	51.73	101.39	RW2
Namibia	OTJOZONDJUPA	1987	70.36	50.96	96.56	RW2
Namibia	OTJOZONDJUPA	1988	68.10	49.90	92.45	RW2
Namibia	OTJOZONDJUPA	1989	66.31	48.89	89.65	RW2
Namibia	OTJOZONDJUPA	1990	64.54	47.88	85.96	RW2
Namibia	OTJOZONDJUPA	1991	63.51	48.04	83.19	RW2
Namibia	OTJOZONDJUPA	1992	63.07	48.43	81.87	RW2
Namibia	OTJOZONDJUPA	1993	63.29	48.54	82.13	RW2
Namibia	OTJOZONDJUPA	1994	64.07	48.95	83.36	RW2
Namibia	OTJOZONDJUPA	1995	65.38	50.24	84.75	RW2
Namibia	OTJOZONDJUPA	1996	66.62	51.64	85.57	RW2
Namibia	OTJOZONDJUPA	1997	67.82	52.99	86.59	RW2
Namibia	OTJOZONDJUPA	1998	68.92	53.71	87.93	RW2
Namibia	OTJOZONDJUPA	1999	69.53	53.94	89.25	RW2
Namibia	OTJOZONDJUPA	2000	69.81	54.44	89.41	RW2
Namibia	OTJOZONDJUPA	2001	69.43	54.41	88.31	RW2
Namibia	OTJOZONDJUPA	2002	68.36	53.76	86.66	RW2
Namibia	OTJOZONDJUPA	2003	66.87	52.15	85.95	RW2
Namibia	OTJOZONDJUPA	2004	64.79	49.75	84.30	RW2
Namibia	OTJOZONDJUPA	2005	62.23	47.31	81.38	RW2
Namibia	OTJOZONDJUPA	2006	59.76	45.32	78.44	RW2
Namibia	OTJOZONDJUPA	2007	57.32	43.08	76.02	RW2
Namibia	OTJOZONDJUPA	2008	54.97	40.71	74.05	RW2
Namibia	OTJOZONDJUPA	2009	52.79	38.02	72.92	RW2
Namibia	OTJOZONDJUPA	2010	50.74	35.71	71.63	RW2
Namibia	OTJOZONDJUPA	2011	48.83	33.96	69.65	RW2
Namibia	OTJOZONDJUPA	2012	47.01	32.12	68.05	RW2
Namibia	OTJOZONDJUPA	2013	45.15	29.65	67.97	RW2
Namibia	OTJOZONDJUPA	2014	43.41	26.27	70.88	RW2
Namibia	OTJOZONDJUPA	2015	41.69	21.68	78.00	RW2
Namibia	OTJOZONDJUPA	2016	40.05	17.85	87.48	RW2
Namibia	OTJOZONDJUPA	2017	38.40	14.06	100.31	RW2
Namibia	OTJOZONDJUPA	2018	37.08	10.80	119.11	RW2
Namibia	OTJOZONDJUPA	2019	35.45	8.24	144.13	RW2
Niger	ALL	1980	316.20	307.31	324.93	IHME
Niger	ALL	1980	314.93	242.57	397.50	RW2
Niger	ALL	1980	319.60	298.00	342.80	UN

Continued on next page

Country	Region	Year	Median	Lower	Upper	Method
Niger	ALL	1981	314.66	305.98	323.35	IHME
Niger	ALL	1981	319.23	267.09	376.11	RW2
Niger	ALL	1981	319.10	298.00	341.80	UN
Niger	ALL	1982	313.57	305.25	321.79	IHME
Niger	ALL	1982	323.58	275.14	376.00	RW2
Niger	ALL	1982	321.00	300.30	343.40	UN
Niger	ALL	1983	312.14	303.94	320.16	IHME
Niger	ALL	1983	327.42	272.60	386.54	RW2
Niger	ALL	1983	324.50	304.20	346.70	UN
Niger	ALL	1984	312.09	304.16	320.09	IHME
Niger	ALL	1984	331.37	270.20	396.63	RW2
Niger	ALL	1984	328.90	308.60	351.00	UN
Niger	ALL	1985	311.07	303.08	318.84	IHME
Niger	ALL	1985	334.45	279.58	395.41	RW2
Niger	ALL	1985	332.90	312.60	355.20	UN
Niger	ALL	1986	309.49	301.57	317.13	IHME
Niger	ALL	1986	336.40	285.71	391.61	RW2
Niger	ALL	1986	335.80	315.20	358.00	UN
Niger	ALL	1987	307.24	299.77	314.34	IHME
Niger	ALL	1987	337.13	288.88	389.81	RW2
Niger	ALL	1987	336.90	316.30	358.70	UN
Niger	ALL	1988	303.90	296.92	311.24	IHME
Niger	ALL	1988	336.06	284.77	390.74	RW2
Niger	ALL	1988	335.70	315.50	357.10	UN
Niger	ALL	1989	300.35	293.09	307.96	IHME
Niger	ALL	1989	333.21	278.93	391.54	RW2
Niger	ALL	1989	332.90	312.90	354.00	UN
Niger	ALL	1990	296.08	288.57	303.94	IHME
Niger	ALL	1990	328.69	275.18	386.66	RW2
Niger	ALL	1990	328.20	309.00	349.00	UN
Niger	ALL	1991	291.11	283.84	298.92	IHME
Niger	ALL	1991	321.90	272.27	374.38	RW2
Niger	ALL	1991	321.90	303.00	342.30	UN
Niger	ALL	1992	285.98	278.99	294.29	IHME
Niger	ALL	1992	313.15	266.00	363.71	RW2
Niger	ALL	1992	313.70	294.90	333.50	UN
Niger	ALL	1993	280.04	272.94	287.98	IHME
Niger	ALL	1993	302.81	255.87	355.25	RW2
Niger	ALL	1993	303.80	285.50	323.00	UN
Niger	ALL	1994	273.08	265.69	281.04	IHME
Niger	ALL	1994	291.23	242.74	348.01	RW2
Niger	ALL	1994	292.20	274.60	310.80	UN
Niger	ALL	1995	265.81	258.47	273.61	IHME
Niger	ALL	1995	278.41	230.49	331.09	RW2
Niger	ALL	1995	279.50	262.50	297.20	UN
Niger	ALL	1996	257.80	250.28	265.32	IHME
Niger	ALL	1996	266.51	222.99	315.87	RW2
Niger	ALL	1996	267.10	250.20	284.20	UN
Niger	ALL	1997	249.88	242.38	257.49	IHME
Niger	ALL	1997	255.29	214.72	300.99	RW2
Niger	ALL	1997	255.90	239.40	272.80	UN
Niger	ALL	1998	241.19	233.76	248.71	IHME
Niger	ALL	1998	244.98	203.97	292.14	RW2
Niger	ALL	1998	245.80	229.70	262.70	UN
Niger	ALL	1999	232.41	224.78	240.15	IHME
Niger	ALL	1999	235.23	192.28	282.70	RW2
Niger	ALL	1999	236.40	220.60	253.60	UN
Niger	ALL	2000	223.68	216.23	231.35	IHME
Niger	ALL	2000	226.61	185.77	274.66	RW2
Niger	ALL	2000	227.30	211.90	244.80	UN
Niger	ALL	2001	214.43	206.95	221.79	IHME
Niger	ALL	2001	217.00	179.21	260.74	RW2
Niger	ALL	2001	217.60	202.50	235.20	UN

Continued on next page

Country	Region	Year	Median	Lower	Upper	Method
Niger	ALL	2002	204.70	197.55	211.79	IHME
Niger	ALL	2002	206.89	171.86	246.95	RW2
Niger	ALL	2002	207.00	192.30	223.90	UN
Niger	ALL	2003	194.96	187.91	202.03	IHME
Niger	ALL	2003	196.32	161.72	236.08	RW2
Niger	ALL	2003	195.70	181.60	211.70	UN
Niger	ALL	2004	185.18	177.97	192.46	IHME
Niger	ALL	2004	184.98	148.49	227.58	RW2
Niger	ALL	2004	184.10	170.60	199.20	UN
Niger	ALL	2005	175.88	168.56	183.36	IHME
Niger	ALL	2005	173.30	135.64	217.58	RW2
Niger	ALL	2005	172.70	159.60	187.40	UN
Niger	ALL	2006	166.58	158.94	173.96	IHME
Niger	ALL	2006	161.97	128.78	200.68	RW2
Niger	ALL	2006	161.80	148.60	176.10	UN
Niger	ALL	2007	158.17	150.05	165.88	IHME
Niger	ALL	2007	151.05	123.25	183.50	RW2
Niger	ALL	2007	151.30	138.00	165.80	UN
Niger	ALL	2008	149.98	141.85	158.26	IHME
Niger	ALL	2008	140.75	113.14	174.46	RW2
Niger	ALL	2008	141.30	127.60	156.80	UN
Niger	ALL	2009	143.16	134.56	151.69	IHME
Niger	ALL	2009	130.78	94.97	178.47	RW2
Niger	ALL	2009	132.10	117.30	148.80	UN
Niger	ALL	2010	137.04	128.09	146.66	IHME
Niger	ALL	2010	121.53	72.81	198.17	RW2
Niger	ALL	2010	123.60	107.30	142.40	UN
Niger	ALL	2011	131.73	122.32	142.41	IHME
Niger	ALL	2011	112.98	54.49	219.39	RW2
Niger	ALL	2011	116.10	97.80	137.20	UN
Niger	ALL	2012	126.84	116.69	138.36	IHME
Niger	ALL	2012	104.75	39.32	248.89	RW2
Niger	ALL	2012	109.60	89.90	133.60	UN
Niger	ALL	2013	121.99	111.28	134.74	IHME
Niger	ALL	2013	97.44	27.98	284.27	RW2
Niger	ALL	2013	104.10	82.50	131.10	UN
Niger	ALL	2014	117.20	105.88	130.55	IHME
Niger	ALL	2014	90.22	19.34	328.28	RW2
Niger	ALL	2014	99.60	76.20	129.50	UN
Niger	ALL	2015	112.77	101.27	127.06	IHME
Niger	ALL	2015	83.27	13.29	376.12	RW2
Niger	ALL	2015	95.50	70.50	128.30	UN
Niger	ALL	2016	77.64	9.01	439.12	RW2
Niger	ALL	2017	71.44	5.80	504.48	RW2
Niger	ALL	2018	66.06	3.82	578.44	RW2
Niger	ALL	2019	60.88	2.23	638.18	RW2
Niger	ALL	15-19	71.45	5.85	499.15	RW2
Niger	DOSSO	1980	264.42	205.10	335.35	RW2
Niger	DOSSO	1981	268.36	225.64	316.57	RW2
Niger	DOSSO	1982	272.35	233.14	314.69	RW2
Niger	DOSSO	1983	275.85	232.77	323.52	RW2
Niger	DOSSO	1984	279.15	232.86	329.67	RW2
Niger	DOSSO	1985	281.97	238.90	328.87	RW2
Niger	DOSSO	1986	283.01	244.70	325.56	RW2
Niger	DOSSO	1987	282.79	246.61	322.05	RW2
Niger	DOSSO	1988	280.86	242.79	322.08	RW2
Niger	DOSSO	1989	277.21	236.49	322.20	RW2
Niger	DOSSO	1990	272.23	231.79	316.18	RW2
Niger	DOSSO	1991	266.48	229.33	305.55	RW2
Niger	DOSSO	1992	259.71	224.98	297.04	RW2
Niger	DOSSO	1993	252.78	218.04	292.04	RW2
Niger	DOSSO	1994	246.09	209.05	288.48	RW2
Niger	DOSSO	1995	239.06	202.03	280.27	RW2

Continued on next page

Country	Region	Year	Median	Lower	Upper	Method
Niger	DOSSO	1996	233.52	199.97	270.10	RW2
Niger	DOSSO	1997	228.99	197.52	264.39	RW2
Niger	DOSSO	1998	225.59	193.11	261.64	RW2
Niger	DOSSO	1999	222.72	187.86	260.79	RW2
Niger	DOSSO	2000	220.95	187.14	260.29	RW2
Niger	DOSSO	2001	218.41	187.08	253.91	RW2
Niger	DOSSO	2002	215.20	185.81	248.11	RW2
Niger	DOSSO	2003	211.18	180.38	245.48	RW2
Niger	DOSSO	2004	206.39	172.39	244.33	RW2
Niger	DOSSO	2005	200.47	164.05	241.43	RW2
Niger	DOSSO	2006	194.51	161.75	232.11	RW2
Niger	DOSSO	2007	188.59	159.59	221.93	RW2
Niger	DOSSO	2008	182.62	151.15	219.65	RW2
Niger	DOSSO	2009	176.55	133.50	230.20	RW2
Niger	DOSSO	2010	171.00	109.79	256.97	RW2
Niger	DOSSO	2011	165.38	89.24	287.04	RW2
Niger	DOSSO	2012	159.88	70.06	324.39	RW2
Niger	DOSSO	2013	154.58	53.88	368.68	RW2
Niger	DOSSO	2014	148.91	39.60	418.61	RW2
Niger	DOSSO	2015	144.44	29.46	479.11	RW2
Niger	DOSSO	2016	139.14	20.96	540.48	RW2
Niger	DOSSO	2017	134.97	15.00	607.73	RW2
Niger	DOSSO	2018	129.59	10.54	669.92	RW2
Niger	DOSSO	2019	125.87	7.38	732.23	RW2
Niger	MARADI	1980	362.82	291.94	440.40	RW2
Niger	MARADI	1981	369.10	320.76	422.09	RW2
Niger	MARADI	1982	375.38	330.55	422.49	RW2
Niger	MARADI	1983	381.42	330.12	434.67	RW2
Niger	MARADI	1984	387.17	331.78	444.14	RW2
Niger	MARADI	1985	392.90	342.68	446.44	RW2
Niger	MARADI	1986	397.26	351.62	444.59	RW2
Niger	MARADI	1987	400.33	357.07	444.63	RW2
Niger	MARADI	1988	401.72	356.13	448.83	RW2
Niger	MARADI	1989	401.37	352.63	452.52	RW2
Niger	MARADI	1990	398.26	349.85	448.66	RW2
Niger	MARADI	1991	393.00	347.83	438.54	RW2
Niger	MARADI	1992	384.72	342.42	428.62	RW2
Niger	MARADI	1993	373.64	330.55	420.22	RW2
Niger	MARADI	1994	360.59	314.91	411.21	RW2
Niger	MARADI	1995	345.41	300.31	393.57	RW2
Niger	MARADI	1996	329.97	288.91	373.75	RW2
Niger	MARADI	1997	314.77	277.39	355.69	RW2
Niger	MARADI	1998	299.69	261.52	341.50	RW2
Niger	MARADI	1999	284.84	243.83	328.44	RW2
Niger	MARADI	2000	270.51	231.17	314.95	RW2
Niger	MARADI	2001	254.88	220.01	294.09	RW2
Niger	MARADI	2002	238.57	206.54	273.38	RW2
Niger	MARADI	2003	221.40	189.35	257.09	RW2
Niger	MARADI	2004	203.96	170.52	242.61	RW2
Niger	MARADI	2005	186.23	151.95	225.39	RW2
Niger	MARADI	2006	169.28	139.48	203.30	RW2
Niger	MARADI	2007	153.19	127.99	182.06	RW2
Niger	MARADI	2008	138.21	112.31	168.59	RW2
Niger	MARADI	2009	124.55	91.90	166.13	RW2
Niger	MARADI	2010	112.06	69.72	174.70	RW2
Niger	MARADI	2011	100.56	51.68	185.54	RW2
Niger	MARADI	2012	90.37	37.17	201.06	RW2
Niger	MARADI	2013	80.97	26.79	220.93	RW2
Niger	MARADI	2014	72.31	18.30	244.33	RW2
Niger	MARADI	2015	64.64	12.25	277.38	RW2
Niger	MARADI	2016	57.61	7.94	310.83	RW2
Niger	MARADI	2017	51.57	5.25	352.91	RW2
Niger	MARADI	2018	45.67	3.34	397.43	RW2

Continued on next page

Country	Region	Year	Median	Lower	Upper	Method
Niger	MARADI	2019	40.98	2.09	459.61	RW2
Niger	NIAMEY	1980	143.08	104.71	192.94	RW2
Niger	NIAMEY	1981	147.02	117.73	182.54	RW2
Niger	NIAMEY	1982	150.77	124.07	181.87	RW2
Niger	NIAMEY	1983	154.49	125.59	188.26	RW2
Niger	NIAMEY	1984	157.95	126.57	194.30	RW2
Niger	NIAMEY	1985	161.33	132.48	195.48	RW2
Niger	NIAMEY	1986	163.67	136.98	194.09	RW2
Niger	NIAMEY	1987	165.04	139.38	194.20	RW2
Niger	NIAMEY	1988	165.36	138.31	196.19	RW2
Niger	NIAMEY	1989	164.57	135.99	197.49	RW2
Niger	NIAMEY	1990	162.68	134.28	195.12	RW2
Niger	NIAMEY	1991	160.11	133.97	189.80	RW2
Niger	NIAMEY	1992	156.73	131.77	185.03	RW2
Niger	NIAMEY	1993	152.81	127.80	181.95	RW2
Niger	NIAMEY	1994	148.90	122.71	179.72	RW2
Niger	NIAMEY	1995	144.46	118.67	174.45	RW2
Niger	NIAMEY	1996	140.78	117.02	168.38	RW2
Niger	NIAMEY	1997	137.40	115.03	163.88	RW2
Niger	NIAMEY	1998	134.43	111.89	161.12	RW2
Niger	NIAMEY	1999	131.71	107.25	159.72	RW2
Niger	NIAMEY	2000	129.37	105.45	158.06	RW2
Niger	NIAMEY	2001	126.36	103.95	152.75	RW2
Niger	NIAMEY	2002	122.80	101.48	147.85	RW2
Niger	NIAMEY	2003	118.61	96.70	144.91	RW2
Niger	NIAMEY	2004	113.98	90.30	142.76	RW2
Niger	NIAMEY	2005	108.69	83.82	139.44	RW2
Niger	NIAMEY	2006	103.63	79.72	133.40	RW2
Niger	NIAMEY	2007	98.51	74.86	127.78	RW2
Niger	NIAMEY	2008	93.67	68.20	126.57	RW2
Niger	NIAMEY	2009	88.82	58.87	131.11	RW2
Niger	NIAMEY	2010	84.49	47.78	144.92	RW2
Niger	NIAMEY	2011	80.11	37.63	162.03	RW2
Niger	NIAMEY	2012	76.08	28.92	184.29	RW2
Niger	NIAMEY	2013	72.37	21.70	215.95	RW2
Niger	NIAMEY	2014	68.85	15.82	249.66	RW2
Niger	NIAMEY	2015	65.39	11.37	292.60	RW2
Niger	NIAMEY	2016	61.83	8.00	342.33	RW2
Niger	NIAMEY	2017	58.94	5.59	403.39	RW2
Niger	NIAMEY	2018	55.58	3.77	471.92	RW2
Niger	NIAMEY	2019	52.85	2.53	549.07	RW2
Niger	TASHOUA/AGADEZ	1980	322.20	256.37	397.36	RW2
Niger	TASHOUA/AGADEZ	1981	325.61	279.09	376.98	RW2
Niger	TASHOUA/AGADEZ	1982	329.06	286.22	374.80	RW2
Niger	TASHOUA/AGADEZ	1983	331.99	283.92	383.03	RW2
Niger	TASHOUA/AGADEZ	1984	334.55	282.65	390.35	RW2
Niger	TASHOUA/AGADEZ	1985	336.80	289.53	387.95	RW2
Niger	TASHOUA/AGADEZ	1986	337.23	295.32	382.39	RW2
Niger	TASHOUA/AGADEZ	1987	336.08	295.87	378.58	RW2
Niger	TASHOUA/AGADEZ	1988	333.15	291.23	377.54	RW2
Niger	TASHOUA/AGADEZ	1989	328.21	283.16	376.11	RW2
Niger	TASHOUA/AGADEZ	1990	321.28	276.60	367.70	RW2
Niger	TASHOUA/AGADEZ	1991	312.58	271.88	355.45	RW2
Niger	TASHOUA/AGADEZ	1992	302.11	264.81	343.05	RW2
Niger	TASHOUA/AGADEZ	1993	290.61	253.19	332.79	RW2
Niger	TASHOUA/AGADEZ	1994	278.11	237.98	322.29	RW2
Niger	TASHOUA/AGADEZ	1995	265.22	226.28	307.48	RW2
Niger	TASHOUA/AGADEZ	1996	253.30	218.64	291.92	RW2
Niger	TASHOUA/AGADEZ	1997	242.02	210.19	277.35	RW2
Niger	TASHOUA/AGADEZ	1998	231.92	199.11	267.83	RW2
Niger	TASHOUA/AGADEZ	1999	222.24	187.38	259.84	RW2
Niger	TASHOUA/AGADEZ	2000	213.62	180.41	251.77	RW2
Niger	TASHOUA/AGADEZ	2001	204.14	173.90	238.81	RW2

Continued on next page

Country	Region	Year	Median	Lower	Upper	Method
Niger	TASHOUA/AGADEZ	2002	194.43	166.30	226.01	RW2
Niger	TASHOUA/AGADEZ	2003	183.84	154.98	215.99	RW2
Niger	TASHOUA/AGADEZ	2004	173.02	142.61	208.22	RW2
Niger	TASHOUA/AGADEZ	2005	161.58	130.43	197.96	RW2
Niger	TASHOUA/AGADEZ	2006	150.52	122.71	182.88	RW2
Niger	TASHOUA/AGADEZ	2007	139.83	115.72	167.56	RW2
Niger	TASHOUA/AGADEZ	2008	129.85	105.12	159.26	RW2
Niger	TASHOUA/AGADEZ	2009	120.32	88.61	161.48	RW2
Niger	TASHOUA/AGADEZ	2010	111.58	69.97	174.55	RW2
Niger	TASHOUA/AGADEZ	2011	102.95	53.34	189.66	RW2
Niger	TASHOUA/AGADEZ	2012	95.19	39.96	209.22	RW2
Niger	TASHOUA/AGADEZ	2013	88.02	29.16	235.80	RW2
Niger	TASHOUA/AGADEZ	2014	81.45	20.74	267.24	RW2
Niger	TASHOUA/AGADEZ	2015	75.06	14.66	307.46	RW2
Niger	TASHOUA/AGADEZ	2016	69.33	9.84	347.94	RW2
Niger	TASHOUA/AGADEZ	2017	63.81	6.68	400.05	RW2
Niger	TASHOUA/AGADEZ	2018	58.82	4.53	461.57	RW2
Niger	TASHOUA/AGADEZ	2019	54.32	2.84	519.38	RW2
Niger	TILLABERI	1980	264.79	203.71	336.06	RW2
Niger	TILLABERI	1981	270.77	226.34	320.21	RW2
Niger	TILLABERI	1982	276.52	236.31	320.81	RW2
Niger	TILLABERI	1983	282.56	237.86	330.89	RW2
Niger	TILLABERI	1984	287.48	239.09	339.25	RW2
Niger	TILLABERI	1985	292.05	247.96	340.27	RW2
Niger	TILLABERI	1986	294.23	254.55	337.08	RW2
Niger	TILLABERI	1987	294.40	256.72	335.36	RW2
Niger	TILLABERI	1988	292.21	253.25	334.02	RW2
Niger	TILLABERI	1989	287.86	246.49	333.73	RW2
Niger	TILLABERI	1990	281.45	240.60	325.97	RW2
Niger	TILLABERI	1991	273.42	236.34	313.72	RW2
Niger	TILLABERI	1992	264.34	229.55	301.94	RW2
Niger	TILLABERI	1993	254.81	219.61	293.86	RW2
Niger	TILLABERI	1994	245.05	207.98	287.16	RW2
Niger	TILLABERI	1995	235.38	198.63	275.61	RW2
Niger	TILLABERI	1996	226.81	193.50	263.71	RW2
Niger	TILLABERI	1997	219.22	187.94	253.83	RW2
Niger	TILLABERI	1998	212.58	181.18	247.46	RW2
Niger	TILLABERI	1999	206.61	172.97	243.47	RW2
Niger	TILLABERI	2000	201.90	169.84	238.98	RW2
Niger	TILLABERI	2001	196.30	167.05	229.79	RW2
Niger	TILLABERI	2002	190.27	163.05	221.17	RW2
Niger	TILLABERI	2003	183.55	155.33	215.30	RW2
Niger	TILLABERI	2004	176.21	146.15	211.29	RW2
Niger	TILLABERI	2005	168.21	136.24	204.72	RW2
Niger	TILLABERI	2006	160.20	131.13	194.03	RW2
Niger	TILLABERI	2007	152.59	126.49	182.64	RW2
Niger	TILLABERI	2008	144.95	117.31	178.91	RW2
Niger	TILLABERI	2009	137.53	101.33	184.87	RW2
Niger	TILLABERI	2010	130.50	81.45	203.47	RW2
Niger	TILLABERI	2011	124.13	64.59	224.42	RW2
Niger	TILLABERI	2012	117.75	49.50	253.13	RW2
Niger	TILLABERI	2013	110.96	36.94	284.78	RW2
Niger	TILLABERI	2014	105.61	27.39	331.17	RW2
Niger	TILLABERI	2015	99.89	19.72	374.35	RW2
Niger	TILLABERI	2016	94.75	13.76	438.73	RW2
Niger	TILLABERI	2017	90.22	9.62	498.08	RW2
Niger	TILLABERI	2018	85.46	6.58	561.23	RW2
Niger	TILLABERI	2019	80.15	4.31	635.71	RW2
Niger	ZINDA/DIFFA	1980	362.09	287.49	444.52	RW2
Niger	ZINDA/DIFFA	1981	365.77	313.19	422.10	RW2
Niger	ZINDA/DIFFA	1982	369.18	321.53	419.27	RW2
Niger	ZINDA/DIFFA	1983	372.26	319.83	427.23	RW2
Niger	ZINDA/DIFFA	1984	374.95	319.55	432.44	RW2

Continued on next page

Country	Region	Year	Median	Lower	Upper	Method
Niger	ZINDA/DIFFA	1985	377.40	326.48	430.86	RW2
Niger	ZINDA/DIFFA	1986	378.37	332.72	425.63	RW2
Niger	ZINDA/DIFFA	1987	377.89	335.34	421.80	RW2
Niger	ZINDA/DIFFA	1988	375.65	330.51	422.19	RW2
Niger	ZINDA/DIFFA	1989	371.46	323.18	421.92	RW2
Niger	ZINDA/DIFFA	1990	365.06	317.57	414.82	RW2
Niger	ZINDA/DIFFA	1991	356.75	313.46	402.48	RW2
Niger	ZINDA/DIFFA	1992	346.29	305.27	389.68	RW2
Niger	ZINDA/DIFFA	1993	333.95	292.88	378.44	RW2
Niger	ZINDA/DIFFA	1994	320.37	276.91	368.61	RW2
Niger	ZINDA/DIFFA	1995	305.68	262.66	351.66	RW2
Niger	ZINDA/DIFFA	1996	291.88	252.90	333.77	RW2
Niger	ZINDA/DIFFA	1997	278.75	242.77	317.48	RW2
Niger	ZINDA/DIFFA	1998	266.42	230.21	306.45	RW2
Niger	ZINDA/DIFFA	1999	254.78	216.22	297.13	RW2
Niger	ZINDA/DIFFA	2000	244.10	206.50	286.07	RW2
Niger	ZINDA/DIFFA	2001	232.46	199.16	269.83	RW2
Niger	ZINDA/DIFFA	2002	220.27	189.42	254.58	RW2
Niger	ZINDA/DIFFA	2003	207.17	176.15	242.19	RW2
Niger	ZINDA/DIFFA	2004	193.66	160.91	231.21	RW2
Niger	ZINDA/DIFFA	2005	179.60	145.39	219.05	RW2
Niger	ZINDA/DIFFA	2006	166.04	135.64	201.30	RW2
Niger	ZINDA/DIFFA	2007	152.94	126.56	183.51	RW2
Niger	ZINDA/DIFFA	2008	140.63	113.55	173.19	RW2
Niger	ZINDA/DIFFA	2009	128.97	95.02	173.16	RW2
Niger	ZINDA/DIFFA	2010	118.52	73.26	185.61	RW2
Niger	ZINDA/DIFFA	2011	108.25	55.94	199.49	RW2
Niger	ZINDA/DIFFA	2012	99.25	41.23	218.51	RW2
Niger	ZINDA/DIFFA	2013	90.52	29.40	244.10	RW2
Niger	ZINDA/DIFFA	2014	82.70	20.60	270.61	RW2
Niger	ZINDA/DIFFA	2015	75.57	14.38	313.31	RW2
Niger	ZINDA/DIFFA	2016	68.85	9.84	355.84	RW2
Niger	ZINDA/DIFFA	2017	62.88	6.49	409.91	RW2
Niger	ZINDA/DIFFA	2018	56.91	4.08	463.28	RW2
Niger	ZINDA/DIFFA	2019	51.70	2.71	519.85	RW2
Nigeria	ALL	1980	223.97	216.34	232.08	IHME
Nigeria	ALL	1980	210.04	163.07	265.74	RW2
Nigeria	ALL	1980	214.40	197.70	232.30	UN
Nigeria	ALL	1981	220.19	212.93	227.88	IHME
Nigeria	ALL	1981	210.31	176.19	248.53	RW2
Nigeria	ALL	1981	211.50	195.40	228.50	UN
Nigeria	ALL	1982	216.92	210.18	224.73	IHME
Nigeria	ALL	1982	210.62	179.41	245.61	RW2
Nigeria	ALL	1982	209.60	194.20	226.10	UN
Nigeria	ALL	1983	214.92	208.45	222.21	IHME
Nigeria	ALL	1983	210.69	176.26	249.84	RW2
Nigeria	ALL	1983	208.90	193.90	224.80	UN
Nigeria	ALL	1984	213.61	206.97	220.44	IHME
Nigeria	ALL	1984	211.12	173.24	254.27	RW2
Nigeria	ALL	1984	208.80	194.40	224.30	UN
Nigeria	ALL	1985	211.91	205.57	218.77	IHME
Nigeria	ALL	1985	211.08	176.51	250.44	RW2
Nigeria	ALL	1985	209.50	195.50	224.70	UN
Nigeria	ALL	1986	211.24	204.85	217.91	IHME
Nigeria	ALL	1986	211.33	179.34	247.22	RW2
Nigeria	ALL	1986	210.60	196.70	225.40	UN
Nigeria	ALL	1987	211.06	204.72	217.38	IHME
Nigeria	ALL	1987	211.66	181.26	246.16	RW2
Nigeria	ALL	1987	211.50	197.90	226.10	UN
Nigeria	ALL	1988	210.59	204.01	216.99	IHME
Nigeria	ALL	1988	211.84	179.81	247.77	RW2
Nigeria	ALL	1988	212.20	198.70	226.50	UN
Nigeria	ALL	1989	209.54	203.15	215.96	IHME

Continued on next page

Country	Region	Year	Median	Lower	Upper	Method
Nigeria	ALL	1989	211.98	178.07	250.39	RW2
Nigeria	ALL	1989	212.60	199.10	226.60	UN
Nigeria	ALL	1990	207.88	201.67	214.16	IHME
Nigeria	ALL	1990	212.10	178.50	250.64	RW2
Nigeria	ALL	1990	212.50	199.20	226.30	UN
Nigeria	ALL	1991	206.28	200.16	212.66	IHME
Nigeria	ALL	1991	211.92	180.21	247.23	RW2
Nigeria	ALL	1991	212.20	199.00	225.90	UN
Nigeria	ALL	1992	204.52	198.59	210.83	IHME
Nigeria	ALL	1992	211.44	180.53	245.62	RW2
Nigeria	ALL	1992	211.70	198.50	225.20	UN
Nigeria	ALL	1993	202.48	196.10	208.97	IHME
Nigeria	ALL	1993	210.60	178.95	245.91	RW2
Nigeria	ALL	1993	211.00	197.90	224.30	UN
Nigeria	ALL	1994	200.35	193.94	206.59	IHME
Nigeria	ALL	1994	209.16	175.72	247.33	RW2
Nigeria	ALL	1994	209.70	196.90	223.00	UN
Nigeria	ALL	1995	198.20	192.16	204.09	IHME
Nigeria	ALL	1995	207.40	174.68	244.51	RW2
Nigeria	ALL	1995	207.80	195.00	220.80	UN
Nigeria	ALL	1996	195.53	189.49	201.47	IHME
Nigeria	ALL	1996	204.66	174.08	239.63	RW2
Nigeria	ALL	1996	205.10	192.40	218.00	UN
Nigeria	ALL	1997	192.56	186.47	198.49	IHME
Nigeria	ALL	1997	201.21	172.04	234.00	RW2
Nigeria	ALL	1997	201.40	189.00	214.20	UN
Nigeria	ALL	1998	189.51	183.47	195.32	IHME
Nigeria	ALL	1998	197.11	167.47	231.41	RW2
Nigeria	ALL	1998	197.00	184.90	209.50	UN
Nigeria	ALL	1999	186.40	180.16	192.25	IHME
Nigeria	ALL	1999	192.31	161.38	227.32	RW2
Nigeria	ALL	1999	192.00	180.40	204.20	UN
Nigeria	ALL	2000	183.06	177.12	189.01	IHME
Nigeria	ALL	2000	186.80	156.65	220.71	RW2
Nigeria	ALL	2000	186.80	175.40	198.60	UN
Nigeria	ALL	2001	179.61	174.04	185.50	IHME
Nigeria	ALL	2001	181.19	153.23	212.67	RW2
Nigeria	ALL	2001	181.30	170.00	192.90	UN
Nigeria	ALL	2002	176.05	170.57	182.25	IHME
Nigeria	ALL	2002	175.37	149.25	205.07	RW2
Nigeria	ALL	2002	175.60	164.80	187.10	UN
Nigeria	ALL	2003	171.91	166.25	178.06	IHME
Nigeria	ALL	2003	169.57	143.69	199.25	RW2
Nigeria	ALL	2003	169.90	159.10	181.10	UN
Nigeria	ALL	2004	166.90	161.18	173.15	IHME
Nigeria	ALL	2004	163.58	136.40	194.77	RW2
Nigeria	ALL	2004	164.10	153.40	175.10	UN
Nigeria	ALL	2005	161.50	155.75	167.84	IHME
Nigeria	ALL	2005	157.91	131.30	188.88	RW2
Nigeria	ALL	2005	158.10	147.80	169.10	UN
Nigeria	ALL	2006	155.99	150.04	161.88	IHME
Nigeria	ALL	2006	152.14	127.85	179.98	RW2
Nigeria	ALL	2006	152.20	141.90	163.20	UN
Nigeria	ALL	2007	149.98	144.00	156.00	IHME
Nigeria	ALL	2007	146.55	124.05	172.24	RW2
Nigeria	ALL	2007	146.40	135.90	157.70	UN
Nigeria	ALL	2008	144.30	137.62	150.82	IHME
Nigeria	ALL	2008	141.18	118.59	167.37	RW2
Nigeria	ALL	2008	140.90	129.50	153.10	UN
Nigeria	ALL	2009	138.52	131.57	145.69	IHME
Nigeria	ALL	2009	135.74	111.96	164.02	RW2
Nigeria	ALL	2009	135.50	122.70	149.50	UN
Nigeria	ALL	2010	132.58	125.30	140.40	IHME

Continued on next page

Country	Region	Year	Median	Lower	Upper	Method
Nigeria	ALL	2010	130.52	105.91	160.55	RW2
Nigeria	ALL	2010	130.30	115.80	146.40	UN
Nigeria	ALL	2011	126.12	117.90	135.07	IHME
Nigeria	ALL	2011	125.54	102.87	152.36	RW2
Nigeria	ALL	2011	125.50	109.00	143.60	UN
Nigeria	ALL	2012	120.64	111.35	130.46	IHME
Nigeria	ALL	2012	120.64	100.42	144.15	RW2
Nigeria	ALL	2012	120.90	102.50	141.90	UN
Nigeria	ALL	2013	115.12	105.37	126.09	IHME
Nigeria	ALL	2013	115.98	95.02	140.62	RW2
Nigeria	ALL	2013	116.60	95.70	140.60	UN
Nigeria	ALL	2014	110.23	99.44	122.15	IHME
Nigeria	ALL	2014	111.38	83.75	146.49	RW2
Nigeria	ALL	2014	112.50	89.20	140.00	UN
Nigeria	ALL	2015	103.84	92.56	116.28	IHME
Nigeria	ALL	2015	106.84	68.28	163.44	RW2
Nigeria	ALL	2015	108.80	83.40	139.70	UN
Nigeria	ALL	2016	102.78	55.61	183.21	RW2
Nigeria	ALL	2017	98.46	43.71	208.39	RW2
Nigeria	ALL	2018	94.46	33.98	241.16	RW2
Nigeria	ALL	2019	90.50	24.96	276.50	RW2
Nigeria	ALL	15-19	98.46	44.34	205.34	RW2
Nigeria	NORTH CENTRAL	1980	168.35	130.06	215.61	RW2
Nigeria	NORTH CENTRAL	1981	167.43	138.64	201.39	RW2
Nigeria	NORTH CENTRAL	1982	166.29	140.41	195.90	RW2
Nigeria	NORTH CENTRAL	1983	165.15	138.05	196.31	RW2
Nigeria	NORTH CENTRAL	1984	163.89	135.44	196.43	RW2
Nigeria	NORTH CENTRAL	1985	162.69	137.10	192.03	RW2
Nigeria	NORTH CENTRAL	1986	161.13	138.06	186.78	RW2
Nigeria	NORTH CENTRAL	1987	159.30	137.75	183.49	RW2
Nigeria	NORTH CENTRAL	1988	157.29	135.17	182.40	RW2
Nigeria	NORTH CENTRAL	1989	155.15	132.12	181.63	RW2
Nigeria	NORTH CENTRAL	1990	152.76	130.02	178.49	RW2
Nigeria	NORTH CENTRAL	1991	150.90	130.05	174.45	RW2
Nigeria	NORTH CENTRAL	1992	149.21	129.11	171.68	RW2
Nigeria	NORTH CENTRAL	1993	147.72	127.18	170.75	RW2
Nigeria	NORTH CENTRAL	1994	146.59	124.60	170.95	RW2
Nigeria	NORTH CENTRAL	1995	145.62	124.23	170.13	RW2
Nigeria	NORTH CENTRAL	1996	144.24	124.36	166.55	RW2
Nigeria	NORTH CENTRAL	1997	142.41	123.57	163.72	RW2
Nigeria	NORTH CENTRAL	1998	140.15	121.20	161.73	RW2
Nigeria	NORTH CENTRAL	1999	137.46	116.91	160.32	RW2
Nigeria	NORTH CENTRAL	2000	134.17	114.02	156.60	RW2
Nigeria	NORTH CENTRAL	2001	130.96	112.54	151.40	RW2
Nigeria	NORTH CENTRAL	2002	127.61	110.48	146.94	RW2
Nigeria	NORTH CENTRAL	2003	124.19	106.92	144.09	RW2
Nigeria	NORTH CENTRAL	2004	120.79	102.50	141.75	RW2
Nigeria	NORTH CENTRAL	2005	117.37	99.49	138.26	RW2
Nigeria	NORTH CENTRAL	2006	113.77	97.59	132.51	RW2
Nigeria	NORTH CENTRAL	2007	109.93	94.51	127.26	RW2
Nigeria	NORTH CENTRAL	2008	105.98	90.20	124.03	RW2
Nigeria	NORTH CENTRAL	2009	101.79	85.02	121.42	RW2
Nigeria	NORTH CENTRAL	2010	97.59	80.14	118.48	RW2
Nigeria	NORTH CENTRAL	2011	93.41	77.30	112.58	RW2
Nigeria	NORTH CENTRAL	2012	89.36	74.79	106.54	RW2
Nigeria	NORTH CENTRAL	2013	85.44	70.01	104.29	RW2
Nigeria	NORTH CENTRAL	2014	81.68	61.76	107.27	RW2
Nigeria	NORTH CENTRAL	2015	78.08	51.05	117.51	RW2
Nigeria	NORTH CENTRAL	2016	74.50	41.68	129.32	RW2
Nigeria	NORTH CENTRAL	2017	71.25	33.32	145.55	RW2
Nigeria	NORTH CENTRAL	2018	67.89	25.94	166.27	RW2
Nigeria	NORTH CENTRAL	2019	64.86	19.87	192.93	RW2
Nigeria	NORTH EAST	1980	249.17	198.63	308.20	RW2

Continued on next page

Country	Region	Year	Median	Lower	Upper	Method
Nigeria	NORTH EAST	1981	252.11	215.40	292.78	RW2
Nigeria	NORTH EAST	1982	255.09	221.59	290.94	RW2
Nigeria	NORTH EAST	1983	257.76	221.47	297.80	RW2
Nigeria	NORTH EAST	1984	260.45	221.62	302.96	RW2
Nigeria	NORTH EAST	1985	262.91	226.30	302.00	RW2
Nigeria	NORTH EAST	1986	264.55	231.60	300.51	RW2
Nigeria	NORTH EAST	1987	265.74	234.50	299.44	RW2
Nigeria	NORTH EAST	1988	266.19	233.63	301.67	RW2
Nigeria	NORTH EAST	1989	265.99	231.26	304.71	RW2
Nigeria	NORTH EAST	1990	265.09	230.33	302.98	RW2
Nigeria	NORTH EAST	1991	264.58	232.26	298.84	RW2
Nigeria	NORTH EAST	1992	263.75	232.97	296.79	RW2
Nigeria	NORTH EAST	1993	263.01	231.64	297.49	RW2
Nigeria	NORTH EAST	1994	262.17	228.15	299.14	RW2
Nigeria	NORTH EAST	1995	261.01	227.63	298.24	RW2
Nigeria	NORTH EAST	1996	258.56	227.92	291.53	RW2
Nigeria	NORTH EAST	1997	254.92	225.83	286.90	RW2
Nigeria	NORTH EAST	1998	250.05	220.20	282.78	RW2
Nigeria	NORTH EAST	1999	243.69	212.10	278.28	RW2
Nigeria	NORTH EAST	2000	236.02	205.19	270.03	RW2
Nigeria	NORTH EAST	2001	227.93	199.85	258.72	RW2
Nigeria	NORTH EAST	2002	219.24	193.26	248.03	RW2
Nigeria	NORTH EAST	2003	210.25	183.84	239.43	RW2
Nigeria	NORTH EAST	2004	201.18	173.31	231.57	RW2
Nigeria	NORTH EAST	2005	192.18	165.01	222.58	RW2
Nigeria	NORTH EAST	2006	183.07	159.03	210.29	RW2
Nigeria	NORTH EAST	2007	174.24	152.04	199.26	RW2
Nigeria	NORTH EAST	2008	165.57	142.79	191.20	RW2
Nigeria	NORTH EAST	2009	156.99	132.70	184.65	RW2
Nigeria	NORTH EAST	2010	148.75	123.12	178.35	RW2
Nigeria	NORTH EAST	2011	140.86	117.35	168.17	RW2
Nigeria	NORTH EAST	2012	133.28	111.64	158.30	RW2
Nigeria	NORTH EAST	2013	126.04	102.97	153.35	RW2
Nigeria	NORTH EAST	2014	119.02	89.58	155.95	RW2
Nigeria	NORTH EAST	2015	112.57	73.52	168.35	RW2
Nigeria	NORTH EAST	2016	106.20	59.20	182.35	RW2
Nigeria	NORTH EAST	2017	100.40	46.76	201.65	RW2
Nigeria	NORTH EAST	2018	94.50	36.14	224.72	RW2
Nigeria	NORTH EAST	2019	89.37	27.53	253.70	RW2
Nigeria	NORTH WEST	1980	278.41	223.97	339.63	RW2
Nigeria	NORTH WEST	1981	282.26	243.85	325.04	RW2
Nigeria	NORTH WEST	1982	286.10	250.78	324.13	RW2
Nigeria	NORTH WEST	1983	289.74	250.60	331.90	RW2
Nigeria	NORTH WEST	1984	293.16	251.42	338.15	RW2
Nigeria	NORTH WEST	1985	296.37	258.05	338.13	RW2
Nigeria	NORTH WEST	1986	298.70	263.57	336.14	RW2
Nigeria	NORTH WEST	1987	300.09	266.73	335.66	RW2
Nigeria	NORTH WEST	1988	300.47	265.71	338.28	RW2
Nigeria	NORTH WEST	1989	300.09	263.18	340.98	RW2
Nigeria	NORTH WEST	1990	298.17	261.36	338.31	RW2
Nigeria	NORTH WEST	1991	296.34	261.61	332.94	RW2
Nigeria	NORTH WEST	1992	293.80	260.78	329.00	RW2
Nigeria	NORTH WEST	1993	290.62	256.74	327.21	RW2
Nigeria	NORTH WEST	1994	286.98	250.94	326.03	RW2
Nigeria	NORTH WEST	1995	283.08	248.32	321.36	RW2
Nigeria	NORTH WEST	1996	277.52	245.18	312.30	RW2
Nigeria	NORTH WEST	1997	271.14	241.08	303.88	RW2
Nigeria	NORTH WEST	1998	263.65	232.94	297.42	RW2
Nigeria	NORTH WEST	1999	255.38	222.56	290.90	RW2
Nigeria	NORTH WEST	2000	246.16	213.86	281.06	RW2
Nigeria	NORTH WEST	2001	237.07	208.19	268.67	RW2
Nigeria	NORTH WEST	2002	228.10	201.13	257.22	RW2
Nigeria	NORTH WEST	2003	219.25	192.04	249.31	RW2

Continued on next page

Country	Region	Year	Median	Lower	Upper	Method
Nigeria	NORTH WEST	2004	210.83	182.42	242.78	RW2
Nigeria	NORTH WEST	2005	202.85	174.97	234.24	RW2
Nigeria	NORTH WEST	2006	194.76	169.73	222.60	RW2
Nigeria	NORTH WEST	2007	186.76	163.65	212.35	RW2
Nigeria	NORTH WEST	2008	178.85	155.04	205.11	RW2
Nigeria	NORTH WEST	2009	171.12	145.76	199.76	RW2
Nigeria	NORTH WEST	2010	163.37	136.72	193.79	RW2
Nigeria	NORTH WEST	2011	155.90	131.56	183.66	RW2
Nigeria	NORTH WEST	2012	148.73	126.94	173.39	RW2
Nigeria	NORTH WEST	2013	141.74	119.04	168.62	RW2
Nigeria	NORTH WEST	2014	134.97	104.62	172.90	RW2
Nigeria	NORTH WEST	2015	128.55	86.19	188.41	RW2
Nigeria	NORTH WEST	2016	122.29	69.87	205.05	RW2
Nigeria	NORTH WEST	2017	116.47	55.77	227.39	RW2
Nigeria	NORTH WEST	2018	110.50	43.28	254.08	RW2
Nigeria	NORTH WEST	2019	105.34	32.93	289.94	RW2
Nigeria	SOUTH EAST	1980	131.93	99.38	173.99	RW2
Nigeria	SOUTH EAST	1981	132.89	107.81	163.48	RW2
Nigeria	SOUTH EAST	1982	133.92	111.14	160.58	RW2
Nigeria	SOUTH EAST	1983	134.74	111.03	162.46	RW2
Nigeria	SOUTH EAST	1984	135.57	110.93	164.85	RW2
Nigeria	SOUTH EAST	1985	136.44	113.59	162.90	RW2
Nigeria	SOUTH EAST	1986	136.99	116.34	160.69	RW2
Nigeria	SOUTH EAST	1987	137.37	117.52	159.86	RW2
Nigeria	SOUTH EAST	1988	137.64	117.32	160.89	RW2
Nigeria	SOUTH EAST	1989	137.83	116.30	162.66	RW2
Nigeria	SOUTH EAST	1990	137.87	116.33	161.93	RW2
Nigeria	SOUTH EAST	1991	138.36	118.14	161.12	RW2
Nigeria	SOUTH EAST	1992	139.11	119.82	161.32	RW2
Nigeria	SOUTH EAST	1993	140.32	120.31	163.38	RW2
Nigeria	SOUTH EAST	1994	141.65	119.58	166.11	RW2
Nigeria	SOUTH EAST	1995	143.59	121.80	168.55	RW2
Nigeria	SOUTH EAST	1996	145.10	124.52	168.74	RW2
Nigeria	SOUTH EAST	1997	146.21	126.35	168.66	RW2
Nigeria	SOUTH EAST	1998	147.08	126.24	170.49	RW2
Nigeria	SOUTH EAST	1999	147.27	125.03	172.24	RW2
Nigeria	SOUTH EAST	2000	146.83	124.89	171.51	RW2
Nigeria	SOUTH EAST	2001	146.15	125.72	169.38	RW2
Nigeria	SOUTH EAST	2002	145.24	125.72	167.33	RW2
Nigeria	SOUTH EAST	2003	143.78	123.44	166.55	RW2
Nigeria	SOUTH EAST	2004	142.17	120.56	166.83	RW2
Nigeria	SOUTH EAST	2005	140.27	118.81	165.20	RW2
Nigeria	SOUTH EAST	2006	137.81	117.81	160.69	RW2
Nigeria	SOUTH EAST	2007	135.01	115.84	156.43	RW2
Nigeria	SOUTH EAST	2008	132.03	112.03	154.60	RW2
Nigeria	SOUTH EAST	2009	128.64	106.70	154.21	RW2
Nigeria	SOUTH EAST	2010	125.06	101.78	152.92	RW2
Nigeria	SOUTH EAST	2011	121.31	98.46	148.45	RW2
Nigeria	SOUTH EAST	2012	117.70	95.40	144.08	RW2
Nigeria	SOUTH EAST	2013	114.16	89.73	143.83	RW2
Nigeria	SOUTH EAST	2014	110.71	80.42	149.81	RW2
Nigeria	SOUTH EAST	2015	107.26	68.06	165.15	RW2
Nigeria	SOUTH EAST	2016	103.98	56.30	182.32	RW2
Nigeria	SOUTH EAST	2017	100.67	45.70	205.80	RW2
Nigeria	SOUTH EAST	2018	97.52	36.52	235.95	RW2
Nigeria	SOUTH EAST	2019	94.57	27.97	270.16	RW2
Nigeria	SOUTH SOUTH	1980	143.82	109.65	186.99	RW2
Nigeria	SOUTH SOUTH	1981	142.78	117.26	173.40	RW2
Nigeria	SOUTH SOUTH	1982	141.64	118.73	168.54	RW2
Nigeria	SOUTH SOUTH	1983	140.80	116.55	168.73	RW2
Nigeria	SOUTH SOUTH	1984	139.62	114.34	168.69	RW2
Nigeria	SOUTH SOUTH	1985	138.71	115.51	165.15	RW2
Nigeria	SOUTH SOUTH	1986	137.50	116.48	161.03	RW2

Continued on next page

Country	Region	Year	Median	Lower	Upper	Method
Nigeria	SOUTH SOUTH	1987	136.29	116.42	158.72	RW2
Nigeria	SOUTH SOUTH	1988	135.04	114.98	157.66	RW2
Nigeria	SOUTH SOUTH	1989	133.93	113.03	158.44	RW2
Nigeria	SOUTH SOUTH	1990	132.78	112.10	156.48	RW2
Nigeria	SOUTH SOUTH	1991	132.07	112.93	153.94	RW2
Nigeria	SOUTH SOUTH	1992	131.74	113.35	152.43	RW2
Nigeria	SOUTH SOUTH	1993	131.83	112.85	153.26	RW2
Nigeria	SOUTH SOUTH	1994	132.08	111.71	155.22	RW2
Nigeria	SOUTH SOUTH	1995	132.82	112.67	156.03	RW2
Nigeria	SOUTH SOUTH	1996	133.01	114.11	154.66	RW2
Nigeria	SOUTH SOUTH	1997	132.84	114.54	153.58	RW2
Nigeria	SOUTH SOUTH	1998	132.18	113.58	153.49	RW2
Nigeria	SOUTH SOUTH	1999	130.94	110.98	153.82	RW2
Nigeria	SOUTH SOUTH	2000	129.18	109.62	151.51	RW2
Nigeria	SOUTH SOUTH	2001	127.09	109.05	147.79	RW2
Nigeria	SOUTH SOUTH	2002	124.66	107.66	144.34	RW2
Nigeria	SOUTH SOUTH	2003	121.87	104.40	141.91	RW2
Nigeria	SOUTH SOUTH	2004	118.79	100.65	140.00	RW2
Nigeria	SOUTH SOUTH	2005	115.57	97.57	136.37	RW2
Nigeria	SOUTH SOUTH	2006	111.84	95.43	130.85	RW2
Nigeria	SOUTH SOUTH	2007	108.01	92.60	125.42	RW2
Nigeria	SOUTH SOUTH	2008	103.83	88.16	122.24	RW2
Nigeria	SOUTH SOUTH	2009	99.43	82.59	119.38	RW2
Nigeria	SOUTH SOUTH	2010	94.92	77.20	116.10	RW2
Nigeria	SOUTH SOUTH	2011	90.63	74.05	110.10	RW2
Nigeria	SOUTH SOUTH	2012	86.36	70.81	104.48	RW2
Nigeria	SOUTH SOUTH	2013	82.11	65.57	101.72	RW2
Nigeria	SOUTH SOUTH	2014	78.21	57.72	104.81	RW2
Nigeria	SOUTH SOUTH	2015	74.38	47.59	113.71	RW2
Nigeria	SOUTH SOUTH	2016	70.77	38.59	126.02	RW2
Nigeria	SOUTH SOUTH	2017	67.45	30.67	140.94	RW2
Nigeria	SOUTH SOUTH	2018	64.16	23.83	159.90	RW2
Nigeria	SOUTH SOUTH	2019	60.75	17.99	185.80	RW2
Nigeria	SOUTH WEST	1980	160.25	119.91	211.96	RW2
Nigeria	SOUTH WEST	1981	156.09	125.76	192.87	RW2
Nigeria	SOUTH WEST	1982	151.89	125.20	183.38	RW2
Nigeria	SOUTH WEST	1983	147.67	121.05	179.09	RW2
Nigeria	SOUTH WEST	1984	143.52	117.22	174.39	RW2
Nigeria	SOUTH WEST	1985	139.47	115.67	166.95	RW2
Nigeria	SOUTH WEST	1986	135.35	114.27	159.25	RW2
Nigeria	SOUTH WEST	1987	131.19	112.01	152.94	RW2
Nigeria	SOUTH WEST	1988	127.07	107.76	149.15	RW2
Nigeria	SOUTH WEST	1989	123.12	103.36	146.10	RW2
Nigeria	SOUTH WEST	1990	119.24	100.16	141.29	RW2
Nigeria	SOUTH WEST	1991	116.14	98.65	136.32	RW2
Nigeria	SOUTH WEST	1992	113.58	96.86	132.57	RW2
Nigeria	SOUTH WEST	1993	111.51	94.76	130.55	RW2
Nigeria	SOUTH WEST	1994	109.90	92.25	130.11	RW2
Nigeria	SOUTH WEST	1995	108.84	91.66	128.76	RW2
Nigeria	SOUTH WEST	1996	107.64	91.59	125.93	RW2
Nigeria	SOUTH WEST	1997	106.35	91.14	123.48	RW2
Nigeria	SOUTH WEST	1998	104.85	89.52	122.69	RW2
Nigeria	SOUTH WEST	1999	103.15	86.97	122.14	RW2
Nigeria	SOUTH WEST	2000	101.14	84.97	119.31	RW2
Nigeria	SOUTH WEST	2001	99.23	84.67	115.84	RW2
Nigeria	SOUTH WEST	2002	97.33	83.54	113.13	RW2
Nigeria	SOUTH WEST	2003	95.35	81.37	111.61	RW2
Nigeria	SOUTH WEST	2004	93.44	78.68	110.60	RW2
Nigeria	SOUTH WEST	2005	91.57	76.70	109.15	RW2
Nigeria	SOUTH WEST	2006	89.46	75.66	105.63	RW2
Nigeria	SOUTH WEST	2007	87.16	74.00	102.39	RW2
Nigeria	SOUTH WEST	2008	84.75	71.15	100.69	RW2
Nigeria	SOUTH WEST	2009	82.10	67.50	99.71	RW2

Continued on next page

Country	Region	Year	Median	Lower	Upper	Method
Nigeria	SOUTH WEST	2010	79.43	63.53	98.72	RW2
Nigeria	SOUTH WEST	2011	76.62	61.40	95.49	RW2
Nigeria	SOUTH WEST	2012	73.97	59.05	92.51	RW2
Nigeria	SOUTH WEST	2013	71.28	55.09	92.17	RW2
Nigeria	SOUTH WEST	2014	68.74	49.02	95.51	RW2
Nigeria	SOUTH WEST	2015	66.31	41.15	105.83	RW2
Nigeria	SOUTH WEST	2016	63.91	34.06	117.70	RW2
Nigeria	SOUTH WEST	2017	61.65	27.34	134.35	RW2
Nigeria	SOUTH WEST	2018	59.26	21.24	154.54	RW2
Nigeria	SOUTH WEST	2019	57.05	16.59	179.41	RW2
Rwanda	ALL	1980	203.05	192.59	215.35	IHME
Rwanda	ALL	1980	214.17	148.90	294.16	RW2
Rwanda	ALL	1980	218.20	203.60	233.60	UN
Rwanda	ALL	1981	191.19	181.51	201.29	IHME
Rwanda	ALL	1981	200.87	155.70	251.70	RW2
Rwanda	ALL	1981	201.60	188.50	215.60	UN
Rwanda	ALL	1982	181.05	171.17	190.84	IHME
Rwanda	ALL	1982	188.22	150.09	233.71	RW2
Rwanda	ALL	1982	186.50	174.30	199.20	UN
Rwanda	ALL	1983	173.80	164.82	182.29	IHME
Rwanda	ALL	1983	176.42	137.25	226.83	RW2
Rwanda	ALL	1983	174.40	162.70	186.40	UN
Rwanda	ALL	1984	169.52	160.86	177.98	IHME
Rwanda	ALL	1984	166.62	126.23	221.27	RW2
Rwanda	ALL	1984	166.00	155.30	177.20	UN
Rwanda	ALL	1985	166.43	157.44	175.19	IHME
Rwanda	ALL	1985	157.23	120.00	200.87	RW2
Rwanda	ALL	1985	160.10	150.10	171.10	UN
Rwanda	ALL	1986	161.66	153.18	170.85	IHME
Rwanda	ALL	1986	151.88	119.02	190.69	RW2
Rwanda	ALL	1986	155.30	145.80	165.70	UN
Rwanda	ALL	1987	155.03	147.10	163.48	IHME
Rwanda	ALL	1987	149.46	119.27	187.01	RW2
Rwanda	ALL	1987	151.60	142.50	161.30	UN
Rwanda	ALL	1988	150.27	142.06	158.65	IHME
Rwanda	ALL	1988	150.00	118.24	189.74	RW2
Rwanda	ALL	1988	149.40	140.30	159.00	UN
Rwanda	ALL	1989	150.42	143.01	158.40	IHME
Rwanda	ALL	1989	153.71	119.31	197.02	RW2
Rwanda	ALL	1989	149.30	140.40	158.80	UN
Rwanda	ALL	1990	154.43	146.27	162.85	IHME
Rwanda	ALL	1990	160.77	126.65	207.62	RW2
Rwanda	ALL	1990	151.80	142.50	161.30	UN
Rwanda	ALL	1991	159.63	151.50	168.09	IHME
Rwanda	ALL	1991	170.39	136.58	213.95	RW2
Rwanda	ALL	1991	157.30	147.60	167.30	UN
Rwanda	ALL	1992	165.94	158.01	174.80	IHME
Rwanda	ALL	1992	182.46	146.53	225.03	RW2
Rwanda	ALL	1992	165.90	154.80	177.80	UN
Rwanda	ALL	1993	171.67	162.61	181.82	IHME
Rwanda	ALL	1993	196.12	155.18	240.57	RW2
Rwanda	ALL	1993	185.40	170.40	202.20	UN
Rwanda	ALL	1994	355.64	178.00	566.20	IHME
Rwanda	ALL	1994	209.67	161.89	259.35	RW2
Rwanda	ALL	1994	299.60	270.60	332.90	UN
Rwanda	ALL	1995	182.98	170.24	192.73	IHME
Rwanda	ALL	1995	223.66	178.20	279.42	RW2
Rwanda	ALL	1995	268.30	238.60	303.00	UN
Rwanda	ALL	1996	179.69	170.04	189.79	IHME
Rwanda	ALL	1996	230.58	185.57	283.04	RW2
Rwanda	ALL	1996	203.30	179.90	231.30	UN
Rwanda	ALL	1997	180.07	170.08	190.86	IHME
Rwanda	ALL	1997	231.02	186.94	279.97	RW2

Continued on next page

Country	Region	Year	Median	Lower	Upper	Method
Rwanda	ALL	1997	223.00	198.00	252.60	UN
Rwanda	ALL	1998	175.48	166.40	185.55	IHME
Rwanda	ALL	1998	224.35	180.49	276.49	RW2
Rwanda	ALL	1998	234.00	209.90	262.10	UN
Rwanda	ALL	1999	168.19	159.11	177.47	IHME
Rwanda	ALL	1999	211.30	167.70	265.01	RW2
Rwanda	ALL	1999	200.60	183.10	220.50	UN
Rwanda	ALL	2000	158.50	149.80	167.12	IHME
Rwanda	ALL	2000	191.74	148.14	237.47	RW2
Rwanda	ALL	2000	183.80	170.30	198.70	UN
Rwanda	ALL	2001	149.20	140.30	158.28	IHME
Rwanda	ALL	2001	172.97	136.05	214.30	RW2
Rwanda	ALL	2001	170.20	158.80	182.20	UN
Rwanda	ALL	2002	138.34	130.35	147.27	IHME
Rwanda	ALL	2002	154.44	123.41	192.54	RW2
Rwanda	ALL	2002	154.70	144.70	165.50	UN
Rwanda	ALL	2003	127.90	119.92	136.83	IHME
Rwanda	ALL	2003	137.53	109.46	174.11	RW2
Rwanda	ALL	2003	139.40	130.10	149.10	UN
Rwanda	ALL	2004	115.77	108.21	123.20	IHME
Rwanda	ALL	2004	122.38	94.86	158.66	RW2
Rwanda	ALL	2004	124.40	115.70	133.50	UN
Rwanda	ALL	2005	103.56	96.91	110.88	IHME
Rwanda	ALL	2005	109.66	84.19	143.14	RW2
Rwanda	ALL	2005	110.70	102.40	119.40	UN
Rwanda	ALL	2006	92.65	86.38	99.80	IHME
Rwanda	ALL	2006	98.20	76.39	126.10	RW2
Rwanda	ALL	2006	98.60	90.90	106.90	UN
Rwanda	ALL	2007	84.48	77.81	91.66	IHME
Rwanda	ALL	2007	88.25	69.16	111.97	RW2
Rwanda	ALL	2007	87.80	80.70	95.60	UN
Rwanda	ALL	2008	78.81	71.88	85.98	IHME
Rwanda	ALL	2008	79.60	61.44	102.16	RW2
Rwanda	ALL	2008	78.30	71.60	85.80	UN
Rwanda	ALL	2009	75.80	68.35	83.63	IHME
Rwanda	ALL	2009	71.79	53.72	94.94	RW2
Rwanda	ALL	2009	70.60	64.00	78.00	UN
Rwanda	ALL	2010	73.65	65.67	82.21	IHME
Rwanda	ALL	2010	65.02	47.75	89.10	RW2
Rwanda	ALL	2010	64.10	57.10	71.90	UN
Rwanda	ALL	2011	72.72	63.54	82.46	IHME
Rwanda	ALL	2011	58.89	43.68	79.21	RW2
Rwanda	ALL	2011	57.70	50.20	66.30	UN
Rwanda	ALL	2012	71.79	61.32	83.51	IHME
Rwanda	ALL	2012	53.33	40.26	70.20	RW2
Rwanda	ALL	2012	52.10	43.60	62.30	UN
Rwanda	ALL	2013	70.39	58.61	84.25	IHME
Rwanda	ALL	2013	48.32	35.38	65.37	RW2
Rwanda	ALL	2013	47.80	38.00	60.30	UN
Rwanda	ALL	2014	68.42	56.09	83.64	IHME
Rwanda	ALL	2014	43.71	27.99	67.32	RW2
Rwanda	ALL	2014	44.30	33.50	58.90	UN
Rwanda	ALL	2015	65.64	53.52	81.81	IHME
Rwanda	ALL	2015	39.44	19.83	76.54	RW2
Rwanda	ALL	2015	41.70	29.90	58.40	UN
Rwanda	ALL	2016	35.75	14.04	88.37	RW2
Rwanda	ALL	2017	32.20	9.46	104.65	RW2
Rwanda	ALL	2018	29.06	6.29	127.91	RW2
Rwanda	ALL	2019	26.18	3.86	154.73	RW2
Rwanda	ALL	15-19	32.19	9.67	102.31	RW2
Rwanda	EAST	1980	203.28	143.80	279.75	RW2
Rwanda	EAST	1981	193.05	151.76	242.68	RW2
Rwanda	EAST	1982	183.34	148.78	224.25	RW2

Continued on next page

Country	Region	Year	Median	Lower	Upper	Method
Rwanda	EAST	1983	174.27	139.38	217.21	RW2
Rwanda	EAST	1984	166.70	131.78	211.89	RW2
Rwanda	EAST	1985	160.20	128.21	197.04	RW2
Rwanda	EAST	1986	157.16	129.94	188.61	RW2
Rwanda	EAST	1987	157.10	131.65	186.65	RW2
Rwanda	EAST	1988	160.38	133.32	192.08	RW2
Rwanda	EAST	1989	166.96	136.54	202.43	RW2
Rwanda	EAST	1990	177.20	146.57	214.35	RW2
Rwanda	EAST	1991	190.00	160.52	225.18	RW2
Rwanda	EAST	1992	205.12	175.38	239.45	RW2
Rwanda	EAST	1993	221.79	187.52	258.96	RW2
Rwanda	EAST	1994	238.06	196.19	280.02	RW2
Rwanda	EAST	1995	254.16	213.21	300.56	RW2
Rwanda	EAST	1996	264.20	225.23	307.20	RW2
Rwanda	EAST	1997	267.76	230.56	307.68	RW2
Rwanda	EAST	1998	264.53	225.89	307.19	RW2
Rwanda	EAST	1999	254.32	213.71	300.86	RW2
Rwanda	EAST	2000	237.28	196.75	279.44	RW2
Rwanda	EAST	2001	219.01	184.63	256.72	RW2
Rwanda	EAST	2002	199.79	170.13	233.93	RW2
Rwanda	EAST	2003	180.47	151.45	214.42	RW2
Rwanda	EAST	2004	162.42	133.21	197.67	RW2
Rwanda	EAST	2005	145.97	118.97	179.09	RW2
Rwanda	EAST	2006	130.65	107.85	158.01	RW2
Rwanda	EAST	2007	116.90	97.03	139.74	RW2
Rwanda	EAST	2008	104.74	85.31	127.18	RW2
Rwanda	EAST	2009	93.75	73.59	118.09	RW2
Rwanda	EAST	2010	84.09	64.52	109.44	RW2
Rwanda	EAST	2011	75.22	57.62	97.61	RW2
Rwanda	EAST	2012	67.38	51.71	86.90	RW2
Rwanda	EAST	2013	60.32	44.09	81.30	RW2
Rwanda	EAST	2014	53.95	34.74	81.86	RW2
Rwanda	EAST	2015	48.12	25.22	89.37	RW2
Rwanda	EAST	2016	42.96	17.70	98.42	RW2
Rwanda	EAST	2017	38.27	12.13	112.30	RW2
Rwanda	EAST	2018	34.11	8.14	131.78	RW2
Rwanda	EAST	2019	30.43	5.15	155.01	RW2
Rwanda	KIGALI	1980	140.15	89.32	208.07	RW2
Rwanda	KIGALI	1981	133.34	94.85	180.26	RW2
Rwanda	KIGALI	1982	126.70	94.43	166.36	RW2
Rwanda	KIGALI	1983	120.39	90.60	159.05	RW2
Rwanda	KIGALI	1984	115.47	87.06	152.77	RW2
Rwanda	KIGALI	1985	111.00	85.33	142.55	RW2
Rwanda	KIGALI	1986	109.04	86.54	136.41	RW2
Rwanda	KIGALI	1987	109.16	88.07	135.05	RW2
Rwanda	KIGALI	1988	111.31	89.36	138.21	RW2
Rwanda	KIGALI	1989	115.65	91.84	145.17	RW2
Rwanda	KIGALI	1990	122.06	98.00	152.87	RW2
Rwanda	KIGALI	1991	129.65	106.72	158.66	RW2
Rwanda	KIGALI	1992	138.23	114.93	166.09	RW2
Rwanda	KIGALI	1993	146.80	120.26	176.89	RW2
Rwanda	KIGALI	1994	154.49	124.05	187.95	RW2
Rwanda	KIGALI	1995	160.96	131.17	196.80	RW2
Rwanda	KIGALI	1996	163.04	134.04	195.68	RW2
Rwanda	KIGALI	1997	160.78	132.91	191.76	RW2
Rwanda	KIGALI	1998	154.22	126.13	186.49	RW2
Rwanda	KIGALI	1999	143.83	115.36	178.22	RW2
Rwanda	KIGALI	2000	130.39	103.24	160.17	RW2
Rwanda	KIGALI	2001	117.36	94.73	143.09	RW2
Rwanda	KIGALI	2002	104.81	85.63	127.81	RW2
Rwanda	KIGALI	2003	93.17	75.02	115.35	RW2
Rwanda	KIGALI	2004	82.90	65.28	105.40	RW2
Rwanda	KIGALI	2005	73.96	57.84	94.71	RW2

Continued on next page

Country	Region	Year	Median	Lower	Upper	Method
Rwanda	KIGALI	2006	66.04	52.08	83.61	RW2
Rwanda	KIGALI	2007	59.10	46.83	74.35	RW2
Rwanda	KIGALI	2008	52.95	41.15	68.06	RW2
Rwanda	KIGALI	2009	47.58	35.54	63.23	RW2
Rwanda	KIGALI	2010	42.84	30.86	59.30	RW2
Rwanda	KIGALI	2011	38.59	27.66	53.98	RW2
Rwanda	KIGALI	2012	34.76	24.57	49.19	RW2
Rwanda	KIGALI	2013	31.30	20.95	46.83	RW2
Rwanda	KIGALI	2014	28.19	16.72	47.43	RW2
Rwanda	KIGALI	2015	25.40	12.26	51.85	RW2
Rwanda	KIGALI	2016	22.85	8.80	57.93	RW2
Rwanda	KIGALI	2017	20.53	6.14	66.44	RW2
Rwanda	KIGALI	2018	18.49	4.16	78.85	RW2
Rwanda	KIGALI	2019	16.64	2.70	95.21	RW2
Rwanda	NORTH	1980	237.30	168.61	324.08	RW2
Rwanda	NORTH	1981	218.75	172.33	276.22	RW2
Rwanda	NORTH	1982	201.42	163.02	247.73	RW2
Rwanda	NORTH	1983	185.63	147.93	232.26	RW2
Rwanda	NORTH	1984	172.03	135.79	218.05	RW2
Rwanda	NORTH	1985	160.24	128.16	197.76	RW2
Rwanda	NORTH	1986	152.69	125.38	184.01	RW2
Rwanda	NORTH	1987	148.60	123.85	177.20	RW2
Rwanda	NORTH	1988	148.06	122.19	178.57	RW2
Rwanda	NORTH	1989	151.15	122.99	184.73	RW2
Rwanda	NORTH	1990	157.64	129.82	192.79	RW2
Rwanda	NORTH	1991	167.00	140.00	198.93	RW2
Rwanda	NORTH	1992	178.57	151.30	209.44	RW2
Rwanda	NORTH	1993	191.34	160.25	225.13	RW2
Rwanda	NORTH	1994	203.96	167.41	242.84	RW2
Rwanda	NORTH	1995	215.66	179.83	257.85	RW2
Rwanda	NORTH	1996	220.83	186.38	259.33	RW2
Rwanda	NORTH	1997	219.63	187.47	255.77	RW2
Rwanda	NORTH	1998	211.33	178.80	249.28	RW2
Rwanda	NORTH	1999	197.17	163.43	237.34	RW2
Rwanda	NORTH	2000	177.72	144.61	213.38	RW2
Rwanda	NORTH	2001	158.86	131.79	189.23	RW2
Rwanda	NORTH	2002	140.90	117.96	167.25	RW2
Rwanda	NORTH	2003	124.69	103.19	150.78	RW2
Rwanda	NORTH	2004	110.72	89.64	137.20	RW2
Rwanda	NORTH	2005	98.93	79.43	123.34	RW2
Rwanda	NORTH	2006	88.34	71.93	108.51	RW2
Rwanda	NORTH	2007	78.97	64.67	96.28	RW2
Rwanda	NORTH	2008	70.62	56.51	87.64	RW2
Rwanda	NORTH	2009	63.17	48.76	81.18	RW2
Rwanda	NORTH	2010	56.51	42.31	75.26	RW2
Rwanda	NORTH	2011	50.46	37.55	67.54	RW2
Rwanda	NORTH	2012	45.08	33.20	60.67	RW2
Rwanda	NORTH	2013	40.23	28.27	56.85	RW2
Rwanda	NORTH	2014	35.83	22.24	56.92	RW2
Rwanda	NORTH	2015	31.91	16.10	62.01	RW2
Rwanda	NORTH	2016	28.38	11.31	68.43	RW2
Rwanda	NORTH	2017	25.30	7.79	78.18	RW2
Rwanda	NORTH	2018	22.41	5.16	90.88	RW2
Rwanda	NORTH	2019	20.02	3.33	110.31	RW2
Rwanda	SOUTH	1980	187.31	133.64	254.01	RW2
Rwanda	SOUTH	1981	180.15	143.31	222.74	RW2
Rwanda	SOUTH	1982	172.93	142.01	208.91	RW2
Rwanda	SOUTH	1983	166.47	134.17	206.10	RW2
Rwanda	SOUTH	1984	161.06	127.50	203.39	RW2
Rwanda	SOUTH	1985	156.43	125.64	191.91	RW2
Rwanda	SOUTH	1986	154.84	127.90	185.41	RW2
Rwanda	SOUTH	1987	155.75	130.67	185.16	RW2
Rwanda	SOUTH	1988	159.49	132.31	191.66	RW2

Continued on next page

Country	Region	Year	Median	Lower	Upper	Method
Rwanda	SOUTH	1989	165.97	135.71	201.66	RW2
Rwanda	SOUTH	1990	175.29	145.00	213.06	RW2
Rwanda	SOUTH	1991	186.38	157.66	221.13	RW2
Rwanda	SOUTH	1992	198.59	169.16	231.73	RW2
Rwanda	SOUTH	1993	210.88	177.61	246.19	RW2
Rwanda	SOUTH	1994	222.09	183.03	261.82	RW2
Rwanda	SOUTH	1995	231.50	193.66	275.00	RW2
Rwanda	SOUTH	1996	234.68	198.98	273.65	RW2
Rwanda	SOUTH	1997	231.59	197.96	268.24	RW2
Rwanda	SOUTH	1998	222.16	188.93	260.05	RW2
Rwanda	SOUTH	1999	207.42	172.39	248.09	RW2
Rwanda	SOUTH	2000	187.62	153.38	223.60	RW2
Rwanda	SOUTH	2001	168.33	140.15	199.03	RW2
Rwanda	SOUTH	2002	149.40	126.03	176.69	RW2
Rwanda	SOUTH	2003	131.96	110.12	158.80	RW2
Rwanda	SOUTH	2004	116.57	94.78	143.39	RW2
Rwanda	SOUTH	2005	103.39	83.48	128.23	RW2
Rwanda	SOUTH	2006	91.90	75.34	112.23	RW2
Rwanda	SOUTH	2007	82.01	67.38	99.18	RW2
Rwanda	SOUTH	2008	73.59	59.27	90.64	RW2
Rwanda	SOUTH	2009	66.24	51.54	84.38	RW2
Rwanda	SOUTH	2010	60.00	45.51	79.15	RW2
Rwanda	SOUTH	2011	54.36	41.24	71.74	RW2
Rwanda	SOUTH	2012	49.33	37.48	65.21	RW2
Rwanda	SOUTH	2013	44.80	32.49	62.44	RW2
Rwanda	SOUTH	2014	40.69	26.05	63.69	RW2
Rwanda	SOUTH	2015	36.92	19.19	70.28	RW2
Rwanda	SOUTH	2016	33.42	13.83	78.91	RW2
Rwanda	SOUTH	2017	30.37	9.68	91.72	RW2
Rwanda	SOUTH	2018	27.43	6.53	109.20	RW2
Rwanda	SOUTH	2019	24.88	4.31	133.40	RW2
Rwanda	WEST	1980	240.83	173.58	324.77	RW2
Rwanda	WEST	1981	219.83	175.33	271.58	RW2
Rwanda	WEST	1982	200.28	164.20	241.61	RW2
Rwanda	WEST	1983	182.41	146.96	226.25	RW2
Rwanda	WEST	1984	167.23	132.62	211.15	RW2
Rwanda	WEST	1985	154.00	122.70	189.43	RW2
Rwanda	WEST	1986	144.99	119.02	175.30	RW2
Rwanda	WEST	1987	139.71	116.30	166.97	RW2
Rwanda	WEST	1988	137.97	113.48	166.80	RW2
Rwanda	WEST	1989	139.70	112.94	171.78	RW2
Rwanda	WEST	1990	145.26	118.66	178.79	RW2
Rwanda	WEST	1991	153.92	128.43	184.13	RW2
Rwanda	WEST	1992	165.25	139.47	194.49	RW2
Rwanda	WEST	1993	178.70	149.54	210.89	RW2
Rwanda	WEST	1994	192.76	157.67	230.15	RW2
Rwanda	WEST	1995	206.18	170.96	248.17	RW2
Rwanda	WEST	1996	213.62	180.02	251.25	RW2
Rwanda	WEST	1997	214.22	182.10	250.78	RW2
Rwanda	WEST	1998	207.48	174.93	245.38	RW2
Rwanda	WEST	1999	194.05	160.82	233.99	RW2
Rwanda	WEST	2000	175.19	143.02	210.35	RW2
Rwanda	WEST	2001	156.89	130.21	186.63	RW2
Rwanda	WEST	2002	139.46	117.07	165.69	RW2
Rwanda	WEST	2003	124.06	102.72	149.73	RW2
Rwanda	WEST	2004	111.02	89.63	136.77	RW2
Rwanda	WEST	2005	100.23	80.40	124.68	RW2
Rwanda	WEST	2006	90.81	74.25	111.32	RW2
Rwanda	WEST	2007	82.76	68.21	100.45	RW2
Rwanda	WEST	2008	75.66	61.05	93.36	RW2
Rwanda	WEST	2009	69.24	53.87	88.34	RW2
Rwanda	WEST	2010	63.62	47.97	84.26	RW2
Rwanda	WEST	2011	58.40	44.00	77.63	RW2

Continued on next page

Country	Region	Year	Median	Lower	Upper	Method
Rwanda	WEST	2012	53.63	40.12	71.82	RW2
Rwanda	WEST	2013	49.24	34.95	69.53	RW2
Rwanda	WEST	2014	45.15	28.11	71.94	RW2
Rwanda	WEST	2015	41.48	20.97	80.60	RW2
Rwanda	WEST	2016	38.00	15.17	91.72	RW2
Rwanda	WEST	2017	34.94	10.70	107.92	RW2
Rwanda	WEST	2018	31.91	7.33	128.65	RW2
Rwanda	WEST	2019	29.37	4.93	156.79	RW2
Senegal	ALL	1980	195.96	190.93	201.05	IHME
Senegal	ALL	1980	208.35	158.91	267.68	RW2
Senegal	ALL	1980	205.20	195.00	216.00	UN
Senegal	ALL	1981	189.34	184.65	194.10	IHME
Senegal	ALL	1981	200.03	165.46	239.38	RW2
Senegal	ALL	1981	198.60	188.80	208.80	UN
Senegal	ALL	1982	182.51	178.09	187.06	IHME
Senegal	ALL	1982	192.05	161.24	227.03	RW2
Senegal	ALL	1982	192.50	182.90	202.20	UN
Senegal	ALL	1983	176.24	172.04	180.47	IHME
Senegal	ALL	1983	184.12	151.09	222.29	RW2
Senegal	ALL	1983	186.50	177.30	195.70	UN
Senegal	ALL	1984	170.66	166.49	174.73	IHME
Senegal	ALL	1984	176.83	141.70	217.81	RW2
Senegal	ALL	1984	179.90	171.20	189.00	UN
Senegal	ALL	1985	165.18	161.29	169.01	IHME
Senegal	ALL	1985	169.53	138.30	205.48	RW2
Senegal	ALL	1985	172.60	164.20	181.40	UN
Senegal	ALL	1986	160.20	156.55	163.88	IHME
Senegal	ALL	1986	162.81	134.88	194.49	RW2
Senegal	ALL	1986	164.50	156.60	172.80	UN
Senegal	ALL	1987	155.43	151.92	159.14	IHME
Senegal	ALL	1987	156.61	131.16	186.39	RW2
Senegal	ALL	1987	156.60	148.90	164.70	UN
Senegal	ALL	1988	151.14	147.53	154.76	IHME
Senegal	ALL	1988	150.91	125.26	181.50	RW2
Senegal	ALL	1988	149.60	142.20	157.40	UN
Senegal	ALL	1989	147.42	144.02	151.14	IHME
Senegal	ALL	1989	146.09	119.93	178.32	RW2
Senegal	ALL	1989	144.10	136.80	151.60	UN
Senegal	ALL	1990	144.23	140.95	147.83	IHME
Senegal	ALL	1990	141.76	115.90	172.30	RW2
Senegal	ALL	1990	140.40	133.20	147.80	UN
Senegal	ALL	1991	141.53	138.15	145.17	IHME
Senegal	ALL	1991	139.35	115.48	167.08	RW2
Senegal	ALL	1991	138.70	131.60	145.90	UN
Senegal	ALL	1992	139.46	136.00	143.16	IHME
Senegal	ALL	1992	138.35	115.23	165.11	RW2
Senegal	ALL	1992	138.50	131.30	145.60	UN
Senegal	ALL	1993	137.66	134.30	141.48	IHME
Senegal	ALL	1993	138.66	114.66	166.19	RW2
Senegal	ALL	1993	139.30	132.20	146.50	UN
Senegal	ALL	1994	135.84	132.44	139.54	IHME
Senegal	ALL	1994	139.83	113.81	169.66	RW2
Senegal	ALL	1994	140.60	133.60	148.10	UN
Senegal	ALL	1995	134.01	130.65	137.70	IHME
Senegal	ALL	1995	142.43	117.40	173.74	RW2
Senegal	ALL	1995	142.10	134.80	149.90	UN
Senegal	ALL	1996	131.63	128.24	135.17	IHME
Senegal	ALL	1996	143.56	119.43	173.08	RW2
Senegal	ALL	1996	143.20	135.80	151.20	UN
Senegal	ALL	1997	129.02	125.57	132.44	IHME
Senegal	ALL	1997	143.68	119.92	171.15	RW2
Senegal	ALL	1997	143.40	135.70	151.80	UN
Senegal	ALL	1998	125.79	122.43	129.16	IHME

Continued on next page

Country	Region	Year	Median	Lower	Upper	Method
Senegal	ALL	1998	142.35	117.79	171.00	RW2
Senegal	ALL	1998	142.40	134.50	151.00	UN
Senegal	ALL	1999	122.01	118.62	125.44	IHME
Senegal	ALL	1999	139.26	113.63	168.49	RW2
Senegal	ALL	1999	139.70	131.50	148.40	UN
Senegal	ALL	2000	117.63	114.35	121.06	IHME
Senegal	ALL	2000	134.28	109.45	162.38	RW2
Senegal	ALL	2000	134.90	126.90	143.90	UN
Senegal	ALL	2001	112.72	109.51	115.93	IHME
Senegal	ALL	2001	128.17	105.52	153.93	RW2
Senegal	ALL	2001	128.40	120.60	137.20	UN
Senegal	ALL	2002	107.97	104.91	111.02	IHME
Senegal	ALL	2002	120.97	100.50	144.95	RW2
Senegal	ALL	2002	120.70	113.10	129.20	UN
Senegal	ALL	2003	102.56	99.61	105.43	IHME
Senegal	ALL	2003	113.17	93.77	136.75	RW2
Senegal	ALL	2003	112.50	105.10	120.50	UN
Senegal	ALL	2004	97.34	94.45	100.37	IHME
Senegal	ALL	2004	104.94	85.50	129.08	RW2
Senegal	ALL	2004	104.20	97.00	111.90	UN
Senegal	ALL	2005	91.99	89.13	94.95	IHME
Senegal	ALL	2005	96.64	77.91	118.80	RW2
Senegal	ALL	2005	96.20	89.10	103.80	UN
Senegal	ALL	2006	87.01	84.08	89.94	IHME
Senegal	ALL	2006	89.04	72.72	108.30	RW2
Senegal	ALL	2006	88.80	81.70	96.40	UN
Senegal	ALL	2007	82.02	79.04	84.97	IHME
Senegal	ALL	2007	82.09	67.66	99.27	RW2
Senegal	ALL	2007	82.00	74.80	89.70	UN
Senegal	ALL	2008	77.44	74.35	80.41	IHME
Senegal	ALL	2008	75.90	62.01	92.75	RW2
Senegal	ALL	2008	75.80	68.50	83.70	UN
Senegal	ALL	2009	73.00	69.83	76.09	IHME
Senegal	ALL	2009	70.24	56.19	87.69	RW2
Senegal	ALL	2009	70.10	62.50	78.70	UN
Senegal	ALL	2010	68.90	65.73	72.31	IHME
Senegal	ALL	2010	65.38	51.56	83.52	RW2
Senegal	ALL	2010	64.80	56.60	74.30	UN
Senegal	ALL	2011	64.97	61.66	68.71	IHME
Senegal	ALL	2011	60.83	48.52	76.33	RW2
Senegal	ALL	2011	60.00	50.70	71.00	UN
Senegal	ALL	2012	61.43	57.87	65.39	IHME
Senegal	ALL	2012	56.62	45.96	69.57	RW2
Senegal	ALL	2012	55.90	45.50	68.60	UN
Senegal	ALL	2013	58.07	54.37	62.41	IHME
Senegal	ALL	2013	52.75	42.01	65.75	RW2
Senegal	ALL	2013	52.50	41.20	66.90	UN
Senegal	ALL	2014	55.02	51.10	59.58	IHME
Senegal	ALL	2014	49.08	35.44	67.36	RW2
Senegal	ALL	2014	49.70	37.50	65.60	UN
Senegal	ALL	2015	52.25	48.23	57.16	IHME
Senegal	ALL	2015	45.57	27.38	74.82	RW2
Senegal	ALL	2015	47.20	34.30	64.70	UN
Senegal	ALL	2016	42.46	21.19	83.86	RW2
Senegal	ALL	2017	39.38	15.79	95.93	RW2
Senegal	ALL	2018	36.58	11.64	112.51	RW2
Senegal	ALL	2019	33.93	8.08	131.05	RW2
Senegal	ALL	15-19	39.37	16.06	94.27	RW2
Senegal	DAKAR	1980	142.65	104.39	191.96	RW2
Senegal	DAKAR	1981	137.72	110.14	170.92	RW2
Senegal	DAKAR	1982	132.82	108.76	161.00	RW2
Senegal	DAKAR	1983	127.96	103.42	157.01	RW2
Senegal	DAKAR	1984	123.22	98.65	152.31	RW2

Continued on next page

Country	Region	Year	Median	Lower	Upper	Method
Senegal	DAKAR	1985	118.74	96.43	144.68	RW2
Senegal	DAKAR	1986	114.12	94.38	136.61	RW2
Senegal	DAKAR	1987	109.51	91.65	130.11	RW2
Senegal	DAKAR	1988	105.08	87.24	126.30	RW2
Senegal	DAKAR	1989	101.06	82.96	123.39	RW2
Senegal	DAKAR	1990	97.11	79.45	118.03	RW2
Senegal	DAKAR	1991	94.79	78.64	114.15	RW2
Senegal	DAKAR	1992	93.53	77.97	112.07	RW2
Senegal	DAKAR	1993	93.28	77.29	112.19	RW2
Senegal	DAKAR	1994	93.85	76.47	114.31	RW2
Senegal	DAKAR	1995	95.54	78.30	117.06	RW2
Senegal	DAKAR	1996	96.54	79.75	116.73	RW2
Senegal	DAKAR	1997	96.98	80.41	116.12	RW2
Senegal	DAKAR	1998	96.49	79.41	116.40	RW2
Senegal	DAKAR	1999	94.88	76.86	115.92	RW2
Senegal	DAKAR	2000	92.13	74.38	112.38	RW2
Senegal	DAKAR	2001	88.33	72.43	106.62	RW2
Senegal	DAKAR	2002	83.71	69.12	100.70	RW2
Senegal	DAKAR	2003	78.40	64.50	95.31	RW2
Senegal	DAKAR	2004	72.91	59.26	89.99	RW2
Senegal	DAKAR	2005	67.08	53.81	82.96	RW2
Senegal	DAKAR	2006	62.01	50.34	76.19	RW2
Senegal	DAKAR	2007	57.43	46.85	70.39	RW2
Senegal	DAKAR	2008	53.47	43.04	66.40	RW2
Senegal	DAKAR	2009	49.95	39.26	63.50	RW2
Senegal	DAKAR	2010	47.19	36.12	61.77	RW2
Senegal	DAKAR	2011	44.40	33.95	58.24	RW2
Senegal	DAKAR	2012	41.95	31.76	55.20	RW2
Senegal	DAKAR	2013	39.60	28.73	54.13	RW2
Senegal	DAKAR	2014	37.41	24.69	55.65	RW2
Senegal	DAKAR	2015	35.30	19.88	61.71	RW2
Senegal	DAKAR	2016	33.30	15.78	69.11	RW2
Senegal	DAKAR	2017	31.45	12.10	79.95	RW2
Senegal	DAKAR	2018	29.57	8.94	93.48	RW2
Senegal	DAKAR	2019	27.86	6.65	110.73	RW2
Senegal	DIOURBEL	1980	246.27	190.34	312.39	RW2
Senegal	DIOURBEL	1981	236.60	197.20	281.57	RW2
Senegal	DIOURBEL	1982	227.33	192.96	266.38	RW2
Senegal	DIOURBEL	1983	218.37	182.41	259.21	RW2
Senegal	DIOURBEL	1984	209.65	172.12	251.30	RW2
Senegal	DIOURBEL	1985	201.34	168.35	238.78	RW2
Senegal	DIOURBEL	1986	193.00	163.84	225.20	RW2
Senegal	DIOURBEL	1987	184.97	158.42	214.61	RW2
Senegal	DIOURBEL	1988	177.44	150.65	208.03	RW2
Senegal	DIOURBEL	1989	170.64	143.61	203.54	RW2
Senegal	DIOURBEL	1990	164.54	137.74	194.28	RW2
Senegal	DIOURBEL	1991	161.02	136.54	188.67	RW2
Senegal	DIOURBEL	1992	159.55	136.61	185.47	RW2
Senegal	DIOURBEL	1993	159.89	135.67	187.35	RW2
Senegal	DIOURBEL	1994	161.81	135.43	191.20	RW2
Senegal	DIOURBEL	1995	165.60	139.66	196.93	RW2
Senegal	DIOURBEL	1996	168.45	144.17	197.66	RW2
Senegal	DIOURBEL	1997	170.41	145.90	197.66	RW2
Senegal	DIOURBEL	1998	170.56	144.96	198.90	RW2
Senegal	DIOURBEL	1999	168.78	141.13	198.70	RW2
Senegal	DIOURBEL	2000	164.77	137.77	194.88	RW2
Senegal	DIOURBEL	2001	158.50	134.23	185.67	RW2
Senegal	DIOURBEL	2002	150.48	128.39	175.85	RW2
Senegal	DIOURBEL	2003	141.10	119.53	166.09	RW2
Senegal	DIOURBEL	2004	130.95	109.28	157.23	RW2
Senegal	DIOURBEL	2005	120.30	99.40	144.25	RW2
Senegal	DIOURBEL	2006	110.94	92.75	131.78	RW2
Senegal	DIOURBEL	2007	102.71	86.62	121.81	RW2

Continued on next page

Country	Region	Year	Median	Lower	Upper	Method
Senegal	DIOURBEL	2008	95.60	79.75	114.71	RW2
Senegal	DIOURBEL	2009	89.70	72.76	109.96	RW2
Senegal	DIOURBEL	2010	85.00	68.01	106.72	RW2
Senegal	DIOURBEL	2011	80.60	64.94	99.89	RW2
Senegal	DIOURBEL	2012	76.59	62.50	94.00	RW2
Senegal	DIOURBEL	2013	72.84	57.68	91.16	RW2
Senegal	DIOURBEL	2014	69.24	50.24	94.76	RW2
Senegal	DIOURBEL	2015	65.82	40.22	105.63	RW2
Senegal	DIOURBEL	2016	62.40	32.35	117.84	RW2
Senegal	DIOURBEL	2017	59.22	24.77	135.19	RW2
Senegal	DIOURBEL	2018	56.30	18.85	156.21	RW2
Senegal	DIOURBEL	2019	53.48	13.91	182.16	RW2
Senegal	FATICK	1980	226.54	174.16	289.49	RW2
Senegal	FATICK	1981	218.47	181.40	261.24	RW2
Senegal	FATICK	1982	210.55	176.85	247.78	RW2
Senegal	FATICK	1983	202.92	168.05	242.33	RW2
Senegal	FATICK	1984	195.38	159.48	236.30	RW2
Senegal	FATICK	1985	188.31	156.14	224.83	RW2
Senegal	FATICK	1986	180.93	152.43	212.16	RW2
Senegal	FATICK	1987	173.68	147.80	202.75	RW2
Senegal	FATICK	1988	166.73	141.28	196.20	RW2
Senegal	FATICK	1989	160.52	134.44	191.56	RW2
Senegal	FATICK	1990	154.58	129.02	183.45	RW2
Senegal	FATICK	1991	151.05	128.07	177.92	RW2
Senegal	FATICK	1992	149.18	127.39	173.94	RW2
Senegal	FATICK	1993	148.93	126.24	174.63	RW2
Senegal	FATICK	1994	149.99	125.07	177.99	RW2
Senegal	FATICK	1995	152.72	128.53	182.34	RW2
Senegal	FATICK	1996	154.22	131.09	181.32	RW2
Senegal	FATICK	1997	154.85	132.24	180.16	RW2
Senegal	FATICK	1998	153.71	130.18	179.82	RW2
Senegal	FATICK	1999	150.66	125.35	179.23	RW2
Senegal	FATICK	2000	145.54	121.00	173.12	RW2
Senegal	FATICK	2001	138.40	116.51	162.64	RW2
Senegal	FATICK	2002	129.69	109.90	152.26	RW2
Senegal	FATICK	2003	119.84	101.02	142.11	RW2
Senegal	FATICK	2004	109.68	91.06	132.58	RW2
Senegal	FATICK	2005	99.07	81.03	119.56	RW2
Senegal	FATICK	2006	89.95	74.49	107.94	RW2
Senegal	FATICK	2007	81.78	67.96	98.13	RW2
Senegal	FATICK	2008	74.81	61.45	90.79	RW2
Senegal	FATICK	2009	69.03	55.29	85.78	RW2
Senegal	FATICK	2010	64.25	50.37	81.76	RW2
Senegal	FATICK	2011	59.82	47.30	75.68	RW2
Senegal	FATICK	2012	55.85	44.25	70.14	RW2
Senegal	FATICK	2013	52.19	40.08	66.91	RW2
Senegal	FATICK	2014	48.80	34.26	68.06	RW2
Senegal	FATICK	2015	45.47	27.41	74.47	RW2
Senegal	FATICK	2016	42.39	21.32	81.94	RW2
Senegal	FATICK	2017	39.62	16.28	92.63	RW2
Senegal	FATICK	2018	36.73	12.15	107.26	RW2
Senegal	FATICK	2019	34.24	8.82	124.85	RW2
Senegal	KAOLACK	1980	223.38	171.78	285.76	RW2
Senegal	KAOLACK	1981	216.24	179.67	258.40	RW2
Senegal	KAOLACK	1982	209.24	176.98	245.48	RW2
Senegal	KAOLACK	1983	202.33	168.52	241.01	RW2
Senegal	KAOLACK	1984	195.46	160.45	235.41	RW2
Senegal	KAOLACK	1985	189.03	157.60	224.62	RW2
Senegal	KAOLACK	1986	182.31	154.32	212.81	RW2
Senegal	KAOLACK	1987	175.48	149.95	203.57	RW2
Senegal	KAOLACK	1988	169.19	143.60	198.30	RW2
Senegal	KAOLACK	1989	163.44	137.26	194.69	RW2
Senegal	KAOLACK	1990	157.94	132.34	186.53	RW2

Continued on next page

Country	Region	Year	Median	Lower	Upper	Method
Senegal	KAOLACK	1991	154.82	131.74	181.31	RW2
Senegal	KAOLACK	1992	153.52	131.58	178.50	RW2
Senegal	KAOLACK	1993	153.97	131.20	179.72	RW2
Senegal	KAOLACK	1994	155.71	130.56	183.67	RW2
Senegal	KAOLACK	1995	159.21	134.49	189.05	RW2
Senegal	KAOLACK	1996	161.60	138.04	188.76	RW2
Senegal	KAOLACK	1997	163.02	139.73	189.06	RW2
Senegal	KAOLACK	1998	162.78	138.88	189.36	RW2
Senegal	KAOLACK	1999	160.45	134.56	189.10	RW2
Senegal	KAOLACK	2000	155.75	130.43	183.60	RW2
Senegal	KAOLACK	2001	148.90	126.46	173.91	RW2
Senegal	KAOLACK	2002	140.01	119.89	162.71	RW2
Senegal	KAOLACK	2003	129.96	110.60	153.32	RW2
Senegal	KAOLACK	2004	119.04	99.79	142.91	RW2
Senegal	KAOLACK	2005	107.64	89.29	128.42	RW2
Senegal	KAOLACK	2006	97.59	82.12	115.31	RW2
Senegal	KAOLACK	2007	88.64	75.14	104.29	RW2
Senegal	KAOLACK	2008	80.81	67.80	96.16	RW2
Senegal	KAOLACK	2009	74.09	60.67	90.13	RW2
Senegal	KAOLACK	2010	68.52	55.21	85.57	RW2
Senegal	KAOLACK	2011	63.42	51.29	77.94	RW2
Senegal	KAOLACK	2012	58.74	48.16	71.45	RW2
Senegal	KAOLACK	2013	54.49	43.45	67.77	RW2
Senegal	KAOLACK	2014	50.44	36.50	68.79	RW2
Senegal	KAOLACK	2015	46.72	28.58	75.29	RW2
Senegal	KAOLACK	2016	43.32	22.17	82.88	RW2
Senegal	KAOLACK	2017	39.99	16.67	92.51	RW2
Senegal	KAOLACK	2018	36.88	12.37	105.59	RW2
Senegal	KAOLACK	2019	34.17	8.93	124.43	RW2
Senegal	KOLDA	1980	261.52	202.11	331.10	RW2
Senegal	KOLDA	1981	256.86	214.63	304.49	RW2
Senegal	KOLDA	1982	252.21	214.43	293.99	RW2
Senegal	KOLDA	1983	247.33	207.68	291.38	RW2
Senegal	KOLDA	1984	242.35	201.02	288.64	RW2
Senegal	KOLDA	1985	237.55	199.78	278.47	RW2
Senegal	KOLDA	1986	231.55	198.53	267.24	RW2
Senegal	KOLDA	1987	225.12	194.50	258.60	RW2
Senegal	KOLDA	1988	218.56	187.63	253.69	RW2
Senegal	KOLDA	1989	212.27	179.93	249.87	RW2
Senegal	KOLDA	1990	205.84	174.27	241.49	RW2
Senegal	KOLDA	1991	202.42	173.03	235.34	RW2
Senegal	KOLDA	1992	201.04	173.22	232.22	RW2
Senegal	KOLDA	1993	202.00	173.09	234.31	RW2
Senegal	KOLDA	1994	204.73	172.91	239.40	RW2
Senegal	KOLDA	1995	209.76	179.26	246.33	RW2
Senegal	KOLDA	1996	213.58	184.43	247.12	RW2
Senegal	KOLDA	1997	216.38	187.74	247.90	RW2
Senegal	KOLDA	1998	217.14	186.33	249.78	RW2
Senegal	KOLDA	1999	215.12	182.23	250.07	RW2
Senegal	KOLDA	2000	210.35	178.51	244.94	RW2
Senegal	KOLDA	2001	202.49	173.34	234.09	RW2
Senegal	KOLDA	2002	192.13	165.85	221.66	RW2
Senegal	KOLDA	2003	179.59	153.59	209.51	RW2
Senegal	KOLDA	2004	166.12	140.25	197.38	RW2
Senegal	KOLDA	2005	151.71	126.26	180.07	RW2
Senegal	KOLDA	2006	139.19	117.39	164.01	RW2
Senegal	KOLDA	2007	128.11	108.60	150.61	RW2
Senegal	KOLDA	2008	118.71	99.51	140.97	RW2
Senegal	KOLDA	2009	110.84	90.80	134.34	RW2
Senegal	KOLDA	2010	104.57	84.47	129.74	RW2
Senegal	KOLDA	2011	98.85	80.51	121.28	RW2
Senegal	KOLDA	2012	93.61	77.32	113.16	RW2
Senegal	KOLDA	2013	88.78	71.38	109.99	RW2

Continued on next page

Country	Region	Year	Median	Lower	Upper	Method
Senegal	KOLDA	2014	84.13	61.72	113.55	RW2
Senegal	KOLDA	2015	79.70	49.41	125.84	RW2
Senegal	KOLDA	2016	75.38	39.00	140.61	RW2
Senegal	KOLDA	2017	71.42	30.08	162.81	RW2
Senegal	KOLDA	2018	67.63	22.66	186.80	RW2
Senegal	KOLDA	2019	64.11	16.97	216.09	RW2
Senegal	LOUGA	1980	212.52	162.34	273.52	RW2
Senegal	LOUGA	1981	203.33	167.39	246.16	RW2
Senegal	LOUGA	1982	194.80	162.34	231.81	RW2
Senegal	LOUGA	1983	186.28	153.18	224.95	RW2
Senegal	LOUGA	1984	177.87	144.13	216.69	RW2
Senegal	LOUGA	1985	169.93	139.97	204.12	RW2
Senegal	LOUGA	1986	161.64	135.79	191.00	RW2
Senegal	LOUGA	1987	153.51	129.95	179.91	RW2
Senegal	LOUGA	1988	145.68	122.91	172.51	RW2
Senegal	LOUGA	1989	138.40	115.20	166.65	RW2
Senegal	LOUGA	1990	131.33	108.95	156.88	RW2
Senegal	LOUGA	1991	126.58	106.77	149.61	RW2
Senegal	LOUGA	1992	123.36	104.40	145.01	RW2
Senegal	LOUGA	1993	121.71	102.48	143.90	RW2
Senegal	LOUGA	1994	121.15	100.05	144.87	RW2
Senegal	LOUGA	1995	122.30	101.70	147.45	RW2
Senegal	LOUGA	1996	122.55	103.05	145.61	RW2
Senegal	LOUGA	1997	122.32	102.92	144.09	RW2
Senegal	LOUGA	1998	121.01	100.94	143.60	RW2
Senegal	LOUGA	1999	118.38	97.40	142.03	RW2
Senegal	LOUGA	2000	114.44	94.02	137.72	RW2
Senegal	LOUGA	2001	109.15	90.74	129.94	RW2
Senegal	LOUGA	2002	102.71	86.27	121.82	RW2
Senegal	LOUGA	2003	95.63	79.50	114.85	RW2
Senegal	LOUGA	2004	88.33	72.77	107.77	RW2
Senegal	LOUGA	2005	80.69	65.63	98.24	RW2
Senegal	LOUGA	2006	74.34	61.37	89.74	RW2
Senegal	LOUGA	2007	68.81	57.17	82.78	RW2
Senegal	LOUGA	2008	64.16	52.62	78.06	RW2
Senegal	LOUGA	2009	60.37	48.31	75.16	RW2
Senegal	LOUGA	2010	57.41	45.23	73.35	RW2
Senegal	LOUGA	2011	54.71	43.38	69.02	RW2
Senegal	LOUGA	2012	52.24	41.83	65.32	RW2
Senegal	LOUGA	2013	50.00	39.02	63.94	RW2
Senegal	LOUGA	2014	47.82	34.21	66.72	RW2
Senegal	LOUGA	2015	45.65	27.71	74.42	RW2
Senegal	LOUGA	2016	43.63	22.23	83.85	RW2
Senegal	LOUGA	2017	41.64	17.45	96.95	RW2
Senegal	LOUGA	2018	39.76	13.20	114.69	RW2
Senegal	LOUGA	2019	38.05	9.87	137.21	RW2
Senegal	MATAM	1980	240.67	180.58	310.21	RW2
Senegal	MATAM	1981	234.32	189.86	282.92	RW2
Senegal	MATAM	1982	228.19	189.43	270.40	RW2
Senegal	MATAM	1983	221.62	182.93	265.10	RW2
Senegal	MATAM	1984	215.12	176.76	258.62	RW2
Senegal	MATAM	1985	208.60	174.17	247.19	RW2
Senegal	MATAM	1986	201.07	170.79	234.29	RW2
Senegal	MATAM	1987	193.05	165.90	224.16	RW2
Senegal	MATAM	1988	184.55	157.06	216.84	RW2
Senegal	MATAM	1989	176.14	147.83	210.12	RW2
Senegal	MATAM	1990	167.52	140.10	198.64	RW2
Senegal	MATAM	1991	161.06	136.41	189.82	RW2
Senegal	MATAM	1992	156.14	133.33	182.96	RW2
Senegal	MATAM	1993	152.84	128.99	180.32	RW2
Senegal	MATAM	1994	150.79	125.59	179.16	RW2
Senegal	MATAM	1995	150.36	126.05	180.05	RW2
Senegal	MATAM	1996	148.98	126.36	176.12	RW2

Continued on next page

Country	Region	Year	Median	Lower	Upper	Method
Senegal	MATAM	1997	146.81	124.83	171.98	RW2
Senegal	MATAM	1998	143.54	120.73	168.89	RW2
Senegal	MATAM	1999	138.75	114.92	165.56	RW2
Senegal	MATAM	2000	132.34	109.49	158.32	RW2
Senegal	MATAM	2001	124.56	104.05	147.64	RW2
Senegal	MATAM	2002	115.76	97.39	136.99	RW2
Senegal	MATAM	2003	106.23	88.55	126.88	RW2
Senegal	MATAM	2004	96.72	79.51	117.85	RW2
Senegal	MATAM	2005	87.13	70.77	106.11	RW2
Senegal	MATAM	2006	79.15	65.00	95.53	RW2
Senegal	MATAM	2007	72.15	59.72	87.05	RW2
Senegal	MATAM	2008	66.42	54.20	81.07	RW2
Senegal	MATAM	2009	61.60	49.03	77.14	RW2
Senegal	MATAM	2010	57.94	45.26	74.47	RW2
Senegal	MATAM	2011	54.44	42.77	69.29	RW2
Senegal	MATAM	2012	51.35	40.67	65.28	RW2
Senegal	MATAM	2013	48.52	37.26	63.29	RW2
Senegal	MATAM	2014	45.83	32.16	65.12	RW2
Senegal	MATAM	2015	43.25	25.89	71.50	RW2
Senegal	MATAM	2016	40.83	20.40	80.23	RW2
Senegal	MATAM	2017	38.50	15.81	91.98	RW2
Senegal	MATAM	2018	36.22	12.06	106.30	RW2
Senegal	MATAM	2019	34.23	8.85	126.07	RW2
Senegal	SAINT-LOUIS	1980	222.63	168.02	288.99	RW2
Senegal	SAINT-LOUIS	1981	214.28	174.99	259.66	RW2
Senegal	SAINT-LOUIS	1982	205.82	171.28	245.01	RW2
Senegal	SAINT-LOUIS	1983	197.55	162.18	237.97	RW2
Senegal	SAINT-LOUIS	1984	189.22	153.48	230.28	RW2
Senegal	SAINT-LOUIS	1985	181.07	149.33	217.82	RW2
Senegal	SAINT-LOUIS	1986	172.14	144.05	203.63	RW2
Senegal	SAINT-LOUIS	1987	162.80	137.50	192.21	RW2
Senegal	SAINT-LOUIS	1988	153.37	128.62	182.62	RW2
Senegal	SAINT-LOUIS	1989	144.28	120.00	174.19	RW2
Senegal	SAINT-LOUIS	1990	135.39	111.75	162.72	RW2
Senegal	SAINT-LOUIS	1991	128.59	107.60	153.01	RW2
Senegal	SAINT-LOUIS	1992	123.34	103.78	146.33	RW2
Senegal	SAINT-LOUIS	1993	119.65	99.97	142.61	RW2
Senegal	SAINT-LOUIS	1994	117.24	96.65	141.12	RW2
Senegal	SAINT-LOUIS	1995	116.51	96.10	141.20	RW2
Senegal	SAINT-LOUIS	1996	115.08	96.15	137.86	RW2
Senegal	SAINT-LOUIS	1997	113.45	94.76	134.49	RW2
Senegal	SAINT-LOUIS	1998	110.92	91.74	132.28	RW2
Senegal	SAINT-LOUIS	1999	107.54	87.92	130.09	RW2
Senegal	SAINT-LOUIS	2000	103.10	83.79	124.77	RW2
Senegal	SAINT-LOUIS	2001	97.67	80.49	117.26	RW2
Senegal	SAINT-LOUIS	2002	91.35	75.95	109.30	RW2
Senegal	SAINT-LOUIS	2003	84.55	69.93	102.24	RW2
Senegal	SAINT-LOUIS	2004	77.67	63.21	95.60	RW2
Senegal	SAINT-LOUIS	2005	70.62	56.91	86.86	RW2
Senegal	SAINT-LOUIS	2006	64.76	52.73	79.15	RW2
Senegal	SAINT-LOUIS	2007	59.70	48.80	73.09	RW2
Senegal	SAINT-LOUIS	2008	55.50	44.74	68.88	RW2
Senegal	SAINT-LOUIS	2009	52.04	40.75	66.44	RW2
Senegal	SAINT-LOUIS	2010	49.45	37.80	64.92	RW2
Senegal	SAINT-LOUIS	2011	47.01	35.90	61.82	RW2
Senegal	SAINT-LOUIS	2012	44.78	34.07	59.28	RW2
Senegal	SAINT-LOUIS	2013	42.71	31.52	58.41	RW2
Senegal	SAINT-LOUIS	2014	40.76	27.65	60.63	RW2
Senegal	SAINT-LOUIS	2015	38.89	22.40	67.22	RW2
Senegal	SAINT-LOUIS	2016	37.19	18.11	75.75	RW2
Senegal	SAINT-LOUIS	2017	35.36	14.10	88.32	RW2
Senegal	SAINT-LOUIS	2018	33.77	10.79	103.29	RW2
Senegal	SAINT-LOUIS	2019	32.25	8.09	125.14	RW2

Continued on next page

Country	Region	Year	Median	Lower	Upper	Method
Senegal	TAMBACOUNDA	1980	228.90	171.96	296.29	RW2
Senegal	TAMBACOUNDA	1981	226.29	183.60	274.83	RW2
Senegal	TAMBACOUNDA	1982	223.61	184.82	265.96	RW2
Senegal	TAMBACOUNDA	1983	220.68	180.96	265.00	RW2
Senegal	TAMBACOUNDA	1984	217.67	177.50	263.52	RW2
Senegal	TAMBACOUNDA	1985	214.58	177.90	255.71	RW2
Senegal	TAMBACOUNDA	1986	210.71	178.29	247.06	RW2
Senegal	TAMBACOUNDA	1987	206.39	176.51	240.19	RW2
Senegal	TAMBACOUNDA	1988	201.74	171.67	236.11	RW2
Senegal	TAMBACOUNDA	1989	197.26	166.55	234.26	RW2
Senegal	TAMBACOUNDA	1990	192.62	161.83	226.49	RW2
Senegal	TAMBACOUNDA	1991	190.52	162.62	222.12	RW2
Senegal	TAMBACOUNDA	1992	189.99	163.47	219.43	RW2
Senegal	TAMBACOUNDA	1993	191.45	163.71	222.57	RW2
Senegal	TAMBACOUNDA	1994	194.52	164.09	227.95	RW2
Senegal	TAMBACOUNDA	1995	199.37	169.24	235.30	RW2
Senegal	TAMBACOUNDA	1996	203.10	174.47	235.84	RW2
Senegal	TAMBACOUNDA	1997	205.42	177.02	236.77	RW2
Senegal	TAMBACOUNDA	1998	205.84	175.96	237.97	RW2
Senegal	TAMBACOUNDA	1999	204.03	172.37	238.75	RW2
Senegal	TAMBACOUNDA	2000	199.40	168.45	233.27	RW2
Senegal	TAMBACOUNDA	2001	192.07	164.62	222.39	RW2
Senegal	TAMBACOUNDA	2002	182.49	156.80	210.91	RW2
Senegal	TAMBACOUNDA	2003	171.12	146.21	200.41	RW2
Senegal	TAMBACOUNDA	2004	158.93	133.79	189.16	RW2
Senegal	TAMBACOUNDA	2005	145.79	121.28	173.26	RW2
Senegal	TAMBACOUNDA	2006	134.49	113.30	158.87	RW2
Senegal	TAMBACOUNDA	2007	124.29	105.32	145.80	RW2
Senegal	TAMBACOUNDA	2008	115.56	96.84	137.55	RW2
Senegal	TAMBACOUNDA	2009	108.32	88.65	131.60	RW2
Senegal	TAMBACOUNDA	2010	102.53	82.64	127.57	RW2
Senegal	TAMBACOUNDA	2011	97.10	78.95	119.22	RW2
Senegal	TAMBACOUNDA	2012	92.11	75.84	111.49	RW2
Senegal	TAMBACOUNDA	2013	87.53	70.38	107.92	RW2
Senegal	TAMBACOUNDA	2014	83.12	60.99	111.48	RW2
Senegal	TAMBACOUNDA	2015	78.79	49.14	123.64	RW2
Senegal	TAMBACOUNDA	2016	74.62	39.29	138.14	RW2
Senegal	TAMBACOUNDA	2017	70.87	30.25	157.41	RW2
Senegal	TAMBACOUNDA	2018	67.02	22.87	180.87	RW2
Senegal	TAMBACOUNDA	2019	63.43	17.14	211.80	RW2
Senegal	THIES	1980	174.11	130.74	228.36	RW2
Senegal	THIES	1981	166.40	134.50	204.16	RW2
Senegal	THIES	1982	159.00	131.21	191.69	RW2
Senegal	THIES	1983	151.81	123.29	185.01	RW2
Senegal	THIES	1984	144.73	115.99	178.67	RW2
Senegal	THIES	1985	138.28	112.67	168.18	RW2
Senegal	THIES	1986	131.75	109.41	157.08	RW2
Senegal	THIES	1987	125.45	105.03	148.45	RW2
Senegal	THIES	1988	119.49	99.71	142.66	RW2
Senegal	THIES	1989	114.15	94.32	138.58	RW2
Senegal	THIES	1990	109.10	89.98	131.37	RW2
Senegal	THIES	1991	105.94	88.74	125.99	RW2
Senegal	THIES	1992	104.19	87.81	123.12	RW2
Senegal	THIES	1993	103.54	86.60	123.40	RW2
Senegal	THIES	1994	104.00	85.91	125.02	RW2
Senegal	THIES	1995	105.56	87.85	128.06	RW2
Senegal	THIES	1996	106.41	89.29	127.10	RW2
Senegal	THIES	1997	106.54	89.74	125.86	RW2
Senegal	THIES	1998	105.53	87.94	125.59	RW2
Senegal	THIES	1999	103.20	85.00	124.15	RW2
Senegal	THIES	2000	99.53	81.58	120.05	RW2
Senegal	THIES	2001	94.51	78.70	112.87	RW2
Senegal	THIES	2002	88.51	74.17	105.31	RW2

Continued on next page

Country	Region	Year	Median	Lower	Upper	Method
Senegal	THIES	2003	81.78	68.01	98.54	RW2
Senegal	THIES	2004	74.88	61.21	92.18	RW2
Senegal	THIES	2005	67.73	54.67	83.23	RW2
Senegal	THIES	2006	61.57	50.37	75.23	RW2
Senegal	THIES	2007	56.10	46.04	68.20	RW2
Senegal	THIES	2008	51.42	41.62	63.57	RW2
Senegal	THIES	2009	47.42	37.42	59.97	RW2
Senegal	THIES	2010	44.20	34.14	57.61	RW2
Senegal	THIES	2011	41.12	31.78	53.23	RW2
Senegal	THIES	2012	38.40	29.64	49.43	RW2
Senegal	THIES	2013	35.89	26.80	47.48	RW2
Senegal	THIES	2014	33.53	22.97	48.29	RW2
Senegal	THIES	2015	31.21	18.18	52.36	RW2
Senegal	THIES	2016	29.12	14.33	58.00	RW2
Senegal	THIES	2017	27.20	11.00	65.88	RW2
Senegal	THIES	2018	25.37	8.29	76.66	RW2
Senegal	THIES	2019	23.64	5.92	90.98	RW2
Senegal	ZUGUINCHOR	1980	224.57	163.56	300.89	RW2
Senegal	ZUGUINCHOR	1981	217.42	170.82	272.82	RW2
Senegal	ZUGUINCHOR	1982	210.15	169.72	257.63	RW2
Senegal	ZUGUINCHOR	1983	203.02	163.05	249.22	RW2
Senegal	ZUGUINCHOR	1984	195.90	156.08	242.09	RW2
Senegal	ZUGUINCHOR	1985	189.04	153.31	230.19	RW2
Senegal	ZUGUINCHOR	1986	181.56	150.71	217.50	RW2
Senegal	ZUGUINCHOR	1987	173.96	145.51	206.75	RW2
Senegal	ZUGUINCHOR	1988	166.60	138.30	199.74	RW2
Senegal	ZUGUINCHOR	1989	159.70	131.77	193.72	RW2
Senegal	ZUGUINCHOR	1990	152.85	125.90	183.91	RW2
Senegal	ZUGUINCHOR	1991	148.33	123.80	176.52	RW2
Senegal	ZUGUINCHOR	1992	145.51	122.47	172.27	RW2
Senegal	ZUGUINCHOR	1993	144.52	120.70	171.70	RW2
Senegal	ZUGUINCHOR	1994	144.84	119.59	173.79	RW2
Senegal	ZUGUINCHOR	1995	146.56	121.71	176.77	RW2
Senegal	ZUGUINCHOR	1996	147.38	124.13	174.84	RW2
Senegal	ZUGUINCHOR	1997	147.06	124.15	173.55	RW2
Senegal	ZUGUINCHOR	1998	145.36	121.57	172.31	RW2
Senegal	ZUGUINCHOR	1999	141.70	116.82	170.17	RW2
Senegal	ZUGUINCHOR	2000	135.89	111.75	163.75	RW2
Senegal	ZUGUINCHOR	2001	128.26	106.59	153.37	RW2
Senegal	ZUGUINCHOR	2002	119.02	99.16	142.13	RW2
Senegal	ZUGUINCHOR	2003	108.76	89.73	131.52	RW2
Senegal	ZUGUINCHOR	2004	98.33	80.00	121.06	RW2
Senegal	ZUGUINCHOR	2005	87.64	70.06	108.74	RW2
Senegal	ZUGUINCHOR	2006	78.57	63.35	96.83	RW2
Senegal	ZUGUINCHOR	2007	70.69	57.06	87.30	RW2
Senegal	ZUGUINCHOR	2008	64.09	50.73	80.62	RW2
Senegal	ZUGUINCHOR	2009	58.59	45.19	75.65	RW2
Senegal	ZUGUINCHOR	2010	54.20	40.80	72.34	RW2
Senegal	ZUGUINCHOR	2011	50.23	37.41	67.36	RW2
Senegal	ZUGUINCHOR	2012	46.67	34.39	63.03	RW2
Senegal	ZUGUINCHOR	2013	43.39	30.67	60.87	RW2
Senegal	ZUGUINCHOR	2014	40.33	25.99	61.99	RW2
Senegal	ZUGUINCHOR	2015	37.43	20.65	67.19	RW2
Senegal	ZUGUINCHOR	2016	34.88	15.92	74.04	RW2
Senegal	ZUGUINCHOR	2017	32.24	12.14	83.90	RW2
Senegal	ZUGUINCHOR	2018	29.92	9.00	96.33	RW2
Senegal	ZUGUINCHOR	2019	27.90	6.45	112.92	RW2
Sierra Leone	ALL	1980	275.05	260.44	289.33	IHME
Sierra Leone	ALL	1980	279.36	192.54	385.69	RW2
Sierra Leone	ALL	1980	287.60	258.00	319.40	UN
Sierra Leone	ALL	1981	273.24	259.96	285.89	IHME
Sierra Leone	ALL	1981	278.52	209.77	358.94	RW2
Sierra Leone	ALL	1981	284.00	254.90	315.50	UN

Continued on next page

Country	Region	Year	Median	Lower	Upper	Method
Sierra Leone	ALL	1982	270.83	258.91	282.58	IHME
Sierra Leone	ALL	1982	277.90	219.57	344.56	RW2
Sierra Leone	ALL	1982	280.90	252.10	311.40	UN
Sierra Leone	ALL	1983	268.44	257.48	279.28	IHME
Sierra Leone	ALL	1983	276.70	221.36	339.70	RW2
Sierra Leone	ALL	1983	277.90	249.90	307.30	UN
Sierra Leone	ALL	1984	265.68	255.59	275.97	IHME
Sierra Leone	ALL	1984	276.07	219.96	339.23	RW2
Sierra Leone	ALL	1984	275.20	248.20	303.50	UN
Sierra Leone	ALL	1985	263.01	253.54	272.42	IHME
Sierra Leone	ALL	1985	274.68	223.19	333.40	RW2
Sierra Leone	ALL	1985	272.80	246.50	299.50	UN
Sierra Leone	ALL	1986	260.24	251.04	269.39	IHME
Sierra Leone	ALL	1986	273.36	224.75	328.19	RW2
Sierra Leone	ALL	1986	270.50	245.20	296.40	UN
Sierra Leone	ALL	1987	257.03	248.62	265.74	IHME
Sierra Leone	ALL	1987	271.94	225.62	324.60	RW2
Sierra Leone	ALL	1987	268.20	244.00	293.30	UN
Sierra Leone	ALL	1988	253.58	245.57	261.97	IHME
Sierra Leone	ALL	1988	270.07	223.16	323.02	RW2
Sierra Leone	ALL	1988	266.70	243.70	290.50	UN
Sierra Leone	ALL	1989	249.81	242.31	257.69	IHME
Sierra Leone	ALL	1989	268.11	220.70	322.01	RW2
Sierra Leone	ALL	1989	265.20	243.30	288.10	UN
Sierra Leone	ALL	1990	245.64	238.57	253.63	IHME
Sierra Leone	ALL	1990	265.91	219.74	317.79	RW2
Sierra Leone	ALL	1990	264.30	243.20	286.30	UN
Sierra Leone	ALL	1991	242.15	235.07	250.01	IHME
Sierra Leone	ALL	1991	263.91	220.20	311.91	RW2
Sierra Leone	ALL	1991	263.40	243.10	284.60	UN
Sierra Leone	ALL	1992	238.43	231.55	246.02	IHME
Sierra Leone	ALL	1992	261.95	219.41	308.61	RW2
Sierra Leone	ALL	1992	262.60	242.70	283.20	UN
Sierra Leone	ALL	1993	234.39	227.95	241.79	IHME
Sierra Leone	ALL	1993	260.08	217.02	307.75	RW2
Sierra Leone	ALL	1993	261.50	242.20	282.00	UN
Sierra Leone	ALL	1994	230.81	224.14	238.18	IHME
Sierra Leone	ALL	1994	257.99	213.33	308.50	RW2
Sierra Leone	ALL	1994	259.90	240.80	280.40	UN
Sierra Leone	ALL	1995	227.38	220.61	234.57	IHME
Sierra Leone	ALL	1995	256.16	212.67	305.56	RW2
Sierra Leone	ALL	1995	257.50	238.80	277.90	UN
Sierra Leone	ALL	1996	222.53	216.29	229.23	IHME
Sierra Leone	ALL	1996	253.31	211.96	300.83	RW2
Sierra Leone	ALL	1996	254.40	235.70	274.40	UN
Sierra Leone	ALL	1997	219.57	213.36	225.99	IHME
Sierra Leone	ALL	1997	249.88	209.98	295.09	RW2
Sierra Leone	ALL	1997	250.50	232.00	270.20	UN
Sierra Leone	ALL	1998	218.84	211.90	226.46	IHME
Sierra Leone	ALL	1998	245.87	205.53	292.71	RW2
Sierra Leone	ALL	1998	246.00	227.80	265.10	UN
Sierra Leone	ALL	1999	216.68	209.05	225.07	IHME
Sierra Leone	ALL	1999	241.11	199.64	288.13	RW2
Sierra Leone	ALL	1999	241.10	223.30	259.60	UN
Sierra Leone	ALL	2000	210.36	204.53	216.51	IHME
Sierra Leone	ALL	2000	235.44	195.29	280.48	RW2
Sierra Leone	ALL	2000	235.80	218.50	254.00	UN
Sierra Leone	ALL	2001	205.34	199.17	211.14	IHME
Sierra Leone	ALL	2001	229.77	191.97	272.13	RW2
Sierra Leone	ALL	2001	229.90	213.30	247.70	UN
Sierra Leone	ALL	2002	199.62	193.42	205.48	IHME
Sierra Leone	ALL	2002	223.76	187.94	264.12	RW2
Sierra Leone	ALL	2002	223.90	207.60	241.20	UN

Continued on next page

Country	Region	Year	Median	Lower	Upper	Method
Sierra Leone	ALL	2003	193.63	187.42	199.44	IHME
Sierra Leone	ALL	2003	217.66	181.94	257.85	RW2
Sierra Leone	ALL	2003	217.40	201.50	234.70	UN
Sierra Leone	ALL	2004	187.57	181.41	193.36	IHME
Sierra Leone	ALL	2004	210.92	173.66	252.61	RW2
Sierra Leone	ALL	2004	210.80	195.20	227.80	UN
Sierra Leone	ALL	2005	181.49	175.37	187.32	IHME
Sierra Leone	ALL	2005	204.45	168.75	246.01	RW2
Sierra Leone	ALL	2005	203.70	188.30	220.90	UN
Sierra Leone	ALL	2006	175.31	169.00	181.60	IHME
Sierra Leone	ALL	2006	196.80	164.01	234.32	RW2
Sierra Leone	ALL	2006	196.20	180.90	213.20	UN
Sierra Leone	ALL	2007	169.06	162.65	175.45	IHME
Sierra Leone	ALL	2007	188.60	158.10	223.32	RW2
Sierra Leone	ALL	2007	188.00	173.10	204.50	UN
Sierra Leone	ALL	2008	162.66	155.85	169.59	IHME
Sierra Leone	ALL	2008	179.94	149.57	215.18	RW2
Sierra Leone	ALL	2008	179.10	164.60	195.50	UN
Sierra Leone	ALL	2009	156.71	149.69	163.98	IHME
Sierra Leone	ALL	2009	170.52	139.21	208.10	RW2
Sierra Leone	ALL	2009	169.90	155.50	185.80	UN
Sierra Leone	ALL	2010	150.56	143.18	158.00	IHME
Sierra Leone	ALL	2010	160.68	128.57	199.03	RW2
Sierra Leone	ALL	2010	160.20	145.40	176.70	UN
Sierra Leone	ALL	2011	144.62	136.81	152.43	IHME
Sierra Leone	ALL	2011	151.33	122.18	185.39	RW2
Sierra Leone	ALL	2011	150.60	134.80	168.30	UN
Sierra Leone	ALL	2012	138.39	130.24	147.07	IHME
Sierra Leone	ALL	2012	142.12	116.34	172.15	RW2
Sierra Leone	ALL	2012	141.60	124.50	160.40	UN
Sierra Leone	ALL	2013	131.99	123.37	141.18	IHME
Sierra Leone	ALL	2013	133.36	107.04	164.89	RW2
Sierra Leone	ALL	2013	133.40	114.70	153.80	UN
Sierra Leone	ALL	2014	128.46	119.39	138.14	IHME
Sierra Leone	ALL	2014	124.97	91.41	168.38	RW2
Sierra Leone	ALL	2014	126.40	105.80	148.50	UN
Sierra Leone	ALL	2015	122.40	113.03	132.22	IHME
Sierra Leone	ALL	2015	117.03	72.07	184.66	RW2
Sierra Leone	ALL	2015	120.40	97.80	145.30	UN
Sierra Leone	ALL	2016	109.82	56.52	203.25	RW2
Sierra Leone	ALL	2017	102.55	42.65	227.27	RW2
Sierra Leone	ALL	2018	95.89	31.78	258.95	RW2
Sierra Leone	ALL	2019	89.50	22.27	292.52	RW2
Sierra Leone	ALL	15-19	102.58	43.29	223.53	RW2
Sierra Leone	EASTERN	1980	320.52	222.57	437.89	RW2
Sierra Leone	EASTERN	1981	321.35	240.40	414.25	RW2
Sierra Leone	EASTERN	1982	322.29	251.80	401.70	RW2
Sierra Leone	EASTERN	1983	323.04	256.69	397.26	RW2
Sierra Leone	EASTERN	1984	323.98	258.99	396.12	RW2
Sierra Leone	EASTERN	1985	324.29	262.83	392.26	RW2
Sierra Leone	EASTERN	1986	324.87	266.92	387.95	RW2
Sierra Leone	EASTERN	1987	324.58	269.34	385.67	RW2
Sierra Leone	EASTERN	1988	324.53	270.09	383.38	RW2
Sierra Leone	EASTERN	1989	323.99	271.14	381.95	RW2
Sierra Leone	EASTERN	1990	323.08	273.23	377.21	RW2
Sierra Leone	EASTERN	1991	321.88	275.66	372.30	RW2
Sierra Leone	EASTERN	1992	320.35	275.99	368.07	RW2
Sierra Leone	EASTERN	1993	318.50	274.09	366.64	RW2
Sierra Leone	EASTERN	1994	316.20	269.80	366.92	RW2
Sierra Leone	EASTERN	1995	313.06	267.49	362.76	RW2
Sierra Leone	EASTERN	1996	309.22	266.11	356.52	RW2
Sierra Leone	EASTERN	1997	304.27	262.82	349.58	RW2
Sierra Leone	EASTERN	1998	298.35	256.41	344.16	RW2

Continued on next page

Country	Region	Year	Median	Lower	Upper	Method
Sierra Leone	EASTERN	1999	291.50	248.65	339.19	RW2
Sierra Leone	EASTERN	2000	283.47	241.72	329.15	RW2
Sierra Leone	EASTERN	2001	275.26	237.39	317.36	RW2
Sierra Leone	EASTERN	2002	266.53	229.95	306.28	RW2
Sierra Leone	EASTERN	2003	257.12	220.20	296.95	RW2
Sierra Leone	EASTERN	2004	247.38	209.71	288.63	RW2
Sierra Leone	EASTERN	2005	237.25	201.04	277.45	RW2
Sierra Leone	EASTERN	2006	226.37	193.35	262.81	RW2
Sierra Leone	EASTERN	2007	214.86	184.47	249.72	RW2
Sierra Leone	EASTERN	2008	202.97	172.08	237.55	RW2
Sierra Leone	EASTERN	2009	190.64	158.75	227.60	RW2
Sierra Leone	EASTERN	2010	178.29	145.37	216.14	RW2
Sierra Leone	EASTERN	2011	166.20	136.04	201.27	RW2
Sierra Leone	EASTERN	2012	154.67	127.04	187.11	RW2
Sierra Leone	EASTERN	2013	143.66	113.73	178.83	RW2
Sierra Leone	EASTERN	2014	133.22	96.47	180.26	RW2
Sierra Leone	EASTERN	2015	123.63	76.93	191.92	RW2
Sierra Leone	EASTERN	2016	114.47	60.31	204.85	RW2
Sierra Leone	EASTERN	2017	105.83	45.92	223.49	RW2
Sierra Leone	EASTERN	2018	97.91	34.04	248.11	RW2
Sierra Leone	EASTERN	2019	90.31	24.84	277.99	RW2
Sierra Leone	NORTHERN	1980	196.73	124.89	290.97	RW2
Sierra Leone	NORTHERN	1981	201.04	140.05	275.72	RW2
Sierra Leone	NORTHERN	1982	205.24	151.13	269.63	RW2
Sierra Leone	NORTHERN	1983	208.85	158.93	268.49	RW2
Sierra Leone	NORTHERN	1984	213.05	164.93	269.54	RW2
Sierra Leone	NORTHERN	1985	216.83	171.69	269.95	RW2
Sierra Leone	NORTHERN	1986	220.27	177.68	269.75	RW2
Sierra Leone	NORTHERN	1987	223.45	182.36	270.72	RW2
Sierra Leone	NORTHERN	1988	225.90	185.48	271.89	RW2
Sierra Leone	NORTHERN	1989	227.99	187.87	274.04	RW2
Sierra Leone	NORTHERN	1990	229.29	190.41	273.10	RW2
Sierra Leone	NORTHERN	1991	230.29	194.08	271.43	RW2
Sierra Leone	NORTHERN	1992	231.01	195.85	270.60	RW2
Sierra Leone	NORTHERN	1993	231.12	195.00	271.97	RW2
Sierra Leone	NORTHERN	1994	230.91	193.56	273.20	RW2
Sierra Leone	NORTHERN	1995	229.99	193.74	271.37	RW2
Sierra Leone	NORTHERN	1996	228.53	193.88	267.29	RW2
Sierra Leone	NORTHERN	1997	226.26	193.03	263.76	RW2
Sierra Leone	NORTHERN	1998	223.37	189.68	261.32	RW2
Sierra Leone	NORTHERN	1999	219.51	184.47	258.93	RW2
Sierra Leone	NORTHERN	2000	215.42	181.54	252.82	RW2
Sierra Leone	NORTHERN	2001	210.87	179.53	245.57	RW2
Sierra Leone	NORTHERN	2002	206.20	176.59	239.23	RW2
Sierra Leone	NORTHERN	2003	201.10	170.92	234.21	RW2
Sierra Leone	NORTHERN	2004	195.83	164.91	230.61	RW2
Sierra Leone	NORTHERN	2005	190.18	160.74	223.82	RW2
Sierra Leone	NORTHERN	2006	183.96	157.01	214.11	RW2
Sierra Leone	NORTHERN	2007	177.19	152.34	204.84	RW2
Sierra Leone	NORTHERN	2008	169.77	144.82	198.45	RW2
Sierra Leone	NORTHERN	2009	162.07	135.40	192.77	RW2
Sierra Leone	NORTHERN	2010	153.91	125.70	186.43	RW2
Sierra Leone	NORTHERN	2011	146.00	120.40	175.98	RW2
Sierra Leone	NORTHERN	2012	138.22	114.64	165.45	RW2
Sierra Leone	NORTHERN	2013	130.68	105.20	161.02	RW2
Sierra Leone	NORTHERN	2014	123.46	91.10	164.91	RW2
Sierra Leone	NORTHERN	2015	116.74	73.78	179.13	RW2
Sierra Leone	NORTHERN	2016	110.18	58.99	195.45	RW2
Sierra Leone	NORTHERN	2017	103.86	46.16	216.26	RW2
Sierra Leone	NORTHERN	2018	98.03	35.30	243.70	RW2
Sierra Leone	NORTHERN	2019	92.48	26.14	276.52	RW2
Sierra Leone	SOUTHERN	1980	332.10	221.27	465.83	RW2
Sierra Leone	SOUTHERN	1981	330.58	237.47	439.49	RW2

Continued on next page

Country	Region	Year	Median	Lower	Upper	Method
Sierra Leone	SOUTHERN	1982	328.38	247.67	420.04	RW2
Sierra Leone	SOUTHERN	1983	326.32	252.64	409.07	RW2
Sierra Leone	SOUTHERN	1984	324.09	254.32	401.41	RW2
Sierra Leone	SOUTHERN	1985	322.09	258.95	392.12	RW2
Sierra Leone	SOUTHERN	1986	319.83	261.60	383.19	RW2
Sierra Leone	SOUTHERN	1987	317.37	263.13	376.72	RW2
Sierra Leone	SOUTHERN	1988	314.82	262.24	372.32	RW2
Sierra Leone	SOUTHERN	1989	312.10	260.87	368.12	RW2
Sierra Leone	SOUTHERN	1990	309.06	260.36	361.98	RW2
Sierra Leone	SOUTHERN	1991	306.26	261.11	355.05	RW2
Sierra Leone	SOUTHERN	1992	303.07	259.61	349.67	RW2
Sierra Leone	SOUTHERN	1993	299.54	256.30	346.31	RW2
Sierra Leone	SOUTHERN	1994	296.14	251.52	343.99	RW2
Sierra Leone	SOUTHERN	1995	292.17	248.99	339.32	RW2
Sierra Leone	SOUTHERN	1996	287.74	247.20	331.77	RW2
Sierra Leone	SOUTHERN	1997	282.54	243.94	325.17	RW2
Sierra Leone	SOUTHERN	1998	276.65	238.37	319.34	RW2
Sierra Leone	SOUTHERN	1999	269.99	229.58	313.94	RW2
Sierra Leone	SOUTHERN	2000	262.22	223.07	305.31	RW2
Sierra Leone	SOUTHERN	2001	253.93	217.62	293.92	RW2
Sierra Leone	SOUTHERN	2002	244.73	210.73	282.66	RW2
Sierra Leone	SOUTHERN	2003	234.72	201.13	272.67	RW2
Sierra Leone	SOUTHERN	2004	224.19	189.94	262.36	RW2
Sierra Leone	SOUTHERN	2005	213.23	181.14	249.72	RW2
Sierra Leone	SOUTHERN	2006	201.80	173.42	233.87	RW2
Sierra Leone	SOUTHERN	2007	190.06	163.54	219.24	RW2
Sierra Leone	SOUTHERN	2008	178.37	151.75	208.29	RW2
Sierra Leone	SOUTHERN	2009	166.64	138.95	198.57	RW2
Sierra Leone	SOUTHERN	2010	155.21	126.95	188.34	RW2
Sierra Leone	SOUTHERN	2011	144.29	118.40	174.65	RW2
Sierra Leone	SOUTHERN	2012	133.96	110.28	161.68	RW2
Sierra Leone	SOUTHERN	2013	124.25	99.11	154.91	RW2
Sierra Leone	SOUTHERN	2014	115.13	84.05	155.32	RW2
Sierra Leone	SOUTHERN	2015	106.62	66.84	165.37	RW2
Sierra Leone	SOUTHERN	2016	98.49	52.27	177.07	RW2
Sierra Leone	SOUTHERN	2017	91.16	39.91	193.99	RW2
Sierra Leone	SOUTHERN	2018	83.99	29.58	215.81	RW2
Sierra Leone	SOUTHERN	2019	77.59	21.53	244.02	RW2
Sierra Leone	WESTERN	1980	255.10	140.66	442.41	RW2
Sierra Leone	WESTERN	1981	249.59	149.67	405.39	RW2
Sierra Leone	WESTERN	1982	244.54	155.37	375.19	RW2
Sierra Leone	WESTERN	1983	238.82	158.47	353.71	RW2
Sierra Leone	WESTERN	1984	233.70	159.95	333.47	RW2
Sierra Leone	WESTERN	1985	228.32	161.06	314.76	RW2
Sierra Leone	WESTERN	1986	222.47	162.26	299.75	RW2
Sierra Leone	WESTERN	1987	217.04	162.03	285.59	RW2
Sierra Leone	WESTERN	1988	211.44	159.72	275.20	RW2
Sierra Leone	WESTERN	1989	205.78	156.23	267.10	RW2
Sierra Leone	WESTERN	1990	200.41	152.60	258.55	RW2
Sierra Leone	WESTERN	1991	195.79	149.44	249.74	RW2
Sierra Leone	WESTERN	1992	191.08	145.73	243.54	RW2
Sierra Leone	WESTERN	1993	187.05	141.95	239.49	RW2
Sierra Leone	WESTERN	1994	183.64	137.53	236.52	RW2
Sierra Leone	WESTERN	1995	180.34	134.68	232.59	RW2
Sierra Leone	WESTERN	1996	177.45	133.24	226.62	RW2
Sierra Leone	WESTERN	1997	174.87	132.02	223.27	RW2
Sierra Leone	WESTERN	1998	172.79	130.75	220.20	RW2
Sierra Leone	WESTERN	1999	170.59	129.44	217.35	RW2
Sierra Leone	WESTERN	2000	168.68	129.63	213.70	RW2
Sierra Leone	WESTERN	2001	167.24	130.43	209.10	RW2
Sierra Leone	WESTERN	2002	165.83	131.25	205.27	RW2
Sierra Leone	WESTERN	2003	164.50	130.85	202.96	RW2
Sierra Leone	WESTERN	2004	163.25	129.80	201.29	RW2

Continued on next page

Country	Region	Year	Median	Lower	Upper	Method
Sierra Leone	WESTERN	2005	161.98	129.89	199.39	RW2
Sierra Leone	WESTERN	2006	160.24	130.64	195.31	RW2
Sierra Leone	WESTERN	2007	158.40	129.90	192.24	RW2
Sierra Leone	WESTERN	2008	156.17	126.60	191.54	RW2
Sierra Leone	WESTERN	2009	153.42	121.79	191.85	RW2
Sierra Leone	WESTERN	2010	150.43	116.40	192.34	RW2
Sierra Leone	WESTERN	2011	147.35	113.21	190.60	RW2
Sierra Leone	WESTERN	2012	144.14	109.20	190.03	RW2
Sierra Leone	WESTERN	2013	140.91	102.71	194.03	RW2
Sierra Leone	WESTERN	2014	137.61	92.08	204.86	RW2
Sierra Leone	WESTERN	2015	134.87	78.49	227.11	RW2
Sierra Leone	WESTERN	2016	131.76	65.21	253.03	RW2
Sierra Leone	WESTERN	2017	129.12	53.05	287.12	RW2
Sierra Leone	WESTERN	2018	125.94	42.15	326.99	RW2
Sierra Leone	WESTERN	2019	123.54	32.97	375.29	RW2
Tanzania	ALL	1980	175.21	170.28	180.41	IHME
Tanzania	ALL	1980	182.08	137.66	236.40	RW2
Tanzania	ALL	1980	181.10	170.20	193.10	UN
Tanzania	ALL	1981	173.37	168.31	178.12	IHME
Tanzania	ALL	1981	180.70	148.37	217.88	RW2
Tanzania	ALL	1981	179.70	168.70	191.30	UN
Tanzania	ALL	1982	171.60	166.81	176.10	IHME
Tanzania	ALL	1982	179.38	150.26	212.63	RW2
Tanzania	ALL	1982	178.70	168.20	190.10	UN
Tanzania	ALL	1983	169.82	165.09	174.35	IHME
Tanzania	ALL	1983	177.82	146.31	214.28	RW2
Tanzania	ALL	1983	178.30	167.70	189.20	UN
Tanzania	ALL	1984	167.83	163.28	172.25	IHME
Tanzania	ALL	1984	176.55	142.30	216.33	RW2
Tanzania	ALL	1984	177.50	167.30	188.40	UN
Tanzania	ALL	1985	165.56	161.12	169.86	IHME
Tanzania	ALL	1985	174.87	143.65	211.17	RW2
Tanzania	ALL	1985	176.30	166.20	187.20	UN
Tanzania	ALL	1986	163.70	159.49	167.81	IHME
Tanzania	ALL	1986	173.27	144.44	206.25	RW2
Tanzania	ALL	1986	174.50	164.50	185.30	UN
Tanzania	ALL	1987	161.88	157.84	165.75	IHME
Tanzania	ALL	1987	171.63	144.50	203.17	RW2
Tanzania	ALL	1987	172.10	162.40	182.40	UN
Tanzania	ALL	1988	159.56	155.59	163.45	IHME
Tanzania	ALL	1988	169.79	141.74	202.36	RW2
Tanzania	ALL	1988	169.40	159.80	179.50	UN
Tanzania	ALL	1989	157.39	153.48	161.30	IHME
Tanzania	ALL	1989	167.95	138.84	202.34	RW2
Tanzania	ALL	1989	167.10	157.40	177.00	UN
Tanzania	ALL	1990	155.17	151.18	159.19	IHME
Tanzania	ALL	1990	165.95	137.29	199.53	RW2
Tanzania	ALL	1990	165.20	155.60	175.20	UN
Tanzania	ALL	1991	152.86	148.88	156.88	IHME
Tanzania	ALL	1991	164.30	137.52	194.87	RW2
Tanzania	ALL	1991	163.90	154.40	173.60	UN
Tanzania	ALL	1992	150.59	146.71	154.79	IHME
Tanzania	ALL	1992	162.77	136.82	192.18	RW2
Tanzania	ALL	1992	162.60	153.40	172.30	UN
Tanzania	ALL	1993	148.28	144.29	152.40	IHME
Tanzania	ALL	1993	161.27	134.72	191.39	RW2
Tanzania	ALL	1993	161.50	152.30	171.10	UN
Tanzania	ALL	1994	145.74	141.75	149.88	IHME
Tanzania	ALL	1994	159.47	131.41	191.76	RW2
Tanzania	ALL	1994	159.90	150.70	169.60	UN
Tanzania	ALL	1995	142.86	138.73	146.91	IHME
Tanzania	ALL	1995	157.81	130.83	189.98	RW2
Tanzania	ALL	1995	157.60	148.40	167.50	UN

Continued on next page

Country	Region	Year	Median	Lower	Upper	Method
Tanzania	ALL	1996	139.54	135.50	143.51	IHME
Tanzania	ALL	1996	154.54	129.29	184.57	RW2
Tanzania	ALL	1996	154.30	145.00	164.20	UN
Tanzania	ALL	1997	135.65	131.59	139.77	IHME
Tanzania	ALL	1997	150.16	126.17	177.87	RW2
Tanzania	ALL	1997	149.90	140.60	160.00	UN
Tanzania	ALL	1998	131.31	127.26	135.49	IHME
Tanzania	ALL	1998	144.65	120.65	173.12	RW2
Tanzania	ALL	1998	144.50	135.30	154.50	UN
Tanzania	ALL	1999	126.54	122.45	130.62	IHME
Tanzania	ALL	1999	138.02	113.60	166.52	RW2
Tanzania	ALL	1999	137.90	129.00	147.50	UN
Tanzania	ALL	2000	121.48	117.34	125.64	IHME
Tanzania	ALL	2000	130.26	106.78	157.01	RW2
Tanzania	ALL	2000	130.60	122.00	139.80	UN
Tanzania	ALL	2001	116.21	112.04	120.46	IHME
Tanzania	ALL	2001	122.44	101.24	146.76	RW2
Tanzania	ALL	2001	122.60	114.30	131.60	UN
Tanzania	ALL	2002	110.90	106.70	115.04	IHME
Tanzania	ALL	2002	114.46	95.35	136.87	RW2
Tanzania	ALL	2002	114.70	106.40	123.50	UN
Tanzania	ALL	2003	105.69	101.43	109.97	IHME
Tanzania	ALL	2003	106.70	88.54	128.42	RW2
Tanzania	ALL	2003	106.90	98.50	115.60	UN
Tanzania	ALL	2004	100.75	96.61	105.12	IHME
Tanzania	ALL	2004	99.10	80.88	121.11	RW2
Tanzania	ALL	2004	99.40	90.90	108.20	UN
Tanzania	ALL	2005	96.02	91.56	100.59	IHME
Tanzania	ALL	2005	92.03	74.68	112.86	RW2
Tanzania	ALL	2005	92.40	83.90	101.30	UN
Tanzania	ALL	2006	91.77	87.03	96.66	IHME
Tanzania	ALL	2006	85.45	70.10	103.69	RW2
Tanzania	ALL	2006	85.80	77.10	95.30	UN
Tanzania	ALL	2007	87.64	82.63	93.00	IHME
Tanzania	ALL	2007	79.47	65.67	95.84	RW2
Tanzania	ALL	2007	79.50	70.20	89.80	UN
Tanzania	ALL	2008	83.69	78.20	89.27	IHME
Tanzania	ALL	2008	74.10	60.68	90.21	RW2
Tanzania	ALL	2008	73.70	63.80	85.00	UN
Tanzania	ALL	2009	80.02	74.21	85.95	IHME
Tanzania	ALL	2009	69.07	55.50	85.79	RW2
Tanzania	ALL	2009	68.60	57.90	80.80	UN
Tanzania	ALL	2010	76.24	70.26	83.12	IHME
Tanzania	ALL	2010	64.62	51.23	81.87	RW2
Tanzania	ALL	2010	63.40	52.10	76.80	UN
Tanzania	ALL	2011	72.68	66.28	80.01	IHME
Tanzania	ALL	2011	60.47	48.46	75.38	RW2
Tanzania	ALL	2011	58.90	46.90	73.70	UN
Tanzania	ALL	2012	69.12	62.53	77.10	IHME
Tanzania	ALL	2012	56.58	46.06	69.31	RW2
Tanzania	ALL	2012	55.70	43.10	72.00	UN
Tanzania	ALL	2013	66.00	59.18	74.23	IHME
Tanzania	ALL	2013	52.98	42.26	66.06	RW2
Tanzania	ALL	2013	53.30	39.80	71.20	UN
Tanzania	ALL	2014	63.04	55.95	71.42	IHME
Tanzania	ALL	2014	49.55	35.95	67.81	RW2
Tanzania	ALL	2014	50.50	36.50	70.20	UN
Tanzania	ALL	2015	60.08	52.89	69.16	IHME
Tanzania	ALL	2015	46.26	28.16	75.08	RW2
Tanzania	ALL	2015	48.70	33.80	70.30	UN
Tanzania	ALL	2016	43.34	22.08	83.90	RW2
Tanzania	ALL	2017	40.41	16.69	95.59	RW2
Tanzania	ALL	2018	37.75	12.49	111.57	RW2

Continued on next page

Country	Region	Year	Median	Lower	Upper	Method
Tanzania	ALL	2019	35.21	8.82	129.37	RW2
Tanzania	ALL	15-19	40.41	16.95	93.98	RW2
Tanzania	ARUSHA	1980	111.44	76.91	158.98	RW2
Tanzania	ARUSHA	1981	110.69	81.93	147.83	RW2
Tanzania	ARUSHA	1982	109.76	83.65	142.99	RW2
Tanzania	ARUSHA	1983	108.85	83.12	141.05	RW2
Tanzania	ARUSHA	1984	107.89	82.35	139.87	RW2
Tanzania	ARUSHA	1985	106.90	83.27	136.03	RW2
Tanzania	ARUSHA	1986	105.69	84.49	131.95	RW2
Tanzania	ARUSHA	1987	104.34	84.33	128.67	RW2
Tanzania	ARUSHA	1988	102.98	82.98	127.19	RW2
Tanzania	ARUSHA	1989	101.53	81.72	125.95	RW2
Tanzania	ARUSHA	1990	99.87	80.77	122.98	RW2
Tanzania	ARUSHA	1991	98.41	80.55	119.68	RW2
Tanzania	ARUSHA	1992	97.02	80.03	117.25	RW2
Tanzania	ARUSHA	1993	95.79	78.54	116.04	RW2
Tanzania	ARUSHA	1994	94.47	76.77	115.51	RW2
Tanzania	ARUSHA	1995	93.03	75.67	113.93	RW2
Tanzania	ARUSHA	1996	90.93	74.91	109.94	RW2
Tanzania	ARUSHA	1997	88.06	72.80	106.25	RW2
Tanzania	ARUSHA	1998	84.65	69.55	102.63	RW2
Tanzania	ARUSHA	1999	80.61	65.50	98.90	RW2
Tanzania	ARUSHA	2000	75.87	61.41	93.21	RW2
Tanzania	ARUSHA	2001	71.06	58.08	86.68	RW2
Tanzania	ARUSHA	2002	66.07	54.13	80.41	RW2
Tanzania	ARUSHA	2003	61.09	49.64	75.00	RW2
Tanzania	ARUSHA	2004	56.33	45.28	70.05	RW2
Tanzania	ARUSHA	2005	51.69	41.11	64.91	RW2
Tanzania	ARUSHA	2006	47.51	38.04	59.20	RW2
Tanzania	ARUSHA	2007	43.68	34.98	54.50	RW2
Tanzania	ARUSHA	2008	40.24	31.65	51.02	RW2
Tanzania	ARUSHA	2009	37.14	28.57	48.18	RW2
Tanzania	ARUSHA	2010	34.38	25.86	45.86	RW2
Tanzania	ARUSHA	2011	31.86	23.75	42.66	RW2
Tanzania	ARUSHA	2012	29.54	21.84	39.75	RW2
Tanzania	ARUSHA	2013	27.37	19.55	38.01	RW2
Tanzania	ARUSHA	2014	25.36	16.72	38.12	RW2
Tanzania	ARUSHA	2015	23.48	13.48	40.63	RW2
Tanzania	ARUSHA	2016	21.82	10.55	43.97	RW2
Tanzania	ARUSHA	2017	20.13	8.18	48.92	RW2
Tanzania	ARUSHA	2018	18.63	6.17	55.19	RW2
Tanzania	ARUSHA	2019	17.33	4.51	63.61	RW2
Tanzania	DAR ES SALAAM	1980	205.93	144.49	284.42	RW2
Tanzania	DAR ES SALAAM	1981	202.81	154.73	260.87	RW2
Tanzania	DAR ES SALAAM	1982	199.67	157.97	249.19	RW2
Tanzania	DAR ES SALAAM	1983	196.03	155.95	244.32	RW2
Tanzania	DAR ES SALAAM	1984	192.42	152.52	240.73	RW2
Tanzania	DAR ES SALAAM	1985	187.70	150.67	232.16	RW2
Tanzania	DAR ES SALAAM	1986	182.38	148.27	223.11	RW2
Tanzania	DAR ES SALAAM	1987	176.01	144.02	214.67	RW2
Tanzania	DAR ES SALAAM	1988	168.82	137.84	206.22	RW2
Tanzania	DAR ES SALAAM	1989	161.25	130.09	198.42	RW2
Tanzania	DAR ES SALAAM	1990	153.62	124.08	188.76	RW2
Tanzania	DAR ES SALAAM	1991	146.63	119.25	178.72	RW2
Tanzania	DAR ES SALAAM	1992	140.82	114.94	170.83	RW2
Tanzania	DAR ES SALAAM	1993	136.12	110.33	165.90	RW2
Tanzania	DAR ES SALAAM	1994	132.46	105.98	163.04	RW2
Tanzania	DAR ES SALAAM	1995	129.82	104.02	160.77	RW2
Tanzania	DAR ES SALAAM	1996	126.99	102.29	155.82	RW2
Tanzania	DAR ES SALAAM	1997	124.16	100.51	152.09	RW2
Tanzania	DAR ES SALAAM	1998	121.18	96.92	149.25	RW2
Tanzania	DAR ES SALAAM	1999	117.55	93.06	146.16	RW2
Tanzania	DAR ES SALAAM	2000	113.44	89.69	142.13	RW2

Continued on next page

Country	Region	Year	Median	Lower	Upper	Method
Tanzania	DAR ES SALAAM	2001	109.62	86.94	136.46	RW2
Tanzania	DAR ES SALAAM	2002	105.42	83.86	131.37	RW2
Tanzania	DAR ES SALAAM	2003	101.27	80.28	127.13	RW2
Tanzania	DAR ES SALAAM	2004	97.22	76.37	123.67	RW2
Tanzania	DAR ES SALAAM	2005	93.36	73.07	118.56	RW2
Tanzania	DAR ES SALAAM	2006	89.76	70.89	113.53	RW2
Tanzania	DAR ES SALAAM	2007	86.67	68.98	109.00	RW2
Tanzania	DAR ES SALAAM	2008	83.77	66.45	105.46	RW2
Tanzania	DAR ES SALAAM	2009	81.20	63.77	103.00	RW2
Tanzania	DAR ES SALAAM	2010	79.06	61.79	101.18	RW2
Tanzania	DAR ES SALAAM	2011	76.90	60.77	97.24	RW2
Tanzania	DAR ES SALAAM	2012	74.89	59.24	94.10	RW2
Tanzania	DAR ES SALAAM	2013	72.85	55.65	94.78	RW2
Tanzania	DAR ES SALAAM	2014	70.90	49.32	101.09	RW2
Tanzania	DAR ES SALAAM	2015	68.97	40.98	114.32	RW2
Tanzania	DAR ES SALAAM	2016	66.95	33.17	130.48	RW2
Tanzania	DAR ES SALAAM	2017	65.19	26.52	152.30	RW2
Tanzania	DAR ES SALAAM	2018	63.53	20.24	180.76	RW2
Tanzania	DAR ES SALAAM	2019	61.49	15.48	217.00	RW2
Tanzania	DODOMA	1980	231.42	165.81	311.12	RW2
Tanzania	DODOMA	1981	230.98	176.74	293.80	RW2
Tanzania	DODOMA	1982	230.50	182.51	286.23	RW2
Tanzania	DODOMA	1983	229.92	183.14	283.76	RW2
Tanzania	DODOMA	1984	229.04	182.92	282.64	RW2
Tanzania	DODOMA	1985	228.30	185.88	276.92	RW2
Tanzania	DODOMA	1986	227.14	188.55	270.52	RW2
Tanzania	DODOMA	1987	225.55	188.96	266.26	RW2
Tanzania	DODOMA	1988	223.46	187.12	264.52	RW2
Tanzania	DODOMA	1989	220.95	183.80	264.00	RW2
Tanzania	DODOMA	1990	217.82	181.58	259.68	RW2
Tanzania	DODOMA	1991	214.85	180.85	253.96	RW2
Tanzania	DODOMA	1992	211.88	178.86	249.29	RW2
Tanzania	DODOMA	1993	208.32	174.90	246.72	RW2
Tanzania	DODOMA	1994	204.53	170.29	243.73	RW2
Tanzania	DODOMA	1995	200.28	167.09	239.47	RW2
Tanzania	DODOMA	1996	194.41	163.09	230.41	RW2
Tanzania	DODOMA	1997	187.07	157.41	220.54	RW2
Tanzania	DODOMA	1998	178.26	148.75	211.97	RW2
Tanzania	DODOMA	1999	168.34	139.09	202.29	RW2
Tanzania	DODOMA	2000	157.45	128.88	189.88	RW2
Tanzania	DODOMA	2001	146.37	120.93	175.85	RW2
Tanzania	DODOMA	2002	135.41	111.98	162.58	RW2
Tanzania	DODOMA	2003	124.66	102.15	151.31	RW2
Tanzania	DODOMA	2004	114.59	92.39	141.52	RW2
Tanzania	DODOMA	2005	105.09	84.02	130.48	RW2
Tanzania	DODOMA	2006	96.60	77.66	119.82	RW2
Tanzania	DODOMA	2007	88.92	71.27	110.22	RW2
Tanzania	DODOMA	2008	82.11	64.86	103.58	RW2
Tanzania	DODOMA	2009	76.02	58.69	98.00	RW2
Tanzania	DODOMA	2010	70.70	53.35	93.69	RW2
Tanzania	DODOMA	2011	65.65	49.17	87.40	RW2
Tanzania	DODOMA	2012	61.09	45.21	82.16	RW2
Tanzania	DODOMA	2013	56.84	40.52	79.30	RW2
Tanzania	DODOMA	2014	52.87	34.81	79.73	RW2
Tanzania	DODOMA	2015	49.02	27.96	84.38	RW2
Tanzania	DODOMA	2016	45.54	22.26	91.25	RW2
Tanzania	DODOMA	2017	42.35	17.26	101.03	RW2
Tanzania	DODOMA	2018	39.33	13.13	114.42	RW2
Tanzania	DODOMA	2019	36.48	9.49	131.94	RW2
Tanzania	IRINGA	1980	229.84	170.49	303.77	RW2
Tanzania	IRINGA	1981	223.54	177.32	279.55	RW2
Tanzania	IRINGA	1982	217.90	176.52	266.60	RW2
Tanzania	IRINGA	1983	212.12	171.24	259.85	RW2

Continued on next page

Country	Region	Year	Median	Lower	Upper	Method
Tanzania	IRINGA	1984	206.46	165.98	254.53	RW2
Tanzania	IRINGA	1985	201.22	163.46	244.31	RW2
Tanzania	IRINGA	1986	195.82	161.94	234.71	RW2
Tanzania	IRINGA	1987	191.00	158.95	226.97	RW2
Tanzania	IRINGA	1988	186.21	154.89	222.34	RW2
Tanzania	IRINGA	1989	181.97	150.42	219.03	RW2
Tanzania	IRINGA	1990	177.72	146.88	212.81	RW2
Tanzania	IRINGA	1991	174.00	145.68	206.39	RW2
Tanzania	IRINGA	1992	170.72	143.88	201.13	RW2
Tanzania	IRINGA	1993	167.58	140.45	198.88	RW2
Tanzania	IRINGA	1994	164.10	135.82	196.14	RW2
Tanzania	IRINGA	1995	160.95	133.13	193.11	RW2
Tanzania	IRINGA	1996	156.35	130.54	186.07	RW2
Tanzania	IRINGA	1997	150.91	126.58	178.54	RW2
Tanzania	IRINGA	1998	144.47	120.21	172.25	RW2
Tanzania	IRINGA	1999	137.03	112.84	165.08	RW2
Tanzania	IRINGA	2000	128.85	105.79	155.60	RW2
Tanzania	IRINGA	2001	120.34	99.57	144.26	RW2
Tanzania	IRINGA	2002	111.80	93.09	133.71	RW2
Tanzania	IRINGA	2003	103.48	85.83	124.74	RW2
Tanzania	IRINGA	2004	95.40	77.94	116.70	RW2
Tanzania	IRINGA	2005	87.68	71.31	107.45	RW2
Tanzania	IRINGA	2006	80.70	65.99	98.12	RW2
Tanzania	IRINGA	2007	74.21	60.92	90.28	RW2
Tanzania	IRINGA	2008	68.42	55.38	84.39	RW2
Tanzania	IRINGA	2009	63.11	49.81	79.69	RW2
Tanzania	IRINGA	2010	58.33	45.10	75.49	RW2
Tanzania	IRINGA	2011	53.84	41.32	70.15	RW2
Tanzania	IRINGA	2012	49.78	37.80	65.17	RW2
Tanzania	IRINGA	2013	46.02	33.71	62.43	RW2
Tanzania	IRINGA	2014	42.49	28.66	62.84	RW2
Tanzania	IRINGA	2015	39.26	22.80	66.52	RW2
Tanzania	IRINGA	2016	36.19	17.95	71.77	RW2
Tanzania	IRINGA	2017	33.47	13.64	79.33	RW2
Tanzania	IRINGA	2018	30.80	10.25	89.55	RW2
Tanzania	IRINGA	2019	28.43	7.46	102.96	RW2
Tanzania	KAGERA	1980	212.30	151.83	288.63	RW2
Tanzania	KAGERA	1981	209.75	161.59	268.51	RW2
Tanzania	KAGERA	1982	207.25	164.22	258.57	RW2
Tanzania	KAGERA	1983	204.56	162.65	254.07	RW2
Tanzania	KAGERA	1984	202.04	161.01	250.58	RW2
Tanzania	KAGERA	1985	199.48	161.43	243.55	RW2
Tanzania	KAGERA	1986	196.99	162.67	236.61	RW2
Tanzania	KAGERA	1987	194.22	162.24	230.68	RW2
Tanzania	KAGERA	1988	191.72	159.77	228.48	RW2
Tanzania	KAGERA	1989	189.18	156.50	226.82	RW2
Tanzania	KAGERA	1990	186.56	154.94	223.20	RW2
Tanzania	KAGERA	1991	184.54	155.13	218.24	RW2
Tanzania	KAGERA	1992	182.72	154.40	214.90	RW2
Tanzania	KAGERA	1993	180.97	152.24	213.51	RW2
Tanzania	KAGERA	1994	179.25	149.26	212.71	RW2
Tanzania	KAGERA	1995	177.53	148.62	211.37	RW2
Tanzania	KAGERA	1996	174.34	147.02	205.32	RW2
Tanzania	KAGERA	1997	170.31	144.24	200.01	RW2
Tanzania	KAGERA	1998	164.83	138.45	194.89	RW2
Tanzania	KAGERA	1999	158.01	130.97	189.18	RW2
Tanzania	KAGERA	2000	150.03	123.62	180.34	RW2
Tanzania	KAGERA	2001	141.41	117.36	169.44	RW2
Tanzania	KAGERA	2002	132.20	109.84	158.44	RW2
Tanzania	KAGERA	2003	122.84	101.10	148.88	RW2
Tanzania	KAGERA	2004	113.51	92.00	139.61	RW2
Tanzania	KAGERA	2005	104.40	83.99	129.05	RW2
Tanzania	KAGERA	2006	95.96	77.36	118.70	RW2

Continued on next page

Country	Region	Year	Median	Lower	Upper	Method
Tanzania	KAGERA	2007	88.31	70.71	109.47	RW2
Tanzania	KAGERA	2008	81.30	64.18	102.50	RW2
Tanzania	KAGERA	2009	75.03	57.56	97.22	RW2
Tanzania	KAGERA	2010	69.44	51.74	92.28	RW2
Tanzania	KAGERA	2011	64.28	47.42	86.25	RW2
Tanzania	KAGERA	2012	59.43	43.23	80.98	RW2
Tanzania	KAGERA	2013	55.02	38.44	77.74	RW2
Tanzania	KAGERA	2014	50.82	32.68	77.69	RW2
Tanzania	KAGERA	2015	46.93	26.30	82.45	RW2
Tanzania	KAGERA	2016	43.50	20.71	88.25	RW2
Tanzania	KAGERA	2017	40.21	15.77	96.72	RW2
Tanzania	KAGERA	2018	36.98	11.89	109.98	RW2
Tanzania	KAGERA	2019	34.17	8.60	124.54	RW2
Tanzania	KIGOMA	1980	213.07	147.32	296.69	RW2
Tanzania	KIGOMA	1981	210.26	155.77	276.48	RW2
Tanzania	KIGOMA	1982	207.12	159.07	265.02	RW2
Tanzania	KIGOMA	1983	204.21	158.46	258.46	RW2
Tanzania	KIGOMA	1984	201.13	157.51	253.75	RW2
Tanzania	KIGOMA	1985	198.65	158.63	245.11	RW2
Tanzania	KIGOMA	1986	195.55	159.74	236.99	RW2
Tanzania	KIGOMA	1987	192.71	159.60	230.51	RW2
Tanzania	KIGOMA	1988	189.94	157.45	227.57	RW2
Tanzania	KIGOMA	1989	187.09	154.33	225.66	RW2
Tanzania	KIGOMA	1990	183.94	152.18	220.56	RW2
Tanzania	KIGOMA	1991	181.31	151.52	215.71	RW2
Tanzania	KIGOMA	1992	178.62	150.03	210.88	RW2
Tanzania	KIGOMA	1993	175.91	147.05	209.07	RW2
Tanzania	KIGOMA	1994	172.67	142.57	207.37	RW2
Tanzania	KIGOMA	1995	169.73	140.31	204.55	RW2
Tanzania	KIGOMA	1996	165.08	137.28	197.35	RW2
Tanzania	KIGOMA	1997	159.45	133.19	190.06	RW2
Tanzania	KIGOMA	1998	152.68	126.86	183.37	RW2
Tanzania	KIGOMA	1999	144.81	119.02	175.65	RW2
Tanzania	KIGOMA	2000	136.01	111.11	164.81	RW2
Tanzania	KIGOMA	2001	126.89	104.65	153.10	RW2
Tanzania	KIGOMA	2002	117.60	97.11	141.85	RW2
Tanzania	KIGOMA	2003	108.37	88.77	131.99	RW2
Tanzania	KIGOMA	2004	99.36	80.12	122.98	RW2
Tanzania	KIGOMA	2005	90.85	72.53	113.23	RW2
Tanzania	KIGOMA	2006	83.19	66.34	103.60	RW2
Tanzania	KIGOMA	2007	76.07	60.33	95.44	RW2
Tanzania	KIGOMA	2008	69.88	54.33	89.25	RW2
Tanzania	KIGOMA	2009	64.28	48.64	84.39	RW2
Tanzania	KIGOMA	2010	59.30	43.64	80.39	RW2
Tanzania	KIGOMA	2011	54.79	39.56	75.13	RW2
Tanzania	KIGOMA	2012	50.62	35.88	70.33	RW2
Tanzania	KIGOMA	2013	46.73	31.77	67.48	RW2
Tanzania	KIGOMA	2014	43.23	27.05	67.76	RW2
Tanzania	KIGOMA	2015	39.81	21.69	71.36	RW2
Tanzania	KIGOMA	2016	36.71	17.20	76.55	RW2
Tanzania	KIGOMA	2017	33.85	13.14	85.36	RW2
Tanzania	KIGOMA	2018	31.19	9.84	95.08	RW2
Tanzania	KIGOMA	2019	28.82	7.18	107.87	RW2
Tanzania	KILIMANJARO	1980	115.58	77.37	171.57	RW2
Tanzania	KILIMANJARO	1981	111.42	80.72	153.19	RW2
Tanzania	KILIMANJARO	1982	107.16	80.35	142.31	RW2
Tanzania	KILIMANJARO	1983	103.14	77.93	135.59	RW2
Tanzania	KILIMANJARO	1984	99.02	75.30	129.38	RW2
Tanzania	KILIMANJARO	1985	95.19	73.93	121.99	RW2
Tanzania	KILIMANJARO	1986	91.55	72.31	115.33	RW2
Tanzania	KILIMANJARO	1987	87.96	70.25	109.65	RW2
Tanzania	KILIMANJARO	1988	84.46	67.15	105.68	RW2
Tanzania	KILIMANJARO	1989	81.34	64.06	102.77	RW2

Continued on next page

Country	Region	Year	Median	Lower	Upper	Method
Tanzania	KILIMANJARO	1990	78.38	61.74	98.94	RW2
Tanzania	KILIMANJARO	1991	75.96	60.06	95.30	RW2
Tanzania	KILIMANJARO	1992	73.94	58.43	92.10	RW2
Tanzania	KILIMANJARO	1993	72.21	56.62	90.88	RW2
Tanzania	KILIMANJARO	1994	70.85	54.98	90.07	RW2
Tanzania	KILIMANJARO	1995	69.83	53.89	89.26	RW2
Tanzania	KILIMANJARO	1996	68.63	53.34	87.22	RW2
Tanzania	KILIMANJARO	1997	67.17	52.23	84.96	RW2
Tanzania	KILIMANJARO	1998	65.55	50.85	83.62	RW2
Tanzania	KILIMANJARO	1999	63.57	48.88	81.97	RW2
Tanzania	KILIMANJARO	2000	61.28	47.00	79.19	RW2
Tanzania	KILIMANJARO	2001	58.80	45.23	75.98	RW2
Tanzania	KILIMANJARO	2002	56.18	43.18	72.71	RW2
Tanzania	KILIMANJARO	2003	53.48	40.53	70.57	RW2
Tanzania	KILIMANJARO	2004	50.72	37.81	68.28	RW2
Tanzania	KILIMANJARO	2005	48.01	35.19	65.65	RW2
Tanzania	KILIMANJARO	2006	45.53	32.91	62.97	RW2
Tanzania	KILIMANJARO	2007	43.25	30.82	60.88	RW2
Tanzania	KILIMANJARO	2008	41.23	28.50	59.50	RW2
Tanzania	KILIMANJARO	2009	39.37	26.33	58.83	RW2
Tanzania	KILIMANJARO	2010	37.84	24.33	59.08	RW2
Tanzania	KILIMANJARO	2011	36.26	22.62	58.34	RW2
Tanzania	KILIMANJARO	2012	34.77	21.11	57.93	RW2
Tanzania	KILIMANJARO	2013	33.43	19.38	58.75	RW2
Tanzania	KILIMANJARO	2014	32.23	17.07	61.89	RW2
Tanzania	KILIMANJARO	2015	30.94	14.40	67.17	RW2
Tanzania	KILIMANJARO	2016	29.75	11.84	75.12	RW2
Tanzania	KILIMANJARO	2017	28.55	9.55	85.11	RW2
Tanzania	KILIMANJARO	2018	27.52	7.50	99.29	RW2
Tanzania	KILIMANJARO	2019	26.55	5.74	116.72	RW2
Tanzania	LINDI	1980	243.30	180.36	318.98	RW2
Tanzania	LINDI	1981	245.34	194.90	303.62	RW2
Tanzania	LINDI	1982	247.49	201.70	299.74	RW2
Tanzania	LINDI	1983	249.68	203.23	301.97	RW2
Tanzania	LINDI	1984	251.49	204.80	304.66	RW2
Tanzania	LINDI	1985	253.37	209.43	303.24	RW2
Tanzania	LINDI	1986	254.64	213.24	300.17	RW2
Tanzania	LINDI	1987	255.57	216.25	298.95	RW2
Tanzania	LINDI	1988	255.73	216.08	300.34	RW2
Tanzania	LINDI	1989	255.75	214.52	302.29	RW2
Tanzania	LINDI	1990	254.66	214.18	299.73	RW2
Tanzania	LINDI	1991	253.41	215.48	295.37	RW2
Tanzania	LINDI	1992	251.71	215.32	292.36	RW2
Tanzania	LINDI	1993	249.70	212.61	290.09	RW2
Tanzania	LINDI	1994	246.44	208.37	289.04	RW2
Tanzania	LINDI	1995	242.18	204.78	285.07	RW2
Tanzania	LINDI	1996	235.56	200.78	274.78	RW2
Tanzania	LINDI	1997	226.39	192.94	263.95	RW2
Tanzania	LINDI	1998	214.94	181.95	252.11	RW2
Tanzania	LINDI	1999	201.54	168.29	239.99	RW2
Tanzania	LINDI	2000	186.41	154.13	223.39	RW2
Tanzania	LINDI	2001	170.84	141.83	204.32	RW2
Tanzania	LINDI	2002	155.18	128.61	186.58	RW2
Tanzania	LINDI	2003	140.14	114.68	170.38	RW2
Tanzania	LINDI	2004	125.79	101.28	155.66	RW2
Tanzania	LINDI	2005	112.20	89.26	139.89	RW2
Tanzania	LINDI	2006	100.29	79.53	125.69	RW2
Tanzania	LINDI	2007	89.68	70.49	113.15	RW2
Tanzania	LINDI	2008	80.17	61.82	103.38	RW2
Tanzania	LINDI	2009	71.91	53.79	95.53	RW2
Tanzania	LINDI	2010	64.65	46.79	88.18	RW2
Tanzania	LINDI	2011	58.04	41.21	80.71	RW2
Tanzania	LINDI	2012	52.20	36.13	74.16	RW2

Continued on next page

Country	Region	Year	Median	Lower	Upper	Method
Tanzania	LINDI	2013	46.83	30.85	69.22	RW2
Tanzania	LINDI	2014	42.03	25.40	67.34	RW2
Tanzania	LINDI	2015	37.72	19.78	69.12	RW2
Tanzania	LINDI	2016	33.82	15.20	72.52	RW2
Tanzania	LINDI	2017	30.16	11.24	77.86	RW2
Tanzania	LINDI	2018	27.05	8.24	84.93	RW2
Tanzania	LINDI	2019	24.20	5.90	94.21	RW2
Tanzania	MARA	1980	209.17	149.83	284.47	RW2
Tanzania	MARA	1981	207.98	159.53	266.69	RW2
Tanzania	MARA	1982	206.91	163.52	258.58	RW2
Tanzania	MARA	1983	206.13	163.93	256.04	RW2
Tanzania	MARA	1984	204.83	163.50	253.74	RW2
Tanzania	MARA	1985	203.72	165.77	248.36	RW2
Tanzania	MARA	1986	202.45	167.79	242.22	RW2
Tanzania	MARA	1987	200.91	168.89	237.43	RW2
Tanzania	MARA	1988	199.47	167.15	235.70	RW2
Tanzania	MARA	1989	197.92	165.40	235.53	RW2
Tanzania	MARA	1990	196.22	164.17	232.38	RW2
Tanzania	MARA	1991	195.14	165.82	228.23	RW2
Tanzania	MARA	1992	194.43	166.05	226.18	RW2
Tanzania	MARA	1993	194.07	164.97	226.03	RW2
Tanzania	MARA	1994	193.66	162.93	228.15	RW2
Tanzania	MARA	1995	193.76	162.88	228.76	RW2
Tanzania	MARA	1996	192.26	163.45	224.92	RW2
Tanzania	MARA	1997	189.85	162.10	221.04	RW2
Tanzania	MARA	1998	186.03	157.91	217.87	RW2
Tanzania	MARA	1999	180.49	151.44	213.91	RW2
Tanzania	MARA	2000	173.54	144.87	206.24	RW2
Tanzania	MARA	2001	165.52	139.35	195.67	RW2
Tanzania	MARA	2002	156.57	132.37	184.75	RW2
Tanzania	MARA	2003	146.87	122.99	174.95	RW2
Tanzania	MARA	2004	136.92	113.14	165.29	RW2
Tanzania	MARA	2005	126.92	104.12	153.80	RW2
Tanzania	MARA	2006	117.27	96.76	141.65	RW2
Tanzania	MARA	2007	108.27	89.44	130.38	RW2
Tanzania	MARA	2008	99.91	81.56	122.24	RW2
Tanzania	MARA	2009	92.24	73.29	115.42	RW2
Tanzania	MARA	2010	85.45	66.23	109.36	RW2
Tanzania	MARA	2011	78.91	60.86	101.66	RW2
Tanzania	MARA	2012	72.96	55.76	94.59	RW2
Tanzania	MARA	2013	67.41	49.46	90.48	RW2
Tanzania	MARA	2014	62.32	42.12	90.32	RW2
Tanzania	MARA	2015	57.44	33.53	95.65	RW2
Tanzania	MARA	2016	52.89	26.19	103.86	RW2
Tanzania	MARA	2017	48.75	19.83	113.23	RW2
Tanzania	MARA	2018	44.98	14.86	128.78	RW2
Tanzania	MARA	2019	41.49	11.05	144.55	RW2
Tanzania	MBEYA	1980	151.95	106.39	212.71	RW2
Tanzania	MBEYA	1981	151.40	114.08	198.48	RW2
Tanzania	MBEYA	1982	150.90	117.15	192.15	RW2
Tanzania	MBEYA	1983	150.40	116.98	191.34	RW2
Tanzania	MBEYA	1984	150.12	116.87	190.67	RW2
Tanzania	MBEYA	1985	149.74	119.07	186.76	RW2
Tanzania	MBEYA	1986	149.57	120.83	183.69	RW2
Tanzania	MBEYA	1987	149.47	122.12	181.66	RW2
Tanzania	MBEYA	1988	149.54	122.09	182.09	RW2
Tanzania	MBEYA	1989	149.90	121.64	183.30	RW2
Tanzania	MBEYA	1990	149.90	122.07	182.87	RW2
Tanzania	MBEYA	1991	150.53	124.36	181.27	RW2
Tanzania	MBEYA	1992	150.96	125.42	180.87	RW2
Tanzania	MBEYA	1993	151.12	124.88	181.52	RW2
Tanzania	MBEYA	1994	151.10	123.86	183.26	RW2
Tanzania	MBEYA	1995	150.93	123.90	183.29	RW2

Continued on next page

Country	Region	Year	Median	Lower	Upper	Method
Tanzania	MBEYA	1996	149.14	123.63	179.09	RW2
Tanzania	MBEYA	1997	146.17	121.63	175.05	RW2
Tanzania	MBEYA	1998	142.04	117.28	170.93	RW2
Tanzania	MBEYA	1999	136.85	111.84	166.32	RW2
Tanzania	MBEYA	2000	130.34	106.27	158.76	RW2
Tanzania	MBEYA	2001	123.66	101.26	149.95	RW2
Tanzania	MBEYA	2002	116.83	96.05	141.03	RW2
Tanzania	MBEYA	2003	109.78	89.60	134.06	RW2
Tanzania	MBEYA	2004	103.21	83.12	127.61	RW2
Tanzania	MBEYA	2005	96.76	77.18	120.59	RW2
Tanzania	MBEYA	2006	91.10	72.83	112.88	RW2
Tanzania	MBEYA	2007	85.99	68.50	107.05	RW2
Tanzania	MBEYA	2008	81.48	64.08	103.18	RW2
Tanzania	MBEYA	2009	77.40	59.52	99.88	RW2
Tanzania	MBEYA	2010	74.00	55.58	97.80	RW2
Tanzania	MBEYA	2011	70.71	52.86	93.86	RW2
Tanzania	MBEYA	2012	67.65	50.11	90.73	RW2
Tanzania	MBEYA	2013	64.75	46.30	90.00	RW2
Tanzania	MBEYA	2014	61.98	41.04	92.95	RW2
Tanzania	MBEYA	2015	59.12	34.11	101.21	RW2
Tanzania	MBEYA	2016	56.65	27.81	112.21	RW2
Tanzania	MBEYA	2017	54.08	21.95	126.56	RW2
Tanzania	MBEYA	2018	51.98	17.22	147.10	RW2
Tanzania	MBEYA	2019	49.66	13.01	171.99	RW2
Tanzania	MOROGORO	1980	222.66	163.60	296.05	RW2
Tanzania	MOROGORO	1981	221.14	173.60	278.12	RW2
Tanzania	MOROGORO	1982	219.91	177.06	269.77	RW2
Tanzania	MOROGORO	1983	218.35	175.45	267.63	RW2
Tanzania	MOROGORO	1984	216.58	174.42	265.26	RW2
Tanzania	MOROGORO	1985	214.95	176.45	259.48	RW2
Tanzania	MOROGORO	1986	213.02	177.27	253.26	RW2
Tanzania	MOROGORO	1987	210.91	177.94	248.06	RW2
Tanzania	MOROGORO	1988	208.58	175.53	245.89	RW2
Tanzania	MOROGORO	1989	206.10	172.08	244.93	RW2
Tanzania	MOROGORO	1990	203.46	170.47	241.13	RW2
Tanzania	MOROGORO	1991	201.16	170.15	235.69	RW2
Tanzania	MOROGORO	1992	199.03	169.83	231.65	RW2
Tanzania	MOROGORO	1993	196.96	166.93	230.71	RW2
Tanzania	MOROGORO	1994	194.50	162.90	229.72	RW2
Tanzania	MOROGORO	1995	191.88	161.24	227.25	RW2
Tanzania	MOROGORO	1996	187.36	158.82	220.24	RW2
Tanzania	MOROGORO	1997	181.36	153.86	212.41	RW2
Tanzania	MOROGORO	1998	173.73	145.95	205.45	RW2
Tanzania	MOROGORO	1999	164.34	136.42	197.35	RW2
Tanzania	MOROGORO	2000	153.68	126.29	185.29	RW2
Tanzania	MOROGORO	2001	142.64	117.70	171.65	RW2
Tanzania	MOROGORO	2002	131.36	108.20	158.67	RW2
Tanzania	MOROGORO	2003	120.23	97.82	146.93	RW2
Tanzania	MOROGORO	2004	109.54	87.71	135.97	RW2
Tanzania	MOROGORO	2005	99.36	78.50	124.60	RW2
Tanzania	MOROGORO	2006	90.33	71.45	113.80	RW2
Tanzania	MOROGORO	2007	82.00	64.46	103.77	RW2
Tanzania	MOROGORO	2008	74.64	57.60	96.15	RW2
Tanzania	MOROGORO	2009	68.08	51.14	89.89	RW2
Tanzania	MOROGORO	2010	62.34	45.65	84.29	RW2
Tanzania	MOROGORO	2011	56.91	41.21	77.87	RW2
Tanzania	MOROGORO	2012	52.12	37.25	72.33	RW2
Tanzania	MOROGORO	2013	47.63	32.61	68.18	RW2
Tanzania	MOROGORO	2014	43.49	27.65	67.35	RW2
Tanzania	MOROGORO	2015	39.71	21.90	69.86	RW2
Tanzania	MOROGORO	2016	36.26	17.19	74.38	RW2
Tanzania	MOROGORO	2017	33.06	13.03	81.21	RW2
Tanzania	MOROGORO	2018	30.15	9.61	89.25	RW2

Continued on next page

Country	Region	Year	Median	Lower	Upper	Method
Tanzania	MOROGORO	2019	27.48	7.02	100.80	RW2
Tanzania	MTWARA	1980	194.97	136.98	269.11	RW2
Tanzania	MTWARA	1981	197.61	149.72	255.35	RW2
Tanzania	MTWARA	1982	200.69	157.67	251.09	RW2
Tanzania	MTWARA	1983	203.32	160.86	252.71	RW2
Tanzania	MTWARA	1984	206.00	163.87	255.83	RW2
Tanzania	MTWARA	1985	208.98	168.65	255.05	RW2
Tanzania	MTWARA	1986	211.80	174.63	254.00	RW2
Tanzania	MTWARA	1987	214.38	178.65	254.30	RW2
Tanzania	MTWARA	1988	216.56	180.88	257.31	RW2
Tanzania	MTWARA	1989	218.70	181.69	261.51	RW2
Tanzania	MTWARA	1990	220.02	183.39	261.94	RW2
Tanzania	MTWARA	1991	221.08	186.35	260.72	RW2
Tanzania	MTWARA	1992	221.31	187.59	259.61	RW2
Tanzania	MTWARA	1993	220.47	185.50	260.20	RW2
Tanzania	MTWARA	1994	218.01	181.77	259.50	RW2
Tanzania	MTWARA	1995	214.26	178.67	256.04	RW2
Tanzania	MTWARA	1996	207.73	174.22	246.87	RW2
Tanzania	MTWARA	1997	198.77	166.80	235.25	RW2
Tanzania	MTWARA	1998	187.45	155.80	224.22	RW2
Tanzania	MTWARA	1999	174.36	142.90	211.44	RW2
Tanzania	MTWARA	2000	160.02	130.15	195.02	RW2
Tanzania	MTWARA	2001	145.70	118.50	177.70	RW2
Tanzania	MTWARA	2002	131.38	106.55	160.59	RW2
Tanzania	MTWARA	2003	117.99	94.19	146.35	RW2
Tanzania	MTWARA	2004	105.60	82.77	133.36	RW2
Tanzania	MTWARA	2005	94.14	72.48	120.55	RW2
Tanzania	MTWARA	2006	84.34	64.79	108.50	RW2
Tanzania	MTWARA	2007	75.50	57.60	98.29	RW2
Tanzania	MTWARA	2008	67.92	50.72	90.00	RW2
Tanzania	MTWARA	2009	61.22	44.63	83.51	RW2
Tanzania	MTWARA	2010	55.48	39.30	77.96	RW2
Tanzania	MTWARA	2011	50.31	34.90	71.98	RW2
Tanzania	MTWARA	2012	45.64	30.83	66.97	RW2
Tanzania	MTWARA	2013	41.35	26.68	63.73	RW2
Tanzania	MTWARA	2014	37.57	22.22	62.81	RW2
Tanzania	MTWARA	2015	33.92	17.39	65.03	RW2
Tanzania	MTWARA	2016	30.69	13.44	69.04	RW2
Tanzania	MTWARA	2017	27.81	10.03	75.23	RW2
Tanzania	MTWARA	2018	25.15	7.35	83.26	RW2
Tanzania	MTWARA	2019	22.80	5.27	94.57	RW2
Tanzania	MWANZA	1980	180.92	124.28	255.81	RW2
Tanzania	MWANZA	1981	180.05	133.47	238.05	RW2
Tanzania	MWANZA	1982	179.22	137.82	229.49	RW2
Tanzania	MWANZA	1983	178.08	138.37	225.58	RW2
Tanzania	MWANZA	1984	177.11	138.80	223.22	RW2
Tanzania	MWANZA	1985	175.96	140.98	217.52	RW2
Tanzania	MWANZA	1986	174.70	143.02	211.36	RW2
Tanzania	MWANZA	1987	173.11	143.68	207.37	RW2
Tanzania	MWANZA	1988	171.47	142.54	205.57	RW2
Tanzania	MWANZA	1989	169.62	140.08	204.22	RW2
Tanzania	MWANZA	1990	167.36	138.40	201.23	RW2
Tanzania	MWANZA	1991	165.53	138.85	196.47	RW2
Tanzania	MWANZA	1992	163.70	138.12	192.96	RW2
Tanzania	MWANZA	1993	161.99	135.88	191.67	RW2
Tanzania	MWANZA	1994	160.12	132.83	190.91	RW2
Tanzania	MWANZA	1995	158.50	132.00	188.84	RW2
Tanzania	MWANZA	1996	155.62	130.76	184.24	RW2
Tanzania	MWANZA	1997	152.09	128.51	178.74	RW2
Tanzania	MWANZA	1998	147.74	123.92	175.00	RW2
Tanzania	MWANZA	1999	142.26	117.92	170.78	RW2
Tanzania	MWANZA	2000	136.12	112.40	163.66	RW2
Tanzania	MWANZA	2001	129.14	107.53	154.47	RW2

Continued on next page

Country	Region	Year	Median	Lower	Upper	Method
Tanzania	MWANZA	2002	121.68	101.33	145.21	RW2
Tanzania	MWANZA	2003	114.06	94.69	137.69	RW2
Tanzania	MWANZA	2004	106.22	86.95	129.44	RW2
Tanzania	MWANZA	2005	98.47	79.87	121.00	RW2
Tanzania	MWANZA	2006	91.30	74.62	111.47	RW2
Tanzania	MWANZA	2007	84.64	69.17	103.28	RW2
Tanzania	MWANZA	2008	78.63	63.44	97.18	RW2
Tanzania	MWANZA	2009	73.17	57.58	92.71	RW2
Tanzania	MWANZA	2010	68.34	52.54	88.57	RW2
Tanzania	MWANZA	2011	63.79	48.65	83.11	RW2
Tanzania	MWANZA	2012	59.56	44.94	78.61	RW2
Tanzania	MWANZA	2013	55.62	40.26	76.12	RW2
Tanzania	MWANZA	2014	51.88	34.46	77.17	RW2
Tanzania	MWANZA	2015	48.33	27.78	82.51	RW2
Tanzania	MWANZA	2016	45.16	22.04	91.15	RW2
Tanzania	MWANZA	2017	41.92	16.98	100.91	RW2
Tanzania	MWANZA	2018	39.36	12.89	114.96	RW2
Tanzania	MWANZA	2019	36.64	9.36	132.40	RW2
Tanzania	PWANI	1980	239.08	169.20	325.17	RW2
Tanzania	PWANI	1981	237.95	180.90	305.53	RW2
Tanzania	PWANI	1982	235.93	185.50	295.14	RW2
Tanzania	PWANI	1983	234.35	185.41	291.05	RW2
Tanzania	PWANI	1984	232.19	184.80	287.90	RW2
Tanzania	PWANI	1985	229.68	186.61	280.05	RW2
Tanzania	PWANI	1986	226.11	187.55	270.61	RW2
Tanzania	PWANI	1987	221.73	185.72	262.94	RW2
Tanzania	PWANI	1988	216.20	180.85	256.61	RW2
Tanzania	PWANI	1989	210.34	174.71	251.60	RW2
Tanzania	PWANI	1990	203.74	169.05	243.02	RW2
Tanzania	PWANI	1991	197.56	165.80	233.35	RW2
Tanzania	PWANI	1992	191.84	162.04	225.83	RW2
Tanzania	PWANI	1993	186.69	156.30	221.24	RW2
Tanzania	PWANI	1994	181.89	150.52	217.67	RW2
Tanzania	PWANI	1995	177.32	146.79	212.27	RW2
Tanzania	PWANI	1996	171.61	142.93	204.07	RW2
Tanzania	PWANI	1997	164.98	137.95	195.69	RW2
Tanzania	PWANI	1998	157.48	130.67	187.95	RW2
Tanzania	PWANI	1999	148.86	122.15	180.04	RW2
Tanzania	PWANI	2000	139.52	114.02	169.22	RW2
Tanzania	PWANI	2001	130.08	106.67	157.30	RW2
Tanzania	PWANI	2002	120.52	99.06	145.75	RW2
Tanzania	PWANI	2003	111.47	90.71	136.94	RW2
Tanzania	PWANI	2004	102.72	82.13	128.13	RW2
Tanzania	PWANI	2005	94.49	74.77	118.85	RW2
Tanzania	PWANI	2006	87.13	68.81	110.01	RW2
Tanzania	PWANI	2007	80.56	63.08	102.69	RW2
Tanzania	PWANI	2008	74.67	57.55	96.77	RW2
Tanzania	PWANI	2009	69.44	51.99	92.43	RW2
Tanzania	PWANI	2010	64.78	47.32	88.45	RW2
Tanzania	PWANI	2011	60.52	43.62	83.71	RW2
Tanzania	PWANI	2012	56.58	40.07	79.60	RW2
Tanzania	PWANI	2013	52.78	35.98	77.27	RW2
Tanzania	PWANI	2014	49.28	31.08	78.04	RW2
Tanzania	PWANI	2015	45.96	25.06	82.81	RW2
Tanzania	PWANI	2016	42.88	20.17	89.51	RW2
Tanzania	PWANI	2017	39.92	15.57	98.86	RW2
Tanzania	PWANI	2018	37.41	11.74	112.91	RW2
Tanzania	PWANI	2019	34.73	8.79	131.48	RW2
Tanzania	RUKWA	1980	249.16	178.40	337.58	RW2
Tanzania	RUKWA	1981	242.17	186.51	309.86	RW2
Tanzania	RUKWA	1982	235.46	186.37	293.69	RW2
Tanzania	RUKWA	1983	228.68	182.48	283.54	RW2
Tanzania	RUKWA	1984	222.30	177.49	274.44	RW2

Continued on next page

Country	Region	Year	Median	Lower	Upper	Method
Tanzania	RUKWA	1985	216.04	176.15	262.46	RW2
Tanzania	RUKWA	1986	210.24	174.80	251.03	RW2
Tanzania	RUKWA	1987	204.51	171.76	241.46	RW2
Tanzania	RUKWA	1988	199.20	167.16	235.60	RW2
Tanzania	RUKWA	1989	194.08	161.82	231.12	RW2
Tanzania	RUKWA	1990	189.18	158.27	224.54	RW2
Tanzania	RUKWA	1991	184.86	156.70	217.34	RW2
Tanzania	RUKWA	1992	180.81	153.96	210.91	RW2
Tanzania	RUKWA	1993	176.78	149.30	208.03	RW2
Tanzania	RUKWA	1994	172.64	143.94	205.57	RW2
Tanzania	RUKWA	1995	168.49	140.35	201.46	RW2
Tanzania	RUKWA	1996	163.19	137.10	193.53	RW2
Tanzania	RUKWA	1997	156.88	131.82	185.03	RW2
Tanzania	RUKWA	1998	149.55	124.54	178.01	RW2
Tanzania	RUKWA	1999	141.45	116.78	170.16	RW2
Tanzania	RUKWA	2000	132.68	108.95	160.03	RW2
Tanzania	RUKWA	2001	124.08	102.86	148.32	RW2
Tanzania	RUKWA	2002	115.62	96.44	138.12	RW2
Tanzania	RUKWA	2003	107.62	89.14	129.23	RW2
Tanzania	RUKWA	2004	100.33	81.91	122.34	RW2
Tanzania	RUKWA	2005	93.58	75.98	114.22	RW2
Tanzania	RUKWA	2006	87.78	71.86	106.55	RW2
Tanzania	RUKWA	2007	82.71	67.98	100.10	RW2
Tanzania	RUKWA	2008	78.53	63.80	96.20	RW2
Tanzania	RUKWA	2009	74.85	59.69	93.32	RW2
Tanzania	RUKWA	2010	71.86	56.27	91.55	RW2
Tanzania	RUKWA	2011	69.09	54.10	88.18	RW2
Tanzania	RUKWA	2012	66.50	52.01	85.05	RW2
Tanzania	RUKWA	2013	64.11	48.49	85.03	RW2
Tanzania	RUKWA	2014	61.79	42.86	89.32	RW2
Tanzania	RUKWA	2015	59.55	35.40	98.72	RW2
Tanzania	RUKWA	2016	57.23	28.85	111.69	RW2
Tanzania	RUKWA	2017	55.10	23.03	127.72	RW2
Tanzania	RUKWA	2018	52.92	17.95	149.68	RW2
Tanzania	RUKWA	2019	51.22	13.66	174.66	RW2
Tanzania	RUVUMA	1980	143.80	98.63	205.28	RW2
Tanzania	RUVUMA	1981	145.52	107.07	194.20	RW2
Tanzania	RUVUMA	1982	147.43	112.38	190.37	RW2
Tanzania	RUVUMA	1983	149.27	114.74	191.85	RW2
Tanzania	RUVUMA	1984	151.01	116.72	193.18	RW2
Tanzania	RUVUMA	1985	153.14	120.84	191.90	RW2
Tanzania	RUVUMA	1986	154.96	124.97	190.62	RW2
Tanzania	RUVUMA	1987	157.05	128.25	190.71	RW2
Tanzania	RUVUMA	1988	159.08	130.03	193.26	RW2
Tanzania	RUVUMA	1989	161.05	131.06	196.50	RW2
Tanzania	RUVUMA	1990	162.89	133.13	197.95	RW2
Tanzania	RUVUMA	1991	164.95	136.44	198.05	RW2
Tanzania	RUVUMA	1992	166.73	138.63	198.96	RW2
Tanzania	RUVUMA	1993	168.02	139.24	201.23	RW2
Tanzania	RUVUMA	1994	168.58	138.55	203.32	RW2
Tanzania	RUVUMA	1995	168.47	139.13	204.00	RW2
Tanzania	RUVUMA	1996	166.47	138.54	199.64	RW2
Tanzania	RUVUMA	1997	162.34	135.54	193.73	RW2
Tanzania	RUVUMA	1998	156.57	129.89	188.25	RW2
Tanzania	RUVUMA	1999	149.04	121.90	180.71	RW2
Tanzania	RUVUMA	2000	139.88	114.27	169.90	RW2
Tanzania	RUVUMA	2001	130.30	107.00	157.79	RW2
Tanzania	RUVUMA	2002	120.22	98.87	145.64	RW2
Tanzania	RUVUMA	2003	110.26	89.74	135.04	RW2
Tanzania	RUVUMA	2004	100.76	80.86	125.29	RW2
Tanzania	RUVUMA	2005	91.65	72.48	114.96	RW2
Tanzania	RUVUMA	2006	83.51	65.86	105.11	RW2
Tanzania	RUVUMA	2007	76.18	59.79	96.59	RW2

Continued on next page

Country	Region	Year	Median	Lower	Upper	Method
Tanzania	RUVUMA	2008	69.64	53.57	90.22	RW2
Tanzania	RUVUMA	2009	63.81	47.63	84.82	RW2
Tanzania	RUVUMA	2010	58.77	42.64	80.40	RW2
Tanzania	RUVUMA	2011	54.05	38.43	75.14	RW2
Tanzania	RUVUMA	2012	49.75	34.62	70.56	RW2
Tanzania	RUVUMA	2013	45.82	30.47	67.98	RW2
Tanzania	RUVUMA	2014	42.15	25.74	67.61	RW2
Tanzania	RUVUMA	2015	38.86	20.73	71.53	RW2
Tanzania	RUVUMA	2016	35.75	16.15	76.24	RW2
Tanzania	RUVUMA	2017	32.77	12.45	83.56	RW2
Tanzania	RUVUMA	2018	30.12	9.21	94.94	RW2
Tanzania	RUVUMA	2019	27.74	6.75	105.99	RW2
Tanzania	SHINYANGA	1980	193.83	142.55	259.36	RW2
Tanzania	SHINYANGA	1981	190.85	150.53	239.73	RW2
Tanzania	SHINYANGA	1982	188.38	152.08	231.02	RW2
Tanzania	SHINYANGA	1983	185.73	149.85	228.28	RW2
Tanzania	SHINYANGA	1984	182.89	147.21	225.29	RW2
Tanzania	SHINYANGA	1985	180.59	147.57	218.90	RW2
Tanzania	SHINYANGA	1986	178.04	147.99	212.14	RW2
Tanzania	SHINYANGA	1987	175.41	147.85	207.38	RW2
Tanzania	SHINYANGA	1988	173.07	145.08	205.37	RW2
Tanzania	SHINYANGA	1989	170.70	142.25	204.03	RW2
Tanzania	SHINYANGA	1990	168.38	140.39	200.58	RW2
Tanzania	SHINYANGA	1991	166.55	140.83	195.90	RW2
Tanzania	SHINYANGA	1992	164.97	140.55	192.69	RW2
Tanzania	SHINYANGA	1993	163.68	138.77	191.84	RW2
Tanzania	SHINYANGA	1994	162.23	135.83	192.17	RW2
Tanzania	SHINYANGA	1995	160.81	135.02	190.80	RW2
Tanzania	SHINYANGA	1996	158.37	134.16	186.05	RW2
Tanzania	SHINYANGA	1997	155.11	132.09	180.90	RW2
Tanzania	SHINYANGA	1998	150.79	127.50	176.81	RW2
Tanzania	SHINYANGA	1999	145.32	121.53	172.62	RW2
Tanzania	SHINYANGA	2000	138.78	115.94	164.99	RW2
Tanzania	SHINYANGA	2001	132.03	110.87	155.48	RW2
Tanzania	SHINYANGA	2002	124.63	105.52	146.89	RW2
Tanzania	SHINYANGA	2003	117.21	98.46	139.09	RW2
Tanzania	SHINYANGA	2004	109.76	91.13	132.10	RW2
Tanzania	SHINYANGA	2005	102.53	84.54	123.57	RW2
Tanzania	SHINYANGA	2006	95.87	79.92	114.82	RW2
Tanzania	SHINYANGA	2007	89.83	75.06	107.21	RW2
Tanzania	SHINYANGA	2008	84.35	69.79	101.70	RW2
Tanzania	SHINYANGA	2009	79.46	64.54	97.92	RW2
Tanzania	SHINYANGA	2010	75.16	60.00	94.03	RW2
Tanzania	SHINYANGA	2011	71.09	57.26	88.27	RW2
Tanzania	SHINYANGA	2012	67.31	54.50	82.95	RW2
Tanzania	SHINYANGA	2013	63.73	50.20	80.50	RW2
Tanzania	SHINYANGA	2014	60.32	43.75	82.56	RW2
Tanzania	SHINYANGA	2015	57.10	35.43	90.48	RW2
Tanzania	SHINYANGA	2016	54.00	28.54	100.08	RW2
Tanzania	SHINYANGA	2017	51.00	22.26	113.15	RW2
Tanzania	SHINYANGA	2018	48.09	17.01	128.35	RW2
Tanzania	SHINYANGA	2019	45.53	12.85	149.81	RW2
Tanzania	SINGIDA	1980	160.05	110.47	226.23	RW2
Tanzania	SINGIDA	1981	157.09	116.13	209.36	RW2
Tanzania	SINGIDA	1982	154.03	117.04	200.73	RW2
Tanzania	SINGIDA	1983	151.13	115.27	194.76	RW2
Tanzania	SINGIDA	1984	148.22	113.79	190.84	RW2
Tanzania	SINGIDA	1985	145.64	113.95	184.07	RW2
Tanzania	SINGIDA	1986	142.90	114.11	177.27	RW2
Tanzania	SINGIDA	1987	140.44	113.86	171.96	RW2
Tanzania	SINGIDA	1988	138.20	112.10	169.25	RW2
Tanzania	SINGIDA	1989	136.06	110.24	167.33	RW2
Tanzania	SINGIDA	1990	133.96	109.08	163.45	RW2

Continued on next page

Country	Region	Year	Median	Lower	Upper	Method
Tanzania	SINGIDA	1991	132.12	109.02	159.12	RW2
Tanzania	SINGIDA	1992	130.46	108.70	156.07	RW2
Tanzania	SINGIDA	1993	128.76	106.64	154.37	RW2
Tanzania	SINGIDA	1994	126.78	103.90	153.58	RW2
Tanzania	SINGIDA	1995	124.72	102.17	151.72	RW2
Tanzania	SINGIDA	1996	121.46	100.45	146.70	RW2
Tanzania	SINGIDA	1997	117.30	97.10	141.11	RW2
Tanzania	SINGIDA	1998	112.11	92.11	135.91	RW2
Tanzania	SINGIDA	1999	106.16	86.05	130.65	RW2
Tanzania	SINGIDA	2000	99.26	79.99	122.49	RW2
Tanzania	SINGIDA	2001	92.23	74.77	113.40	RW2
Tanzania	SINGIDA	2002	85.09	68.92	104.64	RW2
Tanzania	SINGIDA	2003	78.14	62.60	97.17	RW2
Tanzania	SINGIDA	2004	71.39	56.47	90.52	RW2
Tanzania	SINGIDA	2005	65.04	50.73	83.04	RW2
Tanzania	SINGIDA	2006	59.30	46.19	75.70	RW2
Tanzania	SINGIDA	2007	54.20	41.98	69.54	RW2
Tanzania	SINGIDA	2008	49.58	37.76	64.81	RW2
Tanzania	SINGIDA	2009	45.44	33.60	61.00	RW2
Tanzania	SINGIDA	2010	41.88	30.21	57.78	RW2
Tanzania	SINGIDA	2011	38.56	27.47	53.77	RW2
Tanzania	SINGIDA	2012	35.48	24.89	50.19	RW2
Tanzania	SINGIDA	2013	32.72	22.05	47.83	RW2
Tanzania	SINGIDA	2014	30.19	18.78	47.48	RW2
Tanzania	SINGIDA	2015	27.68	15.13	49.91	RW2
Tanzania	SINGIDA	2016	25.54	11.94	53.41	RW2
Tanzania	SINGIDA	2017	23.55	9.26	58.71	RW2
Tanzania	SINGIDA	2018	21.55	6.80	65.69	RW2
Tanzania	SINGIDA	2019	19.87	5.05	76.31	RW2
Tanzania	TABORA	1980	178.54	124.70	248.35	RW2
Tanzania	TABORA	1981	176.32	131.79	231.09	RW2
Tanzania	TABORA	1982	174.06	133.95	223.10	RW2
Tanzania	TABORA	1983	171.98	133.28	218.98	RW2
Tanzania	TABORA	1984	170.01	131.67	216.00	RW2
Tanzania	TABORA	1985	168.32	132.90	209.94	RW2
Tanzania	TABORA	1986	166.63	134.24	203.91	RW2
Tanzania	TABORA	1987	165.06	134.87	200.08	RW2
Tanzania	TABORA	1988	163.64	134.15	197.75	RW2
Tanzania	TABORA	1989	162.52	132.63	197.82	RW2
Tanzania	TABORA	1990	161.11	132.32	194.81	RW2
Tanzania	TABORA	1991	160.42	133.86	191.23	RW2
Tanzania	TABORA	1992	159.69	134.21	188.89	RW2
Tanzania	TABORA	1993	158.79	133.16	188.36	RW2
Tanzania	TABORA	1994	157.48	130.87	187.73	RW2
Tanzania	TABORA	1995	156.07	130.19	186.07	RW2
Tanzania	TABORA	1996	152.87	129.04	180.67	RW2
Tanzania	TABORA	1997	148.61	125.95	174.21	RW2
Tanzania	TABORA	1998	142.97	120.58	168.95	RW2
Tanzania	TABORA	1999	136.14	113.35	162.94	RW2
Tanzania	TABORA	2000	128.24	106.24	153.52	RW2
Tanzania	TABORA	2001	119.92	100.20	142.70	RW2
Tanzania	TABORA	2002	111.45	93.62	132.50	RW2
Tanzania	TABORA	2003	103.08	85.85	123.68	RW2
Tanzania	TABORA	2004	95.10	78.01	116.15	RW2
Tanzania	TABORA	2005	87.53	71.24	106.91	RW2
Tanzania	TABORA	2006	80.72	66.05	98.12	RW2
Tanzania	TABORA	2007	74.64	60.97	90.89	RW2
Tanzania	TABORA	2008	69.17	55.79	85.17	RW2
Tanzania	TABORA	2009	64.39	50.81	81.17	RW2
Tanzania	TABORA	2010	60.18	46.42	77.87	RW2
Tanzania	TABORA	2011	56.28	43.27	72.82	RW2
Tanzania	TABORA	2012	52.73	40.21	68.62	RW2
Tanzania	TABORA	2013	49.34	36.46	66.59	RW2

Continued on next page

Country	Region	Year	Median	Lower	Upper	Method
Tanzania	TABORA	2014	46.26	31.44	67.27	RW2
Tanzania	TABORA	2015	43.20	25.50	72.62	RW2
Tanzania	TABORA	2016	40.48	20.27	79.29	RW2
Tanzania	TABORA	2017	37.76	15.62	88.47	RW2
Tanzania	TABORA	2018	35.42	11.86	101.04	RW2
Tanzania	TABORA	2019	33.10	8.70	117.06	RW2
Tanzania	TANGA	1980	177.53	124.21	244.39	RW2
Tanzania	TANGA	1981	179.03	134.99	231.43	RW2
Tanzania	TANGA	1982	180.27	140.69	225.96	RW2
Tanzania	TANGA	1983	181.49	143.13	226.83	RW2
Tanzania	TANGA	1984	182.54	144.54	227.59	RW2
Tanzania	TANGA	1985	183.04	147.85	223.65	RW2
Tanzania	TANGA	1986	183.14	151.22	220.23	RW2
Tanzania	TANGA	1987	182.44	152.50	217.34	RW2
Tanzania	TANGA	1988	181.12	151.05	215.83	RW2
Tanzania	TANGA	1989	179.23	148.96	215.28	RW2
Tanzania	TANGA	1990	176.48	147.14	210.61	RW2
Tanzania	TANGA	1991	173.96	146.75	205.27	RW2
Tanzania	TANGA	1992	171.19	145.38	200.96	RW2
Tanzania	TANGA	1993	168.41	141.79	198.85	RW2
Tanzania	TANGA	1994	165.32	137.46	196.79	RW2
Tanzania	TANGA	1995	162.32	135.08	194.08	RW2
Tanzania	TANGA	1996	157.88	132.36	187.75	RW2
Tanzania	TANGA	1997	152.45	127.51	180.43	RW2
Tanzania	TANGA	1998	145.91	121.64	174.20	RW2
Tanzania	TANGA	1999	138.39	113.76	167.31	RW2
Tanzania	TANGA	2000	129.90	106.31	157.81	RW2
Tanzania	TANGA	2001	121.10	99.60	146.65	RW2
Tanzania	TANGA	2002	112.02	92.40	135.42	RW2
Tanzania	TANGA	2003	102.92	83.92	126.10	RW2
Tanzania	TANGA	2004	94.26	75.53	117.07	RW2
Tanzania	TANGA	2005	85.86	67.95	107.69	RW2
Tanzania	TANGA	2006	78.29	61.98	98.19	RW2
Tanzania	TANGA	2007	71.42	56.22	90.37	RW2
Tanzania	TANGA	2008	65.20	50.27	84.20	RW2
Tanzania	TANGA	2009	59.72	44.75	79.15	RW2
Tanzania	TANGA	2010	54.78	39.73	74.90	RW2
Tanzania	TANGA	2011	50.29	35.91	69.57	RW2
Tanzania	TANGA	2012	46.20	32.24	65.30	RW2
Tanzania	TANGA	2013	42.38	28.24	62.41	RW2
Tanzania	TANGA	2014	38.91	23.86	62.11	RW2
Tanzania	TANGA	2015	35.71	19.11	64.77	RW2
Tanzania	TANGA	2016	32.79	15.02	69.84	RW2
Tanzania	TANGA	2017	29.95	11.41	76.40	RW2
Tanzania	TANGA	2018	27.49	8.48	85.84	RW2
Tanzania	TANGA	2019	25.24	6.19	97.46	RW2
Togo	ALL	1980	168.86	162.69	175.00	IHME
Togo	ALL	1980	178.01	133.38	233.24	RW2
Togo	ALL	1980	177.50	164.20	191.70	UN
Togo	ALL	1981	165.07	159.14	171.14	IHME
Togo	ALL	1981	173.86	141.43	211.38	RW2
Togo	ALL	1981	173.40	160.40	187.20	UN
Togo	ALL	1982	161.80	156.03	167.68	IHME
Togo	ALL	1982	169.82	141.27	202.86	RW2
Togo	ALL	1982	169.50	156.90	182.90	UN
Togo	ALL	1983	159.37	153.97	165.06	IHME
Togo	ALL	1983	165.69	135.56	201.28	RW2
Togo	ALL	1983	165.70	153.50	178.90	UN
Togo	ALL	1984	156.95	151.61	162.47	IHME
Togo	ALL	1984	162.10	129.83	200.49	RW2
Togo	ALL	1984	162.30	150.40	175.10	UN
Togo	ALL	1985	154.67	149.39	160.01	IHME
Togo	ALL	1985	158.19	128.66	192.87	RW2

Continued on next page

Country	Region	Year	Median	Lower	Upper	Method
Togo	ALL	1985	159.00	147.40	171.50	UN
Togo	ALL	1986	152.55	147.19	157.84	IHME
Togo	ALL	1986	155.08	127.86	186.65	RW2
Togo	ALL	1986	156.10	144.60	168.00	UN
Togo	ALL	1987	150.77	145.59	155.72	IHME
Togo	ALL	1987	152.43	126.84	182.40	RW2
Togo	ALL	1987	153.30	142.20	164.90	UN
Togo	ALL	1988	148.78	143.68	153.92	IHME
Togo	ALL	1988	150.07	123.75	180.48	RW2
Togo	ALL	1988	150.80	139.90	162.00	UN
Togo	ALL	1989	146.74	141.71	151.88	IHME
Togo	ALL	1989	148.05	120.88	179.64	RW2
Togo	ALL	1989	148.50	138.00	159.30	UN
Togo	ALL	1990	144.53	139.63	149.59	IHME
Togo	ALL	1990	146.49	120.22	177.97	RW2
Togo	ALL	1990	146.20	136.20	156.90	UN
Togo	ALL	1991	142.41	137.40	147.48	IHME
Togo	ALL	1991	144.66	119.99	173.23	RW2
Togo	ALL	1991	144.10	134.40	154.60	UN
Togo	ALL	1992	140.40	135.47	145.46	IHME
Togo	ALL	1992	142.71	118.84	170.12	RW2
Togo	ALL	1992	142.10	132.60	152.50	UN
Togo	ALL	1993	139.14	134.04	144.22	IHME
Togo	ALL	1993	140.57	116.47	168.65	RW2
Togo	ALL	1993	140.00	130.70	150.30	UN
Togo	ALL	1994	137.39	132.30	142.55	IHME
Togo	ALL	1994	138.12	113.04	168.24	RW2
Togo	ALL	1994	137.70	128.60	147.90	UN
Togo	ALL	1995	135.15	130.09	140.09	IHME
Togo	ALL	1995	135.46	110.67	164.56	RW2
Togo	ALL	1995	135.40	126.20	145.40	UN
Togo	ALL	1996	132.73	127.48	137.77	IHME
Togo	ALL	1996	132.63	109.39	160.26	RW2
Togo	ALL	1996	132.70	123.80	142.70	UN
Togo	ALL	1997	130.07	124.91	135.17	IHME
Togo	ALL	1997	129.71	107.59	155.66	RW2
Togo	ALL	1997	129.90	121.20	139.70	UN
Togo	ALL	1998	127.76	122.62	132.94	IHME
Togo	ALL	1998	126.78	104.48	153.66	RW2
Togo	ALL	1998	127.00	118.40	136.50	UN
Togo	ALL	1999	125.33	120.11	130.55	IHME
Togo	ALL	1999	123.74	100.63	150.95	RW2
Togo	ALL	1999	123.90	115.40	133.10	UN
Togo	ALL	2000	123.00	117.62	128.38	IHME
Togo	ALL	2000	120.65	97.82	147.81	RW2
Togo	ALL	2000	120.80	112.40	129.80	UN
Togo	ALL	2001	120.68	115.41	125.84	IHME
Togo	ALL	2001	117.55	95.79	143.37	RW2
Togo	ALL	2001	117.60	109.30	126.50	UN
Togo	ALL	2002	118.22	112.94	123.54	IHME
Togo	ALL	2002	114.39	93.70	139.03	RW2
Togo	ALL	2002	114.40	106.20	123.20	UN
Togo	ALL	2003	115.60	110.27	120.83	IHME
Togo	ALL	2003	111.31	90.86	135.74	RW2
Togo	ALL	2003	111.20	103.10	120.00	UN
Togo	ALL	2004	112.94	107.66	118.19	IHME
Togo	ALL	2004	108.04	87.03	133.23	RW2
Togo	ALL	2004	108.10	99.90	116.90	UN
Togo	ALL	2005	110.45	105.11	115.73	IHME
Togo	ALL	2005	105.00	84.46	129.82	RW2
Togo	ALL	2005	105.10	96.80	113.90	UN
Togo	ALL	2006	107.33	101.97	112.75	IHME
Togo	ALL	2006	101.92	82.72	124.81	RW2

Continued on next page

Country	Region	Year	Median	Lower	Upper	Method
Togo	ALL	2006	102.10	93.70	111.10	UN
Togo	ALL	2007	104.38	99.02	110.06	IHME
Togo	ALL	2007	98.97	80.80	120.62	RW2
Togo	ALL	2007	99.20	90.70	108.40	UN
Togo	ALL	2008	101.33	95.85	107.02	IHME
Togo	ALL	2008	96.23	77.93	118.31	RW2
Togo	ALL	2008	96.40	87.70	105.80	UN
Togo	ALL	2009	98.22	92.54	104.23	IHME
Togo	ALL	2009	93.38	74.48	116.79	RW2
Togo	ALL	2009	93.60	84.50	103.40	UN
Togo	ALL	2010	95.02	89.31	101.46	IHME
Togo	ALL	2010	90.71	71.56	114.96	RW2
Togo	ALL	2010	90.90	81.40	101.20	UN
Togo	ALL	2011	91.41	85.36	98.29	IHME
Togo	ALL	2011	88.18	70.27	110.14	RW2
Togo	ALL	2011	88.30	78.30	99.10	UN
Togo	ALL	2012	87.89	81.60	95.11	IHME
Togo	ALL	2012	85.66	69.02	105.78	RW2
Togo	ALL	2012	85.80	75.20	97.20	UN
Togo	ALL	2013	84.63	78.21	92.40	IHME
Togo	ALL	2013	83.27	65.34	105.40	RW2
Togo	ALL	2013	83.30	71.80	95.40	UN
Togo	ALL	2014	81.42	74.85	89.39	IHME
Togo	ALL	2014	80.87	57.69	112.15	RW2
Togo	ALL	2014	80.80	68.60	94.30	UN
Togo	ALL	2015	78.04	71.17	86.48	IHME
Togo	ALL	2015	78.43	47.20	127.72	RW2
Togo	ALL	2015	78.40	65.10	93.90	UN
Togo	ALL	2016	76.34	38.53	146.59	RW2
Togo	ALL	2017	73.96	30.34	170.90	RW2
Togo	ALL	2018	71.78	23.63	202.99	RW2
Togo	ALL	2019	69.58	17.37	238.60	RW2
Togo	ALL	15-19	73.96	30.80	168.21	RW2
Togo	CENTRALE	1980	193.68	145.29	254.56	RW2
Togo	CENTRALE	1981	187.27	150.27	231.88	RW2
Togo	CENTRALE	1982	180.74	148.40	219.03	RW2
Togo	CENTRALE	1983	174.62	142.49	212.98	RW2
Togo	CENTRALE	1984	168.92	136.46	207.62	RW2
Togo	CENTRALE	1985	163.51	134.09	197.81	RW2
Togo	CENTRALE	1986	159.33	132.81	189.57	RW2
Togo	CENTRALE	1987	156.01	131.35	184.24	RW2
Togo	CENTRALE	1988	153.56	128.62	181.90	RW2
Togo	CENTRALE	1989	151.74	126.09	180.75	RW2
Togo	CENTRALE	1990	150.78	126.06	179.87	RW2
Togo	CENTRALE	1991	149.32	126.23	176.01	RW2
Togo	CENTRALE	1992	147.43	124.95	172.89	RW2
Togo	CENTRALE	1993	145.03	122.28	171.11	RW2
Togo	CENTRALE	1994	142.50	118.56	169.99	RW2
Togo	CENTRALE	1995	139.19	115.16	166.23	RW2
Togo	CENTRALE	1996	136.66	113.97	162.03	RW2
Togo	CENTRALE	1997	134.42	112.71	159.14	RW2
Togo	CENTRALE	1998	132.66	110.88	157.69	RW2
Togo	CENTRALE	1999	131.29	107.88	157.64	RW2
Togo	CENTRALE	2000	130.39	107.00	157.81	RW2
Togo	CENTRALE	2001	129.23	106.60	155.56	RW2
Togo	CENTRALE	2002	127.78	105.82	153.48	RW2
Togo	CENTRALE	2003	125.91	103.88	152.22	RW2
Togo	CENTRALE	2004	123.79	101.07	150.84	RW2
Togo	CENTRALE	2005	121.16	98.82	147.71	RW2
Togo	CENTRALE	2006	118.65	97.60	143.59	RW2
Togo	CENTRALE	2007	116.02	95.32	140.18	RW2
Togo	CENTRALE	2008	113.52	92.27	138.75	RW2
Togo	CENTRALE	2009	110.96	88.78	137.78	RW2

Continued on next page

Country	Region	Year	Median	Lower	Upper	Method
Togo	CENTRALE	2010	108.69	85.97	136.84	RW2
Togo	CENTRALE	2011	106.33	84.56	133.31	RW2
Togo	CENTRALE	2012	104.10	83.13	130.09	RW2
Togo	CENTRALE	2013	101.94	79.19	131.24	RW2
Togo	CENTRALE	2014	99.84	71.52	138.14	RW2
Togo	CENTRALE	2015	97.73	60.63	154.24	RW2
Togo	CENTRALE	2016	95.51	50.61	173.52	RW2
Togo	CENTRALE	2017	93.60	41.31	199.46	RW2
Togo	CENTRALE	2018	91.35	32.76	232.16	RW2
Togo	CENTRALE	2019	89.44	25.55	273.47	RW2
Togo	GRANDE AGGLOMÉRATION DE LOMÉ	1980	131.08	89.57	186.95	RW2
Togo	GRANDE AGGLOMÉRATION DE LOMÉ	1981	126.88	93.13	169.14	RW2
Togo	GRANDE AGGLOMÉRATION DE LOMÉ	1982	122.84	93.10	158.92	RW2
Togo	GRANDE AGGLOMÉRATION DE LOMÉ	1983	118.79	90.93	153.79	RW2
Togo	GRANDE AGGLOMÉRATION DE LOMÉ	1984	115.29	88.56	148.74	RW2
Togo	GRANDE AGGLOMÉRATION DE LOMÉ	1985	111.83	87.11	141.43	RW2
Togo	GRANDE AGGLOMÉRATION DE LOMÉ	1986	109.17	87.06	136.12	RW2
Togo	GRANDE AGGLOMÉRATION DE LOMÉ	1987	107.29	86.74	131.81	RW2
Togo	GRANDE AGGLOMÉRATION DE LOMÉ	1988	105.90	85.60	129.99	RW2
Togo	GRANDE AGGLOMÉRATION DE LOMÉ	1989	104.85	84.32	129.46	RW2
Togo	GRANDE AGGLOMÉRATION DE LOMÉ	1990	104.47	84.62	128.83	RW2
Togo	GRANDE AGGLOMÉRATION DE LOMÉ	1991	103.66	84.89	125.80	RW2
Togo	GRANDE AGGLOMÉRATION DE LOMÉ	1992	102.33	84.44	123.61	RW2
Togo	GRANDE AGGLOMÉRATION DE LOMÉ	1993	100.68	82.93	122.45	RW2
Togo	GRANDE AGGLOMÉRATION DE LOMÉ	1994	98.71	80.29	121.47	RW2
Togo	GRANDE AGGLOMÉRATION DE LOMÉ	1995	95.97	77.64	118.26	RW2
Togo	GRANDE AGGLOMÉRATION DE LOMÉ	1996	93.56	76.48	114.06	RW2
Togo	GRANDE AGGLOMÉRATION DE LOMÉ	1997	91.22	74.99	111.29	RW2
Togo	GRANDE AGGLOMÉRATION DE LOMÉ	1998	89.08	72.80	109.06	RW2
Togo	GRANDE AGGLOMÉRATION DE LOMÉ	1999	86.91	70.19	107.25	RW2
Togo	GRANDE AGGLOMÉRATION DE LOMÉ	2000	85.04	68.62	105.67	RW2
Togo	GRANDE AGGLOMÉRATION DE LOMÉ	2001	82.88	67.10	102.33	RW2
Togo	GRANDE AGGLOMÉRATION DE LOMÉ	2002	80.51	65.34	99.10	RW2
Togo	GRANDE AGGLOMÉRATION DE LOMÉ	2003	77.95	62.78	96.44	RW2
Togo	GRANDE AGGLOMÉRATION DE LOMÉ	2004	75.29	59.90	93.85	RW2
Togo	GRANDE AGGLOMÉRATION DE LOMÉ	2005	72.41	57.37	90.62	RW2
Togo	GRANDE AGGLOMÉRATION DE LOMÉ	2006	69.65	55.66	86.82	RW2
Togo	GRANDE AGGLOMÉRATION DE LOMÉ	2007	67.10	53.64	83.63	RW2
Togo	GRANDE AGGLOMÉRATION DE LOMÉ	2008	64.68	51.04	81.53	RW2
Togo	GRANDE AGGLOMÉRATION DE LOMÉ	2009	62.32	48.35	79.84	RW2
Togo	GRANDE AGGLOMÉRATION DE LOMÉ	2010	60.24	45.98	78.45	RW2
Togo	GRANDE AGGLOMÉRATION DE LOMÉ	2011	58.20	44.54	75.76	RW2
Togo	GRANDE AGGLOMÉRATION DE LOMÉ	2012	56.26	42.86	73.49	RW2
Togo	GRANDE AGGLOMÉRATION DE LOMÉ	2013	54.39	40.08	73.40	RW2
Togo	GRANDE AGGLOMÉRATION DE LOMÉ	2014	52.51	35.51	76.77	RW2
Togo	GRANDE AGGLOMÉRATION DE LOMÉ	2015	50.82	29.75	85.54	RW2
Togo	GRANDE AGGLOMÉRATION DE LOMÉ	2016	49.05	24.35	96.22	RW2
Togo	GRANDE AGGLOMÉRATION DE LOMÉ	2017	47.49	19.54	111.08	RW2
Togo	GRANDE AGGLOMÉRATION DE LOMÉ	2018	45.76	15.34	129.52	RW2
Togo	GRANDE AGGLOMÉRATION DE LOMÉ	2019	44.35	11.87	153.57	RW2
Togo	KARA	1980	209.57	157.50	273.35	RW2
Togo	KARA	1981	203.30	164.33	249.83	RW2
Togo	KARA	1982	197.15	162.69	237.23	RW2
Togo	KARA	1983	191.23	156.68	231.85	RW2
Togo	KARA	1984	185.81	151.41	226.52	RW2
Togo	KARA	1985	180.62	149.15	217.14	RW2
Togo	KARA	1986	176.76	148.24	209.26	RW2
Togo	KARA	1987	173.80	147.19	203.91	RW2
Togo	KARA	1988	171.69	144.80	202.12	RW2
Togo	KARA	1989	170.38	142.60	201.86	RW2

Continued on next page

Country	Region	Year	Median	Lower	Upper	Method
Togo	KARA	1990	169.74	143.06	201.29	RW2
Togo	KARA	1991	168.61	143.21	197.17	RW2
Togo	KARA	1992	166.91	142.63	194.20	RW2
Togo	KARA	1993	164.53	139.93	192.82	RW2
Togo	KARA	1994	161.77	136.02	192.00	RW2
Togo	KARA	1995	158.28	132.44	187.37	RW2
Togo	KARA	1996	155.25	130.80	182.62	RW2
Togo	KARA	1997	152.79	129.67	179.22	RW2
Togo	KARA	1998	150.70	127.09	178.04	RW2
Togo	KARA	1999	149.05	123.84	177.81	RW2
Togo	KARA	2000	147.86	122.45	178.01	RW2
Togo	KARA	2001	146.19	121.88	175.12	RW2
Togo	KARA	2002	144.22	120.39	172.02	RW2
Togo	KARA	2003	141.65	117.60	170.27	RW2
Togo	KARA	2004	138.74	114.16	168.45	RW2
Togo	KARA	2005	135.26	110.91	164.05	RW2
Togo	KARA	2006	131.57	108.72	158.37	RW2
Togo	KARA	2007	127.72	105.84	153.46	RW2
Togo	KARA	2008	123.84	101.47	150.17	RW2
Togo	KARA	2009	120.09	96.92	147.73	RW2
Togo	KARA	2010	116.38	92.77	144.95	RW2
Togo	KARA	2011	112.67	90.37	139.60	RW2
Togo	KARA	2012	109.15	87.83	134.64	RW2
Togo	KARA	2013	105.66	82.98	133.86	RW2
Togo	KARA	2014	102.18	73.87	139.79	RW2
Togo	KARA	2015	98.84	61.63	155.45	RW2
Togo	KARA	2016	95.52	50.45	173.31	RW2
Togo	KARA	2017	92.47	40.66	197.28	RW2
Togo	KARA	2018	89.16	31.82	226.33	RW2
Togo	KARA	2019	86.44	24.41	265.63	RW2
Togo	MARITIME (SANS AGGLOMÉRATION DE LOMÉ)	1980	172.40	126.83	230.25	RW2
Togo	MARITIME (SANS AGGLOMÉRATION DE LOMÉ)	1981	167.04	131.68	209.46	RW2
Togo	MARITIME (SANS AGGLOMÉRATION DE LOMÉ)	1982	161.86	130.60	198.58	RW2
Togo	MARITIME (SANS AGGLOMÉRATION DE LOMÉ)	1983	156.72	125.86	193.55	RW2
Togo	MARITIME (SANS AGGLOMÉRATION DE LOMÉ)	1984	152.09	121.35	189.70	RW2
Togo	MARITIME (SANS AGGLOMÉRATION DE LOMÉ)	1985	147.63	119.15	181.04	RW2
Togo	MARITIME (SANS AGGLOMÉRATION DE LOMÉ)	1986	144.21	118.60	174.23	RW2
Togo	MARITIME (SANS AGGLOMÉRATION DE LOMÉ)	1987	141.55	117.38	169.54	RW2
Togo	MARITIME (SANS AGGLOMÉRATION DE LOMÉ)	1988	139.66	115.62	167.29	RW2
Togo	MARITIME (SANS AGGLOMÉRATION DE LOMÉ)	1989	138.23	113.57	166.40	RW2
Togo	MARITIME (SANS AGGLOMÉRATION DE LOMÉ)	1990	137.57	113.87	165.18	RW2
Togo	MARITIME (SANS AGGLOMÉRATION DE LOMÉ)	1991	136.24	114.09	161.95	RW2
Togo	MARITIME (SANS AGGLOMÉRATION DE LOMÉ)	1992	134.42	113.56	159.05	RW2
Togo	MARITIME (SANS AGGLOMÉRATION DE LOMÉ)	1993	132.24	111.24	157.27	RW2
Togo	MARITIME (SANS AGGLOMÉRATION DE LOMÉ)	1994	129.41	107.14	155.41	RW2
Togo	MARITIME (SANS AGGLOMÉRATION DE LOMÉ)	1995	126.05	103.92	151.50	RW2
Togo	MARITIME (SANS AGGLOMÉRATION DE LOMÉ)	1996	123.04	102.34	147.31	RW2
Togo	MARITIME (SANS AGGLOMÉRATION DE LOMÉ)	1997	120.03	100.33	143.21	RW2
Togo	MARITIME (SANS AGGLOMÉRATION DE LOMÉ)	1998	117.45	97.18	141.12	RW2
Togo	MARITIME (SANS AGGLOMÉRATION DE LOMÉ)	1999	114.83	93.58	139.55	RW2
Togo	MARITIME (SANS AGGLOMÉRATION DE LOMÉ)	2000	112.63	91.48	138.13	RW2
Togo	MARITIME (SANS AGGLOMÉRATION DE LOMÉ)	2001	110.00	89.40	135.01	RW2
Togo	MARITIME (SANS AGGLOMÉRATION DE LOMÉ)	2002	107.37	86.99	131.77	RW2
Togo	MARITIME (SANS AGGLOMÉRATION DE LOMÉ)	2003	104.27	83.46	129.09	RW2
Togo	MARITIME (SANS AGGLOMÉRATION DE LOMÉ)	2004	101.18	79.92	127.03	RW2
Togo	MARITIME (SANS AGGLOMÉRATION DE LOMÉ)	2005	97.79	76.78	123.45	RW2
Togo	MARITIME (SANS AGGLOMÉRATION DE LOMÉ)	2006	94.49	74.23	119.19	RW2
Togo	MARITIME (SANS AGGLOMÉRATION DE LOMÉ)	2007	91.32	71.49	115.39	RW2
Togo	MARITIME (SANS AGGLOMÉRATION DE LOMÉ)	2008	88.50	68.39	113.23	RW2
Togo	MARITIME (SANS AGGLOMÉRATION DE LOMÉ)	2009	85.71	65.00	111.97	RW2

Continued on next page

Country	Region	Year	Median	Lower	Upper	Method
Togo	MARITIME (SANS AGGLOMÉRATION DE LOMÉ)	2010	83.19	62.39	110.31	RW2
Togo	MARITIME (SANS AGGLOMÉRATION DE LOMÉ)	2011	80.59	60.24	107.09	RW2
Togo	MARITIME (SANS AGGLOMÉRATION DE LOMÉ)	2012	78.25	58.30	104.24	RW2
Togo	MARITIME (SANS AGGLOMÉRATION DE LOMÉ)	2013	75.98	54.94	104.32	RW2
Togo	MARITIME (SANS AGGLOMÉRATION DE LOMÉ)	2014	73.80	49.44	108.80	RW2
Togo	MARITIME (SANS AGGLOMÉRATION DE LOMÉ)	2015	71.58	41.95	120.43	RW2
Togo	MARITIME (SANS AGGLOMÉRATION DE LOMÉ)	2016	69.48	34.63	134.19	RW2
Togo	MARITIME (SANS AGGLOMÉRATION DE LOMÉ)	2017	67.36	28.01	153.65	RW2
Togo	MARITIME (SANS AGGLOMÉRATION DE LOMÉ)	2018	65.35	22.27	179.43	RW2
Togo	MARITIME (SANS AGGLOMÉRATION DE LOMÉ)	2019	63.47	16.92	209.50	RW2
Togo	PLATEAUX	1980	181.35	137.25	236.49	RW2
Togo	PLATEAUX	1981	175.14	142.76	213.56	RW2
Togo	PLATEAUX	1982	168.94	140.90	202.02	RW2
Togo	PLATEAUX	1983	163.42	134.74	197.03	RW2
Togo	PLATEAUX	1984	157.98	128.81	192.36	RW2
Togo	PLATEAUX	1985	152.97	126.27	183.37	RW2
Togo	PLATEAUX	1986	148.97	125.10	175.98	RW2
Togo	PLATEAUX	1987	145.89	123.48	171.40	RW2
Togo	PLATEAUX	1988	143.56	120.96	168.85	RW2
Togo	PLATEAUX	1989	141.89	118.31	168.91	RW2
Togo	PLATEAUX	1990	141.04	118.11	168.29	RW2
Togo	PLATEAUX	1991	139.50	117.97	164.64	RW2
Togo	PLATEAUX	1992	137.63	116.83	161.37	RW2
Togo	PLATEAUX	1993	135.41	114.27	159.82	RW2
Togo	PLATEAUX	1994	132.71	110.61	158.74	RW2
Togo	PLATEAUX	1995	129.54	107.37	154.62	RW2
Togo	PLATEAUX	1996	126.77	106.12	150.36	RW2
Togo	PLATEAUX	1997	124.30	104.54	146.93	RW2
Togo	PLATEAUX	1998	122.15	102.37	145.06	RW2
Togo	PLATEAUX	1999	120.26	99.29	144.46	RW2
Togo	PLATEAUX	2000	119.00	98.29	143.77	RW2
Togo	PLATEAUX	2001	117.32	97.40	141.07	RW2
Togo	PLATEAUX	2002	115.44	96.10	138.49	RW2
Togo	PLATEAUX	2003	113.25	93.54	136.54	RW2
Togo	PLATEAUX	2004	110.78	90.76	134.93	RW2
Togo	PLATEAUX	2005	108.06	88.08	131.48	RW2
Togo	PLATEAUX	2006	105.29	86.43	127.66	RW2
Togo	PLATEAUX	2007	102.81	84.43	124.29	RW2
Togo	PLATEAUX	2008	100.29	81.49	123.15	RW2
Togo	PLATEAUX	2009	97.83	77.98	122.20	RW2
Togo	PLATEAUX	2010	95.57	75.05	121.17	RW2
Togo	PLATEAUX	2011	93.51	73.76	117.89	RW2
Togo	PLATEAUX	2012	91.40	72.18	115.38	RW2
Togo	PLATEAUX	2013	89.20	68.45	115.84	RW2
Togo	PLATEAUX	2014	87.30	61.86	122.79	RW2
Togo	PLATEAUX	2015	85.28	52.34	136.50	RW2
Togo	PLATEAUX	2016	83.39	43.48	155.55	RW2
Togo	PLATEAUX	2017	81.68	35.38	178.61	RW2
Togo	PLATEAUX	2018	79.85	28.11	207.65	RW2
Togo	PLATEAUX	2019	77.71	21.66	246.58	RW2
Togo	SAVANES	1980	224.65	173.09	286.03	RW2
Togo	SAVANES	1981	217.30	180.51	258.64	RW2
Togo	SAVANES	1982	209.93	177.93	245.83	RW2
Togo	SAVANES	1983	202.84	169.73	240.95	RW2
Togo	SAVANES	1984	196.34	162.60	235.51	RW2
Togo	SAVANES	1985	190.12	158.46	225.41	RW2
Togo	SAVANES	1986	185.42	156.86	217.06	RW2
Togo	SAVANES	1987	181.77	155.29	211.09	RW2
Togo	SAVANES	1988	179.09	151.93	208.92	RW2
Togo	SAVANES	1989	177.13	148.90	208.22	RW2
Togo	SAVANES	1990	176.09	149.31	207.27	RW2
Togo	SAVANES	1991	174.39	149.52	202.96	RW2

Continued on next page

Country	Region	Year	Median	Lower	Upper	Method
Togo	SAVANES	1992	172.15	148.10	199.16	RW2
Togo	SAVANES	1993	169.18	144.97	196.66	RW2
Togo	SAVANES	1994	165.62	139.99	195.35	RW2
Togo	SAVANES	1995	161.23	135.26	190.14	RW2
Togo	SAVANES	1996	157.43	133.05	184.67	RW2
Togo	SAVANES	1997	153.88	130.77	179.90	RW2
Togo	SAVANES	1998	150.66	127.41	177.72	RW2
Togo	SAVANES	1999	147.75	123.20	176.38	RW2
Togo	SAVANES	2000	145.26	120.56	174.12	RW2
Togo	SAVANES	2001	142.25	119.13	169.60	RW2
Togo	SAVANES	2002	138.90	116.49	165.34	RW2
Togo	SAVANES	2003	134.91	112.50	161.62	RW2
Togo	SAVANES	2004	130.56	107.70	157.77	RW2
Togo	SAVANES	2005	125.67	102.94	152.56	RW2
Togo	SAVANES	2006	120.68	99.42	145.76	RW2
Togo	SAVANES	2007	115.57	95.24	139.45	RW2
Togo	SAVANES	2008	110.56	90.04	134.93	RW2
Togo	SAVANES	2009	105.51	84.41	131.07	RW2
Togo	SAVANES	2010	100.82	78.88	127.50	RW2
Togo	SAVANES	2011	96.02	75.25	121.48	RW2
Togo	SAVANES	2012	91.61	71.19	116.15	RW2
Togo	SAVANES	2013	87.24	65.19	114.46	RW2
Togo	SAVANES	2014	83.08	56.96	117.62	RW2
Togo	SAVANES	2015	79.10	46.97	129.28	RW2
Togo	SAVANES	2016	75.23	38.14	142.75	RW2
Togo	SAVANES	2017	71.62	29.98	161.81	RW2
Togo	SAVANES	2018	67.92	22.76	184.72	RW2
Togo	SAVANES	2019	64.51	17.37	212.72	RW2
Uganda	ALL	1980	196.61	190.11	204.38	IHME
Uganda	ALL	1980	216.80	167.12	275.78	RW2
Uganda	ALL	1980	215.60	200.80	232.20	UN
Uganda	ALL	1981	195.38	188.88	202.68	IHME
Uganda	ALL	1981	212.95	177.37	252.82	RW2
Uganda	ALL	1981	214.40	200.00	230.40	UN
Uganda	ALL	1982	193.12	186.63	200.28	IHME
Uganda	ALL	1982	209.18	177.15	245.28	RW2
Uganda	ALL	1982	210.40	197.00	225.20	UN
Uganda	ALL	1983	190.08	183.89	197.44	IHME
Uganda	ALL	1983	205.26	170.44	245.24	RW2
Uganda	ALL	1983	205.00	192.40	218.70	UN
Uganda	ALL	1984	187.00	180.91	193.98	IHME
Uganda	ALL	1984	201.83	164.04	245.49	RW2
Uganda	ALL	1984	199.80	187.40	212.90	UN
Uganda	ALL	1985	184.09	178.15	190.53	IHME
Uganda	ALL	1985	198.01	163.71	237.40	RW2
Uganda	ALL	1985	195.80	183.60	208.20	UN
Uganda	ALL	1986	181.33	175.60	187.38	IHME
Uganda	ALL	1986	194.79	163.37	230.41	RW2
Uganda	ALL	1986	193.20	181.40	205.20	UN
Uganda	ALL	1987	176.29	171.84	180.66	IHME
Uganda	ALL	1987	191.88	162.41	225.78	RW2
Uganda	ALL	1987	191.80	180.60	203.50	UN
Uganda	ALL	1988	173.34	169.27	177.62	IHME
Uganda	ALL	1988	189.11	158.54	223.94	RW2
Uganda	ALL	1988	190.90	180.00	202.50	UN
Uganda	ALL	1989	170.61	166.41	175.00	IHME
Uganda	ALL	1989	186.59	154.70	223.33	RW2
Uganda	ALL	1989	189.50	178.90	201.30	UN
Uganda	ALL	1990	167.93	163.67	172.44	IHME
Uganda	ALL	1990	184.31	152.97	220.82	RW2
Uganda	ALL	1990	187.10	176.50	198.60	UN
Uganda	ALL	1991	165.36	161.24	169.87	IHME
Uganda	ALL	1991	182.01	152.64	215.24	RW2

Continued on next page

Country	Region	Year	Median	Lower	Upper	Method
Uganda	ALL	1991	183.70	173.00	194.80	UN
Uganda	ALL	1992	162.56	158.57	167.23	IHME
Uganda	ALL	1992	179.66	151.28	211.64	RW2
Uganda	ALL	1992	179.60	169.00	190.50	UN
Uganda	ALL	1993	159.61	155.62	164.11	IHME
Uganda	ALL	1993	177.19	148.45	210.01	RW2
Uganda	ALL	1993	175.60	165.10	186.50	UN
Uganda	ALL	1994	156.04	151.87	160.52	IHME
Uganda	ALL	1994	174.40	144.33	209.60	RW2
Uganda	ALL	1994	172.20	162.10	183.10	UN
Uganda	ALL	1995	152.25	148.16	156.58	IHME
Uganda	ALL	1995	171.44	142.18	205.68	RW2
Uganda	ALL	1995	169.50	159.70	180.20	UN
Uganda	ALL	1996	148.42	144.32	152.71	IHME
Uganda	ALL	1996	167.84	140.53	200.05	RW2
Uganda	ALL	1996	166.90	157.20	177.50	UN
Uganda	ALL	1997	144.22	140.11	148.39	IHME
Uganda	ALL	1997	163.73	137.71	193.67	RW2
Uganda	ALL	1997	163.90	154.20	174.20	UN
Uganda	ALL	1998	139.84	135.81	144.00	IHME
Uganda	ALL	1998	159.08	132.75	189.85	RW2
Uganda	ALL	1998	159.90	150.50	170.20	UN
Uganda	ALL	1999	135.34	131.22	139.59	IHME
Uganda	ALL	1999	153.65	126.45	184.49	RW2
Uganda	ALL	1999	154.80	145.50	165.10	UN
Uganda	ALL	2000	130.97	126.88	135.15	IHME
Uganda	ALL	2000	147.67	121.80	177.77	RW2
Uganda	ALL	2000	148.40	139.30	158.40	UN
Uganda	ALL	2001	126.40	122.33	130.48	IHME
Uganda	ALL	2001	140.67	116.88	168.15	RW2
Uganda	ALL	2001	141.00	132.00	150.80	UN
Uganda	ALL	2002	122.05	118.05	125.99	IHME
Uganda	ALL	2002	132.93	111.13	158.31	RW2
Uganda	ALL	2002	132.80	124.20	142.10	UN
Uganda	ALL	2003	117.50	113.59	121.41	IHME
Uganda	ALL	2003	124.74	103.87	149.54	RW2
Uganda	ALL	2003	124.20	116.10	133.20	UN
Uganda	ALL	2004	113.17	109.18	117.19	IHME
Uganda	ALL	2004	116.16	95.17	141.53	RW2
Uganda	ALL	2004	115.60	107.80	124.10	UN
Uganda	ALL	2005	108.73	104.64	112.93	IHME
Uganda	ALL	2005	107.48	87.12	131.14	RW2
Uganda	ALL	2005	107.40	99.70	115.40	UN
Uganda	ALL	2006	104.56	100.19	108.77	IHME
Uganda	ALL	2006	99.58	81.61	120.44	RW2
Uganda	ALL	2006	99.70	92.10	107.60	UN
Uganda	ALL	2007	100.39	95.71	104.82	IHME
Uganda	ALL	2007	92.36	76.33	111.23	RW2
Uganda	ALL	2007	92.50	85.00	100.30	UN
Uganda	ALL	2008	96.12	91.36	100.94	IHME
Uganda	ALL	2008	85.97	70.44	104.62	RW2
Uganda	ALL	2008	85.90	78.30	94.00	UN
Uganda	ALL	2009	92.13	87.12	97.14	IHME
Uganda	ALL	2009	80.10	64.31	99.59	RW2
Uganda	ALL	2009	80.10	72.20	88.90	UN
Uganda	ALL	2010	88.77	83.45	94.47	IHME
Uganda	ALL	2010	75.08	59.22	95.79	RW2
Uganda	ALL	2010	75.20	66.30	85.10	UN
Uganda	ALL	2011	84.67	79.15	90.89	IHME
Uganda	ALL	2011	70.37	55.52	89.24	RW2
Uganda	ALL	2011	69.80	59.80	81.30	UN
Uganda	ALL	2012	79.97	74.11	86.63	IHME
Uganda	ALL	2012	65.98	51.93	83.64	RW2

Continued on next page

Country	Region	Year	Median	Lower	Upper	Method
Uganda	ALL	2012	64.10	53.20	77.30	UN
Uganda	ALL	2013	76.14	69.99	83.44	IHME
Uganda	ALL	2013	61.93	46.89	81.31	RW2
Uganda	ALL	2013	60.30	48.40	75.80	UN
Uganda	ALL	2014	72.64	66.31	80.34	IHME
Uganda	ALL	2014	58.04	39.88	83.88	RW2
Uganda	ALL	2014	56.90	44.10	74.30	UN
Uganda	ALL	2015	69.35	62.82	77.42	IHME
Uganda	ALL	2015	54.28	31.54	92.38	RW2
Uganda	ALL	2015	54.60	40.60	74.20	UN
Uganda	ALL	2016	50.97	24.85	103.19	RW2
Uganda	ALL	2017	47.61	18.87	117.47	RW2
Uganda	ALL	2018	44.56	14.17	136.90	RW2
Uganda	ALL	2019	41.63	10.04	158.39	RW2
Uganda	ALL	15-19	47.60	19.15	115.65	RW2
Uganda	CENTRAL	1980	216.04	170.00	271.63	RW2
Uganda	CENTRAL	1981	210.42	177.77	247.63	RW2
Uganda	CENTRAL	1982	204.95	176.19	237.36	RW2
Uganda	CENTRAL	1983	199.53	168.83	234.51	RW2
Uganda	CENTRAL	1984	194.35	162.10	231.36	RW2
Uganda	CENTRAL	1985	189.07	159.59	222.43	RW2
Uganda	CENTRAL	1986	184.20	157.89	213.38	RW2
Uganda	CENTRAL	1987	179.27	154.58	207.13	RW2
Uganda	CENTRAL	1988	174.76	149.35	202.95	RW2
Uganda	CENTRAL	1989	170.43	144.31	200.27	RW2
Uganda	CENTRAL	1990	166.32	140.77	195.25	RW2
Uganda	CENTRAL	1991	162.60	139.02	189.39	RW2
Uganda	CENTRAL	1992	159.19	136.63	184.37	RW2
Uganda	CENTRAL	1993	156.09	133.31	181.76	RW2
Uganda	CENTRAL	1994	153.18	129.04	180.73	RW2
Uganda	CENTRAL	1995	150.30	126.75	177.37	RW2
Uganda	CENTRAL	1996	147.26	125.39	172.31	RW2
Uganda	CENTRAL	1997	143.82	123.07	167.26	RW2
Uganda	CENTRAL	1998	139.91	118.88	163.68	RW2
Uganda	CENTRAL	1999	135.42	113.78	160.54	RW2
Uganda	CENTRAL	2000	130.25	109.31	154.53	RW2
Uganda	CENTRAL	2001	124.36	105.78	146.25	RW2
Uganda	CENTRAL	2002	117.73	100.25	138.02	RW2
Uganda	CENTRAL	2003	110.43	93.20	130.59	RW2
Uganda	CENTRAL	2004	102.88	85.62	123.64	RW2
Uganda	CENTRAL	2005	94.96	78.24	114.50	RW2
Uganda	CENTRAL	2006	87.63	72.68	105.12	RW2
Uganda	CENTRAL	2007	80.71	67.20	97.18	RW2
Uganda	CENTRAL	2008	74.38	60.89	90.41	RW2
Uganda	CENTRAL	2009	68.52	54.73	85.41	RW2
Uganda	CENTRAL	2010	63.42	49.14	81.24	RW2
Uganda	CENTRAL	2011	58.54	44.58	75.92	RW2
Uganda	CENTRAL	2012	54.12	40.18	71.31	RW2
Uganda	CENTRAL	2013	50.01	34.90	68.74	RW2
Uganda	CENTRAL	2014	46.12	29.22	69.59	RW2
Uganda	CENTRAL	2015	42.55	23.26	74.08	RW2
Uganda	CENTRAL	2016	39.20	18.24	79.56	RW2
Uganda	CENTRAL	2017	36.07	13.92	87.75	RW2
Uganda	CENTRAL	2018	33.24	10.36	98.99	RW2
Uganda	CENTRAL	2019	30.54	7.61	113.25	RW2
Uganda	EASTERN	1980	225.68	175.61	283.32	RW2
Uganda	EASTERN	1981	222.61	187.02	261.60	RW2
Uganda	EASTERN	1982	219.43	188.12	253.95	RW2
Uganda	EASTERN	1983	215.95	183.10	253.19	RW2
Uganda	EASTERN	1984	212.75	178.27	251.70	RW2
Uganda	EASTERN	1985	209.19	177.40	245.25	RW2
Uganda	EASTERN	1986	205.39	176.59	237.80	RW2
Uganda	EASTERN	1987	201.33	173.92	232.24	RW2

Continued on next page

Country	Region	Year	Median	Lower	Upper	Method
Uganda	EASTERN	1988	196.80	168.90	228.20	RW2
Uganda	EASTERN	1989	192.03	163.16	225.26	RW2
Uganda	EASTERN	1990	186.72	158.32	218.91	RW2
Uganda	EASTERN	1991	181.33	155.69	210.81	RW2
Uganda	EASTERN	1992	175.87	151.70	203.41	RW2
Uganda	EASTERN	1993	170.16	145.59	198.19	RW2
Uganda	EASTERN	1994	164.41	139.12	193.25	RW2
Uganda	EASTERN	1995	158.53	134.62	186.32	RW2
Uganda	EASTERN	1996	152.47	130.42	177.36	RW2
Uganda	EASTERN	1997	146.22	125.57	169.51	RW2
Uganda	EASTERN	1998	139.85	118.97	163.27	RW2
Uganda	EASTERN	1999	133.12	111.35	157.56	RW2
Uganda	EASTERN	2000	126.58	105.88	149.79	RW2
Uganda	EASTERN	2001	119.66	101.08	140.48	RW2
Uganda	EASTERN	2002	112.74	95.84	131.91	RW2
Uganda	EASTERN	2003	105.72	89.13	124.57	RW2
Uganda	EASTERN	2004	98.96	82.45	118.57	RW2
Uganda	EASTERN	2005	92.17	76.34	110.51	RW2
Uganda	EASTERN	2006	86.26	72.01	102.67	RW2
Uganda	EASTERN	2007	80.94	67.92	96.11	RW2
Uganda	EASTERN	2008	76.16	63.13	91.95	RW2
Uganda	EASTERN	2009	71.98	58.02	89.10	RW2
Uganda	EASTERN	2010	68.36	53.47	87.59	RW2
Uganda	EASTERN	2011	64.95	50.35	84.54	RW2
Uganda	EASTERN	2012	61.75	46.94	82.06	RW2
Uganda	EASTERN	2013	58.72	42.50	82.22	RW2
Uganda	EASTERN	2014	55.85	36.88	85.65	RW2
Uganda	EASTERN	2015	53.16	30.19	93.87	RW2
Uganda	EASTERN	2016	50.52	24.40	104.15	RW2
Uganda	EASTERN	2017	47.97	19.32	117.51	RW2
Uganda	EASTERN	2018	45.63	14.97	135.67	RW2
Uganda	EASTERN	2019	43.39	11.24	158.25	RW2
Uganda	NORTHERN	1980	257.95	202.02	323.27	RW2
Uganda	NORTHERN	1981	253.98	212.78	300.41	RW2
Uganda	NORTHERN	1982	249.73	213.39	289.85	RW2
Uganda	NORTHERN	1983	245.58	207.68	287.87	RW2
Uganda	NORTHERN	1984	241.41	201.62	285.80	RW2
Uganda	NORTHERN	1985	237.39	201.25	277.75	RW2
Uganda	NORTHERN	1986	233.36	200.64	269.07	RW2
Uganda	NORTHERN	1987	229.33	198.72	263.06	RW2
Uganda	NORTHERN	1988	225.38	194.10	260.08	RW2
Uganda	NORTHERN	1989	221.50	189.14	257.70	RW2
Uganda	NORTHERN	1990	217.61	185.89	252.88	RW2
Uganda	NORTHERN	1991	214.21	185.11	246.45	RW2
Uganda	NORTHERN	1992	210.86	182.91	241.52	RW2
Uganda	NORTHERN	1993	207.56	179.23	238.84	RW2
Uganda	NORTHERN	1994	204.49	174.45	237.39	RW2
Uganda	NORTHERN	1995	201.22	172.06	234.20	RW2
Uganda	NORTHERN	1996	197.42	170.41	227.54	RW2
Uganda	NORTHERN	1997	192.88	167.37	221.52	RW2
Uganda	NORTHERN	1998	187.52	162.04	216.16	RW2
Uganda	NORTHERN	1999	181.22	153.94	211.00	RW2
Uganda	NORTHERN	2000	173.96	147.80	203.21	RW2
Uganda	NORTHERN	2001	165.64	141.98	191.90	RW2
Uganda	NORTHERN	2002	156.37	134.78	180.69	RW2
Uganda	NORTHERN	2003	146.41	125.38	170.88	RW2
Uganda	NORTHERN	2004	136.29	114.88	161.36	RW2
Uganda	NORTHERN	2005	125.94	105.17	149.70	RW2
Uganda	NORTHERN	2006	116.60	98.14	137.88	RW2
Uganda	NORTHERN	2007	108.02	90.69	127.80	RW2
Uganda	NORTHERN	2008	100.41	82.93	120.83	RW2
Uganda	NORTHERN	2009	93.53	75.24	115.50	RW2
Uganda	NORTHERN	2010	87.71	68.74	111.69	RW2

Continued on next page

Country	Region	Year	Median	Lower	Upper	Method
Uganda	NORTHERN	2011	82.15	63.42	106.09	RW2
Uganda	NORTHERN	2012	77.07	58.24	101.44	RW2
Uganda	NORTHERN	2013	72.37	52.07	100.13	RW2
Uganda	NORTHERN	2014	67.92	44.62	102.15	RW2
Uganda	NORTHERN	2015	63.66	36.21	109.77	RW2
Uganda	NORTHERN	2016	59.57	28.89	119.43	RW2
Uganda	NORTHERN	2017	55.90	22.54	133.25	RW2
Uganda	NORTHERN	2018	52.21	17.09	151.27	RW2
Uganda	NORTHERN	2019	48.93	12.73	174.92	RW2
Uganda	WESTERN	1980	188.62	146.72	239.90	RW2
Uganda	WESTERN	1981	187.30	157.48	221.42	RW2
Uganda	WESTERN	1982	186.03	159.31	215.50	RW2
Uganda	WESTERN	1983	184.60	155.85	217.56	RW2
Uganda	WESTERN	1984	183.44	152.78	218.48	RW2
Uganda	WESTERN	1985	182.35	153.16	214.56	RW2
Uganda	WESTERN	1986	181.34	154.89	211.08	RW2
Uganda	WESTERN	1987	180.71	155.52	208.57	RW2
Uganda	WESTERN	1988	180.25	154.11	209.41	RW2
Uganda	WESTERN	1989	179.96	152.28	211.63	RW2
Uganda	WESTERN	1990	179.90	152.19	211.28	RW2
Uganda	WESTERN	1991	180.35	154.21	209.03	RW2
Uganda	WESTERN	1992	180.72	155.61	208.68	RW2
Uganda	WESTERN	1993	181.24	155.60	210.64	RW2
Uganda	WESTERN	1994	181.66	153.94	213.37	RW2
Uganda	WESTERN	1995	181.56	153.97	213.90	RW2
Uganda	WESTERN	1996	180.49	154.67	209.95	RW2
Uganda	WESTERN	1997	178.30	153.54	207.12	RW2
Uganda	WESTERN	1998	174.92	149.60	203.97	RW2
Uganda	WESTERN	1999	169.92	143.60	199.81	RW2
Uganda	WESTERN	2000	163.71	138.55	192.99	RW2
Uganda	WESTERN	2001	156.14	133.25	182.30	RW2
Uganda	WESTERN	2002	147.43	126.51	171.40	RW2
Uganda	WESTERN	2003	138.01	117.44	161.70	RW2
Uganda	WESTERN	2004	128.35	107.51	152.32	RW2
Uganda	WESTERN	2005	118.47	98.26	141.33	RW2
Uganda	WESTERN	2006	109.50	91.76	129.99	RW2
Uganda	WESTERN	2007	101.47	85.29	120.26	RW2
Uganda	WESTERN	2008	94.31	78.26	113.07	RW2
Uganda	WESTERN	2009	87.89	71.44	107.41	RW2
Uganda	WESTERN	2010	82.47	65.73	103.19	RW2
Uganda	WESTERN	2011	77.37	61.63	96.96	RW2
Uganda	WESTERN	2012	72.68	57.38	91.70	RW2
Uganda	WESTERN	2013	68.31	51.78	89.57	RW2
Uganda	WESTERN	2014	64.10	44.28	91.70	RW2
Uganda	WESTERN	2015	60.24	35.87	99.62	RW2
Uganda	WESTERN	2016	56.48	28.45	109.21	RW2
Uganda	WESTERN	2017	53.10	22.15	122.74	RW2
Uganda	WESTERN	2018	49.67	16.87	139.30	RW2
Uganda	WESTERN	2019	46.73	12.67	160.80	RW2
Zambia	ALL	1980	160.55	151.46	170.16	IHME
Zambia	ALL	1980	152.41	114.83	199.40	RW2
Zambia	ALL	1980	156.80	146.40	167.80	UN
Zambia	ALL	1981	159.61	149.64	169.37	IHME
Zambia	ALL	1981	157.23	129.13	189.98	RW2
Zambia	ALL	1981	158.50	148.00	169.50	UN
Zambia	ALL	1982	158.97	150.24	167.94	IHME
Zambia	ALL	1982	162.21	135.57	192.81	RW2
Zambia	ALL	1982	160.90	150.40	171.90	UN
Zambia	ALL	1983	160.43	151.75	169.54	IHME
Zambia	ALL	1983	167.00	136.70	201.98	RW2
Zambia	ALL	1983	164.50	153.70	175.50	UN
Zambia	ALL	1984	163.84	155.15	172.87	IHME
Zambia	ALL	1984	171.99	137.80	211.36	RW2

Continued on next page

Country	Region	Year	Median	Lower	Upper	Method
Zambia	ALL	1984	169.20	158.30	180.20	UN
Zambia	ALL	1985	168.10	159.09	177.17	IHME
Zambia	ALL	1985	176.59	145.03	213.66	RW2
Zambia	ALL	1985	174.70	163.90	185.90	UN
Zambia	ALL	1986	171.96	162.98	181.03	IHME
Zambia	ALL	1986	180.70	150.88	214.91	RW2
Zambia	ALL	1986	180.10	169.30	191.70	UN
Zambia	ALL	1987	172.76	163.76	181.39	IHME
Zambia	ALL	1987	184.25	155.43	217.51	RW2
Zambia	ALL	1987	184.70	173.60	196.40	UN
Zambia	ALL	1988	170.66	161.82	179.41	IHME
Zambia	ALL	1988	186.91	156.21	221.93	RW2
Zambia	ALL	1988	188.00	176.80	199.70	UN
Zambia	ALL	1989	169.06	160.69	177.70	IHME
Zambia	ALL	1989	188.75	156.10	226.57	RW2
Zambia	ALL	1989	190.00	178.60	201.60	UN
Zambia	ALL	1990	168.91	161.09	177.34	IHME
Zambia	ALL	1990	189.65	156.85	227.29	RW2
Zambia	ALL	1990	190.60	179.30	202.40	UN
Zambia	ALL	1991	169.28	160.91	177.88	IHME
Zambia	ALL	1991	189.83	158.92	224.39	RW2
Zambia	ALL	1991	190.30	178.90	202.20	UN
Zambia	ALL	1992	169.42	161.47	178.45	IHME
Zambia	ALL	1992	189.20	159.28	222.83	RW2
Zambia	ALL	1992	189.40	178.10	201.00	UN
Zambia	ALL	1993	168.37	159.58	177.56	IHME
Zambia	ALL	1993	187.84	157.47	222.77	RW2
Zambia	ALL	1993	187.50	176.20	199.20	UN
Zambia	ALL	1994	165.83	156.94	175.24	IHME
Zambia	ALL	1994	185.70	153.71	223.58	RW2
Zambia	ALL	1994	184.50	173.50	196.00	UN
Zambia	ALL	1995	161.63	153.43	170.35	IHME
Zambia	ALL	1995	182.93	151.36	219.83	RW2
Zambia	ALL	1995	181.10	170.40	192.40	UN
Zambia	ALL	1996	157.03	148.66	165.14	IHME
Zambia	ALL	1996	179.50	149.97	214.37	RW2
Zambia	ALL	1996	178.10	167.30	189.30	UN
Zambia	ALL	1997	153.48	144.29	162.00	IHME
Zambia	ALL	1997	175.49	147.26	207.92	RW2
Zambia	ALL	1997	175.70	164.80	186.50	UN
Zambia	ALL	1998	152.10	143.32	161.41	IHME
Zambia	ALL	1998	170.88	142.16	204.18	RW2
Zambia	ALL	1998	172.90	162.30	184.00	UN
Zambia	ALL	1999	150.83	142.39	160.27	IHME
Zambia	ALL	1999	165.39	135.54	198.80	RW2
Zambia	ALL	1999	169.10	158.40	180.40	UN
Zambia	ALL	2000	147.30	137.62	156.69	IHME
Zambia	ALL	2000	159.47	131.17	192.81	RW2
Zambia	ALL	2000	163.10	152.40	174.90	UN
Zambia	ALL	2001	140.44	131.66	150.11	IHME
Zambia	ALL	2001	151.92	125.74	182.39	RW2
Zambia	ALL	2001	154.20	143.90	165.60	UN
Zambia	ALL	2002	131.21	122.85	140.37	IHME
Zambia	ALL	2002	143.29	119.27	171.39	RW2
Zambia	ALL	2002	142.90	133.60	153.10	UN
Zambia	ALL	2003	122.29	114.02	130.78	IHME
Zambia	ALL	2003	133.97	111.06	161.41	RW2
Zambia	ALL	2003	131.20	122.30	140.70	UN
Zambia	ALL	2004	114.74	106.63	122.74	IHME
Zambia	ALL	2004	124.15	101.22	152.23	RW2
Zambia	ALL	2004	120.60	111.70	129.40	UN
Zambia	ALL	2005	108.61	100.92	117.05	IHME
Zambia	ALL	2005	114.04	91.38	139.84	RW2

Continued on next page

Country	Region	Year	Median	Lower	Upper	Method
Zambia	ALL	2005	111.70	103.10	120.50	UN
Zambia	ALL	2006	103.84	96.33	112.21	IHME
Zambia	ALL	2006	105.37	85.21	128.48	RW2
Zambia	ALL	2006	104.50	96.10	113.00	UN
Zambia	ALL	2007	98.97	91.64	106.93	IHME
Zambia	ALL	2007	97.79	79.65	119.04	RW2
Zambia	ALL	2007	98.00	90.20	106.00	UN
Zambia	ALL	2008	94.03	86.20	102.67	IHME
Zambia	ALL	2008	91.49	73.85	112.49	RW2
Zambia	ALL	2008	93.10	85.50	101.20	UN
Zambia	ALL	2009	89.64	81.63	98.89	IHME
Zambia	ALL	2009	86.03	68.23	107.64	RW2
Zambia	ALL	2009	87.40	80.00	95.70	UN
Zambia	ALL	2010	83.00	75.23	90.84	IHME
Zambia	ALL	2010	81.87	64.54	104.57	RW2
Zambia	ALL	2010	82.10	74.60	90.40	UN
Zambia	ALL	2011	77.32	69.63	85.57	IHME
Zambia	ALL	2011	77.91	62.18	97.79	RW2
Zambia	ALL	2011	78.60	70.60	87.60	UN
Zambia	ALL	2012	73.30	65.43	82.07	IHME
Zambia	ALL	2012	74.30	60.14	91.62	RW2
Zambia	ALL	2012	74.40	65.40	84.80	UN
Zambia	ALL	2013	69.63	60.72	79.07	IHME
Zambia	ALL	2013	71.00	56.04	89.27	RW2
Zambia	ALL	2013	70.20	59.40	82.40	UN
Zambia	ALL	2014	67.12	57.74	78.19	IHME
Zambia	ALL	2014	67.73	48.54	93.91	RW2
Zambia	ALL	2014	66.60	54.10	81.10	UN
Zambia	ALL	2015	65.93	55.36	78.00	IHME
Zambia	ALL	2015	64.44	38.67	106.15	RW2
Zambia	ALL	2015	64.00	49.40	81.30	UN
Zambia	ALL	2016	61.57	30.89	121.34	RW2
Zambia	ALL	2017	58.55	23.77	141.15	RW2
Zambia	ALL	2018	55.78	18.10	167.73	RW2
Zambia	ALL	2019	53.06	12.99	197.39	RW2
Zambia	ALL	15-19	58.53	24.18	138.98	RW2
Zambia	CENTRAL	1980	132.52	99.58	174.88	RW2
Zambia	CENTRAL	1981	136.96	110.93	168.46	RW2
Zambia	CENTRAL	1982	141.57	116.88	170.38	RW2
Zambia	CENTRAL	1983	146.04	119.33	177.29	RW2
Zambia	CENTRAL	1984	150.56	122.07	184.42	RW2
Zambia	CENTRAL	1985	155.09	128.19	186.36	RW2
Zambia	CENTRAL	1986	159.11	134.44	187.46	RW2
Zambia	CENTRAL	1987	162.69	138.68	189.81	RW2
Zambia	CENTRAL	1988	165.74	140.78	194.03	RW2
Zambia	CENTRAL	1989	168.12	141.34	198.61	RW2
Zambia	CENTRAL	1990	169.74	142.81	199.75	RW2
Zambia	CENTRAL	1991	170.76	145.45	199.11	RW2
Zambia	CENTRAL	1992	171.00	146.99	198.55	RW2
Zambia	CENTRAL	1993	170.66	145.99	199.21	RW2
Zambia	CENTRAL	1994	169.41	142.52	199.55	RW2
Zambia	CENTRAL	1995	167.68	141.20	197.98	RW2
Zambia	CENTRAL	1996	165.10	140.44	193.59	RW2
Zambia	CENTRAL	1997	161.59	138.21	188.30	RW2
Zambia	CENTRAL	1998	157.51	133.44	184.67	RW2
Zambia	CENTRAL	1999	152.34	127.23	180.55	RW2
Zambia	CENTRAL	2000	146.58	122.40	174.54	RW2
Zambia	CENTRAL	2001	139.73	117.44	165.71	RW2
Zambia	CENTRAL	2002	132.34	111.45	156.54	RW2
Zambia	CENTRAL	2003	124.22	103.44	148.37	RW2
Zambia	CENTRAL	2004	116.14	95.16	141.24	RW2
Zambia	CENTRAL	2005	107.82	87.21	132.11	RW2
Zambia	CENTRAL	2006	100.37	81.29	122.99	RW2

Continued on next page

Country	Region	Year	Median	Lower	Upper	Method
Zambia	CENTRAL	2007	93.70	75.69	115.02	RW2
Zambia	CENTRAL	2008	87.99	70.13	109.37	RW2
Zambia	CENTRAL	2009	82.91	64.76	105.19	RW2
Zambia	CENTRAL	2010	78.73	60.97	101.54	RW2
Zambia	CENTRAL	2011	74.61	57.86	95.90	RW2
Zambia	CENTRAL	2012	70.95	55.16	90.75	RW2
Zambia	CENTRAL	2013	67.52	51.04	88.64	RW2
Zambia	CENTRAL	2014	64.24	44.86	91.03	RW2
Zambia	CENTRAL	2015	60.99	36.95	99.68	RW2
Zambia	CENTRAL	2016	57.97	29.73	109.94	RW2
Zambia	CENTRAL	2017	55.02	23.45	124.70	RW2
Zambia	CENTRAL	2018	52.26	18.19	144.47	RW2
Zambia	CENTRAL	2019	49.68	13.50	167.56	RW2
Zambia	COPPERBELT	1980	116.40	86.35	154.66	RW2
Zambia	COPPERBELT	1981	121.63	97.94	149.75	RW2
Zambia	COPPERBELT	1982	126.89	104.81	152.72	RW2
Zambia	COPPERBELT	1983	132.57	108.40	160.56	RW2
Zambia	COPPERBELT	1984	137.98	111.88	168.14	RW2
Zambia	COPPERBELT	1985	143.59	118.95	171.88	RW2
Zambia	COPPERBELT	1986	148.61	125.71	174.40	RW2
Zambia	COPPERBELT	1987	153.20	130.91	178.43	RW2
Zambia	COPPERBELT	1988	157.11	133.76	183.41	RW2
Zambia	COPPERBELT	1989	160.34	135.13	189.76	RW2
Zambia	COPPERBELT	1990	162.65	137.12	192.08	RW2
Zambia	COPPERBELT	1991	164.09	140.05	191.69	RW2
Zambia	COPPERBELT	1992	164.66	141.30	191.13	RW2
Zambia	COPPERBELT	1993	164.44	140.20	192.17	RW2
Zambia	COPPERBELT	1994	163.22	137.20	193.31	RW2
Zambia	COPPERBELT	1995	161.36	135.31	191.44	RW2
Zambia	COPPERBELT	1996	158.44	133.99	186.74	RW2
Zambia	COPPERBELT	1997	154.71	131.13	181.77	RW2
Zambia	COPPERBELT	1998	150.14	126.46	177.47	RW2
Zambia	COPPERBELT	1999	144.70	119.83	173.30	RW2
Zambia	COPPERBELT	2000	138.85	114.85	167.01	RW2
Zambia	COPPERBELT	2001	131.92	109.70	157.97	RW2
Zambia	COPPERBELT	2002	124.36	103.61	148.80	RW2
Zambia	COPPERBELT	2003	116.33	95.86	140.50	RW2
Zambia	COPPERBELT	2004	108.21	87.97	132.84	RW2
Zambia	COPPERBELT	2005	100.08	80.18	123.39	RW2
Zambia	COPPERBELT	2006	92.79	74.49	114.72	RW2
Zambia	COPPERBELT	2007	86.53	69.20	107.14	RW2
Zambia	COPPERBELT	2008	80.95	63.85	102.12	RW2
Zambia	COPPERBELT	2009	76.06	58.68	97.85	RW2
Zambia	COPPERBELT	2010	72.06	54.68	94.57	RW2
Zambia	COPPERBELT	2011	68.41	51.78	89.60	RW2
Zambia	COPPERBELT	2012	64.98	48.83	85.53	RW2
Zambia	COPPERBELT	2013	61.63	44.81	83.63	RW2
Zambia	COPPERBELT	2014	58.64	39.40	86.38	RW2
Zambia	COPPERBELT	2015	55.61	32.43	93.58	RW2
Zambia	COPPERBELT	2016	52.82	26.16	104.68	RW2
Zambia	COPPERBELT	2017	50.26	20.65	118.39	RW2
Zambia	COPPERBELT	2018	47.72	15.90	136.17	RW2
Zambia	COPPERBELT	2019	45.08	11.86	161.04	RW2
Zambia	EASTERN	1980	217.78	169.17	276.27	RW2
Zambia	EASTERN	1981	221.37	185.81	262.09	RW2
Zambia	EASTERN	1982	224.84	192.10	261.50	RW2
Zambia	EASTERN	1983	228.16	192.37	268.28	RW2
Zambia	EASTERN	1984	231.30	193.54	273.40	RW2
Zambia	EASTERN	1985	234.27	199.02	273.17	RW2
Zambia	EASTERN	1986	236.60	204.64	271.31	RW2
Zambia	EASTERN	1987	238.22	208.27	270.72	RW2
Zambia	EASTERN	1988	239.02	207.42	273.40	RW2
Zambia	EASTERN	1989	238.98	205.22	276.24	RW2

Continued on next page

Country	Region	Year	Median	Lower	Upper	Method
Zambia	EASTERN	1990	237.96	204.61	274.87	RW2
Zambia	EASTERN	1991	236.45	205.54	270.64	RW2
Zambia	EASTERN	1992	234.19	204.27	266.86	RW2
Zambia	EASTERN	1993	231.17	200.65	264.71	RW2
Zambia	EASTERN	1994	227.51	194.69	264.01	RW2
Zambia	EASTERN	1995	223.33	190.60	259.69	RW2
Zambia	EASTERN	1996	218.55	187.79	252.36	RW2
Zambia	EASTERN	1997	213.14	183.92	244.93	RW2
Zambia	EASTERN	1998	207.03	177.63	239.91	RW2
Zambia	EASTERN	1999	200.22	169.40	234.85	RW2
Zambia	EASTERN	2000	192.88	162.52	226.59	RW2
Zambia	EASTERN	2001	184.52	157.23	215.20	RW2
Zambia	EASTERN	2002	175.47	150.04	204.20	RW2
Zambia	EASTERN	2003	165.72	140.83	194.57	RW2
Zambia	EASTERN	2004	155.95	130.67	185.55	RW2
Zambia	EASTERN	2005	145.94	120.56	174.91	RW2
Zambia	EASTERN	2006	137.02	113.74	163.87	RW2
Zambia	EASTERN	2007	128.95	107.11	154.29	RW2
Zambia	EASTERN	2008	121.95	100.15	147.58	RW2
Zambia	EASTERN	2009	115.71	93.43	142.42	RW2
Zambia	EASTERN	2010	110.72	87.88	138.91	RW2
Zambia	EASTERN	2011	105.69	84.55	132.22	RW2
Zambia	EASTERN	2012	101.23	81.09	126.38	RW2
Zambia	EASTERN	2013	96.90	75.22	124.75	RW2
Zambia	EASTERN	2014	92.81	66.27	128.84	RW2
Zambia	EASTERN	2015	88.80	54.81	142.34	RW2
Zambia	EASTERN	2016	84.93	44.77	158.12	RW2
Zambia	EASTERN	2017	81.30	35.41	180.19	RW2
Zambia	EASTERN	2018	77.54	27.06	206.58	RW2
Zambia	EASTERN	2019	74.05	20.78	238.61	RW2
Zambia	LUAPULA	1980	204.71	157.07	261.12	RW2
Zambia	LUAPULA	1981	211.01	175.13	251.21	RW2
Zambia	LUAPULA	1982	217.43	184.88	254.15	RW2
Zambia	LUAPULA	1983	223.95	188.50	264.12	RW2
Zambia	LUAPULA	1984	230.32	191.46	273.21	RW2
Zambia	LUAPULA	1985	236.34	200.38	277.10	RW2
Zambia	LUAPULA	1986	241.53	208.06	278.60	RW2
Zambia	LUAPULA	1987	245.78	213.64	281.35	RW2
Zambia	LUAPULA	1988	248.89	214.71	286.37	RW2
Zambia	LUAPULA	1989	250.55	214.54	291.52	RW2
Zambia	LUAPULA	1990	251.20	215.10	290.77	RW2
Zambia	LUAPULA	1991	250.68	216.68	288.08	RW2
Zambia	LUAPULA	1992	249.16	216.84	284.49	RW2
Zambia	LUAPULA	1993	246.38	212.72	283.75	RW2
Zambia	LUAPULA	1994	242.55	206.92	282.21	RW2
Zambia	LUAPULA	1995	237.62	202.03	277.32	RW2
Zambia	LUAPULA	1996	231.57	198.94	268.82	RW2
Zambia	LUAPULA	1997	224.31	192.51	259.24	RW2
Zambia	LUAPULA	1998	215.59	183.65	251.26	RW2
Zambia	LUAPULA	1999	205.93	172.56	242.58	RW2
Zambia	LUAPULA	2000	195.48	163.08	232.28	RW2
Zambia	LUAPULA	2001	183.86	154.13	217.68	RW2
Zambia	LUAPULA	2002	171.80	143.99	203.65	RW2
Zambia	LUAPULA	2003	159.46	132.09	190.60	RW2
Zambia	LUAPULA	2004	147.16	119.74	179.39	RW2
Zambia	LUAPULA	2005	135.14	108.52	166.38	RW2
Zambia	LUAPULA	2006	124.40	99.66	153.51	RW2
Zambia	LUAPULA	2007	114.88	91.65	143.06	RW2
Zambia	LUAPULA	2008	106.38	83.52	134.79	RW2
Zambia	LUAPULA	2009	99.10	75.65	128.35	RW2
Zambia	LUAPULA	2010	92.85	69.70	123.08	RW2
Zambia	LUAPULA	2011	87.04	64.75	115.91	RW2
Zambia	LUAPULA	2012	81.65	60.18	110.49	RW2

Continued on next page

Country	Region	Year	Median	Lower	Upper	Method
Zambia	LUAPULA	2013	76.60	54.08	107.25	RW2
Zambia	LUAPULA	2014	71.85	46.85	109.30	RW2
Zambia	LUAPULA	2015	67.42	37.81	117.85	RW2
Zambia	LUAPULA	2016	63.03	30.45	127.86	RW2
Zambia	LUAPULA	2017	59.00	23.33	142.88	RW2
Zambia	LUAPULA	2018	55.34	17.74	161.07	RW2
Zambia	LUAPULA	2019	51.84	13.08	183.53	RW2
Zambia	LUSAKA	1980	113.80	85.12	150.49	RW2
Zambia	LUSAKA	1981	118.33	96.09	144.87	RW2
Zambia	LUSAKA	1982	122.93	101.61	147.33	RW2
Zambia	LUSAKA	1983	127.71	104.70	154.63	RW2
Zambia	LUSAKA	1984	132.48	107.57	161.76	RW2
Zambia	LUSAKA	1985	137.33	113.75	164.93	RW2
Zambia	LUSAKA	1986	141.78	119.68	166.69	RW2
Zambia	LUSAKA	1987	145.87	124.50	170.17	RW2
Zambia	LUSAKA	1988	149.46	127.00	175.06	RW2
Zambia	LUSAKA	1989	152.50	128.08	180.49	RW2
Zambia	LUSAKA	1990	154.85	130.23	183.06	RW2
Zambia	LUSAKA	1991	156.60	133.41	183.62	RW2
Zambia	LUSAKA	1992	157.54	134.97	183.01	RW2
Zambia	LUSAKA	1993	157.71	134.23	184.46	RW2
Zambia	LUSAKA	1994	157.13	131.82	186.32	RW2
Zambia	LUSAKA	1995	155.93	130.66	185.25	RW2
Zambia	LUSAKA	1996	153.87	130.00	181.17	RW2
Zambia	LUSAKA	1997	151.24	128.35	177.08	RW2
Zambia	LUSAKA	1998	147.70	124.51	174.16	RW2
Zambia	LUSAKA	1999	143.52	119.05	171.97	RW2
Zambia	LUSAKA	2000	138.72	114.86	166.52	RW2
Zambia	LUSAKA	2001	132.87	110.95	158.01	RW2
Zambia	LUSAKA	2002	126.34	105.79	150.20	RW2
Zambia	LUSAKA	2003	119.22	99.09	142.92	RW2
Zambia	LUSAKA	2004	112.01	91.56	136.55	RW2
Zambia	LUSAKA	2005	104.44	84.02	128.21	RW2
Zambia	LUSAKA	2006	97.84	78.89	120.25	RW2
Zambia	LUSAKA	2007	91.73	73.68	113.40	RW2
Zambia	LUSAKA	2008	86.44	68.65	108.03	RW2
Zambia	LUSAKA	2009	82.04	63.93	104.36	RW2
Zambia	LUSAKA	2010	78.26	60.05	101.07	RW2
Zambia	LUSAKA	2011	74.70	57.78	96.23	RW2
Zambia	LUSAKA	2012	71.43	55.14	92.05	RW2
Zambia	LUSAKA	2013	68.38	51.14	90.22	RW2
Zambia	LUSAKA	2014	65.50	45.18	93.29	RW2
Zambia	LUSAKA	2015	62.56	37.65	102.86	RW2
Zambia	LUSAKA	2016	59.76	30.44	114.20	RW2
Zambia	LUSAKA	2017	57.24	24.15	129.99	RW2
Zambia	LUSAKA	2018	54.39	18.75	151.12	RW2
Zambia	LUSAKA	2019	51.98	14.16	176.26	RW2
Zambia	NORTH-WESTERN	1980	144.22	109.76	187.54	RW2
Zambia	NORTH-WESTERN	1981	147.22	121.39	177.83	RW2
Zambia	NORTH-WESTERN	1982	150.23	126.00	178.28	RW2
Zambia	NORTH-WESTERN	1983	153.10	126.87	183.65	RW2
Zambia	NORTH-WESTERN	1984	155.93	127.79	188.91	RW2
Zambia	NORTH-WESTERN	1985	158.80	132.18	189.04	RW2
Zambia	NORTH-WESTERN	1986	160.94	136.77	188.21	RW2
Zambia	NORTH-WESTERN	1987	162.66	139.38	188.73	RW2
Zambia	NORTH-WESTERN	1988	163.87	139.57	191.32	RW2
Zambia	NORTH-WESTERN	1989	164.40	138.38	193.88	RW2
Zambia	NORTH-WESTERN	1990	164.14	138.88	193.46	RW2
Zambia	NORTH-WESTERN	1991	163.44	139.48	190.54	RW2
Zambia	NORTH-WESTERN	1992	161.98	139.22	187.56	RW2
Zambia	NORTH-WESTERN	1993	159.95	136.73	186.42	RW2
Zambia	NORTH-WESTERN	1994	157.23	132.46	185.51	RW2
Zambia	NORTH-WESTERN	1995	153.95	129.71	182.07	RW2

Continued on next page

Country	Region	Year	Median	Lower	Upper	Method
Zambia	NORTH-WESTERN	1996	150.04	127.35	176.03	RW2
Zambia	NORTH-WESTERN	1997	145.59	124.02	170.13	RW2
Zambia	NORTH-WESTERN	1998	140.52	118.35	165.42	RW2
Zambia	NORTH-WESTERN	1999	134.71	111.94	160.54	RW2
Zambia	NORTH-WESTERN	2000	128.57	106.76	153.97	RW2
Zambia	NORTH-WESTERN	2001	121.55	101.36	144.97	RW2
Zambia	NORTH-WESTERN	2002	114.14	95.51	136.02	RW2
Zambia	NORTH-WESTERN	2003	106.27	87.83	128.03	RW2
Zambia	NORTH-WESTERN	2004	98.48	80.16	120.76	RW2
Zambia	NORTH-WESTERN	2005	90.59	72.58	112.04	RW2
Zambia	NORTH-WESTERN	2006	83.74	67.26	103.64	RW2
Zambia	NORTH-WESTERN	2007	77.61	62.09	96.52	RW2
Zambia	NORTH-WESTERN	2008	72.29	57.09	90.97	RW2
Zambia	NORTH-WESTERN	2009	67.69	52.47	86.64	RW2
Zambia	NORTH-WESTERN	2010	63.78	48.93	83.04	RW2
Zambia	NORTH-WESTERN	2011	60.22	46.33	78.11	RW2
Zambia	NORTH-WESTERN	2012	56.88	43.96	73.64	RW2
Zambia	NORTH-WESTERN	2013	53.79	40.43	71.73	RW2
Zambia	NORTH-WESTERN	2014	50.85	35.33	73.06	RW2
Zambia	NORTH-WESTERN	2015	48.08	28.78	79.72	RW2
Zambia	NORTH-WESTERN	2016	45.37	23.01	88.21	RW2
Zambia	NORTH-WESTERN	2017	42.90	17.97	101.52	RW2
Zambia	NORTH-WESTERN	2018	40.54	13.72	116.05	RW2
Zambia	NORTH-WESTERN	2019	38.36	10.42	134.18	RW2
Zambia	NORTHERN	1980	179.30	138.13	229.46	RW2
Zambia	NORTHERN	1981	185.02	154.04	220.34	RW2
Zambia	NORTHERN	1982	190.76	162.05	222.89	RW2
Zambia	NORTHERN	1983	196.50	164.95	232.61	RW2
Zambia	NORTHERN	1984	202.10	167.78	241.16	RW2
Zambia	NORTHERN	1985	207.69	175.43	244.27	RW2
Zambia	NORTHERN	1986	212.62	182.53	245.52	RW2
Zambia	NORTHERN	1987	216.70	187.81	248.09	RW2
Zambia	NORTHERN	1988	220.25	189.70	253.52	RW2
Zambia	NORTHERN	1989	222.78	190.06	259.23	RW2
Zambia	NORTHERN	1990	224.33	191.96	260.03	RW2
Zambia	NORTHERN	1991	225.07	194.95	258.66	RW2
Zambia	NORTHERN	1992	225.01	195.99	256.95	RW2
Zambia	NORTHERN	1993	224.09	194.30	257.18	RW2
Zambia	NORTHERN	1994	222.16	190.05	257.97	RW2
Zambia	NORTHERN	1995	219.35	187.15	255.27	RW2
Zambia	NORTHERN	1996	215.39	185.46	248.42	RW2
Zambia	NORTHERN	1997	210.29	181.73	242.11	RW2
Zambia	NORTHERN	1998	204.01	175.60	236.12	RW2
Zambia	NORTHERN	1999	196.56	166.50	230.40	RW2
Zambia	NORTHERN	2000	188.02	158.72	221.13	RW2
Zambia	NORTHERN	2001	178.26	151.84	208.44	RW2
Zambia	NORTHERN	2002	167.44	143.19	195.06	RW2
Zambia	NORTHERN	2003	156.32	132.56	184.31	RW2
Zambia	NORTHERN	2004	144.79	120.84	173.27	RW2
Zambia	NORTHERN	2005	133.23	109.98	159.76	RW2
Zambia	NORTHERN	2006	122.87	101.86	146.98	RW2
Zambia	NORTHERN	2007	113.57	94.02	136.07	RW2
Zambia	NORTHERN	2008	105.22	86.05	127.68	RW2
Zambia	NORTHERN	2009	97.89	78.45	121.04	RW2
Zambia	NORTHERN	2010	91.56	72.38	115.49	RW2
Zambia	NORTHERN	2011	85.76	67.60	107.32	RW2
Zambia	NORTHERN	2012	80.31	63.39	100.84	RW2
Zambia	NORTHERN	2013	75.30	57.50	97.44	RW2
Zambia	NORTHERN	2014	70.45	49.31	99.12	RW2
Zambia	NORTHERN	2015	65.98	39.80	107.66	RW2
Zambia	NORTHERN	2016	61.84	31.65	117.91	RW2
Zambia	NORTHERN	2017	57.70	24.42	130.84	RW2
Zambia	NORTHERN	2018	53.79	18.57	148.17	RW2

Continued on next page

Country	Region	Year	Median	Lower	Upper	Method
Zambia	NORTHERN	2019	50.38	13.76	172.63	RW2
Zambia	SOUTHERN	1980	125.95	95.05	164.97	RW2
Zambia	SOUTHERN	1981	129.20	105.71	157.55	RW2
Zambia	SOUTHERN	1982	132.73	110.52	158.35	RW2
Zambia	SOUTHERN	1983	136.14	112.27	164.42	RW2
Zambia	SOUTHERN	1984	139.45	113.67	169.49	RW2
Zambia	SOUTHERN	1985	142.82	118.64	170.83	RW2
Zambia	SOUTHERN	1986	145.74	123.57	171.18	RW2
Zambia	SOUTHERN	1987	148.34	126.71	172.43	RW2
Zambia	SOUTHERN	1988	150.48	128.02	176.11	RW2
Zambia	SOUTHERN	1989	152.02	127.61	180.16	RW2
Zambia	SOUTHERN	1990	152.80	128.50	180.50	RW2
Zambia	SOUTHERN	1991	153.21	130.68	178.96	RW2
Zambia	SOUTHERN	1992	152.92	130.77	177.65	RW2
Zambia	SOUTHERN	1993	152.03	129.55	177.77	RW2
Zambia	SOUTHERN	1994	150.34	126.06	178.01	RW2
Zambia	SOUTHERN	1995	148.29	124.10	176.07	RW2
Zambia	SOUTHERN	1996	145.36	122.96	171.06	RW2
Zambia	SOUTHERN	1997	141.89	120.29	166.23	RW2
Zambia	SOUTHERN	1998	137.73	116.01	162.65	RW2
Zambia	SOUTHERN	1999	132.90	110.60	158.55	RW2
Zambia	SOUTHERN	2000	127.64	105.98	153.00	RW2
Zambia	SOUTHERN	2001	121.50	101.73	144.45	RW2
Zambia	SOUTHERN	2002	114.71	96.65	136.02	RW2
Zambia	SOUTHERN	2003	107.64	89.53	129.06	RW2
Zambia	SOUTHERN	2004	100.50	82.59	122.30	RW2
Zambia	SOUTHERN	2005	93.16	75.37	114.17	RW2
Zambia	SOUTHERN	2006	86.83	70.55	106.33	RW2
Zambia	SOUTHERN	2007	81.15	65.82	99.66	RW2
Zambia	SOUTHERN	2008	76.17	60.92	94.66	RW2
Zambia	SOUTHERN	2009	71.95	56.43	91.06	RW2
Zambia	SOUTHERN	2010	68.35	53.03	88.13	RW2
Zambia	SOUTHERN	2011	65.06	50.69	83.18	RW2
Zambia	SOUTHERN	2012	61.99	48.42	79.19	RW2
Zambia	SOUTHERN	2013	59.17	44.85	77.66	RW2
Zambia	SOUTHERN	2014	56.41	39.44	80.42	RW2
Zambia	SOUTHERN	2015	53.69	32.34	88.36	RW2
Zambia	SOUTHERN	2016	51.15	26.16	98.21	RW2
Zambia	SOUTHERN	2017	48.67	20.71	111.90	RW2
Zambia	SOUTHERN	2018	46.32	15.82	130.30	RW2
Zambia	SOUTHERN	2019	44.19	11.94	153.32	RW2
Zambia	WESTERN	1980	203.48	157.46	258.95	RW2
Zambia	WESTERN	1981	205.98	171.73	245.11	RW2
Zambia	WESTERN	1982	208.66	176.79	244.44	RW2
Zambia	WESTERN	1983	210.93	176.43	250.18	RW2
Zambia	WESTERN	1984	213.22	176.97	254.53	RW2
Zambia	WESTERN	1985	215.42	181.30	254.02	RW2
Zambia	WESTERN	1986	216.94	185.47	251.73	RW2
Zambia	WESTERN	1987	218.04	188.40	251.44	RW2
Zambia	WESTERN	1988	218.35	187.14	253.32	RW2
Zambia	WESTERN	1989	217.95	184.70	255.15	RW2
Zambia	WESTERN	1990	216.76	184.27	252.87	RW2
Zambia	WESTERN	1991	214.93	184.69	248.78	RW2
Zambia	WESTERN	1992	212.31	183.76	244.30	RW2
Zambia	WESTERN	1993	208.99	179.21	242.31	RW2
Zambia	WESTERN	1994	204.91	173.85	239.74	RW2
Zambia	WESTERN	1995	200.08	169.33	235.00	RW2
Zambia	WESTERN	1996	194.46	166.22	226.97	RW2
Zambia	WESTERN	1997	187.87	161.04	218.55	RW2
Zambia	WESTERN	1998	180.56	153.28	211.44	RW2
Zambia	WESTERN	1999	172.33	144.12	204.69	RW2
Zambia	WESTERN	2000	163.30	136.01	195.20	RW2
Zambia	WESTERN	2001	153.31	128.18	182.52	RW2

Continued on next page

Country	Region	Year	Median	Lower	Upper	Method
Zambia	WESTERN	2002	142.81	119.49	170.26	RW2
Zambia	WESTERN	2003	131.84	108.84	158.75	RW2
Zambia	WESTERN	2004	121.07	98.19	148.66	RW2
Zambia	WESTERN	2005	110.30	88.03	136.88	RW2
Zambia	WESTERN	2006	100.97	80.33	125.54	RW2
Zambia	WESTERN	2007	92.42	73.22	116.01	RW2
Zambia	WESTERN	2008	85.10	66.16	108.39	RW2
Zambia	WESTERN	2009	78.57	59.71	102.43	RW2
Zambia	WESTERN	2010	73.20	54.64	97.40	RW2
Zambia	WESTERN	2011	68.04	50.68	90.34	RW2
Zambia	WESTERN	2012	63.39	47.05	84.92	RW2
Zambia	WESTERN	2013	59.13	42.33	81.44	RW2
Zambia	WESTERN	2014	55.10	36.42	81.82	RW2
Zambia	WESTERN	2015	51.28	29.57	87.01	RW2
Zambia	WESTERN	2016	47.73	23.37	94.82	RW2
Zambia	WESTERN	2017	44.38	18.13	105.61	RW2
Zambia	WESTERN	2018	41.15	13.85	118.66	RW2
Zambia	WESTERN	2019	38.34	10.18	136.80	RW2
Zimbabwe	ALL	1980	89.58	85.95	93.29	IHME
Zimbabwe	ALL	1980	106.33	72.43	152.64	RW2
Zimbabwe	ALL	1980	103.40	95.30	111.90	UN
Zimbabwe	ALL	1981	86.73	83.27	90.15	IHME
Zimbabwe	ALL	1981	100.01	76.20	129.05	RW2
Zimbabwe	ALL	1981	99.40	91.90	107.60	UN
Zimbabwe	ALL	1982	83.62	80.33	86.93	IHME
Zimbabwe	ALL	1982	94.02	73.89	119.25	RW2
Zimbabwe	ALL	1982	94.70	87.70	102.50	UN
Zimbabwe	ALL	1983	80.23	77.17	83.42	IHME
Zimbabwe	ALL	1983	88.41	67.79	115.74	RW2
Zimbabwe	ALL	1983	89.80	83.10	96.90	UN
Zimbabwe	ALL	1984	76.83	74.07	79.81	IHME
Zimbabwe	ALL	1984	83.73	62.37	113.02	RW2
Zimbabwe	ALL	1984	84.90	78.70	91.50	UN
Zimbabwe	ALL	1985	73.69	71.07	76.42	IHME
Zimbabwe	ALL	1985	79.15	59.60	103.44	RW2
Zimbabwe	ALL	1985	80.60	74.70	86.80	UN
Zimbabwe	ALL	1986	70.91	68.35	73.55	IHME
Zimbabwe	ALL	1986	76.31	58.81	97.92	RW2
Zimbabwe	ALL	1986	77.10	71.20	83.10	UN
Zimbabwe	ALL	1987	68.65	66.27	71.00	IHME
Zimbabwe	ALL	1987	74.69	58.49	95.16	RW2
Zimbabwe	ALL	1987	74.60	68.90	80.60	UN
Zimbabwe	ALL	1988	66.88	64.55	69.23	IHME
Zimbabwe	ALL	1988	74.23	57.32	95.33	RW2
Zimbabwe	ALL	1988	73.50	67.70	79.50	UN
Zimbabwe	ALL	1989	65.70	63.38	68.06	IHME
Zimbabwe	ALL	1989	74.94	56.93	97.51	RW2
Zimbabwe	ALL	1989	73.90	67.90	80.10	UN
Zimbabwe	ALL	1990	65.62	63.23	67.96	IHME
Zimbabwe	ALL	1990	77.01	58.95	101.20	RW2
Zimbabwe	ALL	1990	75.80	69.70	82.00	UN
Zimbabwe	ALL	1991	65.97	63.46	68.40	IHME
Zimbabwe	ALL	1991	79.62	61.78	102.36	RW2
Zimbabwe	ALL	1991	78.80	72.50	85.20	UN
Zimbabwe	ALL	1992	66.77	64.29	69.29	IHME
Zimbabwe	ALL	1992	82.90	64.61	105.54	RW2
Zimbabwe	ALL	1992	82.70	76.30	89.30	UN
Zimbabwe	ALL	1993	67.86	65.26	70.36	IHME
Zimbabwe	ALL	1993	86.67	67.01	110.87	RW2
Zimbabwe	ALL	1993	87.00	80.40	93.90	UN
Zimbabwe	ALL	1994	68.90	66.20	71.38	IHME
Zimbabwe	ALL	1994	90.60	68.86	117.94	RW2
Zimbabwe	ALL	1994	91.20	84.30	98.60	UN

Continued on next page

Country	Region	Year	Median	Lower	Upper	Method
Zimbabwe	ALL	1995	71.32	68.64	73.88	IHME
Zimbabwe	ALL	1995	94.82	72.52	123.98	RW2
Zimbabwe	ALL	1995	95.50	88.10	103.30	UN
Zimbabwe	ALL	1996	73.31	70.59	76.01	IHME
Zimbabwe	ALL	1996	98.38	76.02	127.53	RW2
Zimbabwe	ALL	1996	99.20	91.30	107.50	UN
Zimbabwe	ALL	1997	74.49	71.70	77.40	IHME
Zimbabwe	ALL	1997	101.36	78.87	129.89	RW2
Zimbabwe	ALL	1997	102.00	93.70	110.70	UN
Zimbabwe	ALL	1998	75.41	72.57	78.38	IHME
Zimbabwe	ALL	1998	103.64	80.11	134.33	RW2
Zimbabwe	ALL	1998	103.90	95.20	113.00	UN
Zimbabwe	ALL	1999	76.30	73.40	79.37	IHME
Zimbabwe	ALL	1999	105.02	80.08	136.88	RW2
Zimbabwe	ALL	1999	105.20	96.00	114.70	UN
Zimbabwe	ALL	2000	77.87	75.03	80.97	IHME
Zimbabwe	ALL	2000	105.30	79.90	136.72	RW2
Zimbabwe	ALL	2000	105.80	96.30	115.80	UN
Zimbabwe	ALL	2001	79.33	76.36	82.49	IHME
Zimbabwe	ALL	2001	105.25	80.64	135.58	RW2
Zimbabwe	ALL	2001	105.60	95.70	115.80	UN
Zimbabwe	ALL	2002	81.06	78.03	84.25	IHME
Zimbabwe	ALL	2002	104.63	80.91	134.16	RW2
Zimbabwe	ALL	2002	105.10	95.10	115.30	UN
Zimbabwe	ALL	2003	82.53	79.30	85.96	IHME
Zimbabwe	ALL	2003	103.74	79.72	134.00	RW2
Zimbabwe	ALL	2003	104.00	94.20	114.30	UN
Zimbabwe	ALL	2004	83.99	80.44	87.52	IHME
Zimbabwe	ALL	2004	102.34	76.88	134.82	RW2
Zimbabwe	ALL	2004	103.00	93.40	113.40	UN
Zimbabwe	ALL	2005	83.02	79.55	86.50	IHME
Zimbabwe	ALL	2005	100.96	74.72	134.68	RW2
Zimbabwe	ALL	2005	101.90	92.50	112.00	UN
Zimbabwe	ALL	2006	81.63	78.07	85.29	IHME
Zimbabwe	ALL	2006	99.35	75.34	129.51	RW2
Zimbabwe	ALL	2006	100.00	91.00	110.20	UN
Zimbabwe	ALL	2007	81.70	77.77	85.61	IHME
Zimbabwe	ALL	2007	97.75	76.49	124.03	RW2
Zimbabwe	ALL	2007	98.00	89.00	108.40	UN
Zimbabwe	ALL	2008	81.99	77.64	86.24	IHME
Zimbabwe	ALL	2008	96.26	73.64	125.15	RW2
Zimbabwe	ALL	2008	95.40	86.20	106.00	UN
Zimbabwe	ALL	2009	81.43	76.62	86.37	IHME
Zimbabwe	ALL	2009	94.51	64.22	137.95	RW2
Zimbabwe	ALL	2009	92.90	83.10	104.10	UN
Zimbabwe	ALL	2010	76.95	71.81	82.35	IHME
Zimbabwe	ALL	2010	92.87	50.90	166.23	RW2
Zimbabwe	ALL	2010	89.50	79.00	102.20	UN
Zimbabwe	ALL	2011	71.87	66.38	77.87	IHME
Zimbabwe	ALL	2011	91.39	39.36	199.25	RW2
Zimbabwe	ALL	2011	85.60	73.80	100.70	UN
Zimbabwe	ALL	2012	66.34	60.58	72.98	IHME
Zimbabwe	ALL	2012	89.71	29.32	244.17	RW2
Zimbabwe	ALL	2012	78.50	65.20	96.20	UN
Zimbabwe	ALL	2013	63.99	57.90	71.53	IHME
Zimbabwe	ALL	2013	88.46	21.57	299.18	RW2
Zimbabwe	ALL	2013	74.50	59.10	95.10	UN
Zimbabwe	ALL	2014	62.75	55.94	71.15	IHME
Zimbabwe	ALL	2014	86.83	15.38	367.35	RW2
Zimbabwe	ALL	2014	72.30	54.60	96.30	UN
Zimbabwe	ALL	2015	54.81	48.09	63.31	IHME
Zimbabwe	ALL	2015	84.97	10.92	441.84	RW2
Zimbabwe	ALL	2015	70.70	50.90	97.60	UN

Continued on next page

Country	Region	Year	Median	Lower	Upper	Method
Zimbabwe	ALL	2016	84.17	7.64	533.30	RW2
Zimbabwe	ALL	2017	82.18	5.05	622.85	RW2
Zimbabwe	ALL	2018	80.73	3.42	712.95	RW2
Zimbabwe	ALL	2019	79.05	2.03	779.77	RW2
Zimbabwe	ALL	15-19	82.18	5.10	617.28	RW2
Zimbabwe	BULAWAYO	1980	64.36	39.04	103.68	RW2
Zimbabwe	BULAWAYO	1981	60.62	40.84	89.08	RW2
Zimbabwe	BULAWAYO	1982	57.09	39.62	81.16	RW2
Zimbabwe	BULAWAYO	1983	53.82	37.30	77.17	RW2
Zimbabwe	BULAWAYO	1984	51.02	35.35	73.80	RW2
Zimbabwe	BULAWAYO	1985	48.45	34.01	68.19	RW2
Zimbabwe	BULAWAYO	1986	46.86	33.84	64.59	RW2
Zimbabwe	BULAWAYO	1987	46.05	33.80	62.64	RW2
Zimbabwe	BULAWAYO	1988	46.03	33.64	62.62	RW2
Zimbabwe	BULAWAYO	1989	46.88	33.97	64.56	RW2
Zimbabwe	BULAWAYO	1990	48.67	35.25	66.69	RW2
Zimbabwe	BULAWAYO	1991	51.44	37.83	69.73	RW2
Zimbabwe	BULAWAYO	1992	54.99	40.81	73.47	RW2
Zimbabwe	BULAWAYO	1993	59.48	43.90	79.94	RW2
Zimbabwe	BULAWAYO	1994	64.63	47.03	87.70	RW2
Zimbabwe	BULAWAYO	1995	70.42	51.38	96.95	RW2
Zimbabwe	BULAWAYO	1996	75.41	55.43	102.53	RW2
Zimbabwe	BULAWAYO	1997	79.09	58.25	107.31	RW2
Zimbabwe	BULAWAYO	1998	81.29	59.54	110.88	RW2
Zimbabwe	BULAWAYO	1999	82.09	59.48	113.52	RW2
Zimbabwe	BULAWAYO	2000	80.74	57.69	111.25	RW2
Zimbabwe	BULAWAYO	2001	78.97	56.90	108.45	RW2
Zimbabwe	BULAWAYO	2002	76.72	54.83	105.85	RW2
Zimbabwe	BULAWAYO	2003	74.14	52.32	104.02	RW2
Zimbabwe	BULAWAYO	2004	71.60	49.47	101.96	RW2
Zimbabwe	BULAWAYO	2005	69.01	47.24	99.42	RW2
Zimbabwe	BULAWAYO	2006	66.62	46.13	95.29	RW2
Zimbabwe	BULAWAYO	2007	64.08	44.71	90.62	RW2
Zimbabwe	BULAWAYO	2008	61.67	41.59	90.64	RW2
Zimbabwe	BULAWAYO	2009	59.49	36.09	96.79	RW2
Zimbabwe	BULAWAYO	2010	57.28	28.74	112.43	RW2
Zimbabwe	BULAWAYO	2011	55.20	22.20	132.49	RW2
Zimbabwe	BULAWAYO	2012	52.98	16.54	161.48	RW2
Zimbabwe	BULAWAYO	2013	51.02	12.05	198.43	RW2
Zimbabwe	BULAWAYO	2014	49.13	8.39	245.13	RW2
Zimbabwe	BULAWAYO	2015	47.21	5.81	309.88	RW2
Zimbabwe	BULAWAYO	2016	45.15	3.95	384.02	RW2
Zimbabwe	BULAWAYO	2017	43.69	2.54	468.19	RW2
Zimbabwe	BULAWAYO	2018	41.72	1.63	552.68	RW2
Zimbabwe	BULAWAYO	2019	39.99	1.07	646.04	RW2
Zimbabwe	HARARE	1980	75.64	46.93	119.54	RW2
Zimbabwe	HARARE	1981	71.82	49.47	102.87	RW2
Zimbabwe	HARARE	1982	68.02	48.34	94.86	RW2
Zimbabwe	HARARE	1983	64.59	45.68	90.96	RW2
Zimbabwe	HARARE	1984	61.62	43.41	87.40	RW2
Zimbabwe	HARARE	1985	58.99	42.31	81.48	RW2
Zimbabwe	HARARE	1986	57.39	42.21	77.05	RW2
Zimbabwe	HARARE	1987	56.62	42.46	75.22	RW2
Zimbabwe	HARARE	1988	56.80	42.44	75.53	RW2
Zimbabwe	HARARE	1989	58.01	43.07	77.84	RW2
Zimbabwe	HARARE	1990	60.40	44.74	81.13	RW2
Zimbabwe	HARARE	1991	63.84	47.99	84.53	RW2
Zimbabwe	HARARE	1992	68.38	51.75	89.95	RW2
Zimbabwe	HARARE	1993	73.93	55.64	97.51	RW2
Zimbabwe	HARARE	1994	80.21	59.63	106.98	RW2
Zimbabwe	HARARE	1995	87.54	64.88	118.17	RW2
Zimbabwe	HARARE	1996	93.51	70.07	125.47	RW2
Zimbabwe	HARARE	1997	98.38	73.55	130.84	RW2

Continued on next page

Country	Region	Year	Median	Lower	Upper	Method
Zimbabwe	HARARE	1998	101.20	75.13	135.55	RW2
Zimbabwe	HARARE	1999	102.27	75.22	139.00	RW2
Zimbabwe	HARARE	2000	100.91	72.63	136.77	RW2
Zimbabwe	HARARE	2001	99.15	71.81	134.55	RW2
Zimbabwe	HARARE	2002	96.61	70.00	131.69	RW2
Zimbabwe	HARARE	2003	93.82	67.06	129.68	RW2
Zimbabwe	HARARE	2004	91.00	63.47	128.16	RW2
Zimbabwe	HARARE	2005	88.23	60.94	125.75	RW2
Zimbabwe	HARARE	2006	85.53	59.65	120.92	RW2
Zimbabwe	HARARE	2007	82.82	58.21	116.72	RW2
Zimbabwe	HARARE	2008	80.21	54.63	116.35	RW2
Zimbabwe	HARARE	2009	77.54	47.58	124.76	RW2
Zimbabwe	HARARE	2010	75.12	38.01	144.38	RW2
Zimbabwe	HARARE	2011	72.71	29.68	170.35	RW2
Zimbabwe	HARARE	2012	70.19	22.10	207.09	RW2
Zimbabwe	HARARE	2013	67.69	16.24	250.93	RW2
Zimbabwe	HARARE	2014	65.31	11.62	306.00	RW2
Zimbabwe	HARARE	2015	63.22	7.84	375.78	RW2
Zimbabwe	HARARE	2016	61.46	5.46	455.17	RW2
Zimbabwe	HARARE	2017	58.79	3.59	550.50	RW2
Zimbabwe	HARARE	2018	56.99	2.36	632.26	RW2
Zimbabwe	HARARE	2019	55.21	1.52	725.31	RW2
Zimbabwe	MANICALAND	1980	124.51	84.07	180.40	RW2
Zimbabwe	MANICALAND	1981	117.89	88.82	154.99	RW2
Zimbabwe	MANICALAND	1982	111.47	86.49	143.14	RW2
Zimbabwe	MANICALAND	1983	105.93	80.53	138.58	RW2
Zimbabwe	MANICALAND	1984	100.81	75.39	134.21	RW2
Zimbabwe	MANICALAND	1985	96.44	72.97	125.31	RW2
Zimbabwe	MANICALAND	1986	93.66	72.79	119.01	RW2
Zimbabwe	MANICALAND	1987	92.40	72.84	116.46	RW2
Zimbabwe	MANICALAND	1988	92.72	72.70	117.24	RW2
Zimbabwe	MANICALAND	1989	94.82	73.25	122.04	RW2
Zimbabwe	MANICALAND	1990	98.70	76.28	127.01	RW2
Zimbabwe	MANICALAND	1991	104.29	81.89	132.11	RW2
Zimbabwe	MANICALAND	1992	111.70	88.37	139.81	RW2
Zimbabwe	MANICALAND	1993	120.84	94.71	152.04	RW2
Zimbabwe	MANICALAND	1994	131.00	100.72	167.50	RW2
Zimbabwe	MANICALAND	1995	142.76	110.22	185.05	RW2
Zimbabwe	MANICALAND	1996	152.56	118.43	196.30	RW2
Zimbabwe	MANICALAND	1997	160.44	124.69	205.41	RW2
Zimbabwe	MANICALAND	1998	165.52	128.25	212.93	RW2
Zimbabwe	MANICALAND	1999	167.59	127.92	218.22	RW2
Zimbabwe	MANICALAND	2000	166.45	125.82	215.92	RW2
Zimbabwe	MANICALAND	2001	164.36	124.84	212.74	RW2
Zimbabwe	MANICALAND	2002	161.21	122.54	209.20	RW2
Zimbabwe	MANICALAND	2003	157.45	117.87	206.66	RW2
Zimbabwe	MANICALAND	2004	153.50	113.08	204.91	RW2
Zimbabwe	MANICALAND	2005	149.86	109.03	201.34	RW2
Zimbabwe	MANICALAND	2006	145.73	107.66	194.47	RW2
Zimbabwe	MANICALAND	2007	142.18	106.13	187.47	RW2
Zimbabwe	MANICALAND	2008	138.39	100.01	189.38	RW2
Zimbabwe	MANICALAND	2009	134.42	87.28	203.05	RW2
Zimbabwe	MANICALAND	2010	130.55	70.07	234.05	RW2
Zimbabwe	MANICALAND	2011	127.26	55.00	271.51	RW2
Zimbabwe	MANICALAND	2012	123.64	41.37	321.87	RW2
Zimbabwe	MANICALAND	2013	119.14	30.12	377.50	RW2
Zimbabwe	MANICALAND	2014	116.33	21.75	454.53	RW2
Zimbabwe	MANICALAND	2015	112.70	15.17	524.33	RW2
Zimbabwe	MANICALAND	2016	109.63	10.18	617.07	RW2
Zimbabwe	MANICALAND	2017	107.15	6.85	694.01	RW2
Zimbabwe	MANICALAND	2018	104.10	4.48	764.86	RW2
Zimbabwe	MANICALAND	2019	99.93	2.79	833.77	RW2
Zimbabwe	MASHONALAND CENTRAL	1980	165.98	115.57	231.30	RW2

Continued on next page

Country	Region	Year	Median	Lower	Upper	Method
Zimbabwe	MASHONALAND CENTRAL	1981	154.92	120.66	195.86	RW2
Zimbabwe	MASHONALAND CENTRAL	1982	144.33	115.15	179.33	RW2
Zimbabwe	MASHONALAND CENTRAL	1983	134.56	104.77	172.14	RW2
Zimbabwe	MASHONALAND CENTRAL	1984	125.96	96.51	164.10	RW2
Zimbabwe	MASHONALAND CENTRAL	1985	118.21	90.90	151.01	RW2
Zimbabwe	MASHONALAND CENTRAL	1986	112.70	88.76	141.24	RW2
Zimbabwe	MASHONALAND CENTRAL	1987	108.94	87.10	135.23	RW2
Zimbabwe	MASHONALAND CENTRAL	1988	107.02	84.45	134.46	RW2
Zimbabwe	MASHONALAND CENTRAL	1989	106.95	82.89	136.55	RW2
Zimbabwe	MASHONALAND CENTRAL	1990	108.70	84.25	139.36	RW2
Zimbabwe	MASHONALAND CENTRAL	1991	112.32	88.24	142.21	RW2
Zimbabwe	MASHONALAND CENTRAL	1992	117.62	92.79	147.53	RW2
Zimbabwe	MASHONALAND CENTRAL	1993	124.25	97.36	156.22	RW2
Zimbabwe	MASHONALAND CENTRAL	1994	131.69	101.26	168.23	RW2
Zimbabwe	MASHONALAND CENTRAL	1995	140.20	108.39	181.41	RW2
Zimbabwe	MASHONALAND CENTRAL	1996	146.72	113.93	188.16	RW2
Zimbabwe	MASHONALAND CENTRAL	1997	151.09	117.82	192.35	RW2
Zimbabwe	MASHONALAND CENTRAL	1998	152.68	118.64	196.25	RW2
Zimbabwe	MASHONALAND CENTRAL	1999	151.52	116.12	197.35	RW2
Zimbabwe	MASHONALAND CENTRAL	2000	147.10	110.82	190.51	RW2
Zimbabwe	MASHONALAND CENTRAL	2001	142.19	108.33	183.36	RW2
Zimbabwe	MASHONALAND CENTRAL	2002	136.55	104.06	176.54	RW2
Zimbabwe	MASHONALAND CENTRAL	2003	130.43	98.27	170.89	RW2
Zimbabwe	MASHONALAND CENTRAL	2004	124.57	92.07	165.66	RW2
Zimbabwe	MASHONALAND CENTRAL	2005	119.04	86.81	160.41	RW2
Zimbabwe	MASHONALAND CENTRAL	2006	113.44	84.33	150.76	RW2
Zimbabwe	MASHONALAND CENTRAL	2007	108.03	81.81	141.30	RW2
Zimbabwe	MASHONALAND CENTRAL	2008	102.95	75.63	138.76	RW2
Zimbabwe	MASHONALAND CENTRAL	2009	97.82	64.56	146.75	RW2
Zimbabwe	MASHONALAND CENTRAL	2010	93.26	50.00	169.09	RW2
Zimbabwe	MASHONALAND CENTRAL	2011	88.30	38.20	196.15	RW2
Zimbabwe	MASHONALAND CENTRAL	2012	84.09	27.99	232.04	RW2
Zimbabwe	MASHONALAND CENTRAL	2013	79.62	19.72	279.48	RW2
Zimbabwe	MASHONALAND CENTRAL	2014	75.62	13.63	330.65	RW2
Zimbabwe	MASHONALAND CENTRAL	2015	71.88	9.38	406.93	RW2
Zimbabwe	MASHONALAND CENTRAL	2016	68.13	6.31	482.66	RW2
Zimbabwe	MASHONALAND CENTRAL	2017	64.80	4.06	570.97	RW2
Zimbabwe	MASHONALAND CENTRAL	2018	60.98	2.47	651.23	RW2
Zimbabwe	MASHONALAND CENTRAL	2019	57.69	1.60	725.97	RW2
Zimbabwe	MASHONALAND EAST	1980	95.73	61.59	144.28	RW2
Zimbabwe	MASHONALAND EAST	1981	90.98	64.89	125.09	RW2
Zimbabwe	MASHONALAND EAST	1982	86.48	63.93	116.29	RW2
Zimbabwe	MASHONALAND EAST	1983	82.44	60.49	112.28	RW2
Zimbabwe	MASHONALAND EAST	1984	78.97	57.24	108.38	RW2
Zimbabwe	MASHONALAND EAST	1985	75.79	56.04	101.55	RW2
Zimbabwe	MASHONALAND EAST	1986	73.98	56.21	96.57	RW2
Zimbabwe	MASHONALAND EAST	1987	73.30	56.76	94.29	RW2
Zimbabwe	MASHONALAND EAST	1988	73.84	56.73	95.44	RW2
Zimbabwe	MASHONALAND EAST	1989	75.59	57.54	99.13	RW2
Zimbabwe	MASHONALAND EAST	1990	78.93	60.11	102.88	RW2
Zimbabwe	MASHONALAND EAST	1991	83.62	64.63	107.67	RW2
Zimbabwe	MASHONALAND EAST	1992	89.86	70.33	113.95	RW2
Zimbabwe	MASHONALAND EAST	1993	97.32	75.47	124.19	RW2
Zimbabwe	MASHONALAND EAST	1994	105.76	80.83	136.30	RW2
Zimbabwe	MASHONALAND EAST	1995	115.34	88.78	150.46	RW2
Zimbabwe	MASHONALAND EAST	1996	123.57	96.36	159.57	RW2
Zimbabwe	MASHONALAND EAST	1997	130.21	101.43	166.36	RW2
Zimbabwe	MASHONALAND EAST	1998	134.25	104.17	172.88	RW2
Zimbabwe	MASHONALAND EAST	1999	136.03	103.90	176.83	RW2
Zimbabwe	MASHONALAND EAST	2000	134.76	101.42	175.59	RW2
Zimbabwe	MASHONALAND EAST	2001	132.76	100.63	172.37	RW2
Zimbabwe	MASHONALAND EAST	2002	130.03	98.63	169.27	RW2
Zimbabwe	MASHONALAND EAST	2003	126.88	94.85	166.44	RW2

Continued on next page

Country	Region	Year	Median	Lower	Upper	Method
Zimbabwe	MASHONALAND EAST	2004	123.46	90.45	165.10	RW2
Zimbabwe	MASHONALAND EAST	2005	120.40	87.77	162.36	RW2
Zimbabwe	MASHONALAND EAST	2006	117.00	86.93	155.04	RW2
Zimbabwe	MASHONALAND EAST	2007	113.79	86.49	148.64	RW2
Zimbabwe	MASHONALAND EAST	2008	110.50	81.74	148.44	RW2
Zimbabwe	MASHONALAND EAST	2009	107.45	70.81	160.32	RW2
Zimbabwe	MASHONALAND EAST	2010	104.30	56.78	187.03	RW2
Zimbabwe	MASHONALAND EAST	2011	101.38	44.16	218.98	RW2
Zimbabwe	MASHONALAND EAST	2012	98.40	33.74	265.51	RW2
Zimbabwe	MASHONALAND EAST	2013	95.38	24.25	315.63	RW2
Zimbabwe	MASHONALAND EAST	2014	92.45	17.50	385.32	RW2
Zimbabwe	MASHONALAND EAST	2015	90.12	11.77	468.26	RW2
Zimbabwe	MASHONALAND EAST	2016	86.84	8.28	544.62	RW2
Zimbabwe	MASHONALAND EAST	2017	84.10	5.25	631.29	RW2
Zimbabwe	MASHONALAND EAST	2018	81.87	3.48	707.08	RW2
Zimbabwe	MASHONALAND EAST	2019	79.53	2.19	774.95	RW2
Zimbabwe	MASHONALAND WEST	1980	121.11	80.87	176.94	RW2
Zimbabwe	MASHONALAND WEST	1981	114.89	85.64	152.11	RW2
Zimbabwe	MASHONALAND WEST	1982	108.89	83.35	140.48	RW2
Zimbabwe	MASHONALAND WEST	1983	103.48	78.53	135.91	RW2
Zimbabwe	MASHONALAND WEST	1984	98.72	74.05	131.74	RW2
Zimbabwe	MASHONALAND WEST	1985	94.50	71.93	122.67	RW2
Zimbabwe	MASHONALAND WEST	1986	91.84	71.87	115.92	RW2
Zimbabwe	MASHONALAND WEST	1987	90.50	72.15	113.05	RW2
Zimbabwe	MASHONALAND WEST	1988	90.63	71.81	113.86	RW2
Zimbabwe	MASHONALAND WEST	1989	92.35	71.86	117.65	RW2
Zimbabwe	MASHONALAND WEST	1990	95.75	74.55	122.33	RW2
Zimbabwe	MASHONALAND WEST	1991	100.81	79.81	127.32	RW2
Zimbabwe	MASHONALAND WEST	1992	107.49	85.73	133.54	RW2
Zimbabwe	MASHONALAND WEST	1993	115.61	91.30	144.41	RW2
Zimbabwe	MASHONALAND WEST	1994	124.75	96.57	158.49	RW2
Zimbabwe	MASHONALAND WEST	1995	135.22	105.44	174.24	RW2
Zimbabwe	MASHONALAND WEST	1996	143.85	112.80	183.21	RW2
Zimbabwe	MASHONALAND WEST	1997	150.79	118.72	190.35	RW2
Zimbabwe	MASHONALAND WEST	1998	154.84	121.38	196.84	RW2
Zimbabwe	MASHONALAND WEST	1999	156.31	120.67	202.28	RW2
Zimbabwe	MASHONALAND WEST	2000	154.45	117.73	198.51	RW2
Zimbabwe	MASHONALAND WEST	2001	151.87	116.73	193.61	RW2
Zimbabwe	MASHONALAND WEST	2002	148.42	114.19	189.96	RW2
Zimbabwe	MASHONALAND WEST	2003	144.45	109.82	187.11	RW2
Zimbabwe	MASHONALAND WEST	2004	140.65	104.59	185.73	RW2
Zimbabwe	MASHONALAND WEST	2005	136.82	100.23	182.72	RW2
Zimbabwe	MASHONALAND WEST	2006	133.05	99.01	176.11	RW2
Zimbabwe	MASHONALAND WEST	2007	129.02	97.04	169.69	RW2
Zimbabwe	MASHONALAND WEST	2008	125.19	91.24	169.70	RW2
Zimbabwe	MASHONALAND WEST	2009	121.78	79.67	182.93	RW2
Zimbabwe	MASHONALAND WEST	2010	118.03	63.29	210.63	RW2
Zimbabwe	MASHONALAND WEST	2011	114.40	50.09	246.70	RW2
Zimbabwe	MASHONALAND WEST	2012	110.87	37.39	295.36	RW2
Zimbabwe	MASHONALAND WEST	2013	107.53	27.02	349.20	RW2
Zimbabwe	MASHONALAND WEST	2014	104.74	19.12	417.77	RW2
Zimbabwe	MASHONALAND WEST	2015	101.27	13.78	500.25	RW2
Zimbabwe	MASHONALAND WEST	2016	97.90	9.05	577.22	RW2
Zimbabwe	MASHONALAND WEST	2017	95.43	6.06	658.77	RW2
Zimbabwe	MASHONALAND WEST	2018	91.02	3.98	739.36	RW2
Zimbabwe	MASHONALAND WEST	2019	88.24	2.51	803.95	RW2
Zimbabwe	MASVINGO	1980	111.37	74.52	162.54	RW2
Zimbabwe	MASVINGO	1981	103.84	77.50	137.29	RW2
Zimbabwe	MASVINGO	1982	96.91	74.41	125.33	RW2
Zimbabwe	MASVINGO	1983	90.37	68.39	119.44	RW2
Zimbabwe	MASVINGO	1984	84.75	63.59	113.45	RW2
Zimbabwe	MASVINGO	1985	79.74	60.41	104.11	RW2
Zimbabwe	MASVINGO	1986	76.23	59.31	97.08	RW2

Continued on next page

Country	Region	Year	Median	Lower	Upper	Method
Zimbabwe	MASVINGO	1987	74.03	58.78	93.35	RW2
Zimbabwe	MASVINGO	1988	73.09	57.37	92.99	RW2
Zimbabwe	MASVINGO	1989	73.48	56.62	94.72	RW2
Zimbabwe	MASVINGO	1990	75.24	58.21	96.83	RW2
Zimbabwe	MASVINGO	1991	78.28	61.47	99.53	RW2
Zimbabwe	MASVINGO	1992	82.54	65.47	103.72	RW2
Zimbabwe	MASVINGO	1993	87.91	68.63	111.36	RW2
Zimbabwe	MASVINGO	1994	93.94	72.13	120.52	RW2
Zimbabwe	MASVINGO	1995	100.81	77.66	131.80	RW2
Zimbabwe	MASVINGO	1996	106.29	82.49	137.90	RW2
Zimbabwe	MASVINGO	1997	110.08	85.56	142.00	RW2
Zimbabwe	MASVINGO	1998	111.95	86.23	145.31	RW2
Zimbabwe	MASVINGO	1999	111.62	84.80	147.10	RW2
Zimbabwe	MASVINGO	2000	108.61	81.40	142.81	RW2
Zimbabwe	MASVINGO	2001	105.20	79.25	137.64	RW2
Zimbabwe	MASVINGO	2002	101.24	76.49	132.71	RW2
Zimbabwe	MASVINGO	2003	96.89	72.11	128.15	RW2
Zimbabwe	MASVINGO	2004	92.69	67.78	124.83	RW2
Zimbabwe	MASVINGO	2005	88.62	64.36	120.33	RW2
Zimbabwe	MASVINGO	2006	84.76	62.88	112.75	RW2
Zimbabwe	MASVINGO	2007	80.81	61.48	105.75	RW2
Zimbabwe	MASVINGO	2008	77.21	56.90	103.86	RW2
Zimbabwe	MASVINGO	2009	73.53	48.32	111.28	RW2
Zimbabwe	MASVINGO	2010	70.25	37.58	129.79	RW2
Zimbabwe	MASVINGO	2011	66.77	28.60	150.46	RW2
Zimbabwe	MASVINGO	2012	63.53	21.32	183.72	RW2
Zimbabwe	MASVINGO	2013	60.58	15.07	222.25	RW2
Zimbabwe	MASVINGO	2014	57.67	10.35	272.07	RW2
Zimbabwe	MASVINGO	2015	54.93	7.17	331.02	RW2
Zimbabwe	MASVINGO	2016	52.32	4.65	408.64	RW2
Zimbabwe	MASVINGO	2017	49.69	3.09	492.43	RW2
Zimbabwe	MASVINGO	2018	46.91	2.06	571.89	RW2
Zimbabwe	MASVINGO	2019	44.87	1.27	661.26	RW2
Zimbabwe	MATABELELAND NORTH	1980	110.08	72.67	163.14	RW2
Zimbabwe	MATABELELAND NORTH	1981	102.65	75.28	138.18	RW2
Zimbabwe	MATABELELAND NORTH	1982	95.63	72.40	125.40	RW2
Zimbabwe	MATABELELAND NORTH	1983	89.23	66.78	119.29	RW2
Zimbabwe	MATABELELAND NORTH	1984	83.59	61.78	113.32	RW2
Zimbabwe	MATABELELAND NORTH	1985	78.59	58.80	103.89	RW2
Zimbabwe	MATABELELAND NORTH	1986	75.12	57.55	96.86	RW2
Zimbabwe	MATABELELAND NORTH	1987	72.76	56.63	92.77	RW2
Zimbabwe	MATABELELAND NORTH	1988	71.88	55.38	92.44	RW2
Zimbabwe	MATABELELAND NORTH	1989	72.23	54.75	94.44	RW2
Zimbabwe	MATABELELAND NORTH	1990	73.94	56.07	96.70	RW2
Zimbabwe	MATABELELAND NORTH	1991	76.90	59.07	99.59	RW2
Zimbabwe	MATABELELAND NORTH	1992	81.17	62.64	104.03	RW2
Zimbabwe	MATABELELAND NORTH	1993	86.56	66.24	111.40	RW2
Zimbabwe	MATABELELAND NORTH	1994	92.58	69.47	121.02	RW2
Zimbabwe	MATABELELAND NORTH	1995	99.55	74.90	132.09	RW2
Zimbabwe	MATABELELAND NORTH	1996	105.11	79.47	138.02	RW2
Zimbabwe	MATABELELAND NORTH	1997	109.17	82.65	143.15	RW2
Zimbabwe	MATABELELAND NORTH	1998	111.22	84.09	146.71	RW2
Zimbabwe	MATABELELAND NORTH	1999	111.23	82.72	148.91	RW2
Zimbabwe	MATABELELAND NORTH	2000	108.57	79.49	145.01	RW2
Zimbabwe	MATABELELAND NORTH	2001	105.64	77.87	140.94	RW2
Zimbabwe	MATABELELAND NORTH	2002	101.85	75.15	136.13	RW2
Zimbabwe	MATABELELAND NORTH	2003	98.20	71.39	133.59	RW2
Zimbabwe	MATABELELAND NORTH	2004	94.20	67.08	130.40	RW2
Zimbabwe	MATABELELAND NORTH	2005	90.53	63.99	126.00	RW2
Zimbabwe	MATABELELAND NORTH	2006	86.83	62.35	119.38	RW2
Zimbabwe	MATABELELAND NORTH	2007	83.38	60.63	113.30	RW2
Zimbabwe	MATABELELAND NORTH	2008	79.92	56.46	111.95	RW2
Zimbabwe	MATABELELAND NORTH	2009	76.59	48.70	119.05	RW2

Continued on next page

Country	Region	Year	Median	Lower	Upper	Method
Zimbabwe	MATABELELAND NORTH	2010	73.20	38.47	137.48	RW2
Zimbabwe	MATABELELAND NORTH	2011	70.29	29.10	159.44	RW2
Zimbabwe	MATABELELAND NORTH	2012	67.06	21.77	194.89	RW2
Zimbabwe	MATABELELAND NORTH	2013	64.24	15.78	237.86	RW2
Zimbabwe	MATABELELAND NORTH	2014	61.20	10.82	290.21	RW2
Zimbabwe	MATABELELAND NORTH	2015	58.94	7.50	361.39	RW2
Zimbabwe	MATABELELAND NORTH	2016	56.76	5.04	437.27	RW2
Zimbabwe	MATABELELAND NORTH	2017	53.90	3.28	512.61	RW2
Zimbabwe	MATABELELAND NORTH	2018	51.15	2.16	597.11	RW2
Zimbabwe	MATABELELAND NORTH	2019	49.21	1.36	692.89	RW2
Zimbabwe	MATABELELAND SOUTH	1980	76.92	50.60	114.68	RW2
Zimbabwe	MATABELELAND SOUTH	1981	72.37	53.18	97.51	RW2
Zimbabwe	MATABELELAND SOUTH	1982	68.08	51.31	89.86	RW2
Zimbabwe	MATABELELAND SOUTH	1983	64.09	47.62	86.28	RW2
Zimbabwe	MATABELELAND SOUTH	1984	60.68	44.45	83.23	RW2
Zimbabwe	MATABELELAND SOUTH	1985	57.70	42.58	76.94	RW2
Zimbabwe	MATABELELAND SOUTH	1986	55.68	42.31	72.63	RW2
Zimbabwe	MATABELELAND SOUTH	1987	54.59	42.04	70.52	RW2
Zimbabwe	MATABELELAND SOUTH	1988	54.51	41.66	71.00	RW2
Zimbabwe	MATABELELAND SOUTH	1989	55.42	41.64	73.09	RW2
Zimbabwe	MATABELELAND SOUTH	1990	57.35	43.38	75.93	RW2
Zimbabwe	MATABELELAND SOUTH	1991	60.40	46.11	78.85	RW2
Zimbabwe	MATABELELAND SOUTH	1992	64.43	49.63	83.15	RW2
Zimbabwe	MATABELELAND SOUTH	1993	69.46	53.07	90.10	RW2
Zimbabwe	MATABELELAND SOUTH	1994	75.08	56.22	98.85	RW2
Zimbabwe	MATABELELAND SOUTH	1995	81.53	61.48	108.98	RW2
Zimbabwe	MATABELELAND SOUTH	1996	86.86	65.65	115.23	RW2
Zimbabwe	MATABELELAND SOUTH	1997	91.04	68.86	120.23	RW2
Zimbabwe	MATABELELAND SOUTH	1998	93.51	69.85	124.53	RW2
Zimbabwe	MATABELELAND SOUTH	1999	94.04	69.37	126.88	RW2
Zimbabwe	MATABELELAND SOUTH	2000	92.52	67.45	124.74	RW2
Zimbabwe	MATABELELAND SOUTH	2001	90.51	65.97	122.17	RW2
Zimbabwe	MATABELELAND SOUTH	2002	88.05	64.21	119.35	RW2
Zimbabwe	MATABELELAND SOUTH	2003	85.13	60.96	117.08	RW2
Zimbabwe	MATABELELAND SOUTH	2004	82.30	57.90	115.35	RW2
Zimbabwe	MATABELELAND SOUTH	2005	79.50	55.21	112.72	RW2
Zimbabwe	MATABELELAND SOUTH	2006	76.78	54.14	107.75	RW2
Zimbabwe	MATABELELAND SOUTH	2007	74.10	52.72	103.22	RW2
Zimbabwe	MATABELELAND SOUTH	2008	71.55	49.29	102.65	RW2
Zimbabwe	MATABELELAND SOUTH	2009	69.03	42.86	109.66	RW2
Zimbabwe	MATABELELAND SOUTH	2010	66.43	34.18	127.12	RW2
Zimbabwe	MATABELELAND SOUTH	2011	64.18	26.39	150.71	RW2
Zimbabwe	MATABELELAND SOUTH	2012	61.72	19.89	182.04	RW2
Zimbabwe	MATABELELAND SOUTH	2013	59.50	14.48	225.90	RW2
Zimbabwe	MATABELELAND SOUTH	2014	57.27	10.30	273.48	RW2
Zimbabwe	MATABELELAND SOUTH	2015	55.43	7.06	341.17	RW2
Zimbabwe	MATABELELAND SOUTH	2016	53.19	4.67	418.04	RW2
Zimbabwe	MATABELELAND SOUTH	2017	51.42	3.08	520.39	RW2
Zimbabwe	MATABELELAND SOUTH	2018	49.64	2.00	601.00	RW2
Zimbabwe	MATABELELAND SOUTH	2019	48.07	1.33	679.60	RW2
Zimbabwe	MIDLANDS	1980	113.04	76.31	163.12	RW2
Zimbabwe	MIDLANDS	1981	105.84	79.68	139.18	RW2
Zimbabwe	MIDLANDS	1982	99.29	76.52	127.62	RW2
Zimbabwe	MIDLANDS	1983	93.17	70.78	122.84	RW2
Zimbabwe	MIDLANDS	1984	87.80	65.55	117.55	RW2
Zimbabwe	MIDLANDS	1985	83.05	62.68	108.50	RW2
Zimbabwe	MIDLANDS	1986	79.79	61.99	101.97	RW2
Zimbabwe	MIDLANDS	1987	77.86	61.28	98.13	RW2
Zimbabwe	MIDLANDS	1988	77.29	60.57	98.24	RW2
Zimbabwe	MIDLANDS	1989	78.07	60.04	100.78	RW2
Zimbabwe	MIDLANDS	1990	80.19	61.89	103.30	RW2
Zimbabwe	MIDLANDS	1991	83.82	65.99	106.16	RW2
Zimbabwe	MIDLANDS	1992	88.76	70.21	111.11	RW2

Continued on next page

Country	Region	Year	Median	Lower	Upper	Method
Zimbabwe	MIDLANDS	1993	94.94	74.73	119.33	RW2
Zimbabwe	MIDLANDS	1994	101.73	78.29	129.82	RW2
Zimbabwe	MIDLANDS	1995	109.70	84.95	142.33	RW2
Zimbabwe	MIDLANDS	1996	115.94	90.57	148.78	RW2
Zimbabwe	MIDLANDS	1997	120.66	94.29	153.83	RW2
Zimbabwe	MIDLANDS	1998	123.09	95.69	158.44	RW2
Zimbabwe	MIDLANDS	1999	123.23	94.70	160.33	RW2
Zimbabwe	MIDLANDS	2000	120.75	91.38	156.83	RW2
Zimbabwe	MIDLANDS	2001	117.69	89.70	152.00	RW2
Zimbabwe	MIDLANDS	2002	113.79	87.18	147.19	RW2
Zimbabwe	MIDLANDS	2003	109.74	82.44	144.13	RW2
Zimbabwe	MIDLANDS	2004	105.70	78.26	141.01	RW2
Zimbabwe	MIDLANDS	2005	101.70	74.41	137.00	RW2
Zimbabwe	MIDLANDS	2006	97.88	73.21	129.80	RW2
Zimbabwe	MIDLANDS	2007	94.12	71.73	122.81	RW2
Zimbabwe	MIDLANDS	2008	90.42	66.66	121.62	RW2
Zimbabwe	MIDLANDS	2009	86.92	57.12	130.88	RW2
Zimbabwe	MIDLANDS	2010	83.26	44.85	152.66	RW2
Zimbabwe	MIDLANDS	2011	79.99	34.33	178.07	RW2
Zimbabwe	MIDLANDS	2012	76.66	25.35	214.83	RW2
Zimbabwe	MIDLANDS	2013	73.78	18.23	260.70	RW2
Zimbabwe	MIDLANDS	2014	70.70	12.85	320.65	RW2
Zimbabwe	MIDLANDS	2015	67.60	8.76	388.42	RW2
Zimbabwe	MIDLANDS	2016	64.79	5.86	462.76	RW2
Zimbabwe	MIDLANDS	2017	61.92	3.92	547.15	RW2
Zimbabwe	MIDLANDS	2018	59.23	2.45	637.67	RW2
Zimbabwe	MIDLANDS	2019	57.03	1.55	720.86	RW2

4 Bibliography

[1] L. Alkema and J.R. New. Global estimation of child mortality using a Bayesian B-spline bias-reduction model. *The Annals of Applied Statistics*, 8:2122–2149, 2014.

[2] P.D. Allison. *Event History and Survival Analysis, Second Edition*, volume 46. SAGE publications, 2014.

[3] J. Besag, J. York, and A. Mollié. Bayesian image restoration with two applications in spatial statistics. *Annals of the Institute of Statistics and Mathematics*, 43:1–59, 1991.

[4] S. Bhatt, E. Cameron, S.R. Flaxman, D.J. Weiss, D.L. Smith, and P.W. Gething. Improved prediction accuracy for disease risk mapping using Gaussian process stacked generalization. *Journal of The Royal Society Interface*, 14:20170520, 2017.

[5] D.A. Binder. On the variances of asymptotically normal estimators from complex surveys. *International Statistical Review*, 51:279–292, 1983.

[6] Marshall Burke, Sam Heft-Neal, and Eran Bendavid. Sources of variation in under-5 mortality across sub-Saharan Africa: a spatial analysis. *The Lancet Global Health*, 4:e936–e945, 2016.

[7] C. Chen, J. Wakefield, and T. Lumley. The use of sample weights in Bayesian hierarchical models for small area estimation. *Spatial and Spatio-Temporal Epidemiology*, 11:33–43, 2014.

[8] P. Congdon and P. Lloyd. Estimating small area diabetes prevalence in the US using the behavioral risk factor surveillance system. *Journal of Data Science*, 8:235–252, 2010.

[9] Laura Dwyer-Lindgren, Frank Kakungu, Peter Hangoma, Marie Ng, Haidong Wang, Abraham D Flaxman, Felix Masiye, and Emmanuela Gakidou. Estimation of district-level under-5 mortality in Zambia using birth history data, 1980–2010. *Spatial and Spatio-Temporal Epidemiology*, 11:89–107, 2014.

[10] GBD 2016 Mortality Collaborators. Global, regional, and national under-5 mortality, adult mortality, age-specific mortality, and life expectancy, 1970–2016: a systematic analysis for the Global Burden of Disease Study 2016. *The Lancet*, 390:1084–1150, 2017.

[11] P. Gething, A. Tatem, T. Bird, and C.R. Burgert-Brucker. Creating spatial interpolation surfaces with DHS data. Technical report, ICF International, 2015. DHS Spatial Analysis Reports No. 11.

[12] N. Golding, R. Burstein, J. Longbottom, A.J. Browne, N. Fullman, A. Osgood-Zimmerman, L. Earl, S. Bhatt, E. Cameron, D.C. Casey, L. Dwyer-Lindgren, T.H. Farag, A.D. Flaxman, M.S. Fraser, P.W. Gething, H.S. Gibson, N. Graetz, L.K. Krause, X.R. Kulikoff, S.S. Lim, B. Mappin, C. Morozoff, R.C. Reiner, A. Sligar, D.L. Smith, H. Wang, D.J. Weiss, C.J.L. Murray, C.L. Moyes, and S.I. Hay. Mapping under-5 and neonatal mortality in Africa, 2000–15: a baseline analysis for the Sustainable Development Goals. *The Lancet*, 2017. Available online, September 25th, 2017.

[13] Timothy Hallett, Sarah-Jane Anderson, Cynthia Adobea Asante, Noah Bartlett, Victoria Bendaud, Samir Bhatt, Clara Burgert, Diego Fernando Cuadros, Janet Dzangare, Daniela Fecht, et al. Evaluation of geospatial methods to generate subnational HIV prevalence estimates for local level planning. *AIDS*, 30:1467–1474, 2016.

[14] L. Knorr-Held. Bayesian modelling of inseparable space-time variation in disease risk. *Statistics in Medicine*, 19:2555–2567, 2000.

[15] Joseph Larmarange and Victoria Bendaud. HIV estimates at second subnational level from national population-based surveys. *AIDS*, 28:S469–S476, 2014.

[16] B.G. Leroux, X. Lei, and N. Breslow. Estimation of disease rates in small areas: A new mixed model for spatial dependence. In M.E. Halloran and D.A Berry, editors, *Statistical Models in Epidemiology, the Environment and Clinical Trials*, pages 179–192. Springer, New York, 1999.

[17] F. Lindgren, H. Rue, and J. Lindström. An explicit link between Gaussian fields and Gaussian Markov random fields: the stochastic differential equation approach (with discussion). *Journal of the Royal Statistical Society, Series B*, 73:423–498, 2011.

[18] S.L. Lohr. *Sampling: Design and Analysis, Second Edition.* Brooks/Cole Cengage Learning, Boston, 2010.

[19] Bryan D Martin, Zehang R Li, Yuan Hsiao, Jessica Godwin, Jon Wakefield, and Samuel J Clark. *SUMMER: Spatio-Temporal Under-Five Mortality Methods for Estimation*, 2018. R package version 0.2.1.

[20] L. Mercer, J. Wakefield, A. Pantazis, A. Lutambi, H. Mosanja, and S. Clark. Small area estimation of childhood of childhood mortality in the absence of vital registration. *Annals of Applied Statistics*, 9:1889–1905, 2015.

[21] C. Pezzulo, E.C. Utazi, T.J. Bird A. Sorichetta, A.J. Tatem, J. Yourkavitch, T. Pullum, and C.R. Burgert-Brucker. Subnational modelling of child mortality and its drivers across 27 countries in Sub-Saharan Africa. Technical report, Paper presented at PAA Meeting, 2017.

[22] Aaron T Porter, Scott H Holan, Christopher K Wikle, and Noel Cressie. Spatial Fay–Herriot models for small area estimation with functional covariates. *Spatial Statistics*, 10:27–42, 2014.

[23] J.N.K. Rao and I. Molina. *Small Area Estimation, Second Edition.* John Wiley, New York, 2015.

[24] H. Rue and L. Held. *Gaussian Markov random fields: theory and application.* Chapman and Hall/CRC Press, Boca Raton, 2005.

[25] H. Rue, S. Martino, and N. Chopin. Approximate Bayesian inference for latent Gaussian models using integrated nested Laplace approximations (with discussion). *Journal of the Royal Statistical Society, Series B*, 71:319–392, 2009.

[26] Mark J Van der Laan, Eric C Polley, and Alan E Hubbard. Super learner. *Statistical Applications in Genetics and Molecular Biology*, 6, 2007.

[27] Yannick Vandendijck, Christel Faes, Russel S Kirby, A Lawson, and Niel Hens. Model-based inference for small area estimation with sampling weights. *Spatial Statistics*, 18:455–473, 2016.

[28] J. Wakefield. Ecologic studies revisited. *Annual Review of Public Health*, 29:75–90, 2008.

[29] Jon Wakefield, Geir-Arne Fuglstad, Andrea Riebler, Jessica Godwin, Katie Wilson, and Samuel J Clark. Estimating under five mortality in space and time in a developing world context. *Statistical Methods in Medical Research*, 2018. To Appear.

[30] Neff Walker, Kenneth Hill, and Fengmin Zhao. Child mortality estimation: methods used to adjust for bias due to aids in estimating trends in under-five mortality. *PLoS Medicine*, 9(8):e1001298, 2012.

[31] L.A. Waller and C.A. Gotway. *Applied Spatial Statistics for Public Health Data*. John Wiley and Sons, 2004.

[32] Kevin Watjou, Christel Faes, A Lawson, RS Kirby, M Aregay, R Carroll, and Yannick Vandendijck. Spatial small area smoothing models for handling survey data with nonresponse. *Statistics in medicine*, 36(23):3708–3745, 2017.

[33] K. Wilson and J. Wakefield. Pointless continuous spatial surface reconstruction. *arXiv:1709.09659*, 2017.

[34] D.H. Wolpert. Stacked generalization. *Neural Networks*, 5:241–259, 1992.

[35] Yong You and Qian M Zhou. Hierarchical Bayes small area estimation under a spatial model with application to health survey data. *Survey Methodology*, 37:25–37, 2011.
